# High‐grade B‐cell lymphoma not otherwise specified, with diffuse large B‐cell lymphoma gene expression signatures: Genomic analysis and potential therapeutics

**DOI:** 10.1002/ajh.27513

**Published:** 2024-11-16

**Authors:** Waseem Lone, Alyssa Bouska, Tyler A. Herek, Catalina Amador, Joo Song, Alexander M. Xu, Dylan Jochum, Issa Ismail Issa, Dennis D. Weisenburger, Xuan Zhang, Sharath Kumar Bhagavathi, Tayla B. Heavican‐Foral, Sunandini Sharma, Ab Rauf Shah, Abdul Rouf Mir, Aisha Ahmad Alkhinji, Dalia El‐Gamal, Bhavana J. Dave, Keenan Hartert, Jiayu Yu, Mallick Saumyaranjan, Timothy C. Greiner, Julie Vose, Timothy W. McKeithan, Kai Fu, Michael Green, Chengfeng Bi, Akil Merchant, Wing C. Chan, Javeed Iqbal

**Affiliations:** ^1^ Pathology, Microbiology and Immunology University of Nebraska Medical Center Omaha Nebraska USA; ^2^ Department of Pathology University of Miami Miami Florida USA; ^3^ Department of Pathology City of Hope National Medical Center Duarte California USA; ^4^ Cedars‐Sinai Medical Center and Samuel Oschin Comprehensive Cancer Institute Los Angeles California USA; ^5^ Department of Pathology University of Iowa Iowa City Iowa USA; ^6^ Department of Medical Oncology Dana‐Farber Cancer Institute Boston Massachusetts USA; ^7^ Eppley Institute for Cancer and Allied Diseases University of Nebraska Medical Center Omaha Nebraska USA; ^8^ Human Genetics Laboratory University of Nebraska Medical Center Omaha Nebraska USA; ^9^ Department of Biological Sciences Minnesota State University Mankato Minnesota USA; ^10^ All India Institute of Medical Sciences New Delhi India; ^11^ Division of Hematology and Oncology, Department of Internal Medicine University of Nebraska Medical Center Omaha Nebraska USA; ^12^ Department of Pathology Roswell Park Comprehensive Cancer Center Buffalo New York USA; ^13^ Department of Lymphoma/Myeloma and Genomic Medicine University of Texas MD Anderson Cancer Center Houston Texas USA

## Abstract

High‐grade B‐cell lymphoma not otherwise specified (HGBCL, NOS) has overlapping morphological and genetic features with diffuse large B‐cell lymphoma (DLBCL) and Burkitt lymphoma (BL), leading to uncertainty in its diagnosis and clinical management. Using functional genomic approaches, we previously characterized HGBCL and NOS, that demonstrate gene expression profiling (GEP), and genetic signatures similar to BL. Herein, we characterize distinct HGBCL, NOS, cohort (*n* = 55) in adults (*n* = 45) and in children (*n* = 10), and compared the GEP, genomic DNA copy number (CN), and mutational spectrum with *de novo* DLBCL (*n* = 85) and BL (*n* = 52). This subgroup, representing ~60% of HGBCL, NOS, lack gene‐expression signature of BL and double hit/dark zone lymphoma, but express DLBCL like signatures and are characterized by either GCB‐ or ABC‐like mRNA signatures and exhibit higher genomic complexity, similar to *de novo* DLBCL, and show alteration in genes regulating B‐cell activation (*CD79B*, *MYD88*, *PRDM1*, *TBLIXR1*, *CARD11*), epigenome (*KMT2D*, *TET2*) and cell cycle transition (*TP53*, *ASPM*). However, recurrent mutations in genes often mutated in BL (DDX3X, GNA13, CCND3), but rare in DLBCL, are also present in HGBCL‐NOS, highlighting genetic heterogeneity. Consistent with mutation spectrum, frequent genomic CN alterations in genes regulating B‐cell activation (del‐*PRDM1*, gain‐*BCL6*, ‐*REL*, ‐*STAT3*) and cell cycle regulators (del‐*TP53*, del‐*CDKN2A*, del‐*RB1*, gain‐*CCND3*) were observed. Pediatric cases showed GCB‐DLBCL‐like mRNA signatures, but also featured hallmark mutations of pediatric BL. Frequent oncogenic *PIM1* mutations were present in adult HGBCL, NOS. *In vitro* analyses with pharmacologic or genetic inhibition of *PIM1 expression* triggered B‐cell activation and NF‐κB‐induced apoptosis, suggesting that *PIM1* is a rational therapeutic target.

## 1 INTRODUCTION

High‐grade B‐cell lymphoma (HGBCL) is a morphologically aggressive subtype of non‐Hodgkin lymphoma (NHL) characterized by high proliferation rate, demonstrated by Ki67 expression, and an aggressive clinical course.^1^ Unfortunately, polychemotherapy, immunochemotherapy, or intensive regimens have not resulted in a survival advantage, and no established treatment regimens are recommended.^2^ HGBCL is a heterogenous group that includes DLBCL/HGBCL‐*MYC*/*BCL2* (i.e., harboring *MYC* and *BCL2* rearrangements) representing about 70% of HGBCL.^3^ The other 30% are not classifiable as DLBCL or BL using established morphologic and immunophenotypic criteria and are therefore categorized as HGBCL, not otherwise specified (HGBCL, NOS).^4^, ^5^ The latter exhibits blastoid or Burkitt‐like morphology with a high proliferative index and does not include the hallmark double‐hit or triple‐hit rearrangements involving *MYC*, *BCL2*, or *BCL6*. This rare tumor subtype is biologically heterogenous, but is yet to be characterized exclusively using molecular analysis. Historically, these cases were described as Burkitt‐like lymphoma (BLL), small non‐cleaved B‐cell lymphoma (SNC‐BCL), and atypical BL gray‐zone lymphomas (GZL).^6^ While, DLBCL and BL are extensively characterized and are classified by distinct differences in morphology, cytogenetics, and molecular signatures,^7^, ^8^ the diagnostic criteria for HGBCL without a hallmark translocation remain ambiguous, and thus limit the clinical utility and impede progress in translational research.

Molecular studies have previosuly estimated that one‐sixth of adult *de novo* DLBCLs show a BL‐like gene expression profile (GEP) designated as molecular BL (mBL).^9^, ^10^ These cases were inaccurately diagnosed by an expert panel of hematopathologists accentuating the need for an increased understanding of their pathogenesis.^11^ Previously, we reported on a subset of adult HGBCL cases that share molecular (i.e., GEP and genetic) features with BL, with the *MYC*‐ARF‐p53 axis as the primary deregulated signaling pathway.^6^ A subset of these cases lacked *MYC* rearrangement, however the *MYC* function was driven by onco‐miR17~92 due to a focal gain of *MIR17HG* (>30% cases) resulting in B‐cell receptor (BCR) signaling activation and susceptibility to BCR‐BTK inhibitor (ibrutinib) treatment *in vitro*.^6^ This subgroup included cases with the double hit (DHIT) *MYC/BCL2* translocation, and genetic profiles resembling BL, and was referred to as mBL. Subsequent GEP studies demonstrated that the cases with *MYC* and *BCL2* translocations may represent a distinct molecular subtype referred to as “dark zone signature positive (DZ‐sig^+^)” cases and termed as molecular high grade (MGH) subgroup.^12^, ^13^ The “DZ‐sig^+^” also defines 20% *de novo* GCB‐DLBCL with a poor prognosis that harbor a transcriptomic profile resembling BL.

Herein, we characterized a subgroup of HGBCL, NOS, that do not express either mBL or DZ‐sig or MGH signatures, to delineate their underlying pathobiology in comparison to *de novo* DLBCL and mBL. Thus, the overarching aim is to elucidate whether these cases should be considered as a DLBCL‐like molecular entity based on their genomic and transcriptomic profiles and ultimately infer potential therapeutic benefits. We have demonstrated that these HGBCL, NOS cases have genetic features resembling *de novo* DLBCL, and that they lack hallmark BL, DZ‐sig, or MHG genetic features. We explored the relationship of crucial GEP‐defined cell‐of‐origin subtypes within the tumor‐milieu and identified the critical role of *PIM1* mutation frequent in these cases. Overall, HGBCL, NOS, cases with GEP signatures resembling DLBCL exhibit a spectrum of genetic and transcriptomic features that overlap significantly with DLBCL, however, they lack the characteristics of mBL and DHIT/DZ‐sig lymphoma.

## 2 MATERIALS AND METHODS

### 2.1 Patient samples and B‐cell lines

The clinical and pathological characteristics of HGBCL, NOS, including their molecular classification and pathological diagnoses are detailed in Table S1. The clinical and pathological characteristics of BL^6^, ^14^ and DLBCL^15^, ^16^ used for comparison are described in their respective manuscripts. The pathology review, immunohistochemistry, and cytogenetic evaluation of HGBCL, NOS, was reported in an earlier study.^17^ The GEP molecular diagnoses were performed using the Dave, et al.^9^ or Lenz, et al.^15^ gene signatures for BL and DLBCL subgrouping simultaneously, or DLBCL subgrouping, respectively. DZ‐signatures were defined using the DLBCL90 nCounter classifier.^13^ Since cases were assembled from several institutes, upon molecular classification, cases were further re‐reviewed for consensus diagnosis (DDW, WCC, KF, JS, or Lymphoma/Leukemia Molecular Profiling Project (LLMPP)) to ensure the accuracy and relevance of our findings. The expert hematopathology panel carefully evaluated all cases, and cases with blastoid morphology were not included in our analysis. This distinction, frequently made at the time of diagnosis, is essential for exploring molecular changes specific to different lymphoma subtypes. This decision aligns with the need to maintain a clear focus on genomic profiles characteristic of HGBCL in comparison with DLBCL and BL. The study was approved by the Institutional Review Board of the University of Nebraska Medical Center. Genomic data from previously published BL^6^, ^18^ and DLBCL^6^, ^18^ series were included for comparative analysis.

Of the six DLBCL cell lines included in the study, four (TMD8, HBL1, U‐2932, and DHL16) were cultured in RPMI 1640 (Hyclone™‐RPMI 1640 with 2.05 mM L‐Glutamine), whereas two (OCI‐Ly3, OCI‐Ly8) were cultured in IMDM (Lonza Biowhittaker), supplemented with 10% fetal bovine serum (FBS), penicillin G (100 U/mL) and streptomycin (100 μg/mL), and maintained at 37°C in 5% CO_2_.

### 2.2 Structural and functional genomic analysis

#### 2.2.1 Array‐CGH analysis

Following genomic DNA (gDNA) isolation (Qiagen, Texas), DNA copy number (CN) analysis using the Human Mapping 250 K Nsp Array (Affymetrix Inc., California) was performed according to the manufacturer's protocol, and analytical details were described previously.^19^

*Whole exome sequencing* (WES) *and/or target gene deep sequencing*: 45 adult and 10 pediatric HGBCL, NOS, cases were profiled by sequencing. Of these,14 adult and 9 pediatric cases were profiled by both WES and targeted sequencing with a lymphoma gene panel; 23 adult cases were profiled only by WES, 5 cases were analyzed by whole genome sequencing and 2 cases only had targeted sequencing. The targeted DNA sequencing data was from our two earlier studies^6^, ^18^ including a gene panel of 380 common‐mutated genes in B‐NHL. The detailed methodology for variant calling has been described in an earlier study^6^ and in the Supplementary Materials.

#### 2.2.2 Gene expression analysis

Total RNA was isolated for GEP analysis utilizing HG‐U133 plus 2.0 (Affymetrix Inc., California) arrays, and a detailed analysis of subclassification was described in earlier studies.^20^ Of the 55 HGBCL, NOS, cases, 53  cases were profiled by nCounter (NanoString Technologies, WA) and subclassified using the DLBCL90 classifier to assess the dark‐zone gene expression signature (DZ‐sig)^13^ and DLBCL cell‐of‐origin classification (i.e., ABC versus GCB) of the cohort, as described in Ennishi, et al.^13^ See Supplementary Materials for analysis details.

### 2.3 Classification of lymphoma samples by genetic subtyping

HGBCL, NOS and *de novo* DLBCL samples were submitted for genetic subtype classification using the LymphGen 1.0 portal (https://llmpp.nih.gov/lymphgen/index.php).^21^

### 2.4 Tissue imaging mass cytometry (IMC) and cell lineage assignments

Tissue microarrays (TMAs) were generated and a panel of antibodies marking B‐cells, cells within the tumor microenvironment (TME) milieu, and other functional biomarkers were used for TME analysis using the Hyperion Imaging system (Standard Biotools, CA). Raw images were visualized using MCD Viewer (Standard Biotools). Complete details are provided in the Supplementary Materials.

### 2.5 Survival outcome analysis

Overall survival (OS) was estimated using the Kaplan–Meier method, and differences were assessed using the log‐rank test. Statistical analyses were performed with GraphPad Prism (Prism 8.0.2), with *p* < 0.05 considered significant.

### 2.6 *In vitro* functional analysis to elucidate the role of *PIM1*

Experimental details for *PIM1* knockdown (KD) using shRNA and knockout (KO) using CRISPR/cas9, or pharmacological inhibition using AZD1208 are provided in the Supplementary Materials. Standard assays to assess BCR signaling, apoptosis, and cell viability were performed as detailed in the Supplementary Material.

A detailed Materials and Methods section is provided in the Supplementary Materials.

## 3 RESULTS

### 3.1 Patient characteristics and molecular classification by gene expression profiling (GEP)

**FIGURE 1:**
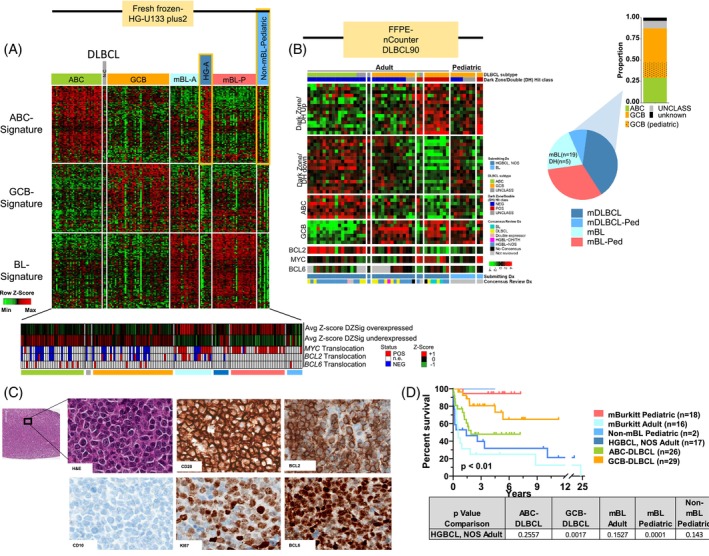
Classification and clinical data for high‐grade B‐cell lymphoma (HGBCL), not otherwise specified (NOS), cases. (A) Heatmap of diffuse large B‐cell lymphoma (DLBCL) and Burkitt lymphoma (BL) Affymetrix classification signatures as described in Dave, et al.,^9^ Lenz, et al.,^15^ and Bouska, et al.^6^ The Dark Zone/DHL‐Signature status (represented by average Z‐score of genes described in Ennishi, et al.^13^) and *MYC*/*BCL2*/*BCL6* translocation status are displayed below. (B) Heatmap of adult HGBCL, NOS, cases and non‐mBL pediatric cases profiled on the DLBCL90 nCounter panel.^13^ An additional cohort of HGBCL, NOS, and pediatric non‐molecular BL (initially diagnosed as BL but lacking the BL GEP signature) were profiled using the nCounter DLBCL90 classifier to determine DLBCL subtypes (ABC‐DLBCL or GCB‐DLBCL) and to identify DZ‐lymphomas. (C) Hematoxylin and eosin (H&E) staining and immunohistochemistry of CD20, BCL2, CD10, Ki67, and BCL6 in a representative HGBCL, NOS, case. This case, HGBCL‐30, is also shown in Figure S1. All images are at 20× magnification. (D) Kaplan–Meier curve comparing overall survival of molecular BL, non‐molecular Burkitt lymphoma (non‐mBL) pediatric cases with DLBCL signatures, HGBCL, NOS, with DLBCL signatures, and *de novo* DLBCL cases. The *p* values (log‐rank) for the different groups compared with HGBCL, NOS, are noted in the table below. [Color figure can be viewed at wileyonlinelibrary.com]

The clinical characteristics of this cohort of HGBCLs, including their molecular classification, genetic status, and pathological diagnoses, are described in Table S1. In our earlier study^6^ 36% of HGBCL, NOS were classified into the mBL subgroup using GEP signatures, and upon review, five cases were DHIT, and others exhibited a focal gain 13q31/miR17~92, del11q21, and a mutational spectrum similar to classical BL and their clinico‐pathological and genomic characteristics described in earlier studies.^6^, ^13^ The remaining HGBCL, NOS cases were classified into molecular DLBCL subtypes using the HG‐U133plus2 platform/profile (Affymetrix Inc.) and are part of the current studies. Figure 1A illustrates the molecular classifier that distinguishes between the molecular subtypes of DLBCL and BL, and HGBCL, NOS cases with the DLBCL signatures show downregulated BL mRNA signature^9^ or the “DZ‐sig”^13^ and lack DHIT translocations, when compared with previously re‐classified mBLs.^22^ These HGBCL, NOS cases from our previous study,^6^ and an additional subset of cases (*n* = 34), and the pediatric non‐molecular BL (initial diagnosis as BL, but lacked the BL GEP signature) were also profiled using the nCounter based‐ DLBCL90 classifier^13^, ^23^ to define DLBCL subtypes (ABC‐DLBCL or GCB‐DLBCL) and DZ‐lymphomas^13^ (Figure 1B). Representative H&E and IHC images of HGBCL, NOS case with a DLBCL GEP are shown (Figure 1C) and additional H&Es and IHC have been included in the supplemental Figure S1 and IHC data presented in Table S1. As expected, the clinical outcomes of adult HGBCL, NOS cases were inferior compared with *de novo* GCB‐DLBCL, but similar to that of BL (Figure 1D), consistent with a recent HGBCL, NOS cohort,^2^ underscoring the need for tailored therapeutic approaches for these high‐risk patients.

### 3.2 Genomic copy number abnormalities (gCNA) in HGBCL, NOS cases expressing DLBCL signatures

#### 3.2.1 Adult HGBCL, NOS

**FIGURE 2:**
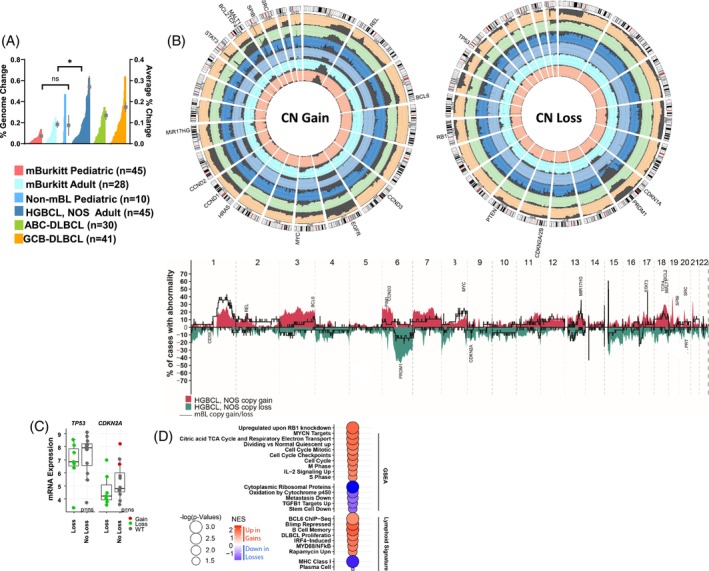
Genomic analysis of HGBCL, NOS, cases with DLBCL signatures. (A) Histogram of % genome‐change per case (left axis) in molecular BL, HGBCL, NOS, with DLBCL signatures, and *de novo* DLBCL cases. The mean and standard error of the mean are shown as in‐laid points and correspond to the right‐axis scale. (B) The upper panel shows circos plots comparing the frequency of gains and losses found in the noted lymphoma types. The scale lines represent 20% increments. The lower panel depicts the frequency plot of gains and losses in adult HGBCL, NOS, and adult mBL. (C) Analysis of mRNA expression and CNA status in adult HGBCL, NOS shows that frequent deletions in *TP53* and *CDKN2A* correlate with decreased mRNA expression levels. (D) Bubble graph of pathways enriched for genes demonstrating both upregulation and CN gain or downregulation and CN loss (compared with WT) in HGBCL, NOS, adult cases. [Color figure can be viewed at wileyonlinelibrary.com]

The cases expressing DLBCL signatures had a significantly higher genomic complexity (estimated as % aberrant genome) compared with cases with mBL, but comparable with *de novo* DLBCL (Figure 2A), suggesting more genetic alterations are essential for lymphomagenesis or aggressive clinical behavior as observed in DLBCL, but unlike BL which predominantly driven by t(8;14) or constitutive *MYC* oncogene overexpression. Adult HGBCL, NOS cases showed distinct genomic alterations with recurrent gains at 1q, 3q27 (*BCL6*), chr7, 18q21 (*BCL2*, *MATL1*, *TCF4*), 19p and 19q13 gain (*SPIB*), with losses including 9p21(*CDKN2A*), 6q21 (*PRDM1*), and 17p (*TP53*) with similar or higher frequency to those observed in *de novo* ABC‐DLBCL (Figures 2B and S2), indicating that genetic aberrations target genes promoting cell‐cycle transitions, inhibiting apoptosis or are essential for B‐cell differentiation and activation. The correlation of mRNA expression with CNA status in adult HGBCL, NOS demonstrated that losses in *TP53* and *CDKN2A* were marginally associated with low mRNA expression (Figure 2C). Gene expression enrichment analysis (GSEA) of genes with concordant CN gain and associated mRNA expression (upregulated) in adult HGBCL, NOS and pediatric non‐mBL cases (compared with WT‐status cases) demonstrated enrichment for genes promoting proliferation (*MYC* targets), cell cycle regulation (RB1 dependent), B‐cell differentiation and activation (*BCL6* targets up or BLIMP1 dependent, NF‐κB, IRF4 targets) and metabolic reprograming (mTOR, TCA cycle), thus underscoring the metabolic adaptations and increased proliferative capacity of neoplastic cells. Conversely, genes showing CN loss and corresponding decreased/low mRNA expression included genes associated with TGFβ1, MHC class 1, negative regulators of stemness, and plasma cells (Figure 2D).

#### 3.2.2 Pediatric HGBCL, NOS

These cases showed recurrent del17p (*TP53*) and gain12q13 (*STAT6*) at similar frequencies (24% and 15%, respectively) to that of GCB‐DLBCL^24^ suggesting overlapping pathogenic mechanisms (Figure 2B).

### 3.3 Mutational landscape in molecular HGBCL, NOS cases expressing DLBCL signatures

#### 3.3.1 Adult HGBCL, NOS

**FIGURE 3:**
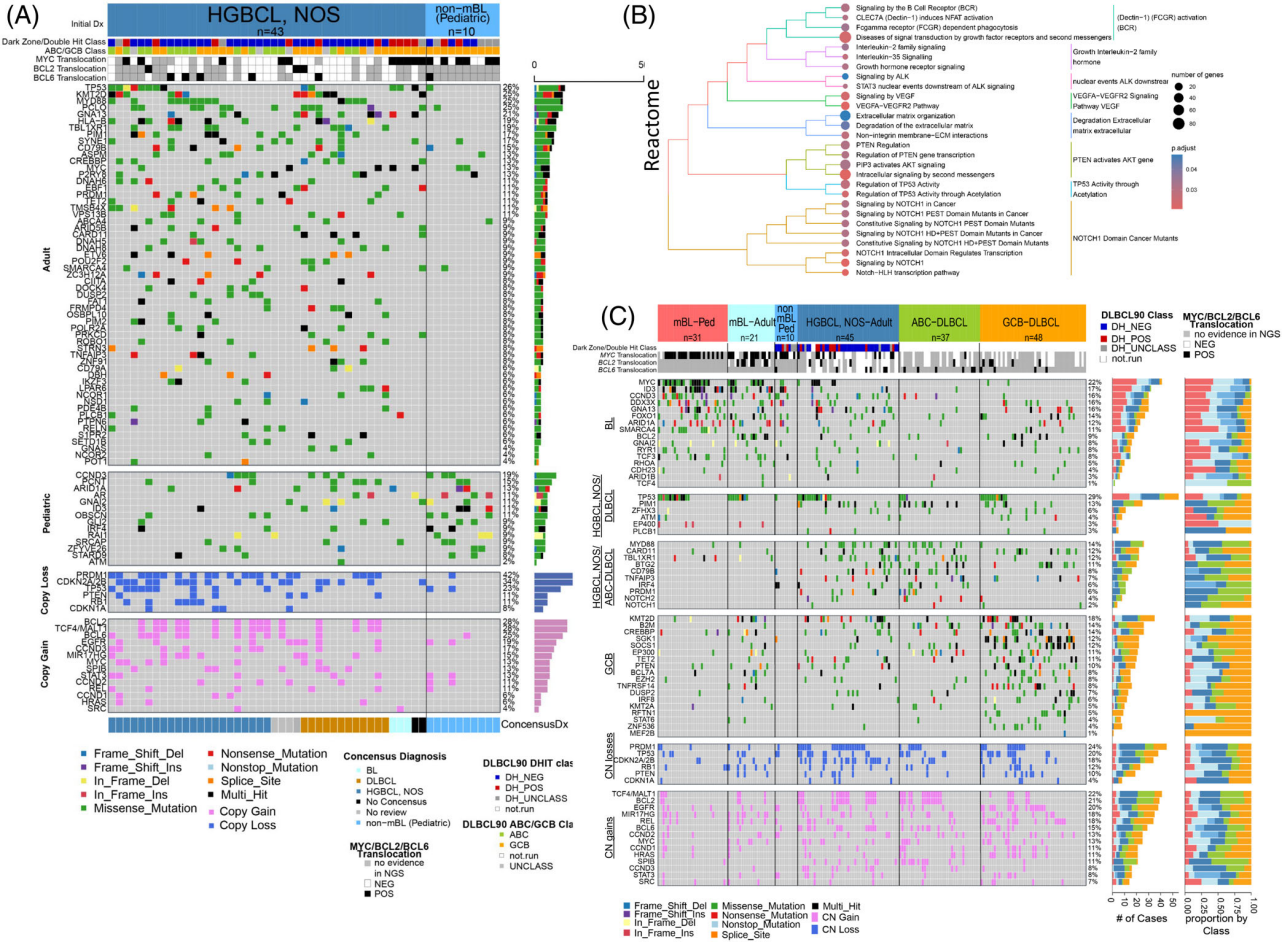
Mutational profiling of HGBCL, NOS, cases with DLBCL signatures. (A) Mutation status of hallmark genes identified by WES in 43 adult HGBCL, NOS, and 10 pediatric non‐molecular BL cases with DLBCL GEP signatures. The panels represent genes common in the adult cases or pediatric cases or genes affected by DNA CN loss or gain. (B) Bubble graph of pathways enriched for mutations in DHL‐signature negative HGBCL, NOS, adult cases. (C) Mutation status of hallmark genes within the noted lymphoma types. *MYC*/*BCL2*/*BCL6* translocation status and gene expression classification are displayed above according to in‐figure key. Right axis corresponds to mutation count and proportion within subtypes. The panels show genes common in (1) BL, (2) HGBCL, NOS, and DLBCL, (3) HGBCL, NOS and ABC‐DLBCL, and (4) GCB‐DLBCL. The lower 2 panels show DNA CN losses and DNA CN gains. [Color figure can be viewed at wileyonlinelibrary.com]

Initial analysis of the WES‐derived mutational landscape indicated an aberrant gene spectrum comparable with *de novo* DLBCL, with frequent mutation (>20%) of *TP53*, *KMT2D*, *MYD88*, *GNA13*, and *PIM1*, highlighting common pathogenic pathways (Figure 3A). *In silico* pathway enrichment analysis of the mutated genes indicated dysregulated B‐cell differentiation (e.g., *PRDM1*, *TBL1XR1*), BCR and B‐cell activation (*CD79A/B*, *CARD11*, *MYD88*, *PIM1*), NOTCH‐ (*NOTCH1/2*), PTEN‐ (*PTEN*) signaling, epigenomic dysregulation (*KMTD2*, *TET2*), cell‐cycle progression (*TP53*), and DNA damage and repair (*P2RY8*) (Figure 3B and Table S2). To validate and compare these findings with *de novo* DLBCL and BL, we used a lymphoma‐targeted custom gene panel from our previous studies.^6^, ^18^ The results showed high concordance between WES and the lymphoma‐targeted panel. The mutation spectrum of HGBCL, NOS closely resembled DLBCL but not BL, except for three genes (*CCND3*, *GNAI3*, *DDX3X*) which were frequently present in HGBCL, NOS (Figure 3C). Adult mBL had frequent mutations in *BCL2* and *MYC*, and have been associated with translocation status,^10^ but these were either not present or infrequent in HGBCL, NOS. *PIM1* mutations were frequent in adult HGBCL, NOS (20%, 9/45 HGBCL, NOS) and comparable with DLBCL subtypes (i.e., 24% in ABC‐DLBCL [9/37] and 15% in GCB‐DLBCL [7/48]), but were not observed in adult mBL. While *TP53* mutations and copy loss were frequently identified in HGBCL, NOS and DLBCL subtypes, they co‐ocurred more frequently with *PIM1* mutations in GCB‐DLBCL than in either HGBCL, NOS or ABC‐DLBCL(Figure 3C). Genes involved in BCR signaling and B‐cell activation (*MYD88*, *CARD11*, *TBL1XR1*, *CD79B*, *TNFAIP3*) or epigenetic regulators (*KMT2D*, *CREBBP*, *TET2*) were observed at frequencies comparable with *de novo* DLBCL subtypes. However, *PRDM1* loss and/or mutations were present in 53% (24/45) of cases, similar to *TP53* loss/mutation (~50%, 23/45).

**FIGURE 4:**
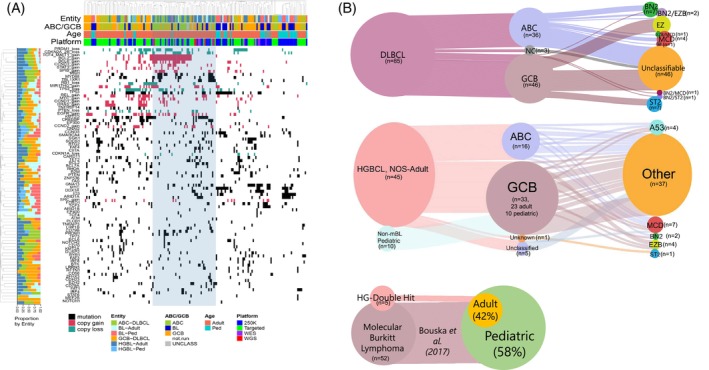
Genetic classifications for HGBCL, NOS, cases with DLBCL signatures. (A) Hierarchical clustering of frequent mutation and CNAs in the noted lymphoma subtypes. (B) Sankey plot of genetic classifications for HGBCL, NOS, cases with DLBCL signatures based on the Lymphgen1.0 classifier.^21^ Nodes represent classifications with the migration of cases represented by connecting lines. Line width is proportional to the number of cases. [Color figure can be viewed at wileyonlinelibrary.com]

We performed hierarchical clustering of the recurrently mutated genes and CNAs identified in HGBCL, NOS, *de novo* DLBCL, and BL. We observed that the HGBCL, NOS (with DLBCL molecular signatures), mainly clustered within the *de novo* DLBCLs (Figure 4A). These clusters were driven by frequent del17p13 (*TP53*), del6q21(*PRDM1*), del9p21(*CDKN2A*), and gain of 18q21(*BCL2*, *TCF4*, *MALT1*) or gain of 3q27 (*BCL6*), and mutations in *PIM1* and *MYD88*.

Furthemore, we examined the re‐classified HGBCL, NOS cases using the newly‐described genetic DLBCL classifier (i.e., LymphGen genetic subtype classifier)^21^, ^25^ and the resultant classification, including the *de novo* DLBCL cohort, is depicted in Figure 4B. As expected, adult HGBCL, NOS, cases were classified largely as “other” (62%, 27/45), and a subset was classified as MCD (15%, 7/45), EZB (8%, 4/45) or A53 subtypes (8%, 4/45). The proportion of “other” observed in the adult cases is consistent with *de novo* DLBCL, as in earlier studies,^21^, ^25^ indicating that HGBCL, NOS, cases are genetically heterogenous.

Pediatric HGBCL, NOS: These cases were associated with GCB‐DLBCL molecular profile and shared mutations in genes commonly associated with BL, including *TCF3*, *ID3*, *CCND3* and *MYC*. (Figure 3C). Hierarchical clustering of pediatric HGBCL, and NOS cases showed that they were interspersed with pediatric BL cases (Figure 4A), driven by the relative lack of CNAs in these samples. When classified using the LymphGen genetic subtype classifier,^21^, ^25^ all pediatric HGBCL, NOS cases were grouped into the ‘other’ category (Figure 4B).

### 3.4 GEP and tumor microenvironment milieu analysis of HGBCL, NOS expressing DLBCL signatures

#### 3.4.1 Adult HGBCL, NOS

**FIGURE 5:**
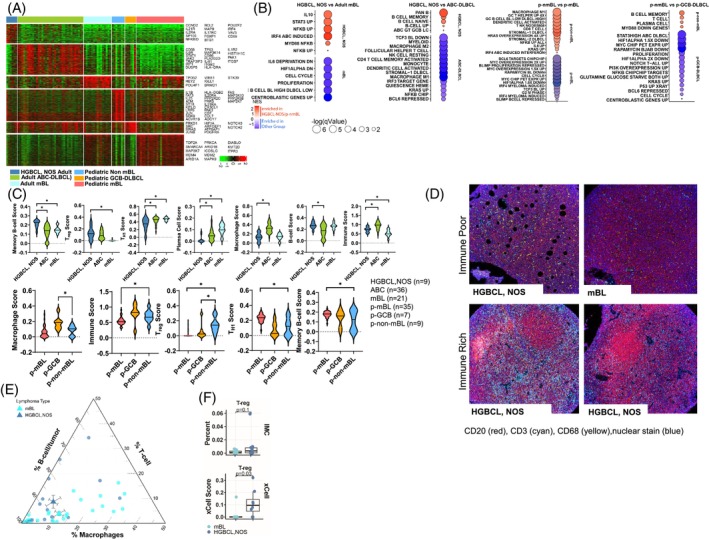
Gene expression analysis of HGBCL, NOS, adult cases. (A) Heatmap of differentially expressed genes between the denoted lymphoma groups. (B) Bubble graph of significantly enriched GSEA pathways in adult HGBCL, NOS as compared with BL and ABC‐DLBCL (left) and pediatric non‐mBL as compared with pediatric BL and pediatric GCB‐DLBCL (right). Bubble size and color correspond to in‐figure‐key. (C) Immune cell enrichment analysis (xCell) for adult HGBCL, NOS, ABC‐DLBCL, and BL (upper panel). Immune cell enrichment analysis (xCell) for pediatric non‐mBL, pediatric GCB‐DLBCL, and pediatric BL (lower panel). Asterisks (*p* < 0.05) denote significant differences between groups. (D) Representative imaging mass cytometry (IMC) images of immune rich and immune poor cases. (E) Ternary plot displaying the percent of B‐cells, T‐cells, and macrophages in HGBCL, NOS, and mBL cases analyzed by IMC. The circles represent individual samples, and the triangles the average values for the groups. (F) Boxplots comparing the percent of regulatory T‐cells (T_reg_ cells) from IMC data in mBL versus HGBCL, NOS, with the T_reg_ score (lower insert) estimated from gene expression data using xCell in the same cases. [Color figure can be viewed at wileyonlinelibrary.com]

As HGBCL, NOS, cases differ in morphological characteristics, transcriptional expression, and genetic classification when compared with DLBCL or BL, we sought to identify the underlying GEP differences among these subsets, representing the overall expression patterns from the neoplastic cell and their corresponding microenvironment. (Figure 5A).

GEP analysis of the reclassified adult HGBCL, NOS in comparison with adult mBL cases, showed enrichment of NF‐κB, MYD88, IRF4, IL10, and STAT3 signaling pathways, whereas the mBL cases were enriched for cell‐cycle, proliferation, and centroblast signatures (Figure 5B). Next, we examined the differential GE in HGBCL, NOS, with the ABC signature compared with *de novo* ABC‐DLBCL and observed an increase in multiple B‐cell signatures, including pan B‐cell, memory B‐cell, and naïve‐B cell signatures. Adult HGBCL, NOS, cases had lower expression of IRF3, KRAS, and NF‐κB‐related signatures, and lower expression of stromal, myeloid, macrophage, and monocyte signatures when compared with *de novo* ABC‐DLBCL (Figure 5B).

We used GEP data to examine the gene expression associated with the TME using xCell^26^ algorithm, and adult HGBCL, NOS had a TME signature enriched for memory B‐cell and regulatory T cell (T_reg_) phenotype, but displayed low expression of T_H1_ cell and plasma cell signature/markers (Figure 5C, top). To validate these findings, spatial proteomic analysis was performed imaging Mass Cytometry (IMC) using a 34‐antibody immune panel for comparative analysis versus mBL (Figure 5D). Integrated analysis of the cell types indicated a similarly high percentage of tumor‐rich B‐cells in HGBCL, NOS, and mBL. HGBCL, NOS had a higher percentage of T‐cells in the TME in contrast with mBL which showed more macrophages (Figure 5E). The increase in T_reg_ cells observed in the GEP data using xCell^26^ estimation was also observed in IMC analysis, although it exhibited a non‐significant trend due to the small sample size (Figure 5F).

#### 3.4.2 Pediatric HGBCL, NOS

GEP analysis of the pediatric non‐mBL cases, which expressed a GCB‐like signature versus pediatric mBL (Figure 5A), showed an expected reduced enrichment for BL‐related pathways (cell‐cycle‐*MYC*‐related), but displayed enrichment for signatures associated with IL‐6 signaling, DLBCL‐related stroma, KRAS, and NF‐κB (Figure 5B). Comparison of pediatric non‐mBL cases with the GCB‐signature against a pediatric GCB‐DLBCL cohort showed enrichment in memory B‐cell and plasma cell signatures, and lower expression of cell‐cycle, PI3K, STAT3, and *MYC* signatures (Figure 5B). The immune signature analysis in pediatric non‐mBL cases versus pediatric GCB‐DLBCL also demonstrated differences in macrophage and T_reg_ cell enrichment (Figure 5C, lower). Compared with mBL, the immune signature analysis^27^ of the TME for the pediatric non‐mBL cases revealed elevated immune and T_reg_ signatures and lower expression of the T_H1_ and memory B‐cell scores (Figure 5C, lower).

### 3.5 *PIM1* gain of function mutation as a rational target in HGBCL, NOS expressing DLBCL signatures

Adult HGBCL, NOS patients have a poor prognosis compared with *de novo* DLBCL, and have frequent *PIM1* mutations, which are not seen in HBGCL, NOS, with a BL signature.^6^ *PIM1* mutations are prognostic in DLBCL,^28^ associated with the clinically aggressive MCD genetic subtype^6^, ^25^ frequent in ABC‐DLBCL, and associated with active BCR signaling (*MYD88* ^*mut*^, *CD79B* ^*mut*^, gain of *SPIB*, gain of *REL*), cell‐cycle regulation (del‐*CDKN2A*, del‐*PRDM1*, *ZFHX3*) and apoptosis (gain‐*BCL2*).^18^ *PIM1* mutations affect the kinase domain, leading to stability and sustained kinase activity that promotes NF‐κB signaling.^29^ GEP analysis of cases with *PIM1* ^mutant^ versus *PIM1* ^WT^ in HGBCL, NOS, ABC‐DLBCL, and GCB‐DLBCL were compared, and the consensus of upregulated genes showed enrichment of genes related to *MYC* and NF‐κB‐related pathways, whereas downregulated pathways included genes involved in KRAS signaling and the p53 responses (Figure S3). Other key upregulated pathways including cell‐cycle and HIF1α, highlighting the roles in tumor proliferation, survival, and aggressiveness (Figure S3).

**FIGURE 6:**
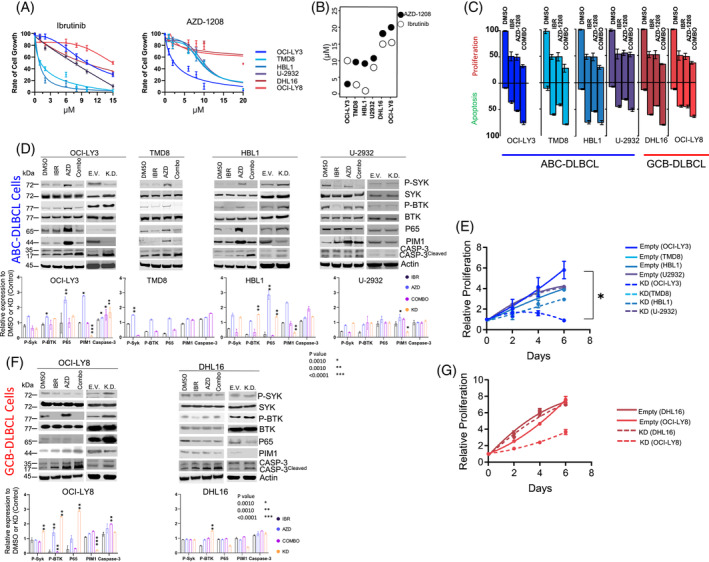
*In vitro* investigation of *PIM1* inhibition in genetically classified DLBCL cell lines. (A) Representative IC_50_ curves for ibrutinib (IBR; BCR inhibitor) and AZD‐1208 (AZD; pan PIM‐inhibitor) in the listed cell lines at 48 h. (B) IC_50_ values for IBR and AZD in the noted cell lines. (C) Cell proliferation/growth or apoptosis relative to vehicle control (DMSO) for single‐ (AZD or IBR) or double‐agent (Combo) treatments for the listed cell lines. Agents were treated at their respective IC_50_ values for 48 h. Error bars represent SEM from three independent experiments. (D) Representative western blots for the indicated targets following drug treatments of the noted ABC‐DLBCL cell lines at the respective IC_50_ values (48 h) or after knockdown of *PIM1*, the bottom panel represents the protein quantification from three independent biological experiments. Vehicle DMSO (D), ibrutinib (I), AZD‐1208 (A) and combination treatment (C), empty vector (EV), knockdown (KD). Phosphorylation of SYK was probed at Tyr525/526 (P‐SYK) and BTK at Tyr223 (P‐BTK). CASP‐3; caspase (E) Proliferation (normalized to day 1) for the listed ABC‐DLBCL cells lines. Error bars represent SEM from three independent experiments. Asterisks (*p* < 0.05) denote significant differences between groups. (F) Representative western blots for the indicated targets following drug treatments of the noted GCB‐DLBCL cell lines at the respective IC_50_ values (48 h) or after knockdown of *PIM1*. The bottom panel represents the protein quantification from three independent biological experiments. (G) Proliferation (normalized to Day 1) for the listed GCB‐DLBCL cells lines. Error bars represent SEM from three independent experiments. Asterisks (*p* < 0.05) denote significant differences between groups. [Color figure can be viewed at wileyonlinelibrary.com]

To further delineate the role of *PIM1*, we tested *PIM1* inhibition *in vitro* using pharmacologic and genetic approaches using *PIM1* ^mutant^ ABC‐DLBCL (OCI‐Ly3, TMD8, HBL1), GCB‐DLBCL (OCI‐Ly8) and a *PIM1* ^WT^ ABC‐DLBCL cell line (U‐2932) and GCB (DHL16) cell line (Table S3). Of these, the OCI‐Ly3 cell line shared a similar genetic profile to that of HGBCL, NOS, expressing the ABC‐DLBCL signature described herein (i.e., *PIM1* ^*mut*^, *CDKN2A/B* ^*−/−*^, *TP53* ^*−/−*^, *MYD88* ^*mut*^). As shown here or noted earlier,^30^ OCI‐Ly3 cells were resistant to ibrutinib but highly sensitive to *PIM1* inhibition with AZD1208 (Figure 6A). In contrast, the HBL1 and TMD8 cell lines (*MYD88* ^*mut*^, *CD79B* ^*mut*^, *PIM1* ^*mut*^ or *PIM1* ^*+/*−^) were sensitive to ibrutinib (see IC_50_, Figure 6B). In agreement with the literature,^11^ the IC_50_ values for ibrutinib in HBL‐1 and TMD8 cell lines are significantly lower, often reported in the low nanomolar range. Specifically, studies indicate that the IC_50_ for ibrutinib in HBL‐1 cells is approximately 0.001 μM (1 nM), and similarly low in TMD8 cells.^31^ The GCB‐DLBCL cell lines (OCI‐Ly8 and DHL16) were resistant to the *PIM1* inhibitor, AZD1208 (IC_50_ ~ 20 μM), and showed a moderate response to ibrutinib.

We explored combined inhibitor treatment (at their IC_50_), which resulted in an additive decrease in cell growth/proliferation in three of the four ABC‐DLBCL cell lines, suggesting a positive association, and observed increased apoptosis post‐48 h following combination treatment compared with single agent (Figure 6C).

Next, we examined BCR and NF‐κB signaling following pharmacologic *PIM1* inhibition with or without BCR induction (i.e., +/‐ α‐IgM stimulation), and ibrutinib treatment was used for comparative analysis. To our surprise, *PIM1* inhibition with AZD‐1208 led to enhanced NF‐κB activation, as estimated by phosphorylation of p65 (RELA), and potentiated BCR activation, as estimated by downstream kinases (pSYK^Tyr525/526^ and pBTK^Tyr223^), in three ABC‐DLBCL cell lines with *PIM1* mutation. This was much more prominent upon BCR activation (Figures 6D and S4). To confirm these findings, we performed *PIM1* knockdown (KD) using shRNA, which reduced *PIM1* protein expression to varying levels in the above cell lines, except for TMD8 cells, which failed *PIM1* KD. Consistent with the pharmacological inhibition of *PIM1*, upon *PIM1* KD in ABC cell lines, OCI‐LY3 cells showed the most significant growth inhibition. HBL1 and TMD8 cell lines also exhibited poor growth upon *PIM1* KD, while the *PIM1* ^WT^ U‐2932 cell line showed minimal effects on proliferation (Figure 6E). We observed higher p65 levels as a measure of active NF‐κB pathway, and higher apoptosis estimated by cleaved caspase‐3 in *PIM* ^mut^ ABC‐DLBCL lines (OCI‐LY3, HBL1). In contrast, higher BCR signaling (measured by pSYK and pBTK) was only observed upon KD in HBL1, but not in the other ABC‐DLBCL cell lines (Figure 6D).

The GCB‐DLBCL cell line, OCI‐Ly8 showed an increase in p‐BTK^Tyr223^ upon AZD‐1208 treatment without BCR stimulation (Figure 6F; left panel), but *PIM1* ^WT^ ABC‐DLBCL (U‐2932) and GCB‐DLBCL (DHL16) cell lines showed no noticeable effect following AZD‐1208 treatment, suggesting that *PIM1* inhibition exerts a role in BCR or downstream NF‐κB signaling (Figure 6F; right panel). The effect was reduced by ibrutinib to varying degrees in the three ABC‐DLBCL cell lines tested. We observed a similar effect in the GCB‐DLBCL cell line (OCI‐Ly8) with *PIM1* ^mut^ upon *PIM1* KD, which increased pSYK, pBTK, and p65 expression. No appreciable change was observed in the *PIM1* ^WT^ DHL16 or U‐2932 cells. Upon KD of *PIM1*, the GCB‐DLBCL cell lines also showed reduced proliferation, with significant effects observed in the OCI‐LY8 cell line, while the DHL16 cell line, wild‐type for *PIM1*, exhibited minimal impact (Figure 6G). To enhance experimental rigor, we performed CRISPR/Cas9‐induced *PIM* deletion in OCI‐Ly3 cells and validated increased NF‐κB signaling and cell apoptosis (Figure S5). Overall, these *in vitro* experiments indicate that cell activation via B‐cell survival and NF‐κB signaling are regulated by *PIM1*, supporting the therapeutic targeting *PIM1* in HGBCL, NOS.

## 4 DISCUSSION

HGBCL, NOS included in the 2022 WHO lymphoma classification,^4^ encompasses a diverse group of aggressive lymphomas with several prior designations (e.g., SNC, BLL, or HGBCL‐unclassifiable)^3^ and includes diagnostically challenging cases with features intermediately between DLBCL and BL.^3^ HGBCL, NOS is a diagnosis of exclusion, and the classification of these cases is not very robust, with variable percentages of cases reclassified as DLBCL by expert panels. We performed an extensive genomic/molecular analysis trying to identify unique biological entities within this category. Earlier, we characterized HGBCL cases in adults that had a BL gene expression signature and included DHIT or miR17~92 dependent transcriptional program.^6^ DHL cases represent a distinct molecular subtype with some features overlapping with GCB‐DLBCL,^12^, ^13^ and the oncogenic transcriptome is overwhelmingly regulated by *MYC* overexpression, resulting in shared transcriptomic features with BL. However, the remaining cases did not have a BL‐like transcriptomic profile nor DHL characteristics and, instead, had DLBCL‐like molecular features. Four out of ten pediatric cases initially diagnosed as BLL or atypical BL were confirmed to lack *MYC* translocations, with only those harboring *MYC* translocations exhibiting recurrent *MYC* mutations (Figure S6). While our current analysis supports the molecular distinction of these cases from classical BL, the potential presence of cryptic *MYC* translocations, although rare, may warrant future investigations using whole genome sequencing. Herein, we show that HGBCL, NOS expressing DLBCL signatures are clinically aggressive despite a lack of DHL characteristics, consistent with earlier studies.^32^, ^33^, ^34^ This aggressive presentation is likely due to higher genomic instability (del/mutation *TP53* and/or del‐*CDKN2A*) resulting in therapeutic resistance.^32^, ^35^ HGBCL, NOS expressing DLBCL signatures demonstrated higher genomic complexity compared with BL, but comparable with *de novo* DLBCL. The BL genomic profile is dominated by *MYC* transcriptional programs and may require fewer further genetic alterations during lymphomagenesis,^9^ whereas DLBCL display a more aberrant genome for oncogenic transformation.

Within our series, distinct differences between adult and pediatric HGBCL, NOS cases were apparent.^6^ Adult cases were classified into both ABC‐, and GCB‐DLBCL subtypes (~40% and 60%, respectively), whereas the pediatric cases were classified as GCB‐DLBCL, but shared some mutations commonly observed in BL. The pediatric cases had del17p (*TP53*) and gain of 12q13 (*STAT6*) at a similar frequency (~20%) to that of GCB‐DLBCL. Adult HGBCL, NOS exhibited higher genomic complexity, like DLBCL, featuring aberrations in key tumor suppressor genes (TSGs) such as *PRDM1* (25%), *TP53* (24%), *CDKN2A* (33%), and *RB1* (30%), as well as gains in genes with oncogenic function (*REL*, *BCL2*, *TCF4*, *MALT1*). Adult HGBCL, NOS, also displayed gene mutations regulating the cell cycle, BCR/B‐cell activation, and the epigenome comparable with DLBCL. Notably, mutations in *CCND3*, *DDX3A*, and *GNAI3*, were frequently observed in BL but not in DLBCL, highlighting the genetic heterogeneity within this patient cohort. Genetic subclassification was examined using the LymphGen classification^21^ in this cohort of HGBCL, NOS, cases. Notably, most cases displayed genomic heterogeneity, as they were primarily classified as “other”, whereas a subset exhibited MCD or EZB subtypes, aligning with ABC‐ and GCB‐DLBCL genetic subtypes, respectively. In contrast, pediatric cases were genetically unclassifiable, showcasing a mutational spectrum distinct from *de novo* DLBCL. We observed losses in TSGs (*TP53*, *CDKN2A*, *RB1*), mutations in *PIM1*, and dysregulation of plasmacytic cell differentiation, with *PRDM1* loss and an IRF4‐mediated signature in adult HGBCL, NOS. While the mutational spectrum of the adult HGBCL, NOS cases aligns more closely with ABC‐DLBCL, the GEP indicates biological heterogeneity and further studies with large cohorts are essential to delineate the biological differences or to better understand their clinical implications.

Our integrative analysis of GEP, CN, and mutation data indicated perturbed signaling pathways related to B‐cell differentiation and aberrant BCR signaling, concurrently with aberrations in cell cycle regulators. These findings highlight a significant overlap of these cases with DLBCL subtypes, including GCB‐ or ABC‐like GEP signatures and higher genomic complexity. However, mutations in *CCND3*, *GNA13*, and *DDX3X*, often observed in BL, indicate genetic heterogeneity in some of these cases. Our study further reveals a distinct immune landscape in HGBCL, NOS, which is characterized by increased T_regs_ and memory B cells, and decreased T_H_1 cells and plasma cells. Understanding these immune signatures is crucial for developing targeted therapies and immunotherapies tailored to HGBCL, NOS.

The prognostic significance of *PIM1* mutation or expression observed in earlier B‐cell lymphoma studies is due to its association with ABC‐DLBCL,^6^, ^18^, ^25^ wherein *PIM1* mutations are thought to stabilize its nuclear expression resulting in a gain‐of‐function.^32^ *PIM1* mutations were frequent in adult HGBCL, NOS, and comparable with ABC‐DLBCL with concurrent aberrations in BCR signaling and cell‐cycle regulators. The BCR delivers critical signals to promote B‐cell survival and growth.^36^ However, *PIM1* mutations affect the kinase domain leading to increased stability and prolonged kinase activity culminating in enhanced NF‐κB signaling,^29^ a major downstream effector of BCR pathway activation.^36^ Our study reveals that *PIM1* mutations in HGBCL, NOS, enhance NF‐κB signaling by sustaining kinase activity and promoting phosphorylation of the NF‐κB p65 subunit. This increases NF‐κB activity, upregulating genes that promote cell survival and proliferation. *PIM1* mutations also amplify BCR signaling‐induced NF‐κB activation. To investigate *PIM1*'s relevance in B‐cell activation, *PIM1* was inhibited via pharmacologic or genetic ablation in ABC‐ or GCB‐DLBCL cell lines, including those genetically classified as MCD B‐cell lines, with or without BCR stimulation (i.e., +/− anti‐IgM treatment). Indeed, we identified an unrecognized role of *PIM1* in B‐cell activation and apoptosis, a process important in the negative selection of autoreactive BCRs during B‐cell maturation.^37^, ^38^ As anticipated, upon B‐cell activation in MCD B‐cell lines (OC1‐Ly3, TMD8, HBL1), we observed enhanced NF‐κB signaling and cell proliferation. This was inhibited by ibrutinib, leading to apoptosis or cell‐cycle arrest in cells harboring *MYD88* or *CD79B* mutations (i.e., TMD8, HBL1) consistent with earlier studies.^30^ Currently, ibrutinib is approved for several B‐cell malignancies,^39^ and is active for ABC‐DLBCL with *MYD88*/*CD79A/B* mutations.^11^ *PIM1* mutations have also been associated with intrinsic ibrutinib resistance in ABC‐DLBCL, but combination with *PIM1* inhibitors can circumvent the resistance.^30^ The pharmacologic, or genetic targeting of *PIM1* in the MCD B‐cell lines, resulted in enhanced BCR➔NF‐κB signaling and caspase 3‐induced apoptosis. The role of *PIM1* in BCR‐ligation‐mediated apoptosis is quite intriguing, as malignant B‐cells are more sensitive to BCR‐directed monoclonal antibody killing.^40^ Thus, pharmacologic activation of BCR‐mediated cell death pathways may prove to be therapeutically useful, in addition to targeting pro‐survival BCR signaling, particularly in patients who are resistant to ibrutinib.

In conclusion, this study has shown diagnostic and clinical importance in molecularly categorizing adult HGBCL, NOS cases into DLBCL (~64%) and mBL (~36%) subtypes.^6^ While adult HGBCL, NOS harboring the mBL signature are characterized by a *MYC*‐ or miR17~92‐dependent transcriptional program with simultaneous loss of *TP53*, and the *in vitro* inhibitor studies showed sensitivity to ibrutinib.^6^ These mechanisms are distinct from what is observed in DHL (or triple hit) cases, which can be defined by a temporal sequence of events with the t(14;18) as the first hit, followed by subsequent t(8;14) or dual translocations in *MYC* and *BCL6* in the germinal center.^35^ Our current study revealed that HGBCL, NOS expressing DLBCL‐like signatures have higher genomic aberrations and are characterized by deletion of well‐characterized TSGs (e.g., *PRDM1*, *CDKN2A*, *TP53*, *RB1*), and recurrent mutations in *MYD88* and *PIM1* and may be therapeutically vulnerable to BCR and/or *PIM1* inhibition as rational therapeutic approach. Future studies with *PIM1* inhibition could be efficacious even in *de novo* DLBCL harboring *PIM1* mutations. Further mechanistic studies exploring the role of *PIM1* in B‐cell activation and survival are warranted. One study limitation is the lack of complete or standardized therapies for the current cohort, as these patients have been treated with several intensive immunochemo therapy regimens across different institutions. We believe that future clinical trials may include a biology‐based treatment approach and may benefit from combining R‐CHOP therapy with BCR and *PIM1* inhibitors. Additionally, *PIM1* inhibition may prove effective even in de novo DLBCL cases harboring *PIM1* mutations. Further mechanistic studies exploring the role of *PIM1* in B‐cell activation and survival are warranted, which could open new avenues for targeted therapies.

## AUTHOR CONTRIBUTIONS

W. L., T. A. H., A. B., W. C. C., M. G. and J. I. designed and performed the research. W. L., T. A. H., A. B., X. Z., T. B. H., C. B., and K. H. collected, analyzed/and or interpreted the data. C. A., D. E., M. S., B. J. D., T. C. G., J. V., D. D. W., T. W. M., K. F., and M. G. provided materials, conducted the pathological review, and/or contributed clinical data. D. E. provided input for the *in vitro* experiments, data interpretation and assisted in the review of the manuscript along with the co‐authors. T. A. H., A. B., W. L., and J. I. wrote the manuscript; all authors edited and approved the manuscript.

## CONFLICT OF INTEREST STATEMENT

The authors declare no competing financial interests.

## Supporting information


**Data S1.** Figures.

**Data S2.** Supporting Information.

**Data S3.** Tables.

## Data Availability

Gene expression or any other relevant data can be requested by emailing the corresponding author.
