# Supplementary material for: High‐grade B‐cell lymphoma not otherwise specified, with diffuse large B‐cell lymphoma gene expression signatures: Genomic analysis and potential therapeutics
Source: Am J Hematol. 2024 Nov 16;100(1):10–22. doi: 10.1002/ajh.27513 (PMC11625982; doi:10.1002/ajh.27513)
Supplement: Supplementary file 1 — Data S1. Figures. [file AJH-100-10-s002.pdf]

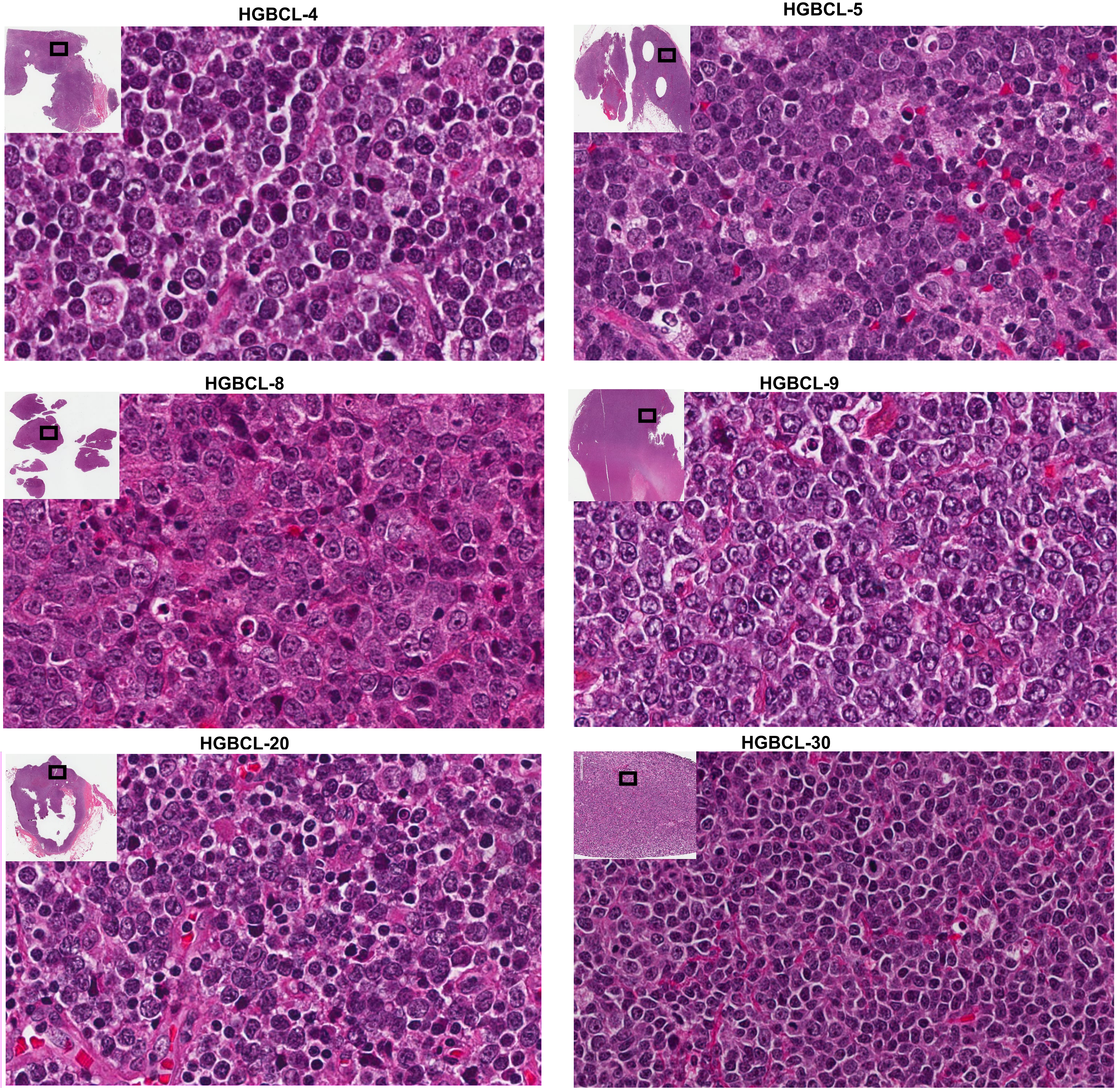

Figure S1: Representative H&E Images of Cases Diagnosed as HGBCL, NOS

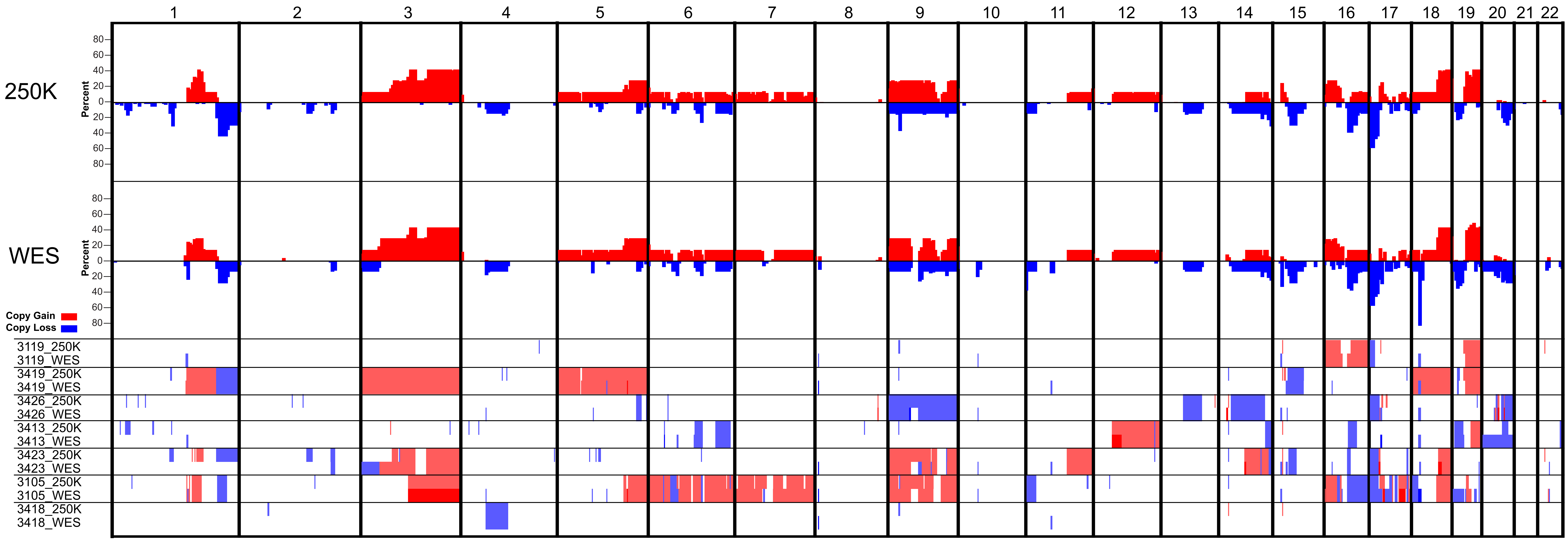

Figure S2. Comparison of copy number alterations detected using the Affymetrix 250K SNP array and by whole exome sequencing (WES) in adult HGBCL, NOS cases profiled by both. The frequency plots for the group is shown above while the individual segmentation for each case comparing the 2 methods is show in the lower panel. Red indicates copy gain and blue loss.

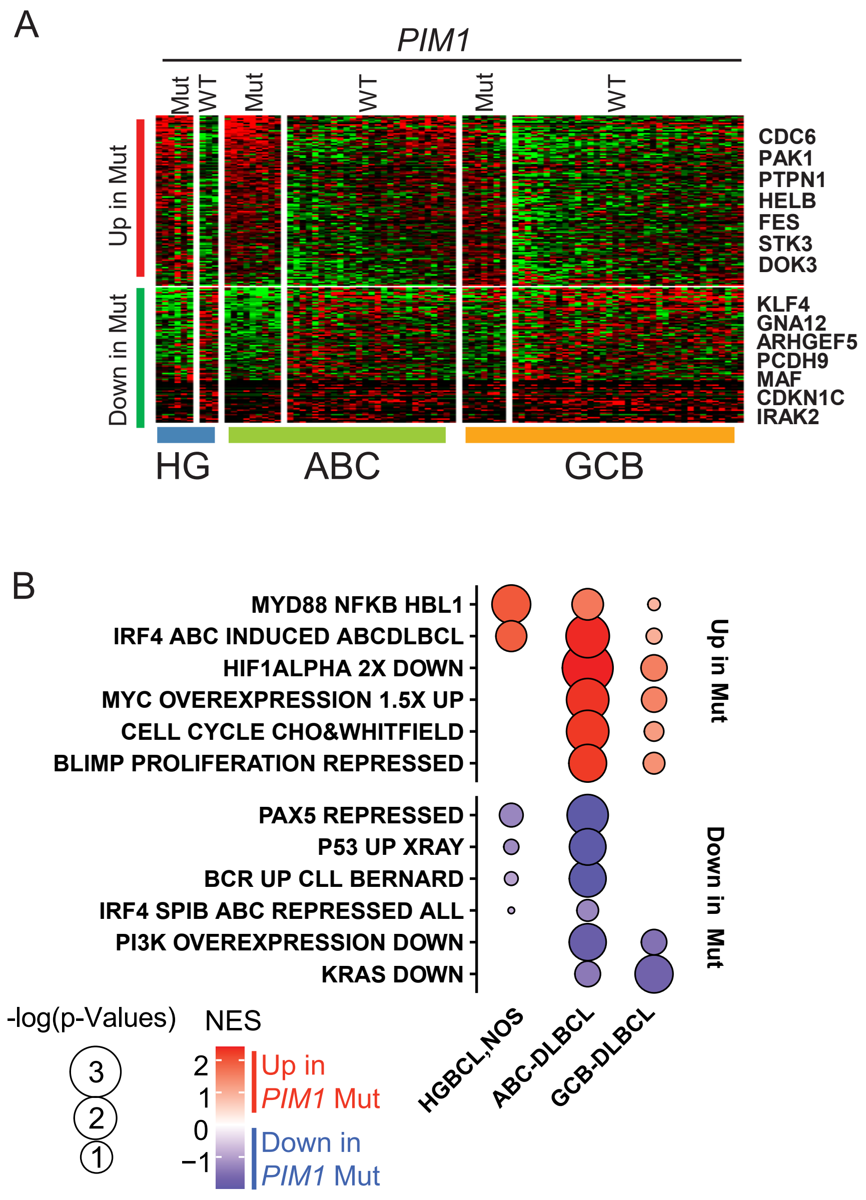

Figure S3. A. Heat map of genes differentially expressed by PIM1 mutation status in HGBCL, NOS, ABC-DLBCL, and GCB-DLBCL cases. B. Bubble plot comparing the pathway enrichment in PIM1 mutant comparted to PIM1 WT cases in HGBCL, NOS, ABC-DLBCL, and GCB-DLBCL

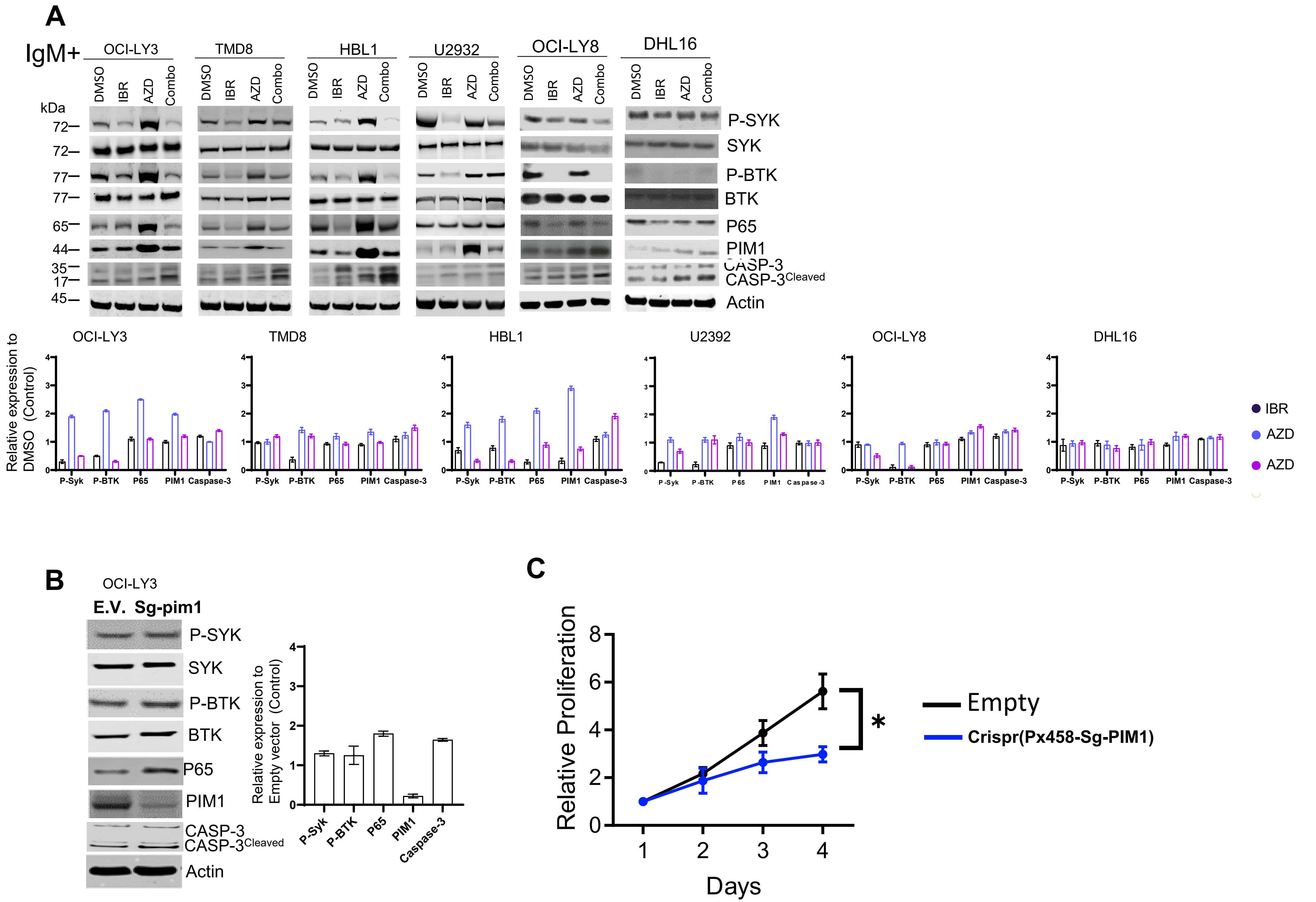

Figure S4. A. Representative Western blots for the indicated targets following drug treatments of the noted ABC cell lines at the respective IC50 values (48 h) with IgM stimula-tion. DMSO (D), ibrutinib (I), AZD-1208 (A) and combination treatment (C), empty vector (E.V.), knock-down (K.D.). Phosphorylation of SYK was probed at Tyr525/526 (p-SYK) and BTK at Tyr223 (p-BTK). CASP3; caspase.
B. Western blot comparing expression of the noted proteins in control (E.V) or *PIM1* knock-out OCI-LY3 . C.Proliferation in control and *PIM1* knockout OCI-LY3 cells.

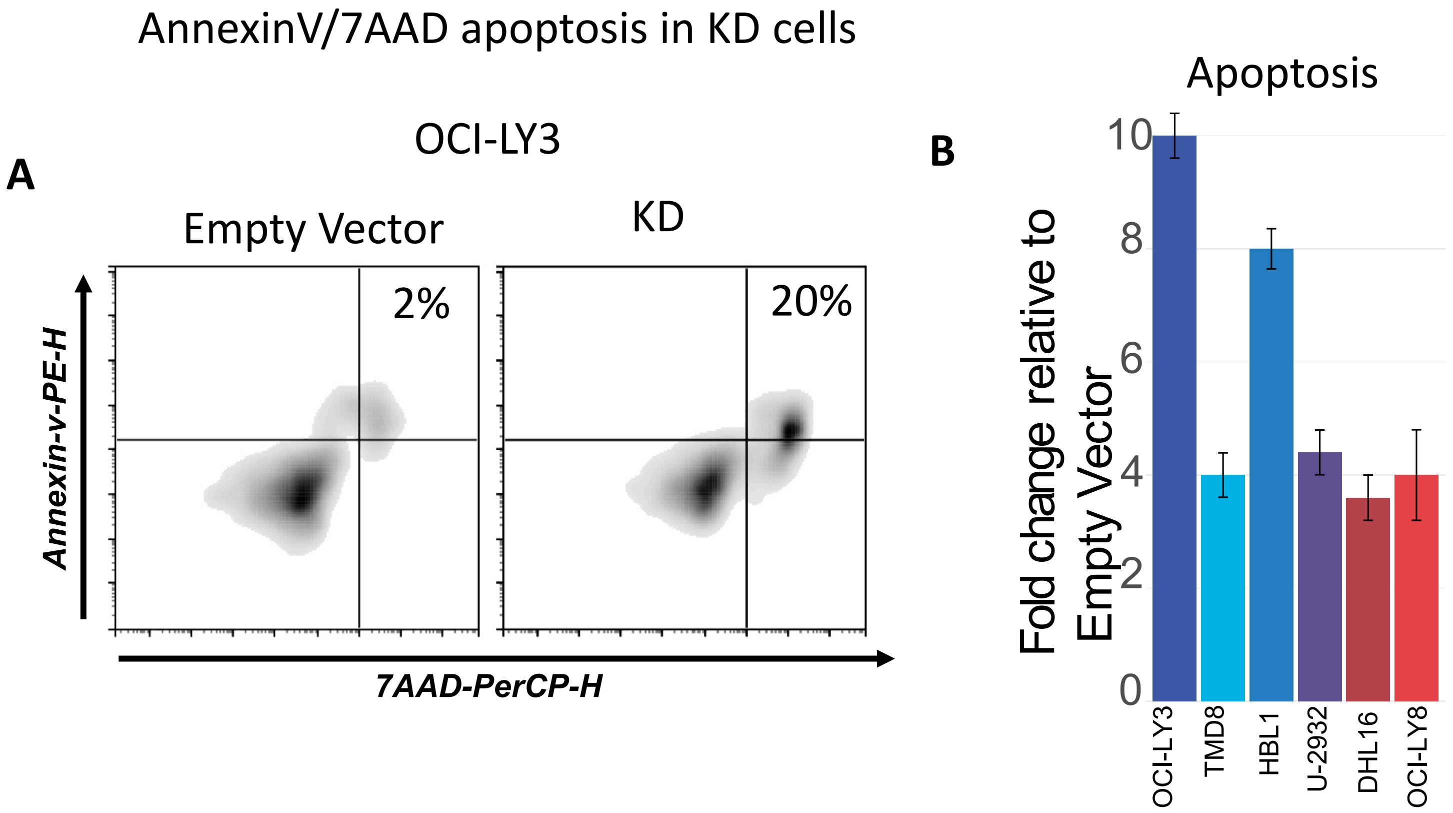

Figure S5. Apoptosis upon *PIM1* knock-down. A. Representative Flow cytometry contour plot of AnnexinV/7AAD staining in OCI-LY3 cells in control and *PIM1* knock-down cells. B. Bar plot depicting fold change in apoptosis upon *PIM1* knock-down in the noted cell lines.

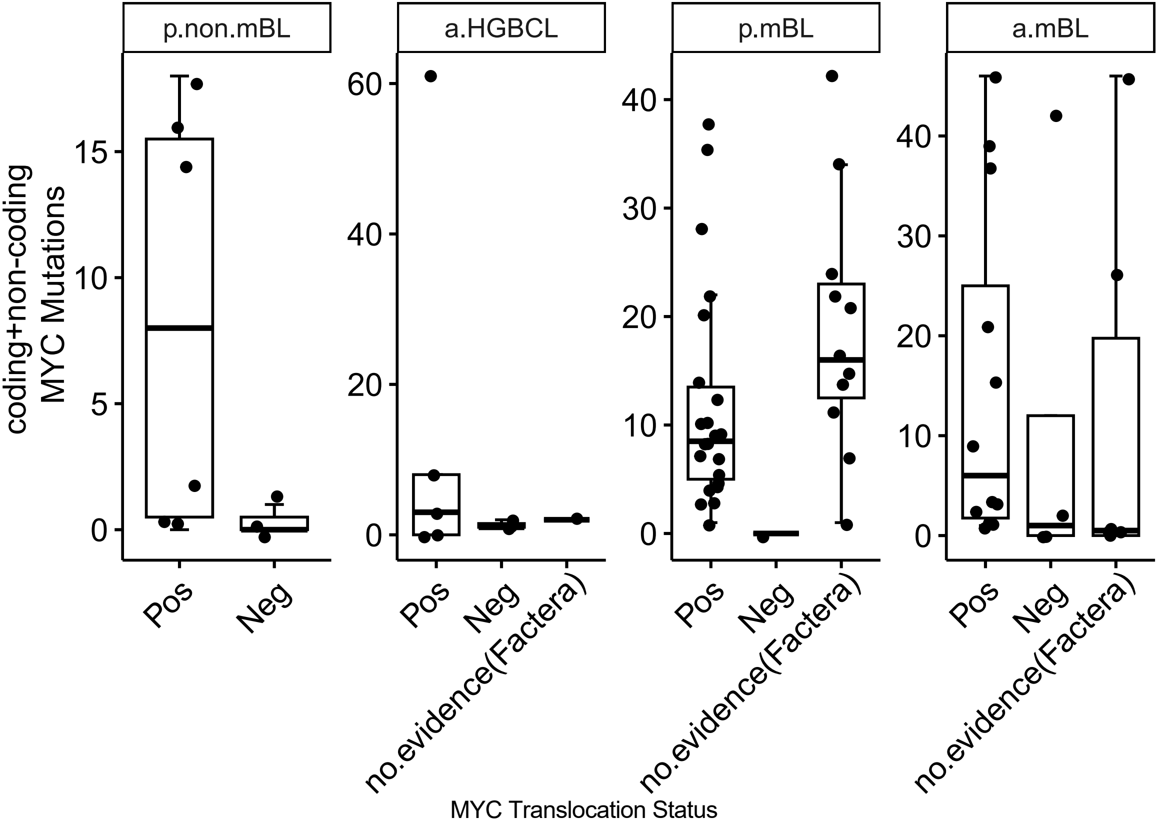

Supplemental Figure 6: Analysis of MYC mutations in coding and non-coding regions of pediatric non-molecular BL (non-mBL) cases. Cases without MYC translocations exhibited an absence of MYC mutations, while those with MYC translocations frequen[illegible] harbored mutations in MYC. This supports the role of AID-mediated mutations in MYC and highlights the absence of alternative MYC translocations in these pediatric non-mBL cases
