# Supplementary material for: High‐grade B‐cell lymphoma not otherwise specified, with diffuse large B‐cell lymphoma gene expression signatures: Genomic analysis and potential therapeutics
Source: Am J Hematol. 2024 Nov 16;100(1):10–22. doi: 10.1002/ajh.27513 (PMC11625982; doi:10.1002/ajh.27513)
Supplement: Supplementary file 2 — Data S2. Supporting Information. [file AJH-100-10-s003.docx]

**Supplementary Material**

**Materials and Methods**

***Patient samples and B-cell lines:***

The clinical and pathological characteristics of HGBCL, NOS patients are provided (***Table-S1***). The clinical and pathological characteristics of BL^1,2^ and DLBCL^3,4^ used for comparison are described in their respective manuscripts. The pathology review, immunohistochemistry, and cytogenetic evaluation of HGBCL, NOS was reported in an earlier study^5^. The GEP molecular diagnoses were performed using the Dave *et al.* or Lenz *et al.* gene signature for BL and DLBCL subgrouping simultaneously^6^ and/or the DLBCL90 nCounter classifier^7^. All HGBCL-NOS cases profiled by WES were analyzed by the DLBCL90 classifier for consistency. Since cases were assembled from several institutes, upon molecular classification, cases were further re-reviewed for consensus diagnosis (DDW, WCC, KF, JS, or Lymphoma/Leukemia Molecular Profiling Project (LLMPP)). The study was approved by the Institutional Review Board of the University of Nebraska Medical Center. Genomic data from previously published BL and DLBCL series^1,8^ were included for comparative analysis.

The study was approved by the Institutional Review Board of the University of Nebraska Medical Center. Genomic data from previously published BL^1^,^2^ and DLBCL^3,4^ series were included for comparative analysis.

Four DLBCL cell lines (TMD8, HBL1, U2392, DHL16) were cultured in RPMI 1640 (Hyclone^TM^-RPMI 1640 with 2.05 mM L-Glutamine), whereas OCI-LY3 and OCI-LY8 were cultured in IMDM (Lonza Biowhittaker) and supplemented with 10% fetal bovine serum (FBS), penicillin G (100 U/ml) and streptomycin (100 μg/mL) and maintained at 37 ºC in 5%CO_2_.

***Cell Culture and Treatment with AZD-1208 and Ibrutinib:***

The DLBCL cell lines used in this study (OCI-LY3, TMD8, HBL-1, U2932, OCI-Ly8, DHL16) were cultured in RPMI-1640 or IMDM media, supplemented with 10% FBS and 1% penicillin/streptomycin. Cells were maintained at 37°C in a humidified incubator with 5% CO₂. The expression profiles, mutation status, and resistance to ibrutinib and AZD 1208 are presented in **Supplemental Table 3**. Cell viability assays were conducted to determine the half-maximal inhibitory concentration (IC50) of Ibrutinib and AZD1208 in both ABC and GCB cell lines. AZD1208, a potent ATP-competitive inhibitor of all three Pim kinase isoforms, and Ibrutinib, a selective inhibitor of Bruton's tyrosine kinase (BTK), were used for treatment. Cells were exposed to varying concentrations of Ibrutinib (0.0001 µM to 10 µM) or AZD1208 (0.01 µM to 10 µM) for 72 hours. IC_50_ values were determined using cell viability curves generated with Presto Blue™ cell viability reagent, following the manufacturer’s protocol (Invitrogen Inc). For combination treatments, cells were treated with both Ibrutinib and AZD1208 at their respective IC50 concentrations.

***Pathological Criteria for the Diagnosis of HGBCL, NOS:***

All HGBCL, NOS cases underwent extensive evaluation by hematopathologists (DDW, WCC, KF, JS or Lymphoma/Leukemia Molecular Profiling Project (LLMPP)), with any diagnostic discrepancies resolved by consensus. Morphological assessment centered on identifying high-grade cytological features, including large neoplastic cells with vesicular chromatin, prominent nucleoli, and frequent mitoses, differentiating these cases from conventional DLBCL. Immunophenotypic profiling was conducted via IHC for B-cell markers (CD20, CD19) and germinal center markers (BCL6, CD10), while a Ki-67 index exceeding 70% confirmed heightened proliferative activity. FISH was performed to exclude MYC, BCL2, and/or BCL6 rearrangements, and only cases without these aberrations were designated as HGBCL, NOS. Burkitt lymphoma was ruled out based on morphology and the absence of MYC rearrangement, and cases fitting the profile of DLBCL without high-grade features were excluded.

To further substantiate the diagnostic criteria, we evaluated MYC mutations in both coding and non-coding regions of these pediatric non-molecular BL cases (Supplemental Figure 6). Our findings demonstrated that pediatric non-mBL cases without MYC translocations also lacked MYC mutations, whereas those with MYC translocations often harbored MYC mutations. This observation aligns with the well-established role of MYC as a gene frequently mutated by AID, providing further evidence that these cases do not carry alternative MYC translocations.

***DNA/RNA isolation and library preparation:***

For formalin-fixed-paraffin-embedded (FFPE) HGBCL, NOS (n=36) samples, total DNA and RNA were extracted using RNAstorm^TM^/DNAStorm^TM^ Kit (Celldata, #: CD506, CD507) as per manufacturer’s guidelines. 3-6 unstained slides were used for extraction. For fresh-frozen samples, DNA and RNA were extracted using the AllPrep DNA/RNA kit (Qiagen, Catalog# 80204). DNA and RNA quality and quantity were examined using Agilent Tapestation 2200 and gDNA and RNA screen tapes (Catalog#:5067-5365, 5067-5366, 5067-5576, 5067-5577), NanoDrop spectrophotometer (ThermoFisher Scientific), and Qubit fluorometric quantification (Catalog#: Q32851, Q32852). The gDNA was sheared to 250 bp and library prepped using the KAPA DNA HyperPrep kit (Roche Diagnostic). For WES, libraries were hybridized overnight to baits provided in the SureSelect XT HS Human All Exon V7 kit (Agilent Technologies). The captured libraries were amplified and sequenced on Illumina NovaSeq 6000^TM^.

***SNP Array genomic copy number analysis:***

250 ng of gDNA isolated from fresh-frozen tissue was DNA copy number (CN) analysis using the Human Mapping 250K Nsp Array (Affymetrix, Inc) was performed according to the manufacturer’s protocols, and analytical details were described previously^1,9^. Briefly, single-nucleotide polymorphism genotypes and probe-intensity log2 ratios were generated using Genotyping Console 4.1 software (Affymetrix). Segmentation of the log2 ratios was done using the circular binary segmentation^10^ (CBS) within the DNAcopy Bioconductor package. Segments with a CN of ≥ 2.3 were considered gains, while ≥3.6 copies were considered an amplification. Segments with a CN ≤ 1.7 were categorized as CN losses, and CN ≤ 0.35 were considered homozygous losses. 35 HGBCL-NOS and molecular Burkitt cases and 55 pediatric non-molecular Burkitt and molecular Burkitt cases^1^ had SNP array analysis performed. The percent aberrant genome per case was calculated by taking the total size of the aberrant regions divided by the total genome size.

***Whole exome sequencing (WES) and target gene deep sequencing:***

Whole exome/ genome sequencing was done on 43 adults (40 WES, 3 WGS) and 10 pediatric cases. The average WES sequencing depth was 100.1x (range 27-242x). The quality of the raw reads was assessed by FastQC (v 0.11.7) (<https://www.bioinformatics.babraham.ac.uk/projects/fastqc/>) and adapter sequences and poor-quality WES reads were subject to trimming with Trimmomatic (v0.36)^11^. Reads were further mapped to the human genome (hg38) with BWA (0.7.17-r1188), and duplicate reads were marked with Picard (v2.9.0) (https://broadinstitute.github.io/picard/). Variants were called by dual variant calling with VarScan2 (v2.4.4) and GATK Mutect2 (v4.1.8.1)^12-14^. The variants were annotated using Annovar (http://annovar.openbioinformatics.org). Variants retained were supported by at least four reads in the tumor sample, with a minimal variant allele fraction (VAF) of 5%, and with variant reads present on both the plus and minus strand^15^. Variants were further excluded if they satisfied either of the following criteria: 1) existed in the avSNP150NotFlagged database (<http://annovar.openbioinformatics.org/en/latest/user-guide/filter>), 2) were present at >1% in gnomAD non-cancer sample database (<https://gnomad.broadinstitute.org/>), 3) were in a region of segmental duplication (SuperDups, Annovar), 4) did not change protein-coding sequence or affect canonical splice sites, 5) were recurrent in an unrelated set of 91 normal samples. Variants retained were identified by both variant callers if they passed the above criteria in at least 1 variant caller (***Table-S4***).

Targeted sequencing data using a Pan Lymphoma gene panel was available on 25 (16 adult and 9 pediatric) cases. All but 2 of the adult Targeted-sequencing cases were also profiled by WES. The average sequencing depth was 387.6x (range 136.2-795.9x). The targeted DNA sequencing data was from our two earlier studies ^1^,*^8^* using a gene panel of 380 commonly mutated in B-NHLs. The alignment and analysis were performed similarly to the description above and a detailed methodology for variant calling was described in an earlier study^1^. Targeted variants were aligned in hg19 genomic coordinates and thus were lifted over to hg38 for comparison with the WES data. In cases with both WES and targeted sequencing the mutations called by both targeted sequencing compared and >90% of were also called in the WES data, ensuring sufficient coverage depth in WES.

To assess copy number abnormalities in cases that lacked the SNP array data, the targeted WES, and WGS sequencing data were used. Bam files from the targeted data were analyzed by CopyWriteR^16^ with 100kB windows and bam files from WES and WGS were analyzed by CNVkit (https://github.com/etal/cnvkit). Segmentation was done using the circular binary segmentation^10^ (CBS) algorithm and segments with a CN of ≥ 2.3 were considered gains, while ≥3.6 copies were considered an amplification. Segments with a CN ≤ 1.7 were categorized as CN losses, and CN ≤ 0.35 were considered homozygous losses. Cases with SNP array data and CNA by sequencing were compared **(*Figure S2*)** and showed concordant results. For cases with SNP array data was available, that was used for the final analysis.

***Evaluation of MYC, BCL2, and BCL6 translocation status:***

*MYC*, *BCL2,* and *BCL6* translocation status was known in a subset of cases based on clinical cytogenetics data or FISH. If the status was not known the targeted and/or WES data was analyzed by Factera^17^ to look for evidence of MYC, BCL2, or BCL6 translocations as previously described^1^.

***Gene expression analysis:***

Total RNA was isolated for GEP analysis utilizing HGU133 plus 2.0 (Affymetrix Inc) arrays and the detailed analysis of subclassification has been described in earlier studies^18^. The raw data was normalized (MAS5.0) using BRB Array Tools (<http://linus.nci.nih.gov>/BRB-ArrayTools.html). The GEP data from BL cases were used to classify samples as BL or DLBCL using a Bayesian classifier described previously^6^. The classification of the BL *vs.* DLBCL cases was performed using the training cohort from a previous study^6^. Among the 55 HGBCL, NOS cases, 53 were subjected to profiling using nCounter (NanoString, Inc.). Employing 200 nanograms of RNA, we quantified the expression of 90 genes pertinent to the DLBCL90 classifier. This dataset facilitated subclassification, enabling the evaluation of both the double-hit (dHIT) gene expression signature^7^ and the DLBCL subclassification (i.e., ABC versus GCB) within the cohort, by the methodology described by Ennishi et al^7^.

Differential gene expression was identified using BRB Array Tools with t-test and other standard approaches described previously^18^. The *in-silico* pathway and gene signature analysis was performed through GSEA using the lymphoid signature^19^ (http://lymphochip.nih.gov /signaturedb/) and Broad Institute signature databases^20^ (<http://software.broadinstitute.org/>gsea/msigdb). Gene pathway and gene ontology analyses were conducted using Ingenuity Pathway Analysis^21^ (IPA; QIAGEN, Inc). Immune signature analysis was completed using xCell (http://xCell.ucsf.edu/)^22^. We compared the average mRNA expression level of genes by CN status (gain, loss, 2N) using a one-sided Student's t-test to determine significance. IPA (https://www.qiagenbioinformatics.com/products/ingenuity-variant-analysis) or DAVID^23^ were used for the molecular and functional annotation of the genes within aberrant regions.

***Classification of DLBCL samples by genetic subtyping:***

HGBCL, NOS and *de novo* DLBCL samples were submitted for genetic subtype classification using the LymphGen 1.0 portal (https://llmpp.nih.gov/lymphgen/index.php)^24^. CN status was used, and BCL2 and BCL6 status was provided for cases where it was available.

***Tissue Imaging Mass Cytometry, and cell lineage assignments***

Tissue Microarrays (TMAs) were dewaxed in 3 washes of xylene and rehydrated by successive washes in 100-70%, ethanol and immersed in Tris-EDTA antigen retrieval solution for 30 minutes at 95^o^C after the antigen retrieval step the slides were blocked with 3% BSA for 45 minutes and stained overnight with the antibody panel at 4^o^C. The panel included antibodies representing B-cells and tumor environment milieu and other functional biomarkers (Fluidigm Inc.) in conjugated form or were conjugated using MaxPar kits. The slides were then incubated with 191 Iridium, a nuclear stain, for 40 minutes and ablated using the Hyperion/Helios Imaging Mass Cytometry platform (Standard Biotools) at a rate of 200 H, and images were acquired for analysis.

Raw images were generated using MCD Viewer (Standard Biotools). Segmentation of image data was performed using Mesmer^25^. Segmented cell data was processed using Python 3.7 and R 4.2.1. Cells smaller than 5μm^2^ or larger than 400μm^2^ were removed from analysis, and ROIs with fewer than 1000 cells were removed from analysis. Mean cell protein values were transformed by hyperbolic arcs in with a cofactor of 5, censored at the top 1%, scaled, and all clustering steps were performed using Rphenograph with k=15. Phenograph cluster labels were assigned by calculating z-scores of protein expression for each cluster and labeling each cluster with all relevant cell types based on protein expression. Phenograph was performed within each cluster and manual corrections were made. The remaining cells unable to be classified were subjected to a final clustering step and z-scores of cell expression were used to assign cell types, with multiple cell type assignments per cell allowed. UMAPs were generated using the uwot package for all cells as well as cell subtypes using default settings. Statistical tests were performed by measuring cell type proportions for each patient, stratifying patients using Mclust ^26^and using Cox regression analysis or generalized linear models to test for the significance of cell type proportions.

***Survival outcome analysis:***

Overall survival (OS) -death from any cause- was estimated using Kaplan-Meier and differences were assessed using the log-rank test. Statistical analyses were performed with GraphPad Prism (Prism 8.0.2) with P < 0.05 considered significant.

***In vitro functional analysis:***

*Evaluation of BCR signaling:* ABC-DLBCL cell lines were treated with 10 μg/mL anti-IgM (Southern Biotech) to cross-link BCR for 5-10 min, followed by snap freeze in liquid nitrogen. BCR activation was evaluated using Western blots for phospho-SYK (Tyr525/526) and phospho-BTK (Tyr223).

*Apoptosis Assay:* Apoptosis of lentivirus-transduced cells was quantified using a FACSCalibur flow cytometer (BD Biosciences) after staining using the Apoptosis Detection Kit (BD Pharmingen) according to the manufacturer’s instructions.

*Prestoblue Assay:* Cell viability was performed in 96 well plates using PrestoBlue™ Cell Viability Reagent (Invitrogen) per the manufacturer’s protocol.

*PIM1 knockdown using RNA:* Knockdown was achieved using three siRNA (hs.Ri.PIM1.13.1-13.3, IDT) with the TriFECT kit (Company). siRNAs were mixed in buffer “R” and 1E^6^ cells were electroporated using the Neon® Transfection System (Invitrogen). Cells were plated in 1 mL of medium and were grown for 48 h in 12 well plates. Cells were collected 48 h later for downstream analyses.

*PIM1 knockout using CRISPR:* Cell lines were CRISPR-edited using the lentiCRISPR v2 construct (Addgene #52961) and two guide RNAs (# SC1678, GenScript). Lentivirus was grown in 293T cells transduced using the calcium phosphate method. The virus was collected at 48 and 72 h, precipitated using the PEG Virus Precipitation Kit (Abcam), and immediately utilized for cell line transfection. Cells were spinoculated (350xg, 1 h) twice and then selected for 7 days in puromycin (1 μg/ml).

*Western blot:* Whole-cell extract (50 ng) was resolved using 10% sodium dodecyl sulfate-polyacrylamide gel electrophoresis and transferred to a polyvinylidene difluoride membrane and incubated in blocking buffer at room temperature (1 h) and in specific primary antibody (4°C, overnight), followed by appropriate secondary antibody (***Table-S5***). The immunoblots were visualized using an Odyssey CLX (LI-COR).

**References**

1. Bouska, A.*, et al.* Adult high-grade B-cell lymphoma with Burkitt lymphoma signature: genomic features and potential therapeutic targets. *Blood* **130**, 1819-1831 (2017).

2. Scholtysik, R.*, et al.* Detection of genomic aberrations in molecularly defined Burkitt's lymphoma by array-based, high resolution, single nucleotide polymorphism analysis. *Haematologica* **95**, 2047-2055 (2010).

3. Lenz, G.*, et al.* Molecular subtypes of diffuse large B-cell lymphoma arise by distinct genetic pathways. *Proceedings of the National Academy of Sciences of the United States of America* **105**, 13520-13525 (2008).

4. Scandurra, M.*, et al.* Genomic lesions associated with a different clinical outcome in diffuse large B-Cell lymphoma treated with R-CHOP-21. *British journal of haematology* **151**, 221-231 (2010).

5. Perry, A.M.*, et al.* B-cell lymphoma, unclassifiable, with features intermediate between diffuse large B-cell lymphoma and burkitt lymphoma: study of 39 cases. *British journal of haematology* **162**, 40-49 (2013).

6. Dave, S.S.*, et al.* Molecular diagnosis of Burkitt's lymphoma. *The New England journal of medicine* **354**, 2431-2442 (2006).

7. Ennishi, D.*, et al.* Double-Hit Gene Expression Signature Defines a Distinct Subgroup of Germinal Center B-Cell-Like Diffuse Large B-Cell Lymphoma. *J Clin Oncol* **37**, 190-201 (2019).

8. Ma, M.C.J.*, et al.* Subtype-specific and co-occurring genetic alterations in B-cell non-Hodgkin lymphoma. *Haematologica* (2021).

9. Bouska, A.*, et al.* Genome-wide copy-number analyses reveal genomic abnormalities involved in transformation of follicular lymphoma. *Blood* **123**, 1681-1690 (2014).

10. Olshen, A.B., Venkatraman, E.S., Lucito, R. & Wigler, M. Circular binary segmentation for the analysis of array-based DNA copy number data. *Biostatistics* **5**, 557-572 (2004).

11. Spruance, S.L., Reid, J.E., Grace, M. & Samore, M. Hazard ratio in clinical trials. *Antimicrob Agents Chemother* **48**, 2787-2792 (2004).

12. McKenna, A.*, et al.* The Genome Analysis Toolkit: a MapReduce framework for analyzing next-generation DNA sequencing data. *Genome Res* **20**, 1297-1303 (2010).

13. Tian, S., Yan, H., Kalmbach, M. & Slager, S.L. Impact of post-alignment processing in variant discovery from whole exome data. *BMC Bioinformatics* **17**, 403 (2016).

14. Koboldt, D.C.*, et al.* VarScan 2: somatic mutation and copy number alteration discovery in cancer by exome sequencing. *Genome Res* **22**, 568-576 (2012).

15. Strom, S.P. Current practices and guidelines for clinical next-generation sequencing oncology testing. *Cancer Biol Med* **13**, 3-11 (2016).

16. Kuilman, T.*, et al.* CopywriteR: DNA copy number detection from off-target sequence data. *Genome Biol* **16**, 49 (2015).

17. Newman, A.M.*, et al.* FACTERA: a practical method for the discovery of genomic rearrangements at breakpoint resolution. *Bioinformatics* **30**, 3390-3393 (2014).

18. Iqbal, J.*, et al.* Genome-wide miRNA profiling of mantle cell lymphoma reveals a distinct subgroup with poor prognosis. *Blood* **119**, 4939-4948 (2012).

19. Shaffer, A.L.*, et al.* A library of gene expression signatures to illuminate normal and pathological lymphoid biology. *Immunol Rev* **210**, 67-85 (2006).

20. Liberzon, A.*, et al.* The Molecular Signatures Database (MSigDB) hallmark gene set collection. *Cell Syst* **1**, 417-425 (2015).

21. Kramer, A., Green, J., Pollard, J., Jr. & Tugendreich, S. Causal analysis approaches in Ingenuity Pathway Analysis. *Bioinformatics* **30**, 523-530 (2014).

22. Aran, D., Hu, Z. & Butte, A.J. xCell: digitally portraying the tissue cellular heterogeneity landscape. *Genome Biol* **18**, 220 (2017).

23. Huang da, W., Sherman, B.T. & Lempicki, R.A. Systematic and integrative analysis of large gene lists using DAVID bioinformatics resources. *Nat Protoc* **4**, 44-57 (2009).

24. Wright, G.W.*, et al.* A Probabilistic Classification Tool for Genetic Subtypes of Diffuse Large B Cell Lymphoma with Therapeutic Implications. *Cancer Cell* **37**, 551-568 e514 (2020).

25. Greenwald, N.F.*, et al.* Whole-cell segmentation of tissue images with human-level performance using large-scale data annotation and deep learning. *Nat Biotechnol* **40**, 555-565 (2022).

26. Scrucca, L., Fop, M., Murphy, T.B. & Raftery, A.E. mclust 5: Clustering, Classification and Density Estimation Using Gaussian Finite Mixture Models. *R J* **8**, 289-317 (2016).
