# Supplementary material for: High‐grade B‐cell lymphoma not otherwise specified, with diffuse large B‐cell lymphoma gene expression signatures: Genomic analysis and potential therapeutics
Source: Am J Hematol. 2024 Nov 16;100(1):10–22. doi: 10.1002/ajh.27513 (PMC11625982; doi:10.1002/ajh.27513)
Supplement: Supplementary file 3 — Data S3. Tables. [file AJH-100-10-s001.pdf]

Table S1: Clinical and Pathological Data of HGBCL, NOS Cases														FISH	FISH	FISH	IHC	IHC	IHC	IHC	IHC	IHC
	Name	Institutional Pathology Review Report	Final Pathology Review Report	Age	Gender	Time (Years)	Status at Follow-up	HG-U133 plus2 molecular class	DLBCL90 (nCounter) DLBCLcall	DLBCL90 (nCounter) DHITsig Call	Affymetrix U133 plus2	Affymetrix 250K SNP array	Sequencing	MYC-Translocation	BCL2-Translocation	BCL6-Translocation	MYC	CD10	CD20	BCL2	BCL6	Ki-67
HGBCL, NOS	HGBCL-21	HGBCL, NOS	ABC-DLBCL	62	Female	6.64	Dead	Not Run	GCB	POS			WES	unknown	unknown	Pos	+	-	+	+	+	+
HGBCL, NOS	HGBCL-1	HGBCL, NOS	BL	60	Male	0.17	Dead	Not Run	GCB	POS			Targeted/WES	Pos	unknown	unknown		+	+	-	+	+
HGBCL, NOS	HGBCL-12	HGBCL, NOS	BL	70	Female	NA	NA	Not Run	GCB	POS				Pos	unknown	unknown		+	+	-	+	+
HGBCL, NOS	HGBCL-13	HGBCL, NOS	BL	58	Male	NA	NA	Not Run	GCB	POS			WES	Pos	unknown	unknown	+	+	+	-	+	+
HGBCL, NOS	HGBCL-14	HGBCL, NOS	BL	37	Male	NA	NA	Not Run	GCB	POS			WES	Pos	Neg	Neg	+	+	+	-	+	+
HGBCL, NOS	HGBCL-2	BLL	BL	51	Female	NA	NA	Not Run	GCB	POS				Pos	Neg	Neg		+	+	-	+	+
HGBCL, NOS	HGBCL-22	HGBL	DLBCL	78	Male	4.96	alive	Not Run	ABC	NEG				Neg	Neg	Pos	+	-	+	+	+	+
HGBCL, NOS	HGBCL-15	Low grade Lymphoma	DLBCL	35	Male	NA	NA	Not Run	GCB	POS			WES					+	+	-	+	+
HGBCL, NOS	HGBCL-3	HGBCL, NOS	DLBCL, Double expresser	54	Female	9.66	alive	Not Run	ABC	NEG			Targeted/WES				+	+	+	+	+	+
HGBCL, NOS	HGBCL-23	DLBCL	HGBCL, NOS	57	Male	3.14	Dead	Not Run	GCB	POS				Neg	Pos	Neg	-	+	+	+	+	+
HGBCL, NOS	HGBCL-31	SNC-NB	No concensus	61	Male	0.17	Dead	GCB-DLBCL	GCB	UNCLASS	x	x	Targeted/WES	Fail	unknown	unknown	+	+	+	-	-	+
HGBCL, NOS	HGBCL-10	HGBCL, NOS	HGBCL, NOS	69	Female	not specified	Dead	Not Run	ABC	NEG							+	+	+	+	-	+
HGBCL, NOS	HGBCL-17	HGBCL, NOS	HGBCL, NOS		Female	NA	NA	Not Run	GCB	POS				Neg	Pos	Neg	+	+	+	-	+	+
HGBCL, NOS	HGBCL-18	HGBCL, NOS	HGBCL, NOS		Female	NA	NA	Not Run	ABC	NEG				Neg	Neg	Neg	+	+	+	-	+	+
HGBCL, NOS	HGBCL-19	BLL	HGBCL, NOS	70	Male	NA	NA	Not Run	GCB	UNCLASS				Neg	Neg	Neg						
HGBCL, NOS	HGBCL-20		HGBCL, NOS	75	Female	NA	NA	Not Run	GCB	UNCLASS			WES	Pos	unknown	unknown	+	+	+	+	+	+
HGBCL, NOS	HGBCL-24	HGBCL	HGBCL, NOS	41	Female	2.59	Dead	Not Run	ABC	NEG			WES	Neg	Pos	Neg	+	+	+	+	+	+
HGBCL, NOS	HGBCL-25	HG-B CELL	HGBCL, NOS	72	Male	3.12	alive	Not Run	ABC	NEG			WES				+	-	+	+	+	+
HGBCL, NOS	HGBCL-26	SNC-NB	HGBCL, NOS	66	Male	1.41	Dead	DLBCL-NC	ABC	POS	x	x	Targeted/WES	unknown	unknown	unknown	+	-	+	+	+	+
HGBCL, NOS	HGBCL-27	SNC-NB	HGBCL, NOS	79	Male	0.07	Dead	ABC-DLBCL	ABC	POS	x	x	Targeted/WES	Pos	unknown	unknown	+	-	+	+	+	+
HGBCL, NOS	HGBCL-28	SNC-B	HGBCL, NOS	83	Male	0.32	Dead	GCB-DLBCL	GCB	POS	x	x	Targeted/WES	Neg	unknown	unknown	+	-	+	+	+	+
HGBCL, NOS	HGBCL-29	SNC-B	HGBCL, NOS	81	Male	0.04	Dead	ABC-DLBCL	GCB	NEG	x	x	Targeted/WES				+	-	+	+	+	+
HGBCL, NOS	HGBCL-30	SNC-NB	HGBCL, NOS	79	Female	3.36	Dead	ABC-DLBCL	UNCLASS	NEG	x	x	Targeted/WES	Neg	unknown	unknown	+	-	+	+	+	+
HGBCL, NOS	HGBCL-33	SNC-NB	HGBCL, NOS	82	Male	0.03	Dead	ABC-DLBCL	ABC	NEG	x	x	Targeted/WES	Pos	unknown	unknown	+	-	+	+	-	+
HGBCL, NOS	HGBCL-4	B-BLL	HGBCL, NOS	70	Female	0.12	Dead	Not Run	GCB	NEG			Targeted/WES	Neg	Neg	unknown		+	+	+	+	+
HGBCL, NOS	HGBCL-5	BLL	HGBCL, NOS	71	Male	10.10	Dead	Not Run	ABC	NEG			Targeted/WES	Neg	Neg	Neg		+	+	+	+	+
HGBCL, NOS	HGBCL-6	B-UCL-HG	HGBCL, NOS	65	Female	11.28	alive	Not Run	GCB	UNCLASS			Targeted/WES	Neg	Neg	Neg	-	+	+	+	+	+
HGBCL, NOS	HGBCL-7	HGBCL, NOS	HGBCL, NOS	65	Male	not specified	Dead	Not Run	Not Run	Not Run				Neg	Neg	Neg		+				
HGBCL, NOS	HGBCL-71	HGBL-NOS	HGBCL, NOS	70	Male	NA	NA	Not Run	GCB	NEG			WGS	Neg	Pos	Neg						
HGBCL, NOS	HGBCL-72	HGBL-NOS	HGBCL, NOS	54	Female	NA	NA	Not Run	GCB	NEG			WGS	Neg	Neg	Neg						
HGBCL, NOS	HGBCL-73	HGBL-NOS	HGBCL, NOS	87	Female	NA	NA	Not Run	ABC	NEG			WGS	Neg	Neg	Pos						
HGBCL, NOS	HGBCL-74	HGBL-NOS	HGBCL, NOS	68	Male	NA	NA	Not Run	ABC	NEG			WGS	Neg	Neg	Neg						
HGBCL, NOS	HGBCL-75	HGBL-NOS	HGBCL, NOS	82	Female	NA	NA	Not Run	GCB	NEG			WGS	Neg	Neg	Pos						
HGBCL, NOS	HGBCL-76	HGBL-NOS	HGBCL, NOS	85	Male	NA	NA	Not Run	GCB	NEG			WES	Neg	Neg	Neg						
HGBCL, NOS	HGBCL-77	HGBL-NOS	HGBCL, NOS	83	Male	NA	NA	Not Run	ABC	NEG			WES	Neg	Neg	Neg						
HGBCL, NOS	HGBCL-78	HGBL-NOS	HGBCL, NOS	65	Male	NA	NA	Not Run	GCB	NEG			WES	Neg	Pos	Neg						
HGBCL, NOS	HGBCL-79	HGBL-NOS	HGBCL, NOS	69	Male	NA	NA	Not Run	ABC	NEG			WES	Neg	Neg	Neg						
HGBCL, NOS	HGBCL-8	HGBCL, NOS	HGBCL, NOS	55	Male	not specified	Dead	Not Run	ABC	NEG			WES	Neg	Neg	Neg	+	-	+	-	+	+
HGBCL, NOS	HGBCL-80	HGBL-NOS	HGBCL, NOS	78	Male	NA	NA	Not Run	UNCLASS	NEG			WES	Neg	Fail	Fail						
HGBCL, NOS	HGBCL-81	HGBL-NOS	HGBCL, NOS	65	Female	NA	NA	Not Run	GCB	NEG			WES	Neg	Neg	Neg						
HGBCL, NOS	HGBCL-82	HGBL-NOS	HGBCL, NOS	19	Male	NA	NA	Not Run	ABC	NEG			WES	Neg	Neg	Neg						
HGBCL, NOS	HGBCL-83	HGBL-NOS	HGBCL, NOS	70	Male	NA	NA	Not Run	UNCLASS	NEG			WES	Neg	Neg	Neg						
HGBCL, NOS	HGBCL-84	HGBL-NOS	HGBCL, NOS	73	Female	NA	NA	Not Run	UNCLASS	NEG			WES	Neg	Neg	Neg						
HGBCL, NOS	HGBCL-85	HGBL-NOS	HGBCL, NOS	73	Male	NA	NA	Not Run	GCB	NEG			WES	Neg	Neg	Neg						
HGBCL, NOS	HGBCL-86	HGBL-NOS	HGBCL, NOS	77	Female	NA	NA	Not Run	GCB	NEG			WES	Neg	Neg	Neg						
HGBCL, NOS	HGBCL-87	HGBL-NOS	HGBCL, NOS	83	Male	NA	NA	Not Run	GCB	NEG			WES	Neg	unknown	Neg						
HGBCL, NOS	HGBCL-88	HGBL-NOS	HGBCL, NOS	74	Female	NA	NA	Not Run	ABC	NEG			WES	Neg	Neg	Pos						
HGBCL, NOS	HGBCL-9	HGBCL, NOS	HGBCL, NOS	41	Male	not specified	Dead	Not Run	ABC	NEG			WES	Neg	Neg	Pos	+	-	+	+	+	+
HGBCL, NOS	HGBCL-16	HGBCL, NOS	No concensus		Male	NA	NA	Not Run	GCB	POS			WES	Pos	Neg	Neg	+	+	+	-	+	+
HGBCL, NOS	HGBCL-11	HGBCL, NOS	HGBCL, NOS	29	Male	not specified	Dead	Not Run	ABC	NEG			WES	Neg	Neg	Neg	+	+	+	-	+	+
HGBCL, NOS	HGBCL-32	SNC-NB	HGBCL, NOS	85	Female	0.17	Dead	ABC-DLBCL	ABC	NEG	x	x	Targeted/WES	Pos								
HGBCL, NOS	HGBCL-34	BLL	HGBCL, NOS	75	Female	1.01	alive	GCB-DLBCL	UNCLASS	POS	x	x	Targeted/WES	Pos						-		
HGBCL, NOS	HGBCL-35	BLL	HGBCL, NOS	6	Male	NA	NA	DLBCL-NC	GCB	POS	x	x	Targeted/WES	Pos				+	+			
HGBCL, NOS	HGBCL-36	BLL	HGBCL, NOS	2	Male	NA	NA	GCB-DLBCL	GCB	UNCLASS	x	x	Targeted/WES	Neg								
HGBCL, NOS	HGBCL-37	BLL	HGBCL, NOS	11	Female	4.45	alive	DLBCL-NC	GCB	NEG	x	x	Targeted/WES	Pos								
HGBCL, NOS	HGBCL-38	BLL	HGBCL, NOS	2	Male	3.88	alive	ABC-DLBCL	GCB	NEG	x	x	Targeted/WES	Pos								
HGBCL, NOS	HGBCL-39	BLL	HGBCL, NOS	5	Male	NA	NA	GCB-DLBCL	GCB	NEG	x	x	Targeted/WES	Neg								
HGBCL, NOS	HGBCL-40	BLL	HGBCL, NOS	4	Female	NA	NA	GCB-DLBCL	GCB	UNCLASS	x	x	Targeted/WES	Pos								
HGBCL, NOS	HGBCL-41	BLL	HGBCL, NOS	10	Male	NA	NA	GCB-DLBCL	GCB	NEG	x	x	Targeted/WES	Neg								
HGBCL, NOS	HGBCL-42	BLL	HGBCL, NOS	6	Male	NA	NA	GCB-DLBCL	GCB	POS	x	x	Targeted/WES	Pos								
HGBCL, NOS	HGBCL-43	BLL	HGBCL, NOS	6	Male	NA	NA	GCB-DLBCL	GCB	UNCLASS	x	x	Targeted/WES	Pos				+	+			
HGBCL, NOS	HGBCL-44	BLL	HGBCL, NOS	10	Female	NA	NA	GCB_DLBCL	GCB	UNCLASS	x	x		Neg								
HGBCL, NOS	HGBCL-89		HGBCL, NOS					Not Run	Not Run	Not Run			Targeted									
HGBCL, NOS	HGBCL-90		HGBCL, NOS			15.10	alive	Not Run	Not Run	Not Run			Targeted									
HGBCL, NOS	HGBCL-91	HGBCL	HGBCL, NOS	73	Male	not specified	Dead	ABC-DLBCL	ABC	NEG			WES	Neg	Neg	Neg	+	-	+	+	+	+
HGBCL, NOS-IMC only	HGBCL-57	SNC-NB	IMC only Case	65		NA	NA	ABC-DLBCL	Not Run	Not Run	x	x	Targeted									
HGBCL, NOS-IMC only	HGBCL-67	SNC-BLL	IMC only Case	12		NA	NA	DLBCL-NC	Not Run	Not Run	x	x	Targeted									
mBL from IMC	HGBCL-45	SNC-NB	IMC only Case, mBL from Bouska et al.Blood (2017)		Male	22.93	alive	BL	Not Run	Not Run	x	x	Targeted									
mBL from IMC	HGBCL-46	SNC-NB	IMC only Case, mBL from Bouska et al.Blood (2017)	20		12.96	alive	BL	Not Run	Not Run	x	x	Targeted									
mBL from IMC	HGBCL-47	SNC-BLL	IMC only Case, mBL from Bouska et al.Blood (2017)	21		NA	NA	BL	Not Run	Not Run	x	x	Targeted									
mBL from IMC	HGBCL-48	SNC-B	IMC only Case, mBL from Bouska et al.Blood (2017)	55	Male	0.11	Dead	BL	Not Run	Not Run	x	x	Targeted									
mBL from IMC	HGBCL-49	SNC-NB	IMC only Case, mBL from Bouska et al.Blood (2017)	12		15.53	alive	BL	Not Run	Not Run	x	x	Targeted									
mBL from IMC	HGBCL-50	SNC-NB	IMC only Case, mBL from Bouska et al.Blood (2017)	78	Male	0.92	Dead	BL	Not Run	Not Run	x	x	Targeted									
mBL from IMC	HGBCL-51	SNC-NB	IMC only Case, mBL from Bouska et al.Blood (2017)	73	Female	0.39	Dead	BL	Not Run	Not Run	x	x	Targeted									
mBL from IMC	HGBCL-52	SNC-NB	IMC only Case, mBL from Bouska et al.Blood (2017)	39	Male	0.38	Dead	BL	Not Run	Not Run	x	x	Targeted									
mBL from IMC	HGBCL-53	SNC-B	IMC only Case, mBL from Bouska et al.Blood (2017)	4		13.07	alive	BL	Not Run	Not Run	x	x	Targeted									
mBL from IMC	HGBCL-54	SNC-NB	IMC only Case, mBL from Bouska et al.Blood (2017)	71	Female	0.18	Dead	BL	Not Run	Not Run	x	x	Targeted									
mBL from IMC	HGBCL-55	SNC-B	IMC only Case, mBL from Bouska et al.Blood (2017)	6		4.92	alive	BL	Not Run	Not Run	x	x	Targeted									
mBL from IMC	HGBCL-56	BLL	IMC only Case, mBL from Bouska et al.Blood (2017)	38		9.82	alive	BL	Not Run	Not Run	x	x	Targeted									
mBL from IMC	HGBCL-58	SNC-B	IMC only Case, mBL from Bouska et al.Blood (2017)	6	Male	0.95	alive	BL	Not Run	Not Run	x	x	Targeted									
mBL from IMC	HGBCL-59	SNC-BLL	IMC only Case, mBL from Bouska et al.Blood (2017)	7		NA	NA	BL	Not Run	Not Run	x	x	Targeted									
mBL from IMC	HGBCL-60	BLL	IMC only Case, mBL from Bouska et al.Blood (2017)	43	Male	0.53	Dead	BL	Not Run	Not Run	x	x	Targeted									
mBL from IMC	HGBCL-61	SNC-NB	IMC only Case, mBL from Bouska et al.Blood (2017)	50	Female	0.55	Dead	BL	Not Run	Not Run	x	x	Targeted									
mBL from IMC	HGBCL-62	SNC-NB	IMC only Case, mBL from Bouska et al.Blood (2017)	43	Male	8.83	Dead	BL	Not Run	Not Run	x	x	Targeted									
mBL from IMC	HGBCL-65	SNC-BLL	IMC only Case, mBL from Bouska et al.Blood (2017)	61	Female	0.05	Dead	BL	Not Run	Not Run	x	x	Targeted									
mBL from IMC	HGBCL-66	SNC_BLL	IMC only Case, mBL from Bouska et al.Blood (2017)	20	Male	5.09	alive	BL	Not Run	Not Run	x	x	Targeted									
mBL from IMC	HGBCL-68	B-BLL	IMC only Case, mBL from Bouska et al.Blood (2017)	78	Male	2.48	alive	BL	Not Run	Not Run	x	x	Targeted									
mBL from IMC	HGBCL-69	B-BLL	IMC only Case, mBL from Bouska et al.Blood (2017)	67	Female	4.99	alive	BL	Not Run	Not Run	x	x	Targeted									
mBL from IMC	HGBCL-70	B-BLL	IMC only Case, mBL from Bouska et al.Blood (2017)	27	Male	2.91	alive	BL	Not Run	Not Run	x	x	Targeted									

Table S2: Pathway enrichment of genes muted in DH-negative HGBCL, NOS

Pathway	Description	GeneRatio	BgRatio	pvalue	p.adjust	qvalue	geneID	Count	Database
R-HSA-1980143	Signaling by NOTCH1	23/1354	74/10955	1.69E-05	0.0206301	0.018904	DTX1;NCOR1;ITCH;EP300;MIB2;ADAM17;CUL1;HDAC1;HDAC10;HDAC2;HDAC3;HDAC4;HDAC7;KAT2A;MAML1;MAML2;MIB1;NBEA;NEURL1B;NUMB;TBL1X;TLE2;TLE3	23	Reactome
R-HSA-9006925	Intracellular signaling by second messengers	63/1354	309/10955	3.4E-05	0.0206301	0.018904	PP2R1B;PRKACB;PRKAR1B;PSMC4;PSMC5;PSMD13;PSME4;RAC2;RBBP4;RNF2;TNRC6A;TRIM27;USP7	63	Reactome
R-HSA-4420097	VEGFA-VEGFR2 Pathway	27/1354	99/10955	4.52E-05	0.0206301	0.018904	CTNND1;ELMO1;HSP90AA1;ITPR3;PRKCA;VEGFA;AXL;AKT2;DOCK1;ITPR1;ITPR2;KRAS;MAPK11;MAPKAPK2;MTOR;NCKAP1;NOS3;PAK2;PAK3;PIK3CA;PIK3CB;PLCG1;PRKACB;PRKCB;ROCK2;SHB;SHC2	27	Reactome
R-HSA-350054	Notch-HLH transcription pathway	12/1354	28/10955	5.48E-05	0.0206301	0.018904	NCOR1;HDAC1;HDAC10;HDAC2;HDAC3;HDAC4;HDAC7;KAT2A;MAML1;MAML2;NOTCH4;TBL1X	12	Reactome
R-HSA-194138	Signaling by VEGF	28/1354	108/10955	8.79E-05	0.0239075	0.021907	CTNND1;ELMO1;HSP90AA1;ITPR3;PRKCA;VEGFA;AXL;AKT2;DOCK1;FLT1;ITPR1;ITPR2;KRAS;MAPK11;MAPKAPK2;MTOR;NCKAP1;NOS3;PAK2;PAK3;PIK3CA;PIK3CB;PLCG1;PRKACB;PRKCB;ROCK2;SHB;SHC2	28	Reactome
R-HSA-5663202	second messengers	80/1354	433/10955	0.00012	0.0239075	0.021907	KRAS;LCK;LYN;MAML1;MAML2;MECP2;MIB1;MTOR;MYH9;MYO18A;NCBP1;NEURL1B;NRG3;PDGFRA;PIK3AP1;PIK3CA;PIK3CB;PLCG1;POLR2B;PPP2R1B;PSMC4;PSMC5;PSMD13;PSME4;RAC2;RNF43;SMAD2;STAT1;TBL1X;TGFBR2;TRIM24;VCP	80	Reactome
R-HSA-6804758	Regulation of TP53 Activity through Acetylation	12/1354	30/10955	0.000123	0.0239075	0.021907	CHD4;EP300;AKT2;BRD1;GATAD2B;HDAC1;HDAC2;MBD3;MTA2;PIP4K2C;PML;RBBP4	12	Reactome
R-HSA-2122947	NOTCH1 Intracellular Domain Regulates Transcription	16/1354	48/10955	0.000127	0.0239075	0.021907	NCOR1;EP300;CUL1;HDAC1;HDAC10;HDAC2;HDAC3;HDAC4;HDAC7;KAT2A;MAML1;MAML2;NBEA;TBL1X;TLE2;TLE3	16	Reactome
R-HSA-3000171	Non-integrin membrane-ECM interactions	18/1354	59/10955	0.000178	0.0281907	0.025832	NRXN1;AGRN;COL4A3;COL5A3;ITGB5;LAMA2;LAMA3;LAMA5;PRKCA;COL1A1;COL11A2;DAG1;DDR2;HSPG2;LAMB3;LAMC1;THBS1;TNC	18	Reactome
R-HSA-8984722	Interleukin-35 Signalling	7/1354	12/10955	0.000195	0.0281907	0.025832	STAT3;STAT4;EBI3;IL12A;IL6ST;STAT1;TYK2	7	Reactome
R-HSA-9679506	SARS-CoV Infections	76/1354	414/10955	0.000217	0.0281907	0.025832	1;NUP133;NUP188;NUP214;NUP85;NUP98;PARP6;RBBP4;RIPK2;ROCK2;RPS11;RPS16;RPS7;RPS8;RUNX1;SEC24A;SFTPD;ST3GAL4;STAT1;SYK;TJP1;TLR2;TLR9;TRIM25;TYK2;VCP;VPS33A;ZDHHC2	76	Reactome
R-HSA-5633007	Regulation of TP53 Activity	36/1354	160/10955	0.000225	0.0281907	0.025832	RAD50;CDK5R1;CHD4;POU4F1;EP300;SGK1;TAF1;AKT2;BRD1;CSNK2A1;CSNK2B;EHMT2;EXO1;GATAD2B;HDAC1;HDAC2;HIPK1;MAPK11;MBD3;MTA2;MTOR;PIP4K2C;PML;PPP2R1B;RBBP4;RFC2;RFC4;RNF34;SMYD2;TAF3;TAF4B;TP63;TP73;TPX2;USP2;USP7	36	Reactome
R-HSA-8943724	Regulation of PTEN gene transcription	18/1354	61/10955	0.000283	0.0309701	0.028379	EGR1;CBX6;CHD4;PHC3;RRAGC;ATN1;GATAD2B;HDAC1;HDAC2;HDAC3;HDAC7;MBD3;MTA1;MTA2;MTA3;MTOR;RBBP4;RNF2	18	Reactome
R-HSA-982772	Growth hormone receptor signaling	10/1354	24/10955	0.000306	0.0309701	0.028379	PTPN6;SOCS1;IRS2;STAT3;ADAM17;GHR;LYN;SOCS2;SOCS3;STAT1	10	Reactome
R-HSA-6807070	PTEN Regulation	32/1354	140/10955	0.000364	0.0309701	0.028379	EGR1;CBX6;CHD4;PHC3;PSMB10;RRAGC;PSMA3;AKT2;ATN1;CSNK2A1;CSNK2B;GATAD2B;HDAC1;HDAC2;HDAC3;HDAC7;MBD3;MTA1;MTA2;MTA3;MTOR;NEDD4;PML;PSMC4;PSMC5;PSMD13;PSME4;RBBP4;RNF2;TNRC6A;TRIM27;USP7	32	Reactome
R-HSA-5607763	CLEC7A (Dectin-1) induces NFAT activation	7/1354	13/10955	0.000378	0.0309701	0.028379	ITPR3;PPP3CB;ITPR1;ITPR2;NFATC1;NFATC3;PPP3R1	7	Reactome
R-HSA-983705	Signaling by the B Cell Receptor (BCR)	27/1354	112/10955	0.000421	0.0309701	0.028379	BTK;CD79A;PTPN6;ITPR3;PSMB10;PPP3CB;PSMA3;CD19;CHUK;CUL1;IKBKB;ITPR1;ITPR2;KRAS;LYN;NFATC1;NFATC3;NFKBIB;PIK3AP1;PPP3R1;PRKCB;PSMC4;PSMC5;PSMD13;PSME4;SYK;TRPC1	27	Reactome
R-HSA-2644602	Signaling by NOTCH1 PEST Domain Mutants in Cancer	17/1354	58/10955	0.000452	0.0309701	0.028379	NCOR1;EP300;MIB2;ADAM17;CUL1;HDAC1;HDAC10;HDAC2;HDAC3;HDAC4;HDAC7;KAT2A;MAML1;MAML2;MIB1;NEURL1B;TBL1X	17	Reactome
R-HSA-2644603	Signaling by NOTCH1 in Cancer	17/1354	58/10955	0.000452	0.0309701	0.028379	NCOR1;EP300;MIB2;ADAM17;CUL1;HDAC1;HDAC10;HDAC2;HDAC3;HDAC4;HDAC7;KAT2A;MAML1;MAML2;MIB1;NEURL1B;TBL1X	17	Reactome
R-HSA-2644606	Constitutive Signaling by NOTCH1 PEST Domain Mutants	17/1354	58/10955	0.000452	0.0309701	0.028379	NCOR1;EP300;MIB2;ADAM17;CUL1;HDAC1;HDAC10;HDAC2;HDAC3;HDAC4;HDAC7;KAT2A;MAML1;MAML2;MIB1;NEURL1B;TBL1X	17	Reactome
R-HSA-2894858	Signaling by NOTCH1 HD+PEST Domain Mutants in Cancer	17/1354	58/10955	0.000452	0.0309701	0.028379	NCOR1;EP300;MIB2;ADAM17;CUL1;HDAC1;HDAC10;HDAC2;HDAC3;HDAC4;HDAC7;KAT2A;MAML1;MAML2;MIB1;NEURL1B;TBL1X	17	Reactome
R-HSA-2894862	Constitutive Signaling by NOTCH1 HD+PEST Domain Mutants	17/1354	58/10955	0.000452	0.0309701	0.028379	NCOR1;EP300;MIB2;ADAM17;CUL1;HDAC1;HDAC10;HDAC2;HDAC3;HDAC4;HDAC7;KAT2A;MAML1;MAML2;MIB1;NEURL1B;TBL1X	17	Reactome
R-HSA-9701898	STAT3 nuclear events downstream of ALK signaling	6/1354	10/10955	0.000475	0.0311272	0.028523	EP300;STAT3;CD274;HDAC1;HDAC2;HDAC3	6	Reactome
R-HSA-1257604	PIP3 activates AKT signaling	52/1354	267/10955	0.000515	0.032307	0.029604	NRC6A;TRIM27;USP7	52	Reactome
R-HSA-451927	Interleukin-2 family signaling	14/1354	44/10955	0.00057	0.0342413	0.031376	PTPN6;SOS2;STAT3;STAT4;CSF2RB;IL21R;IL2RB;IL3RA;INPP5D;LCK;PIK3CA;PIK3CB;STAT1;SYK	14	Reactome
R-HSA-2029480	Fcgamma receptor (FCGR) dependent phagocytosis	22/1354	86/10955	0.000591	0.0342413	0.031376	BTK;ELMO1;HSP90AA1;ITPR3;WAS;ABL1;ACTR3;ARPC1B;DOCK1;FGR;HSP90AB1;ITPR1;ITPR2;LYN;MYH9;MYO1C;MYO9B;NCKAP1;PIK3CA;PIK3CB;PLCG1;SYK	22	Reactome
R-HSA-9679191	Potential therapeutics for SARS	24/1354	99/10955	0.000789	0.0426185	0.039053	BTK;CD79A;BRD4;CHD4;HSP90AA1;VEGFA;ATP1A3;FKBP4;GATAD2B;HDAC1;HDAC2;HSP90AB1;IFNGR1;KEAP1;MBD3;MTA1;MTA2;MTA3;NR3C1;RBBP4;ROCK2;SYK;TLR9;TYK2	24	Reactome
R-HSA-1474228	Degradation of the extracellular matrix	31/1354	140/10955	0.000792	0.0426185	0.039053	COL19A1;COL4A3;COL5A3;COL6A2;LAMA3;LAMA5;COL6A3;COL1A1;MMP2;ADAM17;ADAM9;CAPN12;CAPN14;CAPN3;CAPN8;CAPNS1;CAPNS2;CASP3;CD44;COL11A2;COL15A1;COL23A1;COL7A1;FBN1;HSPG2;LAMB3;LAMC1;MMP1;MMP9;SCUBE3;TLL1	31	Reactome
R-HSA-201556	Signaling by ALK	10/1354	27/10955	0.000928	0.047186	0.043238	PTPN6;EP300;STAT3;CD274;HDAC1;HDAC2;HDAC3;PIK3CA;PIK3CB;PLCG1	10	Reactome
R-HSA-1474244	Extracellular matrix organization	56/1354	300/10955	0.00094	0.047186	0.043238	LOXL4;LTBP2;MMP1;MMP9;SCUBE3;TGFB3;THBS1;TLL1;TNC;TNN	56	Reactome

Table S3. Cell Line Characteristics.	HBL1	TMD8	OCI-LY3	U2932	OCI-Ly8	DHL16
Expression Class	ABC-DLBCL[1]	ABC-DLBCL[1]	ABC-DLBCL[1]	ABC-DLBCL[1]	GCB-DLBCL[2]	GCB-DLBCL[7]
Genetic Class	MCD[5]	MCD[5]	MCD[5]	Unclassified[5]	EZB[5]	EZB[5]
CDKN2A/B Status	WT[5]	Homozygous loss[5]	Homozygous loss[5]	WT[5]	Homozygous loss (CDKN2A)[5]	Homozygous loss[5]
PIM1 Status	MUT[4]	MUT[4]	MUT[4]	WT[4]	MUT[4]	WT[7]
MYD88 Status	MUT[3]	MUT[3]	MUT[3]	WT[6]	WT	WT[7]
CD79b	MUT[5]	MUT[5]	WT[5]	WT[6]	WT[2]	WT[7]
AZD-1208	Resistant[4]	Responsive[4]	Sensitive[4]	Resistant[4]	Resistant[4]	Resistant[7]
Ibrutinib	Sensitive[2]	Sensitive[2]	Resistant[6]	Resistant[6]	Resistant[2]	Sensitive[7]

1. Phelan, et. al. (2018)- PMID: 29925955
2.Wilson, et. al. (2021)- PMID: 26193343
3. Ngo, et. al. (2011)- PMID: 21179087
4. Kuo et. al. (2016)- PMID: 27904766
5. Wright et. al. (2020)- PMID: 32289277
6. Kozaki et. al. (2018)- PMID: 29690649
7. Chapuy et. al. (2018)- PMID: 29713087

Table S4: WES and Targeted Sequencing Mutations

ID	Chr	Start	End	Ref	Alt	Func.refGene	Gene.refGene	reads1	reads2	VAF	Variant_Classification	AAChange	Mutation.Compile.Group	Group
BCL-1	chr9	123452275	123452275	C	T	splicing	DENND1A	76	3	0.0375	Splice_Site	.	Non-Targeted Panel Gene	Adult HGBCL, NOS
BCL-1	chr10	114210749	114210749	G	C	splicing	TDRD1	50	2	0.0377	Splice_Site	.	Non-Targeted Panel Gene	Adult HGBCL, NOS
BCL-1	chr21	36288812	36288812	G	A	splicing	DOPEY2	48	3	0.0588	Splice_Site	.	Non-Targeted Panel Gene	Adult HGBCL, NOS
BCL-1	chr3	48309903	48309903	C	T	splicing	SPINK8	30	2	0.0625	Splice_Site	.	Non-Targeted Panel Gene	Adult HGBCL, NOS
BCL-1	chr17	58304337	58304337	C	T	splicing	TSPOAP1	44	3	0.0638	Splice_Site	.	Non-Targeted Panel Gene	Adult HGBCL, NOS
BCL-1	chr6	131671328	131671328	G	A	splicing	ENPP3	56	4	0.0656	Splice_Site	.	Non-Targeted Panel Gene	Adult HGBCL, NOS
BCL-1	chr5	113294328	113294328	C	T	splicing	MCC	26	2	0.0714	Splice_Site	.	Non-Targeted Panel Gene	Adult HGBCL, NOS
BCL-1	chr11	74351382	74351382	C	T	splicing	PGM2L1	35	3	0.0789	Splice_Site	.	Non-Targeted Panel Gene	Adult HGBCL, NOS
BCL-1	chr6	43560923	43560923	C	T	splicing	XPO5	43	4	0.0851	Splice_Site	.	Non-Targeted Panel Gene	Adult HGBCL, NOS
BCL-1	chr13	111273953	111273953	G	A	splicing	ARHGEF7	31	3	0.0882	Splice_Site	.	Non-Targeted Panel Gene	Adult HGBCL, NOS
BCL-1	chr4	75657556	75657556	C	T	splicing	G3BP2	17	2	0.1053	Splice_Site	.	Non-Targeted Panel Gene	Adult HGBCL, NOS
BCL-1	chrX	20187827	20187827	C	T	splicing	RPS6KA3	17	2	0.1053	Splice_Site	.	Non-Targeted Panel Gene	Adult HGBCL, NOS
BCL-1	chr7	48467046	48467046	G	A	splicing	ABCA13	48	6	0.1111	Splice_Site	.	Non-Targeted Panel Gene	Adult HGBCL, NOS
BCL-1	chr2	166410325	166410325	G	A	splicing	SCN7A	24	3	0.1111	Splice_Site	.	Non-Targeted Panel Gene	Adult HGBCL, NOS
BCL-1	chr14	20356032	20356032	G	A	splicing	PARP2	31	4	0.1143	Splice_Site	.	Non-Targeted Panel Gene	Adult HGBCL, NOS
BCL-1	chr2	188993460	188993460	G	A	splicing	COL3A1	15	2	0.1176	Splice_Site	.	Non-Targeted Panel Gene	Adult HGBCL, NOS
BCL-1	chr8	144512143	144512143	C	T	splicing	RECQL4	19	3	0.1364	Splice_Site	.	Non-Targeted Panel Gene	Adult HGBCL, NOS
BCL-1	chr16	57755496	57755496	C	T	splicing	KATNB1	12	2	0.1429	Splice_Site	.	Non-Targeted Panel Gene	Adult HGBCL, NOS
BCL-1	chrX	65529593	65529593	C	T	splicing	LAS1L	12	2	0.1429	Splice_Site	.	Non-Targeted Panel Gene	Adult HGBCL, NOS
BCL-1	chr9	98823426	98823426	G	A	splicing	GALNT12	9	2	0.1818	Splice_Site	.	Non-Targeted Panel Gene	Adult HGBCL, NOS
BCL-1	chrX	65735431	65735431	G	A	splicing	MSN	7	2	0.2222	Splice_Site	.	Non-Targeted Panel Gene	Adult HGBCL, NOS
BCL-1	chr12	109090008	109090008	C	T	splicing	ALKBH2	6	2	0.25	Splice_Site	.	Non-Targeted Panel Gene	Adult HGBCL, NOS
BCL-1	chr22	26506360	26506363	TCTG	-	exonic	TFIP11	60	2	0.0323	Frame_Shift_Del	p.Q154fs	Non-Targeted Panel Gene	Adult HGBCL, NOS
BCL-1	chr5	94940102	94940102	T	-	exonic	MCTP1	12	16	0.5517	Frame_Shift_Del	p.E385fs	Non-Targeted Panel Gene	Adult HGBCL, NOS
BCL-1	chrX	48934747	48934747	-	CC	exonic	OTUD5	9	10	0.5263	Frame_Shift_Ins	p.Y292fs	Non-Targeted Panel Gene	Adult HGBCL, NOS
BCL-1	chr17	50370091	50370091	-	AT	exonic	MRPL27	11	13	0.5417	Frame_Shift_Ins	p.V61fs	Non-Targeted Panel Gene	Adult HGBCL, NOS
BCL-1	chr4	87435029	87435037	TGGTTCTAA	-	exonic	NUDT9	66	2	0.0294	In_Frame_Del	p.2_5del	Non-Targeted Panel Gene	Adult HGBCL, NOS
BCL-1	chrX	41346372	41346372	T	C	exonic	DDX3X	2	88	0.9778	Missense_Mutation	p.F487L	Targerted Panel Gene	Adult HGBCL, NOS
BCL-1	chr1	201209568	201209568	G	C	exonic	IGFN1	269	32	0.1063	Missense_Mutation	p.G1559R	Targerted Panel Gene	Adult HGBCL, NOS
BCL-1	chr17	7674252	7674252	C	T	exonic	TP53	1	133	0.9925	Missense_Mutation	p.M198I	Targerted Panel Gene	Adult HGBCL, NOS
BCL-1	chr3	49375465	49375465	T	G	exonic	RHOA	82	78	0.4875	Missense_Mutation	p.Y42S	Targerted Panel Gene	Adult HGBCL, NOS
BCL-1	chr19	47000599	47000599	C	T	exonic	ARHGAP35	60	2	0.0323	Missense_Mutation	p.P1471S	Non-Targeted Panel Gene	Adult HGBCL, NOS
BCL-1	chr7	15431069	15431069	C	T	exonic	AGMO	58	2	0.0333	Missense_Mutation	p.S150N	Non-Targeted Panel Gene	Adult HGBCL, NOS
BCL-1	chr1	111982053	111982053	G	T	exonic	KCND3	58	2	0.0333	Missense_Mutation	p.A225D	Non-Targeted Panel Gene	Adult HGBCL, NOS
BCL-1	chr14	53046800	53046800	C	T	exonic	DDHD1	57	2	0.0339	Missense_Mutation	p.D891N	Non-Targeted Panel Gene	Adult HGBCL, NOS
BCL-1	chr6	30647256	30647256	G	A	exonic	C6orf136	56	2	0.0345	Missense_Mutation	p.A9T	Non-Targeted Panel Gene	Adult HGBCL, NOS
BCL-1	chr16	23106408	23106408	G	A	exonic	USP31	56	2	0.0345	Missense_Mutation	p.A284V	Non-Targeted Panel Gene	Adult HGBCL, NOS
BCL-1	chr18	58948125	58948125	C	T	exonic	ZNF532	83	3	0.0345	Missense_Mutation	p.R922C	Non-Targeted Panel Gene	Adult HGBCL, NOS
BCL-1	chr19	8909354	8909354	C	T	exonic	MUC16	55	2	0.0351	Missense_Mutation	p.E12489K	Non-Targeted Panel Gene	Adult HGBCL, NOS
BCL-1	chr13	105472626	105472626	G	A	exonic	DAOA	54	2	0.0357	Missense_Mutation	p.R10K	Non-Targeted Panel Gene	Adult HGBCL, NOS
BCL-1	chr2	161204749	161204749	C	A	exonic	TANK	54	2	0.0357	Missense_Mutation	p.Q95K	Non-Targeted Panel Gene	Adult HGBCL, NOS
BCL-1	chr1	147294495	147294495	G	A	exonic	CHD1L	53	2	0.0364	Missense_Mutation	p.A793T	Non-Targeted Panel Gene	Adult HGBCL, NOS
BCL-1	chr7	85012788	85012788	C	G	exonic	SEMA3D	53	2	0.0364	Missense_Mutation	p.E588Q	Non-Targeted Panel Gene	Adult HGBCL, NOS
BCL-1	chr13	42301651	42301651	G	A	exonic	AKAP11	51	2	0.0377	Missense_Mutation	p.E969K	Non-Targeted Panel Gene	Adult HGBCL, NOS
BCL-1	chr14	50998157	50998157	G	A	exonic	TRIM9	51	2	0.0377	Missense_Mutation	p.T499I	Non-Targeted Panel Gene	Adult HGBCL, NOS
BCL-1	chr2	195875683	195875683	C	T	exonic	DNAH7	50	2	0.0385	Missense_Mutation	p.G2093E	Non-Targeted Panel Gene	Adult HGBCL, NOS
BCL-1	chr20	10646001	10646001	C	T	exonic	JAG1	50	2	0.0385	Missense_Mutation	p.D657N	Non-Targeted Panel Gene	Adult HGBCL, NOS
BCL-1	chr5	141477503	141477503	G	A	exonic	PCDHGC3	50	2	0.0385	Missense_Mutation	p.D463N	Non-Targeted Panel Gene	Adult HGBCL, NOS
BCL-1	chr22	41877364	41877364	G	A	exonic	SREBF2	50	2	0.0385	Missense_Mutation	p.D508N	Non-Targeted Panel Gene	Adult HGBCL, NOS
BCL-1	chr17	1726515	1726515	G	A	exonic	WDR81	50	2	0.0385	Missense_Mutation	p.S519N	Non-Targeted Panel Gene	Adult HGBCL, NOS
BCL-1	chr6	106619289	106619289	G	A	exonic	RTN4IP1	74	3	0.039	Missense_Mutation	p.A178V	Non-Targeted Panel Gene	Adult HGBCL, NOS
BCL-1	chr2	232790791	232790791	G	A	exonic	GIGYF2	49	2	0.0392	Missense_Mutation	p.R269Q	Non-Targeted Panel Gene	Adult HGBCL, NOS
BCL-1	chr7	135188459	135188459	C	T	exonic	WDR91	73	3	0.0395	Missense_Mutation	p.V619M	Non-Targeted Panel Gene	Adult HGBCL, NOS
BCL-1	chr2	99360544	99360544	G	A	exonic	EIF5B	48	2	0.04	Missense_Mutation	p.V81M	Non-Targeted Panel Gene	Adult HGBCL, NOS
BCL-1	chr11	105753065	105753065	G	A	exonic	GRIA4	48	2	0.04	Missense_Mutation	p.S111N	Non-Targeted Panel Gene	Adult HGBCL, NOS
BCL-1	chr20	23365557	23365557	G	A	exonic	GZF1	47	2	0.04	Missense_Mutation	p.V392M	Non-Targeted Panel Gene	Adult HGBCL, NOS
BCL-1	chr2	63604807	63604807	G	A	exonic	MDH1	48	2	0.04	Missense_Mutation	p.G204R	Non-Targeted Panel Gene	Adult HGBCL, NOS
BCL-1	chr9	120437428	120437428	C	T	exonic	CDK5RAP2	47	2	0.0408	Missense_Mutation	p.M1274I	Non-Targeted Panel Gene	Adult HGBCL, NOS
BCL-1	chr14	33950403	33950403	C	T	exonic	EGLN3	47	2	0.0408	Missense_Mutation	p.R117K	Non-Targeted Panel Gene	Adult HGBCL, NOS
BCL-1	chr5	141341261	141341261	C	T	exonic	PCDHGA2	93	4	0.0412	Missense_Mutation	p.R764W	Non-Targeted Panel Gene	Adult HGBCL, NOS
BCL-1	chr5	157490436	157490436	G	A	exonic	ADAM19	46	2	0.0417	Missense_Mutation	p.A705V	Non-Targeted Panel Gene	Adult HGBCL, NOS
BCL-1	chr4	174331492	174331492	C	T	exonic	CEP44	46	2	0.0417	Missense_Mutation	p.A366V	Non-Targeted Panel Gene	Adult HGBCL, NOS
BCL-1	chr3	119582401	119582401	C	A	exonic	ADPRH	45	2	0.0426	Missense_Mutation	p.Q78K	Non-Targeted Panel Gene	Adult HGBCL, NOS
BCL-1	chr19	1398848	1398848	G	A	exonic	GAMT	45	2	0.0426	Missense_Mutation	p.A213V	Non-Targeted Panel Gene	Adult HGBCL, NOS
BCL-1	chr7	24680393	24680393	C	T	exonic	MPP6	45	2	0.0426	Missense_Mutation	p.A440V	Non-Targeted Panel Gene	Adult HGBCL, NOS
BCL-1	chr7	104168382	104168382	C	T	exonic	ORC5	45	2	0.0426	Missense_Mutation	p.R323K	Non-Targeted Panel Gene	Adult HGBCL, NOS
BCL-1	chr3	48660849	48660849	C	T	exonic	CELSR3	44	2	0.0435	Missense_Mutation	p.D596N	Non-Targeted Panel Gene	Adult HGBCL, NOS
BCL-1	chr5	151528091	151528091	T	C	exonic	FAT2	43	2	0.0444	Missense_Mutation	p.T3357A	Non-Targeted Panel Gene	Adult HGBCL, NOS
BCL-1	chr6	79202275	79202275	C	T	exonic;splicing	HMGN3;HMGN3	43	2	0.0444	Missense_Mutation	p.V88I	Non-Targeted Panel Gene	Adult HGBCL, NOS
BCL-1	chr13	41193684	41193684	C	T	exonic	KBTBD7	43	2	0.0444	Missense_Mutation	p.A192T	Non-Targeted Panel Gene	Adult HGBCL, NOS
BCL-1	chr14	64209444	64209444	C	A	exonic	SYNE2	42	2	0.0444	Missense_Mutation	p.R6136S	Non-Targeted Panel Gene	Adult HGBCL, NOS
BCL-1	chr16	86554661	86554661	C	T	exonic	MTHFSD	64	3	0.0448	Missense_Mutation	p.R35K	Non-Targeted Panel Gene	Adult HGBCL, NOS
BCL-1	chr11	20364389	20364389	G	A	exonic	HTATIP2	42	2	0.0455	Missense_Mutation	p.G51D	Non-Targeted Panel Gene	Adult HGBCL, NOS
BCL-1	chr12	105031914	105031914	C	G	exonic	ALDH1L2	41	2	0.0465	Missense_Mutation	p.M755I	Non-Targeted Panel Gene	Adult HGBCL, NOS
BCL-1	chr6	100647397	100647397	G	A	exonic	ASCC3	40	2	0.0465	Missense_Mutation	p.P1103S	Non-Targeted Panel Gene	Adult HGBCL, NOS
BCL-1	chr6	41806779	41806779	C	T	exonic	USP49	41	2	0.0465	Missense_Mutation	p.V69I	Non-Targeted Panel Gene	Adult HGBCL, NOS
BCL-1	chr1	9823651	9823651	G	A	exonic	CLSTN1	60	3	0.0469	Missense_Mutation	p.A28V	Non-Targeted Panel Gene	Adult HGBCL, NOS
BCL-1	chr19	14409338	14409338	C	T	exonic	DDX39A	39	2	0.0476	Missense_Mutation	p.D362N	Non-Targeted Panel Gene	Adult HGBCL, NOS
BCL-1	chr22	50240283	50240283	C	T	exonic	TUBGCP6	60	3	0.0476	Missense_Mutation	p.V276M	Non-Targeted Panel Gene	Adult HGBCL, NOS
BCL-1	chr7	48106890	48106890	G	T	exonic	UPP1	40	2	0.0476	Missense_Mutation	p.V152L	Non-Targeted Panel Gene	Adult HGBCL, NOS

BCL-1	chr17	31296184	31296184	G	A	exonic	OMG	59	3	0.0484	Missense_Mutation	p.P50S	Non-Targeted Panel Gene	Adult HGBCL, NOS
BCL-1	chr1	225783345	225783345	G	A	exonic	SRP9	98	5	0.0485	Missense_Mutation	p.V40I	Non-Targeted Panel Gene	Adult HGBCL, NOS
BCL-1	chr11	34097759	34097759	G	A	exonic	CAPRIN1	39	2	0.0488	Missense_Mutation	p.R688Q	Non-Targeted Panel Gene	Adult HGBCL, NOS
BCL-1	chr16	20965032	20965032	T	C	exonic	DNAH3	39	2	0.0488	Missense_Mutation	p.K2951R	Non-Targeted Panel Gene	Adult HGBCL, NOS
BCL-1	chr5	146157748	146157748	C	A	exonic	LARS	39	2	0.0488	Missense_Mutation	p.E273D	Non-Targeted Panel Gene	Adult HGBCL, NOS
BCL-1	chr2	164898558	164898558	C	T	exonic	SLC38A11	38	2	0.0488	Missense_Mutation	p.C367Y	Non-Targeted Panel Gene	Adult HGBCL, NOS
BCL-1	chr2	178577030	178577030	G	A	exonic	TTN	39	2	0.0488	Missense_Mutation	p.T23102I	Non-Targeted Panel Gene	Adult HGBCL, NOS
BCL-1	chr10	101534696	101534696	C	T	exonic	BTRC	77	4	0.0494	Missense_Mutation	p.T378M	Non-Targeted Panel Gene	Adult HGBCL, NOS
BCL-1	chr8	99875502	99875502	C	T	exonic	VPS13B	38	2	0.05	Missense_Mutation	p.P3944S	Non-Targeted Panel Gene	Adult HGBCL, NOS
BCL-1	chr19	57398690	57398690	G	A	exonic	ZNF548	38	2	0.05	Missense_Mutation	p.D147N	Non-Targeted Panel Gene	Adult HGBCL, NOS
BCL-1	chr1	161214182	161214182	G	A	exonic	NDUFS2	56	3	0.0508	Missense_Mutation	p.V461I	Non-Targeted Panel Gene	Adult HGBCL, NOS
BCL-1	chr1	145746894	145746894	C	T	exonic	RNF115	56	3	0.0508	Missense_Mutation	p.S296N	Non-Targeted Panel Gene	Adult HGBCL, NOS
BCL-1	chr11	64299673	64299673	C	T	exonic	KCNK4	37	2	0.0513	Missense_Mutation	p.P377S	Non-Targeted Panel Gene	Adult HGBCL, NOS
BCL-1	chr14	64225339	64225339	C	T	exonic	SYNE2	37	2	0.0513	Missense_Mutation	p.P6846L	Non-Targeted Panel Gene	Adult HGBCL, NOS
BCL-1	chr16	84657537	84657537	G	A	exonic	KLHL36	72	4	0.0526	Missense_Mutation	p.D244N	Non-Targeted Panel Gene	Adult HGBCL, NOS
BCL-1	chr19	50382577	50382577	C	T	exonic	NR1H2	35	2	0.0526	Missense_Mutation	p.S453L	Non-Targeted Panel Gene	Adult HGBCL, NOS
BCL-1	chr4	6323451	6323451	G	A	exonic	PPP2R2C	36	2	0.0526	Missense_Mutation	p.R382C	Non-Targeted Panel Gene	Adult HGBCL, NOS
BCL-1	chr2	9868340	9868340	G	A	exonic	TAF1B	72	4	0.0526	Missense_Mutation	p.S155N	Non-Targeted Panel Gene	Adult HGBCL, NOS
BCL-1	chr12	30991901	30991901	C	T	exonic	TSPAN11	36	2	0.0526	Missense_Mutation	p.R250W	Non-Targeted Panel Gene	Adult HGBCL, NOS
BCL-1	chr19	2137015	2137015	C	T	exonic	AP3D1	53	3	0.0536	Missense_Mutation	p.R117H	Non-Targeted Panel Gene	Adult HGBCL, NOS
BCL-1	chr18	31546408	31546408	C	A	exonic	DSG2	35	2	0.0541	Missense_Mutation	p.L1008I	Non-Targeted Panel Gene	Adult HGBCL, NOS
BCL-1	chr17	30080234	30080234	G	A	exonic	EFCAB5	35	2	0.0541	Missense_Mutation	p.G1064R	Non-Targeted Panel Gene	Adult HGBCL, NOS
BCL-1	chr14	35689275	35689275	G	T	exonic	RALGAPA1	35	2	0.0541	Missense_Mutation	p.P1046T	Non-Targeted Panel Gene	Adult HGBCL, NOS
BCL-1	chr11	8730970	8730970	G	A	exonic	ST5	35	2	0.0541	Missense_Mutation	p.S107L	Non-Targeted Panel Gene	Adult HGBCL, NOS
BCL-1	chr12	885329	885329	C	A	exonic	WNK1	35	2	0.0541	Missense_Mutation	p.L1761I	Non-Targeted Panel Gene	Adult HGBCL, NOS
BCL-1	chr2	218644153	218644153	G	A	exonic	ZNF142	35	2	0.0541	Missense_Mutation	p.P788L	Non-Targeted Panel Gene	Adult HGBCL, NOS
BCL-1	chr8	124977677	124977677	G	A	exonic	ZNF572	35	2	0.0541	Missense_Mutation	p.C470Y	Non-Targeted Panel Gene	Adult HGBCL, NOS
BCL-1	chr3	185586486	185586486	G	A	exonic	SENP2	51	3	0.0545	Missense_Mutation	p.A25T	Non-Targeted Panel Gene	Adult HGBCL, NOS
BCL-1	chr3	132642036	132642036	G	A	exonic	ACAD11	69	4	0.0548	Missense_Mutation	p.S158F	Non-Targeted Panel Gene	Adult HGBCL, NOS
BCL-1	chr12	56244916	56244916	C	A	exonic	ANKRD52	34	2	0.0556	Missense_Mutation	p.A856S	Non-Targeted Panel Gene	Adult HGBCL, NOS
BCL-1	chr13	76886063	76886063	G	A	exonic	KCTD12	34	2	0.0556	Missense_Mutation	p.P29L	Non-Targeted Panel Gene	Adult HGBCL, NOS
BCL-1	chr16	11556648	11556648	G	A	exonic	LITAF	34	2	0.0556	Missense_Mutation	p.A28V	Non-Targeted Panel Gene	Adult HGBCL, NOS
BCL-1	chr4	105473959	105473959	G	A	exonic	PPA2	34	2	0.0556	Missense_Mutation	p.A31V	Non-Targeted Panel Gene	Adult HGBCL, NOS
BCL-1	chr19	9529518	9529518	C	T	exonic	ZNF426	51	3	0.0556	Missense_Mutation	p.C176Y	Non-Targeted Panel Gene	Adult HGBCL, NOS
BCL-1	chr2	178557063	178557063	C	T	exonic	TTN	50	3	0.0566	Missense_Mutation	p.G29364D	Non-Targeted Panel Gene	Adult HGBCL, NOS
BCL-1	chr9	132392169	132392169	G	A	exonic	TTF1	33	2	0.0571	Missense_Mutation	p.H632Y	Non-Targeted Panel Gene	Adult HGBCL, NOS
BCL-1	chr6	158502131	158502131	C	T	exonic	TULP4	33	2	0.0571	Missense_Mutation	p.P823L	Non-Targeted Panel Gene	Adult HGBCL, NOS
BCL-1	chr14	20556706	20556706	G	A	exonic	RNASE9	49	3	0.0577	Missense_Mutation	p.P127S	Non-Targeted Panel Gene	Adult HGBCL, NOS
BCL-1	chr13	24905916	24905916	C	T	exonic	CENPJ	47	3	0.0588	Missense_Mutation	p.E708K	Non-Targeted Panel Gene	Adult HGBCL, NOS
BCL-1	chr1	108937363	108937363	G	A	exonic	CLCC1	32	2	0.0588	Missense_Mutation	p.P316L	Non-Targeted Panel Gene	Adult HGBCL, NOS
BCL-1	chr19	8962467	8962467	C	T	exonic	MUC16	32	2	0.0588	Missense_Mutation	p.S4768N	Non-Targeted Panel Gene	Adult HGBCL, NOS
BCL-1	chr17	31330303	31330303	G	T	exonic	NF1	31	2	0.0588	Missense_Mutation	p.A1873S	Non-Targeted Panel Gene	Adult HGBCL, NOS
BCL-1	chrX	71299975	71299975	G	A	exonic	NONO	32	2	0.0588	Missense_Mutation	p.A439T	Non-Targeted Panel Gene	Adult HGBCL, NOS
BCL-1	chr5	181125246	181125246	G	A	exonic	OR2V1	31	2	0.0588	Missense_Mutation	p.S20F	Non-Targeted Panel Gene	Adult HGBCL, NOS
BCL-1	chr11	65646646	65646646	G	A	exonic	SIPA1	32	2	0.0588	Missense_Mutation	p.D538N	Non-Targeted Panel Gene	Adult HGBCL, NOS
BCL-1	chr12	20862845	20862845	G	A	exonic	SLCO1B3	32	2	0.0588	Missense_Mutation	p.V240I	Non-Targeted Panel Gene	Adult HGBCL, NOS
BCL-1	chr7	2271951	2271951	C	A	exonic	SNX8	32	2	0.0588	Missense_Mutation	p.A147S	Non-Targeted Panel Gene	Adult HGBCL, NOS
BCL-1	chr1	26282776	26282776	G	A	exonic	UBXN11	48	3	0.0588	Missense_Mutation	p.P389S	Non-Targeted Panel Gene	Adult HGBCL, NOS
BCL-1	chr16	71454059	71454059	C	A	exonic	ZNF23	32	2	0.0588	Missense_Mutation	p.G6V	Non-Targeted Panel Gene	Adult HGBCL, NOS
BCL-1	chr11	102696456	102696456	G	A	exonic	MMP27	47	3	0.06	Missense_Mutation	p.P273S	Non-Targeted Panel Gene	Adult HGBCL, NOS
BCL-1	chr15	88858107	88858107	C	T	exonic	ACAN	31	2	0.0606	Missense_Mutation	p.P1841L	Non-Targeted Panel Gene	Adult HGBCL, NOS
BCL-1	chr17	6590519	6590519	C	T	exonic	KIAA0753	31	2	0.0606	Missense_Mutation	p.C851Y	Non-Targeted Panel Gene	Adult HGBCL, NOS
BCL-1	chr18	51176478	51176478	G	A	exonic	MEX3C	31	2	0.0606	Missense_Mutation	p.A618V	Non-Targeted Panel Gene	Adult HGBCL, NOS
BCL-1	chr22	30020228	30020228	G	A	exonic	MTMR3	31	2	0.0606	Missense_Mutation	p.V857I	Non-Targeted Panel Gene	Adult HGBCL, NOS
BCL-1	chr12	54962997	54962997	G	A	exonic	TESPA1	30	2	0.0606	Missense_Mutation	p.P301S	Non-Targeted Panel Gene	Adult HGBCL, NOS
BCL-1	chr11	66294961	66294961	C	T	exonic	TMEM151A	31	2	0.0606	Missense_Mutation	p.R239W	Non-Targeted Panel Gene	Adult HGBCL, NOS
BCL-1	chr1	55097708	55097708	C	A	exonic	USP24	31	2	0.0606	Missense_Mutation	p.V1869L	Non-Targeted Panel Gene	Adult HGBCL, NOS
BCL-1	chr11	10853030	10853030	G	A	exonic	ZBED5	31	2	0.0606	Missense_Mutation	p.A639V	Non-Targeted Panel Gene	Adult HGBCL, NOS
BCL-1	chr2	3702453	3702453	G	A	exonic	ALLC	46	3	0.0612	Missense_Mutation	p.G356R	Non-Targeted Panel Gene	Adult HGBCL, NOS
BCL-1	chr6	100642688	100642688	G	A	exonic	ASCC3	46	3	0.0612	Missense_Mutation	p.S1265F	Non-Targeted Panel Gene	Adult HGBCL, NOS
BCL-1	chr12	49069053	49069053	C	T	exonic	RHEBL1	59	4	0.0615	Missense_Mutation	p.D36N	Non-Targeted Panel Gene	Adult HGBCL, NOS
BCL-1	chr7	87164970	87164970	C	T	exonic	DMTF1	29	2	0.0625	Missense_Mutation	p.T10I	Non-Targeted Panel Gene	Adult HGBCL, NOS
BCL-1	chr12	52647836	52647836	G	A	exonic	KRT2	44	3	0.0625	Missense_Mutation	p.T381I	Non-Targeted Panel Gene	Adult HGBCL, NOS
BCL-1	chr11	47819450	47819450	G	A	exonic	NUP160	30	2	0.0625	Missense_Mutation	p.A429V	Non-Targeted Panel Gene	Adult HGBCL, NOS
BCL-1	chr11	16055876	16055876	C	T	exonic	SOX6	29	2	0.0625	Missense_Mutation	p.S376N	Non-Targeted Panel Gene	Adult HGBCL, NOS
BCL-1	chr7	149224319	149224319	G	A	exonic	ZNF282	30	2	0.0625	Missense_Mutation	p.R563H	Non-Targeted Panel Gene	Adult HGBCL, NOS
BCL-1	chrX	103274410	103274410	C	T	exonic	TCEAL5	44	3	0.0638	Missense_Mutation	p.G52R	Non-Targeted Panel Gene	Adult HGBCL, NOS
BCL-1	chr6	35079842	35079842	G	A	exonic	ANKS1A	29	2	0.0645	Missense_Mutation	p.G820S	Non-Targeted Panel Gene	Adult HGBCL, NOS
BCL-1	chr2	240918783	240918783	G	A	exonic	CROCC2	29	2	0.0645	Missense_Mutation	p.V46M	Non-Targeted Panel Gene	Adult HGBCL, NOS
BCL-1	chr7	128723219	128723219	C	T	exonic	FAM71F1	29	2	0.0645	Missense_Mutation	p.A138V	Non-Targeted Panel Gene	Adult HGBCL, NOS
BCL-1	chr9	95480476	95480476	C	T	exonic	PTCH1	29	2	0.0645	Missense_Mutation	p.V136I	Non-Targeted Panel Gene	Adult HGBCL, NOS
BCL-1	chr19	17504494	17504494	G	A	exonic	SLC27A1	29	2	0.0645	Missense_Mutation	p.G608D	Non-Targeted Panel Gene	Adult HGBCL, NOS
BCL-1	chr19	43733410	43733410	G	T	exonic	SMG9	28	2	0.0645	Missense_Mutation	p.P418Q	Non-Targeted Panel Gene	Adult HGBCL, NOS
BCL-1	chr14	69952232	69952232	C	T	exonic	SMOC1	29	2	0.0645	Missense_Mutation	p.S65F	Non-Targeted Panel Gene	Adult HGBCL, NOS
BCL-1	chr2	197453567	197453567	G	A	exonic	COQ10B	56	4	0.0667	Missense_Mutation	p.A3T	Non-Targeted Panel Gene	Adult HGBCL, NOS
BCL-1	chr11	65607723	65607723	G	A	exonic	MAP3K11	28	2	0.0667	Missense_Mutation	p.P388L	Non-Targeted Panel Gene	Adult HGBCL, NOS
BCL-1	chr4	681099	681099	C	T	exonic	MYL5	28	2	0.0667	Missense_Mutation	p.R127C	Non-Targeted Panel Gene	Adult HGBCL, NOS
BCL-1	chr17	12744222	12744222	C	T	exonic	MYOCD	28	2	0.0667	Missense_Mutation	p.P253S	Non-Targeted Panel Gene	Adult HGBCL, NOS
BCL-1	chr19	49599641	49599641	G	A	exonic	PRR12	28	2	0.0667	Missense_Mutation	p.A1350T	Non-Targeted Panel Gene	Adult HGBCL, NOS
BCL-1	chr16	69339266	69339266	C	T	exonic	COG8	27	2	0.069	Missense_Mutation	p.C96Y	Non-Targeted Panel Gene	Adult HGBCL, NOS
BCL-1	chr19	10256786	10256786	G	A	exonic	MRPL4	27	2	0.069	Missense_Mutation	p.A136T	Non-Targeted Panel Gene	Adult HGBCL, NOS
BCL-1	chr19	55622467	55622467	C	T	exonic	ZNF784	27	2	0.069	Missense_Mutation	p.G86S	Non-Targeted Panel Gene	Adult HGBCL, NOS

BCL-1	chr4	182914961	182914961	C	T	exonic	DCTD	67	5	0.0694	Missense_Mutation	p.R69K	Non-Targeted Panel Gene	Adult HGBCL, NOS
BCL-1	chrX	1309554	1309554	G	A	exonic	CSF2RA	39	3	0.0714	Missense_Mutation	p.V367M	Non-Targeted Panel Gene	Adult HGBCL, NOS
BCL-1	chr1	36322524	36322524	C	T	exonic	EVA1B	26	2	0.0714	Missense_Mutation	p.G90D	Non-Targeted Panel Gene	Adult HGBCL, NOS
BCL-1	chr3	121510218	121510218	G	A	exonic	POLQ	39	3	0.0714	Missense_Mutation	p.T546I	Non-Targeted Panel Gene	Adult HGBCL, NOS
BCL-1	chr3	137765398	137765398	C	T	exonic	SOX14	26	2	0.0714	Missense_Mutation	p.T205I	Non-Targeted Panel Gene	Adult HGBCL, NOS
BCL-1	chr16	67961614	67961614	C	A	exonic	SLC12A4	24	2	0.0741	Missense_Mutation	p.E101D	Non-Targeted Panel Gene	Adult HGBCL, NOS
BCL-1	chr13	36879476	36879476	G	A	exonic	SMAD9	25	2	0.0741	Missense_Mutation	p.P72S	Non-Targeted Panel Gene	Adult HGBCL, NOS
BCL-1	chr12	57004307	57004307	G	T	exonic	ZBTB39	24	2	0.0741	Missense_Mutation	p.P204Q	Non-Targeted Panel Gene	Adult HGBCL, NOS
BCL-1	chr7	36522083	36522083	G	T	exonic	AOAH	24	2	0.0769	Missense_Mutation	p.P519T	Non-Targeted Panel Gene	Adult HGBCL, NOS
BCL-1	chr7	151024395	151024395	C	T	exonic	ATG9B	24	2	0.0769	Missense_Mutation	p.R10K	Non-Targeted Panel Gene	Adult HGBCL, NOS
BCL-1	chr10	101605141	101605141	C	A	exonic	DPCD	23	2	0.0769	Missense_Mutation	p.S22R	Non-Targeted Panel Gene	Adult HGBCL, NOS
BCL-1	chr20	34559776	34559776	C	A	exonic	MAP1LC3A	24	2	0.0769	Missense_Mutation	p.L86M	Non-Targeted Panel Gene	Adult HGBCL, NOS
BCL-1	chr4	158599259	158599259	C	T	exonic	RXFP1	60	5	0.0769	Missense_Mutation	p.L101F	Non-Targeted Panel Gene	Adult HGBCL, NOS
BCL-1	chr16	30722643	30722643	G	A	exonic	SRCAP	36	3	0.0769	Missense_Mutation	p.V1263M	Non-Targeted Panel Gene	Adult HGBCL, NOS
BCL-1	chr9	132901698	132901698	G	A	exonic	TSC1	24	2	0.0769	Missense_Mutation	p.T797M	Non-Targeted Panel Gene	Adult HGBCL, NOS
BCL-1	chr2	106145232	106145232	C	T	exonic	UXS1	48	4	0.0769	Missense_Mutation	p.E139K	Non-Targeted Panel Gene	Adult HGBCL, NOS
BCL-1	chr1	224153200	224153200	G	A	exonic	FBXO28	47	4	0.0784	Missense_Mutation	p.R192Q	Non-Targeted Panel Gene	Adult HGBCL, NOS
BCL-1	chr13	73813276	73813276	G	A	exonic	KLF12	35	3	0.0789	Missense_Mutation	p.P228S	Non-Targeted Panel Gene	Adult HGBCL, NOS
BCL-1	chr10	5451911	5451911	G	A	exonic	NET1	35	3	0.0789	Missense_Mutation	p.V113I	Non-Targeted Panel Gene	Adult HGBCL, NOS
BCL-1	chr12	117056852	117056852	G	A	exonic	TESC	34	3	0.0789	Missense_Mutation	p.L55F	Non-Targeted Panel Gene	Adult HGBCL, NOS
BCL-1	chr2	178568205	178568205	G	A	exonic	TTN	35	3	0.0789	Missense_Mutation	p.A25976V	Non-Targeted Panel Gene	Adult HGBCL, NOS
BCL-1	chr6	75126422	75126422	G	A	exonic	COL12A1	23	2	0.08	Missense_Mutation	p.T2130I	Non-Targeted Panel Gene	Adult HGBCL, NOS
BCL-1	chr21	31999113	31999113	C	T	exonic	HUNK	22	2	0.08	Missense_Mutation	p.P692S	Non-Targeted Panel Gene	Adult HGBCL, NOS
BCL-1	chr14	62849670	62849670	C	T	exonic	KCNH5	46	4	0.08	Missense_Mutation	p.G518S	Non-Targeted Panel Gene	Adult HGBCL, NOS
BCL-1	chr19	50517966	50517966	C	T	exonic	LRRC4B	23	2	0.08	Missense_Mutation	p.V583M	Non-Targeted Panel Gene	Adult HGBCL, NOS
BCL-1	chr15	49037732	49037732	G	A	exonic	SECISBP2L	23	2	0.08	Missense_Mutation	p.P21L	Non-Targeted Panel Gene	Adult HGBCL, NOS
BCL-1	chr7	84011249	84011249	G	A	exonic	SEMA3A	22	2	0.08	Missense_Mutation	p.L287F	Non-Targeted Panel Gene	Adult HGBCL, NOS
BCL-1	chr20	38132395	38132395	C	T	exonic	TGM2	23	2	0.08	Missense_Mutation	p.S574N	Non-Targeted Panel Gene	Adult HGBCL, NOS
BCL-1	chr16	27204135	27204135	G	A	exonic	KDM8	34	3	0.0811	Missense_Mutation	p.G15R	Non-Targeted Panel Gene	Adult HGBCL, NOS
BCL-1	chr4	76897606	76897606	C	T	exonic	SOWAHB	34	3	0.0811	Missense_Mutation	p.G82R	Non-Targeted Panel Gene	Adult HGBCL, NOS
BCL-1	chr7	93468802	93468802	G	A	exonic	CALCR	22	2	0.0833	Missense_Mutation	p.A163V	Non-Targeted Panel Gene	Adult HGBCL, NOS
BCL-1	chrX	114848123	114848123	G	A	exonic	HTR2C	22	2	0.0833	Missense_Mutation	p.R157H	Non-Targeted Panel Gene	Adult HGBCL, NOS
BCL-1	chr6	73363627	73363627	C	T	exonic	KHDC3L	22	2	0.0833	Missense_Mutation	p.R141W	Non-Targeted Panel Gene	Adult HGBCL, NOS
BCL-1	chr18	61554333	61554333	G	A	exonic	CDH20	43	4	0.0851	Missense_Mutation	p.A682T	Non-Targeted Panel Gene	Adult HGBCL, NOS
BCL-1	chr12	63802252	63802252	C	T	exonic	TMEM5	32	3	0.0857	Missense_Mutation	p.A197V	Non-Targeted Panel Gene	Adult HGBCL, NOS
BCL-1	chr6	32761713	32761713	T	G	exonic	HLA-DQB2	42	4	0.087	Missense_Mutation	p.K104T	Non-Targeted Panel Gene	Adult HGBCL, NOS
BCL-1	chr19	47752536	47752536	C	T	exonic	NOP53	21	2	0.087	Missense_Mutation	p.P232S	Non-Targeted Panel Gene	Adult HGBCL, NOS
BCL-1	chr11	63217434	63217434	G	A	exonic	SLC22A25	21	2	0.087	Missense_Mutation	p.T237I	Non-Targeted Panel Gene	Adult HGBCL, NOS
BCL-1	chr18	76849637	76849637	G	A	exonic	ZNF236	21	2	0.087	Missense_Mutation	p.R54H	Non-Targeted Panel Gene	Adult HGBCL, NOS
BCL-1	chr19	5456468	5456468	C	T	exonic	ZNRF4	21	2	0.087	Missense_Mutation	p.P326L	Non-Targeted Panel Gene	Adult HGBCL, NOS
BCL-1	chr17	31330304	31330304	C	T	exonic	NF1	31	3	0.0882	Missense_Mutation	p.A1873V	Non-Targeted Panel Gene	Adult HGBCL, NOS
BCL-1	chr9	20929491	20929491	C	T	exonic	FOCAD	41	4	0.0889	Missense_Mutation	p.A1071V	Non-Targeted Panel Gene	Adult HGBCL, NOS
BCL-1	chr8	41693919	41693919	G	A	exonic	ANK1	30	3	0.0909	Missense_Mutation	p.R1212C	Non-Targeted Panel Gene	Adult HGBCL, NOS
BCL-1	chr2	29073424	29073424	C	T	exonic	C2orf71	30	3	0.0909	Missense_Mutation	p.V280M	Non-Targeted Panel Gene	Adult HGBCL, NOS
BCL-1	chr9	68780234	68780234	G	A	exonic	FAM122A	20	2	0.0909	Missense_Mutation	p.G66S	Non-Targeted Panel Gene	Adult HGBCL, NOS
BCL-1	chr15	73343509	73343509	C	T	exonic	HCN4	19	2	0.0909	Missense_Mutation	p.R362H	Non-Targeted Panel Gene	Adult HGBCL, NOS
BCL-1	chr19	39307944	39307944	G	A	exonic	LRFN1	20	2	0.0909	Missense_Mutation	p.P669S	Non-Targeted Panel Gene	Adult HGBCL, NOS
BCL-1	chr20	43711539	43711539	G	A	exonic	MYBL2	19	2	0.0909	Missense_Mutation	p.A553T	Non-Targeted Panel Gene	Adult HGBCL, NOS
BCL-1	chrX	18940030	18940030	G	A	exonic	PHKA2	39	4	0.093	Missense_Mutation	p.R295C	Non-Targeted Panel Gene	Adult HGBCL, NOS
BCL-1	chr14	21394967	21394967	G	A	exonic	CHD8	29	3	0.0938	Missense_Mutation	p.R1500C	Non-Targeted Panel Gene	Adult HGBCL, NOS
BCL-1	chr20	32898599	32898599	C	T	exonic	EFCAB8	19	2	0.0952	Missense_Mutation	p.P355L	Non-Targeted Panel Gene	Adult HGBCL, NOS
BCL-1	chr19	18071297	18071297	G	A	exonic	IL12RB1	19	2	0.0952	Missense_Mutation	p.P353L	Non-Targeted Panel Gene	Adult HGBCL, NOS
BCL-1	chr3	9949580	9949580	C	T	exonic	PRRT3	19	2	0.0952	Missense_Mutation	p.G179E	Non-Targeted Panel Gene	Adult HGBCL, NOS
BCL-1	chr17	44558157	44558157	G	A	exonic	FZD2	28	3	0.0968	Missense_Mutation	p.E157K	Non-Targeted Panel Gene	Adult HGBCL, NOS
BCL-1	chr13	98721871	98721871	G	A	exonic	SLC15A1	28	3	0.0968	Missense_Mutation	p.A133V	Non-Targeted Panel Gene	Adult HGBCL, NOS
BCL-1	chr10	76944773	76944773	C	T	exonic	KCNMA1	18	2	0.1	Missense_Mutation	p.G759R	Non-Targeted Panel Gene	Adult HGBCL, NOS
BCL-1	chr11	12256915	12256915	G	A	exonic	MICAL2	27	3	0.1	Missense_Mutation	p.C1029Y	Non-Targeted Panel Gene	Adult HGBCL, NOS
BCL-1	chr15	50482039	50482039	G	A	exonic	USP8	18	2	0.1	Missense_Mutation	p.V593M	Non-Targeted Panel Gene	Adult HGBCL, NOS
BCL-1	chr3	130563167	130563167	G	A	exonic	COL6A6	35	4	0.1026	Missense_Mutation	p.S55N	Non-Targeted Panel Gene	Adult HGBCL, NOS
BCL-1	chr11	124224742	124224742	G	A	exonic	OR8G2P	35	4	0.1026	Missense_Mutation	p.G17E	Non-Targeted Panel Gene	Adult HGBCL, NOS
BCL-1	chr8	24331317	24331317	G	A	exonic	ADAM28	26	3	0.1034	Missense_Mutation	p.G424E	Non-Targeted Panel Gene	Adult HGBCL, NOS
BCL-1	chr17	49969485	49969485	G	A	exonic	DLX4	26	3	0.1034	Missense_Mutation	p.C6Y	Non-Targeted Panel Gene	Adult HGBCL, NOS
BCL-1	chr12	110582946	110582946	C	T	exonic	PPTC7	26	3	0.1034	Missense_Mutation	p.G29D	Non-Targeted Panel Gene	Adult HGBCL, NOS
BCL-1	chr5	94692232	94692232	G	A	exonic	SLF1	26	3	0.1034	Missense_Mutation	p.G891S	Non-Targeted Panel Gene	Adult HGBCL, NOS
BCL-1	chr13	25170071	25170071	C	T	exonic	AMER2	17	2	0.1053	Missense_Mutation	p.V398I	Non-Targeted Panel Gene	Adult HGBCL, NOS
BCL-1	chr6	30730511	30730511	C	T	exonic	FLOT1	17	2	0.1053	Missense_Mutation	p.E336K	Non-Targeted Panel Gene	Adult HGBCL, NOS
BCL-1	chr12	120353355	120353355	G	A	exonic	MSI1	17	2	0.1053	Missense_Mutation	p.T226I	Non-Targeted Panel Gene	Adult HGBCL, NOS
BCL-1	chrX	110452361	110452361	G	A	exonic	RTL9	17	2	0.1053	Missense_Mutation	p.A582T	Non-Targeted Panel Gene	Adult HGBCL, NOS
BCL-1	chr8	27879752	27879752	G	A	exonic	SCARA5	17	2	0.1053	Missense_Mutation	p.P390S	Non-Targeted Panel Gene	Adult HGBCL, NOS
BCL-1	chr17	79940914	79940914	C	T	exonic	TBC1D16	17	2	0.1053	Missense_Mutation	p.R750K	Non-Targeted Panel Gene	Adult HGBCL, NOS
BCL-1	chr17	10514057	10514057	C	T	exonic	MYH1	25	3	0.1071	Missense_Mutation	p.V201I	Non-Targeted Panel Gene	Adult HGBCL, NOS
BCL-1	chr11	19934189	19934189	G	A	exonic	NAV2	25	3	0.1071	Missense_Mutation	p.G672R	Non-Targeted Panel Gene	Adult HGBCL, NOS
BCL-1	chr19	56190845	56190845	T	G	exonic	ZSCAN5B	33	4	0.1081	Missense_Mutation	p.K244T	Non-Targeted Panel Gene	Adult HGBCL, NOS
BCL-1	chr12	2651598	2651598	C	T	exonic	CACNA1C	24	3	0.1111	Missense_Mutation	p.L1324F	Non-Targeted Panel Gene	Adult HGBCL, NOS
BCL-1	chr14	90401402	90401402	G	A	exonic	CALM1	16	2	0.1111	Missense_Mutation	p.G60S	Non-Targeted Panel Gene	Adult HGBCL, NOS
BCL-1	chr10	100075973	100075973	C	T	exonic	CPN1	16	2	0.1111	Missense_Mutation	p.D120N	Non-Targeted Panel Gene	Adult HGBCL, NOS
BCL-1	chr5	38258809	38258809	G	A	exonic	EGFLAM	24	3	0.1111	Missense_Mutation	p.G19R	Non-Targeted Panel Gene	Adult HGBCL, NOS
BCL-1	chr22	20992316	20992316	G	A	exonic	LZTR1	16	2	0.1111	Missense_Mutation	p.G366S	Non-Targeted Panel Gene	Adult HGBCL, NOS
BCL-1	chr8	24953542	24953542	C	T	exonic	NEFL	16	2	0.1111	Missense_Mutation	p.E475K	Non-Targeted Panel Gene	Adult HGBCL, NOS
BCL-1	chr8	76705767	76705767	C	T	exonic	ZFHX4	24	3	0.1111	Missense_Mutation	p.A560V	Non-Targeted Panel Gene	Adult HGBCL, NOS
BCL-1	chr19	58489328	58489328	C	T	exonic	ZNF446	16	2	0.1111	Missense_Mutation	p.H281Y	Non-Targeted Panel Gene	Adult HGBCL, NOS
BCL-1	chr2	119559944	119559944	C	T	exonic	CFAP221	23	3	0.1154	Missense_Mutation	p.P115L	Non-Targeted Panel Gene	Adult HGBCL, NOS

BCL-1	chr1	43970600	43970600	C	T	exonic	DPH2	23	3	0.1154	Missense_Mutation	p.A51V	Non-Targeted Panel Gene	Adult HGBCL, NOS
BCL-1	chr1	31739614	31739614	G	A	exonic	ADGRB2	15	2	0.1176	Missense_Mutation	p.P730L	Non-Targeted Panel Gene	Adult HGBCL, NOS
BCL-1	chr2	27055711	27055711	G	A	exonic	AGBL5	30	4	0.1176	Missense_Mutation	p.R313H	Non-Targeted Panel Gene	Adult HGBCL, NOS
BCL-1	chr18	46216337	46216337	G	A	exonic	C18orf25	15	2	0.1176	Missense_Mutation	p.A153T	Non-Targeted Panel Gene	Adult HGBCL, NOS
BCL-1	chr17	79794978	79794978	G	A	exonic	CBX8	15	2	0.1176	Missense_Mutation	p.A276V	Non-Targeted Panel Gene	Adult HGBCL, NOS
BCL-1	chr15	24679322	24679322	G	A	exonic	NPAP1	30	4	0.1176	Missense_Mutation	p.C1152Y	Non-Targeted Panel Gene	Adult HGBCL, NOS
BCL-1	chr14	90890575	90890575	C	T	exonic	RPS6KA5	15	2	0.1176	Missense_Mutation	p.C504Y	Non-Targeted Panel Gene	Adult HGBCL, NOS
BCL-1	chr18	62369293	62369293	C	T	exonic	TNFRSF11A	15	2	0.1176	Missense_Mutation	p.P459L	Non-Targeted Panel Gene	Adult HGBCL, NOS
BCL-1	chr17	78102504	78102504	G	A	exonic	TNRC6C	15	2	0.1176	Missense_Mutation	p.R1547K	Non-Targeted Panel Gene	Adult HGBCL, NOS
BCL-1	chr6	24596240	24596240	C	A	exonic	KIAA0319	22	3	0.12	Missense_Mutation	p.G136V	Non-Targeted Panel Gene	Adult HGBCL, NOS
BCL-1	chr7	128074622	128074622	G	A	exonic	SND1	22	3	0.12	Missense_Mutation	p.A634T	Non-Targeted Panel Gene	Adult HGBCL, NOS
BCL-1	chr17	30922943	30922943	C	T	exonic	ADAP2	14	2	0.125	Missense_Mutation	p.P33L	Non-Targeted Panel Gene	Adult HGBCL, NOS
BCL-1	chr8	52565072	52565072	C	T	exonic	ALKAL1	14	2	0.125	Missense_Mutation	p.S62N	Non-Targeted Panel Gene	Adult HGBCL, NOS
BCL-1	chr2	219232558	219232558	G	T	exonic	ANKZF1	14	2	0.125	Missense_Mutation	p.D145Y	Non-Targeted Panel Gene	Adult HGBCL, NOS
BCL-1	chr7	105614776	105614776	C	T	exonic	ATXN7L1	14	2	0.125	Missense_Mutation	p.A520T	Non-Targeted Panel Gene	Adult HGBCL, NOS
BCL-1	chr16	89738641	89738641	G	A	exonic	FANCA	14	2	0.125	Missense_Mutation	p.A1443V	Non-Targeted Panel Gene	Adult HGBCL, NOS
BCL-1	chr14	85622128	85622128	G	A	exonic	FLRT2	14	2	0.125	Missense_Mutation	p.S205N	Non-Targeted Panel Gene	Adult HGBCL, NOS
BCL-1	chr16	70471233	70471233	C	T	exonic	FUK	14	2	0.125	Missense_Mutation	p.H408Y	Non-Targeted Panel Gene	Adult HGBCL, NOS
BCL-1	chr17	41524878	41524878	C	T	exonic	KRT19	21	3	0.125	Missense_Mutation	p.E209K	Non-Targeted Panel Gene	Adult HGBCL, NOS
BCL-1	chr5	179837650	179837650	C	T	exonic	MRNIP	14	2	0.125	Missense_Mutation	p.G258D	Non-Targeted Panel Gene	Adult HGBCL, NOS
BCL-1	chr8	70156212	70156212	T	C	exonic	NCOA2	14	2	0.125	Missense_Mutation	p.E718G	Non-Targeted Panel Gene	Adult HGBCL, NOS
BCL-1	chr3	122704719	122704719	C	A	exonic	PARP14	14	2	0.125	Missense_Mutation	p.L1171M	Non-Targeted Panel Gene	Adult HGBCL, NOS
BCL-1	chr17	75093639	75093639	G	A	exonic	SLC16A5	14	2	0.125	Missense_Mutation	p.M1I	Non-Targeted Panel Gene	Adult HGBCL, NOS
BCL-1	chr17	62746882	62746882	C	T	exonic	MARCHF10	20	3	0.1304	Missense_Mutation	p.S160N	Non-Targeted Panel Gene	Adult HGBCL, NOS
BCL-1	chr6	44310409	44310409	G	A	exonic	AARS2	20	3	0.1304	Missense_Mutation	p.R262W	Non-Targeted Panel Gene	Adult HGBCL, NOS
BCL-1	chr22	50548557	50548557	C	T	exonic	KLHDC7B	20	3	0.1304	Missense_Mutation	p.R131W	Non-Targeted Panel Gene	Adult HGBCL, NOS
BCL-1	chr9	116697906	116697906	G	A	exonic	TRIM32	20	3	0.1304	Missense_Mutation	p.S55N	Non-Targeted Panel Gene	Adult HGBCL, NOS
BCL-1	chr5	140402179	140402179	G	A	exonic	ANKHD1;ANKHD1-	13	2	0.1333	Missense_Mutation	p.G71E	Non-Targeted Panel Gene	Adult HGBCL, NOS
BCL-1	chr2	105242543	105242543	G	A	exonic	GPR45	13	2	0.1333	Missense_Mutation	p.V229M	Non-Targeted Panel Gene	Adult HGBCL, NOS
BCL-1	chr2	200490255	200490255	C	T	exonic	KCTD18	13	2	0.1333	Missense_Mutation	p.V376M	Non-Targeted Panel Gene	Adult HGBCL, NOS
BCL-1	chr1	149949763	149949763	G	A	exonic	OTUD7B	13	2	0.1333	Missense_Mutation	p.P330L	Non-Targeted Panel Gene	Adult HGBCL, NOS
BCL-1	chr14	64971983	64971983	C	T	exonic	RAB15	13	2	0.1333	Missense_Mutation	p.E32K	Non-Targeted Panel Gene	Adult HGBCL, NOS
BCL-1	chr15	43256590	43256590	G	A	exonic	TGM5	13	2	0.1333	Missense_Mutation	p.P178L	Non-Targeted Panel Gene	Adult HGBCL, NOS
BCL-1	chr1	11501396	11501396	G	A	exonic	DISP3	19	3	0.1364	Missense_Mutation	p.R135Q	Non-Targeted Panel Gene	Adult HGBCL, NOS
BCL-1	chr19	53810878	53810878	C	T	exonic	NLRP12	19	3	0.1364	Missense_Mutation	p.E261K	Non-Targeted Panel Gene	Adult HGBCL, NOS
BCL-1	chr7	108180264	108180264	C	T	exonic	NRCAM	19	3	0.1364	Missense_Mutation	p.G937D	Non-Targeted Panel Gene	Adult HGBCL, NOS
BCL-1	chr4	76896442	76896442	C	T	exonic	SOWAHB	19	3	0.1364	Missense_Mutation	p.V470I	Non-Targeted Panel Gene	Adult HGBCL, NOS
BCL-1	chr7	100746651	100746651	C	T	exonic	ZAN	19	3	0.1364	Missense_Mutation	p.L294F	Non-Targeted Panel Gene	Adult HGBCL, NOS
BCL-1	chr11	130411578	130411578	G	A	exonic	ADAMTS8	12	2	0.1429	Missense_Mutation	p.A530V	Non-Targeted Panel Gene	Adult HGBCL, NOS
BCL-1	chr22	17738133	17738133	G	T	exonic	BID	12	2	0.1429	Missense_Mutation	p.L200M	Non-Targeted Panel Gene	Adult HGBCL, NOS
BCL-1	chr4	15003815	15003815	C	T	exonic	CPEB2	12	2	0.1429	Missense_Mutation	p.T381I	Non-Targeted Panel Gene	Adult HGBCL, NOS
BCL-1	chr6	43186403	43186403	C	A	exonic	CUL9	12	2	0.1429	Missense_Mutation	p.A400D	Non-Targeted Panel Gene	Adult HGBCL, NOS
BCL-1	chr6	43072950	43072950	G	A	exonic	KLC4	18	3	0.1429	Missense_Mutation	p.V539M	Non-Targeted Panel Gene	Adult HGBCL, NOS
BCL-1	chr5	163512056	163512056	C	T	exonic	MAT2B	12	2	0.1429	Missense_Mutation	p.L29F	Non-Targeted Panel Gene	Adult HGBCL, NOS
BCL-1	chr17	29043304	29043304	G	A	exonic	PIPOX	12	2	0.1429	Missense_Mutation	p.A27T	Non-Targeted Panel Gene	Adult HGBCL, NOS
BCL-1	chr1	75791536	75791536	G	A	exonic	RABGGTB	24	4	0.1429	Missense_Mutation	p.G182S	Non-Targeted Panel Gene	Adult HGBCL, NOS
BCL-1	chr17	28969757	28969757	C	T	exonic	SEZ6	12	2	0.1429	Missense_Mutation	p.A352T	Non-Targeted Panel Gene	Adult HGBCL, NOS
BCL-1	chr10	105158971	105158971	C	T	exonic	SORCS3	12	2	0.1429	Missense_Mutation	p.A570V	Non-Targeted Panel Gene	Adult HGBCL, NOS
BCL-1	chr17	3876309	3876309	C	T	exonic	CAMKK1	23	4	0.1481	Missense_Mutation	p.D342N	Non-Targeted Panel Gene	Adult HGBCL, NOS
BCL-1	chr22	31934345	31934345	G	A	exonic	C22orf24	17	3	0.15	Missense_Mutation	p.H86Y	Non-Targeted Panel Gene	Adult HGBCL, NOS
BCL-1	chr10	15214301	15214301	C	A	exonic	FAM171A1	11	2	0.1538	Missense_Mutation	p.E429D	Non-Targeted Panel Gene	Adult HGBCL, NOS
BCL-1	chr4	47403705	47403705	G	A	exonic	GABRB1	11	2	0.1538	Missense_Mutation	p.A277T	Non-Targeted Panel Gene	Adult HGBCL, NOS
BCL-1	chr8	143569819	143569819	C	T	exonic	MROH6	11	2	0.1538	Missense_Mutation	p.A394T	Non-Targeted Panel Gene	Adult HGBCL, NOS
BCL-1	chr13	29025954	29025954	G	A	exonic	MTUS2	11	2	0.1538	Missense_Mutation	p.G429D	Non-Targeted Panel Gene	Adult HGBCL, NOS
BCL-1	chr16	15031869	15031869	G	A	exonic	PDXDC1	11	2	0.1538	Missense_Mutation	p.V512I	Non-Targeted Panel Gene	Adult HGBCL, NOS
BCL-1	chrX	110454320	110454320	C	T	exonic	RTL9	11	2	0.1538	Missense_Mutation	p.L1235F	Non-Targeted Panel Gene	Adult HGBCL, NOS
BCL-1	chr17	28497117	28497117	C	T	exonic	SLC13A2	11	2	0.1538	Missense_Mutation	p.L472F	Non-Targeted Panel Gene	Adult HGBCL, NOS
BCL-1	chr1	151173499	151173499	C	T	exonic	TMOD4	11	2	0.1538	Missense_Mutation	p.A133T	Non-Targeted Panel Gene	Adult HGBCL, NOS
BCL-1	chr8	124553560	124553560	G	A	exonic	MTSS1	16	3	0.1579	Missense_Mutation	p.P571L	Non-Targeted Panel Gene	Adult HGBCL, NOS
BCL-1	chr1	150942921	150942921	C	T	exonic	SETDB1	16	3	0.1579	Missense_Mutation	p.A248V	Non-Targeted Panel Gene	Adult HGBCL, NOS
BCL-1	chr12	56248515	56248515	C	T	exonic	ANKRD52	10	2	0.1667	Missense_Mutation	p.V586I	Non-Targeted Panel Gene	Adult HGBCL, NOS
BCL-1	chr5	180619106	180619106	G	T	exonic	FLT4	10	2	0.1667	Missense_Mutation	p.P922H	Non-Targeted Panel Gene	Adult HGBCL, NOS
BCL-1	chr2	76748907	76748907	G	A	exonic	LRRTM4	10	2	0.1667	Missense_Mutation	p.H521Y	Non-Targeted Panel Gene	Adult HGBCL, NOS
BCL-1	chr1	205569106	205569106	C	T	exonic	MFSD4A	10	2	0.1667	Missense_Mutation	p.R13C	Non-Targeted Panel Gene	Adult HGBCL, NOS
BCL-1	chr17	18145878	18145878	C	T	exonic	MYO15A	10	2	0.1667	Missense_Mutation	p.R2094C	Non-Targeted Panel Gene	Adult HGBCL, NOS
BCL-1	chr2	191386056	191386056	G	T	exonic	MYO1B	10	2	0.1667	Missense_Mutation	p.C509F	Non-Targeted Panel Gene	Adult HGBCL, NOS
BCL-1	chr19	45155817	45155817	C	T	exonic	NKPD1	10	2	0.1667	Missense_Mutation	p.C210Y	Non-Targeted Panel Gene	Adult HGBCL, NOS
BCL-1	chr19	15170141	15170141	C	T	exonic	NOTCH3	10	2	0.1667	Missense_Mutation	p.G1715E	Non-Targeted Panel Gene	Adult HGBCL, NOS
BCL-1	chr15	69055351	69055351	C	T	exonic	NOX5	10	2	0.1667	Missense_Mutation	p.H673Y	Non-Targeted Panel Gene	Adult HGBCL, NOS
BCL-1	chr6	31969018	31969018	G	A	exonic	SKIV2L	10	2	0.1667	Missense_Mutation	p.A1110T	Non-Targeted Panel Gene	Adult HGBCL, NOS
BCL-1	chr8	141218984	141218984	G	A	exonic	SLC45A4	14	3	0.1765	Missense_Mutation	p.T168I	Non-Targeted Panel Gene	Adult HGBCL, NOS
BCL-1	chr15	100879945	100879945	C	T	exonic	ALDH1A3	9	2	0.1818	Missense_Mutation	p.P13L	Non-Targeted Panel Gene	Adult HGBCL, NOS
BCL-1	chr7	27143288	27143288	G	A	exonic	HOXA5	9	2	0.1818	Missense_Mutation	p.A107V	Non-Targeted Panel Gene	Adult HGBCL, NOS
BCL-1	chr19	42352417	42352417	G	A	exonic	MEGF8	9	2	0.1818	Missense_Mutation	p.R1104Q	Non-Targeted Panel Gene	Adult HGBCL, NOS
BCL-1	chr22	29480459	29480459	G	A	exonic	NEFH	9	2	0.1818	Missense_Mutation	p.R66H	Non-Targeted Panel Gene	Adult HGBCL, NOS
BCL-1	chrX	72139628	72139628	G	A	exonic	NHSL2	9	2	0.1818	Missense_Mutation	p.V694M	Non-Targeted Panel Gene	Adult HGBCL, NOS
BCL-1	chr12	132144855	132144855	C	T	exonic	NOC4L	9	2	0.1818	Missense_Mutation	p.S40F	Non-Targeted Panel Gene	Adult HGBCL, NOS
BCL-1	chr2	219635868	219635868	C	T	exonic	SLC4A3	9	2	0.1818	Missense_Mutation	p.A750V	Non-Targeted Panel Gene	Adult HGBCL, NOS
BCL-1	chr5	1293654	1293654	G	A	exonic	TERT	9	2	0.1818	Missense_Mutation	p.T411M	Non-Targeted Panel Gene	Adult HGBCL, NOS
BCL-1	chr9	109166925	109166925	C	T	exonic	FRRS1L	13	3	0.1875	Missense_Mutation	p.D123N	Non-Targeted Panel Gene	Adult HGBCL, NOS
BCL-1	chr11	62910448	62910448	C	T	exonic	CHRM1	8	2	0.2	Missense_Mutation	p.R218Q	Non-Targeted Panel Gene	Adult HGBCL, NOS
BCL-1	chr1	57011272	57011272	G	A	exonic	DAB1	8	2	0.2	Missense_Mutation	p.P482L	Non-Targeted Panel Gene	Adult HGBCL, NOS

BCL-1	chr15	51397500	51397500	C	T	exonic	GLDN	8	2	0.2	Missense_Mutation	p.P240L	Non-Targeted Panel Gene	Adult HGBCL, NOS
BCL-1	chr6	34036023	34036023	G	A	exonic	GRM4	8	2	0.2	Missense_Mutation	p.P563L	Non-Targeted Panel Gene	Adult HGBCL, NOS
BCL-1	chr15	73323212	73323212	G	A	exonic	HCN4	8	2	0.2	Missense_Mutation	p.P961S	Non-Targeted Panel Gene	Adult HGBCL, NOS
BCL-1	chr6	33211438	33211438	C	T	exonic	RING1	8	2	0.2	Missense_Mutation	p.P246S	Non-Targeted Panel Gene	Adult HGBCL, NOS
BCL-1	chr16	1968861	1968861	C	T	exonic	RNF151	8	2	0.2	Missense_Mutation	p.A225V	Non-Targeted Panel Gene	Adult HGBCL, NOS
BCL-1	chr6	46688216	46688216	G	A	exonic	TDRD6	8	2	0.2	Missense_Mutation	p.V30M	Non-Targeted Panel Gene	Adult HGBCL, NOS
BCL-1	chr19	50662587	50662587	C	T	exonic	SHANK1	11	3	0.2143	Missense_Mutation	p.G1955E	Non-Targeted Panel Gene	Adult HGBCL, NOS
BCL-1	chr1	150496624	150496624	G	A	exonic	TARS2	7	2	0.2222	Missense_Mutation	p.G306E	Non-Targeted Panel Gene	Adult HGBCL, NOS
BCL-1	chr12	64824982	64824982	C	T	exonic	TBC1D30	7	2	0.2222	Missense_Mutation	p.R35C	Non-Targeted Panel Gene	Adult HGBCL, NOS
BCL-1	chr17	81547578	81547578	C	T	exonic	FAAP100	9	3	0.25	Missense_Mutation	p.G502S	Non-Targeted Panel Gene	Adult HGBCL, NOS
BCL-1	chr19	42362572	42362572	G	A	exonic	MEGF8	9	3	0.25	Missense_Mutation	p.M2011I	Non-Targeted Panel Gene	Adult HGBCL, NOS
BCL-1	chr1	231209150	231209150	G	A	exonic	TRIM67	6	2	0.25	Missense_Mutation	p.V613M	Non-Targeted Panel Gene	Adult HGBCL, NOS
BCL-1	chr16	88534272	88534272	G	A	exonic	ZFPM1	6	2	0.25	Missense_Mutation	p.G772R	Non-Targeted Panel Gene	Adult HGBCL, NOS
BCL-1	chr8	100240715	100240715	G	T	exonic	SPAG1	66	28	0.2979	Missense_Mutation	p.D865Y	Non-Targeted Panel Gene	Adult HGBCL, NOS
BCL-1	chr1	150579488	150579488	A	C	exonic	MCL1	150	66	0.3056	Missense_Mutation	p.Y15D	Non-Targeted Panel Gene	Adult HGBCL, NOS
BCL-1	chr11	124570753	124570753	A	C	exonic	OR8A1	19	9	0.3103	Missense_Mutation	p.T229P	Non-Targeted Panel Gene	Adult HGBCL, NOS
BCL-1	chr4	15533245	15533245	A	G	exonic	CC2D2A	45	21	0.3182	Missense_Mutation	p.K507E	Non-Targeted Panel Gene	Adult HGBCL, NOS
BCL-1	chr4	169438208	169438208	T	G	exonic	NEK1	34	16	0.32	Missense_Mutation	p.K880T	Non-Targeted Panel Gene	Adult HGBCL, NOS
BCL-1	chr2	214728798	214728798	T	C	exonic	BARD1	27	15	0.3571	Missense_Mutation	p.I738V	Non-Targeted Panel Gene	Adult HGBCL, NOS
BCL-1	chr16	30927416	30927416	A	G	exonic	FBXL19	32	19	0.3654	Missense_Mutation	p.I116V	Non-Targeted Panel Gene	Adult HGBCL, NOS
BCL-1	chr19	31276720	31276720	A	G	exonic	TSHZ3	24	14	0.3684	Missense_Mutation	p.S1025P	Non-Targeted Panel Gene	Adult HGBCL, NOS
BCL-1	chr2	74881744	74881744	G	A	exonic	HK2	10	6	0.375	Missense_Mutation	p.G535E	Non-Targeted Panel Gene	Adult HGBCL, NOS
BCL-1	chr3	158645674	158645674	A	G	exonic	GFM1	29	18	0.383	Missense_Mutation	p.N43D	Non-Targeted Panel Gene	Adult HGBCL, NOS
BCL-1	chr7	42025312	42025312	C	A	exonic	GLI3	20	14	0.4118	Missense_Mutation	p.K436N	Non-Targeted Panel Gene	Adult HGBCL, NOS
BCL-1	chr1	226895515	226895515	T	A	exonic	PSEN2	25	18	0.4186	Missense_Mutation	p.F427Y	Non-Targeted Panel Gene	Adult HGBCL, NOS
BCL-1	chr6	44426689	44426689	C	T	exonic	CDC5L	41	30	0.4225	Missense_Mutation	p.P620S	Non-Targeted Panel Gene	Adult HGBCL, NOS
BCL-1	chr2	102362700	102362700	A	G	exonic	IL18R1	28	21	0.4286	Missense_Mutation	p.I14V	Non-Targeted Panel Gene	Adult HGBCL, NOS
BCL-1	chr12	57628849	57628849	T	C	exonic	B4GALNT1	13	10	0.4348	Missense_Mutation	p.Y289C	Non-Targeted Panel Gene	Adult HGBCL, NOS
BCL-1	chr2	39013966	39013966	G	A	exonic	SOS1	30	24	0.4444	Missense_Mutation	p.P655L	Non-Targeted Panel Gene	Adult HGBCL, NOS
BCL-1	chr2	178555065	178555065	G	A	exonic	TTN	15	12	0.4444	Missense_Mutation	p.S29465F	Non-Targeted Panel Gene	Adult HGBCL, NOS
BCL-1	chr1	159934744	159934744	T	C	exonic	IGSF9	22	18	0.45	Missense_Mutation	p.E251G	Non-Targeted Panel Gene	Adult HGBCL, NOS
BCL-1	chr12	46778584	46778584	C	A	exonic	SLC38A4	17	14	0.4516	Missense_Mutation	p.G304C	Non-Targeted Panel Gene	Adult HGBCL, NOS
BCL-1	chr15	99690352	99690352	A	G	exonic	MEF2A	18	15	0.4545	Missense_Mutation	p.N261S	Non-Targeted Panel Gene	Adult HGBCL, NOS
BCL-1	chr20	53255986	53255986	G	T	exonic	TSHZ2	10	9	0.4737	Missense_Mutation	p.G843V	Non-Targeted Panel Gene	Adult HGBCL, NOS
BCL-1	chr7	134934052	134934052	T	C	exonic	CALD1	15	14	0.4828	Missense_Mutation	p.L428P	Non-Targeted Panel Gene	Adult HGBCL, NOS
BCL-1	chr3	38885402	38885402	T	G	exonic	SCN11A	24	23	0.4894	Missense_Mutation	p.K984Q	Non-Targeted Panel Gene	Adult HGBCL, NOS
BCL-1	chr2	38937399	38937399	G	T	exonic	ARHGEF33	5	5	0.5	Missense_Mutation	p.Q210H	Non-Targeted Panel Gene	Adult HGBCL, NOS
BCL-1	chr17	18021850	18021850	C	T	exonic	ATPAF2	9	9	0.5	Missense_Mutation	p.V171M	Non-Targeted Panel Gene	Adult HGBCL, NOS
BCL-1	chr9	5126715	5126715	A	G	exonic	JAK2	32	32	0.5	Missense_Mutation	p.N1108S	Non-Targeted Panel Gene	Adult HGBCL, NOS
BCL-1	chr8	142664692	142664692	G	A	exonic	JRK	16	16	0.5	Missense_Mutation	p.T456M	Non-Targeted Panel Gene	Adult HGBCL, NOS
BCL-1	chr12	21054594	21054594	T	A	exonic	SLCO1B7	28	30	0.5172	Missense_Mutation	p.L500H	Non-Targeted Panel Gene	Adult HGBCL, NOS
BCL-1	chr16	89738893	89738893	G	C	exonic	FANCA	67	72	0.518	Missense_Mutation	p.T1418R	Non-Targeted Panel Gene	Adult HGBCL, NOS
BCL-1	chr10	86912289	86912289	G	C	exonic	BMPR1A	49	56	0.5333	Missense_Mutation	p.E194Q	Non-Targeted Panel Gene	Adult HGBCL, NOS
BCL-1	chr17	81868726	81868726	C	T	exonic	ARHGDIA	5	6	0.5455	Missense_Mutation	p.R218Q	Non-Targeted Panel Gene	Adult HGBCL, NOS
BCL-1	chr6	24437175	24437175	T	C	exonic	GPLD1	25	31	0.5536	Missense_Mutation	p.D712G	Non-Targeted Panel Gene	Adult HGBCL, NOS
BCL-1	chr4	6301692	6301692	G	C	exonic	WFS1	10	15	0.6	Missense_Mutation	p.V633L	Non-Targeted Panel Gene	Adult HGBCL, NOS
BCL-1	chr9	137018735	137018735	G	C	exonic	ABCA2	5	9	0.6429	Missense_Mutation	p.N631K	Non-Targeted Panel Gene	Adult HGBCL, NOS
BCL-1	chr1	45607588	45607588	A	C	exonic	NASP	6	12	0.6667	Missense_Mutation	p.E226A	Non-Targeted Panel Gene	Adult HGBCL, NOS
BCL-1	chr9	122093271	122093271	T	C	exonic	TTLL11	5	10	0.6667	Missense_Mutation	p.N50D	Non-Targeted Panel Gene	Adult HGBCL, NOS
BCL-1	chr1	10304582	10304582	C	T	exonic	KIF1B	9	19	0.6786	Missense_Mutation	p.P1133S	Non-Targeted Panel Gene	Adult HGBCL, NOS
BCL-1	chr17	14206977	14206977	G	T	exonic	COX10	7	16	0.6957	Missense_Mutation	p.V366L	Non-Targeted Panel Gene	Adult HGBCL, NOS
BCL-1	chr20	8802095	8802095	G	T	exonic	PLCB1	71	73	0.5069	Nonsense_Mutation	p.E1155X	Targerted Panel Gene	Adult HGBCL, NOS
BCL-1	chr4	4417369	4417369	G	A	exonic	NSG1	49	2	0.0392	Nonsense_Mutation	p.W164X	Non-Targeted Panel Gene	Adult HGBCL, NOS
BCL-1	chr14	104177586	104177586	G	T	exonic	KIF26A	47	2	0.04	Nonsense_Mutation	p.E1600X	Non-Targeted Panel Gene	Adult HGBCL, NOS
BCL-1	chr10	102424052	102424052	G	A	exonic	CUEDC2	46	2	0.0417	Nonsense_Mutation	p.Q180X	Non-Targeted Panel Gene	Adult HGBCL, NOS
BCL-1	chr17	8797416	8797416	G	A	exonic	MFSD6L	45	2	0.0426	Nonsense_Mutation	p.Q569X	Non-Targeted Panel Gene	Adult HGBCL, NOS
BCL-1	chr12	98614313	98614313	G	A	exonic	IKBIP	53	3	0.0526	Nonsense_Mutation	p.Q109X	Non-Targeted Panel Gene	Adult HGBCL, NOS
BCL-1	chr20	63928361	63928361	C	T	exonic	DNAJC5	29	2	0.0645	Nonsense_Mutation	p.Q6X	Non-Targeted Panel Gene	Adult HGBCL, NOS
BCL-1	chr2	68822563	68822563	C	A	exonic	ARHGAP25	26	2	0.0714	Nonsense_Mutation	p.S475X	Non-Targeted Panel Gene	Adult HGBCL, NOS
BCL-1	chrX	123668275	123668275	G	A	exonic	THOC2	48	4	0.0769	Nonsense_Mutation	p.R301X	Non-Targeted Panel Gene	Adult HGBCL, NOS
BCL-1	chr10	73291412	73291412	G	A	exonic	CFAP70	34	3	0.0811	Nonsense_Mutation	p.R755X	Non-Targeted Panel Gene	Adult HGBCL, NOS
BCL-1	chr17	7906872	7906872	C	T	exonic	CHD3	22	2	0.0833	Nonsense_Mutation	p.Q1503X	Non-Targeted Panel Gene	Adult HGBCL, NOS
BCL-1	chr11	4989726	4989726	C	T	exonic	MMP26	22	2	0.0833	Nonsense_Mutation	p.Q60X	Non-Targeted Panel Gene	Adult HGBCL, NOS
BCL-1	chr5	157813195	157813195	G	A	exonic	CLINT1	46	5	0.098	Nonsense_Mutation	p.R129X	Non-Targeted Panel Gene	Adult HGBCL, NOS
BCL-1	chrX	85957918	85957918	G	A	exonic	CHM	36	4	0.1	Nonsense_Mutation	p.R145X	Non-Targeted Panel Gene	Adult HGBCL, NOS
BCL-1	chr17	37330348	37330348	G	A	exonic	ACACA	35	4	0.1026	Nonsense_Mutation	p.R18X	Non-Targeted Panel Gene	Adult HGBCL, NOS
BCL-1	chr6	34606620	34606620	G	A	exonic	C6orf106	23	3	0.1154	Nonsense_Mutation	p.Q266X	Non-Targeted Panel Gene	Adult HGBCL, NOS
BCL-1	chr12	119672330	119672330	G	A	exonic	PRKAB1	15	2	0.1176	Nonsense_Mutation	p.W63X	Non-Targeted Panel Gene	Adult HGBCL, NOS
BCL-1	chr1	152410511	152410511	G	A	exonic	CRNN	21	3	0.12	Nonsense_Mutation	p.Q191X	Non-Targeted Panel Gene	Adult HGBCL, NOS
BCL-1	chr11	45227068	45227068	G	T	exonic	PRDM11	14	2	0.125	Nonsense_Mutation	p.E815X	Non-Targeted Panel Gene	Adult HGBCL, NOS
BCL-1	chr1	35895132	35895132	C	T	exonic	AGO1	13	2	0.1333	Nonsense_Mutation	p.Q295X	Non-Targeted Panel Gene	Adult HGBCL, NOS
BCL-1	chr11	66021599	66021599	G	A	exonic	CATSPER1	13	2	0.1333	Nonsense_Mutation	p.Q530X	Non-Targeted Panel Gene	Adult HGBCL, NOS
BCL-1	chr20	44626497	44626497	C	T	exonic	ADA	12	2	0.1429	Nonsense_Mutation	p.W11X	Non-Targeted Panel Gene	Adult HGBCL, NOS
BCL-1	chr16	58574748	58574748	G	A	exonic	CNOT1	12	2	0.1429	Nonsense_Mutation	p.Q614X	Non-Targeted Panel Gene	Adult HGBCL, NOS
BCL-1	chr2	100988181	100988181	C	T	exonic	NPAS2	16	3	0.1579	Nonsense_Mutation	p.Q578X	Non-Targeted Panel Gene	Adult HGBCL, NOS
BCL-1	chr1	35563219	35563219	G	A	exonic	NCDN	8	2	0.2	Nonsense_Mutation	p.W468X	Non-Targeted Panel Gene	Adult HGBCL, NOS
BCL-1	chr19	4525773	4525773	G	A	exonic	PLIN5	8	2	0.2	Nonsense_Mutation	p.Q194X	Non-Targeted Panel Gene	Adult HGBCL, NOS
BCL-1	chr17	74592189	74592189	G	A	exonic	C17orf77;CD300LD	18	12	0.4	Nonsense_Mutation	p.W48X	Non-Targeted Panel Gene	Adult HGBCL, NOS
BCL-10	chr6	106088450	106088450	G	A	splicing	PRDM1	19	55	0.7432	Splice_Site	.	Targerted Panel Gene	Adult HGBCL, NOS
BCL-10	chr17	40141568	40141568	A	T	splicing	CASC3	39	2	0.0488	Splice_Site	.	Non-Targeted Panel Gene	Adult HGBCL, NOS
BCL-10	chr16	66995830	66995830	G	A	splicing	CES4A	31	3	0.0882	Splice_Site	.	Non-Targeted Panel Gene	Adult HGBCL, NOS
BCL-10	chr7	144051308	144051309	AG	-	exonic	OR2A5	37	25	0.4032	Frame_Shift_Del	p.R303fs	Non-Targeted Panel Gene	Adult HGBCL, NOS

BCL-10	chr7	51190896	51190907	CAGGGACTTGGA	-	exonic	COBL	56	29	0.3412	In_Frame_Del	p.210_213del	Non-Targeted Panel Gene	Adult HGBCL, NOS
BCL-10	chr5	13780894	13780894	-	TTT	exonic	DNAH5	60	28	0.3182	In_Frame_Ins	p.K2962delinsKK	Non-Targeted Panel Gene	Adult HGBCL, NOS
BCL-10	chr3	38140768	38140768	T	C	exonic	MYD88	60	42	0.4118	Missense_Mutation	p.M232T	Targerted Panel Gene	Adult HGBCL, NOS
BCL-10	chr17	63929438	63929438	T	G	exonic	CD79B	31	15	0.3261	Missense_Mutation	p.Y197S	Targerted Panel Gene	Adult HGBCL, NOS
BCL-10	chr17	63929230	63929230	T	G	exonic	CD79B	16	8	0.3333	Missense_Mutation	p.E230A	Targerted Panel Gene	Adult HGBCL, NOS
BCL-10	chr17	63929243	63929243	G	C	exonic	CD79B	19	11	0.3667	Missense_Mutation	p.H226D	Targerted Panel Gene	Adult HGBCL, NOS
BCL-10	chr7	83155436	83155436	G	T	exonic	PCLO	50	30	0.375	Missense_Mutation	p.A402D	Targerted Panel Gene	Adult HGBCL, NOS
BCL-10	chr7	2944517	2944517	G	A	exonic	CARD11	15	10	0.4	Missense_Mutation	p.L127F	Targerted Panel Gene	Adult HGBCL, NOS
BCL-10	chr2	96144175	96144175	G	C	exonic	DUSP2	79	56	0.4148	Missense_Mutation	p.Q237E	Targerted Panel Gene	Adult HGBCL, NOS
BCL-10	chr11	118489802	118489802	A	T	exonic	KMT2A	23	20	0.4651	Missense_Mutation	p.E1497V	Targerted Panel Gene	Adult HGBCL, NOS
BCL-10	chr11	118489819	118489819	A	G	exonic	KMT2A	24	21	0.4667	Missense_Mutation	p.N1503D	Targerted Panel Gene	Adult HGBCL, NOS
BCL-10	chr13	38691533	38691533	G	T	exonic	FREM2	57	55	0.4911	Missense_Mutation	p.D1397Y	Targerted Panel Gene	Adult HGBCL, NOS
BCL-10	chr15	93009227	93009227	T	C	exonic	CHD2	47	60	0.5607	Missense_Mutation	p.L1499P	Targerted Panel Gene	Adult HGBCL, NOS
BCL-10	chr19	6586103	6586103	G	A	exonic	CD70	11	106	0.906	Missense_Mutation	p.L167F	Targerted Panel Gene	Adult HGBCL, NOS
BCL-10	chr3	185919473	185919473	C	T	exonic	TRA2B	54	2	0.0357	Missense_Mutation	p.G249E	Non-Targeted Panel Gene	Adult HGBCL, NOS
BCL-10	chr6	109516474	109516474	C	T	exonic	AK9	53	2	0.0364	Missense_Mutation	p.G1268R	Non-Targeted Panel Gene	Adult HGBCL, NOS
BCL-10	chr11	116821207	116821207	C	T	exonic	APOA4	53	2	0.0364	Missense_Mutation	p.G284E	Non-Targeted Panel Gene	Adult HGBCL, NOS
BCL-10	chr6	31889551	31889551	C	T	exonic	EHMT2	48	2	0.04	Missense_Mutation	p.E306K	Non-Targeted Panel Gene	Adult HGBCL, NOS
BCL-10	chr1	197055877	197055877	G	A	exonic	F13B	48	2	0.04	Missense_Mutation	p.H398Y	Non-Targeted Panel Gene	Adult HGBCL, NOS
BCL-10	chr17	41190328	41190328	T	A	exonic	KRTAP9-1	42	2	0.0455	Missense_Mutation	p.S148T	Non-Targeted Panel Gene	Adult HGBCL, NOS
BCL-10	chr12	120737017	120737017	C	T	exonic	ACADS	35	2	0.0541	Missense_Mutation	p.A81V	Non-Targeted Panel Gene	Adult HGBCL, NOS
BCL-10	chr16	67236993	67236993	C	T	exonic	FHOD1	51	3	0.0556	Missense_Mutation	p.G372D	Non-Targeted Panel Gene	Adult HGBCL, NOS
BCL-10	chr19	7647511	7647511	G	A	exonic	STXBP2	41	3	0.0682	Missense_Mutation	p.G566S	Non-Targeted Panel Gene	Adult HGBCL, NOS
BCL-10	chr6	69048295	69048295	G	A	exonic	ADGRB3	25	2	0.0741	Missense_Mutation	p.V740I	Non-Targeted Panel Gene	Adult HGBCL, NOS
BCL-10	chr4	185436324	185436324	G	A	exonic	C4orf47	13	2	0.1333	Missense_Mutation	p.A167T	Non-Targeted Panel Gene	Adult HGBCL, NOS
BCL-10	chr17	3449126	3449126	G	A	exonic	SPATA22	12	2	0.1429	Missense_Mutation	p.T118I	Non-Targeted Panel Gene	Adult HGBCL, NOS
BCL-10	chr18	63407471	63407471	C	T	exonic	VPS4B	10	2	0.1667	Missense_Mutation	p.D109N	Non-Targeted Panel Gene	Adult HGBCL, NOS
BCL-10	chr10	46550408	46550408	C	T	exonic	GPRIN2	45	17	0.2742	Missense_Mutation	p.R110Q	Non-Targeted Panel Gene	Adult HGBCL, NOS
BCL-10	chr15	67825940	67825940	G	A	exonic	SKOR1	39	18	0.3158	Missense_Mutation	p.G224S	Non-Targeted Panel Gene	Adult HGBCL, NOS
BCL-10	chr15	73927060	73927060	C	T	exonic	LOXL1	12	6	0.3333	Missense_Mutation	p.R93W	Non-Targeted Panel Gene	Adult HGBCL, NOS
BCL-10	chr4	88139884	88139884	T	G	exonic	ABCG2	35	18	0.3396	Missense_Mutation	p.S38R	Non-Targeted Panel Gene	Adult HGBCL, NOS
BCL-10	chr4	119037164	119037164	C	T	exonic	SYNPO2	22	12	0.3529	Missense_Mutation	p.P1102L	Non-Targeted Panel Gene	Adult HGBCL, NOS
BCL-10	chr15	34870887	34870887	A	C	exonic	AQR	18	11	0.3793	Missense_Mutation	p.F1211L	Non-Targeted Panel Gene	Adult HGBCL, NOS
BCL-10	chr4	15625608	15625608	A	C	exonic	FBXL5	26	16	0.381	Missense_Mutation	p.H498Q	Non-Targeted Panel Gene	Adult HGBCL, NOS
BCL-10	chr1	180020218	180020218	C	G	exonic	CEP350	32	20	0.3846	Missense_Mutation	p.A815G	Non-Targeted Panel Gene	Adult HGBCL, NOS
BCL-10	chr4	102304999	102304999	G	A	exonic	SLC39A8	8	5	0.3846	Missense_Mutation	p.T222I	Non-Targeted Panel Gene	Adult HGBCL, NOS
BCL-10	chr17	63421933	63421933	C	T	exonic	TANC2	64	41	0.3905	Missense_Mutation	p.P1984L	Non-Targeted Panel Gene	Adult HGBCL, NOS
BCL-10	chr5	141625705	141625705	C	T	exonic	HDAC3	155	100	0.3922	Missense_Mutation	p.E347K	Non-Targeted Panel Gene	Adult HGBCL, NOS
BCL-10	chr11	55820350	55820350	A	G	exonic	OR5D18	9	6	0.4	Missense_Mutation	p.T241A	Non-Targeted Panel Gene	Adult HGBCL, NOS
BCL-10	chr16	23636231	23636231	C	G	exonic	PALB2	40	27	0.403	Missense_Mutation	p.E105D	Non-Targeted Panel Gene	Adult HGBCL, NOS
BCL-10	chr16	722333	722333	C	T	exonic	FAM173A	26	18	0.4091	Missense_Mutation	p.R162W	Non-Targeted Panel Gene	Adult HGBCL, NOS
BCL-10	chr1	51408191	51408191	C	T	exonic	EPS15	37	26	0.4127	Missense_Mutation	p.A473T	Non-Targeted Panel Gene	Adult HGBCL, NOS
BCL-10	chr16	89199042	89199042	G	C	exonic	SLC22A31	33	24	0.4211	Missense_Mutation	p.L37V	Non-Targeted Panel Gene	Adult HGBCL, NOS
BCL-10	chr4	947749	947749	C	T	exonic	TMEM175	15	11	0.4231	Missense_Mutation	p.P4S	Non-Targeted Panel Gene	Adult HGBCL, NOS
BCL-10	chr1	100277299	100277299	C	T	exonic	RTCA	90	68	0.4304	Missense_Mutation	p.S274L	Non-Targeted Panel Gene	Adult HGBCL, NOS
BCL-10	chr9	37744727	37744727	A	T	exonic	FRMPD1	34	26	0.4333	Missense_Mutation	p.T899S	Non-Targeted Panel Gene	Adult HGBCL, NOS
BCL-10	chr5	33658328	33658328	T	G	exonic	ADAMTS12	56	44	0.44	Missense_Mutation	p.D349A	Non-Targeted Panel Gene	Adult HGBCL, NOS
BCL-10	chr18	65880668	65880668	T	G	exonic	CDH7	28	22	0.44	Missense_Mutation	p.I711S	Non-Targeted Panel Gene	Adult HGBCL, NOS
BCL-10	chr5	151822915	151822915	C	T	exonic	GLRA1	70	55	0.44	Missense_Mutation	p.G378S	Non-Targeted Panel Gene	Adult HGBCL, NOS
BCL-10	chr8	43122177	43122177	A	G	exonic	POMK	56	44	0.44	Missense_Mutation	p.D118G	Non-Targeted Panel Gene	Adult HGBCL, NOS
BCL-10	chr2	38779072	38779072	A	G	exonic	GEMIN6	52	41	0.4409	Missense_Mutation	p.N28D	Non-Targeted Panel Gene	Adult HGBCL, NOS
BCL-10	chr15	85580826	85580826	A	G	exonic	AKAP13	30	24	0.4444	Missense_Mutation	p.S920G	Non-Targeted Panel Gene	Adult HGBCL, NOS
BCL-10	chr8	6835454	6835454	C	T	exonic	XKR5	5	4	0.4444	Missense_Mutation	p.A14T	Non-Targeted Panel Gene	Adult HGBCL, NOS
BCL-10	chr14	73246074	73246074	G	A	exonic	PAPLN	6	5	0.4545	Missense_Mutation	p.S78N	Non-Targeted Panel Gene	Adult HGBCL, NOS
BCL-10	chr16	89280675	89280675	T	C	exonic	ANKRD11	38	32	0.4571	Missense_Mutation	p.Q1956R	Non-Targeted Panel Gene	Adult HGBCL, NOS
BCL-10	chr11	56663738	56663738	C	A	exonic	OR5AR1	50	43	0.4624	Missense_Mutation	p.T18N	Non-Targeted Panel Gene	Adult HGBCL, NOS
BCL-10	chr2	160016701	160016701	C	G	exonic	PLA2R1	32	31	0.4921	Missense_Mutation	p.W488C	Non-Targeted Panel Gene	Adult HGBCL, NOS
BCL-10	chr16	88434042	88434042	A	G	exonic	ZNF469	35	34	0.4928	Missense_Mutation	p.D2163G	Non-Targeted Panel Gene	Adult HGBCL, NOS
BCL-10	chr2	219223912	219223912	G	A	exonic	ATG9A	113	111	0.4955	Missense_Mutation	p.S459L	Non-Targeted Panel Gene	Adult HGBCL, NOS
BCL-10	chr13	87676950	87676950	C	T	exonic	SLITRK5	59	58	0.4957	Missense_Mutation	p.P521L	Non-Targeted Panel Gene	Adult HGBCL, NOS
BCL-10	chr11	34071770	34071770	G	C	exonic	CAPRIN1	11	11	0.5	Missense_Mutation	p.R87S	Non-Targeted Panel Gene	Adult HGBCL, NOS
BCL-10	chr3	165830318	165830318	G	A	exonic	BCHE	21	22	0.5116	Missense_Mutation	p.P239L	Non-Targeted Panel Gene	Adult HGBCL, NOS
BCL-10	chr7	12337083	12337083	C	A	exonic	VWDE	25	27	0.5192	Missense_Mutation	p.S1488I	Non-Targeted Panel Gene	Adult HGBCL, NOS
BCL-10	chr15	101178472	101178472	A	G	exonic	CHSY1	43	47	0.5222	Missense_Mutation	p.M442T	Non-Targeted Panel Gene	Adult HGBCL, NOS
BCL-10	chr8	17085292	17085292	A	G	exonic	MICU3	52	57	0.5229	Missense_Mutation	p.M251V	Non-Targeted Panel Gene	Adult HGBCL, NOS
BCL-10	chr9	91723689	91723689	G	C	exonic	ROR2	15	17	0.5312	Missense_Mutation	p.D935E	Non-Targeted Panel Gene	Adult HGBCL, NOS
BCL-10	chr1	171785906	171785906	T	C	exonic	METTL13	46	53	0.5354	Missense_Mutation	p.L314P	Non-Targeted Panel Gene	Adult HGBCL, NOS
BCL-10	chr20	3237566	3237566	C	A	exonic	SLC4A11	64	76	0.5429	Missense_Mutation	p.Q65H	Non-Targeted Panel Gene	Adult HGBCL, NOS
BCL-10	chr16	31490477	31490477	A	G	exonic	SLC5A2	26	34	0.5667	Missense_Mutation	p.N654S	Non-Targeted Panel Gene	Adult HGBCL, NOS
BCL-10	chr1	1072406	1072406	C	G	exonic	RNF223	22	29	0.5686	Missense_Mutation	p.C54S	Non-Targeted Panel Gene	Adult HGBCL, NOS
BCL-10	chr11	102790760	102790760	T	C	exonic	MMP1	21	28	0.5714	Missense_Mutation	p.I415V	Non-Targeted Panel Gene	Adult HGBCL, NOS
BCL-10	chr1	20633904	20633904	G	A	exonic	PINK1	7	10	0.5882	Missense_Mutation	p.R119Q	Non-Targeted Panel Gene	Adult HGBCL, NOS
BCL-10	chr6	137206153	137206153	A	G	exonic	IFNGR1	3	31	0.9118	Missense_Mutation	p.F119S	Non-Targeted Panel Gene	Adult HGBCL, NOS
BCL-10	chrX	115190982	115190982	G	T	exonic	RBMXL3	5	59	0.9219	Missense_Mutation	p.R514L	Non-Targeted Panel Gene	Adult HGBCL, NOS
BCL-10	chrX	105219223	105219223	A	T	exonic	TEX13A	0	129	1	Missense_Mutation	p.V324D	Non-Targeted Panel Gene	Adult HGBCL, NOS
BCL-10	chr11	92882766	92882766	C	T	exonic	FAT3	95	76	0.4444	Nonsense_Mutation	p.R4104X	Targerted Panel Gene	Adult HGBCL, NOS
BCL-10	chr6	137877221	137877221	G	A	exonic	TNFAIP3	7	29	0.8056	Nonsense_Mutation	p.W317X	Targerted Panel Gene	Adult HGBCL, NOS
BCL-10	chr6	75284516	75284516	C	T	exonic	TMEM30A	2	30	0.9375	Nonsense_Mutation	p.W41X	Targerted Panel Gene	Adult HGBCL, NOS
BCL-10	chr6	109443598	109443598	G	A	exonic	SMPD2	19	2	0.0952	Nonsense_Mutation	p.W322X	Non-Targeted Panel Gene	Adult HGBCL, NOS
BCL-10	chr2	62859288	62859288	G	T	exonic	EHBP1	20	16	0.4444	Nonsense_Mutation	p.E287X	Non-Targeted Panel Gene	Adult HGBCL, NOS
BCL-10	chr4	5015208	5015208	G	T	exonic	CYTL1	31	39	0.5571	Nonsense_Mutation	p.C118X	Non-Targeted Panel Gene	Adult HGBCL, NOS
BCL-11	chr4	93769452	93769452	T	G	splicing	GRID2	109	77	0.414	Splice_Site	.	Targerted Panel Gene	Adult HGBCL, NOS

BCL-11	chr11	128463507	128463507	A	C	splicing	ETS1	22	22	0.5	Splice_Site	.	Targerted Panel Gene	Adult HGBCL, NOS
BCL-11	chr12	19320019	19320019	A	C	splicing	PLEKHA5	45	30	0.4	Splice_Site	.	Non-Targeted Panel Gene	Adult HGBCL, NOS
BCL-11	chr1	20345594	20345594	G	C	splicing	VWA5B1	16	22	0.5789	Splice_Site	.	Non-Targeted Panel Gene	Adult HGBCL, NOS
BCL-11	chrX	126551193	126551193	A	C	splicing	DCAF12L1	3	14	0.8235	Splice_Site	.	Non-Targeted Panel Gene	Adult HGBCL, NOS
BCL-11	chr2	74455592	74455604	CCAAGAGGTGAGG	-	exonic	INO80B	85	19	0.1827	Frame_Shift_Del	p.T82fs	Non-Targeted Panel Gene	Adult HGBCL, NOS
BCL-11	chr7	122554598	122554598	-	T	exonic	CADPS2	62	50	0.4464	Frame_Shift_Ins	p.I476fs	Non-Targeted Panel Gene	Adult HGBCL, NOS
BCL-11	chr17	17796724	17796726	AGG	-	exonic	RAI1	44	23	0.3433	In_Frame_Del	p.1259_1260del	Non-Targeted Panel Gene	Adult HGBCL, NOS
BCL-11	chr16	67202031	67202031	-	ACA	exonic	ELMO3	32	27	0.4426	In_Frame_Ins	p.D422delinsDN	Non-Targeted Panel Gene	Adult HGBCL, NOS
BCL-11	chr2	167210872	167210872	G	A	exonic	XIRP2	35	3	0.0789	Missense_Mutation	p.D267N	Targerted Panel Gene	Adult HGBCL, NOS
BCL-11	chr7	100956356	100956356	G	A	exonic	MUC3A	415	37	0.0799	Missense_Mutation	p.S1526N	Targerted Panel Gene	Adult HGBCL, NOS
BCL-11	chr8	3052642	3052642	A	G	exonic	CSMD1	37	22	0.3729	Missense_Mutation	p.S2494P	Targerted Panel Gene	Adult HGBCL, NOS
BCL-11	chr11	92809975	92809975	A	C	exonic	FAT3	80	51	0.3893	Missense_Mutation	p.N3127T	Targerted Panel Gene	Adult HGBCL, NOS
BCL-11	chr16	685736	685736	C	T	exonic	WDR24	74	57	0.4351	Missense_Mutation	p.G541S	Targerted Panel Gene	Adult HGBCL, NOS
BCL-11	chr10	121565644	121565644	G	A	exonic	FGFR2	107	88	0.4513	Missense_Mutation	p.S57L	Targerted Panel Gene	Adult HGBCL, NOS
BCL-11	chr2	49943749	49943749	C	T	exonic	NRXN1	114	98	0.4623	Missense_Mutation	p.D1431N	Targerted Panel Gene	Adult HGBCL, NOS
BCL-11	chr13	40666151	40666151	C	T	exonic	FOXO1	15	13	0.4643	Missense_Mutation	p.R21H	Targerted Panel Gene	Adult HGBCL, NOS
BCL-11	chr12	92145462	92145462	A	G	exonic	BTG1	45	40	0.4706	Missense_Mutation	p.F25S	Targerted Panel Gene	Adult HGBCL, NOS
BCL-11	chr7	82950542	82950542	A	C	exonic	PCLO	32	29	0.4754	Missense_Mutation	p.F3349C	Targerted Panel Gene	Adult HGBCL, NOS
BCL-11	chr12	53471801	53471801	A	G	exonic	PCBP2	33	33	0.5	Missense_Mutation	p.N349S	Targerted Panel Gene	Adult HGBCL, NOS
BCL-11	chr2	140525966	140525966	T	G	exonic	LRP1B	44	47	0.5165	Missense_Mutation	p.K2635T	Targerted Panel Gene	Adult HGBCL, NOS
BCL-11	chr1	7737964	7737964	A	G	exonic	CAMTA1	10	11	0.5238	Missense_Mutation	p.R1222G	Targerted Panel Gene	Adult HGBCL, NOS
BCL-11	chr1	237566656	237566656	T	G	exonic	RYR2	47	56	0.5437	Missense_Mutation	p.Y1102D	Targerted Panel Gene	Adult HGBCL, NOS
BCL-11	chr2	11773711	11773711	C	A	exonic	LPIN1	70	4	0.0533	Missense_Mutation	p.P279T	Non-Targeted Panel Gene	Adult HGBCL, NOS
BCL-11	chr2	96689736	96689736	C	A	exonic	FER1L5	28	3	0.0968	Missense_Mutation	p.A873D	Non-Targeted Panel Gene	Adult HGBCL, NOS
BCL-11	chr15	40997557	40997557	A	C	exonic	INO80	116	14	0.1077	Missense_Mutation	p.L1181R	Non-Targeted Panel Gene	Adult HGBCL, NOS
BCL-11	chr6	137493660	137493660	G	A	exonic	OLIG3	13	2	0.1333	Missense_Mutation	p.P171S	Non-Targeted Panel Gene	Adult HGBCL, NOS
BCL-11	chr11	67421075	67421075	C	G	exonic	CARNS1	9	3	0.25	Missense_Mutation	p.D494E	Non-Targeted Panel Gene	Adult HGBCL, NOS
BCL-11	chr3	184248762	184248762	A	C	exonic	ALG3	14	5	0.2632	Missense_Mutation	p.V60G	Non-Targeted Panel Gene	Adult HGBCL, NOS
BCL-11	chr10	48981381	48981381	A	C	exonic	WDFY4	45	19	0.2969	Missense_Mutation	p.K3131Q	Non-Targeted Panel Gene	Adult HGBCL, NOS
BCL-11	chrX	54134969	54134969	G	T	exonic	FAM120C	110	52	0.319	Missense_Mutation	p.A493D	Non-Targeted Panel Gene	Adult HGBCL, NOS
BCL-11	chr7	40459197	40459197	C	T	exonic	SUGCT	41	20	0.3279	Missense_Mutation	p.R299W	Non-Targeted Panel Gene	Adult HGBCL, NOS
BCL-11	chr7	121976211	121976211	T	G	exonic	PTPRZ1	42	21	0.3333	Missense_Mutation	p.S165R	Non-Targeted Panel Gene	Adult HGBCL, NOS
BCL-11	chr10	34382662	34382662	T	G	exonic	PARD3	45	24	0.3478	Missense_Mutation	p.H426P	Non-Targeted Panel Gene	Adult HGBCL, NOS
BCL-11	chr2	216259619	216259619	T	G	exonic	MARCHF4	64	35	0.3535	Missense_Mutation	p.N309T	Non-Targeted Panel Gene	Adult HGBCL, NOS
BCL-11	chr10	122087293	122087293	A	C	exonic	TACC2	22	13	0.3714	Missense_Mutation	p.K1598T	Non-Targeted Panel Gene	Adult HGBCL, NOS
BCL-11	chr1	74250696	74250696	T	G	exonic	FPGT-TNNI3K;TNN	63	38	0.3762	Missense_Mutation	p.L201R	Non-Targeted Panel Gene	Adult HGBCL, NOS
BCL-11	chr5	35065463	35065463	T	G	exonic	PRLR	49	30	0.3797	Missense_Mutation	p.T499P	Non-Targeted Panel Gene	Adult HGBCL, NOS
BCL-11	chr12	20877862	20877862	G	C	exonic	SLCO1B3	89	57	0.3878	Missense_Mutation	p.G354A	Non-Targeted Panel Gene	Adult HGBCL, NOS
BCL-11	chr9	121781517	121781517	C	T	exonic	DAB2IP	36	23	0.3898	Missense_Mutation	p.A1095V	Non-Targeted Panel Gene	Adult HGBCL, NOS
BCL-11	chr19	15923951	15923951	C	T	exonic	CYP4F11	99	64	0.3926	Missense_Mutation	p.C260Y	Non-Targeted Panel Gene	Adult HGBCL, NOS
BCL-11	chr11	400622	400622	G	A	exonic	PKP3	32	21	0.3962	Missense_Mutation	p.G567S	Non-Targeted Panel Gene	Adult HGBCL, NOS
BCL-11	chr4	65365017	65365017	T	G	exonic	EPHA5	70	46	0.3966	Missense_Mutation	p.S746R	Non-Targeted Panel Gene	Adult HGBCL, NOS
BCL-11	chr1	85200296	85200296	T	G	exonic	SYDE2	40	28	0.4058	Missense_Mutation	p.N234T	Non-Targeted Panel Gene	Adult HGBCL, NOS
BCL-11	chr7	112767250	112767250	A	C	exonic	TMEM168	38	26	0.4062	Missense_Mutation	p.F681V	Non-Targeted Panel Gene	Adult HGBCL, NOS
BCL-11	chr19	48286782	48286782	A	C	exonic	ZNF114	50	35	0.407	Missense_Mutation	p.K386N	Non-Targeted Panel Gene	Adult HGBCL, NOS
BCL-11	chr11	11967011	11967011	T	C	exonic	DKK3	23	16	0.4103	Missense_Mutation	p.T206A	Non-Targeted Panel Gene	Adult HGBCL, NOS
BCL-11	chr12	13213782	13213782	A	C	exonic	EMP1	63	44	0.4112	Missense_Mutation	p.N93H	Non-Targeted Panel Gene	Adult HGBCL, NOS
BCL-11	chr7	99606769	99606769	T	G	exonic	TMEM225B	103	72	0.4114	Missense_Mutation	p.V77G	Non-Targeted Panel Gene	Adult HGBCL, NOS
BCL-11	chr11	47349779	47349779	T	C	exonic	MYBPC3	20	14	0.4118	Missense_Mutation	p.S217G	Non-Targeted Panel Gene	Adult HGBCL, NOS
BCL-11	chr10	69000515	69000515	T	C	exonic	KIF1BP	61	43	0.4135	Missense_Mutation	p.M173T	Non-Targeted Panel Gene	Adult HGBCL, NOS
BCL-11	chr3	113363307	113363307	A	C	exonic	CFAP44	48	34	0.4146	Missense_Mutation	p.S924R	Non-Targeted Panel Gene	Adult HGBCL, NOS
BCL-11	chr4	46974265	46974265	T	G	exonic	GABRA4	73	52	0.416	Missense_Mutation	p.T211P	Non-Targeted Panel Gene	Adult HGBCL, NOS
BCL-11	chr2	238141194	238141194	T	G	exonic	KLHL30	22	16	0.4211	Missense_Mutation	p.L147R	Non-Targeted Panel Gene	Adult HGBCL, NOS
BCL-11	chr16	30953441	30953441	T	A	exonic	ORAI3	53	39	0.4239	Missense_Mutation	p.F162Y	Non-Targeted Panel Gene	Adult HGBCL, NOS
BCL-11	chr2	98300115	98300115	A	G	exonic	VWA3B	69	51	0.425	Missense_Mutation	p.K1107E	Non-Targeted Panel Gene	Adult HGBCL, NOS
BCL-11	chr2	109574710	109574710	T	G	exonic	SEPTIN10	35	26	0.4262	Missense_Mutation	p.E157D	Non-Targeted Panel Gene	Adult HGBCL, NOS
BCL-11	chr3	156678592	156678592	G	C	exonic	TIPARP	76	58	0.4296	Missense_Mutation	p.D299H	Non-Targeted Panel Gene	Adult HGBCL, NOS
BCL-11	chr7	21601176	21601176	A	G	exonic	DNAH11	49	37	0.4302	Missense_Mutation	p.D1141G	Non-Targeted Panel Gene	Adult HGBCL, NOS
BCL-11	chr17	59270945	59270945	A	C	exonic	GDPD1	71	54	0.432	Missense_Mutation	p.E240D	Non-Targeted Panel Gene	Adult HGBCL, NOS
BCL-11	chr2	189036655	189036655	T	G	exonic	COL5A2	23	18	0.439	Missense_Mutation	p.K1358N	Non-Targeted Panel Gene	Adult HGBCL, NOS
BCL-11	chr4	73491992	73491992	A	G	exonic	AFM	56	44	0.44	Missense_Mutation	p.R322G	Non-Targeted Panel Gene	Adult HGBCL, NOS
BCL-11	chr10	27532528	27532528	T	G	exonic	RAB18	89	70	0.4403	Missense_Mutation	p.F99V	Non-Targeted Panel Gene	Adult HGBCL, NOS
BCL-11	chr3	171010024	171010024	A	C	exonic	SLC2A2	78	62	0.4429	Missense_Mutation	p.F144V	Non-Targeted Panel Gene	Adult HGBCL, NOS
BCL-11	chr11	67421107	67421107	T	A	exonic	CARNS1	10	8	0.4444	Missense_Mutation	p.M505K	Non-Targeted Panel Gene	Adult HGBCL, NOS
BCL-11	chr21	21324407	21324407	A	C	exonic	NCAM2	44	36	0.45	Missense_Mutation	p.Q215P	Non-Targeted Panel Gene	Adult HGBCL, NOS
BCL-11	chr8	95052195	95052195	G	A	exonic	NDUFAF6	78	64	0.4507	Missense_Mutation	p.V280I	Non-Targeted Panel Gene	Adult HGBCL, NOS
BCL-11	chr5	77298834	77298834	T	G	exonic	PDE8B	42	35	0.4545	Missense_Mutation	p.V24G	Non-Targeted Panel Gene	Adult HGBCL, NOS
BCL-11	chr15	54567938	54567938	A	C	exonic	UNC13C	36	30	0.4545	Missense_Mutation	p.T2031P	Non-Targeted Panel Gene	Adult HGBCL, NOS
BCL-11	chr3	123732916	123732916	C	G	exonic	MYLK	32	27	0.4576	Missense_Mutation	p.S499T	Non-Targeted Panel Gene	Adult HGBCL, NOS
BCL-11	chr14	47035213	47035213	T	G	exonic	MDGA2	122	103	0.4578	Missense_Mutation	p.K241N	Non-Targeted Panel Gene	Adult HGBCL, NOS
BCL-11	chr11	56346844	56346844	A	C	exonic	OR8K1	65	55	0.4583	Missense_Mutation	p.K269T	Non-Targeted Panel Gene	Adult HGBCL, NOS
BCL-11	chr3	32534565	32534565	T	G	exonic	DYNC1LI1	83	71	0.461	Missense_Mutation	p.Y305S	Non-Targeted Panel Gene	Adult HGBCL, NOS
BCL-11	chr11	105611043	105611043	T	C	exonic	GRIA4	32	28	0.4667	Missense_Mutation	p.W16R	Non-Targeted Panel Gene	Adult HGBCL, NOS
BCL-11	chr2	99361166	99361166	G	C	exonic	EIF5B	41	37	0.4744	Missense_Mutation	p.E89Q	Non-Targeted Panel Gene	Adult HGBCL, NOS
BCL-11	chr2	115840754	115840754	A	C	exonic	DPP10	53	48	0.4752	Missense_Mutation	p.K679N	Non-Targeted Panel Gene	Adult HGBCL, NOS
BCL-11	chr7	137463566	137463566	A	C	exonic	DGKI	42	39	0.4815	Missense_Mutation	p.S894R	Non-Targeted Panel Gene	Adult HGBCL, NOS
BCL-11	chr15	39988002	39988002	G	A	exonic	EIF2AK4	44	41	0.4824	Missense_Mutation	p.S808N	Non-Targeted Panel Gene	Adult HGBCL, NOS
BCL-11	chr15	26880913	26880913	A	G	exonic	GABRA5	53	50	0.4854	Missense_Mutation	p.I52V	Non-Targeted Panel Gene	Adult HGBCL, NOS
BCL-11	chr13	32319134	32319134	A	G	exonic	BRCA2	88	85	0.4857	Missense_Mutation	p.Y42C	Non-Targeted Panel Gene	Adult HGBCL, NOS
BCL-11	chr2	55867136	55867136	T	C	exonic	EFEMP1	60	57	0.4872	Missense_Mutation	p.I473M	Non-Targeted Panel Gene	Adult HGBCL, NOS
BCL-11	chr13	31777398	31777398	A	C	exonic	RXFP2	102	98	0.49	Missense_Mutation	p.T222P	Non-Targeted Panel Gene	Adult HGBCL, NOS
BCL-11	chr5	55220785	55220785	G	A	exonic	MCIDAS	26	25	0.4902	Missense_Mutation	p.R247W	Non-Targeted Panel Gene	Adult HGBCL, NOS

BCL-11	chr10	78004816	78004816	A	C	exonic	POLR3A	31	30	0.4918	Missense_Mutation	p.L716W	Non-Targeted Panel Gene	Adult HGBCL, NOS
BCL-11	chr3	142542691	142542691	T	C	exonic	ATR	38	37	0.4933	Missense_Mutation	p.S1142G	Non-Targeted Panel Gene	Adult HGBCL, NOS
BCL-11	chr6	64307042	64307042	A	T	exonic	EYS	44	44	0.5	Missense_Mutation	p.V2040D	Non-Targeted Panel Gene	Adult HGBCL, NOS
BCL-11	chr1	6601986	6601986	G	A	exonic	KLHL21	15	15	0.5	Missense_Mutation	p.P278S	Non-Targeted Panel Gene	Adult HGBCL, NOS
BCL-11	chr12	21456015	21456015	A	C	exonic	PYROXD1	54	54	0.5	Missense_Mutation	p.S224R	Non-Targeted Panel Gene	Adult HGBCL, NOS
BCL-11	chr4	79407425	79407425	T	C	exonic	GK2	79	81	0.5062	Missense_Mutation	p.D259G	Non-Targeted Panel Gene	Adult HGBCL, NOS
BCL-11	chr8	80499761	80499761	G	A	exonic	ZBTB10	80	82	0.5062	Missense_Mutation	p.V414I	Non-Targeted Panel Gene	Adult HGBCL, NOS
BCL-11	chr10	19175279	19175279	A	T	exonic	MALRD1	67	69	0.5074	Missense_Mutation	p.E634D	Non-Targeted Panel Gene	Adult HGBCL, NOS
BCL-11	chr17	16623015	16623015	A	G	exonic	ZNF624	32	33	0.5077	Missense_Mutation	p.M624T	Non-Targeted Panel Gene	Adult HGBCL, NOS
BCL-11	chr6	31782360	31782360	G	C	exonic	VARS	26	27	0.5094	Missense_Mutation	p.A692G	Non-Targeted Panel Gene	Adult HGBCL, NOS
BCL-11	chr2	178361702	178361702	G	T	exonic	OSBPL6	48	50	0.5102	Missense_Mutation	p.A417S	Non-Targeted Panel Gene	Adult HGBCL, NOS
BCL-11	chr15	48784114	48784114	T	C	exonic	CEP152	38	40	0.5128	Missense_Mutation	p.I394V	Non-Targeted Panel Gene	Adult HGBCL, NOS
BCL-11	chr9	5065003	5065003	C	G	exonic	JAK2	40	43	0.5181	Missense_Mutation	p.L393V	Non-Targeted Panel Gene	Adult HGBCL, NOS
BCL-11	chr1	97082391	97082391	T	A	exonic	DPYD	73	80	0.5229	Missense_Mutation	p.D949V	Non-Targeted Panel Gene	Adult HGBCL, NOS
BCL-11	chr11	65917494	65917494	C	G	exonic	C11orf68	9	10	0.5263	Missense_Mutation	p.V282L	Non-Targeted Panel Gene	Adult HGBCL, NOS
BCL-11	chr17	44354167	44354167	C	T	exonic	FAM171A2	22	25	0.5319	Missense_Mutation	p.D683N	Non-Targeted Panel Gene	Adult HGBCL, NOS
BCL-11	chr5	146129003	146129003	T	C	exonic	LARS	48	57	0.5429	Missense_Mutation	p.N915S	Non-Targeted Panel Gene	Adult HGBCL, NOS
BCL-11	chr15	89319073	89319073	A	G	exonic	POLG	65	78	0.5455	Missense_Mutation	p.V1044A	Non-Targeted Panel Gene	Adult HGBCL, NOS
BCL-11	chr11	95026971	95026971	C	A	exonic	KDM4E	19	24	0.5581	Missense_Mutation	p.Q472K	Non-Targeted Panel Gene	Adult HGBCL, NOS
BCL-11	chr14	21385663	21385663	C	G	exonic	CHD8	81	103	0.5598	Missense_Mutation	p.D2287H	Non-Targeted Panel Gene	Adult HGBCL, NOS
BCL-11	chr16	89920138	89920138	G	C	exonic	MC1R	145	189	0.5625	Missense_Mutation	p.D294H	Non-Targeted Panel Gene	Adult HGBCL, NOS
BCL-11	chr16	77194464	77194464	C	G	exonic	MON1B	34	44	0.5641	Missense_Mutation	p.T202R	Non-Targeted Panel Gene	Adult HGBCL, NOS
BCL-11	chr5	151109759	151109759	G	A	exonic	ANXA6	23	30	0.566	Missense_Mutation	p.R560W	Non-Targeted Panel Gene	Adult HGBCL, NOS
BCL-11	chr14	102928833	102928833	C	T	exonic	AMN	40	55	0.5789	Missense_Mutation	p.A124V	Non-Targeted Panel Gene	Adult HGBCL, NOS
BCL-11	chr7	4220234	4220234	G	A	exonic	SDK1	16	22	0.5789	Missense_Mutation	p.E1889K	Non-Targeted Panel Gene	Adult HGBCL, NOS
BCL-11	chr16	1220150	1220150	C	T	exonic	CACNA1H	18	25	0.5814	Missense_Mutation	p.T2073I	Non-Targeted Panel Gene	Adult HGBCL, NOS
BCL-11	chr7	122886087	122886087	C	A	exonic	CADPS2	8	12	0.6	Missense_Mutation	p.R84L	Non-Targeted Panel Gene	Adult HGBCL, NOS
BCL-11	chr12	20855032	20855032	T	G	exonic	SLCO1B3	16	24	0.6	Missense_Mutation	p.F30C	Non-Targeted Panel Gene	Adult HGBCL, NOS
BCL-11	chrX	1465681	1465681	T	G	exonic	P2RY8	13	95	0.8796	Missense_Mutation	p.Y293S	Non-Targeted Panel Gene	Adult HGBCL, NOS
BCL-11	chrX	1465731	1465731	C	G	exonic	P2RY8	12	97	0.8899	Missense_Mutation	p.K276N	Non-Targeted Panel Gene	Adult HGBCL, NOS
BCL-11	chrX	111254402	111254402	T	C	exonic	CAPN6	1	12	0.9231	Missense_Mutation	p.D56G	Non-Targeted Panel Gene	Adult HGBCL, NOS
BCL-11	chr6	152350676	152350676	A	G	exonic	SYNE1	3	63	0.9545	Missense_Mutation	p.L3892S	Non-Targeted Panel Gene	Adult HGBCL, NOS
BCL-11	chrX	152931727	152931727	G	C	exonic	ZNF185	0	38	1	Missense_Mutation	p.D324H	Non-Targeted Panel Gene	Adult HGBCL, NOS
BCL-11	chr7	65974701	65974701	G	A	exonic	GUSB	63	4	0.0597	Nonsense_Mutation	p.R357X	Non-Targeted Panel Gene	Adult HGBCL, NOS
BCL-11	chr2	102351877	102351877	A	T	exonic	IL1RL1	23	12	0.3429	Nonsense_Mutation	p.R543X	Non-Targeted Panel Gene	Adult HGBCL, NOS
BCL-11	chr8	16155085	16155085	G	A	exonic	MSR1	42	34	0.4474	Nonsense_Mutation	p.R293X	Non-Targeted Panel Gene	Adult HGBCL, NOS
BCL-11	chr2	73519879	73519879	C	G	exonic	ALMS1	47	54	0.5347	Nonsense_Mutation	p.S3215X	Non-Targeted Panel Gene	Adult HGBCL, NOS
BCL-11	chr6	105116813	105116813	A	C	exonic	BVES	7	45	0.8654	Nonsense_Mutation	p.L235X	Non-Targeted Panel Gene	Adult HGBCL, NOS
BCL-13	chr2	25131912	25131912	G	A	splicing	EFR3B	52	3	0.0545	Splice_Site	.	Non-Targeted Panel Gene	Adult HGBCL, NOS
BCL-13	chr10	19049138	19049138	G	A	splicing	MALRD1	35	3	0.0789	Splice_Site	.	Non-Targeted Panel Gene	Adult HGBCL, NOS
BCL-13	chr2	152664136	152664136	A	G	splicing	PRPF40A	178	18	0.0918	Splice_Site	.	Non-Targeted Panel Gene	Adult HGBCL, NOS
BCL-13	chr14	34608029	34608029	C	T	splicing	SNX6	67	62	0.4806	Splice_Site	.	Non-Targeted Panel Gene	Adult HGBCL, NOS
BCL-13	chr4	7433777	7433777	T	-	exonic	PSAPL1	65	42	0.3925	Frame_Shift_Del	p.N368fs	Non-Targeted Panel Gene	Adult HGBCL, NOS
BCL-13	chr19	12112876	12112876	A	-	exonic	ZNF788	68	55	0.4472	Frame_Shift_Del	p.A443fs	Non-Targeted Panel Gene	Adult HGBCL, NOS
BCL-13	chr10	125788972	125788973	GC	-	exonic	UROS	24	26	0.52	Frame_Shift_Del	p.A258fs	Non-Targeted Panel Gene	Adult HGBCL, NOS
BCL-13	chr16	69150666	69150666	-	T	exonic	UTP4	55	56	0.5	Frame_Shift_Ins	p.V290fs	Non-Targeted Panel Gene	Adult HGBCL, NOS
BCL-13	chr11	129855861	129855863	TCA	-	exonic	TMEM45B	119	19	0.1377	In_Frame_Del	p.180_181del	Non-Targeted Panel Gene	Adult HGBCL, NOS
BCL-13	chrX	71141301	71141301	-	CAGCAACACCAG	exonic	MED12	17	14	0.3784	In_Frame_Ins	p.Q2113delinsQQ	Targerted Panel Gene	Adult HGBCL, NOS
BCL-13	chr6	26124542	26124542	G	A	exonic	HIST1H2AC	344	27	0.0728	Missense_Mutation	p.A104T	Targerted Panel Gene	Adult HGBCL, NOS
BCL-13	chr22	23181118	23181118	G	A	exonic	BCR	80	30	0.2727	Missense_Mutation	p.R53H	Targerted Panel Gene	Adult HGBCL, NOS
BCL-13	chr13	40666136	40666136	G	A	exonic	FOXO1	24	11	0.3143	Missense_Mutation	p.P26L	Targerted Panel Gene	Adult HGBCL, NOS
BCL-13	chr4	20486211	20486211	A	G	exonic	SLIT2	117	56	0.3237	Missense_Mutation	p.N184S	Targerted Panel Gene	Adult HGBCL, NOS
BCL-13	chr16	689552	689552	T	C	exonic	WDR24	97	62	0.3875	Missense_Mutation	p.N30S	Targerted Panel Gene	Adult HGBCL, NOS
BCL-13	chr19	1612367	1612367	A	T	exonic	TCF3	73	64	0.4672	Missense_Mutation	p.N551K	Targerted Panel Gene	Adult HGBCL, NOS
BCL-13	chr12	57194410	57194410	C	T	exonic	LRP1	27	25	0.4808	Missense_Mutation	p.P2659S	Targerted Panel Gene	Adult HGBCL, NOS
BCL-13	chr5	112819272	112819272	C	T	exonic	APC	113	107	0.4864	Missense_Mutation	p.R414C	Targerted Panel Gene	Adult HGBCL, NOS
BCL-13	chr2	21006643	21006643	T	C	exonic	APOB	376	389	0.5085	Missense_Mutation	p.S3409G	Targerted Panel Gene	Adult HGBCL, NOS
BCL-13	chr19	856153	856153	A	G	exonic	ELANE	75	3	0.0385	Missense_Mutation	p.R265G	Non-Targeted Panel Gene	Adult HGBCL, NOS
BCL-13	chr19	14151405	14151405	C	T	exonic	ADGRL1	73	3	0.0395	Missense_Mutation	p.S1298N	Non-Targeted Panel Gene	Adult HGBCL, NOS
BCL-13	chr19	7917827	7917827	C	T	exonic	TGFBR3L	71	3	0.0405	Missense_Mutation	p.A284V	Non-Targeted Panel Gene	Adult HGBCL, NOS
BCL-13	chr19	44878267	44878267	G	A	exonic	NECTIN2	84	4	0.0455	Missense_Mutation	p.G363R	Non-Targeted Panel Gene	Adult HGBCL, NOS
BCL-13	chr1	70036587	70036587	G	A	exonic	LRRC7	61	3	0.0469	Missense_Mutation	p.A718T	Non-Targeted Panel Gene	Adult HGBCL, NOS
BCL-13	chr1	202881213	202881213	G	A	exonic	RABIF	54	3	0.0526	Missense_Mutation	p.P46L	Non-Targeted Panel Gene	Adult HGBCL, NOS
BCL-13	chr16	89710874	89710874	G	A	exonic	VPS9D1	53	3	0.0526	Missense_Mutation	p.R324W	Non-Targeted Panel Gene	Adult HGBCL, NOS
BCL-13	chr19	11575227	11575227	C	T	exonic	ACP5	52	3	0.0545	Missense_Mutation	p.G254D	Non-Targeted Panel Gene	Adult HGBCL, NOS
BCL-13	chr8	96785178	96785178	A	C	exonic	CPQ	96	6	0.0588	Missense_Mutation	p.N94T	Non-Targeted Panel Gene	Adult HGBCL, NOS
BCL-13	chr11	67467362	67467362	T	G	exonic	TMEM134	107	8	0.0696	Missense_Mutation	p.K119T	Non-Targeted Panel Gene	Adult HGBCL, NOS
BCL-13	chr8	89953686	89953686	C	A	exonic	NBN	40	3	0.0698	Missense_Mutation	p.R386M	Non-Targeted Panel Gene	Adult HGBCL, NOS
BCL-13	chr5	11384835	11384835	G	A	exonic	CTNND2	38	3	0.0732	Missense_Mutation	p.T336I	Non-Targeted Panel Gene	Adult HGBCL, NOS
BCL-13	chr17	1006091	1006091	T	G	exonic	ABR	132	11	0.0769	Missense_Mutation	p.T857P	Non-Targeted Panel Gene	Adult HGBCL, NOS
BCL-13	chr9	73158571	73158571	G	T	exonic	ANXA1	107	9	0.0776	Missense_Mutation	p.W12C	Non-Targeted Panel Gene	Adult HGBCL, NOS
BCL-13	chr9	2060866	2060866	G	T	exonic	SMARCA2	178	15	0.0777	Missense_Mutation	p.R524S	Non-Targeted Panel Gene	Adult HGBCL, NOS
BCL-13	chr16	30369705	30369705	G	A	exonic	TBC1D10B	45	4	0.0816	Missense_Mutation	p.T160M	Non-Targeted Panel Gene	Adult HGBCL, NOS
BCL-13	chr1	25863806	25863806	G	A	exonic	PAQR7	30	3	0.0909	Missense_Mutation	p.P12S	Non-Targeted Panel Gene	Adult HGBCL, NOS
BCL-13	chr17	7294365	7294365	C	A	exonic	YBX2	19	2	0.0952	Missense_Mutation	p.G46W	Non-Targeted Panel Gene	Adult HGBCL, NOS
BCL-13	chr5	128301485	128301485	A	C	exonic	FBN2	194	40	0.1709	Missense_Mutation	p.F1981L	Non-Targeted Panel Gene	Adult HGBCL, NOS
BCL-13	chr15	84783840	84783840	C	T	exonic	ZNF592	71	29	0.2871	Missense_Mutation	p.R389W	Non-Targeted Panel Gene	Adult HGBCL, NOS
BCL-13	chr2	27083128	27083128	C	A	exonic	EMILIN1	16	7	0.3043	Missense_Mutation	p.H519Q	Non-Targeted Panel Gene	Adult HGBCL, NOS
BCL-13	chr12	84876504	84876504	G	A	exonic	SLC6A15	34	16	0.32	Missense_Mutation	p.T287I	Non-Targeted Panel Gene	Adult HGBCL, NOS
BCL-13	chr16	88435691	88435691	G	C	exonic	ZNF469	34	18	0.3462	Missense_Mutation	p.G2713R	Non-Targeted Panel Gene	Adult HGBCL, NOS
BCL-13	chr16	89154148	89154148	C	T	exonic	ACSF3	47	25	0.3472	Missense_Mutation	p.R558W	Non-Targeted Panel Gene	Adult HGBCL, NOS
BCL-13	chr11	64829636	64829636	C	T	exonic	CDC42BPG	67	38	0.3619	Missense_Mutation	p.E1268K	Non-Targeted Panel Gene	Adult HGBCL, NOS

BCL-13	chr12	85979367	85979367	T	C	exonic	MGAT4C	104	67	0.3918	Missense_Mutation	p.I453M	Non-Targeted Panel Gene	Adult HGBCL, NOS
BCL-13	chr19	8585484	8585484	G	A	exonic	ADAMTS10	17	11	0.3929	Missense_Mutation	p.P946L	Non-Targeted Panel Gene	Adult HGBCL, NOS
BCL-13	chr16	4262622	4262622	C	T	exonic	TFAP4	90	60	0.4	Missense_Mutation	p.E57K	Non-Targeted Panel Gene	Adult HGBCL, NOS
BCL-13	chr16	54285992	54285992	G	A	exonic	IRX3	13	9	0.4091	Missense_Mutation	p.P20L	Non-Targeted Panel Gene	Adult HGBCL, NOS
BCL-13	chr3	38950110	38950110	A	G	exonic	SCN11A	46	32	0.4103	Missense_Mutation	p.Y85H	Non-Targeted Panel Gene	Adult HGBCL, NOS
BCL-13	chr2	237829401	237829401	A	G	exonic	RBM44	120	84	0.4118	Missense_Mutation	p.N930D	Non-Targeted Panel Gene	Adult HGBCL, NOS
BCL-13	chr16	71656593	71656593	C	T	exonic	PHLPP2	62	44	0.4151	Missense_Mutation	p.E790K	Non-Targeted Panel Gene	Adult HGBCL, NOS
BCL-13	chr10	102834088	102834088	C	G	exonic	CYP17A1	83	63	0.4315	Missense_Mutation	p.S234T	Non-Targeted Panel Gene	Adult HGBCL, NOS
BCL-13	chr7	66082440	66082440	C	T	exonic	ASL	66	51	0.4359	Missense_Mutation	p.R94C	Non-Targeted Panel Gene	Adult HGBCL, NOS
BCL-13	chr7	11412707	11412707	C	G	exonic	THSD7A	144	120	0.4545	Missense_Mutation	p.E1211Q	Non-Targeted Panel Gene	Adult HGBCL, NOS
BCL-13	chr15	73323445	73323445	G	C	exonic	HCN4	16	14	0.4667	Missense_Mutation	p.P883R	Non-Targeted Panel Gene	Adult HGBCL, NOS
BCL-13	chr6	31269989	31269989	A	T	exonic	HLA-C	118	106	0.4732	Missense_Mutation	p.M331K	Non-Targeted Panel Gene	Adult HGBCL, NOS
BCL-13	chr5	132393688	132393688	G	A	exonic	SLC22A5	100	91	0.474	Missense_Mutation	p.R512H	Non-Targeted Panel Gene	Adult HGBCL, NOS
BCL-13	chr7	5388790	5388790	G	A	exonic	TNRC18	12	11	0.4783	Missense_Mutation	p.P345L	Non-Targeted Panel Gene	Adult HGBCL, NOS
BCL-13	chr12	125114520	125114520	A	G	exonic	AACS	61	56	0.4786	Missense_Mutation	p.N320S	Non-Targeted Panel Gene	Adult HGBCL, NOS
BCL-13	chr15	24676177	24676177	A	T	exonic	NPAP1	29	27	0.4821	Missense_Mutation	p.N104Y	Non-Targeted Panel Gene	Adult HGBCL, NOS
BCL-13	chr12	57622572	57622572	C	T	exonic	SLC26A10	75	71	0.4863	Missense_Mutation	p.S241F	Non-Targeted Panel Gene	Adult HGBCL, NOS
BCL-13	chr11	74634606	74634606	T	C	exonic	POLD3	78	75	0.4902	Missense_Mutation	p.Y344H	Non-Targeted Panel Gene	Adult HGBCL, NOS
BCL-13	chr9	18777353	18777353	T	C	exonic	ADAMTSL1	19	19	0.5	Missense_Mutation	p.W1042R	Non-Targeted Panel Gene	Adult HGBCL, NOS
BCL-13	chr10	119147812	119147812	C	T	exonic	SFXN4	31	31	0.5	Missense_Mutation	p.A261T	Non-Targeted Panel Gene	Adult HGBCL, NOS
BCL-13	chr3	129976921	129976921	C	T	exonic	TRH	42	42	0.5	Missense_Mutation	p.A145V	Non-Targeted Panel Gene	Adult HGBCL, NOS
BCL-13	chr8	31081176	31081176	G	T	exonic	WRN	98	101	0.5075	Missense_Mutation	p.L383F	Non-Targeted Panel Gene	Adult HGBCL, NOS
BCL-13	chr1	226368240	226368240	T	C	exonic	PARP1	44	46	0.5111	Missense_Mutation	p.M746V	Non-Targeted Panel Gene	Adult HGBCL, NOS
BCL-13	chr17	43091002	43091002	G	C	exonic	BRCA1	110	116	0.5133	Missense_Mutation	p.T1376R	Non-Targeted Panel Gene	Adult HGBCL, NOS
BCL-13	chr3	165046998	165046998	A	C	exonic	SI	59	65	0.5242	Missense_Mutation	p.V577G	Non-Targeted Panel Gene	Adult HGBCL, NOS
BCL-13	chr19	49211240	49211240	C	T	exonic	TRPM4	45	51	0.5312	Missense_Mutation	p.P1204L	Non-Targeted Panel Gene	Adult HGBCL, NOS
BCL-13	chr11	48125168	48125168	G	C	exonic	PTPRJ	51	58	0.5321	Missense_Mutation	p.A359P	Non-Targeted Panel Gene	Adult HGBCL, NOS
BCL-13	chr8	31141445	31141445	G	A	exonic	WRN	35	41	0.5395	Missense_Mutation	p.A995T	Non-Targeted Panel Gene	Adult HGBCL, NOS
BCL-13	chr20	33659553	33659553	G	C	exonic	NECAB3	47	58	0.5524	Missense_Mutation	p.Q275E	Non-Targeted Panel Gene	Adult HGBCL, NOS
BCL-13	chr20	36198013	36198013	C	G	exonic	EPB41L1	42	54	0.5625	Missense_Mutation	p.P547R	Non-Targeted Panel Gene	Adult HGBCL, NOS
BCL-13	chr17	7285729	7285729	G	A	exonic	SLC2A4	28	40	0.5882	Missense_Mutation	p.V383I	Non-Targeted Panel Gene	Adult HGBCL, NOS
BCL-13	chr12	4810088	4810088	G	C	exonic	KCNA6	3	7	0.7	Missense_Mutation	p.R16P	Non-Targeted Panel Gene	Adult HGBCL, NOS
BCL-13	chrX	102717169	102717169	T	A	exonic	ARMCX5-GPRASP2	0	44	1	Missense_Mutation	p.L767H	Non-Targeted Panel Gene	Adult HGBCL, NOS
BCL-13	chr22	23181180	23181180	C	T	exonic	BCR	70	7	0.0909	Nonsense_Mutation	p.R74X	Targerted Panel Gene	Adult HGBCL, NOS
BCL-13	chr19	17830507	17830507	G	T	exonic	JAK3	41	3	0.0682	Nonsense_Mutation	p.S1031X	Non-Targeted Panel Gene	Adult HGBCL, NOS
BCL-13	chr11	56318538	56318538	G	T	exonic	OR8K3	149	55	0.2696	Nonsense_Mutation	p.G78X	Non-Targeted Panel Gene	Adult HGBCL, NOS
BCL-14	chr4	102657901	102657901	C	A	splicing	MANBA	49	3	0.0577	Splice_Site	.	Non-Targeted Panel Gene	Adult HGBCL, NOS
BCL-14	chr10	104915934	104915934	T	G	splicing	SORCS3	69	8	0.1039	Splice_Site	.	Non-Targeted Panel Gene	Adult HGBCL, NOS
BCL-14	chr16	25161100	25161100	A	G	splicing	LCMT1	41	43	0.5119	Splice_Site	.	Non-Targeted Panel Gene	Adult HGBCL, NOS
BCL-14	chr1	26697069	26697069	C	-	exonic	ARID1A	221	94	0.2984	Frame_Shift_Del	p.Y222fs	Targerted Panel Gene	Adult HGBCL, NOS
BCL-14	chr15	75392656	75392660	TTATC	-	exonic	SIN3A	138	5	0.035	Frame_Shift_Del	p.K811fs	Non-Targeted Panel Gene	Adult HGBCL, NOS
BCL-14	chr3	183309733	183309733	-	T	exonic	MCF2L2	68	35	0.3365	Frame_Shift_Ins	p.L366fs	Non-Targeted Panel Gene	Adult HGBCL, NOS
BCL-14	chr8	127738423	127738423	-	GTT	exonic	MYC	16	15	0.4839	In_Frame_Ins	p.E69delinsEL	Targerted Panel Gene	Adult HGBCL, NOS
BCL-14	chr7	100954049	100954049	C	T	exonic	MUC3A	254	25	0.0874	Missense_Mutation	p.T757I	Targerted Panel Gene	Adult HGBCL, NOS
BCL-14	chr8	127738598	127738598	C	G	exonic	MYC	159	21	0.1167	Missense_Mutation	p.N127K	Targerted Panel Gene	Adult HGBCL, NOS
BCL-14	chr3	49375489	49375489	T	C	exonic	RHOA	146	28	0.1609	Missense_Mutation	p.Y34C	Targerted Panel Gene	Adult HGBCL, NOS
BCL-14	chr17	7673802	7673802	C	T	exonic	TP53	64	14	0.1795	Missense_Mutation	p.R234H	Targerted Panel Gene	Adult HGBCL, NOS
BCL-14	chr7	55200352	55200352	G	A	exonic	EGFR	71	62	0.4662	Missense_Mutation	p.R962H	Targerted Panel Gene	Adult HGBCL, NOS
BCL-14	chr8	127738279	127738279	G	A	exonic	MYC	43	43	0.5	Missense_Mutation	p.S21N	Targerted Panel Gene	Adult HGBCL, NOS
BCL-14	chr8	127738790	127738790	G	C	exonic	MYC	33	35	0.5147	Missense_Mutation	p.L191F	Targerted Panel Gene	Adult HGBCL, NOS
BCL-14	chrX	41346590	41346590	G	A	exonic	DDX3X	31	107	0.7754	Missense_Mutation	p.R528H	Targerted Panel Gene	Adult HGBCL, NOS
BCL-14	chr19	51389135	51389135	G	A	exonic	C19orf84	52	2	0.037	Missense_Mutation	p.A137V	Non-Targeted Panel Gene	Adult HGBCL, NOS
BCL-14	chr11	45810877	45810877	G	A	exonic	SLC35C1	75	3	0.0385	Missense_Mutation	p.A200T	Non-Targeted Panel Gene	Adult HGBCL, NOS
BCL-14	chr12	111457314	111457314	G	A	exonic	ATXN2	69	3	0.0417	Missense_Mutation	p.T1139I	Non-Targeted Panel Gene	Adult HGBCL, NOS
BCL-14	chr18	76927150	76927150	G	A	exonic	ZNF236	87	4	0.044	Missense_Mutation	p.D1379N	Non-Targeted Panel Gene	Adult HGBCL, NOS
BCL-14	chr2	232459058	232459058	G	A	exonic	ALPI	43	2	0.0444	Missense_Mutation	p.C500Y	Non-Targeted Panel Gene	Adult HGBCL, NOS
BCL-14	chr20	968051	968051	A	G	exonic	RSPO4	42	2	0.0444	Missense_Mutation	p.L56P	Non-Targeted Panel Gene	Adult HGBCL, NOS
BCL-14	chr10	73914862	73914862	G	A	exonic	PLAU	126	6	0.0455	Missense_Mutation	p.D306N	Non-Targeted Panel Gene	Adult HGBCL, NOS
BCL-14	chr11	134153295	134153295	C	T	exonic	NCAPD3	82	4	0.0465	Missense_Mutation	p.A1441T	Non-Targeted Panel Gene	Adult HGBCL, NOS
BCL-14	chr13	24224404	24224404	G	A	exonic	SPATA13	61	3	0.0469	Missense_Mutation	p.S554N	Non-Targeted Panel Gene	Adult HGBCL, NOS
BCL-14	chr1	230255334	230255334	G	A	exonic	GALNT2	40	2	0.0476	Missense_Mutation	p.V376I	Non-Targeted Panel Gene	Adult HGBCL, NOS
BCL-14	chr2	219547869	219547869	C	A	exonic	TMEM198	39	2	0.0488	Missense_Mutation	p.A177D	Non-Targeted Panel Gene	Adult HGBCL, NOS
BCL-14	chr20	59192065	59192065	G	A	exonic	ZNF831	58	3	0.0492	Missense_Mutation	p.R349H	Non-Targeted Panel Gene	Adult HGBCL, NOS
BCL-14	chr12	30637033	30637033	C	T	exonic	IPO8	57	3	0.05	Missense_Mutation	p.V882I	Non-Targeted Panel Gene	Adult HGBCL, NOS
BCL-14	chr7	41965160	41965160	C	A	exonic	GLI3	36	2	0.0513	Missense_Mutation	p.A1305S	Non-Targeted Panel Gene	Adult HGBCL, NOS
BCL-14	chr3	52471918	52471918	G	T	exonic	NISCH	55	3	0.0517	Missense_Mutation	p.D172Y	Non-Targeted Panel Gene	Adult HGBCL, NOS
BCL-14	chr11	83469367	83469367	G	A	exonic	DLG2	72	4	0.0526	Missense_Mutation	p.T818M	Non-Targeted Panel Gene	Adult HGBCL, NOS
BCL-14	chr9	114350938	114350938	G	A	exonic	AKNA	34	2	0.0556	Missense_Mutation	p.P1048S	Non-Targeted Panel Gene	Adult HGBCL, NOS
BCL-14	chr20	63873738	63873738	G	A	exonic	TPD52L2	34	2	0.0556	Missense_Mutation	p.R79K	Non-Targeted Panel Gene	Adult HGBCL, NOS
BCL-14	chr8	144040409	144040409	G	A	exonic	SPATC1	50	3	0.0566	Missense_Mutation	p.G238S	Non-Targeted Panel Gene	Adult HGBCL, NOS
BCL-14	chr17	1229518	1229518	C	T	exonic	ABR	32	2	0.0588	Missense_Mutation	p.R38H	Non-Targeted Panel Gene	Adult HGBCL, NOS
BCL-14	chr12	51822298	51822298	C	T	exonic	FIGNL2	32	2	0.0588	Missense_Mutation	p.R39H	Non-Targeted Panel Gene	Adult HGBCL, NOS
BCL-14	chr2	226796335	226796335	C	T	exonic	IRS1	46	3	0.06	Missense_Mutation	p.A802T	Non-Targeted Panel Gene	Adult HGBCL, NOS
BCL-14	chr6	107634228	107634228	C	T	exonic	SOBP	60	4	0.0625	Missense_Mutation	p.P462S	Non-Targeted Panel Gene	Adult HGBCL, NOS
BCL-14	chr6	43145343	43145343	G	T	exonic	PTK7	29	2	0.0645	Missense_Mutation	p.G851W	Non-Targeted Panel Gene	Adult HGBCL, NOS
BCL-14	chr1	150552544	150552544	C	A	exonic	ADAMTSL4	27	2	0.0667	Missense_Mutation	p.P8T	Non-Targeted Panel Gene	Adult HGBCL, NOS
BCL-14	chr5	31995711	31995711	G	A	exonic	PDZD2	42	3	0.0667	Missense_Mutation	p.A372T	Non-Targeted Panel Gene	Adult HGBCL, NOS
BCL-14	chr17	80065562	80065562	G	A	exonic	CCDC40	41	3	0.0682	Missense_Mutation	p.M506I	Non-Targeted Panel Gene	Adult HGBCL, NOS
BCL-14	chr16	2527907	2527907	G	A	exonic	AMDHD2	36	3	0.0769	Missense_Mutation	p.V184M	Non-Targeted Panel Gene	Adult HGBCL, NOS
BCL-14	chr16	88432967	88432967	G	A	exonic	ZNF469	31	3	0.0882	Missense_Mutation	p.G1805R	Non-Targeted Panel Gene	Adult HGBCL, NOS
BCL-14	chr1	154429426	154429426	G	A	exonic	IL6R	30	3	0.0909	Missense_Mutation	p.V106M	Non-Targeted Panel Gene	Adult HGBCL, NOS
BCL-14	chr7	130023704	130023704	G	A	exonic	ZC3HC1	19	2	0.0952	Missense_Mutation	p.P326L	Non-Targeted Panel Gene	Adult HGBCL, NOS

BCL-14	chr1	155309898	155309898	C	T	exonic	FDPS	17	2	0.1053	Missense_Mutation	p.H37Y	Non-Targeted Panel Gene	Adult HGBCL, NOS
BCL-14	chr1	109114187	109114187	G	A	exonic	KIAA1324	17	2	0.1053	Missense_Mutation	p.A2T	Non-Targeted Panel Gene	Adult HGBCL, NOS
BCL-14	chr14	21092572	21092572	G	A	exonic	ZNF219	16	2	0.1111	Missense_Mutation	p.S242L	Non-Targeted Panel Gene	Adult HGBCL, NOS
BCL-14	chr4	88008256	88008256	C	T	exonic	PKD2	14	2	0.125	Missense_Mutation	p.H175Y	Non-Targeted Panel Gene	Adult HGBCL, NOS
BCL-14	chr2	28525283	28525283	C	T	exonic	PLB1	28	4	0.125	Missense_Mutation	p.T87M	Non-Targeted Panel Gene	Adult HGBCL, NOS
BCL-14	chr6	134918432	134918432	T	G	exonic	ALDH8A1	68	10	0.1282	Missense_Mutation	p.I483L	Non-Targeted Panel Gene	Adult HGBCL, NOS
BCL-14	chr10	113582128	113582128	C	T	exonic	HABP2	13	2	0.1333	Missense_Mutation	p.T364I	Non-Targeted Panel Gene	Adult HGBCL, NOS
BCL-14	chr20	4182368	4182368	G	A	exonic	SMOX	18	3	0.1429	Missense_Mutation	p.G297R	Non-Targeted Panel Gene	Adult HGBCL, NOS
BCL-14	chr1	160218349	160218349	T	C	exonic	DCAF8	112	19	0.145	Missense_Mutation	p.H551R	Non-Targeted Panel Gene	Adult HGBCL, NOS
BCL-14	chr8	58587670	58587670	C	G	exonic	NSMAF	41	17	0.2931	Missense_Mutation	p.G748A	Non-Targeted Panel Gene	Adult HGBCL, NOS
BCL-14	chr5	116486891	116486891	T	C	exonic	SEMA6A	77	37	0.3246	Missense_Mutation	p.T274A	Non-Targeted Panel Gene	Adult HGBCL, NOS
BCL-14	chr1	186351377	186351377	A	C	exonic	TPR	109	59	0.3512	Missense_Mutation	p.L855V	Non-Targeted Panel Gene	Adult HGBCL, NOS
BCL-14	chr1	70038420	70038420	C	A	exonic	LRRC7	79	45	0.36	Missense_Mutation	p.H833N	Non-Targeted Panel Gene	Adult HGBCL, NOS
BCL-14	chr17	35119588	35119588	C	G	exonic	RAD51D	34	21	0.3818	Missense_Mutation	p.C9S	Non-Targeted Panel Gene	Adult HGBCL, NOS
BCL-14	chr6	154833130	154833130	A	T	exonic	SCAF8	62	40	0.3922	Missense_Mutation	p.Q1250L	Non-Targeted Panel Gene	Adult HGBCL, NOS
BCL-14	chr10	17321281	17321281	A	C	exonic	ST8SIA6	36	24	0.4	Missense_Mutation	p.F114C	Non-Targeted Panel Gene	Adult HGBCL, NOS
BCL-14	chr3	184323540	184323540	A	G	exonic	EIF4G1	43	30	0.411	Missense_Mutation	p.T748A	Non-Targeted Panel Gene	Adult HGBCL, NOS
BCL-14	chr2	159752433	159752433	G	C	exonic	MARCHF7	53	38	0.4176	Missense_Mutation	p.G549R	Non-Targeted Panel Gene	Adult HGBCL, NOS
BCL-14	chr1	173485364	173485364	A	G	exonic	PRDX6	35	27	0.4355	Missense_Mutation	p.I86V	Non-Targeted Panel Gene	Adult HGBCL, NOS
BCL-14	chr8	492691	492691	C	A	exonic	TDRP	47	37	0.4405	Missense_Mutation	p.W89L	Non-Targeted Panel Gene	Adult HGBCL, NOS
BCL-14	chr6	43040301	43040301	T	G	exonic	CUL7	55	44	0.4444	Missense_Mutation	p.E1383D	Non-Targeted Panel Gene	Adult HGBCL, NOS
BCL-14	chr1	6213137	6213137	C	T	exonic	RNF207	51	42	0.4516	Missense_Mutation	p.P536S	Non-Targeted Panel Gene	Adult HGBCL, NOS
BCL-14	chr17	31169939	31169939	T	A	exonic	NF1	57	47	0.4519	Missense_Mutation	p.D176E	Non-Targeted Panel Gene	Adult HGBCL, NOS
BCL-14	chr3	10359892	10359892	C	T	exonic	ATP2B2	45	38	0.4578	Missense_Mutation	p.V631M	Non-Targeted Panel Gene	Adult HGBCL, NOS
BCL-14	chr1	225085722	225085722	C	T	exonic	DNAH14	87	74	0.4596	Missense_Mutation	p.A1169V	Non-Targeted Panel Gene	Adult HGBCL, NOS
BCL-14	chr19	3646019	3646019	G	A	exonic	PIP5K1C	36	31	0.4627	Missense_Mutation	p.R434C	Non-Targeted Panel Gene	Adult HGBCL, NOS
BCL-14	chr2	135788521	135788521	C	G	exonic	LCT	44	39	0.4643	Missense_Mutation	p.V1863L	Non-Targeted Panel Gene	Adult HGBCL, NOS
BCL-14	chr6	161973317	161973317	G	A	exonic	PRKN	49	43	0.4674	Missense_Mutation	p.T240M	Non-Targeted Panel Gene	Adult HGBCL, NOS
BCL-14	chr12	64875291	64875291	G	A	exonic	TBC1D30	35	31	0.4697	Missense_Mutation	p.E483K	Non-Targeted Panel Gene	Adult HGBCL, NOS
BCL-14	chr10	91951452	91951452	G	T	exonic	BTAF1	61	57	0.4831	Missense_Mutation	p.Q150H	Non-Targeted Panel Gene	Adult HGBCL, NOS
BCL-14	chr14	91461523	91461523	C	G	exonic	PPP4R3A	55	53	0.4862	Missense_Mutation	p.S737T	Non-Targeted Panel Gene	Adult HGBCL, NOS
BCL-14	chr9	34648170	34648170	A	G	exonic	GALT	39	37	0.4868	Missense_Mutation	p.Q188R	Non-Targeted Panel Gene	Adult HGBCL, NOS
BCL-14	chr14	71026249	71026249	A	G	exonic	PCNX1	34	33	0.4925	Missense_Mutation	p.N1106D	Non-Targeted Panel Gene	Adult HGBCL, NOS
BCL-14	chr5	160092951	160092951	A	G	exonic	PWWP2A	100	99	0.495	Missense_Mutation	p.Y350H	Non-Targeted Panel Gene	Adult HGBCL, NOS
BCL-14	chr15	73323445	73323445	G	C	exonic	HCN4	18	19	0.5135	Missense_Mutation	p.P883R	Non-Targeted Panel Gene	Adult HGBCL, NOS
BCL-14	chr1	157769925	157769925	G	T	exonic	FCRL2	42	45	0.5172	Missense_Mutation	p.A179E	Non-Targeted Panel Gene	Adult HGBCL, NOS
BCL-14	chr22	29517041	29517041	C	T	exonic	THOC5	51	58	0.5321	Missense_Mutation	p.E557K	Non-Targeted Panel Gene	Adult HGBCL, NOS
BCL-14	chr9	94301252	94301252	C	A	exonic	ZNF169	30	35	0.5385	Missense_Mutation	p.S565Y	Non-Targeted Panel Gene	Adult HGBCL, NOS
BCL-14	chr16	71951582	71951582	C	T	exonic	PKD1L3	11	13	0.5417	Missense_Mutation	p.E1058K	Non-Targeted Panel Gene	Adult HGBCL, NOS
BCL-14	chr14	104883203	104883203	C	A	exonic	CEP170B	29	37	0.5606	Missense_Mutation	p.T249K	Non-Targeted Panel Gene	Adult HGBCL, NOS
BCL-14	chr12	6329485	6329485	C	T	exonic	TNFRSF1A	61	82	0.5734	Missense_Mutation	p.A246T	Non-Targeted Panel Gene	Adult HGBCL, NOS
BCL-14	chr6	42050564	42050564	C	A	exonic	TAF8	38	63	0.6238	Missense_Mutation	p.A8D	Non-Targeted Panel Gene	Adult HGBCL, NOS
BCL-14	chr7	107701183	107701183	T	C	exonic	SLC26A4	33	58	0.6374	Missense_Mutation	p.L597S	Non-Targeted Panel Gene	Adult HGBCL, NOS
BCL-14	chr17	65056395	65056395	G	A	exonic	GNA13	120	7	0.0551	Nonsense_Mutation	p.Q67X	Targerted Panel Gene	Adult HGBCL, NOS
BCL-14	chr16	67200204	67200204	G	T	exonic	ELMO3	21	2	0.087	Nonsense_Mutation	p.E139X	Non-Targeted Panel Gene	Adult HGBCL, NOS
BCL-14	chr4	143440166	143440166	C	T	exonic	GAB1	101	41	0.2887	Nonsense_Mutation	p.R457X	Non-Targeted Panel Gene	Adult HGBCL, NOS
BCL-14	chr18	46844834	46844834	G	C	exonic	PIAS2	35	47	0.5732	Nonsense_Mutation	p.Y280X	Non-Targeted Panel Gene	Adult HGBCL, NOS
BCL-15	chr4	75883076	75883076	C	T	splicing	PPEF2	44	2	0.0426	Splice_Site	.	Non-Targeted Panel Gene	Adult HGBCL, NOS
BCL-15	chr20	5944161	5944161	C	T	splicing	TRMT6	39	2	0.0488	Splice_Site	.	Non-Targeted Panel Gene	Adult HGBCL, NOS
BCL-15	chr17	41367907	41367907	C	T	splicing	KRT33B	45	3	0.0625	Splice_Site	.	Non-Targeted Panel Gene	Adult HGBCL, NOS
BCL-15	chr2	15275483	15275483	C	T	splicing	NBAS	27	2	0.069	Splice_Site	.	Non-Targeted Panel Gene	Adult HGBCL, NOS
BCL-15	chr11	72294324	72294324	C	T	splicing	CLPB	35	3	0.0769	Splice_Site	.	Non-Targeted Panel Gene	Adult HGBCL, NOS
BCL-15	chr21	32413014	32413014	G	A	splicing	EVA1C	24	2	0.0769	Splice_Site	.	Non-Targeted Panel Gene	Adult HGBCL, NOS
BCL-15	chr10	70736794	70736794	G	A	splicing	ADAMTS14	31	3	0.0882	Splice_Site	.	Non-Targeted Panel Gene	Adult HGBCL, NOS
BCL-15	chr3	195291705	195291705	C	T	splicing	ACAP2	32	4	0.1111	Splice_Site	.	Non-Targeted Panel Gene	Adult HGBCL, NOS
BCL-15	chr14	31387119	31387119	C	T	splicing	HEATR5A	11	2	0.1538	Splice_Site	.	Non-Targeted Panel Gene	Adult HGBCL, NOS
BCL-15	chr9	114905826	114905826	A	T	splicing	TNFSF8	11	6	0.3529	Splice_Site	.	Non-Targeted Panel Gene	Adult HGBCL, NOS
BCL-15	chr11	1030684	1030684	C	-	exonic	MUC6	15	9	0.375	Frame_Shift_Del	p.A261fs	Non-Targeted Panel Gene	Adult HGBCL, NOS
BCL-15	chr9	128822602	128822605	AGTA	-	exonic	ENDOG	7	7	0.5	Frame_Shift_Del	p.S296fs	Non-Targeted Panel Gene	Adult HGBCL, NOS
BCL-15	chr1	157834704	157834704	C	-	exonic	CD5L	13	23	0.6389	Frame_Shift_Del	p.A141fs	Non-Targeted Panel Gene	Adult HGBCL, NOS
BCL-15	chr17	65014746	65014746	-	T	exonic	GNA13	101	19	0.157	Frame_Shift_Ins	p.N215fs	Targerted Panel Gene	Adult HGBCL, NOS
BCL-15	chr11	61121641	61121641	-	G	exonic	CD5	112	13	0.104	Frame_Shift_Ins	p.V279fs	Non-Targeted Panel Gene	Adult HGBCL, NOS
BCL-15	chr4	17705076	17705078	TCT	-	exonic	FAM184B	38	17	0.3091	In_Frame_Del	p.433_434del	Non-Targeted Panel Gene	Adult HGBCL, NOS
BCL-15	chr9	15591449	15591451	AGA	-	exonic	CCDC171	23	17	0.425	In_Frame_Del	p.53_53del	Non-Targeted Panel Gene	Adult HGBCL, NOS
BCL-15	chr11	9091462	9091470	GCAGCAGCA	-	exonic	SCUBE2	6	5	0.4545	In_Frame_Del	p.20_23del	Non-Targeted Panel Gene	Adult HGBCL, NOS
BCL-15	chr7	21430345	21430347	CAG	-	exonic	SP4	23	30	0.566	In_Frame_Del	p.394_394del	Non-Targeted Panel Gene	Adult HGBCL, NOS
BCL-15	chr14	23525483	23525483	-	CCCACAGGC	exonic	ZFHX2	19	5	0.2083	In_Frame_Ins	p.K1487delinsACGI	Targerted Panel Gene	Adult HGBCL, NOS
BCL-15	chr8	47886034	47886034	C	A	exonic	PRKDC	53	2	0.0364	Missense_Mutation	p.L1562F	Targerted Panel Gene	Adult HGBCL, NOS
BCL-15	chr6	56635632	56635632	G	A	exonic	DST	48	2	0.04	Missense_Mutation	p.S1015L	Targerted Panel Gene	Adult HGBCL, NOS
BCL-15	chr4	186596999	186596999	C	T	exonic	FAT1	48	2	0.04	Missense_Mutation	p.E4181K	Targerted Panel Gene	Adult HGBCL, NOS
BCL-15	chr4	174976658	174976658	C	T	exonic	ADAM29	38	3	0.0732	Missense_Mutation	p.T378I	Targerted Panel Gene	Adult HGBCL, NOS
BCL-15	chr14	23525609	23525609	C	T	exonic	ZFHX2	21	2	0.087	Missense_Mutation	p.E1445K	Targerted Panel Gene	Adult HGBCL, NOS
BCL-15	chr11	118481715	118481715	C	T	exonic	KMT2A	17	2	0.1053	Missense_Mutation	p.A1212V	Targerted Panel Gene	Adult HGBCL, NOS
BCL-15	chr1	119925741	119925741	G	A	exonic	NOTCH2	13	2	0.1333	Missense_Mutation	p.H1359Y	Targerted Panel Gene	Adult HGBCL, NOS
BCL-15	chr6	26273072	26273072	A	G	exonic	HIST1H2BI	169	33	0.1634	Missense_Mutation	p.S33G	Targerted Panel Gene	Adult HGBCL, NOS
BCL-15	chr8	127738633	127738633	T	C	exonic	MYC	73	16	0.1798	Missense_Mutation	p.F139S	Targerted Panel Gene	Adult HGBCL, NOS
BCL-15	chr8	127738647	127738647	A	C	exonic	MYC	73	17	0.1889	Missense_Mutation	p.I144L	Targerted Panel Gene	Adult HGBCL, NOS
BCL-15	chr8	127740694	127740694	G	C	exonic	MYC	93	24	0.2051	Missense_Mutation	p.E367D	Targerted Panel Gene	Adult HGBCL, NOS
BCL-15	chr8	39788236	39788236	T	C	exonic	ADAM2	26	7	0.2121	Missense_Mutation	p.N220D	Targerted Panel Gene	Adult HGBCL, NOS
BCL-15	chr8	127738675	127738675	T	G	exonic	MYC	44	12	0.2143	Missense_Mutation	p.F153C	Targerted Panel Gene	Adult HGBCL, NOS
BCL-15	chr2	27217941	27217941	C	G	exonic	CAD	82	23	0.219	Missense_Mutation	p.I49M	Targerted Panel Gene	Adult HGBCL, NOS
BCL-15	chr8	127738852	127738852	A	G	exonic	MYC	40	12	0.2308	Missense_Mutation	p.Y212C	Targerted Panel Gene	Adult HGBCL, NOS

BCL-15	chr12	53471660	53471660	G	A	exonic	PCBP2	46	14	0.2333	Missense_Mutation	p.R302H	Targerted Panel Gene	Adult HGBCL, NOS
BCL-15	chr7	100955705	100955705	C	T	exonic	MUC3A	80	37	0.3162	Missense_Mutation	p.A1309V	Targerted Panel Gene	Adult HGBCL, NOS
BCL-15	chr10	128103969	128103969	T	G	exonic	MKI67	42	41	0.494	Missense_Mutation	p.Q2624P	Targerted Panel Gene	Adult HGBCL, NOS
BCL-15	chr8	3214640	3214640	C	A	exonic	CSMD1	15	15	0.5	Missense_Mutation	p.R1575I	Targerted Panel Gene	Adult HGBCL, NOS
BCL-15	chr11	92353103	92353103	C	A	exonic	FAT3	21	21	0.5	Missense_Mutation	p.Q331K	Targerted Panel Gene	Adult HGBCL, NOS
BCL-15	chr6	106105017	106105017	G	C	exonic	PRDM1	41	43	0.5119	Missense_Mutation	p.R286P	Targerted Panel Gene	Adult HGBCL, NOS
BCL-15	chr6	52083252	52083252	C	T	exonic	PKHD1	15	27	0.6429	Missense_Mutation	p.R19H	Targerted Panel Gene	Adult HGBCL, NOS
BCL-15	chr2	85542290	85542290	G	A	exonic	MAT2A	73	2	0.0267	Missense_Mutation	p.A229T	Non-Targeted Panel Gene	Adult HGBCL, NOS
BCL-15	chr15	61940706	61940706	C	A	exonic	VPS13C	68	2	0.0286	Missense_Mutation	p.A1848S	Non-Targeted Panel Gene	Adult HGBCL, NOS
BCL-15	chr22	20115438	20115438	G	A	exonic	TRMT2A	62	2	0.0312	Missense_Mutation	p.R240C	Non-Targeted Panel Gene	Adult HGBCL, NOS
BCL-15	chr1	1321934	1321934	C	T	exonic	INTS11	60	2	0.0323	Missense_Mutation	p.G7E	Non-Targeted Panel Gene	Adult HGBCL, NOS
BCL-15	chr12	54054257	54054257	C	T	exonic	HOXC4	58	2	0.0328	Missense_Mutation	p.P112L	Non-Targeted Panel Gene	Adult HGBCL, NOS
BCL-15	chrX	65533661	65533661	C	T	exonic	LAS1L	59	2	0.0328	Missense_Mutation	p.G104D	Non-Targeted Panel Gene	Adult HGBCL, NOS
BCL-15	chr1	155018561	155018561	G	A	exonic	DCST2	58	2	0.0333	Missense_Mutation	p.H769Y	Non-Targeted Panel Gene	Adult HGBCL, NOS
BCL-15	chr1	147167791	147167791	C	T	exonic	PRKAB2	58	2	0.0333	Missense_Mutation	p.S100N	Non-Targeted Panel Gene	Adult HGBCL, NOS
BCL-15	chr7	140097550	140097550	C	T	exonic	KDM7A	57	2	0.0339	Missense_Mutation	p.A671T	Non-Targeted Panel Gene	Adult HGBCL, NOS
BCL-15	chr8	12731779	12731779	C	T	exonic	LONRF1	57	2	0.0339	Missense_Mutation	p.E549K	Non-Targeted Panel Gene	Adult HGBCL, NOS
BCL-15	chr3	51312849	51312849	G	T	exonic	DOCK3	56	2	0.0345	Missense_Mutation	p.G1067V	Non-Targeted Panel Gene	Adult HGBCL, NOS
BCL-15	chr1	200979641	200979641	C	T	exonic	KIF21B	55	2	0.0351	Missense_Mutation	p.V1352I	Non-Targeted Panel Gene	Adult HGBCL, NOS
BCL-15	chr19	40075151	40075151	G	A	exonic	ZNF780A	55	2	0.0351	Missense_Mutation	p.H431Y	Non-Targeted Panel Gene	Adult HGBCL, NOS
BCL-15	chr7	103304028	103304028	C	T	exonic	PMPCB	53	2	0.0364	Missense_Mutation	p.T215I	Non-Targeted Panel Gene	Adult HGBCL, NOS
BCL-15	chr5	123573402	123573402	C	A	exonic	CSNK1G3	51	2	0.0377	Missense_Mutation	p.P100H	Non-Targeted Panel Gene	Adult HGBCL, NOS
BCL-15	chr13	30974855	30974855	T	C	exonic	TEX26	51	2	0.0377	Missense_Mutation	p.I273T	Non-Targeted Panel Gene	Adult HGBCL, NOS
BCL-15	chr3	171228193	171228193	G	A	exonic	TNIK	51	2	0.0377	Missense_Mutation	p.A51V	Non-Targeted Panel Gene	Adult HGBCL, NOS
BCL-15	chr20	49249021	49249021	G	A	exonic	ZNFX1	51	2	0.0377	Missense_Mutation	p.L1335F	Non-Targeted Panel Gene	Adult HGBCL, NOS
BCL-15	chr7	41965576	41965576	C	T	exonic	GLI3	50	2	0.0385	Missense_Mutation	p.S1166N	Non-Targeted Panel Gene	Adult HGBCL, NOS
BCL-15	chr20	23394989	23394989	G	A	exonic	NAPB	50	2	0.0385	Missense_Mutation	p.T118I	Non-Targeted Panel Gene	Adult HGBCL, NOS
BCL-15	chr2	77519729	77519729	C	T	exonic	LRRTM4	49	2	0.0392	Missense_Mutation	p.C47Y	Non-Targeted Panel Gene	Adult HGBCL, NOS
BCL-15	chr11	77040194	77040194	G	A	exonic	B3GNT6	73	3	0.0395	Missense_Mutation	p.A215T	Non-Targeted Panel Gene	Adult HGBCL, NOS
BCL-15	chr1	15410194	15410194	C	T	exonic	EFHD2	48	2	0.04	Missense_Mutation	p.P75S	Non-Targeted Panel Gene	Adult HGBCL, NOS
BCL-15	chr1	176699393	176699393	G	T	exonic	PAPPA2	48	2	0.04	Missense_Mutation	p.D1014Y	Non-Targeted Panel Gene	Adult HGBCL, NOS
BCL-15	chr8	9710021	9710021	G	A	exonic	TNKS	48	2	0.04	Missense_Mutation	p.A549T	Non-Targeted Panel Gene	Adult HGBCL, NOS
BCL-15	chr11	11940441	11940441	C	T	exonic	USP47	48	2	0.04	Missense_Mutation	p.P756S	Non-Targeted Panel Gene	Adult HGBCL, NOS
BCL-15	chr1	150553733	150553733	C	T	exonic	ADAMTSL4	71	3	0.0405	Missense_Mutation	p.P248S	Non-Targeted Panel Gene	Adult HGBCL, NOS
BCL-15	chr2	86490968	86490968	C	T	exonic	KDM3A	70	3	0.0411	Missense_Mutation	p.R1221C	Non-Targeted Panel Gene	Adult HGBCL, NOS
BCL-15	chr1	28280796	28280796	G	A	exonic	SESN2	46	2	0.0417	Missense_Mutation	p.M479I	Non-Targeted Panel Gene	Adult HGBCL, NOS
BCL-15	chr11	65133507	65133507	G	A	exonic	SYVN1	68	3	0.0423	Missense_Mutation	p.T32I	Non-Targeted Panel Gene	Adult HGBCL, NOS
BCL-15	chr1	46006287	46006287	C	T	exonic	MAST2	45	2	0.0426	Missense_Mutation	p.P272L	Non-Targeted Panel Gene	Adult HGBCL, NOS
BCL-15	chr1	202948369	202948369	G	A	exonic	ADIPOR1	65	3	0.0429	Missense_Mutation	p.R65W	Non-Targeted Panel Gene	Adult HGBCL, NOS
BCL-15	chr6	47009554	47009554	C	T	exonic	ADGRF1	43	2	0.0435	Missense_Mutation	p.M627I	Non-Targeted Panel Gene	Adult HGBCL, NOS
BCL-15	chr4	151650175	151650175	C	T	exonic	FAM160A1	44	2	0.0435	Missense_Mutation	p.P712S	Non-Targeted Panel Gene	Adult HGBCL, NOS
BCL-15	chr7	142930420	142930420	G	A	exonic	TRPV5	44	2	0.0435	Missense_Mutation	p.A52V	Non-Targeted Panel Gene	Adult HGBCL, NOS
BCL-15	chr6	131674163	131674163	G	A	exonic	ENPP3	43	2	0.0444	Missense_Mutation	p.G215D	Non-Targeted Panel Gene	Adult HGBCL, NOS
BCL-15	chr17	50358483	50358483	G	A	exonic	XYLT2	64	3	0.0448	Missense_Mutation	p.E740K	Non-Targeted Panel Gene	Adult HGBCL, NOS
BCL-15	chr17	42405063	42405063	G	A	exonic	CAVIN1	84	4	0.0455	Missense_Mutation	p.T266M	Non-Targeted Panel Gene	Adult HGBCL, NOS
BCL-15	chr6	556530	556530	A	G	exonic	EXOC2	42	2	0.0455	Missense_Mutation	p.L629P	Non-Targeted Panel Gene	Adult HGBCL, NOS
BCL-15	chr7	6154612	6154612	C	T	exonic	USP42	42	2	0.0455	Missense_Mutation	p.H1020Y	Non-Targeted Panel Gene	Adult HGBCL, NOS
BCL-15	chr15	40899689	40899689	G	A	exonic	VPS18	42	2	0.0455	Missense_Mutation	p.G291S	Non-Targeted Panel Gene	Adult HGBCL, NOS
BCL-15	chr18	76496460	76496460	G	A	exonic	C18orf65	61	3	0.0462	Missense_Mutation	p.R87H	Non-Targeted Panel Gene	Adult HGBCL, NOS
BCL-15	chr17	75181146	75181146	C	T	exonic	SUMO2	41	2	0.0465	Missense_Mutation	p.V22M	Non-Targeted Panel Gene	Adult HGBCL, NOS
BCL-15	chr10	5747829	5747829	G	A	exonic	FAM208B	40	2	0.0476	Missense_Mutation	p.V1470I	Non-Targeted Panel Gene	Adult HGBCL, NOS
BCL-15	chr20	44186671	44186671	C	T	exonic	JPH2	60	3	0.0476	Missense_Mutation	p.G12E	Non-Targeted Panel Gene	Adult HGBCL, NOS
BCL-15	chr1	247815453	247815453	G	A	exonic	OR14A16	40	2	0.0476	Missense_Mutation	p.L93F	Non-Targeted Panel Gene	Adult HGBCL, NOS
BCL-15	chr8	94250410	94250410	C	T	exonic	GEM	59	3	0.0484	Missense_Mutation	p.R264K	Non-Targeted Panel Gene	Adult HGBCL, NOS
BCL-15	chr2	195816859	195816859	G	A	exonic	DNAH7	39	2	0.0488	Missense_Mutation	p.A3177V	Non-Targeted Panel Gene	Adult HGBCL, NOS
BCL-15	chr19	8948682	8948682	G	A	exonic	MUC16	39	2	0.0488	Missense_Mutation	p.T9363I	Non-Targeted Panel Gene	Adult HGBCL, NOS
BCL-15	chr6	31647685	31647685	C	T	exonic	BAG6	38	2	0.05	Missense_Mutation	p.E232K	Non-Targeted Panel Gene	Adult HGBCL, NOS
BCL-15	chr10	93389035	93389035	C	T	exonic	MYOF	38	2	0.05	Missense_Mutation	p.G526R	Non-Targeted Panel Gene	Adult HGBCL, NOS
BCL-15	chr12	94578473	94578473	G	A	exonic	TMCC3	38	2	0.05	Missense_Mutation	p.T351I	Non-Targeted Panel Gene	Adult HGBCL, NOS
BCL-15	chr4	143697830	143697830	C	T	exonic	FREM3	36	2	0.0526	Missense_Mutation	p.S949N	Non-Targeted Panel Gene	Adult HGBCL, NOS
BCL-15	chr7	128395149	128395149	C	A	exonic	IMPDH1	54	3	0.0526	Missense_Mutation	p.A463S	Non-Targeted Panel Gene	Adult HGBCL, NOS
BCL-15	chr22	19275749	19275749	C	T	exonic	CLTCL1	35	2	0.0541	Missense_Mutation	p.E42K	Non-Targeted Panel Gene	Adult HGBCL, NOS
BCL-15	chr3	50300551	50300551	C	T	exonic	HYAL1	35	2	0.0541	Missense_Mutation	p.V414M	Non-Targeted Panel Gene	Adult HGBCL, NOS
BCL-15	chr6	160010754	160010754	G	A	exonic	IGF2R	35	2	0.0541	Missense_Mutation	p.C161Y	Non-Targeted Panel Gene	Adult HGBCL, NOS
BCL-15	chr17	4938425	4938425	C	T	exonic	SLC25A11	35	2	0.0541	Missense_Mutation	p.C184Y	Non-Targeted Panel Gene	Adult HGBCL, NOS
BCL-15	chr9	105546476	105546476	C	T	exonic	FSD1L	34	2	0.0556	Missense_Mutation	p.T508I	Non-Targeted Panel Gene	Adult HGBCL, NOS
BCL-15	chrX	119943466	119943466	C	T	exonic	NKAP	34	2	0.0556	Missense_Mutation	p.C47Y	Non-Targeted Panel Gene	Adult HGBCL, NOS
BCL-15	chr8	132839514	132839514	C	A	exonic	PHF20L1	51	3	0.0556	Missense_Mutation	p.H773Q	Non-Targeted Panel Gene	Adult HGBCL, NOS
BCL-15	chr15	92444782	92444782	G	A	exonic	ST8SIA2	34	2	0.0556	Missense_Mutation	p.S232N	Non-Targeted Panel Gene	Adult HGBCL, NOS
BCL-15	chr2	233618497	233618497	C	T	exonic	UGT1A8	34	2	0.0556	Missense_Mutation	p.P264S	Non-Targeted Panel Gene	Adult HGBCL, NOS
BCL-15	chr10	86500338	86500338	G	A	exonic	WAPL	67	4	0.0563	Missense_Mutation	p.T302M	Non-Targeted Panel Gene	Adult HGBCL, NOS
BCL-15	chr1	202015238	202015238	G	A	exonic	ELF3	66	4	0.0571	Missense_Mutation	p.R344Q	Non-Targeted Panel Gene	Adult HGBCL, NOS
BCL-15	chr8	41987472	41987472	G	A	exonic	KAT6A	33	2	0.0571	Missense_Mutation	p.A231V	Non-Targeted Panel Gene	Adult HGBCL, NOS
BCL-15	chr12	103733046	103733046	C	T	exonic	STAB2	33	2	0.0571	Missense_Mutation	p.T1775I	Non-Targeted Panel Gene	Adult HGBCL, NOS
BCL-15	chr13	23358350	23358350	C	T	exonic	SACS	82	5	0.0575	Missense_Mutation	p.V197I	Non-Targeted Panel Gene	Adult HGBCL, NOS
BCL-15	chr11	46668827	46668827	C	A	exonic	ATG13	31	2	0.0588	Missense_Mutation	p.P422T	Non-Targeted Panel Gene	Adult HGBCL, NOS
BCL-15	chr17	43522147	43522147	G	A	exonic	DHX8	32	2	0.0588	Missense_Mutation	p.E1122K	Non-Targeted Panel Gene	Adult HGBCL, NOS
BCL-15	chr21	44893435	44893435	C	T	exonic	ITGB2	32	2	0.0588	Missense_Mutation	p.G329D	Non-Targeted Panel Gene	Adult HGBCL, NOS
BCL-15	chr9	35957548	35957548	G	T	exonic	OR2S2	32	2	0.0588	Missense_Mutation	p.A184D	Non-Targeted Panel Gene	Adult HGBCL, NOS
BCL-15	chr2	47798899	47798899	G	A	exonic	MSH6	31	2	0.0606	Missense_Mutation	p.G4R	Non-Targeted Panel Gene	Adult HGBCL, NOS
BCL-15	chr12	117286252	117286252	G	A	exonic	NOS1	31	2	0.0606	Missense_Mutation	p.A381V	Non-Targeted Panel Gene	Adult HGBCL, NOS
BCL-15	chr17	80846475	80846475	C	A	exonic	RPTOR	29	2	0.0606	Missense_Mutation	p.H405Q	Non-Targeted Panel Gene	Adult HGBCL, NOS

BCL-15	chr16	13935457	13935457	G	A	exonic	ERCC4	29	2	0.0625	Missense_Mutation	p.D509N	Non-Targeted Panel Gene	Adult HGBCL, NOS
BCL-15	chr10	100176046	100176046	G	A	exonic	ERLIN1	30	2	0.0625	Missense_Mutation	p.T110I	Non-Targeted Panel Gene	Adult HGBCL, NOS
BCL-15	chr4	186251254	186251254	C	A	exonic	KLKB1	30	2	0.0625	Missense_Mutation	p.T227K	Non-Targeted Panel Gene	Adult HGBCL, NOS
BCL-15	chr21	30319951	30319951	G	A	exonic	KRTAP26-1	30	2	0.0625	Missense_Mutation	p.L29F	Non-Targeted Panel Gene	Adult HGBCL, NOS
BCL-15	chr2	178072289	178072289	C	T	exonic	PDE11A	30	2	0.0625	Missense_Mutation	p.G50D	Non-Targeted Panel Gene	Adult HGBCL, NOS
BCL-15	chr20	20032563	20032563	A	G	exonic	NAA20	86	6	0.0652	Missense_Mutation	p.N109D	Non-Targeted Panel Gene	Adult HGBCL, NOS
BCL-15	chr5	141370776	141370776	G	A	exonic	PCDHGB3	57	4	0.0656	Missense_Mutation	p.D128N	Non-Targeted Panel Gene	Adult HGBCL, NOS
BCL-15	chr6	42704499	42704499	C	T	exonic	PRPH2	55	4	0.0667	Missense_Mutation	p.A232T	Non-Targeted Panel Gene	Adult HGBCL, NOS
BCL-15	chr10	72007800	72007800	G	A	exonic	CHST3	55	4	0.0678	Missense_Mutation	p.V257M	Non-Targeted Panel Gene	Adult HGBCL, NOS
BCL-15	chr9	34506785	34506785	G	A	exonic	DNAI1	27	2	0.069	Missense_Mutation	p.V408M	Non-Targeted Panel Gene	Adult HGBCL, NOS
BCL-15	chr8	11771502	11771502	G	T	exonic	NEIL2	25	2	0.069	Missense_Mutation	p.G19C	Non-Targeted Panel Gene	Adult HGBCL, NOS
BCL-15	chr2	28538287	28538287	C	T	exonic	PLB1	26	2	0.069	Missense_Mutation	p.A186V	Non-Targeted Panel Gene	Adult HGBCL, NOS
BCL-15	chr22	26512410	26512410	C	T	exonic	TFIP11	27	2	0.069	Missense_Mutation	p.R8H	Non-Targeted Panel Gene	Adult HGBCL, NOS
BCL-15	chr2	70696315	70696315	C	T	exonic	ADD2	26	2	0.0714	Missense_Mutation	p.C135Y	Non-Targeted Panel Gene	Adult HGBCL, NOS
BCL-15	chr10	113604875	113604875	C	A	exonic	NRAP	26	2	0.0714	Missense_Mutation	p.G1321W	Non-Targeted Panel Gene	Adult HGBCL, NOS
BCL-15	chr19	30005363	30005363	G	A	exonic	URI1	26	2	0.0714	Missense_Mutation	p.V124I	Non-Targeted Panel Gene	Adult HGBCL, NOS
BCL-15	chr19	55622034	55622034	C	A	exonic	ZNF784	26	2	0.0714	Missense_Mutation	p.G230V	Non-Targeted Panel Gene	Adult HGBCL, NOS
BCL-15	chr11	67423693	67423693	G	A	exonic	CARNS1	25	2	0.0741	Missense_Mutation	p.A649T	Non-Targeted Panel Gene	Adult HGBCL, NOS
BCL-15	chr14	77475624	77475624	C	A	exonic	ISM2	25	2	0.0741	Missense_Mutation	p.A563S	Non-Targeted Panel Gene	Adult HGBCL, NOS
BCL-15	chr8	28339268	28339268	G	A	exonic	PNOC	49	4	0.0755	Missense_Mutation	p.G119S	Non-Targeted Panel Gene	Adult HGBCL, NOS
BCL-15	chr11	67523318	67523318	G	A	exonic	CABP2	36	3	0.0769	Missense_Mutation	p.T8I	Non-Targeted Panel Gene	Adult HGBCL, NOS
BCL-15	chr9	17464502	17464502	C	T	exonic	CNTLN	23	2	0.0769	Missense_Mutation	p.S1137F	Non-Targeted Panel Gene	Adult HGBCL, NOS
BCL-15	chr1	227921685	227921685	G	A	exonic	WNT9A	35	3	0.0769	Missense_Mutation	p.R311C	Non-Targeted Panel Gene	Adult HGBCL, NOS
BCL-15	chr5	79237122	79237122	G	T	exonic	JMY	23	2	0.08	Missense_Mutation	p.D158Y	Non-Targeted Panel Gene	Adult HGBCL, NOS
BCL-15	chr4	30721916	30721916	C	T	exonic	PCDH7	21	2	0.087	Missense_Mutation	p.P165L	Non-Targeted Panel Gene	Adult HGBCL, NOS
BCL-15	chr14	50916701	50916701	G	A	exonic	PYGL	21	2	0.087	Missense_Mutation	p.L345F	Non-Targeted Panel Gene	Adult HGBCL, NOS
BCL-15	chr17	65225393	65225393	C	T	exonic	RGS9	21	2	0.087	Missense_Mutation	p.S600L	Non-Targeted Panel Gene	Adult HGBCL, NOS
BCL-15	chr1	167854472	167854472	C	A	exonic	ADCY10	52	5	0.0877	Missense_Mutation	p.S730I	Non-Targeted Panel Gene	Adult HGBCL, NOS
BCL-15	chr22	31806169	31806169	G	A	exonic	DEPDC5	52	5	0.0877	Missense_Mutation	p.R422Q	Non-Targeted Panel Gene	Adult HGBCL, NOS
BCL-15	chr2	206543269	206543269	G	A	exonic	ADAM23	31	3	0.0882	Missense_Mutation	p.D225N	Non-Targeted Panel Gene	Adult HGBCL, NOS
BCL-15	chr17	10462873	10462873	C	T	exonic	MYH4	31	3	0.0882	Missense_Mutation	p.A334T	Non-Targeted Panel Gene	Adult HGBCL, NOS
BCL-15	chr11	8715814	8715814	G	A	exonic	ST5	31	3	0.0882	Missense_Mutation	p.S545F	Non-Targeted Panel Gene	Adult HGBCL, NOS
BCL-15	chr20	63701835	63701835	C	T	exonic	ARFRP1	30	3	0.0909	Missense_Mutation	p.V138M	Non-Targeted Panel Gene	Adult HGBCL, NOS
BCL-15	chr3	129520243	129520243	G	A	exonic	IFT122	30	3	0.0909	Missense_Mutation	p.C1286Y	Non-Targeted Panel Gene	Adult HGBCL, NOS
BCL-15	chr22	30888340	30888340	G	A	exonic	OSBP2	20	2	0.0909	Missense_Mutation	p.R308K	Non-Targeted Panel Gene	Adult HGBCL, NOS
BCL-15	chr20	1598895	1598895	G	A	exonic	SIRPB1	20	2	0.0909	Missense_Mutation	p.L376F	Non-Targeted Panel Gene	Adult HGBCL, NOS
BCL-15	chr17	38720512	38720512	G	A	exonic	MLLT6	29	3	0.0938	Missense_Mutation	p.A766T	Non-Targeted Panel Gene	Adult HGBCL, NOS
BCL-15	chr4	24799864	24799864	G	A	exonic	SOD3	29	3	0.0938	Missense_Mutation	p.V115M	Non-Targeted Panel Gene	Adult HGBCL, NOS
BCL-15	chr4	109869810	109869810	C	T	exonic	LRIT3	19	2	0.0952	Missense_Mutation	p.P354L	Non-Targeted Panel Gene	Adult HGBCL, NOS
BCL-15	chr3	94064542	94064542	G	A	exonic	NSUN3	19	2	0.0952	Missense_Mutation	p.V40I	Non-Targeted Panel Gene	Adult HGBCL, NOS
BCL-15	chr19	2917447	2917447	G	A	exonic	ZNF57	28	3	0.0968	Missense_Mutation	p.G276R	Non-Targeted Panel Gene	Adult HGBCL, NOS
BCL-15	chr22	19721899	19721899	G	A	exonic	SEPTIN5	27	3	0.1	Missense_Mutation	p.V298M	Non-Targeted Panel Gene	Adult HGBCL, NOS
BCL-15	chr7	95528314	95528314	G	A	exonic	ASB4	27	3	0.1	Missense_Mutation	p.C330Y	Non-Targeted Panel Gene	Adult HGBCL, NOS
BCL-15	chr7	128809597	128809597	C	A	exonic	CCDC136	18	2	0.1	Missense_Mutation	p.L585M	Non-Targeted Panel Gene	Adult HGBCL, NOS
BCL-15	chr11	66042586	66042586	C	T	exonic	GAL3ST3	18	2	0.1	Missense_Mutation	p.R406Q	Non-Targeted Panel Gene	Adult HGBCL, NOS
BCL-15	chr19	17251939	17251939	C	T	exonic	USHBP1	18	2	0.1	Missense_Mutation	p.A591T	Non-Targeted Panel Gene	Adult HGBCL, NOS
BCL-15	chr1	75919020	75919020	G	A	exonic	ASB17	25	3	0.1071	Missense_Mutation	p.L274F	Non-Targeted Panel Gene	Adult HGBCL, NOS
BCL-15	chr2	69328420	69328420	A	C	exonic	GFPT1	25	3	0.1071	Missense_Mutation	p.Y582D	Non-Targeted Panel Gene	Adult HGBCL, NOS
BCL-15	chr12	2691108	2691108	G	A	exonic	CACNA1C	16	2	0.1111	Missense_Mutation	p.G2192E	Non-Targeted Panel Gene	Adult HGBCL, NOS
BCL-15	chr7	100174761	100174761	G	A	exonic	GPC2	24	3	0.1111	Missense_Mutation	p.T218I	Non-Targeted Panel Gene	Adult HGBCL, NOS
BCL-15	chr9	27017016	27017016	C	A	exonic	IFT74	16	2	0.1111	Missense_Mutation	p.S300Y	Non-Targeted Panel Gene	Adult HGBCL, NOS
BCL-15	chr6	42968918	42968918	C	T	exonic	PEX6	24	3	0.1111	Missense_Mutation	p.V391I	Non-Targeted Panel Gene	Adult HGBCL, NOS
BCL-15	chr17	50278911	50278911	G	T	exonic	TMEM92	24	3	0.1111	Missense_Mutation	p.C94F	Non-Targeted Panel Gene	Adult HGBCL, NOS
BCL-15	chr6	80036595	80036595	C	A	exonic	TTK	16	2	0.1111	Missense_Mutation	p.S682Y	Non-Targeted Panel Gene	Adult HGBCL, NOS
BCL-15	chr1	225331543	225331543	C	T	exonic	DNAH14	78	10	0.1136	Missense_Mutation	p.A3184V	Non-Targeted Panel Gene	Adult HGBCL, NOS
BCL-15	chr10	22345771	22345771	C	T	exonic	SPAG6	23	3	0.1154	Missense_Mutation	p.A25V	Non-Targeted Panel Gene	Adult HGBCL, NOS
BCL-15	chr2	218873795	218873795	G	A	exonic	WNT6	23	3	0.1154	Missense_Mutation	p.V350I	Non-Targeted Panel Gene	Adult HGBCL, NOS
BCL-15	chr22	38214520	38214520	C	T	exonic	MAFF	15	2	0.1176	Missense_Mutation	p.A46V	Non-Targeted Panel Gene	Adult HGBCL, NOS
BCL-15	chr19	38915625	38915625	C	T	exonic	SARS2	15	2	0.1176	Missense_Mutation	p.G515D	Non-Targeted Panel Gene	Adult HGBCL, NOS
BCL-15	chr10	109877857	109877857	C	T	exonic	XPNPEP1	15	2	0.1176	Missense_Mutation	p.D418N	Non-Targeted Panel Gene	Adult HGBCL, NOS
BCL-15	chr12	120160162	120160162	C	T	exonic	GCN1	22	3	0.12	Missense_Mutation	p.V844I	Non-Targeted Panel Gene	Adult HGBCL, NOS
BCL-15	chr3	183833529	183833529	C	T	exonic	PARL	22	3	0.12	Missense_Mutation	p.D331N	Non-Targeted Panel Gene	Adult HGBCL, NOS
BCL-15	chr11	120327380	120327380	C	T	exonic	TMEM136	22	3	0.12	Missense_Mutation	p.T2I	Non-Targeted Panel Gene	Adult HGBCL, NOS
BCL-15	chr6	42104680	42104680	G	A	exonic	C6orf132	14	2	0.125	Missense_Mutation	p.P1078S	Non-Targeted Panel Gene	Adult HGBCL, NOS
BCL-15	chr2	232535212	232535212	C	T	exonic	CHRND	21	3	0.125	Missense_Mutation	p.T384I	Non-Targeted Panel Gene	Adult HGBCL, NOS
BCL-15	chr4	967883	967883	G	A	exonic	DGKQ	14	2	0.125	Missense_Mutation	p.P270S	Non-Targeted Panel Gene	Adult HGBCL, NOS
BCL-15	chr14	44505257	44505257	C	A	exonic	FSCB	14	2	0.125	Missense_Mutation	p.Q577H	Non-Targeted Panel Gene	Adult HGBCL, NOS
BCL-15	chr10	25175758	25175758	C	T	exonic	GPR158	14	2	0.125	Missense_Mutation	p.P113L	Non-Targeted Panel Gene	Adult HGBCL, NOS
BCL-15	chr20	38223540	38223540	G	T	exonic	KIAA1755	14	2	0.125	Missense_Mutation	p.Q756K	Non-Targeted Panel Gene	Adult HGBCL, NOS
BCL-15	chrX	68096947	68096947	C	T	exonic	OPHN1	14	2	0.125	Missense_Mutation	p.E537K	Non-Targeted Panel Gene	Adult HGBCL, NOS
BCL-15	chr3	142962367	142962367	C	T	exonic	PAQR9	21	3	0.125	Missense_Mutation	p.D324N	Non-Targeted Panel Gene	Adult HGBCL, NOS
BCL-15	chr5	131479543	131479543	C	T	exonic	RAPGEF6	28	4	0.125	Missense_Mutation	p.R684Q	Non-Targeted Panel Gene	Adult HGBCL, NOS
BCL-15	chr9	133690497	133690497	G	A	exonic	SARDH	14	2	0.125	Missense_Mutation	p.A651V	Non-Targeted Panel Gene	Adult HGBCL, NOS
BCL-15	chr19	1220609	1220609	C	A	exonic	STK11	14	2	0.125	Missense_Mutation	p.T209N	Non-Targeted Panel Gene	Adult HGBCL, NOS
BCL-15	chr17	39684715	39684715	G	A	exonic	PGAP3	27	4	0.129	Missense_Mutation	p.P105L	Non-Targeted Panel Gene	Adult HGBCL, NOS
BCL-15	chr1	42735884	42735884	G	A	exonic	CLDN19	13	2	0.1333	Missense_Mutation	p.A207V	Non-Targeted Panel Gene	Adult HGBCL, NOS
BCL-15	chr2	3742045	3742045	G	A	exonic	DCDC2C	13	2	0.1333	Missense_Mutation	p.R181Q	Non-Targeted Panel Gene	Adult HGBCL, NOS
BCL-15	chr3	69937822	69937822	G	A	exonic	MITF	13	2	0.1333	Missense_Mutation	p.V103M	Non-Targeted Panel Gene	Adult HGBCL, NOS
BCL-15	chr7	151385100	151385100	G	A	exonic	WDR86	13	2	0.1333	Missense_Mutation	p.H284Y	Non-Targeted Panel Gene	Adult HGBCL, NOS
BCL-15	chr22	41528510	41528510	G	A	exonic	ACO2	12	2	0.1429	Missense_Mutation	p.G747E	Non-Targeted Panel Gene	Adult HGBCL, NOS
BCL-15	chr4	95125090	95125090	G	A	exonic	BMPR1B	12	2	0.1429	Missense_Mutation	p.S185N	Non-Targeted Panel Gene	Adult HGBCL, NOS
BCL-15	chr11	67421170	67421170	G	A	exonic	CARNS1	12	2	0.1429	Missense_Mutation	p.G526D	Non-Targeted Panel Gene	Adult HGBCL, NOS

BCL-15	chr13	110495459	110495459	G	A	exonic	COL4A2	12	2	0.1429	Missense_Mutation	p.G1251E	Non-Targeted Panel Gene	Adult HGBCL, NOS
BCL-15	chr9	77020682	77020682	C	T	exonic	FOXB2	18	3	0.1429	Missense_Mutation	p.A343V	Non-Targeted Panel Gene	Adult HGBCL, NOS
BCL-15	chr7	56083675	56083675	C	T	exonic	PHKG1	30	5	0.1429	Missense_Mutation	p.V152I	Non-Targeted Panel Gene	Adult HGBCL, NOS
BCL-15	chr1	20139504	20139504	G	A	exonic	PLA2G2F	12	2	0.1429	Missense_Mutation	p.G26D	Non-Targeted Panel Gene	Adult HGBCL, NOS
BCL-15	chr2	195718262	195718262	C	A	exonic	SLC39A10	12	2	0.1429	Missense_Mutation	p.F692L	Non-Targeted Panel Gene	Adult HGBCL, NOS
BCL-15	chr1	25458444	25458444	G	A	exonic	TMEM57	18	3	0.1429	Missense_Mutation	p.G236S	Non-Targeted Panel Gene	Adult HGBCL, NOS
BCL-15	chr7	6654010	6654010	G	A	exonic	ZNF316	12	2	0.1429	Missense_Mutation	p.C805Y	Non-Targeted Panel Gene	Adult HGBCL, NOS
BCL-15	chr16	31436491	31436491	G	A	exonic	ZNF843	12	2	0.1429	Missense_Mutation	p.P120L	Non-Targeted Panel Gene	Adult HGBCL, NOS
BCL-15	chr17	52985691	52985691	C	T	exonic	C17orf112	35	6	0.1463	Missense_Mutation	p.L5F	Non-Targeted Panel Gene	Adult HGBCL, NOS
BCL-15	chr9	2073320	2073320	G	A	exonic	SMARCA2	17	3	0.15	Missense_Mutation	p.A619T	Non-Targeted Panel Gene	Adult HGBCL, NOS
BCL-15	chr9	89378711	89378711	C	T	exonic	SEMA4D	11	2	0.1538	Missense_Mutation	p.G861E	Non-Targeted Panel Gene	Adult HGBCL, NOS
BCL-15	chr22	27982303	27982303	G	A	exonic	TTC28	11	2	0.1538	Missense_Mutation	p.P2455L	Non-Targeted Panel Gene	Adult HGBCL, NOS
BCL-15	chr18	46542727	46542727	C	T	exonic	LOXHD1	16	3	0.1579	Missense_Mutation	p.G1250S	Non-Targeted Panel Gene	Adult HGBCL, NOS
BCL-15	chr20	43570656	43570656	C	T	exonic	SGK2	16	3	0.1579	Missense_Mutation	p.P134S	Non-Targeted Panel Gene	Adult HGBCL, NOS
BCL-15	chr12	64390319	64390319	C	T	exonic	C12orf56	10	2	0.1667	Missense_Mutation	p.D83N	Non-Targeted Panel Gene	Adult HGBCL, NOS
BCL-15	chr4	1403429	1403429	G	A	exonic	NKX1-1	10	2	0.1667	Missense_Mutation	p.R284W	Non-Targeted Panel Gene	Adult HGBCL, NOS
BCL-15	chr15	38498812	38498812	C	T	exonic	RASGRP1	10	2	0.1667	Missense_Mutation	p.A619T	Non-Targeted Panel Gene	Adult HGBCL, NOS
BCL-15	chr17	73438008	73438008	C	T	exonic	SDK2	15	3	0.1667	Missense_Mutation	p.S291N	Non-Targeted Panel Gene	Adult HGBCL, NOS
BCL-15	chr3	39185800	39185800	G	T	exonic	XIRP1	10	2	0.1667	Missense_Mutation	p.P1216T	Non-Targeted Panel Gene	Adult HGBCL, NOS
BCL-15	chr17	7577584	7577584	A	G	exonic	EIF4A1	24	5	0.1724	Missense_Mutation	p.T262A	Non-Targeted Panel Gene	Adult HGBCL, NOS
BCL-15	chr1	151401326	151401326	T	G	exonic	PSMB4	59	13	0.1781	Missense_Mutation	p.Y222D	Non-Targeted Panel Gene	Adult HGBCL, NOS
BCL-15	chr16	50722629	50722629	G	C	exonic	NOD2	69	15	0.1786	Missense_Mutation	p.G908R	Non-Targeted Panel Gene	Adult HGBCL, NOS
BCL-15	chr6	149863917	149863917	C	T	exonic	LRP11	9	2	0.1818	Missense_Mutation	p.G35D	Non-Targeted Panel Gene	Adult HGBCL, NOS
BCL-15	chr17	42536627	42536627	G	A	exonic	NAGLU	9	2	0.1818	Missense_Mutation	p.G119R	Non-Targeted Panel Gene	Adult HGBCL, NOS
BCL-15	chr15	99131857	99131857	G	A	exonic	SYNM	9	2	0.1818	Missense_Mutation	p.G1166E	Non-Targeted Panel Gene	Adult HGBCL, NOS
BCL-15	chr2	188993422	188993422	C	G	exonic	COL3A1	31	7	0.1842	Missense_Mutation	p.P371R	Non-Targeted Panel Gene	Adult HGBCL, NOS
BCL-15	chr5	150848245	150848245	A	G	exonic	IRGM	22	5	0.1852	Missense_Mutation	p.D41G	Non-Targeted Panel Gene	Adult HGBCL, NOS
BCL-15	chr6	30642214	30642214	G	T	exonic	ATAT1	61	14	0.1867	Missense_Mutation	p.D231Y	Non-Targeted Panel Gene	Adult HGBCL, NOS
BCL-15	chr9	128753204	128753204	C	T	exonic	ZER1	13	3	0.1875	Missense_Mutation	p.D236N	Non-Targeted Panel Gene	Adult HGBCL, NOS
BCL-15	chr5	7826768	7826768	C	T	exonic	ADCY2	17	4	0.1905	Missense_Mutation	p.T1058I	Non-Targeted Panel Gene	Adult HGBCL, NOS
BCL-15	chr1	152155013	152155013	T	C	exonic	RPTN	63	15	0.1923	Missense_Mutation	p.S696G	Non-Targeted Panel Gene	Adult HGBCL, NOS
BCL-15	chr7	14574263	14574263	T	A	exonic	DGKB	61	15	0.1974	Missense_Mutation	p.K532N	Non-Targeted Panel Gene	Adult HGBCL, NOS
BCL-15	chr17	36989406	36989406	G	A	exonic	AATF	12	3	0.2	Missense_Mutation	p.E437K	Non-Targeted Panel Gene	Adult HGBCL, NOS
BCL-15	chr10	35640952	35640952	C	T	exonic	FZD8	7	2	0.2222	Missense_Mutation	p.A160T	Non-Targeted Panel Gene	Adult HGBCL, NOS
BCL-15	chr2	219572477	219572477	G	A	exonic	INHA	14	4	0.2222	Missense_Mutation	p.A35T	Non-Targeted Panel Gene	Adult HGBCL, NOS
BCL-15	chr17	41190493	41190493	C	G	exonic	KRTAP9-1	42	12	0.2222	Missense_Mutation	p.Q203E	Non-Targeted Panel Gene	Adult HGBCL, NOS
BCL-15	chr17	80288173	80288173	C	T	exonic	RNF213	20	6	0.2222	Missense_Mutation	p.A207V	Non-Targeted Panel Gene	Adult HGBCL, NOS
BCL-15	chr1	185174442	185174442	C	G	exonic	SWT1	34	10	0.2273	Missense_Mutation	p.L99V	Non-Targeted Panel Gene	Adult HGBCL, NOS
BCL-15	chr11	108160996	108160996	T	C	exonic	NPAT	54	16	0.2286	Missense_Mutation	p.R1364G	Non-Targeted Panel Gene	Adult HGBCL, NOS
BCL-15	chr12	21202584	21202584	T	C	exonic	SLCO1B1	53	16	0.2319	Missense_Mutation	p.F410S	Non-Targeted Panel Gene	Adult HGBCL, NOS
BCL-15	chr14	96542796	96542796	A	T	exonic	PAPOLA	62	19	0.2346	Missense_Mutation	p.I398F	Non-Targeted Panel Gene	Adult HGBCL, NOS
BCL-15	chr6	14118039	14118039	G	C	exonic	CD83	29	9	0.2368	Missense_Mutation	p.V43L	Non-Targeted Panel Gene	Adult HGBCL, NOS
BCL-15	chr3	179250771	179250771	T	C	exonic	KCNMB3	28	9	0.2432	Missense_Mutation	p.T56A	Non-Targeted Panel Gene	Adult HGBCL, NOS
BCL-15	chr2	208124294	208124294	G	A	exonic	CRYGD	8	3	0.25	Missense_Mutation	p.P24S	Non-Targeted Panel Gene	Adult HGBCL, NOS
BCL-15	chrX	75784613	75784613	T	A	exonic	MAGEE2	12	4	0.25	Missense_Mutation	p.I147F	Non-Targeted Panel Gene	Adult HGBCL, NOS
BCL-15	chr5	36241714	36241714	C	T	exonic	NADK2	9	3	0.25	Missense_Mutation	p.G29R	Non-Targeted Panel Gene	Adult HGBCL, NOS
BCL-15	chr16	2804403	2804403	C	T	exonic	PRSS41	6	2	0.25	Missense_Mutation	p.P199S	Non-Targeted Panel Gene	Adult HGBCL, NOS
BCL-15	chr15	62675230	62675230	G	A	exonic	TLN2	12	4	0.25	Missense_Mutation	p.C289Y	Non-Targeted Panel Gene	Adult HGBCL, NOS
BCL-15	chr16	4874227	4874227	C	T	exonic	UBN1	6	2	0.25	Missense_Mutation	p.P606L	Non-Targeted Panel Gene	Adult HGBCL, NOS
BCL-15	chr3	39187633	39187633	C	T	exonic	XIRP1	62	21	0.253	Missense_Mutation	p.E605K	Non-Targeted Panel Gene	Adult HGBCL, NOS
BCL-15	chr5	16067520	16067520	G	A	exonic	MARCHF11	29	10	0.2564	Missense_Mutation	p.S387L	Non-Targeted Panel Gene	Adult HGBCL, NOS
BCL-15	chr1	220100585	220100585	A	G	exonic	IARS2	31	11	0.2619	Missense_Mutation	p.I162M	Non-Targeted Panel Gene	Adult HGBCL, NOS
BCL-15	chr15	56142802	56142802	T	G	exonic	RFX7	27	10	0.2703	Missense_Mutation	p.K126T	Non-Targeted Panel Gene	Adult HGBCL, NOS
BCL-15	chr9	135818548	135818548	T	C	exonic	CAMSAP1	16	6	0.2727	Missense_Mutation	p.E1343G	Non-Targeted Panel Gene	Adult HGBCL, NOS
BCL-15	chr2	27329171	27329171	C	A	exonic	GTF3C2	21	8	0.2759	Missense_Mutation	p.W663C	Non-Targeted Panel Gene	Adult HGBCL, NOS
BCL-15	chr17	14206977	14206977	G	T	exonic	COX10	39	15	0.2778	Missense_Mutation	p.V366L	Non-Targeted Panel Gene	Adult HGBCL, NOS
BCL-15	chr14	95191629	95191629	G	T	exonic	CLMN	40	16	0.2857	Missense_Mutation	p.L982I	Non-Targeted Panel Gene	Adult HGBCL, NOS
BCL-15	chr1	156077407	156077407	C	T	exonic	MEX3A	32	13	0.2889	Missense_Mutation	p.A244T	Non-Targeted Panel Gene	Adult HGBCL, NOS
BCL-15	chr3	81648904	81648904	A	G	exonic	GBE1	12	5	0.2941	Missense_Mutation	p.S215P	Non-Targeted Panel Gene	Adult HGBCL, NOS
BCL-15	chr19	36819434	36819434	G	A	exonic	ZNF790	26	11	0.2973	Missense_Mutation	p.H304Y	Non-Targeted Panel Gene	Adult HGBCL, NOS
BCL-15	chr12	8850216	8850216	G	T	exonic	A2ML1	40	18	0.3103	Missense_Mutation	p.D726Y	Non-Targeted Panel Gene	Adult HGBCL, NOS
BCL-15	chr4	39745790	39745790	T	A	exonic	UBE2K	50	23	0.3151	Missense_Mutation	p.Y66N	Non-Targeted Panel Gene	Adult HGBCL, NOS
BCL-15	chr18	10794984	10794984	G	A	exonic	PIEZO2	13	6	0.3158	Missense_Mutation	p.H516Y	Non-Targeted Panel Gene	Adult HGBCL, NOS
BCL-15	chr2	113241644	113241644	G	T	exonic	PAX8	30	14	0.3182	Missense_Mutation	p.F228L	Non-Targeted Panel Gene	Adult HGBCL, NOS
BCL-15	chrX	78755608	78755608	G	A	exonic	LPAR4	29	14	0.3256	Missense_Mutation	p.V247I	Non-Targeted Panel Gene	Adult HGBCL, NOS
BCL-15	chrX	56565258	56565258	T	C	exonic	UBQLN2	20	10	0.3333	Missense_Mutation	p.L462P	Non-Targeted Panel Gene	Adult HGBCL, NOS
BCL-15	chr15	37095570	37095570	G	T	exonic	MEIS2	23	12	0.3429	Missense_Mutation	p.D144E	Non-Targeted Panel Gene	Adult HGBCL, NOS
BCL-15	chr5	40950009	40950009	C	T	exonic	C7	19	10	0.3448	Missense_Mutation	p.A363V	Non-Targeted Panel Gene	Adult HGBCL, NOS
BCL-15	chrX	47244739	47244739	G	A	exonic	USP11	17	9	0.3462	Missense_Mutation	p.R677Q	Non-Targeted Panel Gene	Adult HGBCL, NOS
BCL-15	chr7	81710220	81710220	T	G	exonic	HGF	40	22	0.3548	Missense_Mutation	p.T485P	Non-Targeted Panel Gene	Adult HGBCL, NOS
BCL-15	chr7	11037070	11037070	G	C	exonic	PHF14	26	15	0.3659	Missense_Mutation	p.K653N	Non-Targeted Panel Gene	Adult HGBCL, NOS
BCL-15	chr3	67498167	67498167	A	T	exonic	SUCLG2	40	25	0.3846	Missense_Mutation	p.Y296N	Non-Targeted Panel Gene	Adult HGBCL, NOS
BCL-15	chr1	181483764	181483764	C	T	exonic	CACNA1E	28	18	0.3913	Missense_Mutation	p.A7V	Non-Targeted Panel Gene	Adult HGBCL, NOS
BCL-15	chr6	96604809	96604809	T	G	exonic	FHL5	17	11	0.3929	Missense_Mutation	p.N73K	Non-Targeted Panel Gene	Adult HGBCL, NOS
BCL-15	chr1	41583694	41583694	T	G	exonic	HIVEP3	26	17	0.3953	Missense_Mutation	p.L368F	Non-Targeted Panel Gene	Adult HGBCL, NOS
BCL-15	chr4	30722657	30722657	T	A	exonic	PCDH7	24	16	0.4	Missense_Mutation	p.V412E	Non-Targeted Panel Gene	Adult HGBCL, NOS
BCL-15	chr12	101786174	101786174	G	C	exonic	GNPTAB	10	7	0.4118	Missense_Mutation	p.P137A	Non-Targeted Panel Gene	Adult HGBCL, NOS
BCL-15	chr14	24163059	24163059	T	G	exonic	IRF9	27	19	0.413	Missense_Mutation	p.S92A	Non-Targeted Panel Gene	Adult HGBCL, NOS
BCL-15	chr19	9094026	9094026	G	A	exonic	OR1M1	8	6	0.4286	Missense_Mutation	p.C261Y	Non-Targeted Panel Gene	Adult HGBCL, NOS
BCL-15	chr9	340257	340257	C	G	exonic	DOCK8	36	28	0.4375	Missense_Mutation	p.R539G	Non-Targeted Panel Gene	Adult HGBCL, NOS
BCL-15	chr9	131519447	131519447	C	G	exonic	POMT1	22	18	0.439	Missense_Mutation	p.S537R	Non-Targeted Panel Gene	Adult HGBCL, NOS
BCL-15	chrX	75784253	75784253	G	T	exonic	MAGEE2	19	15	0.4412	Missense_Mutation	p.L267M	Non-Targeted Panel Gene	Adult HGBCL, NOS

BCL-15	chr1	1959988	1959988	T	G	exonic	CFAP74	15	13	0.4483	Missense_Mutation	p.E579D	Non-Targeted Panel Gene	Adult HGBCL, NOS
BCL-15	chr17	45996480	45996480	C	T	exonic	MAPT	41	34	0.4533	Missense_Mutation	p.P548L	Non-Targeted Panel Gene	Adult HGBCL, NOS
BCL-15	chr5	177433378	177433378	T	A	exonic	GRK6	16	14	0.4667	Missense_Mutation	p.Y189N	Non-Targeted Panel Gene	Adult HGBCL, NOS
BCL-15	chr14	100734621	100734621	A	G	exonic	DLK1	19	17	0.4722	Missense_Mutation	p.N293D	Non-Targeted Panel Gene	Adult HGBCL, NOS
BCL-15	chr2	178732691	178732691	C	A	exonic	TTN	12	11	0.4783	Missense_Mutation	p.G5457V	Non-Targeted Panel Gene	Adult HGBCL, NOS
BCL-15	chr10	97599137	97599137	C	A	exonic	HOGA1	15	14	0.4828	Missense_Mutation	p.A130D	Non-Targeted Panel Gene	Adult HGBCL, NOS
BCL-15	chr2	177468456	177468456	A	G	exonic	AGPS	19	18	0.4865	Missense_Mutation	p.E346G	Non-Targeted Panel Gene	Adult HGBCL, NOS
BCL-15	chr19	35527014	35527014	C	A	exonic	SBSN	28	27	0.4909	Missense_Mutation	p.G423V	Non-Targeted Panel Gene	Adult HGBCL, NOS
BCL-15	chr17	43092841	43092841	G	A	exonic	BRCA1	16	16	0.5	Missense_Mutation	p.P897L	Non-Targeted Panel Gene	Adult HGBCL, NOS
BCL-15	chr19	48196606	48196606	C	A	exonic	C19orf68	10	10	0.5	Missense_Mutation	p.H848N	Non-Targeted Panel Gene	Adult HGBCL, NOS
BCL-15	chr10	73154363	73154363	T	C	exonic	ECD	23	23	0.5	Missense_Mutation	p.R226G	Non-Targeted Panel Gene	Adult HGBCL, NOS
BCL-15	chr6	75633608	75633608	A	G	exonic	SENP6	17	17	0.5	Missense_Mutation	p.N79D	Non-Targeted Panel Gene	Adult HGBCL, NOS
BCL-15	chr15	61983924	61983924	T	C	exonic	VPS13C	20	20	0.5	Missense_Mutation	p.T604A	Non-Targeted Panel Gene	Adult HGBCL, NOS
BCL-15	chr10	104168772	104168772	G	C	exonic	CFAP43	29	31	0.5167	Missense_Mutation	p.S888W	Non-Targeted Panel Gene	Adult HGBCL, NOS
BCL-15	chr1	34904398	34904398	C	T	exonic	DLGAP3	11	12	0.5217	Missense_Mutation	p.R329Q	Non-Targeted Panel Gene	Adult HGBCL, NOS
BCL-15	chr5	81123660	81123660	C	T	exonic	RASGRF2	21	23	0.5227	Missense_Mutation	p.P830L	Non-Targeted Panel Gene	Adult HGBCL, NOS
BCL-15	chr22	37723371	37723371	C	A	exonic	TRIOBP	21	24	0.5333	Missense_Mutation	p.T272K	Non-Targeted Panel Gene	Adult HGBCL, NOS
BCL-15	chr4	51886616	51886616	A	G	exonic	DCUN1D4	26	31	0.5439	Missense_Mutation	p.D71G	Non-Targeted Panel Gene	Adult HGBCL, NOS
BCL-15	chr17	76088896	76088896	C	A	exonic	EXOC7	5	6	0.5455	Missense_Mutation	p.G382W	Non-Targeted Panel Gene	Adult HGBCL, NOS
BCL-15	chr14	74357856	74357856	G	A	exonic	VRTN	9	11	0.55	Missense_Mutation	p.G358D	Non-Targeted Panel Gene	Adult HGBCL, NOS
BCL-15	chr6	54940343	54940343	G	A	exonic	FAM83B	29	37	0.5606	Missense_Mutation	p.D458N	Non-Targeted Panel Gene	Adult HGBCL, NOS
BCL-15	chr1	1923014	1923014	T	C	exonic	CFAP74	7	9	0.5625	Missense_Mutation	p.I1552V	Non-Targeted Panel Gene	Adult HGBCL, NOS
BCL-15	chr9	37441933	37441933	G	C	exonic	ZBTB5	10	13	0.5652	Missense_Mutation	p.P207A	Non-Targeted Panel Gene	Adult HGBCL, NOS
BCL-15	chr11	17533291	17533291	T	C	exonic	USH1C	12	16	0.5714	Missense_Mutation	p.K23R	Non-Targeted Panel Gene	Adult HGBCL, NOS
BCL-15	chr7	41967848	41967848	C	T	exonic	GLI3	16	22	0.5789	Missense_Mutation	p.G727R	Non-Targeted Panel Gene	Adult HGBCL, NOS
BCL-15	chr2	178561093	178561093	T	C	exonic	TTN	20	28	0.5833	Missense_Mutation	p.I28347V	Non-Targeted Panel Gene	Adult HGBCL, NOS
BCL-15	chr3	150678417	150678417	A	G	exonic	ERICH6	17	24	0.5854	Missense_Mutation	p.C417R	Non-Targeted Panel Gene	Adult HGBCL, NOS
BCL-15	chr18	12337411	12337411	C	T	exonic	AFG3L2	62	91	0.5948	Missense_Mutation	p.R702Q	Non-Targeted Panel Gene	Adult HGBCL, NOS
BCL-15	chr19	38802356	38802356	T	G	exonic	LGALS4	8	12	0.6	Missense_Mutation	p.T207P	Non-Targeted Panel Gene	Adult HGBCL, NOS
BCL-15	chr21	46416699	46416699	G	C	exonic	PCNT	9	14	0.6087	Missense_Mutation	p.G2261R	Non-Targeted Panel Gene	Adult HGBCL, NOS
BCL-15	chr7	56019663	56019663	G	A	exonic	PSPH	7	11	0.6111	Missense_Mutation	p.P71L	Non-Targeted Panel Gene	Adult HGBCL, NOS
BCL-15	chr15	54235044	54235044	T	C	exonic	UNC13C	17	27	0.6136	Missense_Mutation	p.L1029P	Non-Targeted Panel Gene	Adult HGBCL, NOS
BCL-15	chr5	83541332	83541332	A	G	exonic	VCAN	11	18	0.6207	Missense_Mutation	p.T2777A	Non-Targeted Panel Gene	Adult HGBCL, NOS
BCL-15	chr19	18435391	18435391	C	A	exonic	ISYNA1	4	7	0.6364	Missense_Mutation	p.E449D	Non-Targeted Panel Gene	Adult HGBCL, NOS
BCL-15	chr4	165341906	165341906	A	G	exonic	MSMO1	8	14	0.6364	Missense_Mutation	p.Y281C	Non-Targeted Panel Gene	Adult HGBCL, NOS
BCL-15	chr17	61685878	61685878	T	G	exonic	BRIP1	16	29	0.6444	Missense_Mutation	p.N955H	Non-Targeted Panel Gene	Adult HGBCL, NOS
BCL-15	chr8	11555420	11555420	C	G	exonic	BLK	22	41	0.6508	Missense_Mutation	p.I236M	Non-Targeted Panel Gene	Adult HGBCL, NOS
BCL-15	chr20	33374197	33374197	G	C	exonic	CDK5RAP1	13	28	0.6829	Missense_Mutation	p.H375D	Non-Targeted Panel Gene	Adult HGBCL, NOS
BCL-15	chr11	7960555	7960555	C	T	exonic	NLRP10	16	35	0.6863	Missense_Mutation	p.V353I	Non-Targeted Panel Gene	Adult HGBCL, NOS
BCL-15	chr14	73739791	73739791	A	G	exonic	ELMSAN1	5	12	0.7059	Missense_Mutation	p.L73P	Non-Targeted Panel Gene	Adult HGBCL, NOS
BCL-15	chr1	170544804	170544804	G	T	exonic	GORAB	15	36	0.7059	Missense_Mutation	p.R52S	Non-Targeted Panel Gene	Adult HGBCL, NOS
BCL-15	chrX	124392152	124392152	C	T	exonic	TENM1	0	35	1	Missense_Mutation	p.R1862K	Non-Targeted Panel Gene	Adult HGBCL, NOS
BCL-15	chr8	39788091	39788091	A	C	exonic	ADAM2	43	7	0.14	Nonsense_Mutation	p.L268X	Targerted Panel Gene	Adult HGBCL, NOS
BCL-15	chr2	27217943	27217943	T	A	exonic	CAD	84	23	0.215	Nonsense_Mutation	p.L50X	Targerted Panel Gene	Adult HGBCL, NOS
BCL-15	chr1	150816372	150816372	G	A	exonic	ARNT	53	2	0.0364	Nonsense_Mutation	p.Q604X	Non-Targeted Panel Gene	Adult HGBCL, NOS
BCL-15	chr13	66304821	66304821	C	T	exonic	PCDH9	52	2	0.037	Nonsense_Mutation	p.W1183X	Non-Targeted Panel Gene	Adult HGBCL, NOS
BCL-15	chr4	109828056	109828056	C	A	exonic	RRH	51	2	0.0377	Nonsense_Mutation	p.S10X	Non-Targeted Panel Gene	Adult HGBCL, NOS
BCL-15	chr5	45695787	45695787	G	A	exonic	HCN1	48	2	0.04	Nonsense_Mutation	p.Q103X	Non-Targeted Panel Gene	Adult HGBCL, NOS
BCL-15	chr3	125594645	125594645	C	T	exonic	OSBPL11	43	2	0.0444	Nonsense_Mutation	p.W52X	Non-Targeted Panel Gene	Adult HGBCL, NOS
BCL-15	chr11	62296417	62296417	C	T	exonic	SCGB1D4	43	2	0.0444	Nonsense_Mutation	p.W82X	Non-Targeted Panel Gene	Adult HGBCL, NOS
BCL-15	chr12	117330469	117330469	C	A	exonic	NOS1	42	2	0.0455	Nonsense_Mutation	p.E201X	Non-Targeted Panel Gene	Adult HGBCL, NOS
BCL-15	chr2	232790763	232790763	C	T	exonic	GIGYF2	47	3	0.06	Nonsense_Mutation	p.R260X	Non-Targeted Panel Gene	Adult HGBCL, NOS
BCL-15	chr17	75563132	75563132	G	A	exonic	LLGL2	31	2	0.0606	Nonsense_Mutation	p.W216X	Non-Targeted Panel Gene	Adult HGBCL, NOS
BCL-15	chr4	2250666	2250666	C	A	exonic	MXD4	28	2	0.0645	Nonsense_Mutation	p.E170X	Non-Targeted Panel Gene	Adult HGBCL, NOS
BCL-15	chr16	69351657	69351657	G	A	exonic	TMED6	27	2	0.0667	Nonsense_Mutation	p.Q33X	Non-Targeted Panel Gene	Adult HGBCL, NOS
BCL-15	chr7	73683240	73683240	G	A	exonic	DNAJC30	39	3	0.0714	Nonsense_Mutation	p.Q62X	Non-Targeted Panel Gene	Adult HGBCL, NOS
BCL-15	chr10	93669847	93669847	A	T	exonic	FRA10AC1	26	2	0.0714	Nonsense_Mutation	p.Y309X	Non-Targeted Panel Gene	Adult HGBCL, NOS
BCL-15	chr2	25742427	25742427	G	A	exonic	ASXL2	23	2	0.08	Nonsense_Mutation	p.Q1304X	Non-Targeted Panel Gene	Adult HGBCL, NOS
BCL-15	chr15	40853766	40853766	G	A	exonic	SPINT1	29	3	0.0909	Nonsense_Mutation	p.W266X	Non-Targeted Panel Gene	Adult HGBCL, NOS
BCL-15	chr9	77815685	77815685	C	T	exonic	GNAQ	18	2	0.1	Nonsense_Mutation	p.W136X	Non-Targeted Panel Gene	Adult HGBCL, NOS
BCL-15	chr11	66295290	66295290	G	A	exonic	TMEM151A	17	2	0.1053	Nonsense_Mutation	p.W348X	Non-Targeted Panel Gene	Adult HGBCL, NOS
BCL-15	chr14	24332892	24332892	G	A	exonic	ADCY4	13	2	0.1333	Nonsense_Mutation	p.Q86X	Non-Targeted Panel Gene	Adult HGBCL, NOS
BCL-15	chr19	17808309	17808309	C	T	exonic	B3GNT3	12	2	0.1429	Nonsense_Mutation	p.Q168X	Non-Targeted Panel Gene	Adult HGBCL, NOS
BCL-15	chr19	1787105	1787105	G	A	exonic	ATP8B3	11	2	0.1538	Nonsense_Mutation	p.Q1051X	Non-Targeted Panel Gene	Adult HGBCL, NOS
BCL-15	chr7	54757510	54757510	T	A	exonic	SEC61G	95	18	0.1593	Nonsense_Mutation	p.K27X	Non-Targeted Panel Gene	Adult HGBCL, NOS
BCL-15	chr11	63764915	63764915	C	T	exonic	C11orf95	8	2	0.2	Nonsense_Mutation	p.W326X	Non-Targeted Panel Gene	Adult HGBCL, NOS
BCL-15	chr6	85637139	85637139	-	ATATCT	exonic	SYNCRIP	9	5	0.3571	Nonsense_Mutation	p.F165delinsX	Non-Targeted Panel Gene	Adult HGBCL, NOS
BCL-15	chrX	48910260	48910260	A	C	exonic	SLC35A2	24	16	0.4	Nonsense_Mutation	p.L50X	Non-Targeted Panel Gene	Adult HGBCL, NOS
BCL-15	chr2	189741436	189741436	C	G	exonic	ANKAR	8	8	0.5	Nonsense_Mutation	p.Y1265X	Non-Targeted Panel Gene	Adult HGBCL, NOS
BCL-15	chr1	23519112	23519112	G	T	exonic	E2F2	25	27	0.5192	Nonsense_Mutation	p.Y252X	Non-Targeted Panel Gene	Adult HGBCL, NOS
BCL-16	chr7	142172196	142172196	T	C	splicing	MGAM2	173	21	0.1082	Splice_Site	.	Non-Targeted Panel Gene	Adult HGBCL, NOS
BCL-16	chr17	7674216	7674219	CCTC	-	exonic	TP53	241	30	0.1103	Frame_Shift_Del	p.R209fs	Targerted Panel Gene	Adult HGBCL, NOS
BCL-16	chr11	121160349	121160349	C	-	exonic	TECTA	63	26	0.2921	Frame_Shift_Del	p.T1635fs	Non-Targeted Panel Gene	Adult HGBCL, NOS
BCL-16	chr14	24137401	24137402	AG	-	exonic	PSME1	109	76	0.4086	Frame_Shift_Del	p.K72fs	Non-Targeted Panel Gene	Adult HGBCL, NOS
BCL-16	chr13	113653373	113653373	A	-	exonic	ATP4B	64	47	0.4234	Frame_Shift_Del	p.S101fs	Non-Targeted Panel Gene	Adult HGBCL, NOS
BCL-16	chr17	7674213	7674213	-	GTGA	exonic	TP53	243	28	0.1033	Frame_Shift_Ins	p.P211fs	Targerted Panel Gene	Adult HGBCL, NOS
BCL-16	chr22	23181607	23181607	-	CCAC	exonic	BCR	181	110	0.378	Frame_Shift_Ins	p.Q216fs	Targerted Panel Gene	Adult HGBCL, NOS
BCL-16	chr7	101038758	101038758	-	C	exonic	MUC17	96	79	0.4413	Frame_Shift_Ins	p.T2448fs	Non-Targeted Panel Gene	Adult HGBCL, NOS
BCL-16	chrX	48773374	48773374	-	CA	exonic	GLOD5	2	42	0.9545	Frame_Shift_Ins	p.S141fs	Non-Targeted Panel Gene	Adult HGBCL, NOS
BCL-16	chrX	67545317	67545340	GCAGCAGCAGCAG	-	exonic	AR	17	6	0.25	In_Frame_Del	p.57_65del	Non-Targeted Panel Gene	Adult HGBCL, NOS
BCL-16	chr19	50382119	50382142	TGCAGCAGCCCTA	-	exonic	NR1H2	24	11	0.3143	In_Frame_Del	p.394_402del	Non-Targeted Panel Gene	Adult HGBCL, NOS
BCL-16	chr7	23200679	23200690	ATCCAAGAAATT	-	exonic	NUPL2	53	30	0.3614	In_Frame_Del	p.402_406del	Non-Targeted Panel Gene	Adult HGBCL, NOS

BCL-16	chr5	35654692	35654694	TAA	-	exonic	SPEF2	70	47	0.4017	In_Frame_Del	p.315_316del	Non-Targeted Panel Gene	Adult HGBCL, NOS
BCL-16	chr5	156757715	156757717	AGA	-	exonic	SGCD	37	26	0.4127	In_Frame_Del	p.237_238del	Non-Targeted Panel Gene	Adult HGBCL, NOS
BCL-16	chr8	127738450	127738450	C	T	exonic	MYC	179	8	0.0428	Missense_Mutation	p.P78L	Targerted Panel Gene	Adult HGBCL, NOS
BCL-16	chr10	52133863	52133863	T	G	exonic	PRKG1	62	3	0.0462	Missense_Mutation	p.V320G	Targerted Panel Gene	Adult HGBCL, NOS
BCL-16	chr17	7674220	7674220	C	A	exonic	TP53	244	30	0.1095	Missense_Mutation	p.R209L	Targerted Panel Gene	Adult HGBCL, NOS
BCL-16	chr6	26123577	26123577	G	T	exonic	HIST1H2BC	135	32	0.1916	Missense_Mutation	p.H110N	Targerted Panel Gene	Adult HGBCL, NOS
BCL-16	chr17	7673778	7673778	T	C	exonic	TP53	55	15	0.2143	Missense_Mutation	p.D242G	Targerted Panel Gene	Adult HGBCL, NOS
BCL-16	chr8	112244408	112244408	A	C	exonic	CSMD3	73	20	0.2151	Missense_Mutation	p.V3423G	Targerted Panel Gene	Adult HGBCL, NOS
BCL-16	chr4	93455885	93455885	T	G	exonic	GRID2	87	42	0.3256	Missense_Mutation	p.L590R	Targerted Panel Gene	Adult HGBCL, NOS
BCL-16	chr13	40666019	40666019	G	C	exonic	FOXO1	21	13	0.3824	Missense_Mutation	p.A65G	Targerted Panel Gene	Adult HGBCL, NOS
BCL-16	chr19	1612350	1612350	A	G	exonic	TCF3	58	38	0.3958	Missense_Mutation	p.V557A	Targerted Panel Gene	Adult HGBCL, NOS
BCL-16	chr17	65014564	65014564	A	C	exonic	GNA13	59	45	0.4327	Missense_Mutation	p.V276G	Targerted Panel Gene	Adult HGBCL, NOS
BCL-16	chr19	38543804	38543804	C	T	exonic	RYR1	54	42	0.4375	Missense_Mutation	p.H3981Y	Targerted Panel Gene	Adult HGBCL, NOS
BCL-16	chr8	109430014	109430014	T	G	exonic	PKHD1L1	54	44	0.449	Missense_Mutation	p.V1069G	Targerted Panel Gene	Adult HGBCL, NOS
BCL-16	chr1	236806198	236806198	A	G	exonic	MTR	44	36	0.45	Missense_Mutation	p.S102G	Targerted Panel Gene	Adult HGBCL, NOS
BCL-16	chr11	111379174	111379174	G	A	exonic	POU2AF1	96	79	0.4514	Missense_Mutation	p.L2F	Targerted Panel Gene	Adult HGBCL, NOS
BCL-16	chr13	40666152	40666152	G	C	exonic	FOXO1	15	13	0.4643	Missense_Mutation	p.R21G	Targerted Panel Gene	Adult HGBCL, NOS
BCL-16	chr17	65018275	65018275	T	G	exonic	GNA13	10	9	0.4737	Missense_Mutation	p.N180T	Targerted Panel Gene	Adult HGBCL, NOS
BCL-16	chr17	65014505	65014505	G	C	exonic	GNA13	59	56	0.487	Missense_Mutation	p.L296V	Targerted Panel Gene	Adult HGBCL, NOS
BCL-16	chr8	127736612	127736612	G	A	exonic	MYC	14	18	0.5625	Missense_Mutation	p.V7M	Targerted Panel Gene	Adult HGBCL, NOS
BCL-16	chr17	7674250	7674250	C	T	exonic	TP53	103	165	0.6157	Missense_Mutation	p.C199Y	Targerted Panel Gene	Adult HGBCL, NOS
BCL-16	chr6	28572212	28572212	T	G	exonic	ZBED9	61	3	0.0469	Missense_Mutation	p.K1075T	Non-Targeted Panel Gene	Adult HGBCL, NOS
BCL-16	chr11	12925046	12925046	A	C	exonic	TEAD1	80	4	0.0476	Missense_Mutation	p.K336N	Non-Targeted Panel Gene	Adult HGBCL, NOS
BCL-16	chr2	189006361	189006361	A	G	exonic	COL3A1	109	11	0.0917	Missense_Mutation	p.N1037S	Non-Targeted Panel Gene	Adult HGBCL, NOS
BCL-16	chr7	122039560	122039560	T	G	exonic	PTPRZ1	106	21	0.1654	Missense_Mutation	p.F1870C	Non-Targeted Panel Gene	Adult HGBCL, NOS
BCL-16	chr8	22194466	22194466	A	G	exonic	BMP1	144	50	0.2577	Missense_Mutation	p.K440R	Non-Targeted Panel Gene	Adult HGBCL, NOS
BCL-16	chr3	64594407	64594407	T	C	exonic	ADAMTS9	103	37	0.2643	Missense_Mutation	p.R1403G	Non-Targeted Panel Gene	Adult HGBCL, NOS
BCL-16	chr14	33774434	33774434	C	G	exonic	NPAS3	76	28	0.2692	Missense_Mutation	p.A304G	Non-Targeted Panel Gene	Adult HGBCL, NOS
BCL-16	chr14	33774436	33774436	T	G	exonic	NPAS3	75	28	0.2718	Missense_Mutation	p.L305V	Non-Targeted Panel Gene	Adult HGBCL, NOS
BCL-16	chr10	116674986	116674986	T	C	exonic	HSPA12A	176	74	0.296	Missense_Mutation	p.D625G	Non-Targeted Panel Gene	Adult HGBCL, NOS
BCL-16	chr5	40854251	40854251	A	C	exonic	CARD6	79	36	0.313	Missense_Mutation	p.K973N	Non-Targeted Panel Gene	Adult HGBCL, NOS
BCL-16	chr16	20792092	20792092	T	G	exonic	ACSM3	63	30	0.3226	Missense_Mutation	p.F473V	Non-Targeted Panel Gene	Adult HGBCL, NOS
BCL-16	chr2	229025714	229025714	T	C	exonic	PID1	156	75	0.3247	Missense_Mutation	p.H222R	Non-Targeted Panel Gene	Adult HGBCL, NOS
BCL-16	chr6	111349255	111349255	C	T	exonic	REV3L	22	12	0.3529	Missense_Mutation	p.R2383Q	Non-Targeted Panel Gene	Adult HGBCL, NOS
BCL-16	chr6	152140027	152140027	C	T	exonic	SYNE1	29	16	0.3556	Missense_Mutation	p.E8461K	Non-Targeted Panel Gene	Adult HGBCL, NOS
BCL-16	chr12	9152270	9152270	C	G	exonic	PZP	29	17	0.3696	Missense_Mutation	p.D1388H	Non-Targeted Panel Gene	Adult HGBCL, NOS
BCL-16	chr7	64521271	64521271	A	C	exonic	ZNF680	20	12	0.375	Missense_Mutation	p.C495G	Non-Targeted Panel Gene	Adult HGBCL, NOS
BCL-16	chr14	47131746	47131746	T	G	exonic	MDGA2	19	12	0.3871	Missense_Mutation	p.K229T	Non-Targeted Panel Gene	Adult HGBCL, NOS
BCL-16	chr3	142459276	142459276	G	C	exonic	ATR	64	41	0.3905	Missense_Mutation	p.P2434A	Non-Targeted Panel Gene	Adult HGBCL, NOS
BCL-16	chr2	178549089	178549089	A	G	exonic	TTN	84	54	0.3913	Missense_Mutation	p.V30846A	Non-Targeted Panel Gene	Adult HGBCL, NOS
BCL-16	chr2	159169304	159169304	C	G	exonic	TANC1	106	69	0.3943	Missense_Mutation	p.A334G	Non-Targeted Panel Gene	Adult HGBCL, NOS
BCL-16	chr3	184342837	184342837	A	G	exonic	FAM131A	121	81	0.401	Missense_Mutation	p.D201G	Non-Targeted Panel Gene	Adult HGBCL, NOS
BCL-16	chr19	58262076	58262076	A	C	exonic	ZNF544	60	41	0.4059	Missense_Mutation	p.K490N	Non-Targeted Panel Gene	Adult HGBCL, NOS
BCL-16	chr3	119447030	119447030	A	G	exonic	TMEM39A	64	44	0.4074	Missense_Mutation	p.F188S	Non-Targeted Panel Gene	Adult HGBCL, NOS
BCL-16	chr4	1806842	1806842	C	T	exonic	FGFR3	29	20	0.4082	Missense_Mutation	p.R730W	Non-Targeted Panel Gene	Adult HGBCL, NOS
BCL-16	chr14	91313612	91313612	C	T	exonic	CCDC88C	43	30	0.411	Missense_Mutation	p.R735H	Non-Targeted Panel Gene	Adult HGBCL, NOS
BCL-16	chr9	132329641	132329641	G	T	exonic	SETX	34	24	0.4138	Missense_Mutation	p.Q653K	Non-Targeted Panel Gene	Adult HGBCL, NOS
BCL-16	chr15	23645096	23645096	C	T	exonic	MAGEL2	74	53	0.4173	Missense_Mutation	p.G883S	Non-Targeted Panel Gene	Adult HGBCL, NOS
BCL-16	chr19	54218377	54218377	A	G	exonic	LILRB3	76	56	0.4242	Missense_Mutation	p.V543A	Non-Targeted Panel Gene	Adult HGBCL, NOS
BCL-16	chr12	63802131	63802131	G	A	exonic	TMEM5	37	28	0.4308	Missense_Mutation	p.V157I	Non-Targeted Panel Gene	Adult HGBCL, NOS
BCL-16	chr16	55691975	55691975	G	A	exonic	SLC6A2	85	65	0.4333	Missense_Mutation	p.G281S	Non-Targeted Panel Gene	Adult HGBCL, NOS
BCL-16	chr11	94544737	94544737	G	A	exonic	FUT4	30	23	0.434	Missense_Mutation	p.D202N	Non-Targeted Panel Gene	Adult HGBCL, NOS
BCL-16	chr11	36574361	36574361	T	G	exonic	RAG1	115	89	0.4363	Missense_Mutation	p.S353A	Non-Targeted Panel Gene	Adult HGBCL, NOS
BCL-16	chr5	19503056	19503056	C	A	exonic	CDH18	23	18	0.439	Missense_Mutation	p.R522S	Non-Targeted Panel Gene	Adult HGBCL, NOS
BCL-16	chr4	55110542	55110542	C	G	exonic	KDR	34	27	0.4426	Missense_Mutation	p.E372D	Non-Targeted Panel Gene	Adult HGBCL, NOS
BCL-16	chr3	130571259	130571259	T	G	exonic	COL6A6	99	79	0.4438	Missense_Mutation	p.I948S	Non-Targeted Panel Gene	Adult HGBCL, NOS
BCL-16	chr3	171856689	171856689	C	A	exonic	TMEM212	17	14	0.4516	Missense_Mutation	p.N190K	Non-Targeted Panel Gene	Adult HGBCL, NOS
BCL-16	chr9	132329791	132329791	T	C	exonic	SETX	53	44	0.4536	Missense_Mutation	p.N603D	Non-Targeted Panel Gene	Adult HGBCL, NOS
BCL-16	chr12	6453269	6453269	T	G	exonic	TAPBPL	74	62	0.4559	Missense_Mutation	p.D89E	Non-Targeted Panel Gene	Adult HGBCL, NOS
BCL-16	chr8	10613245	10613245	C	G	exonic	RP1L1	102	86	0.4574	Missense_Mutation	p.V285L	Non-Targeted Panel Gene	Adult HGBCL, NOS
BCL-16	chr1	20671998	20671998	C	T	exonic	KIF17	83	70	0.4575	Missense_Mutation	p.E888K	Non-Targeted Panel Gene	Adult HGBCL, NOS
BCL-16	chr3	132453503	132453503	C	T	exonic	DNAJC13	39	33	0.4583	Missense_Mutation	p.S248L	Non-Targeted Panel Gene	Adult HGBCL, NOS
BCL-16	chr16	67886075	67886075	C	A	exonic	NRN1L	80	69	0.4631	Missense_Mutation	p.A105D	Non-Targeted Panel Gene	Adult HGBCL, NOS
BCL-16	chr15	48738638	48738638	A	G	exonic	CEP152	51	44	0.4632	Missense_Mutation	p.S1582P	Non-Targeted Panel Gene	Adult HGBCL, NOS
BCL-16	chr2	43846221	43846221	C	A	exonic	ABCG8	89	78	0.4671	Missense_Mutation	p.P78T	Non-Targeted Panel Gene	Adult HGBCL, NOS
BCL-16	chr8	31091835	31091835	C	G	exonic	WRN	41	36	0.4675	Missense_Mutation	p.S612C	Non-Targeted Panel Gene	Adult HGBCL, NOS
BCL-16	chr17	44375704	44375704	G	T	exonic	ITGA2B	77	68	0.469	Missense_Mutation	p.L872M	Non-Targeted Panel Gene	Adult HGBCL, NOS
BCL-16	chr19	57356773	57356773	A	G	exonic	ZNF304	60	53	0.469	Missense_Mutation	p.T210A	Non-Targeted Panel Gene	Adult HGBCL, NOS
BCL-16	chr13	48001233	48001233	C	T	exonic	SUCLA2	35	31	0.4697	Missense_Mutation	p.V13M	Non-Targeted Panel Gene	Adult HGBCL, NOS
BCL-16	chr3	66382333	66382333	G	C	exonic	LRIG1	65	58	0.4715	Missense_Mutation	p.Q853E	Non-Targeted Panel Gene	Adult HGBCL, NOS
BCL-16	chr11	62678032	62678032	C	G	exonic	UBXN1	19	17	0.4722	Missense_Mutation	p.R126T	Non-Targeted Panel Gene	Adult HGBCL, NOS
BCL-16	chr12	70310971	70310971	T	C	exonic	CNOT2	74	67	0.4752	Missense_Mutation	p.M42T	Non-Targeted Panel Gene	Adult HGBCL, NOS
BCL-16	chr15	89683702	89683702	T	C	exonic	PEX11A	106	97	0.4778	Missense_Mutation	p.Y140C	Non-Targeted Panel Gene	Adult HGBCL, NOS
BCL-16	chr11	60847952	60847952	C	G	exonic	CCDC86	38	35	0.4795	Missense_Mutation	p.L263V	Non-Targeted Panel Gene	Adult HGBCL, NOS
BCL-16	chr14	63983761	63983761	A	G	exonic	SYNE2	38	35	0.4795	Missense_Mutation	p.K676E	Non-Targeted Panel Gene	Adult HGBCL, NOS
BCL-16	chr6	26240712	26240712	G	A	exonic	HIST1H4F	137	127	0.4811	Missense_Mutation	p.R96H	Non-Targeted Panel Gene	Adult HGBCL, NOS
BCL-16	chr11	119298451	119298451	C	T	exonic	CBL	102	95	0.4822	Missense_Mutation	p.P782L	Non-Targeted Panel Gene	Adult HGBCL, NOS
BCL-16	chr3	47124079	47124079	G	A	exonic	SETD2	90	85	0.4857	Missense_Mutation	p.P186L	Non-Targeted Panel Gene	Adult HGBCL, NOS
BCL-16	chr5	134576771	134576771	G	A	exonic	JADE2	75	71	0.4863	Missense_Mutation	p.R519Q	Non-Targeted Panel Gene	Adult HGBCL, NOS
BCL-16	chr12	40939357	40939357	G	T	exonic	CNTN1	58	55	0.4867	Missense_Mutation	p.M417I	Non-Targeted Panel Gene	Adult HGBCL, NOS
BCL-16	chr19	2834107	2834107	A	C	exonic	ZNF554	115	110	0.4889	Missense_Mutation	p.H291P	Non-Targeted Panel Gene	Adult HGBCL, NOS
BCL-16	chr15	50997734	50997734	A	G	exonic	AP4E1	33	33	0.5	Missense_Mutation	p.M919V	Non-Targeted Panel Gene	Adult HGBCL, NOS

BCL-16	chr3	134549149	134549149	C	A	exonic	CEP63	11	11	0.5	Missense_Mutation	p.N385K	Non-Targeted Panel Gene	Adult HGBCL, NOS
BCL-16	chr13	20189571	20189571	C	T	exonic	GJB2	38	38	0.5	Missense_Mutation	p.G4D	Non-Targeted Panel Gene	Adult HGBCL, NOS
BCL-16	chr10	102413944	102413944	G	A	exonic	PSD	50	51	0.5	Missense_Mutation	p.P81S	Non-Targeted Panel Gene	Adult HGBCL, NOS
BCL-16	chr3	47701316	47701316	G	A	exonic	SMARCC1	42	42	0.5	Missense_Mutation	p.P376L	Non-Targeted Panel Gene	Adult HGBCL, NOS
BCL-16	chr14	64022021	64022021	A	T	exonic	SYNE2	10	10	0.5	Missense_Mutation	p.L1839F	Non-Targeted Panel Gene	Adult HGBCL, NOS
BCL-16	chr19	57291422	57291422	A	G	exonic	ZNF460	133	136	0.5056	Missense_Mutation	p.N294S	Non-Targeted Panel Gene	Adult HGBCL, NOS
BCL-16	chr13	102737136	102737136	T	C	exonic	CCDC168	33	34	0.5075	Missense_Mutation	p.I4521V	Non-Targeted Panel Gene	Adult HGBCL, NOS
BCL-16	chr1	225862833	225862833	A	C	exonic	TMEM63A	105	111	0.5139	Missense_Mutation	p.C255W	Non-Targeted Panel Gene	Adult HGBCL, NOS
BCL-16	chr9	21385272	21385272	A	G	exonic	IFNA2	136	146	0.5177	Missense_Mutation	p.C20R	Non-Targeted Panel Gene	Adult HGBCL, NOS
BCL-16	chr4	113353362	113353362	C	T	exonic	ANK2	52	56	0.5185	Missense_Mutation	p.R1582W	Non-Targeted Panel Gene	Adult HGBCL, NOS
BCL-16	chr5	141957842	141957842	G	A	exonic	PCDH12	45	49	0.5213	Missense_Mutation	p.L4F	Non-Targeted Panel Gene	Adult HGBCL, NOS
BCL-16	chr16	30026791	30026791	C	T	exonic	FAM57B	141	155	0.5236	Missense_Mutation	p.D88N	Non-Targeted Panel Gene	Adult HGBCL, NOS
BCL-16	chr1	203217835	203217835	C	T	exonic	CHIT1	40	44	0.5238	Missense_Mutation	p.G354R	Non-Targeted Panel Gene	Adult HGBCL, NOS
BCL-16	chr11	60873190	60873190	A	G	exonic	ZP1	60	70	0.5385	Missense_Mutation	p.S381G	Non-Targeted Panel Gene	Adult HGBCL, NOS
BCL-16	chr3	142512277	142512277	T	C	exonic	ATR	41	49	0.5444	Missense_Mutation	p.N1612S	Non-Targeted Panel Gene	Adult HGBCL, NOS
BCL-16	chr7	16421291	16421291	G	C	exonic	ISPD	9	11	0.55	Missense_Mutation	p.P11R	Non-Targeted Panel Gene	Adult HGBCL, NOS
BCL-16	chr12	18701711	18701711	T	A	exonic	PLCZ1	13	16	0.5517	Missense_Mutation	p.R310S	Non-Targeted Panel Gene	Adult HGBCL, NOS
BCL-16	chr4	183319947	183319947	C	T	exonic	CLDN22	76	94	0.5529	Missense_Mutation	p.G91E	Non-Targeted Panel Gene	Adult HGBCL, NOS
BCL-16	chr19	50286528	50286528	G	A	exonic	MYH14	32	40	0.5556	Missense_Mutation	p.R1529H	Non-Targeted Panel Gene	Adult HGBCL, NOS
BCL-16	chr7	78160034	78160034	A	T	exonic	MAGI2	63	79	0.5563	Missense_Mutation	p.S946T	Non-Targeted Panel Gene	Adult HGBCL, NOS
BCL-16	chr11	66495413	66495413	A	G	exonic	DPP3	47	59	0.5566	Missense_Mutation	p.T501A	Non-Targeted Panel Gene	Adult HGBCL, NOS
BCL-16	chr12	57796359	57796359	G	A	exonic	TSFM	74	93	0.5569	Missense_Mutation	p.V273I	Non-Targeted Panel Gene	Adult HGBCL, NOS
BCL-16	chr15	50948126	50948126	A	G	exonic	AP4E1	33	42	0.56	Missense_Mutation	p.N428S	Non-Targeted Panel Gene	Adult HGBCL, NOS
BCL-16	chr16	30085048	30085048	G	A	exonic	PPP4C	39	51	0.5667	Missense_Mutation	p.D304N	Non-Targeted Panel Gene	Adult HGBCL, NOS
BCL-16	chr21	45984315	45984315	C	T	exonic	COL6A1	60	81	0.5745	Missense_Mutation	p.H92Y	Non-Targeted Panel Gene	Adult HGBCL, NOS
BCL-16	chr22	41254445	41254445	C	T	exonic	RANGAP1	29	44	0.6027	Missense_Mutation	p.E375K	Non-Targeted Panel Gene	Adult HGBCL, NOS
BCL-16	chr5	178994593	178994593	G	T	exonic	GRM6	5	9	0.6429	Missense_Mutation	p.R118S	Non-Targeted Panel Gene	Adult HGBCL, NOS
BCL-16	chr8	10608739	10608739	A	C	exonic	RP1L1	12	22	0.6471	Missense_Mutation	p.L1787V	Non-Targeted Panel Gene	Adult HGBCL, NOS
BCL-16	chrX	53985746	53985746	T	C	exonic	PHF8	6	47	0.8868	Missense_Mutation	p.S693G	Non-Targeted Panel Gene	Adult HGBCL, NOS
BCL-16	chr17	65018272	65018272	A	T	exonic	GNA13	10	8	0.4444	Nonsense_Mutation	p.L181X	Targerted Panel Gene	Adult HGBCL, NOS
BCL-16	chrX	41339060	41339060	T	G	exonic	DDX3X	6	54	0.9	Nonsense_Mutation	p.L43X	Targerted Panel Gene	Adult HGBCL, NOS
BCL-16	chr5	66013613	66013613	G	T	exonic	ERBIN	80	11	0.1209	Nonsense_Mutation	p.E151X	Non-Targeted Panel Gene	Adult HGBCL, NOS
BCL-16	chr19	12527882	12527882	C	A	exonic	ZNF564	43	37	0.4625	Nonsense_Mutation	p.E76X	Non-Targeted Panel Gene	Adult HGBCL, NOS
BCL-16	chr11	116790781	116790781	G	A	exonic	APOA5	49	46	0.4842	Nonsense_Mutation	p.Q150X	Non-Targeted Panel Gene	Adult HGBCL, NOS
BCL-16	chr17	41731703	41731703	G	A	exonic	HAP1	31	31	0.5	Nonsense_Mutation	p.Q313X	Non-Targeted Panel Gene	Adult HGBCL, NOS
BCL-20	chr2	205745891	205745891	G	A	splicing	NRP2	87	3	0.0333	Splice_Site	.	Non-Targeted Panel Gene	Adult HGBCL, NOS
BCL-20	chr17	10401274	10401274	C	T	splicing	MYH8	9	2	0.1818	Splice_Site	.	Non-Targeted Panel Gene	Adult HGBCL, NOS
BCL-20	chrX	119620053	119620058	TGAAAA	-	splicing	SEPTIN1	42	12	0.2222	Splice_Site	.	Non-Targeted Panel Gene	Adult HGBCL, NOS
BCL-20	chr14	81220488	81220488	C	G	splicing	GTF2A1	9	4	0.3077	Splice_Site	.	Non-Targeted Panel Gene	Adult HGBCL, NOS
BCL-20	chr3	180609707	180609707	C	-	exonic	TTC14	25	21	0.4565	Frame_Shift_Del	p.S493fs	Non-Targeted Panel Gene	Adult HGBCL, NOS
BCL-20	chr20	43726487	43726487	C	-	exonic	GTSF1L	69	76	0.5241	Frame_Shift_Del	p.E70fs	Non-Targeted Panel Gene	Adult HGBCL, NOS
BCL-20	chr12	27735480	27735480	-	GTAA	exonic	MRPS35	29	9	0.2368	Frame_Shift_Ins	p.A186fs	Non-Targeted Panel Gene	Adult HGBCL, NOS
BCL-20	chr1	203305608	203305608	T	G	exonic	BTG2	68	8	0.1053	Missense_Mutation	p.M1R	Targerted Panel Gene	Adult HGBCL, NOS
BCL-20	chr9	37002663	37002663	G	C	exonic	PAX5	34	6	0.15	Missense_Mutation	p.R197G	Targerted Panel Gene	Adult HGBCL, NOS
BCL-20	chr1	236895403	236895403	G	A	exonic	MTR	91	26	0.2222	Missense_Mutation	p.A1151T	Targerted Panel Gene	Adult HGBCL, NOS
BCL-20	chr7	83135444	83135444	C	G	exonic	PCLO	174	68	0.281	Missense_Mutation	p.K702N	Targerted Panel Gene	Adult HGBCL, NOS
BCL-20	chr17	65056494	65056494	T	C	exonic	GNA13	114	54	0.3214	Missense_Mutation	p.I34V	Targerted Panel Gene	Adult HGBCL, NOS
BCL-20	chr6	134174577	134174577	T	G	exonic	SGK1	37	18	0.3273	Missense_Mutation	p.K124T	Targerted Panel Gene	Adult HGBCL, NOS
BCL-20	chr4	125415248	125415248	C	A	exonic	FAT4	41	22	0.3492	Missense_Mutation	p.N2095K	Targerted Panel Gene	Adult HGBCL, NOS
BCL-20	chr2	151697571	151697571	A	T	exonic	NEB	42	23	0.3538	Missense_Mutation	p.D410E	Targerted Panel Gene	Adult HGBCL, NOS
BCL-20	chrX	32343255	32343255	A	G	exonic	DMD	49	33	0.4024	Missense_Mutation	p.L1750P	Targerted Panel Gene	Adult HGBCL, NOS
BCL-20	chr6	26056240	26056240	T	G	exonic	HIST1H1C	42	35	0.4545	Missense_Mutation	p.K63N	Targerted Panel Gene	Adult HGBCL, NOS
BCL-20	chr12	40274594	40274594	T	A	exonic	LRRK2	15	13	0.4643	Missense_Mutation	p.N556K	Targerted Panel Gene	Adult HGBCL, NOS
BCL-20	chr15	88639412	88639412	G	A	exonic	ISG20	42	2	0.0455	Missense_Mutation	p.G16R	Non-Targeted Panel Gene	Adult HGBCL, NOS
BCL-20	chr22	26365561	26365561	G	A	exonic	SEZ6L	76	4	0.05	Missense_Mutation	p.C930Y	Non-Targeted Panel Gene	Adult HGBCL, NOS
BCL-20	chr13	109782108	109782108	G	A	exonic	IRS2	88	5	0.0538	Missense_Mutation	p.P1316S	Non-Targeted Panel Gene	Adult HGBCL, NOS
BCL-20	chr6	169744235	169744235	G	A	exonic	TCTE3	54	4	0.069	Missense_Mutation	p.H54Y	Non-Targeted Panel Gene	Adult HGBCL, NOS
BCL-20	chr19	45885644	45885644	C	T	exonic	IRF2BP1	40	3	0.0698	Missense_Mutation	p.R44H	Non-Targeted Panel Gene	Adult HGBCL, NOS
BCL-20	chrX	139796324	139796324	A	C	exonic	ATP11C	36	3	0.0769	Missense_Mutation	p.I388M	Non-Targeted Panel Gene	Adult HGBCL, NOS
BCL-20	chr9	121142199	121142199	G	A	exonic	CNTRL	28	3	0.0968	Missense_Mutation	p.D934N	Non-Targeted Panel Gene	Adult HGBCL, NOS
BCL-20	chr6	152310425	152310425	T	G	exonic	SYNE1	74	9	0.1084	Missense_Mutation	p.S5664R	Non-Targeted Panel Gene	Adult HGBCL, NOS
BCL-20	chr2	135130611	135130611	C	G	exonic	RAB3GAP1	29	5	0.1471	Missense_Mutation	p.H376D	Non-Targeted Panel Gene	Adult HGBCL, NOS
BCL-20	chr3	49098932	49098932	C	G	exonic	QARS	119	22	0.156	Missense_Mutation	p.V606L	Non-Targeted Panel Gene	Adult HGBCL, NOS
BCL-20	chr11	130428205	130428205	G	A	exonic	ADAMTS8	10	2	0.1667	Missense_Mutation	p.P28S	Non-Targeted Panel Gene	Adult HGBCL, NOS
BCL-20	chr21	29067063	29067063	T	G	exonic	CCT8	15	3	0.1667	Missense_Mutation	p.E111D	Non-Targeted Panel Gene	Adult HGBCL, NOS
BCL-20	chr13	70107243	70107243	C	T	exonic	KLHL1	154	35	0.1852	Missense_Mutation	p.D153N	Non-Targeted Panel Gene	Adult HGBCL, NOS
BCL-20	chrX	108128828	108128828	T	C	exonic	ATG4A	19	5	0.2083	Missense_Mutation	p.Y57H	Non-Targeted Panel Gene	Adult HGBCL, NOS
BCL-20	chr6	348135	348135	T	C	exonic	DUSP22	90	24	0.2105	Missense_Mutation	p.V99A	Non-Targeted Panel Gene	Adult HGBCL, NOS
BCL-20	chr6	152310825	152310825	T	A	exonic	SYNE1	30	8	0.2105	Missense_Mutation	p.T5587S	Non-Targeted Panel Gene	Adult HGBCL, NOS
BCL-20	chr7	81702610	81702610	T	G	exonic	HGF	22	6	0.2143	Missense_Mutation	p.I715L	Non-Targeted Panel Gene	Adult HGBCL, NOS
BCL-20	chrX	139619699	139619699	G	A	exonic	MCF2	10	3	0.2308	Missense_Mutation	p.T292I	Non-Targeted Panel Gene	Adult HGBCL, NOS
BCL-20	chr2	178536008	178536008	G	C	exonic	TTN	36	11	0.234	Missense_Mutation	p.S33580C	Non-Targeted Panel Gene	Adult HGBCL, NOS
BCL-20	chr1	247157081	247157081	T	G	exonic	ZNF124	51	16	0.2388	Missense_Mutation	p.K119Q	Non-Targeted Panel Gene	Adult HGBCL, NOS
BCL-20	chr6	82365504	82365504	C	G	exonic	TPBG	59	20	0.2532	Missense_Mutation	p.N181K	Non-Targeted Panel Gene	Adult HGBCL, NOS
BCL-20	chr13	87676893	87676893	A	G	exonic	SLITRK5	100	35	0.2593	Missense_Mutation	p.D502G	Non-Targeted Panel Gene	Adult HGBCL, NOS
BCL-20	chrX	80025628	80025628	A	G	exonic	TBX22	105	37	0.2606	Missense_Mutation	p.M42V	Non-Targeted Panel Gene	Adult HGBCL, NOS
BCL-20	chr12	27735481	27735481	C	A	exonic	MRPS35	28	10	0.2632	Missense_Mutation	p.A186E	Non-Targeted Panel Gene	Adult HGBCL, NOS
BCL-20	chr13	32658315	32658315	A	C	exonic	PDS5B	28	10	0.2632	Missense_Mutation	p.Y130S	Non-Targeted Panel Gene	Adult HGBCL, NOS
BCL-20	chrX	85255675	85255675	A	C	exonic	ZNF711	82	30	0.2679	Missense_Mutation	p.N166H	Non-Targeted Panel Gene	Adult HGBCL, NOS
BCL-20	chr9	89402875	89402875	T	C	exonic	SEMA4D	70	30	0.3	Missense_Mutation	p.H83R	Non-Targeted Panel Gene	Adult HGBCL, NOS
BCL-20	chrX	108680711	108680711	G	T	exonic	COL4A5	30	13	0.3023	Missense_Mutation	p.M1319I	Non-Targeted Panel Gene	Adult HGBCL, NOS
BCL-20	chr3	122617069	122617069	G	C	exonic	PARP15	36	16	0.3077	Missense_Mutation	p.G302A	Non-Targeted Panel Gene	Adult HGBCL, NOS

BCL-20	chr4	441904	441904	G	C	exonic	ZNF721	9	4	0.3077	Missense_Mutation	p.H855D	Non-Targeted Panel Gene	Adult HGBCL, NOS
BCL-20	chr16	28986659	28986659	C	A	exonic	LAT	69	31	0.31	Missense_Mutation	p.P151T	Non-Targeted Panel Gene	Adult HGBCL, NOS
BCL-20	chr5	138430378	138430378	C	G	exonic	KDM3B	131	60	0.3141	Missense_Mutation	p.Q1675E	Non-Targeted Panel Gene	Adult HGBCL, NOS
BCL-20	chr9	104793223	104793223	C	T	exonic	ABCA1	77	36	0.3186	Missense_Mutation	p.V1862M	Non-Targeted Panel Gene	Adult HGBCL, NOS
BCL-20	chr17	7336746	7336746	G	C	exonic	ACAP1	60	29	0.3258	Missense_Mutation	p.K4N	Non-Targeted Panel Gene	Adult HGBCL, NOS
BCL-20	chr13	83881105	83881105	G	A	exonic	SLITRK1	47	23	0.3286	Missense_Mutation	p.L135F	Non-Targeted Panel Gene	Adult HGBCL, NOS
BCL-20	chr11	77040533	77040533	G	A	exonic	B3GNT6	102	50	0.3289	Missense_Mutation	p.G328S	Non-Targeted Panel Gene	Adult HGBCL, NOS
BCL-20	chr1	197135239	197135239	A	C	exonic	ASPM	42	21	0.3333	Missense_Mutation	p.I677S	Non-Targeted Panel Gene	Adult HGBCL, NOS
BCL-20	chr5	158823268	158823268	G	A	exonic	EBF1	46	23	0.3333	Missense_Mutation	p.S229F	Non-Targeted Panel Gene	Adult HGBCL, NOS
BCL-20	chr19	7083721	7083721	A	G	exonic	ZNF557	30	15	0.3333	Missense_Mutation	p.R424G	Non-Targeted Panel Gene	Adult HGBCL, NOS
BCL-20	chr2	196902727	196902727	A	G	exonic	PGAP1	21	11	0.3438	Missense_Mutation	p.V48A	Non-Targeted Panel Gene	Adult HGBCL, NOS
BCL-20	chr9	27524922	27524922	T	G	exonic	IFNK	19	10	0.3448	Missense_Mutation	p.Y196D	Non-Targeted Panel Gene	Adult HGBCL, NOS
BCL-20	chr14	23308120	23308120	T	A	exonic	BCL2L2;BCL2L2-PA	74	42	0.3621	Missense_Mutation	p.L118Q	Non-Targeted Panel Gene	Adult HGBCL, NOS
BCL-20	chr2	178776298	178776298	C	T	exonic	TTN	74	44	0.3729	Missense_Mutation	p.E1856K	Non-Targeted Panel Gene	Adult HGBCL, NOS
BCL-20	chr11	118786111	118786111	G	C	exonic	DDX6	30	18	0.375	Missense_Mutation	p.N47K	Non-Targeted Panel Gene	Adult HGBCL, NOS
BCL-20	chr17	81700729	81700729	C	G	exonic	HGS	10	6	0.375	Missense_Mutation	p.A684G	Non-Targeted Panel Gene	Adult HGBCL, NOS
BCL-20	chr1	52388470	52388470	T	C	exonic	ORC1	37	23	0.3833	Missense_Mutation	p.H452R	Non-Targeted Panel Gene	Adult HGBCL, NOS
BCL-20	chrX	9946728	9946728	C	T	exonic	SHROOM2	158	99	0.3852	Missense_Mutation	p.L1548F	Non-Targeted Panel Gene	Adult HGBCL, NOS
BCL-20	chr13	51773987	51773987	A	T	exonic	DHRS12	35	22	0.386	Missense_Mutation	p.N186K	Non-Targeted Panel Gene	Adult HGBCL, NOS
BCL-20	chr6	136583713	136583713	C	G	exonic	MAP3K5	27	17	0.3864	Missense_Mutation	p.E1085Q	Non-Targeted Panel Gene	Adult HGBCL, NOS
BCL-20	chr10	19347885	19347885	A	G	exonic	MALRD1	50	33	0.3976	Missense_Mutation	p.E1339G	Non-Targeted Panel Gene	Adult HGBCL, NOS
BCL-20	chr11	56318485	56318485	A	T	exonic	OR8K3	64	48	0.4286	Missense_Mutation	p.Y60F	Non-Targeted Panel Gene	Adult HGBCL, NOS
BCL-20	chr6	152463486	152463486	T	C	exonic	SYNE1	27	21	0.4375	Missense_Mutation	p.Q655R	Non-Targeted Panel Gene	Adult HGBCL, NOS
BCL-20	chr12	10806648	10806648	T	C	exonic	TAS2R8	37	29	0.4394	Missense_Mutation	p.I111M	Non-Targeted Panel Gene	Adult HGBCL, NOS
BCL-20	chr17	66788864	66788864	G	A	exonic	PRKCA	56	45	0.4412	Missense_Mutation	p.R580Q	Non-Targeted Panel Gene	Adult HGBCL, NOS
BCL-20	chr7	117590400	117590400	G	C	exonic	CFTR	5	4	0.4444	Missense_Mutation	p.G576A	Non-Targeted Panel Gene	Adult HGBCL, NOS
BCL-20	chr19	38385500	38385500	C	T	exonic	GGN	45	36	0.4444	Missense_Mutation	p.V588M	Non-Targeted Panel Gene	Adult HGBCL, NOS
BCL-20	chr7	92532545	92532545	T	G	exonic	RBM48	22	18	0.45	Missense_Mutation	p.N148K	Non-Targeted Panel Gene	Adult HGBCL, NOS
BCL-20	chr12	55707527	55707527	G	C	exonic	ITGA7	55	47	0.4608	Missense_Mutation	p.F52L	Non-Targeted Panel Gene	Adult HGBCL, NOS
BCL-20	chr14	67767816	67767816	C	A	exonic	ZFYVE26	26	23	0.4694	Missense_Mutation	p.S1893I	Non-Targeted Panel Gene	Adult HGBCL, NOS
BCL-20	chr12	49742234	49742234	G	T	exonic	TMBIM6	42	38	0.475	Missense_Mutation	p.G33V	Non-Targeted Panel Gene	Adult HGBCL, NOS
BCL-20	chr16	31508171	31508171	G	A	exonic	C16orf58	42	39	0.4815	Missense_Mutation	p.P68L	Non-Targeted Panel Gene	Adult HGBCL, NOS
BCL-20	chr12	96247186	96247186	C	A	exonic	ELK3	32	31	0.4921	Missense_Mutation	p.Q152K	Non-Targeted Panel Gene	Adult HGBCL, NOS
BCL-20	chr8	117161850	117161850	A	T	exonic	SLC30A8	35	34	0.4928	Missense_Mutation	p.I180F	Non-Targeted Panel Gene	Adult HGBCL, NOS
BCL-20	chr18	49044168	49044168	C	T	exonic	DYM	14	14	0.5	Missense_Mutation	p.E633K	Non-Targeted Panel Gene	Adult HGBCL, NOS
BCL-20	chr5	1293652	1293652	G	A	exonic	TERT	23	24	0.5106	Missense_Mutation	p.H412Y	Non-Targeted Panel Gene	Adult HGBCL, NOS
BCL-20	chr14	34774430	34774430	T	A	exonic	BAZ1A	16	17	0.5152	Missense_Mutation	p.D965V	Non-Targeted Panel Gene	Adult HGBCL, NOS
BCL-20	chr12	50961088	50961088	G	A	exonic	HIGD1C	18	20	0.5263	Missense_Mutation	p.G72E	Non-Targeted Panel Gene	Adult HGBCL, NOS
BCL-20	chr6	155248147	155248147	A	T	exonic	TIAM2	10	12	0.5455	Missense_Mutation	p.D1267V	Non-Targeted Panel Gene	Adult HGBCL, NOS
BCL-20	chr11	118894508	118894508	T	C	exonic	CXCR5	36	47	0.5663	Missense_Mutation	p.Y322H	Non-Targeted Panel Gene	Adult HGBCL, NOS
BCL-20	chr22	45295673	45295673	C	T	exonic	UPK3A	27	36	0.5714	Missense_Mutation	p.P273L	Non-Targeted Panel Gene	Adult HGBCL, NOS
BCL-20	chr5	39331792	39331792	G	A	exonic	C9	30	43	0.589	Missense_Mutation	p.P167S	Non-Targeted Panel Gene	Adult HGBCL, NOS
BCL-20	chr2	48968790	48968790	C	G	exonic	FSHR	46	72	0.6102	Missense_Mutation	p.K254N	Non-Targeted Panel Gene	Adult HGBCL, NOS
BCL-20	chr18	10704581	10704581	A	C	exonic	PIEZO2	52	86	0.6232	Missense_Mutation	p.F1911C	Non-Targeted Panel Gene	Adult HGBCL, NOS
BCL-20	chr15	64162976	64162976	A	C	exonic	PPIB	11	19	0.6333	Missense_Mutation	p.L4R	Non-Targeted Panel Gene	Adult HGBCL, NOS
BCL-20	chr15	43534760	43534760	C	T	exonic	PPIP5K1	20	35	0.6364	Missense_Mutation	p.G1406S	Non-Targeted Panel Gene	Adult HGBCL, NOS
BCL-20	chr3	67654538	67654538	C	A	exonic	SUCLG2	3	10	0.7692	Missense_Mutation	p.A17S	Non-Targeted Panel Gene	Adult HGBCL, NOS
BCL-20	chr1	10336710	10336710	A	G	exonic	KIF1B	6	26	0.8125	Missense_Mutation	p.K987E	Non-Targeted Panel Gene	Adult HGBCL, NOS
BCL-20	chr11	284390	284390	C	T	exonic	NLRP6	67	3	0.0429	Nonsense_Mutation	p.Q788X	Non-Targeted Panel Gene	Adult HGBCL, NOS
BCL-20	chr9	977212	977212	C	T	exonic	DMRT3	37	2	0.0513	Nonsense_Mutation	p.Q71X	Non-Targeted Panel Gene	Adult HGBCL, NOS
BCL-20	chrX	54333149	54333149	C	T	exonic	WNK3	82	6	0.0682	Nonsense_Mutation	p.W175X	Non-Targeted Panel Gene	Adult HGBCL, NOS
BCL-20	chr6	31581036	31581036	G	C	exonic	LTB	31	4	0.1143	Nonsense_Mutation	p.Y136X	Non-Targeted Panel Gene	Adult HGBCL, NOS
BCL-20	chr12	31981122	31981122	C	G	exonic	KIAA1551	42	10	0.1923	Nonsense_Mutation	p.S56X	Non-Targeted Panel Gene	Adult HGBCL, NOS
BCL-20	chr12	27735489	27735489	A	T	exonic	MRPS35	32	13	0.2889	Nonsense_Mutation	p.K189X	Non-Targeted Panel Gene	Adult HGBCL, NOS
BCL-20	chr1	15725484	15725484	G	T	exonic	PLEKHM2	15	9	0.375	Nonsense_Mutation	p.E294X	Non-Targeted Panel Gene	Adult HGBCL, NOS
BCL-20	chr4	67641188	67641188	A	C	exonic	UBA6	8	7	0.4667	Nonsense_Mutation	p.L506X	Non-Targeted Panel Gene	Adult HGBCL, NOS
BCL-20	chr2	165154511	165154511	T	A	exonic	SCN3A	6	9	0.6	Nonsense_Mutation	p.K441X	Non-Targeted Panel Gene	Adult HGBCL, NOS
BCL-21	chr2	86456453	86456456	TTAG	-	exonic	KDM3A	31	6	0.1622	Frame_Shift_Del	p.L190fs	Non-Targeted Panel Gene	Adult HGBCL, NOS
BCL-21	chr17	10418779	10418780	AC	-	exonic	MYH8	69	32	0.3168	Frame_Shift_Del	p.V126fs	Non-Targeted Panel Gene	Adult HGBCL, NOS
BCL-21	chr2	85049513	85049513	C	-	exonic	KCMF1	113	59	0.343	Frame_Shift_Del	p.T250fs	Non-Targeted Panel Gene	Adult HGBCL, NOS
BCL-21	chr4	93085236	93085236	-	TA	exonic	GRID2	52	67	0.5537	Frame_Shift_Ins	p.E162fs	Targerted Panel Gene	Adult HGBCL, NOS
BCL-21	chrX	120535836	120535838	AAA	-	exonic	CUL4B	101	49	0.3267	In_Frame_Del	p.736_736del	Non-Targeted Panel Gene	Adult HGBCL, NOS
BCL-21	chr14	23054576	23054578	CCC	-	exonic	CDH24	48	53	0.5248	In_Frame_Del	p.238_238del	Non-Targeted Panel Gene	Adult HGBCL, NOS
BCL-21	chr19	21116969	21116971	ATA	-	exonic	ZNF714	28	148	0.8409	In_Frame_Del	p.102_103del	Non-Targeted Panel Gene	Adult HGBCL, NOS
BCL-21	chr7	122886180	122886180	-	CGC	exonic	CADPS2	59	27	0.3103	In_Frame_Ins	p.A53delinsGA	Non-Targeted Panel Gene	Adult HGBCL, NOS
BCL-21	chr7	100953923	100953923	C	T	exonic	MUC3A	1087	95	0.0803	Missense_Mutation	p.P715L	Targerted Panel Gene	Adult HGBCL, NOS
BCL-21	chr3	183555426	183555426	C	G	exonic	KLHL6	99	27	0.2143	Missense_Mutation	p.L76F	Targerted Panel Gene	Adult HGBCL, NOS
BCL-21	chr7	83156240	83156240	C	A	exonic	PCLO	136	61	0.3096	Missense_Mutation	p.S134I	Targerted Panel Gene	Adult HGBCL, NOS
BCL-21	chr1	203305689	203305689	G	C	exonic	BTG2	41	22	0.3492	Missense_Mutation	p.G28A	Targerted Panel Gene	Adult HGBCL, NOS
BCL-21	chr9	37034026	37034026	A	T	exonic	PAX5	65	37	0.3627	Missense_Mutation	p.D2E	Targerted Panel Gene	Adult HGBCL, NOS
BCL-21	chr17	65014307	65014307	C	T	exonic	GNA13	78	45	0.3659	Missense_Mutation	p.V362M	Targerted Panel Gene	Adult HGBCL, NOS
BCL-21	chr12	49033564	49033564	C	T	exonic	KMT2D	127	83	0.3952	Missense_Mutation	p.R3714K	Targerted Panel Gene	Adult HGBCL, NOS
BCL-21	chr19	22089299	22089299	T	C	exonic	ZNF257	177	167	0.4855	Missense_Mutation	p.C441R	Targerted Panel Gene	Adult HGBCL, NOS
BCL-21	chr22	22888252	22888252	T	C	exonic	IGLL5	6	14	0.7	Missense_Mutation	p.W67R	Targerted Panel Gene	Adult HGBCL, NOS
BCL-21	chr16	81928573	81928573	C	T	exonic	PLCG2	40	177	0.8157	Missense_Mutation	p.P844S	Targerted Panel Gene	Adult HGBCL, NOS
BCL-21	chr17	4076983	4076983	C	A	exonic	ZZEF1	51	3	0.0556	Missense_Mutation	p.G999V	Non-Targeted Panel Gene	Adult HGBCL, NOS
BCL-21	chr9	2729591	2729591	G	A	exonic	KCNV2	159	10	0.0592	Missense_Mutation	p.S501N	Non-Targeted Panel Gene	Adult HGBCL, NOS
BCL-21	chr2	117821708	117821708	G	A	exonic	DDX18	171	11	0.0604	Missense_Mutation	p.A237T	Non-Targeted Panel Gene	Adult HGBCL, NOS
BCL-21	chr1	23778606	23778606	C	A	exonic	PITHD1	23	2	0.08	Missense_Mutation	p.L31M	Non-Targeted Panel Gene	Adult HGBCL, NOS
BCL-21	chr4	505742	505742	G	T	exonic	PIGG	33	3	0.0833	Missense_Mutation	p.G7C	Non-Targeted Panel Gene	Adult HGBCL, NOS
BCL-21	chr9	742211	742211	C	G	exonic	KANK1	120	14	0.1037	Missense_Mutation	p.Q1235E	Non-Targeted Panel Gene	Adult HGBCL, NOS
BCL-21	chr13	77273599	77273599	G	A	exonic	MYCBP2	82	18	0.18	Missense_Mutation	p.S273F	Non-Targeted Panel Gene	Adult HGBCL, NOS

BCL-21	chr6	64591915	64591915	G	T	exonic	EYS	140	31	0.1813	Missense_Mutation	p.P1318T	Non-Targeted Panel Gene	Adult HGBCL, NOS
BCL-21	chr3	63664036	63664036	A	C	exonic	SNTN	155	36	0.1875	Missense_Mutation	p.S159R	Non-Targeted Panel Gene	Adult HGBCL, NOS
BCL-21	chr3	69118713	69118713	C	A	exonic	LMOD3	155	36	0.1885	Missense_Mutation	p.A548S	Non-Targeted Panel Gene	Adult HGBCL, NOS
BCL-21	chr3	174280992	174280992	G	A	exonic	NLGN1	365	87	0.1925	Missense_Mutation	p.D721N	Non-Targeted Panel Gene	Adult HGBCL, NOS
BCL-21	chr18	57436312	57436312	G	A	exonic	ONECUT2	587	159	0.2131	Missense_Mutation	p.R199H	Non-Targeted Panel Gene	Adult HGBCL, NOS
BCL-21	chr18	50924101	50924101	C	T	exonic	ME2	81	22	0.2136	Missense_Mutation	p.R354W	Non-Targeted Panel Gene	Adult HGBCL, NOS
BCL-21	chr6	37649264	37649264	G	A	exonic	MDGA1	13	4	0.2353	Missense_Mutation	p.P538S	Non-Targeted Panel Gene	Adult HGBCL, NOS
BCL-21	chr6	6318588	6318588	T	C	exonic	F13A1	147	49	0.2487	Missense_Mutation	p.D26G	Non-Targeted Panel Gene	Adult HGBCL, NOS
BCL-21	chr3	126416364	126416364	T	C	exonic	CFAP100	369	127	0.256	Missense_Mutation	p.S92P	Non-Targeted Panel Gene	Adult HGBCL, NOS
BCL-21	chr3	51388923	51388923	G	A	exonic	MANF	126	49	0.28	Missense_Mutation	p.S131N	Non-Targeted Panel Gene	Adult HGBCL, NOS
BCL-21	chr19	45638589	45638589	A	C	exonic	EML2	90	37	0.2913	Missense_Mutation	p.M179R	Non-Targeted Panel Gene	Adult HGBCL, NOS
BCL-21	chr19	48596899	48596899	G	A	exonic	SULT2B1	146	72	0.3303	Missense_Mutation	p.R269H	Non-Targeted Panel Gene	Adult HGBCL, NOS
BCL-21	chr19	36115600	36115600	T	C	exonic	TBCB	177	93	0.3444	Missense_Mutation	p.F14L	Non-Targeted Panel Gene	Adult HGBCL, NOS
BCL-21	chr8	90645122	90645122	C	T	exonic	TMEM64	57	30	0.3448	Missense_Mutation	p.A262T	Non-Targeted Panel Gene	Adult HGBCL, NOS
BCL-21	chrX	41473593	41473593	G	A	exonic	NYX	55	29	0.3452	Missense_Mutation	p.R47H	Non-Targeted Panel Gene	Adult HGBCL, NOS
BCL-21	chr1	40238826	40238826	G	A	exonic	RLF	67	37	0.3558	Missense_Mutation	p.C1375Y	Non-Targeted Panel Gene	Adult HGBCL, NOS
BCL-21	chr16	20780759	20780759	G	T	exonic	ACSM3	145	82	0.3612	Missense_Mutation	p.E228D	Non-Targeted Panel Gene	Adult HGBCL, NOS
BCL-21	chr17	8798648	8798648	C	A	exonic	MFSD6L	99	58	0.3694	Missense_Mutation	p.G158V	Non-Targeted Panel Gene	Adult HGBCL, NOS
BCL-21	chr1	84303244	84303244	G	A	exonic	SAMD13	69	41	0.3727	Missense_Mutation	p.V4I	Non-Targeted Panel Gene	Adult HGBCL, NOS
BCL-21	chr4	2063974	2063974	G	C	exonic	NAT8L	68	41	0.3761	Missense_Mutation	p.K252N	Non-Targeted Panel Gene	Adult HGBCL, NOS
BCL-21	chr4	157136578	157136578	A	C	exonic	GLRB	116	70	0.3763	Missense_Mutation	p.K136T	Non-Targeted Panel Gene	Adult HGBCL, NOS
BCL-21	chr11	73390822	73390822	G	T	exonic	RELT	46	28	0.3784	Missense_Mutation	p.S63I	Non-Targeted Panel Gene	Adult HGBCL, NOS
BCL-21	chr1	171283203	171283203	A	G	exonic	FMO1	80	51	0.3893	Missense_Mutation	p.N415D	Non-Targeted Panel Gene	Adult HGBCL, NOS
BCL-21	chrX	148885900	148885900	A	G	exonic	AFF2	127	83	0.3952	Missense_Mutation	p.D425G	Non-Targeted Panel Gene	Adult HGBCL, NOS
BCL-21	chr10	113847315	113847315	C	A	exonic	DCLRE1A	42	28	0.4	Missense_Mutation	p.A716S	Non-Targeted Panel Gene	Adult HGBCL, NOS
BCL-21	chrX	153943946	153943946	G	A	exonic	RENBP	63	42	0.4	Missense_Mutation	p.R80C	Non-Targeted Panel Gene	Adult HGBCL, NOS
BCL-21	chr2	1922631	1922631	G	T	exonic	MYT1L	107	73	0.4056	Missense_Mutation	p.L380M	Non-Targeted Panel Gene	Adult HGBCL, NOS
BCL-21	chrX	106612228	106612228	C	G	exonic	CXorf57	70	48	0.4068	Missense_Mutation	p.L50V	Non-Targeted Panel Gene	Adult HGBCL, NOS
BCL-21	chr14	68789795	68789795	G	A	exonic	ZFP36L1	35	24	0.4068	Missense_Mutation	p.A321V	Non-Targeted Panel Gene	Adult HGBCL, NOS
BCL-21	chr17	28725956	28725956	G	C	exonic	TLCD1	42	29	0.4085	Missense_Mutation	p.L48V	Non-Targeted Panel Gene	Adult HGBCL, NOS
BCL-21	chr17	8103459	8103459	G	A	exonic	ALOXE3	36	25	0.4098	Missense_Mutation	p.S607L	Non-Targeted Panel Gene	Adult HGBCL, NOS
BCL-21	chrX	107981828	107981828	G	C	exonic	TEX13B	121	84	0.4098	Missense_Mutation	p.H100Q	Non-Targeted Panel Gene	Adult HGBCL, NOS
BCL-21	chr18	23544434	23544434	C	A	exonic	NPC1	57	40	0.4124	Missense_Mutation	p.L680F	Non-Targeted Panel Gene	Adult HGBCL, NOS
BCL-21	chrX	49217953	49217953	A	G	exonic	CACNA1F	149	105	0.4134	Missense_Mutation	p.I1005T	Non-Targeted Panel Gene	Adult HGBCL, NOS
BCL-21	chr19	12112461	12112461	G	T	exonic	ZNF788	58	41	0.4141	Missense_Mutation	p.W305L	Non-Targeted Panel Gene	Adult HGBCL, NOS
BCL-21	chrX	117920358	117920358	G	T	exonic	KLHL13	59	42	0.4158	Missense_Mutation	p.L69I	Non-Targeted Panel Gene	Adult HGBCL, NOS
BCL-21	chr5	74763760	74763760	T	C	exonic	GFM2	35	25	0.4167	Missense_Mutation	p.T27A	Non-Targeted Panel Gene	Adult HGBCL, NOS
BCL-21	chr2	171028359	171028359	T	C	exonic	TLK1	76	55	0.4198	Missense_Mutation	p.R406G	Non-Targeted Panel Gene	Adult HGBCL, NOS
BCL-21	chr1	155034536	155034536	G	A	exonic	DCST1	44	32	0.4211	Missense_Mutation	p.G55S	Non-Targeted Panel Gene	Adult HGBCL, NOS
BCL-21	chrX	110453542	110453542	G	A	exonic	RTL9	75	55	0.4231	Missense_Mutation	p.M975I	Non-Targeted Panel Gene	Adult HGBCL, NOS
BCL-21	chr2	241559646	241559646	C	G	exonic	BOK	27	20	0.4255	Missense_Mutation	p.R55G	Non-Targeted Panel Gene	Adult HGBCL, NOS
BCL-21	chrX	154532969	154532969	G	A	exonic	G6PD	184	137	0.4268	Missense_Mutation	p.L342F	Non-Targeted Panel Gene	Adult HGBCL, NOS
BCL-21	chrX	123253384	123253384	G	T	exonic	GRIA3	143	108	0.4303	Missense_Mutation	p.C117F	Non-Targeted Panel Gene	Adult HGBCL, NOS
BCL-21	chr1	161175087	161175087	C	T	exonic	B4GALT3	38	29	0.4328	Missense_Mutation	p.R132H	Non-Targeted Panel Gene	Adult HGBCL, NOS
BCL-21	chr6	152505255	152505255	C	T	exonic	SYNE1	83	64	0.4354	Missense_Mutation	p.A242T	Non-Targeted Panel Gene	Adult HGBCL, NOS
BCL-21	chr17	8832982	8832982	C	G	exonic	PIK3R6	72	56	0.4375	Missense_Mutation	p.A101P	Non-Targeted Panel Gene	Adult HGBCL, NOS
BCL-21	chr4	38049838	38049838	T	G	exonic	TBC1D1	122	96	0.4404	Missense_Mutation	p.V617G	Non-Targeted Panel Gene	Adult HGBCL, NOS
BCL-21	chr22	25040515	25040515	G	A	exonic	KIAA1671	60	48	0.4444	Missense_Mutation	p.A1129T	Non-Targeted Panel Gene	Adult HGBCL, NOS
BCL-21	chr1	161212389	161212389	G	T	exonic	NDUFS2	70	57	0.4488	Missense_Mutation	p.R342I	Non-Targeted Panel Gene	Adult HGBCL, NOS
BCL-21	chr6	31356805	31356805	T	C	exonic	HLA-B	104	85	0.4497	Missense_Mutation	p.I76V	Non-Targeted Panel Gene	Adult HGBCL, NOS
BCL-21	chr6	31356803	31356803	T	C	exonic	HLA-B	102	86	0.4574	Missense_Mutation	p.I76M	Non-Targeted Panel Gene	Adult HGBCL, NOS
BCL-21	chr16	653538	653538	C	T	exonic	WDR90	117	99	0.4583	Missense_Mutation	p.A416V	Non-Targeted Panel Gene	Adult HGBCL, NOS
BCL-21	chr4	61517350	61517350	G	A	exonic	ADGRL3	44	38	0.4634	Missense_Mutation	p.A31T	Non-Targeted Panel Gene	Adult HGBCL, NOS
BCL-21	chr5	142907736	142907736	T	C	exonic	ARHGAP26	68	60	0.4651	Missense_Mutation	p.C289R	Non-Targeted Panel Gene	Adult HGBCL, NOS
BCL-21	chr8	109575220	109575220	G	T	exonic	SYBU	78	68	0.4658	Missense_Mutation	p.P559T	Non-Targeted Panel Gene	Adult HGBCL, NOS
BCL-21	chr12	31987314	31987314	A	G	exonic	KIAA1551	35	31	0.4697	Missense_Mutation	p.Q1693R	Non-Targeted Panel Gene	Adult HGBCL, NOS
BCL-21	chr16	3013456	3013456	G	A	exonic	CLDN9	124	111	0.4723	Missense_Mutation	p.V32M	Non-Targeted Panel Gene	Adult HGBCL, NOS
BCL-21	chr16	3299794	3299794	A	T	exonic	TIGD7	63	57	0.475	Missense_Mutation	p.V274D	Non-Targeted Panel Gene	Adult HGBCL, NOS
BCL-21	chr3	9475796	9475796	C	G	exonic	SETD5	98	89	0.4759	Missense_Mutation	p.A1247G	Non-Targeted Panel Gene	Adult HGBCL, NOS
BCL-21	chr17	37633373	37633373	G	C	exonic	DDX52	51	47	0.4796	Missense_Mutation	p.S3C	Non-Targeted Panel Gene	Adult HGBCL, NOS
BCL-21	chr10	86437994	86437994	C	T	exonic	WAPL	40	37	0.4805	Missense_Mutation	p.E1145K	Non-Targeted Panel Gene	Adult HGBCL, NOS
BCL-21	chr9	15189781	15189781	C	A	exonic	TTC39B	28	26	0.4815	Missense_Mutation	p.V373L	Non-Targeted Panel Gene	Adult HGBCL, NOS
BCL-21	chr8	10765460	10765460	C	T	exonic	PINX1	103	96	0.4824	Missense_Mutation	p.E310K	Non-Targeted Panel Gene	Adult HGBCL, NOS
BCL-21	chr8	30144566	30144566	T	C	exonic	MBOAT4	46	43	0.4831	Missense_Mutation	p.I12M	Non-Targeted Panel Gene	Adult HGBCL, NOS
BCL-21	chr2	117944076	117944076	T	A	exonic	CCDC93	70	66	0.4853	Missense_Mutation	p.D454V	Non-Targeted Panel Gene	Adult HGBCL, NOS
BCL-21	chr22	29311807	29311807	C	G	exonic	GAS2L1	18	17	0.4857	Missense_Mutation	p.D452E	Non-Targeted Panel Gene	Adult HGBCL, NOS
BCL-21	chr11	66858282	66858282	C	G	exonic	LRFN4	130	124	0.4882	Missense_Mutation	p.L180V	Non-Targeted Panel Gene	Adult HGBCL, NOS
BCL-21	chr9	131447697	131447697	G	T	exonic	PRRC2B	33	32	0.4923	Missense_Mutation	p.R338L	Non-Targeted Panel Gene	Adult HGBCL, NOS
BCL-21	chr2	29193514	29193514	T	C	exonic	ALK	69	67	0.4926	Missense_Mutation	p.K1525E	Non-Targeted Panel Gene	Adult HGBCL, NOS
BCL-21	chr2	75493502	75493502	G	A	exonic	EVA1A	66	65	0.4962	Missense_Mutation	p.R65W	Non-Targeted Panel Gene	Adult HGBCL, NOS
BCL-21	chr9	90613357	90613357	G	T	exonic	DIRAS2	47	47	0.5	Missense_Mutation	p.N157K	Non-Targeted Panel Gene	Adult HGBCL, NOS
BCL-21	chr1	171204051	171204051	G	C	exonic	FMO2	46	46	0.5	Missense_Mutation	p.E272Q	Non-Targeted Panel Gene	Adult HGBCL, NOS
BCL-21	chr22	31140081	31140081	C	A	exonic	PLA2G3	24	24	0.5	Missense_Mutation	p.A92S	Non-Targeted Panel Gene	Adult HGBCL, NOS
BCL-21	chr17	10333158	10333158	A	C	exonic	MYH13	41	42	0.506	Missense_Mutation	p.L697R	Non-Targeted Panel Gene	Adult HGBCL, NOS
BCL-21	chr4	150588124	150588124	G	T	exonic	LRBA	104	108	0.5094	Missense_Mutation	p.T2096N	Non-Targeted Panel Gene	Adult HGBCL, NOS
BCL-21	chr21	17547108	17547108	A	G	exonic	CXADR	52	56	0.5185	Missense_Mutation	p.K42R	Non-Targeted Panel Gene	Adult HGBCL, NOS
BCL-21	chr6	127829647	127829647	G	A	exonic	THEMIS	112	121	0.5193	Missense_Mutation	p.R83C	Non-Targeted Panel Gene	Adult HGBCL, NOS
BCL-21	chr12	98626159	98626159	A	C	exonic	IKBIP	56	61	0.5214	Missense_Mutation	p.M302R	Non-Targeted Panel Gene	Adult HGBCL, NOS
BCL-21	chr11	62392238	62392238	A	C	exonic	ASRGL1	30	33	0.5238	Missense_Mutation	p.H294P	Non-Targeted Panel Gene	Adult HGBCL, NOS
BCL-21	chr2	55344400	55344400	G	T	exonic	CCDC88A	36	40	0.5263	Missense_Mutation	p.Q386K	Non-Targeted Panel Gene	Adult HGBCL, NOS
BCL-21	chr12	122341210	122341210	T	C	exonic	CLIP1	56	64	0.5333	Missense_Mutation	p.K665R	Non-Targeted Panel Gene	Adult HGBCL, NOS
BCL-21	chr5	141639541	141639541	G	C	exonic	RELL2	62	74	0.5441	Missense_Mutation	p.C132S	Non-Targeted Panel Gene	Adult HGBCL, NOS

BCL-21	chr19	7627438	7627438	C	G	exonic	XAB2	10	12	0.5455	Missense_Mutation	p.M109I	Non-Targeted Panel Gene	Adult HGBCL, NOS
BCL-21	chr1	150487946	150487946	G	A	exonic	TARS2	44	54	0.551	Missense_Mutation	p.S52N	Non-Targeted Panel Gene	Adult HGBCL, NOS
BCL-21	chrX	153778461	153778461	A	G	exonic	PLXNB3	26	32	0.5517	Missense_Mutation	p.E1870G	Non-Targeted Panel Gene	Adult HGBCL, NOS
BCL-21	chr9	128940682	128940682	A	G	exonic	PHYHD1	8	10	0.5556	Missense_Mutation	p.N224D	Non-Targeted Panel Gene	Adult HGBCL, NOS
BCL-21	chr16	56983380	56983380	A	G	exonic	CETP	47	59	0.5566	Missense_Mutation	p.D459G	Non-Targeted Panel Gene	Adult HGBCL, NOS
BCL-21	chr8	6442133	6442133	T	C	exonic	MCPH1	80	102	0.5604	Missense_Mutation	p.I216T	Non-Targeted Panel Gene	Adult HGBCL, NOS
BCL-21	chr5	72196503	72196503	G	A	exonic	MAP1B	30	39	0.5652	Missense_Mutation	p.D1050N	Non-Targeted Panel Gene	Adult HGBCL, NOS
BCL-21	chr12	64126076	64126076	G	A	exonic	SRGAP1	51	67	0.5678	Missense_Mutation	p.R775H	Non-Targeted Panel Gene	Adult HGBCL, NOS
BCL-21	chr14	93928496	93928496	G	T	exonic	FAM181A	33	45	0.5769	Missense_Mutation	p.G133W	Non-Targeted Panel Gene	Adult HGBCL, NOS
BCL-21	chr19	50379816	50379816	G	A	exonic	NR1H2	110	154	0.5833	Missense_Mutation	p.E322K	Non-Targeted Panel Gene	Adult HGBCL, NOS
BCL-21	chrX	101079111	101079111	T	A	exonic	TMEM35A	39	58	0.5979	Missense_Mutation	p.Y37N	Non-Targeted Panel Gene	Adult HGBCL, NOS
BCL-21	chr7	48273732	48273732	T	A	exonic	ABCA13	92	138	0.6	Missense_Mutation	p.C1356S	Non-Targeted Panel Gene	Adult HGBCL, NOS
BCL-21	chr19	13904873	13904873	A	G	exonic	C19orf57	34	53	0.6092	Missense_Mutation	p.L7P	Non-Targeted Panel Gene	Adult HGBCL, NOS
BCL-21	chr16	88533856	88533856	A	G	exonic	ZFPM1	3	5	0.625	Missense_Mutation	p.D633G	Non-Targeted Panel Gene	Adult HGBCL, NOS
BCL-21	chr7	121267289	121267289	A	T	exonic	CPED1	70	129	0.6482	Missense_Mutation	p.H903L	Non-Targeted Panel Gene	Adult HGBCL, NOS
BCL-21	chr17	18154689	18154689	G	A	exonic	MYO15A	9	18	0.6667	Missense_Mutation	p.D2720N	Non-Targeted Panel Gene	Adult HGBCL, NOS
BCL-21	chr18	50986990	50986990	G	A	exonic	ELAC1	48	108	0.6923	Missense_Mutation	p.A333T	Non-Targeted Panel Gene	Adult HGBCL, NOS
BCL-21	chr3	172523359	172523359	C	A	exonic	TNFSF10	60	150	0.7143	Missense_Mutation	p.G9V	Non-Targeted Panel Gene	Adult HGBCL, NOS
BCL-21	chr4	76367433	76367433	A	G	exonic	CCDC158	24	107	0.8168	Missense_Mutation	p.I564T	Non-Targeted Panel Gene	Adult HGBCL, NOS
BCL-21	chr17	63929466	63929466	T	A	exonic	CD79B	85	26	0.2342	Nonsense_Mutation	p.K188X	Targerted Panel Gene	Adult HGBCL, NOS
BCL-21	chr1	151776451	151776451	A	C	exonic	TDRKH	81	8	0.0899	Nonsense_Mutation	p.Y344X	Non-Targeted Panel Gene	Adult HGBCL, NOS
BCL-21	chr11	129164357	129164357	G	A	exonic	ARHGAP32	137	52	0.2751	Nonsense_Mutation	p.R63X	Non-Targeted Panel Gene	Adult HGBCL, NOS
BCL-21	chr1	92840587	92840587	C	T	exonic	RPL5	69	37	0.3458	Nonsense_Mutation	p.R248X	Non-Targeted Panel Gene	Adult HGBCL, NOS
BCL-24	chr13	24796296	24796296	G	T	splicing	RNF17	51	3	0.0556	Splice_Site	.	Non-Targeted Panel Gene	Adult HGBCL, NOS
BCL-24	chr12	196883	196883	C	T	splicing	SLC6A12	57	5	0.0806	Splice_Site	.	Non-Targeted Panel Gene	Adult HGBCL, NOS
BCL-24	chr13	102649407	102649407	G	T	splicing	TPP2	14	2	0.125	Splice_Site	.	Non-Targeted Panel Gene	Adult HGBCL, NOS
BCL-24	chr17	81665585	81665590	CTGAGG	-	splicing	OXLD1	77	20	0.2062	Splice_Site	.	Non-Targeted Panel Gene	Adult HGBCL, NOS
BCL-24	chr11	64361242	64361242	G	A	splicing	RPS6KA4	0	24	1	Splice_Site	.	Non-Targeted Panel Gene	Adult HGBCL, NOS
BCL-24	chr1	221739187	221739197	ACTCTTGTTGT	-	exonic	DUSP10	78	11	0.1236	Frame_Shift_Del	p.Y183fs	Non-Targeted Panel Gene	Adult HGBCL, NOS
BCL-24	chr17	67030593	67030594	TT	-	exonic	CACNG4	50	23	0.3151	Frame_Shift_Del	p.S191fs	Non-Targeted Panel Gene	Adult HGBCL, NOS
BCL-24	chr20	31538217	31538217	-	TA	exonic	HM13	156	42	0.21	Frame_Shift_Ins	p.M107fs	Non-Targeted Panel Gene	Adult HGBCL, NOS
BCL-24	chr14	96547875	96547875	-	C	exonic	PAPOLA	82	32	0.2807	Frame_Shift_Ins	p.L493fs	Non-Targeted Panel Gene	Adult HGBCL, NOS
BCL-24	chr12	103302206	103302206	-	AA	exonic	C12orf42	40	21	0.3443	Frame_Shift_Ins	p.K234fs	Non-Targeted Panel Gene	Adult HGBCL, NOS
BCL-24	chr2	231460731	231460775	TCGTCGTCGTCATC	-	exonic	NCL	180	7	0.0374	In_Frame_Del	p.235_250del	Non-Targeted Panel Gene	Adult HGBCL, NOS
BCL-24	chr7	100956356	100956356	G	A	exonic	MUC3A	888	84	0.0846	Missense_Mutation	p.S1526N	Targerted Panel Gene	Adult HGBCL, NOS
BCL-24	chr8	112636925	112636925	T	A	exonic	CSMD3	261	32	0.1092	Missense_Mutation	p.T1163S	Targerted Panel Gene	Adult HGBCL, NOS
BCL-24	chr1	201212609	201212609	G	T	exonic	IGFN1	17	3	0.15	Missense_Mutation	p.L2572F	Targerted Panel Gene	Adult HGBCL, NOS
BCL-24	chr15	33728975	33728975	G	C	exonic	RYR3	108	29	0.2117	Missense_Mutation	p.L2384F	Targerted Panel Gene	Adult HGBCL, NOS
BCL-24	chr5	79731464	79731464	C	T	exonic	CMYA5	145	44	0.2328	Missense_Mutation	p.A900V	Targerted Panel Gene	Adult HGBCL, NOS
BCL-24	chr7	111788695	111788695	C	A	exonic	DOCK4	109	41	0.2733	Missense_Mutation	p.G1123V	Targerted Panel Gene	Adult HGBCL, NOS
BCL-24	chr2	151656008	151656008	C	G	exonic	NEB	85	41	0.3254	Missense_Mutation	p.D2171H	Targerted Panel Gene	Adult HGBCL, NOS
BCL-24	chr1	228287904	228287904	G	T	exonic	OBSCN	58	30	0.3409	Missense_Mutation	p.G3681V	Targerted Panel Gene	Adult HGBCL, NOS
BCL-24	chr1	216246840	216246840	T	A	exonic	USH2A	54	32	0.3721	Missense_Mutation	p.T852S	Targerted Panel Gene	Adult HGBCL, NOS
BCL-24	chr7	128842905	128842905	C	T	exonic	FLNC	145	128	0.4689	Missense_Mutation	p.T834M	Targerted Panel Gene	Adult HGBCL, NOS
BCL-24	chr5	168047515	168047515	C	A	exonic	TENM2	23	37	0.6167	Missense_Mutation	p.N425K	Targerted Panel Gene	Adult HGBCL, NOS
BCL-24	chr11	103114163	103114163	G	C	exonic	DYNC2H1	3	29	0.9062	Missense_Mutation	p.A143P	Targerted Panel Gene	Adult HGBCL, NOS
BCL-24	chr17	7673764	7673764	C	T	exonic	TP53	4	65	0.942	Missense_Mutation	p.E247K	Targerted Panel Gene	Adult HGBCL, NOS
BCL-24	chr19	49432647	49432647	C	A	exonic	SLC17A7	52	2	0.037	Missense_Mutation	p.G341V	Non-Targeted Panel Gene	Adult HGBCL, NOS
BCL-24	chr15	71901204	71901204	G	A	exonic	MYO9A	300	16	0.0505	Missense_Mutation	p.S1046F	Non-Targeted Panel Gene	Adult HGBCL, NOS
BCL-24	chr17	57983386	57983386	T	A	exonic	VEZF1	118	7	0.056	Missense_Mutation	p.E5V	Non-Targeted Panel Gene	Adult HGBCL, NOS
BCL-24	chr18	2906813	2906813	C	A	exonic	EMILIN2	83	5	0.0568	Missense_Mutation	p.A797D	Non-Targeted Panel Gene	Adult HGBCL, NOS
BCL-24	chr11	65543526	65543526	C	G	exonic	LTBP3	42	3	0.0667	Missense_Mutation	p.D793H	Non-Targeted Panel Gene	Adult HGBCL, NOS
BCL-24	chr5	6620138	6620138	G	T	exonic	NSUN2	56	4	0.0667	Missense_Mutation	p.F261L	Non-Targeted Panel Gene	Adult HGBCL, NOS
BCL-24	chr8	130780869	130780869	G	A	exonic	ADCY8	106	8	0.0702	Missense_Mutation	p.H1093Y	Non-Targeted Panel Gene	Adult HGBCL, NOS
BCL-24	chr9	34996793	34996793	A	C	exonic	DNAJB5	607	46	0.0702	Missense_Mutation	p.K360T	Non-Targeted Panel Gene	Adult HGBCL, NOS
BCL-24	chr19	20949334	20949334	A	T	exonic	ZNF85	219	17	0.072	Missense_Mutation	p.T304S	Non-Targeted Panel Gene	Adult HGBCL, NOS
BCL-24	chr6	132323957	132323957	G	T	exonic	MOXD1	38	3	0.0732	Missense_Mutation	p.H363N	Non-Targeted Panel Gene	Adult HGBCL, NOS
BCL-24	chr3	129570889	129570889	T	A	exonic	PLXND1	513	49	0.0872	Missense_Mutation	p.K1216M	Non-Targeted Panel Gene	Adult HGBCL, NOS
BCL-24	chr3	165019666	165019666	G	T	exonic	SI	240	26	0.0974	Missense_Mutation	p.T1120K	Non-Targeted Panel Gene	Adult HGBCL, NOS
BCL-24	chr7	156950659	156950659	G	C	exonic	NOM1	53	6	0.1017	Missense_Mutation	p.V308L	Non-Targeted Panel Gene	Adult HGBCL, NOS
BCL-24	chr3	184140539	184140539	C	T	exonic	EIF2B5	279	32	0.1029	Missense_Mutation	p.T322I	Non-Targeted Panel Gene	Adult HGBCL, NOS
BCL-24	chr3	122685313	122685313	A	G	exonic	PARP14	226	26	0.1032	Missense_Mutation	p.T106A	Non-Targeted Panel Gene	Adult HGBCL, NOS
BCL-24	chr19	58247381	58247381	C	T	exonic	ZNF544	17	2	0.1053	Missense_Mutation	p.A115V	Non-Targeted Panel Gene	Adult HGBCL, NOS
BCL-24	chr8	94488822	94488822	G	T	exonic	VIRMA	456	58	0.1126	Missense_Mutation	p.R1775S	Non-Targeted Panel Gene	Adult HGBCL, NOS
BCL-24	chr3	147396170	147396170	T	A	exonic	ZIC4	265	34	0.1137	Missense_Mutation	p.I124F	Non-Targeted Panel Gene	Adult HGBCL, NOS
BCL-24	chr8	138722040	138722040	G	T	exonic	COL22A1	201	26	0.1145	Missense_Mutation	p.T766N	Non-Targeted Panel Gene	Adult HGBCL, NOS
BCL-24	chr11	3760535	3760535	T	C	exonic	NUP98	169	22	0.1152	Missense_Mutation	p.N393S	Non-Targeted Panel Gene	Adult HGBCL, NOS
BCL-24	chr19	16525262	16525262	G	T	exonic	CHERP	15	2	0.1176	Missense_Mutation	p.P574H	Non-Targeted Panel Gene	Adult HGBCL, NOS
BCL-24	chr10	101064843	101064843	G	C	exonic	KAZALD1	173	25	0.1263	Missense_Mutation	p.G280R	Non-Targeted Panel Gene	Adult HGBCL, NOS
BCL-24	chr10	98259061	98259061	G	A	exonic	LOXL4	118	18	0.1324	Missense_Mutation	p.P290L	Non-Targeted Panel Gene	Adult HGBCL, NOS
BCL-24	chr7	134163540	134163540	G	T	exonic	LRGUK	52	8	0.1333	Missense_Mutation	p.K313N	Non-Targeted Panel Gene	Adult HGBCL, NOS
BCL-24	chr19	1754084	1754084	C	A	exonic	ONECUT3	13	2	0.1333	Missense_Mutation	p.P141H	Non-Targeted Panel Gene	Adult HGBCL, NOS
BCL-24	chr12	120640707	120640707	G	A	exonic	CABP1	12	2	0.1429	Missense_Mutation	p.A8T	Non-Targeted Panel Gene	Adult HGBCL, NOS
BCL-24	chr6	38756020	38756020	A	T	exonic	DNAH8	64	11	0.1467	Missense_Mutation	p.I486F	Non-Targeted Panel Gene	Adult HGBCL, NOS
BCL-24	chr1	16198733	16198733	C	A	exonic	ARHGEF19	81	17	0.1735	Missense_Mutation	p.G755W	Non-Targeted Panel Gene	Adult HGBCL, NOS
BCL-24	chr7	44139569	44139569	C	A	exonic	MYL7	19	4	0.1739	Missense_Mutation	p.E126D	Non-Targeted Panel Gene	Adult HGBCL, NOS
BCL-24	chr1	113957257	113957257	A	T	exonic	HIPK1	77	17	0.1809	Missense_Mutation	p.S576C	Non-Targeted Panel Gene	Adult HGBCL, NOS
BCL-24	chr19	55482035	55482035	A	C	exonic	ZNF628	35	8	0.1818	Missense_Mutation	p.E281A	Non-Targeted Panel Gene	Adult HGBCL, NOS
BCL-24	chr1	89185338	89185338	C	A	exonic	GBP4	35	8	0.186	Missense_Mutation	p.M613I	Non-Targeted Panel Gene	Adult HGBCL, NOS
BCL-24	chr2	7073240	7073240	C	A	exonic	RNF144A	233	58	0.1986	Missense_Mutation	p.P276T	Non-Targeted Panel Gene	Adult HGBCL, NOS
BCL-24	chr9	127214751	127214751	G	T	exonic	RALGPS1	137	34	0.1988	Missense_Mutation	p.G546V	Non-Targeted Panel Gene	Adult HGBCL, NOS
BCL-24	chr19	12691423	12691423	T	A	exonic	FBXW9	84	21	0.2	Missense_Mutation	p.H237L	Non-Targeted Panel Gene	Adult HGBCL, NOS

BCL-24	chr18	6997839	6997839	T	C	exonic	LAMA1	124	32	0.2038	Missense_Mutation	p.E1570G	Non-Targeted Panel Gene	Adult HGBCL, NOS
BCL-24	chr19	48376251	48376251	G	C	exonic	SYNGR4	194	50	0.2041	Missense_Mutation	p.S213T	Non-Targeted Panel Gene	Adult HGBCL, NOS
BCL-24	chr6	27831398	27831398	C	G	exonic	HIST1H4K	49	13	0.2097	Missense_Mutation	p.V44L	Non-Targeted Panel Gene	Adult HGBCL, NOS
BCL-24	chr8	86478949	86478949	T	G	exonic	RMDN1	209	56	0.2113	Missense_Mutation	p.T235P	Non-Targeted Panel Gene	Adult HGBCL, NOS
BCL-24	chr4	155796435	155796435	T	A	exonic	GUCY1B3	127	35	0.216	Missense_Mutation	p.I233N	Non-Targeted Panel Gene	Adult HGBCL, NOS
BCL-24	chr21	46399604	46399604	A	T	exonic	PCNT	36	10	0.2174	Missense_Mutation	p.Q1533H	Non-Targeted Panel Gene	Adult HGBCL, NOS
BCL-24	chr7	100648810	100648810	G	A	exonic	ACTL6B	98	28	0.2222	Missense_Mutation	p.R161W	Non-Targeted Panel Gene	Adult HGBCL, NOS
BCL-24	chr1	1041499	1041499	C	G	exonic	AGRN	184	54	0.2269	Missense_Mutation	p.P325R	Non-Targeted Panel Gene	Adult HGBCL, NOS
BCL-24	chr8	6811353	6811353	G	C	exonic	XKR5	92	27	0.2269	Missense_Mutation	p.L473V	Non-Targeted Panel Gene	Adult HGBCL, NOS
BCL-24	chr1	6470521	6470521	G	C	exonic	PLEKHG5	78	23	0.2277	Missense_Mutation	p.S632R	Non-Targeted Panel Gene	Adult HGBCL, NOS
BCL-24	chr6	31356262	31356262	C	T	exonic	HLA-B	50	15	0.2308	Missense_Mutation	p.R175H	Non-Targeted Panel Gene	Adult HGBCL, NOS
BCL-24	chr19	51873160	51873160	T	C	exonic	ZNF577	80	24	0.2308	Missense_Mutation	p.K277R	Non-Targeted Panel Gene	Adult HGBCL, NOS
BCL-24	chr14	64490436	64490436	A	C	exonic	ZBTB25	75	23	0.2347	Missense_Mutation	p.V33G	Non-Targeted Panel Gene	Adult HGBCL, NOS
BCL-24	chr2	61265986	61265986	T	A	exonic	USP34	130	40	0.2353	Missense_Mutation	p.Q1872L	Non-Targeted Panel Gene	Adult HGBCL, NOS
BCL-24	chr12	96519031	96519031	G	T	exonic	CFAP54	74	23	0.2371	Missense_Mutation	p.C301F	Non-Targeted Panel Gene	Adult HGBCL, NOS
BCL-24	chr5	79069539	79069539	A	T	exonic	DMGDH	57	18	0.24	Missense_Mutation	p.S28T	Non-Targeted Panel Gene	Adult HGBCL, NOS
BCL-24	chr19	44328010	44328010	A	T	exonic	ZNF112	204	65	0.2416	Missense_Mutation	p.I655K	Non-Targeted Panel Gene	Adult HGBCL, NOS
BCL-24	chr7	39987717	39987717	A	T	exonic	CDK13	98	32	0.2462	Missense_Mutation	p.T444S	Non-Targeted Panel Gene	Adult HGBCL, NOS
BCL-24	chr6	111373184	111373184	C	A	exonic	REV3L	86	29	0.2522	Missense_Mutation	p.S1646I	Non-Targeted Panel Gene	Adult HGBCL, NOS
BCL-24	chr4	121037424	121037424	C	G	exonic	NDNF	97	33	0.2538	Missense_Mutation	p.D183H	Non-Targeted Panel Gene	Adult HGBCL, NOS
BCL-24	chr5	69514074	69514074	A	T	exonic	OCLN	73	25	0.2551	Missense_Mutation	p.I286F	Non-Targeted Panel Gene	Adult HGBCL, NOS
BCL-24	chr9	111738366	111738366	A	C	exonic	C9orf84	103	36	0.2571	Missense_Mutation	p.L380R	Non-Targeted Panel Gene	Adult HGBCL, NOS
BCL-24	chr13	41358393	41358393	G	T	exonic	NAA16	59	22	0.2683	Missense_Mutation	p.A393S	Non-Targeted Panel Gene	Adult HGBCL, NOS
BCL-24	chr1	179575806	179575806	G	A	exonic	NPHS2	98	37	0.2741	Missense_Mutation	p.P20L	Non-Targeted Panel Gene	Adult HGBCL, NOS
BCL-24	chr10	80509410	80509410	A	C	exonic	TSPAN14	58	22	0.275	Missense_Mutation	p.N130T	Non-Targeted Panel Gene	Adult HGBCL, NOS
BCL-24	chr11	62727072	62727072	G	C	exonic	HNRNPUL2	94	36	0.2769	Missense_Mutation	p.L29V	Non-Targeted Panel Gene	Adult HGBCL, NOS
BCL-24	chr5	160687820	160687820	A	T	exonic	ATP10B	54	21	0.28	Missense_Mutation	p.N85K	Non-Targeted Panel Gene	Adult HGBCL, NOS
BCL-24	chr3	49661403	49661403	C	A	exonic	BSN	74	29	0.2816	Missense_Mutation	p.S3186R	Non-Targeted Panel Gene	Adult HGBCL, NOS
BCL-24	chr6	31697021	31697021	C	G	exonic	ABHD16A	137	55	0.285	Missense_Mutation	p.W119S	Non-Targeted Panel Gene	Adult HGBCL, NOS
BCL-24	chr11	19055419	19055419	A	T	exonic	MRGPRX2	96	39	0.2889	Missense_Mutation	p.S328R	Non-Targeted Panel Gene	Adult HGBCL, NOS
BCL-24	chr13	113732894	113732894	A	T	exonic	GRK1	72	30	0.2941	Missense_Mutation	p.K402M	Non-Targeted Panel Gene	Adult HGBCL, NOS
BCL-24	chr5	136029083	136029083	C	T	exonic	TGFBI	47	20	0.2985	Missense_Mutation	p.L10F	Non-Targeted Panel Gene	Adult HGBCL, NOS
BCL-24	chr2	207861219	207861219	T	G	exonic	PLEKHM3	64	28	0.3043	Missense_Mutation	p.K665T	Non-Targeted Panel Gene	Adult HGBCL, NOS
BCL-24	chr4	150256162	150256162	C	A	exonic	DCLK2	43	19	0.3065	Missense_Mutation	p.T756N	Non-Targeted Panel Gene	Adult HGBCL, NOS
BCL-24	chr2	108911061	108911061	G	T	exonic	EDAR	24	11	0.3143	Missense_Mutation	p.Q181K	Non-Targeted Panel Gene	Adult HGBCL, NOS
BCL-24	chr5	132575823	132575823	G	A	exonic	RAD50	30	14	0.3182	Missense_Mutation	p.R87H	Non-Targeted Panel Gene	Adult HGBCL, NOS
BCL-24	chr6	52465012	52465012	A	T	exonic	EFHC1	91	44	0.3259	Missense_Mutation	p.Y326F	Non-Targeted Panel Gene	Adult HGBCL, NOS
BCL-24	chr3	160226023	160226023	G	A	exonic	C3orf80	118	62	0.3444	Missense_Mutation	p.G130R	Non-Targeted Panel Gene	Adult HGBCL, NOS
BCL-24	chr6	116399613	116399613	A	T	exonic	DSE	67	36	0.3495	Missense_Mutation	p.E121D	Non-Targeted Panel Gene	Adult HGBCL, NOS
BCL-24	chr20	11890769	11890769	T	C	exonic	BTBD3	69	43	0.3839	Missense_Mutation	p.M1T	Non-Targeted Panel Gene	Adult HGBCL, NOS
BCL-24	chr7	6150172	6150172	G	T	exonic	USP42	187	117	0.3849	Missense_Mutation	p.G659V	Non-Targeted Panel Gene	Adult HGBCL, NOS
BCL-24	chr4	168685513	168685513	G	A	exonic	PALLD	147	98	0.3984	Missense_Mutation	p.R430Q	Non-Targeted Panel Gene	Adult HGBCL, NOS
BCL-24	chr12	109596542	109596542	G	A	exonic	MVK	69	46	0.4	Missense_Mutation	p.D386N	Non-Targeted Panel Gene	Adult HGBCL, NOS
BCL-24	chr18	10680365	10680365	T	A	exonic	PIEZO2	23	17	0.425	Missense_Mutation	p.M2483L	Non-Targeted Panel Gene	Adult HGBCL, NOS
BCL-24	chr7	123662804	123662804	A	T	exonic	LMOD2	32	24	0.4286	Missense_Mutation	p.K406N	Non-Targeted Panel Gene	Adult HGBCL, NOS
BCL-24	chr18	26389998	26389998	G	A	exonic	TAF4B	69	53	0.4344	Missense_Mutation	p.A859T	Non-Targeted Panel Gene	Adult HGBCL, NOS
BCL-24	chr14	99891225	99891225	C	G	exonic	EML1	53	41	0.4362	Missense_Mutation	p.A201G	Non-Targeted Panel Gene	Adult HGBCL, NOS
BCL-24	chr14	104887603	104887603	C	T	exonic	CEP170B	95	76	0.4444	Missense_Mutation	p.R1122W	Non-Targeted Panel Gene	Adult HGBCL, NOS
BCL-24	chr1	179109058	179109058	C	A	exonic	ABL2	59	49	0.4537	Missense_Mutation	p.G737C	Non-Targeted Panel Gene	Adult HGBCL, NOS
BCL-24	chr19	14564835	14564835	A	G	exonic	TECR	75	63	0.4565	Missense_Mutation	p.N195S	Non-Targeted Panel Gene	Adult HGBCL, NOS
BCL-24	chr20	63530199	63530199	G	C	exonic	PTK6	60	53	0.469	Missense_Mutation	p.I349M	Non-Targeted Panel Gene	Adult HGBCL, NOS
BCL-24	chr19	8950776	8950776	G	T	exonic	MUC16	63	56	0.4706	Missense_Mutation	p.A8665D	Non-Targeted Panel Gene	Adult HGBCL, NOS
BCL-24	chr20	36198013	36198013	C	G	exonic	EPB41L1	77	70	0.473	Missense_Mutation	p.P547R	Non-Targeted Panel Gene	Adult HGBCL, NOS
BCL-24	chr19	35513159	35513159	A	G	exonic	DMKN	91	83	0.4743	Missense_Mutation	p.L106P	Non-Targeted Panel Gene	Adult HGBCL, NOS
BCL-24	chr20	56516981	56516981	T	A	exonic	RTFDC1	36	34	0.4857	Missense_Mutation	p.V243D	Non-Targeted Panel Gene	Adult HGBCL, NOS
BCL-24	chr1	94063212	94063212	G	A	exonic	ABCA4	95	90	0.4865	Missense_Mutation	p.P554S	Non-Targeted Panel Gene	Adult HGBCL, NOS
BCL-24	chr8	13567443	13567443	A	G	exonic	C8orf48	44	44	0.5	Missense_Mutation	p.H151R	Non-Targeted Panel Gene	Adult HGBCL, NOS
BCL-24	chr7	43876804	43876804	G	A	exonic	URGCP	73	74	0.5034	Missense_Mutation	p.P844S	Non-Targeted Panel Gene	Adult HGBCL, NOS
BCL-24	chr16	68822063	68822063	G	A	exonic	CDH1	99	102	0.505	Missense_Mutation	p.A592T	Non-Targeted Panel Gene	Adult HGBCL, NOS
BCL-24	chr7	107694642	107694642	A	T	exonic	SLC26A4	123	126	0.506	Missense_Mutation	p.I455F	Non-Targeted Panel Gene	Adult HGBCL, NOS
BCL-24	chr4	73078753	73078753	C	T	exonic	ANKRD17	59	63	0.5164	Missense_Mutation	p.A2433T	Non-Targeted Panel Gene	Adult HGBCL, NOS
BCL-24	chr7	129203555	129203555	G	A	exonic	SMO	43	47	0.5222	Missense_Mutation	p.R168H	Non-Targeted Panel Gene	Adult HGBCL, NOS
BCL-24	chr7	8141973	8141973	C	A	exonic	ICA1	64	70	0.5224	Missense_Mutation	p.G328C	Non-Targeted Panel Gene	Adult HGBCL, NOS
BCL-24	chr18	2937701	2937701	T	C	exonic	LPIN2	26	29	0.5273	Missense_Mutation	p.K387E	Non-Targeted Panel Gene	Adult HGBCL, NOS
BCL-24	chr18	13124698	13124698	G	T	exonic	CEP192	58	65	0.5285	Missense_Mutation	p.L2514F	Non-Targeted Panel Gene	Adult HGBCL, NOS
BCL-24	chr2	61348792	61348792	T	A	exonic	USP34	103	119	0.536	Missense_Mutation	p.R546S	Non-Targeted Panel Gene	Adult HGBCL, NOS
BCL-24	chr20	46035777	46035777	G	A	exonic	SLC12A5	23	29	0.5577	Missense_Mutation	p.A94T	Non-Targeted Panel Gene	Adult HGBCL, NOS
BCL-24	chr17	70176289	70176289	A	G	exonic	KCNJ2	55	71	0.5635	Missense_Mutation	p.E417G	Non-Targeted Panel Gene	Adult HGBCL, NOS
BCL-24	chr3	38316418	38316418	T	A	exonic	SLC22A14	162	215	0.5703	Missense_Mutation	p.F543I	Non-Targeted Panel Gene	Adult HGBCL, NOS
BCL-24	chr2	154699386	154699386	T	C	exonic	KCNJ3	54	78	0.5909	Missense_Mutation	p.M204T	Non-Targeted Panel Gene	Adult HGBCL, NOS
BCL-24	chr14	54981577	54981577	A	C	exonic	WDHD1	61	90	0.596	Missense_Mutation	p.W676G	Non-Targeted Panel Gene	Adult HGBCL, NOS
BCL-24	chr16	69385910	69385910	G	A	exonic	TERF2	35	58	0.6237	Missense_Mutation	p.A21V	Non-Targeted Panel Gene	Adult HGBCL, NOS
BCL-24	chr2	144401226	144401226	G	T	exonic	ZEB2	39	65	0.625	Missense_Mutation	p.L297M	Non-Targeted Panel Gene	Adult HGBCL, NOS
BCL-24	chr10	5889326	5889326	A	C	exonic	ANKRD16	8	14	0.6364	Missense_Mutation	p.L10R	Non-Targeted Panel Gene	Adult HGBCL, NOS
BCL-24	chr1	179108904	179108904	G	T	exonic	ABL2	66	128	0.6598	Missense_Mutation	p.S788Y	Non-Targeted Panel Gene	Adult HGBCL, NOS
BCL-24	chr2	96689657	96689657	A	G	exonic	FER1L5	18	35	0.6604	Missense_Mutation	p.I847V	Non-Targeted Panel Gene	Adult HGBCL, NOS
BCL-24	chr12	113119240	113119240	A	T	exonic	RASAL1	38	81	0.6807	Missense_Mutation	p.F177Y	Non-Targeted Panel Gene	Adult HGBCL, NOS
BCL-24	chr3	165829781	165829781	C	A	exonic	BCHE	86	211	0.7104	Missense_Mutation	p.G418V	Non-Targeted Panel Gene	Adult HGBCL, NOS
BCL-24	chr8	96160524	96160524	C	T	exonic	GDF6	102	299	0.7438	Missense_Mutation	p.D57N	Non-Targeted Panel Gene	Adult HGBCL, NOS
BCL-24	chr11	28097851	28097851	C	G	exonic	KIF18A	9	134	0.9371	Missense_Mutation	p.V33L	Non-Targeted Panel Gene	Adult HGBCL, NOS
BCL-24	chr11	64667484	64667484	G	A	exonic	NRXN2	1	57	0.9828	Missense_Mutation	p.R522C	Non-Targeted Panel Gene	Adult HGBCL, NOS
BCL-24	chrX	120627081	120627081	C	T	exonic	C1GALT1C1	0	269	1	Missense_Mutation	p.R29K	Non-Targeted Panel Gene	Adult HGBCL, NOS

BCL-24	chr14	20243657	20243657	C	A	exonic	OR11H4	92	4	0.0417	Nonsense_Mutation	p.S289X	Non-Targeted Panel Gene	Adult HGBCL, NOS
BCL-24	chr10	7965584	7965584	G	T	exonic	TAF3	69	3	0.0417	Nonsense_Mutation	p.E692X	Non-Targeted Panel Gene	Adult HGBCL, NOS
BCL-24	chr3	198021520	198021520	C	A	exonic	LMLN	227	31	0.1197	Nonsense_Mutation	p.Y517X	Non-Targeted Panel Gene	Adult HGBCL, NOS
BCL-24	chr3	183987901	183987901	G	A	exonic	ABCC5	177	27	0.1324	Nonsense_Mutation	p.Q154X	Non-Targeted Panel Gene	Adult HGBCL, NOS
BCL-24	chr20	18526463	18526463	A	T	exonic	SEC23B	90	20	0.1818	Nonsense_Mutation	p.K309X	Non-Targeted Panel Gene	Adult HGBCL, NOS
BCL-24	chr2	84697710	84697710	G	T	exonic	DNAH6	89	20	0.1835	Nonsense_Mutation	p.E2554X	Non-Targeted Panel Gene	Adult HGBCL, NOS
BCL-24	chr1	3412424	3412424	C	T	exonic	PRDM16	294	70	0.1923	Nonsense_Mutation	p.R743X	Non-Targeted Panel Gene	Adult HGBCL, NOS
BCL-24	chr7	74338825	74338825	C	T	exonic	CLIP2	176	57	0.2436	Nonsense_Mutation	p.Q167X	Non-Targeted Panel Gene	Adult HGBCL, NOS
BCL-24	chr2	144401211	144401211	G	A	exonic	ZEB2	39	60	0.6061	Nonsense_Mutation	p.R302X	Non-Targeted Panel Gene	Adult HGBCL, NOS
BCL-24	chr5	115524895	115524895	C	A	exonic	FEM1C	40	82	0.6721	Nonsense_Mutation	p.E423X	Non-Targeted Panel Gene	Adult HGBCL, NOS
BCL-24	chr13	36335618	36335618	C	T	exonic	SPG20	28	76	0.7308	Nonsense_Mutation	p.W71X	Non-Targeted Panel Gene	Adult HGBCL, NOS
BCL-26	chr3	44720318	44720318	T	A	splicing	ZNF502	46	22	0.3235	Splice_Site	.	Non-Targeted Panel Gene	Adult HGBCL, NOS
BCL-26	chr2	206755506	206755506	C	G	splicing	MDH1B	55	37	0.4022	Splice_Site	.	Non-Targeted Panel Gene	Adult HGBCL, NOS
BCL-26	chr19	16572259	16572262	GCAG	-	exonic	SLC35E1	84	24	0.2222	Frame_Shift_Del	p.L35fs	Non-Targeted Panel Gene	Adult HGBCL, NOS
BCL-26	chr8	40767679	40767680	CC	-	exonic	ZMAT4	33	24	0.4211	Frame_Shift_Del	p.R51fs	Non-Targeted Panel Gene	Adult HGBCL, NOS
BCL-26	chr19	7938143	7938144	TA	-	exonic	TIMM44	9	8	0.4706	Frame_Shift_Del	p.D65fs	Non-Targeted Panel Gene	Adult HGBCL, NOS
BCL-26	chr1	93133736	93133736	G	-	exonic	MTF2	13	18	0.5806	Frame_Shift_Del	p.K296fs	Non-Targeted Panel Gene	Adult HGBCL, NOS
BCL-26	chr16	57026311	57026311	-	CT	exonic	NLRC5	74	9	0.1071	Frame_Shift_Ins	p.S456fs	Non-Targeted Panel Gene	Adult HGBCL, NOS
BCL-26	chr11	17531408	17531408	-	G	exonic	USH1C	96	13	0.1193	Frame_Shift_Ins	p.R80fs	Non-Targeted Panel Gene	Adult HGBCL, NOS
BCL-26	chr8	91991861	91991861	-	CT	exonic	RUNX1T1	69	36	0.3429	Frame_Shift_Ins	p.P230fs	Non-Targeted Panel Gene	Adult HGBCL, NOS
BCL-26	chr3	18416062	18416062	-	T	exonic	SATB1	69	51	0.425	Frame_Shift_Ins	p.A154fs	Non-Targeted Panel Gene	Adult HGBCL, NOS
BCL-26	chr5	177386232	177386252	GTCCCCAAGCTGCC	-	exonic	SLC34A1	125	44	0.2604	In_Frame_Del	p.91_97del	Non-Targeted Panel Gene	Adult HGBCL, NOS
BCL-26	chr13	27920292	27920300	GGCGCCCTG	-	exonic	PDX1	69	32	0.3168	In_Frame_Del	p.52_54del	Non-Targeted Panel Gene	Adult HGBCL, NOS
BCL-26	chr15	101646745	101646765	CGCACCGTGCAGTT	-	exonic	TM2D3	98	67	0.4061	In_Frame_Del	p.154_161del	Non-Targeted Panel Gene	Adult HGBCL, NOS
BCL-26	chr2	176080411	176080437	GCCGCCGCCGCCG	-	exonic	EVX2	6	5	0.4545	In_Frame_Del	p.367_376del	Non-Targeted Panel Gene	Adult HGBCL, NOS
BCL-26	chr7	81961947	81961947	G	T	exonic	CACNA2D1	466	242	0.3418	Missense_Mutation	p.F971L	Targerted Panel Gene	Adult HGBCL, NOS
BCL-26	chr1	7467879	7467879	G	A	exonic	CAMTA1	292	238	0.4491	Missense_Mutation	p.R163Q	Targerted Panel Gene	Adult HGBCL, NOS
BCL-26	chr7	150477383	150477383	T	C	exonic	GIMAP8	830	315	0.2749	Missense_Mutation	p.L534P	Targerted Panel Gene	Adult HGBCL, NOS
BCL-26	chr6	37171461	37171461	C	T	exonic	PIM1	388	157	0.2881	Missense_Mutation	p.L193F	Targerted Panel Gene	Adult HGBCL, NOS
BCL-26	chr17	7674953	7674953	T	C	exonic	TP53	44	176	0.8	Missense_Mutation	p.H154R	Targerted Panel Gene	Adult HGBCL, NOS
BCL-26	chr16	2088569	2088569	C	T	exonic	TSC2	51	27	0.3462	Missense_Mutation	p.R1795C	Targerted Panel Gene	Adult HGBCL, NOS
BCL-26	chr2	53774377	53774377	G	C	exonic	CHAC2	63	3	0.0455	Missense_Mutation	p.G80A	Non-Targeted Panel Gene	Adult HGBCL, NOS
BCL-26	chr12	29371597	29371597	C	A	exonic	ERGIC2	59	3	0.0484	Missense_Mutation	p.V13L	Non-Targeted Panel Gene	Adult HGBCL, NOS
BCL-26	chr2	185789354	185789354	C	A	exonic	FSIP2	65	4	0.058	Missense_Mutation	p.R740S	Non-Targeted Panel Gene	Adult HGBCL, NOS
BCL-26	chr6	24405194	24405194	G	T	exonic	MRS2	79	5	0.0595	Missense_Mutation	p.D73Y	Non-Targeted Panel Gene	Adult HGBCL, NOS
BCL-26	chr7	44140781	44140781	T	C	exonic	MYL7	168	11	0.0615	Missense_Mutation	p.S42G	Non-Targeted Panel Gene	Adult HGBCL, NOS
BCL-26	chr9	34637398	34637398	G	C	exonic	SIGMAR1	180	12	0.0625	Missense_Mutation	p.F58L	Non-Targeted Panel Gene	Adult HGBCL, NOS
BCL-26	chr11	66816133	66816133	A	G	exonic	C11orf80	81	6	0.069	Missense_Mutation	p.T424A	Non-Targeted Panel Gene	Adult HGBCL, NOS
BCL-26	chr16	66885279	66885279	T	A	exonic	PDP2	179	14	0.0725	Missense_Mutation	p.I332N	Non-Targeted Panel Gene	Adult HGBCL, NOS
BCL-26	chr10	97599789	97599789	T	C	exonic	HOGA1	61	5	0.0758	Missense_Mutation	p.V193A	Non-Targeted Panel Gene	Adult HGBCL, NOS
BCL-26	chr4	68229509	68229509	T	C	exonic	TMPRSS11B	66	6	0.0833	Missense_Mutation	p.N232D	Non-Targeted Panel Gene	Adult HGBCL, NOS
BCL-26	chr11	5778064	5778064	T	C	exonic	OR52N5	53	7	0.1167	Missense_Mutation	p.M191V	Non-Targeted Panel Gene	Adult HGBCL, NOS
BCL-26	chr17	43091841	43091841	T	G	exonic	BRCA1	27	4	0.129	Missense_Mutation	p.L1230F	Non-Targeted Panel Gene	Adult HGBCL, NOS
BCL-26	chr17	41806870	41806870	G	A	exonic	P3H4	51	8	0.1356	Missense_Mutation	p.L358F	Non-Targeted Panel Gene	Adult HGBCL, NOS
BCL-26	chr7	74045258	74045258	T	G	exonic	ELN	107	22	0.1705	Missense_Mutation	p.V169G	Non-Targeted Panel Gene	Adult HGBCL, NOS
BCL-26	chr10	123688000	123688000	G	A	exonic	GPR26	213	44	0.1712	Missense_Mutation	p.S285N	Non-Targeted Panel Gene	Adult HGBCL, NOS
BCL-26	chr20	57391748	57391748	G	A	exonic	RBM38	66	14	0.175	Missense_Mutation	p.G56D	Non-Targeted Panel Gene	Adult HGBCL, NOS
BCL-26	chr16	27470358	27470358	C	T	exonic	GTF3C1	193	48	0.1992	Missense_Mutation	p.E1522K	Non-Targeted Panel Gene	Adult HGBCL, NOS
BCL-26	chr1	234429613	234429613	G	C	exonic	TARBP1	62	16	0.2051	Missense_Mutation	p.Q892E	Non-Targeted Panel Gene	Adult HGBCL, NOS
BCL-26	chr7	123147234	123147234	T	A	exonic	SLC13A1	105	30	0.2222	Missense_Mutation	p.Y122F	Non-Targeted Panel Gene	Adult HGBCL, NOS
BCL-26	chr1	156955725	156955725	C	A	exonic	ARHGEF11	209	71	0.2536	Missense_Mutation	p.K582N	Non-Targeted Panel Gene	Adult HGBCL, NOS
BCL-26	chr9	127826563	127826563	G	A	exonic	ENG	79	27	0.2547	Missense_Mutation	p.T157I	Non-Targeted Panel Gene	Adult HGBCL, NOS
BCL-26	chr1	90937910	90937910	T	C	exonic	ZNF644	29	10	0.2564	Missense_Mutation	p.K1088R	Non-Targeted Panel Gene	Adult HGBCL, NOS
BCL-26	chr19	49706302	49706302	C	A	exonic	CPT1C	80	29	0.2661	Missense_Mutation	p.A411D	Non-Targeted Panel Gene	Adult HGBCL, NOS
BCL-26	chr17	73390315	73390315	C	G	exonic	SDK2	70	26	0.2708	Missense_Mutation	p.E1388D	Non-Targeted Panel Gene	Adult HGBCL, NOS
BCL-26	chr15	101737357	101737357	A	G	exonic	LOC100128108	8	3	0.2727	Missense_Mutation	p.S49P	Non-Targeted Panel Gene	Adult HGBCL, NOS
BCL-26	chr7	149179208	149179208	C	T	exonic	ZNF398	112	45	0.2866	Missense_Mutation	p.R275W	Non-Targeted Panel Gene	Adult HGBCL, NOS
BCL-26	chrX	42777769	42777769	G	C	exonic	PPP1R2P9	78	32	0.2909	Missense_Mutation	p.T104S	Non-Targeted Panel Gene	Adult HGBCL, NOS
BCL-26	chr19	1434894	1434894	C	A	exonic	DAZAP1	123	66	0.3492	Missense_Mutation	p.F402L	Non-Targeted Panel Gene	Adult HGBCL, NOS
BCL-26	chr8	55523739	55523739	A	T	exonic	XKR4	96	52	0.3514	Missense_Mutation	p.I489F	Non-Targeted Panel Gene	Adult HGBCL, NOS
BCL-26	chr13	101454090	101454090	C	G	exonic	ITGBL1	121	68	0.3598	Missense_Mutation	p.S102R	Non-Targeted Panel Gene	Adult HGBCL, NOS
BCL-26	chr12	53306851	53306851	T	G	exonic	C12orf10	135	80	0.3721	Missense_Mutation	p.F313V	Non-Targeted Panel Gene	Adult HGBCL, NOS
BCL-26	chr2	36522274	36522274	C	A	exonic	CRIM1	44	27	0.3803	Missense_Mutation	p.P797T	Non-Targeted Panel Gene	Adult HGBCL, NOS
BCL-26	chr14	68789942	68789942	C	G	exonic	ZFP36L1	69	43	0.3839	Missense_Mutation	p.S272T	Non-Targeted Panel Gene	Adult HGBCL, NOS
BCL-26	chr2	222424750	222424750	C	A	exonic	SGPP2	11	7	0.3889	Missense_Mutation	p.H50N	Non-Targeted Panel Gene	Adult HGBCL, NOS
BCL-26	chr19	2111735	2111735	C	T	exonic	AP3D1	25	16	0.3902	Missense_Mutation	p.E961K	Non-Targeted Panel Gene	Adult HGBCL, NOS
BCL-26	chr4	86769789	86769789	T	A	exonic	PTPN13	23	15	0.3947	Missense_Mutation	p.L1509I	Non-Targeted Panel Gene	Adult HGBCL, NOS
BCL-26	chr2	135807396	135807396	G	A	exonic	LCT	61	40	0.396	Missense_Mutation	p.A1302V	Non-Targeted Panel Gene	Adult HGBCL, NOS
BCL-26	chr11	66863857	66863857	C	T	exonic	PC	68	47	0.4087	Missense_Mutation	p.V429I	Non-Targeted Panel Gene	Adult HGBCL, NOS
BCL-26	chr11	64230898	64230898	G	A	exonic	DNAJC4	49	35	0.4167	Missense_Mutation	p.R15H	Non-Targeted Panel Gene	Adult HGBCL, NOS
BCL-26	chr19	40395382	40395382	A	T	exonic	PRX	80	59	0.4245	Missense_Mutation	p.D990E	Non-Targeted Panel Gene	Adult HGBCL, NOS
BCL-26	chr2	215423395	215423395	G	C	exonic	FN1	43	32	0.4267	Missense_Mutation	p.Q450E	Non-Targeted Panel Gene	Adult HGBCL, NOS
BCL-26	chr19	8511830	8511830	C	G	exonic	ZNF414	43	33	0.4342	Missense_Mutation	p.E221Q	Non-Targeted Panel Gene	Adult HGBCL, NOS
BCL-26	chr11	67498726	67498726	C	T	exonic	PITPNM1	58	46	0.4423	Missense_Mutation	p.D452N	Non-Targeted Panel Gene	Adult HGBCL, NOS
BCL-26	chr1	10656684	10656684	C	T	exonic	CASZ1	39	31	0.4429	Missense_Mutation	p.E488K	Non-Targeted Panel Gene	Adult HGBCL, NOS
BCL-26	chr15	43521914	43521914	T	A	exonic	MAP1A	141	113	0.4449	Missense_Mutation	p.D147E	Non-Targeted Panel Gene	Adult HGBCL, NOS
BCL-26	chr4	15563510	15563510	A	C	exonic	CC2D2A	47	38	0.4471	Missense_Mutation	p.K1057T	Non-Targeted Panel Gene	Adult HGBCL, NOS
BCL-26	chr8	86578718	86578718	G	A	exonic	CNGB3	48	39	0.4483	Missense_Mutation	p.L692F	Non-Targeted Panel Gene	Adult HGBCL, NOS
BCL-26	chr11	112025527	112025527	G	C	exonic	DLAT	48	39	0.4483	Missense_Mutation	p.E19Q	Non-Targeted Panel Gene	Adult HGBCL, NOS
BCL-26	chr19	8088098	8088098	G	A	exonic	FBN3	62	51	0.4513	Missense_Mutation	p.A2153V	Non-Targeted Panel Gene	Adult HGBCL, NOS
BCL-26	chr19	4174654	4174654	G	C	exonic	SIRT6	17	14	0.4516	Missense_Mutation	p.P344R	Non-Targeted Panel Gene	Adult HGBCL, NOS
BCL-26	chr15	45117826	45117826	G	C	exonic	DUOXA2	74	61	0.4519	Missense_Mutation	p.G294R	Non-Targeted Panel Gene	Adult HGBCL, NOS

BCL-26	chr14	104774982	104774982	T	G	exonic	AKT1	47	40	0.4598	Missense_Mutation	p.T197P	Non-Targeted Panel Gene	Adult HGBCL, NOS
BCL-26	chr1	153561536	153561536	C	A	exonic	S100A2	35	32	0.4776	Missense_Mutation	p.S66I	Non-Targeted Panel Gene	Adult HGBCL, NOS
BCL-26	chr14	60713606	60713606	T	C	exonic	SIX4	47	43	0.4778	Missense_Mutation	p.Q716R	Non-Targeted Panel Gene	Adult HGBCL, NOS
BCL-26	chr21	33263141	33263141	A	G	exonic	IFNAR2	155	145	0.4833	Missense_Mutation	p.R397G	Non-Targeted Panel Gene	Adult HGBCL, NOS
BCL-26	chr1	197143330	197143330	T	C	exonic	ASPM	37	36	0.4865	Missense_Mutation	p.I308V	Non-Targeted Panel Gene	Adult HGBCL, NOS
BCL-26	chr4	41954085	41954085	A	T	exonic	TMEM33	19	18	0.4865	Missense_Mutation	p.E210D	Non-Targeted Panel Gene	Adult HGBCL, NOS
BCL-26	chr14	49782933	49782933	A	G	exonic	KLHDC2	20	19	0.4872	Missense_Mutation	p.N401D	Non-Targeted Panel Gene	Adult HGBCL, NOS
BCL-26	chr22	29050428	29050428	G	T	exonic	ZNRF3	53	51	0.4904	Missense_Mutation	p.E649D	Non-Targeted Panel Gene	Adult HGBCL, NOS
BCL-26	chr2	96500627	96500627	G	A	exonic	NEURL3	54	52	0.4906	Missense_Mutation	p.A109V	Non-Targeted Panel Gene	Adult HGBCL, NOS
BCL-26	chr20	48644418	48644418	T	A	exonic	PREX1	33	32	0.4923	Missense_Mutation	p.M1198L	Non-Targeted Panel Gene	Adult HGBCL, NOS
BCL-26	chr21	37714611	37714611	A	T	exonic	KCNJ6	73	75	0.5068	Missense_Mutation	p.N182K	Non-Targeted Panel Gene	Adult HGBCL, NOS
BCL-26	chr11	112086941	112086941	G	A	exonic	SDHD	29	31	0.5167	Missense_Mutation	p.G12S	Non-Targeted Panel Gene	Adult HGBCL, NOS
BCL-26	chr15	65058568	65058568	A	G	exonic	RASL12	64	70	0.5224	Missense_Mutation	p.L95P	Non-Targeted Panel Gene	Adult HGBCL, NOS
BCL-26	chr10	95384072	95384072	C	T	exonic	SORBS1	33	37	0.5286	Missense_Mutation	p.S438N	Non-Targeted Panel Gene	Adult HGBCL, NOS
BCL-26	chr10	104036581	104036581	G	A	exonic	COL17A1	45	54	0.5455	Missense_Mutation	p.P1110L	Non-Targeted Panel Gene	Adult HGBCL, NOS
BCL-26	chr4	99287581	99287581	G	A	exonic	ADH1A	13	16	0.5517	Missense_Mutation	p.H35Y	Non-Targeted Panel Gene	Adult HGBCL, NOS
BCL-26	chr2	21141516	21141516	T	C	exonic	TDRD15	25	31	0.5536	Missense_Mutation	p.V1350A	Non-Targeted Panel Gene	Adult HGBCL, NOS
BCL-26	chr6	5404666	5404666	C	T	exonic	FARS2	70	88	0.557	Missense_Mutation	p.T246M	Non-Targeted Panel Gene	Adult HGBCL, NOS
BCL-26	chr14	90680533	90680533	A	T	exonic	TTC7B	17	22	0.5641	Missense_Mutation	p.I216N	Non-Targeted Panel Gene	Adult HGBCL, NOS
BCL-26	chr12	109783719	109783719	C	T	exonic	TRPV4	42	56	0.5714	Missense_Mutation	p.E840K	Non-Targeted Panel Gene	Adult HGBCL, NOS
BCL-26	chr17	48769901	48769901	G	C	exonic	TTLL6	40	60	0.6	Missense_Mutation	p.P746R	Non-Targeted Panel Gene	Adult HGBCL, NOS
BCL-26	chr5	178986162	178986162	G	C	exonic	GRM6	41	73	0.6404	Missense_Mutation	p.L698V	Non-Targeted Panel Gene	Adult HGBCL, NOS
BCL-26	chr9	128626536	128626536	G	C	exonic	SPTAN1	51	92	0.6434	Missense_Mutation	p.R2142P	Non-Targeted Panel Gene	Adult HGBCL, NOS
BCL-26	chr6	160050546	160050546	G	T	exonic	IGF2R	59	113	0.657	Missense_Mutation	p.G863V	Non-Targeted Panel Gene	Adult HGBCL, NOS
BCL-26	chr6	75151986	75151986	G	C	exonic	COL12A1	28	55	0.6627	Missense_Mutation	p.A1294G	Non-Targeted Panel Gene	Adult HGBCL, NOS
BCL-26	chr11	1929123	1929123	C	T	exonic	TNNT3	16	50	0.7576	Missense_Mutation	p.T29I	Non-Targeted Panel Gene	Adult HGBCL, NOS
BCL-26	chr16	88429934	88429934	G	A	exonic	ZNF469	19	157	0.892	Missense_Mutation	p.A822T	Non-Targeted Panel Gene	Adult HGBCL, NOS
BCL-26	chrX	48905143	48905143	T	C	exonic	SLC35A2	17	301	0.9465	Missense_Mutation	p.T284A	Non-Targeted Panel Gene	Adult HGBCL, NOS
BCL-26	chrX	111682233	111682233	C	T	exonic	ALG13	0	39	1	Missense_Mutation	p.T3I	Non-Targeted Panel Gene	Adult HGBCL, NOS
BCL-26	chr17	63929437	63929437	G	C	exonic	CD79B	168	658	0.7956	Nonsense_Mutation	p.Y197X	Targerted Panel Gene	Adult HGBCL, NOS
BCL-26	chr6	106099345	106099345	G	T	exonic	PRDM1	57	259	0.8196	Nonsense_Mutation	p.E153X	Targerted Panel Gene	Adult HGBCL, NOS
BCL-26	chr1	37481780	37481780	G	T	exonic	ZC3H12A	191	14	0.0683	Nonsense_Mutation	p.E255X	Non-Targeted Panel Gene	Adult HGBCL, NOS
BCL-26	chr5	131744626	131744626	G	A	exonic	FNIP1	15	3	0.1667	Nonsense_Mutation	p.R53X	Non-Targeted Panel Gene	Adult HGBCL, NOS
BCL-26	chr19	16572251	16572251	G	T	exonic	SLC35E1	78	35	0.3097	Nonsense_Mutation	p.Y38X	Non-Targeted Panel Gene	Adult HGBCL, NOS
BCL-26	chr5	170080262	170080262	C	T	exonic	DOCK2	178	90	0.3358	Nonsense_Mutation	p.Q1756X	Non-Targeted Panel Gene	Adult HGBCL, NOS
BCL-26	chr5	132749362	132749362	C	A	exonic	CCNI2	39	20	0.339	Nonsense_Mutation	p.Y192X	Non-Targeted Panel Gene	Adult HGBCL, NOS
BCL-26	chr19	57980073	57980073	G	A	exonic	ZNF606	53	34	0.3908	Nonsense_Mutation	p.Q203X	Non-Targeted Panel Gene	Adult HGBCL, NOS
BCL-26	chr11	66366142	66366142	G	A	exonic	SLC29A2	54	41	0.4316	Nonsense_Mutation	p.Q275X	Non-Targeted Panel Gene	Adult HGBCL, NOS
BCL-26	chr5	140969343	140969343	G	A	exonic	PCDHAC2	89	146	0.6186	Nonsense_Mutation	p.W859X	Non-Targeted Panel Gene	Adult HGBCL, NOS
BCL-27	chr7	81959719	81959719	C	G	splicing	CACNA2D1	111	102	0.4789	Splice_Site	.	Targerted Panel Gene	Adult HGBCL, NOS
BCL-27	chr19	581593	581593	T	C	splicing	BSG	33	32	0.4923	Splice_Site	.	Non-Targeted Panel Gene	Adult HGBCL, NOS
BCL-27	chr2	238338361	238338361	G	C	splicing	TRAF3IP1	18	18	0.5	Splice_Site	.	Non-Targeted Panel Gene	Adult HGBCL, NOS
BCL-27	chr6	49472859	49472859	G	A	splicing	CENPQ	7	10	0.5882	Splice_Site	.	Non-Targeted Panel Gene	Adult HGBCL, NOS
BCL-27	chr9	121037879	121037879	A	G	splicing	C5	2	13	0.8667	Splice_Site	.	Non-Targeted Panel Gene	Adult HGBCL, NOS
BCL-27	chr10	75176082	75176082	G	-	exonic	SAMD8	65	49	0.4298	Frame_Shift_Del	p.W270fs	Non-Targeted Panel Gene	Adult HGBCL, NOS
BCL-27	chr12	69654943	69654944	TA	-	exonic	BEST3	31	24	0.4364	Frame_Shift_Del	p.I657fs	Non-Targeted Panel Gene	Adult HGBCL, NOS
BCL-27	chr11	104037323	104037323	-	GCGGAAGCCCTGC	exonic	DDI1	218	65	0.2289	Frame_Shift_Ins	p.L167fs	Non-Targeted Panel Gene	Adult HGBCL, NOS
BCL-27	chr10	70255278	70255278	-	GC	exonic	NPFFR1	71	63	0.4632	Frame_Shift_Ins	p.A324fs	Non-Targeted Panel Gene	Adult HGBCL, NOS
BCL-27	chr19	37565905	37565905	-	A	exonic	ZNF571	28	26	0.4815	Frame_Shift_Ins	p.T175fs	Non-Targeted Panel Gene	Adult HGBCL, NOS
BCL-27	chr17	7675201	7675212	CAGTTGGCAAAA	-	exonic	TP53	76	179	0.702	In_Frame_Del	p.95_98del	Targerted Panel Gene	Adult HGBCL, NOS
BCL-27	chr4	78870998	78871018	CAGCAGCAGCAGC	-	exonic	BMP2K	93	40	0.2857	In_Frame_Del	p.483_489del	Non-Targeted Panel Gene	Adult HGBCL, NOS
BCL-27	chr1	150963556	150963579	AAATCAACCCGAG	-	exonic	SETDB1	73	36	0.3303	In_Frame_Del	p.1162_1169del	Non-Targeted Panel Gene	Adult HGBCL, NOS
BCL-27	chr4	6302180	6302180	-	GAC	exonic	WFS1	248	202	0.444	In_Frame_Ins	p.E795delinsED	Non-Targeted Panel Gene	Adult HGBCL, NOS
BCL-27	chr8	3162183	3162183	C	G	exonic	CSMD1	195	204	0.5113	Missense_Mutation	p.Q1940H	Targerted Panel Gene	Adult HGBCL, NOS
BCL-27	chr1	186087256	186087256	A	T	exonic	HMCN1	198	173	0.4663	Missense_Mutation	p.D3029V	Targerted Panel Gene	Adult HGBCL, NOS
BCL-27	chr12	49026823	49026823	C	T	exonic	KMT2D	331	303	0.4764	Missense_Mutation	p.R5048H	Targerted Panel Gene	Adult HGBCL, NOS
BCL-27	chr4	102600996	102600996	A	T	exonic	NFKB1	180	165	0.4783	Missense_Mutation	p.N580I	Targerted Panel Gene	Adult HGBCL, NOS
BCL-27	chr19	11030823	11030823	G	A	exonic	SMARCA4	225	190	0.4578	Missense_Mutation	p.G1159E	Targerted Panel Gene	Adult HGBCL, NOS
BCL-27	chr12	49630611	49630611	A	G	exonic	PRPF40B	31	4	0.1143	Missense_Mutation	p.M24V	Non-Targeted Panel Gene	Adult HGBCL, NOS
BCL-27	chr10	27538015	27538015	A	G	exonic	RAB18	68	37	0.3524	Missense_Mutation	p.E173G	Non-Targeted Panel Gene	Adult HGBCL, NOS
BCL-27	chr12	8933039	8933039	C	G	exonic	PHC1	37	22	0.3729	Missense_Mutation	p.Q528E	Non-Targeted Panel Gene	Adult HGBCL, NOS
BCL-27	chr12	21765733	21765733	G	A	exonic	KCNJ8	66	42	0.3889	Missense_Mutation	p.S422L	Non-Targeted Panel Gene	Adult HGBCL, NOS
BCL-27	chr19	56574664	56574664	A	T	exonic	ZNF470	50	32	0.3902	Missense_Mutation	p.I72F	Non-Targeted Panel Gene	Adult HGBCL, NOS
BCL-27	chr4	185374353	185374353	T	C	exonic	LRP2BP	63	42	0.4	Missense_Mutation	p.K147E	Non-Targeted Panel Gene	Adult HGBCL, NOS
BCL-27	chr5	40798176	40798176	C	A	exonic	PRKAA1	9	6	0.4	Missense_Mutation	p.S5I	Non-Targeted Panel Gene	Adult HGBCL, NOS
BCL-27	chr1	75791473	75791473	G	A	exonic	RABGGTB	42	28	0.4	Missense_Mutation	p.A161T	Non-Targeted Panel Gene	Adult HGBCL, NOS
BCL-27	chr3	17009693	17009693	G	C	exonic	PLCL2	26	18	0.4091	Missense_Mutation	p.R116T	Non-Targeted Panel Gene	Adult HGBCL, NOS
BCL-27	chr22	46231943	46231943	C	T	exonic	PPARA	116	82	0.4141	Missense_Mutation	p.T288M	Non-Targeted Panel Gene	Adult HGBCL, NOS
BCL-27	chr8	23439911	23439911	T	A	exonic	ENTPD4	49	35	0.4167	Missense_Mutation	p.E296V	Non-Targeted Panel Gene	Adult HGBCL, NOS
BCL-27	chr16	786353	786353	T	C	exonic	RPUSD1	81	58	0.4173	Missense_Mutation	p.H179R	Non-Targeted Panel Gene	Adult HGBCL, NOS
BCL-27	chr11	123729686	123729686	C	T	exonic	ZNF202	78	57	0.4222	Missense_Mutation	p.S181N	Non-Targeted Panel Gene	Adult HGBCL, NOS
BCL-27	chr2	205301599	205301599	A	G	exonic	PARD3B	82	60	0.4225	Missense_Mutation	p.K843R	Non-Targeted Panel Gene	Adult HGBCL, NOS
BCL-27	chr6	132553587	132553587	G	A	exonic	TAAR8	45	34	0.4304	Missense_Mutation	p.A299T	Non-Targeted Panel Gene	Adult HGBCL, NOS
BCL-27	chr1	214643214	214643214	G	A	exonic	CENPF	79	62	0.4397	Missense_Mutation	p.A1626T	Non-Targeted Panel Gene	Adult HGBCL, NOS
BCL-27	chr1	170166714	170166714	C	G	exonic	METTL11B	98	80	0.4494	Missense_Mutation	p.F181L	Non-Targeted Panel Gene	Adult HGBCL, NOS
BCL-27	chr19	45500557	45500557	T	C	exonic	PPM1N	65	54	0.4538	Missense_Mutation	p.C387R	Non-Targeted Panel Gene	Adult HGBCL, NOS
BCL-27	chr4	83455470	83455470	A	G	exonic	HELQ	150	125	0.4545	Missense_Mutation	p.L75P	Non-Targeted Panel Gene	Adult HGBCL, NOS
BCL-27	chr1	173811138	173811138	C	A	exonic	CENPL	49	41	0.4556	Missense_Mutation	p.Q54H	Non-Targeted Panel Gene	Adult HGBCL, NOS
BCL-27	chr4	161459244	161459244	C	G	exonic	FSTL5	37	31	0.4559	Missense_Mutation	p.G562R	Non-Targeted Panel Gene	Adult HGBCL, NOS
BCL-27	chr22	19196540	19196540	C	A	exonic	CLTCL1	166	143	0.4628	Missense_Mutation	p.Q1330H	Non-Targeted Panel Gene	Adult HGBCL, NOS
BCL-27	chr5	140822431	140822431	C	A	exonic	PCDHA5	61	53	0.4649	Missense_Mutation	p.P219H	Non-Targeted Panel Gene	Adult HGBCL, NOS
BCL-27	chr15	34860142	34860142	G	A	exonic	AQR	25	22	0.4681	Missense_Mutation	p.P1348L	Non-Targeted Panel Gene	Adult HGBCL, NOS

BCL-27	chr19	45764114	45764114	A	G	exonic	BHMG1	76	68	0.4722	Missense_Mutation	p.Q638R	Non-Targeted Panel Gene	Adult HGBCL, NOS
BCL-27	chr6	79203620	79203620	G	A	exonic	HMGN3	38	34	0.4722	Missense_Mutation	p.P36L	Non-Targeted Panel Gene	Adult HGBCL, NOS
BCL-27	chr2	25161179	25161179	G	C	exonic	POMC	100	90	0.4737	Missense_Mutation	p.R236G	Non-Targeted Panel Gene	Adult HGBCL, NOS
BCL-27	chr4	31142588	31142588	G	C	exonic	PCDH7	131	119	0.476	Missense_Mutation	p.K1161N	Non-Targeted Panel Gene	Adult HGBCL, NOS
BCL-27	chr18	22993593	22993593	G	A	exonic	RBBP8	38	35	0.4795	Missense_Mutation	p.R589H	Non-Targeted Panel Gene	Adult HGBCL, NOS
BCL-27	chr5	35860987	35860987	T	C	exonic	IL7R	77	73	0.4867	Missense_Mutation	p.I73T	Non-Targeted Panel Gene	Adult HGBCL, NOS
BCL-27	chr22	46248230	46248230	T	C	exonic	CDPF1	103	99	0.4901	Missense_Mutation	p.T19A	Non-Targeted Panel Gene	Adult HGBCL, NOS
BCL-27	chr2	219484934	219484934	C	T	exonic	SPEG	32	31	0.4921	Missense_Mutation	p.L2491F	Non-Targeted Panel Gene	Adult HGBCL, NOS
BCL-27	chr6	29723842	29723842	G	T	exonic	HLA-F	45	44	0.4944	Missense_Mutation	p.W83C	Non-Targeted Panel Gene	Adult HGBCL, NOS
BCL-27	chr1	68444785	68444785	A	G	exonic	RPE65	57	57	0.5	Missense_Mutation	p.I115T	Non-Targeted Panel Gene	Adult HGBCL, NOS
BCL-27	chr6	63280281	63280281	C	G	exonic	LGSN	87	88	0.5029	Missense_Mutation	p.V424L	Non-Targeted Panel Gene	Adult HGBCL, NOS
BCL-27	chr17	62736075	62736075	T	C	exonic	MARCHF10	60	61	0.5041	Missense_Mutation	p.N597S	Non-Targeted Panel Gene	Adult HGBCL, NOS
BCL-27	chr20	3752108	3752108	C	T	exonic	HSPA12B	141	154	0.522	Missense_Mutation	p.A668V	Non-Targeted Panel Gene	Adult HGBCL, NOS
BCL-27	chr6	20152711	20152711	T	A	exonic	MBOAT1	28	31	0.5254	Missense_Mutation	p.Y53F	Non-Targeted Panel Gene	Adult HGBCL, NOS
BCL-27	chr1	13773125	13773125	G	A	exonic	PRDM2	90	100	0.5263	Missense_Mutation	p.D187N	Non-Targeted Panel Gene	Adult HGBCL, NOS
BCL-27	chr5	75597986	75597986	C	T	exonic	POLK	17	19	0.5278	Missense_Mutation	p.P771S	Non-Targeted Panel Gene	Adult HGBCL, NOS
BCL-27	chr3	14148578	14148578	C	T	exonic	XPC	70	82	0.5395	Missense_Mutation	p.G802S	Non-Targeted Panel Gene	Adult HGBCL, NOS
BCL-27	chr8	21982715	21982715	G	A	exonic	XPO7	51	62	0.5487	Missense_Mutation	p.V394I	Non-Targeted Panel Gene	Adult HGBCL, NOS
BCL-27	chr7	10982419	10982419	G	C	exonic	PHF14	9	11	0.55	Missense_Mutation	p.G54R	Non-Targeted Panel Gene	Adult HGBCL, NOS
BCL-27	chr13	32339951	32339951	T	C	exonic	BRCA2	48	66	0.5789	Missense_Mutation	p.F1866L	Non-Targeted Panel Gene	Adult HGBCL, NOS
BCL-27	chrX	140091991	140091991	G	A	exonic	LOC389895	18	159	0.8983	Missense_Mutation	p.M83I	Non-Targeted Panel Gene	Adult HGBCL, NOS
BCL-27	chr9	129322336	129322336	C	T	exonic	C9orf106	4	143	0.9728	Missense_Mutation	p.P175S	Non-Targeted Panel Gene	Adult HGBCL, NOS
BCL-27	chr2	178756428	178756428	A	T	exonic	TTN	58	42	0.42	Nonsense_Mutation	p.L3683X	Non-Targeted Panel Gene	Adult HGBCL, NOS
BCL-27	chr7	100779731	100779731	G	A	exonic	ZAN	37	50	0.5747	Nonsense_Mutation	p.W2201X	Non-Targeted Panel Gene	Adult HGBCL, NOS
BCL-28	chr2	54968769	54968769	G	A	splicing	EML6	100	5	0.0476	Splice_Site	.	Non-Targeted Panel Gene	Adult HGBCL, NOS
BCL-28	chr1	201807950	201807950	T	C	splicing	NAV1	38	37	0.4933	Splice_Site	.	Non-Targeted Panel Gene	Adult HGBCL, NOS
BCL-28	chrX	12976245	12976245	G	A	splicing	TMSB4X	2	9	0.8182	Splice_Site	.	Non-Targeted Panel Gene	Adult HGBCL, NOS
BCL-28	chr2	79122078	79122079	GG	-	exonic	REG1A	76	29	0.2762	Frame_Shift_Del	p.G92fs	Non-Targeted Panel Gene	Adult HGBCL, NOS
BCL-28	chr2	19982521	19982528	CAATTTTG	-	exonic	WDR35	24	10	0.2941	Frame_Shift_Del	p.A50fs	Non-Targeted Panel Gene	Adult HGBCL, NOS
BCL-28	chr2	227094257	227094273	GAAATCCTTGTGG[illegible]	-	exonic	COL4A4	20	9	0.3103	Frame_Shift_Del	p.P407fs	Non-Targeted Panel Gene	Adult HGBCL, NOS
BCL-28	chr2	241441444	241441444	G	-	exonic	FARP2	61	45	0.4245	Frame_Shift_Del	p.Q433fs	Non-Targeted Panel Gene	Adult HGBCL, NOS
BCL-28	chr1	145848392	145848396	TAAAA	-	exonic	NUDT17	37	28	0.4308	Frame_Shift_Del	p.C300fs	Non-Targeted Panel Gene	Adult HGBCL, NOS
BCL-28	chr2	164497277	164497277	-	T	exonic	GRB14	55	34	0.3736	Frame_Shift_Ins	p.G410fs	Non-Targeted Panel Gene	Adult HGBCL, NOS
BCL-28	chr1	28537946	28537946	-	A	exonic	RCC1	45	56	0.5545	Frame_Shift_Ins	p.L402fs	Non-Targeted Panel Gene	Adult HGBCL, NOS
BCL-28	chr4	125316841	125316841	T	C	exonic	FAT4	847	693	0.4488	Missense_Mutation	p.F144L	Targerted Panel Gene	Adult HGBCL, NOS
BCL-28	chr6	26056040	26056040	T	C	exonic	HIST1H1C	317	222	0.4111	Missense_Mutation	p.K130R	Targerted Panel Gene	Adult HGBCL, NOS
BCL-28	chr6	26056065	26056065	T	C	exonic	HIST1H1C	359	251	0.4101	Missense_Mutation	p.K122E	Targerted Panel Gene	Adult HGBCL, NOS
BCL-28	chr6	26123577	26123577	G	A	exonic	HIST1H2BC	261	162	0.383	Missense_Mutation	p.H110Y	Targerted Panel Gene	Adult HGBCL, NOS
BCL-28	chr22	22888181	22888181	T	C	exonic	IGLL5	214	81	0.2746	Missense_Mutation	p.V43A	Targerted Panel Gene	Adult HGBCL, NOS
BCL-28	chr6	37171028	37171028	G	C	exonic	PIM1	105	208	0.6645	Missense_Mutation	p.E79D	Targerted Panel Gene	Adult HGBCL, NOS
BCL-28	chr6	37170839	37170839	G	A	exonic	PIM1	43	117	0.7267	Missense_Mutation	p.G50D	Targerted Panel Gene	Adult HGBCL, NOS
BCL-28	chr2	1664964	1664964	T	C	exonic	PXDN	729	528	0.4194	Missense_Mutation	p.K468E	Targerted Panel Gene	Adult HGBCL, NOS
BCL-28	chr19	38565704	38565704	T	G	exonic	RYR1	203	156	0.4345	Missense_Mutation	p.M4457R	Targerted Panel Gene	Adult HGBCL, NOS
BCL-28	chr3	177034264	177034264	T	C	exonic	TBL1XR1	145	298	0.6727	Missense_Mutation	p.Y395C	Targerted Panel Gene	Adult HGBCL, NOS
BCL-28	chr1	216418543	216418543	C	T	exonic	USH2A	382	329	0.4627	Missense_Mutation	p.V208M	Targerted Panel Gene	Adult HGBCL, NOS
BCL-28	chr8	26508283	26508283	A	G	exonic	PNMA2	150	7	0.0446	Missense_Mutation	p.L158P	Non-Targeted Panel Gene	Adult HGBCL, NOS
BCL-28	chr17	4786286	4786286	A	G	exonic	VMO1	169	10	0.0559	Missense_Mutation	p.C23R	Non-Targeted Panel Gene	Adult HGBCL, NOS
BCL-28	chr8	132882523	132882523	T	C	exonic	TG	29	2	0.0625	Missense_Mutation	p.L267P	Non-Targeted Panel Gene	Adult HGBCL, NOS
BCL-28	chr4	169107172	169107172	C	A	exonic	SH3RF1	43	3	0.0652	Missense_Mutation	p.G725C	Non-Targeted Panel Gene	Adult HGBCL, NOS
BCL-28	chrX	48918642	48918642	C	T	exonic	PIM2	53	4	0.0702	Missense_Mutation	p.G22D	Non-Targeted Panel Gene	Adult HGBCL, NOS
BCL-28	chr1	6462033	6462033	C	T	exonic	TNFRSF25	73	7	0.0875	Missense_Mutation	p.V305M	Non-Targeted Panel Gene	Adult HGBCL, NOS
BCL-28	chr3	57445382	57445382	T	C	exonic	DNAH12	40	4	0.0909	Missense_Mutation	p.Y1406C	Non-Targeted Panel Gene	Adult HGBCL, NOS
BCL-28	chr14	45246961	45246961	T	C	exonic	MIS18BP1	15	2	0.1176	Missense_Mutation	p.E109G	Non-Targeted Panel Gene	Adult HGBCL, NOS
BCL-28	chr10	49739758	49739758	G	C	exonic	OGDHL	63	11	0.1486	Missense_Mutation	p.A741G	Non-Targeted Panel Gene	Adult HGBCL, NOS
BCL-28	chr19	54465336	54465336	G	A	exonic	CDC42EP5	14	4	0.2222	Missense_Mutation	p.P71L	Non-Targeted Panel Gene	Adult HGBCL, NOS
BCL-28	chr4	176225336	176225336	A	G	exonic	ASB5	30	9	0.2308	Missense_Mutation	p.W68R	Non-Targeted Panel Gene	Adult HGBCL, NOS
BCL-28	chr2	79122076	79122076	G	C	exonic	REG1A	76	30	0.283	Missense_Mutation	p.S91T	Non-Targeted Panel Gene	Adult HGBCL, NOS
BCL-28	chr2	188569278	188569278	C	A	exonic	GULP1	16	7	0.3043	Missense_Mutation	p.L147M	Non-Targeted Panel Gene	Adult HGBCL, NOS
BCL-28	chr5	80078118	80078118	G	A	exonic	THBS4	163	83	0.3374	Missense_Mutation	p.C628Y	Non-Targeted Panel Gene	Adult HGBCL, NOS
BCL-28	chr4	119260618	119260618	G	T	exonic	USP53	53	27	0.3375	Missense_Mutation	p.V263F	Non-Targeted Panel Gene	Adult HGBCL, NOS
BCL-28	chr10	93351823	93351823	G	A	exonic	MYOF	60	31	0.3407	Missense_Mutation	p.L1169F	Non-Targeted Panel Gene	Adult HGBCL, NOS
BCL-28	chr10	105216952	105216952	C	T	exonic	SORCS3	216	116	0.3494	Missense_Mutation	p.T855I	Non-Targeted Panel Gene	Adult HGBCL, NOS
BCL-28	chr10	113679900	113679900	T	G	exonic	CASP7	11	6	0.3529	Missense_Mutation	p.L49R	Non-Targeted Panel Gene	Adult HGBCL, NOS
BCL-28	chr7	13935716	13935716	C	A	exonic	ETV1	135	75	0.3571	Missense_Mutation	p.M182I	Non-Targeted Panel Gene	Adult HGBCL, NOS
BCL-28	chr7	117777619	117777619	T	A	exonic	CTTNBP2	114	64	0.3596	Missense_Mutation	p.E890D	Non-Targeted Panel Gene	Adult HGBCL, NOS
BCL-28	chr5	173234860	173234860	C	T	exonic	NKX2-5	33	19	0.3654	Missense_Mutation	p.R75H	Non-Targeted Panel Gene	Adult HGBCL, NOS
BCL-28	chr1	153561561	153561561	G	T	exonic	S100A2	32	19	0.3725	Missense_Mutation	p.L58M	Non-Targeted Panel Gene	Adult HGBCL, NOS
BCL-28	chr1	176711896	176711896	G	A	exonic	PAPPA2	56	34	0.3736	Missense_Mutation	p.C1238Y	Non-Targeted Panel Gene	Adult HGBCL, NOS
BCL-28	chr2	84781638	84781638	A	C	exonic	DNAH6	30	18	0.375	Missense_Mutation	p.T3617P	Non-Targeted Panel Gene	Adult HGBCL, NOS
BCL-28	chr18	31400955	31400955	A	G	exonic	DSG4	13	8	0.381	Missense_Mutation	p.E451G	Non-Targeted Panel Gene	Adult HGBCL, NOS
BCL-28	chr5	122077822	122077822	A	C	exonic	LOX	111	69	0.3833	Missense_Mutation	p.F55C	Non-Targeted Panel Gene	Adult HGBCL, NOS
BCL-28	chr1	24339692	24339692	C	T	exonic	GRHL3	90	56	0.3836	Missense_Mutation	p.T326I	Non-Targeted Panel Gene	Adult HGBCL, NOS
BCL-28	chr16	15032863	15032863	A	G	exonic	PDXDC1	33	21	0.3889	Missense_Mutation	p.Y525C	Non-Targeted Panel Gene	Adult HGBCL, NOS
BCL-28	chr19	45777755	45777755	C	G	exonic	DMPK	75	48	0.3902	Missense_Mutation	p.G291A	Non-Targeted Panel Gene	Adult HGBCL, NOS
BCL-28	chr11	8932215	8932215	G	A	exonic	C11orf16	45	29	0.3919	Missense_Mutation	p.P32S	Non-Targeted Panel Gene	Adult HGBCL, NOS
BCL-28	chr2	132731737	132731737	C	T	exonic	NCKAP5	172	113	0.3965	Missense_Mutation	p.G1815R	Non-Targeted Panel Gene	Adult HGBCL, NOS
BCL-28	chr11	63764256	63764256	C	G	exonic	C11orf95	21	14	0.4	Missense_Mutation	p.R456P	Non-Targeted Panel Gene	Adult HGBCL, NOS
BCL-28	chr9	83679902	83679902	A	C	exonic	UBQLN1	50	34	0.4048	Missense_Mutation	p.V195G	Non-Targeted Panel Gene	Adult HGBCL, NOS
BCL-28	chr11	56542618	56542618	A	T	exonic	OR5M11	136	95	0.4113	Missense_Mutation	p.L214M	Non-Targeted Panel Gene	Adult HGBCL, NOS
BCL-28	chr16	70556252	70556252	T	A	exonic	SF3B3	107	75	0.4121	Missense_Mutation	p.V595E	Non-Targeted Panel Gene	Adult HGBCL, NOS
BCL-28	chr3	44396465	44396465	A	G	exonic	TCAIM	35	25	0.4167	Missense_Mutation	p.Q254R	Non-Targeted Panel Gene	Adult HGBCL, NOS
BCL-28	chr9	101429774	101429774	C	T	exonic	ALDOB	182	131	0.4185	Missense_Mutation	p.G102E	Non-Targeted Panel Gene	Adult HGBCL, NOS

BCL-28	chr19	20006591	20006591	T	C	exonic	ZNF682	52	38	0.4222	Missense_Mutation	p.K304R	Non-Targeted Panel Gene	Adult HGBCL, NOS
BCL-28	chr22	38294488	38294488	T	C	exonic	CSNK1E;LOC40092	12	9	0.4286	Missense_Mutation	p.H311R	Non-Targeted Panel Gene	Adult HGBCL, NOS
BCL-28	chr20	63695563	63695563	G	T	exonic	RTEL1	28	21	0.4286	Missense_Mutation	p.Q1245H	Non-Targeted Panel Gene	Adult HGBCL, NOS
BCL-28	chr8	143728611	143728611	G	A	exonic	FAM83H	133	101	0.4316	Missense_Mutation	p.L284F	Non-Targeted Panel Gene	Adult HGBCL, NOS
BCL-28	chr2	218871610	218871610	C	T	exonic	WNT6	19	16	0.4571	Missense_Mutation	p.P143S	Non-Targeted Panel Gene	Adult HGBCL, NOS
BCL-28	chr2	178675734	178675734	G	T	exonic	TTN	26	22	0.4583	Missense_Mutation	p.P11492T	Non-Targeted Panel Gene	Adult HGBCL, NOS
BCL-28	chr2	74361590	74361590	G	A	exonic	DCTN1	91	79	0.4647	Missense_Mutation	p.T1249I	Non-Targeted Panel Gene	Adult HGBCL, NOS
BCL-28	chr8	70055357	70055357	G	T	exonic	PRDM14	47	41	0.4659	Missense_Mutation	p.N477K	Non-Targeted Panel Gene	Adult HGBCL, NOS
BCL-28	chr2	223052202	223052202	C	T	exonic	KCNE4	101	90	0.4712	Missense_Mutation	p.R20W	Non-Targeted Panel Gene	Adult HGBCL, NOS
BCL-28	chr5	109037450	109037450	A	G	exonic	FER	37	33	0.4714	Missense_Mutation	p.D562G	Non-Targeted Panel Gene	Adult HGBCL, NOS
BCL-28	chr1	147908696	147908696	T	G	exonic	GJA8	103	92	0.4718	Missense_Mutation	p.I247M	Non-Targeted Panel Gene	Adult HGBCL, NOS
BCL-28	chr19	44672615	44672615	G	C	exonic	CEACAM19	48	43	0.4725	Missense_Mutation	p.W25C	Non-Targeted Panel Gene	Adult HGBCL, NOS
BCL-28	chr16	30888913	30888913	C	T	exonic	BCL7C	41	37	0.4744	Missense_Mutation	p.E159K	Non-Targeted Panel Gene	Adult HGBCL, NOS
BCL-28	chr8	130780407	130780407	T	G	exonic	ADCY8	34	32	0.4848	Missense_Mutation	p.K1247Q	Non-Targeted Panel Gene	Adult HGBCL, NOS
BCL-28	chr2	28894726	28894726	G	T	exonic	WDR43	34	32	0.4848	Missense_Mutation	p.D10Y	Non-Targeted Panel Gene	Adult HGBCL, NOS
BCL-28	chr17	42666441	42666441	G	A	exonic	TUBG2	55	52	0.486	Missense_Mutation	p.R381Q	Non-Targeted Panel Gene	Adult HGBCL, NOS
BCL-28	chr1	223759399	223759399	A	G	exonic	CAPN2	44	42	0.4884	Missense_Mutation	p.I483V	Non-Targeted Panel Gene	Adult HGBCL, NOS
BCL-28	chr1	201649345	201649345	G	A	exonic	NAV1	104	101	0.4927	Missense_Mutation	p.R226H	Non-Targeted Panel Gene	Adult HGBCL, NOS
BCL-28	chr8	53881028	53881028	G	A	exonic	RGS20	63	62	0.496	Missense_Mutation	p.D5N	Non-Targeted Panel Gene	Adult HGBCL, NOS
BCL-28	chr9	130636050	130636050	T	A	exonic	FUBP3	39	39	0.5	Missense_Mutation	p.M545K	Non-Targeted Panel Gene	Adult HGBCL, NOS
BCL-28	chr10	104008235	104008235	C	G	exonic	SLK	9	9	0.5	Missense_Mutation	p.T888S	Non-Targeted Panel Gene	Adult HGBCL, NOS
BCL-28	chr13	49915066	49915066	G	C	exonic	SPRYD7	9	9	0.5	Missense_Mutation	p.F196L	Non-Targeted Panel Gene	Adult HGBCL, NOS
BCL-28	chr14	92833223	92833223	T	G	exonic	GOLGA5	57	58	0.5043	Missense_Mutation	p.S607R	Non-Targeted Panel Gene	Adult HGBCL, NOS
BCL-28	chr7	117189353	117189353	T	C	exonic	ST7	46	47	0.5054	Missense_Mutation	p.Y371H	Non-Targeted Panel Gene	Adult HGBCL, NOS
BCL-28	chr6	20546397	20546397	T	C	exonic	CDKAL1	26	27	0.5094	Missense_Mutation	p.I16T	Non-Targeted Panel Gene	Adult HGBCL, NOS
BCL-28	chr19	51693668	51693668	C	T	exonic	SPACA6	53	56	0.5138	Missense_Mutation	p.R48W	Non-Targeted Panel Gene	Adult HGBCL, NOS
BCL-28	chr7	841954	841954	A	C	exonic	SUN1	59	63	0.5164	Missense_Mutation	p.K113T	Non-Targeted Panel Gene	Adult HGBCL, NOS
BCL-28	chr9	136849470	136849470	A	G	exonic	PHPT1	29	31	0.5167	Missense_Mutation	p.I14V	Non-Targeted Panel Gene	Adult HGBCL, NOS
BCL-28	chr8	144261720	144261720	C	G	exonic	MROH1	12	13	0.52	Missense_Mutation	p.R1636G	Non-Targeted Panel Gene	Adult HGBCL, NOS
BCL-28	chr19	14396364	14396364	C	A	exonic	ADGRE5	46	50	0.5208	Missense_Mutation	p.N123K	Non-Targeted Panel Gene	Adult HGBCL, NOS
BCL-28	chr11	44114268	44114268	C	T	exonic	EXT2	52	57	0.5229	Missense_Mutation	p.S237L	Non-Targeted Panel Gene	Adult HGBCL, NOS
BCL-28	chr2	238239160	238239160	G	C	exonic	HES6	95	105	0.525	Missense_Mutation	p.C114W	Non-Targeted Panel Gene	Adult HGBCL, NOS
BCL-28	chr20	63738209	63738209	C	G	exonic	LIME1	13	15	0.5357	Missense_Mutation	p.L99V	Non-Targeted Panel Gene	Adult HGBCL, NOS
BCL-28	chr10	68174589	68174589	G	A	exonic	MYPN	35	44	0.557	Missense_Mutation	p.V539I	Non-Targeted Panel Gene	Adult HGBCL, NOS
BCL-28	chr1	231626931	231626931	G	A	exonic	DISC1	11	15	0.5769	Missense_Mutation	p.A22T	Non-Targeted Panel Gene	Adult HGBCL, NOS
BCL-28	chr18	62574129	62574129	A	G	exonic	ZCCHC2	38	57	0.6	Missense_Mutation	p.Q683R	Non-Targeted Panel Gene	Adult HGBCL, NOS
BCL-28	chr6	30921617	30921617	G	C	exonic	VARS2	12	19	0.6129	Missense_Mutation	p.R554P	Non-Targeted Panel Gene	Adult HGBCL, NOS
BCL-28	chr15	55418422	55418422	C	T	exonic	C15orf65	13	21	0.6176	Missense_Mutation	p.P73L	Non-Targeted Panel Gene	Adult HGBCL, NOS
BCL-28	chr8	14090453	14090453	A	G	exonic	SGCZ	19	50	0.7246	Missense_Mutation	p.L310P	Non-Targeted Panel Gene	Adult HGBCL, NOS
BCL-28	chr17	9943154	9943154	A	G	exonic	GAS7	26	82	0.7593	Missense_Mutation	p.M233T	Non-Targeted Panel Gene	Adult HGBCL, NOS
BCL-28	chr6	46722884	46722884	G	A	exonic	PLA2G7	5	19	0.7917	Missense_Mutation	p.P3L	Non-Targeted Panel Gene	Adult HGBCL, NOS
BCL-28	chrX	91436161	91436161	G	C	exonic	PABPC5	21	157	0.882	Missense_Mutation	p.R195T	Non-Targeted Panel Gene	Adult HGBCL, NOS
BCL-28	chr17	16940415	16940415	G	T	exonic	TNFRSF13B	1	26	0.963	Missense_Mutation	p.A181E	Non-Targeted Panel Gene	Adult HGBCL, NOS
BCL-28	chrX	47915116	47915116	A	G	exonic	ZNF81	0	21	1	Missense_Mutation	p.N157S	Non-Targeted Panel Gene	Adult HGBCL, NOS
BCL-28	chrX	107603230	107603230	-	AGT	exonic	FRMPD3	28	25	0.4464	Nonsense_Mutation	p.Q1764delinsQX	Targeted Panel Gene	Adult HGBCL, NOS
BCL-28	chr10	62091189	62091189	G	T	exonic	ARID5B	67	38	0.3619	Nonsense_Mutation	p.E576X	Non-Targeted Panel Gene	Adult HGBCL, NOS
BCL-28	chr11	4587591	4587591	C	G	exonic	OR52I2	54	31	0.3647	Nonsense_Mutation	p.S260X	Non-Targeted Panel Gene	Adult HGBCL, NOS
BCL-28	chr1	62268486	62268486	C	A	exonic	KANK4	46	31	0.4026	Nonsense_Mutation	p.E678X	Non-Targeted Panel Gene	Adult HGBCL, NOS
BCL-28	chr4	42656828	42656828	C	A	exonic	ATP8A1	16	12	0.4286	Nonsense_Mutation	p.E16X	Non-Targeted Panel Gene	Adult HGBCL, NOS
BCL-28	chr11	73649084	73649084	C	T	exonic	PLEKHB1	49	38	0.4368	Nonsense_Mutation	p.Q31X	Non-Targeted Panel Gene	Adult HGBCL, NOS
BCL-28	chr17	41308297	41308297	G	T	exonic	KRTAP16-1	188	155	0.4519	Nonsense_Mutation	p.C319X	Non-Targeted Panel Gene	Adult HGBCL, NOS
BCL-28	chr11	75276868	75276868	G	C	exonic	ARRB1	104	99	0.4877	Nonsense_Mutation	p.Y249X	Non-Targeted Panel Gene	Adult HGBCL, NOS
BCL-28	chr6	42721764	42721764	C	A	exonic	PRPH2	25	35	0.5833	Nonsense_Mutation	p.E191X	Non-Targeted Panel Gene	Adult HGBCL, NOS
BCL-28	chr3	38141150	38141150	T	C	exonic	MYD88	322	235	0.4204	Nonstop_Mutation	p.L265P	Targerted Panel Gene	Adult HGBCL, NOS
BCL-29	chr6	37170980	37170980	G	A	splicing	PIM1	361	297	0.4514	Splice_Site	.	Targerted Panel Gene	Adult HGBCL, NOS
BCL-29	chr6	37170772	37170772	G	C	splicing	PIM1	186	115	0.3821	Splice_Site	.	Targerted Panel Gene	Adult HGBCL, NOS
BCL-29	chr1	37475461	37475461	G	C	splicing	ZC3H12A	53	27	0.3375	Splice_Site	.	Non-Targeted Panel Gene	Adult HGBCL, NOS
BCL-29	chr3	102456192	102456192	G	A	splicing	ZPLD1	20	16	0.4444	Splice_Site	.	Non-Targeted Panel Gene	Adult HGBCL, NOS
BCL-29	chr12	11650161	11650161	G	A	splicing	ETV6	46	44	0.4889	Splice_Site	.	Non-Targeted Panel Gene	Adult HGBCL, NOS
BCL-29	chr1	17410052	17410052	C	T	splicing	RCC2	29	36	0.5538	Splice_Site	.	Non-Targeted Panel Gene	Adult HGBCL, NOS
BCL-29	chr22	22888169	22888181	TGCGCCCAATGGT	-	exonic	IGLL5	80	98	0.5506	Frame_Shift_Del	p.C4fs	Targerted Panel Gene	Adult HGBCL, NOS
BCL-29	chr6	27824296	27824303	GTGCTGAA	-	exonic	HIST1H4J	124	9	0.0677	Frame_Shift_Del	p.V58fs	Non-Targeted Panel Gene	Adult HGBCL, NOS
BCL-29	chr12	122986255	122986259	GCGCA	-	exonic	PITPNM2	119	46	0.2788	Frame_Shift_Del	p.L1273fs	Non-Targeted Panel Gene	Adult HGBCL, NOS
BCL-29	chr1	186313985	186313986	CT	-	exonic	TPR	19	8	0.2963	Frame_Shift_Del	p.R2359fs	Non-Targeted Panel Gene	Adult HGBCL, NOS
BCL-29	chr4	105842131	105842137	ATAGGTA	-	exonic	GSTCD	104	51	0.329	Frame_Shift_Del	p.I501fs	Non-Targeted Panel Gene	Adult HGBCL, NOS
BCL-29	chr17	82752244	82752244	G	-	exonic	TBCD	28	15	0.3488	Frame_Shift_Del	p.E17fs	Non-Targeted Panel Gene	Adult HGBCL, NOS
BCL-29	chr14	69572231	69572231	-	CGTTG	exonic	CCDC177	39	21	0.35	Frame_Shift_Ins	p.R464fs	Non-Targeted Panel Gene	Adult HGBCL, NOS
BCL-29	chr3	170084683	170084683	-	AAAAT	exonic	GPR160	26	14	0.35	Frame_Shift_Ins	p.K237fs	Non-Targeted Panel Gene	Adult HGBCL, NOS
BCL-29	chr2	241498298	241498298	-	G	exonic	STK25	38	55	0.5914	Frame_Shift_Ins	p.P323fs	Non-Targeted Panel Gene	Adult HGBCL, NOS
BCL-29	chr7	39951010	39951018	GCCCCAGCC	-	exonic	CDK13	42	21	0.3333	In_Frame_Del	p.123_126del	Non-Targeted Panel Gene	Adult HGBCL, NOS
BCL-29	chr17	38558311	38558313	TCC	-	exonic	SRCIN1	53	45	0.4592	In_Frame_Del	p.705_706del	Non-Targeted Panel Gene	Adult HGBCL, NOS
BCL-29	chr16	67884914	67884925	GCTGCCGCCGCC	-	exonic	NRN1L	40	46	0.5349	In_Frame_Del	p.4_8del	Non-Targeted Panel Gene	Adult HGBCL, NOS
BCL-29	chr12	92145533	92145533	C	T	exonic	BTG1	369	189	0.3387	Missense_Mutation	p.M1I	Targerted Panel Gene	Adult HGBCL, NOS
BCL-29	chr12	92145456	92145456	C	T	exonic	BTG1	345	171	0.3314	Missense_Mutation	p.R27H	Targerted Panel Gene	Adult HGBCL, NOS
BCL-29	chr1	33587133	33587133	G	A	exonic	CSMD2	328	39	0.1054	Missense_Mutation	p.L2300F	Targerted Panel Gene	Adult HGBCL, NOS
BCL-29	chr7	154587875	154587875	C	G	exonic	DPP6	392	278	0.4131	Missense_Mutation	p.P285A	Targerted Panel Gene	Adult HGBCL, NOS
BCL-29	chr4	186611716	186611716	T	C	exonic	FAT1	221	183	0.453	Missense_Mutation	p.N3175D	Targerted Panel Gene	Adult HGBCL, NOS
BCL-29	chr4	125319882	125319882	T	A	exonic	FAT4	374	289	0.4359	Missense_Mutation	p.N1157K	Targerted Panel Gene	Adult HGBCL, NOS
BCL-29	chr13	40665754	40665754	G	C	exonic	FOXO1	143	85	0.3728	Missense_Mutation	p.S153R	Targerted Panel Gene	Adult HGBCL, NOS
BCL-29	chr22	22888214	22888214	C	T	exonic	IGLL5	33	98	0.7481	Missense_Mutation	p.P19S	Targerted Panel Gene	Adult HGBCL, NOS
BCL-29	chr22	22888211	22888211	G	A	exonic	IGLL5	34	102	0.75	Missense_Mutation	p.E18K	Targerted Panel Gene	Adult HGBCL, NOS
BCL-29	chr22	22888213	22888213	G	T	exonic	IGLL5	34	98	0.7424	Missense_Mutation	p.E18D	Targerted Panel Gene	Adult HGBCL, NOS

BCL-29	chr12	124355560	124355560	G	A	exonic	NCOR2	343	134	0.2809	Missense_Mutation	p.P1085S	Targerted Panel Gene	Adult HGBCL, NOS
BCL-29	chr6	37171200	37171200	C	T	exonic	PIM1	469	339	0.4196	Missense_Mutation	p.L106F	Targerted Panel Gene	Adult HGBCL, NOS
BCL-29	chr6	37171263	37171263	C	G	exonic	PIM1	407	342	0.4566	Missense_Mutation	p.Q127E	Targerted Panel Gene	Adult HGBCL, NOS
BCL-29	chr6	37171374	37171374	C	G	exonic	PIM1	327	293	0.4711	Missense_Mutation	p.L164V	Targerted Panel Gene	Adult HGBCL, NOS
BCL-29	chr6	37170643	37170643	C	A	exonic	PIM1	152	123	0.4473	Missense_Mutation	p.T23N	Targerted Panel Gene	Adult HGBCL, NOS
BCL-29	chr6	37170824	37170824	G	T	exonic	PIM1	229	185	0.4469	Missense_Mutation	p.G45V	Targerted Panel Gene	Adult HGBCL, NOS
BCL-29	chr6	37170647	37170647	G	C	exonic	PIM1	145	117	0.4466	Missense_Mutation	p.K24N	Targerted Panel Gene	Adult HGBCL, NOS
BCL-29	chr6	37170604	37170604	C	T	exonic	PIM1	172	129	0.4286	Missense_Mutation	p.A10V	Targerted Panel Gene	Adult HGBCL, NOS
BCL-29	chr8	109405113	109405113	A	G	exonic	PKHD1L1	160	132	0.4521	Missense_Mutation	p.Y551C	Targerted Panel Gene	Adult HGBCL, NOS
BCL-29	chr20	8760425	8760425	C	A	exonic	PLCB1	182	117	0.3913	Missense_Mutation	p.A892D	Targerted Panel Gene	Adult HGBCL, NOS
BCL-29	chr16	11254950	11254950	G	C	exonic	SOCS1	295	222	0.4286	Missense_Mutation	p.L177V	Targerted Panel Gene	Adult HGBCL, NOS
BCL-29	chr3	177033124	177033124	A	C	exonic	TBL1XR1	72	257	0.7812	Missense_Mutation	p.D421E	Targerted Panel Gene	Adult HGBCL, NOS
BCL-29	chr16	72793599	72793599	A	G	exonic	ZFHX3	518	445	0.4616	Missense_Mutation	p.L3028S	Targerted Panel Gene	Adult HGBCL, NOS
BCL-29	chr19	23362455	23362455	C	G	exonic	ZNF91	43	164	0.7923	Missense_Mutation	p.R175T	Targerted Panel Gene	Adult HGBCL, NOS
BCL-29	chr16	3738616	3738616	C	T	exonic	CREBBP	301	252	0.4557	Missense_Mutation	p.R1446H	Targerted Panel Gene	Adult HGBCL, NOS
BCL-29	chr7	78194945	78194945	T	A	exonic	MAGI2	167	16	0.0874	Missense_Mutation	p.E733V	Non-Targeted Panel Gene	Adult HGBCL, NOS
BCL-29	chr6	144797882	144797882	C	T	exonic	UTRN	76	9	0.1059	Missense_Mutation	p.P3046L	Non-Targeted Panel Gene	Adult HGBCL, NOS
BCL-29	chr3	125489456	125489456	G	A	exonic	SNX4	28	10	0.2632	Missense_Mutation	p.S202F	Non-Targeted Panel Gene	Adult HGBCL, NOS
BCL-29	chr20	499909	499909	C	A	exonic	CSNK2A1	19	7	0.2692	Missense_Mutation	p.R80L	Non-Targeted Panel Gene	Adult HGBCL, NOS
BCL-29	chr7	123129452	123129452	G	T	exonic	SLC13A1	20	8	0.2857	Missense_Mutation	p.T197N	Non-Targeted Panel Gene	Adult HGBCL, NOS
BCL-29	chr12	100391041	100391041	T	C	exonic	SLC17A8	89	37	0.2937	Missense_Mutation	p.I132T	Non-Targeted Panel Gene	Adult HGBCL, NOS
BCL-29	chr11	66068676	66068676	A	C	exonic	SF3B2	27	13	0.325	Missense_Mutation	p.Q873H	Non-Targeted Panel Gene	Adult HGBCL, NOS
BCL-29	chr17	1270748	1270748	G	A	exonic	BHLHA9	45	22	0.3284	Missense_Mutation	p.R62Q	Non-Targeted Panel Gene	Adult HGBCL, NOS
BCL-29	chr6	167304012	167304012	C	A	exonic	UNC93A	54	27	0.3333	Missense_Mutation	p.P240H	Non-Targeted Panel Gene	Adult HGBCL, NOS
BCL-29	chr7	64521861	64521861	T	G	exonic	ZNF680	6	3	0.3333	Missense_Mutation	p.E298A	Non-Targeted Panel Gene	Adult HGBCL, NOS
BCL-29	chr19	43718910	43718910	C	T	exonic	IRGC	167	88	0.3451	Missense_Mutation	p.P118S	Non-Targeted Panel Gene	Adult HGBCL, NOS
BCL-29	chr3	62874606	62874606	C	T	exonic	CADPS	30	16	0.3478	Missense_Mutation	p.A142T	Non-Targeted Panel Gene	Adult HGBCL, NOS
BCL-29	chr1	84144389	84144389	A	C	exonic	PRKACB	24	14	0.3684	Missense_Mutation	p.N10H	Non-Targeted Panel Gene	Adult HGBCL, NOS
BCL-29	chr11	123577513	123577513	C	T	exonic	GRAMD1B	65	39	0.375	Missense_Mutation	p.P17L	Non-Targeted Panel Gene	Adult HGBCL, NOS
BCL-29	chr5	90647501	90647501	C	A	exonic	ADGRV1	81	49	0.3769	Missense_Mutation	p.P1009H	Non-Targeted Panel Gene	Adult HGBCL, NOS
BCL-29	chr12	109409623	109409623	G	T	exonic	MYO1H	43	27	0.3857	Missense_Mutation	p.G392C	Non-Targeted Panel Gene	Adult HGBCL, NOS
BCL-29	chr3	107377662	107377662	A	C	exonic	CCDC54	27	17	0.3864	Missense_Mutation	p.R25S	Non-Targeted Panel Gene	Adult HGBCL, NOS
BCL-29	chr5	140822127	140822127	G	C	exonic	PCDHA5	87	58	0.4	Missense_Mutation	p.V118L	Non-Targeted Panel Gene	Adult HGBCL, NOS
BCL-29	chr1	66332609	66332609	G	A	exonic	PDE4B	67	45	0.4018	Missense_Mutation	p.A246T	Non-Targeted Panel Gene	Adult HGBCL, NOS
BCL-29	chr20	10408774	10408774	T	C	exonic	MKKS	25	17	0.4048	Missense_Mutation	p.I339V	Non-Targeted Panel Gene	Adult HGBCL, NOS
BCL-29	chr12	14771051	14771051	C	G	exonic	HIST4H4	45	31	0.4079	Missense_Mutation	p.G12R	Non-Targeted Panel Gene	Adult HGBCL, NOS
BCL-29	chr11	9574341	9574341	C	G	exonic	WEE1	24	17	0.4146	Missense_Mutation	p.S136R	Non-Targeted Panel Gene	Adult HGBCL, NOS
BCL-29	chr10	104142341	104142341	C	A	exonic	CFAP43	25	18	0.4186	Missense_Mutation	p.R1404I	Non-Targeted Panel Gene	Adult HGBCL, NOS
BCL-29	chr8	51409225	51409225	G	A	exonic	PXDNL	135	98	0.4206	Missense_Mutation	p.T800M	Non-Targeted Panel Gene	Adult HGBCL, NOS
BCL-29	chr16	75478880	75478880	C	T	exonic	CHST6	183	134	0.4227	Missense_Mutation	p.E317K	Non-Targeted Panel Gene	Adult HGBCL, NOS
BCL-29	chr10	43644333	43644333	C	T	exonic	ZNF32	93	69	0.4259	Missense_Mutation	p.R180K	Non-Targeted Panel Gene	Adult HGBCL, NOS
BCL-29	chr9	122228355	122228355	C	T	exonic	LHX6	24	18	0.4286	Missense_Mutation	p.M1I	Non-Targeted Panel Gene	Adult HGBCL, NOS
BCL-29	chr9	87887441	87887441	G	A	exonic	SPATA31E1	165	124	0.4291	Missense_Mutation	p.R985K	Non-Targeted Panel Gene	Adult HGBCL, NOS
BCL-29	chr10	116096759	116096759	C	T	exonic	GFRA1	61	46	0.4299	Missense_Mutation	p.R259H	Non-Targeted Panel Gene	Adult HGBCL, NOS
BCL-29	chr11	24729204	24729204	A	G	exonic	LUZP2	49	37	0.4302	Missense_Mutation	p.E33G	Non-Targeted Panel Gene	Adult HGBCL, NOS
BCL-29	chr1	247423943	247423943	G	A	exonic	NLRP3	129	98	0.4317	Missense_Mutation	p.R167H	Non-Targeted Panel Gene	Adult HGBCL, NOS
BCL-29	chr2	68775221	68775221	C	T	exonic	ARHGAP25	125	95	0.4318	Missense_Mutation	p.A21V	Non-Targeted Panel Gene	Adult HGBCL, NOS
BCL-29	chr2	73450660	73450660	C	T	exonic	ALMS1	71	54	0.432	Missense_Mutation	p.S1378F	Non-Targeted Panel Gene	Adult HGBCL, NOS
BCL-29	chr11	6214533	6214533	G	A	exonic	FAM160A2	46	35	0.4321	Missense_Mutation	p.A826V	Non-Targeted Panel Gene	Adult HGBCL, NOS
BCL-29	chr7	34811855	34811855	T	C	exonic	NPSR1	63	48	0.4324	Missense_Mutation	p.L157P	Non-Targeted Panel Gene	Adult HGBCL, NOS
BCL-29	chr1	84650522	84650522	C	T	exonic	SSX2IP	38	29	0.4328	Missense_Mutation	p.D504N	Non-Targeted Panel Gene	Adult HGBCL, NOS
BCL-29	chr20	3671615	3671615	A	G	exonic	ADAM33	115	91	0.4417	Missense_Mutation	p.L624P	Non-Targeted Panel Gene	Adult HGBCL, NOS
BCL-29	chr1	34988591	34988591	C	T	exonic	ZMYM6	24	19	0.4419	Missense_Mutation	p.A831T	Non-Targeted Panel Gene	Adult HGBCL, NOS
BCL-29	chr3	42941289	42941289	C	A	exonic	KRBOX1	77	61	0.442	Missense_Mutation	p.P34T	Non-Targeted Panel Gene	Adult HGBCL, NOS
BCL-29	chr4	118352513	118352513	G	A	exonic	PRSS12	50	40	0.4444	Missense_Mutation	p.R70W	Non-Targeted Panel Gene	Adult HGBCL, NOS
BCL-29	chr1	190099109	190099109	T	A	exonic	BRINP3	67	54	0.4463	Missense_Mutation	p.I404F	Non-Targeted Panel Gene	Adult HGBCL, NOS
BCL-29	chr9	70108286	70108286	C	T	exonic	MAMDC2	57	46	0.4466	Missense_Mutation	p.A75V	Non-Targeted Panel Gene	Adult HGBCL, NOS
BCL-29	chr18	35689258	35689258	T	C	exonic	GALNT1	41	34	0.4474	Missense_Mutation	p.W316R	Non-Targeted Panel Gene	Adult HGBCL, NOS
BCL-29	chr17	32487771	32487771	C	T	exonic	CDK5R1	154	125	0.448	Missense_Mutation	p.P51S	Non-Targeted Panel Gene	Adult HGBCL, NOS
BCL-29	chr6	70156197	70156197	G	T	exonic	COL19A1	80	65	0.4483	Missense_Mutation	p.G717V	Non-Targeted Panel Gene	Adult HGBCL, NOS
BCL-29	chr17	18249143	18249143	T	A	exonic	FLII	39	32	0.4507	Missense_Mutation	p.T640S	Non-Targeted Panel Gene	Adult HGBCL, NOS
BCL-29	chr6	41805911	41805911	C	A	exonic	USP49	197	162	0.4513	Missense_Mutation	p.C358F	Non-Targeted Panel Gene	Adult HGBCL, NOS
BCL-29	chr15	99130087	99130087	A	G	exonic	SYNM	183	153	0.4554	Missense_Mutation	p.E576G	Non-Targeted Panel Gene	Adult HGBCL, NOS
BCL-29	chr4	159353650	159353650	G	T	exonic	RAPGEF2	98	82	0.4556	Missense_Mutation	p.D1444Y	Non-Targeted Panel Gene	Adult HGBCL, NOS
BCL-29	chr7	107937149	107937149	T	C	exonic	LAMB1	41	35	0.4605	Missense_Mutation	p.N1297S	Non-Targeted Panel Gene	Adult HGBCL, NOS
BCL-29	chr17	58358526	58358526	C	T	exonic	RNF43	81	70	0.4636	Missense_Mutation	p.G417E	Non-Targeted Panel Gene	Adult HGBCL, NOS
BCL-29	chr6	31356882	31356882	C	T	exonic	HLA-B	37	32	0.4638	Missense_Mutation	p.G50D	Non-Targeted Panel Gene	Adult HGBCL, NOS
BCL-29	chr15	44657156	44657156	C	T	exonic	SPG11	77	67	0.4653	Missense_Mutation	p.V270I	Non-Targeted Panel Gene	Adult HGBCL, NOS
BCL-29	chr2	12718438	12718438	G	A	exonic	TRIB2	120	106	0.469	Missense_Mutation	p.G44D	Non-Targeted Panel Gene	Adult HGBCL, NOS
BCL-29	chr2	232486228	232486228	G	T	exonic	ECEL1	27	24	0.4706	Missense_Mutation	p.D142E	Non-Targeted Panel Gene	Adult HGBCL, NOS
BCL-29	chr15	43239030	43239030	C	T	exonic	TGM5	100	91	0.4764	Missense_Mutation	p.V378I	Non-Targeted Panel Gene	Adult HGBCL, NOS
BCL-29	chr14	26472376	26472376	G	A	exonic	NOVA1	61	56	0.4786	Missense_Mutation	p.P155S	Non-Targeted Panel Gene	Adult HGBCL, NOS
BCL-29	chr11	10803249	10803249	T	C	exonic	EIF4G2	25	23	0.4792	Missense_Mutation	p.S287G	Non-Targeted Panel Gene	Adult HGBCL, NOS
BCL-29	chr6	19838139	19838139	G	A	exonic	ID4	59	55	0.4825	Missense_Mutation	p.G129R	Non-Targeted Panel Gene	Adult HGBCL, NOS
BCL-29	chr9	37746702	37746702	A	G	exonic	FRMPD1	145	137	0.4858	Missense_Mutation	p.Q1557R	Non-Targeted Panel Gene	Adult HGBCL, NOS
BCL-29	chr3	122284583	122284583	G	A	exonic	CASR	148	141	0.4879	Missense_Mutation	p.A887T	Non-Targeted Panel Gene	Adult HGBCL, NOS
BCL-29	chr12	8838341	8838341	C	A	exonic	A2ML1	31	30	0.4918	Missense_Mutation	p.D287E	Non-Targeted Panel Gene	Adult HGBCL, NOS
BCL-29	chr11	66424983	66424983	C	A	exonic	NPAS4	140	136	0.4928	Missense_Mutation	p.A698D	Non-Targeted Panel Gene	Adult HGBCL, NOS
BCL-29	chr8	142701696	142701696	G	A	exonic	LY6K	73	71	0.4931	Missense_Mutation	p.C67Y	Non-Targeted Panel Gene	Adult HGBCL, NOS
BCL-29	chr20	16379115	16379115	C	T	exonic	KIF16B	43	42	0.4941	Missense_Mutation	p.A963T	Non-Targeted Panel Gene	Adult HGBCL, NOS
BCL-29	chr15	31484093	31484093	G	A	exonic	OTUD7A	45	45	0.5	Missense_Mutation	p.A661V	Non-Targeted Panel Gene	Adult HGBCL, NOS
BCL-29	chr12	10847212	10847212	G	A	exonic	PRR4	84	85	0.5	Missense_Mutation	p.P86S	Non-Targeted Panel Gene	Adult HGBCL, NOS

BCL-29	chr7	64526478	64526478	A	T	exonic	ZNF680	12	12	0.5	Missense_Mutation	p.V98D	Non-Targeted Panel Gene	Adult HGBCL, NOS
BCL-29	chr2	240895634	240895634	G	A	exonic	C2orf54	43	45	0.5114	Missense_Mutation	p.R122W	Non-Targeted Panel Gene	Adult HGBCL, NOS
BCL-29	chr15	73323445	73323445	G	C	exonic	HCN4	41	43	0.5119	Missense_Mutation	p.P883R	Non-Targeted Panel Gene	Adult HGBCL, NOS
BCL-29	chr15	40955535	40955535	G	C	exonic	CHAC1	160	168	0.5122	Missense_Mutation	p.V186L	Non-Targeted Panel Gene	Adult HGBCL, NOS
BCL-29	chr5	171309516	171309516	C	T	exonic	TLX3	55	58	0.5133	Missense_Mutation	p.R51C	Non-Targeted Panel Gene	Adult HGBCL, NOS
BCL-29	chr14	24262228	24262228	G	T	exonic	TGM1	110	118	0.5175	Missense_Mutation	p.S42Y	Non-Targeted Panel Gene	Adult HGBCL, NOS
BCL-29	chr16	30724027	30724027	C	G	exonic	SRCAP	37	40	0.5195	Missense_Mutation	p.P1535A	Non-Targeted Panel Gene	Adult HGBCL, NOS
BCL-29	chr3	31981133	31981133	C	T	exonic	OSBPL10	8	9	0.5294	Missense_Mutation	p.S16N	Non-Targeted Panel Gene	Adult HGBCL, NOS
BCL-29	chr3	31981139	31981139	C	A	exonic	OSBPL10	8	9	0.5294	Missense_Mutation	p.S14I	Non-Targeted Panel Gene	Adult HGBCL, NOS
BCL-29	chr4	13577426	13577426	G	A	exonic	BOD1L1	19	22	0.5366	Missense_Mutation	p.S2954F	Non-Targeted Panel Gene	Adult HGBCL, NOS
BCL-29	chr18	24014873	24014873	T	C	exonic	TTC39C	18	22	0.55	Missense_Mutation	p.M1T	Non-Targeted Panel Gene	Adult HGBCL, NOS
BCL-29	chr8	142478331	142478331	C	T	exonic	ADGRB1	22	27	0.551	Missense_Mutation	p.T511I	Non-Targeted Panel Gene	Adult HGBCL, NOS
BCL-29	chr3	31980995	31980995	G	A	exonic	OSBPL10	16	21	0.5676	Missense_Mutation	p.A62V	Non-Targeted Panel Gene	Adult HGBCL, NOS
BCL-29	chr1	156861050	156861050	C	A	exonic	NTRK1	22	30	0.5769	Missense_Mutation	p.A39D	Non-Targeted Panel Gene	Adult HGBCL, NOS
BCL-29	chr3	31981143	31981143	C	A	exonic	OSBPL10	6	9	0.6	Missense_Mutation	p.G13C	Non-Targeted Panel Gene	Adult HGBCL, NOS
BCL-29	chr12	123034538	123034538	C	T	exonic	PITPNM2	45	87	0.6591	Missense_Mutation	p.R18H	Non-Targeted Panel Gene	Adult HGBCL, NOS
BCL-29	chr19	15458581	15458581	A	G	exonic	RASAL3	30	108	0.7826	Missense_Mutation	p.V246A	Non-Targeted Panel Gene	Adult HGBCL, NOS
BCL-29	chr19	7520368	7520368	C	T	exonic	ZNF358	6	39	0.8667	Missense_Mutation	p.R376C	Non-Targeted Panel Gene	Adult HGBCL, NOS
BCL-29	chrX	105220368	105220368	G	T	exonic	TEX13A	0	22	1	Missense_Mutation	p.S10R	Non-Targeted Panel Gene	Adult HGBCL, NOS
BCL-29	chr2	178014454	178014454	G	A	exonic	PDE11A	36	15	0.2941	Nonsense_Mutation	p.R57X	Non-Targeted Panel Gene	Adult HGBCL, NOS
BCL-29	chr9	132678511	132678511	G	T	exonic	GTF3C4	75	45	0.375	Nonsense_Mutation	p.E298X	Non-Targeted Panel Gene	Adult HGBCL, NOS
BCL-29	chr12	27780432	27780432	C	A	exonic	KLHL42	34	23	0.4035	Nonsense_Mutation	p.Y34X	Non-Targeted Panel Gene	Adult HGBCL, NOS
BCL-29	chr10	19692464	19692464	G	A	exonic	MALRD1	28	20	0.4167	Nonsense_Mutation	p.W2075X	Non-Targeted Panel Gene	Adult HGBCL, NOS
BCL-29	chr6	31356707	31356707	G	C	exonic	HLA-B	48	38	0.4419	Nonsense_Mutation	p.Y108X	Non-Targeted Panel Gene	Adult HGBCL, NOS
BCL-29	chr6	26032003	26032003	G	A	exonic	HIST1H3B	46	47	0.5054	Nonsense_Mutation	p.Q20X	Non-Targeted Panel Gene	Adult HGBCL, NOS
BCL-29	chr3	38141150	38141150	T	C	exonic	MYD88	417	256	0.3793	Nonstop_Mutation	p.L265P	Targerted Panel Gene	Adult HGBCL, NOS
BCL-3	chr15	22786835	22786835	G	A	splicing	NIPA1	22	2	0.0833	Splice_Site	.	Non-Targeted Panel Gene	Adult HGBCL, NOS
BCL-3	chr5	148088541	148088541	G	T	splicing	SPINK5	46	5	0.098	Splice_Site	.	Non-Targeted Panel Gene	Adult HGBCL, NOS
BCL-3	chr11	72088296	72088296	G	A	splicing	LRTOMT	56	21	0.2727	Splice_Site	.	Non-Targeted Panel Gene	Adult HGBCL, NOS
BCL-3	chr9	117039967	117039967	T	C	splicing	ASTN2	67	69	0.5074	Splice_Site	.	Non-Targeted Panel Gene	Adult HGBCL, NOS
BCL-3	chr12	109561518	109561518	C	T	splicing	MMAB	9	17	0.6538	Splice_Site	.	Non-Targeted Panel Gene	Adult HGBCL, NOS
BCL-3	chr4	73420257	73420257	A	-	exonic	ALB	18	5	0.2174	Frame_Shift_Del	p.K597fs	Non-Targeted Panel Gene	Adult HGBCL, NOS
BCL-3	chr14	92652183	92652183	G	-	exonic	RIN3	56	33	0.3708	Frame_Shift_Del	p.A378fs	Non-Targeted Panel Gene	Adult HGBCL, NOS
BCL-3	chr13	20189256	20189269	CCCTTGATGAACTT	-	exonic	GJB2	83	63	0.4315	Frame_Shift_Del	p.K105fs	Non-Targeted Panel Gene	Adult HGBCL, NOS
BCL-3	chr11	45927574	45927575	TC	-	exonic	LARGE2	84	68	0.4474	Frame_Shift_Del	p.S498fs	Non-Targeted Panel Gene	Adult HGBCL, NOS
BCL-3	chr16	71626645	71626653	AGCCGCCGC	-	exonic	MARVELD3	9	6	0.4	In_Frame_Del	p.139_142del	Non-Targeted Panel Gene	Adult HGBCL, NOS
BCL-3	chr5	180620672	180620672	-	CCG	exonic	FLT4	68	72	0.507	In_Frame_Ins	p.G781delinsGG	Non-Targeted Panel Gene	Adult HGBCL, NOS
BCL-3	chr4	174977861	174977861	C	G	exonic	ADAM29	184	166	0.4716	Missense_Mutation	p.P779R	Targerted Panel Gene	Adult HGBCL, NOS
BCL-3	chr15	44711548	44711548	T	A	exonic	B2M	32	108	0.7714	Missense_Mutation	p.M1K	Targerted Panel Gene	Adult HGBCL, NOS
BCL-3	chr1	7738328	7738328	G	C	exonic	CAMTA1	153	117	0.4301	Missense_Mutation	p.S1343T	Targerted Panel Gene	Adult HGBCL, NOS
BCL-3	chr7	2944519	2944519	C	T	exonic	CARD11	72	56	0.4375	Missense_Mutation	p.G126D	Targerted Panel Gene	Adult HGBCL, NOS
BCL-3	chr7	2939861	2939861	A	G	exonic	CARD11	222	170	0.4326	Missense_Mutation	p.L251P	Targerted Panel Gene	Adult HGBCL, NOS
BCL-3	chr19	19149316	19149316	C	G	exonic	MEF2B,MEF2BNB-I	199	168	0.4565	Missense_Mutation	p.Q56H	Targerted Panel Gene	Adult HGBCL, NOS
BCL-3	chr6	37171459	37171459	T	A	exonic	PIM1	147	142	0.4913	Missense_Mutation	p.L192Q	Targerted Panel Gene	Adult HGBCL, NOS
BCL-3	chr10	87933038	87933038	T	A	exonic	PTEN	216	172	0.4433	Missense_Mutation	p.H266Q	Targerted Panel Gene	Adult HGBCL, NOS
BCL-3	chr3	38140801	38140801	G	A	exonic	MYD88	149	131	0.4679	Missense_Mutation	p.S243N	Targerted Panel Gene	Adult HGBCL, NOS
BCL-3	chr17	81645337	81645337	G	A	exonic	TSPAN10	58	2	0.0333	Missense_Mutation	p.G166R	Non-Targeted Panel Gene	Adult HGBCL, NOS
BCL-3	chr12	76030855	76030855	C	T	exonic	PHLDA1	50	2	0.0385	Missense_Mutation	p.R296Q	Non-Targeted Panel Gene	Adult HGBCL, NOS
BCL-3	chr16	30783220	30783220	G	A	exonic	ZNF629	72	3	0.04	Missense_Mutation	p.R356C	Non-Targeted Panel Gene	Adult HGBCL, NOS
BCL-3	chr6	30589944	30589944	G	A	exonic	ABCF1	45	2	0.0426	Missense_Mutation	p.A735T	Non-Targeted Panel Gene	Adult HGBCL, NOS
BCL-3	chr20	34746915	34746915	G	A	exonic	NCOA6	44	2	0.0435	Missense_Mutation	p.R936C	Non-Targeted Panel Gene	Adult HGBCL, NOS
BCL-3	chr11	66337753	66337753	C	T	exonic	BRMS1	43	2	0.0444	Missense_Mutation	p.C263Y	Non-Targeted Panel Gene	Adult HGBCL, NOS
BCL-3	chr9	16552559	16552559	G	A	exonic	BNC2	190	9	0.0452	Missense_Mutation	p.R214W	Non-Targeted Panel Gene	Adult HGBCL, NOS
BCL-3	chr17	28042943	28042943	G	A	exonic	NLK	39	2	0.0488	Missense_Mutation	p.A24T	Non-Targeted Panel Gene	Adult HGBCL, NOS
BCL-3	chr9	111649526	111649526	C	A	exonic	DNAJC25	38	2	0.05	Missense_Mutation	p.A188D	Non-Targeted Panel Gene	Adult HGBCL, NOS
BCL-3	chrX	153954924	153954924	C	T	exonic	HCFC1	38	2	0.05	Missense_Mutation	p.A1159T	Non-Targeted Panel Gene	Adult HGBCL, NOS
BCL-3	chr1	205529529	205529529	C	T	exonic	CDK18	65	4	0.058	Missense_Mutation	p.T426M	Non-Targeted Panel Gene	Adult HGBCL, NOS
BCL-3	chr22	50467686	50467686	G	A	exonic	SBF1	48	3	0.0588	Missense_Mutation	p.T95M	Non-Targeted Panel Gene	Adult HGBCL, NOS
BCL-3	chr9	124860233	124860233	G	A	exonic	RPL35	31	2	0.0606	Missense_Mutation	p.L58F	Non-Targeted Panel Gene	Adult HGBCL, NOS
BCL-3	chr4	792501	792501	C	T	exonic	CPLX1	30	2	0.0625	Missense_Mutation	p.E47K	Non-Targeted Panel Gene	Adult HGBCL, NOS
BCL-3	chr11	62787247	62787247	C	T	exonic	TAF6L	44	3	0.0638	Missense_Mutation	p.P607L	Non-Targeted Panel Gene	Adult HGBCL, NOS
BCL-3	chr2	97805113	97805113	C	T	exonic	TMEM131	56	4	0.0667	Missense_Mutation	p.G793R	Non-Targeted Panel Gene	Adult HGBCL, NOS
BCL-3	chr13	21168361	21168361	A	G	exonic	SKA3	106	9	0.0783	Missense_Mutation	p.S124P	Non-Targeted Panel Gene	Adult HGBCL, NOS
BCL-3	chrX	117910004	117910004	C	A	exonic	KLHL13	68	6	0.0811	Missense_Mutation	p.K205N	Non-Targeted Panel Gene	Adult HGBCL, NOS
BCL-3	chr22	39662323	39662323	C	T	exonic	CACNA1I	31	3	0.0882	Missense_Mutation	p.P1087L	Non-Targeted Panel Gene	Adult HGBCL, NOS
BCL-3	chr8	140559485	140559485	C	T	exonic	AGO2	30	3	0.0909	Missense_Mutation	p.V234I	Non-Targeted Panel Gene	Adult HGBCL, NOS
BCL-3	chr4	121819529	121819529	G	A	exonic	CCNA2	30	3	0.0909	Missense_Mutation	p.T282I	Non-Targeted Panel Gene	Adult HGBCL, NOS
BCL-3	chr6	168308633	168308633	C	T	exonic	DACT2	40	4	0.0909	Missense_Mutation	p.G375D	Non-Targeted Panel Gene	Adult HGBCL, NOS
BCL-3	chr7	6653425	6653425	C	T	exonic	ZNF316	17	2	0.1053	Missense_Mutation	p.P610L	Non-Targeted Panel Gene	Adult HGBCL, NOS
BCL-3	chrX	67546229	67546229	T	G	exonic	AR	106	14	0.1167	Missense_Mutation	p.S361R	Non-Targeted Panel Gene	Adult HGBCL, NOS
BCL-3	chr6	135466004	135466004	C	T	exonic	AHI1	22	3	0.12	Missense_Mutation	p.E187K	Non-Targeted Panel Gene	Adult HGBCL, NOS
BCL-3	chr13	61412015	61412015	C	A	exonic	PCDH20	58	8	0.1212	Missense_Mutation	p.R695M	Non-Targeted Panel Gene	Adult HGBCL, NOS
BCL-3	chr19	56088546	56088546	G	A	exonic	ZNF787	20	3	0.1304	Missense_Mutation	p.A209V	Non-Targeted Panel Gene	Adult HGBCL, NOS
BCL-3	chr1	2586877	2586877	G	A	exonic	FAM213B	19	3	0.1364	Missense_Mutation	p.A28T	Non-Targeted Panel Gene	Adult HGBCL, NOS
BCL-3	chr18	8370925	8370925	G	T	exonic	PTPRM	19	3	0.1364	Missense_Mutation	p.E1030D	Non-Targeted Panel Gene	Adult HGBCL, NOS
BCL-3	chr8	97690979	97690979	C	T	exonic	MTDH	63	12	0.16	Missense_Mutation	p.T280I	Non-Targeted Panel Gene	Adult HGBCL, NOS
BCL-3	chr16	54285183	54285183	A	G	exonic	IRX3	26	5	0.1613	Missense_Mutation	p.L233P	Non-Targeted Panel Gene	Adult HGBCL, NOS
BCL-3	chrX	72262747	72262747	T	C	exonic	PIN4	71	14	0.1647	Missense_Mutation	p.L118P	Non-Targeted Panel Gene	Adult HGBCL, NOS
BCL-3	chr10	113590855	113590855	C	G	exonic	NRAP	150	31	0.1713	Missense_Mutation	p.G1560A	Non-Targeted Panel Gene	Adult HGBCL, NOS
BCL-3	chr19	19149316	19149316	C	G	exonic	BORCS8-MEF2B;M	69	15	0.1786	Missense_Mutation	p.Q56H	Non-Targeted Panel Gene	Adult HGBCL, NOS
BCL-3	chr6	499683	499683	T	G	exonic	EXOC2	23	5	0.1786	Missense_Mutation	p.K800Q	Non-Targeted Panel Gene	Adult HGBCL, NOS
BCL-3	chr2	79122054	79122054	G	T	exonic	REG1A	67	15	0.1807	Missense_Mutation	p.V84L	Non-Targeted Panel Gene	Adult HGBCL, NOS

BCL-3	chr6	33405368	33405368	G	A	exonic	KIFC1	83	19	0.1845	Missense_Mutation	p.G425R	Non-Targeted Panel Gene	Adult HGBCL, NOS
BCL-3	chr12	48783277	48783277	G	A	exonic	ADCY6	78	19	0.1939	Missense_Mutation	p.P53L	Non-Targeted Panel Gene	Adult HGBCL, NOS
BCL-3	chr17	65529987	65529987	G	A	exonic	AXIN2	54	13	0.194	Missense_Mutation	p.R841W	Non-Targeted Panel Gene	Adult HGBCL, NOS
BCL-3	chr1	158638195	158638195	C	A	exonic	SPTA1	45	11	0.1964	Missense_Mutation	p.S1676I	Non-Targeted Panel Gene	Adult HGBCL, NOS
BCL-3	chr1	55096621	55096621	C	G	exonic	USP24	16	4	0.2	Missense_Mutation	p.G1980R	Non-Targeted Panel Gene	Adult HGBCL, NOS
BCL-3	chr13	70107411	70107411	G	A	exonic	KLHL1	112	30	0.2113	Missense_Mutation	p.L97F	Non-Targeted Panel Gene	Adult HGBCL, NOS
BCL-3	chr19	36950133	36950133	T	C	exonic	ZNF568	55	15	0.2143	Missense_Mutation	p.I327T	Non-Targeted Panel Gene	Adult HGBCL, NOS
BCL-3	chrX	1466176	1466176	G	A	exonic	P2RY8	102	28	0.2154	Missense_Mutation	p.P128L	Non-Targeted Panel Gene	Adult HGBCL, NOS
BCL-3	chr1	28714334	28714334	G	T	exonic	GMEB1	111	36	0.2449	Missense_Mutation	p.S418I	Non-Targeted Panel Gene	Adult HGBCL, NOS
BCL-3	chrX	123417591	123417591	A	G	exonic	GRIA3	79	26	0.2476	Missense_Mutation	p.I564V	Non-Targeted Panel Gene	Adult HGBCL, NOS
BCL-3	chr12	123327957	123327957	C	T	exonic	SBNO1	24	8	0.25	Missense_Mutation	p.G455D	Non-Targeted Panel Gene	Adult HGBCL, NOS
BCL-3	chr1	156671888	156671888	C	A	exonic	NES	26	9	0.2571	Missense_Mutation	p.R767M	Non-Targeted Panel Gene	Adult HGBCL, NOS
BCL-3	chr17	4034256	4034256	G	A	exonic	ZZEF1	129	45	0.2586	Missense_Mutation	p.L2115F	Non-Targeted Panel Gene	Adult HGBCL, NOS
BCL-3	chr1	152111566	152111566	T	C	exonic	TCHH	18	7	0.28	Missense_Mutation	p.K551E	Non-Targeted Panel Gene	Adult HGBCL, NOS
BCL-3	chr10	73782407	73782407	T	G	exonic	CHCHD1	61	24	0.2824	Missense_Mutation	p.I70S	Non-Targeted Panel Gene	Adult HGBCL, NOS
BCL-3	chrX	87622212	87622212	A	G	exonic	KLHL4	17	7	0.2917	Missense_Mutation	p.E309G	Non-Targeted Panel Gene	Adult HGBCL, NOS
BCL-3	chr19	10565966	10565966	C	T	exonic	KRI1	6	3	0.3333	Missense_Mutation	p.V18M	Non-Targeted Panel Gene	Adult HGBCL, NOS
BCL-3	chr2	196201348	196201348	G	A	exonic	HECW2	29	15	0.3409	Missense_Mutation	p.P1550S	Non-Targeted Panel Gene	Adult HGBCL, NOS
BCL-3	chr1	145960333	145960333	G	C	exonic	ANKRD34A	36	19	0.3455	Missense_Mutation	p.T476S	Non-Targeted Panel Gene	Adult HGBCL, NOS
BCL-3	chr18	46577725	46577725	A	T	exonic	LOXHD1	120	69	0.3651	Missense_Mutation	p.V651E	Non-Targeted Panel Gene	Adult HGBCL, NOS
BCL-3	chr3	130574222	130574222	G	A	exonic	COL6A6	51	31	0.378	Missense_Mutation	p.A1082T	Non-Targeted Panel Gene	Adult HGBCL, NOS
BCL-3	chr3	122284634	122284634	G	A	exonic	CASR	88	54	0.3803	Missense_Mutation	p.V904I	Non-Targeted Panel Gene	Adult HGBCL, NOS
BCL-3	chr11	74976953	74976953	T	G	exonic	SPCS2	40	25	0.3846	Missense_Mutation	p.F197L	Non-Targeted Panel Gene	Adult HGBCL, NOS
BCL-3	chr4	109516552	109516552	T	C	exonic	SEC24B	39	26	0.4	Missense_Mutation	p.Y710H	Non-Targeted Panel Gene	Adult HGBCL, NOS
BCL-3	chr17	81935132	81935132	G	A	exonic	PYCR1	76	53	0.4077	Missense_Mutation	p.R112W	Non-Targeted Panel Gene	Adult HGBCL, NOS
BCL-3	chr16	3256340	3256340	G	T	exonic	MEFV	26	18	0.4091	Missense_Mutation	p.A83D	Non-Targeted Panel Gene	Adult HGBCL, NOS
BCL-3	chr19	6151716	6151716	G	A	exonic	ACSBG2	31	23	0.4259	Missense_Mutation	p.E103K	Non-Targeted Panel Gene	Adult HGBCL, NOS
BCL-3	chr5	72460390	72460390	C	G	exonic	ZNF366	59	44	0.4272	Missense_Mutation	p.E369D	Non-Targeted Panel Gene	Adult HGBCL, NOS
BCL-3	chr12	102912801	102912801	C	T	exonic	PAH	49	37	0.4302	Missense_Mutation	p.R53H	Non-Targeted Panel Gene	Adult HGBCL, NOS
BCL-3	chr3	129411168	129411168	C	T	exonic	EFCAB12	13	10	0.4348	Missense_Mutation	p.R342Q	Non-Targeted Panel Gene	Adult HGBCL, NOS
BCL-3	chr22	23744138	23744138	G	A	exonic	ZNF70	48	39	0.4483	Missense_Mutation	p.P335S	Non-Targeted Panel Gene	Adult HGBCL, NOS
BCL-3	chr11	36594147	36594147	C	T	exonic	RAG2	28	23	0.451	Missense_Mutation	p.V8I	Non-Targeted Panel Gene	Adult HGBCL, NOS
BCL-3	chrX	124056160	124056160	G	T	exonic	STAG2	65	55	0.4583	Missense_Mutation	p.C410F	Non-Targeted Panel Gene	Adult HGBCL, NOS
BCL-3	chr17	28534833	28534833	C	G	exonic	FOXN1	41	35	0.4605	Missense_Mutation	p.P421R	Non-Targeted Panel Gene	Adult HGBCL, NOS
BCL-3	chr20	63529711	63529711	T	C	exonic	PTK6	14	12	0.4615	Missense_Mutation	p.H394R	Non-Targeted Panel Gene	Adult HGBCL, NOS
BCL-3	chr3	154122056	154122056	T	G	exonic	ARHGEF26	23	20	0.4651	Missense_Mutation	p.S22A	Non-Targeted Panel Gene	Adult HGBCL, NOS
BCL-3	chr15	74446143	74446143	C	A	exonic	UBL7	40	35	0.4667	Missense_Mutation	p.G404C	Non-Targeted Panel Gene	Adult HGBCL, NOS
BCL-3	chr14	104887903	104887903	G	A	exonic	CEP170B	41	37	0.4744	Missense_Mutation	p.A1222T	Non-Targeted Panel Gene	Adult HGBCL, NOS
BCL-3	chr16	68822063	68822063	G	A	exonic	CDH1	54	49	0.4757	Missense_Mutation	p.A592T	Non-Targeted Panel Gene	Adult HGBCL, NOS
BCL-3	chr17	49796882	49796882	C	T	exonic	KAT7	58	54	0.4821	Missense_Mutation	p.S99L	Non-Targeted Panel Gene	Adult HGBCL, NOS
BCL-3	chr14	53951994	53951994	C	T	exonic	BMP4	77	72	0.4832	Missense_Mutation	p.A77T	Non-Targeted Panel Gene	Adult HGBCL, NOS
BCL-3	chr10	77993342	77993342	T	A	exonic	POLR3A	44	42	0.4884	Missense_Mutation	p.D881V	Non-Targeted Panel Gene	Adult HGBCL, NOS
BCL-3	chr9	128125404	128125404	T	C	exonic	PTGES2	36	35	0.493	Missense_Mutation	p.Q106R	Non-Targeted Panel Gene	Adult HGBCL, NOS
BCL-3	chr1	112711275	112711275	A	C	exonic	PPM1J	44	44	0.5	Missense_Mutation	p.M346R	Non-Targeted Panel Gene	Adult HGBCL, NOS
BCL-3	chr2	137276009	137276009	A	G	exonic	THSD7B	49	51	0.51	Missense_Mutation	p.K828R	Non-Targeted Panel Gene	Adult HGBCL, NOS
BCL-3	chr10	49738227	49738227	G	C	exonic	OGDHL	23	24	0.5106	Missense_Mutation	p.F785L	Non-Targeted Panel Gene	Adult HGBCL, NOS
BCL-3	chr13	66631279	66631279	C	T	exonic	PCDH9	91	95	0.5108	Missense_Mutation	p.D1091N	Non-Targeted Panel Gene	Adult HGBCL, NOS
BCL-3	chrX	108601436	108601436	G	T	exonic	COL4A5	36	38	0.5135	Missense_Mutation	p.K664N	Non-Targeted Panel Gene	Adult HGBCL, NOS
BCL-3	chr21	46432135	46432135	G	A	exonic	PCNT	27	30	0.5263	Missense_Mutation	p.A2891T	Non-Targeted Panel Gene	Adult HGBCL, NOS
BCL-3	chr22	29313555	29313555	G	A	exonic	RASL10A	41	47	0.5341	Missense_Mutation	p.P120S	Non-Targeted Panel Gene	Adult HGBCL, NOS
BCL-3	chr17	29631662	29631662	C	A	exonic	SSH2	44	53	0.5408	Missense_Mutation	p.D1178Y	Non-Targeted Panel Gene	Adult HGBCL, NOS
BCL-3	chr6	105373389	105373389	T	C	exonic	PREP	10	12	0.5455	Missense_Mutation	p.Q192R	Non-Targeted Panel Gene	Adult HGBCL, NOS
BCL-3	chr1	47282457	47282457	G	A	exonic	STIL	30	36	0.5455	Missense_Mutation	p.S332F	Non-Targeted Panel Gene	Adult HGBCL, NOS
BCL-3	chr19	8904998	8904998	G	T	exonic	MUC16	130	161	0.5533	Missense_Mutation	p.H12717N	Non-Targeted Panel Gene	Adult HGBCL, NOS
BCL-3	chr9	129719285	129719285	G	A	exonic	PRRX2	66	82	0.5541	Missense_Mutation	p.R105Q	Non-Targeted Panel Gene	Adult HGBCL, NOS
BCL-3	chr8	31639995	31639995	G	C	exonic	NRG1	24	31	0.5636	Missense_Mutation	p.R4P	Non-Targeted Panel Gene	Adult HGBCL, NOS
BCL-3	chr1	117896842	117896842	T	C	exonic	GDAP2	35	47	0.5732	Missense_Mutation	p.H315R	Non-Targeted Panel Gene	Adult HGBCL, NOS
BCL-3	chr12	52054803	52054803	G	A	exonic	NR4A1	19	26	0.5778	Missense_Mutation	p.G159S	Non-Targeted Panel Gene	Adult HGBCL, NOS
BCL-3	chr15	40900688	40900688	C	G	exonic	VPS18	70	99	0.5824	Missense_Mutation	p.R624G	Non-Targeted Panel Gene	Adult HGBCL, NOS
BCL-3	chr16	2148802	2148802	C	T	exonic	RAB26	4	6	0.6	Missense_Mutation	p.P7S	Non-Targeted Panel Gene	Adult HGBCL, NOS
BCL-3	chrX	19537779	19537779	G	A	exonic	SH3KBP1	66	3	0.0435	Nonsense_Mutation	p.R632X	Non-Targeted Panel Gene	Adult HGBCL, NOS
BCL-3	chr17	75620472	75620472	C	T	exonic	MYO15B	47	4	0.0784	Nonsense_Mutation	p.Q2483X	Non-Targeted Panel Gene	Adult HGBCL, NOS
BCL-3	chr17	42709916	42709916	C	A	exonic	EZH1	124	31	0.2	Nonsense_Mutation	p.E475X	Non-Targeted Panel Gene	Adult HGBCL, NOS
BCL-3	chr7	17796878	17796878	G	A	exonic	SNX13	25	7	0.2188	Nonsense_Mutation	p.R656X	Non-Targeted Panel Gene	Adult HGBCL, NOS
BCL-3	chr17	50684767	50684767	C	G	exonic	ABCC3	86	60	0.411	Nonsense_Mutation	p.S1391X	Non-Targeted Panel Gene	Adult HGBCL, NOS
BCL-3	chr12	80255165	80255165	C	T	exonic	OTOGL	18	14	0.4375	Nonsense_Mutation	p.Q514X	Non-Targeted Panel Gene	Adult HGBCL, NOS
BCL-3	chr20	34998373	34998373	C	T	exonic	MYH7B	57	69	0.5476	Nonsense_Mutation	p.Q1318X	Non-Targeted Panel Gene	Adult HGBCL, NOS
BCL-3	chr2	20283081	20283081	A	-	exonic	PUM2	15	21	0.5833	Nonsense_Mutation	p.L529X	Non-Targeted Panel Gene	Adult HGBCL, NOS
BCL-30	chr2	142885537	142885537	G	A	splicing	KYNU	43	28	0.3944	Splice_Site	.	Non-Targeted Panel Gene	Adult HGBCL, NOS
BCL-30	chr6	26508500	26508500	G	A	splicing	BTN1A1	102	84	0.4516	Splice_Site	.	Non-Targeted Panel Gene	Adult HGBCL, NOS
BCL-30	chr12	11650161	11650161	G	A	splicing	ETV6	46	51	0.5258	Splice_Site	.	Non-Targeted Panel Gene	Adult HGBCL, NOS
BCL-30	chr16	71982140	71982140	A	-	exonic	PKD1L3	129	53	0.2912	Frame_Shift_Del	p.T354fs	Non-Targeted Panel Gene	Adult HGBCL, NOS
BCL-30	chr7	94588716	94588717	TC	-	exonic	SGCE	29	15	0.3409	Frame_Shift_Del	p.Q394fs	Non-Targeted Panel Gene	Adult HGBCL, NOS
BCL-30	chr2	182928863	182928863	C	-	exonic	NCKAP1	70	47	0.4017	Frame_Shift_Del	p.C1003fs	Non-Targeted Panel Gene	Adult HGBCL, NOS
BCL-30	chr14	68790349	68790362	GAGCTGGTTCTGG	-	exonic	ZFP36L1	52	40	0.4348	Frame_Shift_Del	p.H132fs	Non-Targeted Panel Gene	Adult HGBCL, NOS
BCL-30	chr10	47350206	47350206	-	CACACCCGCACGG	exonic	RBP3	59	5	0.0781	Frame_Shift_Ins	p.L574fs	Non-Targeted Panel Gene	Adult HGBCL, NOS
BCL-30	chr12	6959949	6959949	-	C	exonic	PTPN6	33	15	0.3125	Frame_Shift_Ins	p.T462fs	Non-Targeted Panel Gene	Adult HGBCL, NOS
BCL-30	chr10	115519304	115519304	-	T	exonic	ATRNL1	14	9	0.3913	Frame_Shift_Ins	p.Q1232fs	Non-Targeted Panel Gene	Adult HGBCL, NOS
BCL-30	chr5	176578058	176578058	-	G	exonic	CDHR2	72	60	0.4545	Frame_Shift_Ins	p.W513fs	Non-Targeted Panel Gene	Adult HGBCL, NOS
BCL-30	chr1	2504031	2504031	-	A	exonic	PLCH2	33	32	0.4776	Frame_Shift_Ins	p.P1023fs	Non-Targeted Panel Gene	Adult HGBCL, NOS
BCL-30	chr6	4892224	4892224	-	CG	exonic	CDYL	53	55	0.5093	Frame_Shift_Ins	p.P179fs	Non-Targeted Panel Gene	Adult HGBCL, NOS
BCL-30	chr1	153261291	153261308	CTCCTCCGGTGGC	-	exonic	LOR	15	7	0.3182	In_Frame_Del	p.114_120del	Non-Targeted Panel Gene	Adult HGBCL, NOS

BCL-30	chr19	17778637	17778642	CCCCTC	-	exonic	FCHO1	17	12	0.4138	In_Frame_Del	p.460_462del	Non-Targeted Panel Gene	Adult HGBCL, NOS
BCL-30	chr5	177386232	177386252	GTCCCCAAGCTGCC	-	exonic	SLC34A1	64	48	0.4286	In_Frame_Del	p.91_97del	Non-Targeted Panel Gene	Adult HGBCL, NOS
BCL-30	chrX	12976796	12976798	AGC	-	exonic	TMSB4X	14	11	0.44	In_Frame_Del	p.40_41del	Non-Targeted Panel Gene	Adult HGBCL, NOS
BCL-30	chr19	507779	507779	-	CTG	exonic	TPGS1	48	16	0.25	In_Frame_Ins	p.L91delinsLL	Non-Targeted Panel Gene	Adult HGBCL, NOS
BCL-30	chr1	116544507	116544507	C	A	exonic	CD58	264	219	0.4534	Missense_Mutation	p.W56C	Targerted Panel Gene	Adult HGBCL, NOS
BCL-30	chr1	116544547	116544547	A	G	exonic	CD58	263	213	0.4475	Missense_Mutation	p.F43S	Targerted Panel Gene	Adult HGBCL, NOS
BCL-30	chr10	71793370	71793370	G	A	exonic	CDH23	329	327	0.4985	Missense_Mutation	p.D2148N	Targerted Panel Gene	Adult HGBCL, NOS
BCL-30	chr16	3729745	3729745	G	A	exonic	CREBBP	627	187	0.2297	Missense_Mutation	p.R1768C	Targerted Panel Gene	Adult HGBCL, NOS
BCL-30	chr15	48446750	48446750	C	T	exonic	FBN1	288	233	0.4472	Missense_Mutation	p.R1915H	Targerted Panel Gene	Adult HGBCL, NOS
BCL-30	chr1	240392559	240392559	A	C	exonic	FMN2	164	140	0.4605	Missense_Mutation	p.K1640T	Targerted Panel Gene	Adult HGBCL, NOS
BCL-30	chr6	26156648	26156648	C	G	exonic	HIST1H1E	414	311	0.4284	Missense_Mutation	p.S86R	Targerted Panel Gene	Adult HGBCL, NOS
BCL-30	chr22	22888214	22888214	C	T	exonic	IGLL5	137	104	0.4315	Missense_Mutation	p.P19S	Targerted Panel Gene	Adult HGBCL, NOS
BCL-30	chr22	22888234	22888234	T	C	exonic	IGLL5	112	91	0.4461	Missense_Mutation	p.S61P	Targerted Panel Gene	Adult HGBCL, NOS
BCL-30	chr2	140867655	140867655	A	C	exonic	LRP1B	314	255	0.4474	Missense_Mutation	p.V1505G	Targerted Panel Gene	Adult HGBCL, NOS
BCL-30	chr7	82955794	82955794	A	G	exonic	PCLO	324	349	0.5186	Missense_Mutation	p.L1720P	Targerted Panel Gene	Adult HGBCL, NOS
BCL-30	chr6	37171434	37171434	C	T	exonic	PIM1	283	228	0.4453	Missense_Mutation	p.L184F	Targerted Panel Gene	Adult HGBCL, NOS
BCL-30	chr6	37170778	37170778	G	A	exonic	PIM1	157	134	0.4605	Missense_Mutation	p.E30K	Targerted Panel Gene	Adult HGBCL, NOS
BCL-30	chr7	124892290	124892290	G	A	exonic	POT1	152	136	0.4706	Missense_Mutation	p.P34S	Targerted Panel Gene	Adult HGBCL, NOS
BCL-30	chr16	30981064	30981064	C	T	exonic	SETD1A	567	247	0.3034	Missense_Mutation	p.R1566W	Targerted Panel Gene	Adult HGBCL, NOS
BCL-30	chr3	177033005	177033005	G	T	exonic	TBL1XR1	42	499	0.9224	Missense_Mutation	p.S461Y	Targerted Panel Gene	Adult HGBCL, NOS
BCL-30	chr16	72794329	72794329	G	T	exonic	ZFHX3	829	399	0.3249	Missense_Mutation	p.L2785I	Targerted Panel Gene	Adult HGBCL, NOS
BCL-30	chr21	44457102	44457102	T	A	exonic	LRRC3	163	29	0.151	Missense_Mutation	p.L153Q	Non-Targeted Panel Gene	Adult HGBCL, NOS
BCL-30	chr19	55279677	55279677	G	A	exonic	HSPBP1	149	34	0.1858	Missense_Mutation	p.R24C	Non-Targeted Panel Gene	Adult HGBCL, NOS
BCL-30	chr3	52905266	52905266	C	G	exonic	SFMBT1	59	14	0.1918	Missense_Mutation	p.G824A	Non-Targeted Panel Gene	Adult HGBCL, NOS
BCL-30	chr19	31277811	31277811	T	A	exonic	TSHZ3	218	65	0.2289	Missense_Mutation	p.D661V	Non-Targeted Panel Gene	Adult HGBCL, NOS
BCL-30	chr16	81264677	81264677	C	T	exonic	BCO1	126	52	0.2921	Missense_Mutation	p.T170M	Non-Targeted Panel Gene	Adult HGBCL, NOS
BCL-30	chr12	85019717	85019717	T	C	exonic	TSPAN19	7	3	0.3	Missense_Mutation	p.D120G	Non-Targeted Panel Gene	Adult HGBCL, NOS
BCL-30	chr16	24823491	24823491	G	T	exonic	TNRC6A	205	91	0.3074	Missense_Mutation	p.R1858L	Non-Targeted Panel Gene	Adult HGBCL, NOS
BCL-30	chr1	158700522	158700522	A	C	exonic	OR6K2	66	30	0.3125	Missense_Mutation	p.V44G	Non-Targeted Panel Gene	Adult HGBCL, NOS
BCL-30	chr16	31224328	31224328	C	T	exonic	TRIM72	158	78	0.3305	Missense_Mutation	p.A336V	Non-Targeted Panel Gene	Adult HGBCL, NOS
BCL-30	chr1	152812520	152812520	T	C	exonic	LCE1B	50	25	0.3333	Missense_Mutation	p.L25P	Non-Targeted Panel Gene	Adult HGBCL, NOS
BCL-30	chr14	24189551	24189551	C	G	exonic	TM9SF1	62	32	0.3404	Missense_Mutation	p.G562A	Non-Targeted Panel Gene	Adult HGBCL, NOS
BCL-30	chr2	97759702	97759702	G	A	exonic	TMEM131	48	25	0.3425	Missense_Mutation	p.T1719I	Non-Targeted Panel Gene	Adult HGBCL, NOS
BCL-30	chr1	6554179	6554179	G	T	exonic	NOL9	19	10	0.3448	Missense_Mutation	p.H108Q	Non-Targeted Panel Gene	Adult HGBCL, NOS
BCL-30	chr9	4662378	4662378	G	A	exonic	PLPP6	17	9	0.3462	Missense_Mutation	p.M1I	Non-Targeted Panel Gene	Adult HGBCL, NOS
BCL-30	chr1	235441844	235441844	A	G	exonic	TBCE	20	11	0.3548	Missense_Mutation	p.K485R	Non-Targeted Panel Gene	Adult HGBCL, NOS
BCL-30	chr18	54587855	54587855	A	G	exonic	DYNAP	20	12	0.375	Missense_Mutation	p.Y3C	Non-Targeted Panel Gene	Adult HGBCL, NOS
BCL-30	chrX	11112065	11112065	G	A	exonic	HCCS	35	21	0.375	Missense_Mutation	p.G2D	Non-Targeted Panel Gene	Adult HGBCL, NOS
BCL-30	chr12	41016749	41016749	G	C	exonic	CNTN1	63	38	0.3762	Missense_Mutation	p.G751A	Non-Targeted Panel Gene	Adult HGBCL, NOS
BCL-30	chr20	44750695	44750695	G	A	exonic	KCNK15	39	24	0.381	Missense_Mutation	p.V284M	Non-Targeted Panel Gene	Adult HGBCL, NOS
BCL-30	chr5	177512379	177512379	G	A	exonic	DDX41	94	58	0.3816	Missense_Mutation	p.R522C	Non-Targeted Panel Gene	Adult HGBCL, NOS
BCL-30	chrX	53985846	53985846	T	C	exonic	PHF8	56	36	0.3913	Missense_Mutation	p.Q700R	Non-Targeted Panel Gene	Adult HGBCL, NOS
BCL-30	chr6	113953377	113953377	T	C	exonic	HDAC2	20	13	0.3939	Missense_Mutation	p.H180R	Non-Targeted Panel Gene	Adult HGBCL, NOS
BCL-30	chrX	48683918	48683918	T	C	exonic	WAS	110	72	0.3956	Missense_Mutation	p.I22T	Non-Targeted Panel Gene	Adult HGBCL, NOS
BCL-30	chr8	144333241	144333241	C	G	exonic	SCRT1	97	64	0.3975	Missense_Mutation	p.G331R	Non-Targeted Panel Gene	Adult HGBCL, NOS
BCL-30	chr12	11650160	11650160	G	C	exonic	ETV6	59	39	0.398	Missense_Mutation	p.K11N	Non-Targeted Panel Gene	Adult HGBCL, NOS
BCL-30	chr9	97854458	97854458	C	G	exonic	FOXE1	18	12	0.4	Missense_Mutation	p.P182A	Non-Targeted Panel Gene	Adult HGBCL, NOS
BCL-30	chr8	1501366	1501366	A	G	exonic	DLGAP2	98	66	0.4024	Missense_Mutation	p.E36G	Non-Targeted Panel Gene	Adult HGBCL, NOS
BCL-30	chr17	43074471	43074471	C	A	exonic	BRCA1	35	24	0.4068	Missense_Mutation	p.S1533I	Non-Targeted Panel Gene	Adult HGBCL, NOS
BCL-30	chr4	110622324	110622324	G	A	exonic	PITX2	49	35	0.4118	Missense_Mutation	p.S46L	Non-Targeted Panel Gene	Adult HGBCL, NOS
BCL-30	chr6	32443194	32443194	A	G	exonic	HLA-DRA	124	87	0.4123	Missense_Mutation	p.E113G	Non-Targeted Panel Gene	Adult HGBCL, NOS
BCL-30	chr3	38010410	38010410	G	T	exonic	PLCD1	108	76	0.413	Missense_Mutation	p.L315M	Non-Targeted Panel Gene	Adult HGBCL, NOS
BCL-30	chrX	48918549	48918549	G	A	exonic	PIM2	132	93	0.4133	Missense_Mutation	p.T53I	Non-Targeted Panel Gene	Adult HGBCL, NOS
BCL-30	chrX	105909269	105909269	A	C	exonic	NRK	156	110	0.4135	Missense_Mutation	p.N543T	Non-Targeted Panel Gene	Adult HGBCL, NOS
BCL-30	chr19	7520581	7520581	G	A	exonic	ZNF358	30	22	0.4231	Missense_Mutation	p.G447S	Non-Targeted Panel Gene	Adult HGBCL, NOS
BCL-30	chr5	179774265	179774265	G	C	exonic	MAML1	118	87	0.4244	Missense_Mutation	p.K813N	Non-Targeted Panel Gene	Adult HGBCL, NOS
BCL-30	chrX	106205482	106205482	C	A	exonic	MUM1L1	46	34	0.425	Missense_Mutation	p.A17E	Non-Targeted Panel Gene	Adult HGBCL, NOS
BCL-30	chr22	42619804	42619804	C	T	exonic	CYB5R3	35	26	0.4262	Missense_Mutation	p.G269D	Non-Targeted Panel Gene	Adult HGBCL, NOS
BCL-30	chr10	48963913	48963913	G	C	exonic	WDFY4	51	38	0.427	Missense_Mutation	p.Q2765H	Non-Targeted Panel Gene	Adult HGBCL, NOS
BCL-30	chrX	10213787	10213787	G	C	exonic	CLCN4	210	158	0.4293	Missense_Mutation	p.W561C	Non-Targeted Panel Gene	Adult HGBCL, NOS
BCL-30	chr6	168310281	168310281	G	A	exonic	DACT2	77	58	0.4296	Missense_Mutation	p.T182I	Non-Targeted Panel Gene	Adult HGBCL, NOS
BCL-30	chr2	214767641	214767641	T	C	exonic	BARD1	41	31	0.4306	Missense_Mutation	p.N470S	Non-Targeted Panel Gene	Adult HGBCL, NOS
BCL-30	chr12	93572964	93572964	G	A	exonic	SOCS2	91	69	0.4312	Missense_Mutation	p.A23T	Non-Targeted Panel Gene	Adult HGBCL, NOS
BCL-30	chr9	86945827	86945827	C	T	exonic	GAS1	21	16	0.4324	Missense_Mutation	p.S318N	Non-Targeted Panel Gene	Adult HGBCL, NOS
BCL-30	chr14	31107243	31107243	C	A	exonic	HECTD1	30	23	0.434	Missense_Mutation	p.G2210V	Non-Targeted Panel Gene	Adult HGBCL, NOS
BCL-30	chr2	60791804	60791804	G	A	exonic	PAPOLG	9	7	0.4375	Missense_Mutation	p.M480I	Non-Targeted Panel Gene	Adult HGBCL, NOS
BCL-30	chr13	20222886	20222886	A	T	exonic	GJB6	32	25	0.4386	Missense_Mutation	p.S199T	Non-Targeted Panel Gene	Adult HGBCL, NOS
BCL-30	chr2	178589692	178589692	G	T	exonic	TTN	48	38	0.4419	Missense_Mutation	p.A20678E	Non-Targeted Panel Gene	Adult HGBCL, NOS
BCL-30	chr12	91055542	91055542	T	C	exonic	KERA	49	39	0.4432	Missense_Mutation	p.N247S	Non-Targeted Panel Gene	Adult HGBCL, NOS
BCL-30	chr5	154927495	154927495	A	G	exonic	GEMIN5	35	28	0.4444	Missense_Mutation	p.F324L	Non-Targeted Panel Gene	Adult HGBCL, NOS
BCL-30	chr14	68790160	68790160	C	G	exonic	ZFP36L1	70	57	0.4488	Missense_Mutation	p.K199N	Non-Targeted Panel Gene	Adult HGBCL, NOS
BCL-30	chr2	84701254	84701254	G	T	exonic	DNAH6	40	33	0.4521	Missense_Mutation	p.R2659L	Non-Targeted Panel Gene	Adult HGBCL, NOS
BCL-30	chr19	419031	419031	T	C	exonic	SHC2	52	43	0.4526	Missense_Mutation	p.E549G	Non-Targeted Panel Gene	Adult HGBCL, NOS
BCL-30	chr10	82985266	82985266	A	T	exonic	NRG3	41	34	0.4533	Missense_Mutation	p.E387D	Non-Targeted Panel Gene	Adult HGBCL, NOS
BCL-30	chr12	113172530	113172530	T	G	exonic	DDX54	106	88	0.4536	Missense_Mutation	p.I368L	Non-Targeted Panel Gene	Adult HGBCL, NOS
BCL-30	chr18	6995389	6995389	C	A	exonic	LAMA1	24	20	0.4545	Missense_Mutation	p.V1622F	Non-Targeted Panel Gene	Adult HGBCL, NOS
BCL-30	chr20	20513191	20513191	C	G	exonic	RALGAPA2	86	72	0.4557	Missense_Mutation	p.G1393A	Non-Targeted Panel Gene	Adult HGBCL, NOS
BCL-30	chr12	71556624	71556624	T	A	exonic	LGR5	25	21	0.4565	Missense_Mutation	p.L217H	Non-Targeted Panel Gene	Adult HGBCL, NOS
BCL-30	chr9	133547119	133547119	C	T	exonic	ADAMTSL2	85	72	0.4586	Missense_Mutation	p.T282M	Non-Targeted Panel Gene	Adult HGBCL, NOS
BCL-30	chr9	97602476	97602476	A	C	exonic	TSTD2	33	28	0.459	Missense_Mutation	p.L515R	Non-Targeted Panel Gene	Adult HGBCL, NOS
BCL-30	chr13	25170887	25170887	C	T	exonic	AMER2	20	17	0.4595	Missense_Mutation	p.A245T	Non-Targeted Panel Gene	Adult HGBCL, NOS
BCL-30	chr5	124744478	124744478	C	A	exonic	ZNF608	27	23	0.46	Missense_Mutation	p.S171I	Non-Targeted Panel Gene	Adult HGBCL, NOS

BCL-30	chr2	17931111	17931111	C	T	exonic	KCNS3	122	104	0.4602	Missense_Mutation	p.R35W	Non-Targeted Panel Gene	Adult HGBCL, NOS
BCL-30	chr8	124486731	124486731	C	T	exonic	RNF139	43	37	0.4625	Missense_Mutation	p.T361I	Non-Targeted Panel Gene	Adult HGBCL, NOS
BCL-30	chr5	124744752	124744752	C	G	exonic	ZNF608	41	36	0.4675	Missense_Mutation	p.A80P	Non-Targeted Panel Gene	Adult HGBCL, NOS
BCL-30	chr17	45266583	45266583	C	G	exonic	MAP3K14	97	86	0.4699	Missense_Mutation	p.M844I	Non-Targeted Panel Gene	Adult HGBCL, NOS
BCL-30	chr4	158868850	158868850	G	T	exonic	FNIP2	142	126	0.4701	Missense_Mutation	p.M738I	Non-Targeted Panel Gene	Adult HGBCL, NOS
BCL-30	chr2	42719057	42719057	G	T	exonic	MTA3	37	33	0.4714	Missense_Mutation	p.C475F	Non-Targeted Panel Gene	Adult HGBCL, NOS
BCL-30	chr20	13221898	13221898	G	A	exonic	ISM1	10	9	0.4737	Missense_Mutation	p.S41N	Non-Targeted Panel Gene	Adult HGBCL, NOS
BCL-30	chr5	143298691	143298691	A	T	exonic	NR3C1	40	36	0.4737	Missense_Mutation	p.F623L	Non-Targeted Panel Gene	Adult HGBCL, NOS
BCL-30	chr15	25701999	25701999	G	T	exonic	ATP10A	73	66	0.4748	Missense_Mutation	p.Q893K	Non-Targeted Panel Gene	Adult HGBCL, NOS
BCL-30	chr11	60292287	60292287	C	A	exonic	MS4A4A	45	41	0.4767	Missense_Mutation	p.P16H	Non-Targeted Panel Gene	Adult HGBCL, NOS
BCL-30	chr21	30497070	30497070	C	T	exonic	KRTAP19-4	107	98	0.478	Missense_Mutation	p.G14D	Non-Targeted Panel Gene	Adult HGBCL, NOS
BCL-30	chr7	88760014	88760014	G	A	exonic	ZNF804B	111	102	0.4789	Missense_Mutation	p.S13N	Non-Targeted Panel Gene	Adult HGBCL, NOS
BCL-30	chr14	73712877	73712877	G	C	exonic	PNMA1	102	94	0.4796	Missense_Mutation	p.Q255E	Non-Targeted Panel Gene	Adult HGBCL, NOS
BCL-30	chr12	55942029	55942029	C	T	exonic	DGKA	104	96	0.48	Missense_Mutation	p.R428W	Non-Targeted Panel Gene	Adult HGBCL, NOS
BCL-30	chr7	73597463	73597463	G	C	exonic	MLXIPL	13	12	0.48	Missense_Mutation	p.P441R	Non-Targeted Panel Gene	Adult HGBCL, NOS
BCL-30	chr2	178608393	178608393	T	G	exonic	TTN	40	37	0.4805	Missense_Mutation	p.N17497T	Non-Targeted Panel Gene	Adult HGBCL, NOS
BCL-30	chr1	109266174	109266174	C	T	exonic	CELSR2	54	50	0.4808	Missense_Mutation	p.P1994L	Non-Targeted Panel Gene	Adult HGBCL, NOS
BCL-30	chr12	102844348	102844348	A	T	exonic	PAH	27	25	0.4808	Missense_Mutation	p.F351L	Non-Targeted Panel Gene	Adult HGBCL, NOS
BCL-30	chr5	6739745	6739745	G	A	exonic	PAPD7	69	64	0.4812	Missense_Mutation	p.V51M	Non-Targeted Panel Gene	Adult HGBCL, NOS
BCL-30	chr10	62091595	62091595	G	T	exonic	ARID5B	99	93	0.4844	Missense_Mutation	p.G711V	Non-Targeted Panel Gene	Adult HGBCL, NOS
BCL-30	chr1	207049600	207049600	G	C	exonic	YOD1	66	62	0.4844	Missense_Mutation	p.A112G	Non-Targeted Panel Gene	Adult HGBCL, NOS
BCL-30	chr17	65534045	65534045	C	T	exonic	AXIN2	46	44	0.4889	Missense_Mutation	p.A758T	Non-Targeted Panel Gene	Adult HGBCL, NOS
BCL-30	chr11	44309981	44309981	G	A	exonic	ALX4	75	72	0.4898	Missense_Mutation	p.R28W	Non-Targeted Panel Gene	Adult HGBCL, NOS
BCL-30	chr2	173364984	173364984	A	G	exonic	CDCA7	56	54	0.4909	Missense_Mutation	p.M297V	Non-Targeted Panel Gene	Adult HGBCL, NOS
BCL-30	chr1	145961595	145961595	G	C	exonic	ANKRD34A	87	85	0.4942	Missense_Mutation	p.N55K	Non-Targeted Panel Gene	Adult HGBCL, NOS
BCL-30	chr19	18131938	18131938	A	T	exonic	MAST3	46	45	0.4945	Missense_Mutation	p.M459L	Non-Targeted Panel Gene	Adult HGBCL, NOS
BCL-30	chrX	106205856	106205856	C	T	exonic	MUM1L1	50	49	0.4949	Missense_Mutation	p.P142S	Non-Targeted Panel Gene	Adult HGBCL, NOS
BCL-30	chr11	124310511	124310511	G	T	exonic	OR8D1	57	56	0.4956	Missense_Mutation	p.L86I	Non-Targeted Panel Gene	Adult HGBCL, NOS
BCL-30	chr4	148435963	148435963	G	A	exonic	NR3C2	120	118	0.4958	Missense_Mutation	p.P300S	Non-Targeted Panel Gene	Adult HGBCL, NOS
BCL-30	chr10	62091444	62091444	G	C	exonic	ARID5B	126	126	0.5	Missense_Mutation	p.D661H	Non-Targeted Panel Gene	Adult HGBCL, NOS
BCL-30	chr9	35736073	35736073	C	G	exonic	CREB3	63	63	0.5	Missense_Mutation	p.L213V	Non-Targeted Panel Gene	Adult HGBCL, NOS
BCL-30	chr2	232486521	232486521	C	A	exonic	ECEL1	9	9	0.5	Missense_Mutation	p.A45S	Non-Targeted Panel Gene	Adult HGBCL, NOS
BCL-30	chr3	105702188	105702188	C	G	exonic	CBLB	80	81	0.5031	Missense_Mutation	p.S226T	Non-Targeted Panel Gene	Adult HGBCL, NOS
BCL-30	chrX	85330991	85330991	A	T	exonic	POF1B	49	50	0.5051	Missense_Mutation	p.L271Q	Non-Targeted Panel Gene	Adult HGBCL, NOS
BCL-30	chr17	70132650	70132650	C	T	exonic	KCNJ16	65	67	0.5076	Missense_Mutation	p.A223V	Non-Targeted Panel Gene	Adult HGBCL, NOS
BCL-30	chr14	74407587	74407587	A	T	exonic	SYNDIG1L	96	99	0.5077	Missense_Mutation	p.V222E	Non-Targeted Panel Gene	Adult HGBCL, NOS
BCL-30	chr7	149278469	149278469	G	C	exonic	ZNF783	30	31	0.5082	Missense_Mutation	p.Q248H	Non-Targeted Panel Gene	Adult HGBCL, NOS
BCL-30	chr7	14149220	14149220	T	A	exonic	DGKB	55	57	0.5089	Missense_Mutation	p.N734Y	Non-Targeted Panel Gene	Adult HGBCL, NOS
BCL-30	chr12	109783719	109783719	C	T	exonic	TRPV4	67	70	0.5109	Missense_Mutation	p.E840K	Non-Targeted Panel Gene	Adult HGBCL, NOS
BCL-30	chr1	32226210	32226210	G	A	exonic	EIF3I	87	91	0.5112	Missense_Mutation	p.R97Q	Non-Targeted Panel Gene	Adult HGBCL, NOS
BCL-30	chr1	56876075	56876075	A	T	exonic	C8A	120	129	0.5181	Missense_Mutation	p.K110N	Non-Targeted Panel Gene	Adult HGBCL, NOS
BCL-30	chr2	173364904	173364904	T	C	exonic	CDCA7	53	57	0.5182	Missense_Mutation	p.L270P	Non-Targeted Panel Gene	Adult HGBCL, NOS
BCL-30	chr1	66332610	66332610	C	G	exonic	PDE4B	50	54	0.5192	Missense_Mutation	p.A246G	Non-Targeted Panel Gene	Adult HGBCL, NOS
BCL-30	chr6	49551378	49551378	C	T	exonic	C6orf141	147	165	0.5272	Missense_Mutation	p.R196C	Non-Targeted Panel Gene	Adult HGBCL, NOS
BCL-30	chr3	42686309	42686309	C	T	exonic	KLHL40	59	67	0.5317	Missense_Mutation	p.R231C	Non-Targeted Panel Gene	Adult HGBCL, NOS
BCL-30	chrX	118825354	118825354	T	A	exonic	ZCCHC12	87	99	0.5323	Missense_Mutation	p.M37K	Non-Targeted Panel Gene	Adult HGBCL, NOS
BCL-30	chr6	144803071	144803071	G	A	exonic	UTRN	26	30	0.5357	Missense_Mutation	p.C3094Y	Non-Targeted Panel Gene	Adult HGBCL, NOS
BCL-30	chr13	110210015	110210015	C	A	exonic	COL4A1	64	75	0.5396	Missense_Mutation	p.G194C	Non-Targeted Panel Gene	Adult HGBCL, NOS
BCL-30	chr2	209817006	209817006	T	C	exonic	UNC80	60	71	0.542	Missense_Mutation	p.L478P	Non-Targeted Panel Gene	Adult HGBCL, NOS
BCL-30	chr17	38329654	38329654	C	A	exonic	GPR179	34	42	0.5526	Missense_Mutation	p.M1305I	Non-Targeted Panel Gene	Adult HGBCL, NOS
BCL-30	chr5	38406234	38406234	T	C	exonic	EGFLAM	62	77	0.554	Missense_Mutation	p.F274S	Non-Targeted Panel Gene	Adult HGBCL, NOS
BCL-30	chr15	98649664	98649664	C	G	exonic	IGF1R	27	35	0.5645	Missense_Mutation	p.T28R	Non-Targeted Panel Gene	Adult HGBCL, NOS
BCL-30	chr12	107319772	107319772	C	G	exonic	BTBD11	23	30	0.566	Missense_Mutation	p.P278A	Non-Targeted Panel Gene	Adult HGBCL, NOS
BCL-30	chr5	55956036	55956036	C	T	exonic	IL6ST	13	17	0.5667	Missense_Mutation	p.C419Y	Non-Targeted Panel Gene	Adult HGBCL, NOS
BCL-30	chr9	92186757	92186757	C	T	exonic	PRSS47	65	86	0.5695	Missense_Mutation	p.E203K	Non-Targeted Panel Gene	Adult HGBCL, NOS
BCL-30	chr20	44624224	44624224	G	C	exonic	ADA	43	57	0.57	Missense_Mutation	p.P195R	Non-Targeted Panel Gene	Adult HGBCL, NOS
BCL-30	chr11	76976304	76976304	C	A	exonic	ACER3	6	8	0.5714	Missense_Mutation	p.P95T	Non-Targeted Panel Gene	Adult HGBCL, NOS
BCL-30	chr11	64766112	64766112	C	T	exonic	SF1	7	10	0.5882	Missense_Mutation	p.A541T	Non-Targeted Panel Gene	Adult HGBCL, NOS
BCL-30	chrX	130402256	130402256	C	T	exonic	RBMX2	3	5	0.625	Missense_Mutation	p.P3S	Non-Targeted Panel Gene	Adult HGBCL, NOS
BCL-30	chr19	37635467	37635467	T	A	exonic	ZFP30	19	54	0.7397	Missense_Mutation	p.E358D	Non-Targeted Panel Gene	Adult HGBCL, NOS
BCL-30	chr6	137878706	137878706	C	-	exonic	TNFAIP3	416	338	0.4483	Nonsense_Mutation	p.L421X	Targerted Panel Gene	Adult HGBCL, NOS
BCL-30	chr17	7670685	7670685	G	A	exonic	TP53	14	106	0.8833	Nonsense_Mutation	p.R303X	Targerted Panel Gene	Adult HGBCL, NOS
BCL-30	chr5	72875696	72875696	C	T	exonic	TNPO1	22	10	0.3125	Nonsense_Mutation	p.R246X	Non-Targeted Panel Gene	Adult HGBCL, NOS
BCL-30	chr2	157443940	157443940	G	C	exonic	CYTIP	60	39	0.3939	Nonsense_Mutation	p.Y27X	Non-Targeted Panel Gene	Adult HGBCL, NOS
BCL-30	chr2	163610148	163610148	C	A	exonic	FIGN	73	60	0.4511	Nonsense_Mutation	p.E562X	Non-Targeted Panel Gene	Adult HGBCL, NOS
BCL-30	chr20	61923462	61923462	C	A	exonic	CDH4	105	94	0.4724	Nonsense_Mutation	p.Y462X	Non-Targeted Panel Gene	Adult HGBCL, NOS
BCL-30	chr9	35612930	35612930	G	T	exonic	CD72	36	35	0.493	Nonsense_Mutation	p.S251X	Non-Targeted Panel Gene	Adult HGBCL, NOS
BCL-30	chr3	38141150	38141150	T	C	exonic	MYD88	299	255	0.4595	Nonstop_Mutation	p.L265P	Targerted Panel Gene	Adult HGBCL, NOS
BCL-30	chr2	37253178	37253178	T	A	exonic	PRKD3	44	34	0.4359	Nonstop_Mutation	p.X891L	Non-Targeted Panel Gene	Adult HGBCL, NOS
BCL-31	chr11	111379160	111379160	A	C	splicing	POU2AF1	200	95	0.322	Splice_Site	.	Targerted Panel Gene	Adult HGBCL, NOS
BCL-31	chr4	25662614	25662614	T	G	splicing	SLC34A2	170	18	0.0957	Splice_Site	.	Non-Targeted Panel Gene	Adult HGBCL, NOS
BCL-31	chr3	108454143	108454143	C	A	splicing	MYH15	44	8	0.1538	Splice_Site	.	Non-Targeted Panel Gene	Adult HGBCL, NOS
BCL-31	chr4	146707552	146707552	C	A	splicing	TTC29	18	9	0.3333	Splice_Site	.	Non-Targeted Panel Gene	Adult HGBCL, NOS
BCL-31	chr1	2557827	2557831	CAGTC	-	exonic	TNFRSF14	79	12	0.1319	Frame_Shift_Del	p.C57fs	Targerted Panel Gene	Adult HGBCL, NOS
BCL-31	chr12	49049858	49049858	C	-	exonic	KMT2D	274	120	0.3046	Frame_Shift_Del	p.V1244fs	Targerted Panel Gene	Adult HGBCL, NOS
BCL-31	chr2	70816719	70816719	T	-	exonic	CLEC4F	34	28	0.4516	Frame_Shift_Del	p.N221fs	Non-Targeted Panel Gene	Adult HGBCL, NOS
BCL-31	chr4	6301570	6301571	TG	-	exonic	WFS1	39	114	0.7451	Frame_Shift_Del	p.L592fs	Non-Targeted Panel Gene	Adult HGBCL, NOS
BCL-31	chr14	91462162	91462162	-	A	exonic	PPP4R3A	15	5	0.25	Frame_Shift_Ins	p.N671fs	Non-Targeted Panel Gene	Adult HGBCL, NOS
BCL-31	chr7	100112246	100112248	CTC	-	exonic	TAF6	52	24	0.3158	In_Frame_Del	p.194_194del	Non-Targeted Panel Gene	Adult HGBCL, NOS
BCL-31	chr13	108210145	108210147	TTA	-	exonic	LIG4	17	12	0.4138	In_Frame_Del	p.374_375del	Non-Targeted Panel Gene	Adult HGBCL, NOS
BCL-31	chr9	129994934	129994934	-	TTT	exonic	FNBP1	19	10	0.3333	In_Frame_Ins	p.H17delinsKH	Non-Targeted Panel Gene	Adult HGBCL, NOS
BCL-31	chr4	78776612	78776612	-	GGCGGGGCC	exonic	BMP2K	9	12	0.5217	In_Frame_Ins	p.A23delinsAGGA	Non-Targeted Panel Gene	Adult HGBCL, NOS

BCL-31	chr1	203307377	203307377	A	G	exonic	BTG2	184	70	0.2756	Missense_Mutation	p.N139S	Targerted Panel Gene	Adult HGBCL, NOS
BCL-31	chr1	203307262	203307262	A	G	exonic	BTG2	204	91	0.3064	Missense_Mutation	p.T101A	Targerted Panel Gene	Adult HGBCL, NOS
BCL-31	chr7	2945909	2945909	C	A	exonic	CARD11	348	151	0.3026	Missense_Mutation	p.V90F	Targerted Panel Gene	Adult HGBCL, NOS
BCL-31	chr16	3738605	3738605	A	T	exonic	CREBBP	149	235	0.612	Missense_Mutation	p.Y1450N	Targerted Panel Gene	Adult HGBCL, NOS
BCL-31	chr8	113278639	113278639	G	A	exonic	CSMD3	307	102	0.2494	Missense_Mutation	p.T116I	Targerted Panel Gene	Adult HGBCL, NOS
BCL-31	chr4	125446343	125446343	G	T	exonic	FAT4	350	157	0.3091	Missense_Mutation	p.G2415V	Targerted Panel Gene	Adult HGBCL, NOS
BCL-31	chr3	50256271	50256271	A	G	exonic	GNAI2	253	83	0.247	Missense_Mutation	p.T166A	Targerted Panel Gene	Adult HGBCL, NOS
BCL-31	chr6	26271050	26271050	G	A	exonic	HIST1H3G	50	27	0.3506	Missense_Mutation	p.A112V	Targerted Panel Gene	Adult HGBCL, NOS
BCL-31	chr22	22888259	22888259	G	C	exonic	IGLL5	111	26	0.1884	Missense_Mutation	p.G34R	Targerted Panel Gene	Adult HGBCL, NOS
BCL-31	chr12	49034475	49034475	T	C	exonic	KMT2D	232	117	0.3352	Missense_Mutation	p.E3481G	Targerted Panel Gene	Adult HGBCL, NOS
BCL-31	chr7	83155362	83155362	G	T	exonic	PCLO	322	179	0.3573	Missense_Mutation	p.L427I	Targerted Panel Gene	Adult HGBCL, NOS
BCL-31	chr19	11013030	11013030	A	G	exonic	SMARCA4	356	146	0.2908	Missense_Mutation	p.T786A	Targerted Panel Gene	Adult HGBCL, NOS
BCL-31	chr1	2557825	2557825	T	G	exonic	TNFRSF14	75	12	0.1379	Missense_Mutation	p.C57G	Targerted Panel Gene	Adult HGBCL, NOS
BCL-31	chr11	130110644	130110644	C	A	exonic	APLP2	67	4	0.0563	Missense_Mutation	p.T129K	Non-Targeted Panel Gene	Adult HGBCL, NOS
BCL-31	chrX	71294297	71294297	G	A	exonic	NONO	122	8	0.0615	Missense_Mutation	p.R140H	Non-Targeted Panel Gene	Adult HGBCL, NOS
BCL-31	chr16	983736	983736	G	A	exonic	SOX8	102	10	0.0893	Missense_Mutation	p.S144N	Non-Targeted Panel Gene	Adult HGBCL, NOS
BCL-31	chr2	178663287	178663287	G	A	exonic	TTN	70	7	0.0909	Missense_Mutation	p.P12227S	Non-Targeted Panel Gene	Adult HGBCL, NOS
BCL-31	chr1	2408856	2408856	A	T	exonic	PEX10	201	28	0.1223	Missense_Mutation	p.Y66N	Non-Targeted Panel Gene	Adult HGBCL, NOS
BCL-31	chr9	137013480	137013480	C	T	exonic	ABCA2	40	6	0.1304	Missense_Mutation	p.E1541K	Non-Targeted Panel Gene	Adult HGBCL, NOS
BCL-31	chr3	53186644	53186644	A	G	exonic	PRKCD	322	53	0.1413	Missense_Mutation	p.D434G	Non-Targeted Panel Gene	Adult HGBCL, NOS
BCL-31	chr1	1332310	1332310	T	A	exonic	TAS1R3	29	5	0.1471	Missense_Mutation	p.L260Q	Non-Targeted Panel Gene	Adult HGBCL, NOS
BCL-31	chr13	94403042	94403042	G	A	exonic	GPC6	115	33	0.223	Missense_Mutation	p.G498D	Non-Targeted Panel Gene	Adult HGBCL, NOS
BCL-31	chr10	22209637	22209637	A	T	exonic	EBLN1	72	21	0.2258	Missense_Mutation	p.L116H	Non-Targeted Panel Gene	Adult HGBCL, NOS
BCL-31	chr20	49025374	49025374	T	C	exonic	ARFGEF2	56	18	0.2432	Missense_Mutation	p.M1606T	Non-Targeted Panel Gene	Adult HGBCL, NOS
BCL-31	chr16	65004673	65004673	T	G	exonic	CDH11	62	22	0.2619	Missense_Mutation	p.Y66S	Non-Targeted Panel Gene	Adult HGBCL, NOS
BCL-31	chr4	134200100	134200100	T	G	exonic	PABPC4L	79	29	0.2685	Missense_Mutation	p.E365A	Non-Targeted Panel Gene	Adult HGBCL, NOS
BCL-31	chr15	43775829	43775829	G	C	exonic	ELL3	92	34	0.2698	Missense_Mutation	p.Q126E	Non-Targeted Panel Gene	Adult HGBCL, NOS
BCL-31	chr20	9580488	9580488	T	G	exonic	PAK5	78	29	0.271	Missense_Mutation	p.K216T	Non-Targeted Panel Gene	Adult HGBCL, NOS
BCL-31	chr8	19418738	19418738	A	G	exonic	CSGALNACT1	64	26	0.2889	Missense_Mutation	p.F382S	Non-Targeted Panel Gene	Adult HGBCL, NOS
BCL-31	chr21	44940104	44940104	T	C	exonic	FAM207A	33	14	0.2979	Missense_Mutation	p.V16A	Non-Targeted Panel Gene	Adult HGBCL, NOS
BCL-31	chr2	207555673	207555673	G	A	exonic	CREB1	63	27	0.3	Missense_Mutation	p.G13E	Non-Targeted Panel Gene	Adult HGBCL, NOS
BCL-31	chr15	41457818	41457818	A	G	exonic	RTF1	39	17	0.3036	Missense_Mutation	p.K202E	Non-Targeted Panel Gene	Adult HGBCL, NOS
BCL-31	chr17	11669485	11669485	A	G	exonic	DNAH9	222	97	0.3041	Missense_Mutation	p.Y1015C	Non-Targeted Panel Gene	Adult HGBCL, NOS
BCL-31	chr12	119713568	119713568	A	T	exonic	CIT	178	79	0.3074	Missense_Mutation	p.F1463I	Non-Targeted Panel Gene	Adult HGBCL, NOS
BCL-31	chr10	100990486	100990486	A	G	exonic	TWNK	74	33	0.3084	Missense_Mutation	p.H512R	Non-Targeted Panel Gene	Adult HGBCL, NOS
BCL-31	chr7	151074468	151074468	A	T	exonic	SLC4A2	137	63	0.315	Missense_Mutation	p.I954F	Non-Targeted Panel Gene	Adult HGBCL, NOS
BCL-31	chr2	106843670	106843670	T	C	exonic	ST6GAL2	78	36	0.3158	Missense_Mutation	p.Q103R	Non-Targeted Panel Gene	Adult HGBCL, NOS
BCL-31	chr20	48632627	48632627	A	G	exonic	PREX1	211	99	0.3194	Missense_Mutation	p.F1427S	Non-Targeted Panel Gene	Adult HGBCL, NOS
BCL-31	chr3	109308065	109308065	C	T	exonic	DPPA2	36	18	0.3333	Missense_Mutation	p.V209I	Non-Targeted Panel Gene	Adult HGBCL, NOS
BCL-31	chr7	838885	838885	T	G	exonic	SUN1	85	45	0.3462	Missense_Mutation	p.S76R	Non-Targeted Panel Gene	Adult HGBCL, NOS
BCL-31	chr10	61902183	61902183	C	T	exonic	ARID5B	30	16	0.3478	Missense_Mutation	p.H16Y	Non-Targeted Panel Gene	Adult HGBCL, NOS
BCL-31	chr4	62070201	62070201	G	C	exonic	ADGRL3	35	20	0.3636	Missense_Mutation	p.E1303Q	Non-Targeted Panel Gene	Adult HGBCL, NOS
BCL-31	chr8	31154721	31154721	C	G	exonic	WRN	17	10	0.3704	Missense_Mutation	p.T1262R	Non-Targeted Panel Gene	Adult HGBCL, NOS
BCL-31	chr7	47978712	47978712	T	G	exonic	HUS1	131	83	0.3879	Missense_Mutation	p.S53R	Non-Targeted Panel Gene	Adult HGBCL, NOS
BCL-31	chr6	158071666	158071666	G	A	exonic	SYNJ2	158	101	0.39	Missense_Mutation	p.G669S	Non-Targeted Panel Gene	Adult HGBCL, NOS
BCL-31	chr9	129994940	129994940	C	G	exonic	FNBP1	18	12	0.4	Missense_Mutation	p.E15Q	Non-Targeted Panel Gene	Adult HGBCL, NOS
BCL-31	chr6	75862605	75862605	A	G	exonic	MYO6	42	28	0.4	Missense_Mutation	p.E519G	Non-Targeted Panel Gene	Adult HGBCL, NOS
BCL-31	chr5	129016047	129016047	G	A	exonic	SLC27A6	26	19	0.4222	Missense_Mutation	p.G378R	Non-Targeted Panel Gene	Adult HGBCL, NOS
BCL-31	chr11	116790222	116790222	A	G	exonic	APOA5	163	124	0.4321	Missense_Mutation	p.V336A	Non-Targeted Panel Gene	Adult HGBCL, NOS
BCL-31	chr17	16940352	16940352	C	T	exonic	TNFRSF13B	60	48	0.4444	Missense_Mutation	p.R202H	Non-Targeted Panel Gene	Adult HGBCL, NOS
BCL-31	chr6	28995060	28995060	A	T	exonic	ZNF311	91	73	0.4451	Missense_Mutation	p.S600T	Non-Targeted Panel Gene	Adult HGBCL, NOS
BCL-31	chr19	39423028	39423028	T	A	exonic	PLEKHG2	88	71	0.4465	Missense_Mutation	p.S658R	Non-Targeted Panel Gene	Adult HGBCL, NOS
BCL-31	chr9	130458549	130458549	G	T	exonic	ASS1	16	13	0.4483	Missense_Mutation	p.R108L	Non-Targeted Panel Gene	Adult HGBCL, NOS
BCL-31	chr19	57720899	57720899	G	A	exonic	ZNF671	48	39	0.4483	Missense_Mutation	p.P319L	Non-Targeted Panel Gene	Adult HGBCL, NOS
BCL-31	chr6	132552721	132552721	T	G	exonic	TAAR8	35	29	0.4531	Missense_Mutation	p.V10G	Non-Targeted Panel Gene	Adult HGBCL, NOS
BCL-31	chr2	70816177	70816177	C	G	exonic	CLEC4F	61	51	0.4554	Missense_Mutation	p.A402P	Non-Targeted Panel Gene	Adult HGBCL, NOS
BCL-31	chr2	178720104	178720104	G	C	exonic	TTN	51	44	0.4632	Missense_Mutation	p.F7846L	Non-Targeted Panel Gene	Adult HGBCL, NOS
BCL-31	chr21	38814899	38814899	C	G	exonic	ETS2	209	189	0.4737	Missense_Mutation	p.N281K	Non-Targeted Panel Gene	Adult HGBCL, NOS
BCL-31	chr2	113193901	113193901	C	A	exonic	PSD4	119	109	0.4781	Missense_Mutation	p.L712M	Non-Targeted Panel Gene	Adult HGBCL, NOS
BCL-31	chr11	88509224	88509224	C	T	exonic	GRM5	37	34	0.4789	Missense_Mutation	p.V1003M	Non-Targeted Panel Gene	Adult HGBCL, NOS
BCL-31	chr9	14808038	14808038	C	T	exonic	FREM1	43	42	0.4941	Missense_Mutation	p.G997E	Non-Targeted Panel Gene	Adult HGBCL, NOS
BCL-31	chr5	151277584	151277584	G	A	exonic	SLC36A3	138	135	0.4945	Missense_Mutation	p.L449F	Non-Targeted Panel Gene	Adult HGBCL, NOS
BCL-31	chr10	89206643	89206643	C	A	exonic	CH25H	171	168	0.4956	Missense_Mutation	p.G217V	Non-Targeted Panel Gene	Adult HGBCL, NOS
BCL-31	chr9	32572946	32572946	T	C	exonic	NDUFB6	32	32	0.5	Missense_Mutation	p.K39E	Non-Targeted Panel Gene	Adult HGBCL, NOS
BCL-31	chr2	9488319	9488319	G	A	exonic	IAH1	53	54	0.5047	Missense_Mutation	p.G133E	Non-Targeted Panel Gene	Adult HGBCL, NOS
BCL-31	chr14	58645935	58645935	G	T	exonic	DACT1	140	144	0.507	Missense_Mutation	p.G438C	Non-Targeted Panel Gene	Adult HGBCL, NOS
BCL-31	chr11	118203534	118203534	T	G	exonic	JAML	89	104	0.5389	Missense_Mutation	p.Q222H	Non-Targeted Panel Gene	Adult HGBCL, NOS
BCL-31	chr20	3147035	3147035	A	C	exonic	FASTKD5	86	102	0.5426	Missense_Mutation	p.M679R	Non-Targeted Panel Gene	Adult HGBCL, NOS
BCL-31	chr11	58955788	58955788	G	A	exonic	GLYATL1	36	45	0.5556	Missense_Mutation	p.D224N	Non-Targeted Panel Gene	Adult HGBCL, NOS
BCL-31	chr7	150397317	150397317	G	A	exonic	ZNF775	40	52	0.5652	Missense_Mutation	p.R279H	Non-Targeted Panel Gene	Adult HGBCL, NOS
BCL-31	chr9	23692664	23692664	T	C	exonic	ELAVL2	38	52	0.5778	Missense_Mutation	p.T353A	Non-Targeted Panel Gene	Adult HGBCL, NOS
BCL-31	chr16	724734	724734	T	G	exonic	CCDC78	21	85	0.8019	Missense_Mutation	p.K238Q	Non-Targeted Panel Gene	Adult HGBCL, NOS
BCL-31	chr4	7786252	7786252	T	C	exonic	AFAP1	16	91	0.8505	Missense_Mutation	p.Y491C	Non-Targeted Panel Gene	Adult HGBCL, NOS
BCL-31	chrX	74741775	74741775	G	A	exonic	KIAA2022	0	80	1	Missense_Mutation	p.L928F	Non-Targeted Panel Gene	Adult HGBCL, NOS
BCL-31	chr12	50352196	50352196	G	A	exonic	FAM186A	213	121	0.358	Nonsense_Mutation	p.Q1546X	Targerted Panel Gene	Adult HGBCL, NOS
BCL-31	chr12	49031861	49031861	G	A	exonic	KMT2D	278	117	0.2962	Nonsense_Mutation	p.R4282X	Targerted Panel Gene	Adult HGBCL, NOS
BCL-31	chr3	165030719	165030719	C	T	exonic	SI	17	4	0.1905	Nonsense_Mutation	p.W962X	Non-Targeted Panel Gene	Adult HGBCL, NOS
BCL-31	chr5	159099385	159099385	G	A	exonic	EBF1	42	18	0.3	Nonsense_Mutation	p.Q32X	Non-Targeted Panel Gene	Adult HGBCL, NOS
BCL-32	chr5	111120995	111120995	G	A	splicing	WDR36	20	10	0.3333	Splice_Site	.	Non-Targeted Panel Gene	Adult HGBCL, NOS
BCL-32	chr3	180659457	180659458	CA	-	exonic	CCDC39	52	5	0.0877	Frame_Shift_Del	p.C244fs	Non-Targeted Panel Gene	Adult HGBCL, NOS
BCL-32	chr3	17009705	17009708	AGAC	-	exonic	PLCL2	40	14	0.2593	Frame_Shift_Del	p.K120fs	Non-Targeted Panel Gene	Adult HGBCL, NOS
BCL-32	chr13	72775296	72775296	T	G	exonic	DIS3	252	122	0.3262	Missense_Mutation	p.Q301P	Targerted Panel Gene	Adult HGBCL, NOS

BCL-32	chr3	65621993	65621993	G	T	exonic	MAGI1	383	282	0.4241	Missense_Mutation	p.L137I	Targerted Panel Gene	Adult HGBCL, NOS
BCL-32	chr19	19149236	19149236	T	A	exonic	MEF2B,MEF2BNB-I	242	145	0.3747	Missense_Mutation	p.D83V	Targerted Panel Gene	Adult HGBCL, NOS
BCL-32	chr6	37170999	37170999	G	C	exonic	PIM1	310	159	0.339	Missense_Mutation	p.E70Q	Targerted Panel Gene	Adult HGBCL, NOS
BCL-32	chr6	106088409	106088409	C	A	exonic	PRDM1	382	214	0.3591	Missense_Mutation	p.P84Q	Targerted Panel Gene	Adult HGBCL, NOS
BCL-32	chr7	103535440	103535440	G	A	exonic	RELN	309	154	0.3319	Missense_Mutation	p.P2409S	Targerted Panel Gene	Adult HGBCL, NOS
BCL-32	chr15	44711549	44711549	G	A	exonic	B2M	94	128	0.5766	Missense_Mutation	p.M1I	Targerted Panel Gene	Adult HGBCL, NOS
BCL-32	chr11	26443810	26443810	T	C	exonic	ANO3	65	2	0.0299	Missense_Mutation	p.F157S	Non-Targeted Panel Gene	Adult HGBCL, NOS
BCL-32	chr10	132404913	132404913	T	C	exonic	PWWP2B	53	3	0.0536	Missense_Mutation	p.L138P	Non-Targeted Panel Gene	Adult HGBCL, NOS
BCL-32	chr19	50668413	50668413	T	G	exonic	SHANK1	54	5	0.0847	Missense_Mutation	p.T1183P	Non-Targeted Panel Gene	Adult HGBCL, NOS
BCL-32	chr5	45353199	45353199	A	T	exonic	HCN1	34	7	0.1707	Missense_Mutation	p.D426E	Non-Targeted Panel Gene	Adult HGBCL, NOS
BCL-32	chr1	116768722	116768722	G	A	exonic	CD2	52	11	0.1746	Missense_Mutation	p.R332Q	Non-Targeted Panel Gene	Adult HGBCL, NOS
BCL-32	chr7	83961538	83961538	T	G	exonic	SEMA3A	69	27	0.2812	Missense_Mutation	p.T717P	Non-Targeted Panel Gene	Adult HGBCL, NOS
BCL-32	chr16	4875433	4875433	C	T	exonic	UBN1	74	30	0.2885	Missense_Mutation	p.S1008L	Non-Targeted Panel Gene	Adult HGBCL, NOS
BCL-32	chr19	758627	758627	G	A	exonic	MISP	112	47	0.2956	Missense_Mutation	p.V561M	Non-Targeted Panel Gene	Adult HGBCL, NOS
BCL-32	chr12	49295621	49295621	G	T	exonic	PRPH	137	64	0.3168	Missense_Mutation	p.D141Y	Non-Targeted Panel Gene	Adult HGBCL, NOS
BCL-32	chr15	90225767	90225767	G	A	exonic	SEMA4B	44	21	0.3231	Missense_Mutation	p.C543Y	Non-Targeted Panel Gene	Adult HGBCL, NOS
BCL-32	chr9	132987297	132987297	C	G	exonic	GFI1B	60	29	0.3258	Missense_Mutation	p.A39G	Non-Targeted Panel Gene	Adult HGBCL, NOS
BCL-32	chr5	74685474	74685474	C	T	exonic	HEXB	139	68	0.3285	Missense_Mutation	p.L72F	Non-Targeted Panel Gene	Adult HGBCL, NOS
BCL-32	chr1	43688998	43688998	G	A	exonic	KDM4A	162	82	0.3361	Missense_Mutation	p.R647Q	Non-Targeted Panel Gene	Adult HGBCL, NOS
BCL-32	chr19	46660902	46660902	C	A	exonic	DACT3	58	32	0.3516	Missense_Mutation	p.A55S	Non-Targeted Panel Gene	Adult HGBCL, NOS
BCL-32	chr6	155314376	155314376	A	C	exonic	TFB1M	148	86	0.3675	Missense_Mutation	p.I18S	Non-Targeted Panel Gene	Adult HGBCL, NOS
BCL-32	chr22	38682371	38682371	C	T	exonic	TOMM22	210	125	0.3731	Missense_Mutation	p.P56S	Non-Targeted Panel Gene	Adult HGBCL, NOS
BCL-32	chr20	33097628	33097628	G	C	exonic	BPIFB4	80	48	0.375	Missense_Mutation	p.Q470H	Non-Targeted Panel Gene	Adult HGBCL, NOS
BCL-32	chr2	218217471	218217471	A	T	exonic	ARPC2	81	50	0.3817	Missense_Mutation	p.M1L	Non-Targeted Panel Gene	Adult HGBCL, NOS
BCL-32	chr11	69810393	69810393	C	T	exonic	FGF3	58	36	0.383	Missense_Mutation	p.R211Q	Non-Targeted Panel Gene	Adult HGBCL, NOS
BCL-32	chr21	46649582	46649582	C	T	exonic	PRMT2	108	68	0.3864	Missense_Mutation	p.A166V	Non-Targeted Panel Gene	Adult HGBCL, NOS
BCL-32	chr20	46174583	46174583	T	C	exonic	CDH22	56	37	0.3978	Missense_Mutation	p.S804G	Non-Targeted Panel Gene	Adult HGBCL, NOS
BCL-32	chr7	122697880	122697880	C	T	exonic	RNF133	59	41	0.41	Missense_Mutation	p.D347N	Non-Targeted Panel Gene	Adult HGBCL, NOS
BCL-32	chr12	81753986	81753986	G	A	exonic	PPFIA2	78	55	0.4135	Missense_Mutation	p.S79L	Non-Targeted Panel Gene	Adult HGBCL, NOS
BCL-32	chr12	7922994	7922994	T	A	exonic	SLC2A3	41	30	0.4225	Missense_Mutation	p.I367F	Non-Targeted Panel Gene	Adult HGBCL, NOS
BCL-32	chr20	32918427	32918427	G	C	exonic	EFCAB8	166	122	0.4236	Missense_Mutation	p.E709D	Non-Targeted Panel Gene	Adult HGBCL, NOS
BCL-32	chr11	119019402	119019402	A	G	exonic	TRAPPC4	34	25	0.4237	Missense_Mutation	p.T5A	Non-Targeted Panel Gene	Adult HGBCL, NOS
BCL-32	chr6	43045282	43045282	C	T	exonic	CUL7	91	67	0.4241	Missense_Mutation	p.A995T	Non-Targeted Panel Gene	Adult HGBCL, NOS
BCL-32	chr4	81456661	81456661	C	T	exonic	RASGEF1B	76	58	0.4328	Missense_Mutation	p.S143N	Non-Targeted Panel Gene	Adult HGBCL, NOS
BCL-32	chr11	47338631	47338631	G	A	exonic	MYBPC3	77	59	0.4338	Missense_Mutation	p.R733C	Non-Targeted Panel Gene	Adult HGBCL, NOS
BCL-32	chr19	19149236	19149236	T	A	exonic	BORCS8-MEF2B;M	48	38	0.4419	Missense_Mutation	p.D83V	Non-Targeted Panel Gene	Adult HGBCL, NOS
BCL-32	chr9	106927434	106927434	A	C	exonic	ZNF462	113	90	0.4433	Missense_Mutation	p.Q1174H	Non-Targeted Panel Gene	Adult HGBCL, NOS
BCL-32	chr11	10000945	10000945	T	G	exonic	SBF2	23	19	0.4524	Missense_Mutation	p.H277P	Non-Targeted Panel Gene	Adult HGBCL, NOS
BCL-32	chr2	195824302	195824302	T	A	exonic	DNAH7	29	24	0.4528	Missense_Mutation	p.T3082S	Non-Targeted Panel Gene	Adult HGBCL, NOS
BCL-32	chr19	46755877	46755877	C	A	exonic	FKRP	71	60	0.458	Missense_Mutation	p.R143S	Non-Targeted Panel Gene	Adult HGBCL, NOS
BCL-32	chr12	122341069	122341069	G	C	exonic	CLIP1	30	26	0.4643	Missense_Mutation	p.A712G	Non-Targeted Panel Gene	Adult HGBCL, NOS
BCL-32	chr1	46189973	46189973	C	T	exonic	POMGNT1	49	45	0.4787	Missense_Mutation	p.D556N	Non-Targeted Panel Gene	Adult HGBCL, NOS
BCL-32	chr16	20955013	20955013	A	G	exonic	DNAH3	90	83	0.4798	Missense_Mutation	p.I3624T	Non-Targeted Panel Gene	Adult HGBCL, NOS
BCL-32	chr8	144512318	144512318	C	T	exonic	RECQL4	107	103	0.4905	Missense_Mutation	p.R1021Q	Non-Targeted Panel Gene	Adult HGBCL, NOS
BCL-32	chr10	110784367	110784367	C	T	exonic	RBM20	92	89	0.4917	Missense_Mutation	p.S455L	Non-Targeted Panel Gene	Adult HGBCL, NOS
BCL-32	chr1	45331467	45331467	G	A	exonic	MUTYH	62	61	0.4959	Missense_Mutation	p.R398C	Non-Targeted Panel Gene	Adult HGBCL, NOS
BCL-32	chr7	6154753	6154753	G	A	exonic	USP42	66	65	0.4962	Missense_Mutation	p.A1067T	Non-Targeted Panel Gene	Adult HGBCL, NOS
BCL-32	chr13	113239444	113239444	T	A	exonic	CUL4A	118	126	0.5164	Missense_Mutation	p.L210I	Non-Targeted Panel Gene	Adult HGBCL, NOS
BCL-32	chr4	52863280	52863280	G	C	exonic	RASL11B	43	46	0.5169	Missense_Mutation	p.R52P	Non-Targeted Panel Gene	Adult HGBCL, NOS
BCL-32	chr5	156344565	156344565	T	C	exonic	SGCD	62	68	0.5231	Missense_Mutation	p.I27T	Non-Targeted Panel Gene	Adult HGBCL, NOS
BCL-32	chr7	116759459	116759459	A	G	exonic	MET	68	76	0.5278	Missense_Mutation	p.N778S	Non-Targeted Panel Gene	Adult HGBCL, NOS
BCL-32	chr7	149210597	149210597	T	G	exonic	ZNF282	59	67	0.5317	Missense_Mutation	p.L282R	Non-Targeted Panel Gene	Adult HGBCL, NOS
BCL-32	chr14	95107916	95107916	C	T	exonic	DICER1	22	28	0.56	Missense_Mutation	p.A872T	Non-Targeted Panel Gene	Adult HGBCL, NOS
BCL-32	chr8	10608998	10608998	G	T	exonic	RP1L1	24	31	0.5636	Missense_Mutation	p.H1700Q	Non-Targeted Panel Gene	Adult HGBCL, NOS
BCL-32	chr4	82359581	82359581	A	C	exonic	HNRNPD	11	19	0.6333	Missense_Mutation	p.F117V	Non-Targeted Panel Gene	Adult HGBCL, NOS
BCL-32	chr5	141485576	141485576	C	T	exonic	PCDHGC4	235	431	0.6471	Missense_Mutation	p.R135W	Non-Targeted Panel Gene	Adult HGBCL, NOS
BCL-32	chrX	150512849	150512849	T	A	exonic	MAMLD1	53	101	0.6558	Missense_Mutation	p.F925I	Non-Targeted Panel Gene	Adult HGBCL, NOS
BCL-32	chr1	156767935	156767935	C	G	exonic	PRCC	28	54	0.6585	Missense_Mutation	p.P55R	Non-Targeted Panel Gene	Adult HGBCL, NOS
BCL-32	chr4	15003785	15003785	C	T	exonic	CPEB2	3	6	0.6667	Missense_Mutation	p.A371V	Non-Targeted Panel Gene	Adult HGBCL, NOS
BCL-32	chr19	54131332	54131332	C	T	exonic	PRPF31	141	284	0.6682	Missense_Mutation	p.A467V	Non-Targeted Panel Gene	Adult HGBCL, NOS
BCL-32	chrX	53234278	53234278	C	T	exonic	IQSEC2	6	15	0.7143	Missense_Mutation	p.A1470T	Non-Targeted Panel Gene	Adult HGBCL, NOS
BCL-32	chrX	48686087	48686087	G	A	exonic	WAS	24	126	0.84	Missense_Mutation	p.R171K	Non-Targeted Panel Gene	Adult HGBCL, NOS
BCL-32	chr6	106104961	106104961	C	G	exonic	PRDM1	399	264	0.3982	Nonsense_Mutation	p.Y267X	Targerted Panel Gene	Adult HGBCL, NOS
BCL-32	chr11	124030547	124030547	C	T	exonic	OR10G8	89	40	0.3101	Nonsense_Mutation	p.Q309X	Non-Targeted Panel Gene	Adult HGBCL, NOS
BCL-32	chr1	118085977	118085977	C	T	exonic	SPAG17	60	62	0.5082	Nonsense_Mutation	p.W569X	Non-Targeted Panel Gene	Adult HGBCL, NOS
BCL-33	chr4	154315746	154315747	AC	-	splicing	DCHS2	221	111	0.3343	Splice_Site	.	Targerted Panel Gene	Adult HGBCL, NOS
BCL-33	chr6	393097	393097	G	T	splicing	IRF4	98	30	0.2326	Splice_Site	.	Targerted Panel Gene	Adult HGBCL, NOS
BCL-33	chr6	37171492	37171492	G	C	splicing	PIM1	188	93	0.3298	Splice_Site	.	Targerted Panel Gene	Adult HGBCL, NOS
BCL-33	chr18	3450506	3450506	G	A	splicing	TGIF1	155	9	0.0545	Splice_Site	.	Non-Targeted Panel Gene	Adult HGBCL, NOS
BCL-33	chr11	35139371	35139371	G	T	splicing	CD44	39	36	0.48	Splice_Site	.	Non-Targeted Panel Gene	Adult HGBCL, NOS
BCL-33	chr6	14118038	14118082	GGTTCCCTACACGG	-	exonic	CD83	137	11	0.0743	Frame_Shift_Del	p.Q42fs	Non-Targeted Panel Gene	Adult HGBCL, NOS
BCL-33	chr21	34887055	34887092	GCGCGGTGGAAGG	-	exonic	RUNX1	113	11	0.0887	Frame_Shift_Del	p.A34fs	Non-Targeted Panel Gene	Adult HGBCL, NOS
BCL-33	chr3	56080930	56080930	T	-	exonic	ERC2	94	15	0.1376	Frame_Shift_Del	p.T510fs	Non-Targeted Panel Gene	Adult HGBCL, NOS
BCL-33	chr5	140968658	140968680	ACCTCTTTAAGGTA	-	exonic	PCDHAC2	178	56	0.2393	Frame_Shift_Del	p.D631fs	Non-Targeted Panel Gene	Adult HGBCL, NOS
BCL-33	chr1	37480343	37480343	G	-	exonic	ZC3H12A	191	86	0.3105	Frame_Shift_Del	p.W166fs	Non-Targeted Panel Gene	Adult HGBCL, NOS
BCL-33	chr7	2520145	2520145	C	-	exonic	LFNG	42	49	0.5385	Frame_Shift_Del	p.A95fs	Non-Targeted Panel Gene	Adult HGBCL, NOS
BCL-33	chr5	43453681	43453681	-	A	exonic	TMEM267	39	16	0.2909	Frame_Shift_Ins	p.L97fs	Non-Targeted Panel Gene	Adult HGBCL, NOS
BCL-33	chr4	39460842	39460842	-	T	exonic	LIAS	51	30	0.3659	Frame_Shift_Ins	p.D33fs	Non-Targeted Panel Gene	Adult HGBCL, NOS
BCL-33	chr10	87863967	87863967	-	GCGGCG	exonic	PTEN	56	23	0.284	In_Frame_Ins	p.E6delinsEAA	Targerted Panel Gene	Adult HGBCL, NOS
BCL-33	chrX	71537865	71537865	-	CTG	exonic	OGT	48	61	0.5596	In_Frame_Ins	p.N75delinsNL	Non-Targeted Panel Gene	Adult HGBCL, NOS
BCL-33	chr12	92145427	92145427	G	T	exonic	BTG1	126	82	0.3942	Missense_Mutation	p.L37M	Targerted Panel Gene	Adult HGBCL, NOS
BCL-33	chr11	103120689	103120689	C	A	exonic	DYNC2H1	357	91	0.2031	Missense_Mutation	p.P379T	Targerted Panel Gene	Adult HGBCL, NOS

BCL-33	chr4	186603344	186603344	C	T	exonic	FAT1	204	209	0.5061	Missense_Mutation	p.V3728I	Targerted Panel Gene	Adult HGBCL, NOS
BCL-33	chr4	186707007	186707007	G	C	exonic	FAT1	278	235	0.4581	Missense_Mutation	p.P941A	Targerted Panel Gene	Adult HGBCL, NOS
BCL-33	chr13	38692153	38692153	C	A	exonic	FREM2	468	201	0.3004	Missense_Mutation	p.S1603R	Targerted Panel Gene	Adult HGBCL, NOS
BCL-33	chr6	26156757	26156757	G	C	exonic	HIST1H1E	375	154	0.2906	Missense_Mutation	p.A123P	Targerted Panel Gene	Adult HGBCL, NOS
BCL-33	chr6	26124362	26124362	G	A	exonic	HIST1H2AC	242	101	0.2945	Missense_Mutation	p.V44I	Targerted Panel Gene	Adult HGBCL, NOS
BCL-33	chr22	22893792	22893792	G	A	exonic	IGLL5	150	129	0.4607	Missense_Mutation	p.G100E	Targerted Panel Gene	Adult HGBCL, NOS
BCL-33	chr6	393256	393256	G	A	exonic	IRF4	350	34	0.0885	Missense_Mutation	p.G35D	Targerted Panel Gene	Adult HGBCL, NOS
BCL-33	chr19	38613910	38613910	C	G	exonic	MAP4K1	238	126	0.3452	Missense_Mutation	p.R168T	Targerted Panel Gene	Adult HGBCL, NOS
BCL-33	chr9	13158073	13158073	C	T	exonic	MPDZ	325	299	0.4792	Missense_Mutation	p.G1133S	Targerted Panel Gene	Adult HGBCL, NOS
BCL-33	chr8	127738379	127738379	G	C	exonic	MYC	325	181	0.3577	Missense_Mutation	p.E54D	Targerted Panel Gene	Adult HGBCL, NOS
BCL-33	chr7	82965911	82965911	T	C	exonic	PCLO	442	205	0.3164	Missense_Mutation	p.T1293A	Targerted Panel Gene	Adult HGBCL, NOS
BCL-33	chr3	79589859	79589859	G	A	exonic	ROBO1	174	465	0.7277	Missense_Mutation	p.S18F	Targerted Panel Gene	Adult HGBCL, NOS
BCL-33	chr4	20553855	20553855	C	T	exonic	SLIT2	323	170	0.3441	Missense_Mutation	p.S871F	Targerted Panel Gene	Adult HGBCL, NOS
BCL-33	chr17	7675125	7675125	A	G	exonic	TP53	137	133	0.4926	Missense_Mutation	p.Y124H	Targerted Panel Gene	Adult HGBCL, NOS
BCL-33	chr17	59839859	59839859	G	A	exonic	VMP1	386	164	0.2982	Missense_Mutation	p.S390N	Targerted Panel Gene	Adult HGBCL, NOS
BCL-33	chrX	48918627	48918627	G	A	exonic	PIM2	118	10	0.0781	Missense_Mutation	p.A27V	Non-Targeted Panel Gene	Adult HGBCL, NOS
BCL-33	chr19	12792133	12792133	C	T	exonic	JUNB	294	32	0.0982	Missense_Mutation	p.A121V	Non-Targeted Panel Gene	Adult HGBCL, NOS
BCL-33	chrX	24057492	24057492	T	C	exonic	EIF2S3	16	2	0.1111	Missense_Mutation	p.F69L	Non-Targeted Panel Gene	Adult HGBCL, NOS
BCL-33	chr3	143120979	143120979	G	A	exonic	CHST2	93	13	0.1226	Missense_Mutation	p.A55T	Non-Targeted Panel Gene	Adult HGBCL, NOS
BCL-33	chr18	68846324	68846324	C	G	exonic	CCDC102B	100	15	0.1304	Missense_Mutation	p.A280G	Non-Targeted Panel Gene	Adult HGBCL, NOS
BCL-33	chr21	34887097	34887097	C	T	exonic;splicing	RUNX1;RUNX1	94	15	0.1376	Missense_Mutation	p.D6N	Non-Targeted Panel Gene	Adult HGBCL, NOS
BCL-33	chr17	10498659	10498659	G	A	exonic	MYH1	61	12	0.1644	Missense_Mutation	p.A1383V	Non-Targeted Panel Gene	Adult HGBCL, NOS
BCL-33	chr1	75238528	75238528	A	C	exonic	SLC44A5	30	8	0.2105	Missense_Mutation	p.L172R	Non-Targeted Panel Gene	Adult HGBCL, NOS
BCL-33	chr3	50571109	50571109	A	C	exonic	HEMK1	163	49	0.2311	Missense_Mutation	p.E2A	Non-Targeted Panel Gene	Adult HGBCL, NOS
BCL-33	chr9	6986415	6986415	A	C	exonic	KDM4C	181	55	0.2331	Missense_Mutation	p.S476R	Non-Targeted Panel Gene	Adult HGBCL, NOS
BCL-33	chr1	12267899	12267899	G	A	exonic	VPS13D	96	30	0.2381	Missense_Mutation	p.D594N	Non-Targeted Panel Gene	Adult HGBCL, NOS
BCL-33	chr4	6062412	6062412	C	T	exonic	JAKMIP1	28	9	0.2432	Missense_Mutation	p.R487H	Non-Targeted Panel Gene	Adult HGBCL, NOS
BCL-33	chr1	56674462	56674462	G	A	exonic	PRKAA2	14	5	0.2632	Missense_Mutation	p.G59E	Non-Targeted Panel Gene	Adult HGBCL, NOS
BCL-33	chr3	49510793	49510793	A	G	exonic	DAG1	231	83	0.2643	Missense_Mutation	p.I87V	Non-Targeted Panel Gene	Adult HGBCL, NOS
BCL-33	chr1	89380611	89380611	G	T	exonic	GBP6	47	17	0.2656	Missense_Mutation	p.G284V	Non-Targeted Panel Gene	Adult HGBCL, NOS
BCL-33	chr6	31356365	31356365	C	T	exonic	HLA-B	151	56	0.2705	Missense_Mutation	p.A141T	Non-Targeted Panel Gene	Adult HGBCL, NOS
BCL-33	chr10	127738747	127738747	G	A	exonic	FOXI2	80	30	0.2727	Missense_Mutation	p.G247S	Non-Targeted Panel Gene	Adult HGBCL, NOS
BCL-33	chr6	165416205	165416205	C	T	exonic	PDE10A	69	26	0.2737	Missense_Mutation	p.D349N	Non-Targeted Panel Gene	Adult HGBCL, NOS
BCL-33	chr19	35890026	35890026	G	A	exonic	NFKBID	58	22	0.275	Missense_Mutation	p.A251V	Non-Targeted Panel Gene	Adult HGBCL, NOS
BCL-33	chr1	17282940	17282940	C	T	exonic	PADI3	52	20	0.2778	Missense_Mutation	p.S619F	Non-Targeted Panel Gene	Adult HGBCL, NOS
BCL-33	chr7	37314864	37314864	A	G	exonic	ELMO1	37	15	0.2885	Missense_Mutation	p.Y60H	Non-Targeted Panel Gene	Adult HGBCL, NOS
BCL-33	chr20	32898494	32898494	C	T	exonic	EFCAB8	142	58	0.2886	Missense_Mutation	p.A320V	Non-Targeted Panel Gene	Adult HGBCL, NOS
BCL-33	chr19	12792184	12792184	G	A	exonic	JUNB	233	96	0.2918	Missense_Mutation	p.G138D	Non-Targeted Panel Gene	Adult HGBCL, NOS
BCL-33	chr20	62323655	62323655	G	C	exonic	LAMA5	63	26	0.2921	Missense_Mutation	p.F1955L	Non-Targeted Panel Gene	Adult HGBCL, NOS
BCL-33	chr12	12718942	12718942	C	G	exonic	CDKN1B	29	12	0.2927	Missense_Mutation	p.T198R	Non-Targeted Panel Gene	Adult HGBCL, NOS
BCL-33	chr5	128084066	128084066	G	A	exonic	SLC12A2	14	6	0.3	Missense_Mutation	p.A38T	Non-Targeted Panel Gene	Adult HGBCL, NOS
BCL-33	chr19	12791827	12791827	G	A	exonic	JUNB	184	79	0.3004	Missense_Mutation	p.G19E	Non-Targeted Panel Gene	Adult HGBCL, NOS
BCL-33	chr17	78358698	78358698	G	A	exonic	SOCS3	243	105	0.3017	Missense_Mutation	p.A133V	Non-Targeted Panel Gene	Adult HGBCL, NOS
BCL-33	chr11	13963041	13963041	G	C	exonic	SPON1	105	47	0.3072	Missense_Mutation	p.R46P	Non-Targeted Panel Gene	Adult HGBCL, NOS
BCL-33	chr11	59211049	59211049	G	A	exonic	MPEG1	144	64	0.3077	Missense_Mutation	p.A606V	Non-Targeted Panel Gene	Adult HGBCL, NOS
BCL-33	chr7	45610383	45610383	G	A	exonic	ADCY1	173	78	0.3108	Missense_Mutation	p.R40Q	Non-Targeted Panel Gene	Adult HGBCL, NOS
BCL-33	chr6	31356948	31356948	G	A	exonic	HLA-B	53	24	0.3117	Missense_Mutation	p.S28F	Non-Targeted Panel Gene	Adult HGBCL, NOS
BCL-33	chr19	38838497	38838497	G	A	exonic	HNRNPL	320	148	0.3162	Missense_Mutation	p.S486F	Non-Targeted Panel Gene	Adult HGBCL, NOS
BCL-33	chr16	30474226	30474226	G	A	exonic	ITGAL	92	43	0.3185	Missense_Mutation	p.R31Q	Non-Targeted Panel Gene	Adult HGBCL, NOS
BCL-33	chr19	35787296	35787296	G	A	exonic	ARHGAP33	70	33	0.3204	Missense_Mutation	p.V750M	Non-Targeted Panel Gene	Adult HGBCL, NOS
BCL-33	chr4	108650759	108650759	C	T	exonic	OSTC	53	25	0.3205	Missense_Mutation	p.A35V	Non-Targeted Panel Gene	Adult HGBCL, NOS
BCL-33	chr1	81942041	81942041	A	T	exonic	ADGRL2	58	28	0.3256	Missense_Mutation	p.Q135H	Non-Targeted Panel Gene	Adult HGBCL, NOS
BCL-33	chr22	50414630	50414630	C	G	exonic	PPP6R2	141	69	0.3286	Missense_Mutation	p.L165V	Non-Targeted Panel Gene	Adult HGBCL, NOS
BCL-33	chr15	52196367	52196367	G	A	exonic	MYO5C	75	37	0.3304	Missense_Mutation	p.T1646I	Non-Targeted Panel Gene	Adult HGBCL, NOS
BCL-33	chr12	125349659	125349659	G	A	exonic	TMEM132B	130	66	0.3367	Missense_Mutation	p.S87N	Non-Targeted Panel Gene	Adult HGBCL, NOS
BCL-33	chr13	93227539	93227539	G	A	exonic	GPC6	102	52	0.3377	Missense_Mutation	p.R28Q	Non-Targeted Panel Gene	Adult HGBCL, NOS
BCL-33	chr19	36102794	36102794	T	A	exonic	WDR62	104	54	0.3418	Missense_Mutation	p.F1088Y	Non-Targeted Panel Gene	Adult HGBCL, NOS
BCL-33	chr17	78358938	78358938	C	G	exonic	SOCS3	193	103	0.348	Missense_Mutation	p.G53A	Non-Targeted Panel Gene	Adult HGBCL, NOS
BCL-33	chr17	59088361	59088361	C	T	exonic	TRIM37	37	20	0.3509	Missense_Mutation	p.E71K	Non-Targeted Panel Gene	Adult HGBCL, NOS
BCL-33	chr1	180155053	180155053	C	A	exonic	QSOX1	45	25	0.3571	Missense_Mutation	p.T49K	Non-Targeted Panel Gene	Adult HGBCL, NOS
BCL-33	chr16	27403175	27403175	G	A	exonic	IL21R	183	103	0.3601	Missense_Mutation	p.S2N	Non-Targeted Panel Gene	Adult HGBCL, NOS
BCL-33	chr21	30642953	30642953	C	T	exonic	KRTAP20-3	72	41	0.3628	Missense_Mutation	p.T22I	Non-Targeted Panel Gene	Adult HGBCL, NOS
BCL-33	chr17	61793626	61793626	T	C	exonic	BRIP1	14	8	0.3636	Missense_Mutation	p.I482V	Non-Targeted Panel Gene	Adult HGBCL, NOS
BCL-33	chr2	1494006	1494006	G	A	exonic	TPO	136	78	0.3645	Missense_Mutation	p.G658E	Non-Targeted Panel Gene	Adult HGBCL, NOS
BCL-33	chr14	94383039	94383039	G	A	exonic	SERPINA1	432	266	0.3811	Missense_Mutation	p.H67Y	Non-Targeted Panel Gene	Adult HGBCL, NOS
BCL-33	chr3	85802133	85802133	G	T	exonic	CADM2	63	39	0.3824	Missense_Mutation	p.D59Y	Non-Targeted Panel Gene	Adult HGBCL, NOS
BCL-33	chr1	1204457	1204457	C	A	exonic	TNFRSF18	71	49	0.4083	Missense_Mutation	p.D114Y	Non-Targeted Panel Gene	Adult HGBCL, NOS
BCL-33	chr1	234608447	234608447	C	T	exonic	IRF2BP2	37	26	0.4127	Missense_Mutation	p.V350I	Non-Targeted Panel Gene	Adult HGBCL, NOS
BCL-33	chr2	120990472	120990472	G	A	exonic	GLI2	90	69	0.434	Missense_Mutation	p.D1520N	Non-Targeted Panel Gene	Adult HGBCL, NOS
BCL-33	chr13	101868778	101868778	C	T	exonic	FGF14	68	54	0.4426	Missense_Mutation	p.V35M	Non-Targeted Panel Gene	Adult HGBCL, NOS
BCL-33	chr8	100213137	100213137	A	C	exonic	SPAG1	81	67	0.4527	Missense_Mutation	p.M382L	Non-Targeted Panel Gene	Adult HGBCL, NOS
BCL-33	chr1	206685447	206685447	G	A	exonic	MAPKAPK2	50	42	0.4565	Missense_Mutation	p.G73D	Non-Targeted Panel Gene	Adult HGBCL, NOS
BCL-33	chr15	75932753	75932753	C	A	exonic	FBXO22	165	141	0.4608	Missense_Mutation	p.A288D	Non-Targeted Panel Gene	Adult HGBCL, NOS
BCL-33	chr12	102855313	102855313	C	T	exonic	PAH	69	59	0.4609	Missense_Mutation	p.V177M	Non-Targeted Panel Gene	Adult HGBCL, NOS
BCL-33	chr6	34527384	34527384	G	A	exonic	PACSIN1	142	123	0.4642	Missense_Mutation	p.C39Y	Non-Targeted Panel Gene	Adult HGBCL, NOS
BCL-33	chr5	141956965	141956965	C	T	exonic	PCDH12	238	207	0.4652	Missense_Mutation	p.S296N	Non-Targeted Panel Gene	Adult HGBCL, NOS
BCL-33	chr5	37153769	37153769	G	T	exonic	C5orf42	31	27	0.4655	Missense_Mutation	p.P2728T	Non-Targeted Panel Gene	Adult HGBCL, NOS
BCL-33	chr17	76265917	76265917	G	A	exonic	UBALD2	24	21	0.4667	Missense_Mutation	p.S44N	Non-Targeted Panel Gene	Adult HGBCL, NOS
BCL-33	chr18	46216233	46216233	C	T	exonic	C18orf25	57	53	0.4818	Missense_Mutation	p.S118F	Non-Targeted Panel Gene	Adult HGBCL, NOS
BCL-33	chr14	34536151	34536151	C	G	exonic	EAPP	26	25	0.4902	Missense_Mutation	p.A67P	Non-Targeted Panel Gene	Adult HGBCL, NOS
BCL-33	chr16	22140166	22140166	G	T	exonic	VWA3A	46	48	0.5106	Missense_Mutation	p.D769Y	Non-Targeted Panel Gene	Adult HGBCL, NOS
BCL-33	chr5	141224019	141224019	A	G	exonic	PCDHB14	58	62	0.5167	Missense_Mutation	p.I172V	Non-Targeted Panel Gene	Adult HGBCL, NOS

BCL-33	chr14	24262228	24262228	G	T	exonic	TGM1	111	119	0.5174	Missense_Mutation	p.S42Y	Non-Targeted Panel Gene	Adult HGBCL, NOS
BCL-33	chr12	50222445	50222445	A	G	exonic	LIMA1	65	72	0.5255	Missense_Mutation	p.L69P	Non-Targeted Panel Gene	Adult HGBCL, NOS
BCL-33	chr1	33013276	33013276	C	T	exonic	AK2	62	70	0.5303	Missense_Mutation	p.A161T	Non-Targeted Panel Gene	Adult HGBCL, NOS
BCL-33	chr19	4433343	4433343	A	G	exonic	CHAF1A	169	193	0.5331	Missense_Mutation	p.Q826R	Non-Targeted Panel Gene	Adult HGBCL, NOS
BCL-33	chr15	67344174	67344174	G	C	exonic	IQCH	46	53	0.5354	Missense_Mutation	p.R207P	Non-Targeted Panel Gene	Adult HGBCL, NOS
BCL-33	chr13	69839021	69839021	T	C	exonic	KLHL1	11	14	0.56	Missense_Mutation	p.T457A	Non-Targeted Panel Gene	Adult HGBCL, NOS
BCL-33	chr20	63775843	63775843	G	T	exonic	ZBTB46	64	82	0.5616	Missense_Mutation	p.L353I	Non-Targeted Panel Gene	Adult HGBCL, NOS
BCL-33	chr14	50420105	50420105	C	T	exonic	MAP4K5	16	22	0.5789	Missense_Mutation	p.V819I	Non-Targeted Panel Gene	Adult HGBCL, NOS
BCL-33	chr1	12242532	12242532	C	G	exonic	VPS13D	24	33	0.5789	Missense_Mutation	p.N39K	Non-Targeted Panel Gene	Adult HGBCL, NOS
BCL-33	chr5	111119021	111119021	A	G	exonic	WDR36	23	33	0.5893	Missense_Mutation	p.D658G	Non-Targeted Panel Gene	Adult HGBCL, NOS
BCL-33	chr9	108999218	108999218	C	A	exonic	CTNNAL1	10	16	0.6154	Missense_Mutation	p.K60N	Non-Targeted Panel Gene	Adult HGBCL, NOS
BCL-33	chrX	48918642	48918642	C	T	exonic	PIM2	45	73	0.6186	Missense_Mutation	p.G22D	Non-Targeted Panel Gene	Adult HGBCL, NOS
BCL-33	chrX	2949384	2949384	G	C	exonic	ARSE	85	146	0.632	Missense_Mutation	p.N283K	Non-Targeted Panel Gene	Adult HGBCL, NOS
BCL-33	chr3	186790246	186790246	C	T	exonic	RFC4	24	63	0.7241	Missense_Mutation	p.D298N	Non-Targeted Panel Gene	Adult HGBCL, NOS
BCL-33	chrX	111163565	111163565	T	C	exonic	PAK3	10	28	0.7368	Missense_Mutation	p.Y238H	Non-Targeted Panel Gene	Adult HGBCL, NOS
BCL-33	chrX	142202886	142202886	T	A	exonic	MAGEC2	44	136	0.7556	Missense_Mutation	p.N368Y	Non-Targeted Panel Gene	Adult HGBCL, NOS
BCL-33	chr16	89102704	89102704	G	T	exonic	ACSF3	53	170	0.7623	Missense_Mutation	p.C256F	Non-Targeted Panel Gene	Adult HGBCL, NOS
BCL-33	chr4	118282175	118282175	G	A	exonic	PRSS12	31	112	0.7832	Missense_Mutation	p.R797C	Non-Targeted Panel Gene	Adult HGBCL, NOS
BCL-33	chrX	64191491	64191491	T	C	exonic	AMER1	2	78	0.975	Missense_Mutation	p.Y599C	Non-Targeted Panel Gene	Adult HGBCL, NOS
BCL-33	chr18	32770306	32770306	G	A	exonic	KLHL14	158	87	0.3551	Nonsense_Mutation	p.Q96X	Non-Targeted Panel Gene	Adult HGBCL, NOS
BCL-33	chr17	77193544	77193544	A	T	exonic	SEC14L1	49	32	0.3951	Nonsense_Mutation	p.K157X	Non-Targeted Panel Gene	Adult HGBCL, NOS
BCL-33	chr16	89282455	89282455	G	A	exonic	ANKRD11	33	61	0.6489	Nonsense_Mutation	p.R1363X	Non-Targeted Panel Gene	Adult HGBCL, NOS
BCL-34	chr20	36660385	36660385	C	A	splicing	NDRG3	15	2	0.1176	Splice_Site	.	Non-Targeted Panel Gene	Adult HGBCL, NOS
BCL-34	chr2	24164309	24164309	C	-	exonic	FAM228B	60	50	0.4545	Frame_Shift_Del	p.S165fs	Non-Targeted Panel Gene	Adult HGBCL, NOS
BCL-34	chrX	135546882	135546882	G	-	exonic	INTS6L	77	76	0.4967	Frame_Shift_Del	p.G204fs	Non-Targeted Panel Gene	Adult HGBCL, NOS
BCL-34	chr4	78541102	78541104	TAC	-	exonic	FRAS1	173	113	0.3951	In_Frame_Del	p.4006_4007del	Targerted Panel Gene	Adult HGBCL, NOS
BCL-34	chr1	17826035	17826043	TGACTCAGA	-	exonic	ACTL8	156	15	0.0877	In_Frame_Del	p.206_209del	Non-Targeted Panel Gene	Adult HGBCL, NOS
BCL-34	chr4	54100786	54100809	GCGGCGGCGGCAG	-	exonic	GSX2	42	20	0.3226	In_Frame_Del	p.148_155del	Non-Targeted Panel Gene	Adult HGBCL, NOS
BCL-34	chrX	41345220	41345220	G	A	exonic	DDX3X	309	47	0.132	Missense_Mutation	p.G356R	Targerted Panel Gene	Adult HGBCL, NOS
BCL-34	chr13	72771144	72771144	C	T	exonic	DIS3	112	145	0.562	Missense_Mutation	p.R536K	Targerted Panel Gene	Adult HGBCL, NOS
BCL-34	chrX	31444566	31444566	C	T	exonic	DMD	332	50	0.1309	Missense_Mutation	p.R2877H	Targerted Panel Gene	Adult HGBCL, NOS
BCL-34	chr7	148811636	148811636	A	T	exonic	EZH2	197	76	0.2774	Missense_Mutation	p.Y646N	Targerted Panel Gene	Adult HGBCL, NOS
BCL-34	chr3	50252136	50252136	A	G	exonic	GNAI2	141	32	0.185	Missense_Mutation	p.Q36R	Targerted Panel Gene	Adult HGBCL, NOS
BCL-34	chr22	22888154	22888154	T	G	exonic	IGLL5	85	57	0.4014	Missense_Mutation	p.V34G	Targerted Panel Gene	Adult HGBCL, NOS
BCL-34	chr8	127736613	127736613	T	C	exonic	MYC	129	91	0.4136	Missense_Mutation	p.V7A	Targerted Panel Gene	Adult HGBCL, NOS
BCL-34	chr8	127738927	127738927	C	G	exonic	MYC	114	45	0.283	Missense_Mutation	p.S237C	Targerted Panel Gene	Adult HGBCL, NOS
BCL-34	chr8	127739017	127739017	C	G	exonic	MYC	139	42	0.232	Missense_Mutation	p.S267C	Targerted Panel Gene	Adult HGBCL, NOS
BCL-34	chr8	127738642	127738642	A	C	exonic	MYC	148	43	0.2251	Missense_Mutation	p.N142T	Targerted Panel Gene	Adult HGBCL, NOS
BCL-34	chr8	127738343	127738343	G	C	exonic	MYC	148	41	0.2147	Missense_Mutation	p.E42D	Targerted Panel Gene	Adult HGBCL, NOS
BCL-34	chr8	127738371	127738371	C	G	exonic	MYC	140	32	0.186	Missense_Mutation	p.Q52E	Targerted Panel Gene	Adult HGBCL, NOS
BCL-34	chr8	127738378	127738378	A	C	exonic	MYC	143	31	0.1782	Missense_Mutation	p.E54A	Targerted Panel Gene	Adult HGBCL, NOS
BCL-34	chr10	87933148	87933148	G	A	exonic	PTEN	223	84	0.2718	Missense_Mutation	p.R303Q	Targerted Panel Gene	Adult HGBCL, NOS
BCL-34	chr15	41882718	41882718	G	A	exonic	SPTBN5	47	15	0.2419	Missense_Mutation	p.T638I	Targerted Panel Gene	Adult HGBCL, NOS
BCL-34	chrX	51895273	51895273	A	C	exonic	MAGED1	215	27	0.1116	Missense_Mutation	p.N145T	Non-Targeted Panel Gene	Adult HGBCL, NOS
BCL-34	chrX	71291853	71291853	T	G	exonic	NONO	92	12	0.1154	Missense_Mutation	p.F77V	Non-Targeted Panel Gene	Adult HGBCL, NOS
BCL-34	chr17	58651880	58651880	T	G	exonic	TEX14	70	11	0.1358	Missense_Mutation	p.K41T	Non-Targeted Panel Gene	Adult HGBCL, NOS
BCL-34	chr5	124744298	124744298	G	A	exonic	ZNF608	50	8	0.1379	Missense_Mutation	p.S231F	Non-Targeted Panel Gene	Adult HGBCL, NOS
BCL-34	chr12	75207487	75207487	A	G	exonic	KCNC2	24	4	0.1429	Missense_Mutation	p.L166P	Non-Targeted Panel Gene	Adult HGBCL, NOS
BCL-34	chr1	69980408	69980408	G	T	exonic	LRRC7	24	4	0.1429	Missense_Mutation	p.L214F	Non-Targeted Panel Gene	Adult HGBCL, NOS
BCL-34	chr12	827187	827187	C	T	exonic	WNK1	104	18	0.1475	Missense_Mutation	p.P360S	Non-Targeted Panel Gene	Adult HGBCL, NOS
BCL-34	chr6	13595480	13595480	G	A	exonic	SIRT5	28	5	0.1515	Missense_Mutation	p.S160N	Non-Targeted Panel Gene	Adult HGBCL, NOS
BCL-34	chr6	149451021	149451021	C	T	exonic	ZC3H12D	22	4	0.1538	Missense_Mutation	p.E416K	Non-Targeted Panel Gene	Adult HGBCL, NOS
BCL-34	chrX	124420615	124420615	G	T	exonic	TENM1	171	32	0.1576	Missense_Mutation	p.H1559N	Non-Targeted Panel Gene	Adult HGBCL, NOS
BCL-34	chr11	66334836	66334836	G	C	exonic	RIN1	64	12	0.1579	Missense_Mutation	p.S321R	Non-Targeted Panel Gene	Adult HGBCL, NOS
BCL-34	chr19	56782079	56782079	C	T	exonic	ZIM2	110	21	0.1603	Missense_Mutation	p.E174K	Non-Targeted Panel Gene	Adult HGBCL, NOS
BCL-34	chr12	109085901	109085901	C	G	exonic	USP30	73	14	0.1609	Missense_Mutation	p.H477Q	Non-Targeted Panel Gene	Adult HGBCL, NOS
BCL-34	chr1	8356159	8356159	G	A	exonic	RERE	89	18	0.1682	Missense_Mutation	p.P1476L	Non-Targeted Panel Gene	Adult HGBCL, NOS
BCL-34	chrX	47626088	47626088	C	T	exonic	CFP	67	14	0.1728	Missense_Mutation	p.R405H	Non-Targeted Panel Gene	Adult HGBCL, NOS
BCL-34	chr13	49566705	49566705	C	T	exonic	RCBTB1	50	12	0.1935	Missense_Mutation	p.V64I	Non-Targeted Panel Gene	Adult HGBCL, NOS
BCL-34	chr22	41528566	41528566	T	G	exonic	ACO2	77	20	0.2062	Missense_Mutation	p.F766V	Non-Targeted Panel Gene	Adult HGBCL, NOS
BCL-34	chr18	50729702	50729702	G	A	exonic	MAPK4	83	22	0.2095	Missense_Mutation	p.D538N	Non-Targeted Panel Gene	Adult HGBCL, NOS
BCL-34	chr16	89646671	89646671	G	A	exonic	CHMP1A	80	25	0.2381	Missense_Mutation	p.P142L	Non-Targeted Panel Gene	Adult HGBCL, NOS
BCL-34	chr3	186784569	186784569	C	G	exonic	EIF4A2	62	20	0.2439	Missense_Mutation	p.N27K	Non-Targeted Panel Gene	Adult HGBCL, NOS
BCL-34	chr17	44323328	44323328	A	C	exonic	SLC25A39	27	9	0.25	Missense_Mutation	p.V34G	Non-Targeted Panel Gene	Adult HGBCL, NOS
BCL-34	chr12	32878461	32878461	G	A	exonic	PKP2	144	50	0.2577	Missense_Mutation	p.S140F	Non-Targeted Panel Gene	Adult HGBCL, NOS
BCL-34	chrX	129788489	129788489	A	G	exonic	SASH3	185	68	0.2688	Missense_Mutation	p.K71R	Non-Targeted Panel Gene	Adult HGBCL, NOS
BCL-34	chr1	85093621	85093621	T	G	exonic	WDR63	59	22	0.2716	Missense_Mutation	p.C341G	Non-Targeted Panel Gene	Adult HGBCL, NOS
BCL-34	chr6	152471655	152471655	G	T	exonic	SYNE1	72	27	0.2727	Missense_Mutation	p.S525Y	Non-Targeted Panel Gene	Adult HGBCL, NOS
BCL-34	chrX	23000216	23000216	T	G	exonic	DDX53	63	28	0.3077	Missense_Mutation	p.F53L	Non-Targeted Panel Gene	Adult HGBCL, NOS
BCL-34	chr3	14715326	14715326	A	G	exonic	C3orf20	106	50	0.3205	Missense_Mutation	p.T451A	Non-Targeted Panel Gene	Adult HGBCL, NOS
BCL-34	chr10	27035490	27035490	A	G	exonic	ANKRD26	20	10	0.3333	Missense_Mutation	p.M987T	Non-Targeted Panel Gene	Adult HGBCL, NOS
BCL-34	chr8	91249823	91249823	G	A	exonic	SLC26A7	57	29	0.3372	Missense_Mutation	p.A58T	Non-Targeted Panel Gene	Adult HGBCL, NOS
BCL-34	chr7	93477612	93477612	C	G	exonic	CALCR	79	44	0.3577	Missense_Mutation	p.G106R	Non-Targeted Panel Gene	Adult HGBCL, NOS
BCL-34	chr17	47709109	47709109	G	T	exonic	TBKBP1	21	14	0.4	Missense_Mutation	p.R459L	Non-Targeted Panel Gene	Adult HGBCL, NOS
BCL-34	chr5	90720961	90720961	C	T	exonic	ADGRV1	68	48	0.4138	Missense_Mutation	p.A3217V	Non-Targeted Panel Gene	Adult HGBCL, NOS
BCL-34	chrX	65499195	65499195	A	G	exonic	ZC3H12B	106	77	0.4208	Missense_Mutation	p.I315M	Non-Targeted Panel Gene	Adult HGBCL, NOS
BCL-34	chr17	410412	410412	G	A	exonic	C17orf97	25	19	0.4318	Missense_Mutation	p.A24T	Non-Targeted Panel Gene	Adult HGBCL, NOS
BCL-34	chr16	89920138	89920138	G	C	exonic	MC1R	71	55	0.4365	Missense_Mutation	p.D294H	Non-Targeted Panel Gene	Adult HGBCL, NOS
BCL-34	chr19	17843141	17843141	G	C	exonic	JAK3	28	23	0.451	Missense_Mutation	p.P151R	Non-Targeted Panel Gene	Adult HGBCL, NOS
BCL-34	chr7	43438073	43438073	G	A	exonic	HECW1	85	70	0.4516	Missense_Mutation	p.R291H	Non-Targeted Panel Gene	Adult HGBCL, NOS
BCL-34	chr11	2422320	2422320	A	T	exonic	TRPM5	40	33	0.4521	Missense_Mutation	p.F40Y	Non-Targeted Panel Gene	Adult HGBCL, NOS
BCL-34	chr11	2403316	2403316	G	C	exonic	TSSC4	116	96	0.4528	Missense_Mutation	p.R164T	Non-Targeted Panel Gene	Adult HGBCL, NOS

BCL-34	chr9	136687272	136687272	T	C	exonic	AGPAT2	73	62	0.4559	Missense_Mutation	p.K29R	Non-Targeted Panel Gene	Adult HGBCL, NOS
BCL-34	chr7	51083136	51083136	G	C	exonic	COBL	35	31	0.4697	Missense_Mutation	p.P397A	Non-Targeted Panel Gene	Adult HGBCL, NOS
BCL-34	chr5	71011298	71011298	C	G	exonic	NAIP	27	24	0.4706	Missense_Mutation	p.K215N	Non-Targeted Panel Gene	Adult HGBCL, NOS
BCL-34	chr19	7647436	7647436	G	A	exonic	STXBP2	63	57	0.475	Missense_Mutation	p.G541S	Non-Targeted Panel Gene	Adult HGBCL, NOS
BCL-34	chr21	42220015	42220015	T	C	exonic	ABCG1	34	31	0.4769	Missense_Mutation	p.V15A	Non-Targeted Panel Gene	Adult HGBCL, NOS
BCL-34	chr9	127501036	127501036	G	A	exonic	LRSAM1	71	69	0.4894	Missense_Mutation	p.V647I	Non-Targeted Panel Gene	Adult HGBCL, NOS
BCL-34	chr9	136395326	136395326	G	C	exonic	SNAPC4	49	47	0.4896	Missense_Mutation	p.P148R	Non-Targeted Panel Gene	Adult HGBCL, NOS
BCL-34	chr5	148827322	148827322	C	T	exonic	ADRB2	108	104	0.4906	Missense_Mutation	p.T164I	Non-Targeted Panel Gene	Adult HGBCL, NOS
BCL-34	chr11	47413496	47413496	C	T	exonic	SLC39A13	36	35	0.493	Missense_Mutation	p.R212W	Non-Targeted Panel Gene	Adult HGBCL, NOS
BCL-34	chr22	17121024	17121024	C	A	exonic	CECR6	6	6	0.5	Missense_Mutation	p.G35V	Non-Targeted Panel Gene	Adult HGBCL, NOS
BCL-34	chr2	178620762	178620762	T	C	exonic	TTN	24	24	0.5	Missense_Mutation	p.N15283S	Non-Targeted Panel Gene	Adult HGBCL, NOS
BCL-34	chr17	65207993	65207993	A	C	exonic	RGS9	47	48	0.5053	Missense_Mutation	p.E425D	Non-Targeted Panel Gene	Adult HGBCL, NOS
BCL-34	chr16	89546737	89546737	C	T	exonic	SPG7	56	61	0.5214	Missense_Mutation	p.A510V	Non-Targeted Panel Gene	Adult HGBCL, NOS
BCL-34	chr14	23398856	23398856	T	G	exonic	MYH6	122	133	0.5216	Missense_Mutation	p.D588A	Non-Targeted Panel Gene	Adult HGBCL, NOS
BCL-34	chr17	74357571	74357571	A	G	exonic	BTBD17	34	42	0.5526	Missense_Mutation	p.Y175H	Non-Targeted Panel Gene	Adult HGBCL, NOS
BCL-34	chr4	2932389	2932389	A	T	exonic	MFSD10	43	58	0.5743	Missense_Mutation	p.F219I	Non-Targeted Panel Gene	Adult HGBCL, NOS
BCL-34	chr3	16885062	16885062	G	A	exonic	PLCL2	8	11	0.5789	Missense_Mutation	p.G8D	Non-Targeted Panel Gene	Adult HGBCL, NOS
BCL-34	chr14	77482346	77482346	C	T	exonic	ISM2	14	20	0.5882	Missense_Mutation	p.V317I	Non-Targeted Panel Gene	Adult HGBCL, NOS
BCL-34	chrX	23736002	23736002	C	T	exonic	ACOT9	38	56	0.5957	Missense_Mutation	p.G12D	Non-Targeted Panel Gene	Adult HGBCL, NOS
BCL-34	chr12	7495116	7495116	C	T	exonic	CD163	24	38	0.6129	Missense_Mutation	p.C462Y	Non-Targeted Panel Gene	Adult HGBCL, NOS
BCL-34	chr6	89938148	89938148	T	C	exonic	BACH2	18	34	0.6538	Missense_Mutation	p.K680R	Non-Targeted Panel Gene	Adult HGBCL, NOS
BCL-34	chr19	34428497	34428497	T	A	exonic	UBA2	7	18	0.72	Missense_Mutation	p.V22E	Non-Targeted Panel Gene	Adult HGBCL, NOS
BCL-34	chr1	3430978	3430978	G	A	exonic	PRDM16	14	51	0.7846	Missense_Mutation	p.E1131K	Non-Targeted Panel Gene	Adult HGBCL, NOS
BCL-34	chr19	45713021	45713021	C	T	exonic	FBXO46	17	64	0.7901	Missense_Mutation	p.G159S	Non-Targeted Panel Gene	Adult HGBCL, NOS
BCL-34	chr1	11766604	11766604	G	A	exonic	C1orf167	19	113	0.8561	Missense_Mutation	p.G273D	Non-Targeted Panel Gene	Adult HGBCL, NOS
BCL-34	chr19	52951108	52951108	A	C	exonic	ZNF816	5	32	0.8649	Missense_Mutation	p.C223G	Non-Targeted Panel Gene	Adult HGBCL, NOS
BCL-34	chr19	45785839	45785839	C	T	exonic	DMWD	8	61	0.8841	Missense_Mutation	p.G553S	Non-Targeted Panel Gene	Adult HGBCL, NOS
BCL-34	chr22	37925771	37925771	A	G	exonic	MICALL1	0	38	1	Missense_Mutation	p.D398G	Non-Targeted Panel Gene	Adult HGBCL, NOS
BCL-34	chr22	45422301	45422301	A	G	exonic	RIBC2	0	129	1	Missense_Mutation	p.T190A	Non-Targeted Panel Gene	Adult HGBCL, NOS
BCL-34	chr1	149886512	149886512	G	C	exonic	HIST2H2BE	147	24	0.1404	Nonsense_Mutation	p.Y43X	Targerted Panel Gene	Adult HGBCL, NOS
BCL-34	chr4	105275983	105275983	C	T	exonic	TET2	210	62	0.2279	Nonsense_Mutation	p.Q1825X	Targerted Panel Gene	Adult HGBCL, NOS
BCL-34	chr1	100504891	100504891	A	T	exonic	CDC14A	6	9	0.6	Nonstop_Mutation	p.X611Y	Non-Targeted Panel Gene	Adult HGBCL, NOS
BCL-35	chr12	52381123	52381123	A	-	exonic	KRT84	83	68	0.4503	Frame_Shift_Del	p.L387fs	Non-Targeted Panel Gene	Adult HGBCL, NOS
BCL-35	chr1	28466459	28466459	A	-	exonic	PHACTR4	56	50	0.4717	Frame_Shift_Del	p.K180fs	Non-Targeted Panel Gene	Adult HGBCL, NOS
BCL-35	chr17	75617272	75617278	AGGACCC	-	exonic	MYO15B	19	20	0.5128	Frame_Shift_Del	p.E2223fs	Non-Targeted Panel Gene	Adult HGBCL, NOS
BCL-35	chrX	67545316	67545316	-	GCAGCA	exonic	AR	17	8	0.2963	In_Frame_Ins	p.L57delinsLQQ	Non-Targeted Panel Gene	Adult HGBCL, NOS
BCL-35	chr16	30724027	30724027	C	G	exonic	SRCAP	41	21	0.3387	Missense_Mutation	p.P1535A	Non-Targeted Panel Gene	Adult HGBCL, NOS
BCL-35	chr13	27920364	27920364	G	A	exonic	PDX1	52	35	0.4023	Missense_Mutation	p.D76N	Non-Targeted Panel Gene	Adult HGBCL, NOS
BCL-35	chr6	43782081	43782081	C	G	exonic	VEGFA	39	30	0.4348	Missense_Mutation	p.T387S	Non-Targeted Panel Gene	Adult HGBCL, NOS
BCL-35	chr17	45983906	45983906	G	T	exonic	MAPT	64	51	0.4397	Missense_Mutation	p.V368F	Non-Targeted Panel Gene	Adult HGBCL, NOS
BCL-35	chr19	6381470	6381470	C	T	exonic	GTF2F1	55	44	0.4444	Missense_Mutation	p.E303K	Non-Targeted Panel Gene	Adult HGBCL, NOS
BCL-35	chr19	49153871	49153871	C	T	exonic	HRC	84	69	0.451	Missense_Mutation	p.G456E	Non-Targeted Panel Gene	Adult HGBCL, NOS
BCL-35	chr11	118212513	118212513	G	T	exonic	JAML	62	52	0.4561	Missense_Mutation	p.T31K	Non-Targeted Panel Gene	Adult HGBCL, NOS
BCL-35	chr3	59047248	59047248	C	A	exonic	C3orf67	19	16	0.4571	Missense_Mutation	p.A10S	Non-Targeted Panel Gene	Adult HGBCL, NOS
BCL-35	chr5	149042718	149042718	A	G	exonic	SH3TC2	37	33	0.4714	Missense_Mutation	p.Y169H	Non-Targeted Panel Gene	Adult HGBCL, NOS
BCL-35	chr17	81809839	81809839	G	A	exonic	GCGR	48	43	0.4725	Missense_Mutation	p.G40S	Non-Targeted Panel Gene	Adult HGBCL, NOS
BCL-35	chr11	126271475	126271475	A	C	exonic	FOXRED1	78	72	0.4768	Missense_Mutation	p.K42Q	Non-Targeted Panel Gene	Adult HGBCL, NOS
BCL-35	chr3	11362900	11362900	A	G	exonic	ATG7	61	56	0.4786	Missense_Mutation	p.M591V	Non-Targeted Panel Gene	Adult HGBCL, NOS
BCL-35	chr9	134817038	134817038	C	T	exonic	COL5A1	41	38	0.481	Missense_Mutation	p.P1379S	Non-Targeted Panel Gene	Adult HGBCL, NOS
BCL-35	chr11	13366750	13366750	C	G	exonic	ARNTL	31	29	0.4833	Missense_Mutation	p.T170S	Non-Targeted Panel Gene	Adult HGBCL, NOS
BCL-35	chr10	96397209	96397209	T	G	exonic	TLL2	46	45	0.4945	Missense_Mutation	p.K454T	Non-Targeted Panel Gene	Adult HGBCL, NOS
BCL-35	chr8	143924876	143924876	G	A	exonic	PLEC	19	19	0.5	Missense_Mutation	p.R1712W	Non-Targeted Panel Gene	Adult HGBCL, NOS
BCL-35	chr6	43285312	43285312	A	T	exonic	TTBK1	32	36	0.5294	Missense_Mutation	p.Q1301L	Non-Targeted Panel Gene	Adult HGBCL, NOS
BCL-35	chr6	158503742	158503742	A	G	exonic	TULP4	95	107	0.5297	Missense_Mutation	p.K1360R	Non-Targeted Panel Gene	Adult HGBCL, NOS
BCL-35	chr2	195864771	195864771	A	G	exonic	DNAH7	34	39	0.5342	Missense_Mutation	p.I2295T	Non-Targeted Panel Gene	Adult HGBCL, NOS
BCL-35	chr12	52652138	52652138	C	G	exonic	KRT2	24	28	0.5385	Missense_Mutation	p.S2T	Non-Targeted Panel Gene	Adult HGBCL, NOS
BCL-35	chr17	75617176	75617176	A	G	exonic	MYO15B	35	42	0.5455	Missense_Mutation	p.K2191R	Non-Targeted Panel Gene	Adult HGBCL, NOS
BCL-35	chr3	57323493	57323493	A	C	exonic	DNAH12	23	28	0.549	Missense_Mutation	p.S3373A	Non-Targeted Panel Gene	Adult HGBCL, NOS
BCL-35	chr6	33443896	33443896	T	C	exonic	SYNGAP1	39	59	0.602	Missense_Mutation	p.I1115T	Non-Targeted Panel Gene	Adult HGBCL, NOS
BCL-35	chr13	69839002	69839002	G	A	exonic	KLHL1	8	13	0.619	Missense_Mutation	p.A463V	Non-Targeted Panel Gene	Adult HGBCL, NOS
BCL-36	chr9	98099993	98100000	TCATGATG	-	exonic	TRIM14	51	44	0.4632	Frame_Shift_Del	p.D156fs	Non-Targeted Panel Gene	Adult HGBCL, NOS
BCL-36	chr17	17796724	17796726	AGG	-	exonic	RAI1	118	98	0.4537	In_Frame_Del	p.1259_1260del	Non-Targeted Panel Gene	Adult HGBCL, NOS
BCL-36	chr19	45761789	45761789	-	TCCAGCTCCAGC	exonic	BHMG1	109	23	0.1742	In_Frame_Ins	p.P452delinsPSSSS	Non-Targeted Panel Gene	Adult HGBCL, NOS
BCL-36	chr1	197090909	197090909	G	A	exonic	ASPM	39	22	0.3607	Missense_Mutation	p.R3193C	Non-Targeted Panel Gene	Adult HGBCL, NOS
BCL-36	chr3	197161651	197161651	T	G	exonic	DLG1	39	24	0.381	Missense_Mutation	p.T191P	Non-Targeted Panel Gene	Adult HGBCL, NOS
BCL-36	chr4	55102430	55102430	G	A	exonic	KDR	59	38	0.3918	Missense_Mutation	p.T689M	Non-Targeted Panel Gene	Adult HGBCL, NOS
BCL-36	chr17	78560826	78560826	C	T	exonic	DNAH17	39	28	0.4179	Missense_Mutation	p.D649N	Non-Targeted Panel Gene	Adult HGBCL, NOS
BCL-36	chr7	87431528	87431528	C	T	exonic	ABCB4	45	33	0.4231	Missense_Mutation	p.R590Q	Non-Targeted Panel Gene	Adult HGBCL, NOS
BCL-36	chr7	144765897	144765897	C	T	exonic	TPK1	8	6	0.4286	Missense_Mutation	p.R33H	Non-Targeted Panel Gene	Adult HGBCL, NOS
BCL-36	chr9	97913416	97913416	G	C	exonic	TRMO	105	79	0.4293	Missense_Mutation	p.L132V	Non-Targeted Panel Gene	Adult HGBCL, NOS
BCL-36	chr13	99552234	99552234	C	T	exonic	TM9SF2	104	80	0.4348	Missense_Mutation	p.P466S	Non-Targeted Panel Gene	Adult HGBCL, NOS
BCL-36	chr7	84060554	84060554	T	C	exonic	SEMA3A	34	27	0.4426	Missense_Mutation	p.N153S	Non-Targeted Panel Gene	Adult HGBCL, NOS
BCL-36	chr5	134376671	134376671	C	T	exonic	UBE2B	19	16	0.4571	Missense_Mutation	p.P43L	Non-Targeted Panel Gene	Adult HGBCL, NOS
BCL-36	chr14	67805304	67805304	C	A	exonic	ZFYVE26	84	72	0.4615	Missense_Mutation	p.G395V	Non-Targeted Panel Gene	Adult HGBCL, NOS
BCL-36	chr6	143846068	143846068	G	T	exonic	LTV1	33	29	0.4677	Missense_Mutation	p.R51S	Non-Targeted Panel Gene	Adult HGBCL, NOS
BCL-36	chr8	144056492	144056492	C	T	exonic	OPLAH	160	141	0.4684	Missense_Mutation	p.E626K	Non-Targeted Panel Gene	Adult HGBCL, NOS
BCL-36	chr6	34155354	34155354	C	T	exonic	GRM4	10	9	0.4737	Missense_Mutation	p.G13S	Non-Targeted Panel Gene	Adult HGBCL, NOS
BCL-36	chr10	106688257	106688257	T	C	exonic	SORCS1	32	29	0.4754	Missense_Mutation	p.R499G	Non-Targeted Panel Gene	Adult HGBCL, NOS
BCL-36	chr12	8838341	8838341	C	A	exonic	A2ML1	33	31	0.4844	Missense_Mutation	p.D287E	Non-Targeted Panel Gene	Adult HGBCL, NOS
BCL-36	chr3	49675717	49675717	C	T	exonic	APEH	166	158	0.4877	Missense_Mutation	p.S99L	Non-Targeted Panel Gene	Adult HGBCL, NOS
BCL-36	chr19	1234252	1234252	C	T	exonic	CBARP	51	49	0.49	Missense_Mutation	p.G236D	Non-Targeted Panel Gene	Adult HGBCL, NOS
BCL-36	chr14	91313360	91313360	T	C	exonic	CCDC88C	58	59	0.5	Missense_Mutation	p.D819G	Non-Targeted Panel Gene	Adult HGBCL, NOS

BCL-36	chr2	80313616	80313616	A	G	exonic	CTNNA2	64	64	0.5	Missense_Mutation	p.M19V	Non-Targeted Panel Gene	Adult HGBCL, NOS
BCL-36	chr1	108983305	108983305	T	C	exonic	WDR47	55	56	0.5045	Missense_Mutation	p.N692S	Non-Targeted Panel Gene	Adult HGBCL, NOS
BCL-36	chr6	79916753	79916753	A	G	exonic	ELOVL4	20	21	0.5122	Missense_Mutation	p.I267T	Non-Targeted Panel Gene	Adult HGBCL, NOS
BCL-36	chr6	44254998	44254998	T	G	exonic	SLC35B2	225	237	0.513	Missense_Mutation	p.E336A	Non-Targeted Panel Gene	Adult HGBCL, NOS
BCL-36	chr14	24137366	24137366	T	G	exonic	PSME1	96	103	0.5176	Missense_Mutation	p.L61V	Non-Targeted Panel Gene	Adult HGBCL, NOS
BCL-36	chr16	23558279	23558279	G	C	exonic	UBFD1	63	68	0.5191	Missense_Mutation	p.G119R	Non-Targeted Panel Gene	Adult HGBCL, NOS
BCL-36	chr15	55371924	55371924	T	A	exonic	CCPG1	52	57	0.5229	Missense_Mutation	p.D192V	Non-Targeted Panel Gene	Adult HGBCL, NOS
BCL-36	chr6	151468416	151468416	T	C	exonic	ARMT1	16	18	0.5294	Missense_Mutation	p.L211P	Non-Targeted Panel Gene	Adult HGBCL, NOS
BCL-36	chr2	97815268	97815268	A	T	exonic	TMEM131	9	11	0.55	Missense_Mutation	p.I408K	Non-Targeted Panel Gene	Adult HGBCL, NOS
BCL-36	chr11	67310068	67310068	G	A	exonic	SSH3	72	89	0.5528	Missense_Mutation	p.R471H	Non-Targeted Panel Gene	Adult HGBCL, NOS
BCL-36	chr3	128486117	128486117	G	C	exonic	GATA2	29	36	0.5538	Missense_Mutation	p.P161A	Non-Targeted Panel Gene	Adult HGBCL, NOS
BCL-36	chr14	103337125	103337125	C	T	exonic	EIF5	8	12	0.6	Missense_Mutation	p.P113S	Non-Targeted Panel Gene	Adult HGBCL, NOS
BCL-36	chrX	50311090	50311090	G	A	exonic	CCNB3	1	110	0.991	Missense_Mutation	p.S974N	Non-Targeted Panel Gene	Adult HGBCL, NOS
BCL-36	chrX	64191491	64191491	T	C	exonic	AMER1	0	74	1	Missense_Mutation	p.Y599C	Non-Targeted Panel Gene	Adult HGBCL, NOS
BCL-36	chrX	49257547	49257547	G	A	exonic	FOXP3	0	37	1	Missense_Mutation	p.H112Y	Non-Targeted Panel Gene	Adult HGBCL, NOS
BCL-36	chr19	5866721	5866721	C	T	exonic	FUT5	52	77	0.5969	Nonsense_Mutation	p.W335X	Non-Targeted Panel Gene	Adult HGBCL, NOS
BCL-37	chr4	5460101	5460101	A	T	splicing	STK32B	71	18	0.2022	Splice_Site	.	Non-Targeted Panel Gene	Adult HGBCL, NOS
BCL-37	chr1	169596989	169596989	A	G	splicing	SELP	53	31	0.369	Splice_Site	.	Non-Targeted Panel Gene	Adult HGBCL, NOS
BCL-37	chr2	99560379	99560382	TTGT	-	exonic	AFF3	90	48	0.3478	Frame_Shift_Del	p.K1058fs	Non-Targeted Panel Gene	Adult HGBCL, NOS
BCL-37	chr11	60842491	60842491	-	A	exonic	CCDC86	64	88	0.5789	Frame_Shift_Ins	p.E123fs	Non-Targeted Panel Gene	Adult HGBCL, NOS
BCL-37	chr8	30849295	30849295	T	C	exonic	TEX15	44	5	0.102	Missense_Mutation	p.N291S	Non-Targeted Panel Gene	Adult HGBCL, NOS
BCL-37	chr3	26710022	26710022	C	T	exonic	LRRC3B	68	9	0.1169	Missense_Mutation	p.T117I	Non-Targeted Panel Gene	Adult HGBCL, NOS
BCL-37	chr4	65602150	65602150	A	C	exonic	EPHA5	79	11	0.1222	Missense_Mutation	p.L134R	Non-Targeted Panel Gene	Adult HGBCL, NOS
BCL-37	chr17	8111466	8111466	G	A	exonic	ALOXE3	88	14	0.1373	Missense_Mutation	p.P284S	Non-Targeted Panel Gene	Adult HGBCL, NOS
BCL-37	chr4	182754904	182754904	G	C	exonic	TENM3	74	12	0.1395	Missense_Mutation	p.V1513L	Non-Targeted Panel Gene	Adult HGBCL, NOS
BCL-37	chr6	15496610	15496610	G	C	exonic	JARID2	124	22	0.1507	Missense_Mutation	p.G462A	Non-Targeted Panel Gene	Adult HGBCL, NOS
BCL-37	chr11	123943723	123943723	G	T	exonic	OR6T1	212	44	0.1719	Missense_Mutation	p.A39D	Non-Targeted Panel Gene	Adult HGBCL, NOS
BCL-37	chr5	110618522	110618522	G	T	exonic	TMEM232	48	10	0.1724	Missense_Mutation	p.A270D	Non-Targeted Panel Gene	Adult HGBCL, NOS
BCL-37	chr18	27142725	27142725	T	C	exonic	CHST9	40	9	0.1837	Missense_Mutation	p.M29V	Non-Targeted Panel Gene	Adult HGBCL, NOS
BCL-37	chr1	210387504	210387504	T	C	exonic	HHAT	94	23	0.1966	Missense_Mutation	p.W66R	Non-Targeted Panel Gene	Adult HGBCL, NOS
BCL-37	chr22	37507842	37507842	T	G	exonic	CARD10	24	6	0.2	Missense_Mutation	p.K393T	Non-Targeted Panel Gene	Adult HGBCL, NOS
BCL-37	chr11	62705316	62705316	A	G	exonic	BSCL2	124	35	0.2201	Missense_Mutation	p.V66A	Non-Targeted Panel Gene	Adult HGBCL, NOS
BCL-37	chr6	117270846	117270846	C	T	exonic	VGLL2	24	7	0.2258	Missense_Mutation	p.A232V	Non-Targeted Panel Gene	Adult HGBCL, NOS
BCL-37	chr2	101387184	101387184	C	T	exonic	CREG2	27	10	0.2703	Missense_Mutation	p.A92T	Non-Targeted Panel Gene	Adult HGBCL, NOS
BCL-37	chr10	70266121	70266121	C	G	exonic	NPFFR1	195	73	0.2724	Missense_Mutation	p.G93A	Non-Targeted Panel Gene	Adult HGBCL, NOS
BCL-37	chr20	51676203	51676203	C	G	exonic	ATP9A	59	23	0.2805	Missense_Mutation	p.V269L	Non-Targeted Panel Gene	Adult HGBCL, NOS
BCL-37	chr8	21982755	21982755	C	T	exonic	XPO7	67	27	0.2872	Missense_Mutation	p.P407L	Non-Targeted Panel Gene	Adult HGBCL, NOS
BCL-37	chr2	240933251	240933251	C	A	exonic	CROCC2	42	17	0.2881	Missense_Mutation	p.Q458K	Non-Targeted Panel Gene	Adult HGBCL, NOS
BCL-37	chr14	102139972	102139972	T	A	exonic	WDR20	103	46	0.3087	Missense_Mutation	p.F17I	Non-Targeted Panel Gene	Adult HGBCL, NOS
BCL-37	chr12	80610581	80610581	A	G	exonic	PTPRQ	42	21	0.3333	Missense_Mutation	p.E1625G	Non-Targeted Panel Gene	Adult HGBCL, NOS
BCL-37	chr7	137016040	137016040	T	C	exonic	CHRM2	106	54	0.3375	Missense_Mutation	p.I392T	Non-Targeted Panel Gene	Adult HGBCL, NOS
BCL-37	chr3	112581120	112581120	A	G	exonic	SLC35A5	40	21	0.3443	Missense_Mutation	p.M335V	Non-Targeted Panel Gene	Adult HGBCL, NOS
BCL-37	chr7	89334787	89334787	T	G	exonic	ZNF804B	76	40	0.3448	Missense_Mutation	p.L602R	Non-Targeted Panel Gene	Adult HGBCL, NOS
BCL-37	chr10	100256404	100256404	T	C	exonic	CWF19L1	39	21	0.35	Missense_Mutation	p.E121G	Non-Targeted Panel Gene	Adult HGBCL, NOS
BCL-37	chr7	1496221	1496221	A	G	exonic	INTS1	86	47	0.3534	Missense_Mutation	p.M549T	Non-Targeted Panel Gene	Adult HGBCL, NOS
BCL-37	chr1	156658657	156658657	G	A	exonic	BCAN	181	101	0.3582	Missense_Mutation	p.R851H	Non-Targeted Panel Gene	Adult HGBCL, NOS
BCL-37	chr17	37207678	37207678	C	T	exonic	ACACA	96	54	0.36	Missense_Mutation	p.R1240Q	Non-Targeted Panel Gene	Adult HGBCL, NOS
BCL-37	chr8	144110556	144110556	G	A	exonic	WDR97	102	59	0.3665	Missense_Mutation	p.D687N	Non-Targeted Panel Gene	Adult HGBCL, NOS
BCL-37	chr2	206767112	206767112	C	G	exonic	FASTKD2	43	25	0.3676	Missense_Mutation	p.S140C	Non-Targeted Panel Gene	Adult HGBCL, NOS
BCL-37	chr17	44322845	44322845	C	A	exonic	SLC25A39	104	62	0.3735	Missense_Mutation	p.M51I	Non-Targeted Panel Gene	Adult HGBCL, NOS
BCL-37	chr13	57633942	57633942	A	T	exonic	PCDH17	214	129	0.3761	Missense_Mutation	p.N466Y	Non-Targeted Panel Gene	Adult HGBCL, NOS
BCL-37	chr1	204982002	204982002	G	A	exonic	NFASC	181	110	0.378	Missense_Mutation	p.G818S	Non-Targeted Panel Gene	Adult HGBCL, NOS
BCL-37	chr10	102475962	102475962	C	T	exonic	MFSD13A	68	42	0.3818	Missense_Mutation	p.T511I	Non-Targeted Panel Gene	Adult HGBCL, NOS
BCL-37	chr9	136013291	136013291	G	A	exonic	NACC2	17	11	0.3929	Missense_Mutation	p.T388M	Non-Targeted Panel Gene	Adult HGBCL, NOS
BCL-37	chr1	211978466	211978466	G	A	exonic	INTS7	109	73	0.4011	Missense_Mutation	p.L426F	Non-Targeted Panel Gene	Adult HGBCL, NOS
BCL-37	chr12	112252462	112252462	T	G	exonic	HECTD4	56	39	0.4105	Missense_Mutation	p.K1038Q	Non-Targeted Panel Gene	Adult HGBCL, NOS
BCL-37	chr1	1054941	1054941	C	T	exonic	AGRN	17	12	0.4138	Missense_Mutation	p.A2033V	Non-Targeted Panel Gene	Adult HGBCL, NOS
BCL-37	chr9	130362964	130362964	G	A	exonic	HMCN2	57	41	0.4184	Missense_Mutation	p.R2069H	Non-Targeted Panel Gene	Adult HGBCL, NOS
BCL-37	chr10	114299295	114299295	T	C	exonic	AFAP1L2	22	16	0.4211	Missense_Mutation	p.K664R	Non-Targeted Panel Gene	Adult HGBCL, NOS
BCL-37	chr5	141375270	141375270	A	T	exonic	PCDHGA6	50	37	0.4253	Missense_Mutation	p.K396I	Non-Targeted Panel Gene	Adult HGBCL, NOS
BCL-37	chr17	82062949	82062949	G	A	exonic	DUS1L	96	72	0.4286	Missense_Mutation	p.S141F	Non-Targeted Panel Gene	Adult HGBCL, NOS
BCL-37	chr15	73702921	73702921	G	T	exonic	CD276	65	56	0.4628	Missense_Mutation	p.V190L	Non-Targeted Panel Gene	Adult HGBCL, NOS
BCL-37	chr17	61847211	61847211	G	A	exonic	BRIP1	16	14	0.4667	Missense_Mutation	p.R173C	Non-Targeted Panel Gene	Adult HGBCL, NOS
BCL-37	chr2	218679575	218679575	C	G	exonic	STK36	121	106	0.467	Missense_Mutation	p.A265G	Non-Targeted Panel Gene	Adult HGBCL, NOS
BCL-37	chr15	82538329	82538329	T	C	exonic	RPS17	83	73	0.4679	Missense_Mutation	p.T102A	Non-Targeted Panel Gene	Adult HGBCL, NOS
BCL-37	chr3	74334896	74334896	T	C	exonic	CNTN3	53	49	0.4804	Missense_Mutation	p.T503A	Non-Targeted Panel Gene	Adult HGBCL, NOS
BCL-37	chr22	18126361	18126361	C	T	exonic	TUBA8	46	48	0.5106	Missense_Mutation	p.A128V	Non-Targeted Panel Gene	Adult HGBCL, NOS
BCL-37	chr3	165032541	165032541	G	C	exonic	SI	15	16	0.5161	Missense_Mutation	p.T906S	Non-Targeted Panel Gene	Adult HGBCL, NOS
BCL-37	chr2	202467669	202467669	C	T	exonic	BMPR2	22	24	0.5217	Missense_Mutation	p.P133L	Non-Targeted Panel Gene	Adult HGBCL, NOS
BCL-37	chr17	61808730	61808730	A	G	exonic	BRIP1	30	33	0.5238	Missense_Mutation	p.C219R	Non-Targeted Panel Gene	Adult HGBCL, NOS
BCL-37	chr6	139373580	139373580	T	C	exonic	CITED2	78	86	0.5244	Missense_Mutation	p.Q122R	Non-Targeted Panel Gene	Adult HGBCL, NOS
BCL-37	chrX	75784214	75784214	T	C	exonic	MAGEE2	34	42	0.5526	Missense_Mutation	p.S280G	Non-Targeted Panel Gene	Adult HGBCL, NOS
BCL-37	chr6	89868323	89868323	A	G	exonic	CASP8AP2	29	41	0.5857	Missense_Mutation	p.H1678R	Non-Targeted Panel Gene	Adult HGBCL, NOS
BCL-37	chr4	69639507	69639507	A	T	exonic	UGT2A1;UGT2A2	20	31	0.6078	Missense_Mutation	p.I246N	Non-Targeted Panel Gene	Adult HGBCL, NOS
BCL-37	chr5	90461508	90461508	T	C	exonic	MBLAC2	43	82	0.656	Missense_Mutation	p.M167V	Non-Targeted Panel Gene	Adult HGBCL, NOS
BCL-37	chr4	40893383	40893383	A	G	exonic	APBB2	31	69	0.69	Missense_Mutation	p.V428A	Non-Targeted Panel Gene	Adult HGBCL, NOS
BCL-37	chr9	19080561	19080561	C	T	exonic	HAUS6	17	41	0.7069	Missense_Mutation	p.A328T	Non-Targeted Panel Gene	Adult HGBCL, NOS
BCL-37	chr4	530650	530650	C	G	exonic	PIGG	16	41	0.7193	Missense_Mutation	p.L460V	Non-Targeted Panel Gene	Adult HGBCL, NOS
BCL-37	chr11	104037404	104037404	G	C	exonic	DDI1	112	341	0.7528	Missense_Mutation	p.E194D	Non-Targeted Panel Gene	Adult HGBCL, NOS
BCL-37	chrX	154364590	154364590	T	C	exonic	FLNA	46	190	0.8051	Missense_Mutation	p.D653G	Non-Targeted Panel Gene	Adult HGBCL, NOS
BCL-37	chr18	43273632	43273632	A	C	exonic	SYT4	54	10	0.1562	Nonsense_Mutation	p.L266X	Non-Targeted Panel Gene	Adult HGBCL, NOS
BCL-37	chr8	16155085	16155085	G	A	exonic	MSR1	33	12	0.2667	Nonsense_Mutation	p.R293X	Non-Targeted Panel Gene	Adult HGBCL, NOS

BCL-37	chr11	44936899	44936899	C	T	exonic	TP53I11	58	22	0.275	Nonsense_Mutation	p.W30X	Non-Targeted Panel Gene	Adult HGBCL, NOS
BCL-37	chr5	116010890	116010890	T	A	exonic	LVRN	20	8	0.2857	Nonsense_Mutation	p.L748X	Non-Targeted Panel Gene	Adult HGBCL, NOS
BCL-37	chr5	160370571	160370571	C	A	exonic	C1QTNF2	17	7	0.2917	Nonsense_Mutation	p.E23X	Non-Targeted Panel Gene	Adult HGBCL, NOS
BCL-37	chr9	101429901	101429901	G	A	exonic	ALDOB	92	97	0.5132	Nonsense_Mutation	p.R60X	Non-Targeted Panel Gene	Adult HGBCL, NOS
BCL-37	chr17	18277777	18277777	G	A	exonic	TOP3A	70	97	0.5808	Nonsense_Mutation	p.Q909X	Non-Targeted Panel Gene	Adult HGBCL, NOS
BCL-37	chr11	118212472	118212472	G	A	exonic	JAML	53	123	0.6989	Nonsense_Mutation	p.Q45X	Non-Targeted Panel Gene	Adult HGBCL, NOS
BCL-38	chr19	45872511	45872512	TG	-	exonic	FOXA3	109	96	0.4683	Frame_Shift_Del	p.L169fs	Non-Targeted Panel Gene	Adult HGBCL, NOS
BCL-38	chr3	20114897	20114899	AGA	-	exonic	KAT2B	56	33	0.3708	In_Frame_Del	p.353_354del	Non-Targeted Panel Gene	Adult HGBCL, NOS
BCL-38	chr1	150226676	150226681	CTTCAT	-	exonic	ANP32E	10	8	0.4444	In_Frame_Del	p.203_205del	Non-Targeted Panel Gene	Adult HGBCL, NOS
BCL-38	chr4	87616155	87616163	AGCAGCGAC	-	exonic	DSPP	4	6	0.6	In_Frame_Del	p.1165_1167del	Non-Targeted Panel Gene	Adult HGBCL, NOS
BCL-38	chr5	108381011	108381011	-	CCG	exonic	FBXL17	11	5	0.3125	In_Frame_Ins	p.G227delinsGG	Non-Targeted Panel Gene	Adult HGBCL, NOS
BCL-38	chr5	115543102	115543102	A	T	exonic	FEM1C	127	7	0.0522	Missense_Mutation	p.I131K	Non-Targeted Panel Gene	Adult HGBCL, NOS
BCL-38	chr7	122010986	122010986	C	A	exonic	PTPRZ1	163	10	0.0578	Missense_Mutation	p.S647Y	Non-Targeted Panel Gene	Adult HGBCL, NOS
BCL-38	chr5	115543193	115543193	A	C	exonic	FEM1C	157	12	0.071	Missense_Mutation	p.S101A	Non-Targeted Panel Gene	Adult HGBCL, NOS
BCL-38	chr5	154891441	154891441	A	T	exonic	GEMIN5	111	13	0.1048	Missense_Mutation	p.S1354R	Non-Targeted Panel Gene	Adult HGBCL, NOS
BCL-38	chr19	3148707	3148707	G	C	exonic	GNA15	59	7	0.1061	Missense_Mutation	p.V88L	Non-Targeted Panel Gene	Adult HGBCL, NOS
BCL-38	chr15	36644286	36644286	A	G	exonic	C15orf41	38	13	0.2549	Missense_Mutation	p.Q37R	Non-Targeted Panel Gene	Adult HGBCL, NOS
BCL-38	chr7	135364021	135364021	T	C	exonic	CNOT4	110	47	0.2994	Missense_Mutation	p.D558G	Non-Targeted Panel Gene	Adult HGBCL, NOS
BCL-38	chr10	119826953	119826953	T	C	exonic	INPP5F	105	54	0.3396	Missense_Mutation	p.Y858H	Non-Targeted Panel Gene	Adult HGBCL, NOS
BCL-38	chr13	51799629	51799629	T	C	exonic	DHRS12	69	50	0.4202	Missense_Mutation	p.M11V	Non-Targeted Panel Gene	Adult HGBCL, NOS
BCL-38	chr21	33534666	33534666	G	A	exonic	GART	44	33	0.4286	Missense_Mutation	p.A110V	Non-Targeted Panel Gene	Adult HGBCL, NOS
BCL-38	chr4	168237414	168237414	T	C	exonic	DDX60	13	10	0.4348	Missense_Mutation	p.Q1428R	Non-Targeted Panel Gene	Adult HGBCL, NOS
BCL-38	chr7	142946253	142946253	G	A	exonic	KEL	65	50	0.4348	Missense_Mutation	p.A423V	Non-Targeted Panel Gene	Adult HGBCL, NOS
BCL-38	chr19	19637531	19637531	C	A	exonic	GMIP	38	30	0.4412	Missense_Mutation	p.A320S	Non-Targeted Panel Gene	Adult HGBCL, NOS
BCL-38	chr14	39040770	39040770	T	C	exonic	SEC23A	58	47	0.4434	Missense_Mutation	p.M702V	Non-Targeted Panel Gene	Adult HGBCL, NOS
BCL-38	chr17	50189878	50189878	C	T	exonic	COL1A1	81	65	0.4452	Missense_Mutation	p.R865H	Non-Targeted Panel Gene	Adult HGBCL, NOS
BCL-38	chr19	16471929	16471929	A	T	exonic	EPS15L1	6	5	0.4545	Missense_Mutation	p.I6N	Non-Targeted Panel Gene	Adult HGBCL, NOS
BCL-38	chr14	73478625	73478625	T	C	exonic	HEATR4	54	45	0.4545	Missense_Mutation	p.K1021R	Non-Targeted Panel Gene	Adult HGBCL, NOS
BCL-38	chr8	144512318	144512318	C	T	exonic	RECQL4	114	97	0.4575	Missense_Mutation	p.R1021Q	Non-Targeted Panel Gene	Adult HGBCL, NOS
BCL-38	chr21	46402480	46402480	G	T	exonic	PCNT	40	34	0.4595	Missense_Mutation	p.L1704F	Non-Targeted Panel Gene	Adult HGBCL, NOS
BCL-38	chr5	151343594	151343594	C	A	exonic	SLC36A2	168	143	0.4598	Missense_Mutation	p.G87V	Non-Targeted Panel Gene	Adult HGBCL, NOS
BCL-38	chr8	24953563	24953563	C	T	exonic	NEFL	34	30	0.4688	Missense_Mutation	p.D468N	Non-Targeted Panel Gene	Adult HGBCL, NOS
BCL-38	chr17	34992014	34992014	T	C	exonic	LIG3	87	77	0.4695	Missense_Mutation	p.M422T	Non-Targeted Panel Gene	Adult HGBCL, NOS
BCL-38	chr12	48149754	48149754	A	G	exonic	ASB8	101	91	0.474	Missense_Mutation	p.M99T	Non-Targeted Panel Gene	Adult HGBCL, NOS
BCL-38	chr1	196998292	196998292	G	C	exonic	CFHR5	27	25	0.4808	Missense_Mutation	p.V379L	Non-Targeted Panel Gene	Adult HGBCL, NOS
BCL-38	chr19	41220708	41220708	C	T	exonic	AXL	82	78	0.4875	Missense_Mutation	p.T53I	Non-Targeted Panel Gene	Adult HGBCL, NOS
BCL-38	chr6	26033421	26033421	C	A	exonic	HIST1H2AB	97	93	0.4895	Missense_Mutation	p.V50L	Non-Targeted Panel Gene	Adult HGBCL, NOS
BCL-38	chr2	181918840	181918840	G	T	exonic	SSFA2	49	47	0.4896	Missense_Mutation	p.R984L	Non-Targeted Panel Gene	Adult HGBCL, NOS
BCL-38	chr12	29536292	29536292	T	A	exonic	TMTC1	29	29	0.5	Missense_Mutation	p.I460F	Non-Targeted Panel Gene	Adult HGBCL, NOS
BCL-38	chr11	19192439	19192439	A	G	exonic	CSRP3	77	81	0.5094	Missense_Mutation	p.W4R	Non-Targeted Panel Gene	Adult HGBCL, NOS
BCL-38	chr12	45852828	45852828	G	A	exonic	ARID2	73	76	0.5101	Missense_Mutation	p.A1569T	Non-Targeted Panel Gene	Adult HGBCL, NOS
BCL-38	chrX	115306578	115306578	T	A	exonic	LUZP4	88	92	0.5111	Missense_Mutation	p.V239E	Non-Targeted Panel Gene	Adult HGBCL, NOS
BCL-38	chr6	73407605	73407605	G	A	exonic	DDX43	20	21	0.5122	Missense_Mutation	p.G343R	Non-Targeted Panel Gene	Adult HGBCL, NOS
BCL-38	chr19	49183151	49183151	A	C	exonic	TRPM4	81	87	0.5179	Missense_Mutation	p.D561A	Non-Targeted Panel Gene	Adult HGBCL, NOS
BCL-38	chr20	63350059	63350059	G	A	exonic	CHRNA4	37	40	0.5195	Missense_Mutation	p.P275L	Non-Targeted Panel Gene	Adult HGBCL, NOS
BCL-38	chr6	30952984	30952984	T	C	exonic	DPCR1	93	101	0.5206	Missense_Mutation	p.M1350T	Non-Targeted Panel Gene	Adult HGBCL, NOS
BCL-38	chr16	87711408	87711408	C	A	exonic	KLHDC4	71	78	0.5235	Missense_Mutation	p.V291F	Non-Targeted Panel Gene	Adult HGBCL, NOS
BCL-38	chr21	46002580	46002580	G	C	exonic	COL6A1	86	96	0.5275	Missense_Mutation	p.Q768H	Non-Targeted Panel Gene	Adult HGBCL, NOS
BCL-38	chr16	83994324	83994324	C	T	exonic	NECAB2	74	87	0.5404	Missense_Mutation	p.H207Y	Non-Targeted Panel Gene	Adult HGBCL, NOS
BCL-38	chr22	32513700	32513700	A	C	exonic	SYN3	95	126	0.5701	Missense_Mutation	p.S578A	Non-Targeted Panel Gene	Adult HGBCL, NOS
BCL-38	chr22	32498193	32498193	T	G	exonic	FBXO7	56	75	0.5725	Missense_Mutation	p.F411C	Non-Targeted Panel Gene	Adult HGBCL, NOS
BCL-38	chr2	218072486	218072486	G	T	exonic	RUFY4	39	55	0.5851	Missense_Mutation	p.R89L	Non-Targeted Panel Gene	Adult HGBCL, NOS
BCL-38	chr1	81984622	81984622	T	C	exonic	ADGRL2	44	67	0.6036	Missense_Mutation	p.C1108R	Non-Targeted Panel Gene	Adult HGBCL, NOS
BCL-38	chrX	11254686	11254686	T	C	exonic	ARHGAP6	1	93	0.9894	Missense_Mutation	p.M204V	Non-Targeted Panel Gene	Adult HGBCL, NOS
BCL-38	chr5	114496108	114496108	G	T	exonic	KCNN2	81	65	0.4452	Nonsense_Mutation	p.E556X	Non-Targeted Panel Gene	Adult HGBCL, NOS
BCL-38	chr14	50915869	50915869	G	A	exonic	PYGL	41	35	0.4605	Nonsense_Mutation	p.R399X	Non-Targeted Panel Gene	Adult HGBCL, NOS
BCL-39	chr3	184357095	184357095	C	A	splicing	CLCN2	88	79	0.4731	Splice_Site	.	Non-Targeted Panel Gene	Adult HGBCL, NOS
BCL-39	chr7	95804792	95804796	AAGAG	-	exonic	DYNC1I1	42	13	0.2364	Frame_Shift_Del	p.I21fs	Non-Targeted Panel Gene	Adult HGBCL, NOS
BCL-39	chr6	28125701	28125701	A	-	exonic	ZSCAN16	79	65	0.4514	Frame_Shift_Del	p.L86fs	Non-Targeted Panel Gene	Adult HGBCL, NOS
BCL-39	chr7	100201116	100201116	A	-	exonic	STAG3	55	103	0.6519	Frame_Shift_Del	p.V696fs	Non-Targeted Panel Gene	Adult HGBCL, NOS
BCL-39	chr10	122698036	122698036	-	T	exonic	C10orf120	63	5	0.0735	Frame_Shift_Ins	p.N235fs	Non-Targeted Panel Gene	Adult HGBCL, NOS
BCL-39	chr20	23633947	23633947	-	C	exonic	CST3	99	78	0.4407	Frame_Shift_Ins	p.T137fs	Non-Targeted Panel Gene	Adult HGBCL, NOS
BCL-39	chr19	44156475	44156475	-	A	exonic	ZNF234	35	44	0.557	Frame_Shift_Ins	p.Y153fs	Non-Targeted Panel Gene	Adult HGBCL, NOS
BCL-39	chr3	52818266	52818266	-	GGTTGT	exonic	ITIH4	65	34	0.3434	In_Frame_Ins	p.Q724delinsTTQ	Non-Targeted Panel Gene	Adult HGBCL, NOS
BCL-39	chr7	101089039	101089039	-	CGG	exonic	TRIM56	111	125	0.5252	In_Frame_Ins	p.N576delinsNG	Non-Targeted Panel Gene	Adult HGBCL, NOS
BCL-39	chr1	13420819	13420819	C	T	exonic	PRAMEF20	177	11	0.0585	Missense_Mutation	p.T330I	Non-Targeted Panel Gene	Adult HGBCL, NOS
BCL-39	chr3	81578033	81578033	T	G	exonic	GBE1	29	6	0.1714	Missense_Mutation	p.S504R	Non-Targeted Panel Gene	Adult HGBCL, NOS
BCL-39	chr7	5377464	5377464	G	A	exonic	TNRC18	88	25	0.2212	Missense_Mutation	p.R790C	Non-Targeted Panel Gene	Adult HGBCL, NOS
BCL-39	chr6	111387798	111387798	C	G	exonic	REV3L	26	8	0.2353	Missense_Mutation	p.V277L	Non-Targeted Panel Gene	Adult HGBCL, NOS
BCL-39	chr1	230911490	230911490	C	T	exonic	TTC13	11	4	0.2667	Missense_Mutation	p.M763I	Non-Targeted Panel Gene	Adult HGBCL, NOS
BCL-39	chr3	38551486	38551486	C	T	exonic	SCN5A	127	48	0.2743	Missense_Mutation	p.R1629Q	Non-Targeted Panel Gene	Adult HGBCL, NOS
BCL-39	chr2	60546082	60546082	T	C	exonic	BCL11A	80	34	0.2982	Missense_Mutation	p.M92V	Non-Targeted Panel Gene	Adult HGBCL, NOS
BCL-39	chr5	151343594	151343594	C	A	exonic	SLC36A2	211	105	0.3323	Missense_Mutation	p.G87V	Non-Targeted Panel Gene	Adult HGBCL, NOS
BCL-39	chr14	23415054	23415054	C	T	exonic	MYH7	68	34	0.3333	Missense_Mutation	p.A1834T	Non-Targeted Panel Gene	Adult HGBCL, NOS
BCL-39	chr12	52951512	52951512	G	C	exonic	KRT18	45	23	0.3382	Missense_Mutation	p.S230T	Non-Targeted Panel Gene	Adult HGBCL, NOS
BCL-39	chr17	64072022	64072022	C	T	exonic	ERN1	59	31	0.3444	Missense_Mutation	p.S146N	Non-Targeted Panel Gene	Adult HGBCL, NOS
BCL-39	chr12	6856044	6856044	A	G	exonic	USP5	178	94	0.3456	Missense_Mutation	p.E111G	Non-Targeted Panel Gene	Adult HGBCL, NOS
BCL-39	chr5	90652372	90652372	G	A	exonic	ADGRV1	103	59	0.3642	Missense_Mutation	p.G1148D	Non-Targeted Panel Gene	Adult HGBCL, NOS
BCL-39	chr12	124814270	124814270	C	T	exonic	SCARB1	210	121	0.3656	Missense_Mutation	p.V188M	Non-Targeted Panel Gene	Adult HGBCL, NOS
BCL-39	chr2	178555065	178555065	G	A	exonic	TTN	50	29	0.3671	Missense_Mutation	p.S29465F	Non-Targeted Panel Gene	Adult HGBCL, NOS
BCL-39	chr1	243426536	243426536	G	A	exonic	SDCCAG8	32	21	0.3962	Missense_Mutation	p.V354I	Non-Targeted Panel Gene	Adult HGBCL, NOS
BCL-39	chr5	95522240	95522240	A	C	exonic	TTC37	49	34	0.4096	Missense_Mutation	p.L609V	Non-Targeted Panel Gene	Adult HGBCL, NOS

BCL-39	chr12	52675506	52675506	G	A	exonic	KRT1	32	23	0.4182	Missense_Mutation	p.S541F	Non-Targeted Panel Gene	Adult HGBCL, NOS
BCL-39	chr9	4544579	4544579	G	A	exonic	SLC1A1	30	23	0.434	Missense_Mutation	p.G35E	Non-Targeted Panel Gene	Adult HGBCL, NOS
BCL-39	chr3	184337703	184337703	C	G	exonic	FAM131A	94	73	0.4371	Missense_Mutation	p.Q25E	Non-Targeted Panel Gene	Adult HGBCL, NOS
BCL-39	chr11	83169402	83169402	C	G	exonic	PCF11	139	108	0.4372	Missense_Mutation	p.P1023T	Non-Targeted Panel Gene	Adult HGBCL, NOS
BCL-39	chr14	73502964	73502964	T	A	exonic	HEATR4	28	23	0.451	Missense_Mutation	p.E679V	Non-Targeted Panel Gene	Adult HGBCL, NOS
BCL-39	chr5	58457561	58457561	A	T	exonic	PLK2	56	46	0.451	Missense_Mutation	p.Y232N	Non-Targeted Panel Gene	Adult HGBCL, NOS
BCL-39	chr20	23633948	23633948	T	A	exonic	CST3	95	79	0.454	Missense_Mutation	p.T137S	Non-Targeted Panel Gene	Adult HGBCL, NOS
BCL-39	chr4	70783140	70783140	G	A	exonic	RUFY3	19	17	0.4722	Missense_Mutation	p.R262Q	Non-Targeted Panel Gene	Adult HGBCL, NOS
BCL-39	chr3	105702188	105702188	C	G	exonic	CBLB	82	74	0.4744	Missense_Mutation	p.S226T	Non-Targeted Panel Gene	Adult HGBCL, NOS
BCL-39	chr9	36246396	36246396	A	G	exonic	GNE	41	37	0.4744	Missense_Mutation	p.M84T	Non-Targeted Panel Gene	Adult HGBCL, NOS
BCL-39	chr17	66688414	66688414	A	T	exonic	PRKCA	62	56	0.4746	Missense_Mutation	p.M267L	Non-Targeted Panel Gene	Adult HGBCL, NOS
BCL-39	chr11	124870060	124870060	T	C	exonic	ROBO3	107	98	0.478	Missense_Mutation	p.M253T	Non-Targeted Panel Gene	Adult HGBCL, NOS
BCL-39	chr16	30724027	30724027	C	G	exonic	SRCAP	24	22	0.4783	Missense_Mutation	p.P1535A	Non-Targeted Panel Gene	Adult HGBCL, NOS
BCL-39	chr21	34370500	34370500	A	G	exonic	KCNE2	49	45	0.4787	Missense_Mutation	p.T8A	Non-Targeted Panel Gene	Adult HGBCL, NOS
BCL-39	chr14	76499932	76499932	T	C	exonic	ESRRB	39	36	0.48	Missense_Mutation	p.F456L	Non-Targeted Panel Gene	Adult HGBCL, NOS
BCL-39	chr4	73853400	73853400	C	G	exonic	PF4V1	66	62	0.4844	Missense_Mutation	p.T13S	Non-Targeted Panel Gene	Adult HGBCL, NOS
BCL-39	chr19	11832905	11832905	G	C	exonic	ZNF440	72	68	0.4857	Missense_Mutation	p.G577R	Non-Targeted Panel Gene	Adult HGBCL, NOS
BCL-39	chr18	26862459	26862459	A	G	exonic	AQP4	100	95	0.4872	Missense_Mutation	p.I57T	Non-Targeted Panel Gene	Adult HGBCL, NOS
BCL-39	chr13	28197254	28197254	A	G	exonic	PAN3	42	40	0.4878	Missense_Mutation	p.K254E	Non-Targeted Panel Gene	Adult HGBCL, NOS
BCL-39	chr2	232542939	232542939	C	G	exonic	CHRNG	23	22	0.4889	Missense_Mutation	p.A221G	Non-Targeted Panel Gene	Adult HGBCL, NOS
BCL-39	chr10	116860871	116860871	A	G	exonic	ENO4	42	41	0.494	Missense_Mutation	p.I238V	Non-Targeted Panel Gene	Adult HGBCL, NOS
BCL-39	chr14	51722025	51722025	G	T	exonic	FRMD6	47	47	0.5	Missense_Mutation	p.M471I	Non-Targeted Panel Gene	Adult HGBCL, NOS
BCL-39	chr19	15180702	15180702	T	C	exonic	NOTCH3	53	53	0.5	Missense_Mutation	p.R1041G	Non-Targeted Panel Gene	Adult HGBCL, NOS
BCL-39	chr6	84762715	84762715	G	A	exonic	TBX18	12	12	0.5	Missense_Mutation	p.S109F	Non-Targeted Panel Gene	Adult HGBCL, NOS
BCL-39	chr6	33443896	33443896	T	C	exonic	SYNGAP1	38	39	0.5065	Missense_Mutation	p.I1115T	Non-Targeted Panel Gene	Adult HGBCL, NOS
BCL-39	chr20	45967623	45967623	C	T	exonic	ZNF335	54	56	0.5091	Missense_Mutation	p.A276T	Non-Targeted Panel Gene	Adult HGBCL, NOS
BCL-39	chr19	13918779	13918779	C	T	exonic	CC2D1A	21	23	0.5227	Missense_Mutation	p.S327L	Non-Targeted Panel Gene	Adult HGBCL, NOS
BCL-39	chr9	124799309	124799309	G	A	exonic	OLFML2A	76	85	0.528	Missense_Mutation	p.E163K	Non-Targeted Panel Gene	Adult HGBCL, NOS
BCL-39	chr20	45314518	45314518	G	A	exonic	RBPJL	50	57	0.5327	Missense_Mutation	p.G325S	Non-Targeted Panel Gene	Adult HGBCL, NOS
BCL-39	chr16	4681769	4681769	C	A	exonic	MGRN1	37	45	0.5488	Missense_Mutation	p.P451T	Non-Targeted Panel Gene	Adult HGBCL, NOS
BCL-39	chr16	13947713	13947713	T	C	exonic	ERCC4	79	99	0.5562	Missense_Mutation	p.I706T	Non-Targeted Panel Gene	Adult HGBCL, NOS
BCL-39	chr16	87603487	87603487	G	A	exonic	JPH3	10	13	0.5652	Missense_Mutation	p.G114E	Non-Targeted Panel Gene	Adult HGBCL, NOS
BCL-39	chr21	39187213	39187213	C	A	exonic	BRWD1	19	25	0.5682	Missense_Mutation	p.W2259L	Non-Targeted Panel Gene	Adult HGBCL, NOS
BCL-39	chr13	110893154	110893154	A	G	exonic	ANKRD10	27	36	0.5714	Missense_Mutation	p.L220P	Non-Targeted Panel Gene	Adult HGBCL, NOS
BCL-39	chr12	120446750	120446750	A	G	exonic	GATC	68	94	0.5802	Missense_Mutation	p.K59E	Non-Targeted Panel Gene	Adult HGBCL, NOS
BCL-39	chr12	132727423	132727423	T	A	exonic	ANKLE2	83	145	0.636	Missense_Mutation	p.K879I	Non-Targeted Panel Gene	Adult HGBCL, NOS
BCL-39	chr12	121020962	121020962	T	C	exonic	OASL	65	117	0.6429	Missense_Mutation	p.K382E	Non-Targeted Panel Gene	Adult HGBCL, NOS
BCL-39	chr7	155739808	155739808	C	G	exonic	RBM33	7	20	0.7407	Missense_Mutation	p.P611A	Non-Targeted Panel Gene	Adult HGBCL, NOS
BCL-39	chrX	43743810	43743810	G	A	exonic	MAOA	0	214	1	Missense_Mutation	p.V294M	Non-Targeted Panel Gene	Adult HGBCL, NOS
BCL-39	chr12	13564318	13564318	G	A	exonic	GRIN2B	264	78	0.2281	Nonsense_Mutation	p.Q974X	Non-Targeted Panel Gene	Adult HGBCL, NOS
BCL-39	chr22	50286226	50286226	G	T	exonic	PLXNB2	101	85	0.457	Nonsense_Mutation	p.Y608X	Non-Targeted Panel Gene	Adult HGBCL, NOS
BCL-39	chr20	23633961	23633961	C	T	exonic	CST3	96	83	0.4637	Nonsense_Mutation	p.W132X	Non-Targeted Panel Gene	Adult HGBCL, NOS
BCL-39	chr19	2328747	2328747	T	A	exonic	SPPL2B	18	25	0.5814	Nonsense_Mutation	p.L13X	Non-Targeted Panel Gene	Adult HGBCL, NOS
BCL-4	chr3	65470485	65470485	C	G	splicing	MAGI1	55	38	0.4086	Splice_Site	.	Targerted Panel Gene	Adult HGBCL, NOS
BCL-4	chr14	20999562	20999562	G	A	splicing	SLC39A2	53	3	0.0536	Splice_Site	.	Non-Targeted Panel Gene	Adult HGBCL, NOS
BCL-4	chr15	94340277	94340277	T	C	splicing	MCTP2	46	5	0.098	Splice_Site	.	Non-Targeted Panel Gene	Adult HGBCL, NOS
BCL-4	chr7	99042117	99042117	C	T	splicing	SMURF1	14	2	0.125	Splice_Site	.	Non-Targeted Panel Gene	Adult HGBCL, NOS
BCL-4	chr20	49010406	49010406	T	C	splicing	ARFGEF2	68	11	0.1392	Splice_Site	.	Non-Targeted Panel Gene	Adult HGBCL, NOS
BCL-4	chr9	133636712	133636712	T	C	splicing	DBH	45	9	0.1667	Splice_Site	.	Non-Targeted Panel Gene	Adult HGBCL, NOS
BCL-4	chr17	75902019	75902019	C	T	splicing	MRPL38	77	19	0.1979	Splice_Site	.	Non-Targeted Panel Gene	Adult HGBCL, NOS
BCL-4	chr14	20380252	20380258	GGTCCGG	-	exonic	TEP1	102	27	0.2093	Frame_Shift_Del	p.P1660fs	Non-Targeted Panel Gene	Adult HGBCL, NOS
BCL-4	chr17	15975032	15975033	TC	-	exonic	ADORA2B	98	32	0.2462	Frame_Shift_Del	p.I230fs	Non-Targeted Panel Gene	Adult HGBCL, NOS
BCL-4	chr13	73015872	73015872	A	-	exonic	PIBF1	19	8	0.2963	Frame_Shift_Del	p.K772fs	Non-Targeted Panel Gene	Adult HGBCL, NOS
BCL-4	chr2	100477256	100477257	AT	-	exonic	NMS	17	10	0.3704	Frame_Shift_Del	p.I66fs	Non-Targeted Panel Gene	Adult HGBCL, NOS
BCL-4	chr7	34844942	34844942	A	-	exonic	NPSR1	13	10	0.4348	Frame_Shift_Del	p.A268fs	Non-Targeted Panel Gene	Adult HGBCL, NOS
BCL-4	chr1	145848392	145848396	TAAAA	-	exonic	NUDT17	57	69	0.5476	Frame_Shift_Del	p.C300fs	Non-Targeted Panel Gene	Adult HGBCL, NOS
BCL-4	chr4	71236634	71236636	GAA	-	exonic	SLC4A4	58	15	0.2055	In_Frame_Del	p.20_20del	Non-Targeted Panel Gene	Adult HGBCL, NOS
BCL-4	chr17	15975035	15975035	-	TGG	exonic	ADORA2B	93	54	0.3673	In_Frame_Ins	p.H231delinsHG	Non-Targeted Panel Gene	Adult HGBCL, NOS
BCL-4	chr2	21026945	21026945	C	G	exonic	APOB	82	41	0.3333	Missense_Mutation	p.G696A	Targerted Panel Gene	Adult HGBCL, NOS
BCL-4	chr3	135133010	135133010	C	A	exonic	EPHB1	58	39	0.4021	Missense_Mutation	p.P420T	Targerted Panel Gene	Adult HGBCL, NOS
BCL-4	chr17	7675136	7675136	G	T	exonic	TP53	38	29	0.4328	Missense_Mutation	p.A120D	Targerted Panel Gene	Adult HGBCL, NOS
BCL-4	chr17	7675209	7675209	A	G	exonic	TP53	66	26	0.2826	Missense_Mutation	p.C96R	Targerted Panel Gene	Adult HGBCL, NOS
BCL-4	chr18	49257091	49257091	T	C	exonic	DYM	59	2	0.0328	Missense_Mutation	p.H460R	Non-Targeted Panel Gene	Adult HGBCL, NOS
BCL-4	chr19	47544914	47544914	G	A	exonic	ZNF541	58	2	0.0328	Missense_Mutation	p.R539C	Non-Targeted Panel Gene	Adult HGBCL, NOS
BCL-4	chr6	36972765	36972765	C	T	exonic	MTCH1	55	2	0.0351	Missense_Mutation	p.V265I	Non-Targeted Panel Gene	Adult HGBCL, NOS
BCL-4	chr14	64743552	64743552	C	A	exonic	PLEKHG3	53	2	0.0357	Missense_Mutation	p.P1170Q	Non-Targeted Panel Gene	Adult HGBCL, NOS
BCL-4	chr12	110342379	110342379	G	A	exonic	ATP2A2	53	2	0.0364	Missense_Mutation	p.R750Q	Non-Targeted Panel Gene	Adult HGBCL, NOS
BCL-4	chr1	62253184	62253184	G	C	exonic	KANK4	53	2	0.0364	Missense_Mutation	p.N855K	Non-Targeted Panel Gene	Adult HGBCL, NOS
BCL-4	chr11	62559776	62559776	C	T	exonic	EEF1G	79	3	0.0366	Missense_Mutation	p.S406N	Non-Targeted Panel Gene	Adult HGBCL, NOS
BCL-4	chr1	16255754	16255754	C	A	exonic	FBXO42	49	2	0.0392	Missense_Mutation	p.D242Y	Non-Targeted Panel Gene	Adult HGBCL, NOS
BCL-4	chr12	52777026	52777026	C	A	exonic	KRT76	49	2	0.0392	Missense_Mutation	p.G89V	Non-Targeted Panel Gene	Adult HGBCL, NOS
BCL-4	chr15	39589997	39589997	G	A	exonic	THBS1	48	2	0.0392	Missense_Mutation	p.A707T	Non-Targeted Panel Gene	Adult HGBCL, NOS
BCL-4	chr3	133834641	133834641	G	A	exonic	RAB6B	73	3	0.0395	Missense_Mutation	p.L166F	Non-Targeted Panel Gene	Adult HGBCL, NOS
BCL-4	chr17	48544737	48544737	C	A	exonic	HOXB2	48	2	0.04	Missense_Mutation	p.G59C	Non-Targeted Panel Gene	Adult HGBCL, NOS
BCL-4	chr19	13135592	13135592	G	A	exonic	NACC1	68	3	0.0423	Missense_Mutation	p.D129N	Non-Targeted Panel Gene	Adult HGBCL, NOS
BCL-4	chr16	67303176	67303176	C	T	exonic	KCTD19	43	2	0.0444	Missense_Mutation	p.E205K	Non-Targeted Panel Gene	Adult HGBCL, NOS
BCL-4	chr12	56433594	56433594	G	A	exonic	TIMELESS	64	3	0.0448	Missense_Mutation	p.P104S	Non-Targeted Panel Gene	Adult HGBCL, NOS
BCL-4	chr1	16133556	16133556	G	A	exonic	EPHA2	59	3	0.0476	Missense_Mutation	p.P597S	Non-Targeted Panel Gene	Adult HGBCL, NOS
BCL-4	chr15	68218613	68218613	C	T	exonic	CLN6	78	4	0.0488	Missense_Mutation	p.A41T	Non-Targeted Panel Gene	Adult HGBCL, NOS
BCL-4	chr9	131057115	131057115	G	A	exonic	LAMC3	38	2	0.0488	Missense_Mutation	p.C709Y	Non-Targeted Panel Gene	Adult HGBCL, NOS
BCL-4	chr1	205306288	205306288	G	A	exonic	NUAK2	39	2	0.0488	Missense_Mutation	p.S241F	Non-Targeted Panel Gene	Adult HGBCL, NOS
BCL-4	chr22	38822703	38822703	C	T	exonic	NPTXR	37	2	0.0513	Missense_Mutation	p.G470D	Non-Targeted Panel Gene	Adult HGBCL, NOS

BCL-4	chr2	219478003	219478003	C	T	exonic	SPEG	72	4	0.0519	Missense_Mutation	p.A1642V	Non-Targeted Panel Gene	Adult HGBCL, NOS
BCL-4	chr10	128049607	128049607	G	C	exonic	PTPRE	139	8	0.0541	Missense_Mutation	p.E132Q	Non-Targeted Panel Gene	Adult HGBCL, NOS
BCL-4	chr6	17828345	17828345	G	A	exonic	KIF13A	51	3	0.0556	Missense_Mutation	p.T476I	Non-Targeted Panel Gene	Adult HGBCL, NOS
BCL-4	chr12	50732397	50732397	C	T	exonic	DIP2B	50	3	0.0566	Missense_Mutation	p.T1281I	Non-Targeted Panel Gene	Adult HGBCL, NOS
BCL-4	chr12	95879702	95879702	C	T	exonic	CCDC38	33	2	0.0571	Missense_Mutation	p.D362N	Non-Targeted Panel Gene	Adult HGBCL, NOS
BCL-4	chr19	13963169	13963169	G	A	exonic	RFX1	47	3	0.0577	Missense_Mutation	p.R893C	Non-Targeted Panel Gene	Adult HGBCL, NOS
BCL-4	chr16	57997166	57997166	G	A	exonic	ZNF319	48	3	0.0577	Missense_Mutation	p.T367I	Non-Targeted Panel Gene	Adult HGBCL, NOS
BCL-4	chr14	21385858	21385858	G	A	exonic	CHD8	32	2	0.0588	Missense_Mutation	p.P2222S	Non-Targeted Panel Gene	Adult HGBCL, NOS
BCL-4	chr6	24403147	24403147	C	T	exonic	MRS2	30	2	0.0606	Missense_Mutation	p.P34L	Non-Targeted Panel Gene	Adult HGBCL, NOS
BCL-4	chr3	195867996	195867996	G	A	exonic	TNK2	30	2	0.0625	Missense_Mutation	p.H831Y	Non-Targeted Panel Gene	Adult HGBCL, NOS
BCL-4	chr5	146455271	146455271	C	A	exonic	TCERG1	29	2	0.0645	Missense_Mutation	p.P92Q	Non-Targeted Panel Gene	Adult HGBCL, NOS
BCL-4	chr9	35062303	35062303	G	A	exonic	VCP	57	4	0.0656	Missense_Mutation	p.R287C	Non-Targeted Panel Gene	Adult HGBCL, NOS
BCL-4	chr7	132140624	132140624	G	T	exonic	PLXNA4	28	2	0.0667	Missense_Mutation	p.P1805T	Non-Targeted Panel Gene	Adult HGBCL, NOS
BCL-4	chr19	4233182	4233182	C	T	exonic	EBI3	27	2	0.069	Missense_Mutation	p.T85M	Non-Targeted Panel Gene	Adult HGBCL, NOS
BCL-4	chr15	29766335	29766335	G	A	exonic	TJP1	53	4	0.069	Missense_Mutation	p.R178W	Non-Targeted Panel Gene	Adult HGBCL, NOS
BCL-4	chr2	40429224	40429224	C	T	exonic	SLC8A1	26	2	0.0714	Missense_Mutation	p.V353I	Non-Targeted Panel Gene	Adult HGBCL, NOS
BCL-4	chr10	47350595	47350595	G	A	exonic	RBP3	37	3	0.075	Missense_Mutation	p.S704N	Non-Targeted Panel Gene	Adult HGBCL, NOS
BCL-4	chr15	84860058	84860058	G	A	exonic	ALPK3	49	4	0.0755	Missense_Mutation	p.R1574K	Non-Targeted Panel Gene	Adult HGBCL, NOS
BCL-4	chr17	44558560	44558560	C	T	exonic	FZD2	61	5	0.0758	Missense_Mutation	p.S291L	Non-Targeted Panel Gene	Adult HGBCL, NOS
BCL-4	chr19	48196324	48196324	G	A	exonic	C19orf68	24	2	0.0769	Missense_Mutation	p.A754T	Non-Targeted Panel Gene	Adult HGBCL, NOS
BCL-4	chr11	64267437	64267437	C	T	exonic	PLCB3	24	2	0.0769	Missense_Mutation	p.P1196S	Non-Targeted Panel Gene	Adult HGBCL, NOS
BCL-4	chr12	41567851	41567851	A	C	exonic	PDZRN4	68	6	0.0811	Missense_Mutation	p.E512D	Non-Targeted Panel Gene	Adult HGBCL, NOS
BCL-4	chr6	53018353	53018353	G	C	exonic	ICK	44	4	0.0833	Missense_Mutation	p.Q214E	Non-Targeted Panel Gene	Adult HGBCL, NOS
BCL-4	chr15	43330350	43330350	C	T	exonic	LCMT2	22	2	0.0833	Missense_Mutation	p.R47H	Non-Targeted Panel Gene	Adult HGBCL, NOS
BCL-4	chr7	130492330	130492330	G	A	exonic	MEST	22	2	0.0833	Missense_Mutation	p.R6H	Non-Targeted Panel Gene	Adult HGBCL, NOS
BCL-4	chr9	96012860	96012860	G	T	exonic	ERCC6L2	21	2	0.087	Missense_Mutation	p.S1448I	Non-Targeted Panel Gene	Adult HGBCL, NOS
BCL-4	chr22	23834151	23834151	C	T	exonic	SMARCB1	42	4	0.087	Missense_Mutation	p.R377C	Non-Targeted Panel Gene	Adult HGBCL, NOS
BCL-4	chr5	13914634	13914634	A	T	exonic	DNAH5	41	4	0.0889	Missense_Mutation	p.N402K	Non-Targeted Panel Gene	Adult HGBCL, NOS
BCL-4	chr8	99820031	99820031	A	G	exonic	VPS13B	81	8	0.0899	Missense_Mutation	p.N2968S	Non-Targeted Panel Gene	Adult HGBCL, NOS
BCL-4	chr8	118926582	118926582	T	A	exonic	TNFRSF11B	89	9	0.0918	Missense_Mutation	p.Q243H	Non-Targeted Panel Gene	Adult HGBCL, NOS
BCL-4	chr17	68918467	68918467	C	T	exonic	ABCA8	29	3	0.0938	Missense_Mutation	p.R623K	Non-Targeted Panel Gene	Adult HGBCL, NOS
BCL-4	chr10	30336973	30336973	T	C	exonic	MTPAP	46	5	0.098	Missense_Mutation	p.T204A	Non-Targeted Panel Gene	Adult HGBCL, NOS
BCL-4	chr4	150079448	150079448	G	A	exonic	DCLK2	17	2	0.1	Missense_Mutation	p.G141S	Non-Targeted Panel Gene	Adult HGBCL, NOS
BCL-4	chr22	20242952	20242952	G	A	exonic	RTN4R	27	3	0.1	Missense_Mutation	p.R61C	Non-Targeted Panel Gene	Adult HGBCL, NOS
BCL-4	chr11	17555890	17555890	G	A	exonic	OTOG	71	8	0.1013	Missense_Mutation	p.G218S	Non-Targeted Panel Gene	Adult HGBCL, NOS
BCL-4	chr5	2749697	2749697	C	T	exonic	IRX2	25	3	0.1071	Missense_Mutation	p.D114N	Non-Targeted Panel Gene	Adult HGBCL, NOS
BCL-4	chrX	139560770	139560770	C	A	exonic	F9	40	5	0.1111	Missense_Mutation	p.F251L	Non-Targeted Panel Gene	Adult HGBCL, NOS
BCL-4	chr2	27336026	27336026	G	A	exonic	GTF3C2	63	8	0.1127	Missense_Mutation	p.P453L	Non-Targeted Panel Gene	Adult HGBCL, NOS
BCL-4	chr5	157751432	157751432	G	T	exonic	LSM11	127	17	0.1172	Missense_Mutation	p.R164L	Non-Targeted Panel Gene	Adult HGBCL, NOS
BCL-4	chrX	101553381	101553381	G	A	exonic	ARMCX1	30	4	0.1176	Missense_Mutation	p.G151S	Non-Targeted Panel Gene	Adult HGBCL, NOS
BCL-4	chr2	112723146	112723146	C	T	exonic	NT5DC4	104	14	0.1186	Missense_Mutation	p.T172I	Non-Targeted Panel Gene	Adult HGBCL, NOS
BCL-4	chr2	232522025	232522025	G	A	exonic	PRSS56	22	3	0.12	Missense_Mutation	p.R104H	Non-Targeted Panel Gene	Adult HGBCL, NOS
BCL-4	chr17	47742723	47742723	G	C	exonic	TBX21	22	3	0.12	Missense_Mutation	p.G202A	Non-Targeted Panel Gene	Adult HGBCL, NOS
BCL-4	chr1	158330077	158330077	T	G	exonic	CD1B	36	5	0.122	Missense_Mutation	p.I128L	Non-Targeted Panel Gene	Adult HGBCL, NOS
BCL-4	chr7	596165	596165	C	T	exonic	PRKAR1B	14	2	0.125	Missense_Mutation	p.S230N	Non-Targeted Panel Gene	Adult HGBCL, NOS
BCL-4	chrX	84468906	84468906	A	G	exonic	HDX	68	10	0.1282	Missense_Mutation	p.Y273H	Non-Targeted Panel Gene	Adult HGBCL, NOS
BCL-4	chr14	94619305	94619305	C	G	exonic	SERPINA3	54	8	0.129	Missense_Mutation	p.P252A	Non-Targeted Panel Gene	Adult HGBCL, NOS
BCL-4	chr16	4744876	4744876	T	C	exonic	C16orf71	33	5	0.1316	Missense_Mutation	p.M303T	Non-Targeted Panel Gene	Adult HGBCL, NOS
BCL-4	chr2	1943105	1943105	C	T	exonic	MYT1L	13	2	0.1333	Missense_Mutation	p.D128N	Non-Targeted Panel Gene	Adult HGBCL, NOS
BCL-4	chr11	66526181	66526181	T	G	exonic	BBS1	136	21	0.1338	Missense_Mutation	p.M390R	Non-Targeted Panel Gene	Adult HGBCL, NOS
BCL-4	chr10	35530201	35530201	C	G	exonic	CCNY	18	3	0.1429	Missense_Mutation	p.F125L	Non-Targeted Panel Gene	Adult HGBCL, NOS
BCL-4	chr1	216677435	216677435	A	G	exonic	ESRRG	90	15	0.1429	Missense_Mutation	p.F15S	Non-Targeted Panel Gene	Adult HGBCL, NOS
BCL-4	chr19	8812629	8812629	G	A	exonic	ZNF558	12	2	0.1429	Missense_Mutation	p.L120F	Non-Targeted Panel Gene	Adult HGBCL, NOS
BCL-4	chr13	27920235	27920235	C	A	exonic	PDX1	41	7	0.1458	Missense_Mutation	p.P33T	Non-Targeted Panel Gene	Adult HGBCL, NOS
BCL-4	chr2	240931010	240931010	C	A	exonic	CROCC2	17	3	0.15	Missense_Mutation	p.L277M	Non-Targeted Panel Gene	Adult HGBCL, NOS
BCL-4	chr17	9771665	9771665	G	T	exonic	DHRS7C	11	2	0.1538	Missense_Mutation	p.H254Q	Non-Targeted Panel Gene	Adult HGBCL, NOS
BCL-4	chr13	23839654	23839654	G	T	exonic	MIPEP	11	2	0.1538	Missense_Mutation	p.H445N	Non-Targeted Panel Gene	Adult HGBCL, NOS
BCL-4	chr3	47418692	47418692	C	T	exonic	SCAP	11	2	0.1538	Missense_Mutation	p.G698S	Non-Targeted Panel Gene	Adult HGBCL, NOS
BCL-4	chr1	185087591	185087591	T	C	exonic	RNF2	49	9	0.1552	Missense_Mutation	p.L13S	Non-Targeted Panel Gene	Adult HGBCL, NOS
BCL-4	chr17	82086333	82086333	A	C	exonic	FASN	16	3	0.1579	Missense_Mutation	p.L1218R	Non-Targeted Panel Gene	Adult HGBCL, NOS
BCL-4	chr5	141574081	141574081	C	A	exonic	DIAPH1	85	16	0.1584	Missense_Mutation	p.G590V	Non-Targeted Panel Gene	Adult HGBCL, NOS
BCL-4	chr19	55677699	55677699	G	A	exonic	EPN1	37	7	0.1591	Missense_Mutation	p.G66D	Non-Targeted Panel Gene	Adult HGBCL, NOS
BCL-4	chr2	221437063	221437063	T	C	exonic	EPHA4	47	9	0.1607	Missense_Mutation	p.R712G	Non-Targeted Panel Gene	Adult HGBCL, NOS
BCL-4	chr12	63808776	63808776	A	G	exonic	TMEM5	62	12	0.1622	Missense_Mutation	p.Y339C	Non-Targeted Panel Gene	Adult HGBCL, NOS
BCL-4	chr19	48796709	48796709	G	A	exonic	BCAT2	36	7	0.1628	Missense_Mutation	p.R272W	Non-Targeted Panel Gene	Adult HGBCL, NOS
BCL-4	chr2	239115124	239115124	C	T	exonic	HDAC4	10	2	0.1667	Missense_Mutation	p.E569K	Non-Targeted Panel Gene	Adult HGBCL, NOS
BCL-4	chr11	56542670	56542670	A	T	exonic	OR5M11	40	8	0.1667	Missense_Mutation	p.H196Q	Non-Targeted Panel Gene	Adult HGBCL, NOS
BCL-4	chr17	43093786	43093786	G	A	exonic	BRCA1	14	3	0.1765	Missense_Mutation	p.T582M	Non-Targeted Panel Gene	Adult HGBCL, NOS
BCL-4	chr12	114674410	114674410	C	T	exonic	TBX3	14	3	0.1765	Missense_Mutation	p.G509S	Non-Targeted Panel Gene	Adult HGBCL, NOS
BCL-4	chr3	195869508	195869508	A	G	exonic	TNK2	14	3	0.1765	Missense_Mutation	p.F589S	Non-Targeted Panel Gene	Adult HGBCL, NOS
BCL-4	chr14	105498660	105498660	G	A	exonic	C14orf80	9	2	0.1818	Missense_Mutation	p.R260Q	Non-Targeted Panel Gene	Adult HGBCL, NOS
BCL-4	chr11	62605643	62605643	T	G	exonic	EML3	9	2	0.1818	Missense_Mutation	p.K638T	Non-Targeted Panel Gene	Adult HGBCL, NOS
BCL-4	chr4	83421580	83421580	C	T	exonic	HELQ	9	2	0.1818	Missense_Mutation	p.V978M	Non-Targeted Panel Gene	Adult HGBCL, NOS
BCL-4	chr19	1827713	1827713	G	A	exonic	REXO1	9	2	0.1818	Missense_Mutation	p.P359L	Non-Targeted Panel Gene	Adult HGBCL, NOS
BCL-4	chrX	103084769	103084769	T	G	exonic	NXF3	67	15	0.1829	Missense_Mutation	p.Q48P	Non-Targeted Panel Gene	Adult HGBCL, NOS
BCL-4	chr17	5183365	5183365	T	A	exonic	ZNF594	48	11	0.1864	Missense_Mutation	p.N298Y	Non-Targeted Panel Gene	Adult HGBCL, NOS
BCL-4	chr21	44439110	44439110	G	C	exonic	TRPM2	39	9	0.1875	Missense_Mutation	p.R1454P	Non-Targeted Panel Gene	Adult HGBCL, NOS
BCL-4	chr8	134602008	134602008	C	T	exonic	ZFAT	39	9	0.1875	Missense_Mutation	p.A559T	Non-Targeted Panel Gene	Adult HGBCL, NOS
BCL-4	chr19	4548398	4548398	C	G	exonic	SEMA6B	47	11	0.1897	Missense_Mutation	p.G440A	Non-Targeted Panel Gene	Adult HGBCL, NOS
BCL-4	chr20	45967623	45967623	C	T	exonic	ZNF335	38	9	0.1915	Missense_Mutation	p.A276T	Non-Targeted Panel Gene	Adult HGBCL, NOS
BCL-4	chr12	108517025	108517025	T	G	exonic	FICD	82	20	0.1961	Missense_Mutation	p.V18G	Non-Targeted Panel Gene	Adult HGBCL, NOS
BCL-4	chr11	100071715	100071715	A	G	exonic	CNTN5	20	5	0.2	Missense_Mutation	p.E437G	Non-Targeted Panel Gene	Adult HGBCL, NOS

BCL-4	chr1	2498609	2498609	C	A	exonic	PLCH2	8	2	0.2	Missense_Mutation	p.P771T	Non-Targeted Panel Gene	Adult HGBCL, NOS
BCL-4	chr1	153112703	153112703	G	A	exonic	SPRR2F	20	5	0.2	Missense_Mutation	p.P11S	Non-Targeted Panel Gene	Adult HGBCL, NOS
BCL-4	chr19	41988043	41988043	G	A	exonic	ATP1A3	35	9	0.2045	Missense_Mutation	p.R84W	Non-Targeted Panel Gene	Adult HGBCL, NOS
BCL-4	chr11	124265791	124265791	C	T	exonic	OR8G5	81	21	0.2059	Missense_Mutation	p.P322L	Non-Targeted Panel Gene	Adult HGBCL, NOS
BCL-4	chr10	89402632	89402632	C	A	exonic	IFIT1	19	5	0.2083	Missense_Mutation	p.D88E	Non-Targeted Panel Gene	Adult HGBCL, NOS
BCL-4	chr3	51393399	51393399	A	G	exonic	RBM15B	83	22	0.2095	Missense_Mutation	p.H667R	Non-Targeted Panel Gene	Adult HGBCL, NOS
BCL-4	chr2	154855065	154855065	A	G	exonic	KCNJ3	15	4	0.2105	Missense_Mutation	p.S420G	Non-Targeted Panel Gene	Adult HGBCL, NOS
BCL-4	chr19	17282118	17282118	G	A	exonic	ANKLE1	7	2	0.2222	Missense_Mutation	p.A96T	Non-Targeted Panel Gene	Adult HGBCL, NOS
BCL-4	chr18	45955181	45955181	C	T	exonic	EPG5	63	19	0.2317	Missense_Mutation	p.G74E	Non-Targeted Panel Gene	Adult HGBCL, NOS
BCL-4	chr2	219218423	219218423	G	A	exonic	ABCB6	23	7	0.2333	Missense_Mutation	p.A84V	Non-Targeted Panel Gene	Adult HGBCL, NOS
BCL-4	chr2	219237957	219237957	C	T	exonic	GLB1L	22	7	0.2414	Missense_Mutation	p.V448M	Non-Targeted Panel Gene	Adult HGBCL, NOS
BCL-4	chr3	50358686	50358686	G	C	exonic	TMEM115	65	21	0.2442	Missense_Mutation	p.N126K	Non-Targeted Panel Gene	Adult HGBCL, NOS
BCL-4	chr2	218693780	218693780	C	T	exonic	STK36	40	13	0.2453	Missense_Mutation	p.P736S	Non-Targeted Panel Gene	Adult HGBCL, NOS
BCL-4	chr1	186417650	186417650	C	A	exonic	C1orf27	6	2	0.25	Missense_Mutation	p.N431K	Non-Targeted Panel Gene	Adult HGBCL, NOS
BCL-4	chr3	62874992	62874992	T	A	exonic	CADPS	9	3	0.25	Missense_Mutation	p.E13V	Non-Targeted Panel Gene	Adult HGBCL, NOS
BCL-4	chr2	219496473	219496473	G	A	exonic	LOC100996693	21	7	0.25	Missense_Mutation	p.R40Q	Non-Targeted Panel Gene	Adult HGBCL, NOS
BCL-4	chr11	1197591	1197591	G	A	exonic	MUC5AC	6	2	0.25	Missense_Mutation	p.V5329M	Non-Targeted Panel Gene	Adult HGBCL, NOS
BCL-4	chr2	170386193	170386193	T	C	exonic	MYO3B	59	20	0.2532	Missense_Mutation	p.I432T	Non-Targeted Panel Gene	Adult HGBCL, NOS
BCL-4	chr12	95533783	95533783	C	G	exonic	USP44	38	13	0.2549	Missense_Mutation	p.M158I	Non-Targeted Panel Gene	Adult HGBCL, NOS
BCL-4	chrX	124094124	124094124	G	C	exonic	STAG2	32	11	0.2558	Missense_Mutation	p.D1229H	Non-Targeted Panel Gene	Adult HGBCL, NOS
BCL-4	chr1	213997669	213997669	G	T	exonic	PROX1	72	25	0.2577	Missense_Mutation	p.Q378H	Non-Targeted Panel Gene	Adult HGBCL, NOS
BCL-4	chr22	37182547	37182547	A	C	exonic	C1QTNF6	56	20	0.2597	Missense_Mutation	p.F160V	Non-Targeted Panel Gene	Adult HGBCL, NOS
BCL-4	chr16	13932233	13932233	A	C	exonic	ERCC4	17	6	0.2609	Missense_Mutation	p.K350N	Non-Targeted Panel Gene	Adult HGBCL, NOS
BCL-4	chr6	15524474	15524474	G	C	exonic	DTNBP1	57	21	0.2692	Missense_Mutation	p.S288C	Non-Targeted Panel Gene	Adult HGBCL, NOS
BCL-4	chr16	89920138	89920138	G	C	exonic	MC1R	99	37	0.2721	Missense_Mutation	p.D294H	Non-Targeted Panel Gene	Adult HGBCL, NOS
BCL-4	chr9	136475136	136475136	G	A	exonic	SEC16A	60	23	0.2771	Missense_Mutation	p.P827L	Non-Targeted Panel Gene	Adult HGBCL, NOS
BCL-4	chr3	196316185	196316185	C	G	exonic	TCTEX1D2	77	30	0.2804	Missense_Mutation	p.E54Q	Non-Targeted Panel Gene	Adult HGBCL, NOS
BCL-4	chr2	55875027	55875027	A	G	exonic	EFEMP1	61	24	0.2824	Missense_Mutation	p.Y307H	Non-Targeted Panel Gene	Adult HGBCL, NOS
BCL-4	chr19	11577244	11577244	G	A	exonic	ACP5	72	29	0.2871	Missense_Mutation	p.A25V	Non-Targeted Panel Gene	Adult HGBCL, NOS
BCL-4	chr1	200898410	200898410	A	G	exonic	C1orf106	35	15	0.3	Missense_Mutation	p.K103E	Non-Targeted Panel Gene	Adult HGBCL, NOS
BCL-4	chr1	214327634	214327634	G	A	exonic	SMYD2	48	21	0.3043	Missense_Mutation	p.M205I	Non-Targeted Panel Gene	Adult HGBCL, NOS
BCL-4	chr2	218738889	218738889	G	T	exonic	TTLL4	71	32	0.3107	Missense_Mutation	p.D405Y	Non-Targeted Panel Gene	Adult HGBCL, NOS
BCL-4	chr5	116058861	116058861	C	T	exonic	ARL14EPL	66	30	0.3125	Missense_Mutation	p.R125C	Non-Targeted Panel Gene	Adult HGBCL, NOS
BCL-4	chrX	56565073	56565073	C	G	exonic	UBQLN2	54	27	0.3333	Missense_Mutation	p.S400R	Non-Targeted Panel Gene	Adult HGBCL, NOS
BCL-4	chr14	100881768	100881768	C	A	exonic	RTL1	43	22	0.3385	Missense_Mutation	p.R1007S	Non-Targeted Panel Gene	Adult HGBCL, NOS
BCL-4	chr5	90720961	90720961	C	T	exonic	ADGRV1	68	35	0.3398	Missense_Mutation	p.A3217V	Non-Targeted Panel Gene	Adult HGBCL, NOS
BCL-4	chr15	80580504	80580504	G	T	exonic	ARNT2	53	28	0.3457	Missense_Mutation	p.Q569H	Non-Targeted Panel Gene	Adult HGBCL, NOS
BCL-4	chr17	10697492	10697492	G	C	exonic	SCO1	69	38	0.3486	Missense_Mutation	p.L6V	Non-Targeted Panel Gene	Adult HGBCL, NOS
BCL-4	chr1	117092816	117092816	G	C	exonic	TTF2	41	22	0.3492	Missense_Mutation	p.R964P	Non-Targeted Panel Gene	Adult HGBCL, NOS
BCL-4	chr7	134139442	134139442	A	G	exonic	LRGUK	11	6	0.3529	Missense_Mutation	p.N138D	Non-Targeted Panel Gene	Adult HGBCL, NOS
BCL-4	chr1	109494186	109494186	C	G	exonic	CYB561D1	7	4	0.3636	Missense_Mutation	p.P16R	Non-Targeted Panel Gene	Adult HGBCL, NOS
BCL-4	chr3	37111014	37111014	G	C	exonic	LRRFIP2	22	14	0.3889	Missense_Mutation	p.P164A	Non-Targeted Panel Gene	Adult HGBCL, NOS
BCL-4	chr9	98128696	98128696	C	T	exonic	CORO2A	62	41	0.3981	Missense_Mutation	p.D331N	Non-Targeted Panel Gene	Adult HGBCL, NOS
BCL-4	chr6	32097337	32097337	A	T	exonic	TNXB	47	37	0.4405	Missense_Mutation	p.D172E	Non-Targeted Panel Gene	Adult HGBCL, NOS
BCL-4	chr19	2041931	2041931	A	G	exonic	MKNK2	16	14	0.4667	Missense_Mutation	p.L285S	Non-Targeted Panel Gene	Adult HGBCL, NOS
BCL-4	chr8	43122119	43122119	G	T	exonic	POMK	57	2	0.0333	Nonsense_Mutation	p.E99X	Non-Targeted Panel Gene	Adult HGBCL, NOS
BCL-4	chr19	48831748	48831748	G	A	exonic	HSD17B14	30	4	0.1143	Nonsense_Mutation	p.Q97X	Non-Targeted Panel Gene	Adult HGBCL, NOS
BCL-4	chr2	219385528	219385528	C	A	exonic	DNPEP	52	30	0.3659	Nonsense_Mutation	p.E171X	Non-Targeted Panel Gene	Adult HGBCL, NOS
BCL-40	chr19	7058559	7058559	A	G	splicing	MBD3L3	84	8	0.087	Splice_Site	.	Non-Targeted Panel Gene	Adult HGBCL, NOS
BCL-40	chr17	58270865	58270865	T	G	splicing	MPO	87	61	0.4122	Splice_Site	.	Non-Targeted Panel Gene	Adult HGBCL, NOS
BCL-40	chr4	3767645	3767678	CGCGCCAGCTCGC(	-	exonic	ADRA2C	45	12	0.2105	Frame_Shift_Del	p.R347fs	Non-Targeted Panel Gene	Adult HGBCL, NOS
BCL-40	chr4	151671217	151671217	G	-	exonic	GATB	36	17	0.3208	Frame_Shift_Del	p.P544fs	Non-Targeted Panel Gene	Adult HGBCL, NOS
BCL-40	chr11	77961136	77961136	A	-	exonic	INTS4	11	16	0.5926	Frame_Shift_Del	p.H158fs	Non-Targeted Panel Gene	Adult HGBCL, NOS
BCL-40	chr20	57615004	57615004	-	GATGA	exonic	ZBP1	72	61	0.4586	Frame_Shift_Ins	p.A129fs	Non-Targeted Panel Gene	Adult HGBCL, NOS
BCL-40	chr5	1293561	1293563	TCC	-	exonic	TERT	118	73	0.3822	In_Frame_Del	p.441_442del	Non-Targeted Panel Gene	Adult HGBCL, NOS
BCL-40	chr22	46738125	46738125	C	G	exonic	CERK	10	4	0.2857	Missense_Mutation	p.E8D	Non-Targeted Panel Gene	Adult HGBCL, NOS
BCL-40	chr1	167113729	167113729	G	A	exonic	DUSP27	29	13	0.3095	Missense_Mutation	p.A44T	Non-Targeted Panel Gene	Adult HGBCL, NOS
BCL-40	chr7	45084104	45084104	A	T	exonic	NACAD	42	20	0.3226	Missense_Mutation	p.D692E	Non-Targeted Panel Gene	Adult HGBCL, NOS
BCL-40	chr3	17406482	17406482	G	A	exonic	TBC1D5	17	9	0.3462	Missense_Mutation	p.T71I	Non-Targeted Panel Gene	Adult HGBCL, NOS
BCL-40	chr19	50481876	50481876	G	T	exonic	EMC10	31	18	0.3673	Missense_Mutation	p.G232W	Non-Targeted Panel Gene	Adult HGBCL, NOS
BCL-40	chr7	150947833	150947833	G	A	exonic	KCNH2	27	16	0.3721	Missense_Mutation	p.A913V	Non-Targeted Panel Gene	Adult HGBCL, NOS
BCL-40	chr4	83470243	83470243	T	C	exonic	ABRAXAS1	12	8	0.4	Missense_Mutation	p.T146A	Non-Targeted Panel Gene	Adult HGBCL, NOS
BCL-40	chr4	118282175	118282175	G	A	exonic	PRSS12	75	50	0.4	Missense_Mutation	p.R797C	Non-Targeted Panel Gene	Adult HGBCL, NOS
BCL-40	chr4	71022550	71022550	T	C	exonic	DCK	32	22	0.4074	Missense_Mutation	p.Y131H	Non-Targeted Panel Gene	Adult HGBCL, NOS
BCL-40	chr12	109188111	109188111	C	T	exonic	ACACB	103	78	0.4309	Missense_Mutation	p.S698F	Non-Targeted Panel Gene	Adult HGBCL, NOS
BCL-40	chr2	174470428	174470428	C	A	exonic	GPR155	26	20	0.4348	Missense_Mutation	p.A330S	Non-Targeted Panel Gene	Adult HGBCL, NOS
BCL-40	chr11	10560545	10560545	G	C	exonic	LYVE1	47	37	0.4405	Missense_Mutation	p.P218R	Non-Targeted Panel Gene	Adult HGBCL, NOS
BCL-40	chr16	87410288	87410288	T	C	exonic	ZCCHC14	37	31	0.4559	Missense_Mutation	p.T948A	Non-Targeted Panel Gene	Adult HGBCL, NOS
BCL-40	chr2	237324784	237324784	A	G	exonic	COL6A3	71	61	0.4586	Missense_Mutation	p.M3175T	Non-Targeted Panel Gene	Adult HGBCL, NOS
BCL-40	chr16	2497033	2497033	C	G	exonic	TBC1D24	241	209	0.4644	Missense_Mutation	p.F295L	Non-Targeted Panel Gene	Adult HGBCL, NOS
BCL-40	chr5	110625416	110625416	C	T	exonic	TMEM232	8	7	0.4667	Missense_Mutation	p.A207T	Non-Targeted Panel Gene	Adult HGBCL, NOS
BCL-40	chr19	1223126	1223126	C	G	exonic	STK11	169	150	0.4702	Missense_Mutation	p.F354L	Non-Targeted Panel Gene	Adult HGBCL, NOS
BCL-40	chr5	35876149	35876149	A	C	exonic	IL7R	56	50	0.4717	Missense_Mutation	p.N348T	Non-Targeted Panel Gene	Adult HGBCL, NOS
BCL-40	chr19	1007727	1007727	C	G	exonic	GRIN3B	75	67	0.4718	Missense_Mutation	p.R718G	Non-Targeted Panel Gene	Adult HGBCL, NOS
BCL-40	chr19	15265386	15265386	G	C	exonic	BRD4	26	24	0.48	Missense_Mutation	p.P273A	Non-Targeted Panel Gene	Adult HGBCL, NOS
BCL-40	chr1	20633708	20633708	G	T	exonic	PINK1	16	15	0.4839	Missense_Mutation	p.A54S	Non-Targeted Panel Gene	Adult HGBCL, NOS
BCL-40	chr16	89863564	89863564	A	C	exonic	SPIRE2	130	124	0.4882	Missense_Mutation	p.K555T	Non-Targeted Panel Gene	Adult HGBCL, NOS
BCL-40	chr17	63941779	63941779	C	T	exonic	SCN4A	233	224	0.4891	Missense_Mutation	p.M1501I	Non-Targeted Panel Gene	Adult HGBCL, NOS
BCL-40	chr6	42104827	42104827	C	T	exonic	C6orf132	66	66	0.4962	Missense_Mutation	p.G1029R	Non-Targeted Panel Gene	Adult HGBCL, NOS
BCL-40	chr3	129279907	129279907	T	G	exonic	HMCES	30	30	0.5	Missense_Mutation	p.F59V	Non-Targeted Panel Gene	Adult HGBCL, NOS
BCL-40	chr6	13641215	13641215	G	A	exonic	RANBP9	8	8	0.5	Missense_Mutation	p.L440F	Non-Targeted Panel Gene	Adult HGBCL, NOS
BCL-40	chr3	119187938	119187938	T	C	exonic	UPK1B	66	66	0.5	Missense_Mutation	p.V78A	Non-Targeted Panel Gene	Adult HGBCL, NOS

BCL-40	chr4	113360849	113360849	G	A	exonic	ANK2	35	36	0.507	Missense_Mutation	p.E1476K	Non-Targeted Panel Gene	Adult HGBCL, NOS
BCL-40	chr7	149808624	149808624	G	T	exonic	SSPO	81	84	0.5091	Missense_Mutation	p.A3032S	Non-Targeted Panel Gene	Adult HGBCL, NOS
BCL-40	chr1	232986410	232986410	G	T	exonic	PCNX2	90	94	0.5109	Missense_Mutation	p.H1974Q	Non-Targeted Panel Gene	Adult HGBCL, NOS
BCL-40	chr2	26461006	26461006	C	T	exonic	OTOF	90	95	0.5135	Missense_Mutation	p.R1853Q	Non-Targeted Panel Gene	Adult HGBCL, NOS
BCL-40	chr3	187670216	187670216	G	A	exonic	SST	60	67	0.5276	Missense_Mutation	p.P26S	Non-Targeted Panel Gene	Adult HGBCL, NOS
BCL-40	chr9	120913533	120913533	G	A	exonic	TRAF1	76	85	0.528	Missense_Mutation	p.A167V	Non-Targeted Panel Gene	Adult HGBCL, NOS
BCL-40	chr4	24895048	24895048	C	T	exonic	CCDC149	50	56	0.5283	Missense_Mutation	p.V3I	Non-Targeted Panel Gene	Adult HGBCL, NOS
BCL-40	chr22	41873844	41873844	T	C	exonic	SREBF2	17	20	0.5405	Missense_Mutation	p.M305T	Non-Targeted Panel Gene	Adult HGBCL, NOS
BCL-40	chr7	138844385	138844385	G	A	exonic	KIAA1549	55	67	0.5492	Missense_Mutation	p.A1795V	Non-Targeted Panel Gene	Adult HGBCL, NOS
BCL-40	chr11	554154	554154	G	A	exonic	LRRC56	41	50	0.5495	Missense_Mutation	p.E503K	Non-Targeted Panel Gene	Adult HGBCL, NOS
BCL-40	chr11	1866835	1866835	G	A	exonic	LSP1	64	82	0.5616	Missense_Mutation	p.G121S	Non-Targeted Panel Gene	Adult HGBCL, NOS
BCL-40	chr19	18281025	18281025	C	T	exonic	JUND	27	39	0.5909	Missense_Mutation	p.G154S	Non-Targeted Panel Gene	Adult HGBCL, NOS
BCL-40	chr1	241597960	241597960	A	G	exonic	OPN3	16	25	0.6098	Missense_Mutation	p.I244T	Non-Targeted Panel Gene	Adult HGBCL, NOS
BCL-41	chr14	58271000	58271000	T	C	splicing	PSMA3	26	4	0.1333	Splice_Site	.	Non-Targeted Panel Gene	Adult HGBCL, NOS
BCL-41	chr1	145848392	145848396	TAAAA	-	exonic	NUDT17	111	78	0.4127	Frame_Shift_Del	p.C300fs	Non-Targeted Panel Gene	Adult HGBCL, NOS
BCL-41	chr3	51338367	51338367	T	C	exonic	DOCK3	123	6	0.0465	Missense_Mutation	p.M1207T	Non-Targeted Panel Gene	Adult HGBCL, NOS
BCL-41	chr4	1403323	1403323	C	T	exonic	NKX1-1	146	9	0.0581	Missense_Mutation	p.R319H	Non-Targeted Panel Gene	Adult HGBCL, NOS
BCL-41	chr3	130567074	130567074	G	T	exonic	COL6A6	128	9	0.0657	Missense_Mutation	p.S552I	Non-Targeted Panel Gene	Adult HGBCL, NOS
BCL-41	chr9	138118005	138118005	A	T	exonic	CACNA1B	168	13	0.0718	Missense_Mutation	p.D1946V	Non-Targeted Panel Gene	Adult HGBCL, NOS
BCL-41	chr6	22569849	22569849	G	A	exonic	HDGFL1	254	23	0.083	Missense_Mutation	p.D92N	Non-Targeted Panel Gene	Adult HGBCL, NOS
BCL-41	chr7	110663648	110663648	G	T	exonic	IMMP2L	74	7	0.0864	Missense_Mutation	p.S189Y	Non-Targeted Panel Gene	Adult HGBCL, NOS
BCL-41	chr6	32815203	32815203	T	G	exonic	HLA-DOB	86	9	0.0947	Missense_Mutation	p.S68R	Non-Targeted Panel Gene	Adult HGBCL, NOS
BCL-41	chr7	39207413	39207413	T	G	exonic	POU6F2	139	15	0.0974	Missense_Mutation	p.L102V	Non-Targeted Panel Gene	Adult HGBCL, NOS
BCL-41	chr4	5640666	5640666	C	T	exonic	EVC2	83	9	0.0978	Missense_Mutation	p.E440K	Non-Targeted Panel Gene	Adult HGBCL, NOS
BCL-41	chr8	37938750	37938750	A	G	exonic	GOT1L1	193	26	0.1187	Missense_Mutation	p.S83P	Non-Targeted Panel Gene	Adult HGBCL, NOS
BCL-41	chrX	67643323	67643323	A	C	exonic	AR	65	9	0.1216	Missense_Mutation	p.I30L	Non-Targeted Panel Gene	Adult HGBCL, NOS
BCL-41	chrX	1466074	1466074	T	G	exonic	P2RY8	235	34	0.1264	Missense_Mutation	p.D162A	Non-Targeted Panel Gene	Adult HGBCL, NOS
BCL-41	chrX	1466083	1466083	G	A	exonic	P2RY8	238	38	0.1377	Missense_Mutation	p.A159V	Non-Targeted Panel Gene	Adult HGBCL, NOS
BCL-41	chrX	1466129	1466129	A	G	exonic	P2RY8	265	43	0.1396	Missense_Mutation	p.C144R	Non-Targeted Panel Gene	Adult HGBCL, NOS
BCL-41	chrX	19966471	19966471	A	T	exonic	CXorf23	32	8	0.2	Missense_Mutation	p.Y74N	Non-Targeted Panel Gene	Adult HGBCL, NOS
BCL-41	chr4	3074911	3074911	A	C	exonic	HTT	24	6	0.2	Missense_Mutation	p.Q29P	Non-Targeted Panel Gene	Adult HGBCL, NOS
BCL-41	chr11	18933967	18933967	A	T	exonic	MRGPRX1	97	55	0.3618	Missense_Mutation	p.F273Y	Non-Targeted Panel Gene	Adult HGBCL, NOS
BCL-41	chr22	49925494	49925494	G	T	exonic	CRELD2	95	66	0.4099	Missense_Mutation	p.V365F	Non-Targeted Panel Gene	Adult HGBCL, NOS
BCL-41	chr5	43677728	43677728	A	T	exonic	NNT	54	38	0.413	Missense_Mutation	p.Y933F	Non-Targeted Panel Gene	Adult HGBCL, NOS
BCL-41	chr16	24563225	24563225	C	T	exonic	RBBP6	52	39	0.4286	Missense_Mutation	p.A439V	Non-Targeted Panel Gene	Adult HGBCL, NOS
BCL-41	chr16	1210122	1210122	T	C	exonic	CACNA1H	30	23	0.434	Missense_Mutation	p.S1278P	Non-Targeted Panel Gene	Adult HGBCL, NOS
BCL-41	chr16	89546737	89546737	C	T	exonic	SPG7	104	81	0.4378	Missense_Mutation	p.A510V	Non-Targeted Panel Gene	Adult HGBCL, NOS
BCL-41	chr12	57608581	57608581	A	G	exonic	DTX3	198	157	0.4423	Missense_Mutation	p.D271G	Non-Targeted Panel Gene	Adult HGBCL, NOS
BCL-41	chr1	13583923	13583923	C	A	exonic	PDPN	105	91	0.4619	Missense_Mutation	p.P40T	Non-Targeted Panel Gene	Adult HGBCL, NOS
BCL-41	chr7	151071432	151071432	A	G	exonic	SLC4A2	55	48	0.466	Missense_Mutation	p.D673G	Non-Targeted Panel Gene	Adult HGBCL, NOS
BCL-41	chr7	6607989	6607989	T	C	exonic	C7orf26	9	8	0.4706	Missense_Mutation	p.V296A	Non-Targeted Panel Gene	Adult HGBCL, NOS
BCL-41	chr6	148747573	148747573	A	G	exonic	UST	45	40	0.4706	Missense_Mutation	p.Y48C	Non-Targeted Panel Gene	Adult HGBCL, NOS
BCL-41	chr2	112077213	112077213	A	G	exonic	TMEM87B	19	17	0.4722	Missense_Mutation	p.K175E	Non-Targeted Panel Gene	Adult HGBCL, NOS
BCL-41	chr10	125719893	125719893	A	C	exonic	EDRF1	59	53	0.4732	Missense_Mutation	p.E29A	Non-Targeted Panel Gene	Adult HGBCL, NOS
BCL-41	chr2	178617228	178617228	C	T	exonic	TTN	38	35	0.4795	Missense_Mutation	p.G15923R	Non-Targeted Panel Gene	Adult HGBCL, NOS
BCL-41	chr14	67789467	67789467	C	G	exonic	ZFYVE26	116	108	0.4821	Missense_Mutation	p.V963L	Non-Targeted Panel Gene	Adult HGBCL, NOS
BCL-41	chr10	43112906	43112906	G	A	exonic	RET	132	123	0.4824	Missense_Mutation	p.G568S	Non-Targeted Panel Gene	Adult HGBCL, NOS
BCL-41	chr11	376733	376733	C	A	exonic	B4GALNT4	30	28	0.4828	Missense_Mutation	p.S537Y	Non-Targeted Panel Gene	Adult HGBCL, NOS
BCL-41	chr17	11652805	11652805	C	T	exonic	DNAH9	15	14	0.4828	Missense_Mutation	p.L800F	Non-Targeted Panel Gene	Adult HGBCL, NOS
BCL-41	chr19	1453094	1453094	C	A	exonic	APC2	65	62	0.4882	Missense_Mutation	p.D31E	Non-Targeted Panel Gene	Adult HGBCL, NOS
BCL-41	chr5	149362811	149362811	G	A	exonic	PCYOX1L	134	129	0.4905	Missense_Mutation	p.S88N	Non-Targeted Panel Gene	Adult HGBCL, NOS
BCL-41	chr1	45329412	45329412	G	A	exonic	MUTYH	94	91	0.4919	Missense_Mutation	p.S487F	Non-Targeted Panel Gene	Adult HGBCL, NOS
BCL-41	chr10	104050890	104050890	G	A	exonic	COL17A1	70	68	0.4928	Missense_Mutation	p.L684F	Non-Targeted Panel Gene	Adult HGBCL, NOS
BCL-41	chr7	256734	256734	T	C	exonic	FAM20C	131	128	0.4942	Missense_Mutation	p.M445T	Non-Targeted Panel Gene	Adult HGBCL, NOS
BCL-41	chr1	12004835	12004835	G	A	exonic	MFN2	88	88	0.5	Missense_Mutation	p.R468H	Non-Targeted Panel Gene	Adult HGBCL, NOS
BCL-41	chr4	979426	979426	C	G	exonic	SLC26A1	76	80	0.5128	Missense_Mutation	p.E219Q	Non-Targeted Panel Gene	Adult HGBCL, NOS
BCL-41	chr16	89508599	89508599	A	G	exonic	SPG7	18	19	0.5135	Missense_Mutation	p.Q61R	Non-Targeted Panel Gene	Adult HGBCL, NOS
BCL-41	chr7	6017948	6017948	G	C	exonic	AIMP2	44	47	0.5165	Missense_Mutation	p.K81N	Non-Targeted Panel Gene	Adult HGBCL, NOS
BCL-41	chr2	120990472	120990472	G	A	exonic	GLI2	62	70	0.5303	Missense_Mutation	p.D1520N	Non-Targeted Panel Gene	Adult HGBCL, NOS
BCL-41	chr19	34221634	34221634	G	A	exonic	LSM14A	14	16	0.5333	Missense_Mutation	p.G422R	Non-Targeted Panel Gene	Adult HGBCL, NOS
BCL-41	chr11	66337784	66337784	T	C	exonic	BRMS1	79	95	0.546	Missense_Mutation	p.R253G	Non-Targeted Panel Gene	Adult HGBCL, NOS
BCL-41	chr10	104039506	104039506	G	C	exonic	COL17A1	54	81	0.6	Missense_Mutation	p.F945L	Non-Targeted Panel Gene	Adult HGBCL, NOS
BCL-41	chr17	61847211	61847211	G	A	exonic	BRIP1	18	28	0.6087	Missense_Mutation	p.R173C	Non-Targeted Panel Gene	Adult HGBCL, NOS
BCL-41	chr17	5486121	5486121	G	C	exonic	DERL2	8	13	0.619	Missense_Mutation	p.P14R	Non-Targeted Panel Gene	Adult HGBCL, NOS
BCL-41	chr19	52906672	52906672	G	C	exonic	ZNF888	11	5	0.3125	Nonsense_Mutation	p.Y550X	Non-Targeted Panel Gene	Adult HGBCL, NOS
BCL-41	chr8	143991784	143991784	C	T	exonic	GRINA	125	113	0.4748	Nonsense_Mutation	p.R158X	Non-Targeted Panel Gene	Adult HGBCL, NOS
BCL-42	chr2	47832962	47832962	-	AG	splicing	FBXO11	39	14	0.2642	Splice_Site	.	Non-Targeted Panel Gene	Adult HGBCL, NOS
BCL-42	chr1	85096021	85096021	G	A	splicing	WDR63	39	29	0.4265	Splice_Site	.	Non-Targeted Panel Gene	Adult HGBCL, NOS
BCL-42	chr1	50973894	50973894	T	-	exonic	CDKN2C	84	43	0.3386	Frame_Shift_Del	p.V44fs	Non-Targeted Panel Gene	Adult HGBCL, NOS
BCL-42	chr7	20651514	20651514	C	-	exonic	ABCB5	44	34	0.4359	Frame_Shift_Del	p.T476fs	Non-Targeted Panel Gene	Adult HGBCL, NOS
BCL-42	chr1	173961786	173961787	TG	-	exonic	RC3H1	91	134	0.5956	Frame_Shift_Del	p.Q714fs	Non-Targeted Panel Gene	Adult HGBCL, NOS
BCL-42	chr2	152672596	152672596	-	T	exonic	PRPF40A	10	7	0.4118	Frame_Shift_Ins	p.K411fs	Non-Targeted Panel Gene	Adult HGBCL, NOS
BCL-42	chr4	183265087	183265087	-	T	exonic	WWC2	64	78	0.5493	Frame_Shift_Ins	p.V673fs	Non-Targeted Panel Gene	Adult HGBCL, NOS
BCL-42	chr7	6653485	6653508	GCCCGCTCCCGGC	-	exonic	ZNF316	23	4	0.1481	In_Frame_Del	p.630_638del	Non-Targeted Panel Gene	Adult HGBCL, NOS
BCL-42	chr13	111159048	111159050	CCT	-	exonic	ARHGEF7	40	25	0.3846	In_Frame_Del	p.92_92del	Non-Targeted Panel Gene	Adult HGBCL, NOS
BCL-42	chr3	127016593	127016593	A	C	exonic	PLXNA1	133	8	0.0567	Missense_Mutation	p.N1031H	Non-Targeted Panel Gene	Adult HGBCL, NOS
BCL-42	chr15	67164957	67164957	G	A	exonic	SMAD3	179	11	0.0579	Missense_Mutation	p.R90H	Non-Targeted Panel Gene	Adult HGBCL, NOS
BCL-42	chr10	37832979	37832979	C	A	exonic	ZNF248	44	3	0.0638	Missense_Mutation	p.G126C	Non-Targeted Panel Gene	Adult HGBCL, NOS
BCL-42	chr19	50668217	50668217	T	G	exonic	SHANK1	56	4	0.0667	Missense_Mutation	p.N1248T	Non-Targeted Panel Gene	Adult HGBCL, NOS
BCL-42	chr12	12787048	12787048	T	G	exonic	APOLD1	43	4	0.0833	Missense_Mutation	p.L48R	Non-Targeted Panel Gene	Adult HGBCL, NOS
BCL-42	chr12	116881085	116881085	C	T	exonic	HRK	11	2	0.1538	Missense_Mutation	p.A75T	Non-Targeted Panel Gene	Adult HGBCL, NOS
BCL-42	chr21	46418239	46418239	T	G	exonic	PCNT	20	8	0.2857	Missense_Mutation	p.F2319L	Non-Targeted Panel Gene	Adult HGBCL, NOS

BCL-42	chr16	55493217	55493217	C	G	exonic	MMP2	52	25	0.3247	Missense_Mutation	p.P466A	Non-Targeted Panel Gene	Adult HGBCL, NOS
BCL-42	chr2	195972391	195972391	C	A	exonic	DNAH7	97	49	0.3356	Missense_Mutation	p.A637S	Non-Targeted Panel Gene	Adult HGBCL, NOS
BCL-42	chr5	73911561	73911561	A	G	exonic	ARHGEF28	73	37	0.3364	Missense_Mutation	p.D1645G	Non-Targeted Panel Gene	Adult HGBCL, NOS
BCL-42	chr5	88804642	88804642	A	C	exonic	MEF2C	80	44	0.3548	Missense_Mutation	p.Y72D	Non-Targeted Panel Gene	Adult HGBCL, NOS
BCL-42	chr18	43273685	43273685	A	T	exonic	SYT4	73	43	0.3707	Missense_Mutation	p.F248L	Non-Targeted Panel Gene	Adult HGBCL, NOS
BCL-42	chr3	25599484	25599484	T	C	exonic	TOP2B	43	26	0.3768	Missense_Mutation	p.N1554S	Non-Targeted Panel Gene	Adult HGBCL, NOS
BCL-42	chr1	235182536	235182536	A	C	exonic	ARID4B	13	8	0.381	Missense_Mutation	p.Y795D	Non-Targeted Panel Gene	Adult HGBCL, NOS
BCL-42	chr12	50741499	50741499	G	A	exonic	DIP2B	65	41	0.3868	Missense_Mutation	p.E1480K	Non-Targeted Panel Gene	Adult HGBCL, NOS
BCL-42	chr7	18027445	18027445	G	T	exonic	PRPS1L1	113	73	0.3925	Missense_Mutation	p.A113E	Non-Targeted Panel Gene	Adult HGBCL, NOS
BCL-42	chr19	31277602	31277602	T	A	exonic	TSHZ3	111	72	0.3934	Missense_Mutation	p.I731F	Non-Targeted Panel Gene	Adult HGBCL, NOS
BCL-42	chr1	228736002	228736002	C	T	exonic	RHOU	31	21	0.4038	Missense_Mutation	p.S87F	Non-Targeted Panel Gene	Adult HGBCL, NOS
BCL-42	chr12	113154462	113154462	A	G	exonic	CFAP73	57	39	0.4062	Missense_Mutation	p.T173A	Non-Targeted Panel Gene	Adult HGBCL, NOS
BCL-42	chr19	46755906	46755906	C	G	exonic	FKRP	56	39	0.4105	Missense_Mutation	p.S152R	Non-Targeted Panel Gene	Adult HGBCL, NOS
BCL-42	chr6	152334021	152334021	C	T	exonic	SYNE1	24	17	0.4146	Missense_Mutation	p.D4261N	Non-Targeted Panel Gene	Adult HGBCL, NOS
BCL-42	chr3	141443733	141443733	C	T	exonic	ZBTB38	81	58	0.4173	Missense_Mutation	p.H449Y	Non-Targeted Panel Gene	Adult HGBCL, NOS
BCL-42	chr16	1526792	1526792	C	A	exonic	IFT140	59	43	0.4216	Missense_Mutation	p.A802S	Non-Targeted Panel Gene	Adult HGBCL, NOS
BCL-42	chr16	1614814	1614814	C	T	exonic	CRAMP1	29	22	0.4314	Missense_Mutation	p.P59S	Non-Targeted Panel Gene	Adult HGBCL, NOS
BCL-42	chr5	132587981	132587981	G	T	exonic	RAD50	25	19	0.4318	Missense_Mutation	p.V315L	Non-Targeted Panel Gene	Adult HGBCL, NOS
BCL-42	chr4	68332109	68332109	T	G	exonic	YTHDC1	17	13	0.4333	Missense_Mutation	p.K372N	Non-Targeted Panel Gene	Adult HGBCL, NOS
BCL-42	chr8	93799678	93799678	C	T	exonic	TMEM67	23	18	0.439	Missense_Mutation	p.P640S	Non-Targeted Panel Gene	Adult HGBCL, NOS
BCL-42	chr15	90747435	90747435	C	T	exonic	BLM	14	11	0.44	Missense_Mutation	p.R15C	Non-Targeted Panel Gene	Adult HGBCL, NOS
BCL-42	chr11	2587620	2587620	G	T	exonic	KCNQ1	76	61	0.4453	Missense_Mutation	p.K266N	Non-Targeted Panel Gene	Adult HGBCL, NOS
BCL-42	chr6	64388765	64388765	A	T	exonic	EYS	66	53	0.4454	Missense_Mutation	p.H2001Q	Non-Targeted Panel Gene	Adult HGBCL, NOS
BCL-42	chr14	75047840	75047840	T	C	exonic	MLH3	38	31	0.4493	Missense_Mutation	p.T606A	Non-Targeted Panel Gene	Adult HGBCL, NOS
BCL-42	chr2	178589540	178589540	T	C	exonic	TTN	33	29	0.4677	Missense_Mutation	p.M20729V	Non-Targeted Panel Gene	Adult HGBCL, NOS
BCL-42	chr18	10871338	10871338	A	T	exonic	PIEZO2	25	22	0.4681	Missense_Mutation	p.I136N	Non-Targeted Panel Gene	Adult HGBCL, NOS
BCL-42	chr1	35716267	35716267	G	A	exonic	C1orf216	142	130	0.4779	Missense_Mutation	p.P19S	Non-Targeted Panel Gene	Adult HGBCL, NOS
BCL-42	chr17	42693425	42693425	T	C	exonic	CNTNAP1	176	162	0.4793	Missense_Mutation	p.C961R	Non-Targeted Panel Gene	Adult HGBCL, NOS
BCL-42	chr2	1477339	1477339	A	G	exonic	TPO	81	75	0.4808	Missense_Mutation	p.D358G	Non-Targeted Panel Gene	Adult HGBCL, NOS
BCL-42	chr3	113005859	113005859	A	C	exonic	NEPRO	59	55	0.4825	Missense_Mutation	p.L461V	Non-Targeted Panel Gene	Adult HGBCL, NOS
BCL-42	chr2	120990472	120990472	G	A	exonic	GLI2	60	59	0.4958	Missense_Mutation	p.D1520N	Non-Targeted Panel Gene	Adult HGBCL, NOS
BCL-42	chr5	173146900	173146900	T	C	exonic	BNIP1	63	62	0.496	Missense_Mutation	p.L40P	Non-Targeted Panel Gene	Adult HGBCL, NOS
BCL-42	chr11	119187394	119187394	G	A	exonic	PDZD3	105	105	0.5	Missense_Mutation	p.G152E	Non-Targeted Panel Gene	Adult HGBCL, NOS
BCL-42	chr16	89154148	89154148	C	T	exonic	ACSF3	174	176	0.5029	Missense_Mutation	p.R558W	Non-Targeted Panel Gene	Adult HGBCL, NOS
BCL-42	chr5	39382889	39382889	T	C	exonic	DAB2	134	139	0.5073	Missense_Mutation	p.Q357R	Non-Targeted Panel Gene	Adult HGBCL, NOS
BCL-42	chrX	1642868	1642868	C	T	exonic	ASMT	44	47	0.5165	Missense_Mutation	p.L326F	Non-Targeted Panel Gene	Adult HGBCL, NOS
BCL-42	chr5	135360583	135360583	C	A	exonic	H2AFY	96	104	0.52	Missense_Mutation	p.A168S	Non-Targeted Panel Gene	Adult HGBCL, NOS
BCL-42	chr5	115581109	115581109	T	C	exonic	TICAM2;TMED7-TI	59	64	0.5203	Missense_Mutation	p.N219D	Non-Targeted Panel Gene	Adult HGBCL, NOS
BCL-42	chr19	55358611	55358611	C	T	exonic	FAM71E2	120	134	0.5255	Missense_Mutation	p.V753M	Non-Targeted Panel Gene	Adult HGBCL, NOS
BCL-42	chr7	27785260	27785260	A	T	exonic	TAX1BP1	18	20	0.5263	Missense_Mutation	p.D237V	Non-Targeted Panel Gene	Adult HGBCL, NOS
BCL-42	chr21	45176395	45176395	A	G	exonic	ADARB1	184	208	0.5306	Missense_Mutation	p.S232G	Non-Targeted Panel Gene	Adult HGBCL, NOS
BCL-42	chr13	46250458	46250458	T	C	exonic	LRRC63	9	11	0.55	Missense_Mutation	p.I398T	Non-Targeted Panel Gene	Adult HGBCL, NOS
BCL-42	chr5	132588018	132588018	G	A	exonic	RAD50	18	26	0.5778	Missense_Mutation	p.R327H	Non-Targeted Panel Gene	Adult HGBCL, NOS
BCL-42	chr17	78997391	78997391	T	C	exonic	CANT1	84	115	0.5779	Missense_Mutation	p.R78G	Non-Targeted Panel Gene	Adult HGBCL, NOS
BCL-42	chr1	151288106	151288106	C	A	exonic	ZNF687	161	270	0.6265	Missense_Mutation	p.D605E	Non-Targeted Panel Gene	Adult HGBCL, NOS
BCL-42	chrX	47915200	47915200	C	T	exonic	ZNF81	0	32	1	Missense_Mutation	p.S185L	Non-Targeted Panel Gene	Adult HGBCL, NOS
BCL-42	chr1	54139630	54139630	G	A	exonic	CDCP2	66	97	0.5951	Nonsense_Mutation	p.Q414X	Non-Targeted Panel Gene	Adult HGBCL, NOS
BCL-43	chr4	73153878	73153878	A	G	splicing	ANKRD17	58	6	0.0938	Splice_Site	.	Non-Targeted Panel Gene	Adult HGBCL, NOS
BCL-43	chr12	18738642	18738642	-	A	exonic	CAPZA3	62	7	0.1014	Frame_Shift_Ins	p.L125fs	Non-Targeted Panel Gene	Adult HGBCL, NOS
BCL-43	chr1	1627147	1627149	GGA	-	exonic	MIB2	112	13	0.104	In_Frame_Del	p.553_554del	Non-Targeted Panel Gene	Adult HGBCL, NOS
BCL-43	chr17	17796724	17796726	AGG	-	exonic	RAI1	98	87	0.4703	In_Frame_Del	p.1259_1260del	Non-Targeted Panel Gene	Adult HGBCL, NOS
BCL-43	chrX	67545316	67545316	-	GCAGCAGCAGCA	exonic	AR	30	6	0.1579	In_Frame_Ins	p.L57delinsLQQQQ	Non-Targeted Panel Gene	Adult HGBCL, NOS
BCL-43	chr1	247038288	247038288	C	G	exonic	ZNF670	84	4	0.0455	Missense_Mutation	p.V111L	Non-Targeted Panel Gene	Adult HGBCL, NOS
BCL-43	chr2	12723395	12723395	G	A	exonic	TRIB2	220	12	0.0517	Missense_Mutation	p.D136N	Non-Targeted Panel Gene	Adult HGBCL, NOS
BCL-43	chr12	54362900	54362900	T	C	exonic	GPR84	230	14	0.0574	Missense_Mutation	p.T318A	Non-Targeted Panel Gene	Adult HGBCL, NOS
BCL-43	chr11	100074202	100074202	C	A	exonic	CNTN5	61	5	0.0758	Missense_Mutation	p.D496E	Non-Targeted Panel Gene	Adult HGBCL, NOS
BCL-43	chr1	32192378	32192378	C	T	exonic	TXLNA	119	11	0.0846	Missense_Mutation	p.A344V	Non-Targeted Panel Gene	Adult HGBCL, NOS
BCL-43	chr4	119057770	119057770	T	C	exonic	SYNPO2	117	11	0.0859	Missense_Mutation	p.S1208P	Non-Targeted Panel Gene	Adult HGBCL, NOS
BCL-43	chr16	4262612	4262612	C	T	exonic	TFAP4	135	16	0.1053	Missense_Mutation	p.R60H	Non-Targeted Panel Gene	Adult HGBCL, NOS
BCL-43	chr19	21537207	21537207	C	G	exonic	ZNF429	10	5	0.3333	Missense_Mutation	p.T353S	Non-Targeted Panel Gene	Adult HGBCL, NOS
BCL-43	chr15	91268599	91268599	A	G	exonic	SV2B	48	30	0.3846	Missense_Mutation	p.N456S	Non-Targeted Panel Gene	Adult HGBCL, NOS
BCL-43	chr8	143925278	143925278	G	A	exonic	PLEC	21	14	0.4	Missense_Mutation	p.R1578C	Non-Targeted Panel Gene	Adult HGBCL, NOS
BCL-43	chr11	112086941	112086941	G	A	exonic	SDHD	41	29	0.4085	Missense_Mutation	p.G12S	Non-Targeted Panel Gene	Adult HGBCL, NOS
BCL-43	chr3	47163923	47163923	A	G	exonic	SETD2	10	7	0.4118	Missense_Mutation	p.M1T	Non-Targeted Panel Gene	Adult HGBCL, NOS
BCL-43	chr19	55433347	55433347	C	T	exonic	SHISA7	30	21	0.4118	Missense_Mutation	p.A476T	Non-Targeted Panel Gene	Adult HGBCL, NOS
BCL-43	chr9	5090788	5090788	C	T	exonic	JAK2	27	19	0.413	Missense_Mutation	p.T979M	Non-Targeted Panel Gene	Adult HGBCL, NOS
BCL-43	chr10	25024214	25024214	A	G	exonic	THNSL1	71	52	0.4228	Missense_Mutation	p.R331G	Non-Targeted Panel Gene	Adult HGBCL, NOS
BCL-43	chr4	10443613	10443613	C	T	exonic	ZNF518B	61	45	0.4245	Missense_Mutation	p.E906K	Non-Targeted Panel Gene	Adult HGBCL, NOS
BCL-43	chr14	91175949	91175949	A	G	exonic	C14orf159	57	44	0.4356	Missense_Mutation	p.K208R	Non-Targeted Panel Gene	Adult HGBCL, NOS
BCL-43	chr10	20787306	20787306	G	T	exonic	NEBL	37	29	0.4394	Missense_Mutation	p.P922T	Non-Targeted Panel Gene	Adult HGBCL, NOS
BCL-43	chr18	580667	580667	A	G	exonic	CETN1	155	124	0.4444	Missense_Mutation	p.N87D	Non-Targeted Panel Gene	Adult HGBCL, NOS
BCL-43	chr1	53459794	53459794	G	A	exonic	DMRTB1	20	16	0.4444	Missense_Mutation	p.R114H	Non-Targeted Panel Gene	Adult HGBCL, NOS
BCL-43	chr5	132393688	132393688	G	A	exonic	SLC22A5	53	43	0.4479	Missense_Mutation	p.R512H	Non-Targeted Panel Gene	Adult HGBCL, NOS
BCL-43	chr12	76805348	76805348	G	T	exonic	ZDHHC17	34	28	0.4516	Missense_Mutation	p.V77L	Non-Targeted Panel Gene	Adult HGBCL, NOS
BCL-43	chr18	62975543	62975543	C	T	exonic	PHLPP1	95	79	0.454	Missense_Mutation	p.P1301L	Non-Targeted Panel Gene	Adult HGBCL, NOS
BCL-43	chr9	92722783	92722783	C	A	exonic	BICD2	78	65	0.4545	Missense_Mutation	p.R160L	Non-Targeted Panel Gene	Adult HGBCL, NOS
BCL-43	chr19	11100303	11100303	G	T	exonic	LDLR	126	108	0.4615	Missense_Mutation	p.A50S	Non-Targeted Panel Gene	Adult HGBCL, NOS
BCL-43	chr11	17495607	17495607	C	T	exonic	USH1C	90	79	0.4675	Missense_Mutation	p.V873M	Non-Targeted Panel Gene	Adult HGBCL, NOS
BCL-43	chr8	54626758	54626758	C	A	exonic	RP1	27	24	0.4706	Missense_Mutation	p.T959K	Non-Targeted Panel Gene	Adult HGBCL, NOS
BCL-43	chr15	89330184	89330184	G	A	exonic	POLG	101	90	0.4712	Missense_Mutation	p.T251I	Non-Targeted Panel Gene	Adult HGBCL, NOS
BCL-43	chr17	73169918	73169918	A	G	exonic	SSTR2	80	73	0.4771	Missense_Mutation	p.E200G	Non-Targeted Panel Gene	Adult HGBCL, NOS
BCL-43	chr3	125497856	125497856	A	T	exonic	SNX4	12	11	0.4783	Missense_Mutation	p.I176N	Non-Targeted Panel Gene	Adult HGBCL, NOS

BCL-43	chr3	47265781	47265781	G	A	exonic	KIF9	67	62	0.4806	Missense_Mutation	p.H289Y	Non-Targeted Panel Gene	Adult HGBCL, NOS
BCL-43	chr19	44157995	44157995	G	T	exonic	ZNF234	26	26	0.5	Missense_Mutation	p.R660M	Non-Targeted Panel Gene	Adult HGBCL, NOS
BCL-43	chr6	72182890	72182890	G	C	exonic	RIMS1	92	93	0.5027	Missense_Mutation	p.Q473H	Non-Targeted Panel Gene	Adult HGBCL, NOS
BCL-43	chr22	25768308	25768308	T	C	exonic	MYO18B	124	126	0.504	Missense_Mutation	p.L131P	Non-Targeted Panel Gene	Adult HGBCL, NOS
BCL-43	chr11	112025527	112025527	G	C	exonic	DLAT	50	51	0.505	Missense_Mutation	p.E19Q	Non-Targeted Panel Gene	Adult HGBCL, NOS
BCL-43	chr2	237366801	237366801	C	G	exonic	COL6A3	149	155	0.5099	Missense_Mutation	p.A1796P	Non-Targeted Panel Gene	Adult HGBCL, NOS
BCL-43	chr12	55726208	55726208	C	T	exonic	CD63	51	54	0.5143	Missense_Mutation	p.M160I	Non-Targeted Panel Gene	Adult HGBCL, NOS
BCL-43	chr16	67287144	67287144	C	T	exonic	PLEKHG4	83	89	0.5174	Missense_Mutation	p.H1024Y	Non-Targeted Panel Gene	Adult HGBCL, NOS
BCL-43	chr4	16759199	16759199	A	G	exonic	LDB2	34	37	0.5211	Missense_Mutation	p.L65S	Non-Targeted Panel Gene	Adult HGBCL, NOS
BCL-43	chr6	146752699	146752699	G	A	exonic	ADGB	19	21	0.525	Missense_Mutation	p.G1179S	Non-Targeted Panel Gene	Adult HGBCL, NOS
BCL-43	chr15	89321842	89321842	T	C	exonic	POLG	72	81	0.5294	Missense_Mutation	p.Y831C	Non-Targeted Panel Gene	Adult HGBCL, NOS
BCL-43	chr3	44244528	44244528	C	G	exonic	TOPAZ1	23	26	0.5306	Missense_Mutation	p.D674E	Non-Targeted Panel Gene	Adult HGBCL, NOS
BCL-43	chr1	94051698	94051698	C	G	exonic	ABCA4	30	38	0.5588	Missense_Mutation	p.G863A	Non-Targeted Panel Gene	Adult HGBCL, NOS
BCL-43	chr9	15623409	15623409	T	A	exonic	CCDC171	6	9	0.6	Missense_Mutation	p.F180Y	Non-Targeted Panel Gene	Adult HGBCL, NOS
BCL-43	chr21	14594047	14594047	T	C	exonic	LOC388813	11	17	0.6071	Missense_Mutation	p.Y116C	Non-Targeted Panel Gene	Adult HGBCL, NOS
BCL-43	chr21	33403583	33403583	G	A	exonic	IFNGR2	3	6	0.6667	Missense_Mutation	p.G14R	Non-Targeted Panel Gene	Adult HGBCL, NOS
BCL-43	chr9	21031553	21031553	C	T	exonic	HACD4	6	19	0.76	Missense_Mutation	p.R13K	Non-Targeted Panel Gene	Adult HGBCL, NOS
BCL-43	chr3	108634896	108634896	A	G	exonic	DZIP3	1	9	0.9	Missense_Mutation	p.K281R	Non-Targeted Panel Gene	Adult HGBCL, NOS
BCL-43	chrX	83874077	83874077	G	C	exonic	CYLC1	0	12	1	Missense_Mutation	p.G457R	Non-Targeted Panel Gene	Adult HGBCL, NOS
BCL-43	chr1	35451631	35451631	C	A	exonic	KIAA0319L	152	11	0.0675	Nonsense_Mutation	p.E687X	Non-Targeted Panel Gene	Adult HGBCL, NOS
BCL-43	chr3	185519239	185519239	G	-	exonic	LIPH	32	32	0.5	Nonsense_Mutation	p.C263X	Non-Targeted Panel Gene	Adult HGBCL, NOS
BCL-44	chr2	187484810	187484810	C	A	splicing	TFPI	18	13	0.4194	Splice_Site	.	Non-Targeted Panel Gene	Adult HGBCL, NOS
BCL-44	chr19	535924	535924	G	A	splicing	CDC34	63	60	0.4878	Splice_Site	.	Non-Targeted Panel Gene	Adult HGBCL, NOS
BCL-44	chr4	99539321	99539321	G	-	exonic	C4orf17	46	37	0.4458	Frame_Shift_Del	p.A263fs	Non-Targeted Panel Gene	Adult HGBCL, NOS
BCL-44	chr13	99295092	99295092	-	C	exonic	GPR183	61	45	0.4206	Frame_Shift_Ins	p.M352fs	Non-Targeted Panel Gene	Adult HGBCL, NOS
BCL-44	chr8	144138260	144138268	TGGCGGCGC	-	exonic	HGH1	19	10	0.3448	In_Frame_Del	p.142_145del	Non-Targeted Panel Gene	Adult HGBCL, NOS
BCL-44	chr3	46996346	46996346	G	C	exonic	NBEAL2	32	27	0.4576	Missense_Mutation	p.V743L	Targerted Panel Gene	Adult HGBCL, NOS
BCL-44	chr10	121565644	121565644	G	A	exonic	FGFR2	92	93	0.5027	Missense_Mutation	p.S57L	Targerted Panel Gene	Adult HGBCL, NOS
BCL-44	chr7	82914995	82914995	C	T	exonic	PCLO	105	109	0.5093	Missense_Mutation	p.A4331T	Targerted Panel Gene	Adult HGBCL, NOS
BCL-44	chr16	88804656	88804656	G	C	exonic	CDT1	49	51	0.51	Missense_Mutation	p.A114P	Targerted Panel Gene	Adult HGBCL, NOS
BCL-44	chr12	26122565	26122565	C	T	exonic	BHLHE41	10	5	0.3333	Missense_Mutation	p.R317K	Non-Targeted Panel Gene	Adult HGBCL, NOS
BCL-44	chr19	48956251	48956251	C	T	exonic	BAX	83	47	0.3615	Missense_Mutation	p.A96V	Non-Targeted Panel Gene	Adult HGBCL, NOS
BCL-44	chr6	43780743	43780743	G	A	exonic	VEGFA	15	9	0.375	Missense_Mutation	p.R325Q	Non-Targeted Panel Gene	Adult HGBCL, NOS
BCL-44	chr20	3697337	3697337	C	A	exonic	SIGLEC1	128	79	0.3816	Missense_Mutation	p.V710F	Non-Targeted Panel Gene	Adult HGBCL, NOS
BCL-44	chr21	45515837	45515837	C	T	exonic	SLC19A1	133	94	0.4141	Missense_Mutation	p.A533T	Non-Targeted Panel Gene	Adult HGBCL, NOS
BCL-44	chr1	209609088	209609088	G	C	exonic	CAMK1G	67	50	0.4274	Missense_Mutation	p.E248D	Non-Targeted Panel Gene	Adult HGBCL, NOS
BCL-44	chr12	1830937	1830937	A	G	exonic	LRTM2	41	32	0.4384	Missense_Mutation	p.I24V	Non-Targeted Panel Gene	Adult HGBCL, NOS
BCL-44	chr7	92532474	92532474	T	A	exonic	RBM48	20	16	0.4444	Missense_Mutation	p.F125I	Non-Targeted Panel Gene	Adult HGBCL, NOS
BCL-44	chr1	52854610	52854610	T	G	exonic	ZYG11A	67	54	0.4463	Missense_Mutation	p.L79R	Non-Targeted Panel Gene	Adult HGBCL, NOS
BCL-44	chr7	94549621	94549621	G	A	exonic	CASD1	32	26	0.4483	Missense_Mutation	p.R601K	Non-Targeted Panel Gene	Adult HGBCL, NOS
BCL-44	chr3	123629496	123629496	T	G	exonic	MYLK	93	76	0.4497	Missense_Mutation	p.N1698H	Non-Targeted Panel Gene	Adult HGBCL, NOS
BCL-44	chr20	58418300	58418300	C	T	exonic	VAPB	89	73	0.4506	Missense_Mutation	p.R50C	Non-Targeted Panel Gene	Adult HGBCL, NOS
BCL-44	chr2	178576311	178576311	C	T	exonic	TTN	57	47	0.4519	Missense_Mutation	p.G23274D	Non-Targeted Panel Gene	Adult HGBCL, NOS
BCL-44	chr12	40943637	40943637	A	T	exonic	CNTN1	23	19	0.4524	Missense_Mutation	p.I474F	Non-Targeted Panel Gene	Adult HGBCL, NOS
BCL-44	chr11	64343520	64343520	T	G	exonic	CCDC88B	55	46	0.4554	Missense_Mutation	p.L408R	Non-Targeted Panel Gene	Adult HGBCL, NOS
BCL-44	chr19	56423571	56423571	T	G	exonic	ZNF583	43	36	0.4557	Missense_Mutation	p.F305V	Non-Targeted Panel Gene	Adult HGBCL, NOS
BCL-44	chr1	52854620	52854620	G	A	exonic	ZYG11A	64	54	0.4576	Missense_Mutation	p.M82I	Non-Targeted Panel Gene	Adult HGBCL, NOS
BCL-44	chr2	189039508	189039508	G	C	exonic	COL5A2	52	46	0.4694	Missense_Mutation	p.T1230R	Non-Targeted Panel Gene	Adult HGBCL, NOS
BCL-44	chr17	8004153	8004153	G	T	exonic	GUCY2D	26	24	0.48	Missense_Mutation	p.Q341H	Non-Targeted Panel Gene	Adult HGBCL, NOS
BCL-44	chr20	45115023	45115023	C	T	exonic	WFDC5	31	29	0.4833	Missense_Mutation	p.A21T	Non-Targeted Panel Gene	Adult HGBCL, NOS
BCL-44	chr7	129189239	129189239	G	A	exonic	SMO	19	18	0.4865	Missense_Mutation	p.A30T	Non-Targeted Panel Gene	Adult HGBCL, NOS
BCL-44	chr9	95150025	95150025	T	A	exonic	FANCC	53	51	0.4904	Missense_Mutation	p.D195V	Non-Targeted Panel Gene	Adult HGBCL, NOS
BCL-44	chr3	124661013	124661013	C	T	exonic	KALRN	38	37	0.4933	Missense_Mutation	p.P375L	Non-Targeted Panel Gene	Adult HGBCL, NOS
BCL-44	chr2	148783738	148783738	G	A	exonic	EPC2	86	85	0.4971	Missense_Mutation	p.G667S	Non-Targeted Panel Gene	Adult HGBCL, NOS
BCL-44	chr11	64231936	64231936	A	G	exonic	DNAJC4	107	107	0.5	Missense_Mutation	p.K51R	Non-Targeted Panel Gene	Adult HGBCL, NOS
BCL-44	chr11	1836273	1836273	T	C	exonic	SYT8	80	81	0.5031	Missense_Mutation	p.C183R	Non-Targeted Panel Gene	Adult HGBCL, NOS
BCL-44	chr10	132405380	132405380	C	T	exonic	PWWP2B	63	64	0.5039	Missense_Mutation	p.P294S	Non-Targeted Panel Gene	Adult HGBCL, NOS
BCL-44	chr9	136392015	136392015	T	C	exonic	SNAPC4	46	47	0.5054	Missense_Mutation	p.E301G	Non-Targeted Panel Gene	Adult HGBCL, NOS
BCL-44	chr2	128318368	128318368	T	C	exonic	HS6ST1	93	98	0.5104	Missense_Mutation	p.K66E	Non-Targeted Panel Gene	Adult HGBCL, NOS
BCL-44	chr17	42980809	42980809	G	A	exonic	RUNDC1	22	23	0.5111	Missense_Mutation	p.R78Q	Non-Targeted Panel Gene	Adult HGBCL, NOS
BCL-44	chr2	75051257	75051257	T	C	exonic	TACR1	74	78	0.5132	Missense_Mutation	p.N309S	Non-Targeted Panel Gene	Adult HGBCL, NOS
BCL-44	chr8	76983431	76983431	A	G	exonic	PEX2	56	61	0.5214	Missense_Mutation	p.W250R	Non-Targeted Panel Gene	Adult HGBCL, NOS
BCL-44	chr8	144263042	144263042	G	A	exonic	BOP1	42	46	0.5227	Missense_Mutation	p.R569C	Non-Targeted Panel Gene	Adult HGBCL, NOS
BCL-44	chr1	172555995	172555995	T	C	exonic	SUCO	33	37	0.5286	Missense_Mutation	p.S297P	Non-Targeted Panel Gene	Adult HGBCL, NOS
BCL-44	chr12	57455832	57455832	C	T	exonic	INHBE	55	62	0.5299	Missense_Mutation	p.T99I	Non-Targeted Panel Gene	Adult HGBCL, NOS
BCL-44	chrX	153778288	153778288	G	A	exonic	PLXNB3	117	132	0.5301	Missense_Mutation	p.A1836T	Non-Targeted Panel Gene	Adult HGBCL, NOS
BCL-44	chr3	9906385	9906385	A	G	exonic	IL17RE	31	35	0.5303	Missense_Mutation	p.H137R	Non-Targeted Panel Gene	Adult HGBCL, NOS
BCL-44	chr12	12949613	12949613	T	G	exonic	GPRC5D	125	143	0.5336	Missense_Mutation	p.I258L	Non-Targeted Panel Gene	Adult HGBCL, NOS
BCL-44	chr5	42779477	42779477	A	T	exonic	CCDC152	5	6	0.5455	Missense_Mutation	p.K94N	Non-Targeted Panel Gene	Adult HGBCL, NOS
BCL-44	chr3	100368123	100368123	C	G	exonic	TOMM70	12	15	0.5556	Missense_Mutation	p.E532Q	Non-Targeted Panel Gene	Adult HGBCL, NOS
BCL-44	chr17	40260616	40260616	A	G	exonic	WIPF2	84	106	0.5579	Missense_Mutation	p.K49E	Non-Targeted Panel Gene	Adult HGBCL, NOS
BCL-44	chr3	188484607	188484607	C	A	exonic	LPP	21	27	0.5625	Missense_Mutation	p.P70H	Non-Targeted Panel Gene	Adult HGBCL, NOS
BCL-44	chr17	17793558	17793558	C	T	exonic	RAI1	32	42	0.5676	Missense_Mutation	p.P204S	Non-Targeted Panel Gene	Adult HGBCL, NOS
BCL-44	chr12	95878303	95878303	T	C	exonic	CCDC38	8	11	0.5789	Missense_Mutation	p.K396E	Non-Targeted Panel Gene	Adult HGBCL, NOS
BCL-44	chr11	5422900	5422900	G	A	exonic	OR51Q1	26	36	0.5806	Missense_Mutation	p.A234T	Non-Targeted Panel Gene	Adult HGBCL, NOS
BCL-44	chr11	120475418	120475418	G	A	exonic	ARHGEF12	35	59	0.6277	Missense_Mutation	p.S1063N	Non-Targeted Panel Gene	Adult HGBCL, NOS
BCL-5	chr17	63929769	63929769	C	T	splicing	CD79B	73	52	0.416	Splice_Site	.	Targerted Panel Gene	Adult HGBCL, NOS
BCL-5	chr9	127665332	127665332	G	A	splicing	STXBP1	67	2	0.029	Splice_Site	.	Non-Targeted Panel Gene	Adult HGBCL, NOS
BCL-5	chr13	41587511	41587511	C	T	splicing	VWA8	92	3	0.0316	Splice_Site	.	Non-Targeted Panel Gene	Adult HGBCL, NOS
BCL-5	chr16	2233991	2233991	G	A	splicing	E4F1	58	2	0.0333	Splice_Site	.	Non-Targeted Panel Gene	Adult HGBCL, NOS
BCL-5	chr5	55405589	55405589	G	A	splicing	SKIV2L2	86	3	0.0337	Splice_Site	.	Non-Targeted Panel Gene	Adult HGBCL, NOS
BCL-5	chr3	57301734	57301734	C	T	splicing	DNAH12	52	2	0.037	Splice_Site	.	Non-Targeted Panel Gene	Adult HGBCL, NOS

BCL-5	chr11	62597609	62597609	C	T	splicing	MTA2	75	3	0.0385	Splice_Site	.	Non-Targeted Panel Gene	Adult HGBCL, NOS
BCL-5	chr7	2518599	2518599	G	A	splicing	LFNG	48	2	0.04	Splice_Site	.	Non-Targeted Panel Gene	Adult HGBCL, NOS
BCL-5	chr16	15741759	15741759	C	T	splicing	MYH11	68	3	0.0423	Splice_Site	.	Non-Targeted Panel Gene	Adult HGBCL, NOS
BCL-5	chr12	49323753	49323753	G	A	splicing	TROAP	67	3	0.0429	Splice_Site	.	Non-Targeted Panel Gene	Adult HGBCL, NOS
BCL-5	chr12	5575833	5575833	C	T	splicing	ANO2	43	2	0.0444	Splice_Site	.	Non-Targeted Panel Gene	Adult HGBCL, NOS
BCL-5	chr7	43596114	43596114	G	A	splicing	STK17A	61	3	0.0469	Splice_Site	.	Non-Targeted Panel Gene	Adult HGBCL, NOS
BCL-5	chr4	76382609	76382609	C	T	splicing	CCDC158	80	4	0.0476	Splice_Site	.	Non-Targeted Panel Gene	Adult HGBCL, NOS
BCL-5	chrX	11760499	11760499	G	A	splicing	MSL3	60	3	0.0476	Splice_Site	.	Non-Targeted Panel Gene	Adult HGBCL, NOS
BCL-5	chr1	52978368	52978368	G	A	splicing	SCP2	40	2	0.0476	Splice_Site	.	Non-Targeted Panel Gene	Adult HGBCL, NOS
BCL-5	chr2	137616174	137616174	G	A	splicing	THSD7B	40	2	0.0476	Splice_Site	.	Non-Targeted Panel Gene	Adult HGBCL, NOS
BCL-5	chr14	73284080	73284080	C	T	splicing	NUMB	59	3	0.0484	Splice_Site	.	Non-Targeted Panel Gene	Adult HGBCL, NOS
BCL-5	chr1	28529940	28529940	G	A	splicing	RCC1	56	3	0.0508	Splice_Site	.	Non-Targeted Panel Gene	Adult HGBCL, NOS
BCL-5	chr1	3891058	3891058	C	T	splicing	C1orf174	37	2	0.0513	Splice_Site	.	Non-Targeted Panel Gene	Adult HGBCL, NOS
BCL-5	chr1	184754488	184754488	C	T	splicing	EDEM3	103	6	0.055	Splice_Site	.	Non-Targeted Panel Gene	Adult HGBCL, NOS
BCL-5	chr21	46117937	46117937	G	A	splicing	COL6A2	33	2	0.0571	Splice_Site	.	Non-Targeted Panel Gene	Adult HGBCL, NOS
BCL-5	chr15	37097966	37097966	C	T	splicing	MEIS2	33	2	0.0571	Splice_Site	.	Non-Targeted Panel Gene	Adult HGBCL, NOS
BCL-5	chr3	179224822	179224822	G	A	splicing	PIK3CA	45	3	0.0625	Splice_Site	.	Non-Targeted Panel Gene	Adult HGBCL, NOS
BCL-5	chr2	190930569	190930569	G	A	splicing	GLS	29	2	0.0645	Splice_Site	.	Non-Targeted Panel Gene	Adult HGBCL, NOS
BCL-5	chr3	171177325	171177325	C	T	splicing	TNIK	43	3	0.0652	Splice_Site	.	Non-Targeted Panel Gene	Adult HGBCL, NOS
BCL-5	chr16	21033973	21033973	C	T	splicing	DNAH3	28	2	0.0667	Splice_Site	.	Non-Targeted Panel Gene	Adult HGBCL, NOS
BCL-5	chr6	33674266	33674266	G	A	splicing	ITPR3	28	2	0.0667	Splice_Site	.	Non-Targeted Panel Gene	Adult HGBCL, NOS
BCL-5	chr1	43432605	43432605	G	A	splicing	SZT2	41	3	0.0682	Splice_Site	.	Non-Targeted Panel Gene	Adult HGBCL, NOS
BCL-5	chr10	54346364	54346364	C	T	splicing	PCDH15	53	4	0.0702	Splice_Site	.	Non-Targeted Panel Gene	Adult HGBCL, NOS
BCL-5	chr17	81233980	81233980	C	T	splicing	TEPSIN	26	2	0.0714	Splice_Site	.	Non-Targeted Panel Gene	Adult HGBCL, NOS
BCL-5	chr12	132715037	132715037	G	A	splicing	PGAM5	37	3	0.075	Splice_Site	.	Non-Targeted Panel Gene	Adult HGBCL, NOS
BCL-5	chr17	67940657	67940657	G	A	splicing	BPTF	49	4	0.0755	Splice_Site	.	Non-Targeted Panel Gene	Adult HGBCL, NOS
BCL-5	chr14	96471607	96471607	G	A	splicing	AK7	23	2	0.08	Splice_Site	.	Non-Targeted Panel Gene	Adult HGBCL, NOS
BCL-5	chr3	52471978	52471978	G	A	splicing	NISCH	23	2	0.08	Splice_Site	.	Non-Targeted Panel Gene	Adult HGBCL, NOS
BCL-5	chr7	48246031	48246031	G	A	splicing	ABCA13	34	3	0.0811	Splice_Site	.	Non-Targeted Panel Gene	Adult HGBCL, NOS
BCL-5	chr1	153776728	153776728	G	A	splicing	SLC27A3	34	3	0.0811	Splice_Site	.	Non-Targeted Panel Gene	Adult HGBCL, NOS
BCL-5	chr2	74093667	74093667	G	A	splicing	TET3	22	2	0.0833	Splice_Site	.	Non-Targeted Panel Gene	Adult HGBCL, NOS
BCL-5	chr15	42751973	42751973	C	T	splicing	TTBK2	32	3	0.0833	Splice_Site	.	Non-Targeted Panel Gene	Adult HGBCL, NOS
BCL-5	chr12	12519861	12519861	C	T	splicing	DUSP16	32	3	0.0857	Splice_Site	.	Non-Targeted Panel Gene	Adult HGBCL, NOS
BCL-5	chr18	23899066	23899066	G	A	splicing	LAMA3	20	2	0.0909	Splice_Site	.	Non-Targeted Panel Gene	Adult HGBCL, NOS
BCL-5	chr2	215409562	215409562	C	T	splicing	FN1	27	3	0.1	Splice_Site	.	Non-Targeted Panel Gene	Adult HGBCL, NOS
BCL-5	chr12	128888745	128888745	G	A	splicing	GLT1D1	36	4	0.1	Splice_Site	.	Non-Targeted Panel Gene	Adult HGBCL, NOS
BCL-5	chr8	89780161	89780161	G	A	splicing	RIPK2	35	4	0.1026	Splice_Site	.	Non-Targeted Panel Gene	Adult HGBCL, NOS
BCL-5	chr16	21054419	21054419	C	T	splicing	DNAH3	24	3	0.1111	Splice_Site	.	Non-Targeted Panel Gene	Adult HGBCL, NOS
BCL-5	chr11	65647659	65647659	G	A	splicing	SIPA1	24	3	0.1111	Splice_Site	.	Non-Targeted Panel Gene	Adult HGBCL, NOS
BCL-5	chr6	158081511	158081511	G	A	splicing	SYNJ2	13	2	0.1333	Splice_Site	.	Non-Targeted Panel Gene	Adult HGBCL, NOS
BCL-5	chr11	44267493	44267493	C	T	splicing	ALX4	12	2	0.1429	Splice_Site	.	Non-Targeted Panel Gene	Adult HGBCL, NOS
BCL-5	chr14	39179463	39179463	G	A	splicing	PNN	15	3	0.1667	Splice_Site	.	Non-Targeted Panel Gene	Adult HGBCL, NOS
BCL-5	chr17	65014593	65014594	TG	-	exonic	GNA13	179	159	0.4676	Frame_Shift_Del	p.T266fs	Targerted Panel Gene	Adult HGBCL, NOS
BCL-5	chr17	65014433	65014434	TT	-	exonic	GNA13	194	120	0.3822	Frame_Shift_Del	p.L319fs	Targerted Panel Gene	Adult HGBCL, NOS
BCL-5	chr5	98881281	98881282	GG	-	exonic	CHD1	52	2	0.037	Frame_Shift_Del	p.P987fs	Non-Targeted Panel Gene	Adult HGBCL, NOS
BCL-5	chr6	146644846	146644846	A	-	exonic	ADGB	26	15	0.3659	Frame_Shift_Del	p.Q104fs	Non-Targeted Panel Gene	Adult HGBCL, NOS
BCL-5	chr17	17796724	17796726	AGG	-	exonic	RAI1	20	12	0.375	In_Frame_Del	p.1259_1260del	Non-Targeted Panel Gene	Adult HGBCL, NOS
BCL-5	chr20	36803018	36803018	-	TGC	exonic	SOGA1	23	8	0.2581	In_Frame_Ins	p.H821delinsQH	Non-Targeted Panel Gene	Adult HGBCL, NOS
BCL-5	chr1	16996476	16996476	C	A	exonic	ATP13A2	60	9	0.1286	Missense_Mutation	p.G406C	Targerted Panel Gene	Adult HGBCL, NOS
BCL-5	chr6	41935968	41935968	G	A	exonic	CCND3	90	57	0.3851	Missense_Mutation	p.P284L	Targerted Panel Gene	Adult HGBCL, NOS
BCL-5	chr10	17084439	17084439	G	C	exonic	CUBN	40	29	0.4203	Missense_Mutation	p.N711K	Targerted Panel Gene	Adult HGBCL, NOS
BCL-5	chr6	106106393	106106393	G	A	exonic	PRDM1	32	77	0.7064	Missense_Mutation	p.G599E	Targerted Panel Gene	Adult HGBCL, NOS
BCL-5	chr14	100277467	100277467	G	A	exonic	YY1	104	85	0.4497	Missense_Mutation	p.R371H	Targerted Panel Gene	Adult HGBCL, NOS
BCL-5	chr4	147654371	147654371	C	T	exonic	PRMT9	87	2	0.0225	Missense_Mutation	p.S396N	Non-Targeted Panel Gene	Adult HGBCL, NOS
BCL-5	chr16	88429352	88429352	C	T	exonic	ZNF469	77	2	0.025	Missense_Mutation	p.P628S	Non-Targeted Panel Gene	Adult HGBCL, NOS
BCL-5	chr8	90645544	90645544	C	T	exonic	TMEM64	63	2	0.0308	Missense_Mutation	p.R121Q	Non-Targeted Panel Gene	Adult HGBCL, NOS
BCL-5	chr1	225514705	225514705	G	A	exonic	ENAH	61	2	0.0317	Missense_Mutation	p.P370L	Non-Targeted Panel Gene	Adult HGBCL, NOS
BCL-5	chr9	129049450	129049450	G	A	exonic	MIGA2	61	2	0.0317	Missense_Mutation	p.E228K	Non-Targeted Panel Gene	Adult HGBCL, NOS
BCL-5	chr6	25779153	25779153	G	A	exonic	SLC17A4	61	2	0.0317	Missense_Mutation	p.D433N	Non-Targeted Panel Gene	Adult HGBCL, NOS
BCL-5	chrX	152970446	152970446	G	A	exonic	ZNF185	61	2	0.0317	Missense_Mutation	p.G691R	Non-Targeted Panel Gene	Adult HGBCL, NOS
BCL-5	chr19	44908616	44908616	C	T	exonic	APOE	59	2	0.0328	Missense_Mutation	p.T107M	Non-Targeted Panel Gene	Adult HGBCL, NOS
BCL-5	chr5	138346924	138346924	G	T	exonic	FAM53C	58	2	0.0328	Missense_Mutation	p.G382W	Non-Targeted Panel Gene	Adult HGBCL, NOS
BCL-5	chr20	63488309	63488309	C	T	exonic	EEF1A2	58	2	0.0333	Missense_Mutation	p.A461T	Non-Targeted Panel Gene	Adult HGBCL, NOS
BCL-5	chr1	41582819	41582819	G	A	exonic	HIVEP3	87	3	0.0333	Missense_Mutation	p.A660V	Non-Targeted Panel Gene	Adult HGBCL, NOS
BCL-5	chr3	159993767	159993767	G	A	exonic	IL12A	58	2	0.0333	Missense_Mutation	p.A177T	Non-Targeted Panel Gene	Adult HGBCL, NOS
BCL-5	chr7	36357389	36357389	G	A	exonic	KIAA0895	58	2	0.0333	Missense_Mutation	p.S127F	Non-Targeted Panel Gene	Adult HGBCL, NOS
BCL-5	chr1	35560506	35560506	G	A	exonic	NCDN	58	2	0.0333	Missense_Mutation	p.E119K	Non-Targeted Panel Gene	Adult HGBCL, NOS
BCL-5	chr19	49595543	49595543	C	T	exonic	PRR12	58	2	0.0333	Missense_Mutation	p.A403V	Non-Targeted Panel Gene	Adult HGBCL, NOS
BCL-5	chr2	173017431	173017431	G	T	exonic	RAPGEF4	86	3	0.0333	Missense_Mutation	p.K645N	Non-Targeted Panel Gene	Adult HGBCL, NOS
BCL-5	chr1	9749835	9749835	C	A	exonic	CLSTN1	57	2	0.0339	Missense_Mutation	p.G243V	Non-Targeted Panel Gene	Adult HGBCL, NOS
BCL-5	chr11	6224202	6224202	C	T	exonic	FAM160A2	57	2	0.0339	Missense_Mutation	p.G62D	Non-Targeted Panel Gene	Adult HGBCL, NOS
BCL-5	chr4	109463429	109463429	C	T	exonic	SEC24B	85	3	0.0341	Missense_Mutation	p.P252L	Non-Targeted Panel Gene	Adult HGBCL, NOS
BCL-5	chr8	39013976	39013976	C	T	exonic	ADAM9	56	2	0.0345	Missense_Mutation	p.P89L	Non-Targeted Panel Gene	Adult HGBCL, NOS
BCL-5	chr3	124809068	124809068	G	A	exonic	ITGB5	56	2	0.0345	Missense_Mutation	p.S406F	Non-Targeted Panel Gene	Adult HGBCL, NOS
BCL-5	chr22	36294291	36294291	G	A	exonic	MYH9	56	2	0.0345	Missense_Mutation	p.A1213V	Non-Targeted Panel Gene	Adult HGBCL, NOS
BCL-5	chr10	70534445	70534445	A	G	exonic	PALD1	56	2	0.0345	Missense_Mutation	p.K348R	Non-Targeted Panel Gene	Adult HGBCL, NOS
BCL-5	chr12	123914868	123914868	C	T	exonic	DNAH10	55	2	0.0351	Missense_Mutation	p.L3413F	Non-Targeted Panel Gene	Adult HGBCL, NOS
BCL-5	chr11	5234145	5234145	G	A	exonic	HBD	55	2	0.0351	Missense_Mutation	p.A54V	Non-Targeted Panel Gene	Adult HGBCL, NOS
BCL-5	chr11	4907709	4907709	G	A	exonic	OR51A7	55	2	0.0351	Missense_Mutation	p.V114I	Non-Targeted Panel Gene	Adult HGBCL, NOS
BCL-5	chr15	66781449	66781449	G	A	exonic	SMAD6	55	2	0.0351	Missense_Mutation	p.A469T	Non-Targeted Panel Gene	Adult HGBCL, NOS
BCL-5	chr3	196323725	196323725	G	A	exonic	TM4SF19	55	2	0.0351	Missense_Mutation	p.L240F	Non-Targeted Panel Gene	Adult HGBCL, NOS
BCL-5	chr10	73499056	73499056	G	A	exonic	USP54	54	2	0.0351	Missense_Mutation	p.A1543V	Non-Targeted Panel Gene	Adult HGBCL, NOS

BCL-5	chr9	111653124	111653124	G	A	exonic	DNAJC25	54	2	0.0357	Missense_Mutation	p.E329K	Non-Targeted Panel Gene	Adult HGBCL, NOS
BCL-5	chr5	128464751	128464751	G	A	exonic	FBN2	54	2	0.0357	Missense_Mutation	p.P267S	Non-Targeted Panel Gene	Adult HGBCL, NOS
BCL-5	chr11	105933944	105933944	G	A	exonic	GRIA4	54	2	0.0357	Missense_Mutation	p.A757T	Non-Targeted Panel Gene	Adult HGBCL, NOS
BCL-5	chr12	4810723	4810723	G	A	exonic	KCNA6	54	2	0.0357	Missense_Mutation	p.E228K	Non-Targeted Panel Gene	Adult HGBCL, NOS
BCL-5	chr8	143430340	143430340	C	T	exonic	MAFA	53	2	0.0357	Missense_Mutation	p.D23N	Non-Targeted Panel Gene	Adult HGBCL, NOS
BCL-5	chr5	41064527	41064527	C	T	exonic	MROH2B	54	2	0.0357	Missense_Mutation	p.M135I	Non-Targeted Panel Gene	Adult HGBCL, NOS
BCL-5	chr1	154947538	154947538	C	A	exonic	PBXIP1	54	2	0.0357	Missense_Mutation	p.R250M	Non-Targeted Panel Gene	Adult HGBCL, NOS
BCL-5	chr15	70066044	70066044	C	T	exonic	TLE3	81	3	0.0357	Missense_Mutation	p.E183K	Non-Targeted Panel Gene	Adult HGBCL, NOS
BCL-5	chr11	128936559	128936559	C	T	exonic	TP53AIP1	54	2	0.0357	Missense_Mutation	p.A78T	Non-Targeted Panel Gene	Adult HGBCL, NOS
BCL-5	chr9	98087811	98087811	C	T	exonic	TRIM14	54	2	0.0357	Missense_Mutation	p.E330K	Non-Targeted Panel Gene	Adult HGBCL, NOS
BCL-5	chr19	47226613	47226613	G	A	exonic	BBC3	53	2	0.0364	Missense_Mutation	p.A139V	Non-Targeted Panel Gene	Adult HGBCL, NOS
BCL-5	chr9	124032574	124032574	C	T	exonic	LHX2	53	2	0.0364	Missense_Mutation	p.T363M	Non-Targeted Panel Gene	Adult HGBCL, NOS
BCL-5	chr7	30135282	30135282	G	A	exonic	MTURN	53	2	0.0364	Missense_Mutation	p.G49D	Non-Targeted Panel Gene	Adult HGBCL, NOS
BCL-5	chr1	247966178	247966178	C	A	exonic	OR2AK2	53	2	0.0364	Missense_Mutation	p.P283T	Non-Targeted Panel Gene	Adult HGBCL, NOS
BCL-5	chr16	88866471	88866471	C	T	exonic	PABPN1L	53	2	0.0364	Missense_Mutation	p.E46K	Non-Targeted Panel Gene	Adult HGBCL, NOS
BCL-5	chr22	38073774	38073774	C	T	exonic	PICK1	53	2	0.0364	Missense_Mutation	p.S262L	Non-Targeted Panel Gene	Adult HGBCL, NOS
BCL-5	chr3	39137636	39137636	G	A	exonic	TTC21A	53	2	0.0364	Missense_Mutation	p.A1208T	Non-Targeted Panel Gene	Adult HGBCL, NOS
BCL-5	chr14	66965258	66965258	C	T	exonic	GPHN	79	3	0.0366	Missense_Mutation	p.T299I	Non-Targeted Panel Gene	Adult HGBCL, NOS
BCL-5	chr20	46013309	46013309	C	T	exonic	MMP9	52	2	0.037	Missense_Mutation	p.T462M	Non-Targeted Panel Gene	Adult HGBCL, NOS
BCL-5	chr13	67226468	67226468	G	A	exonic	PCDH9	51	2	0.037	Missense_Mutation	p.T658I	Non-Targeted Panel Gene	Adult HGBCL, NOS
BCL-5	chr2	187497049	187497049	G	A	exonic	TFPI	52	2	0.037	Missense_Mutation	p.H51Y	Non-Targeted Panel Gene	Adult HGBCL, NOS
BCL-5	chr5	140807683	140807683	G	A	exonic	PCDHA4	77	3	0.0375	Missense_Mutation	p.A166T	Non-Targeted Panel Gene	Adult HGBCL, NOS
BCL-5	chr10	100348080	100348080	C	T	exonic	SCD	77	3	0.0375	Missense_Mutation	p.T15I	Non-Targeted Panel Gene	Adult HGBCL, NOS
BCL-5	chr15	50592039	50592039	G	A	exonic	TRPM7	77	3	0.0375	Missense_Mutation	p.T1399I	Non-Targeted Panel Gene	Adult HGBCL, NOS
BCL-5	chr20	47238787	47238787	G	A	exonic	ZMYND8	77	3	0.0375	Missense_Mutation	p.T859M	Non-Targeted Panel Gene	Adult HGBCL, NOS
BCL-5	chr5	65242137	65242137	C	T	exonic	ADAMTS6	51	2	0.0377	Missense_Mutation	p.G634R	Non-Targeted Panel Gene	Adult HGBCL, NOS
BCL-5	chr15	58166759	58166759	G	A	exonic	AQP9	51	2	0.0377	Missense_Mutation	p.M66I	Non-Targeted Panel Gene	Adult HGBCL, NOS
BCL-5	chr1	151532549	151532549	G	A	exonic	CGN	51	2	0.0377	Missense_Mutation	p.G907R	Non-Targeted Panel Gene	Adult HGBCL, NOS
BCL-5	chr1	28232992	28232992	C	T	exonic	DNAJC8	51	2	0.0377	Missense_Mutation	p.A3T	Non-Targeted Panel Gene	Adult HGBCL, NOS
BCL-5	chrX	119936612	119936612	C	T	exonic	NKAP	50	2	0.0377	Missense_Mutation	p.E180K	Non-Targeted Panel Gene	Adult HGBCL, NOS
BCL-5	chr11	72004713	72004713	C	T	exonic	NUMA1	51	2	0.0377	Missense_Mutation	p.G1978D	Non-Targeted Panel Gene	Adult HGBCL, NOS
BCL-5	chr4	106233641	106233641	G	A	exonic	TBCK	51	2	0.0377	Missense_Mutation	p.H315Y	Non-Targeted Panel Gene	Adult HGBCL, NOS
BCL-5	chr14	61280312	61280312	G	A	exonic	TMEM30B	51	2	0.0377	Missense_Mutation	p.P279L	Non-Targeted Panel Gene	Adult HGBCL, NOS
BCL-5	chr1	37864360	37864360	C	T	exonic	INPP5B	76	3	0.038	Missense_Mutation	p.E860K	Non-Targeted Panel Gene	Adult HGBCL, NOS
BCL-5	chr19	15372224	15372224	C	T	exonic	AKAP8	50	2	0.0385	Missense_Mutation	p.E329K	Non-Targeted Panel Gene	Adult HGBCL, NOS
BCL-5	chr5	141534415	141534415	C	T	exonic	DIAPH1	50	2	0.0385	Missense_Mutation	p.G834D	Non-Targeted Panel Gene	Adult HGBCL, NOS
BCL-5	chr19	53015405	53015405	G	A	exonic	ERVV-1	74	3	0.0385	Missense_Mutation	p.A439T	Non-Targeted Panel Gene	Adult HGBCL, NOS
BCL-5	chr13	20690795	20690795	G	A	exonic	IFT88	50	2	0.0385	Missense_Mutation	p.S787N	Non-Targeted Panel Gene	Adult HGBCL, NOS
BCL-5	chr10	30047574	30047574	C	T	exonic	JCAD	75	3	0.0385	Missense_Mutation	p.G80E	Non-Targeted Panel Gene	Adult HGBCL, NOS
BCL-5	chr12	53485083	53485083	C	T	exonic	MAP3K12	50	2	0.0385	Missense_Mutation	p.G338D	Non-Targeted Panel Gene	Adult HGBCL, NOS
BCL-5	chr1	43386005	43386005	C	T	exonic	MED8	50	2	0.0385	Missense_Mutation	p.V239I	Non-Targeted Panel Gene	Adult HGBCL, NOS
BCL-5	chrY	14723261	14723261	G	A	exonic	NLGN4Y	50	2	0.0385	Missense_Mutation	p.G206E	Non-Targeted Panel Gene	Adult HGBCL, NOS
BCL-5	chr6	106655689	106655689	G	A	exonic	QRSL1	50	2	0.0385	Missense_Mutation	p.V373M	Non-Targeted Panel Gene	Adult HGBCL, NOS
BCL-5	chr12	71918538	71918538	G	A	exonic	TBC1D15	75	3	0.0385	Missense_Mutation	p.R547Q	Non-Targeted Panel Gene	Adult HGBCL, NOS
BCL-5	chr7	138460813	138460813	C	T	exonic	TRIM24	50	2	0.0385	Missense_Mutation	p.L89F	Non-Targeted Panel Gene	Adult HGBCL, NOS
BCL-5	chr3	11564880	11564880	G	A	exonic	VGLL4	50	2	0.0385	Missense_Mutation	p.P137S	Non-Targeted Panel Gene	Adult HGBCL, NOS
BCL-5	chr11	46701500	46701500	C	T	exonic	ZNF408	50	2	0.0385	Missense_Mutation	p.L52F	Non-Targeted Panel Gene	Adult HGBCL, NOS
BCL-5	chr15	40810134	40810134	G	A	exonic	ZFYVE19	74	3	0.039	Missense_Mutation	p.G202D	Non-Targeted Panel Gene	Adult HGBCL, NOS
BCL-5	chr7	70790776	70790776	C	T	exonic	AUTS2	98	4	0.0392	Missense_Mutation	p.P1187L	Non-Targeted Panel Gene	Adult HGBCL, NOS
BCL-5	chr2	29071657	29071657	C	T	exonic	C2orf71	49	2	0.0392	Missense_Mutation	p.E869K	Non-Targeted Panel Gene	Adult HGBCL, NOS
BCL-5	chr17	47836900	47836900	G	A	exonic	LRRC46	49	2	0.0392	Missense_Mutation	p.S249N	Non-Targeted Panel Gene	Adult HGBCL, NOS
BCL-5	chr12	8942421	8942421	C	T	exonic	M6PR	49	2	0.0392	Missense_Mutation	p.V236I	Non-Targeted Panel Gene	Adult HGBCL, NOS
BCL-5	chr12	52058894	52058894	C	T	exonic	NR4A1	49	2	0.0392	Missense_Mutation	p.P583S	Non-Targeted Panel Gene	Adult HGBCL, NOS
BCL-5	chr3	191218906	191218906	G	T	exonic	OSTN	49	2	0.0392	Missense_Mutation	p.G88W	Non-Targeted Panel Gene	Adult HGBCL, NOS
BCL-5	chr12	120103841	120103841	C	T	exonic	RAB35	49	2	0.0392	Missense_Mutation	p.R71H	Non-Targeted Panel Gene	Adult HGBCL, NOS
BCL-5	chr2	135058024	135058024	G	A	exonic	RAB3GAP1	49	2	0.0392	Missense_Mutation	p.V30I	Non-Targeted Panel Gene	Adult HGBCL, NOS
BCL-5	chr8	120548762	120548762	C	T	exonic	SNTB1	49	2	0.0392	Missense_Mutation	p.A445T	Non-Targeted Panel Gene	Adult HGBCL, NOS
BCL-5	chr2	233458363	233458363	G	A	exonic	DGKD	73	3	0.0395	Missense_Mutation	p.R887Q	Non-Targeted Panel Gene	Adult HGBCL, NOS
BCL-5	chr14	81204010	81204010	G	A	exonic	GTF2A1	73	3	0.0395	Missense_Mutation	p.P26L	Non-Targeted Panel Gene	Adult HGBCL, NOS
BCL-5	chr1	155137681	155137681	G	A	exonic	SLC50A1	73	3	0.0395	Missense_Mutation	p.V135I	Non-Targeted Panel Gene	Adult HGBCL, NOS
BCL-5	chr14	67793690	67793690	G	A	exonic	ZFYVE26	73	3	0.0395	Missense_Mutation	p.S824L	Non-Targeted Panel Gene	Adult HGBCL, NOS
BCL-5	chr11	26441965	26441965	C	T	exonic	ANO3	48	2	0.04	Missense_Mutation	p.P93S	Non-Targeted Panel Gene	Adult HGBCL, NOS
BCL-5	chr14	64260541	64260541	G	A	exonic	ESR2	48	2	0.04	Missense_Mutation	p.A287V	Non-Targeted Panel Gene	Adult HGBCL, NOS
BCL-5	chr19	3586612	3586612	G	A	exonic	GIPC3	48	2	0.04	Missense_Mutation	p.V115I	Non-Targeted Panel Gene	Adult HGBCL, NOS
BCL-5	chr17	58496292	58496292	G	A	exonic	MTMR4	48	2	0.04	Missense_Mutation	p.A617V	Non-Targeted Panel Gene	Adult HGBCL, NOS
BCL-5	chr5	58974902	58974902	G	A	exonic	PDE4D	48	2	0.04	Missense_Mutation	p.T660I	Non-Targeted Panel Gene	Adult HGBCL, NOS
BCL-5	chr19	49654779	49654779	G	A	exonic	SCAF1	72	3	0.04	Missense_Mutation	p.G1176D	Non-Targeted Panel Gene	Adult HGBCL, NOS
BCL-5	chr12	108623527	108623527	C	T	exonic	SELPLG	72	3	0.04	Missense_Mutation	p.A261T	Non-Targeted Panel Gene	Adult HGBCL, NOS
BCL-5	chr2	97834819	97834819	C	T	exonic	TMEM131	72	3	0.04	Missense_Mutation	p.S304N	Non-Targeted Panel Gene	Adult HGBCL, NOS
BCL-5	chr19	43592087	43592087	G	A	exonic	IRGQ	71	3	0.0405	Missense_Mutation	p.A604V	Non-Targeted Panel Gene	Adult HGBCL, NOS
BCL-5	chr17	63831104	63831104	G	A	exonic	PSMC5	70	3	0.0405	Missense_Mutation	p.E250K	Non-Targeted Panel Gene	Adult HGBCL, NOS
BCL-5	chr16	2767010	2767010	G	A	exonic	SRRM2	71	3	0.0405	Missense_Mutation	p.G2161D	Non-Targeted Panel Gene	Adult HGBCL, NOS
BCL-5	chr19	36915164	36915164	G	A	exonic	ZNF829	71	3	0.0405	Missense_Mutation	p.P83S	Non-Targeted Panel Gene	Adult HGBCL, NOS
BCL-5	chr11	419588	419588	G	A	exonic	ANO9	47	2	0.0408	Missense_Mutation	p.T643I	Non-Targeted Panel Gene	Adult HGBCL, NOS
BCL-5	chr19	1468504	1468504	C	T	exonic	APC2	47	2	0.0408	Missense_Mutation	p.H1735Y	Non-Targeted Panel Gene	Adult HGBCL, NOS
BCL-5	chr16	30930430	30930430	C	T	exonic	FBXL19	47	2	0.0408	Missense_Mutation	p.R91W	Non-Targeted Panel Gene	Adult HGBCL, NOS
BCL-5	chr18	21490208	21490208	C	T	exonic	GREB1L	47	2	0.0408	Missense_Mutation	p.P963S	Non-Targeted Panel Gene	Adult HGBCL, NOS
BCL-5	chr1	9245344	9245344	G	A	exonic	H6PD	47	2	0.0408	Missense_Mutation	p.G137D	Non-Targeted Panel Gene	Adult HGBCL, NOS
BCL-5	chr12	2812851	2812851	G	A	exonic	ITFG2	47	2	0.0408	Missense_Mutation	p.D31N	Non-Targeted Panel Gene	Adult HGBCL, NOS
BCL-5	chr9	34371745	34371745	G	A	exonic	KIAA1161	47	2	0.0408	Missense_Mutation	p.S400L	Non-Targeted Panel Gene	Adult HGBCL, NOS
BCL-5	chr19	54252389	54252389	G	A	exonic	LILRB5	47	2	0.0408	Missense_Mutation	p.A517V	Non-Targeted Panel Gene	Adult HGBCL, NOS
BCL-5	chr4	39877134	39877134	G	A	exonic	PDS5A	47	2	0.0408	Missense_Mutation	p.P671L	Non-Targeted Panel Gene	Adult HGBCL, NOS

BCL-5	chr20	438645	438645	C	T	exonic	TBC1D20	47	2	0.0408	Missense_Mutation	p.A385T	Non-Targeted Panel Gene	Adult HGBCL, NOS
BCL-5	chr2	238329194	238329194	G	A	exonic	TRAF3IP1	47	2	0.0408	Missense_Mutation	p.G256E	Non-Targeted Panel Gene	Adult HGBCL, NOS
BCL-5	chr2	113951742	113951742	C	T	exonic	ACTR3	70	3	0.0411	Missense_Mutation	p.S325L	Non-Targeted Panel Gene	Adult HGBCL, NOS
BCL-5	chr6	31624289	31624289	G	A	exonic	PRRC2A	68	3	0.0411	Missense_Mutation	p.E107K	Non-Targeted Panel Gene	Adult HGBCL, NOS
BCL-5	chr2	70228454	70228454	G	A	exonic	TIA1	93	4	0.0412	Missense_Mutation	p.H109Y	Non-Targeted Panel Gene	Adult HGBCL, NOS
BCL-5	chr11	64220575	64220575	G	A	exonic	FERMT3	46	2	0.0417	Missense_Mutation	p.G488D	Non-Targeted Panel Gene	Adult HGBCL, NOS
BCL-5	chr10	94574713	94574713	G	A	exonic	HELLS	46	2	0.0417	Missense_Mutation	p.E165K	Non-Targeted Panel Gene	Adult HGBCL, NOS
BCL-5	chr6	33695017	33695017	G	A	exonic	ITPR3	46	2	0.0417	Missense_Mutation	p.D2627N	Non-Targeted Panel Gene	Adult HGBCL, NOS
BCL-5	chrX	41170030	41170030	G	A	exonic	USP9X	92	4	0.0417	Missense_Mutation	p.R891Q	Non-Targeted Panel Gene	Adult HGBCL, NOS
BCL-5	chr17	34356602	34356602	G	A	exonic	CCL13	68	3	0.0423	Missense_Mutation	p.D26N	Non-Targeted Panel Gene	Adult HGBCL, NOS
BCL-5	chr1	27993543	27993543	G	A	exonic	EYA3	68	3	0.0423	Missense_Mutation	p.T387I	Non-Targeted Panel Gene	Adult HGBCL, NOS
BCL-5	chr14	39122499	39122499	G	A	exonic	GEMIN2	68	3	0.0423	Missense_Mutation	p.V159I	Non-Targeted Panel Gene	Adult HGBCL, NOS
BCL-5	chr2	25749799	25749799	C	T	exonic	ASXL2	45	2	0.0426	Missense_Mutation	p.R586H	Non-Targeted Panel Gene	Adult HGBCL, NOS
BCL-5	chr9	894083	894083	C	T	exonic	DMRT1	45	2	0.0426	Missense_Mutation	p.A237V	Non-Targeted Panel Gene	Adult HGBCL, NOS
BCL-5	chr17	44397576	44397576	C	T	exonic	GPATCH8	45	2	0.0426	Missense_Mutation	p.G1423S	Non-Targeted Panel Gene	Adult HGBCL, NOS
BCL-5	chr19	42405547	42405547	C	T	exonic	LIPE	45	2	0.0426	Missense_Mutation	p.D794N	Non-Targeted Panel Gene	Adult HGBCL, NOS
BCL-5	chr10	73479515	73479515	C	T	exonic	PPP3CB	45	2	0.0426	Missense_Mutation	p.V30I	Non-Targeted Panel Gene	Adult HGBCL, NOS
BCL-5	chr8	17562102	17562102	G	A	exonic	SLC7A2	45	2	0.0426	Missense_Mutation	p.A594T	Non-Targeted Panel Gene	Adult HGBCL, NOS
BCL-5	chr11	59658904	59658904	C	T	exonic	PATL1	67	3	0.0429	Missense_Mutation	p.E130K	Non-Targeted Panel Gene	Adult HGBCL, NOS
BCL-5	chr3	72975109	72975109	G	A	exonic	GXYLT2	111	5	0.0431	Missense_Mutation	p.A428T	Non-Targeted Panel Gene	Adult HGBCL, NOS
BCL-5	chr20	35479719	35479719	G	A	exonic	CEP250	44	2	0.0435	Missense_Mutation	p.G788R	Non-Targeted Panel Gene	Adult HGBCL, NOS
BCL-5	chr6	118565884	118565884	C	T	exonic	CEP85L	66	3	0.0435	Missense_Mutation	p.R225Q	Non-Targeted Panel Gene	Adult HGBCL, NOS
BCL-5	chr6	33164896	33164896	C	T	exonic	COL11A2	66	3	0.0435	Missense_Mutation	p.A1607T	Non-Targeted Panel Gene	Adult HGBCL, NOS
BCL-5	chr12	120353355	120353355	G	A	exonic	MSI1	44	2	0.0435	Missense_Mutation	p.T226I	Non-Targeted Panel Gene	Adult HGBCL, NOS
BCL-5	chr7	39207449	39207449	C	T	exonic	POU6F2	44	2	0.0435	Missense_Mutation	p.P114S	Non-Targeted Panel Gene	Adult HGBCL, NOS
BCL-5	chr1	44827848	44827848	C	T	exonic	PTCH2	44	2	0.0435	Missense_Mutation	p.A685T	Non-Targeted Panel Gene	Adult HGBCL, NOS
BCL-5	chr1	156260552	156260552	G	A	exonic	SMG5	44	2	0.0435	Missense_Mutation	p.L662F	Non-Targeted Panel Gene	Adult HGBCL, NOS
BCL-5	chr15	37936355	37936355	G	A	exonic	TMCO5A	44	2	0.0435	Missense_Mutation	p.R11K	Non-Targeted Panel Gene	Adult HGBCL, NOS
BCL-5	chr11	123726260	123726260	C	T	exonic	ZNF202	44	2	0.0435	Missense_Mutation	p.E562K	Non-Targeted Panel Gene	Adult HGBCL, NOS
BCL-5	chr12	111457291	111457291	G	A	exonic	ATXN2	65	3	0.0441	Missense_Mutation	p.P1147S	Non-Targeted Panel Gene	Adult HGBCL, NOS
BCL-5	chr11	111911559	111911559	G	A	exonic	CRYAB	65	3	0.0441	Missense_Mutation	p.R56W	Non-Targeted Panel Gene	Adult HGBCL, NOS
BCL-5	chr2	160137619	160137619	C	T	exonic	ITGB6	65	3	0.0441	Missense_Mutation	p.C492Y	Non-Targeted Panel Gene	Adult HGBCL, NOS
BCL-5	chr14	21076865	21076865	C	T	exonic	ARHGEF40	43	2	0.0444	Missense_Mutation	p.P670L	Non-Targeted Panel Gene	Adult HGBCL, NOS
BCL-5	chr2	61869466	61869466	G	A	exonic	CCT4	86	4	0.0444	Missense_Mutation	p.R527W	Non-Targeted Panel Gene	Adult HGBCL, NOS
BCL-5	chr11	4803907	4803907	C	A	exonic	OR52R1	43	2	0.0444	Missense_Mutation	p.W158C	Non-Targeted Panel Gene	Adult HGBCL, NOS
BCL-5	chr11	9789185	9789185	C	T	exonic	SBF2	43	2	0.0444	Missense_Mutation	p.S1619N	Non-Targeted Panel Gene	Adult HGBCL, NOS
BCL-5	chr19	44236445	44236445	G	A	exonic	ZNF227	43	2	0.0444	Missense_Mutation	p.S672N	Non-Targeted Panel Gene	Adult HGBCL, NOS
BCL-5	chr11	118628133	118628133	C	T	exonic	PHLDB1	64	3	0.0448	Missense_Mutation	p.T437I	Non-Targeted Panel Gene	Adult HGBCL, NOS
BCL-5	chrX	5893050	5893050	C	T	exonic	NLGN4X	85	4	0.0449	Missense_Mutation	p.D740N	Non-Targeted Panel Gene	Adult HGBCL, NOS
BCL-5	chr12	50059032	50059032	C	T	exonic	ASIC1	63	3	0.0455	Missense_Mutation	p.A89V	Non-Targeted Panel Gene	Adult HGBCL, NOS
BCL-5	chr8	144464582	144464582	G	A	exonic	CYHR1	42	2	0.0455	Missense_Mutation	p.L42F	Non-Targeted Panel Gene	Adult HGBCL, NOS
BCL-5	chr17	17977641	17977641	G	A	exonic	DRC3	42	2	0.0455	Missense_Mutation	p.D15N	Non-Targeted Panel Gene	Adult HGBCL, NOS
BCL-5	chrX	149498150	149498150	G	A	exonic	IDS	42	2	0.0455	Missense_Mutation	p.A222V	Non-Targeted Panel Gene	Adult HGBCL, NOS
BCL-5	chr16	4258100	4258100	C	T	exonic	TFAP4	42	2	0.0455	Missense_Mutation	p.M324I	Non-Targeted Panel Gene	Adult HGBCL, NOS
BCL-5	chr19	51965704	51965704	G	A	exonic	ZNF350	42	2	0.0455	Missense_Mutation	p.T250I	Non-Targeted Panel Gene	Adult HGBCL, NOS
BCL-5	chr5	10258479	10258479	G	A	exonic	CCT5	62	3	0.0462	Missense_Mutation	p.D273N	Non-Targeted Panel Gene	Adult HGBCL, NOS
BCL-5	chr15	41749134	41749134	C	T	exonic	MGA	62	3	0.0462	Missense_Mutation	p.P1843S	Non-Targeted Panel Gene	Adult HGBCL, NOS
BCL-5	chr2	202883619	202883619	C	T	exonic	WDR12	62	3	0.0462	Missense_Mutation	p.D371N	Non-Targeted Panel Gene	Adult HGBCL, NOS
BCL-5	chr4	88121668	88121668	C	T	exonic	ABCG2	41	2	0.0465	Missense_Mutation	p.S219N	Non-Targeted Panel Gene	Adult HGBCL, NOS
BCL-5	chr16	28826986	28826986	G	A	exonic	ATXN2L	82	4	0.0465	Missense_Mutation	p.M247I	Non-Targeted Panel Gene	Adult HGBCL, NOS
BCL-5	chr19	6697378	6697378	A	G	exonic	C3	41	2	0.0465	Missense_Mutation	p.I921T	Non-Targeted Panel Gene	Adult HGBCL, NOS
BCL-5	chr11	82732959	82732959	C	A	exonic	FAM181B	41	2	0.0465	Missense_Mutation	p.K257N	Non-Targeted Panel Gene	Adult HGBCL, NOS
BCL-5	chr18	13642729	13642729	G	A	exonic	LDLRAD4	41	2	0.0465	Missense_Mutation	p.S5N	Non-Targeted Panel Gene	Adult HGBCL, NOS
BCL-5	chr11	281091	281091	G	A	exonic	NLRP6	82	4	0.0465	Missense_Mutation	p.V453I	Non-Targeted Panel Gene	Adult HGBCL, NOS
BCL-5	chr8	23290197	23290197	G	A	exonic	R3HCC1	41	2	0.0465	Missense_Mutation	p.D152N	Non-Targeted Panel Gene	Adult HGBCL, NOS
BCL-5	chr2	171813386	171813386	C	T	exonic	SLC25A12	82	4	0.0465	Missense_Mutation	p.C375Y	Non-Targeted Panel Gene	Adult HGBCL, NOS
BCL-5	chr1	31791244	31791244	C	T	exonic	SPOCD1	41	2	0.0465	Missense_Mutation	p.V1004I	Non-Targeted Panel Gene	Adult HGBCL, NOS
BCL-5	chr11	46704652	46704652	C	T	exonic	ZNF408	41	2	0.0465	Missense_Mutation	p.P318S	Non-Targeted Panel Gene	Adult HGBCL, NOS
BCL-5	chr22	49787851	49787851	G	A	exonic	BRD1	61	3	0.0469	Missense_Mutation	p.A285V	Non-Targeted Panel Gene	Adult HGBCL, NOS
BCL-5	chr7	102457409	102457409	C	A	exonic	ALKBH4	40	2	0.0476	Missense_Mutation	p.Q298H	Non-Targeted Panel Gene	Adult HGBCL, NOS
BCL-5	chr9	97307998	97307998	C	T	exonic	CCDC180	40	2	0.0476	Missense_Mutation	p.H23Y	Non-Targeted Panel Gene	Adult HGBCL, NOS
BCL-5	chr15	40472177	40472177	C	T	exonic	CHST14	40	2	0.0476	Missense_Mutation	p.P322S	Non-Targeted Panel Gene	Adult HGBCL, NOS
BCL-5	chr3	33584878	33584878	G	A	exonic	CLASP2	80	4	0.0476	Missense_Mutation	p.T705I	Non-Targeted Panel Gene	Adult HGBCL, NOS
BCL-5	chr14	94062354	94062354	G	A	exonic	DDX24	80	4	0.0476	Missense_Mutation	p.A329V	Non-Targeted Panel Gene	Adult HGBCL, NOS
BCL-5	chr5	176496516	176496516	C	T	exonic	FAF2	40	2	0.0476	Missense_Mutation	p.P231L	Non-Targeted Panel Gene	Adult HGBCL, NOS
BCL-5	chr17	7848671	7848671	C	T	exonic	KDM6B	40	2	0.0476	Missense_Mutation	p.P795S	Non-Targeted Panel Gene	Adult HGBCL, NOS
BCL-5	chr17	75569994	75569994	C	T	exonic	LLGL2	39	2	0.0476	Missense_Mutation	p.A538V	Non-Targeted Panel Gene	Adult HGBCL, NOS
BCL-5	chr21	36369415	36369415	G	A	exonic	MORC3	40	2	0.0476	Missense_Mutation	p.E612K	Non-Targeted Panel Gene	Adult HGBCL, NOS
BCL-5	chr19	38418488	38418488	C	T	exonic	RASGRP4	40	2	0.0476	Missense_Mutation	p.S247N	Non-Targeted Panel Gene	Adult HGBCL, NOS
BCL-5	chr14	39095914	39095914	C	T	exonic	SEC23A	40	2	0.0476	Missense_Mutation	p.V69I	Non-Targeted Panel Gene	Adult HGBCL, NOS
BCL-5	chr1	40393495	40393495	G	A	exonic	SMAP2	60	3	0.0476	Missense_Mutation	p.V27M	Non-Targeted Panel Gene	Adult HGBCL, NOS
BCL-5	chr19	51969022	51969022	C	T	exonic	ZNF350	60	3	0.0476	Missense_Mutation	p.S42N	Non-Targeted Panel Gene	Adult HGBCL, NOS
BCL-5	chr16	77193522	77193522	C	T	exonic	MON1B	59	3	0.0484	Missense_Mutation	p.R74W	Non-Targeted Panel Gene	Adult HGBCL, NOS
BCL-5	chr9	129081177	129081177	G	A	exonic	DOLPP1	39	2	0.0488	Missense_Mutation	p.V16M	Non-Targeted Panel Gene	Adult HGBCL, NOS
BCL-5	chr3	140688336	140688336	G	A	exonic	TRIM42	39	2	0.0488	Missense_Mutation	p.G552S	Non-Targeted Panel Gene	Adult HGBCL, NOS
BCL-5	chr17	18016176	18016176	G	A	exonic	DRC3	58	3	0.0492	Missense_Mutation	p.C480Y	Non-Targeted Panel Gene	Adult HGBCL, NOS
BCL-5	chr22	50265438	50265438	C	T	exonic	MAPK11	58	3	0.0492	Missense_Mutation	p.A300T	Non-Targeted Panel Gene	Adult HGBCL, NOS
BCL-5	chr1	202895708	202895708	G	A	exonic	KLHL12	77	4	0.0494	Missense_Mutation	p.R317C	Non-Targeted Panel Gene	Adult HGBCL, NOS
BCL-5	chr5	55324188	55324188	G	A	exonic	SKIV2L2	77	4	0.0494	Missense_Mutation	p.C110Y	Non-Targeted Panel Gene	Adult HGBCL, NOS
BCL-5	chr10	133128765	133128765	G	A	exonic	ADGRA1	57	3	0.05	Missense_Mutation	p.V313M	Non-Targeted Panel Gene	Adult HGBCL, NOS
BCL-5	chr1	44813485	44813485	G	A	exonic	BTBD19	56	3	0.05	Missense_Mutation	p.E243K	Non-Targeted Panel Gene	Adult HGBCL, NOS
BCL-5	chr1	3755546	3755546	G	A	exonic	CCDC27	57	3	0.05	Missense_Mutation	p.G178S	Non-Targeted Panel Gene	Adult HGBCL, NOS

BCL-5	chr20	46251171	46251171	G	A	exonic	CDH22	38	2	0.05	Missense_Mutation	p.P42S	Non-Targeted Panel Gene	Adult HGBCL, NOS
BCL-5	chr3	138024622	138024622	C	T	exonic	CLDN18	76	4	0.05	Missense_Mutation	p.A134V	Non-Targeted Panel Gene	Adult HGBCL, NOS
BCL-5	chr19	35737893	35737893	C	T	exonic	KMT2B	38	2	0.05	Missense_Mutation	p.R2565C	Non-Targeted Panel Gene	Adult HGBCL, NOS
BCL-5	chr8	144514997	144514997	C	T	exonic	RECQL4	38	2	0.05	Missense_Mutation	p.S520N	Non-Targeted Panel Gene	Adult HGBCL, NOS
BCL-5	chr14	81371077	81371077	C	T	exonic	STON2	38	2	0.05	Missense_Mutation	p.S161N	Non-Targeted Panel Gene	Adult HGBCL, NOS
BCL-5	chr2	70988680	70988680	G	A	exonic	TEX261	76	4	0.05	Missense_Mutation	p.R171W	Non-Targeted Panel Gene	Adult HGBCL, NOS
BCL-5	chr12	56430963	56430963	G	A	exonic	TIMELESS	57	3	0.05	Missense_Mutation	p.S276F	Non-Targeted Panel Gene	Adult HGBCL, NOS
BCL-5	chr19	56664817	56664817	G	A	exonic	ZNF835	57	3	0.05	Missense_Mutation	p.H128Y	Non-Targeted Panel Gene	Adult HGBCL, NOS
BCL-5	chr19	49832396	49832396	G	A	exonic	MED25	75	4	0.0506	Missense_Mutation	p.R488H	Non-Targeted Panel Gene	Adult HGBCL, NOS
BCL-5	chr2	96115962	96115962	G	A	exonic	ADRA2B	56	3	0.0508	Missense_Mutation	p.T63M	Non-Targeted Panel Gene	Adult HGBCL, NOS
BCL-5	chr13	111233232	111233232	C	T	exonic	ARHGEF7	56	3	0.0508	Missense_Mutation	p.T254I	Non-Targeted Panel Gene	Adult HGBCL, NOS
BCL-5	chr1	58576792	58576792	G	A	exonic	TACSTD2	56	3	0.0508	Missense_Mutation	p.T122M	Non-Targeted Panel Gene	Adult HGBCL, NOS
BCL-5	chr14	102497036	102497036	G	A	exonic	TECPR2	56	3	0.0508	Missense_Mutation	p.V1283M	Non-Targeted Panel Gene	Adult HGBCL, NOS
BCL-5	chr13	114018165	114018165	G	A	exonic	RASA3	37	2	0.0513	Missense_Mutation	p.H344Y	Non-Targeted Panel Gene	Adult HGBCL, NOS
BCL-5	chr16	23069326	23069326	C	T	exonic	USP31	74	4	0.0513	Missense_Mutation	p.D927N	Non-Targeted Panel Gene	Adult HGBCL, NOS
BCL-5	chr6	30649690	30649690	C	T	exonic	C6orf136	55	3	0.0517	Missense_Mutation	p.P250S	Non-Targeted Panel Gene	Adult HGBCL, NOS
BCL-5	chr6	38982353	38982353	G	A	exonic	DNAH8	55	3	0.0517	Missense_Mutation	p.R4281Q	Non-Targeted Panel Gene	Adult HGBCL, NOS
BCL-5	chr19	12792121	12792121	G	A	exonic	JUNB	55	3	0.0517	Missense_Mutation	p.S117N	Non-Targeted Panel Gene	Adult HGBCL, NOS
BCL-5	chr22	50289030	50289030	G	A	exonic	PLXNB2	55	3	0.0517	Missense_Mutation	p.A394V	Non-Targeted Panel Gene	Adult HGBCL, NOS
BCL-5	chr19	3978085	3978085	G	A	exonic	EEF2	73	4	0.0519	Missense_Mutation	p.R601W	Non-Targeted Panel Gene	Adult HGBCL, NOS
BCL-5	chrX	101616482	101616482	C	T	exonic	ARMCX6	54	3	0.0526	Missense_Mutation	p.D47N	Non-Targeted Panel Gene	Adult HGBCL, NOS
BCL-5	chr2	174757630	174757630	C	T	exonic	CHRNA1	54	3	0.0526	Missense_Mutation	p.G119S	Non-Targeted Panel Gene	Adult HGBCL, NOS
BCL-5	chr19	35543467	35543467	C	G	exonic	GAPDHS	36	2	0.0526	Missense_Mutation	p.T290S	Non-Targeted Panel Gene	Adult HGBCL, NOS
BCL-5	chr6	149632823	149632823	C	T	exonic	KATNA1	36	2	0.0526	Missense_Mutation	p.A86T	Non-Targeted Panel Gene	Adult HGBCL, NOS
BCL-5	chr7	20158404	20158404	C	T	exonic	MACC1	72	4	0.0526	Missense_Mutation	p.V653I	Non-Targeted Panel Gene	Adult HGBCL, NOS
BCL-5	chr7	101172300	101172300	G	A	exonic	NAT16	36	2	0.0526	Missense_Mutation	p.L297F	Non-Targeted Panel Gene	Adult HGBCL, NOS
BCL-5	chr16	50633510	50633510	C	T	exonic	NKD1	36	2	0.0526	Missense_Mutation	p.S381F	Non-Targeted Panel Gene	Adult HGBCL, NOS
BCL-5	chr3	136090645	136090645	G	A	exonic	PPP2R3A	54	3	0.0526	Missense_Mutation	p.E969K	Non-Targeted Panel Gene	Adult HGBCL, NOS
BCL-5	chr3	38847035	38847035	G	A	exonic	SCN11A	36	2	0.0526	Missense_Mutation	p.R1679C	Non-Targeted Panel Gene	Adult HGBCL, NOS
BCL-5	chr6	2833906	2833906	C	T	exonic	SERPINB1	72	4	0.0526	Missense_Mutation	p.S281N	Non-Targeted Panel Gene	Adult HGBCL, NOS
BCL-5	chr16	12902567	12902567	G	A	exonic	SHISA9	36	2	0.0526	Missense_Mutation	p.G168D	Non-Targeted Panel Gene	Adult HGBCL, NOS
BCL-5	chr8	117161754	117161754	C	T	exonic	SLC30A8	54	3	0.0526	Missense_Mutation	p.H148Y	Non-Targeted Panel Gene	Adult HGBCL, NOS
BCL-5	chr15	64674245	64674245	G	A	exonic	ZNF609	54	3	0.0526	Missense_Mutation	p.R464H	Non-Targeted Panel Gene	Adult HGBCL, NOS
BCL-5	chr11	129931143	129931143	G	A	exonic	PRDM10	71	4	0.0533	Missense_Mutation	p.T468I	Non-Targeted Panel Gene	Adult HGBCL, NOS
BCL-5	chr5	177269640	177269640	C	T	exonic	NSD1	53	3	0.0536	Missense_Mutation	p.P1512L	Non-Targeted Panel Gene	Adult HGBCL, NOS
BCL-5	chr5	140877213	140877213	G	A	exonic	PCDHA12	53	3	0.0536	Missense_Mutation	p.V581I	Non-Targeted Panel Gene	Adult HGBCL, NOS
BCL-5	chr22	41237493	41237493	G	A	exonic	CHADL	35	2	0.0541	Missense_Mutation	p.H527Y	Non-Targeted Panel Gene	Adult HGBCL, NOS
BCL-5	chr3	49911907	49911907	T	C	exonic	MON1A	35	2	0.0541	Missense_Mutation	p.R175G	Non-Targeted Panel Gene	Adult HGBCL, NOS
BCL-5	chr17	32778519	32778519	G	A	exonic	MYO1D	70	4	0.0541	Missense_Mutation	p.A120V	Non-Targeted Panel Gene	Adult HGBCL, NOS
BCL-5	chr4	185506648	185506648	G	A	exonic	PDLIM3	35	2	0.0541	Missense_Mutation	p.P223S	Non-Targeted Panel Gene	Adult HGBCL, NOS
BCL-5	chr16	1977398	1977398	G	A	exonic	TBL3	35	2	0.0541	Missense_Mutation	p.V572M	Non-Targeted Panel Gene	Adult HGBCL, NOS
BCL-5	chr12	50734196	50734196	G	A	exonic	DIP2B	87	5	0.0543	Missense_Mutation	p.R1348K	Non-Targeted Panel Gene	Adult HGBCL, NOS
BCL-5	chr9	113389449	113389449	C	T	exonic	ALAD	52	3	0.0545	Missense_Mutation	p.V293I	Non-Targeted Panel Gene	Adult HGBCL, NOS
BCL-5	chr2	73813507	73813507	C	T	exonic	C2orf78	52	3	0.0545	Missense_Mutation	p.T43I	Non-Targeted Panel Gene	Adult HGBCL, NOS
BCL-5	chr17	41724898	41724898	C	T	exonic	HAP1	69	4	0.0548	Missense_Mutation	p.V555M	Non-Targeted Panel Gene	Adult HGBCL, NOS
BCL-5	chr14	93540686	93540686	C	T	exonic	UNC79	69	4	0.0548	Missense_Mutation	p.A283V	Non-Targeted Panel Gene	Adult HGBCL, NOS
BCL-5	chr10	7738687	7738687	C	T	exonic	ITIH2	86	5	0.0549	Missense_Mutation	p.T675M	Non-Targeted Panel Gene	Adult HGBCL, NOS
BCL-5	chr7	98281910	98281910	C	T	exonic	BRI3	34	2	0.0556	Missense_Mutation	p.P39S	Non-Targeted Panel Gene	Adult HGBCL, NOS
BCL-5	chr2	84557862	84557862	G	A	exonic	DNAH6	68	4	0.0556	Missense_Mutation	p.C577Y	Non-Targeted Panel Gene	Adult HGBCL, NOS
BCL-5	chr17	50380793	50380793	C	T	exonic	EME1	51	3	0.0556	Missense_Mutation	p.R523C	Non-Targeted Panel Gene	Adult HGBCL, NOS
BCL-5	chr21	38299973	38299973	C	T	exonic	KCNJ15	34	2	0.0556	Missense_Mutation	p.H238Y	Non-Targeted Panel Gene	Adult HGBCL, NOS
BCL-5	chr4	25012475	25012475	G	A	exonic	LGI2	34	2	0.0556	Missense_Mutation	p.P227L	Non-Targeted Panel Gene	Adult HGBCL, NOS
BCL-5	chrX	19380212	19380212	C	T	exonic	MAP3K15	34	2	0.0556	Missense_Mutation	p.D833N	Non-Targeted Panel Gene	Adult HGBCL, NOS
BCL-5	chr11	1016313	1016313	G	A	exonic	MUC6	34	2	0.0556	Missense_Mutation	p.S2163F	Non-Targeted Panel Gene	Adult HGBCL, NOS
BCL-5	chr1	35560851	35560851	G	A	exonic	NCDN	34	2	0.0556	Missense_Mutation	p.D234N	Non-Targeted Panel Gene	Adult HGBCL, NOS
BCL-5	chr5	177455471	177455471	G	A	exonic	PRR7	34	2	0.0556	Missense_Mutation	p.R135Q	Non-Targeted Panel Gene	Adult HGBCL, NOS
BCL-5	chr1	1286158	1286158	G	A	exonic	SCNN1D	34	2	0.0556	Missense_Mutation	p.C264Y	Non-Targeted Panel Gene	Adult HGBCL, NOS
BCL-5	chr8	63187594	63187594	C	T	exonic	YTHDF3	34	2	0.0556	Missense_Mutation	p.P477L	Non-Targeted Panel Gene	Adult HGBCL, NOS
BCL-5	chr11	60875582	60875582	G	A	exonic	ZP1	51	3	0.0556	Missense_Mutation	p.V615I	Non-Targeted Panel Gene	Adult HGBCL, NOS
BCL-5	chr1	225828869	225828869	G	A	exonic	EPHX1	67	4	0.0563	Missense_Mutation	p.S47N	Non-Targeted Panel Gene	Adult HGBCL, NOS
BCL-5	chr1	241857475	241857475	G	A	exonic	EXO1	67	4	0.0563	Missense_Mutation	p.C179Y	Non-Targeted Panel Gene	Adult HGBCL, NOS
BCL-5	chr12	6839082	6839082	G	A	exonic	P3H3	67	4	0.0563	Missense_Mutation	p.R663Q	Non-Targeted Panel Gene	Adult HGBCL, NOS
BCL-5	chr16	67909684	67909684	G	A	exonic	PSKH1	50	3	0.0566	Missense_Mutation	p.G312D	Non-Targeted Panel Gene	Adult HGBCL, NOS
BCL-5	chr1	234214574	234214574	G	A	exonic	SLC35F3	50	3	0.0566	Missense_Mutation	p.G26R	Non-Targeted Panel Gene	Adult HGBCL, NOS
BCL-5	chr2	85326843	85326843	C	T	exonic	TGOLN2	50	3	0.0566	Missense_Mutation	p.E297K	Non-Targeted Panel Gene	Adult HGBCL, NOS
BCL-5	chr3	130379777	130379777	G	A	exonic	COL6A5	33	2	0.0571	Missense_Mutation	p.V343I	Non-Targeted Panel Gene	Adult HGBCL, NOS
BCL-5	chr5	38881701	38881701	G	A	exonic	OSMR	33	2	0.0571	Missense_Mutation	p.D119N	Non-Targeted Panel Gene	Adult HGBCL, NOS
BCL-5	chr19	40394262	40394262	C	T	exonic	PRX	32	2	0.0571	Missense_Mutation	p.G1364R	Non-Targeted Panel Gene	Adult HGBCL, NOS
BCL-5	chr1	19160985	19160985	C	T	exonic	UBR4	33	2	0.0571	Missense_Mutation	p.E1780K	Non-Targeted Panel Gene	Adult HGBCL, NOS
BCL-5	chr8	99103081	99103081	G	A	exonic	VPS13B	66	4	0.0571	Missense_Mutation	p.V181I	Non-Targeted Panel Gene	Adult HGBCL, NOS
BCL-5	chr19	11192837	11192837	C	T	exonic	KANK2	49	3	0.0577	Missense_Mutation	p.A415T	Non-Targeted Panel Gene	Adult HGBCL, NOS
BCL-5	chr3	8994428	8994428	G	A	exonic	SRGAP3	49	3	0.0577	Missense_Mutation	p.H775Y	Non-Targeted Panel Gene	Adult HGBCL, NOS
BCL-5	chr8	28160385	28160385	G	A	exonic	ELP3	65	4	0.058	Missense_Mutation	p.V472I	Non-Targeted Panel Gene	Adult HGBCL, NOS
BCL-5	chr5	132638101	132638101	C	T	exonic	RAD50	65	4	0.058	Missense_Mutation	p.R1166W	Non-Targeted Panel Gene	Adult HGBCL, NOS
BCL-5	chr14	24132711	24132711	G	A	exonic	FITM1	48	3	0.0588	Missense_Mutation	p.G256D	Non-Targeted Panel Gene	Adult HGBCL, NOS
BCL-5	chr19	13765099	13765099	G	A	exonic	MRI1	32	2	0.0588	Missense_Mutation	p.V121I	Non-Targeted Panel Gene	Adult HGBCL, NOS
BCL-5	chr19	9251137	9251137	C	T	exonic	OR7E24	31	2	0.0588	Missense_Mutation	p.L32F	Non-Targeted Panel Gene	Adult HGBCL, NOS
BCL-5	chr16	81171026	81171026	C	T	exonic	PKD1L2	32	2	0.0588	Missense_Mutation	p.E943K	Non-Targeted Panel Gene	Adult HGBCL, NOS
BCL-5	chr7	5064375	5064375	C	T	exonic	RBAK	47	3	0.06	Missense_Mutation	p.H307Y	Non-Targeted Panel Gene	Adult HGBCL, NOS
BCL-5	chr17	3588331	3588331	G	A	exonic	TRPV1	47	3	0.06	Missense_Mutation	p.P361S	Non-Targeted Panel Gene	Adult HGBCL, NOS
BCL-5	chrX	86714607	86714607	G	A	exonic	DACH2	31	2	0.0606	Missense_Mutation	p.A331T	Non-Targeted Panel Gene	Adult HGBCL, NOS
BCL-5	chr1	228425204	228425204	C	T	exonic	HIST3H3	30	2	0.0606	Missense_Mutation	p.R41H	Non-Targeted Panel Gene	Adult HGBCL, NOS

BCL-5	chr22	37128290	37128290	G	A	exonic	IL2RB	31	2	0.0606	Missense_Mutation	p.P488S	Non-Targeted Panel Gene	Adult HGBCL, NOS
BCL-5	chr14	103685004	103685004	G	A	exonic	KLC1	31	2	0.0606	Missense_Mutation	p.G557R	Non-Targeted Panel Gene	Adult HGBCL, NOS
BCL-5	chr11	94545316	94545316	G	A	exonic	FUT4	76	5	0.061	Missense_Mutation	p.V395M	Non-Targeted Panel Gene	Adult HGBCL, NOS
BCL-5	chrX	101239038	101239038	G	A	exonic	DRP2	46	3	0.0612	Missense_Mutation	p.A166T	Non-Targeted Panel Gene	Adult HGBCL, NOS
BCL-5	chr21	30289336	30289336	C	T	exonic	KRTAP25-1	46	3	0.0612	Missense_Mutation	p.G52D	Non-Targeted Panel Gene	Adult HGBCL, NOS
BCL-5	chr3	146528634	146528634	C	T	exonic	PLSCR1	46	3	0.0612	Missense_Mutation	p.G98R	Non-Targeted Panel Gene	Adult HGBCL, NOS
BCL-5	chr1	112712887	112712887	G	A	exonic	PPM1J	46	3	0.0612	Missense_Mutation	p.L196F	Non-Targeted Panel Gene	Adult HGBCL, NOS
BCL-5	chr5	149027942	149027942	G	A	exonic	SH3TC2	46	3	0.0612	Missense_Mutation	p.A597V	Non-Targeted Panel Gene	Adult HGBCL, NOS
BCL-5	chr1	37540431	37540431	C	T	exonic	SNIP1	46	3	0.0612	Missense_Mutation	p.V218M	Non-Targeted Panel Gene	Adult HGBCL, NOS
BCL-5	chr15	88847329	88847329	G	A	exonic	ACAN	61	4	0.0615	Missense_Mutation	p.V506I	Non-Targeted Panel Gene	Adult HGBCL, NOS
BCL-5	chr20	63556243	63556243	G	A	exonic	FNDC11	30	2	0.0625	Missense_Mutation	p.A194T	Non-Targeted Panel Gene	Adult HGBCL, NOS
BCL-5	chr7	42025296	42025296	C	T	exonic	GLI3	45	3	0.0625	Missense_Mutation	p.D442N	Non-Targeted Panel Gene	Adult HGBCL, NOS
BCL-5	chr17	18118994	18118994	C	T	exonic	MYO15A	30	2	0.0625	Missense_Mutation	p.T65I	Non-Targeted Panel Gene	Adult HGBCL, NOS
BCL-5	chr12	57591327	57591327	C	T	exonic	PIP4K2C	45	3	0.0625	Missense_Mutation	p.A13V	Non-Targeted Panel Gene	Adult HGBCL, NOS
BCL-5	chr14	92652361	92652361	G	A	exonic	RIN3	30	2	0.0625	Missense_Mutation	p.V438M	Non-Targeted Panel Gene	Adult HGBCL, NOS
BCL-5	chr20	326417	326417	G	A	exonic	SOX12	30	2	0.0625	Missense_Mutation	p.D165N	Non-Targeted Panel Gene	Adult HGBCL, NOS
BCL-5	chr17	28695493	28695493	G	A	exonic	SUPT6H	60	4	0.0625	Missense_Mutation	p.E1306K	Non-Targeted Panel Gene	Adult HGBCL, NOS
BCL-5	chr19	55578075	55578075	G	A	exonic	ZNF579	30	2	0.0625	Missense_Mutation	p.P522L	Non-Targeted Panel Gene	Adult HGBCL, NOS
BCL-5	chr3	12004624	12004624	G	A	exonic	SYN2	74	5	0.0633	Missense_Mutation	p.D25N	Non-Targeted Panel Gene	Adult HGBCL, NOS
BCL-5	chr16	69321235	69321235	G	A	exonic	VPS4A	59	4	0.0635	Missense_Mutation	p.V346M	Non-Targeted Panel Gene	Adult HGBCL, NOS
BCL-5	chr8	106769779	106769779	G	A	exonic	ABRA	44	3	0.0638	Missense_Mutation	p.L138F	Non-Targeted Panel Gene	Adult HGBCL, NOS
BCL-5	chr3	50337178	50337178	C	T	exonic	RASSF1	44	3	0.0638	Missense_Mutation	p.D48N	Non-Targeted Panel Gene	Adult HGBCL, NOS
BCL-5	chr8	22619665	22619665	C	A	exonic	CCAR2	28	2	0.0645	Missense_Mutation	p.P919T	Non-Targeted Panel Gene	Adult HGBCL, NOS
BCL-5	chr3	48646229	48646229	G	T	exonic	CELSR3	28	2	0.0645	Missense_Mutation	p.P2442T	Non-Targeted Panel Gene	Adult HGBCL, NOS
BCL-5	chr15	57461867	57461867	G	T	exonic	CGNL1	29	2	0.0645	Missense_Mutation	p.R793M	Non-Targeted Panel Gene	Adult HGBCL, NOS
BCL-5	chr18	79899362	79899362	G	A	exonic	KCNG2	29	2	0.0645	Missense_Mutation	p.R316H	Non-Targeted Panel Gene	Adult HGBCL, NOS
BCL-5	chr3	123692822	123692822	C	T	exonic	MYLK	29	2	0.0645	Missense_Mutation	p.A1160T	Non-Targeted Panel Gene	Adult HGBCL, NOS
BCL-5	chr19	5231361	5231361	C	T	exonic	PTPRS	29	2	0.0645	Missense_Mutation	p.V702M	Non-Targeted Panel Gene	Adult HGBCL, NOS
BCL-5	chr1	86735179	86735179	G	A	exonic	SH3GLB1	58	4	0.0645	Missense_Mutation	p.S275N	Non-Targeted Panel Gene	Adult HGBCL, NOS
BCL-5	chr19	38395690	38395690	G	A	exonic	SPRED3	29	2	0.0645	Missense_Mutation	p.A260T	Non-Targeted Panel Gene	Adult HGBCL, NOS
BCL-5	chr6	33250963	33250963	G	A	exonic	VPS52	29	2	0.0645	Missense_Mutation	p.L684F	Non-Targeted Panel Gene	Adult HGBCL, NOS
BCL-5	chr11	62885232	62885232	G	A	exonic	SLC3A2	72	5	0.0649	Missense_Mutation	p.E393K	Non-Targeted Panel Gene	Adult HGBCL, NOS
BCL-5	chr16	16115005	16115005	G	A	exonic	ABCC1	43	3	0.0652	Missense_Mutation	p.V1107I	Non-Targeted Panel Gene	Adult HGBCL, NOS
BCL-5	chr4	8080727	8080727	C	T	exonic	ABLIM2	43	3	0.0652	Missense_Mutation	p.C177Y	Non-Targeted Panel Gene	Adult HGBCL, NOS
BCL-5	chr7	21619963	21619963	C	T	exonic	DNAH11	43	3	0.0652	Missense_Mutation	p.T1462I	Non-Targeted Panel Gene	Adult HGBCL, NOS
BCL-5	chr5	6746383	6746383	G	A	exonic	PAPD7	43	3	0.0652	Missense_Mutation	p.G222E	Non-Targeted Panel Gene	Adult HGBCL, NOS
BCL-5	chr11	118900838	118900838	G	A	exonic	BCL9L	28	2	0.0667	Missense_Mutation	p.P969S	Non-Targeted Panel Gene	Adult HGBCL, NOS
BCL-5	chr8	120168227	120168227	G	A	exonic	COL14A1	28	2	0.0667	Missense_Mutation	p.S139N	Non-Targeted Panel Gene	Adult HGBCL, NOS
BCL-5	chr15	75677760	75677760	C	T	exonic	CSPG4	28	2	0.0667	Missense_Mutation	p.A1693T	Non-Targeted Panel Gene	Adult HGBCL, NOS
BCL-5	chr7	143380754	143380754	G	A	exonic	LOC100507507	28	2	0.0667	Missense_Mutation	p.P10L	Non-Targeted Panel Gene	Adult HGBCL, NOS
BCL-5	chr6	11191138	11191138	G	A	exonic	NEDD9	42	3	0.0667	Missense_Mutation	p.P244L	Non-Targeted Panel Gene	Adult HGBCL, NOS
BCL-5	chr7	100390063	100390063	G	A	exonic	PILRA	70	5	0.0667	Missense_Mutation	p.M210I	Non-Targeted Panel Gene	Adult HGBCL, NOS
BCL-5	chr5	141640009	141640009	G	A	exonic	RELL2	28	2	0.0667	Missense_Mutation	p.G198E	Non-Targeted Panel Gene	Adult HGBCL, NOS
BCL-5	chr5	178138219	178138219	C	T	exonic	RMND5B	28	2	0.0667	Missense_Mutation	p.H34Y	Non-Targeted Panel Gene	Adult HGBCL, NOS
BCL-5	chr1	38859538	38859538	C	T	exonic	RRAGC	42	3	0.0667	Missense_Mutation	p.A37T	Non-Targeted Panel Gene	Adult HGBCL, NOS
BCL-5	chr17	7042404	7042404	C	T	exonic	SLC16A11	42	3	0.0667	Missense_Mutation	p.V260I	Non-Targeted Panel Gene	Adult HGBCL, NOS
BCL-5	chr6	32095866	32095866	C	T	exonic	TNXB	28	2	0.0667	Missense_Mutation	p.V663M	Non-Targeted Panel Gene	Adult HGBCL, NOS
BCL-5	chr11	78200791	78200791	G	A	exonic	USP35	28	2	0.0667	Missense_Mutation	p.V394I	Non-Targeted Panel Gene	Adult HGBCL, NOS
BCL-5	chr17	10652426	10652426	C	T	exonic	MYH3	55	4	0.0678	Missense_Mutation	p.M114I	Non-Targeted Panel Gene	Adult HGBCL, NOS
BCL-5	chr1	246860976	246860976	G	A	exonic	AHCTF1	41	3	0.0682	Missense_Mutation	p.T1361I	Non-Targeted Panel Gene	Adult HGBCL, NOS
BCL-5	chr9	131197992	131197992	G	A	exonic	NUP214	41	3	0.0682	Missense_Mutation	p.A1500T	Non-Targeted Panel Gene	Adult HGBCL, NOS
BCL-5	chrX	154465147	154465147	G	A	exonic	PLXNA3	41	3	0.0682	Missense_Mutation	p.V725M	Non-Targeted Panel Gene	Adult HGBCL, NOS
BCL-5	chrX	54543034	54543034	G	A	exonic	GNL3L	27	2	0.069	Missense_Mutation	p.R129H	Non-Targeted Panel Gene	Adult HGBCL, NOS
BCL-5	chr11	747566	747566	G	A	exonic	TALDO1	27	2	0.069	Missense_Mutation	p.G29S	Non-Targeted Panel Gene	Adult HGBCL, NOS
BCL-5	chr2	178621987	178621987	C	A	exonic	TTN	27	2	0.069	Missense_Mutation	p.A14979S	Non-Targeted Panel Gene	Adult HGBCL, NOS
BCL-5	chr1	32192317	32192317	G	A	exonic	TXLNA	54	4	0.069	Missense_Mutation	p.D324N	Non-Targeted Panel Gene	Adult HGBCL, NOS
BCL-5	chr22	30466999	30466999	C	T	exonic	SEC14L3	40	3	0.0698	Missense_Mutation	p.V91I	Non-Targeted Panel Gene	Adult HGBCL, NOS
BCL-5	chr6	82365217	82365217	C	T	exonic	TPBG	40	3	0.0698	Missense_Mutation	p.P86S	Non-Targeted Panel Gene	Adult HGBCL, NOS
BCL-5	chr7	100822639	100822639	G	A	exonic	EPHB4	26	2	0.0714	Missense_Mutation	p.T147I	Non-Targeted Panel Gene	Adult HGBCL, NOS
BCL-5	chr2	96693548	96693548	C	T	exonic	FER1L5	39	3	0.0714	Missense_Mutation	p.T1112I	Non-Targeted Panel Gene	Adult HGBCL, NOS
BCL-5	chr21	46284392	46284392	G	A	exonic	MCM3AP	39	3	0.0714	Missense_Mutation	p.R299C	Non-Targeted Panel Gene	Adult HGBCL, NOS
BCL-5	chr8	98949043	98949043	C	T	exonic	OSR2	39	3	0.0714	Missense_Mutation	p.P152S	Non-Targeted Panel Gene	Adult HGBCL, NOS
BCL-5	chr19	36111264	36111264	C	T	exonic	OVOL3	26	2	0.0714	Missense_Mutation	p.P21L	Non-Targeted Panel Gene	Adult HGBCL, NOS
BCL-5	chr17	76280902	76280902	C	T	exonic	QRICH2	26	2	0.0714	Missense_Mutation	p.D1273N	Non-Targeted Panel Gene	Adult HGBCL, NOS
BCL-5	chr20	64048195	64048195	C	T	exonic	SOX18	39	3	0.0714	Missense_Mutation	p.V376I	Non-Targeted Panel Gene	Adult HGBCL, NOS
BCL-5	chr13	52029298	52029298	C	T	exonic	UTP14C	26	2	0.0714	Missense_Mutation	p.P165L	Non-Targeted Panel Gene	Adult HGBCL, NOS
BCL-5	chrX	129753172	129753172	C	T	exonic	XPNPEP2	39	3	0.0714	Missense_Mutation	p.T344I	Non-Targeted Panel Gene	Adult HGBCL, NOS
BCL-5	chr2	69181834	69181834	G	A	exonic	ANTXR1	51	4	0.0727	Missense_Mutation	p.A380T	Non-Targeted Panel Gene	Adult HGBCL, NOS
BCL-5	chr14	105464765	105464765	C	T	exonic	MTA1	51	4	0.0727	Missense_Mutation	p.T479M	Non-Targeted Panel Gene	Adult HGBCL, NOS
BCL-5	chr6	17817065	17817065	C	T	exonic	KIF13A	38	3	0.0732	Missense_Mutation	p.S652N	Non-Targeted Panel Gene	Adult HGBCL, NOS
BCL-5	chrX	11156617	11156617	C	T	exonic	ARHGAP6	25	2	0.0741	Missense_Mutation	p.D607N	Non-Targeted Panel Gene	Adult HGBCL, NOS
BCL-5	chr7	138709789	138709789	G	A	exonic	ATP6V0A4	50	4	0.0741	Missense_Mutation	p.S755F	Non-Targeted Panel Gene	Adult HGBCL, NOS
BCL-5	chr7	51065267	51065267	G	A	exonic	COBL	50	4	0.0741	Missense_Mutation	p.L427F	Non-Targeted Panel Gene	Adult HGBCL, NOS
BCL-5	chr8	75014883	75014883	G	T	exonic	CRISPLD1	25	2	0.0741	Missense_Mutation	p.G233V	Non-Targeted Panel Gene	Adult HGBCL, NOS
BCL-5	chr17	4739324	4739324	G	A	exonic	CXCL16	25	2	0.0741	Missense_Mutation	p.R25W	Non-Targeted Panel Gene	Adult HGBCL, NOS
BCL-5	chr2	108929238	108929238	C	T	exonic	EDAR	50	4	0.0741	Missense_Mutation	p.D106N	Non-Targeted Panel Gene	Adult HGBCL, NOS
BCL-5	chr5	150010501	150010501	G	A	exonic	HMGXB3	25	2	0.0741	Missense_Mutation	p.V235I	Non-Targeted Panel Gene	Adult HGBCL, NOS
BCL-5	chr1	31814881	31814881	C	A	exonic	SPOCD1	24	2	0.0741	Missense_Mutation	p.E151D	Non-Targeted Panel Gene	Adult HGBCL, NOS
BCL-5	chr22	30292436	30292436	G	A	exonic	TBC1D10A	25	2	0.0741	Missense_Mutation	p.S489L	Non-Targeted Panel Gene	Adult HGBCL, NOS
BCL-5	chr9	138105767	138105767	G	A	exonic	CACNA1B	37	3	0.075	Missense_Mutation	p.M1796I	Non-Targeted Panel Gene	Adult HGBCL, NOS
BCL-5	chr4	151650365	151650365	C	A	exonic	FAM160A1	37	3	0.075	Missense_Mutation	p.P775H	Non-Targeted Panel Gene	Adult HGBCL, NOS
BCL-5	chr14	41887922	41887922	C	T	exonic	LRFN5	37	3	0.075	Missense_Mutation	p.L433F	Non-Targeted Panel Gene	Adult HGBCL, NOS

BCL-5	chr1	152087283	152087283	C	T	exonic	TCHHL1	49	4	0.0755	Missense_Mutation	p.M133I	Non-Targeted Panel Gene	Adult HGBCL, NOS
BCL-5	chr3	170907781	170907781	G	A	exonic	EIF5A2	24	2	0.0769	Missense_Mutation	p.T9I	Non-Targeted Panel Gene	Adult HGBCL, NOS
BCL-5	chr12	51192356	51192356	G	A	exonic	POU6F1	48	4	0.0769	Missense_Mutation	p.T432I	Non-Targeted Panel Gene	Adult HGBCL, NOS
BCL-5	chr1	155868373	155868373	C	T	exonic	SYT11	36	3	0.0769	Missense_Mutation	p.S148F	Non-Targeted Panel Gene	Adult HGBCL, NOS
BCL-5	chr19	10357852	10357852	G	A	exonic	TYK2	24	2	0.0769	Missense_Mutation	p.T793I	Non-Targeted Panel Gene	Adult HGBCL, NOS
BCL-5	chr10	65966736	65966736	G	A	exonic	CTNNA3	47	4	0.0784	Missense_Mutation	p.P759L	Non-Targeted Panel Gene	Adult HGBCL, NOS
BCL-5	chr16	86511006	86511006	G	A	exonic	FOXF1	35	3	0.0789	Missense_Mutation	p.R146K	Non-Targeted Panel Gene	Adult HGBCL, NOS
BCL-5	chr6	18121777	18121777	G	A	exonic	NHLRC1	35	3	0.0789	Missense_Mutation	p.A277V	Non-Targeted Panel Gene	Adult HGBCL, NOS
BCL-5	chr13	113321703	113321703	G	A	exonic	LAMP1	34	3	0.0811	Missense_Mutation	p.V364M	Non-Targeted Panel Gene	Adult HGBCL, NOS
BCL-5	chr1	226277927	226277927	G	A	exonic	LIN9	34	3	0.0811	Missense_Mutation	p.S193F	Non-Targeted Panel Gene	Adult HGBCL, NOS
BCL-5	chr10	79292246	79292246	G	A	exonic	ZMIZ1	34	3	0.0811	Missense_Mutation	p.A283T	Non-Targeted Panel Gene	Adult HGBCL, NOS
BCL-5	chr3	47124079	47124079	G	A	exonic	SETD2	45	4	0.0816	Missense_Mutation	p.P186L	Non-Targeted Panel Gene	Adult HGBCL, NOS
BCL-5	chr11	418442	418442	C	T	exonic	ANO9	33	3	0.0833	Missense_Mutation	p.G760S	Non-Targeted Panel Gene	Adult HGBCL, NOS
BCL-5	chr17	45406062	45406062	C	T	exonic	ARHGAP27	33	3	0.0833	Missense_Mutation	p.V227M	Non-Targeted Panel Gene	Adult HGBCL, NOS
BCL-5	chr19	45786468	45786468	G	A	exonic	DMWD	44	4	0.0833	Missense_Mutation	p.T343M	Non-Targeted Panel Gene	Adult HGBCL, NOS
BCL-5	chr15	37096332	37096332	G	A	exonic	MEIS2	54	5	0.0833	Missense_Mutation	p.S115F	Non-Targeted Panel Gene	Adult HGBCL, NOS
BCL-5	chr14	64727956	64727956	G	A	exonic	PLEKHG3	55	5	0.0833	Missense_Mutation	p.V109I	Non-Targeted Panel Gene	Adult HGBCL, NOS
BCL-5	chrX	49286812	49286812	A	G	exonic	PPP1R3F	22	2	0.0833	Missense_Mutation	p.S708G	Non-Targeted Panel Gene	Adult HGBCL, NOS
BCL-5	chr8	27922225	27922225	G	T	exonic	SCARA5	22	2	0.0833	Missense_Mutation	p.P88T	Non-Targeted Panel Gene	Adult HGBCL, NOS
BCL-5	chr12	30728713	30728713	C	T	exonic	CAPRIN2	43	4	0.0851	Missense_Mutation	p.A492T	Non-Targeted Panel Gene	Adult HGBCL, NOS
BCL-5	chr20	32930464	32930464	G	A	exonic	EFCAB8	43	4	0.0851	Missense_Mutation	p.G827S	Non-Targeted Panel Gene	Adult HGBCL, NOS
BCL-5	chrX	118643590	118643590	G	A	exonic	DOCK11	32	3	0.0857	Missense_Mutation	p.S1465N	Non-Targeted Panel Gene	Adult HGBCL, NOS
BCL-5	chr11	74836225	74836225	C	T	exonic	RNF169	32	3	0.0857	Missense_Mutation	p.T541I	Non-Targeted Panel Gene	Adult HGBCL, NOS
BCL-5	chr14	99175586	99175586	G	A	exonic	BCL11B	21	2	0.087	Missense_Mutation	p.T417M	Non-Targeted Panel Gene	Adult HGBCL, NOS
BCL-5	chr7	42912052	42912052	C	T	exonic	C7orf25	42	4	0.087	Missense_Mutation	p.E6K	Non-Targeted Panel Gene	Adult HGBCL, NOS
BCL-5	chr20	35472815	35472815	G	A	exonic	CEP250	31	3	0.0882	Missense_Mutation	p.R398K	Non-Targeted Panel Gene	Adult HGBCL, NOS
BCL-5	chr14	45136150	45136150	C	T	exonic	FANCM	31	3	0.0882	Missense_Mutation	p.A40V	Non-Targeted Panel Gene	Adult HGBCL, NOS
BCL-5	chr7	101198236	101198236	G	A	exonic	MOGAT3	31	3	0.0882	Missense_Mutation	p.T208M	Non-Targeted Panel Gene	Adult HGBCL, NOS
BCL-5	chr20	58986842	58986842	G	A	exonic	NELFCD	31	3	0.0882	Missense_Mutation	p.A107T	Non-Targeted Panel Gene	Adult HGBCL, NOS
BCL-5	chr1	228212520	228212520	C	T	exonic;splicing	OBSCN;OBSCN-AS1	31	3	0.0882	Missense_Mutation	p.T246I	Non-Targeted Panel Gene	Adult HGBCL, NOS
BCL-5	chr19	48960987	48960987	C	T	exonic	BAX	41	4	0.0889	Missense_Mutation	p.P183S	Non-Targeted Panel Gene	Adult HGBCL, NOS
BCL-5	chr1	110060989	110060989	C	T	exonic	ALX3	20	2	0.0909	Missense_Mutation	p.C259Y	Non-Targeted Panel Gene	Adult HGBCL, NOS
BCL-5	chr19	45780041	45780041	G	A	exonic	DMPK	20	2	0.0909	Missense_Mutation	p.P7S	Non-Targeted Panel Gene	Adult HGBCL, NOS
BCL-5	chr9	93035582	93035582	C	T	exonic	FGD3	20	2	0.0909	Missense_Mutation	p.A724V	Non-Targeted Panel Gene	Adult HGBCL, NOS
BCL-5	chr15	33154064	33154064	G	A	exonic	FMN1	40	4	0.0909	Missense_Mutation	p.P284L	Non-Targeted Panel Gene	Adult HGBCL, NOS
BCL-5	chr19	47275031	47275031	C	T	exonic	INAFM1	30	3	0.0909	Missense_Mutation	p.L38F	Non-Targeted Panel Gene	Adult HGBCL, NOS
BCL-5	chr20	44186645	44186645	C	T	exonic	JPH2	30	3	0.0909	Missense_Mutation	p.G21R	Non-Targeted Panel Gene	Adult HGBCL, NOS
BCL-5	chr17	3535648	3535648	C	T	exonic	TRPV3	20	2	0.0909	Missense_Mutation	p.A237T	Non-Targeted Panel Gene	Adult HGBCL, NOS
BCL-5	chr11	65048042	65048042	G	A	exonic	NAALADL1	18	2	0.0952	Missense_Mutation	p.A452V	Non-Targeted Panel Gene	Adult HGBCL, NOS
BCL-5	chr17	72121711	72121711	G	A	exonic	SOX9	38	4	0.0952	Missense_Mutation	p.R107Q	Non-Targeted Panel Gene	Adult HGBCL, NOS
BCL-5	chr5	150374744	150374744	G	A	exonic	TCOF1	19	2	0.0952	Missense_Mutation	p.G404E	Non-Targeted Panel Gene	Adult HGBCL, NOS
BCL-5	chr2	46941642	46941642	G	A	exonic	MCFD2;TTC7A	28	3	0.0968	Missense_Mutation	p.R34Q	Non-Targeted Panel Gene	Adult HGBCL, NOS
BCL-5	chr4	163473679	163473679	G	A	exonic	TKTL2	28	3	0.0968	Missense_Mutation	p.T19I	Non-Targeted Panel Gene	Adult HGBCL, NOS
BCL-5	chr7	130192798	130192798	G	A	exonic	TMEM209	28	3	0.0968	Missense_Mutation	p.P200L	Non-Targeted Panel Gene	Adult HGBCL, NOS
BCL-5	chr6	32095871	32095871	C	T	exonic	TNXB	28	3	0.0968	Missense_Mutation	p.R661Q	Non-Targeted Panel Gene	Adult HGBCL, NOS
BCL-5	chr1	205256392	205256392	G	A	exonic	TMCC2	37	4	0.0976	Missense_Mutation	p.M1I	Non-Targeted Panel Gene	Adult HGBCL, NOS
BCL-5	chr1	151532523	151532523	C	T	exonic	CGN	46	5	0.098	Missense_Mutation	p.A898V	Non-Targeted Panel Gene	Adult HGBCL, NOS
BCL-5	chr2	241188657	241188657	C	T	exonic	ANO7	18	2	0.1	Missense_Mutation	p.L16F	Non-Targeted Panel Gene	Adult HGBCL, NOS
BCL-5	chr10	110297717	110297717	G	A	exonic	SMNDC1	36	4	0.1	Missense_Mutation	p.A92V	Non-Targeted Panel Gene	Adult HGBCL, NOS
BCL-5	chr6	41691065	41691065	G	A	exonic	TFEB	18	2	0.1	Missense_Mutation	p.T50I	Non-Targeted Panel Gene	Adult HGBCL, NOS
BCL-5	chr1	165768853	165768853	G	A	exonic	TMCO1	18	2	0.1	Missense_Mutation	p.L18F	Non-Targeted Panel Gene	Adult HGBCL, NOS
BCL-5	chr7	73596405	73596405	G	A	exonic	MLXIPL	26	3	0.1034	Missense_Mutation	p.P633S	Non-Targeted Panel Gene	Adult HGBCL, NOS
BCL-5	chr11	65130346	65130346	C	T	exonic	SYVN1	26	3	0.1034	Missense_Mutation	p.G380D	Non-Targeted Panel Gene	Adult HGBCL, NOS
BCL-5	chr10	56360066	56360066	C	T	exonic	ZWINT	26	3	0.1034	Missense_Mutation	p.E70K	Non-Targeted Panel Gene	Adult HGBCL, NOS
BCL-5	chr6	30160797	30160797	C	T	exonic	TRIM10	17	2	0.1053	Missense_Mutation	p.G21D	Non-Targeted Panel Gene	Adult HGBCL, NOS
BCL-5	chr16	89770580	89770580	C	T	exonic	FANCA	25	3	0.1071	Missense_Mutation	p.V736I	Non-Targeted Panel Gene	Adult HGBCL, NOS
BCL-5	chr10	49325936	49325936	G	A	exonic	C10orf71	24	3	0.1111	Missense_Mutation	p.A1131T	Non-Targeted Panel Gene	Adult HGBCL, NOS
BCL-5	chr19	15878838	15878838	C	T	exonic	CYP4F2	16	2	0.1111	Missense_Mutation	p.R499H	Non-Targeted Panel Gene	Adult HGBCL, NOS
BCL-5	chrX	84336818	84336818	C	T	exonic	HDX	24	3	0.1111	Missense_Mutation	p.V575I	Non-Targeted Panel Gene	Adult HGBCL, NOS
BCL-5	chr12	122992546	122992546	G	A	exonic	PITPNM2	16	2	0.1111	Missense_Mutation	p.P786L	Non-Targeted Panel Gene	Adult HGBCL, NOS
BCL-5	chr8	15540438	15540438	C	T	exonic	TUSC3	16	2	0.1111	Missense_Mutation	p.A3V	Non-Targeted Panel Gene	Adult HGBCL, NOS
BCL-5	chr1	27305164	27305164	G	A	exonic	WDTC1	16	2	0.1111	Missense_Mutation	p.V602I	Non-Targeted Panel Gene	Adult HGBCL, NOS
BCL-5	chr17	17977674	17977674	G	A	exonic	DRC3	23	3	0.1154	Missense_Mutation	p.G26S	Non-Targeted Panel Gene	Adult HGBCL, NOS
BCL-5	chr15	23566353	23566353	G	A	exonic	MKRN3	23	3	0.1154	Missense_Mutation	p.A191T	Non-Targeted Panel Gene	Adult HGBCL, NOS
BCL-5	chr11	798095	798095	C	T	exonic	PANO1	23	3	0.1154	Missense_Mutation	p.P157L	Non-Targeted Panel Gene	Adult HGBCL, NOS
BCL-5	chr5	172770658	172770658	C	T	exonic	DUSP1	15	2	0.1176	Missense_Mutation	p.D99N	Non-Targeted Panel Gene	Adult HGBCL, NOS
BCL-5	chr2	26279148	26279148	C	T	exonic	HADHB	15	2	0.1176	Missense_Mutation	p.S193F	Non-Targeted Panel Gene	Adult HGBCL, NOS
BCL-5	chr1	152699086	152699086	C	T	exonic	LCE2A	15	2	0.1176	Missense_Mutation	p.S62F	Non-Targeted Panel Gene	Adult HGBCL, NOS
BCL-5	chr16	12902602	12902602	C	T	exonic	SHISA9	15	2	0.1176	Missense_Mutation	p.R180W	Non-Targeted Panel Gene	Adult HGBCL, NOS
BCL-5	chr10	123667026	123667026	G	A	exonic	GPR26	22	3	0.12	Missense_Mutation	p.D207N	Non-Targeted Panel Gene	Adult HGBCL, NOS
BCL-5	chr13	20989146	20989146	C	T	exonic	LATS2	22	3	0.12	Missense_Mutation	p.V212M	Non-Targeted Panel Gene	Adult HGBCL, NOS
BCL-5	chr1	118025360	118025360	G	A	exonic	SPAG17	22	3	0.12	Missense_Mutation	p.P1263S	Non-Targeted Panel Gene	Adult HGBCL, NOS
BCL-5	chr11	128980739	128980739	G	A	exonic	ARHGAP32	14	2	0.125	Missense_Mutation	p.S583L	Non-Targeted Panel Gene	Adult HGBCL, NOS
BCL-5	chr6	168308340	168308340	G	A	exonic	DACT2	14	2	0.125	Missense_Mutation	p.P473S	Non-Targeted Panel Gene	Adult HGBCL, NOS
BCL-5	chr9	92517687	92517687	C	T	exonic	ECM2	14	2	0.125	Missense_Mutation	p.V161I	Non-Targeted Panel Gene	Adult HGBCL, NOS
BCL-5	chr10	92692512	92692512	G	A	exonic	HHEX	14	2	0.125	Missense_Mutation	p.R169H	Non-Targeted Panel Gene	Adult HGBCL, NOS
BCL-5	chr6	75914969	75914969	G	A	exonic	MYO6	21	3	0.125	Missense_Mutation	p.R1272Q	Non-Targeted Panel Gene	Adult HGBCL, NOS
BCL-5	chr14	21155049	21155049	G	A	exonic	OR5AU1	35	5	0.125	Missense_Mutation	p.A326V	Non-Targeted Panel Gene	Adult HGBCL, NOS
BCL-5	chr3	61989804	61989804	G	A	exonic	PTPRG	14	2	0.125	Missense_Mutation	p.V124I	Non-Targeted Panel Gene	Adult HGBCL, NOS
BCL-5	chrX	70929679	70929679	C	T	exonic	SLC7A3	14	2	0.125	Missense_Mutation	p.E107K	Non-Targeted Panel Gene	Adult HGBCL, NOS
BCL-5	chr7	12214827	12214827	C	T	exonic	TMEM106B	21	3	0.125	Missense_Mutation	p.S6F	Non-Targeted Panel Gene	Adult HGBCL, NOS
BCL-5	chr16	56485590	56485590	C	T	exonic	BBS2	34	5	0.1282	Missense_Mutation	p.V687I	Non-Targeted Panel Gene	Adult HGBCL, NOS

BCL-5	chr7	55982254	55982254	G	A	exonic	NIPSNAP2	27	4	0.129	Missense_Mutation	p.V140I	Non-Targeted Panel Gene	Adult HGBCL, NOS
BCL-5	chrX	23000554	23000554	G	A	exonic	DDX53	20	3	0.1304	Missense_Mutation	p.R166K	Non-Targeted Panel Gene	Adult HGBCL, NOS
BCL-5	chr6	99412724	99412724	G	A	exonic	PNISR	20	3	0.1304	Missense_Mutation	p.A35V	Non-Targeted Panel Gene	Adult HGBCL, NOS
BCL-5	chr20	2417471	2417471	C	T	exonic	TGM6	20	3	0.1304	Missense_Mutation	p.R526W	Non-Targeted Panel Gene	Adult HGBCL, NOS
BCL-5	chr6	26598542	26598542	C	T	exonic	ABT1	13	2	0.1333	Missense_Mutation	p.A239V	Non-Targeted Panel Gene	Adult HGBCL, NOS
BCL-5	chr19	36141061	36141061	G	A	exonic	CAPNS1	13	2	0.1333	Missense_Mutation	p.G17E	Non-Targeted Panel Gene	Adult HGBCL, NOS
BCL-5	chr14	77026168	77026168	C	T	exonic	IRF2BPL	13	2	0.1333	Missense_Mutation	p.R542H	Non-Targeted Panel Gene	Adult HGBCL, NOS
BCL-5	chr4	4863040	4863040	C	T	exonic	MSX1	13	2	0.1333	Missense_Mutation	p.S270L	Non-Targeted Panel Gene	Adult HGBCL, NOS
BCL-5	chr19	40423210	40423210	C	T	exonic	SERTAD1	13	2	0.1333	Missense_Mutation	p.A113T	Non-Targeted Panel Gene	Adult HGBCL, NOS
BCL-5	chr19	58356470	58356470	G	A	exonic	ZNF497	13	2	0.1333	Missense_Mutation	p.A389V	Non-Targeted Panel Gene	Adult HGBCL, NOS
BCL-5	chr17	3882553	3882553	G	T	exonic	CAMKK1	12	2	0.1429	Missense_Mutation	p.D220E	Non-Targeted Panel Gene	Adult HGBCL, NOS
BCL-5	chr14	69573400	69573400	C	T	exonic	CCDC177	18	3	0.1429	Missense_Mutation	p.D75N	Non-Targeted Panel Gene	Adult HGBCL, NOS
BCL-5	chr14	104883428	104883428	G	A	exonic	CEP170B	12	2	0.1429	Missense_Mutation	p.S324N	Non-Targeted Panel Gene	Adult HGBCL, NOS
BCL-5	chr6	1611860	1611860	C	T	exonic	FOXC1	11	2	0.1429	Missense_Mutation	p.S472L	Non-Targeted Panel Gene	Adult HGBCL, NOS
BCL-5	chr3	50348161	50348161	C	T	exonic	NPRL2	12	2	0.1429	Missense_Mutation	p.G299S	Non-Targeted Panel Gene	Adult HGBCL, NOS
BCL-5	chr15	42087634	42087634	C	T	exonic	PLA2G4D	12	2	0.1429	Missense_Mutation	p.D38N	Non-Targeted Panel Gene	Adult HGBCL, NOS
BCL-5	chr1	1046687	1046687	G	A	exonic	AGRN	17	3	0.15	Missense_Mutation	p.D1068N	Non-Targeted Panel Gene	Adult HGBCL, NOS
BCL-5	chr4	95148923	95148923	G	A	exonic	BMPR1B	17	3	0.15	Missense_Mutation	p.G418S	Non-Targeted Panel Gene	Adult HGBCL, NOS
BCL-5	chr14	60107654	60107654	C	T	exonic	PCNX4	17	3	0.15	Missense_Mutation	p.P6S	Non-Targeted Panel Gene	Adult HGBCL, NOS
BCL-5	chr3	127680283	127680283	G	T	exonic	ABTB1	11	2	0.1538	Missense_Mutation	p.E415D	Non-Targeted Panel Gene	Adult HGBCL, NOS
BCL-5	chr5	72196395	72196395	G	A	exonic	MAP1B	11	2	0.1538	Missense_Mutation	p.E1014K	Non-Targeted Panel Gene	Adult HGBCL, NOS
BCL-5	chr18	21741605	21741605	C	T	exonic	MIB1	11	2	0.1538	Missense_Mutation	p.R8W	Non-Targeted Panel Gene	Adult HGBCL, NOS
BCL-5	chr5	139392089	139392089	G	A	exonic	PROB1	11	2	0.1538	Missense_Mutation	p.P998L	Non-Targeted Panel Gene	Adult HGBCL, NOS
BCL-5	chr5	176810802	176810802	G	A	exonic	UNC5A	11	2	0.1538	Missense_Mutation	p.A18T	Non-Targeted Panel Gene	Adult HGBCL, NOS
BCL-5	chr16	23073880	23073880	G	A	exonic	USP31	11	2	0.1538	Missense_Mutation	p.A726V	Non-Targeted Panel Gene	Adult HGBCL, NOS
BCL-5	chr1	205241785	205241785	G	A	exonic	TMCC2	21	4	0.16	Missense_Mutation	p.R163Q	Non-Targeted Panel Gene	Adult HGBCL, NOS
BCL-5	chr17	18166361	18166361	G	A	exonic	MYO15A	10	2	0.1667	Missense_Mutation	p.G3263D	Non-Targeted Panel Gene	Adult HGBCL, NOS
BCL-5	chr19	50667549	50667549	G	A	exonic	SHANK1	10	2	0.1667	Missense_Mutation	p.P1471S	Non-Targeted Panel Gene	Adult HGBCL, NOS
BCL-5	chr19	15436874	15436874	G	A	exonic	WIZ	10	2	0.1667	Missense_Mutation	p.P154L	Non-Targeted Panel Gene	Adult HGBCL, NOS
BCL-5	chr5	139395071	139395071	G	A	exonic	PROB1	14	3	0.1765	Missense_Mutation	p.A4V	Non-Targeted Panel Gene	Adult HGBCL, NOS
BCL-5	chr15	62068160	62068160	C	T	exonic	C2CD4A	9	2	0.1818	Missense_Mutation	p.R183C	Non-Targeted Panel Gene	Adult HGBCL, NOS
BCL-5	chr19	36141067	36141067	G	A	exonic	CAPNS1	9	2	0.1818	Missense_Mutation	p.G19E	Non-Targeted Panel Gene	Adult HGBCL, NOS
BCL-5	chr19	9214071	9214071	A	G	exonic	OR7D4	40	9	0.1837	Missense_Mutation	p.L256P	Non-Targeted Panel Gene	Adult HGBCL, NOS
BCL-5	chr1	180916257	180916257	C	T	exonic	KIAA1614	13	3	0.1875	Missense_Mutation	p.P52S	Non-Targeted Panel Gene	Adult HGBCL, NOS
BCL-5	chr11	74703461	74703461	C	T	exonic	CHRDL2	16	4	0.2	Missense_Mutation	p.V264M	Non-Targeted Panel Gene	Adult HGBCL, NOS
BCL-5	chr7	78019389	78019389	C	A	exonic	MAGI2	8	2	0.2	Missense_Mutation	p.G1432W	Non-Targeted Panel Gene	Adult HGBCL, NOS
BCL-5	chr7	73596752	73596752	G	A	exonic	MLXIPL	8	2	0.2	Missense_Mutation	p.P570L	Non-Targeted Panel Gene	Adult HGBCL, NOS
BCL-5	chr11	43443570	43443570	G	A	exonic	TTC17	11	3	0.2143	Missense_Mutation	p.E890K	Non-Targeted Panel Gene	Adult HGBCL, NOS
BCL-5	chr3	101565256	101565256	G	A	exonic	TRMT10C	82	23	0.219	Missense_Mutation	p.E159K	Non-Targeted Panel Gene	Adult HGBCL, NOS
BCL-5	chr3	142198932	142198932	A	T	exonic	GK5	39	11	0.22	Missense_Mutation	p.I138K	Non-Targeted Panel Gene	Adult HGBCL, NOS
BCL-5	chrX	153397163	153397163	G	T	exonic	PNMA6E	7	2	0.2222	Missense_Mutation	p.P285T	Non-Targeted Panel Gene	Adult HGBCL, NOS
BCL-5	chr19	15457630	15457630	G	A	exonic	RASAL3	7	2	0.2222	Missense_Mutation	p.R365C	Non-Targeted Panel Gene	Adult HGBCL, NOS
BCL-5	chrX	100665251	100665251	C	T	exonic	SRPX2	7	2	0.2222	Missense_Mutation	p.P181S	Non-Targeted Panel Gene	Adult HGBCL, NOS
BCL-5	chr17	53824436	53824436	G	A	exonic	KIF2B	12	4	0.25	Missense_Mutation	p.G468E	Non-Targeted Panel Gene	Adult HGBCL, NOS
BCL-5	chr15	88843597	88843597	C	A	exonic	ACAN	16	6	0.2727	Missense_Mutation	p.Q334K	Non-Targeted Panel Gene	Adult HGBCL, NOS
BCL-5	chr22	30906007	30906007	G	A	exonic	OSBP2	8	3	0.2727	Missense_Mutation	p.R683H	Non-Targeted Panel Gene	Adult HGBCL, NOS
BCL-5	chr1	21756579	21756579	A	G	exonic	USP48	27	11	0.2895	Missense_Mutation	p.Y127H	Non-Targeted Panel Gene	Adult HGBCL, NOS
BCL-5	chr2	159946854	159946854	G	A	exonic	PLA2R1	67	30	0.3061	Missense_Mutation	p.A1305V	Non-Targeted Panel Gene	Adult HGBCL, NOS
BCL-5	chr3	169768189	169768189	A	C	exonic	ACTRT3	61	27	0.3068	Missense_Mutation	p.M121R	Non-Targeted Panel Gene	Adult HGBCL, NOS
BCL-5	chr12	117272510	117272510	C	T	exonic	NOS1	33	17	0.34	Missense_Mutation	p.V572M	Non-Targeted Panel Gene	Adult HGBCL, NOS
BCL-5	chr15	43151900	43151900	G	C	exonic	TMEM62	23	12	0.3429	Missense_Mutation	p.G326A	Non-Targeted Panel Gene	Adult HGBCL, NOS
BCL-5	chr17	44557999	44557999	A	G	exonic	FZD2	30	16	0.3478	Missense_Mutation	p.E104G	Non-Targeted Panel Gene	Adult HGBCL, NOS
BCL-5	chr5	172670006	172670006	C	T	exonic	NEURL1B	20	11	0.3548	Missense_Mutation	p.R85C	Non-Targeted Panel Gene	Adult HGBCL, NOS
BCL-5	chr2	214781215	214781215	A	G	exonic	BARD1	46	26	0.3611	Missense_Mutation	p.L220S	Non-Targeted Panel Gene	Adult HGBCL, NOS
BCL-5	chr4	153703887	153703887	A	T	exonic	TLR2	51	29	0.3625	Missense_Mutation	p.D327V	Non-Targeted Panel Gene	Adult HGBCL, NOS
BCL-5	chr17	44558002	44558002	A	T	exonic	FZD2	29	17	0.3696	Missense_Mutation	p.Q105L	Non-Targeted Panel Gene	Adult HGBCL, NOS
BCL-5	chr1	179652132	179652132	C	A	exonic	TDRD5	39	23	0.371	Missense_Mutation	p.Q699K	Non-Targeted Panel Gene	Adult HGBCL, NOS
BCL-5	chr9	127730984	127730984	G	A	exonic	TTC16	47	29	0.3816	Missense_Mutation	p.S734N	Non-Targeted Panel Gene	Adult HGBCL, NOS
BCL-5	chr4	46386138	46386138	G	C	exonic	GABRA2	35	22	0.386	Missense_Mutation	p.I41M	Non-Targeted Panel Gene	Adult HGBCL, NOS
BCL-5	chr5	146013994	146013994	C	G	exonic	SH3RF2	41	26	0.3881	Missense_Mutation	p.S331C	Non-Targeted Panel Gene	Adult HGBCL, NOS
BCL-5	chr17	1728340	1728340	G	C	exonic	WDR81	33	21	0.3889	Missense_Mutation	p.E76D	Non-Targeted Panel Gene	Adult HGBCL, NOS
BCL-5	chr8	130800560	130800560	G	C	exonic	ADCY8	14	9	0.3913	Missense_Mutation	p.Q976E	Non-Targeted Panel Gene	Adult HGBCL, NOS
BCL-5	chr21	37164149	37164149	A	C	exonic	TTC3	45	29	0.3919	Missense_Mutation	p.E1108A	Non-Targeted Panel Gene	Adult HGBCL, NOS
BCL-5	chr3	138064686	138064686	A	G	exonic	DZIP1L	10	7	0.4118	Missense_Mutation	p.L695P	Non-Targeted Panel Gene	Adult HGBCL, NOS
BCL-5	chr19	6187677	6187677	A	C	exonic	ACSBG2	35	25	0.4167	Missense_Mutation	p.S587R	Non-Targeted Panel Gene	Adult HGBCL, NOS
BCL-5	chr1	97082391	97082391	T	A	exonic	DPYD	57	42	0.4242	Missense_Mutation	p.D949V	Non-Targeted Panel Gene	Adult HGBCL, NOS
BCL-5	chr2	230446886	230446886	A	C	exonic	SP100	27	20	0.4255	Missense_Mutation	p.Q134H	Non-Targeted Panel Gene	Adult HGBCL, NOS
BCL-5	chr7	104161095	104161095	G	C	exonic	ORC5	47	35	0.4268	Missense_Mutation	p.P376A	Non-Targeted Panel Gene	Adult HGBCL, NOS
BCL-5	chr1	203683371	203683371	A	G	exonic	ATP2B4	20	15	0.4286	Missense_Mutation	p.S56G	Non-Targeted Panel Gene	Adult HGBCL, NOS
BCL-5	chr8	51408751	51408751	T	A	exonic	PXDNL	17	13	0.4333	Missense_Mutation	p.D958V	Non-Targeted Panel Gene	Adult HGBCL, NOS
BCL-5	chr8	38135731	38135731	T	G	exonic	ASH2L	39	30	0.4348	Missense_Mutation	p.Y562D	Non-Targeted Panel Gene	Adult HGBCL, NOS
BCL-5	chr9	34611284	34611284	G	A	exonic	RPP25L	10	8	0.4444	Missense_Mutation	p.R5W	Non-Targeted Panel Gene	Adult HGBCL, NOS
BCL-5	chr19	41256510	41256510	A	G	exonic	AXL	57	47	0.4519	Missense_Mutation	p.N699D	Non-Targeted Panel Gene	Adult HGBCL, NOS
BCL-5	chr1	36301046	36301046	C	T	exonic	THRAP3	15	13	0.4643	Missense_Mutation	p.P822S	Non-Targeted Panel Gene	Adult HGBCL, NOS
BCL-5	chr1	158656639	158656639	A	T	exonic	SPTA1	26	23	0.4694	Missense_Mutation	p.H941Q	Non-Targeted Panel Gene	Adult HGBCL, NOS
BCL-5	chr19	9991795	9991795	C	T	exonic	COL5A3	18	16	0.4706	Missense_Mutation	p.G647E	Non-Targeted Panel Gene	Adult HGBCL, NOS
BCL-5	chr20	45967623	45967623	C	T	exonic	ZNF335	41	38	0.481	Missense_Mutation	p.A276T	Non-Targeted Panel Gene	Adult HGBCL, NOS
BCL-5	chr2	222304103	222304103	G	C	exonic	CCDC140	48	46	0.4894	Missense_Mutation	p.Q67H	Non-Targeted Panel Gene	Adult HGBCL, NOS
BCL-5	chr2	74165221	74165221	T	G	exonic	MOB1A	24	23	0.4894	Missense_Mutation	p.I136L	Non-Targeted Panel Gene	Adult HGBCL, NOS
BCL-5	chr17	50540861	50540861	C	A	exonic	EPN3	28	28	0.4912	Missense_Mutation	p.P350T	Non-Targeted Panel Gene	Adult HGBCL, NOS
BCL-5	chr7	4759380	4759380	C	G	exonic	FOXK1	15	17	0.5	Missense_Mutation	p.P494R	Non-Targeted Panel Gene	Adult HGBCL, NOS
BCL-5	chr12	102843756	102843756	C	G	exonic	PAH	40	41	0.5062	Missense_Mutation	p.K363N	Non-Targeted Panel Gene	Adult HGBCL, NOS

BCL-5	chr1	44313171	44313171	C	G	exonic	ERI3	19	21	0.5122	Missense_Mutation	p.E222Q	Non-Targeted Panel Gene	Adult HGBCL, NOS
BCL-5	chr1	94007731	94007731	G	A	exonic	ABCA4	26	28	0.5185	Missense_Mutation	p.L1970F	Non-Targeted Panel Gene	Adult HGBCL, NOS
BCL-5	chr8	141254156	141254156	G	A	exonic	SLC45A4	20	23	0.5349	Missense_Mutation	p.P25L	Non-Targeted Panel Gene	Adult HGBCL, NOS
BCL-5	chr3	102457837	102457837	A	G	exonic	ZPLD1	103	123	0.5442	Missense_Mutation	p.N189S	Non-Targeted Panel Gene	Adult HGBCL, NOS
BCL-5	chr20	2116766	2116766	G	T	exonic	STK35	19	24	0.5581	Missense_Mutation	p.R331S	Non-Targeted Panel Gene	Adult HGBCL, NOS
BCL-5	chr5	133099615	133099615	C	G	exonic	HSPA4	22	30	0.5769	Missense_Mutation	p.P667R	Non-Targeted Panel Gene	Adult HGBCL, NOS
BCL-5	chr1	197328965	197328965	T	C	exonic	CRB1	13	22	0.6286	Missense_Mutation	p.I136T	Non-Targeted Panel Gene	Adult HGBCL, NOS
BCL-5	chrX	70432927	70432927	G	T	exonic	GDPD2	6	17	0.7391	Missense_Mutation	p.K569N	Non-Targeted Panel Gene	Adult HGBCL, NOS
BCL-5	chrX	153907851	153907851	G	C	exonic	ARHGAP4	3	12	0.8	Missense_Mutation	p.P947A	Non-Targeted Panel Gene	Adult HGBCL, NOS
BCL-5	chr3	25638168	25638168	T	C	exonic	TOP2B	10	80	0.8889	Missense_Mutation	p.T180A	Non-Targeted Panel Gene	Adult HGBCL, NOS
BCL-5	chrX	103707489	103707489	C	T	exonic	GLRA4	0	25	1	Missense_Mutation	p.R370H	Non-Targeted Panel Gene	Adult HGBCL, NOS
BCL-5	chrX	120439216	120439216	C	T	exonic	LAMP2	0	55	1	Missense_Mutation	p.V391I	Non-Targeted Panel Gene	Adult HGBCL, NOS
BCL-5	chr1	26696932	26696932	C	T	exonic	ARID1A	75	17	0.1848	Nonsense_Mutation	p.Q177X	Targerted Panel Gene	Adult HGBCL, NOS
BCL-5	chr5	180567862	180567862	C	T	exonic	CNOT6	58	2	0.0333	Nonsense_Mutation	p.Q296X	Non-Targeted Panel Gene	Adult HGBCL, NOS
BCL-5	chr6	7577810	7577810	C	A	exonic	DSP	56	2	0.0345	Nonsense_Mutation	p.S970X	Non-Targeted Panel Gene	Adult HGBCL, NOS
BCL-5	chr3	184166636	184166636	G	A	exonic	DVL3	54	2	0.0357	Nonsense_Mutation	p.W337X	Non-Targeted Panel Gene	Adult HGBCL, NOS
BCL-5	chr6	138334595	138334595	C	T	exonic	ARFGEF3	52	2	0.037	Nonsense_Mutation	p.Q1917X	Non-Targeted Panel Gene	Adult HGBCL, NOS
BCL-5	chr17	74999599	74999599	C	T	exonic	CDR2L	51	2	0.0377	Nonsense_Mutation	p.Q59X	Non-Targeted Panel Gene	Adult HGBCL, NOS
BCL-5	chr4	25835222	25835222	G	A	exonic	SEL1L3	50	2	0.0385	Nonsense_Mutation	p.R279X	Non-Targeted Panel Gene	Adult HGBCL, NOS
BCL-5	chr1	43437628	43437628	G	A	exonic	SZT2	50	2	0.0385	Nonsense_Mutation	p.W2051X	Non-Targeted Panel Gene	Adult HGBCL, NOS
BCL-5	chr19	49409009	49409009	C	T	exonic	CCDC155	49	2	0.0392	Nonsense_Mutation	p.Q346X	Non-Targeted Panel Gene	Adult HGBCL, NOS
BCL-5	chr1	74205702	74205702	C	A	exonic	FPGT	49	2	0.0392	Nonsense_Mutation	p.S311X	Non-Targeted Panel Gene	Adult HGBCL, NOS
BCL-5	chr13	19668465	19668465	C	T	exonic	MPHOSPH8	73	3	0.0395	Nonsense_Mutation	p.R755X	Non-Targeted Panel Gene	Adult HGBCL, NOS
BCL-5	chr3	143035982	143035982	C	T	exonic	U2SURP	48	2	0.04	Nonsense_Mutation	p.Q215X	Non-Targeted Panel Gene	Adult HGBCL, NOS
BCL-5	chr10	102157217	102157217	C	T	exonic	NOLC1	71	3	0.0405	Nonsense_Mutation	p.Q69X	Non-Targeted Panel Gene	Adult HGBCL, NOS
BCL-5	chr4	39913708	39913708	G	A	exonic	PDS5A	47	2	0.0408	Nonsense_Mutation	p.R299X	Non-Targeted Panel Gene	Adult HGBCL, NOS
BCL-5	chr15	55840711	55840711	G	A	exonic	NEDD4	70	3	0.0411	Nonsense_Mutation	p.Q619X	Non-Targeted Panel Gene	Adult HGBCL, NOS
BCL-5	chr7	50612766	50612766	C	T	exonic	GRB10	46	2	0.0417	Nonsense_Mutation	p.W390X	Non-Targeted Panel Gene	Adult HGBCL, NOS
BCL-5	chr2	47130254	47130254	G	A	exonic	STPG4	64	3	0.0448	Nonsense_Mutation	p.Q136X	Non-Targeted Panel Gene	Adult HGBCL, NOS
BCL-5	chr8	41941039	41941039	G	A	exonic	KAT6A	41	2	0.0465	Nonsense_Mutation	p.R948X	Non-Targeted Panel Gene	Adult HGBCL, NOS
BCL-5	chr14	31144938	31144938	G	A	exonic	HECTD1	61	3	0.0469	Nonsense_Mutation	p.R834X	Non-Targeted Panel Gene	Adult HGBCL, NOS
BCL-5	chr17	37710565	37710565	G	A	exonic	HNF1B	60	3	0.0476	Nonsense_Mutation	p.Q356X	Non-Targeted Panel Gene	Adult HGBCL, NOS
BCL-5	chr9	129900502	129900502	G	A	exonic	FNBP1	39	2	0.0488	Nonsense_Mutation	p.Q492X	Non-Targeted Panel Gene	Adult HGBCL, NOS
BCL-5	chr1	6620133	6620133	C	T	exonic	PHF13	39	2	0.0488	Nonsense_Mutation	p.Q158X	Non-Targeted Panel Gene	Adult HGBCL, NOS
BCL-5	chrX	119837999	119837999	G	A	exonic	UPF3B	39	2	0.0488	Nonsense_Mutation	p.R354X	Non-Targeted Panel Gene	Adult HGBCL, NOS
BCL-5	chr10	97673739	97673739	G	A	exonic	PI4K2A	58	3	0.0492	Nonsense_Mutation	p.W479X	Non-Targeted Panel Gene	Adult HGBCL, NOS
BCL-5	chr8	74350483	74350483	C	T	exonic	GDAP1	38	2	0.05	Nonsense_Mutation	p.Q8X	Non-Targeted Panel Gene	Adult HGBCL, NOS
BCL-5	chr1	175077605	175077605	C	T	exonic	TNN	38	2	0.05	Nonsense_Mutation	p.Q63X	Non-Targeted Panel Gene	Adult HGBCL, NOS
BCL-5	chr3	141066708	141066708	C	T	exonic	SPSB4	56	3	0.0508	Nonsense_Mutation	p.R202X	Non-Targeted Panel Gene	Adult HGBCL, NOS
BCL-5	chr2	99412793	99412793	G	A	exonic	REV1	55	3	0.0517	Nonsense_Mutation	p.R181X	Non-Targeted Panel Gene	Adult HGBCL, NOS
BCL-5	chr9	136944038	136944038	G	A	exonic	FBXW5	36	2	0.0526	Nonsense_Mutation	p.Q16X	Non-Targeted Panel Gene	Adult HGBCL, NOS
BCL-5	chr13	33112899	33112899	G	A	exonic	STARD13	35	2	0.0526	Nonsense_Mutation	p.Q737X	Non-Targeted Panel Gene	Adult HGBCL, NOS
BCL-5	chr20	25278379	25278379	C	T	exonic	PYGB	53	3	0.0536	Nonsense_Mutation	p.Q306X	Non-Targeted Panel Gene	Adult HGBCL, NOS
BCL-5	chr14	103102450	103102450	C	T	exonic	EXOC3L4	35	2	0.0541	Nonsense_Mutation	p.Q243X	Non-Targeted Panel Gene	Adult HGBCL, NOS
BCL-5	chr11	14488581	14488581	G	A	exonic	COPB1	51	3	0.0556	Nonsense_Mutation	p.R204X	Non-Targeted Panel Gene	Adult HGBCL, NOS
BCL-5	chr19	46494381	46494381	C	T	exonic	PNMA8B	34	2	0.0556	Nonsense_Mutation	p.W362X	Non-Targeted Panel Gene	Adult HGBCL, NOS
BCL-5	chr5	140194472	140194472	C	T	exonic	CYSTM1	30	2	0.0625	Nonsense_Mutation	p.Q3X	Non-Targeted Panel Gene	Adult HGBCL, NOS
BCL-5	chrX	119096574	119096574	G	A	exonic	KIAA1210	59	4	0.0635	Nonsense_Mutation	p.Q396X	Non-Targeted Panel Gene	Adult HGBCL, NOS
BCL-5	chr3	9053134	9053134	G	A	exonic	SRGAP3	59	4	0.0635	Nonsense_Mutation	p.R406X	Non-Targeted Panel Gene	Adult HGBCL, NOS
BCL-5	chr1	247301033	247301033	C	T	exonic	ZNF496	58	4	0.0645	Nonsense_Mutation	p.W417X	Non-Targeted Panel Gene	Adult HGBCL, NOS
BCL-5	chr1	36394057	36394057	G	A	exonic	LSM10	43	3	0.0652	Nonsense_Mutation	p.Q25X	Non-Targeted Panel Gene	Adult HGBCL, NOS
BCL-5	chrX	13623902	13623902	G	A	exonic	EGFL6	54	4	0.069	Nonsense_Mutation	p.W421X	Non-Targeted Panel Gene	Adult HGBCL, NOS
BCL-5	chr22	18088023	18088023	G	A	exonic	PEX26	60	5	0.0769	Nonsense_Mutation	p.W289X	Non-Targeted Panel Gene	Adult HGBCL, NOS
BCL-5	chr5	135834860	135834860	C	T	exonic	SLC25A48	33	3	0.0833	Nonsense_Mutation	p.Q5X	Non-Targeted Panel Gene	Adult HGBCL, NOS
BCL-5	chr20	41358818	41358818	C	A	exonic	LPIN3	62	6	0.0882	Nonsense_Mutation	p.C837X	Non-Targeted Panel Gene	Adult HGBCL, NOS
BCL-5	chr16	66912820	66912820	G	A	exonic	CDH16	29	3	0.0938	Nonsense_Mutation	p.Q376X	Non-Targeted Panel Gene	Adult HGBCL, NOS
BCL-5	chr3	51935665	51935665	G	A	exonic	RRP9	29	3	0.0938	Nonsense_Mutation	p.Q255X	Non-Targeted Panel Gene	Adult HGBCL, NOS
BCL-5	chr15	74415801	74415801	C	T	exonic	SEMA7A	17	2	0.1053	Nonsense_Mutation	p.W329X	Non-Targeted Panel Gene	Adult HGBCL, NOS
BCL-5	chr2	53921098	53921098	G	A	exonic	PSME4	16	2	0.1111	Nonsense_Mutation	p.R685X	Non-Targeted Panel Gene	Adult HGBCL, NOS
BCL-5	chrX	11664418	11664418	C	T	exonic	ARHGAP6	13	2	0.1333	Nonsense_Mutation	p.W137X	Non-Targeted Panel Gene	Adult HGBCL, NOS
BCL-5	chr10	77820015	77820015	G	A	exonic	DLG5	11	2	0.1538	Nonsense_Mutation	p.Q1136X	Non-Targeted Panel Gene	Adult HGBCL, NOS
BCL-5	chr6	135195934	135195934	C	T	exonic	MYB	45	26	0.3662	Nonsense_Mutation	p.Q379X	Non-Targeted Panel Gene	Adult HGBCL, NOS
BCL-5	chr1	1923370	1923370	C	A	exonic	CFAP74	18	11	0.3793	Nonsense_Mutation	p.E1507X	Non-Targeted Panel Gene	Adult HGBCL, NOS
BCL-5	chr3	4787962	4787962	C	T	exonic	ITPR1	38	24	0.3871	Nonsense_Mutation	p.R2196X	Non-Targeted Panel Gene	Adult HGBCL, NOS
BCL-5	chr19	42095655	42095655	G	A	exonic	POU2F2	20	17	0.4595	Nonsense_Mutation	p.Q266X	Non-Targeted Panel Gene	Adult HGBCL, NOS
BCL-5	chrX	119581527	119581527	G	T	exonic	UBE2A	15	65	0.8125	Nonsense_Mutation	p.E58X	Non-Targeted Panel Gene	Adult HGBCL, NOS
BCL-6	chr13	48362813	48362813	A	G	splicing	RB1	273	36	0.1165	Splice_Site	.	Targerted Panel Gene	Adult HGBCL, NOS
BCL-6	chr17	74354781	74354781	G	A	splicing	KIF19	35	2	0.0541	Splice_Site	.	Non-Targeted Panel Gene	Adult HGBCL, NOS
BCL-6	chr3	124347267	124347267	T	A	splicing	KALRN	19	2	0.0952	Splice_Site	.	Non-Targeted Panel Gene	Adult HGBCL, NOS
BCL-6	chr6	32934989	32934989	T	C	splicing	HLA-DMB	130	16	0.1096	Splice_Site	.	Non-Targeted Panel Gene	Adult HGBCL, NOS
BCL-6	chr1	196677668	196677668	G	A	splicing	CFH	24	3	0.1111	Splice_Site	.	Non-Targeted Panel Gene	Adult HGBCL, NOS
BCL-6	chr15	65334727	65334727	C	T	splicing	IGDCC3	34	5	0.1282	Splice_Site	.	Non-Targeted Panel Gene	Adult HGBCL, NOS
BCL-6	chr6	146733257	146733257	T	G	splicing	ADGB	13	17	0.5667	Splice_Site	.	Non-Targeted Panel Gene	Adult HGBCL, NOS
BCL-6	chr19	12016471	12016472	AT	-	exonic	ZNF433	53	8	0.1311	Frame_Shift_Del	p.Y97fs	Non-Targeted Panel Gene	Adult HGBCL, NOS
BCL-6	chr5	53485035	53485035	A	-	exonic	FST	109	18	0.1417	Frame_Shift_Del	p.K254fs	Non-Targeted Panel Gene	Adult HGBCL, NOS
BCL-6	chr9	93450479	93450501	CTTTTTCACCGGGA	-	exonic	FAM120AOS	56	15	0.2113	Frame_Shift_Del	p.A221fs	Non-Targeted Panel Gene	Adult HGBCL, NOS
BCL-6	chr17	42323043	42323045	CTT	-	exonic	STAT3	75	11	0.1279	In_Frame_Del	p.616_617del	Non-Targeted Panel Gene	Adult HGBCL, NOS
BCL-6	chr6	89868559	89868567	TTAGCTGTG	-	exonic	CASP8AP2	39	28	0.4179	In_Frame_Del	p.1757_1759del	Non-Targeted Panel Gene	Adult HGBCL, NOS
BCL-6	chr16	3736703	3736703	A	T	exonic	CREBBP	323	38	0.1053	Missense_Mutation	p.Y1503N	Targerted Panel Gene	Adult HGBCL, NOS
BCL-6	chr22	41177344	41177344	C	T	exonic	EP300	141	138	0.4946	Missense_Mutation	p.T1878I	Targerted Panel Gene	Adult HGBCL, NOS
BCL-6	chr7	148826483	148826483	T	C	exonic	EZH2	375	70	0.157	Missense_Mutation	p.D293G	Targerted Panel Gene	Adult HGBCL, NOS
BCL-6	chr1	186145551	186145551	G	T	exonic	HMCN1	165	37	0.1832	Missense_Mutation	p.R4805S	Targerted Panel Gene	Adult HGBCL, NOS

BCL-6	chr1	9720806	9720806	A	T	exonic	PIK3CD	130	22	0.1447	Missense_Mutation	p.D529V	Targerted Panel Gene	Adult HGBCL, NOS
BCL-6	chr1	9720791	9720791	A	C	exonic	PIK3CD	131	19	0.1267	Missense_Mutation	p.Y524S	Targerted Panel Gene	Adult HGBCL, NOS
BCL-6	chr1	183517783	183517783	C	T	exonic	SMG7	191	211	0.5249	Missense_Mutation	p.S92F	Targerted Panel Gene	Adult HGBCL, NOS
BCL-6	chrX	71393422	71393422	A	G	exonic	TAF1	177	185	0.5096	Missense_Mutation	p.E1057G	Targerted Panel Gene	Adult HGBCL, NOS
BCL-6	chr17	7673793	7673793	G	T	exonic	TP53	199	26	0.1156	Missense_Mutation	p.A237D	Targerted Panel Gene	Adult HGBCL, NOS
BCL-6	chr7	144397425	144397425	T	C	exonic	NOBOX	70	3	0.0411	Missense_Mutation	p.T631A	Non-Targeted Panel Gene	Adult HGBCL, NOS
BCL-6	chr22	41833355	41833355	G	A	exonic	SREBF2	41	2	0.0465	Missense_Mutation	p.D29N	Non-Targeted Panel Gene	Adult HGBCL, NOS
BCL-6	chr5	35921048	35921048	G	A	exonic	CAPSL	59	3	0.0484	Missense_Mutation	p.P25S	Non-Targeted Panel Gene	Adult HGBCL, NOS
BCL-6	chr6	30744189	30744189	C	T	exonic	IER3	172	9	0.0497	Missense_Mutation	p.R73H	Non-Targeted Panel Gene	Adult HGBCL, NOS
BCL-6	chr19	54933710	54933710	C	T	exonic	NLRP7	76	4	0.05	Missense_Mutation	p.S834N	Non-Targeted Panel Gene	Adult HGBCL, NOS
BCL-6	chr2	27132366	27132366	G	A	exonic	PREB	56	3	0.0508	Missense_Mutation	p.L264F	Non-Targeted Panel Gene	Adult HGBCL, NOS
BCL-6	chr8	85113084	85113084	G	C	exonic	LRRCC1	71	4	0.0533	Missense_Mutation	p.V177L	Non-Targeted Panel Gene	Adult HGBCL, NOS
BCL-6	chr10	91942568	91942568	G	A	exonic	BTAF1	53	3	0.0536	Missense_Mutation	p.G134S	Non-Targeted Panel Gene	Adult HGBCL, NOS
BCL-6	chr17	17512607	17512607	G	A	exonic	PEMT	69	4	0.0548	Missense_Mutation	p.T86I	Non-Targeted Panel Gene	Adult HGBCL, NOS
BCL-6	chr21	33511348	33511348	C	T	exonic	GART	94	6	0.06	Missense_Mutation	p.V740M	Non-Targeted Panel Gene	Adult HGBCL, NOS
BCL-6	chr10	62805783	62805783	C	T	exonic	ADO	77	5	0.061	Missense_Mutation	p.P242S	Non-Targeted Panel Gene	Adult HGBCL, NOS
BCL-6	chr14	63997039	63997039	T	A	exonic	SYNE2	46	3	0.0612	Missense_Mutation	p.S1011R	Non-Targeted Panel Gene	Adult HGBCL, NOS
BCL-6	chr19	42234617	42234617	C	T	exonic	GSK3A	76	5	0.0617	Missense_Mutation	p.R243H	Non-Targeted Panel Gene	Adult HGBCL, NOS
BCL-6	chr13	77263705	77263705	A	C	exonic	MYCBP2	60	4	0.0625	Missense_Mutation	p.L506V	Non-Targeted Panel Gene	Adult HGBCL, NOS
BCL-6	chr3	171167168	171167168	T	G	exonic	TNIK	75	5	0.0625	Missense_Mutation	p.Q292H	Non-Targeted Panel Gene	Adult HGBCL, NOS
BCL-6	chr5	180261698	180261698	C	T	exonic	MAPK9	29	2	0.0645	Missense_Mutation	p.G146S	Non-Targeted Panel Gene	Adult HGBCL, NOS
BCL-6	chr5	136992574	136992574	G	C	exonic	SPOCK1	115	8	0.065	Missense_Mutation	p.L206V	Non-Targeted Panel Gene	Adult HGBCL, NOS
BCL-6	chr19	45711734	45711734	G	A	exonic	FBXO46	43	3	0.0652	Missense_Mutation	p.P588S	Non-Targeted Panel Gene	Adult HGBCL, NOS
BCL-6	chrX	106694251	106694251	G	T	exonic	RNF128	113	8	0.0656	Missense_Mutation	p.E83D	Non-Targeted Panel Gene	Adult HGBCL, NOS
BCL-6	chr7	124032838	124032838	G	A	exonic	TMEM229A	42	3	0.0667	Missense_Mutation	p.R56C	Non-Targeted Panel Gene	Adult HGBCL, NOS
BCL-6	chr18	6966172	6966172	A	G	exonic	LAMA1	40	3	0.0698	Missense_Mutation	p.L2342P	Non-Targeted Panel Gene	Adult HGBCL, NOS
BCL-6	chr13	102738825	102738825	C	T	exonic	CCDC168	38	3	0.0732	Missense_Mutation	p.G3958R	Non-Targeted Panel Gene	Adult HGBCL, NOS
BCL-6	chr19	16506702	16506702	G	C	exonic	C19orf44	50	4	0.0741	Missense_Mutation	p.E359D	Non-Targeted Panel Gene	Adult HGBCL, NOS
BCL-6	chr20	62139894	62139894	C	T	exonic	PSMA7	50	4	0.0741	Missense_Mutation	p.D79N	Non-Targeted Panel Gene	Adult HGBCL, NOS
BCL-6	chr22	33384290	33384290	G	A	exonic	LARGE1	37	3	0.075	Missense_Mutation	p.L303F	Non-Targeted Panel Gene	Adult HGBCL, NOS
BCL-6	chr20	50891880	50891880	G	A	exonic	ADNP	36	3	0.0769	Missense_Mutation	p.P945L	Non-Targeted Panel Gene	Adult HGBCL, NOS
BCL-6	chr2	237372262	237372262	C	A	exonic	COL6A3	118	10	0.0775	Missense_Mutation	p.R1252L	Non-Targeted Panel Gene	Adult HGBCL, NOS
BCL-6	chr15	82151993	82151993	C	T	exonic	EFL1	35	3	0.0789	Missense_Mutation	p.D821N	Non-Targeted Panel Gene	Adult HGBCL, NOS
BCL-6	chr3	5188250	5188250	G	T	exonic	EDEM1	113	10	0.0813	Missense_Mutation	p.A149S	Non-Targeted Panel Gene	Adult HGBCL, NOS
BCL-6	chr7	31087667	31087667	A	G	exonic	ADCYAP1R1	172	16	0.0851	Missense_Mutation	p.I309V	Non-Targeted Panel Gene	Adult HGBCL, NOS
BCL-6	chr16	3966709	3966709	T	G	exonic	ADCY9	139	13	0.0855	Missense_Mutation	p.Q1043P	Non-Targeted Panel Gene	Adult HGBCL, NOS
BCL-6	chr10	101786510	101786510	C	T	exonic	MGEA5	32	3	0.0857	Missense_Mutation	p.E898K	Non-Targeted Panel Gene	Adult HGBCL, NOS
BCL-6	chrX	105908932	105908932	G	T	exonic	NRK	128	12	0.0857	Missense_Mutation	p.G431W	Non-Targeted Panel Gene	Adult HGBCL, NOS
BCL-6	chr17	18133301	18133301	G	C	exonic	MYO15A	84	8	0.087	Missense_Mutation	p.G1466A	Non-Targeted Panel Gene	Adult HGBCL, NOS
BCL-6	chr4	52079748	52079748	T	A	exonic	SPATA18	105	10	0.087	Missense_Mutation	p.V395E	Non-Targeted Panel Gene	Adult HGBCL, NOS
BCL-6	chr11	724816	724816	A	C	exonic	EPS8L2	132	13	0.0897	Missense_Mutation	p.D516A	Non-Targeted Panel Gene	Adult HGBCL, NOS
BCL-6	chr9	87971223	87971223	T	A	exonic	CDK20	140	14	0.0909	Missense_Mutation	p.Q114L	Non-Targeted Panel Gene	Adult HGBCL, NOS
BCL-6	chr2	74093617	74093617	G	A	exonic	TET3	70	7	0.0909	Missense_Mutation	p.C1073Y	Non-Targeted Panel Gene	Adult HGBCL, NOS
BCL-6	chr19	50276803	50276803	G	A	exonic	MYH14	49	5	0.0926	Missense_Mutation	p.E1243K	Non-Targeted Panel Gene	Adult HGBCL, NOS
BCL-6	chr19	47555588	47555588	T	C	exonic	ZNF541	127	13	0.0929	Missense_Mutation	p.E90G	Non-Targeted Panel Gene	Adult HGBCL, NOS
BCL-6	chrX	70880021	70880021	G	C	exonic	TEX11	37	4	0.0976	Missense_Mutation	p.N57K	Non-Targeted Panel Gene	Adult HGBCL, NOS
BCL-6	chr21	33553394	33553394	T	C	exonic	SON	165	18	0.0984	Missense_Mutation	p.V1388A	Non-Targeted Panel Gene	Adult HGBCL, NOS
BCL-6	chrX	12976299	12976299	T	C	exonic	TMSB4X	64	7	0.0986	Missense_Mutation	p.F13S	Non-Targeted Panel Gene	Adult HGBCL, NOS
BCL-6	chr1	247491683	247491683	G	T	exonic	OR2W5	105	12	0.1026	Missense_Mutation	p.A186S	Non-Targeted Panel Gene	Adult HGBCL, NOS
BCL-6	chr6	143942311	143942311	T	C	exonic	PLAGL1	77	9	0.1047	Missense_Mutation	p.K169E	Non-Targeted Panel Gene	Adult HGBCL, NOS
BCL-6	chr12	131728321	131728321	C	T	exonic	SFSWAP	94	11	0.1048	Missense_Mutation	p.P325L	Non-Targeted Panel Gene	Adult HGBCL, NOS
BCL-6	chr3	187043186	187043186	T	A	exonic	ST6GAL1	143	17	0.1062	Missense_Mutation	p.N161K	Non-Targeted Panel Gene	Adult HGBCL, NOS
BCL-6	chrX	106205994	106205994	C	A	exonic	MUM1L1	42	5	0.1064	Missense_Mutation	p.Q188K	Non-Targeted Panel Gene	Adult HGBCL, NOS
BCL-6	chr2	191061759	191061759	T	G	exonic	STAT4	84	10	0.1064	Missense_Mutation	p.K335T	Non-Targeted Panel Gene	Adult HGBCL, NOS
BCL-6	chr5	123090039	123090039	G	T	exonic	PRDM6	25	3	0.1071	Missense_Mutation	p.G9C	Non-Targeted Panel Gene	Adult HGBCL, NOS
BCL-6	chr19	37699760	37699760	C	G	exonic	ZNF607	25	3	0.1071	Missense_Mutation	p.S124T	Non-Targeted Panel Gene	Adult HGBCL, NOS
BCL-6	chrX	1466045	1466045	T	A	exonic	P2RY8	90	11	0.1089	Missense_Mutation	p.I172F	Non-Targeted Panel Gene	Adult HGBCL, NOS
BCL-6	chr4	161385815	161385815	T	A	exonic	FSTL5	96	12	0.1111	Missense_Mutation	p.N826Y	Non-Targeted Panel Gene	Adult HGBCL, NOS
BCL-6	chr15	74801769	74801769	T	A	exonic	CSK	125	16	0.1135	Missense_Mutation	p.L321Q	Non-Targeted Panel Gene	Adult HGBCL, NOS
BCL-6	chr5	41842737	41842737	A	C	exonic	OXCT1	45	6	0.1176	Missense_Mutation	p.I203M	Non-Targeted Panel Gene	Adult HGBCL, NOS
BCL-6	chr12	7064343	7064343	C	G	exonic	C1S	22	3	0.12	Missense_Mutation	p.C156W	Non-Targeted Panel Gene	Adult HGBCL, NOS
BCL-6	chr12	7064351	7064351	C	G	exonic	C1S	22	3	0.12	Missense_Mutation	p.P159R	Non-Targeted Panel Gene	Adult HGBCL, NOS
BCL-6	chr1	11020574	11020574	C	T	exonic	TARDBP	73	10	0.1205	Missense_Mutation	p.A230V	Non-Targeted Panel Gene	Adult HGBCL, NOS
BCL-6	chrX	56564925	56564925	G	A	exonic	UBQLN2	108	15	0.122	Missense_Mutation	p.G351E	Non-Targeted Panel Gene	Adult HGBCL, NOS
BCL-6	chr10	49324818	49324818	A	G	exonic	C10orf71	105	15	0.125	Missense_Mutation	p.K758R	Non-Targeted Panel Gene	Adult HGBCL, NOS
BCL-6	chrX	153673252	153673252	C	T	exonic	PNCK	14	2	0.125	Missense_Mutation	p.G7S	Non-Targeted Panel Gene	Adult HGBCL, NOS
BCL-6	chrX	123253525	123253525	T	G	exonic	GRIA3	118	17	0.1259	Missense_Mutation	p.L164R	Non-Targeted Panel Gene	Adult HGBCL, NOS
BCL-6	chr2	118994503	118994503	G	A	exonic	MARCO	89	13	0.1275	Missense_Mutation	p.V516M	Non-Targeted Panel Gene	Adult HGBCL, NOS
BCL-6	chr19	45064497	45064497	C	T	exonic	CLASRP	20	3	0.1304	Missense_Mutation	p.R426C	Non-Targeted Panel Gene	Adult HGBCL, NOS
BCL-6	chr1	12776106	12776106	A	G	exonic	PRAMEF12	40	6	0.1304	Missense_Mutation	p.D284G	Non-Targeted Panel Gene	Adult HGBCL, NOS
BCL-6	chr13	36819779	36819779	G	C	exonic	RFXAP	57	9	0.1364	Missense_Mutation	p.C141S	Non-Targeted Panel Gene	Adult HGBCL, NOS
BCL-6	chr15	41836198	41836198	C	T	exonic	JMJD7;JMJD7-PLA2	61	10	0.1408	Missense_Mutation	p.H194Y	Non-Targeted Panel Gene	Adult HGBCL, NOS
BCL-6	chr17	9022800	9022800	G	A	exonic	NTN1	96	16	0.1429	Missense_Mutation	p.E143K	Non-Targeted Panel Gene	Adult HGBCL, NOS
BCL-6	chr1	179341069	179341069	A	G	exonic	SOAT1	47	8	0.1455	Missense_Mutation	p.K180R	Non-Targeted Panel Gene	Adult HGBCL, NOS
BCL-6	chr3	186554938	186554938	G	A	exonic	TBCCD1	52	9	0.1475	Missense_Mutation	p.R336C	Non-Targeted Panel Gene	Adult HGBCL, NOS
BCL-6	chr1	40838369	40838369	G	A	exonic	KCNQ4	72	13	0.1512	Missense_Mutation	p.R645H	Non-Targeted Panel Gene	Adult HGBCL, NOS
BCL-6	chr11	66692628	66692628	G	A	exonic	SPTBN2	39	7	0.1522	Missense_Mutation	p.R1700C	Non-Targeted Panel Gene	Adult HGBCL, NOS
BCL-6	chr17	8046923	8046923	T	C	exonic	ALOX15B	115	21	0.1544	Missense_Mutation	p.I435T	Non-Targeted Panel Gene	Adult HGBCL, NOS
BCL-6	chr15	41836171	41836171	C	A	exonic	JMJD7;JMJD7-PLA2	59	11	0.1571	Missense_Mutation	p.L185I	Non-Targeted Panel Gene	Adult HGBCL, NOS
BCL-6	chr19	45351350	45351350	C	T	exonic	KLC3	21	4	0.16	Missense_Mutation	p.P503L	Non-Targeted Panel Gene	Adult HGBCL, NOS
BCL-6	chr3	5188061	5188061	G	A	exonic	EDEM1	31	6	0.1622	Missense_Mutation	p.A86T	Non-Targeted Panel Gene	Adult HGBCL, NOS
BCL-6	chr2	202036143	202036143	G	T	exonic	FZD7	86	17	0.165	Missense_Mutation	p.W499L	Non-Targeted Panel Gene	Adult HGBCL, NOS

BCL-6	chr15	80921076	80921076	T	C	exonic	CEMIP	60	12	0.1667	Missense_Mutation	p.I683T	Non-Targeted Panel Gene	Adult HGBCL, NOS
BCL-6	chr22	50177515	50177515	C	T	exonic	PANX2	92	19	0.1712	Missense_Mutation	p.A268V	Non-Targeted Panel Gene	Adult HGBCL, NOS
BCL-6	chr3	131162490	131162490	A	G	exonic	NEK11	42	9	0.1765	Missense_Mutation	p.K391E	Non-Targeted Panel Gene	Adult HGBCL, NOS
BCL-6	chr3	113938104	113938104	A	G	exonic	GRAMD1C	18	4	0.1818	Missense_Mutation	p.E551G	Non-Targeted Panel Gene	Adult HGBCL, NOS
BCL-6	chr19	47679519	47679519	A	T	exonic	BICRA	32	8	0.2	Missense_Mutation	p.T117S	Non-Targeted Panel Gene	Adult HGBCL, NOS
BCL-6	chr19	36449531	36449531	T	G	exonic	ZNF566	28	7	0.2	Missense_Mutation	p.T235P	Non-Targeted Panel Gene	Adult HGBCL, NOS
BCL-6	chr19	55481222	55481222	C	T	exonic	ZNF628	24	6	0.2	Missense_Mutation	p.A10V	Non-Targeted Panel Gene	Adult HGBCL, NOS
BCL-6	chr6	31581831	31581831	T	C	exonic	LTB	74	23	0.2371	Missense_Mutation	p.Q64R	Non-Targeted Panel Gene	Adult HGBCL, NOS
BCL-6	chr2	115689700	115689700	C	T	exonic	DPP10	9	3	0.25	Missense_Mutation	p.S102L	Non-Targeted Panel Gene	Adult HGBCL, NOS
BCL-6	chr10	73470765	73470765	T	G	exonic	PPP3CB	17	7	0.28	Missense_Mutation	p.K216Q	Non-Targeted Panel Gene	Adult HGBCL, NOS
BCL-6	chr3	183981744	183981744	A	G	exonic	ABCC5	26	15	0.3659	Missense_Mutation	p.F377S	Non-Targeted Panel Gene	Adult HGBCL, NOS
BCL-6	chr10	102854297	102854297	C	T	exonic	BORCS7	111	65	0.3693	Missense_Mutation	p.T4I	Non-Targeted Panel Gene	Adult HGBCL, NOS
BCL-6	chr14	50188639	50188639	G	C	exonic	SOS2	15	9	0.375	Missense_Mutation	p.P191R	Non-Targeted Panel Gene	Adult HGBCL, NOS
BCL-6	chr10	113089518	113089518	T	C	exonic	TCF7L2	67	43	0.3909	Missense_Mutation	p.V201A	Non-Targeted Panel Gene	Adult HGBCL, NOS
BCL-6	chr2	84813079	84813079	C	A	exonic	DNAH6	44	31	0.4133	Missense_Mutation	p.P3983T	Non-Targeted Panel Gene	Adult HGBCL, NOS
BCL-6	chr5	176578375	176578375	T	C	exonic	CDHR2	43	31	0.4189	Missense_Mutation	p.F529L	Non-Targeted Panel Gene	Adult HGBCL, NOS
BCL-6	chr2	25252079	25252079	C	T	exonic	DNMT3A	9	7	0.4375	Missense_Mutation	p.A6T	Non-Targeted Panel Gene	Adult HGBCL, NOS
BCL-6	chr8	31640695	31640695	G	C	exonic	NRG1	17	14	0.4375	Missense_Mutation	p.K237N	Non-Targeted Panel Gene	Adult HGBCL, NOS
BCL-6	chr1	155291918	155291918	G	A	exonic	PKLR	29	23	0.4423	Missense_Mutation	p.R455W	Non-Targeted Panel Gene	Adult HGBCL, NOS
BCL-6	chr4	151103009	151103009	C	T	exonic	RPS3A	12	10	0.4545	Missense_Mutation	p.R165W	Non-Targeted Panel Gene	Adult HGBCL, NOS
BCL-6	chr19	14056499	14056499	A	G	exonic	PALM3	56	47	0.4563	Missense_Mutation	p.L95P	Non-Targeted Panel Gene	Adult HGBCL, NOS
BCL-6	chr3	142555928	142555928	T	C	exonic	ATR	33	28	0.459	Missense_Mutation	p.K764E	Non-Targeted Panel Gene	Adult HGBCL, NOS
BCL-6	chr1	75761161	75761161	A	G	exonic	ACADM	61	52	0.4602	Missense_Mutation	p.K362E	Non-Targeted Panel Gene	Adult HGBCL, NOS
BCL-6	chr11	126562852	126562852	A	T	exonic	KIRREL3	43	37	0.4625	Missense_Mutation	p.F39Y	Non-Targeted Panel Gene	Adult HGBCL, NOS
BCL-6	chr5	148827322	148827322	C	T	exonic	ADRB2	89	77	0.4639	Missense_Mutation	p.T164I	Non-Targeted Panel Gene	Adult HGBCL, NOS
BCL-6	chr16	18834296	18834296	A	G	exonic	SMG1	86	81	0.4821	Missense_Mutation	p.C2825R	Non-Targeted Panel Gene	Adult HGBCL, NOS
BCL-6	chr12	80708053	80708053	G	T	exonic	MYF6	49	46	0.4842	Missense_Mutation	p.A112S	Non-Targeted Panel Gene	Adult HGBCL, NOS
BCL-6	chr3	44594914	44594914	C	T	exonic	ZNF660	17	16	0.4848	Missense_Mutation	p.R241C	Non-Targeted Panel Gene	Adult HGBCL, NOS
BCL-6	chr8	76983431	76983431	A	G	exonic	PEX2	53	50	0.4854	Missense_Mutation	p.W250R	Non-Targeted Panel Gene	Adult HGBCL, NOS
BCL-6	chr17	53823844	53823844	G	C	exonic	KIF2B	92	88	0.4862	Missense_Mutation	p.D271H	Non-Targeted Panel Gene	Adult HGBCL, NOS
BCL-6	chr12	98533747	98533747	C	G	exonic	TMPO	25	24	0.4898	Missense_Mutation	p.S497C	Non-Targeted Panel Gene	Adult HGBCL, NOS
BCL-6	chr2	26461006	26461006	C	T	exonic	OTOF	34	33	0.4925	Missense_Mutation	p.R1853Q	Non-Targeted Panel Gene	Adult HGBCL, NOS
BCL-6	chrX	47915200	47915200	C	T	exonic	ZNF81	16	16	0.5	Missense_Mutation	p.S185L	Non-Targeted Panel Gene	Adult HGBCL, NOS
BCL-6	chr7	44516100	44516100	A	T	exonic	NPC1L1	23	24	0.5106	Missense_Mutation	p.I1233N	Non-Targeted Panel Gene	Adult HGBCL, NOS
BCL-6	chr8	10609543	10609543	C	G	exonic	RP1L1	37	40	0.5195	Missense_Mutation	p.V1519L	Non-Targeted Panel Gene	Adult HGBCL, NOS
BCL-6	chr17	39727923	39727923	C	A	exonic	ERBB2	61	66	0.5197	Missense_Mutation	p.A1201D	Non-Targeted Panel Gene	Adult HGBCL, NOS
BCL-6	chr1	41580864	41580864	C	G	exonic	HIVEP3	54	65	0.5462	Missense_Mutation	p.D1312H	Non-Targeted Panel Gene	Adult HGBCL, NOS
BCL-6	chr12	32619725	32619725	C	A	exonic	FGD4	24	31	0.5636	Missense_Mutation	p.P112T	Non-Targeted Panel Gene	Adult HGBCL, NOS
BCL-6	chr2	178605503	178605503	T	G	exonic	TTN	37	50	0.5747	Missense_Mutation	p.Q17931P	Non-Targeted Panel Gene	Adult HGBCL, NOS
BCL-6	chr19	44908751	44908751	G	A	exonic	APOE	13	20	0.6061	Missense_Mutation	p.R152Q	Non-Targeted Panel Gene	Adult HGBCL, NOS
BCL-6	chr5	1293652	1293652	G	A	exonic	TERT	11	18	0.6207	Missense_Mutation	p.H412Y	Non-Targeted Panel Gene	Adult HGBCL, NOS
BCL-6	chr12	49026348	49026348	A	C	exonic	KMT2D	273	32	0.1049	Nonsense_Mutation	p.Y5206X	Targerted Panel Gene	Adult HGBCL, NOS
BCL-6	chr1	236686780	236686780	G	A	exonic	ACTN2	48	2	0.04	Nonsense_Mutation	p.W36X	Non-Targeted Panel Gene	Adult HGBCL, NOS
BCL-6	chr14	90292755	90292755	C	T	exonic	NRDE2	48	2	0.04	Nonsense_Mutation	p.W595X	Non-Targeted Panel Gene	Adult HGBCL, NOS
BCL-6	chr16	31466417	31466417	C	A	exonic	ARMC5	43	2	0.0444	Nonsense_Mutation	p.S874X	Non-Targeted Panel Gene	Adult HGBCL, NOS
BCL-6	chr4	122917696	122917696	G	A	exonic	NUDT6	62	4	0.0606	Nonsense_Mutation	p.Q83X	Non-Targeted Panel Gene	Adult HGBCL, NOS
BCL-6	chr2	152670341	152670341	A	-	exonic	PRPF40A	75	7	0.0854	Nonsense_Mutation	p.L507X	Non-Targeted Panel Gene	Adult HGBCL, NOS
BCL-6	chr5	154793619	154793619	T	A	exonic	LARP1	107	11	0.0932	Nonsense_Mutation	p.L178X	Non-Targeted Panel Gene	Adult HGBCL, NOS
BCL-6	chr4	124670759	124670759	C	A	exonic	ANKRD50	43	6	0.1224	Nonsense_Mutation	p.E840X	Non-Targeted Panel Gene	Adult HGBCL, NOS
BCL-6	chr16	7518186	7518186	C	T	exonic	RBFOX1	62	9	0.1268	Nonsense_Mutation	p.Q23X	Non-Targeted Panel Gene	Adult HGBCL, NOS
BCL-6	chr9	132298287	132298287	G	C	exonic	SETX	39	6	0.1333	Nonsense_Mutation	p.Y1858X	Non-Targeted Panel Gene	Adult HGBCL, NOS
BCL-6	chr20	25478930	25478930	G	A	exonic	NINL	22	18	0.45	Nonsense_Mutation	p.Q732X	Non-Targeted Panel Gene	Adult HGBCL, NOS
BCL-6	chr1	35008889	35008889	G	A	exonic	ZMYM6	51	48	0.4848	Nonsense_Mutation	p.R510X	Non-Targeted Panel Gene	Adult HGBCL, NOS
BCL-71	chr13	35142379	35142379	T	C	splicing	NBEA	51	9	0.15	Splice_Site	.	Targerted Panel Gene	Adult HGBCL, NOS
BCL-71	chr1	3683182	3683182	T	G	splicing	TP73	46	12	0.2069	Splice_Site	.	Targerted Panel Gene	Adult HGBCL, NOS
BCL-71	chr12	122022184	122022184	G	A	splicing	BCL7A	66	18	0.2143	Splice_Site	.	Targerted Panel Gene	Adult HGBCL, NOS
BCL-71	chr2	19975528	19975528	A	C	splicing	WDR35	45	9	0.1667	Splice_Site	.	Non-Targeted Panel Gene	Adult HGBCL, NOS
BCL-71	chr15	82709592	82709592	A	G	splicing	AP3B2	79	16	0.1684	Splice_Site	.	Non-Targeted Panel Gene	Adult HGBCL, NOS
BCL-71	chr6	32940752	32940752	C	G	splicing	HLA-DMB	47	16	0.254	Splice_Site	.	Non-Targeted Panel Gene	Adult HGBCL, NOS
BCL-71	chr19	9967345	9967345	-	C	splicing	COL5A3	23	33	0.5893	Splice_Site	.	Non-Targeted Panel Gene	Adult HGBCL, NOS
BCL-71	chr16	3769320	3769321	CT	-	exonic	CREBBP	54	6	0.1	Frame_Shift_Del	p.R971fs	Targerted Panel Gene	Adult HGBCL, NOS
BCL-71	chr17	7673253	7673253	A	-	exonic	TP53	52	8	0.1333	Frame_Shift_Del	p.L297fs	Targerted Panel Gene	Adult HGBCL, NOS
BCL-71	chr4	120060196	120060197	TT	-	exonic	MAD2L1	57	5	0.0806	Frame_Shift_Del	p.E180fs	Non-Targeted Panel Gene	Adult HGBCL, NOS
BCL-71	chr2	47810338	47810339	TC	-	exonic	FBXO11	47	7	0.1296	Frame_Shift_Del	p.R688fs	Targerted Panel Gene	Adult HGBCL, NOS
BCL-71	chr5	177267593	177267593	T	-	exonic	NSD1	47	7	0.1296	Frame_Shift_Del	p.P1457fs	Non-Targeted Panel Gene	Adult HGBCL, NOS
BCL-71	chr1	2645308	2645311	TGTC	-	exonic	TTC34	30	23	0.434	Frame_Shift_Del	p.D314fs	Non-Targeted Panel Gene	Adult HGBCL, NOS
BCL-71	chr8	144261207	144261208	CT	-	exonic	MROH1	51	54	0.5143	Frame_Shift_Del	p.L1589fs	Non-Targeted Panel Gene	Adult HGBCL, NOS
BCL-71	chr17	4672277	4672279	TCC	-	exonic	PELP1	32	19	0.3725	In_Frame_Del	p.757_758del	Non-Targeted Panel Gene	Adult HGBCL, NOS
BCL-71	chr4	55965813	55965815	AAG	-	exonic	CEP135	27	21	0.4375	In_Frame_Del	p.333_334del	Non-Targeted Panel Gene	Adult HGBCL, NOS
BCL-71	chr7	2945821	2945821	A	G	exonic	CARD11	73	6	0.0759	Missense_Mutation	p.V119A	Targerted Panel Gene	Adult HGBCL, NOS
BCL-71	chr7	2945834	2945834	A	G	exonic	CARD11	70	6	0.0789	Missense_Mutation	p.F115L	Targerted Panel Gene	Adult HGBCL, NOS
BCL-71	chr7	100956002	100956002	C	A	exonic	MUC3A	153	15	0.0893	Missense_Mutation	p.T1408K	Targerted Panel Gene	Adult HGBCL, NOS
BCL-71	chr7	82965997	82965997	A	G	exonic	PCLO	87	10	0.1031	Missense_Mutation	p.L1264S	Targerted Panel Gene	Adult HGBCL, NOS
BCL-71	chr8	112921679	112921679	T	A	exonic	CSMD3	81	13	0.1383	Missense_Mutation	p.Q487H	Targerted Panel Gene	Adult HGBCL, NOS
BCL-71	chr3	77632577	77632577	C	G	exonic	ROBO2	65	11	0.1447	Missense_Mutation	p.S1292R	Targerted Panel Gene	Adult HGBCL, NOS
BCL-71	chr19	18857503	18857503	G	A	exonic	UPF1	59	10	0.1449	Missense_Mutation	p.E718K	Targerted Panel Gene	Adult HGBCL, NOS
BCL-71	chr6	27838085	27838085	C	G	exonic	HIST1H2AK	55	11	0.1667	Missense_Mutation	p.Q85H	Targerted Panel Gene	Adult HGBCL, NOS
BCL-71	chr18	63318485	63318485	G	T	exonic	BCL2	79	17	0.1771	Missense_Mutation	p.A61E	Targerted Panel Gene	Adult HGBCL, NOS
BCL-71	chr1	186174537	186174537	A	C	exonic	HMCN1	46	10	0.1786	Missense_Mutation	p.T5280P	Targerted Panel Gene	Adult HGBCL, NOS
BCL-71	chr4	125434406	125434406	T	C	exonic	FAT4	57	13	0.1857	Missense_Mutation	p.S2394P	Targerted Panel Gene	Adult HGBCL, NOS
BCL-71	chr6	26273142	26273142	C	T	exonic	HIST1H2BI	55	13	0.1912	Missense_Mutation	p.S56L	Targerted Panel Gene	Adult HGBCL, NOS
BCL-71	chr7	2944358	2944358	A	C	exonic	CARD11	63	15	0.1923	Missense_Mutation	p.Y180D	Targerted Panel Gene	Adult HGBCL, NOS

BCL-71	chr7	147639234	147639234	A	G	exonic	CNTNAP2	63	15	0.1923	Missense_Mutation	p.T676A	Targerted Panel Gene	Adult HGBCL, NOS
BCL-71	chr1	23559310	23559310	G	T	exonic	ID3	66	16	0.1951	Missense_Mutation	p.S39R	Targerted Panel Gene	Adult HGBCL, NOS
BCL-71	chr18	63318093	63318093	T	G	exonic	BCL2	57	15	0.2083	Missense_Mutation	p.N192H	Targerted Panel Gene	Adult HGBCL, NOS
BCL-71	chr18	63318437	63318437	G	C	exonic	BCL2	71	19	0.2111	Missense_Mutation	p.A77G	Targerted Panel Gene	Adult HGBCL, NOS
BCL-71	chr12	122022182	122022182	T	C	exonic	BCL7A	65	18	0.2169	Missense_Mutation	p.W31R	Targerted Panel Gene	Adult HGBCL, NOS
BCL-71	chr18	63318657	63318657	C	T	exonic	BCL2	72	21	0.2258	Missense_Mutation	p.A4T	Targerted Panel Gene	Adult HGBCL, NOS
BCL-71	chr19	55661172	55661172	C	T	exonic	U2AF2	48	16	0.25	Missense_Mutation	p.P157S	Targerted Panel Gene	Adult HGBCL, NOS
BCL-71	chr8	112573615	112573615	A	C	exonic	CSMD3	61	35	0.3646	Missense_Mutation	p.F1270V	Targerted Panel Gene	Adult HGBCL, NOS
BCL-71	chr14	23522257	23522257	A	T	exonic	ZFHX2	33	38	0.5352	Missense_Mutation	p.F2475Y	Targerted Panel Gene	Adult HGBCL, NOS
BCL-71	chr17	63490960	63490960	T	C	exonic	ACE	14	39	0.7358	Missense_Mutation	p.M883T	Targerted Panel Gene	Adult HGBCL, NOS
BCL-71	chr17	7511410	7511410	A	G	exonic	POLR2A	56	3	0.0508	Missense_Mutation	p.Y1308C	Non-Targeted Panel Gene	Adult HGBCL, NOS
BCL-71	chr6	145926284	145926284	T	A	exonic	SHPRH	53	3	0.0536	Missense_Mutation	p.T1081S	Non-Targeted Panel Gene	Adult HGBCL, NOS
BCL-71	chr2	17931700	17931700	C	T	exonic	KCNS3	65	5	0.0714	Missense_Mutation	p.A231V	Non-Targeted Panel Gene	Adult HGBCL, NOS
BCL-71	chr15	29123389	29123389	G	A	exonic	FAM189A1	61	5	0.0758	Missense_Mutation	p.R524C	Non-Targeted Panel Gene	Adult HGBCL, NOS
BCL-71	chr7	123469340	123469340	A	C	exonic	IQUB	71	7	0.0897	Missense_Mutation	p.I485M	Non-Targeted Panel Gene	Adult HGBCL, NOS
BCL-71	chr4	139344268	139344268	G	A	exonic	NAA15	63	8	0.1127	Missense_Mutation	p.R207K	Non-Targeted Panel Gene	Adult HGBCL, NOS
BCL-71	chr17	47831474	47831474	A	G	exonic	MRPL10	59	8	0.1194	Missense_Mutation	p.L13P	Non-Targeted Panel Gene	Adult HGBCL, NOS
BCL-71	chr8	76705209	76705209	C	T	exonic	ZFHX4	81	11	0.1196	Missense_Mutation	p.S374F	Non-Targeted Panel Gene	Adult HGBCL, NOS
BCL-71	chr16	12902068	12902068	C	T	exonic	SHISA9	66	9	0.12	Missense_Mutation	p.R2C	Non-Targeted Panel Gene	Adult HGBCL, NOS
BCL-71	chr4	88383360	88383360	G	T	exonic	HERC6	51	7	0.1207	Missense_Mutation	p.K113N	Non-Targeted Panel Gene	Adult HGBCL, NOS
BCL-71	chr4	3215191	3215191	A	G	exonic	HTT	55	8	0.127	Missense_Mutation	p.E2345G	Non-Targeted Panel Gene	Adult HGBCL, NOS
BCL-71	chr2	47813823	47813823	C	T	exonic	FBXO11	60	9	0.1304	Missense_Mutation	p.G600E	Non-Targeted Panel Gene	Adult HGBCL, NOS
BCL-71	chr16	8905315	8905315	T	C	exonic	USP7	60	9	0.1304	Missense_Mutation	p.D383G	Non-Targeted Panel Gene	Adult HGBCL, NOS
BCL-71	chr11	251558	251558	C	G	exonic	PSMD13	66	10	0.1316	Missense_Mutation	p.R284G	Non-Targeted Panel Gene	Adult HGBCL, NOS
BCL-71	chr16	67876505	67876505	A	G	exonic	EDC4	65	10	0.1333	Missense_Mutation	p.D86G	Non-Targeted Panel Gene	Adult HGBCL, NOS
BCL-71	chr3	5188107	5188107	G	A	exonic	EDEM1	77	12	0.1348	Missense_Mutation	p.G101D	Non-Targeted Panel Gene	Adult HGBCL, NOS
BCL-71	chr1	169732902	169732902	G	A	exonic	SELE	64	10	0.1351	Missense_Mutation	p.T45I	Non-Targeted Panel Gene	Adult HGBCL, NOS
BCL-71	chr4	99284440	99284440	C	T	exonic	ADH1A	54	9	0.1429	Missense_Mutation	p.G176R	Non-Targeted Panel Gene	Adult HGBCL, NOS
BCL-71	chr1	231770956	231770956	T	C	exonic	DISC1	48	8	0.1429	Missense_Mutation	p.L539P	Non-Targeted Panel Gene	Adult HGBCL, NOS
BCL-71	chr1	16063634	16063634	G	T	exonic	FAM131C	65	11	0.1447	Missense_Mutation	p.L9M	Non-Targeted Panel Gene	Adult HGBCL, NOS
BCL-71	chr19	42095878	42095878	T	A	exonic	POU2F2	74	13	0.1477	Missense_Mutation	p.T223S	Non-Targeted Panel Gene	Adult HGBCL, NOS
BCL-71	chr1	225368030	225368030	G	A	exonic	DNAH14	50	9	0.1525	Missense_Mutation	p.G4013R	Non-Targeted Panel Gene	Adult HGBCL, NOS
BCL-71	chr19	16577619	16577619	T	C	exonic	MED26	66	12	0.1538	Missense_Mutation	p.K71E	Non-Targeted Panel Gene	Adult HGBCL, NOS
BCL-71	chr1	11837060	11837060	A	G	exonic	CLCN6	48	9	0.1579	Missense_Mutation	p.Y681C	Non-Targeted Panel Gene	Adult HGBCL, NOS
BCL-71	chr13	71479241	71479241	T	G	exonic	DACH1	64	12	0.1579	Missense_Mutation	p.M600L	Non-Targeted Panel Gene	Adult HGBCL, NOS
BCL-71	chr22	45354058	45354058	G	C	exonic	SMC1B	37	7	0.1591	Missense_Mutation	p.L1065V	Non-Targeted Panel Gene	Adult HGBCL, NOS
BCL-71	chr3	142791098	142791098	T	G	exonic	TRPC1	74	14	0.1591	Missense_Mutation	p.S459R	Non-Targeted Panel Gene	Adult HGBCL, NOS
BCL-71	chr19	49480456	49480456	A	T	exonic	FLT3LG	58	11	0.1594	Missense_Mutation	p.T214S	Non-Targeted Panel Gene	Adult HGBCL, NOS
BCL-71	chr14	77899140	77899140	A	C	exonic	ADCK1	52	10	0.1613	Missense_Mutation	p.E208A	Non-Targeted Panel Gene	Adult HGBCL, NOS
BCL-71	chr2	240966321	240966321	G	C	exonic	CROCC2	66	13	0.1646	Missense_Mutation	p.G1353A	Non-Targeted Panel Gene	Adult HGBCL, NOS
BCL-71	chr7	137957726	137957726	A	C	exonic	LOC100130880	66	13	0.1646	Missense_Mutation	p.Q72H	Non-Targeted Panel Gene	Adult HGBCL, NOS
BCL-71	chr5	88197746	88197746	T	C	exonic	TMEM161B	71	14	0.1647	Missense_Mutation	p.Y359C	Non-Targeted Panel Gene	Adult HGBCL, NOS
BCL-71	chr6	68930579	68930579	C	T	exonic	ADGRB3	60	12	0.1667	Missense_Mutation	p.H260Y	Non-Targeted Panel Gene	Adult HGBCL, NOS
BCL-71	chr16	30765218	30765218	C	A	exonic	RNF40	60	12	0.1667	Missense_Mutation	p.T270N	Non-Targeted Panel Gene	Adult HGBCL, NOS
BCL-71	chr17	6120519	6120519	G	T	exonic	WSCD1	45	9	0.1667	Missense_Mutation	p.R529L	Non-Targeted Panel Gene	Adult HGBCL, NOS
BCL-71	chr6	28276828	28276828	A	G	exonic	ZSCAN26	53	11	0.1719	Missense_Mutation	p.H256R	Non-Targeted Panel Gene	Adult HGBCL, NOS
BCL-71	chr15	54533050	54533050	G	A	exonic	UNC13C	51	11	0.1774	Missense_Mutation	p.D1892N	Non-Targeted Panel Gene	Adult HGBCL, NOS
BCL-71	chr12	121453128	121453128	A	G	exonic	KDM2B	64	14	0.1795	Missense_Mutation	p.C651R	Non-Targeted Panel Gene	Adult HGBCL, NOS
BCL-71	chr4	138219351	138219351	A	C	exonic	SLC7A11	68	15	0.1807	Missense_Mutation	p.F221V	Non-Targeted Panel Gene	Adult HGBCL, NOS
BCL-71	chr1	227845542	227845542	G	A	exonic	PRSS38	52	12	0.1875	Missense_Mutation	p.G219E	Non-Targeted Panel Gene	Adult HGBCL, NOS
BCL-71	chr6	63646552	63646552	A	G	exonic	PHF3	56	13	0.1884	Missense_Mutation	p.M1V	Non-Targeted Panel Gene	Adult HGBCL, NOS
BCL-71	chr3	149178504	149178504	T	C	exonic	CP	55	13	0.1912	Missense_Mutation	p.Y930C	Non-Targeted Panel Gene	Adult HGBCL, NOS
BCL-71	chr1	211479188	211479188	G	A	exonic	RD3	50	12	0.1935	Missense_Mutation	p.P146S	Non-Targeted Panel Gene	Adult HGBCL, NOS
BCL-71	chr1	43305118	43305118	C	T	exonic	TIE1	57	14	0.1972	Missense_Mutation	p.A109V	Non-Targeted Panel Gene	Adult HGBCL, NOS
BCL-71	chr9	133360334	133360334	A	T	exonic	SURF2	63	16	0.2025	Missense_Mutation	p.D196V	Non-Targeted Panel Gene	Adult HGBCL, NOS
BCL-71	chr15	72139760	72139760	A	G	exonic	SENP8	47	12	0.2034	Missense_Mutation	p.D46G	Non-Targeted Panel Gene	Adult HGBCL, NOS
BCL-71	chr2	19974573	19974573	G	T	exonic	WDR35	43	11	0.2037	Missense_Mutation	p.H211N	Non-Targeted Panel Gene	Adult HGBCL, NOS
BCL-71	chrX	93672521	93672521	C	A	exonic	NAP1L3	27	7	0.2059	Missense_Mutation	p.D262Y	Non-Targeted Panel Gene	Adult HGBCL, NOS
BCL-71	chr17	7512827	7512827	G	A	exonic	POLR2A	38	10	0.2083	Missense_Mutation	p.A1554T	Non-Targeted Panel Gene	Adult HGBCL, NOS
BCL-71	chr2	19974633	19974633	T	A	exonic	WDR35	38	10	0.2083	Missense_Mutation	p.I191L	Non-Targeted Panel Gene	Adult HGBCL, NOS
BCL-71	chr7	30504609	30504609	C	T	exonic	GGCT	60	16	0.2105	Missense_Mutation	p.R34Q	Non-Targeted Panel Gene	Adult HGBCL, NOS
BCL-71	chr19	38422103	38422103	C	T	exonic	RASGRP4	41	11	0.2115	Missense_Mutation	p.R25H	Non-Targeted Panel Gene	Adult HGBCL, NOS
BCL-71	chr6	85487419	85487419	A	C	exonic	NT5E	70	19	0.2135	Missense_Mutation	p.Y345S	Non-Targeted Panel Gene	Adult HGBCL, NOS
BCL-71	chr15	48992735	48992735	T	C	exonic	SECISBP2L	58	16	0.2162	Missense_Mutation	p.T939A	Non-Targeted Panel Gene	Adult HGBCL, NOS
BCL-71	chr1	58576870	58576870	G	A	exonic	TACSTD2	57	16	0.2192	Missense_Mutation	p.A96V	Non-Targeted Panel Gene	Adult HGBCL, NOS
BCL-71	chr22	37878043	37878043	G	A	exonic	EIF3L	32	9	0.2195	Missense_Mutation	p.E483K	Non-Targeted Panel Gene	Adult HGBCL, NOS
BCL-71	chr1	166920744	166920744	G	A	exonic	ILDR2	73	21	0.2234	Missense_Mutation	p.S616L	Non-Targeted Panel Gene	Adult HGBCL, NOS
BCL-71	chr5	141345580	141345580	C	T	exonic	PCDHGA3	51	15	0.2273	Missense_Mutation	p.A516V	Non-Targeted Panel Gene	Adult HGBCL, NOS
BCL-71	chr1	32327583	32327583	A	G	exonic	HDAC1	42	13	0.2364	Missense_Mutation	p.D181G	Non-Targeted Panel Gene	Adult HGBCL, NOS
BCL-71	chr3	3040152	3040152	A	C	exonic	CNTN4	40	13	0.2453	Missense_Mutation	p.Y760S	Non-Targeted Panel Gene	Adult HGBCL, NOS
BCL-71	chr2	209943392	209943392	T	G	exonic	UNC80	49	16	0.2462	Missense_Mutation	p.Y2239D	Non-Targeted Panel Gene	Adult HGBCL, NOS
BCL-71	chr13	26759117	26759117	G	T	exonic	GPR12	51	17	0.25	Missense_Mutation	p.F237L	Non-Targeted Panel Gene	Adult HGBCL, NOS
BCL-71	chr15	28191142	28191142	A	T	exonic	HERC2	39	13	0.25	Missense_Mutation	p.S2852T	Non-Targeted Panel Gene	Adult HGBCL, NOS
BCL-71	chr11	67507379	67507379	T	G	exonic	CDK2AP2	58	21	0.2658	Missense_Mutation	p.E100A	Non-Targeted Panel Gene	Adult HGBCL, NOS
BCL-71	chr12	120144380	120144380	C	T	exonic	GCN1	46	18	0.2812	Missense_Mutation	p.M1807I	Non-Targeted Panel Gene	Adult HGBCL, NOS
BCL-71	chr5	140928751	140928751	C	T	exonic	PCDHAC1	40	16	0.2857	Missense_Mutation	p.T620I	Non-Targeted Panel Gene	Adult HGBCL, NOS
BCL-71	chr6	57147549	57147549	G	C	exonic	ZNF451	41	24	0.3692	Missense_Mutation	p.K488N	Non-Targeted Panel Gene	Adult HGBCL, NOS
BCL-71	chr17	72121749	72121749	C	T	exonic	SOX9	51	30	0.3704	Missense_Mutation	p.R120C	Non-Targeted Panel Gene	Adult HGBCL, NOS
BCL-71	chr15	73873747	73873747	G	C	exonic	TBC1D21	45	27	0.375	Missense_Mutation	p.R13T	Non-Targeted Panel Gene	Adult HGBCL, NOS
BCL-71	chr8	29146472	29146472	C	G	exonic	KIF13B	40	25	0.3846	Missense_Mutation	p.R698T	Non-Targeted Panel Gene	Adult HGBCL, NOS
BCL-71	chr13	70107672	70107672	C	A	exonic	KLHL1	46	30	0.3947	Missense_Mutation	p.D10Y	Non-Targeted Panel Gene	Adult HGBCL, NOS
BCL-71	chr1	95239909	95239909	G	A	exonic	RWDD3	40	27	0.403	Missense_Mutation	p.E14K	Non-Targeted Panel Gene	Adult HGBCL, NOS

BCL-71	chr13	100368479	100368479	G	T	exonic	PCCA	29	20	0.4082	Missense_Mutation	p.V551F	Non-Targeted Panel Gene	Adult HGBCL, NOS
BCL-71	chr3	49174347	49174347	C	T	exonic	KLHDC8B	37	29	0.4394	Missense_Mutation	p.P162L	Non-Targeted Panel Gene	Adult HGBCL, NOS
BCL-71	chr2	20037399	20037399	G	A	exonic	LAPTM4A	38	30	0.4412	Missense_Mutation	p.L117F	Non-Targeted Panel Gene	Adult HGBCL, NOS
BCL-71	chr12	101285813	101285813	C	G	exonic	UTP20	27	22	0.449	Missense_Mutation	p.N86K	Non-Targeted Panel Gene	Adult HGBCL, NOS
BCL-71	chr17	37553123	37553123	G	A	exonic	SYNRG	33	27	0.45	Missense_Mutation	p.P867L	Non-Targeted Panel Gene	Adult HGBCL, NOS
BCL-71	chr6	150865928	150865928	G	T	exonic	MTHFD1L	53	44	0.4536	Missense_Mutation	p.G36C	Non-Targeted Panel Gene	Adult HGBCL, NOS
BCL-71	chr16	1093977	1093977	G	C	exonic	C1QTNF8	51	44	0.4632	Missense_Mutation	p.L95V	Non-Targeted Panel Gene	Adult HGBCL, NOS
BCL-71	chr8	22122838	22122838	C	T	exonic	HR	47	41	0.4659	Missense_Mutation	p.G653R	Non-Targeted Panel Gene	Adult HGBCL, NOS
BCL-71	chr5	151822915	151822915	C	T	exonic	GLRA1	29	26	0.4727	Missense_Mutation	p.G378S	Non-Targeted Panel Gene	Adult HGBCL, NOS
BCL-71	chr19	18066634	18066634	A	G	exonic	IL12RB1	32	29	0.4754	Missense_Mutation	p.V504A	Non-Targeted Panel Gene	Adult HGBCL, NOS
BCL-71	chr20	63312442	63312442	C	T	exonic	COL20A1	33	30	0.4762	Missense_Mutation	p.T609I	Non-Targeted Panel Gene	Adult HGBCL, NOS
BCL-71	chr3	14402595	14402595	T	C	exonic	SLC6A6	48	44	0.4783	Missense_Mutation	p.M1T	Non-Targeted Panel Gene	Adult HGBCL, NOS
BCL-71	chr2	219483539	219483539	C	G	exonic	SPEG	56	52	0.4815	Missense_Mutation	p.R2026G	Non-Targeted Panel Gene	Adult HGBCL, NOS
BCL-71	chr8	38788786	38788786	C	G	exonic	TACC1	31	29	0.4833	Missense_Mutation	p.L82V	Non-Targeted Panel Gene	Adult HGBCL, NOS
BCL-71	chr11	121627569	121627569	G	A	exonic	SORL1	40	38	0.4872	Missense_Mutation	p.A2127T	Non-Targeted Panel Gene	Adult HGBCL, NOS
BCL-71	chr15	65639690	65639690	C	A	exonic	SLC24A1	31	30	0.4918	Missense_Mutation	p.D662E	Non-Targeted Panel Gene	Adult HGBCL, NOS
BCL-71	chr5	42699919	42699919	C	T	exonic	GHR	35	34	0.4928	Missense_Mutation	p.R179C	Non-Targeted Panel Gene	Adult HGBCL, NOS
BCL-71	chr10	96982445	96982445	T	C	exonic	LCOR	38	37	0.4933	Missense_Mutation	p.M662T	Non-Targeted Panel Gene	Adult HGBCL, NOS
BCL-71	chr6	161136550	161136550	A	G	exonic	AGPAT4	40	41	0.5	Missense_Mutation	p.L376P	Non-Targeted Panel Gene	Adult HGBCL, NOS
BCL-71	chr11	61951844	61951844	G	A	exonic	BEST1	31	31	0.5	Missense_Mutation	p.R13H	Non-Targeted Panel Gene	Adult HGBCL, NOS
BCL-71	chr16	29847285	29847285	A	G	exonic	MVP	34	34	0.5	Missense_Mutation	p.Q785R	Non-Targeted Panel Gene	Adult HGBCL, NOS
BCL-71	chr1	233095844	233095844	T	C	exonic	PCNX2	35	35	0.5	Missense_Mutation	p.K1286R	Non-Targeted Panel Gene	Adult HGBCL, NOS
BCL-71	chr12	25648506	25648506	G	T	exonic	LMNTD1	29	30	0.5085	Missense_Mutation	p.Q16K	Non-Targeted Panel Gene	Adult HGBCL, NOS
BCL-71	chr16	57372623	57372623	A	G	exonic	CX3CL1	27	28	0.5091	Missense_Mutation	p.T19A	Non-Targeted Panel Gene	Adult HGBCL, NOS
BCL-71	chr19	58261754	58261754	C	T	exonic	ZNF544	25	26	0.5098	Missense_Mutation	p.T383I	Non-Targeted Panel Gene	Adult HGBCL, NOS
BCL-71	chr9	32489401	32489401	A	T	exonic	DDX58	35	38	0.5205	Missense_Mutation	p.L248I	Non-Targeted Panel Gene	Adult HGBCL, NOS
BCL-71	chr4	161842	161842	A	G	exonic	ZNF718	30	33	0.5238	Missense_Mutation	p.K354R	Non-Targeted Panel Gene	Adult HGBCL, NOS
BCL-71	chr20	25284240	25284240	C	G	exonic	PYGB	34	38	0.5278	Missense_Mutation	p.T586S	Non-Targeted Panel Gene	Adult HGBCL, NOS
BCL-71	chr10	87862083	87862083	C	A	exonic	KLLN	37	43	0.5375	Missense_Mutation	p.K135N	Non-Targeted Panel Gene	Adult HGBCL, NOS
BCL-71	chr4	71763884	71763884	C	G	exonic	GC	41	49	0.5444	Missense_Mutation	p.V195L	Non-Targeted Panel Gene	Adult HGBCL, NOS
BCL-71	chr2	117819753	117819753	G	A	exonic	DDX18	29	35	0.5469	Missense_Mutation	p.D159N	Non-Targeted Panel Gene	Adult HGBCL, NOS
BCL-71	chr3	122262227	122262227	G	A	exonic	CASR	26	32	0.5517	Missense_Mutation	p.D398N	Non-Targeted Panel Gene	Adult HGBCL, NOS
BCL-71	chr18	21496501	21496501	C	T	exonic	GREB1L	34	42	0.5526	Missense_Mutation	p.T1065I	Non-Targeted Panel Gene	Adult HGBCL, NOS
BCL-71	chr2	164621138	164621138	G	C	exonic	GRB14	29	36	0.5538	Missense_Mutation	p.R58G	Non-Targeted Panel Gene	Adult HGBCL, NOS
BCL-71	chr2	209695858	209695858	G	C	exonic	MAP2	36	45	0.5556	Missense_Mutation	p.A1230P	Non-Targeted Panel Gene	Adult HGBCL, NOS
BCL-71	chr11	119193817	119193817	C	G	exonic	CCDC153	27	35	0.5645	Missense_Mutation	p.V69L	Non-Targeted Panel Gene	Adult HGBCL, NOS
BCL-71	chr13	109141050	109141050	C	G	exonic	MYO16	31	41	0.5694	Missense_Mutation	p.A1591G	Non-Targeted Panel Gene	Adult HGBCL, NOS
BCL-71	chr22	29059086	29059086	C	T	exonic	C22orf31	26	36	0.5806	Missense_Mutation	p.D177N	Non-Targeted Panel Gene	Adult HGBCL, NOS
BCL-71	chr19	58212841	58212841	C	G	exonic	ZNF274	31	43	0.5811	Missense_Mutation	p.Q554E	Non-Targeted Panel Gene	Adult HGBCL, NOS
BCL-71	chr17	59088405	59088405	G	A	exonic	TRIM37	22	31	0.5849	Missense_Mutation	p.A56V	Non-Targeted Panel Gene	Adult HGBCL, NOS
BCL-71	chr4	112647203	112647203	G	C	exonic	LARP7	19	27	0.587	Missense_Mutation	p.E224D	Non-Targeted Panel Gene	Adult HGBCL, NOS
BCL-71	chr4	95218981	95218981	C	G	exonic	UNC5C	33	49	0.5976	Missense_Mutation	p.V545L	Non-Targeted Panel Gene	Adult HGBCL, NOS
BCL-71	chr10	114145698	114145698	C	A	exonic	CCDC186	21	32	0.6038	Missense_Mutation	p.G318C	Non-Targeted Panel Gene	Adult HGBCL, NOS
BCL-71	chr15	23644072	23644072	T	A	exonic	MAGEL2	17	26	0.6047	Missense_Mutation	p.E1224V	Non-Targeted Panel Gene	Adult HGBCL, NOS
BCL-71	chr17	69291258	69291258	T	C	exonic	ABCA5	25	41	0.6212	Missense_Mutation	p.T522A	Non-Targeted Panel Gene	Adult HGBCL, NOS
BCL-71	chr8	131987678	131987678	G	A	exonic	EFR3A	25	63	0.7159	Missense_Mutation	p.V681I	Non-Targeted Panel Gene	Adult HGBCL, NOS
BCL-71	chrX	115635045	115635045	A	C	exonic	PLS3	0	24	1	Missense_Mutation	p.E204D	Non-Targeted Panel Gene	Adult HGBCL, NOS
BCL-71	chr7	111868118	111868118	G	A	exonic	DOCK4	68	10	0.1282	Nonsense_Mutation	p.R716X	Targerted Panel Gene	Adult HGBCL, NOS
BCL-71	chr19	18855986	18855986	C	T	exonic	UPF1	65	12	0.1558	Nonsense_Mutation	p.Q536X	Targerted Panel Gene	Adult HGBCL, NOS
BCL-71	chr12	49044960	49044960	G	A	exonic	KMT2D	67	13	0.1625	Nonsense_Mutation	p.Q1583X	Targerted Panel Gene	Adult HGBCL, NOS
BCL-71	chrX	141879303	141879303	G	A	exonic	MAGEC3	31	11	0.2619	Nonsense_Mutation	p.W129X	Targerted Panel Gene	Adult HGBCL, NOS
BCL-71	chr17	65056512	65056512	G	A	exonic	GNA13	48	29	0.3766	Nonsense_Mutation	p.Q28X	Targerted Panel Gene	Adult HGBCL, NOS
BCL-71	chr8	104491506	104491506	G	A	exonic	LRP12	70	8	0.1013	Nonsense_Mutation	p.R583X	Non-Targeted Panel Gene	Adult HGBCL, NOS
BCL-71	chr1	1281426	1281426	G	A	exonic	SCNN1D	71	9	0.1125	Nonsense_Mutation	p.W31X	Non-Targeted Panel Gene	Adult HGBCL, NOS
BCL-71	chr6	46716456	46716456	C	A	exonic	PLA2G7	66	9	0.12	Nonsense_Mutation	p.E102X	Non-Targeted Panel Gene	Adult HGBCL, NOS
BCL-71	chr10	96982213	96982213	C	T	exonic	LCOR	63	10	0.137	Nonsense_Mutation	p.R585X	Non-Targeted Panel Gene	Adult HGBCL, NOS
BCL-71	chr5	158796465	158796465	A	T	exonic	EBF1	76	16	0.1739	Nonsense_Mutation	p.C263X	Non-Targeted Panel Gene	Adult HGBCL, NOS
BCL-71	chr17	75148598	75148598	T	A	exonic	JPT1	49	13	0.2097	Nonsense_Mutation	p.K44X	Non-Targeted Panel Gene	Adult HGBCL, NOS
BCL-71	chr7	20645792	20645792	-	A	exonic	ABCB5	33	37	0.5286	Nonsense_Mutation	p.Y239_S240delins	Non-Targeted Panel Gene	Adult HGBCL, NOS
BCL-72	chr8	47862371	47862371	A	G	splicing	PRKDC	72	14	0.1628	Splice_Site	.	Targerted Panel Gene	Adult HGBCL, NOS
BCL-72	chr12	49046607	49046607	A	T	splicing	KMT2D	57	42	0.4242	Splice_Site	.	Targerted Panel Gene	Adult HGBCL, NOS
BCL-72	chr1	21873924	21873924	C	G	splicing	HSPG2	46	35	0.4321	Splice_Site	.	Targerted Panel Gene	Adult HGBCL, NOS
BCL-72	chr10	74138933	74138933	-	A	splicing	AP3M1	35	7	0.1556	Splice_Site	.	Non-Targeted Panel Gene	Adult HGBCL, NOS
BCL-72	chr9	110375465	110375465	-	A	splicing	SVEP1	24	9	0.2143	Splice_Site	.	Non-Targeted Panel Gene	Adult HGBCL, NOS
BCL-72	chr2	47834786	47834786	A	T	splicing	FBXO11	56	28	0.3333	Splice_Site	.	Non-Targeted Panel Gene	Adult HGBCL, NOS
BCL-72	chr12	64445073	64445073	A	C	splicing	XPOT	48	32	0.4	Splice_Site	.	Non-Targeted Panel Gene	Adult HGBCL, NOS
BCL-72	chr8	23154873	23154873	C	T	splicing	TNFRSF10D	46	31	0.4026	Splice_Site	.	Non-Targeted Panel Gene	Adult HGBCL, NOS
BCL-72	chr7	2230766	2230766	T	A	splicing	MAD1L1	46	37	0.4458	Splice_Site	.	Non-Targeted Panel Gene	Adult HGBCL, NOS
BCL-72	chr10	96628495	96628495	T	C	splicing	PIK3AP1	46	38	0.4524	Splice_Site	.	Non-Targeted Panel Gene	Adult HGBCL, NOS
BCL-72	chr14	30935162	30935162	C	A	splicing	STRN3	46	44	0.4889	Splice_Site	.	Non-Targeted Panel Gene	Adult HGBCL, NOS
BCL-72	chr19	8210864	8210864	T	C	splicing	CERS4	36	36	0.5	Splice_Site	.	Non-Targeted Panel Gene	Adult HGBCL, NOS
BCL-72	chr16	66790725	66790725	T	C	splicing	TERB1	36	43	0.5443	Splice_Site	.	Non-Targeted Panel Gene	Adult HGBCL, NOS
BCL-72	chr2	229777314	229777314	C	T	splicing	TRIP12	13	23	0.6389	Splice_Site	.	Non-Targeted Panel Gene	Adult HGBCL, NOS
BCL-72	chr16	50311754	50311754	T	-	exonic	ADCY7	37	16	0.3019	Frame_Shift_Del	p.F806fs	Non-Targeted Panel Gene	Adult HGBCL, NOS
BCL-72	chr19	56576728	56576728	G	-	exonic	ZNF470	36	28	0.4375	Frame_Shift_Del	p.W100fs	Non-Targeted Panel Gene	Adult HGBCL, NOS
BCL-72	chr3	39265597	39265597	A	-	exonic	CX3CR1	53	42	0.4421	Frame_Shift_Del	p.Y305fs	Non-Targeted Panel Gene	Adult HGBCL, NOS
BCL-72	chr2	162288301	162288301	G	-	exonic	IFIH1	32	35	0.5147	Frame_Shift_Del	p.P310fs	Non-Targeted Panel Gene	Adult HGBCL, NOS
BCL-72	chr17	10315767	10315767	-	T	exonic	MYH13	65	7	0.0972	Frame_Shift_Ins	p.Q1304fs	Non-Targeted Panel Gene	Adult HGBCL, NOS
BCL-72	chr11	123729158	123729158	-	T	exonic	ZNF202	49	37	0.4302	Frame_Shift_Ins	p.E224fs	Non-Targeted Panel Gene	Adult HGBCL, NOS
BCL-72	chr6	156778872	156778874	GGA	-	exonic	ARID1B	49	35	0.4167	In_Frame_Del	p.315_315del	Targerted Panel Gene	Adult HGBCL, NOS
BCL-72	chr7	2355065	2355091	CGAGGCCTCCAGT[illegible]	-	exonic	EIF3B	68	33	0.3267	In_Frame_Del	p.48_57del	Non-Targeted Panel Gene	Adult HGBCL, NOS
BCL-72	chr1	63651792	63651794	GGA	-	exonic	PGM1	44	41	0.4824	In_Frame_Del	p.468_469del	Non-Targeted Panel Gene	Adult HGBCL, NOS

BCL-72	chr9	35906586	35906586	-	CCACCACCACCA	exonic	HRCT1	52	6	0.0909	In_Frame_Ins	p.L100delinsLHHH	Non-Targeted Panel Gene	Adult HGBCL, NOS
BCL-72	chr19	13208877	13208877	-	GGT	exonic	CACNA1A	58	22	0.275	In_Frame_Ins	p.P2220delinsHP	Non-Targeted Panel Gene	Adult HGBCL, NOS
BCL-72	chr5	61332422	61332422	-	GCG	exonic	ZSWIM6	51	32	0.3855	In_Frame_Ins	p.G50delinsGA	Non-Targeted Panel Gene	Adult HGBCL, NOS
BCL-72	chr17	7885064	7885064	-	CCGCCGCCC	exonic	CHD3	30	52	0.5149	In_Frame_Ins	p.P86delinsPPPP	Non-Targeted Panel Gene	Adult HGBCL, NOS
BCL-72	chr7	100954669	100954669	T	A	exonic	MUC3A	93	23	0.1983	Missense_Mutation	p.S964T	Targerted Panel Gene	Adult HGBCL, NOS
BCL-72	chr7	82915741	82915741	G	A	exonic	PCLO	74	24	0.2449	Missense_Mutation	p.T4082I	Targerted Panel Gene	Adult HGBCL, NOS
BCL-72	chr7	111123586	111123586	A	T	exonic	LRRN3	78	35	0.3097	Missense_Mutation	p.I272F	Targerted Panel Gene	Adult HGBCL, NOS
BCL-72	chr7	111123385	111123385	G	A	exonic	LRRN3	81	41	0.3361	Missense_Mutation	p.D205N	Targerted Panel Gene	Adult HGBCL, NOS
BCL-72	chrX	77600552	77600552	T	C	exonic	ATRX	68	39	0.3645	Missense_Mutation	p.N1860S	Targerted Panel Gene	Adult HGBCL, NOS
BCL-72	chr4	84783074	84783074	A	C	exonic	WDFY3	42	25	0.3731	Missense_Mutation	p.L1355V	Targerted Panel Gene	Adult HGBCL, NOS
BCL-72	chr1	215625809	215625809	C	T	exonic	USH2A	45	28	0.3836	Missense_Mutation	p.R5194H	Targerted Panel Gene	Adult HGBCL, NOS
BCL-72	chr2	51028170	51028170	C	A	exonic	NRXN1	40	26	0.3939	Missense_Mutation	p.G35V	Targerted Panel Gene	Adult HGBCL, NOS
BCL-72	chr6	41935959	41935959	A	C	exonic	CCND3	44	32	0.4211	Missense_Mutation	p.V287G	Targerted Panel Gene	Adult HGBCL, NOS
BCL-72	chr14	64793601	64793601	C	T	exonic	SPTB	54	40	0.4255	Missense_Mutation	p.G688R	Targerted Panel Gene	Adult HGBCL, NOS
BCL-72	chr19	10986422	10986422	C	T	exonic	SMARCA4	48	37	0.4353	Missense_Mutation	p.P197S	Targerted Panel Gene	Adult HGBCL, NOS
BCL-72	chr19	4510801	4510801	C	G	exonic	PLIN4	50	42	0.4565	Missense_Mutation	p.Q1039H	Targerted Panel Gene	Adult HGBCL, NOS
BCL-72	chr20	58853749	58853749	A	G	exonic	GNAS	53	50	0.4854	Missense_Mutation	p.M162V	Targerted Panel Gene	Adult HGBCL, NOS
BCL-72	chr13	48364952	48364952	C	T	exonic	RB1	49	47	0.4896	Missense_Mutation	p.T307I	Targerted Panel Gene	Adult HGBCL, NOS
BCL-72	chr1	216198494	216198494	C	A	exonic	USH2A	32	31	0.4921	Missense_Mutation	p.G1301V	Targerted Panel Gene	Adult HGBCL, NOS
BCL-72	chr14	64795395	64795395	G	A	exonic	SPTB	37	39	0.5132	Missense_Mutation	p.A529V	Targerted Panel Gene	Adult HGBCL, NOS
BCL-72	chr7	30455259	30455259	A	T	exonic	NOD1	41	50	0.5495	Missense_Mutation	p.F85Y	Targerted Panel Gene	Adult HGBCL, NOS
BCL-72	chr6	27146509	27146509	G	C	exonic	HIST1H2BK	8	37	0.8222	Missense_Mutation	p.T97R	Targerted Panel Gene	Adult HGBCL, NOS
BCL-72	chr17	7673803	7673803	G	A	exonic	TP53	11	66	0.8571	Missense_Mutation	p.R234C	Targerted Panel Gene	Adult HGBCL, NOS
BCL-72	chr3	53188785	53188785	T	G	exonic	PRKCD	95	5	0.05	Missense_Mutation	p.M494R	Non-Targeted Panel Gene	Adult HGBCL, NOS
BCL-72	chr11	64776507	64776507	C	T	exonic	SF1	117	7	0.0565	Missense_Mutation	p.A51T	Non-Targeted Panel Gene	Adult HGBCL, NOS
BCL-72	chr16	57976622	57976622	G	A	exonic	TEPP	69	5	0.0676	Missense_Mutation	p.V51I	Non-Targeted Panel Gene	Adult HGBCL, NOS
BCL-72	chr10	123149825	123149825	G	A	exonic	HMX2	74	6	0.075	Missense_Mutation	p.S175N	Non-Targeted Panel Gene	Adult HGBCL, NOS
BCL-72	chr4	158171318	158171318	C	T	exonic	FAM198B	86	7	0.0753	Missense_Mutation	p.V20I	Non-Targeted Panel Gene	Adult HGBCL, NOS
BCL-72	chr11	4769008	4769008	C	T	exonic	OR51F1	62	7	0.1014	Missense_Mutation	p.A304T	Non-Targeted Panel Gene	Adult HGBCL, NOS
BCL-72	chr19	54804874	54804874	A	C	exonic	KIR2DL4	77	10	0.1149	Missense_Mutation	p.Y53S	Non-Targeted Panel Gene	Adult HGBCL, NOS
BCL-72	chr19	44328488	44328488	T	G	exonic	ZNF112	77	17	0.1809	Missense_Mutation	p.S496R	Non-Targeted Panel Gene	Adult HGBCL, NOS
BCL-72	chr7	89337009	89337009	A	C	exonic	ZNF804B	81	23	0.2212	Missense_Mutation	p.N1343H	Non-Targeted Panel Gene	Adult HGBCL, NOS
BCL-72	chr7	149475006	149475006	G	A	exonic	ZNF746	149	47	0.2398	Missense_Mutation	p.P438L	Non-Targeted Panel Gene	Adult HGBCL, NOS
BCL-72	chrX	137567446	137567446	C	T	exonic	ZIC3	104	40	0.2778	Missense_Mutation	p.S252L	Non-Targeted Panel Gene	Adult HGBCL, NOS
BCL-72	chr16	50311757	50311757	T	C	exonic	ADCY7	38	16	0.2963	Missense_Mutation	p.Y807H	Non-Targeted Panel Gene	Adult HGBCL, NOS
BCL-72	chr14	21400511	21400511	A	G	exonic	CHD8	71	32	0.3107	Missense_Mutation	p.L1212P	Non-Targeted Panel Gene	Adult HGBCL, NOS
BCL-72	chr2	47834824	47834824	A	T	exonic	FBXO11	66	30	0.3125	Missense_Mutation	p.S171R	Non-Targeted Panel Gene	Adult HGBCL, NOS
BCL-72	chr9	85020228	85020228	C	T	exonic	NTRK2	57	26	0.3133	Missense_Mutation	p.P732L	Non-Targeted Panel Gene	Adult HGBCL, NOS
BCL-72	chr16	50311761	50311761	T	A	exonic	ADCY7	35	16	0.3137	Missense_Mutation	p.I808N	Non-Targeted Panel Gene	Adult HGBCL, NOS
BCL-72	chr4	3767460	3767460	C	T	exonic	ADRA2C	74	35	0.3211	Missense_Mutation	p.P285L	Non-Targeted Panel Gene	Adult HGBCL, NOS
BCL-72	chrX	115640416	115640416	A	C	exonic	PLS3	62	30	0.3261	Missense_Mutation	p.K287N	Non-Targeted Panel Gene	Adult HGBCL, NOS
BCL-72	chr2	47834811	47834811	A	G	exonic	FBXO11	64	31	0.3263	Missense_Mutation	p.Y176H	Non-Targeted Panel Gene	Adult HGBCL, NOS
BCL-72	chr1	224680994	224680994	A	C	exonic	CNIH3	53	27	0.3375	Missense_Mutation	p.S22R	Non-Targeted Panel Gene	Adult HGBCL, NOS
BCL-72	chrX	18246672	18246672	C	T	exonic	SCML2	72	42	0.3684	Missense_Mutation	p.S576N	Non-Targeted Panel Gene	Adult HGBCL, NOS
BCL-72	chr7	23361558	23361558	T	A	exonic	IGF2BP3	55	33	0.375	Missense_Mutation	p.Y126F	Non-Targeted Panel Gene	Adult HGBCL, NOS
BCL-72	chr8	50708953	50708953	T	G	exonic	SNTG1	44	27	0.3803	Missense_Mutation	p.F420C	Non-Targeted Panel Gene	Adult HGBCL, NOS
BCL-72	chr12	69677190	69677190	A	C	exonic	BEST3	55	34	0.382	Missense_Mutation	p.V235G	Non-Targeted Panel Gene	Adult HGBCL, NOS
BCL-72	chr21	30396550	30396550	G	A	exonic	KRTAP13-1	42	26	0.3824	Missense_Mutation	p.R155K	Non-Targeted Panel Gene	Adult HGBCL, NOS
BCL-72	chr2	151373571	151373571	G	C	exonic	TNFAIP6	43	27	0.3857	Missense_Mutation	p.D216H	Non-Targeted Panel Gene	Adult HGBCL, NOS
BCL-72	chr3	169381476	169381476	A	T	exonic	MECOM	49	31	0.3875	Missense_Mutation	p.M29K	Non-Targeted Panel Gene	Adult HGBCL, NOS
BCL-72	chr19	10224119	10224119	A	G	exonic	S1PR2	72	46	0.3898	Missense_Mutation	p.S263P	Non-Targeted Panel Gene	Adult HGBCL, NOS
BCL-72	chr2	195875788	195875788	G	T	exonic	DNAH7	50	32	0.3902	Missense_Mutation	p.P2058H	Non-Targeted Panel Gene	Adult HGBCL, NOS
BCL-72	chr2	196657037	196657037	T	A	exonic	CCDC150	53	34	0.3908	Missense_Mutation	p.N159K	Non-Targeted Panel Gene	Adult HGBCL, NOS
BCL-72	chr1	246565834	246565834	C	G	exonic	TFB2M	59	38	0.3918	Missense_Mutation	p.C102S	Non-Targeted Panel Gene	Adult HGBCL, NOS
BCL-72	chr21	39199118	39199118	A	T	exonic	BRWD1	55	36	0.3956	Missense_Mutation	p.D1766E	Non-Targeted Panel Gene	Adult HGBCL, NOS
BCL-72	chr1	197101048	197101048	A	C	exonic	ASPM	69	46	0.3966	Missense_Mutation	p.F2735V	Non-Targeted Panel Gene	Adult HGBCL, NOS
BCL-72	chr19	48670826	48670826	T	G	exonic	NTN5	60	40	0.4	Missense_Mutation	p.H54P	Non-Targeted Panel Gene	Adult HGBCL, NOS
BCL-72	chr21	21338517	21338517	T	C	exonic	NCAM2	48	33	0.4074	Missense_Mutation	p.F343L	Non-Targeted Panel Gene	Adult HGBCL, NOS
BCL-72	chr19	42095878	42095878	T	A	exonic	POU2F2	71	50	0.4098	Missense_Mutation	p.T223S	Non-Targeted Panel Gene	Adult HGBCL, NOS
BCL-72	chr4	99313902	99313902	T	G	exonic	ADH1B	41	29	0.4143	Missense_Mutation	p.K209N	Non-Targeted Panel Gene	Adult HGBCL, NOS
BCL-72	chr8	17341443	17341443	C	G	exonic	MTMR7	49	35	0.4167	Missense_Mutation	p.E218Q	Non-Targeted Panel Gene	Adult HGBCL, NOS
BCL-72	chr7	5501187	5501187	G	A	exonic	FBXL18	53	38	0.4176	Missense_Mutation	p.S361L	Non-Targeted Panel Gene	Adult HGBCL, NOS
BCL-72	chr16	55485636	55485636	G	A	exonic	MMP2	39	28	0.4179	Missense_Mutation	p.E231K	Non-Targeted Panel Gene	Adult HGBCL, NOS
BCL-72	chr5	154713070	154713070	C	T	exonic	LARP1	43	31	0.4189	Missense_Mutation	p.P49S	Non-Targeted Panel Gene	Adult HGBCL, NOS
BCL-72	chr1	214640958	214640958	C	T	exonic	CENPF	36	26	0.4194	Missense_Mutation	p.H874Y	Non-Targeted Panel Gene	Adult HGBCL, NOS
BCL-72	chr2	30257599	30257599	C	A	exonic	LBH	51	37	0.4205	Missense_Mutation	p.A99E	Non-Targeted Panel Gene	Adult HGBCL, NOS
BCL-72	chr10	113850160	113850160	T	G	exonic	DCLRE1A	48	35	0.4217	Missense_Mutation	p.K315N	Non-Targeted Panel Gene	Adult HGBCL, NOS
BCL-72	chr18	23827325	23827325	A	G	exonic	LAMA3	45	33	0.4231	Missense_Mutation	p.Y894C	Non-Targeted Panel Gene	Adult HGBCL, NOS
BCL-72	chr10	113849231	113849231	T	C	exonic	DCLRE1A	54	40	0.4255	Missense_Mutation	p.E625G	Non-Targeted Panel Gene	Adult HGBCL, NOS
BCL-72	chr19	10224178	10224178	A	G	exonic	S1PR2	70	52	0.4262	Missense_Mutation	p.I243T	Non-Targeted Panel Gene	Adult HGBCL, NOS
BCL-72	chr2	96278284	96278284	A	G	exonic	SNRNP200	43	32	0.4267	Missense_Mutation	p.Y1855H	Non-Targeted Panel Gene	Adult HGBCL, NOS
BCL-72	chr5	180792446	180792446	G	A	exonic	MGAT1	54	41	0.4316	Missense_Mutation	p.H176Y	Non-Targeted Panel Gene	Adult HGBCL, NOS
BCL-72	chr9	122226464	122226464	C	G	exonic	LHX6	51	39	0.4333	Missense_Mutation	p.E125Q	Non-Targeted Panel Gene	Adult HGBCL, NOS
BCL-72	chr6	38594215	38594215	C	T	exonic	BTBD9	47	36	0.4337	Missense_Mutation	p.A100T	Non-Targeted Panel Gene	Adult HGBCL, NOS
BCL-72	chr2	233811905	233811905	A	G	exonic	MROH2A	47	36	0.4337	Missense_Mutation	p.D869G	Non-Targeted Panel Gene	Adult HGBCL, NOS
BCL-72	chr19	13965484	13965484	T	C	exonic	RFX1	49	38	0.4368	Missense_Mutation	p.M726V	Non-Targeted Panel Gene	Adult HGBCL, NOS
BCL-72	chr5	111106140	111106140	G	A	exonic	WDR36	32	25	0.4386	Missense_Mutation	p.A449T	Non-Targeted Panel Gene	Adult HGBCL, NOS
BCL-72	chr17	58696833	58696833	T	G	exonic	RAD51C	46	36	0.439	Missense_Mutation	p.L182R	Non-Targeted Panel Gene	Adult HGBCL, NOS
BCL-72	chr15	54555465	54555465	A	C	exonic	UNC13C	42	33	0.44	Missense_Mutation	p.T1969P	Non-Targeted Panel Gene	Adult HGBCL, NOS
BCL-72	chr16	23836252	23836252	T	G	exonic	PRKCB	68	54	0.4426	Missense_Mutation	p.L26R	Non-Targeted Panel Gene	Adult HGBCL, NOS
BCL-72	chr14	60509612	60509612	C	T	exonic	SIX6	45	36	0.4444	Missense_Mutation	p.H72Y	Non-Targeted Panel Gene	Adult HGBCL, NOS
BCL-72	chr19	50244332	50244332	A	G	exonic	MYH14	36	29	0.4462	Missense_Mutation	p.N402S	Non-Targeted Panel Gene	Adult HGBCL, NOS

BCL-72	chr8	143924486	143924486	G	A	exonic	PLEC	58	48	0.4486	Missense_Mutation	p.R1842W	Non-Targeted Panel Gene	Adult HGBCL, NOS
BCL-72	chr11	112037347	112037347	A	T	exonic	DLAT	43	37	0.4512	Missense_Mutation	p.T288S	Non-Targeted Panel Gene	Adult HGBCL, NOS
BCL-72	chr1	43346584	43346584	A	G	exonic	MPL	39	33	0.4521	Missense_Mutation	p.T374A	Non-Targeted Panel Gene	Adult HGBCL, NOS
BCL-72	chr19	39100782	39100782	G	A	exonic	ACP7	46	38	0.4524	Missense_Mutation	p.V246I	Non-Targeted Panel Gene	Adult HGBCL, NOS
BCL-72	chr2	196656984	196656984	A	G	exonic	CCDC150	46	38	0.4524	Missense_Mutation	p.I142V	Non-Targeted Panel Gene	Adult HGBCL, NOS
BCL-72	chr3	193626114	193626114	T	G	exonic	OPA1	46	38	0.4524	Missense_Mutation	p.L234R	Non-Targeted Panel Gene	Adult HGBCL, NOS
BCL-72	chr2	211420530	211420530	T	A	exonic	ERBB4	41	34	0.4533	Missense_Mutation	p.M1016L	Non-Targeted Panel Gene	Adult HGBCL, NOS
BCL-72	chr19	52322307	52322307	T	G	exonic	ZNF480	47	39	0.4535	Missense_Mutation	p.Y353D	Non-Targeted Panel Gene	Adult HGBCL, NOS
BCL-72	chr11	62801786	62801786	G	A	exonic	NXF1	42	35	0.4545	Missense_Mutation	p.H198Y	Non-Targeted Panel Gene	Adult HGBCL, NOS
BCL-72	chr12	26715325	26715325	T	C	exonic	ITPR2	55	46	0.4554	Missense_Mutation	p.S277G	Non-Targeted Panel Gene	Adult HGBCL, NOS
BCL-72	chr12	117234588	117234588	A	T	exonic	NOS1	49	41	0.4556	Missense_Mutation	p.L1105Q	Non-Targeted Panel Gene	Adult HGBCL, NOS
BCL-72	chr6	149749773	149749773	A	C	exonic	PCMT1	52	44	0.4583	Missense_Mutation	p.S16R	Non-Targeted Panel Gene	Adult HGBCL, NOS
BCL-72	chr4	72283383	72283383	G	T	exonic	ADAMTS3	33	28	0.459	Missense_Mutation	p.P1124H	Non-Targeted Panel Gene	Adult HGBCL, NOS
BCL-72	chr11	60936117	60936117	C	T	exonic	TMEM132A	47	40	0.4598	Missense_Mutation	p.T761I	Non-Targeted Panel Gene	Adult HGBCL, NOS
BCL-72	chr11	7701761	7701761	C	T	exonic	OVCH2	34	29	0.4603	Missense_Mutation	p.A172T	Non-Targeted Panel Gene	Adult HGBCL, NOS
BCL-72	chr20	53255380	53255380	C	T	exonic	TSHZ2	42	36	0.4615	Missense_Mutation	p.P641L	Non-Targeted Panel Gene	Adult HGBCL, NOS
BCL-72	chr2	84797560	84797560	G	A	exonic	DNAH6	44	38	0.4634	Missense_Mutation	p.D3795N	Non-Targeted Panel Gene	Adult HGBCL, NOS
BCL-72	chr11	77590216	77590216	C	G	exonic	AQP11	52	46	0.4694	Missense_Mutation	p.P75R	Non-Targeted Panel Gene	Adult HGBCL, NOS
BCL-72	chr8	1549543	1549543	A	G	exonic	DLGAP2	44	39	0.4699	Missense_Mutation	p.K364E	Non-Targeted Panel Gene	Adult HGBCL, NOS
BCL-72	chr6	65335139	65335139	G	T	exonic	EYS	43	39	0.4756	Missense_Mutation	p.A536E	Non-Targeted Panel Gene	Adult HGBCL, NOS
BCL-72	chr2	159228824	159228824	C	T	exonic	TANC1	43	40	0.4762	Missense_Mutation	p.S1353F	Non-Targeted Panel Gene	Adult HGBCL, NOS
BCL-72	chr20	3166820	3166820	A	G	exonic	LZTS3	49	45	0.4787	Missense_Mutation	p.V115A	Non-Targeted Panel Gene	Adult HGBCL, NOS
BCL-72	chr2	178738347	178738347	G	C	exonic	TTN	42	39	0.4815	Missense_Mutation	p.I4702M	Non-Targeted Panel Gene	Adult HGBCL, NOS
BCL-72	chr12	54183690	54183690	A	G	exonic	SMUG1	43	40	0.4819	Missense_Mutation	p.M84T	Non-Targeted Panel Gene	Adult HGBCL, NOS
BCL-72	chr19	45368706	45368706	T	C	exonic	ERCC2	53	50	0.4854	Missense_Mutation	p.E95G	Non-Targeted Panel Gene	Adult HGBCL, NOS
BCL-72	chr3	51275120	51275120	C	A	exonic	DOCK3	41	39	0.4875	Missense_Mutation	p.H864N	Non-Targeted Panel Gene	Adult HGBCL, NOS
BCL-72	chr18	13057659	13057659	A	G	exonic	CEP192	41	40	0.4878	Missense_Mutation	p.S1395G	Non-Targeted Panel Gene	Adult HGBCL, NOS
BCL-72	chr2	74093580	74093580	C	T	exonic	TET3	42	40	0.4878	Missense_Mutation	p.R1061W	Non-Targeted Panel Gene	Adult HGBCL, NOS
BCL-72	chr22	50579775	50579775	T	C	exonic	CHKB	43	41	0.4881	Missense_Mutation	p.Q328R	Non-Targeted Panel Gene	Adult HGBCL, NOS
BCL-72	chr6	158503337	158503337	T	C	exonic	TULP4	42	41	0.494	Missense_Mutation	p.V1225A	Non-Targeted Panel Gene	Adult HGBCL, NOS
BCL-72	chr8	99481683	99481683	A	G	exonic	VPS13B	44	43	0.4943	Missense_Mutation	p.T1251A	Non-Targeted Panel Gene	Adult HGBCL, NOS
BCL-72	chr5	178249151	178249151	C	A	exonic	COL23A1	45	44	0.4944	Missense_Mutation	p.G372V	Non-Targeted Panel Gene	Adult HGBCL, NOS
BCL-72	chr7	3301754	3301754	G	T	exonic	SDK1	51	50	0.495	Missense_Mutation	p.E56D	Non-Targeted Panel Gene	Adult HGBCL, NOS
BCL-72	chr5	67196566	67196566	G	C	exonic	CD180	34	34	0.5	Missense_Mutation	p.Q26E	Non-Targeted Panel Gene	Adult HGBCL, NOS
BCL-72	chr7	27199650	27199650	C	T	exonic	HOXA13	50	50	0.5	Missense_Mutation	p.G143D	Non-Targeted Panel Gene	Adult HGBCL, NOS
BCL-72	chr1	110339519	110339519	C	G	exonic	RBM15	42	42	0.5	Missense_Mutation	p.D38E	Non-Targeted Panel Gene	Adult HGBCL, NOS
BCL-72	chr1	68153593	68153593	G	T	exonic	WLS	44	44	0.5	Missense_Mutation	p.P243T	Non-Targeted Panel Gene	Adult HGBCL, NOS
BCL-72	chr1	99689245	99689245	C	A	exonic	PALMD	39	40	0.5063	Missense_Mutation	p.P329T	Non-Targeted Panel Gene	Adult HGBCL, NOS
BCL-72	chr14	60154060	60154060	C	T	exonic	DHRS7	36	37	0.5068	Missense_Mutation	p.G98S	Non-Targeted Panel Gene	Adult HGBCL, NOS
BCL-72	chr12	56254913	56254913	C	T	exonic	ANKRD52	42	44	0.5116	Missense_Mutation	p.V168I	Non-Targeted Panel Gene	Adult HGBCL, NOS
BCL-72	chr18	74129610	74129610	A	C	exonic	FBXO15	38	40	0.5128	Missense_Mutation	p.F118V	Non-Targeted Panel Gene	Adult HGBCL, NOS
BCL-72	chr14	94111768	94111768	C	T	exonic	IFI27	49	52	0.5149	Missense_Mutation	p.P29L	Non-Targeted Panel Gene	Adult HGBCL, NOS
BCL-72	chr22	24555669	24555669	C	T	exonic	GUCD1	53	57	0.5182	Missense_Mutation	p.A53T	Non-Targeted Panel Gene	Adult HGBCL, NOS
BCL-72	chr16	47258704	47258704	T	G	exonic	ITFG1	36	39	0.52	Missense_Mutation	p.T420P	Non-Targeted Panel Gene	Adult HGBCL, NOS
BCL-72	chr19	47260811	47260811	A	C	exonic	CCDC9	46	50	0.5208	Missense_Mutation	p.D145A	Non-Targeted Panel Gene	Adult HGBCL, NOS
BCL-72	chr1	176556917	176556917	C	T	exonic	PAPPA2	48	53	0.5248	Missense_Mutation	p.R199C	Non-Targeted Panel Gene	Adult HGBCL, NOS
BCL-72	chr16	53649112	53649112	G	T	exonic	RPGRIP1L	39	44	0.5301	Missense_Mutation	p.T719K	Non-Targeted Panel Gene	Adult HGBCL, NOS
BCL-72	chr16	21000461	21000461	T	G	exonic	DNAH3	38	43	0.5309	Missense_Mutation	p.T2062P	Non-Targeted Panel Gene	Adult HGBCL, NOS
BCL-72	chr5	131498595	131498595	G	A	exonic	RAPGEF6	36	41	0.5325	Missense_Mutation	p.R423C	Non-Targeted Panel Gene	Adult HGBCL, NOS
BCL-72	chr14	23402761	23402761	A	C	exonic	MYH6	42	50	0.5435	Missense_Mutation	p.F313C	Non-Targeted Panel Gene	Adult HGBCL, NOS
BCL-72	chr8	76855807	76855807	A	C	exonic	ZFHX4	29	35	0.5469	Missense_Mutation	p.L2962F	Non-Targeted Panel Gene	Adult HGBCL, NOS
BCL-72	chr20	36770902	36770902	G	A	exonic	DSN1	43	52	0.5474	Missense_Mutation	p.S109L	Non-Targeted Panel Gene	Adult HGBCL, NOS
BCL-72	chr17	40351953	40351953	G	C	exonic	RARA	51	63	0.5526	Missense_Mutation	p.K171N	Non-Targeted Panel Gene	Adult HGBCL, NOS
BCL-72	chr16	519830	519830	T	C	exonic	RAB11FIP3	38	47	0.5529	Missense_Mutation	p.M600T	Non-Targeted Panel Gene	Adult HGBCL, NOS
BCL-72	chr8	72063519	72063519	A	C	exonic	TRPA1	29	36	0.5538	Missense_Mutation	p.I202S	Non-Targeted Panel Gene	Adult HGBCL, NOS
BCL-72	chr2	233960870	233960870	A	T	exonic	TRPM8	28	35	0.5556	Missense_Mutation	p.K486M	Non-Targeted Panel Gene	Adult HGBCL, NOS
BCL-72	chr8	93808880	93808880	A	T	exonic	TMEM67	40	51	0.5604	Missense_Mutation	p.D746V	Non-Targeted Panel Gene	Adult HGBCL, NOS
BCL-72	chr1	17230588	17230588	G	A	exonic	PADI1	45	59	0.5673	Missense_Mutation	p.G357D	Non-Targeted Panel Gene	Adult HGBCL, NOS
BCL-72	chr6	150830940	150830940	G	T	exonic	PLEKHG1	37	49	0.5698	Missense_Mutation	p.S610I	Non-Targeted Panel Gene	Adult HGBCL, NOS
BCL-72	chr15	48738313	48738313	A	G	exonic	CEP152	34	46	0.575	Missense_Mutation	p.L1690S	Non-Targeted Panel Gene	Adult HGBCL, NOS
BCL-72	chr12	108519432	108519432	C	A	exonic	FICD	34	48	0.5854	Missense_Mutation	p.P445H	Non-Targeted Panel Gene	Adult HGBCL, NOS
BCL-72	chr17	64485733	64485733	T	C	exonic	POLG2	34	51	0.6	Missense_Mutation	p.R369G	Non-Targeted Panel Gene	Adult HGBCL, NOS
BCL-72	chr1	201387145	201387145	C	A	exonic	LAD1	41	62	0.6019	Missense_Mutation	p.K72N	Non-Targeted Panel Gene	Adult HGBCL, NOS
BCL-72	chr2	27132652	27132652	C	T	exonic	PREB	30	47	0.6104	Missense_Mutation	p.E235K	Non-Targeted Panel Gene	Adult HGBCL, NOS
BCL-72	chr11	9784428	9784428	C	T	exonic	SBF2	39	62	0.6139	Missense_Mutation	p.G1748R	Non-Targeted Panel Gene	Adult HGBCL, NOS
BCL-72	chr16	50779818	50779818	G	A	exonic	CYLD	25	43	0.6324	Missense_Mutation	p.G431E	Non-Targeted Panel Gene	Adult HGBCL, NOS
BCL-72	chr1	182489056	182489056	A	C	exonic	RGSL1	40	73	0.646	Missense_Mutation	p.K524T	Non-Targeted Panel Gene	Adult HGBCL, NOS
BCL-72	chr7	150947377	150947377	G	A	exonic	KCNH2	55	103	0.6519	Missense_Mutation	p.R1035W	Non-Targeted Panel Gene	Adult HGBCL, NOS
BCL-72	chr1	176690279	176690279	G	T	exonic	PAPPA2	26	55	0.679	Missense_Mutation	p.E760D	Non-Targeted Panel Gene	Adult HGBCL, NOS
BCL-72	chr2	219629278	219629278	C	T	exonic	SLC4A3	9	33	0.7857	Missense_Mutation	p.P118S	Non-Targeted Panel Gene	Adult HGBCL, NOS
BCL-72	chr6	127829583	127829583	T	G	exonic	THEMIS	9	35	0.7955	Missense_Mutation	p.N104T	Non-Targeted Panel Gene	Adult HGBCL, NOS
BCL-72	chr2	219250720	219250720	C	T	exonic	TUBA4A	4	39	0.907	Missense_Mutation	p.D327N	Non-Targeted Panel Gene	Adult HGBCL, NOS
BCL-72	chr1	1232427	1232427	C	A	exonic	B3GALT6	9	109	0.9237	Missense_Mutation	p.P50H	Non-Targeted Panel Gene	Adult HGBCL, NOS
BCL-72	chr12	32850772	32850772	T	C	exonic	PKP2	0	70	1	Missense_Mutation	p.I458V	Non-Targeted Panel Gene	Adult HGBCL, NOS
BCL-72	chr6	156778287	156778287	C	T	exonic	ARID1B	76	43	0.3613	Nonsense_Mutation	p.Q120X	Targerted Panel Gene	Adult HGBCL, NOS
BCL-72	chr6	56632931	56632931	G	C	exonic	DST	39	33	0.4583	Nonsense_Mutation	p.S1210X	Targerted Panel Gene	Adult HGBCL, NOS
BCL-72	chr17	1730924	1730924	C	G	exonic	WDR81	105	7	0.0625	Nonsense_Mutation	p.Y264X	Non-Targeted Panel Gene	Adult HGBCL, NOS
BCL-72	chrX	105195619	105195619	G	A	exonic	IL1RAPL2	78	29	0.271	Nonsense_Mutation	p.W76X	Non-Targeted Panel Gene	Adult HGBCL, NOS
BCL-72	chrX	12718175	12718175	A	T	exonic	FRMPD4	93	39	0.2955	Nonsense_Mutation	p.R1117X	Non-Targeted Panel Gene	Adult HGBCL, NOS
BCL-72	chr13	32398489	32398489	A	T	exonic	BRCA2	44	32	0.4211	Nonsense_Mutation	p.K3326X	Non-Targeted Panel Gene	Adult HGBCL, NOS
BCL-72	chr15	58901266	58901266	C	A	exonic	SLTM	40	45	0.5294	Nonsense_Mutation	p.E195X	Non-Targeted Panel Gene	Adult HGBCL, NOS
BCL-73	chr7	7673311	7673325	AGCAGCAGCAGCA	-	splicing	UMAD1	34	12	0.2553	Splice_Site	.	Non-Targeted Panel Gene	Adult HGBCL, NOS

BCL-73	chr12	49049837	49049864	CTGGACTAACATCC	-	exonic	KMT2D	51	12	0.1905	Frame_Shift_Del	p.L1242fs	Targerted Panel Gene	Adult HGBCL, NOS
BCL-73	chr10	101010782	101010782	T	-	exonic	PDZD7	29	21	0.42	Frame_Shift_Del	p.S703fs	Non-Targeted Panel Gene	Adult HGBCL, NOS
BCL-73	chr9	97662100	97662103	CTTG	-	exonic	NCBP1	38	30	0.4412	Frame_Shift_Del	p.H553fs	Non-Targeted Panel Gene	Adult HGBCL, NOS
BCL-73	chr17	47976759	47976760	CT	-	exonic	CDK5RAP3	21	24	0.5333	Frame_Shift_Del	p.D282fs	Non-Targeted Panel Gene	Adult HGBCL, NOS
BCL-73	chr4	117084337	117084337	-	CA	exonic	TRAM1L1	10	43	0.8113	Frame_Shift_Ins	p.E353fs	Non-Targeted Panel Gene	Adult HGBCL, NOS
BCL-73	chr1	62207433	62207438	GAAAAT	-	exonic	L1TD1	35	20	0.3636	In_Frame_Del	p.269_270del	Non-Targeted Panel Gene	Adult HGBCL, NOS
BCL-73	chr2	219309316	219309324	CGCCGCGGG	-	exonic	PTPRN	25	22	0.4681	In_Frame_Del	p.3_6del	Non-Targeted Panel Gene	Adult HGBCL, NOS
BCL-73	chr2	201757683	201757685	ACC	-	exonic	ALS2	32	33	0.5	In_Frame_Del	p.396_397del	Non-Targeted Panel Gene	Adult HGBCL, NOS
BCL-73	chrX	71141301	71141301	-	CAGCAACACCAG	exonic	MED12	41	18	0.2769	In_Frame_Ins	p.Q2113delinsQQQ	Targerted Panel Gene	Adult HGBCL, NOS
BCL-73	chr3	12004751	12004751	-	GCCCGCGCCGCAG	exonic	SYN2	71	11	0.1294	In_Frame_Ins	p.A67delinsAPAPQ	Non-Targeted Panel Gene	Adult HGBCL, NOS
BCL-73	chr3	38140541	38140541	C	G	exonic	MYD88	49	26	0.3467	Missense_Mutation	p.S219C	Targerted Panel Gene	Adult HGBCL, NOS
BCL-73	chr6	117387987	117387987	A	C	exonic	ROS1	57	6	0.0952	Missense_Mutation	p.S603A	Targerted Panel Gene	Adult HGBCL, NOS
BCL-73	chr20	32437205	32437205	C	T	exonic	ASXL1	34	28	0.4516	Missense_Mutation	p.T1498M	Targerted Panel Gene	Adult HGBCL, NOS
BCL-73	chr2	1660973	1660973	T	C	exonic	PXDN	29	25	0.463	Missense_Mutation	p.N582S	Targerted Panel Gene	Adult HGBCL, NOS
BCL-73	chr1	216084770	216084770	G	T	exonic	USH2A	32	33	0.5077	Missense_Mutation	p.Q1699K	Targerted Panel Gene	Adult HGBCL, NOS
BCL-73	chr16	72959353	72959353	C	A	exonic	ZFHX3	27	31	0.5345	Missense_Mutation	p.D265Y	Targerted Panel Gene	Adult HGBCL, NOS
BCL-73	chr3	52506185	52506185	C	T	exonic	STAB1	37	67	0.6442	Missense_Mutation	p.L589F	Targerted Panel Gene	Adult HGBCL, NOS
BCL-73	chr6	119189835	119189835	T	C	exonic	MAN1A1	43	4	0.0851	Missense_Mutation	p.I459V	Non-Targeted Panel Gene	Adult HGBCL, NOS
BCL-73	chr6	7229331	7229331	G	A	exonic	RREB1	58	14	0.1944	Missense_Mutation	p.S411N	Non-Targeted Panel Gene	Adult HGBCL, NOS
BCL-73	chr3	39136425	39136425	G	A	exonic	TTC21A	70	25	0.2632	Missense_Mutation	p.A1012T	Non-Targeted Panel Gene	Adult HGBCL, NOS
BCL-73	chr7	70764919	70764919	C	T	exonic	AUTS2	20	9	0.3103	Missense_Mutation	p.A461V	Non-Targeted Panel Gene	Adult HGBCL, NOS
BCL-73	chr3	64099608	64099608	C	T	exonic	PRICKLE2	59	27	0.314	Missense_Mutation	p.V660M	Non-Targeted Panel Gene	Adult HGBCL, NOS
BCL-73	chr16	87415312	87415312	G	A	exonic	ZCCHC14	39	19	0.3276	Missense_Mutation	p.A343V	Non-Targeted Panel Gene	Adult HGBCL, NOS
BCL-73	chrX	143630513	143630513	A	C	exonic	SLITRK4	58	29	0.3333	Missense_Mutation	p.L199R	Non-Targeted Panel Gene	Adult HGBCL, NOS
BCL-73	chr9	138023553	138023553	C	T	exonic	CACNA1B	61	31	0.337	Missense_Mutation	p.A937V	Non-Targeted Panel Gene	Adult HGBCL, NOS
BCL-73	chrX	50384187	50384187	G	A	exonic	DGKK	51	26	0.3377	Missense_Mutation	p.H844Y	Non-Targeted Panel Gene	Adult HGBCL, NOS
BCL-73	chr2	165917326	165917326	T	G	exonic	TTC21B	49	25	0.3378	Missense_Mutation	p.E610D	Non-Targeted Panel Gene	Adult HGBCL, NOS
BCL-73	chr7	15626901	15626901	A	C	exonic	MEOX2	32	17	0.3469	Missense_Mutation	p.Y179D	Non-Targeted Panel Gene	Adult HGBCL, NOS
BCL-73	chr3	42690875	42690875	G	T	exonic	KLHL40	58	31	0.3483	Missense_Mutation	p.A542S	Non-Targeted Panel Gene	Adult HGBCL, NOS
BCL-73	chr5	665244	665244	G	A	exonic	TPPP	35	20	0.3636	Missense_Mutation	p.T173M	Non-Targeted Panel Gene	Adult HGBCL, NOS
BCL-73	chr2	54612327	54612327	G	A	exonic	SPTBN1	28	17	0.3778	Missense_Mutation	p.R156H	Non-Targeted Panel Gene	Adult HGBCL, NOS
BCL-73	chr12	11186762	11186762	A	T	exonic	TAS2R42	38	24	0.3871	Missense_Mutation	p.L59Q	Non-Targeted Panel Gene	Adult HGBCL, NOS
BCL-73	chr15	90358912	90358912	C	T	exonic	ZNF774	33	21	0.3889	Missense_Mutation	p.L56F	Non-Targeted Panel Gene	Adult HGBCL, NOS
BCL-73	chr4	166055166	166055166	T	C	exonic	TLL1	36	23	0.3898	Missense_Mutation	p.Y539H	Non-Targeted Panel Gene	Adult HGBCL, NOS
BCL-73	chr3	133777188	133777188	G	A	exonic	TF	55	36	0.3956	Missense_Mutation	p.G671E	Non-Targeted Panel Gene	Adult HGBCL, NOS
BCL-73	chr2	221564258	221564258	A	C	exonic	EPHA4	30	20	0.4	Missense_Mutation	p.I99S	Non-Targeted Panel Gene	Adult HGBCL, NOS
BCL-73	chr17	9728798	9728798	G	T	exonic	USP43	41	28	0.4058	Missense_Mutation	p.E1060D	Non-Targeted Panel Gene	Adult HGBCL, NOS
BCL-73	chr8	144511990	144511990	C	T	exonic	RECQL4	39	27	0.4091	Missense_Mutation	p.G1105D	Non-Targeted Panel Gene	Adult HGBCL, NOS
BCL-73	chr1	175007112	175007112	T	C	exonic	CACYBP	36	25	0.4098	Missense_Mutation	p.L116S	Non-Targeted Panel Gene	Adult HGBCL, NOS
BCL-73	chr7	123452838	123452838	A	G	exonic	IQUB	44	31	0.4133	Missense_Mutation	p.F761L	Non-Targeted Panel Gene	Adult HGBCL, NOS
BCL-73	chr8	54629452	54629452	G	T	exonic	RP1	31	22	0.4151	Missense_Mutation	p.S1857I	Non-Targeted Panel Gene	Adult HGBCL, NOS
BCL-73	chr10	815012	815012	G	A	exonic	LARP4B	31	23	0.4259	Missense_Mutation	p.S585L	Non-Targeted Panel Gene	Adult HGBCL, NOS
BCL-73	chr11	5947984	5947984	C	A	exonic	OR56A3	42	32	0.4324	Missense_Mutation	p.S213Y	Non-Targeted Panel Gene	Adult HGBCL, NOS
BCL-73	chrX	70740769	70740769	T	G	exonic	TEX11	34	26	0.4333	Missense_Mutation	p.N274H	Non-Targeted Panel Gene	Adult HGBCL, NOS
BCL-73	chrX	106787996	106787996	T	A	exonic	RNF128	44	34	0.4359	Missense_Mutation	p.C295S	Non-Targeted Panel Gene	Adult HGBCL, NOS
BCL-73	chr22	24036095	24036095	A	G	exonic	CABIN1	29	23	0.4423	Missense_Mutation	p.I4V	Non-Targeted Panel Gene	Adult HGBCL, NOS
BCL-73	chr22	23834152	23834152	G	A	exonic	SMARCB1	31	25	0.4464	Missense_Mutation	p.R377H	Non-Targeted Panel Gene	Adult HGBCL, NOS
BCL-73	chr19	12792462	12792462	G	T	exonic	JUNB	32	26	0.4483	Missense_Mutation	p.G231C	Non-Targeted Panel Gene	Adult HGBCL, NOS
BCL-73	chr7	117590400	117590400	G	C	exonic	CFTR	40	33	0.4521	Missense_Mutation	p.G576A	Non-Targeted Panel Gene	Adult HGBCL, NOS
BCL-73	chr8	144058849	144058849	C	A	exonic	OPLAH	32	27	0.4576	Missense_Mutation	p.A171S	Non-Targeted Panel Gene	Adult HGBCL, NOS
BCL-73	chrX	50627636	50627636	T	C	exonic	SHROOM4	34	29	0.4603	Missense_Mutation	p.R979G	Non-Targeted Panel Gene	Adult HGBCL, NOS
BCL-73	chr14	35834906	35834906	T	C	exonic	BRMS1L	34	30	0.4688	Missense_Mutation	p.S142P	Non-Targeted Panel Gene	Adult HGBCL, NOS
BCL-73	chr5	21783444	21783444	C	T	exonic	CDH12	36	32	0.4706	Missense_Mutation	p.G436E	Non-Targeted Panel Gene	Adult HGBCL, NOS
BCL-73	chr14	24240746	24240746	G	T	exonic	TINF2	28	25	0.4717	Missense_Mutation	p.S245Y	Non-Targeted Panel Gene	Adult HGBCL, NOS
BCL-73	chr15	78098089	78098089	C	T	exonic	SH2D7	31	28	0.4746	Missense_Mutation	p.P143S	Non-Targeted Panel Gene	Adult HGBCL, NOS
BCL-73	chr2	178598768	178598768	G	A	exonic	TTN	35	32	0.4776	Missense_Mutation	p.A18981V	Non-Targeted Panel Gene	Adult HGBCL, NOS
BCL-73	chr10	3165456	3165456	A	T	exonic	PITRM1	26	24	0.48	Missense_Mutation	p.L164M	Non-Targeted Panel Gene	Adult HGBCL, NOS
BCL-73	chr2	233542299	233542299	G	A	exonic	USP40	36	34	0.4857	Missense_Mutation	p.P356L	Non-Targeted Panel Gene	Adult HGBCL, NOS
BCL-73	chr11	31307717	31307717	G	A	exonic	DCDC1	33	32	0.4923	Missense_Mutation	p.S119L	Non-Targeted Panel Gene	Adult HGBCL, NOS
BCL-73	chr5	140691655	140691655	C	G	exonic	HARS2	37	38	0.5067	Missense_Mutation	p.L3V	Non-Targeted Panel Gene	Adult HGBCL, NOS
BCL-73	chr1	245685520	245685520	C	T	exonic	KIF26B	31	32	0.5079	Missense_Mutation	p.A846V	Non-Targeted Panel Gene	Adult HGBCL, NOS
BCL-73	chr19	35787198	35787198	C	T	exonic	ARHGAP33	32	34	0.5152	Missense_Mutation	p.P717L	Non-Targeted Panel Gene	Adult HGBCL, NOS
BCL-73	chr5	33576377	33576377	T	C	exonic	ADAMTS12	27	29	0.5179	Missense_Mutation	p.M1217V	Non-Targeted Panel Gene	Adult HGBCL, NOS
BCL-73	chr1	245686296	245686296	C	G	exonic	KIF26B	39	42	0.5185	Missense_Mutation	p.P1105A	Non-Targeted Panel Gene	Adult HGBCL, NOS
BCL-73	chr19	11380888	11380888	G	T	exonic	EPOR	25	27	0.5192	Missense_Mutation	p.R275S	Non-Targeted Panel Gene	Adult HGBCL, NOS
BCL-73	chr12	754160	754160	C	G	exonic	WNK1	36	39	0.52	Missense_Mutation	p.Q199E	Non-Targeted Panel Gene	Adult HGBCL, NOS
BCL-73	chr9	121090308	121090308	C	T	exonic	CNTRL	29	32	0.5246	Missense_Mutation	p.T84I	Non-Targeted Panel Gene	Adult HGBCL, NOS
BCL-73	chr19	35868751	35868751	A	C	exonic	APLP1	29	33	0.5323	Missense_Mutation	p.S39R	Non-Targeted Panel Gene	Adult HGBCL, NOS
BCL-73	chr12	80708053	80708053	G	T	exonic	MYF6	29	33	0.5323	Missense_Mutation	p.A112S	Non-Targeted Panel Gene	Adult HGBCL, NOS
BCL-73	chr9	137279854	137279854	G	A	exonic	TOR4A	39	45	0.5357	Missense_Mutation	p.A389T	Non-Targeted Panel Gene	Adult HGBCL, NOS
BCL-73	chr9	128257482	128257482	C	T	exonic	GOLGA2	23	27	0.54	Missense_Mutation	p.G894D	Non-Targeted Panel Gene	Adult HGBCL, NOS
BCL-73	chr15	72350584	72350584	G	A	exonic	HEXA	33	39	0.5417	Missense_Mutation	p.R258W	Non-Targeted Panel Gene	Adult HGBCL, NOS
BCL-73	chr6	31056638	31056638	C	T	exonic	HCG22	37	47	0.5595	Missense_Mutation	p.P202S	Non-Targeted Panel Gene	Adult HGBCL, NOS
BCL-73	chr3	39143393	39143393	G	A	exonic	CSRNP1	41	53	0.5638	Missense_Mutation	p.L478F	Non-Targeted Panel Gene	Adult HGBCL, NOS
BCL-73	chr21	14965389	14965389	A	G	exonic	NRIP1	25	34	0.5763	Missense_Mutation	p.V935A	Non-Targeted Panel Gene	Adult HGBCL, NOS
BCL-73	chr1	151519262	151519262	C	T	exonic	CGN	30	41	0.5775	Missense_Mutation	p.S248L	Non-Targeted Panel Gene	Adult HGBCL, NOS
BCL-73	chr17	11797682	11797682	C	G	exonic	DNAH9	14	23	0.6216	Missense_Mutation	p.P2770R	Non-Targeted Panel Gene	Adult HGBCL, NOS
BCL-73	chr16	84017219	84017219	C	T	exonic	SLC38A8	19	32	0.6275	Missense_Mutation	p.D292N	Non-Targeted Panel Gene	Adult HGBCL, NOS
BCL-73	chr19	45224876	45224876	C	T	exonic	EXOC3L2	16	36	0.6923	Missense_Mutation	p.E148K	Non-Targeted Panel Gene	Adult HGBCL, NOS
BCL-73	chr1	22647383	22647383	G	T	exonic	C1QC	8	21	0.7241	Missense_Mutation	p.R113I	Non-Targeted Panel Gene	Adult HGBCL, NOS
BCL-73	chr20	5302677	5302677	A	C	exonic	PROKR2	12	36	0.75	Missense_Mutation	p.L173R	Non-Targeted Panel Gene	Adult HGBCL, NOS
BCL-73	chr1	244856838	244856838	G	A	exonic	HNRNPU	42	22	0.3438	Nonsense_Mutation	p.Q545X	Non-Targeted Panel Gene	Adult HGBCL, NOS

BCL-73	chr19	42099794	42099794	G	A	exonic	POU2F2	29	18	0.383	Nonsense_Mutation	p.Q111X	Non-Targeted Panel Gene	Adult HGBCL, NOS
BCL-73	chr6	31669842	31669842	C	G	exonic	CSNK2B	27	50	0.6494	Nonsense_Mutation	p.Y188X	Non-Targeted Panel Gene	Adult HGBCL, NOS
BCL-74	chr7	84134792	84134792	A	G	splicing	SEMA3A	41	22	0.3492	Splice_Site	.	Non-Targeted Panel Gene	Adult HGBCL, NOS
BCL-74	chr16	81037476	81037476	G	A	splicing	ATMIN	49	28	0.3636	Splice_Site	.	Non-Targeted Panel Gene	Adult HGBCL, NOS
BCL-74	chr12	11650161	11650161	G	A	splicing	ETV6	41	35	0.4605	Splice_Site	.	Non-Targeted Panel Gene	Adult HGBCL, NOS
BCL-74	chr12	19440371	19440371	G	A	splicing	AEBP2	38	33	0.4648	Splice_Site	.	Non-Targeted Panel Gene	Adult HGBCL, NOS
BCL-74	chr1	111140308	111140308	G	A	splicing	CEPT1	39	34	0.4658	Splice_Site	.	Non-Targeted Panel Gene	Adult HGBCL, NOS
BCL-74	chr3	177051667	177051681	CATTACGGCATCTA	-	exonic	TBL1XR1	52	14	0.2121	Frame_Shift_Del	wholegene	Targerted Panel Gene	Adult HGBCL, NOS
BCL-74	chr6	146304667	146304667	-	GGGAATC	exonic	GRM1	49	22	0.3099	Frame_Shift_Ins	p.G336fs	Non-Targeted Panel Gene	Adult HGBCL, NOS
BCL-74	chr19	41880861	41880905	TCCCAGGGCCTGAA	-	exonic	CD79A	48	8	0.1429	In_Frame_Del	p.152_164del	Targerted Panel Gene	Adult HGBCL, NOS
BCL-74	chr7	100954669	100954669	T	A	exonic	MUC3A	69	23	0.25	Missense_Mutation	p.S964T	Targerted Panel Gene	Adult HGBCL, NOS
BCL-74	chr7	148811635	148811635	T	C	exonic	EZH2	51	25	0.3289	Missense_Mutation	p.Y646C	Targerted Panel Gene	Adult HGBCL, NOS
BCL-74	chrX	141907596	141907596	C	A	exonic	MAGEC1	28	19	0.4043	Missense_Mutation	p.P731H	Targerted Panel Gene	Adult HGBCL, NOS
BCL-74	chrX	80722602	80722602	T	G	exonic	BRWD3	35	24	0.4068	Missense_Mutation	p.K612N	Targerted Panel Gene	Adult HGBCL, NOS
BCL-74	chr22	22888163	22888163	G	A	exonic	IGLL5	52	38	0.4222	Missense_Mutation	p.A2T	Targerted Panel Gene	Adult HGBCL, NOS
BCL-74	chrX	31178687	31178687	T	A	exonic	DMD	41	32	0.4384	Missense_Mutation	p.E3279V	Targerted Panel Gene	Adult HGBCL, NOS
BCL-74	chr12	121804738	121804738	A	G	exonic	SETD1B	35	42	0.5455	Missense_Mutation	p.M1V	Targerted Panel Gene	Adult HGBCL, NOS
BCL-74	chrX	18174236	18174236	T	G	exonic	BEND2	56	4	0.0667	Missense_Mutation	p.K592T	Non-Targeted Panel Gene	Adult HGBCL, NOS
BCL-74	chr3	165190743	165190743	G	T	exonic	SLITRK3	69	5	0.0676	Missense_Mutation	p.P30T	Non-Targeted Panel Gene	Adult HGBCL, NOS
BCL-74	chrX	147948778	147948778	T	C	exonic	FMR1	48	18	0.2727	Missense_Mutation	p.V521A	Non-Targeted Panel Gene	Adult HGBCL, NOS
BCL-74	chr4	128891112	128891112	G	C	exonic	SCLT1	47	20	0.2985	Missense_Mutation	p.H619D	Non-Targeted Panel Gene	Adult HGBCL, NOS
BCL-74	chr12	101661229	101661229	C	A	exonic	MYBPC1	49	23	0.3194	Missense_Mutation	p.P667T	Non-Targeted Panel Gene	Adult HGBCL, NOS
BCL-74	chrX	9725763	9725763	T	C	exonic	GPR143	53	26	0.3291	Missense_Mutation	p.T400A	Non-Targeted Panel Gene	Adult HGBCL, NOS
BCL-74	chrX	26217977	26217977	G	T	exonic	MAGEB5	42	23	0.3538	Missense_Mutation	p.D226Y	Non-Targeted Panel Gene	Adult HGBCL, NOS
BCL-74	chr9	97565951	97565951	C	T	exonic	TMOD1	45	26	0.3662	Missense_Mutation	p.A241V	Non-Targeted Panel Gene	Adult HGBCL, NOS
BCL-74	chr8	87873192	87873192	G	C	exonic	DCAF4L2	34	20	0.3704	Missense_Mutation	p.S260R	Non-Targeted Panel Gene	Adult HGBCL, NOS
BCL-74	chr4	73094203	73094203	T	C	exonic	ANKRD17	45	27	0.375	Missense_Mutation	p.T1735A	Non-Targeted Panel Gene	Adult HGBCL, NOS
BCL-74	chr18	78997164	78997164	G	A	exonic	SALL3	44	27	0.3803	Missense_Mutation	p.A1249T	Non-Targeted Panel Gene	Adult HGBCL, NOS
BCL-74	chrX	72140709	72140709	C	T	exonic	NHSL2	39	25	0.3906	Missense_Mutation	p.S1054L	Non-Targeted Panel Gene	Adult HGBCL, NOS
BCL-74	chr12	29783675	29783675	G	A	exonic	TMTC1	41	27	0.3971	Missense_Mutation	p.P26L	Non-Targeted Panel Gene	Adult HGBCL, NOS
BCL-74	chr3	31980942	31980942	C	T	exonic	OSBPL10	44	29	0.3973	Missense_Mutation	p.V80M	Non-Targeted Panel Gene	Adult HGBCL, NOS
BCL-74	chr6	13205860	13205860	C	T	exonic	PHACTR1	36	24	0.4	Missense_Mutation	p.S237L	Non-Targeted Panel Gene	Adult HGBCL, NOS
BCL-74	chrX	111776531	111776531	T	G	exonic	TRPC5	37	25	0.4032	Missense_Mutation	p.S902R	Non-Targeted Panel Gene	Adult HGBCL, NOS
BCL-74	chr3	31981109	31981109	G	A	exonic	OSBPL10	42	29	0.4085	Missense_Mutation	p.A24V	Non-Targeted Panel Gene	Adult HGBCL, NOS
BCL-74	chr1	44739943	44739943	A	G	exonic	KIF2C	54	39	0.4194	Missense_Mutation	p.D4G	Non-Targeted Panel Gene	Adult HGBCL, NOS
BCL-74	chr11	67436275	67436275	C	T	exonic	PTPRCAP	62	48	0.4364	Missense_Mutation	p.D27N	Non-Targeted Panel Gene	Adult HGBCL, NOS
BCL-74	chr13	36112117	36112117	C	A	exonic	DCLK1	38	30	0.4412	Missense_Mutation	p.A159S	Non-Targeted Panel Gene	Adult HGBCL, NOS
BCL-74	chr15	90228323	90228323	C	G	exonic	SEMA4B	42	34	0.4474	Missense_Mutation	p.P732A	Non-Targeted Panel Gene	Adult HGBCL, NOS
BCL-74	chr19	846348	846348	G	A	exonic	PRTN3	48	39	0.4483	Missense_Mutation	p.V191I	Non-Targeted Panel Gene	Adult HGBCL, NOS
BCL-74	chr2	200490545	200490545	A	C	exonic	KCTD18	36	30	0.4545	Missense_Mutation	p.L279W	Non-Targeted Panel Gene	Adult HGBCL, NOS
BCL-74	chr3	31981127	31981127	C	T	exonic	OSBPL10	41	35	0.4545	Missense_Mutation	p.S18N	Non-Targeted Panel Gene	Adult HGBCL, NOS
BCL-74	chr17	10400756	10400756	T	G	exonic	MYH8	39	33	0.4583	Missense_Mutation	p.E1123D	Non-Targeted Panel Gene	Adult HGBCL, NOS
BCL-74	chr20	56525908	56525908	A	C	exonic	FAM209A	36	31	0.4627	Missense_Mutation	p.E118D	Non-Targeted Panel Gene	Adult HGBCL, NOS
BCL-74	chr12	20369508	20369508	C	T	exonic	PDE3A	37	32	0.4638	Missense_Mutation	p.A75V	Non-Targeted Panel Gene	Adult HGBCL, NOS
BCL-74	chr1	6554312	6554312	T	C	exonic	NOL9	40	35	0.4667	Missense_Mutation	p.E64G	Non-Targeted Panel Gene	Adult HGBCL, NOS
BCL-74	chrX	107154209	107154209	C	T	exonic	NUP62CL	24	21	0.4667	Missense_Mutation	p.E78K	Non-Targeted Panel Gene	Adult HGBCL, NOS
BCL-74	chr3	31981130	31981130	C	T	exonic	OSBPL10	41	37	0.4744	Missense_Mutation	p.S17N	Non-Targeted Panel Gene	Adult HGBCL, NOS
BCL-74	chr9	113988544	113988544	G	A	exonic	ZNF618	35	32	0.4776	Missense_Mutation	p.V101M	Non-Targeted Panel Gene	Adult HGBCL, NOS
BCL-74	chr3	35721688	35721688	G	A	exonic	ARPP21	30	28	0.4828	Missense_Mutation	p.R360K	Non-Targeted Panel Gene	Adult HGBCL, NOS
BCL-74	chr17	39213038	39213038	G	A	exonic	STAC2	35	33	0.4853	Missense_Mutation	p.S363F	Non-Targeted Panel Gene	Adult HGBCL, NOS
BCL-74	chr11	56542777	56542777	G	C	exonic	OR5M11	25	24	0.4898	Missense_Mutation	p.L161V	Non-Targeted Panel Gene	Adult HGBCL, NOS
BCL-74	chr12	51981091	51981091	A	T	exonic	ACVR1B	34	33	0.4925	Missense_Mutation	p.I235L	Non-Targeted Panel Gene	Adult HGBCL, NOS
BCL-74	chr16	31723349	31723349	G	T	exonic	ZNF720	28	28	0.5	Missense_Mutation	p.Q74H	Non-Targeted Panel Gene	Adult HGBCL, NOS
BCL-74	chr20	36815231	36815231	G	C	exonic	SOGA1	33	35	0.5147	Missense_Mutation	p.C499W	Non-Targeted Panel Gene	Adult HGBCL, NOS
BCL-74	chr5	141868997	141868997	G	A	exonic	PCDH1	41	31	0.4306	Nonsense_Mutation	p.Q159X	Non-Targeted Panel Gene	Adult HGBCL, NOS
BCL-74	chr15	41072025	41072025	G	A	exonic	INO80	32	25	0.431	Nonsense_Mutation	p.R477X	Non-Targeted Panel Gene	Adult HGBCL, NOS
BCL-74	chr3	38141150	38141150	T	C	exonic	MYD88	42	27	0.3913	Nonstop_Mutation	p.L265P	Targerted Panel Gene	Adult HGBCL, NOS
BCL-75	chr9	13279563	13279563	C	T	splicing	MPDZ	51	12	0.1905	Splice_Site	.	Targerted Panel Gene	Adult HGBCL, NOS
BCL-75	chr3	136477414	136477414	T	G	splicing	STAG1	54	13	0.194	Splice_Site	.	Non-Targeted Panel Gene	Adult HGBCL, NOS
BCL-75	chr12	6034686	6034686	A	G	splicing	VWF	69	22	0.2418	Splice_Site	.	Non-Targeted Panel Gene	Adult HGBCL, NOS
BCL-75	chr1	165665017	165665017	C	A	splicing	ALDH9A1	39	28	0.4179	Splice_Site	.	Non-Targeted Panel Gene	Adult HGBCL, NOS
BCL-75	chr2	164527137	164527137	T	C	splicing	GRB14	30	24	0.4444	Splice_Site	.	Non-Targeted Panel Gene	Adult HGBCL, NOS
BCL-75	chr11	57646749	57646749	A	C	splicing	YPEL4	27	31	0.5345	Splice_Site	.	Non-Targeted Panel Gene	Adult HGBCL, NOS
BCL-75	chr19	23932865	23932866	CT	-	exonic	ZNF726	37	31	0.4559	Frame_Shift_Del	p.T250fs	Targerted Panel Gene	Adult HGBCL, NOS
BCL-75	chr15	58894144	58894145	TT	-	exonic	SLTM	39	22	0.3607	Frame_Shift_Del	p.K476fs	Non-Targeted Panel Gene	Adult HGBCL, NOS
BCL-75	chr8	69832420	69832429	GCAGAGGGAC	-	exonic	SLCO5A1	30	17	0.3617	Frame_Shift_Del	p.S82fs	Non-Targeted Panel Gene	Adult HGBCL, NOS
BCL-75	chr20	59007101	59007110	GCCGCCACCC	-	exonic	CTSZ	40	23	0.3651	Frame_Shift_Del	p.G7fs	Non-Targeted Panel Gene	Adult HGBCL, NOS
BCL-75	chr8	58146687	58146687	G	-	exonic	FAM110B	34	20	0.3704	Frame_Shift_Del	p.D153fs	Non-Targeted Panel Gene	Adult HGBCL, NOS
BCL-75	chr6	109791505	109791506	CT	-	exonic	FIG4	30	19	0.3878	Frame_Shift_Del	p.G770fs	Non-Targeted Panel Gene	Adult HGBCL, NOS
BCL-75	chr9	77019726	77019726	G	-	exonic	FOXB2	41	26	0.3881	Frame_Shift_Del	p.M24fs	Non-Targeted Panel Gene	Adult HGBCL, NOS
BCL-75	chr2	187496893	187496893	T	-	exonic	TFPI	32	31	0.4921	Frame_Shift_Del	p.M103fs	Non-Targeted Panel Gene	Adult HGBCL, NOS
BCL-75	chr4	105275610	105275610	-	A	exonic	TET2	45	16	0.2623	Frame_Shift_Ins	p.N1700fs	Targerted Panel Gene	Adult HGBCL, NOS
BCL-75	chr22	22893780	22893780	-	CT	exonic	IGLL5	9	20	0.6897	Frame_Shift_Ins	p.C96fs	Targerted Panel Gene	Adult HGBCL, NOS
BCL-75	chr19	48610755	48610755	-	A	exonic	FAM83E	40	21	0.3443	Frame_Shift_Ins	p.L186fs	Non-Targeted Panel Gene	Adult HGBCL, NOS
BCL-75	chr14	24856010	24856010	-	G	exonic	STXBP6	45	31	0.4079	Frame_Shift_Ins	p.L126fs	Non-Targeted Panel Gene	Adult HGBCL, NOS
BCL-75	chr22	31717153	31717155	CCG	-	exonic	PRR14L	51	26	0.3377	In_Frame_Del	p.228_229del	Non-Targeted Panel Gene	Adult HGBCL, NOS
BCL-75	chr19	48610759	48610764	TAGACA	-	exonic	FAM83E	35	20	0.3636	In_Frame_Del	p.183_185del	Non-Targeted Panel Gene	Adult HGBCL, NOS
BCL-75	chr5	95620847	95620847	-	GCCGCC	exonic	GPR150	44	24	0.3429	In_Frame_Ins	p.L191delinsLPP	Non-Targeted Panel Gene	Adult HGBCL, NOS
BCL-75	chr3	38140541	38140541	C	G	exonic	MYD88	45	16	0.2623	Missense_Mutation	p.S219C	Targerted Panel Gene	Adult HGBCL, NOS
BCL-75	chr7	100954669	100954669	T	A	exonic	MUC3A	50	13	0.2063	Missense_Mutation	p.S964T	Targerted Panel Gene	Adult HGBCL, NOS
BCL-75	chr4	125491067	125491067	C	A	exonic	FAT4	45	15	0.25	Missense_Mutation	p.L4749M	Targerted Panel Gene	Adult HGBCL, NOS
BCL-75	chr7	150467288	150467288	G	T	exonic	GIMAP8	45	15	0.25	Missense_Mutation	p.G197V	Targerted Panel Gene	Adult HGBCL, NOS

BCL-75	chr2	1648182	1648182	T	C	exonic	PXDN	42	24	0.3636	Missense_Mutation	p.K1200E	Targerted Panel Gene	Adult HGBCL, NOS
BCL-75	chr4	105269686	105269686	G	A	exonic	TET2	37	22	0.3729	Missense_Mutation	p.C1374Y	Targerted Panel Gene	Adult HGBCL, NOS
BCL-75	chr8	112224839	112224839	C	A	exonic	CSMD3	32	20	0.3846	Missense_Mutation	p.A3646S	Targerted Panel Gene	Adult HGBCL, NOS
BCL-75	chr16	2304004	2304004	A	T	exonic	ABCA3	34	27	0.4426	Missense_Mutation	p.F478I	Targerted Panel Gene	Adult HGBCL, NOS
BCL-75	chr1	23558988	23558988	G	T	exonic	ID3	37	31	0.4559	Missense_Mutation	p.S111Y	Targerted Panel Gene	Adult HGBCL, NOS
BCL-75	chr4	125490312	125490312	A	T	exonic	FAT4	22	19	0.4634	Missense_Mutation	p.E4497V	Targerted Panel Gene	Adult HGBCL, NOS
BCL-75	chr1	23558990	23558990	G	C	exonic	ID3	35	33	0.4853	Missense_Mutation	p.I110M	Targerted Panel Gene	Adult HGBCL, NOS
BCL-75	chr7	83162471	83162471	A	T	exonic	PCLO	29	28	0.4912	Missense_Mutation	p.M41K	Targerted Panel Gene	Adult HGBCL, NOS
BCL-75	chr12	57206493	57206493	T	C	exonic	LRP1	32	54	0.6279	Missense_Mutation	p.Y3871H	Targerted Panel Gene	Adult HGBCL, NOS
BCL-75	chrX	41343280	41343280	C	T	exonic	DDX3X	8	28	0.7778	Missense_Mutation	p.P203L	Targerted Panel Gene	Adult HGBCL, NOS
BCL-75	chr9	13176237	13176237	A	G	exonic	MPDZ	7	73	0.9125	Missense_Mutation	p.Y944H	Targerted Panel Gene	Adult HGBCL, NOS
BCL-75	chr14	34567729	34567729	T	C	exonic	SNX6	63	3	0.0455	Missense_Mutation	p.K387R	Non-Targeted Panel Gene	Adult HGBCL, NOS
BCL-75	chr6	63984444	63984444	C	G	exonic	EYS	54	4	0.069	Missense_Mutation	p.E2332Q	Non-Targeted Panel Gene	Adult HGBCL, NOS
BCL-75	chr4	70335559	70335559	G	C	exonic	CABS1	65	5	0.0714	Missense_Mutation	p.A174P	Non-Targeted Panel Gene	Adult HGBCL, NOS
BCL-75	chr2	185790555	185790555	T	G	exonic	FSIP2	62	5	0.0746	Missense_Mutation	p.L1140R	Non-Targeted Panel Gene	Adult HGBCL, NOS
BCL-75	chr14	102086217	102086217	A	T	exonic	HSP90AA1	74	6	0.075	Missense_Mutation	p.D176E	Non-Targeted Panel Gene	Adult HGBCL, NOS
BCL-75	chr2	161949241	161949241	C	A	exonic	SLC4A10	58	5	0.0794	Missense_Mutation	p.Q768K	Non-Targeted Panel Gene	Adult HGBCL, NOS
BCL-75	chr18	70127664	70127664	T	C	exonic	RTTN	55	5	0.0833	Missense_Mutation	p.D1074G	Non-Targeted Panel Gene	Adult HGBCL, NOS
BCL-75	chr19	1000606	1000606	C	G	exonic	GRIN3B	62	10	0.1389	Missense_Mutation	p.L57V	Non-Targeted Panel Gene	Adult HGBCL, NOS
BCL-75	chr5	6448957	6448957	G	A	exonic	UBE2QL1	51	9	0.15	Missense_Mutation	p.E22K	Non-Targeted Panel Gene	Adult HGBCL, NOS
BCL-75	chr19	38731137	38731137	A	T	exonic	CAPN12	45	10	0.1818	Missense_Mutation	p.S682T	Non-Targeted Panel Gene	Adult HGBCL, NOS
BCL-75	chr16	70251669	70251669	C	T	exonic	EXOSC6	68	16	0.1905	Missense_Mutation	p.G78S	Non-Targeted Panel Gene	Adult HGBCL, NOS
BCL-75	chr10	101131719	101131719	G	A	exonic	TLX1	48	13	0.2131	Missense_Mutation	p.G60R	Non-Targeted Panel Gene	Adult HGBCL, NOS
BCL-75	chr6	44251469	44251469	G	A	exonic	HSP90AB1	44	12	0.2143	Missense_Mutation	p.R392Q	Non-Targeted Panel Gene	Adult HGBCL, NOS
BCL-75	chr17	45115458	45115458	C	A	exonic	PLCD3	37	11	0.2292	Missense_Mutation	p.K482N	Non-Targeted Panel Gene	Adult HGBCL, NOS
BCL-75	chr4	1718347	1718347	G	A	exonic	TMEM129	46	14	0.2333	Missense_Mutation	p.A162V	Non-Targeted Panel Gene	Adult HGBCL, NOS
BCL-75	chr12	55332626	55332626	C	T	exonic	OR6C3	73	23	0.2371	Missense_Mutation	p.A309V	Non-Targeted Panel Gene	Adult HGBCL, NOS
BCL-75	chr5	151567339	151567339	G	T	exonic	FAT2	57	18	0.24	Missense_Mutation	p.F531L	Non-Targeted Panel Gene	Adult HGBCL, NOS
BCL-75	chr15	41757823	41757823	C	G	exonic	MGA	50	17	0.2537	Missense_Mutation	p.A2392G	Non-Targeted Panel Gene	Adult HGBCL, NOS
BCL-75	chr9	108855581	108855581	T	C	exonic	ACTL7B	29	11	0.275	Missense_Mutation	p.H117R	Non-Targeted Panel Gene	Adult HGBCL, NOS
BCL-75	chr21	30289432	30289432	G	T	exonic	KRTAP25-1	47	18	0.2769	Missense_Mutation	p.S20Y	Non-Targeted Panel Gene	Adult HGBCL, NOS
BCL-75	chr15	74956102	74956102	G	A	exonic	RPP25	42	17	0.2881	Missense_Mutation	p.S161L	Non-Targeted Panel Gene	Adult HGBCL, NOS
BCL-75	chr2	106073966	106073966	C	T	exonic	C2orf40	40	17	0.2982	Missense_Mutation	p.R70W	Non-Targeted Panel Gene	Adult HGBCL, NOS
BCL-75	chr2	235041086	235041086	C	T	exonic	SH3BP4	45	20	0.3077	Missense_Mutation	p.S106F	Non-Targeted Panel Gene	Adult HGBCL, NOS
BCL-75	chr12	84863464	84863464	C	A	exonic	SLC6A15	56	25	0.3086	Missense_Mutation	p.G598V	Non-Targeted Panel Gene	Adult HGBCL, NOS
BCL-75	chr1	160213473	160213473	C	T	exonic	PEA15	41	19	0.3167	Missense_Mutation	p.P148L	Non-Targeted Panel Gene	Adult HGBCL, NOS
BCL-75	chr3	69024152	69024152	C	T	exonic	TMF1	37	18	0.3273	Missense_Mutation	p.R1014Q	Non-Targeted Panel Gene	Adult HGBCL, NOS
BCL-75	chr15	27326978	27326978	C	A	exonic	GABRG3	39	19	0.3276	Missense_Mutation	p.P147H	Non-Targeted Panel Gene	Adult HGBCL, NOS
BCL-75	chr2	108486552	108486552	C	A	exonic	GCC2	40	20	0.3333	Missense_Mutation	p.H1278Q	Non-Targeted Panel Gene	Adult HGBCL, NOS
BCL-75	chr5	34821823	34821823	C	A	exonic	RAI14	56	28	0.3333	Missense_Mutation	p.H365Q	Non-Targeted Panel Gene	Adult HGBCL, NOS
BCL-75	chr17	7501342	7501342	A	T	exonic	POLR2A	41	21	0.3387	Missense_Mutation	p.I702F	Non-Targeted Panel Gene	Adult HGBCL, NOS
BCL-75	chr1	973570	973570	T	C	exonic	PLEKHN1	39	20	0.339	Missense_Mutation	p.L455P	Non-Targeted Panel Gene	Adult HGBCL, NOS
BCL-75	chr12	57749222	57749222	A	T	exonic	CDK4	74	38	0.3393	Missense_Mutation	p.V260E	Non-Targeted Panel Gene	Adult HGBCL, NOS
BCL-75	chr6	33671190	33671190	T	C	exonic	ITPR3	44	23	0.3433	Missense_Mutation	p.I871T	Non-Targeted Panel Gene	Adult HGBCL, NOS
BCL-75	chr7	150720719	150720719	C	T	exonic	GIMAP1	40	21	0.3443	Missense_Mutation	p.R239W	Non-Targeted Panel Gene	Adult HGBCL, NOS
BCL-75	chr8	73991487	73991487	T	A	exonic	LY96	53	28	0.3457	Missense_Mutation	p.F15L	Non-Targeted Panel Gene	Adult HGBCL, NOS
BCL-75	chr11	32601809	32601809	A	G	exonic	EIF3M	56	30	0.3488	Missense_Mutation	p.K331E	Non-Targeted Panel Gene	Adult HGBCL, NOS
BCL-75	chr20	22582019	22582019	A	T	exonic	FOXA2	41	22	0.3492	Missense_Mutation	p.L402H	Non-Targeted Panel Gene	Adult HGBCL, NOS
BCL-75	chr7	140694798	140694798	C	T	exonic	ADCK2	39	21	0.35	Missense_Mutation	p.P626S	Non-Targeted Panel Gene	Adult HGBCL, NOS
BCL-75	chr4	25255246	25255246	G	T	exonic	PI4K2B	46	25	0.3521	Missense_Mutation	p.S202I	Non-Targeted Panel Gene	Adult HGBCL, NOS
BCL-75	chrX	129491982	129491982	A	C	exonic	SMARCA1	20	11	0.3548	Missense_Mutation	p.Y592D	Non-Targeted Panel Gene	Adult HGBCL, NOS
BCL-75	chr20	46504262	46504262	C	T	exonic	ZNF334	41	23	0.3594	Missense_Mutation	p.G64R	Non-Targeted Panel Gene	Adult HGBCL, NOS
BCL-75	chr12	88532469	88532469	A	G	exonic	KITLG	57	32	0.3596	Missense_Mutation	p.L55P	Non-Targeted Panel Gene	Adult HGBCL, NOS
BCL-75	chr1	1615536	1615536	C	T	exonic	MIB2	63	36	0.3636	Missense_Mutation	p.P26L	Non-Targeted Panel Gene	Adult HGBCL, NOS
BCL-75	chrX	129492036	129492036	T	C	exonic	SMARCA1	21	12	0.3636	Missense_Mutation	p.T574A	Non-Targeted Panel Gene	Adult HGBCL, NOS
BCL-75	chr19	39435678	39435678	G	C	exonic	RPS16	33	19	0.3654	Missense_Mutation	p.R27G	Non-Targeted Panel Gene	Adult HGBCL, NOS
BCL-75	chr5	13770825	13770825	A	G	exonic	DNAH5	29	17	0.3696	Missense_Mutation	p.S3177P	Non-Targeted Panel Gene	Adult HGBCL, NOS
BCL-75	chr10	94981320	94981320	C	A	exonic	CYP2C9	39	23	0.371	Missense_Mutation	p.P367T	Non-Targeted Panel Gene	Adult HGBCL, NOS
BCL-75	chr11	119363977	119363977	C	T	exonic	USP2	22	13	0.3714	Missense_Mutation	p.V7M	Non-Targeted Panel Gene	Adult HGBCL, NOS
BCL-75	chr8	48729853	48729853	T	C	exonic	EFCAB1	32	19	0.3725	Missense_Mutation	p.D113G	Non-Targeted Panel Gene	Adult HGBCL, NOS
BCL-75	chr3	53820569	53820569	A	T	exonic	CHDH	33	20	0.3774	Missense_Mutation	p.I342N	Non-Targeted Panel Gene	Adult HGBCL, NOS
BCL-75	chr16	89920138	89920138	G	C	exonic	MC1R	52	32	0.381	Missense_Mutation	p.D294H	Non-Targeted Panel Gene	Adult HGBCL, NOS
BCL-75	chr6	39927527	39927527	C	T	exonic	MOCS1	40	25	0.3846	Missense_Mutation	p.G18S	Non-Targeted Panel Gene	Adult HGBCL, NOS
BCL-75	chr2	232521825	232521825	G	T	exonic	PRSS56	41	26	0.3881	Missense_Mutation	p.R72L	Non-Targeted Panel Gene	Adult HGBCL, NOS
BCL-75	chr19	19149416	19149416	T	C	exonic	BORCS8-MEF2B;M	40	26	0.3939	Missense_Mutation	p.K23R	Non-Targeted Panel Gene	Adult HGBCL, NOS
BCL-75	chr13	27924634	27924634	C	T	exonic	PDX1	38	25	0.3968	Missense_Mutation	p.P262L	Non-Targeted Panel Gene	Adult HGBCL, NOS
BCL-75	chr10	97923308	97923308	C	T	exonic	CRTAC1	41	27	0.3971	Missense_Mutation	p.A172T	Non-Targeted Panel Gene	Adult HGBCL, NOS
BCL-75	chr13	32210996	32210996	G	A	exonic	FRY	45	30	0.4	Missense_Mutation	p.R1518H	Non-Targeted Panel Gene	Adult HGBCL, NOS
BCL-75	chr17	4555312	4555312	C	T	exonic	MYBBP1A	46	31	0.4026	Missense_Mutation	p.D5N	Non-Targeted Panel Gene	Adult HGBCL, NOS
BCL-75	chr9	136412128	136412128	A	G	exonic	PMPCA	43	29	0.4028	Missense_Mutation	p.K68R	Non-Targeted Panel Gene	Adult HGBCL, NOS
BCL-75	chr4	57017636	57017636	T	C	exonic	POLR2B	47	32	0.4051	Missense_Mutation	p.M737T	Non-Targeted Panel Gene	Adult HGBCL, NOS
BCL-75	chr19	1754009	1754009	C	T	exonic	ONECUT3	41	28	0.4058	Missense_Mutation	p.P116L	Non-Targeted Panel Gene	Adult HGBCL, NOS
BCL-75	chr20	32911528	32911528	A	G	exonic	EFCAB8	34	24	0.4068	Missense_Mutation	p.T536A	Non-Targeted Panel Gene	Adult HGBCL, NOS
BCL-75	chr4	89248702	89248702	T	C	exonic	GPRIN3	34	24	0.4068	Missense_Mutation	p.Q470R	Non-Targeted Panel Gene	Adult HGBCL, NOS
BCL-75	chr16	31418322	31418322	G	T	exonic	ITGAD	40	28	0.4118	Missense_Mutation	p.D880Y	Non-Targeted Panel Gene	Adult HGBCL, NOS
BCL-75	chr9	106925850	106925850	C	G	exonic	ZNF462	31	22	0.4151	Missense_Mutation	p.D646E	Non-Targeted Panel Gene	Adult HGBCL, NOS
BCL-75	chr10	113774287	113774287	C	G	exonic	PLEKHS1	39	28	0.4179	Missense_Mutation	p.Q245E	Non-Targeted Panel Gene	Adult HGBCL, NOS
BCL-75	chr11	66265707	66265707	G	C	exonic	KLC2	44	32	0.4211	Missense_Mutation	p.G463R	Non-Targeted Panel Gene	Adult HGBCL, NOS
BCL-75	chr5	150379007	150379007	G	A	exonic	TCOF1	34	25	0.4237	Missense_Mutation	p.A815T	Non-Targeted Panel Gene	Adult HGBCL, NOS
BCL-75	chr2	190978924	190978924	C	T	exonic	STAT1	31	23	0.4259	Missense_Mutation	p.R602Q	Non-Targeted Panel Gene	Adult HGBCL, NOS
BCL-75	chr5	159084729	159084729	T	C	exonic	EBF1	37	28	0.4308	Missense_Mutation	p.Y141C	Non-Targeted Panel Gene	Adult HGBCL, NOS
BCL-75	chr1	161169143	161169143	C	G	exonic	PPOX	38	29	0.4328	Missense_Mutation	p.P94R	Non-Targeted Panel Gene	Adult HGBCL, NOS

BCL-75	chr1	15104088	15104088	C	A	exonic	KAZN	35	27	0.4355	Missense_Mutation	p.F649L	Non-Targeted Panel Gene	Adult HGBCL, NOS
BCL-75	chr15	83268191	83268191	T	G	exonic	BNC1	31	24	0.4364	Missense_Mutation	p.K47N	Non-Targeted Panel Gene	Adult HGBCL, NOS
BCL-75	chr10	93500159	93500159	C	G	exonic	CEP55	36	28	0.4375	Missense_Mutation	p.H36Q	Non-Targeted Panel Gene	Adult HGBCL, NOS
BCL-75	chr22	42214881	42214881	T	G	exonic	TCF20	33	26	0.4407	Missense_Mutation	p.Q142P	Non-Targeted Panel Gene	Adult HGBCL, NOS
BCL-75	chr3	53192231	53192231	G	T	exonic	PRKCD	35	28	0.4444	Missense_Mutation	p.V666L	Non-Targeted Panel Gene	Adult HGBCL, NOS
BCL-75	chr6	31753335	31753335	G	A	exonic	MSH5	39	32	0.4507	Missense_Mutation	p.E283K	Non-Targeted Panel Gene	Adult HGBCL, NOS
BCL-75	chr7	100868723	100868723	C	T	exonic	TRIP6	35	29	0.4531	Missense_Mutation	p.P198S	Non-Targeted Panel Gene	Adult HGBCL, NOS
BCL-75	chr18	26546858	26546858	G	A	exonic	KCTD1	42	35	0.4545	Missense_Mutation	p.S560L	Non-Targeted Panel Gene	Adult HGBCL, NOS
BCL-75	chr2	240968192	240968192	C	T	exonic	CROCC2	39	33	0.4583	Missense_Mutation	p.A1444V	Non-Targeted Panel Gene	Adult HGBCL, NOS
BCL-75	chr4	56315228	56315228	C	A	exonic	KIAA1211	27	23	0.46	Missense_Mutation	p.P576T	Non-Targeted Panel Gene	Adult HGBCL, NOS
BCL-75	chr3	53192133	53192133	T	A	exonic	PRKCD	42	37	0.4684	Missense_Mutation	p.F633Y	Non-Targeted Panel Gene	Adult HGBCL, NOS
BCL-75	chr6	109612033	109612033	T	C	exonic	AK9	29	27	0.4821	Missense_Mutation	p.D557G	Non-Targeted Panel Gene	Adult HGBCL, NOS
BCL-75	chr1	20851422	20851422	C	T	exonic	EIF4G3	32	30	0.4839	Missense_Mutation	p.R1153Q	Non-Targeted Panel Gene	Adult HGBCL, NOS
BCL-75	chr15	101806345	101806345	C	T	exonic	OR4F6	37	35	0.4861	Missense_Mutation	p.A209V	Non-Targeted Panel Gene	Adult HGBCL, NOS
BCL-75	chr3	53192183	53192183	A	C	exonic	PRKCD	40	38	0.4872	Missense_Mutation	p.N650H	Non-Targeted Panel Gene	Adult HGBCL, NOS
BCL-75	chr16	71655348	71655348	T	C	exonic	PHLPP2	26	26	0.4906	Missense_Mutation	p.E826G	Non-Targeted Panel Gene	Adult HGBCL, NOS
BCL-75	chr11	72688483	72688483	C	T	exonic	ARAP1	26	26	0.5	Missense_Mutation	p.V1348M	Non-Targeted Panel Gene	Adult HGBCL, NOS
BCL-75	chr2	80303299	80303299	T	C	exonic	LRRTM1	29	29	0.5	Missense_Mutation	p.Q174R	Non-Targeted Panel Gene	Adult HGBCL, NOS
BCL-75	chr6	31622878	31622878	C	G	exonic	PRRC2A	33	33	0.5	Missense_Mutation	p.S30C	Non-Targeted Panel Gene	Adult HGBCL, NOS
BCL-75	chr22	50245956	50245956	A	G	exonic	HDAC10	45	47	0.5109	Missense_Mutation	p.M596T	Non-Targeted Panel Gene	Adult HGBCL, NOS
BCL-75	chr7	128359202	128359202	G	A	exonic	PRRT4	35	41	0.5395	Missense_Mutation	p.P235L	Non-Targeted Panel Gene	Adult HGBCL, NOS
BCL-75	chr3	51933535	51933535	C	T	exonic	RRP9	27	32	0.5424	Missense_Mutation	p.V467I	Non-Targeted Panel Gene	Adult HGBCL, NOS
BCL-75	chr10	96432894	96432894	G	C	exonic	TLL2	29	35	0.5469	Missense_Mutation	p.P145A	Non-Targeted Panel Gene	Adult HGBCL, NOS
BCL-75	chr22	50070568	50070568	T	C	exonic	MLC1	36	45	0.5556	Missense_Mutation	p.I244V	Non-Targeted Panel Gene	Adult HGBCL, NOS
BCL-75	chr5	37038611	37038611	A	G	exonic	NIPBL	28	35	0.5556	Missense_Mutation	p.N1994S	Non-Targeted Panel Gene	Adult HGBCL, NOS
BCL-75	chr2	11771483	11771483	A	G	exonic	LPIN1	27	34	0.5574	Missense_Mutation	p.S183G	Non-Targeted Panel Gene	Adult HGBCL, NOS
BCL-75	chr6	135190227	135190227	A	C	exonic	MYB	30	39	0.5652	Missense_Mutation	p.N136T	Non-Targeted Panel Gene	Adult HGBCL, NOS
BCL-75	chr12	6960757	6960757	C	T	exonic	PTPN6	34	49	0.5904	Missense_Mutation	p.P590S	Non-Targeted Panel Gene	Adult HGBCL, NOS
BCL-75	chr1	94001992	94001992	C	G	exonic	ABCA4	24	37	0.6066	Missense_Mutation	p.V2050L	Non-Targeted Panel Gene	Adult HGBCL, NOS
BCL-75	chr7	81707311	81707311	G	A	exonic	HGF	22	35	0.614	Missense_Mutation	p.A527V	Non-Targeted Panel Gene	Adult HGBCL, NOS
BCL-75	chrX	36099803	36099803	C	A	exonic	CFAP47	15	25	0.625	Missense_Mutation	p.S1684Y	Non-Targeted Panel Gene	Adult HGBCL, NOS
BCL-75	chrX	136745320	136745320	C	T	exonic	ARHGEF6	9	29	0.7632	Missense_Mutation	p.R121H	Non-Targeted Panel Gene	Adult HGBCL, NOS
BCL-75	chr9	5457310	5457310	G	T	exonic	CD274	16	58	0.7838	Missense_Mutation	p.G95V	Non-Targeted Panel Gene	Adult HGBCL, NOS
BCL-75	chr2	32414849	32414849	C	T	exonic	BIRC6	50	5	0.0909	Nonsense_Mutation	p.Q520X	Targerted Panel Gene	Adult HGBCL, NOS
BCL-75	chr16	10907500	10907500	A	T	exonic	CIITA	42	7	0.1429	Nonsense_Mutation	p.R671X	Targerted Panel Gene	Adult HGBCL, NOS
BCL-75	chr7	80671940	80671940	C	A	exonic	CD36	43	31	0.4189	Nonsense_Mutation	p.S342X	Targerted Panel Gene	Adult HGBCL, NOS
BCL-75	chr6	137877213	137877213	C	T	exonic	TNFAIP3	42	36	0.4615	Nonsense_Mutation	p.Q315X	Targerted Panel Gene	Adult HGBCL, NOS
BCL-75	chr6	137876075	137876075	G	A	exonic	TNFAIP3	30	36	0.5455	Nonsense_Mutation	p.W238X	Targerted Panel Gene	Adult HGBCL, NOS
BCL-75	chr15	75381681	75381681	G	A	exonic	SIN3A	58	9	0.1343	Nonsense_Mutation	p.Q1074X	Non-Targeted Panel Gene	Adult HGBCL, NOS
BCL-75	chr13	92144846	92144846	C	A	exonic	GPC5	39	20	0.339	Nonsense_Mutation	p.S473X	Non-Targeted Panel Gene	Adult HGBCL, NOS
BCL-75	chr20	32796858	32796858	C	T	exonic	DNMT3B	28	15	0.3488	Nonsense_Mutation	p.Q456X	Non-Targeted Panel Gene	Adult HGBCL, NOS
BCL-75	chr1	27551761	27551761	G	A	exonic	AHDC1	48	27	0.36	Nonsense_Mutation	p.R119X	Non-Targeted Panel Gene	Adult HGBCL, NOS
BCL-75	chr15	27326814	27326814	C	A	exonic	GABRG3	35	22	0.386	Nonsense_Mutation	p.Y92X	Non-Targeted Panel Gene	Adult HGBCL, NOS
BCL-75	chr1	55120600	55120600	G	A	exonic	USP24	30	34	0.5312	Nonsense_Mutation	p.Q1502X	Non-Targeted Panel Gene	Adult HGBCL, NOS
BCL-76	chr15	44715703	44715703	T	C	splicing	B2M	29	21	0.42	Splice_Site	.	Targerted Panel Gene	Adult HGBCL, NOS
BCL-76	chr20	5178958	5178958	T	A	splicing	CDS2	17	2	0.1053	Splice_Site	.	Non-Targeted Panel Gene	Adult HGBCL, NOS
BCL-76	chr14	74480701	74480701	C	T	splicing	NPC2	24	17	0.4146	Splice_Site	.	Non-Targeted Panel Gene	Adult HGBCL, NOS
BCL-76	chr15	27926126	27926126	C	A	splicing	OCA2	5	4	0.4444	Splice_Site	.	Non-Targeted Panel Gene	Adult HGBCL, NOS
BCL-76	chr6	56616034	56616034	C	-	exonic	DST	14	14	0.5	Frame_Shift_Del	p.R2478fs	Targerted Panel Gene	Adult HGBCL, NOS
BCL-76	chr14	74921415	74921416	CA	-	exonic	RPS6KL1	54	2	0.0357	Frame_Shift_Del	p.P42fs	Non-Targeted Panel Gene	Adult HGBCL, NOS
BCL-76	chr3	57301832	57301832	C	-	exonic	DNAH12	51	2	0.0377	Frame_Shift_Del	p.G3770fs	Non-Targeted Panel Gene	Adult HGBCL, NOS
BCL-76	chr2	219570765	219570766	CT	-	exonic	OBSL1	51	2	0.0377	Frame_Shift_Del	p.E156fs	Non-Targeted Panel Gene	Adult HGBCL, NOS
BCL-76	chr1	156561943	156561944	CA	-	exonic	IQGAP3	48	2	0.04	Frame_Shift_Del	p.L312fs	Non-Targeted Panel Gene	Adult HGBCL, NOS
BCL-76	chr8	22163162	22163163	CC	-	exonic	SFTPC	48	2	0.04	Frame_Shift_Del	p.S95fs	Non-Targeted Panel Gene	Adult HGBCL, NOS
BCL-76	chr2	97792819	97792819	G	-	exonic	TMEM131	45	2	0.0426	Frame_Shift_Del	p.A1237fs	Non-Targeted Panel Gene	Adult HGBCL, NOS
BCL-76	chr16	28169858	28169910	TTGTCTTCAACATG	-	exonic	XPO6	41	2	0.0465	Frame_Shift_Del	p.L122fs	Non-Targeted Panel Gene	Adult HGBCL, NOS
BCL-76	chr1	153817502	153817502	G	-	exonic	GATAD2B	37	2	0.0513	Frame_Shift_Del	p.P257fs	Non-Targeted Panel Gene	Adult HGBCL, NOS
BCL-76	chr1	197142946	197142947	GC	-	exonic	ASPM	36	2	0.0526	Frame_Shift_Del	p.S435fs	Non-Targeted Panel Gene	Adult HGBCL, NOS
BCL-76	chr1	86498775	86498803	TGGACAGCTCCTG	-	exonic	CLCA1	34	2	0.0556	Frame_Shift_Del	p.W773fs	Non-Targeted Panel Gene	Adult HGBCL, NOS
BCL-76	chr14	67761452	67761453	GC	-	exonic	ZFYVE26	34	2	0.0556	Frame_Shift_Del	p.L2167fs	Non-Targeted Panel Gene	Adult HGBCL, NOS
BCL-76	chr20	46251153	46251154	CG	-	exonic	CDH22	66	4	0.0571	Frame_Shift_Del	p.P47fs	Non-Targeted Panel Gene	Adult HGBCL, NOS
BCL-76	chr21	33027530	33027531	CG	-	exonic	OLIG2	31	2	0.0606	Frame_Shift_Del	p.P223fs	Non-Targeted Panel Gene	Adult HGBCL, NOS
BCL-76	chr8	42708761	42708762	CT	-	exonic	CHRNB3	30	2	0.0625	Frame_Shift_Del	p.L33fs	Non-Targeted Panel Gene	Adult HGBCL, NOS
BCL-76	chr17	17136275	17136278	CAGC	-	exonic	MPRIP	30	2	0.0625	Frame_Shift_Del	p.S187fs	Non-Targeted Panel Gene	Adult HGBCL, NOS
BCL-76	chr4	6289119	6289120	GC	-	exonic	WFS1	29	2	0.0645	Frame_Shift_Del	p.A150fs	Non-Targeted Panel Gene	Adult HGBCL, NOS
BCL-76	chr9	93557927	93557939	CCCTGCCCTTCTA	-	exonic	FAM120A	27	2	0.069	Frame_Shift_Del	p.A862fs	Non-Targeted Panel Gene	Adult HGBCL, NOS
BCL-76	chr13	102748531	102748532	TG	-	exonic	CCDC168	25	2	0.0741	Frame_Shift_Del	p.S722fs	Non-Targeted Panel Gene	Adult HGBCL, NOS
BCL-76	chr11	64361704	64361704	C	-	exonic	RPS6KA4	22	2	0.0833	Frame_Shift_Del	p.F238fs	Non-Targeted Panel Gene	Adult HGBCL, NOS
BCL-76	chr9	110697412	110697413	GC	-	exonic	MUSK	29	3	0.0938	Frame_Shift_Del	p.A192fs	Non-Targeted Panel Gene	Adult HGBCL, NOS
BCL-76	chrX	55452506	55452507	CG	-	exonic	MAGEH1	19	2	0.0952	Frame_Shift_Del	p.P44fs	Non-Targeted Panel Gene	Adult HGBCL, NOS
BCL-76	chr20	31798455	31798455	C	-	exonic	TPX2	13	2	0.1333	Frame_Shift_Del	p.A679fs	Non-Targeted Panel Gene	Adult HGBCL, NOS
BCL-76	chr2	185795654	185795657	TTGT	-	exonic	FSIP2	21	13	0.3824	Frame_Shift_Del	p.L2840fs	Non-Targeted Panel Gene	Adult HGBCL, NOS
BCL-76	chr4	117084862	117084862	G	-	exonic	TRAM1L1	10	10	0.5	Frame_Shift_Del	p.H178fs	Non-Targeted Panel Gene	Adult HGBCL, NOS
BCL-76	chr7	152158896	152158896	-	A	exonic	KMT2C	19	7	0.2692	Frame_Shift_Ins	p.Y3879fs	Targerted Panel Gene	Adult HGBCL, NOS
BCL-76	chr20	46251146	46251146	-	CG	exonic	CDH22	67	4	0.0563	Frame_Shift_Ins	p.R50fs	Non-Targeted Panel Gene	Adult HGBCL, NOS
BCL-76	chr15	53615740	53615740	-	A	exonic	WDR72	18	10	0.3333	Frame_Shift_Ins	p.L822fs	Non-Targeted Panel Gene	Adult HGBCL, NOS
BCL-76	chr4	78267406	78267420	GAGTGTGCCAAAG	-	exonic	FRAS1	39	2	0.0488	In_Frame_Del	p.319_323del	Targerted Panel Gene	Adult HGBCL, NOS
BCL-76	chr3	50257024	50257026	AAG	-	exonic	GNAI2	21	13	0.3824	In_Frame_Del	p.255_255del	Targerted Panel Gene	Adult HGBCL, NOS
BCL-76	chr19	12930729	12930731	CCG	-	exonic	FARSA	50	2	0.0385	In_Frame_Del	p.56_56del	Non-Targeted Panel Gene	Adult HGBCL, NOS
BCL-76	chr11	1236493	1236495	GAC	-	exonic	MUC5B	48	2	0.04	In_Frame_Del	p.996_997del	Non-Targeted Panel Gene	Adult HGBCL, NOS
BCL-76	chr2	135715581	135715598	CACCAGCTCAGTC	-	exonic	R3HDM1	44	2	0.0435	In_Frame_Del	p.888_894del	Non-Targeted Panel Gene	Adult HGBCL, NOS
BCL-76	chr5	177332125	177332136	CACCACGGCCCC	-	exonic	LMAN2	58	3	0.0492	In_Frame_Del	p.341_344del	Non-Targeted Panel Gene	Adult HGBCL, NOS

BCL-76	chr5	10264687	10264692	GATTGG	-	exonic	CCT5	35	2	0.0541	In_Frame_Del	p.510_512del	Non-Targeted Panel Gene	Adult HGBCL, NOS
BCL-76	chr6	149451174	149451176	GGA	-	exonic	ZC3H12D	32	2	0.0588	In_Frame_Del	p.364_365del	Non-Targeted Panel Gene	Adult HGBCL, NOS
BCL-76	chr22	37724091	37724099	CCTCCAGAA	-	exonic	TRIOBP	115	9	0.0726	In_Frame_Del	p.512_515del	Non-Targeted Panel Gene	Adult HGBCL, NOS
BCL-76	chr17	18353722	18353742	GGCCTCCAAAACTC	-	exonic	SHMT1	22	2	0.0833	In_Frame_Del	p.58_64del	Non-Targeted Panel Gene	Adult HGBCL, NOS
BCL-76	chr3	101367963	101367965	CTG	-	exonic	SENP7	21	2	0.087	In_Frame_Del	p.281_282del	Non-Targeted Panel Gene	Adult HGBCL, NOS
BCL-76	chrY	12818445	12818447	TAT	-	exonic	USP9Y	22	3	0.12	In_Frame_Del	p.1619_1620del	Non-Targeted Panel Gene	Adult HGBCL, NOS
BCL-76	chr19	5783392	5783412	CAGAACCTTGTCAC	-	exonic	PRR22	44	11	0.2	In_Frame_Del	p.279_285del	Non-Targeted Panel Gene	Adult HGBCL, NOS
BCL-76	chr3	141573155	141573157	CTC	-	exonic	RASA2	14	7	0.3333	In_Frame_Del	p.431_432del	Non-Targeted Panel Gene	Adult HGBCL, NOS
BCL-76	chr10	18539656	18539658	GGA	-	exonic	CACNB2	27	17	0.3864	In_Frame_Del	p.615_615del	Non-Targeted Panel Gene	Adult HGBCL, NOS
BCL-76	chr16	88434448	88434450	CTC	-	exonic	ZNF469	20	14	0.4118	In_Frame_Del	p.2298_2299del	Non-Targeted Panel Gene	Adult HGBCL, NOS
BCL-76	chr12	52848036	52848056	CCCTGCTGGCTGCC	-	exonic	KRT78	31	28	0.4746	In_Frame_Del	p.150_157del	Non-Targeted Panel Gene	Adult HGBCL, NOS
BCL-76	chr16	85666114	85666114	-	AGCTCCTGGACTCT	exonic	GSE1	53	2	0.0364	In_Frame_Ins	p.L966delinsLAPGL	Non-Targeted Panel Gene	Adult HGBCL, NOS
BCL-76	chr19	6415619	6415619	-	GGGGCCGCA	exonic	KHSRP	27	11	0.2821	In_Frame_Ins	p.P601delinsPAAP	Non-Targeted Panel Gene	Adult HGBCL, NOS
BCL-76	chr3	38141147	38141147	G	T	exonic	MYD88	28	14	0.3333	Missense_Mutation	p.R264L	Targerted Panel Gene	Adult HGBCL, NOS
BCL-76	chr7	154892410	154892410	T	A	exonic	DPP6	49	2	0.0392	Missense_Mutation	p.F843Y	Targerted Panel Gene	Adult HGBCL, NOS
BCL-76	chr4	1395799	1395799	C	T	exonic	CRIPAK	76	4	0.05	Missense_Mutation	p.R430C	Targerted Panel Gene	Adult HGBCL, NOS
BCL-76	chr19	23359759	23359759	T	C	exonic	ZNF91	47	4	0.0784	Missense_Mutation	p.R1074G	Targerted Panel Gene	Adult HGBCL, NOS
BCL-76	chr2	32479464	32479464	G	A	exonic	BIRC6	18	2	0.1	Missense_Mutation	p.E2419K	Targerted Panel Gene	Adult HGBCL, NOS
BCL-76	chr2	32479509	32479509	G	A	exonic	BIRC6	18	2	0.1	Missense_Mutation	p.V2434M	Targerted Panel Gene	Adult HGBCL, NOS
BCL-76	chr1	215934718	215934718	C	A	exonic	USH2A	18	2	0.1	Missense_Mutation	p.D2400Y	Targerted Panel Gene	Adult HGBCL, NOS
BCL-76	chr10	87933036	87933036	C	T	exonic	PTEN	49	6	0.1091	Missense_Mutation	p.H266Y	Targerted Panel Gene	Adult HGBCL, NOS
BCL-76	chr10	87864499	87864499	C	A	exonic	PTEN	28	7	0.2	Missense_Mutation	p.S183R	Targerted Panel Gene	Adult HGBCL, NOS
BCL-76	chr3	177033000	177033000	C	T	exonic	TBL1XR1	20	7	0.2593	Missense_Mutation	p.D463N	Targerted Panel Gene	Adult HGBCL, NOS
BCL-76	chr12	113077749	113077749	C	G	exonic	DTX1	58	26	0.3095	Missense_Mutation	p.S195R	Targerted Panel Gene	Adult HGBCL, NOS
BCL-76	chr12	113058448	113058448	A	G	exonic	DTX1	25	12	0.3243	Missense_Mutation	p.T86A	Targerted Panel Gene	Adult HGBCL, NOS
BCL-76	chr11	108268443	108268443	C	T	exonic	ATM	8	4	0.3333	Missense_Mutation	p.S891L	Targerted Panel Gene	Adult HGBCL, NOS
BCL-76	chr6	41935959	41935959	A	T	exonic	CCND3	27	14	0.3415	Missense_Mutation	p.V287D	Targerted Panel Gene	Adult HGBCL, NOS
BCL-76	chr16	3736703	3736703	A	C	exonic	CREBBP	29	19	0.3958	Missense_Mutation	p.Y1503D	Targerted Panel Gene	Adult HGBCL, NOS
BCL-76	chr16	10909127	10909127	C	T	exonic	CIITA	32	23	0.4182	Missense_Mutation	p.P920L	Targerted Panel Gene	Adult HGBCL, NOS
BCL-76	chr3	65356832	65356832	G	A	exonic	MAGI1	47	36	0.4337	Missense_Mutation	p.A1312V	Targerted Panel Gene	Adult HGBCL, NOS
BCL-76	chr16	10916429	10916429	T	C	exonic	CIITA	23	19	0.4524	Missense_Mutation	p.F1012S	Targerted Panel Gene	Adult HGBCL, NOS
BCL-76	chr18	67513510	67513510	G	C	exonic	DSEL	11	10	0.4762	Missense_Mutation	p.H377D	Targerted Panel Gene	Adult HGBCL, NOS
BCL-76	chr6	37170773	37170773	G	T	exonic	PIM1	32	32	0.5	Missense_Mutation	p.G28V	Targerted Panel Gene	Adult HGBCL, NOS
BCL-76	chr17	16092039	16092039	T	G	exonic	NCOR1	6	8	0.5714	Missense_Mutation	p.N947T	Targerted Panel Gene	Adult HGBCL, NOS
BCL-76	chr19	11243676	11243676	G	A	exonic	DOCK6	23	31	0.5741	Missense_Mutation	p.A380V	Targerted Panel Gene	Adult HGBCL, NOS
BCL-76	chr5	112842318	112842318	A	G	exonic	APC	9	19	0.6786	Missense_Mutation	p.S2242G	Targerted Panel Gene	Adult HGBCL, NOS
BCL-76	chrX	141907682	141907682	C	T	exonic	MAGEC1	6	27	0.8182	Missense_Mutation	p.P760S	Targerted Panel Gene	Adult HGBCL, NOS
BCL-76	chr17	63929438	63929438	T	G	exonic	CD79B	8	45	0.8491	Missense_Mutation	p.Y197S	Targerted Panel Gene	Adult HGBCL, NOS
BCL-76	chr17	65014429	65014429	T	C	exonic	GNA13	2	42	0.9545	Missense_Mutation	p.D321G	Targerted Panel Gene	Adult HGBCL, NOS
BCL-76	chr3	112639098	112639098	C	T	exonic	CCDC80	84	2	0.0233	Missense_Mutation	p.E270K	Non-Targeted Panel Gene	Adult HGBCL, NOS
BCL-76	chr8	37966109	37966109	C	T	exonic	ADRB3	65	2	0.0299	Missense_Mutation	p.V121M	Non-Targeted Panel Gene	Adult HGBCL, NOS
BCL-76	chr2	202035003	202035003	C	T	exonic	FZD7	56	2	0.0345	Missense_Mutation	p.P119L	Non-Targeted Panel Gene	Adult HGBCL, NOS
BCL-76	chr17	2694170	2694170	G	A	exonic	CLUH	55	2	0.0351	Missense_Mutation	p.S976L	Non-Targeted Panel Gene	Adult HGBCL, NOS
BCL-76	chr8	22042930	22042930	T	C	exonic	FGF17	54	2	0.0357	Missense_Mutation	p.M1T	Non-Targeted Panel Gene	Adult HGBCL, NOS
BCL-76	chr3	125594767	125594767	C	T	exonic	OSBPL11	52	2	0.037	Missense_Mutation	p.V12I	Non-Targeted Panel Gene	Adult HGBCL, NOS
BCL-76	chr16	68345778	68345778	T	A	exonic	PRMT7	52	2	0.037	Missense_Mutation	p.V344E	Non-Targeted Panel Gene	Adult HGBCL, NOS
BCL-76	chr6	99613912	99613912	C	T	exonic	PRDM13	51	2	0.0377	Missense_Mutation	p.A426V	Non-Targeted Panel Gene	Adult HGBCL, NOS
BCL-76	chr3	57301845	57301845	T	A	exonic	DNAH12	50	2	0.0385	Missense_Mutation	p.S3766C	Non-Targeted Panel Gene	Adult HGBCL, NOS
BCL-76	chr19	55279569	55279569	C	T	exonic	HSPBP1	50	2	0.0385	Missense_Mutation	p.A14T	Non-Targeted Panel Gene	Adult HGBCL, NOS
BCL-76	chr6	34531715	34531715	T	C	exonic	PACSIN1	50	2	0.0385	Missense_Mutation	p.S385P	Non-Targeted Panel Gene	Adult HGBCL, NOS
BCL-76	chr14	64743132	64743132	G	A	exonic	PLEKHG3	50	2	0.0385	Missense_Mutation	p.G1030E	Non-Targeted Panel Gene	Adult HGBCL, NOS
BCL-76	chr14	92651822	92651822	C	T	exonic	RIN3	49	2	0.0392	Missense_Mutation	p.P258L	Non-Targeted Panel Gene	Adult HGBCL, NOS
BCL-76	chr1	202127984	202127984	G	A	exonic	GPR37L1	48	2	0.04	Missense_Mutation	p.A292T	Non-Targeted Panel Gene	Adult HGBCL, NOS
BCL-76	chr20	25500895	25500895	T	C	exonic	NINL	48	2	0.04	Missense_Mutation	p.Q326R	Non-Targeted Panel Gene	Adult HGBCL, NOS
BCL-76	chr7	99388168	99388168	G	A	exonic	ARPC1B	46	2	0.0417	Missense_Mutation	p.R100H	Non-Targeted Panel Gene	Adult HGBCL, NOS
BCL-76	chr20	64205632	64205632	C	A	exonic	MYT1	46	2	0.0417	Missense_Mutation	p.H77N	Non-Targeted Panel Gene	Adult HGBCL, NOS
BCL-76	chr2	97792820	97792820	G	T	exonic	TMEM131	46	2	0.0417	Missense_Mutation	p.A1237D	Non-Targeted Panel Gene	Adult HGBCL, NOS
BCL-76	chr17	8016449	8016449	C	A	exonic	GUCY2D	45	2	0.0426	Missense_Mutation	p.S1077R	Non-Targeted Panel Gene	Adult HGBCL, NOS
BCL-76	chr19	49434794	49434794	C	T	exonic	SLC17A7	45	2	0.0426	Missense_Mutation	p.V175M	Non-Targeted Panel Gene	Adult HGBCL, NOS
BCL-76	chr17	61400435	61400435	G	A	exonic	TBX2	44	2	0.0435	Missense_Mutation	p.A87T	Non-Targeted Panel Gene	Adult HGBCL, NOS
BCL-76	chr11	129944969	129944969	C	A	exonic	PRDM10	43	2	0.0444	Missense_Mutation	p.K188N	Non-Targeted Panel Gene	Adult HGBCL, NOS
BCL-76	chr17	34626421	34626421	C	T	exonic	TMEM132E	42	2	0.0455	Missense_Mutation	p.T121M	Non-Targeted Panel Gene	Adult HGBCL, NOS
BCL-76	chr12	121420278	121420278	G	T	exonic	RNF34	41	2	0.0465	Missense_Mutation	p.D225Y	Non-Targeted Panel Gene	Adult HGBCL, NOS
BCL-76	chr6	30925278	30925278	A	T	exonic	VARS2	41	2	0.0465	Missense_Mutation	p.H893L	Non-Targeted Panel Gene	Adult HGBCL, NOS
BCL-76	chr18	580556	580556	G	A	exonic	CETN1	40	2	0.0476	Missense_Mutation	p.A50T	Non-Targeted Panel Gene	Adult HGBCL, NOS
BCL-76	chr8	144473190	144473190	G	A	exonic	KIFC2	40	2	0.0476	Missense_Mutation	p.E753K	Non-Targeted Panel Gene	Adult HGBCL, NOS
BCL-76	chr22	50468408	50468408	C	T	exonic	SBF1	40	2	0.0476	Missense_Mutation	p.E37K	Non-Targeted Panel Gene	Adult HGBCL, NOS
BCL-76	chr11	130261795	130261795	C	A	exonic	ZBTB44	39	2	0.0488	Missense_Mutation	p.D27Y	Non-Targeted Panel Gene	Adult HGBCL, NOS
BCL-76	chr18	58579716	58579716	C	A	exonic	ALPK2	38	2	0.05	Missense_Mutation	p.V354F	Non-Targeted Panel Gene	Adult HGBCL, NOS
BCL-76	chr10	133383999	133383999	C	A	exonic	PAOX	57	3	0.05	Missense_Mutation	p.P303H	Non-Targeted Panel Gene	Adult HGBCL, NOS
BCL-76	chr2	178773723	178773723	C	A	exonic	TTN	37	2	0.05	Missense_Mutation	p.V2445L	Non-Targeted Panel Gene	Adult HGBCL, NOS
BCL-76	chr2	24918828	24918828	G	A	exonic	ADCY3	56	3	0.0508	Missense_Mutation	p.R54W	Non-Targeted Panel Gene	Adult HGBCL, NOS
BCL-76	chrX	101657251	101657251	C	T	exonic	ARMCX2	37	2	0.0513	Missense_Mutation	p.S113N	Non-Targeted Panel Gene	Adult HGBCL, NOS
BCL-76	chr16	67174941	67174941	G	A	exonic	NOL3	74	4	0.0513	Missense_Mutation	p.E268K	Non-Targeted Panel Gene	Adult HGBCL, NOS
BCL-76	chr15	74906593	74906593	T	C	exonic	FAM219B	36	2	0.0526	Missense_Mutation	p.K70E	Non-Targeted Panel Gene	Adult HGBCL, NOS
BCL-76	chr19	5243973	5243973	G	A	exonic	PTPRS	36	2	0.0526	Missense_Mutation	p.R500W	Non-Targeted Panel Gene	Adult HGBCL, NOS
BCL-76	chr1	226983627	226983627	C	A	exonic	COQ8A	35	2	0.0541	Missense_Mutation	p.P386T	Non-Targeted Panel Gene	Adult HGBCL, NOS
BCL-76	chr16	81202595	81202595	C	A	exonic	PKD1L2	35	2	0.0541	Missense_Mutation	p.A350S	Non-Targeted Panel Gene	Adult HGBCL, NOS
BCL-76	chr1	34985220	34985220	G	C	exonic	TMEM35B	35	2	0.0541	Missense_Mutation	p.S29W	Non-Targeted Panel Gene	Adult HGBCL, NOS
BCL-76	chr12	6620761	6620761	G	A	exonic	LPAR5	69	4	0.0548	Missense_Mutation	p.S163L	Non-Targeted Panel Gene	Adult HGBCL, NOS
BCL-76	chr14	75579445	75579445	C	T	exonic	FLVCR2	51	3	0.0556	Missense_Mutation	p.S158L	Non-Targeted Panel Gene	Adult HGBCL, NOS
BCL-76	chr19	21057261	21057261	G	A	exonic	ZNF430	68	4	0.0556	Missense_Mutation	p.R318K	Non-Targeted Panel Gene	Adult HGBCL, NOS

BCL-76	chr4	7041603	7041603	C	T	exonic	CCDC96	67	4	0.0563	Missense_Mutation	p.V446M	Non-Targeted Panel Gene	Adult HGBCL, NOS
BCL-76	chr16	88709960	88709960	G	A	exonic	CTU2	67	4	0.0563	Missense_Mutation	p.V56I	Non-Targeted Panel Gene	Adult HGBCL, NOS
BCL-76	chr17	58487926	58487926	G	T	exonic	HSF5	32	2	0.0571	Missense_Mutation	p.R117S	Non-Targeted Panel Gene	Adult HGBCL, NOS
BCL-76	chr15	34063142	34063142	G	A	exonic	CHRM5	48	3	0.0588	Missense_Mutation	p.R142H	Non-Targeted Panel Gene	Adult HGBCL, NOS
BCL-76	chr3	52326191	52326191	G	A	exonic	DNAH1	32	2	0.0588	Missense_Mutation	p.C153Y	Non-Targeted Panel Gene	Adult HGBCL, NOS
BCL-76	chr15	75347884	75347884	C	T	exonic	NEIL1	32	2	0.0588	Missense_Mutation	p.P32S	Non-Targeted Panel Gene	Adult HGBCL, NOS
BCL-76	chr19	23654041	23654041	T	G	exonic	ZNF675	32	2	0.0588	Missense_Mutation	p.N298H	Non-Targeted Panel Gene	Adult HGBCL, NOS
BCL-76	chr17	81922791	81922791	C	A	exonic	MAFG	47	3	0.06	Missense_Mutation	p.K101N	Non-Targeted Panel Gene	Adult HGBCL, NOS
BCL-76	chr19	15011261	15011261	C	A	exonic	CCDC105	31	2	0.0606	Missense_Mutation	p.Q146K	Non-Targeted Panel Gene	Adult HGBCL, NOS
BCL-76	chr17	29166594	29166594	G	A	exonic	MYO18A	46	3	0.0612	Missense_Mutation	p.S116L	Non-Targeted Panel Gene	Adult HGBCL, NOS
BCL-76	chr6	144187189	144187189	G	A	exonic	STX11	89	6	0.0632	Missense_Mutation	p.V188M	Non-Targeted Panel Gene	Adult HGBCL, NOS
BCL-76	chr6	30950963	30950963	G	C	exonic	DPCR1	74	5	0.0633	Missense_Mutation	p.K833N	Non-Targeted Panel Gene	Adult HGBCL, NOS
BCL-76	chr8	28750732	28750732	C	T	exonic	EXTL3	43	3	0.0652	Missense_Mutation	p.R876W	Non-Targeted Panel Gene	Adult HGBCL, NOS
BCL-76	chr11	63895583	63895583	G	A	exonic	MARK2	43	3	0.0652	Missense_Mutation	p.A47T	Non-Targeted Panel Gene	Adult HGBCL, NOS
BCL-76	chr3	137765118	137765118	G	A	exonic	SOX14	43	3	0.0652	Missense_Mutation	p.V112M	Non-Targeted Panel Gene	Adult HGBCL, NOS
BCL-76	chr11	64804566	64804566	G	A	exonic	MEN1	56	4	0.0667	Missense_Mutation	p.T539M	Non-Targeted Panel Gene	Adult HGBCL, NOS
BCL-76	chr5	111098782	111098782	C	G	exonic	WDR36	42	3	0.0667	Missense_Mutation	p.H174D	Non-Targeted Panel Gene	Adult HGBCL, NOS
BCL-76	chrX	153347487	153347487	G	A	exonic	ZNF275	28	2	0.0667	Missense_Mutation	p.D268N	Non-Targeted Panel Gene	Adult HGBCL, NOS
BCL-76	chr19	43613683	43613683	A	G	exonic	SRRM5	83	6	0.0674	Missense_Mutation	p.H521R	Non-Targeted Panel Gene	Adult HGBCL, NOS
BCL-76	chr6	28904176	28904176	G	A	exonic	TRIM27	41	3	0.0682	Missense_Mutation	p.S479L	Non-Targeted Panel Gene	Adult HGBCL, NOS
BCL-76	chr20	63752768	63752768	G	A	exonic	ZBTB46	41	3	0.0682	Missense_Mutation	p.S439L	Non-Targeted Panel Gene	Adult HGBCL, NOS
BCL-76	chr3	42659517	42659517	C	T	exonic	ZBTB47	41	3	0.0682	Missense_Mutation	p.R388W	Non-Targeted Panel Gene	Adult HGBCL, NOS
BCL-76	chr19	36237077	36237077	G	A	exonic	ZNF146	41	3	0.0682	Missense_Mutation	p.E213K	Non-Targeted Panel Gene	Adult HGBCL, NOS
BCL-76	chr17	6478052	6478052	C	T	exonic	PITPNM3	27	2	0.069	Missense_Mutation	p.A275T	Non-Targeted Panel Gene	Adult HGBCL, NOS
BCL-76	chrX	107775126	107775126	C	T	exonic	TSC22D3	27	2	0.069	Missense_Mutation	p.M98I	Non-Targeted Panel Gene	Adult HGBCL, NOS
BCL-76	chr11	17406962	17406962	C	T	exonic	ABCC8	40	3	0.0698	Missense_Mutation	p.D1029N	Non-Targeted Panel Gene	Adult HGBCL, NOS
BCL-76	chr10	102919744	102919744	G	A	exonic	CNNM2	40	3	0.0698	Missense_Mutation	p.D422N	Non-Targeted Panel Gene	Adult HGBCL, NOS
BCL-76	chr10	73789763	73789763	G	A	exonic	ZSWIM8	53	4	0.0702	Missense_Mutation	p.R226Q	Non-Targeted Panel Gene	Adult HGBCL, NOS
BCL-76	chr1	37689721	37689721	T	A	exonic	C1orf109	26	2	0.0714	Missense_Mutation	p.N54Y	Non-Targeted Panel Gene	Adult HGBCL, NOS
BCL-76	chr12	113463356	113463356	G	A	exonic	LHX5	64	5	0.0725	Missense_Mutation	p.P348L	Non-Targeted Panel Gene	Adult HGBCL, NOS
BCL-76	chr22	37723805	37723805	A	G	exonic	TRIOBP	64	5	0.0725	Missense_Mutation	p.T417A	Non-Targeted Panel Gene	Adult HGBCL, NOS
BCL-76	chr19	55096078	55096078	C	T	exonic	PPP1R12C	51	4	0.0727	Missense_Mutation	p.E376K	Non-Targeted Panel Gene	Adult HGBCL, NOS
BCL-76	chr19	40224766	40224766	G	A	exonic	CNTD2	25	2	0.0741	Missense_Mutation	p.P105S	Non-Targeted Panel Gene	Adult HGBCL, NOS
BCL-76	chr17	35863075	35863075	T	C	exonic	HEATR9	25	2	0.0741	Missense_Mutation	p.K226E	Non-Targeted Panel Gene	Adult HGBCL, NOS
BCL-76	chr2	98822063	98822063	C	T	exonic	KIAA1211L	50	4	0.0741	Missense_Mutation	p.R737Q	Non-Targeted Panel Gene	Adult HGBCL, NOS
BCL-76	chr17	7502009	7502009	G	A	exonic	POLR2A	37	3	0.075	Missense_Mutation	p.R820H	Non-Targeted Panel Gene	Adult HGBCL, NOS
BCL-76	chr9	107489013	107489013	C	T	exonic	KLF4	36	3	0.0769	Missense_Mutation	p.A15T	Non-Targeted Panel Gene	Adult HGBCL, NOS
BCL-76	chr7	140024790	140024790	C	T	exonic	PARP12	35	3	0.0789	Missense_Mutation	p.E626K	Non-Targeted Panel Gene	Adult HGBCL, NOS
BCL-76	chr17	18353748	18353748	A	G	exonic	SHMT1	23	2	0.08	Missense_Mutation	p.F56L	Non-Targeted Panel Gene	Adult HGBCL, NOS
BCL-76	chr7	27245170	27245170	C	T	exonic	EVX1	44	4	0.0833	Missense_Mutation	p.R2C	Non-Targeted Panel Gene	Adult HGBCL, NOS
BCL-76	chr1	228487511	228487511	C	G	exonic	RNF187	22	2	0.0833	Missense_Mutation	p.A8G	Non-Targeted Panel Gene	Adult HGBCL, NOS
BCL-76	chr19	23654046	23654046	A	G	exonic	ZNF675	32	3	0.0857	Missense_Mutation	p.F296S	Non-Targeted Panel Gene	Adult HGBCL, NOS
BCL-76	chr1	86577922	86577922	G	T	exonic	CLCA4	21	2	0.087	Missense_Mutation	p.D658Y	Non-Targeted Panel Gene	Adult HGBCL, NOS
BCL-76	chr17	42720285	42720285	G	A	exonic	EZH1	21	2	0.087	Missense_Mutation	p.H218Y	Non-Targeted Panel Gene	Adult HGBCL, NOS
BCL-76	chr6	39194261	39194261	A	T	exonic	KCNK5	21	2	0.087	Missense_Mutation	p.V181E	Non-Targeted Panel Gene	Adult HGBCL, NOS
BCL-76	chr9	110379373	110379373	G	T	exonic	SVEP1	21	2	0.087	Missense_Mutation	p.T3461K	Non-Targeted Panel Gene	Adult HGBCL, NOS
BCL-76	chr13	102743915	102743915	A	G	exonic	CCDC168	31	3	0.0882	Missense_Mutation	p.I2261T	Non-Targeted Panel Gene	Adult HGBCL, NOS
BCL-76	chr12	65246209	65246209	C	T	exonic	LEMD3	31	3	0.0882	Missense_Mutation	p.R874C	Non-Targeted Panel Gene	Adult HGBCL, NOS
BCL-76	chr9	137169137	137169137	G	T	exonic	LRRC26	31	3	0.0882	Missense_Mutation	p.P241H	Non-Targeted Panel Gene	Adult HGBCL, NOS
BCL-76	chr4	17522929	17522929	C	A	exonic	CLRN2	20	2	0.0909	Missense_Mutation	p.L107M	Non-Targeted Panel Gene	Adult HGBCL, NOS
BCL-76	chr3	52364869	52364869	G	A	exonic	DNAH1	40	4	0.0909	Missense_Mutation	p.V1790M	Non-Targeted Panel Gene	Adult HGBCL, NOS
BCL-76	chr21	33027533	33027533	C	T	exonic	OLIG2	30	3	0.0909	Missense_Mutation	p.P224L	Non-Targeted Panel Gene	Adult HGBCL, NOS
BCL-76	chr11	17640921	17640921	C	A	exonic	OTOG	20	2	0.0909	Missense_Mutation	p.P2674T	Non-Targeted Panel Gene	Adult HGBCL, NOS
BCL-76	chr3	101367959	101367959	G	T	exonic	SENP7	20	2	0.0909	Missense_Mutation	p.N283K	Non-Targeted Panel Gene	Adult HGBCL, NOS
BCL-76	chr19	30007604	30007604	G	A	exonic	URI1	20	2	0.0909	Missense_Mutation	p.E218K	Non-Targeted Panel Gene	Adult HGBCL, NOS
BCL-76	chr18	37565391	37565391	G	A	exonic	CELF4	39	4	0.093	Missense_Mutation	p.T84M	Non-Targeted Panel Gene	Adult HGBCL, NOS
BCL-76	chr1	52857147	52857147	C	T	exonic	ZYG11A	29	3	0.0938	Missense_Mutation	p.H136Y	Non-Targeted Panel Gene	Adult HGBCL, NOS
BCL-76	chrX	118584826	118584826	G	A	exonic	DOCK11	19	2	0.0952	Missense_Mutation	p.D563N	Non-Targeted Panel Gene	Adult HGBCL, NOS
BCL-76	chr12	66379308	66379308	C	T	exonic	GRIP1	19	2	0.0952	Missense_Mutation	p.E813K	Non-Targeted Panel Gene	Adult HGBCL, NOS
BCL-76	chr6	142761552	142761552	C	A	exonic	HIVEP2	19	2	0.0952	Missense_Mutation	p.K1844N	Non-Targeted Panel Gene	Adult HGBCL, NOS
BCL-76	chr15	66553088	66553088	C	T	exonic	LCTL	38	4	0.0952	Missense_Mutation	p.D365N	Non-Targeted Panel Gene	Adult HGBCL, NOS
BCL-76	chr2	222294146	222294146	G	A	exonic	PAX3	38	4	0.0952	Missense_Mutation	p.R203W	Non-Targeted Panel Gene	Adult HGBCL, NOS
BCL-76	chr10	49664964	49664964	G	A	exonic	CHAT	28	3	0.0968	Missense_Mutation	p.C604Y	Non-Targeted Panel Gene	Adult HGBCL, NOS
BCL-76	chr7	131411345	131411345	A	T	exonic	MKLN1	28	3	0.0968	Missense_Mutation	p.K225M	Non-Targeted Panel Gene	Adult HGBCL, NOS
BCL-76	chr4	113242153	113242153	A	G	exonic	ANK2	18	2	0.1	Missense_Mutation	p.T258A	Non-Targeted Panel Gene	Adult HGBCL, NOS
BCL-76	chr4	15644671	15644671	C	T	exonic	FBXL5	18	2	0.1	Missense_Mutation	p.R41H	Non-Targeted Panel Gene	Adult HGBCL, NOS
BCL-76	chrX	136206467	136206467	A	G	exonic	FHL1	18	2	0.1	Missense_Mutation	p.D12G	Non-Targeted Panel Gene	Adult HGBCL, NOS
BCL-76	chr5	133220840	133220840	C	A	exonic	FSTL4	18	2	0.1	Missense_Mutation	p.V456F	Non-Targeted Panel Gene	Adult HGBCL, NOS
BCL-76	chrX	46606983	46606983	C	T	exonic	SLC9A7	27	3	0.1	Missense_Mutation	p.R716H	Non-Targeted Panel Gene	Adult HGBCL, NOS
BCL-76	chr17	42026997	42026997	G	A	exonic	ZNF385C	18	2	0.1	Missense_Mutation	p.T392I	Non-Targeted Panel Gene	Adult HGBCL, NOS
BCL-76	chr9	74984956	74984956	C	A	exonic	CARNMT1	26	3	0.1034	Missense_Mutation	p.S360I	Non-Targeted Panel Gene	Adult HGBCL, NOS
BCL-76	chr5	122452988	122452988	G	A	exonic	SNCAIP	26	3	0.1034	Missense_Mutation	p.D987N	Non-Targeted Panel Gene	Adult HGBCL, NOS
BCL-76	chr11	870429	870429	C	T	exonic	CHID1	17	2	0.1053	Missense_Mutation	p.E369K	Non-Targeted Panel Gene	Adult HGBCL, NOS
BCL-76	chr1	202122979	202122979	C	T	exonic	GPR37L1	17	2	0.1053	Missense_Mutation	p.P6S	Non-Targeted Panel Gene	Adult HGBCL, NOS
BCL-76	chr16	15622971	15622971	T	A	exonic	MARF1	17	2	0.1053	Missense_Mutation	p.Q808L	Non-Targeted Panel Gene	Adult HGBCL, NOS
BCL-76	chr15	72265966	72265966	C	T	exonic	PARP6	17	2	0.1053	Missense_Mutation	p.R36Q	Non-Targeted Panel Gene	Adult HGBCL, NOS
BCL-76	chr1	90918074	90918074	C	T	exonic	ZNF644	17	2	0.1053	Missense_Mutation	p.E1257K	Non-Targeted Panel Gene	Adult HGBCL, NOS
BCL-76	chr5	56913959	56913959	G	C	exonic	SETD9	25	3	0.1071	Missense_Mutation	p.V226L	Non-Targeted Panel Gene	Adult HGBCL, NOS
BCL-76	chr4	61517386	61517386	G	A	exonic	ADGRL3	16	2	0.1111	Missense_Mutation	p.A43T	Non-Targeted Panel Gene	Adult HGBCL, NOS
BCL-76	chr18	12103215	12103215	G	A	exonic	ANKRD62	16	2	0.1111	Missense_Mutation	p.S293N	Non-Targeted Panel Gene	Adult HGBCL, NOS
BCL-76	chr2	200669722	200669722	G	A	exonic	AOX1	16	2	0.1111	Missense_Mutation	p.E1316K	Non-Targeted Panel Gene	Adult HGBCL, NOS
BCL-76	chr4	95129885	95129885	G	T	exonic	BMPR1B	16	2	0.1111	Missense_Mutation	p.Q203H	Non-Targeted Panel Gene	Adult HGBCL, NOS

BCL-76	chr5	95391393	95391393	C	A	exonic	FAM81B	16	2	0.1111	Missense_Mutation	p.Q2K	Non-Targeted Panel Gene	Adult HGBCL, NOS
BCL-76	chr1	99710916	99710916	C	T	exonic	FRRS1	16	2	0.1111	Missense_Mutation	p.G505E	Non-Targeted Panel Gene	Adult HGBCL, NOS
BCL-76	chr10	94350221	94350221	T	A	exonic	NOC3L	16	2	0.1111	Missense_Mutation	p.E340D	Non-Targeted Panel Gene	Adult HGBCL, NOS
BCL-76	chr2	166477614	166477614	G	A	exonic	SCN7A	16	2	0.1111	Missense_Mutation	p.T28I	Non-Targeted Panel Gene	Adult HGBCL, NOS
BCL-76	chr14	21294762	21294762	C	A	exonic	RPGRIP1	31	4	0.1143	Missense_Mutation	p.H57Q	Non-Targeted Panel Gene	Adult HGBCL, NOS
BCL-76	chr19	48398640	48398640	A	T	exonic	GRIN2D	15	2	0.1176	Missense_Mutation	p.D83V	Non-Targeted Panel Gene	Adult HGBCL, NOS
BCL-76	chr6	160102575	160102575	C	T	exonic	IGF2R	15	2	0.1176	Missense_Mutation	p.S2300L	Non-Targeted Panel Gene	Adult HGBCL, NOS
BCL-76	chr19	48713430	48713430	G	A	exonic	MAMSTR	15	2	0.1176	Missense_Mutation	p.S362F	Non-Targeted Panel Gene	Adult HGBCL, NOS
BCL-76	chr22	30694344	30694344	G	T	exonic	OSBP2	15	2	0.1176	Missense_Mutation	p.R50I	Non-Targeted Panel Gene	Adult HGBCL, NOS
BCL-76	chr11	100976165	100976165	C	T	exonic	ARHGAP42	14	2	0.125	Missense_Mutation	p.P655L	Non-Targeted Panel Gene	Adult HGBCL, NOS
BCL-76	chr2	85550575	85550575	C	A	exonic	GGCX	14	2	0.125	Missense_Mutation	p.K688N	Non-Targeted Panel Gene	Adult HGBCL, NOS
BCL-76	chr16	85071809	85071809	C	A	exonic	KIAA0513	14	2	0.125	Missense_Mutation	p.A119D	Non-Targeted Panel Gene	Adult HGBCL, NOS
BCL-76	chr1	35574024	35574024	C	T	exonic	TFAP2E	14	2	0.125	Missense_Mutation	p.P42L	Non-Targeted Panel Gene	Adult HGBCL, NOS
BCL-76	chr3	126343872	126343872	G	A	exonic	KLF15	20	3	0.1304	Missense_Mutation	p.S369L	Non-Targeted Panel Gene	Adult HGBCL, NOS
BCL-76	chr8	6519940	6519940	G	A	exonic	ANGPT2	13	2	0.1333	Missense_Mutation	p.A285V	Non-Targeted Panel Gene	Adult HGBCL, NOS
BCL-76	chrX	68840429	68840429	C	A	exonic	EFNB1	13	2	0.1333	Missense_Mutation	p.H272Q	Non-Targeted Panel Gene	Adult HGBCL, NOS
BCL-76	chr6	129353333	129353333	G	T	exonic	LAMA2	13	2	0.1333	Missense_Mutation	p.A1565S	Non-Targeted Panel Gene	Adult HGBCL, NOS
BCL-76	chr22	50055321	50055321	T	A	exonic	TTLL8	13	2	0.1333	Missense_Mutation	p.L24F	Non-Targeted Panel Gene	Adult HGBCL, NOS
BCL-76	chr19	8922988	8922988	C	T	exonic	MUC16	19	3	0.1364	Missense_Mutation	p.M12091I	Non-Targeted Panel Gene	Adult HGBCL, NOS
BCL-76	chr21	28966590	28966590	A	T	exonic	LTN1	18	3	0.1429	Missense_Mutation	p.L680H	Non-Targeted Panel Gene	Adult HGBCL, NOS
BCL-76	chr4	25862734	25862734	C	A	exonic	SEL1L3	18	3	0.1429	Missense_Mutation	p.G35C	Non-Targeted Panel Gene	Adult HGBCL, NOS
BCL-76	chrX	123326139	123326139	G	A	exonic	GRIA3	11	2	0.1538	Missense_Mutation	p.E208K	Non-Targeted Panel Gene	Adult HGBCL, NOS
BCL-76	chr14	35625380	35625380	T	A	exonic	RALGAPA1	11	2	0.1538	Missense_Mutation	p.N2304Y	Non-Targeted Panel Gene	Adult HGBCL, NOS
BCL-76	chr1	162773510	162773510	C	A	exonic	DDR2	9	2	0.1818	Missense_Mutation	p.D590E	Non-Targeted Panel Gene	Adult HGBCL, NOS
BCL-76	chr2	73946883	73946883	T	A	exonic	DGUOK	9	2	0.1818	Missense_Mutation	p.F140L	Non-Targeted Panel Gene	Adult HGBCL, NOS
BCL-76	chr16	31111335	31111335	A	G	exonic	BCKDK	25	6	0.1935	Missense_Mutation	p.N294S	Non-Targeted Panel Gene	Adult HGBCL, NOS
BCL-76	chr10	116664860	116664860	C	A	exonic	C10orf82	23	6	0.2069	Missense_Mutation	p.G121V	Non-Targeted Panel Gene	Adult HGBCL, NOS
BCL-76	chrX	36384954	36384954	G	A	exonic	CFAP47	15	4	0.2105	Missense_Mutation	p.R3171K	Non-Targeted Panel Gene	Adult HGBCL, NOS
BCL-76	chr12	101761318	101761318	A	C	exonic	GNPTAB	32	9	0.2195	Missense_Mutation	p.F982V	Non-Targeted Panel Gene	Adult HGBCL, NOS
BCL-76	chr5	13727603	13727603	C	A	exonic	DNAH5	7	2	0.2222	Missense_Mutation	p.E3979D	Non-Targeted Panel Gene	Adult HGBCL, NOS
BCL-76	chr6	52284598	52284598	T	A	exonic	MCM3	7	2	0.2222	Missense_Mutation	p.E71V	Non-Targeted Panel Gene	Adult HGBCL, NOS
BCL-76	chr18	72864821	72864821	G	C	exonic	NETO1	17	5	0.2273	Missense_Mutation	p.I68M	Non-Targeted Panel Gene	Adult HGBCL, NOS
BCL-76	chrY	13324639	13324639	A	T	exonic	UTY	10	3	0.2308	Missense_Mutation	p.V865D	Non-Targeted Panel Gene	Adult HGBCL, NOS
BCL-76	chr6	31581606	31581606	T	C	exonic	LTB	16	5	0.2381	Missense_Mutation	p.E78G	Non-Targeted Panel Gene	Adult HGBCL, NOS
BCL-76	chrX	12706895	12706895	G	A	exonic	FRMPD4	6	2	0.25	Missense_Mutation	p.V423M	Non-Targeted Panel Gene	Adult HGBCL, NOS
BCL-76	chr4	8414888	8414888	T	C	exonic	ACOX3	20	7	0.2593	Missense_Mutation	p.H140R	Non-Targeted Panel Gene	Adult HGBCL, NOS
BCL-76	chr16	87690036	87690036	G	A	exonic	JPH3	50	18	0.2647	Missense_Mutation	p.R559Q	Non-Targeted Panel Gene	Adult HGBCL, NOS
BCL-76	chr12	21936667	21936667	A	G	exonic	ABCC9	38	14	0.2692	Missense_Mutation	p.L3P	Non-Targeted Panel Gene	Adult HGBCL, NOS
BCL-76	chr11	57528887	57528887	G	A	exonic	TIMM10	8	3	0.2727	Missense_Mutation	p.P35S	Non-Targeted Panel Gene	Adult HGBCL, NOS
BCL-76	chr2	43541209	43541209	G	C	exonic	THADA	20	8	0.2857	Missense_Mutation	p.P1072A	Non-Targeted Panel Gene	Adult HGBCL, NOS
BCL-76	chr18	31008132	31008132	C	G	exonic	DSC3	17	7	0.2917	Missense_Mutation	p.G516A	Non-Targeted Panel Gene	Adult HGBCL, NOS
BCL-76	chr5	36995726	36995726	C	T	exonic	NIPBL	17	7	0.2917	Missense_Mutation	p.R1076C	Non-Targeted Panel Gene	Adult HGBCL, NOS
BCL-76	chr2	227259803	227259803	A	C	exonic	COL4A3	12	5	0.2941	Missense_Mutation	p.Y347S	Non-Targeted Panel Gene	Adult HGBCL, NOS
BCL-76	chr14	72892165	72892165	A	C	exonic	DPF3	12	6	0.3158	Missense_Mutation	p.L60R	Non-Targeted Panel Gene	Adult HGBCL, NOS
BCL-76	chr20	31722172	31722172	T	G	exonic	BCL2L1	36	18	0.3333	Missense_Mutation	p.K16T	Non-Targeted Panel Gene	Adult HGBCL, NOS
BCL-76	chr11	55795816	55795816	G	C	exonic	OR5D14	12	6	0.3333	Missense_Mutation	p.L87F	Non-Targeted Panel Gene	Adult HGBCL, NOS
BCL-76	chr1	151234230	151234230	A	G	exonic	PIP5K1A	32	16	0.3333	Missense_Mutation	p.K225E	Non-Targeted Panel Gene	Adult HGBCL, NOS
BCL-76	chr13	79358329	79358329	T	C	exonic	RBM26	10	5	0.3333	Missense_Mutation	p.N545S	Non-Targeted Panel Gene	Adult HGBCL, NOS
BCL-76	chr9	128579718	128579718	T	G	exonic	SPTAN1	13	7	0.3333	Missense_Mutation	p.S435A	Non-Targeted Panel Gene	Adult HGBCL, NOS
BCL-76	chr8	76854533	76854533	A	T	exonic	ZFHX4	31	16	0.3404	Missense_Mutation	p.M2538L	Non-Targeted Panel Gene	Adult HGBCL, NOS
BCL-76	chr6	166324582	166324582	C	T	exonic	SFT2D1	17	9	0.3462	Missense_Mutation	p.G122E	Non-Targeted Panel Gene	Adult HGBCL, NOS
BCL-76	chr9	69516834	69516834	G	T	exonic	APBA1	22	12	0.3529	Missense_Mutation	p.A126D	Non-Targeted Panel Gene	Adult HGBCL, NOS
BCL-76	chr11	7665419	7665419	G	T	exonic	CYB5R2	22	12	0.3529	Missense_Mutation	p.N262K	Non-Targeted Panel Gene	Adult HGBCL, NOS
BCL-76	chr8	2002268	2002268	A	G	exonic	KBTBD11	36	20	0.3571	Missense_Mutation	p.N359S	Non-Targeted Panel Gene	Adult HGBCL, NOS
BCL-76	chr14	75105961	75105961	T	A	exonic	NEK9	16	9	0.36	Missense_Mutation	p.T522S	Non-Targeted Panel Gene	Adult HGBCL, NOS
BCL-76	chr14	75105975	75105975	T	C	exonic	NEK9	16	9	0.36	Missense_Mutation	p.E517G	Non-Targeted Panel Gene	Adult HGBCL, NOS
BCL-76	chr2	215433339	215433339	T	G	exonic	FN1	15	9	0.375	Missense_Mutation	p.S134R	Non-Targeted Panel Gene	Adult HGBCL, NOS
BCL-76	chr2	178741910	178741910	A	G	exonic	TTN	15	9	0.375	Missense_Mutation	p.S3775P	Non-Targeted Panel Gene	Adult HGBCL, NOS
BCL-76	chr9	137034978	137034978	A	G	exonic	C9orf139	29	18	0.383	Missense_Mutation	p.E166G	Non-Targeted Panel Gene	Adult HGBCL, NOS
BCL-76	chr4	79325518	79325518	G	T	exonic	NAA11	24	15	0.3846	Missense_Mutation	p.H120Q	Non-Targeted Panel Gene	Adult HGBCL, NOS
BCL-76	chr2	178574140	178574140	G	A	exonic	TTN	22	14	0.3889	Missense_Mutation	p.R23998C	Non-Targeted Panel Gene	Adult HGBCL, NOS
BCL-76	chr10	97445967	97445967	A	C	exonic	EXOSC1	29	19	0.3958	Missense_Mutation	p.Y7D	Non-Targeted Panel Gene	Adult HGBCL, NOS
BCL-76	chr15	66781278	66781278	G	A	exonic	SMAD6	61	40	0.396	Missense_Mutation	p.V412I	Non-Targeted Panel Gene	Adult HGBCL, NOS
BCL-76	chr15	99733358	99733358	C	A	exonic	LYSMD4	12	8	0.4	Missense_Mutation	p.S29I	Non-Targeted Panel Gene	Adult HGBCL, NOS
BCL-76	chr9	93253042	93253042	G	T	exonic	WNK2	30	20	0.4	Missense_Mutation	p.S665I	Non-Targeted Panel Gene	Adult HGBCL, NOS
BCL-76	chr11	70487445	70487445	T	C	exonic	SHANK2	31	22	0.4151	Missense_Mutation	p.M950V	Non-Targeted Panel Gene	Adult HGBCL, NOS
BCL-76	chr22	37632474	37632474	T	C	exonic	GGA1	39	28	0.4179	Missense_Mutation	p.S517P	Non-Targeted Panel Gene	Adult HGBCL, NOS
BCL-76	chr9	108911096	108911096	T	G	exonic	ELP1	11	8	0.4211	Missense_Mutation	p.N425T	Non-Targeted Panel Gene	Adult HGBCL, NOS
BCL-76	chr8	31058542	31058542	A	G	exonic	WRN	12	9	0.4286	Missense_Mutation	p.K32R	Non-Targeted Panel Gene	Adult HGBCL, NOS
BCL-76	chr15	90477814	90477814	T	G	exonic	IQGAP1	21	16	0.4324	Missense_Mutation	p.L1085R	Non-Targeted Panel Gene	Adult HGBCL, NOS
BCL-76	chr15	64926919	64926919	C	T	exonic	ANKDD1A	10	8	0.4444	Missense_Mutation	p.H164Y	Non-Targeted Panel Gene	Adult HGBCL, NOS
BCL-76	chr1	154568061	154568061	G	A	exonic	CHRNB2	21	17	0.4474	Missense_Mutation	p.G6D	Non-Targeted Panel Gene	Adult HGBCL, NOS
BCL-76	chr11	74457316	74457316	C	T	exonic	KCNE3	18	15	0.4545	Missense_Mutation	p.R83H	Non-Targeted Panel Gene	Adult HGBCL, NOS
BCL-76	chr19	36125520	36125520	G	T	exonic	TBCB	30	25	0.4545	Missense_Mutation	p.G155V	Non-Targeted Panel Gene	Adult HGBCL, NOS
BCL-76	chr6	34858541	34858541	T	G	exonic	UHRF1BP1	32	27	0.4576	Missense_Mutation	p.C729G	Non-Targeted Panel Gene	Adult HGBCL, NOS
BCL-76	chr1	229342102	229342102	G	A	exonic	CCSAP	26	22	0.4583	Missense_Mutation	p.P122S	Non-Targeted Panel Gene	Adult HGBCL, NOS
BCL-76	chr8	47961569	47961569	T	G	exonic	MCM4	23	20	0.4651	Missense_Mutation	p.S42A	Non-Targeted Panel Gene	Adult HGBCL, NOS
BCL-76	chr19	4668630	4668630	T	G	exonic	MYDGF	10	9	0.4737	Missense_Mutation	p.M64L	Non-Targeted Panel Gene	Adult HGBCL, NOS
BCL-76	chr6	26104123	26104123	T	C	exonic	HIST1H4C	22	20	0.4762	Missense_Mutation	p.L59P	Non-Targeted Panel Gene	Adult HGBCL, NOS
BCL-76	chr1	183142610	183142610	G	C	exonic	LAMC1	11	10	0.4762	Missense_Mutation	p.K1550N	Non-Targeted Panel Gene	Adult HGBCL, NOS
BCL-76	chr19	966770	966770	T	G	exonic	ARID3A	14	13	0.4815	Missense_Mutation	p.V466G	Non-Targeted Panel Gene	Adult HGBCL, NOS
BCL-76	chr19	5909095	5909095	G	A	exonic	VMAC	14	13	0.4815	Missense_Mutation	p.A155T	Non-Targeted Panel Gene	Adult HGBCL, NOS

BCL-76	chr16	49396437	49396437	G	T	exonic	C16orf78	15	14	0.4828	Missense_Mutation	p.D137Y	Non-Targeted Panel Gene	Adult HGBCL, NOS
BCL-76	chr11	123977495	123977495	G	T	exonic	OR10S1	15	14	0.4828	Missense_Mutation	p.S66Y	Non-Targeted Panel Gene	Adult HGBCL, NOS
BCL-76	chr1	31738274	31738274	G	T	exonic	ADGRB2	11	11	0.5	Missense_Mutation	p.Q900K	Non-Targeted Panel Gene	Adult HGBCL, NOS
BCL-76	chr12	112068961	112068961	A	T	exonic	NAA25	14	14	0.5	Missense_Mutation	p.F356L	Non-Targeted Panel Gene	Adult HGBCL, NOS
BCL-76	chr16	67259905	67259905	C	T	exonic	SLC9A5	5	5	0.5	Missense_Mutation	p.H601Y	Non-Targeted Panel Gene	Adult HGBCL, NOS
BCL-76	chr7	143444394	143444394	G	T	exonic	TAS2R60	5	5	0.5	Missense_Mutation	p.R314S	Non-Targeted Panel Gene	Adult HGBCL, NOS
BCL-76	chr8	76853740	76853740	G	C	exonic	ZFHX4	25	26	0.5098	Missense_Mutation	p.W2273C	Non-Targeted Panel Gene	Adult HGBCL, NOS
BCL-76	chr7	117322935	117322935	G	A	exonic	WNT2	38	40	0.5128	Missense_Mutation	p.L19F	Non-Targeted Panel Gene	Adult HGBCL, NOS
BCL-76	chr1	234231721	234231721	G	T	exonic	SLC35F3	15	16	0.5161	Missense_Mutation	p.Q196H	Non-Targeted Panel Gene	Adult HGBCL, NOS
BCL-76	chr4	73144765	73144765	A	G	exonic	ANKRD17	11	12	0.5217	Missense_Mutation	p.V646A	Non-Targeted Panel Gene	Adult HGBCL, NOS
BCL-76	chr13	102868215	102868215	A	G	exonic	BIVM-ERCC5;ERCC	11	13	0.5417	Missense_Mutation	p.N1333S	Non-Targeted Panel Gene	Adult HGBCL, NOS
BCL-76	chr11	74005745	74005745	T	C	exonic	UCP3	32	38	0.5429	Missense_Mutation	p.R176G	Non-Targeted Panel Gene	Adult HGBCL, NOS
BCL-76	chr1	89147567	89147567	C	G	exonic	GBP7	5	6	0.5455	Missense_Mutation	p.K455N	Non-Targeted Panel Gene	Adult HGBCL, NOS
BCL-76	chr12	40922290	40922290	A	C	exonic	CNTN1	14	17	0.5484	Missense_Mutation	p.S88R	Non-Targeted Panel Gene	Adult HGBCL, NOS
BCL-76	chr8	31087874	31087874	A	T	exonic	WRN	8	10	0.5556	Missense_Mutation	p.E510D	Non-Targeted Panel Gene	Adult HGBCL, NOS
BCL-76	chr13	103049340	103049340	G	A	exonic	SLC10A2	17	22	0.5641	Missense_Mutation	p.P290S	Non-Targeted Panel Gene	Adult HGBCL, NOS
BCL-76	chr7	41965982	41965982	T	C	exonic	GLI3	15	20	0.5714	Missense_Mutation	p.N1031D	Non-Targeted Panel Gene	Adult HGBCL, NOS
BCL-76	chr7	151181133	151181133	G	A	exonic	ASB10	11	15	0.5769	Missense_Mutation	p.R289C	Non-Targeted Panel Gene	Adult HGBCL, NOS
BCL-76	chr6	152284112	152284112	C	T	exonic	SYNE1	11	15	0.5769	Missense_Mutation	p.A6025T	Non-Targeted Panel Gene	Adult HGBCL, NOS
BCL-76	chr6	137493483	137493483	C	T	exonic	OLIG3	15	22	0.5946	Missense_Mutation	p.G230S	Non-Targeted Panel Gene	Adult HGBCL, NOS
BCL-76	chr2	68186605	68186605	C	G	exonic	PPP3R1	14	21	0.6	Missense_Mutation	p.G110R	Non-Targeted Panel Gene	Adult HGBCL, NOS
BCL-76	chr17	14206977	14206977	G	T	exonic	COX10	21	33	0.6111	Missense_Mutation	p.V366L	Non-Targeted Panel Gene	Adult HGBCL, NOS
BCL-76	chr22	43222650	43222650	G	A	exonic	SCUBE1	10	16	0.6154	Missense_Mutation	p.P474S	Non-Targeted Panel Gene	Adult HGBCL, NOS
BCL-76	chr11	124647396	124647396	G	A	exonic	SIAE	11	19	0.6333	Missense_Mutation	p.T277M	Non-Targeted Panel Gene	Adult HGBCL, NOS
BCL-76	chr19	50668856	50668856	G	A	exonic	SHANK1	5	10	0.6667	Missense_Mutation	p.P1035L	Non-Targeted Panel Gene	Adult HGBCL, NOS
BCL-76	chr12	132816542	132816542	A	C	exonic	GOLGA3	16	44	0.7333	Missense_Mutation	p.L135R	Non-Targeted Panel Gene	Adult HGBCL, NOS
BCL-76	chr16	70664606	70664606	G	A	exonic	MTSS1L	3	11	0.7857	Missense_Mutation	p.P488L	Non-Targeted Panel Gene	Adult HGBCL, NOS
BCL-76	chr17	81198225	81198225	T	C	exonic	CEP131	3	24	0.8889	Missense_Mutation	p.R454G	Non-Targeted Panel Gene	Adult HGBCL, NOS
BCL-76	chrX	47084519	47084519	G	T	exonic	RGN	2	16	0.8889	Missense_Mutation	p.V89F	Non-Targeted Panel Gene	Adult HGBCL, NOS
BCL-76	chr3	38575385	38575385	C	T	exonic	SCN5A	4	32	0.8889	Missense_Mutation	p.R1193Q	Non-Targeted Panel Gene	Adult HGBCL, NOS
BCL-76	chrX	73214476	73214476	T	A	exonic	NAP1L2	3	25	0.8929	Missense_Mutation	p.N6I	Non-Targeted Panel Gene	Adult HGBCL, NOS
BCL-76	chr17	45983180	45983180	C	A	exonic	MAPT	3	26	0.8966	Missense_Mutation	p.P126T	Non-Targeted Panel Gene	Adult HGBCL, NOS
BCL-76	chr17	64213538	64213538	C	T	exonic	TEX2	3	45	0.9375	Missense_Mutation	p.R227Q	Non-Targeted Panel Gene	Adult HGBCL, NOS
BCL-76	chr17	80104893	80104893	T	G	exonic	GAA	3	60	0.9524	Missense_Mutation	p.C103G	Non-Targeted Panel Gene	Adult HGBCL, NOS
BCL-76	chrX	115190457	115190457	C	A	exonic	RBMXL3	0	36	1	Missense_Mutation	p.A339D	Non-Targeted Panel Gene	Adult HGBCL, NOS
BCL-76	chr1	216072958	216072958	G	A	exonic	USH2A	15	2	0.1176	Nonsense_Mutation	p.R1930X	Targerted Panel Gene	Adult HGBCL, NOS
BCL-76	chr6	26123653	26123653	G	C	exonic	HIST1H2BC	59	47	0.4434	Nonsense_Mutation	p.Y84X	Targerted Panel Gene	Adult HGBCL, NOS
BCL-76	chr15	44715447	44715447	C	G	exonic	B2M	17	22	0.5641	Nonsense_Mutation	p.S31X	Targerted Panel Gene	Adult HGBCL, NOS
BCL-76	chr19	48318485	48318485	G	A	exonic	CCDC114	52	2	0.037	Nonsense_Mutation	p.Q51X	Non-Targeted Panel Gene	Adult HGBCL, NOS
BCL-76	chr1	161160704	161160704	C	T	exonic	USP21	88	4	0.0435	Nonsense_Mutation	p.R22X	Non-Targeted Panel Gene	Adult HGBCL, NOS
BCL-76	chr12	85301428	85301428	C	T	exonic	ALX1	55	3	0.0517	Nonsense_Mutation	p.R312X	Non-Targeted Panel Gene	Adult HGBCL, NOS
BCL-76	chr19	11723230	11723230	C	A	exonic	ZNF823	53	3	0.0536	Nonsense_Mutation	p.E102X	Non-Targeted Panel Gene	Adult HGBCL, NOS
BCL-76	chr15	57516819	57516819	C	T	exonic	CGNL1	31	2	0.0606	Nonsense_Mutation	p.Q815X	Non-Targeted Panel Gene	Adult HGBCL, NOS
BCL-76	chr8	144467266	144467266	C	T	exonic	KIFC2	69	5	0.0676	Nonsense_Mutation	p.R132X	Non-Targeted Panel Gene	Adult HGBCL, NOS
BCL-76	chr12	26916154	26916154	G	A	exonic	INTS13	25	2	0.0741	Nonsense_Mutation	p.R366X	Non-Targeted Panel Gene	Adult HGBCL, NOS
BCL-76	chr8	99467606	99467606	C	A	exonic	VPS13B	20	2	0.0909	Nonsense_Mutation	p.S1213X	Non-Targeted Panel Gene	Adult HGBCL, NOS
BCL-76	chr5	90642724	90642724	G	T	exonic	ADGRV1	19	2	0.0952	Nonsense_Mutation	p.E777X	Non-Targeted Panel Gene	Adult HGBCL, NOS
BCL-76	chr7	97022283	97022283	G	A	exonic	DLX5	18	2	0.1	Nonsense_Mutation	p.Q148X	Non-Targeted Panel Gene	Adult HGBCL, NOS
BCL-76	chr22	39015714	39015714	C	A	exonic	APOBEC3C	17	2	0.1053	Nonsense_Mutation	p.S46X	Non-Targeted Panel Gene	Adult HGBCL, NOS
BCL-76	chr11	57804763	57804763	A	T	exonic	CTNND1	16	2	0.1111	Nonsense_Mutation	p.K569X	Non-Targeted Panel Gene	Adult HGBCL, NOS
BCL-76	chr1	209650023	209650023	G	A	exonic	LAMB3	21	3	0.125	Nonsense_Mutation	p.R42X	Non-Targeted Panel Gene	Adult HGBCL, NOS
BCL-76	chr5	151287458	151287458	C	A	exonic	SLC36A3	12	2	0.1429	Nonsense_Mutation	p.E207X	Non-Targeted Panel Gene	Adult HGBCL, NOS
BCL-76	chr2	229768697	229768697	C	A	exonic	TRIP12	11	2	0.1538	Nonsense_Mutation	p.E1942X	Non-Targeted Panel Gene	Adult HGBCL, NOS
BCL-76	chr10	112404725	112404725	C	A	exonic	ACSL5	35	16	0.3137	Nonsense_Mutation	p.Y117X	Non-Targeted Panel Gene	Adult HGBCL, NOS
BCL-76	chr3	196827280	196827280	C	T	exonic	PAK2	20	18	0.4737	Nonsense_Mutation	p.R479X	Non-Targeted Panel Gene	Adult HGBCL, NOS
BCL-76	chr17	10412667	10412667	G	T	exonic	MYH8	10	11	0.5238	Nonsense_Mutation	p.C403X	Non-Targeted Panel Gene	Adult HGBCL, NOS
BCL-76	chr3	38141150	38141150	T	C	exonic	MYD88	6	40	0.8696	Nonstop_Mutation	p.L265P	Targerted Panel Gene	Adult HGBCL, NOS
BCL-77	chr1	205720500	205720500	C	T	splicing	NUCKS1	40	2	0.0476	Splice_Site	.	Non-Targeted Panel Gene	Adult HGBCL, NOS
BCL-77	chr20	35384135	35384136	TG	-	splicing	UQCC1	37	2	0.0513	Splice_Site	.	Non-Targeted Panel Gene	Adult HGBCL, NOS
BCL-77	chr11	124118709	124118709	G	C	splicing	VWA5A	27	2	0.0667	Splice_Site	.	Non-Targeted Panel Gene	Adult HGBCL, NOS
BCL-77	chr12	57269321	57269322	AC	-	splicing	R3HDM2	19	2	0.0952	Splice_Site	.	Non-Targeted Panel Gene	Adult HGBCL, NOS
BCL-77	chr16	22096946	22096947	GT	-	splicing	VWA3A	28	4	0.125	Splice_Site	.	Non-Targeted Panel Gene	Adult HGBCL, NOS
BCL-77	chr8	47800921	47800922	AG	-	exonic	PRKDC	21	2	0.087	Frame_Shift_Del	p.L3329fs	Targerted Panel Gene	Adult HGBCL, NOS
BCL-77	chr5	112827200	112827200	G	-	exonic	APC	29	3	0.0938	Frame_Shift_Del	p.A501fs	Targerted Panel Gene	Adult HGBCL, NOS
BCL-77	chr17	16143690	16143690	C	-	exonic	NCOR1	11	11	0.5	Frame_Shift_Del	p.G363fs	Targerted Panel Gene	Adult HGBCL, NOS
BCL-77	chr18	63564047	63564047	T	-	exonic	SERPINB12	55	2	0.0351	Frame_Shift_Del	p.V191fs	Non-Targeted Panel Gene	Adult HGBCL, NOS
BCL-77	chr6	151366024	151366025	TG	-	exonic	ZBTB2	51	2	0.0377	Frame_Shift_Del	p.D347fs	Non-Targeted Panel Gene	Adult HGBCL, NOS
BCL-77	chr9	76328178	76328179	CC	-	exonic	PCSK5	45	2	0.0426	Frame_Shift_Del	p.F1476fs	Non-Targeted Panel Gene	Adult HGBCL, NOS
BCL-77	chr9	27455247	27455250	CCTG	-	exonic	MOB3B	44	2	0.0435	Frame_Shift_Del	p.Q101fs	Non-Targeted Panel Gene	Adult HGBCL, NOS
BCL-77	chr7	12369824	12369825	CA	-	exonic	VWDE	44	2	0.0435	Frame_Shift_Del	p.L827fs	Non-Targeted Panel Gene	Adult HGBCL, NOS
BCL-77	chr9	122911890	122911894	CTGAT	-	exonic	ZBTB6	42	2	0.0455	Frame_Shift_Del	p.D60fs	Non-Targeted Panel Gene	Adult HGBCL, NOS
BCL-77	chr2	71611248	71611248	G	-	exonic	DYSF	41	2	0.0465	Frame_Shift_Del	p.E1335fs	Non-Targeted Panel Gene	Adult HGBCL, NOS
BCL-77	chr18	47848561	47848562	TC	-	exonic	SMAD2	41	2	0.0465	Frame_Shift_Del	p.D304fs	Non-Targeted Panel Gene	Adult HGBCL, NOS
BCL-77	chr1	17070206	17070207	TC	-	exonic	PADI2	37	2	0.0513	Frame_Shift_Del	p.D549fs	Non-Targeted Panel Gene	Adult HGBCL, NOS
BCL-77	chr1	200974843	200974843	C	-	exonic	KIF21B	36	2	0.0526	Frame_Shift_Del	p.S1562fs	Non-Targeted Panel Gene	Adult HGBCL, NOS
BCL-77	chr22	36937846	36937847	GG	-	exonic	CSF2RB	35	2	0.0541	Frame_Shift_Del	p.G680fs	Non-Targeted Panel Gene	Adult HGBCL, NOS
BCL-77	chr6	108912383	108912383	A	-	exonic	ARMC2	30	2	0.0625	Frame_Shift_Del	p.E392fs	Non-Targeted Panel Gene	Adult HGBCL, NOS
BCL-77	chr11	5234066	5234066	G	-	exonic	HBD	28	2	0.0667	Frame_Shift_Del	p.D80fs	Non-Targeted Panel Gene	Adult HGBCL, NOS
BCL-77	chr10	73274457	73274458	CC	-	exonic	CFAP70	26	2	0.0714	Frame_Shift_Del	p.G1007fs	Non-Targeted Panel Gene	Adult HGBCL, NOS
BCL-77	chr17	16622410	16622414	GTACA	-	exonic	ZNF624	26	2	0.0714	Frame_Shift_Del	p.T824fs	Non-Targeted Panel Gene	Adult HGBCL, NOS
BCL-77	chrX	54470258	54470258	T	-	exonic	FGD1	25	2	0.0741	Frame_Shift_Del	p.S287fs	Non-Targeted Panel Gene	Adult HGBCL, NOS
BCL-77	chr1	225505009	225505022	GACCTGTTGTCAAA	-	exonic	ENAH	24	2	0.0769	Frame_Shift_Del	p.F524fs	Non-Targeted Panel Gene	Adult HGBCL, NOS

BCL-77	chr12	51452149	51452150	CA	-	exonic	SLC4A8	24	2	0.0769	Frame_Shift_Del	p.F48fs	Non-Targeted Panel Gene	Adult HGBCL, NOS
BCL-77	chr17	11752845	11752846	CC	-	exonic	DNAH9	31	3	0.0882	Frame_Shift_Del	p.S2208fs	Non-Targeted Panel Gene	Adult HGBCL, NOS
BCL-77	chr6	118856379	118856380	AG	-	exonic	MCM9	19	2	0.0952	Frame_Shift_Del	p.A439fs	Non-Targeted Panel Gene	Adult HGBCL, NOS
BCL-77	chr7	86880526	86880531	TTTTCC	-	exonic	KIAA1324L	17	2	0.1053	Frame_Shift_Del	p.E897fs	Non-Targeted Panel Gene	Adult HGBCL, NOS
BCL-77	chr1	229465450	229465453	CTAG	-	exonic	NUP133	16	2	0.1111	Frame_Shift_Del	p.L756fs	Non-Targeted Panel Gene	Adult HGBCL, NOS
BCL-77	chr6	125276196	125276196	C	-	exonic	HDDC2	21	4	0.16	Frame_Shift_Del	p.A189fs	Non-Targeted Panel Gene	Adult HGBCL, NOS
BCL-77	chr11	123753856	123753856	G	-	exonic	OR6X1	8	8	0.5	Frame_Shift_Del	p.S221fs	Non-Targeted Panel Gene	Adult HGBCL, NOS
BCL-77	chr3	109114414	109114414	-	AA	exonic	MORC1	53	2	0.0364	Frame_Shift_Ins	p.G30fs	Non-Targeted Panel Gene	Adult HGBCL, NOS
BCL-77	chr19	47802104	47802104	-	CCCGATTT	exonic	TPRX1	38	4	0.0952	Frame_Shift_Ins	p.A303fs	Non-Targeted Panel Gene	Adult HGBCL, NOS
BCL-77	chr19	3013703	3013703	-	T	exonic	TLE2	10	8	0.4444	Frame_Shift_Ins	p.G294fs	Non-Targeted Panel Gene	Adult HGBCL, NOS
BCL-77	chr5	141672297	141672299	TGA	-	exonic	ARAP3	53	2	0.0364	In_Frame_Del	p.463_464del	Non-Targeted Panel Gene	Adult HGBCL, NOS
BCL-77	chr19	12825790	12825792	GGG	-	exonic	RTBDN	49	2	0.0392	In_Frame_Del	p.234_234del	Non-Targeted Panel Gene	Adult HGBCL, NOS
BCL-77	chr4	155374957	155374959	TCA	-	exonic	MAP9	46	2	0.0417	In_Frame_Del	p.46_47del	Non-Targeted Panel Gene	Adult HGBCL, NOS
BCL-77	chr19	38352401	38352403	TAC	-	exonic	CATSPERG	45	2	0.0426	In_Frame_Del	p.322_323del	Non-Targeted Panel Gene	Adult HGBCL, NOS
BCL-77	chr1	111449847	111449849	GAA	-	exonic	ATP5F1	44	2	0.0435	In_Frame_Del	p.17_18del	Non-Targeted Panel Gene	Adult HGBCL, NOS
BCL-77	chr20	33008599	33008610	CTGCTGCTGGCA	-	exonic	BPIFB2	43	2	0.0444	In_Frame_Del	p.9_12del	Non-Targeted Panel Gene	Adult HGBCL, NOS
BCL-77	chr13	113732946	113732954	CAGCCAGGC	-	exonic	GRK1	42	2	0.0455	In_Frame_Del	p.419_422del	Non-Targeted Panel Gene	Adult HGBCL, NOS
BCL-77	chr11	123943522	123943530	AAGAAGTAG	-	exonic	OR6T1	40	2	0.0476	In_Frame_Del	p.103_106del	Non-Targeted Panel Gene	Adult HGBCL, NOS
BCL-77	chr19	18001355	18001357	CGC	-	exonic	ARRDC2	36	2	0.0526	In_Frame_Del	p.14_15del	Non-Targeted Panel Gene	Adult HGBCL, NOS
BCL-77	chr1	156624146	156624148	CCT	-	exonic	HAPLN2	33	2	0.0571	In_Frame_Del	p.142_143del	Non-Targeted Panel Gene	Adult HGBCL, NOS
BCL-77	chr6	30951544	30951549	CACCAT	-	exonic	DPCR1	78	5	0.0602	In_Frame_Del	p.1027_1029del	Non-Targeted Panel Gene	Adult HGBCL, NOS
BCL-77	chr19	3013697	3013702	GGTGAG	-	exonic	TLE2	9	6	0.4	In_Frame_Del	p.294_296del	Non-Targeted Panel Gene	Adult HGBCL, NOS
BCL-77	chr4	109870499	109870501	CTT	-	exonic	LRIT3	25	23	0.4792	In_Frame_Del	p.584_584del	Non-Targeted Panel Gene	Adult HGBCL, NOS
BCL-77	chr19	12825796	12825796	-	CCC	exonic	RTBDN	51	4	0.0727	In_Frame_Ins	p.E232delinsEG	Non-Targeted Panel Gene	Adult HGBCL, NOS
BCL-77	chr1	201209182	201209182	C	T	exonic	IGFN1	59	2	0.0328	Missense_Mutation	p.A1430V	Targerted Panel Gene	Adult HGBCL, NOS
BCL-77	chr5	168262553	168262553	G	A	exonic	TENM2	44	3	0.0638	Missense_Mutation	p.E2681K	Targerted Panel Gene	Adult HGBCL, NOS
BCL-77	chr7	55202570	55202570	C	A	exonic	EGFR	29	2	0.0645	Missense_Mutation	p.D1072E	Targerted Panel Gene	Adult HGBCL, NOS
BCL-77	chr12	50351985	50351985	G	A	exonic	FAM186A	57	4	0.0656	Missense_Mutation	p.T1616I	Targerted Panel Gene	Adult HGBCL, NOS
BCL-77	chr12	50351979	50351979	T	G	exonic	FAM186A	54	4	0.069	Missense_Mutation	p.Q1618P	Targerted Panel Gene	Adult HGBCL, NOS
BCL-77	chr4	186613115	186613115	C	T	exonic	FAT1	20	2	0.0909	Missense_Mutation	p.D3153N	Targerted Panel Gene	Adult HGBCL, NOS
BCL-77	chrX	77682778	77682778	C	A	exonic	ATRX	18	2	0.1	Missense_Mutation	p.K826N	Targerted Panel Gene	Adult HGBCL, NOS
BCL-77	chr19	11041542	11041542	T	C	exonic	SMARCA4	17	2	0.1053	Missense_Mutation	p.V1501A	Targerted Panel Gene	Adult HGBCL, NOS
BCL-77	chr7	103574104	103574104	G	A	exonic	RELN	14	2	0.125	Missense_Mutation	p.T1500I	Targerted Panel Gene	Adult HGBCL, NOS
BCL-77	chr15	33785788	33785788	A	T	exonic	RYR3	23	4	0.1481	Missense_Mutation	p.E3132V	Targerted Panel Gene	Adult HGBCL, NOS
BCL-77	chr15	41875561	41875561	T	A	exonic	SPTBN5	30	9	0.2308	Missense_Mutation	p.Q1395L	Targerted Panel Gene	Adult HGBCL, NOS
BCL-77	chr12	121804905	121804905	C	A	exonic	SETD1B	31	21	0.4038	Missense_Mutation	p.S56R	Targerted Panel Gene	Adult HGBCL, NOS
BCL-77	chr1	228372002	228372002	G	A	exonic	OBSCN	25	19	0.4318	Missense_Mutation	p.S8032N	Targerted Panel Gene	Adult HGBCL, NOS
BCL-77	chr12	111348026	111348026	C	A	exonic	CUX2	35	33	0.4783	Missense_Mutation	p.R1388S	Targerted Panel Gene	Adult HGBCL, NOS
BCL-77	chr6	394888	394888	C	A	exonic	IRF4	18	20	0.5263	Missense_Mutation	p.T95K	Targerted Panel Gene	Adult HGBCL, NOS
BCL-77	chr10	127738639	127738639	A	G	exonic	FOXI2	75	2	0.026	Missense_Mutation	p.S211G	Non-Targeted Panel Gene	Adult HGBCL, NOS
BCL-77	chr11	65547523	65547523	A	T	exonic	LTBP3	67	2	0.029	Missense_Mutation	p.F675I	Non-Targeted Panel Gene	Adult HGBCL, NOS
BCL-77	chr18	63564042	63564042	T	A	exonic	SERPINB12	55	2	0.0351	Missense_Mutation	p.N189K	Non-Targeted Panel Gene	Adult HGBCL, NOS
BCL-77	chr17	39225494	39225494	C	A	exonic	STAC2	54	2	0.0357	Missense_Mutation	p.E3D	Non-Targeted Panel Gene	Adult HGBCL, NOS
BCL-77	chr10	48731167	48731167	C	A	exonic	WDFY4	54	2	0.0357	Missense_Mutation	p.A396D	Non-Targeted Panel Gene	Adult HGBCL, NOS
BCL-77	chr17	74484076	74484076	C	A	exonic	CD300A	52	2	0.037	Missense_Mutation	p.P284T	Non-Targeted Panel Gene	Adult HGBCL, NOS
BCL-77	chr15	88879183	88879183	C	G	exonic	HAPLN3	52	2	0.037	Missense_Mutation	p.V256L	Non-Targeted Panel Gene	Adult HGBCL, NOS
BCL-77	chr6	155314304	155314304	C	T	exonic	TFB1M	49	2	0.0392	Missense_Mutation	p.R42K	Non-Targeted Panel Gene	Adult HGBCL, NOS
BCL-77	chr15	34793253	34793253	C	T	exonic	ACTC1	48	2	0.04	Missense_Mutation	p.R149H	Non-Targeted Panel Gene	Adult HGBCL, NOS
BCL-77	chr22	50142108	50142108	G	A	exonic	MOV10L1	48	2	0.04	Missense_Mutation	p.E700K	Non-Targeted Panel Gene	Adult HGBCL, NOS
BCL-77	chr14	37622765	37622765	G	C	exonic	TTC6	47	2	0.0408	Missense_Mutation	p.S250T	Non-Targeted Panel Gene	Adult HGBCL, NOS
BCL-77	chr20	31605653	31605653	T	A	exonic	ID1	46	2	0.0417	Missense_Mutation	p.V89E	Non-Targeted Panel Gene	Adult HGBCL, NOS
BCL-77	chr1	156913951	156913951	C	T	exonic	PEAR1	46	2	0.0417	Missense_Mutation	p.P938L	Non-Targeted Panel Gene	Adult HGBCL, NOS
BCL-77	chr9	76328180	76328180	A	T	exonic	PCSK5	44	2	0.0435	Missense_Mutation	p.H1477L	Non-Targeted Panel Gene	Adult HGBCL, NOS
BCL-77	chr11	113393588	113393588	T	G	exonic	ANKK1	43	2	0.0444	Missense_Mutation	p.M98R	Non-Targeted Panel Gene	Adult HGBCL, NOS
BCL-77	chr7	149006034	149006034	G	A	exonic	PDIA4	43	2	0.0444	Missense_Mutation	p.A384V	Non-Targeted Panel Gene	Adult HGBCL, NOS
BCL-77	chr19	13963211	13963211	G	A	exonic	RFX1	86	4	0.0444	Missense_Mutation	p.R879W	Non-Targeted Panel Gene	Adult HGBCL, NOS
BCL-77	chr3	52487458	52487458	G	A	exonic	NISCH	42	2	0.0455	Missense_Mutation	p.E656K	Non-Targeted Panel Gene	Adult HGBCL, NOS
BCL-77	chr3	49057176	49057176	C	T	exonic	QRICH1	84	4	0.0455	Missense_Mutation	p.V342I	Non-Targeted Panel Gene	Adult HGBCL, NOS
BCL-77	chr2	71611249	71611249	A	T	exonic	DYSF	41	2	0.0465	Missense_Mutation	p.E1335V	Non-Targeted Panel Gene	Adult HGBCL, NOS
BCL-77	chr17	80113293	80113293	C	T	exonic	GAA	41	2	0.0465	Missense_Mutation	p.L706F	Non-Targeted Panel Gene	Adult HGBCL, NOS
BCL-77	chr16	68352296	68352296	T	C	exonic	PRMT7	41	2	0.0465	Missense_Mutation	p.W488R	Non-Targeted Panel Gene	Adult HGBCL, NOS
BCL-77	chr3	9946693	9946693	C	T	exonic	PRRT3	101	5	0.0472	Missense_Mutation	p.R827Q	Non-Targeted Panel Gene	Adult HGBCL, NOS
BCL-77	chr14	102015983	102015983	C	T	exonic	DYNC1H1	60	3	0.0476	Missense_Mutation	p.S2457L	Non-Targeted Panel Gene	Adult HGBCL, NOS
BCL-77	chr4	48606544	48606544	C	T	exonic	FRYL	40	2	0.0476	Missense_Mutation	p.S212N	Non-Targeted Panel Gene	Adult HGBCL, NOS
BCL-77	chr16	69114400	69114400	G	A	exonic	HAS3	40	2	0.0476	Missense_Mutation	p.A266T	Non-Targeted Panel Gene	Adult HGBCL, NOS
BCL-77	chr5	177135855	177135855	C	A	exonic	NSD1	40	2	0.0476	Missense_Mutation	p.A251E	Non-Targeted Panel Gene	Adult HGBCL, NOS
BCL-77	chr16	30768703	30768703	G	T	exonic	RNF40	38	2	0.05	Missense_Mutation	p.Q688H	Non-Targeted Panel Gene	Adult HGBCL, NOS
BCL-77	chr19	18001375	18001375	T	A	exonic	ARRDC2	37	2	0.0513	Missense_Mutation	p.Y21N	Non-Targeted Panel Gene	Adult HGBCL, NOS
BCL-77	chr16	56981200	56981200	C	A	exonic	CETP	36	2	0.0526	Missense_Mutation	p.L397I	Non-Targeted Panel Gene	Adult HGBCL, NOS
BCL-77	chr10	97459508	97459508	C	T	exonic	MMS19	36	2	0.0526	Missense_Mutation	p.E959K	Non-Targeted Panel Gene	Adult HGBCL, NOS
BCL-77	chr9	83977745	83977745	T	G	exonic	HNRNPK	35	2	0.0541	Missense_Mutation	p.K34Q	Non-Targeted Panel Gene	Adult HGBCL, NOS
BCL-77	chr11	66545910	66545910	C	T	exonic	ZDHHC24	35	2	0.0541	Missense_Mutation	p.E32K	Non-Targeted Panel Gene	Adult HGBCL, NOS
BCL-77	chr14	69573286	69573286	G	A	exonic	CCDC177	69	4	0.0548	Missense_Mutation	p.R113W	Non-Targeted Panel Gene	Adult HGBCL, NOS
BCL-77	chr1	200974844	200974844	T	A	exonic	KIF21B	34	2	0.0556	Missense_Mutation	p.S1562C	Non-Targeted Panel Gene	Adult HGBCL, NOS
BCL-77	chr16	30748845	30748845	G	A	exonic	PHKG2	34	2	0.0556	Missense_Mutation	p.D9N	Non-Targeted Panel Gene	Adult HGBCL, NOS
BCL-77	chr4	1699623	1699623	C	A	exonic	SLBP	50	3	0.0566	Missense_Mutation	p.K140N	Non-Targeted Panel Gene	Adult HGBCL, NOS
BCL-77	chr13	102742087	102742087	C	T	exonic	CCDC168	33	2	0.0571	Missense_Mutation	p.M2870I	Non-Targeted Panel Gene	Adult HGBCL, NOS
BCL-77	chr5	35957262	35957262	G	A	exonic	UGT3A1	32	2	0.0588	Missense_Mutation	p.S334F	Non-Targeted Panel Gene	Adult HGBCL, NOS
BCL-77	chr19	21537468	21537468	G	A	exonic	ZNF429	64	4	0.0588	Missense_Mutation	p.R440K	Non-Targeted Panel Gene	Adult HGBCL, NOS
BCL-77	chr17	81996650	81996650	C	T	exonic	ASPSCR1	63	4	0.0597	Missense_Mutation	p.S246L	Non-Targeted Panel Gene	Adult HGBCL, NOS
BCL-77	chr11	70820544	70820544	G	A	exonic	SHANK2	62	4	0.0606	Missense_Mutation	p.T438M	Non-Targeted Panel Gene	Adult HGBCL, NOS
BCL-77	chr14	90593531	90593531	G	A	exonic	TTC7B	31	2	0.0606	Missense_Mutation	p.P603S	Non-Targeted Panel Gene	Adult HGBCL, NOS

BCL-77	chr19	52439352	52439352	T	A	exonic	ZNF534	45	3	0.0625	Missense_Mutation	p.V644E	Non-Targeted Panel Gene	Adult HGBCL, NOS
BCL-77	chr1	231336335	231336335	C	A	exonic	EXOC8	44	3	0.0638	Missense_Mutation	p.A471S	Non-Targeted Panel Gene	Adult HGBCL, NOS
BCL-77	chr6	30704319	30704319	T	A	exonic	MDC1	85	6	0.0659	Missense_Mutation	p.T1622S	Non-Targeted Panel Gene	Adult HGBCL, NOS
BCL-77	chr11	5234064	5234064	T	G	exonic	HBD	28	2	0.0667	Missense_Mutation	p.N81T	Non-Targeted Panel Gene	Adult HGBCL, NOS
BCL-77	chr15	42081800	42081800	C	A	exonic	PLA2G4D	40	3	0.0682	Missense_Mutation	p.G273V	Non-Targeted Panel Gene	Adult HGBCL, NOS
BCL-77	chr16	67616872	67616872	T	A	exonic	CTCF	27	2	0.069	Missense_Mutation	p.S360R	Non-Targeted Panel Gene	Adult HGBCL, NOS
BCL-77	chr5	55792803	55792803	C	G	exonic	DDX4	27	2	0.069	Missense_Mutation	p.Q489E	Non-Targeted Panel Gene	Adult HGBCL, NOS
BCL-77	chr12	121444467	121444467	C	T	exonic	KDM2B	54	4	0.069	Missense_Mutation	p.A725T	Non-Targeted Panel Gene	Adult HGBCL, NOS
BCL-77	chr19	17187996	17187996	A	T	exonic	MYO9B	27	2	0.069	Missense_Mutation	p.E880V	Non-Targeted Panel Gene	Adult HGBCL, NOS
BCL-77	chr11	5632734	5632734	G	A	exonic	TRIM34;TRIM6-TRI	40	3	0.0698	Missense_Mutation	p.E489K	Non-Targeted Panel Gene	Adult HGBCL, NOS
BCL-77	chr1	113911494	113911494	G	A	exonic	DCLRE1B	53	4	0.0702	Missense_Mutation	p.R301Q	Non-Targeted Panel Gene	Adult HGBCL, NOS
BCL-77	chr5	140857327	140857327	C	T	exonic	PCDHA10	51	4	0.0727	Missense_Mutation	p.R427W	Non-Targeted Panel Gene	Adult HGBCL, NOS
BCL-77	chr5	149863176	149863176	C	T	exonic	PDE6A	50	4	0.0741	Missense_Mutation	p.D817N	Non-Targeted Panel Gene	Adult HGBCL, NOS
BCL-77	chr6	28300956	28300956	G	A	exonic	PGBD1	25	2	0.0741	Missense_Mutation	p.D368N	Non-Targeted Panel Gene	Adult HGBCL, NOS
BCL-77	chr11	13721750	13721750	G	A	exonic	FAR1	37	3	0.075	Missense_Mutation	p.R383H	Non-Targeted Panel Gene	Adult HGBCL, NOS
BCL-77	chr7	142161175	142161175	C	T	exonic	MGAM2	24	2	0.0769	Missense_Mutation	p.A799V	Non-Targeted Panel Gene	Adult HGBCL, NOS
BCL-77	chr17	62689968	62689968	G	T	exonic	MRC2	24	2	0.0769	Missense_Mutation	p.Q1216H	Non-Targeted Panel Gene	Adult HGBCL, NOS
BCL-77	chr4	177360535	177360535	G	A	exonic	NEIL3	36	3	0.0769	Missense_Mutation	p.R498H	Non-Targeted Panel Gene	Adult HGBCL, NOS
BCL-77	chr19	1506185	1506185	G	A	exonic	ADAMTSL5	35	3	0.0789	Missense_Mutation	p.P416S	Non-Targeted Panel Gene	Adult HGBCL, NOS
BCL-77	chr2	201536248	201536248	C	A	exonic	C2CD6	23	2	0.08	Missense_Mutation	p.A427S	Non-Targeted Panel Gene	Adult HGBCL, NOS
BCL-77	chr3	124773762	124773762	C	T	exonic	ITGB5	23	2	0.08	Missense_Mutation	p.C615Y	Non-Targeted Panel Gene	Adult HGBCL, NOS
BCL-77	chr17	18882649	18882649	C	A	exonic	PRPSAP2	34	3	0.0811	Missense_Mutation	p.A165E	Non-Targeted Panel Gene	Adult HGBCL, NOS
BCL-77	chr19	36237197	36237197	T	C	exonic	ZNF146	34	3	0.0811	Missense_Mutation	p.S253P	Non-Targeted Panel Gene	Adult HGBCL, NOS
BCL-77	chr17	77498604	77498604	C	A	exonic	SEPTIN9	22	2	0.0833	Missense_Mutation	p.S405R	Non-Targeted Panel Gene	Adult HGBCL, NOS
BCL-77	chr12	14436172	14436172	G	A	exonic	ATF7IP	42	4	0.087	Missense_Mutation	p.R579Q	Non-Targeted Panel Gene	Adult HGBCL, NOS
BCL-77	chr6	32214137	32214137	T	A	exonic	NOTCH4	21	2	0.087	Missense_Mutation	p.N714Y	Non-Targeted Panel Gene	Adult HGBCL, NOS
BCL-77	chr14	102039502	102039502	G	A	exonic	DYNC1H1	41	4	0.0889	Missense_Mutation	p.D3851N	Non-Targeted Panel Gene	Adult HGBCL, NOS
BCL-77	chr2	69520978	69520978	G	A	exonic	AAK1	20	2	0.0909	Missense_Mutation	p.P356S	Non-Targeted Panel Gene	Adult HGBCL, NOS
BCL-77	chr2	72575542	72575542	C	T	exonic	EXOC6B	20	2	0.0909	Missense_Mutation	p.E266K	Non-Targeted Panel Gene	Adult HGBCL, NOS
BCL-77	chr11	59504338	59504338	A	G	exonic	OR4D11	20	2	0.0909	Missense_Mutation	p.I255V	Non-Targeted Panel Gene	Adult HGBCL, NOS
BCL-77	chr17	4017717	4017717	C	T	exonic	ZZEF1	40	4	0.0909	Missense_Mutation	p.R2552H	Non-Targeted Panel Gene	Adult HGBCL, NOS
BCL-77	chrX	101398033	101398033	G	A	exonic	GLA	29	3	0.0938	Missense_Mutation	p.R356W	Non-Targeted Panel Gene	Adult HGBCL, NOS
BCL-77	chr7	138113714	138113714	G	A	exonic	AKR1D1	19	2	0.0952	Missense_Mutation	p.E294K	Non-Targeted Panel Gene	Adult HGBCL, NOS
BCL-77	chr19	10670974	10670974	C	T	exonic	ILF3	19	2	0.0952	Missense_Mutation	p.R2C	Non-Targeted Panel Gene	Adult HGBCL, NOS
BCL-77	chr10	77999985	77999985	A	G	exonic	POLR3A	19	2	0.0952	Missense_Mutation	p.M871T	Non-Targeted Panel Gene	Adult HGBCL, NOS
BCL-77	chr11	57738976	57738976	A	T	exonic	TMX2	19	2	0.0952	Missense_Mutation	p.Y184F	Non-Targeted Panel Gene	Adult HGBCL, NOS
BCL-77	chr11	120137512	120137512	C	T	exonic	TRIM29	38	4	0.0952	Missense_Mutation	p.D174N	Non-Targeted Panel Gene	Adult HGBCL, NOS
BCL-77	chr17	4177172	4177172	A	G	exonic	ANKFY1	28	3	0.0968	Missense_Mutation	p.L911P	Non-Targeted Panel Gene	Adult HGBCL, NOS
BCL-77	chr19	6467661	6467661	C	A	exonic	DENND1C	18	2	0.1	Missense_Mutation	p.R750L	Non-Targeted Panel Gene	Adult HGBCL, NOS
BCL-77	chr22	37650229	37650229	C	G	exonic	SH3BP1	18	2	0.1	Missense_Mutation	p.A465G	Non-Targeted Panel Gene	Adult HGBCL, NOS
BCL-77	chr5	171310318	171310318	C	T	exonic	TLX3	45	5	0.1	Missense_Mutation	p.A197V	Non-Targeted Panel Gene	Adult HGBCL, NOS
BCL-77	chr11	130470920	130470920	C	T	exonic	ADAMTS15	17	2	0.1053	Missense_Mutation	p.A574V	Non-Targeted Panel Gene	Adult HGBCL, NOS
BCL-77	chr13	102733470	102733470	C	A	exonic	CCDC168	17	2	0.1053	Missense_Mutation	p.V5743F	Non-Targeted Panel Gene	Adult HGBCL, NOS
BCL-77	chr15	29136493	29136493	T	A	exonic	FAM189A1	17	2	0.1053	Missense_Mutation	p.H267L	Non-Targeted Panel Gene	Adult HGBCL, NOS
BCL-77	chr4	2941584	2941584	G	T	exonic	NOP14	17	2	0.1053	Missense_Mutation	p.Q733K	Non-Targeted Panel Gene	Adult HGBCL, NOS
BCL-77	chr1	183232832	183232832	A	T	exonic	LAMC2	16	2	0.1111	Missense_Mutation	p.E732V	Non-Targeted Panel Gene	Adult HGBCL, NOS
BCL-77	chr7	131470904	131470904	A	T	exonic	MKLN1	16	2	0.1111	Missense_Mutation	p.Y641F	Non-Targeted Panel Gene	Adult HGBCL, NOS
BCL-77	chr10	122428282	122428282	C	A	exonic	PLEKHA1	16	2	0.1111	Missense_Mutation	p.Q303K	Non-Targeted Panel Gene	Adult HGBCL, NOS
BCL-77	chr7	138801338	138801338	A	T	exonic	TMEM213	24	3	0.1111	Missense_Mutation	p.S32C	Non-Targeted Panel Gene	Adult HGBCL, NOS
BCL-77	chr1	26029659	26029659	C	G	exonic	EXTL1	14	2	0.1176	Missense_Mutation	p.I311M	Non-Targeted Panel Gene	Adult HGBCL, NOS
BCL-77	chrX	133324192	133324192	C	T	exonic	GPC4	15	2	0.1176	Missense_Mutation	p.A222T	Non-Targeted Panel Gene	Adult HGBCL, NOS
BCL-77	chr1	39743842	39743842	G	T	exonic	PPIE	15	2	0.1176	Missense_Mutation	p.W101L	Non-Targeted Panel Gene	Adult HGBCL, NOS
BCL-77	chr16	21980632	21980632	G	A	exonic	UQCRC2	15	2	0.1176	Missense_Mutation	p.G404S	Non-Targeted Panel Gene	Adult HGBCL, NOS
BCL-77	chrX	153420330	153420330	T	A	exonic	ZFP92	15	2	0.1176	Missense_Mutation	p.I88K	Non-Targeted Panel Gene	Adult HGBCL, NOS
BCL-77	chr6	136366381	136366381	C	A	exonic	MAP7	22	3	0.12	Missense_Mutation	p.R334M	Non-Targeted Panel Gene	Adult HGBCL, NOS
BCL-77	chr1	93226346	93226346	A	T	exonic	CCDC18	14	2	0.125	Missense_Mutation	p.D730V	Non-Targeted Panel Gene	Adult HGBCL, NOS
BCL-77	chr12	96704743	96704743	A	T	exonic	CFAP54	14	2	0.125	Missense_Mutation	p.I2159F	Non-Targeted Panel Gene	Adult HGBCL, NOS
BCL-77	chr3	124773733	124773733	A	T	exonic	ITGB5	14	2	0.125	Missense_Mutation	p.C625S	Non-Targeted Panel Gene	Adult HGBCL, NOS
BCL-77	chr20	5972677	5972677	G	C	exonic	MCM8	28	4	0.125	Missense_Mutation	p.C442S	Non-Targeted Panel Gene	Adult HGBCL, NOS
BCL-77	chr19	40075519	40075519	T	C	exonic	ZNF780A	28	4	0.125	Missense_Mutation	p.K308R	Non-Targeted Panel Gene	Adult HGBCL, NOS
BCL-77	chr8	81525113	81525113	G	A	exonic	FABP12	13	2	0.1333	Missense_Mutation	p.T119I	Non-Targeted Panel Gene	Adult HGBCL, NOS
BCL-77	chr19	8946636	8946636	G	A	exonic	MUC16	24	4	0.1429	Missense_Mutation	p.S10045F	Non-Targeted Panel Gene	Adult HGBCL, NOS
BCL-77	chr19	17188010	17188010	C	T	exonic	MYO9B	24	4	0.1429	Missense_Mutation	p.R885W	Non-Targeted Panel Gene	Adult HGBCL, NOS
BCL-77	chr19	6710749	6710749	T	A	exonic	C3	16	3	0.1579	Missense_Mutation	p.I526F	Non-Targeted Panel Gene	Adult HGBCL, NOS
BCL-77	chr8	38952649	38952649	A	T	exonic	PLEKHA2	10	2	0.1667	Missense_Mutation	p.K216I	Non-Targeted Panel Gene	Adult HGBCL, NOS
BCL-77	chr1	220182300	220182300	C	A	exonic	RAB3GAP2	10	2	0.1667	Missense_Mutation	p.C756F	Non-Targeted Panel Gene	Adult HGBCL, NOS
BCL-77	chr11	16049912	16049912	A	T	exonic	SOX6	14	3	0.1765	Missense_Mutation	p.N426K	Non-Targeted Panel Gene	Adult HGBCL, NOS
BCL-77	chr12	122249877	122249877	G	T	exonic	VPS33A	13	3	0.1875	Missense_Mutation	p.Q257K	Non-Targeted Panel Gene	Adult HGBCL, NOS
BCL-77	chr20	16379114	16379114	G	A	exonic	KIF16B	68	16	0.1905	Missense_Mutation	p.A963V	Non-Targeted Panel Gene	Adult HGBCL, NOS
BCL-77	chrX	12710487	12710487	G	T	exonic	FRMPD4	8	2	0.2	Missense_Mutation	p.R520M	Non-Targeted Panel Gene	Adult HGBCL, NOS
BCL-77	chr22	37937106	37937106	G	T	exonic	MICALL1	11	3	0.2143	Missense_Mutation	p.A779S	Non-Targeted Panel Gene	Adult HGBCL, NOS
BCL-77	chr12	84872640	84872640	G	A	exonic	SLC6A15	7	2	0.2222	Missense_Mutation	p.H422Y	Non-Targeted Panel Gene	Adult HGBCL, NOS
BCL-77	chr20	56452773	56452773	A	G	exonic	CASS4	20	6	0.2308	Missense_Mutation	p.T533A	Non-Targeted Panel Gene	Adult HGBCL, NOS
BCL-77	chr1	152410423	152410423	G	C	exonic	CRNN	42	15	0.2632	Missense_Mutation	p.A220G	Non-Targeted Panel Gene	Adult HGBCL, NOS
BCL-77	chr13	37009732	37009732	A	T	exonic	SUPT20H	18	7	0.28	Missense_Mutation	p.H760Q	Non-Targeted Panel Gene	Adult HGBCL, NOS
BCL-77	chr12	51821609	51821609	C	T	exonic	FIGNL2	32	13	0.2889	Missense_Mutation	p.A269T	Non-Targeted Panel Gene	Adult HGBCL, NOS
BCL-77	chr6	111373959	111373959	G	A	exonic	REV3L	21	9	0.3	Missense_Mutation	p.P1388S	Non-Targeted Panel Gene	Adult HGBCL, NOS
BCL-77	chr18	80037457	80037457	C	T	exonic	RBFA	21	10	0.3226	Missense_Mutation	p.T110I	Non-Targeted Panel Gene	Adult HGBCL, NOS
BCL-77	chr11	124987496	124987496	C	G	exonic	CCDC15	21	12	0.3636	Missense_Mutation	p.Q424E	Non-Targeted Panel Gene	Adult HGBCL, NOS
BCL-77	chr2	29071798	29071798	C	T	exonic	C2orf71	33	19	0.3654	Missense_Mutation	p.E822K	Non-Targeted Panel Gene	Adult HGBCL, NOS
BCL-77	chr12	14770988	14770988	G	A	exonic	HIST4H4	62	36	0.3673	Missense_Mutation	p.P33S	Non-Targeted Panel Gene	Adult HGBCL, NOS
BCL-77	chr2	98726291	98726291	A	C	exonic	MGAT4A	24	14	0.3684	Missense_Mutation	p.F14L	Non-Targeted Panel Gene	Adult HGBCL, NOS

BCL-77	chr3	2902882	2902882	A	G	exonic	CNTN4	22	13	0.3714	Missense_Mutation	p.I362V	Non-Targeted Panel Gene	Adult HGBCL, NOS
BCL-77	chr12	8534809	8534809	G	T	exonic	CLEC4E	20	12	0.375	Missense_Mutation	p.S163R	Non-Targeted Panel Gene	Adult HGBCL, NOS
BCL-77	chr9	107487090	107487090	G	C	exonic	KLF4	45	28	0.3784	Missense_Mutation	p.A401G	Non-Targeted Panel Gene	Adult HGBCL, NOS
BCL-77	chr11	18415260	18415260	G	C	exonic	LDHC	23	15	0.3947	Missense_Mutation	p.G68A	Non-Targeted Panel Gene	Adult HGBCL, NOS
BCL-77	chr1	31059275	31059275	T	C	exonic	PUM1	29	19	0.3958	Missense_Mutation	p.S98G	Non-Targeted Panel Gene	Adult HGBCL, NOS
BCL-77	chr17	17477216	17477216	G	A	exonic	MED9	24	16	0.4	Missense_Mutation	p.E59K	Non-Targeted Panel Gene	Adult HGBCL, NOS
BCL-77	chr2	134986725	134986725	A	C	exonic	MAP3K19	16	11	0.4074	Missense_Mutation	p.L716R	Non-Targeted Panel Gene	Adult HGBCL, NOS
BCL-77	chr19	54158308	54158308	A	T	exonic	LENG1	45	31	0.4079	Missense_Mutation	p.Y96N	Non-Targeted Panel Gene	Adult HGBCL, NOS
BCL-77	chr19	48715280	48715280	G	A	exonic	MAMSTR	23	16	0.4103	Missense_Mutation	p.S136L	Non-Targeted Panel Gene	Adult HGBCL, NOS
BCL-77	chr2	77518334	77518334	C	A	exonic	LRRTM4	27	19	0.413	Missense_Mutation	p.G512V	Non-Targeted Panel Gene	Adult HGBCL, NOS
BCL-77	chr16	1220767	1220767	G	A	exonic	CACNA1H	39	30	0.4348	Missense_Mutation	p.D2279N	Non-Targeted Panel Gene	Adult HGBCL, NOS
BCL-77	chr2	11160474	11160474	T	A	exonic	PQLC3	25	20	0.4348	Missense_Mutation	p.V51E	Non-Targeted Panel Gene	Adult HGBCL, NOS
BCL-77	chr2	177392998	177392998	C	G	exonic	AGPS	55	43	0.4388	Missense_Mutation	p.A70G	Non-Targeted Panel Gene	Adult HGBCL, NOS
BCL-77	chr7	66286533	66286533	G	A	exonic	TPST1	20	16	0.4444	Missense_Mutation	p.V290I	Non-Targeted Panel Gene	Adult HGBCL, NOS
BCL-77	chr19	7125507	7125507	C	T	exonic	INSR	30	25	0.4545	Missense_Mutation	p.V1012M	Non-Targeted Panel Gene	Adult HGBCL, NOS
BCL-77	chr12	8092987	8092987	C	T	exonic	NECAP1	18	15	0.4545	Missense_Mutation	p.S203L	Non-Targeted Panel Gene	Adult HGBCL, NOS
BCL-77	chr8	143021718	143021718	G	C	exonic	LY6E	35	30	0.4615	Missense_Mutation	p.A109P	Non-Targeted Panel Gene	Adult HGBCL, NOS
BCL-77	chr17	2299803	2299803	G	C	exonic	SMG6	29	25	0.463	Missense_Mutation	p.P317R	Non-Targeted Panel Gene	Adult HGBCL, NOS
BCL-77	chr18	21499735	21499735	T	A	exonic	GREB1L	17	15	0.4688	Missense_Mutation	p.I1133N	Non-Targeted Panel Gene	Adult HGBCL, NOS
BCL-77	chr6	1610690	1610690	G	A	exonic	FOXC1	31	28	0.4746	Missense_Mutation	p.S82N	Non-Targeted Panel Gene	Adult HGBCL, NOS
BCL-77	chr2	189039508	189039508	G	C	exonic	COL5A2	12	11	0.4783	Missense_Mutation	p.T1230R	Non-Targeted Panel Gene	Adult HGBCL, NOS
BCL-77	chr14	74551197	74551197	C	A	exonic	LTBP2	25	23	0.4792	Missense_Mutation	p.S518I	Non-Targeted Panel Gene	Adult HGBCL, NOS
BCL-77	chr8	143021705	143021705	T	G	exonic	LY6E	36	34	0.4857	Missense_Mutation	p.D104E	Non-Targeted Panel Gene	Adult HGBCL, NOS
BCL-77	chr9	122567831	122567831	G	A	exonic	OR1L8	4	4	0.5	Missense_Mutation	p.A216V	Non-Targeted Panel Gene	Adult HGBCL, NOS
BCL-77	chr9	128579718	128579718	T	G	exonic	SPTAN1	5	5	0.5	Missense_Mutation	p.S435A	Non-Targeted Panel Gene	Adult HGBCL, NOS
BCL-77	chr5	39331792	39331792	G	A	exonic	C9	19	20	0.5128	Missense_Mutation	p.P167S	Non-Targeted Panel Gene	Adult HGBCL, NOS
BCL-77	chr5	1271177	1271177	C	T	exonic	TERT	17	18	0.5143	Missense_Mutation	p.G804S	Non-Targeted Panel Gene	Adult HGBCL, NOS
BCL-77	chr10	73798442	73798442	T	C	exonic	ZSWIM8	12	13	0.52	Missense_Mutation	p.C1389R	Non-Targeted Panel Gene	Adult HGBCL, NOS
BCL-77	chr11	126271475	126271475	A	C	exonic	FOXRED1	11	12	0.5217	Missense_Mutation	p.K42Q	Non-Targeted Panel Gene	Adult HGBCL, NOS
BCL-77	chr10	94852858	94852858	G	T	exonic	CYP2C19	29	32	0.5246	Missense_Mutation	p.V473F	Non-Targeted Panel Gene	Adult HGBCL, NOS
BCL-77	chr4	158723239	158723239	C	A	exonic	PPID	33	38	0.5352	Missense_Mutation	p.R17L	Non-Targeted Panel Gene	Adult HGBCL, NOS
BCL-77	chr1	171987713	171987713	G	A	exonic	DNM3	16	20	0.5556	Missense_Mutation	p.R98H	Non-Targeted Panel Gene	Adult HGBCL, NOS
BCL-77	chr4	2829931	2829931	G	A	exonic	SH3BP2	28	35	0.5556	Missense_Mutation	p.R342Q	Non-Targeted Panel Gene	Adult HGBCL, NOS
BCL-77	chr1	233382501	233382501	G	C	exonic	MAP3K21	20	29	0.5918	Missense_Mutation	p.Q967H	Non-Targeted Panel Gene	Adult HGBCL, NOS
BCL-77	chr3	12813011	12813011	T	C	exonic	CAND2	13	20	0.6061	Missense_Mutation	p.V260A	Non-Targeted Panel Gene	Adult HGBCL, NOS
BCL-77	chr3	53092439	53092439	G	A	exonic	RFT1	19	32	0.6275	Missense_Mutation	p.A463V	Non-Targeted Panel Gene	Adult HGBCL, NOS
BCL-77	chr5	141658652	141658652	A	C	exonic	ARAP3	13	23	0.6389	Missense_Mutation	p.L1113R	Non-Targeted Panel Gene	Adult HGBCL, NOS
BCL-77	chr15	81303576	81303576	T	G	exonic	IL16	6	11	0.6471	Missense_Mutation	p.L1116V	Non-Targeted Panel Gene	Adult HGBCL, NOS
BCL-77	chr2	219567085	219567085	C	T	exonic	OBSL1	4	8	0.6667	Missense_Mutation	p.V627I	Non-Targeted Panel Gene	Adult HGBCL, NOS
BCL-77	chr7	14736089	14736089	G	A	exonic	DGKB	4	9	0.6923	Missense_Mutation	p.P50S	Non-Targeted Panel Gene	Adult HGBCL, NOS
BCL-77	chr7	21818315	21818315	G	T	exonic	DNAH11	4	10	0.7143	Missense_Mutation	p.G3556V	Non-Targeted Panel Gene	Adult HGBCL, NOS
BCL-77	chr2	219421415	219421415	A	G	exonic	DES	2	10	0.8333	Missense_Mutation	p.I367V	Non-Targeted Panel Gene	Adult HGBCL, NOS
BCL-77	chr15	65497864	65497864	C	T	exonic	DPP8	1	14	0.9333	Missense_Mutation	p.E255K	Non-Targeted Panel Gene	Adult HGBCL, NOS
BCL-77	chrX	154031241	154031241	G	C	exonic	MECP2	0	70	1	Missense_Mutation	p.T103S	Non-Targeted Panel Gene	Adult HGBCL, NOS
BCL-77	chr3	109114415	109114415	C	A	exonic	MORC1	53	2	0.0364	Nonsense_Mutation	p.G30X	Non-Targeted Panel Gene	Adult HGBCL, NOS
BCL-77	chr11	56490452	56490452	T	A	exonic	OR5M8	51	2	0.0377	Nonsense_Mutation	p.K307X	Non-Targeted Panel Gene	Adult HGBCL, NOS
BCL-77	chr12	6390883	6390883	T	A	exonic	LTBR	41	2	0.0465	Nonsense_Mutation	p.C418X	Non-Targeted Panel Gene	Adult HGBCL, NOS
BCL-77	chr3	94043077	94043077	C	A	exonic	ARL13B	37	2	0.0513	Nonsense_Mutation	p.C272X	Non-Targeted Panel Gene	Adult HGBCL, NOS
BCL-77	chr5	75029904	75029904	A	T	exonic	GCNT4	24	2	0.0769	Nonsense_Mutation	p.L45X	Non-Targeted Panel Gene	Adult HGBCL, NOS
BCL-77	chr3	154123073	154123073	A	T	exonic	ARHGEF26	23	2	0.08	Nonsense_Mutation	p.K361X	Non-Targeted Panel Gene	Adult HGBCL, NOS
BCL-77	chr12	14639948	14639948	T	A	exonic	GUCY2C	17	2	0.1053	Nonsense_Mutation	p.K691X	Non-Targeted Panel Gene	Adult HGBCL, NOS
BCL-77	chr15	23567019	23567019	G	T	exonic	MKRN3	17	2	0.1053	Nonsense_Mutation	p.G413X	Non-Targeted Panel Gene	Adult HGBCL, NOS
BCL-77	chr9	127727421	127727421	G	T	exonic	TTC16	17	2	0.1053	Nonsense_Mutation	p.E574X	Non-Targeted Panel Gene	Adult HGBCL, NOS
BCL-77	chr1	19114031	19114031	G	A	exonic	UBR4	25	3	0.1071	Nonsense_Mutation	p.R3748X	Non-Targeted Panel Gene	Adult HGBCL, NOS
BCL-77	chr8	68468701	68468701	C	A	exonic	C8orf34	16	2	0.1111	Nonsense_Mutation	p.S206X	Non-Targeted Panel Gene	Adult HGBCL, NOS
BCL-77	chr17	40882588	40882588	C	A	exonic	KRT20	16	2	0.1111	Nonsense_Mutation	p.E153X	Non-Targeted Panel Gene	Adult HGBCL, NOS
BCL-77	chr9	133647991	133647991	C	A	exonic	DBH	15	2	0.1176	Nonsense_Mutation	p.C390X	Non-Targeted Panel Gene	Adult HGBCL, NOS
BCL-77	chr17	6118178	6118178	G	A	exonic	WSCD1	15	2	0.1176	Nonsense_Mutation	p.W455X	Non-Targeted Panel Gene	Adult HGBCL, NOS
BCL-77	chrX	110450642	110450642	C	T	exonic	RTL9	12	2	0.1429	Nonsense_Mutation	p.R9X	Non-Targeted Panel Gene	Adult HGBCL, NOS
BCL-77	chr19	56664836	56664836	G	T	exonic	ZNF835	48	33	0.4074	Nonsense_Mutation	p.Y121X	Non-Targeted Panel Gene	Adult HGBCL, NOS
BCL-77	chr9	77815650	77815650	G	A	exonic	GNAQ	2	9	0.8182	Nonsense_Mutation	p.R148X	Non-Targeted Panel Gene	Adult HGBCL, NOS
BCL-77	chr12	53158513	53158513	A	T	exonic	CSAD	35	2	0.0541	Nonstop_Mutation	p.X521R	Non-Targeted Panel Gene	Adult HGBCL, NOS
BCL-78	chr15	42421990	42421990	T	A	splicing	ZNF106	51	23	0.3108	Splice_Site	.	Non-Targeted Panel Gene	Adult HGBCL, NOS
BCL-78	chr11	55651615	55651633	TGTGGCTCCCACAT	-	exonic	OR4S2	80	26	0.2453	Frame_Shift_Del	p.C238fs	Non-Targeted Panel Gene	Adult HGBCL, NOS
BCL-78	chr12	55365806	55365806	-	A	exonic	OR6C75	106	33	0.234	Frame_Shift_Ins	p.R232fs	Non-Targeted Panel Gene	Adult HGBCL, NOS
BCL-78	chr4	139045139	139045139	-	TT	exonic	NOCT	69	44	0.3826	Frame_Shift_Ins	p.V321fs	Non-Targeted Panel Gene	Adult HGBCL, NOS
BCL-78	chr19	45677363	45677368	GTACCT	-	exonic	GIPR	31	10	0.2439	In_Frame_Del	p.278_280del	Non-Targeted Panel Gene	Adult HGBCL, NOS
BCL-78	chr3	177038316	177038316	A	T	exonic	TBL1XR1	82	19	0.1881	Missense_Mutation	p.H348Q	Targerted Panel Gene	Adult HGBCL, NOS
BCL-78	chr18	63318657	63318657	C	T	exonic	BCL2	75	30	0.2857	Missense_Mutation	p.A4T	Targerted Panel Gene	Adult HGBCL, NOS
BCL-78	chr1	26780272	26780272	T	G	exonic	ARID1A	143	68	0.3223	Missense_Mutation	p.L1908R	Targerted Panel Gene	Adult HGBCL, NOS
BCL-78	chr4	125319238	125319238	A	G	exonic	FAT4	65	40	0.381	Missense_Mutation	p.N943D	Targerted Panel Gene	Adult HGBCL, NOS
BCL-78	chr12	85046040	85046040	A	C	exonic	LRRIQ1	21	13	0.3824	Missense_Mutation	p.K119N	Targerted Panel Gene	Adult HGBCL, NOS
BCL-78	chr16	11255472	11255472	C	T	exonic	SOCS1	33	22	0.4	Missense_Mutation	p.A3T	Targerted Panel Gene	Adult HGBCL, NOS
BCL-78	chrX	101353876	101353876	C	T	exonic	BTK	19	24	0.5581	Missense_Mutation	p.A616T	Targerted Panel Gene	Adult HGBCL, NOS
BCL-78	chr17	65014661	65014661	G	A	exonic	GNA13	22	70	0.7609	Missense_Mutation	p.L244F	Targerted Panel Gene	Adult HGBCL, NOS
BCL-78	chr22	50459566	50459566	G	T	exonic	SBF1	93	4	0.0412	Missense_Mutation	p.R1198S	Non-Targeted Panel Gene	Adult HGBCL, NOS
BCL-78	chr22	40463272	40463272	G	T	exonic	MKL1	58	3	0.0492	Missense_Mutation	p.L21I	Non-Targeted Panel Gene	Adult HGBCL, NOS
BCL-78	chr5	169601675	169601675	C	A	exonic	SPDL1	54	3	0.0526	Missense_Mutation	p.L503I	Non-Targeted Panel Gene	Adult HGBCL, NOS
BCL-78	chr11	1167877	1167877	C	A	exonic	MUC5AC	50	3	0.0566	Missense_Mutation	p.P463T	Non-Targeted Panel Gene	Adult HGBCL, NOS
BCL-78	chr19	47702276	47702276	C	A	exonic	BICRA	66	4	0.0571	Missense_Mutation	p.P1515H	Non-Targeted Panel Gene	Adult HGBCL, NOS
BCL-78	chr1	59990785	59990785	A	C	exonic	C1orf87	100	12	0.1071	Missense_Mutation	p.L510R	Non-Targeted Panel Gene	Adult HGBCL, NOS
BCL-78	chr2	43947310	43947310	G	T	exonic	LRPPRC	13	2	0.1333	Missense_Mutation	p.Q676K	Non-Targeted Panel Gene	Adult HGBCL, NOS

BCL-78	chr5	91150099	91150099	A	T	exonic	ADGRV1	38	7	0.1556	Missense_Mutation	p.T6168S	Non-Targeted Panel Gene	Adult HGBCL, NOS
BCL-78	chr13	37655127	37655127	C	A	exonic	TRPC4	88	24	0.2143	Missense_Mutation	p.M615I	Non-Targeted Panel Gene	Adult HGBCL, NOS
BCL-78	chr12	49999221	49999221	C	T	exonic	RACGAP1	124	36	0.225	Missense_Mutation	p.E267K	Non-Targeted Panel Gene	Adult HGBCL, NOS
BCL-78	chr7	98619664	98619664	T	A	exonic	NPTX2	98	29	0.2283	Missense_Mutation	p.S150T	Non-Targeted Panel Gene	Adult HGBCL, NOS
BCL-78	chr21	44525667	44525667	G	T	exonic	TSPEAR	118	35	0.2288	Missense_Mutation	p.A373D	Non-Targeted Panel Gene	Adult HGBCL, NOS
BCL-78	chr9	15177750	15177750	C	G	exonic	TTC39B	58	18	0.2368	Missense_Mutation	p.K596N	Non-Targeted Panel Gene	Adult HGBCL, NOS
BCL-78	chr7	89327455	89327455	T	A	exonic	ZNF804B	122	38	0.2375	Missense_Mutation	p.L121I	Non-Targeted Panel Gene	Adult HGBCL, NOS
BCL-78	chr2	187383299	187383299	C	A	exonic	CALCRL	42	14	0.25	Missense_Mutation	p.V20F	Non-Targeted Panel Gene	Adult HGBCL, NOS
BCL-78	chr7	18027124	18027124	T	C	exonic	PRPS1L1	172	61	0.2607	Missense_Mutation	p.D220G	Non-Targeted Panel Gene	Adult HGBCL, NOS
BCL-78	chr9	87887482	87887482	G	A	exonic	SPATA31E1	107	42	0.28	Missense_Mutation	p.A999T	Non-Targeted Panel Gene	Adult HGBCL, NOS
BCL-78	chr11	66492797	66492797	T	C	exonic	DPP3	118	47	0.2848	Missense_Mutation	p.L357P	Non-Targeted Panel Gene	Adult HGBCL, NOS
BCL-78	chr8	143295829	143295829	G	T	exonic	ZNF696	143	57	0.285	Missense_Mutation	p.A52S	Non-Targeted Panel Gene	Adult HGBCL, NOS
BCL-78	chr11	124869536	124869536	G	A	exonic	ROBO3	49	20	0.2899	Missense_Mutation	p.G192S	Non-Targeted Panel Gene	Adult HGBCL, NOS
BCL-78	chr13	52739096	52739096	C	T	exonic	CNMD	81	34	0.2957	Missense_Mutation	p.G50R	Non-Targeted Panel Gene	Adult HGBCL, NOS
BCL-78	chr2	162400267	162400267	C	T	exonic	KCNH7	56	24	0.3	Missense_Mutation	p.D777N	Non-Targeted Panel Gene	Adult HGBCL, NOS
BCL-78	chr2	39067688	39067688	T	G	exonic	SOS1	42	18	0.3	Missense_Mutation	p.L51F	Non-Targeted Panel Gene	Adult HGBCL, NOS
BCL-78	chr2	210647987	210647987	G	A	exonic	CPS1	77	35	0.3125	Missense_Mutation	p.R1095H	Non-Targeted Panel Gene	Adult HGBCL, NOS
BCL-78	chr2	162400272	162400272	C	G	exonic	KCNH7	55	25	0.3125	Missense_Mutation	p.C775S	Non-Targeted Panel Gene	Adult HGBCL, NOS
BCL-78	chr17	3292083	3292083	G	C	exonic	OR3A1	185	85	0.3125	Missense_Mutation	p.S167C	Non-Targeted Panel Gene	Adult HGBCL, NOS
BCL-78	chr10	117545698	117545698	G	A	exonic	EMX2	79	36	0.313	Missense_Mutation	p.R158Q	Non-Targeted Panel Gene	Adult HGBCL, NOS
BCL-78	chr17	30923039	30923039	G	A	exonic	ADAP2	57	28	0.3294	Missense_Mutation	p.R65Q	Non-Targeted Panel Gene	Adult HGBCL, NOS
BCL-78	chr4	3493103	3493103	C	T	exonic	DOK7	120	60	0.3333	Missense_Mutation	p.L373F	Non-Targeted Panel Gene	Adult HGBCL, NOS
BCL-78	chr1	2336847	2336847	C	A	exonic	MORN1	60	30	0.3333	Missense_Mutation	p.R347M	Non-Targeted Panel Gene	Adult HGBCL, NOS
BCL-78	chr3	183736812	183736812	A	C	exonic	YEATS2	62	31	0.3333	Missense_Mutation	p.I303L	Non-Targeted Panel Gene	Adult HGBCL, NOS
BCL-78	chr14	105528936	105528936	G	C	exonic	TMEM121	87	44	0.3359	Missense_Mutation	p.Q34H	Non-Targeted Panel Gene	Adult HGBCL, NOS
BCL-78	chr1	37761612	37761612	C	T	exonic	EPHA10	60	31	0.3407	Missense_Mutation	p.V215M	Non-Targeted Panel Gene	Adult HGBCL, NOS
BCL-78	chr5	82318091	82318091	T	C	exonic	ATP6AP1L	92	48	0.3429	Missense_Mutation	p.S156P	Non-Targeted Panel Gene	Adult HGBCL, NOS
BCL-78	chr10	44373335	44373335	T	C	exonic	CXCL12	59	31	0.3444	Missense_Mutation	p.K92R	Non-Targeted Panel Gene	Adult HGBCL, NOS
BCL-78	chr5	159097067	159097067	G	C	exonic	EBF1	68	37	0.3524	Missense_Mutation	p.N66K	Non-Targeted Panel Gene	Adult HGBCL, NOS
BCL-78	chr12	5044593	5044593	C	T	exonic	KCNA5	117	64	0.3536	Missense_Mutation	p.P149L	Non-Targeted Panel Gene	Adult HGBCL, NOS
BCL-78	chr2	166307037	166307037	C	T	exonic	SCN9A	18	10	0.3571	Missense_Mutation	p.R99H	Non-Targeted Panel Gene	Adult HGBCL, NOS
BCL-78	chr10	61902341	61902341	A	C	exonic	ARID5B	89	50	0.3597	Missense_Mutation	p.Q68H	Non-Targeted Panel Gene	Adult HGBCL, NOS
BCL-78	chr17	2420559	2420559	A	G	exonic	METTL16	91	55	0.3767	Missense_Mutation	p.V367A	Non-Targeted Panel Gene	Adult HGBCL, NOS
BCL-78	chr10	117375081	117375081	C	G	exonic	PDZD8	66	41	0.3832	Missense_Mutation	p.K49N	Non-Targeted Panel Gene	Adult HGBCL, NOS
BCL-78	chr19	16325403	16325403	C	T	exonic	KLF2	66	42	0.3889	Missense_Mutation	p.A88V	Non-Targeted Panel Gene	Adult HGBCL, NOS
BCL-78	chr1	1806476	1806476	T	G	exonic	GNB1	23	15	0.3947	Missense_Mutation	p.K89T	Non-Targeted Panel Gene	Adult HGBCL, NOS
BCL-78	chr5	138145190	138145190	C	T	exonic	BRD8	55	36	0.3956	Missense_Mutation	p.D1142N	Non-Targeted Panel Gene	Adult HGBCL, NOS
BCL-78	chr10	101550747	101550747	G	T	exonic	BTRC	64	43	0.4019	Missense_Mutation	p.V569F	Non-Targeted Panel Gene	Adult HGBCL, NOS
BCL-78	chr2	85542155	85542155	G	A	exonic	MAT2A	41	30	0.4225	Missense_Mutation	p.V184I	Non-Targeted Panel Gene	Adult HGBCL, NOS
BCL-78	chr11	88335010	88335010	C	T	exonic	CTSC	21	17	0.4474	Missense_Mutation	p.G82D	Non-Targeted Panel Gene	Adult HGBCL, NOS
BCL-78	chr7	74060588	74060588	T	C	exonic	ELN	88	94	0.5165	Missense_Mutation	p.S641P	Non-Targeted Panel Gene	Adult HGBCL, NOS
BCL-78	chrX	154449895	154449895	T	G	exonic	FAM50A	12	28	0.7	Missense_Mutation	p.Y265D	Non-Targeted Panel Gene	Adult HGBCL, NOS
BCL-78	chrX	21609311	21609311	G	A	exonic	CNKSR2	17	43	0.7167	Missense_Mutation	p.D796N	Non-Targeted Panel Gene	Adult HGBCL, NOS
BCL-78	chrX	153782783	153782783	G	A	exonic	SRPK3	15	39	0.7222	Missense_Mutation	p.V163M	Non-Targeted Panel Gene	Adult HGBCL, NOS
BCL-78	chr2	228019965	228019965	G	A	exonic	SPHKAP	94	69	0.4233	Nonsense_Mutation	p.Q297X	Targerted Panel Gene	Adult HGBCL, NOS
BCL-78	chr12	49033487	49033487	G	A	exonic	KMT2D	100	101	0.5	Nonsense_Mutation	p.Q3740X	Targerted Panel Gene	Adult HGBCL, NOS
BCL-79	chr8	59118885	59118885	C	T	splicing	TOX	37	12	0.2449	Splice_Site	.	Non-Targeted Panel Gene	Adult HGBCL, NOS
BCL-79	chr20	33359124	33359124	C	A	splicing	CDK5RAP1	17	6	0.2609	Splice_Site	.	Non-Targeted Panel Gene	Adult HGBCL, NOS
BCL-79	chr1	66905376	66905376	C	T	splicing	WDR78	47	18	0.2769	Splice_Site	.	Non-Targeted Panel Gene	Adult HGBCL, NOS
BCL-79	chr15	101313507	101313507	T	C	splicing	PCSK6	24	16	0.4	Splice_Site	.	Non-Targeted Panel Gene	Adult HGBCL, NOS
BCL-79	chr15	42695827	42695828	GG	-	exonic	STARD9	50	2	0.0385	Frame_Shift_Del	p.G4411fs	Targerted Panel Gene	Adult HGBCL, NOS
BCL-79	chr7	98967096	98967096	A	-	exonic	TRRAP	48	3	0.0588	Frame_Shift_Del	p.E2404fs	Targerted Panel Gene	Adult HGBCL, NOS
BCL-79	chr7	100823646	100823647	TG	-	exonic	EPHB4	58	2	0.0333	Frame_Shift_Del	p.I136fs	Non-Targeted Panel Gene	Adult HGBCL, NOS
BCL-79	chr8	16164140	16164143	TCAG	-	exonic	MSR1	50	2	0.0385	Frame_Shift_Del	p.L247fs	Non-Targeted Panel Gene	Adult HGBCL, NOS
BCL-79	chr3	101859367	101859375	ATTAGCTCC	-	exonic	NFKBIZ	37	11	0.2292	Frame_Shift_Del	p.Y618fs	Non-Targeted Panel Gene	Adult HGBCL, NOS
BCL-79	chr2	1493955	1493955	A	-	exonic	TPO	52	19	0.2676	Frame_Shift_Del	p.E641fs	Non-Targeted Panel Gene	Adult HGBCL, NOS
BCL-79	chr18	32770156	32770169	TGGCCGTGTAGAG	-	exonic	KLHL14	128	63	0.3298	Frame_Shift_Del	p.Y141fs	Non-Targeted Panel Gene	Adult HGBCL, NOS
BCL-79	chr8	59118891	59118895	TGCAA	-	exonic	TOX	31	18	0.3673	Frame_Shift_Del	p.Y31fs	Non-Targeted Panel Gene	Adult HGBCL, NOS
BCL-79	chr18	31022493	31022493	A	-	exonic	DSC3	24	18	0.4286	Frame_Shift_Del	p.V262fs	Non-Targeted Panel Gene	Adult HGBCL, NOS
BCL-79	chr1	156051192	156051192	-	C	exonic	UBQLN4	152	2	0.013	Frame_Shift_Ins	p.G132fs	Non-Targeted Panel Gene	Adult HGBCL, NOS
BCL-79	chr20	63319488	63319488	-	A	exonic	COL20A1	48	4	0.0769	Frame_Shift_Ins	p.A936fs	Non-Targeted Panel Gene	Adult HGBCL, NOS
BCL-79	chr6	31271234	31271234	-	CCTCG	exonic	HLA-C	147	32	0.1788	Frame_Shift_Ins	p.D153fs	Non-Targeted Panel Gene	Adult HGBCL, NOS
BCL-79	chr19	41880861	41880905	TCCCAGGGCCTGA	-	exonic	CD79A	70	5	0.0667	In_Frame_Del	p.152_164del	Targerted Panel Gene	Adult HGBCL, NOS
BCL-79	chr1	234607430	234607450	CAGGGTGCACTGG	-	exonic	IRF2BP2	86	26	0.2321	In_Frame_Del	p.484_491del	Non-Targeted Panel Gene	Adult HGBCL, NOS
BCL-79	chr4	13615332	13615346	TCTGGTTTCTCATC	-	exonic	BOD1L1	24	8	0.25	In_Frame_Del	p.175_180del	Non-Targeted Panel Gene	Adult HGBCL, NOS
BCL-79	chr5	179345252	179345252	-	GCAGGA	exonic	ADAMTS2	152	15	0.0898	In_Frame_Ins	p.P26delinsLLP	Non-Targeted Panel Gene	Adult HGBCL, NOS
BCL-79	chr15	42695836	42695836	A	T	exonic	STARD9	47	2	0.0408	Missense_Mutation	p.S4414C	Targerted Panel Gene	Adult HGBCL, NOS
BCL-79	chr19	22089165	22089165	C	T	exonic	ZNF257	61	22	0.2651	Missense_Mutation	p.S396L	Targerted Panel Gene	Adult HGBCL, NOS
BCL-79	chr17	39864120	39864120	C	T	exonic	IKZF3	45	18	0.2857	Missense_Mutation	p.D3N	Targerted Panel Gene	Adult HGBCL, NOS
BCL-79	chr1	149886423	149886423	C	T	exonic	HIST2H2BE	85	36	0.2975	Missense_Mutation	p.R73H	Targerted Panel Gene	Adult HGBCL, NOS
BCL-79	chr2	51028144	51028144	G	A	exonic	NRXN1	66	28	0.2979	Missense_Mutation	p.P44S	Targerted Panel Gene	Adult HGBCL, NOS
BCL-79	chr17	39792705	39792705	T	C	exonic	IKZF3	37	16	0.3019	Missense_Mutation	p.N131S	Targerted Panel Gene	Adult HGBCL, NOS
BCL-79	chr7	148816735	148816735	G	A	exonic	EZH2	27	13	0.325	Missense_Mutation	p.A485V	Targerted Panel Gene	Adult HGBCL, NOS
BCL-79	chr2	124911474	124911474	T	G	exonic	CNTNAP5	27	16	0.3721	Missense_Mutation	p.F1220L	Targerted Panel Gene	Adult HGBCL, NOS
BCL-79	chr3	177047353	177047353	C	G	exonic	TBL1XR1	23	16	0.4103	Missense_Mutation	p.A271P	Targerted Panel Gene	Adult HGBCL, NOS
BCL-79	chr6	106088449	106088449	G	C	exonic	PRDM1	26	21	0.4468	Missense_Mutation	p.E97D	Targerted Panel Gene	Adult HGBCL, NOS
BCL-79	chr22	38520026	38520026	C	A	exonic	DMC1	52	2	0.037	Missense_Mutation	p.K339N	Non-Targeted Panel Gene	Adult HGBCL, NOS
BCL-79	chr10	95336972	95336972	G	A	exonic	SORBS1	49	2	0.0392	Missense_Mutation	p.S1017F	Non-Targeted Panel Gene	Adult HGBCL, NOS
BCL-79	chr7	32869791	32869791	C	T	exonic	KBTBD2	44	2	0.0435	Missense_Mutation	p.G476R	Non-Targeted Panel Gene	Adult HGBCL, NOS
BCL-79	chrX	49039190	49039190	C	G	exonic	TFE3	40	2	0.0476	Missense_Mutation	p.A151P	Non-Targeted Panel Gene	Adult HGBCL, NOS
BCL-79	chr17	67911613	67911613	C	A	exonic	BPTF	38	2	0.0488	Missense_Mutation	p.D1369E	Non-Targeted Panel Gene	Adult HGBCL, NOS
BCL-79	chr17	44378407	44378407	G	C	exonic	ITGA2B	37	2	0.0513	Missense_Mutation	p.H683Q	Non-Targeted Panel Gene	Adult HGBCL, NOS

BCL-79	chr19	12464684	12464684	G	A	exonic	ZNF709	74	4	0.0513	Missense_Mutation	p.T413I	Non-Targeted Panel Gene	Adult HGBCL, NOS
BCL-79	chr17	39151373	39151373	T	C	exonic	PLXDC1	71	4	0.0533	Missense_Mutation	p.Q22R	Non-Targeted Panel Gene	Adult HGBCL, NOS
BCL-79	chr3	10328981	10328981	C	T	exonic	ATP2B2	83	5	0.0568	Missense_Mutation	p.D1189N	Non-Targeted Panel Gene	Adult HGBCL, NOS
BCL-79	chr12	110614183	110614183	A	T	exonic	TCTN1	66	4	0.0571	Missense_Mutation	p.M1L	Non-Targeted Panel Gene	Adult HGBCL, NOS
BCL-79	chr3	97872953	97872953	G	T	exonic	CRYBG3	65	4	0.058	Missense_Mutation	p.D587Y	Non-Targeted Panel Gene	Adult HGBCL, NOS
BCL-79	chr12	11268028	11268028	C	T	exonic	PRB3	63	4	0.058	Missense_Mutation	p.R74H	Non-Targeted Panel Gene	Adult HGBCL, NOS
BCL-79	chr19	21808604	21808604	G	C	exonic	ZNF43	107	7	0.0614	Missense_Mutation	p.A472G	Non-Targeted Panel Gene	Adult HGBCL, NOS
BCL-79	chr19	21537236	21537236	G	A	exonic	ZNF429	65	5	0.0714	Missense_Mutation	p.E363K	Non-Targeted Panel Gene	Adult HGBCL, NOS
BCL-79	chr19	48859116	48859116	G	A	exonic	PLEKHA4	39	4	0.093	Missense_Mutation	p.P239L	Non-Targeted Panel Gene	Adult HGBCL, NOS
BCL-79	chr6	31356882	31356882	C	T	exonic	HLA-B	66	8	0.1081	Missense_Mutation	p.G50D	Non-Targeted Panel Gene	Adult HGBCL, NOS
BCL-79	chr13	102643298	102643298	A	T	exonic	TPP2	72	9	0.1111	Missense_Mutation	p.R699S	Non-Targeted Panel Gene	Adult HGBCL, NOS
BCL-79	chr12	122987294	122987294	C	T	exonic	PITPNM2	13	2	0.1333	Missense_Mutation	p.V1134M	Non-Targeted Panel Gene	Adult HGBCL, NOS
BCL-79	chr2	128183787	128183787	C	G	exonic	UGGT1	50	10	0.1667	Missense_Mutation	p.Q1453E	Non-Targeted Panel Gene	Adult HGBCL, NOS
BCL-79	chr3	94036651	94036651	G	A	exonic	ARL13B	24	5	0.1724	Missense_Mutation	p.A181T	Non-Targeted Panel Gene	Adult HGBCL, NOS
BCL-79	chr16	67233682	67233682	C	G	exonic	FHOD1	43	10	0.1852	Missense_Mutation	p.R700P	Non-Targeted Panel Gene	Adult HGBCL, NOS
BCL-79	chr17	35258854	35258854	G	A	exonic	SLFN5	41	11	0.2115	Missense_Mutation	p.G55E	Non-Targeted Panel Gene	Adult HGBCL, NOS
BCL-79	chr2	217818289	217818289	G	A	exonic	TNS1	29	8	0.2162	Missense_Mutation	p.P1244L	Non-Targeted Panel Gene	Adult HGBCL, NOS
BCL-79	chr4	65495441	65495441	C	T	exonic	EPHA5	25	7	0.2188	Missense_Mutation	p.C338Y	Non-Targeted Panel Gene	Adult HGBCL, NOS
BCL-79	chr1	6231984	6231984	T	C	exonic	ICMT	40	12	0.2308	Missense_Mutation	p.H197R	Non-Targeted Panel Gene	Adult HGBCL, NOS
BCL-79	chr11	46320447	46320447	A	G	exonic	CREB3L1	39	12	0.2353	Missense_Mutation	p.Y481C	Non-Targeted Panel Gene	Adult HGBCL, NOS
BCL-79	chr19	18522007	18522007	C	T	exonic	ELL	95	30	0.24	Missense_Mutation	p.V17I	Non-Targeted Panel Gene	Adult HGBCL, NOS
BCL-79	chr11	124954743	124954743	G	C	exonic	CCDC15	50	16	0.2424	Missense_Mutation	p.S4T	Non-Targeted Panel Gene	Adult HGBCL, NOS
BCL-79	chr17	57885480	57885480	C	T	exonic	CUEDC1	86	28	0.2456	Missense_Mutation	p.A29T	Non-Targeted Panel Gene	Adult HGBCL, NOS
BCL-79	chr7	143511313	143511313	C	T	exonic	OR10AC1	6	2	0.25	Missense_Mutation	p.E200K	Non-Targeted Panel Gene	Adult HGBCL, NOS
BCL-79	chr19	49664477	49664477	C	T	exonic	IRF3	91	31	0.2541	Missense_Mutation	p.S9N	Non-Targeted Panel Gene	Adult HGBCL, NOS
BCL-79	chr18	70630640	70630640	T	A	exonic	GTSCR1	90	32	0.2623	Missense_Mutation	p.S111C	Non-Targeted Panel Gene	Adult HGBCL, NOS
BCL-79	chr5	142314273	142314273	T	A	exonic	SPRY4	45	16	0.2623	Missense_Mutation	p.N302I	Non-Targeted Panel Gene	Adult HGBCL, NOS
BCL-79	chr17	30906170	30906170	C	T	exonic	TEFM	98	36	0.2687	Missense_Mutation	p.G10E	Non-Targeted Panel Gene	Adult HGBCL, NOS
BCL-79	chr6	31355353	31355353	G	A	exonic	HLA-B	44	17	0.2787	Missense_Mutation	p.H287Y	Non-Targeted Panel Gene	Adult HGBCL, NOS
BCL-79	chr8	107303314	107303314	C	A	exonic	ANGPT1	33	13	0.2826	Missense_Mutation	p.D287Y	Non-Targeted Panel Gene	Adult HGBCL, NOS
BCL-79	chr12	116019248	116019248	T	C	exonic	MED13L	17	7	0.2917	Missense_Mutation	p.T329A	Non-Targeted Panel Gene	Adult HGBCL, NOS
BCL-79	chr3	48695192	48695192	G	T	exonic	IP6K2	89	37	0.2937	Missense_Mutation	p.R92S	Non-Targeted Panel Gene	Adult HGBCL, NOS
BCL-79	chr6	1610497	1610497	T	G	exonic	FOXC1	84	35	0.2941	Missense_Mutation	p.Y18D	Non-Targeted Panel Gene	Adult HGBCL, NOS
BCL-79	chr11	66021792	66021792	T	C	exonic	CATSPER1	28	12	0.3	Missense_Mutation	p.Y506C	Non-Targeted Panel Gene	Adult HGBCL, NOS
BCL-79	chr22	38866949	38866949	G	A	exonic	CBX6	180	78	0.3023	Missense_Mutation	p.R167W	Non-Targeted Panel Gene	Adult HGBCL, NOS
BCL-79	chr1	150578381	150578381	G	C	exonic	MCL1	48	21	0.3043	Missense_Mutation	p.L114V	Non-Targeted Panel Gene	Adult HGBCL, NOS
BCL-79	chr9	77922332	77922332	A	C	exonic	GNAQ	34	15	0.3061	Missense_Mutation	p.S50R	Non-Targeted Panel Gene	Adult HGBCL, NOS
BCL-79	chr3	12924581	12924581	T	A	exonic	IQSEC1	63	28	0.3077	Missense_Mutation	p.D591V	Non-Targeted Panel Gene	Adult HGBCL, NOS
BCL-79	chr11	116863816	116863816	G	C	exonic	SIK3	37	17	0.3148	Missense_Mutation	p.S445C	Non-Targeted Panel Gene	Adult HGBCL, NOS
BCL-79	chr17	18254089	18254089	G	C	exonic	FLII	38	18	0.3214	Missense_Mutation	p.S223R	Non-Targeted Panel Gene	Adult HGBCL, NOS
BCL-79	chr4	161459266	161459266	A	C	exonic	FSTL5	38	18	0.3214	Missense_Mutation	p.D554E	Non-Targeted Panel Gene	Adult HGBCL, NOS
BCL-79	chr1	190099111	190099111	C	T	exonic	BRINP3	23	11	0.3235	Missense_Mutation	p.R403H	Non-Targeted Panel Gene	Adult HGBCL, NOS
BCL-79	chr12	4911686	4911686	G	A	exonic	KCNA1	39	19	0.3276	Missense_Mutation	p.R103Q	Non-Targeted Panel Gene	Adult HGBCL, NOS
BCL-79	chr3	14523657	14523657	C	T	exonic	GRIP2	18	9	0.3333	Missense_Mutation	p.V149I	Non-Targeted Panel Gene	Adult HGBCL, NOS
BCL-79	chr17	7508420	7508420	C	T	exonic	POLR2A	34	17	0.3333	Missense_Mutation	p.P1137L	Non-Targeted Panel Gene	Adult HGBCL, NOS
BCL-79	chr19	48729523	48729523	C	T	exonic	RASIP1	143	73	0.338	Missense_Mutation	p.R416H	Non-Targeted Panel Gene	Adult HGBCL, NOS
BCL-79	chr9	96388305	96388305	C	G	exonic	ZNF367	29	15	0.3409	Missense_Mutation	p.E329Q	Non-Targeted Panel Gene	Adult HGBCL, NOS
BCL-79	chr3	71754683	71754683	C	T	exonic	GPR27	344	179	0.3423	Missense_Mutation	p.R212C	Non-Targeted Panel Gene	Adult HGBCL, NOS
BCL-79	chr2	137170839	137170839	T	C	exonic	THSD7B	21	11	0.3438	Missense_Mutation	p.C542R	Non-Targeted Panel Gene	Adult HGBCL, NOS
BCL-79	chr8	144060002	144060002	C	T	exonic	OPLAH	70	37	0.3458	Missense_Mutation	p.A11T	Non-Targeted Panel Gene	Adult HGBCL, NOS
BCL-79	chr4	13544242	13544242	C	A	exonic	NKX3-2	163	94	0.3643	Missense_Mutation	p.R58M	Non-Targeted Panel Gene	Adult HGBCL, NOS
BCL-79	chr14	68790416	68790416	G	A	exonic	ZFP36L1	38	22	0.3667	Missense_Mutation	p.A114V	Non-Targeted Panel Gene	Adult HGBCL, NOS
BCL-79	chr5	149944447	149944447	T	G	exonic	PDE6A	28	17	0.3778	Missense_Mutation	p.K76T	Non-Targeted Panel Gene	Adult HGBCL, NOS
BCL-79	chr1	231163816	231163816	A	C	exonic	TRIM67	368	229	0.3836	Missense_Mutation	p.S221R	Non-Targeted Panel Gene	Adult HGBCL, NOS
BCL-79	chr6	49488626	49488626	G	A	exonic	CENPQ	21	15	0.4167	Missense_Mutation	p.S206N	Non-Targeted Panel Gene	Adult HGBCL, NOS
BCL-79	chr11	17612327	17612327	G	C	exonic	OTOG	20	15	0.4286	Missense_Mutation	p.A2097P	Non-Targeted Panel Gene	Adult HGBCL, NOS
BCL-79	chr3	147412612	147412612	C	A	exonic	ZIC1	63	51	0.4474	Missense_Mutation	p.D359E	Non-Targeted Panel Gene	Adult HGBCL, NOS
BCL-79	chr17	67891970	67891970	T	G	exonic	BPTF	20	17	0.4595	Missense_Mutation	p.L790R	Non-Targeted Panel Gene	Adult HGBCL, NOS
BCL-79	chr18	46546944	46546944	C	A	exonic	LOXHD1	47	44	0.4835	Missense_Mutation	p.R1155S	Non-Targeted Panel Gene	Adult HGBCL, NOS
BCL-79	chrX	18177694	18177694	G	T	exonic	BEND2	9	15	0.625	Missense_Mutation	p.P502H	Non-Targeted Panel Gene	Adult HGBCL, NOS
BCL-79	chr12	49042816	49042816	G	A	exonic	KMT2D	74	27	0.2673	Nonsense_Mutation	p.R1903X	Targerted Panel Gene	Adult HGBCL, NOS
BCL-79	chr18	32769857	32769857	C	T	exonic	KLHL14	90	24	0.2105	Nonsense_Mutation	p.W245X	Non-Targeted Panel Gene	Adult HGBCL, NOS
BCL-79	chr13	30921661	30921661	T	G	exonic	MEDAG	49	15	0.2344	Nonsense_Mutation	p.L201X	Non-Targeted Panel Gene	Adult HGBCL, NOS
BCL-79	chr8	35686557	35686557	G	A	exonic	UNC5D	27	9	0.25	Nonsense_Mutation	p.W311X	Non-Targeted Panel Gene	Adult HGBCL, NOS
BCL-79	chr6	27831290	27831290	T	A	exonic	HIST1H4K	49	20	0.2899	Nonsense_Mutation	p.K80X	Non-Targeted Panel Gene	Adult HGBCL, NOS
BCL-79	chr3	38141150	38141150	T	C	exonic	MYD88	64	24	0.2727	Nonstop_Mutation	p.L265P	Targerted Panel Gene	Adult HGBCL, NOS
BCL-79	chr6	31269169	31269169	T	A	exonic	HLA-C	40	14	0.2593	Nonstop_Mutation	p.X367C	Non-Targeted Panel Gene	Adult HGBCL, NOS
BCL-8	chr8	55999551	55999551	T	C	splicing	LYN	36	25	0.4098	Splice_Site	.	Targerted Panel Gene	Adult HGBCL, NOS
BCL-8	chr7	133024628	133024628	C	A	splicing	CHCHD3	17	2	0.1053	Splice_Site	.	Non-Targeted Panel Gene	Adult HGBCL, NOS
BCL-8	chr13	108866176	108866176	G	A	splicing	MYO16	35	9	0.2045	Splice_Site	.	Non-Targeted Panel Gene	Adult HGBCL, NOS
BCL-8	chr13	23862300	23862300	A	G	splicing	MIPEP	17	8	0.32	Splice_Site	.	Non-Targeted Panel Gene	Adult HGBCL, NOS
BCL-8	chr14	30895581	30895581	C	T	splicing	STRN3	16	10	0.3846	Splice_Site	.	Non-Targeted Panel Gene	Adult HGBCL, NOS
BCL-8	chr18	3187638	3187638	C	T	splicing	MYOM1	51	37	0.4205	Splice_Site	.	Non-Targeted Panel Gene	Adult HGBCL, NOS
BCL-8	chr2	70704460	70704460	C	G	splicing	ADD2	21	18	0.4615	Splice_Site	.	Non-Targeted Panel Gene	Adult HGBCL, NOS
BCL-8	chr3	129478014	129478014	A	G	splicing	IFT122	26	30	0.5357	Splice_Site	.	Non-Targeted Panel Gene	Adult HGBCL, NOS
BCL-8	chr17	39742564	39742564	A	G	splicing	GRB7	28	40	0.5882	Splice_Site	.	Non-Targeted Panel Gene	Adult HGBCL, NOS
BCL-8	chr17	7314515	7314516	TC	-	exonic	GPS2	10	18	0.6429	Frame_Shift_Del	p.R59fs	Non-Targeted Panel Gene	Adult HGBCL, NOS
BCL-8	chr12	49044774	49044774	-	GTCC	exonic	KMT2D	49	15	0.2308	Frame_Shift_Ins	p.L1645fs	Targerted Panel Gene	Adult HGBCL, NOS
BCL-8	chr1	119915812	119915812	-	G	exonic	NOTCH2	72	46	0.3866	Frame_Shift_Ins	p.I2304fs	Targerted Panel Gene	Adult HGBCL, NOS
BCL-8	chr14	103928523	103928549	TCACCATCGAGCAC	-	exonic	TDRD9	63	21	0.25	In_Frame_Del	p.5_14del	Non-Targeted Panel Gene	Adult HGBCL, NOS
BCL-8	chr16	28933449	28933451	TAT	-	exonic	CD19	149	89	0.3739	In_Frame_Del	p.259_259del	Non-Targeted Panel Gene	Adult HGBCL, NOS
BCL-8	chr9	136050055	136050060	GCGGCG	-	exonic	NACC2	11	40	0.7547	In_Frame_Del	p.154_156del	Non-Targeted Panel Gene	Adult HGBCL, NOS

BCL-8	chrX	71141301	71141301	-	CAGCAACACCAG	exonic	MED12	11	16	0.4103	In_Frame_Ins	p.Q2113delinsQQQ	Targerted Panel Gene	Adult HGBCL, NOS
BCL-8	chr7	82443365	82443365	G	A	exonic	CACNA2D1	53	3	0.0536	Missense_Mutation	p.T32I	Targerted Panel Gene	Adult HGBCL, NOS
BCL-8	chr20	8741510	8741510	G	T	exonic	PLCB1	35	7	0.1667	Missense_Mutation	p.M820I	Targerted Panel Gene	Adult HGBCL, NOS
BCL-8	chr3	77607950	77607950	A	C	exonic	ROBO2	31	7	0.1842	Missense_Mutation	p.N504H	Targerted Panel Gene	Adult HGBCL, NOS
BCL-8	chr6	26156634	26156634	C	A	exonic	HIST1H1E	64	21	0.2471	Missense_Mutation	p.L82M	Targerted Panel Gene	Adult HGBCL, NOS
BCL-8	chr1	215728031	215728031	T	G	exonic	USH2A	87	31	0.2627	Missense_Mutation	p.K4022T	Targerted Panel Gene	Adult HGBCL, NOS
BCL-8	chr4	133150861	133150861	C	G	exonic	PCDH10	52	22	0.2973	Missense_Mutation	p.L241V	Targerted Panel Gene	Adult HGBCL, NOS
BCL-8	chr22	23181255	23181255	C	T	exonic	BCR	41	20	0.3279	Missense_Mutation	p.P99S	Targerted Panel Gene	Adult HGBCL, NOS
BCL-8	chr4	105243631	105243631	A	G	exonic	TET2	119	60	0.3352	Missense_Mutation	p.H1219R	Targerted Panel Gene	Adult HGBCL, NOS
BCL-8	chr7	103540202	103540202	C	T	exonic	RELN	84	59	0.4126	Missense_Mutation	p.D2309N	Targerted Panel Gene	Adult HGBCL, NOS
BCL-8	chr22	22888206	22888206	C	T	exonic	IGLL5	21	17	0.4474	Missense_Mutation	p.T16I	Targerted Panel Gene	Adult HGBCL, NOS
BCL-8	chr19	4513520	4513520	A	G	exonic	PLIN4	38	32	0.4571	Missense_Mutation	p.M133T	Targerted Panel Gene	Adult HGBCL, NOS
BCL-8	chr12	50363252	50363252	T	C	exonic	FAM186A	21	18	0.4615	Missense_Mutation	p.K102R	Targerted Panel Gene	Adult HGBCL, NOS
BCL-8	chr8	112954695	112954695	T	C	exonic	CSMD3	10	9	0.4737	Missense_Mutation	p.K430R	Targerted Panel Gene	Adult HGBCL, NOS
BCL-8	chr19	16903842	16903842	G	T	exonic	CPAMD8	101	92	0.4767	Missense_Mutation	p.L1470M	Targerted Panel Gene	Adult HGBCL, NOS
BCL-8	chr12	49034433	49034433	G	A	exonic	KMT2D	70	68	0.4928	Missense_Mutation	p.P3495L	Targerted Panel Gene	Adult HGBCL, NOS
BCL-8	chr3	187726764	187726764	C	G	exonic	BCL6	74	95	0.5556	Missense_Mutation	p.G559R	Targerted Panel Gene	Adult HGBCL, NOS
BCL-8	chr17	7674220	7674220	C	T	exonic	TP53	54	101	0.6516	Missense_Mutation	p.R209Q	Targerted Panel Gene	Adult HGBCL, NOS
BCL-8	chr17	5221306	5221306	C	T	exonic	SCIMP	56	3	0.05	Missense_Mutation	p.D64N	Non-Targeted Panel Gene	Adult HGBCL, NOS
BCL-8	chr19	55517965	55517965	C	A	exonic	SSC5D	36	2	0.0526	Missense_Mutation	p.P1230H	Non-Targeted Panel Gene	Adult HGBCL, NOS
BCL-8	chr14	103127441	103127441	G	C	exonic	TNFAIP2	34	2	0.0541	Missense_Mutation	p.M224I	Non-Targeted Panel Gene	Adult HGBCL, NOS
BCL-8	chr1	222991629	222991629	C	T	exonic	DISP1	45	3	0.0625	Missense_Mutation	p.A258V	Non-Targeted Panel Gene	Adult HGBCL, NOS
BCL-8	chr22	41520204	41520204	G	C	exonic	ACO2	29	2	0.0645	Missense_Mutation	p.D356H	Non-Targeted Panel Gene	Adult HGBCL, NOS
BCL-8	chr3	148865502	148865502	A	T	exonic	CPA3	141	10	0.0662	Missense_Mutation	p.D33V	Non-Targeted Panel Gene	Adult HGBCL, NOS
BCL-8	chr16	4899054	4899054	G	C	exonic	PPL	25	2	0.0741	Missense_Mutation	p.Q279E	Non-Targeted Panel Gene	Adult HGBCL, NOS
BCL-8	chr22	30376707	30376707	G	T	exonic	CCDC157	34	3	0.0811	Missense_Mutation	p.A741S	Non-Targeted Panel Gene	Adult HGBCL, NOS
BCL-8	chr15	65913947	65913947	G	C	exonic	MEGF11	46	9	0.1636	Missense_Mutation	p.P834A	Non-Targeted Panel Gene	Adult HGBCL, NOS
BCL-8	chr3	149520941	149520941	C	T	exonic	WWTR1	107	21	0.1641	Missense_Mutation	p.R356H	Non-Targeted Panel Gene	Adult HGBCL, NOS
BCL-8	chr11	66294956	66294956	C	T	exonic	TMEM151A	60	15	0.2	Missense_Mutation	p.T237M	Non-Targeted Panel Gene	Adult HGBCL, NOS
BCL-8	chr6	110438743	110438743	C	T	exonic	SLC22A16	61	16	0.2078	Missense_Mutation	p.G430S	Non-Targeted Panel Gene	Adult HGBCL, NOS
BCL-8	chr12	20855062	20855062	C	A	exonic	SLCO1B3	30	8	0.2105	Missense_Mutation	p.A40D	Non-Targeted Panel Gene	Adult HGBCL, NOS
BCL-8	chr15	43905913	43905913	C	T	exonic	FRMD5	22	6	0.2143	Missense_Mutation	p.G67S	Non-Targeted Panel Gene	Adult HGBCL, NOS
BCL-8	chr2	237834387	237834387	T	A	exonic	RBM44	11	3	0.2143	Missense_Mutation	p.S1049T	Non-Targeted Panel Gene	Adult HGBCL, NOS
BCL-8	chr17	8112168	8112168	A	T	exonic	ALOXE3	61	17	0.2179	Missense_Mutation	p.L237M	Non-Targeted Panel Gene	Adult HGBCL, NOS
BCL-8	chr12	29464513	29464513	T	A	exonic	OVCH1	37	11	0.2292	Missense_Mutation	p.S707C	Non-Targeted Panel Gene	Adult HGBCL, NOS
BCL-8	chr17	16940415	16940415	G	T	exonic	TNFRSF13B	40	12	0.2308	Missense_Mutation	p.A181E	Non-Targeted Panel Gene	Adult HGBCL, NOS
BCL-8	chr14	47061393	47061393	C	A	exonic	MDGA2	68	22	0.2444	Missense_Mutation	p.D163Y	Non-Targeted Panel Gene	Adult HGBCL, NOS
BCL-8	chr17	47743166	47743166	A	G	exonic	TBX21	117	39	0.25	Missense_Mutation	p.K248E	Non-Targeted Panel Gene	Adult HGBCL, NOS
BCL-8	chr6	14118065	14118065	G	C	exonic	CD83	66	24	0.2667	Missense_Mutation	p.K51N	Non-Targeted Panel Gene	Adult HGBCL, NOS
BCL-8	chr5	141101215	141101215	G	T	exonic	PCDHB3	93	34	0.2677	Missense_Mutation	p.R189M	Non-Targeted Panel Gene	Adult HGBCL, NOS
BCL-8	chr8	17655871	17655871	G	T	exonic	MTUS1	13	5	0.2778	Missense_Mutation	p.Q1034K	Non-Targeted Panel Gene	Adult HGBCL, NOS
BCL-8	chr6	47808235	47808235	C	G	exonic	OPN5	47	19	0.2879	Missense_Mutation	p.P280A	Non-Targeted Panel Gene	Adult HGBCL, NOS
BCL-8	chr17	32487649	32487649	G	C	exonic	CDK5R1	118	48	0.2892	Missense_Mutation	p.S10T	Non-Targeted Panel Gene	Adult HGBCL, NOS
BCL-8	chr16	66912115	66912115	C	T	exonic	CDH16	107	44	0.2914	Missense_Mutation	p.S525N	Non-Targeted Panel Gene	Adult HGBCL, NOS
BCL-8	chr2	27664495	27664495	G	A	exonic	SLC4A1AP	71	30	0.297	Missense_Mutation	p.R248H	Non-Targeted Panel Gene	Adult HGBCL, NOS
BCL-8	chr1	59457024	59457024	C	A	exonic	FGGY	54	23	0.2987	Missense_Mutation	p.F150L	Non-Targeted Panel Gene	Adult HGBCL, NOS
BCL-8	chr11	20095730	20095730	G	T	exonic	NAV2	61	26	0.2989	Missense_Mutation	p.W2051L	Non-Targeted Panel Gene	Adult HGBCL, NOS
BCL-8	chr1	158078073	158078073	C	A	exonic	KIRREL	81	35	0.3017	Missense_Mutation	p.D95E	Non-Targeted Panel Gene	Adult HGBCL, NOS
BCL-8	chr17	80221723	80221723	G	A	exonic	SLC26A11	52	23	0.3067	Missense_Mutation	p.G55S	Non-Targeted Panel Gene	Adult HGBCL, NOS
BCL-8	chr2	11202094	11202094	C	G	exonic	ROCK2	40	18	0.3103	Missense_Mutation	p.M859I	Non-Targeted Panel Gene	Adult HGBCL, NOS
BCL-8	chr2	240726917	240726917	G	A	exonic	KIF1A	62	28	0.3111	Missense_Mutation	p.A1261V	Non-Targeted Panel Gene	Adult HGBCL, NOS
BCL-8	chr20	2637561	2637561	A	T	exonic	TMC2	42	19	0.3115	Missense_Mutation	p.N825Y	Non-Targeted Panel Gene	Adult HGBCL, NOS
BCL-8	chrX	35941310	35941310	T	G	exonic	CFAP47	33	15	0.3125	Missense_Mutation	p.I143M	Non-Targeted Panel Gene	Adult HGBCL, NOS
BCL-8	chr4	88139959	88139959	A	T	exonic	ABCG2	24	11	0.3143	Missense_Mutation	p.S13T	Non-Targeted Panel Gene	Adult HGBCL, NOS
BCL-8	chr17	46031508	46031508	C	T	exonic	KANSL1	43	20	0.3175	Missense_Mutation	p.A1095T	Non-Targeted Panel Gene	Adult HGBCL, NOS
BCL-8	chr5	113064113	113064113	C	T	exonic	MCC	58	27	0.3176	Missense_Mutation	p.C695Y	Non-Targeted Panel Gene	Adult HGBCL, NOS
BCL-8	chr10	89599262	89599262	T	A	exonic	PANK1	49	23	0.3194	Missense_Mutation	p.T209S	Non-Targeted Panel Gene	Adult HGBCL, NOS
BCL-8	chr3	73383591	73383591	C	T	exonic	PDZRN3	53	26	0.3291	Missense_Mutation	p.R992H	Non-Targeted Panel Gene	Adult HGBCL, NOS
BCL-8	chr2	186761664	186761664	A	T	exonic	FAM171B	14	7	0.3333	Missense_Mutation	p.E441V	Non-Targeted Panel Gene	Adult HGBCL, NOS
BCL-8	chr19	39173062	39173062	A	G	exonic	PAK4	20	10	0.3333	Missense_Mutation	p.M117V	Non-Targeted Panel Gene	Adult HGBCL, NOS
BCL-8	chr20	4932562	4932562	T	C	exonic	SLC23A2	24	12	0.3333	Missense_Mutation	p.M1V	Non-Targeted Panel Gene	Adult HGBCL, NOS
BCL-8	chr10	124038533	124038533	A	T	exonic	CHST15	86	45	0.3435	Missense_Mutation	p.L391H	Non-Targeted Panel Gene	Adult HGBCL, NOS
BCL-8	chr2	32089578	32089578	A	C	exonic	SPAST	36	19	0.3455	Missense_Mutation	p.M187L	Non-Targeted Panel Gene	Adult HGBCL, NOS
BCL-8	chr19	32976558	32976558	C	T	exonic	FAAP24	69	37	0.3491	Missense_Mutation	p.P175L	Non-Targeted Panel Gene	Adult HGBCL, NOS
BCL-8	chr5	140927428	140927428	G	A	exonic	PCDHAC1	270	147	0.3525	Missense_Mutation	p.G179D	Non-Targeted Panel Gene	Adult HGBCL, NOS
BCL-8	chr22	41540704	41540704	G	A	exonic	POLR3H	158	87	0.3551	Missense_Mutation	p.T68I	Non-Targeted Panel Gene	Adult HGBCL, NOS
BCL-8	chr11	108509851	108509851	A	C	exonic	EXPH5	52	29	0.358	Missense_Mutation	p.Y1879D	Non-Targeted Panel Gene	Adult HGBCL, NOS
BCL-8	chrX	18893571	18893571	C	T	exonic	PHKA2	90	52	0.3662	Missense_Mutation	p.A1208T	Non-Targeted Panel Gene	Adult HGBCL, NOS
BCL-8	chr2	29071461	29071461	G	A	exonic	C2orf71	15	9	0.375	Missense_Mutation	p.P934L	Non-Targeted Panel Gene	Adult HGBCL, NOS
BCL-8	chr18	31157405	31157405	T	C	exonic	DSC1	31	19	0.38	Missense_Mutation	p.E106G	Non-Targeted Panel Gene	Adult HGBCL, NOS
BCL-8	chr2	164939554	164939554	C	G	exonic	SLC38A11	16	10	0.3846	Missense_Mutation	p.D89H	Non-Targeted Panel Gene	Adult HGBCL, NOS
BCL-8	chr12	2691035	2691035	G	A	exonic	CACNA1C	44	28	0.3889	Missense_Mutation	p.G2168S	Non-Targeted Panel Gene	Adult HGBCL, NOS
BCL-8	chrX	133324335	133324335	C	A	exonic	GPC4	67	43	0.3909	Missense_Mutation	p.R174L	Non-Targeted Panel Gene	Adult HGBCL, NOS
BCL-8	chrX	12976283	12976283	G	C	exonic	TMSB4X	34	22	0.3929	Missense_Mutation	p.A8P	Non-Targeted Panel Gene	Adult HGBCL, NOS
BCL-8	chr17	76626769	76626769	T	C	exonic	ST6GALNAC1	151	98	0.3936	Missense_Mutation	p.K266R	Non-Targeted Panel Gene	Adult HGBCL, NOS
BCL-8	chr5	157502900	157502900	A	G	exonic	ADAM19	35	23	0.3966	Missense_Mutation	p.L404P	Non-Targeted Panel Gene	Adult HGBCL, NOS
BCL-8	chr11	70325537	70325537	G	A	exonic	PPFIA1	18	12	0.4	Missense_Mutation	p.S190N	Non-Targeted Panel Gene	Adult HGBCL, NOS
BCL-8	chr12	132729878	132729878	T	G	exonic	ANKLE2	31	21	0.4038	Missense_Mutation	p.I762L	Non-Targeted Panel Gene	Adult HGBCL, NOS
BCL-8	chr19	50451292	50451292	A	G	exonic	MYBPC2	61	42	0.4078	Missense_Mutation	p.E531G	Non-Targeted Panel Gene	Adult HGBCL, NOS
BCL-8	chr10	17853090	17853090	A	G	exonic	MRC1	29	20	0.4082	Missense_Mutation	p.N458S	Non-Targeted Panel Gene	Adult HGBCL, NOS
BCL-8	chr4	86771280	86771280	A	G	exonic	PTPN13	49	35	0.4167	Missense_Mutation	p.K1643R	Non-Targeted Panel Gene	Adult HGBCL, NOS
BCL-8	chr10	97679868	97679868	G	A	exonic	AVPI1	62	46	0.422	Missense_Mutation	p.P13L	Non-Targeted Panel Gene	Adult HGBCL, NOS

BCL-8	chr11	56137579	56137579	G	C	exonic	OR8J3	66	50	0.431	Missense_Mutation	p.T47S	Non-Targeted Panel Gene	Adult HGBCL, NOS
BCL-8	chr7	140034316	140034316	T	C	exonic	PARP12	47	37	0.4405	Missense_Mutation	p.N447S	Non-Targeted Panel Gene	Adult HGBCL, NOS
BCL-8	chr3	1325907	1325907	C	T	exonic	CNTN6	38	30	0.4412	Missense_Mutation	p.P347S	Non-Targeted Panel Gene	Adult HGBCL, NOS
BCL-8	chr19	10154718	10154718	A	G	exonic	DNMT1	88	71	0.4465	Missense_Mutation	p.L446P	Non-Targeted Panel Gene	Adult HGBCL, NOS
BCL-8	chr6	16295171	16295171	C	G	exonic	GMPR	57	46	0.4466	Missense_Mutation	p.N341K	Non-Targeted Panel Gene	Adult HGBCL, NOS
BCL-8	chr8	93922419	93922419	T	A	exonic	PDP1	42	35	0.4545	Missense_Mutation	p.N145K	Non-Targeted Panel Gene	Adult HGBCL, NOS
BCL-8	chr1	236754055	236754055	G	C	exonic	ACTN2	27	23	0.46	Missense_Mutation	p.G442R	Non-Targeted Panel Gene	Adult HGBCL, NOS
BCL-8	chr1	154571934	154571934	G	A	exonic	CHRNB2	38	33	0.4648	Missense_Mutation	p.E371K	Non-Targeted Panel Gene	Adult HGBCL, NOS
BCL-8	chr14	74797744	74797744	C	T	exonic	YLPM1	61	54	0.4696	Missense_Mutation	p.P816L	Non-Targeted Panel Gene	Adult HGBCL, NOS
BCL-8	chr4	1212275	1212275	C	T	exonic	CTBP1	9	8	0.4706	Missense_Mutation	p.E419K	Non-Targeted Panel Gene	Adult HGBCL, NOS
BCL-8	chrX	55624186	55624186	G	A	exonic	FOXR2	36	32	0.4706	Missense_Mutation	p.A159T	Non-Targeted Panel Gene	Adult HGBCL, NOS
BCL-8	chr11	22625438	22625438	C	T	exonic	FANCF	73	69	0.4859	Missense_Mutation	p.D125N	Non-Targeted Panel Gene	Adult HGBCL, NOS
BCL-8	chr14	20242978	20242978	C	T	exonic	OR11H4	35	34	0.4928	Missense_Mutation	p.P63S	Non-Targeted Panel Gene	Adult HGBCL, NOS
BCL-8	chr19	48043912	48043912	G	A	exonic	CABP5	7	7	0.5	Missense_Mutation	p.P4L	Non-Targeted Panel Gene	Adult HGBCL, NOS
BCL-8	chr8	8377810	8377810	C	G	exonic	PRAG1	79	82	0.5062	Missense_Mutation	p.R200P	Non-Targeted Panel Gene	Adult HGBCL, NOS
BCL-8	chr2	166305834	166305834	C	T	exonic	SCN9A	17	18	0.5143	Missense_Mutation	p.R185H	Non-Targeted Panel Gene	Adult HGBCL, NOS
BCL-8	chr7	77750057	77750057	A	G	exonic	RSBN1L	18	20	0.5263	Missense_Mutation	p.H446R	Non-Targeted Panel Gene	Adult HGBCL, NOS
BCL-8	chr18	22993593	22993593	G	A	exonic	RBBP8	21	24	0.5333	Missense_Mutation	p.R589H	Non-Targeted Panel Gene	Adult HGBCL, NOS
BCL-8	chr11	111753478	111753478	G	T	exonic	PPP2R1B	30	35	0.5385	Missense_Mutation	p.L377I	Non-Targeted Panel Gene	Adult HGBCL, NOS
BCL-8	chr6	142078674	142078674	A	T	exonic	NMBR	26	32	0.5517	Missense_Mutation	p.L70M	Non-Targeted Panel Gene	Adult HGBCL, NOS
BCL-8	chr6	149474426	149474426	G	A	exonic	ZC3H12D	17	21	0.5526	Missense_Mutation	p.L40F	Non-Targeted Panel Gene	Adult HGBCL, NOS
BCL-8	chr5	147828115	147828115	T	C	exonic	SPINK1	19	24	0.5581	Missense_Mutation	p.N34S	Non-Targeted Panel Gene	Adult HGBCL, NOS
BCL-8	chr3	98133051	98133051	G	A	exonic	OR5H1	20	27	0.5745	Missense_Mutation	p.M118I	Non-Targeted Panel Gene	Adult HGBCL, NOS
BCL-8	chr6	152463486	152463486	T	C	exonic	SYNE1	11	18	0.6207	Missense_Mutation	p.Q655R	Non-Targeted Panel Gene	Adult HGBCL, NOS
BCL-8	chr9	87974031	87974031	C	G	exonic	CDK20	15	26	0.6341	Missense_Mutation	p.G40A	Non-Targeted Panel Gene	Adult HGBCL, NOS
BCL-8	chr17	73423413	73423413	G	A	exonic	SDK2	93	163	0.6367	Missense_Mutation	p.R624C	Non-Targeted Panel Gene	Adult HGBCL, NOS
BCL-8	chr6	31355128	31355128	T	C	exonic	HLA-B	45	98	0.6853	Missense_Mutation	p.M331V	Non-Targeted Panel Gene	Adult HGBCL, NOS
BCL-8	chr13	51968544	51968544	A	G	exonic	ATP7B	9	48	0.8421	Missense_Mutation	p.V425A	Non-Targeted Panel Gene	Adult HGBCL, NOS
BCL-8	chr9	114048870	114048870	T	A	exonic	ZNF618	13	86	0.8687	Missense_Mutation	p.L523Q	Non-Targeted Panel Gene	Adult HGBCL, NOS
BCL-8	chr1	183512838	183512838	C	T	exonic	SMG7	41	18	0.3051	Nonsense_Mutation	p.Q40X	Targerted Panel Gene	Adult HGBCL, NOS
BCL-8	chr12	49032761	49032761	G	A	exonic	KMT2D	55	31	0.3563	Nonsense_Mutation	p.R3982X	Targerted Panel Gene	Adult HGBCL, NOS
BCL-8	chr4	20519444	20519444	T	G	exonic	SLIT2	22	16	0.4211	Nonsense_Mutation	p.L374X	Targerted Panel Gene	Adult HGBCL, NOS
BCL-8	chr1	237550657	237550657	C	G	exonic	RYR2	36	27	0.4286	Nonsense_Mutation	p.Y1060X	Targerted Panel Gene	Adult HGBCL, NOS
BCL-8	chr6	24718641	24718641	G	A	exonic	C6orf62	115	30	0.2069	Nonsense_Mutation	p.Q10X	Non-Targeted Panel Gene	Adult HGBCL, NOS
BCL-8	chr3	165036408	165036408	C	T	exonic	SI	41	13	0.2407	Nonsense_Mutation	p.W832X	Non-Targeted Panel Gene	Adult HGBCL, NOS
BCL-8	chr12	95906410	95906410	G	A	exonic	CCDC38	33	16	0.3265	Nonsense_Mutation	p.Q116X	Non-Targeted Panel Gene	Adult HGBCL, NOS
BCL-8	chr1	28571309	28571309	C	T	exonic	TRNAU1AP	90	47	0.3431	Nonsense_Mutation	p.Q222X	Non-Targeted Panel Gene	Adult HGBCL, NOS
BCL-8	chr3	38141150	38141150	T	C	exonic	MYD88	108	50	0.3165	Nonstop_Mutation	p.L265P	Targerted Panel Gene	Adult HGBCL, NOS
BCL-80	chr9	2104002	2104002	G	A	splicing	SMARCA2	27	2	0.069	Splice_Site	.	Non-Targeted Panel Gene	Adult HGBCL, NOS
BCL-80	chr5	90789700	90789700	A	T	splicing	ADGRV1	19	2	0.0952	Splice_Site	.	Non-Targeted Panel Gene	Adult HGBCL, NOS
BCL-80	chr1	192352215	192352215	T	A	splicing	RGS21	36	14	0.28	Splice_Site	.	Non-Targeted Panel Gene	Adult HGBCL, NOS
BCL-80	chr10	127404324	127404324	-	A	splicing	DOCK1	20	12	0.375	Splice_Site	.	Non-Targeted Panel Gene	Adult HGBCL, NOS
BCL-80	chr12	103798585	103798585	A	G	splicing	NT5DC3	31	20	0.3922	Splice_Site	.	Non-Targeted Panel Gene	Adult HGBCL, NOS
BCL-80	chr20	53253789	53253795	CACGTCA	-	exonic	TSHZ2	67	3	0.0429	Frame_Shift_Del	p.H111fs	Non-Targeted Panel Gene	Adult HGBCL, NOS
BCL-80	chr6	46871993	46871993	C	-	exonic	ADGRF5	32	2	0.0588	Frame_Shift_Del	p.D421fs	Non-Targeted Panel Gene	Adult HGBCL, NOS
BCL-80	chr18	43274036	43274036	T	-	exonic	SYT4	40	24	0.375	Frame_Shift_Del	p.K131fs	Non-Targeted Panel Gene	Adult HGBCL, NOS
BCL-80	chr7	82805708	82805708	-	T	exonic	PCLO	31	14	0.3111	Frame_Shift_Ins	p.N4971fs	Targerted Panel Gene	Adult HGBCL, NOS
BCL-80	chr10	70146493	70146493	-	CGGGCCCGCCTCG(	exonic	TYSND1	90	22	0.1964	Frame_Shift_Ins	p.W32fs	Non-Targeted Panel Gene	Adult HGBCL, NOS
BCL-80	chr16	71733073	71733073	-	G	exonic	AP1G1	42	18	0.3	Frame_Shift_Ins	p.P821fs	Non-Targeted Panel Gene	Adult HGBCL, NOS
BCL-80	chr1	226549023	226549023	-	C	exonic	STUM	98	70	0.4142	Frame_Shift_Ins	p.G40fs	Non-Targeted Panel Gene	Adult HGBCL, NOS
BCL-80	chr13	28399071	28399071	-	CC	exonic	FLT1	10	20	0.6667	Frame_Shift_Ins	p.S533fs	Non-Targeted Panel Gene	Adult HGBCL, NOS
BCL-80	chr6	31355390	31355401	AGGCACCACCAC	-	exonic	HLA-B	20	30	0.6	In_Frame_Del	p.271_274del	Non-Targeted Panel Gene	Adult HGBCL, NOS
BCL-80	chr6	138424187	138424187	-	CCTGCCGCCTCCCT	exonic	NHSL1	60	2	0.0323	In_Frame_Ins	p.A1572delinsGAA	Non-Targeted Panel Gene	Adult HGBCL, NOS
BCL-80	chrX	67545320	67545320	-	CAT	exonic	AR	15	3	0.1429	In_Frame_Ins	p.Q58delinsQH	Non-Targeted Panel Gene	Adult HGBCL, NOS
BCL-80	chr12	124356713	124356713	A	T	exonic	NCOR2	57	2	0.0339	Missense_Mutation	p.V1057E	Targerted Panel Gene	Adult HGBCL, NOS
BCL-80	chr1	39422445	39422445	A	C	exonic	MACF1	56	6	0.0968	Missense_Mutation	p.L3234F	Targerted Panel Gene	Adult HGBCL, NOS
BCL-80	chr8	112335353	112335353	C	A	exonic	CSMD3	31	18	0.3673	Missense_Mutation	p.G2341C	Targerted Panel Gene	Adult HGBCL, NOS
BCL-80	chr2	141810354	141810354	T	G	exonic	LRP1B	21	17	0.4474	Missense_Mutation	p.T44P	Targerted Panel Gene	Adult HGBCL, NOS
BCL-80	chr4	105275374	105275374	T	A	exonic	TET2	49	50	0.5051	Missense_Mutation	p.L1622M	Targerted Panel Gene	Adult HGBCL, NOS
BCL-80	chr2	27231488	27231488	C	T	exonic	CAD	19	24	0.5581	Missense_Mutation	p.R770C	Targerted Panel Gene	Adult HGBCL, NOS
BCL-80	chr11	103200112	103200112	C	T	exonic	DYNC2H1	15	22	0.5946	Missense_Mutation	p.H2719Y	Targerted Panel Gene	Adult HGBCL, NOS
BCL-80	chr3	77522779	77522779	G	T	exonic	ROBO2	6	10	0.625	Missense_Mutation	p.D271Y	Targerted Panel Gene	Adult HGBCL, NOS
BCL-80	chr17	47820218	47820218	T	A	exonic	OSBPL7	113	2	0.0174	Missense_Mutation	p.S21C	Non-Targeted Panel Gene	Adult HGBCL, NOS
BCL-80	chr7	108168318	108168318	G	A	exonic	NRCAM	66	2	0.0294	Missense_Mutation	p.P1091L	Non-Targeted Panel Gene	Adult HGBCL, NOS
BCL-80	chr17	4672287	4672287	C	T	exonic	PELP1	55	2	0.0351	Missense_Mutation	p.E755K	Non-Targeted Panel Gene	Adult HGBCL, NOS
BCL-80	chr22	19041899	19041899	G	A	exonic	DGCR2	54	2	0.0357	Missense_Mutation	p.S356F	Non-Targeted Panel Gene	Adult HGBCL, NOS
BCL-80	chr10	23303447	23303447	C	A	exonic	C10orf67	53	2	0.0364	Missense_Mutation	p.V188L	Non-Targeted Panel Gene	Adult HGBCL, NOS
BCL-80	chr1	150472264	150472264	G	A	exonic	RPRD2	78	3	0.037	Missense_Mutation	p.V1106I	Non-Targeted Panel Gene	Adult HGBCL, NOS
BCL-80	chr1	224434012	224434012	G	A	exonic	WDR26	40	2	0.0476	Missense_Mutation	p.L32F	Non-Targeted Panel Gene	Adult HGBCL, NOS
BCL-80	chrX	10136640	10136640	G	A	exonic	WWC3	58	3	0.0492	Missense_Mutation	p.R924H	Non-Targeted Panel Gene	Adult HGBCL, NOS
BCL-80	chr9	89402929	89402929	C	A	exonic	SEMA4D	55	3	0.0517	Missense_Mutation	p.G65V	Non-Targeted Panel Gene	Adult HGBCL, NOS
BCL-80	chrX	47641296	47641296	C	T	exonic	ELK1	63	4	0.0597	Missense_Mutation	p.R49H	Non-Targeted Panel Gene	Adult HGBCL, NOS
BCL-80	chr1	45623849	45623849	G	A	exonic	CCDC17	61	4	0.0615	Missense_Mutation	p.R21C	Non-Targeted Panel Gene	Adult HGBCL, NOS
BCL-80	chr11	124754727	124754727	G	A	exonic	ESAM	57	4	0.0656	Missense_Mutation	p.S215L	Non-Targeted Panel Gene	Adult HGBCL, NOS
BCL-80	chr13	108270085	108270085	G	T	exonic	TNFSF13B	28	2	0.0667	Missense_Mutation	p.V64L	Non-Targeted Panel Gene	Adult HGBCL, NOS
BCL-80	chr18	3457440	3457440	C	A	exonic	TGIF1	53	4	0.0702	Missense_Mutation	p.Q87K	Non-Targeted Panel Gene	Adult HGBCL, NOS
BCL-80	chr2	86137136	86137136	C	A	exonic	PTCD3	39	3	0.0714	Missense_Mutation	p.Q659K	Non-Targeted Panel Gene	Adult HGBCL, NOS
BCL-80	chrX	9705035	9705035	C	A	exonic	TBL1X	19	2	0.0952	Missense_Mutation	p.A386D	Non-Targeted Panel Gene	Adult HGBCL, NOS
BCL-80	chr19	8975567	8975567	T	C	exonic	MUC16	42	5	0.1064	Missense_Mutation	p.T1858A	Non-Targeted Panel Gene	Adult HGBCL, NOS
BCL-80	chr20	31848894	31848894	G	A	exonic	DUSP15	48	7	0.1273	Missense_Mutation	p.P213L	Non-Targeted Panel Gene	Adult HGBCL, NOS
BCL-80	chr1	1071885	1071885	G	A	exonic	RNF223	57	9	0.1364	Missense_Mutation	p.P228S	Non-Targeted Panel Gene	Adult HGBCL, NOS
BCL-80	chr2	118847077	118847077	G	T	exonic	EN1	83	17	0.17	Missense_Mutation	p.L31I	Non-Targeted Panel Gene	Adult HGBCL, NOS

BCL-80	chr16	5071961	5071961	G	A	exonic	ALG1	53	11	0.1719	Missense_Mutation	p.V38M	Non-Targeted Panel Gene	Adult HGBCL, NOS
BCL-80	chr18	57562950	57562950	T	C	exonic	FECH	52	16	0.2353	Missense_Mutation	p.Y216C	Non-Targeted Panel Gene	Adult HGBCL, NOS
BCL-80	chr20	64049416	64049416	C	T	exonic	SOX18	25	9	0.2647	Missense_Mutation	p.G34D	Non-Targeted Panel Gene	Adult HGBCL, NOS
BCL-80	chr6	84152600	84152600	C	T	exonic	CEP162	28	11	0.2821	Missense_Mutation	p.E1192K	Non-Targeted Panel Gene	Adult HGBCL, NOS
BCL-80	chr12	11267968	11267968	G	T	exonic	PRB3	34	14	0.2917	Missense_Mutation	p.P94H	Non-Targeted Panel Gene	Adult HGBCL, NOS
BCL-80	chr2	208493367	208493367	C	T	exonic	PTH2R	23	10	0.303	Missense_Mutation	p.T454I	Non-Targeted Panel Gene	Adult HGBCL, NOS
BCL-80	chr1	171185718	171185718	C	A	exonic	FMO2	31	15	0.3261	Missense_Mutation	p.A2E	Non-Targeted Panel Gene	Adult HGBCL, NOS
BCL-80	chr20	46545904	46545904	A	G	exonic	OCSTAMP	24	12	0.3333	Missense_Mutation	p.L157P	Non-Targeted Panel Gene	Adult HGBCL, NOS
BCL-80	chr8	144247383	144247383	A	G	exonic	MROH1	45	23	0.3382	Missense_Mutation	p.Q985R	Non-Targeted Panel Gene	Adult HGBCL, NOS
BCL-80	chr13	71866486	71866486	C	T	exonic	DACH1	17	9	0.3462	Missense_Mutation	p.G95D	Non-Targeted Panel Gene	Adult HGBCL, NOS
BCL-80	chr4	56314959	56314959	G	A	exonic	KIAA1211	17	9	0.3462	Missense_Mutation	p.G486E	Non-Targeted Panel Gene	Adult HGBCL, NOS
BCL-80	chr4	54267714	54267714	C	A	exonic	PDGFRA	15	8	0.3478	Missense_Mutation	p.T390N	Non-Targeted Panel Gene	Adult HGBCL, NOS
BCL-80	chr2	227259811	227259811	T	C	exonic	COL4A3	20	11	0.3548	Missense_Mutation	p.Y350H	Non-Targeted Panel Gene	Adult HGBCL, NOS
BCL-80	chr3	165036389	165036389	C	G	exonic	SI	34	19	0.3585	Missense_Mutation	p.D839H	Non-Targeted Panel Gene	Adult HGBCL, NOS
BCL-80	chr1	91343482	91343482	T	A	exonic	HFM1	30	18	0.375	Missense_Mutation	p.L761F	Non-Targeted Panel Gene	Adult HGBCL, NOS
BCL-80	chr15	43386318	43386318	C	A	exonic	TUBGCP4	5	3	0.375	Missense_Mutation	p.S334R	Non-Targeted Panel Gene	Adult HGBCL, NOS
BCL-80	chr2	98822744	98822744	G	T	exonic	KIAA1211L	63	38	0.3762	Missense_Mutation	p.A510D	Non-Targeted Panel Gene	Adult HGBCL, NOS
BCL-80	chr5	140875600	140875600	C	A	exonic	PCDHA12	28	17	0.3778	Missense_Mutation	p.T43N	Non-Targeted Panel Gene	Adult HGBCL, NOS
BCL-80	chr1	211575391	211575391	C	A	exonic	SLC30A1	97	59	0.3782	Missense_Mutation	p.L507F	Non-Targeted Panel Gene	Adult HGBCL, NOS
BCL-80	chr16	1775931	1775931	C	G	exonic	EME2	36	22	0.3793	Missense_Mutation	p.P305R	Non-Targeted Panel Gene	Adult HGBCL, NOS
BCL-80	chr19	31592708	31592708	G	A	exonic	THEG5	26	16	0.381	Missense_Mutation	p.R31H	Non-Targeted Panel Gene	Adult HGBCL, NOS
BCL-80	chr13	48412068	48412068	A	C	exonic	LPAR6	45	28	0.3836	Missense_Mutation	p.V119G	Non-Targeted Panel Gene	Adult HGBCL, NOS
BCL-80	chr5	176486379	176486379	G	A	exonic	FAF2	36	23	0.3898	Missense_Mutation	p.E53K	Non-Targeted Panel Gene	Adult HGBCL, NOS
BCL-80	chr19	38905043	38905043	C	G	exonic	NFKBIB	107	69	0.392	Missense_Mutation	p.H70D	Non-Targeted Panel Gene	Adult HGBCL, NOS
BCL-80	chr4	1703648	1703648	T	C	exonic	SLBP	50	33	0.3976	Missense_Mutation	p.S77G	Non-Targeted Panel Gene	Adult HGBCL, NOS
BCL-80	chr17	38841283	38841283	A	G	exonic	C17orf98	72	48	0.4	Missense_Mutation	p.L36P	Non-Targeted Panel Gene	Adult HGBCL, NOS
BCL-80	chr9	24544984	24544984	A	C	exonic	IZUMO3	18	12	0.4	Missense_Mutation	p.F127V	Non-Targeted Panel Gene	Adult HGBCL, NOS
BCL-80	chr2	98370025	98370025	A	G	exonic	CNGA3	36	25	0.4098	Missense_Mutation	p.K17R	Non-Targeted Panel Gene	Adult HGBCL, NOS
BCL-80	chr20	19992257	19992257	T	C	exonic	RIN2	20	14	0.4118	Missense_Mutation	p.Y769H	Non-Targeted Panel Gene	Adult HGBCL, NOS
BCL-80	chr1	54584689	54584689	G	A	exonic	ACOT11	38	27	0.4154	Missense_Mutation	p.R23Q	Non-Targeted Panel Gene	Adult HGBCL, NOS
BCL-80	chr14	69500202	69500202	T	A	exonic	PLEKHD1	35	25	0.4167	Missense_Mutation	p.H79Q	Non-Targeted Panel Gene	Adult HGBCL, NOS
BCL-80	chr12	111749312	111749312	C	A	exonic	ACAD10	32	23	0.4182	Missense_Mutation	p.F959L	Non-Targeted Panel Gene	Adult HGBCL, NOS
BCL-80	chr3	149324887	149324887	C	T	exonic	TM4SF18	29	21	0.42	Missense_Mutation	p.A135T	Non-Targeted Panel Gene	Adult HGBCL, NOS
BCL-80	chr19	32859924	32859924	T	C	exonic	SLC7A9	40	29	0.4203	Missense_Mutation	p.T264A	Non-Targeted Panel Gene	Adult HGBCL, NOS
BCL-80	chr7	44518634	44518634	A	G	exonic	NPC1L1	22	16	0.4211	Missense_Mutation	p.Y1057H	Non-Targeted Panel Gene	Adult HGBCL, NOS
BCL-80	chr5	19473398	19473398	G	C	exonic	CDH18	37	27	0.4219	Missense_Mutation	p.S734C	Non-Targeted Panel Gene	Adult HGBCL, NOS
BCL-80	chr19	3613120	3613120	A	G	exonic	CACTIN	78	57	0.4222	Missense_Mutation	p.V575A	Non-Targeted Panel Gene	Adult HGBCL, NOS
BCL-80	chr6	31763497	31763497	T	G	exonic	SAPCD1	27	20	0.4255	Missense_Mutation	p.F66C	Non-Targeted Panel Gene	Adult HGBCL, NOS
BCL-80	chr1	48411538	48411538	G	T	exonic	SPATA6	24	19	0.4419	Missense_Mutation	p.P111T	Non-Targeted Panel Gene	Adult HGBCL, NOS
BCL-80	chr19	11806648	11806648	G	T	exonic	ZNF491	31	25	0.4464	Missense_Mutation	p.R232M	Non-Targeted Panel Gene	Adult HGBCL, NOS
BCL-80	chr5	171240993	171240993	A	T	exonic	RANBP17	28	23	0.451	Missense_Mutation	p.I830F	Non-Targeted Panel Gene	Adult HGBCL, NOS
BCL-80	chr12	92770158	92770158	C	A	exonic	PLEKHG7	51	42	0.4516	Missense_Mutation	p.S368Y	Non-Targeted Panel Gene	Adult HGBCL, NOS
BCL-80	chr4	53391468	53391468	G	C	exonic	FIP1L1	38	32	0.4571	Missense_Mutation	p.E225D	Non-Targeted Panel Gene	Adult HGBCL, NOS
BCL-80	chr3	49967873	49967873	G	C	exonic	RBM6	28	24	0.4615	Missense_Mutation	p.A150P	Non-Targeted Panel Gene	Adult HGBCL, NOS
BCL-80	chr6	42860286	42860286	C	G	exonic	BICRAL	22	19	0.4634	Missense_Mutation	p.A760G	Non-Targeted Panel Gene	Adult HGBCL, NOS
BCL-80	chr7	150742362	150742362	G	A	exonic	GIMAP1-GIMAP5;(	48	43	0.4674	Missense_Mutation	p.G279R	Non-Targeted Panel Gene	Adult HGBCL, NOS
BCL-80	chr4	182753508	182753508	A	C	exonic	TENM3	33	29	0.4677	Missense_Mutation	p.K1307N	Non-Targeted Panel Gene	Adult HGBCL, NOS
BCL-80	chr3	191275252	191275252	C	T	exonic	UTS2B	25	22	0.4681	Missense_Mutation	p.A112T	Non-Targeted Panel Gene	Adult HGBCL, NOS
BCL-80	chr1	162566094	162566094	C	T	exonic	UAP1	95	84	0.4693	Missense_Mutation	p.T9M	Non-Targeted Panel Gene	Adult HGBCL, NOS
BCL-80	chr7	101087650	101087650	G	A	exonic	TRIM56	53	47	0.47	Missense_Mutation	p.G113E	Non-Targeted Panel Gene	Adult HGBCL, NOS
BCL-80	chr5	889598	889598	G	C	exonic	BRD9	25	23	0.4792	Missense_Mutation	p.A99G	Non-Targeted Panel Gene	Adult HGBCL, NOS
BCL-80	chr22	32493269	32493269	C	T	exonic	FBXO7	38	35	0.4795	Missense_Mutation	p.R378C	Non-Targeted Panel Gene	Adult HGBCL, NOS
BCL-80	chr3	49967870	49967870	G	A	exonic	RBM6	26	24	0.48	Missense_Mutation	p.E149K	Non-Targeted Panel Gene	Adult HGBCL, NOS
BCL-80	chr4	70336096	70336096	C	T	exonic	CABS1	28	26	0.4815	Missense_Mutation	p.P353S	Non-Targeted Panel Gene	Adult HGBCL, NOS
BCL-80	chr12	130348143	130348143	T	G	exonic	PIWIL1	32	30	0.4839	Missense_Mutation	p.Y232D	Non-Targeted Panel Gene	Adult HGBCL, NOS
BCL-80	chr3	107773574	107773574	C	T	exonic	BBX	28	28	0.5	Missense_Mutation	p.A618V	Non-Targeted Panel Gene	Adult HGBCL, NOS
BCL-80	chr2	233163889	233163889	G	T	exonic	INPP5D	37	37	0.5	Missense_Mutation	p.V474L	Non-Targeted Panel Gene	Adult HGBCL, NOS
BCL-80	chr2	98822708	98822708	G	A	exonic	KIAA1211L	47	49	0.5104	Missense_Mutation	p.P522L	Non-Targeted Panel Gene	Adult HGBCL, NOS
BCL-80	chr19	49895864	49895864	C	T	exonic	IL4I1	22	23	0.5111	Missense_Mutation	p.G90D	Non-Targeted Panel Gene	Adult HGBCL, NOS
BCL-80	chr11	96091912	96091912	C	G	exonic	MAML2	22	23	0.5111	Missense_Mutation	p.V707L	Non-Targeted Panel Gene	Adult HGBCL, NOS
BCL-80	chr14	76803418	76803418	C	T	exonic	ANGEL1	14	15	0.5172	Missense_Mutation	p.R524Q	Non-Targeted Panel Gene	Adult HGBCL, NOS
BCL-80	chr7	70762909	70762909	G	A	exonic	AUTS2	43	47	0.5222	Missense_Mutation	p.S261N	Non-Targeted Panel Gene	Adult HGBCL, NOS
BCL-80	chr2	74529461	74529461	A	G	exonic	AUP1	53	60	0.531	Missense_Mutation	p.L30P	Non-Targeted Panel Gene	Adult HGBCL, NOS
BCL-80	chr2	26461006	26461006	C	T	exonic	OTOF	44	50	0.5319	Missense_Mutation	p.R1853Q	Non-Targeted Panel Gene	Adult HGBCL, NOS
BCL-80	chr7	87515355	87515355	A	T	exonic	ABCB1	13	15	0.5357	Missense_Mutation	p.L1123H	Non-Targeted Panel Gene	Adult HGBCL, NOS
BCL-80	chrX	1381100	1381100	A	C	exonic	IL3RA	51	62	0.5487	Missense_Mutation	p.K353T	Non-Targeted Panel Gene	Adult HGBCL, NOS
BCL-80	chr10	116849728	116849728	C	A	exonic	ENO4	13	16	0.5517	Missense_Mutation	p.H54Q	Non-Targeted Panel Gene	Adult HGBCL, NOS
BCL-80	chr11	7001225	7001225	T	A	exonic	ZNF214	28	35	0.5556	Missense_Mutation	p.E153V	Non-Targeted Panel Gene	Adult HGBCL, NOS
BCL-80	chr7	70790202	70790202	G	T	exonic	AUTS2	27	34	0.5574	Missense_Mutation	p.A996S	Non-Targeted Panel Gene	Adult HGBCL, NOS
BCL-80	chr15	64406426	64406426	A	C	exonic	TRIP4	18	23	0.561	Missense_Mutation	p.H265P	Non-Targeted Panel Gene	Adult HGBCL, NOS
BCL-80	chr2	27026325	27026325	C	G	exonic	MAPRE3	27	37	0.5781	Missense_Mutation	p.Q275E	Non-Targeted Panel Gene	Adult HGBCL, NOS
BCL-80	chr5	132385370	132385370	C	T	exonic	SLC22A5	18	26	0.5909	Missense_Mutation	p.T256M	Non-Targeted Panel Gene	Adult HGBCL, NOS
BCL-80	chr2	219541122	219541122	C	T	exonic	CHPF	13	20	0.6061	Missense_Mutation	p.V298M	Non-Targeted Panel Gene	Adult HGBCL, NOS
BCL-80	chr19	15457554	15457554	A	G	exonic	RASAL3	28	47	0.6267	Missense_Mutation	p.L390P	Non-Targeted Panel Gene	Adult HGBCL, NOS
BCL-80	chr13	94439954	94439954	A	C	exonic	DCT	22	42	0.6562	Missense_Mutation	p.Y439D	Non-Targeted Panel Gene	Adult HGBCL, NOS
BCL-80	chr1	2306743	2306743	G	A	exonic	SKI	42	115	0.7325	Missense_Mutation	p.G722D	Non-Targeted Panel Gene	Adult HGBCL, NOS
BCL-80	chr3	53178510	53178510	G	A	exonic	PRKCD	9	35	0.7955	Missense_Mutation	p.V30M	Non-Targeted Panel Gene	Adult HGBCL, NOS
BCL-80	chr14	90304149	90304149	G	A	exonic	NRDE2	10	40	0.8	Missense_Mutation	p.P264L	Non-Targeted Panel Gene	Adult HGBCL, NOS
BCL-80	chr11	64735122	64735122	C	T	exonic	RASGRP2	11	47	0.8103	Missense_Mutation	p.D468N	Non-Targeted Panel Gene	Adult HGBCL, NOS
BCL-80	chr1	111727297	111727297	C	T	exonic	FAM212B	8	64	0.8889	Missense_Mutation	p.E174K	Non-Targeted Panel Gene	Adult HGBCL, NOS
BCL-80	chrX	106694086	106694086	G	T	exonic	RNF128	4	33	0.8919	Missense_Mutation	p.M28I	Non-Targeted Panel Gene	Adult HGBCL, NOS
BCL-80	chrX	139629838	139629838	C	T	exonic	MCF2	0	22	1	Missense_Mutation	p.E159K	Non-Targeted Panel Gene	Adult HGBCL, NOS

BCL-80	chrX	124894320	124894320	C	T	exonic	TENM1	0	15	1	Missense_Mutation	p.E171K	Non-Targeted Panel Gene	Adult HGBCL, NOS
BCL-80	chr17	65056395	65056395	G	A	exonic	GNA13	191	144	0.4286	Nonsense_Mutation	p.Q67X	Targerted Panel Gene	Adult HGBCL, NOS
BCL-80	chr17	65053666	65053666	G	A	exonic	GNA13	43	34	0.4416	Nonsense_Mutation	p.Q116X	Targerted Panel Gene	Adult HGBCL, NOS
BCL-80	chr21	39236702	39236702	G	A	exonic	BRWD1	74	4	0.0513	Nonsense_Mutation	p.R887X	Non-Targeted Panel Gene	Adult HGBCL, NOS
BCL-80	chr16	2765890	2765890	C	T	exonic	SRRM2	113	39	0.2566	Nonsense_Mutation	p.R1788X	Non-Targeted Panel Gene	Adult HGBCL, NOS
BCL-80	chr19	47555733	47555733	G	A	exonic	ZNF541	36	20	0.3571	Nonsense_Mutation	p.R42X	Non-Targeted Panel Gene	Adult HGBCL, NOS
BCL-80	chr4	161542577	161542577	C	A	exonic	FSTL5	18	12	0.4	Nonsense_Mutation	p.G378X	Non-Targeted Panel Gene	Adult HGBCL, NOS
BCL-80	chr21	41741557	41741557	G	A	exonic	RIPK4	37	36	0.4932	Nonsense_Mutation	p.Q546X	Non-Targeted Panel Gene	Adult HGBCL, NOS
BCL-80	chr1	206591828	206591828	G	A	exonic	EIF2D	43	47	0.5222	Nonsense_Mutation	p.R568X	Non-Targeted Panel Gene	Adult HGBCL, NOS
BCL-80	chr1	37475678	37475678	C	A	exonic	ZC3H12A	29	76	0.7238	Nonsense_Mutation	p.S61X	Non-Targeted Panel Gene	Adult HGBCL, NOS
BCL-81	chr2	182775314	182775314	A	T	splicing	DNAJC10	17	2	0.1053	Splice_Site	.	Non-Targeted Panel Gene	Adult HGBCL, NOS
BCL-81	chr17	81622164	81622164	A	T	splicing	NPLOC4	17	2	0.1053	Splice_Site	.	Non-Targeted Panel Gene	Adult HGBCL, NOS
BCL-81	chr2	1792465	1792465	C	A	splicing	MYT1L	16	2	0.1111	Splice_Site	.	Non-Targeted Panel Gene	Adult HGBCL, NOS
BCL-81	chr20	35069295	35069295	C	A	splicing	TRPC4AP	11	2	0.1538	Splice_Site	.	Non-Targeted Panel Gene	Adult HGBCL, NOS
BCL-81	chr11	117986536	117986536	T	G	splicing	IL10RA	66	13	0.1646	Splice_Site	.	Non-Targeted Panel Gene	Adult HGBCL, NOS
BCL-81	chr10	5096408	5096408	A	G	splicing	AKR1C3	3	6	0.6667	Splice_Site	.	Non-Targeted Panel Gene	Adult HGBCL, NOS
BCL-81	chr20	61773156	61773156	G	-	exonic	CDH4	43	2	0.0444	Frame_Shift_Del	p.G184fs	Non-Targeted Panel Gene	Adult HGBCL, NOS
BCL-81	chr12	52365060	52365064	CTCGA	-	exonic	KRT85	35	2	0.0541	Frame_Shift_Del	p.I176fs	Non-Targeted Panel Gene	Adult HGBCL, NOS
BCL-81	chr13	49691256	49691259	GCGC	-	exonic	EBPL	28	2	0.0667	Frame_Shift_Del	p.A56fs	Non-Targeted Panel Gene	Adult HGBCL, NOS
BCL-81	chr8	9011582	9011583	TA	-	exonic	ERI1	27	2	0.069	Frame_Shift_Del	p.Y110fs	Non-Targeted Panel Gene	Adult HGBCL, NOS
BCL-81	chr6	30951185	30951191	CCCATTT	-	exonic	DPCR1	46	4	0.08	Frame_Shift_Del	p.T907fs	Non-Targeted Panel Gene	Adult HGBCL, NOS
BCL-81	chr18	46477689	46477723	CTGCCCCGCTCGAA	-	exonic	LOXHD1	32	3	0.0857	Frame_Shift_Del	p.K2129fs	Non-Targeted Panel Gene	Adult HGBCL, NOS
BCL-81	chr5	34004663	34004664	CA	-	exonic	AMACR	19	2	0.0952	Frame_Shift_Del	p.A154fs	Non-Targeted Panel Gene	Adult HGBCL, NOS
BCL-81	chr2	240018607	240018608	TG	-	exonic	NDUFA10	13	2	0.1333	Frame_Shift_Del	p.F164fs	Non-Targeted Panel Gene	Adult HGBCL, NOS
BCL-81	chrX	35802850	35802850	C	-	exonic	MAGEB16	16	3	0.1579	Frame_Shift_Del	p.N218fs	Non-Targeted Panel Gene	Adult HGBCL, NOS
BCL-81	chr15	43361670	43361671	GA	-	exonic	ZSCAN29	26	7	0.2121	Frame_Shift_Del	p.L654fs	Non-Targeted Panel Gene	Adult HGBCL, NOS
BCL-81	chr20	21706952	21706952	G	-	exonic	PAX1	114	46	0.2857	Frame_Shift_Del	p.M267fs	Non-Targeted Panel Gene	Adult HGBCL, NOS
BCL-81	chr2	229818420	229818430	TGACCAGTAAC	-	exonic	TRIP12	7	5	0.4167	Frame_Shift_Del	p.Q482fs	Non-Targeted Panel Gene	Adult HGBCL, NOS
BCL-81	chr14	69236496	69236496	A	-	exonic	EXD2	9	9	0.5	Frame_Shift_Del	p.K416fs	Non-Targeted Panel Gene	Adult HGBCL, NOS
BCL-81	chr6	30951192	30951192	-	G	exonic	DPCR1	40	5	0.1111	Frame_Shift_Ins	p.T910fs	Non-Targeted Panel Gene	Adult HGBCL, NOS
BCL-81	chr9	137053947	137053947	-	GCGGCGGCCAGCA	exonic	ENTPD2	44	7	0.1346	Frame_Shift_Ins	p.A17fs	Non-Targeted Panel Gene	Adult HGBCL, NOS
BCL-81	chr11	16790827	16790827	-	GCCAGGCAGAGGT	exonic	PLEKHA7	18	4	0.1818	Frame_Shift_Ins	p.E1008fs	Non-Targeted Panel Gene	Adult HGBCL, NOS
BCL-81	chrX	41696668	41696668	-	T	exonic	GPR34	22	8	0.2667	Frame_Shift_Ins	p.R345fs	Non-Targeted Panel Gene	Adult HGBCL, NOS
BCL-81	chr2	46360651	46360651	-	A	exonic	EPAS1	13	5	0.2778	Frame_Shift_Ins	p.G156fs	Non-Targeted Panel Gene	Adult HGBCL, NOS
BCL-81	chr2	127986865	127986865	-	C	exonic	SAP130	10	4	0.2857	Frame_Shift_Ins	p.S652fs	Non-Targeted Panel Gene	Adult HGBCL, NOS
BCL-81	chr15	88530752	88530790	GCGCCTCTTGGCCA	-	exonic	DET1	16	2	0.1111	In_Frame_Del	p.272_284del	Non-Targeted Panel Gene	Adult HGBCL, NOS
BCL-81	chr8	138265767	138265772	TTCCGG	-	exonic	FAM135B	12	2	0.1429	In_Frame_Del	p.76_78del	Non-Targeted Panel Gene	Adult HGBCL, NOS
BCL-81	chr1	7738140	7738140	G	T	exonic	CAMTA1	18	2	0.1	Missense_Mutation	p.K1280N	Targerted Panel Gene	Adult HGBCL, NOS
BCL-81	chr11	92859269	92859269	C	A	exonic	FAT3	17	2	0.1053	Missense_Mutation	p.Q3869K	Targerted Panel Gene	Adult HGBCL, NOS
BCL-81	chr1	33663066	33663066	T	A	exonic	CSMD2	16	2	0.1111	Missense_Mutation	p.E1320V	Targerted Panel Gene	Adult HGBCL, NOS
BCL-81	chr22	23288130	23288130	C	A	exonic	BCR	12	2	0.1429	Missense_Mutation	p.R854S	Targerted Panel Gene	Adult HGBCL, NOS
BCL-81	chr2	215000862	215000862	G	T	exonic	ABCA12	13	3	0.1875	Missense_Mutation	p.H1008N	Targerted Panel Gene	Adult HGBCL, NOS
BCL-81	chrX	32645035	32645035	C	T	exonic	DMD	16	5	0.2381	Missense_Mutation	p.D237N	Targerted Panel Gene	Adult HGBCL, NOS
BCL-81	chr16	85909150	85909150	T	C	exonic	IRF8	9	4	0.3077	Missense_Mutation	p.I112T	Targerted Panel Gene	Adult HGBCL, NOS
BCL-81	chr3	78606993	78606993	G	C	exonic	ROBO1	13	6	0.3158	Missense_Mutation	p.A1495G	Targerted Panel Gene	Adult HGBCL, NOS
BCL-81	chr3	177033053	177033053	A	C	exonic	TBL1XR1	13	12	0.48	Missense_Mutation	p.V445G	Targerted Panel Gene	Adult HGBCL, NOS
BCL-81	chr17	16101620	16101620	G	A	exonic	NCOR1	17	22	0.5641	Missense_Mutation	p.P774S	Targerted Panel Gene	Adult HGBCL, NOS
BCL-81	chr14	20223809	20223809	G	T	exonic	OR11H6	53	2	0.0364	Missense_Mutation	p.G34C	Non-Targeted Panel Gene	Adult HGBCL, NOS
BCL-81	chr17	47840758	47840758	A	G	exonic	SCRN2	53	2	0.0364	Missense_Mutation	p.F29S	Non-Targeted Panel Gene	Adult HGBCL, NOS
BCL-81	chr11	67282318	67282318	C	G	exonic	GRK2	39	2	0.0488	Missense_Mutation	p.D335E	Non-Targeted Panel Gene	Adult HGBCL, NOS
BCL-81	chr10	103449601	103449601	G	A	exonic	CALHM2	37	2	0.0513	Missense_Mutation	p.P114L	Non-Targeted Panel Gene	Adult HGBCL, NOS
BCL-81	chr1	36178782	36178782	G	A	exonic	MAP7D1	37	2	0.0513	Missense_Mutation	p.A630T	Non-Targeted Panel Gene	Adult HGBCL, NOS
BCL-81	chr11	118661185	118661185	A	T	exonic	TREH	35	2	0.0541	Missense_Mutation	p.Y278N	Non-Targeted Panel Gene	Adult HGBCL, NOS
BCL-81	chr6	31111902	31111902	C	T	exonic	C6orf15	33	2	0.0571	Missense_Mutation	p.G153R	Non-Targeted Panel Gene	Adult HGBCL, NOS
BCL-81	chr14	73739674	73739674	C	A	exonic	ELMSAN1	48	3	0.0588	Missense_Mutation	p.G112V	Non-Targeted Panel Gene	Adult HGBCL, NOS
BCL-81	chr19	10499688	10499688	G	A	exonic	KEAP1	47	3	0.06	Missense_Mutation	p.R116W	Non-Targeted Panel Gene	Adult HGBCL, NOS
BCL-81	chr11	72221625	72221625	G	A	exonic	FOLR2	25	2	0.0741	Missense_Mutation	p.A211T	Non-Targeted Panel Gene	Adult HGBCL, NOS
BCL-81	chr13	95587694	95587694	T	C	exonic	DZIP1	21	2	0.087	Missense_Mutation	p.D688G	Non-Targeted Panel Gene	Adult HGBCL, NOS
BCL-81	chr12	92421059	92421059	G	T	exonic	CLLU1OS	20	2	0.0909	Missense_Mutation	p.T86K	Non-Targeted Panel Gene	Adult HGBCL, NOS
BCL-81	chr18	63484562	63484562	G	T	exonic	SERPINB5	20	2	0.0909	Missense_Mutation	p.G45V	Non-Targeted Panel Gene	Adult HGBCL, NOS
BCL-81	chr6	43355568	43355568	T	A	exonic	ZNF318	20	2	0.0909	Missense_Mutation	p.Y589F	Non-Targeted Panel Gene	Adult HGBCL, NOS
BCL-81	chr7	107195847	107195847	C	T	exonic	HBP1	19	2	0.0952	Missense_Mutation	p.P361S	Non-Targeted Panel Gene	Adult HGBCL, NOS
BCL-81	chr22	35327314	35327314	T	A	exonic	TOM1	19	2	0.0952	Missense_Mutation	p.V231E	Non-Targeted Panel Gene	Adult HGBCL, NOS
BCL-81	chrX	110454263	110454263	A	G	exonic	RTL9	18	2	0.1	Missense_Mutation	p.K1216E	Non-Targeted Panel Gene	Adult HGBCL, NOS
BCL-81	chr4	10445460	10445460	C	A	exonic	ZNF518B	18	2	0.1	Missense_Mutation	p.C290F	Non-Targeted Panel Gene	Adult HGBCL, NOS
BCL-81	chr10	7172115	7172115	G	A	exonic	SFMBT2	78	9	0.1034	Missense_Mutation	p.A732V	Non-Targeted Panel Gene	Adult HGBCL, NOS
BCL-81	chr5	34004704	34004704	T	A	exonic	AMACR	17	2	0.1053	Missense_Mutation	p.E141V	Non-Targeted Panel Gene	Adult HGBCL, NOS
BCL-81	chr12	45852216	45852216	G	T	exonic	ARID2	17	2	0.1053	Missense_Mutation	p.D1365Y	Non-Targeted Panel Gene	Adult HGBCL, NOS
BCL-81	chr2	96816470	96816470	C	G	exonic	CNNM3	17	2	0.1053	Missense_Mutation	p.R65G	Non-Targeted Panel Gene	Adult HGBCL, NOS
BCL-81	chr14	59360805	59360805	G	T	exonic	DAAM1	17	2	0.1053	Missense_Mutation	p.M889I	Non-Targeted Panel Gene	Adult HGBCL, NOS
BCL-81	chr12	30673995	30673995	G	T	exonic	IPO8	17	2	0.1053	Missense_Mutation	p.Q302K	Non-Targeted Panel Gene	Adult HGBCL, NOS
BCL-81	chr10	77991152	77991152	G	A	exonic	POLR3A	16	2	0.1053	Missense_Mutation	p.P935S	Non-Targeted Panel Gene	Adult HGBCL, NOS
BCL-81	chr2	54649617	54649617	G	A	exonic	SPTBN1	17	2	0.1053	Missense_Mutation	p.M1735I	Non-Targeted Panel Gene	Adult HGBCL, NOS
BCL-81	chr6	83135852	83135852	T	A	exonic	DOPEY1	16	2	0.1111	Missense_Mutation	p.F1035Y	Non-Targeted Panel Gene	Adult HGBCL, NOS
BCL-81	chr12	2864694	2864694	G	A	exonic	FOXM1	16	2	0.1111	Missense_Mutation	p.P360L	Non-Targeted Panel Gene	Adult HGBCL, NOS
BCL-81	chr11	18291868	18291868	C	T	exonic	HPS5	15	2	0.1111	Missense_Mutation	p.D672N	Non-Targeted Panel Gene	Adult HGBCL, NOS
BCL-81	chr22	39515288	39515288	G	A	exonic	MIEF1	24	3	0.1111	Missense_Mutation	p.S451N	Non-Targeted Panel Gene	Adult HGBCL, NOS
BCL-81	chr5	58457581	58457581	G	A	exonic	PLK2	24	3	0.1111	Missense_Mutation	p.T225M	Non-Targeted Panel Gene	Adult HGBCL, NOS
BCL-81	chr1	182532728	182532728	A	T	exonic	RGSL1	16	2	0.1111	Missense_Mutation	p.N811Y	Non-Targeted Panel Gene	Adult HGBCL, NOS
BCL-81	chr9	128826558	128826558	T	A	exonic	SPOUT1	16	2	0.1111	Missense_Mutation	p.Q147L	Non-Targeted Panel Gene	Adult HGBCL, NOS
BCL-81	chr3	113657806	113657806	C	A	exonic	USF3	16	2	0.1111	Missense_Mutation	p.M1292I	Non-Targeted Panel Gene	Adult HGBCL, NOS
BCL-81	chr15	40955388	40955388	G	A	exonic	CHAC1	23	3	0.1154	Missense_Mutation	p.V137M	Non-Targeted Panel Gene	Adult HGBCL, NOS

BCL-81	chr2	9493812	9493812	T	A	exonic	ADAM17	15	2	0.1176	Missense_Mutation	p.K643I	Non-Targeted Panel Gene	Adult HGBCL, NOS
BCL-81	chr15	42399553	42399553	G	A	exonic	CAPN3	15	2	0.1176	Missense_Mutation	p.D419N	Non-Targeted Panel Gene	Adult HGBCL, NOS
BCL-81	chr10	100218106	100218106	G	T	exonic	CHUK	15	2	0.1176	Missense_Mutation	p.N274K	Non-Targeted Panel Gene	Adult HGBCL, NOS
BCL-81	chr7	71416015	71416015	G	A	exonic	GALNT17	15	2	0.1176	Missense_Mutation	p.G239E	Non-Targeted Panel Gene	Adult HGBCL, NOS
BCL-81	chr2	160195432	160195432	A	G	exonic	ITGB6	30	4	0.1176	Missense_Mutation	p.F177S	Non-Targeted Panel Gene	Adult HGBCL, NOS
BCL-81	chr6	73369239	73369239	G	C	exonic	OOEP	15	2	0.1176	Missense_Mutation	p.L113V	Non-Targeted Panel Gene	Adult HGBCL, NOS
BCL-81	chr5	77211235	77211235	G	A	exonic	PDE8B	93	13	0.1226	Missense_Mutation	p.E104K	Non-Targeted Panel Gene	Adult HGBCL, NOS
BCL-81	chr9	104798477	104798477	A	C	exonic	ABCA1	21	3	0.125	Missense_Mutation	p.F1689V	Non-Targeted Panel Gene	Adult HGBCL, NOS
BCL-81	chr11	66315560	66315560	G	C	exonic	CD248	14	2	0.125	Missense_Mutation	p.P490A	Non-Targeted Panel Gene	Adult HGBCL, NOS
BCL-81	chr10	58227121	58227121	A	T	exonic	IPMK	14	2	0.125	Missense_Mutation	p.F99I	Non-Targeted Panel Gene	Adult HGBCL, NOS
BCL-81	chr7	141662706	141662706	C	A	exonic	KIAA1147	14	2	0.125	Missense_Mutation	p.A440S	Non-Targeted Panel Gene	Adult HGBCL, NOS
BCL-81	chr2	74429844	74429844	G	A	exonic	RTKN	21	3	0.125	Missense_Mutation	p.P234S	Non-Targeted Panel Gene	Adult HGBCL, NOS
BCL-81	chr18	76362553	76362553	C	A	exonic	ZNF516	21	3	0.125	Missense_Mutation	p.R1146I	Non-Targeted Panel Gene	Adult HGBCL, NOS
BCL-81	chr3	58508966	58508966	G	T	exonic	ACOX2	13	2	0.1333	Missense_Mutation	p.A637E	Non-Targeted Panel Gene	Adult HGBCL, NOS
BCL-81	chr15	65395121	65395121	G	A	exonic	IGDCC4	13	2	0.1333	Missense_Mutation	p.P517S	Non-Targeted Panel Gene	Adult HGBCL, NOS
BCL-81	chr9	6986650	6986650	G	T	exonic	KDM4C	13	2	0.1333	Missense_Mutation	p.S554I	Non-Targeted Panel Gene	Adult HGBCL, NOS
BCL-81	chr10	96983533	96983533	A	T	exonic	LCOR	13	2	0.1333	Missense_Mutation	p.R1025W	Non-Targeted Panel Gene	Adult HGBCL, NOS
BCL-81	chr8	30843152	30843152	C	T	exonic	TEX15	13	2	0.1333	Missense_Mutation	p.A2339T	Non-Targeted Panel Gene	Adult HGBCL, NOS
BCL-81	chr16	19309034	19309034	G	T	exonic	CLEC19A	19	3	0.1364	Missense_Mutation	p.S171I	Non-Targeted Panel Gene	Adult HGBCL, NOS
BCL-81	chr16	67842609	67842609	A	G	exonic	THAP11	99	16	0.1391	Missense_Mutation	p.K19E	Non-Targeted Panel Gene	Adult HGBCL, NOS
BCL-81	chr6	167914186	167914186	G	A	exonic	AFDN	12	2	0.1429	Missense_Mutation	p.G645R	Non-Targeted Panel Gene	Adult HGBCL, NOS
BCL-81	chr12	30690525	30690525	T	C	exonic	IPO8	12	2	0.1429	Missense_Mutation	p.D46G	Non-Targeted Panel Gene	Adult HGBCL, NOS
BCL-81	chr9	35548146	35548146	C	T	exonic	RUSC2	83	14	0.1443	Missense_Mutation	p.T542I	Non-Targeted Panel Gene	Adult HGBCL, NOS
BCL-81	chr15	23647325	23647325	C	T	exonic	MAGEL2	47	8	0.1455	Missense_Mutation	p.A140T	Non-Targeted Panel Gene	Adult HGBCL, NOS
BCL-81	chr2	65072292	65072292	C	A	exonic	CEP68	34	6	0.15	Missense_Mutation	p.S399Y	Non-Targeted Panel Gene	Adult HGBCL, NOS
BCL-81	chr3	167576020	167576020	A	T	exonic	WDR49	17	3	0.15	Missense_Mutation	p.N469K	Non-Targeted Panel Gene	Adult HGBCL, NOS
BCL-81	chr12	56255850	56255850	G	T	exonic	ANKRD52	11	2	0.1538	Missense_Mutation	p.S132R	Non-Targeted Panel Gene	Adult HGBCL, NOS
BCL-81	chr17	7897985	7897985	G	A	exonic	CHD3	11	2	0.1538	Missense_Mutation	p.G645E	Non-Targeted Panel Gene	Adult HGBCL, NOS
BCL-81	chr9	137878129	137878129	C	A	exonic	CACNA1B	134	25	0.1572	Missense_Mutation	p.Q66K	Non-Targeted Panel Gene	Adult HGBCL, NOS
BCL-81	chr19	43625916	43625916	G	C	exonic	CADM4	21	4	0.16	Missense_Mutation	p.N250K	Non-Targeted Panel Gene	Adult HGBCL, NOS
BCL-81	chr19	43613273	43613273	G	C	exonic	SRRM5	26	5	0.1613	Missense_Mutation	p.R384S	Non-Targeted Panel Gene	Adult HGBCL, NOS
BCL-81	chr20	48989308	48989308	C	T	exonic	ARFGEF2	10	2	0.1667	Missense_Mutation	p.R853W	Non-Targeted Panel Gene	Adult HGBCL, NOS
BCL-81	chr1	223616096	223616096	A	T	exonic	CAPN8	10	2	0.1667	Missense_Mutation	p.D395E	Non-Targeted Panel Gene	Adult HGBCL, NOS
BCL-81	chrX	71603779	71603779	G	A	exonic	GCNA	45	9	0.1667	Missense_Mutation	p.A168T	Non-Targeted Panel Gene	Adult HGBCL, NOS
BCL-81	chr3	195760893	195760893	C	T	exonic	MUC4	10	2	0.1667	Missense_Mutation	p.A4227T	Non-Targeted Panel Gene	Adult HGBCL, NOS
BCL-81	chr17	56895947	56895947	C	T	exonic	TRIM25	10	2	0.1667	Missense_Mutation	p.E387K	Non-Targeted Panel Gene	Adult HGBCL, NOS
BCL-81	chr1	6461690	6461690	G	A	exonic	TNFRSF25	54	11	0.1692	Missense_Mutation	p.P342L	Non-Targeted Panel Gene	Adult HGBCL, NOS
BCL-81	chr3	197140171	197140171	C	G	exonic	DLG1	24	5	0.1724	Missense_Mutation	p.G261R	Non-Targeted Panel Gene	Adult HGBCL, NOS
BCL-81	chrX	96957891	96957891	G	T	exonic	DIAPH2	19	4	0.1739	Missense_Mutation	p.V560L	Non-Targeted Panel Gene	Adult HGBCL, NOS
BCL-81	chr12	100263531	100263531	T	G	exonic	DEPDC4	14	3	0.1765	Missense_Mutation	p.K174Q	Non-Targeted Panel Gene	Adult HGBCL, NOS
BCL-81	chr11	60941155	60941155	T	C	exonic	SLC15A3	14	3	0.1765	Missense_Mutation	p.M415V	Non-Targeted Panel Gene	Adult HGBCL, NOS
BCL-81	chr4	3317620	3317620	C	T	exonic	RGS12	106	23	0.1783	Missense_Mutation	p.P484S	Non-Targeted Panel Gene	Adult HGBCL, NOS
BCL-81	chr12	75207931	75207931	C	A	exonic	KCNC2	46	10	0.1786	Missense_Mutation	p.R18L	Non-Targeted Panel Gene	Adult HGBCL, NOS
BCL-81	chr4	15533303	15533303	C	A	exonic	CC2D2A	9	2	0.1818	Missense_Mutation	p.T526K	Non-Targeted Panel Gene	Adult HGBCL, NOS
BCL-81	chr4	114870937	114870937	A	T	exonic	NDST4	9	2	0.1818	Missense_Mutation	p.M517K	Non-Targeted Panel Gene	Adult HGBCL, NOS
BCL-81	chr17	58544132	58544132	A	T	exonic	C17orf47	26	6	0.1875	Missense_Mutation	p.S19T	Non-Targeted Panel Gene	Adult HGBCL, NOS
BCL-81	chr5	84060466	84060466	C	A	exonic	EDIL3	13	3	0.1875	Missense_Mutation	p.G324V	Non-Targeted Panel Gene	Adult HGBCL, NOS
BCL-81	chr17	74882578	74882578	A	G	exonic	FADS6	13	3	0.1875	Missense_Mutation	p.F182L	Non-Targeted Panel Gene	Adult HGBCL, NOS
BCL-81	chrX	107927041	107927041	C	T	exonic	MID2	41	10	0.1961	Missense_Mutation	p.P696S	Non-Targeted Panel Gene	Adult HGBCL, NOS
BCL-81	chr20	35463581	35463581	C	A	exonic	CEP250	8	2	0.2	Missense_Mutation	p.Q65K	Non-Targeted Panel Gene	Adult HGBCL, NOS
BCL-81	chr12	32708157	32708157	A	T	exonic	DNM1L	8	2	0.2	Missense_Mutation	p.Y114F	Non-Targeted Panel Gene	Adult HGBCL, NOS
BCL-81	chr2	160367303	160367303	C	A	exonic	RBMS1	12	3	0.2	Missense_Mutation	p.G55V	Non-Targeted Panel Gene	Adult HGBCL, NOS
BCL-81	chr19	46920875	46920875	G	A	exonic	ARHGAP35	15	4	0.2105	Missense_Mutation	p.D734N	Non-Targeted Panel Gene	Adult HGBCL, NOS
BCL-81	chr19	39974383	39974383	G	A	exonic	PSMC4	28	8	0.2162	Missense_Mutation	p.A107T	Non-Targeted Panel Gene	Adult HGBCL, NOS
BCL-81	chr10	122631107	122631107	T	C	exonic	DMBT1	18	5	0.2174	Missense_Mutation	p.C1929R	Non-Targeted Panel Gene	Adult HGBCL, NOS
BCL-81	chr7	4860876	4860876	T	C	exonic	PAPOLB	36	10	0.2174	Missense_Mutation	p.H312R	Non-Targeted Panel Gene	Adult HGBCL, NOS
BCL-81	chr1	151535130	151535130	A	G	exonic	CGN	7	2	0.2222	Missense_Mutation	p.Q998R	Non-Targeted Panel Gene	Adult HGBCL, NOS
BCL-81	chr7	137015220	137015220	T	G	exonic	CHRM2	21	6	0.2222	Missense_Mutation	p.F119V	Non-Targeted Panel Gene	Adult HGBCL, NOS
BCL-81	chr5	38406884	38406884	G	T	exonic	EGFLAM	14	4	0.2222	Missense_Mutation	p.K295N	Non-Targeted Panel Gene	Adult HGBCL, NOS
BCL-81	chr8	105802450	105802450	A	G	exonic	ZFPM2	17	5	0.2273	Missense_Mutation	p.I790V	Non-Targeted Panel Gene	Adult HGBCL, NOS
BCL-81	chr20	44160305	44160305	G	A	exonic	JPH2	27	8	0.2286	Missense_Mutation	p.T161M	Non-Targeted Panel Gene	Adult HGBCL, NOS
BCL-81	chrX	18216559	18216559	C	T	exonic	BEND2	10	3	0.2308	Missense_Mutation	p.G67D	Non-Targeted Panel Gene	Adult HGBCL, NOS
BCL-81	chr13	20988285	20988285	C	T	exonic	LATS2	60	18	0.2308	Missense_Mutation	p.A499T	Non-Targeted Panel Gene	Adult HGBCL, NOS
BCL-81	chr16	57041992	57041992	A	T	exonic	NLRC5	20	6	0.2308	Missense_Mutation	p.N1014Y	Non-Targeted Panel Gene	Adult HGBCL, NOS
BCL-81	chr1	10347802	10347802	G	A	exonic	KIF1B	6	2	0.25	Missense_Mutation	p.C1234Y	Non-Targeted Panel Gene	Adult HGBCL, NOS
BCL-81	chr17	31522006	31522006	C	T	exonic	RAB11FIP4	21	7	0.25	Missense_Mutation	p.L284F	Non-Targeted Panel Gene	Adult HGBCL, NOS
BCL-81	chr8	87873167	87873167	G	A	exonic	DCAF4L2	20	7	0.2593	Missense_Mutation	p.H269Y	Non-Targeted Panel Gene	Adult HGBCL, NOS
BCL-81	chr16	57026405	57026405	G	C	exonic	NLRC5	37	13	0.26	Missense_Mutation	p.G488R	Non-Targeted Panel Gene	Adult HGBCL, NOS
BCL-81	chr11	22227461	22227461	C	G	exonic	ANO5	14	5	0.2632	Missense_Mutation	p.L175V	Non-Targeted Panel Gene	Adult HGBCL, NOS
BCL-81	chr12	107710483	107710483	G	A	exonic	PWP1	8	3	0.2727	Missense_Mutation	p.V457I	Non-Targeted Panel Gene	Adult HGBCL, NOS
BCL-81	chrX	155514192	155514192	T	A	exonic	TMLHE	13	5	0.2778	Missense_Mutation	p.K144N	Non-Targeted Panel Gene	Adult HGBCL, NOS
BCL-81	chr16	3290137	3290137	G	A	exonic	ZNF263	18	7	0.28	Missense_Mutation	p.R544K	Non-Targeted Panel Gene	Adult HGBCL, NOS
BCL-81	chr6	38935614	38935614	A	G	exonic	DNAH8	10	4	0.2857	Missense_Mutation	p.K3827R	Non-Targeted Panel Gene	Adult HGBCL, NOS
BCL-81	chr10	34814971	34814971	G	A	exonic	PARD3	40	16	0.2857	Missense_Mutation	p.R9W	Non-Targeted Panel Gene	Adult HGBCL, NOS
BCL-81	chr2	127986861	127986861	T	C	exonic	SAP130	10	4	0.2857	Missense_Mutation	p.S654G	Non-Targeted Panel Gene	Adult HGBCL, NOS
BCL-81	chr2	127986863	127986863	T	C	exonic	SAP130	10	4	0.2857	Missense_Mutation	p.Q653R	Non-Targeted Panel Gene	Adult HGBCL, NOS
BCL-81	chr1	999751	999751	G	A	exonic	HES4	75	32	0.2991	Missense_Mutation	p.R49C	Non-Targeted Panel Gene	Adult HGBCL, NOS
BCL-81	chr18	62349815	62349815	A	T	exonic	TNFRSF11A	9	4	0.3077	Missense_Mutation	p.K54M	Non-Targeted Panel Gene	Adult HGBCL, NOS
BCL-81	chr5	40981451	40981451	A	T	exonic	C7	34	16	0.3137	Missense_Mutation	p.I804F	Non-Targeted Panel Gene	Adult HGBCL, NOS
BCL-81	chr7	99450724	99450724	C	A	exonic	CPSF4	16	8	0.3333	Missense_Mutation	p.H142Q	Non-Targeted Panel Gene	Adult HGBCL, NOS
BCL-81	chr19	51573348	51573348	T	G	exonic	ZNF175	14	7	0.3333	Missense_Mutation	p.L7V	Non-Targeted Panel Gene	Adult HGBCL, NOS
BCL-81	chrX	108734613	108734613	C	A	exonic	IRS4	32	17	0.3469	Missense_Mutation	p.D578Y	Non-Targeted Panel Gene	Adult HGBCL, NOS

BCL-81	chr2	120988837	120988837	C	T	exonic	GLI2	28	15	0.3488	Missense_Mutation	p.R975W	Non-Targeted Panel Gene	Adult HGBCL, NOS
BCL-81	chr5	16067755	16067755	A	C	exonic	MARCHF11	13	7	0.35	Missense_Mutation	p.F309V	Non-Targeted Panel Gene	Adult HGBCL, NOS
BCL-81	chr14	29597553	29597553	T	A	exonic	PRKD1	11	6	0.3529	Missense_Mutation	p.D703V	Non-Targeted Panel Gene	Adult HGBCL, NOS
BCL-81	chr17	78121152	78121152	C	G	exonic	TMC6	20	11	0.3548	Missense_Mutation	p.A466P	Non-Targeted Panel Gene	Adult HGBCL, NOS
BCL-81	chr1	152356099	152356099	C	T	exonic	FLG2	65	36	0.3564	Missense_Mutation	p.E563K	Non-Targeted Panel Gene	Adult HGBCL, NOS
BCL-81	chr1	155881500	155881500	G	A	exonic	SYT11	14	8	0.3636	Missense_Mutation	p.E430K	Non-Targeted Panel Gene	Adult HGBCL, NOS
BCL-81	chr3	31981160	31981160	C	T	exonic	OSBPL10	36	22	0.3729	Missense_Mutation	p.G7D	Non-Targeted Panel Gene	Adult HGBCL, NOS
BCL-81	chr19	57538221	57538221	A	G	exonic	ZNF549	10	6	0.375	Missense_Mutation	p.D406G	Non-Targeted Panel Gene	Adult HGBCL, NOS
BCL-81	chr8	41646499	41646499	T	G	exonic	NKX6-3	43	26	0.3768	Missense_Mutation	p.K120Q	Non-Targeted Panel Gene	Adult HGBCL, NOS
BCL-81	chr2	202532674	202532674	G	A	exonic	BMPR2	16	10	0.3846	Missense_Mutation	p.M406I	Non-Targeted Panel Gene	Adult HGBCL, NOS
BCL-81	chr21	46132050	46132050	G	A	exonic	COL6A2	54	34	0.3864	Missense_Mutation	p.R853Q	Non-Targeted Panel Gene	Adult HGBCL, NOS
BCL-81	chrX	83873732	83873732	G	T	exonic	CYLC1	9	6	0.4	Missense_Mutation	p.D342Y	Non-Targeted Panel Gene	Adult HGBCL, NOS
BCL-81	chrX	84145504	84145504	C	A	exonic	RPS6KA6	9	6	0.4	Missense_Mutation	p.D159Y	Non-Targeted Panel Gene	Adult HGBCL, NOS
BCL-81	chr17	82661030	82661030	C	G	exonic	RAB40B	10	7	0.4118	Missense_Mutation	p.G74A	Non-Targeted Panel Gene	Adult HGBCL, NOS
BCL-81	chr1	75761293	75761293	G	A	exonic	ACADM	7	5	0.4167	Missense_Mutation	p.V406M	Non-Targeted Panel Gene	Adult HGBCL, NOS
BCL-81	chr2	23926649	23926649	G	C	exonic	ATAD2B	60	48	0.4444	Missense_Mutation	p.S41C	Non-Targeted Panel Gene	Adult HGBCL, NOS
BCL-81	chr12	125076508	125076508	A	C	exonic	AACS	12	10	0.4545	Missense_Mutation	p.K85N	Non-Targeted Panel Gene	Adult HGBCL, NOS
BCL-81	chr17	9612437	9612437	C	A	exonic	CFAP52	7	6	0.4615	Missense_Mutation	p.A328E	Non-Targeted Panel Gene	Adult HGBCL, NOS
BCL-81	chr5	159073448	159073448	T	C	exonic	EBF1	8	7	0.4667	Missense_Mutation	p.K168E	Non-Targeted Panel Gene	Adult HGBCL, NOS
BCL-81	chr17	11705034	11705034	G	A	exonic	DNAH9	11	10	0.4762	Missense_Mutation	p.A1801T	Non-Targeted Panel Gene	Adult HGBCL, NOS
BCL-81	chr17	50114225	50114225	A	G	exonic	SAMD14	14	15	0.5172	Missense_Mutation	p.S330P	Non-Targeted Panel Gene	Adult HGBCL, NOS
BCL-81	chr19	54058386	54058386	C	T	exonic	VSTM1	11	12	0.5217	Missense_Mutation	p.G92E	Non-Targeted Panel Gene	Adult HGBCL, NOS
BCL-81	chr13	49248921	49248921	T	C	exonic	CDADC1	9	10	0.5263	Missense_Mutation	p.W45R	Non-Targeted Panel Gene	Adult HGBCL, NOS
BCL-81	chr7	74060035	74060035	G	C	exonic	ELN	18	20	0.5263	Missense_Mutation	p.G551R	Non-Targeted Panel Gene	Adult HGBCL, NOS
BCL-81	chr2	3575656	3575656	C	T	exonic	RPS7	23	26	0.5306	Missense_Mutation	p.P16L	Non-Targeted Panel Gene	Adult HGBCL, NOS
BCL-81	chr4	103719562	103719562	C	A	exonic	TACR3	75	86	0.5342	Missense_Mutation	p.E38D	Non-Targeted Panel Gene	Adult HGBCL, NOS
BCL-81	chr15	34920417	34920417	T	A	exonic	AQR	4	6	0.6	Missense_Mutation	p.Q379L	Non-Targeted Panel Gene	Adult HGBCL, NOS
BCL-81	chr9	130373118	130373118	G	A	exonic	HMCN2	6	9	0.6	Missense_Mutation	p.V2478I	Non-Targeted Panel Gene	Adult HGBCL, NOS
BCL-81	chr16	29813104	29813104	G	A	exonic	PRRT2	11	18	0.6207	Missense_Mutation	p.S17N	Non-Targeted Panel Gene	Adult HGBCL, NOS
BCL-81	chr14	20118083	20118083	T	C	exonic	OR4K17	6	10	0.625	Missense_Mutation	p.V226A	Non-Targeted Panel Gene	Adult HGBCL, NOS
BCL-81	chr7	99397901	99397901	G	A	exonic	PDAP1	9	17	0.6538	Missense_Mutation	p.R150W	Non-Targeted Panel Gene	Adult HGBCL, NOS
BCL-81	chr1	66372413	66372413	G	A	exonic	PDE4B	6	12	0.6667	Missense_Mutation	p.R649K	Non-Targeted Panel Gene	Adult HGBCL, NOS
BCL-81	chr1	119623045	119623045	C	G	exonic	ZNF697	17	53	0.7571	Missense_Mutation	p.G433A	Non-Targeted Panel Gene	Adult HGBCL, NOS
BCL-81	chr5	90706355	90706355	A	C	exonic	ADGRV1	2	7	0.7778	Missense_Mutation	p.E2897D	Non-Targeted Panel Gene	Adult HGBCL, NOS
BCL-81	chr2	196156626	196156626	A	G	exonic	STK17B	2	8	0.8	Missense_Mutation	p.C50R	Non-Targeted Panel Gene	Adult HGBCL, NOS
BCL-81	chr20	41163444	41163444	C	T	exonic	PLCG1	13	2	0.1333	Nonsense_Mutation	p.R286X	Targerted Panel Gene	Adult HGBCL, NOS
BCL-81	chr2	136114928	136114928	G	A	exonic	CXCR4	15	5	0.25	Nonsense_Mutation	p.R367X	Targerted Panel Gene	Adult HGBCL, NOS
BCL-81	chr1	116544581	116544581	G	A	exonic	CD58	4	5	0.5556	Nonsense_Mutation	p.Q32X	Targerted Panel Gene	Adult HGBCL, NOS
BCL-81	chr22	27796799	27796799	C	A	exonic	MN1	35	3	0.0789	Nonsense_Mutation	p.E1249X	Non-Targeted Panel Gene	Adult HGBCL, NOS
BCL-81	chr3	156678526	156678526	A	T	exonic	TIPARP	17	2	0.1053	Nonsense_Mutation	p.K277X	Non-Targeted Panel Gene	Adult HGBCL, NOS
BCL-81	chr12	96003031	96003031	C	A	exonic	LTA4H	14	2	0.125	Nonsense_Mutation	p.G498X	Non-Targeted Panel Gene	Adult HGBCL, NOS
BCL-81	chr11	6616486	6616486	C	A	exonic	TPP1	20	3	0.1304	Nonsense_Mutation	p.E302X	Non-Targeted Panel Gene	Adult HGBCL, NOS
BCL-81	chr12	536267	536267	G	A	exonic	B4GALNT3	13	2	0.1333	Nonsense_Mutation	p.W108X	Non-Targeted Panel Gene	Adult HGBCL, NOS
BCL-81	chr9	37763746	37763746	C	A	exonic	TRMT10B	23	4	0.1481	Nonsense_Mutation	p.S138X	Non-Targeted Panel Gene	Adult HGBCL, NOS
BCL-81	chr19	58295483	58295483	G	T	exonic	ZNF8	11	2	0.1538	Nonsense_Mutation	p.E559X	Non-Targeted Panel Gene	Adult HGBCL, NOS
BCL-81	chr13	48411930	48411930	G	T	exonic	LPAR6	15	3	0.1667	Nonsense_Mutation	p.S165X	Non-Targeted Panel Gene	Adult HGBCL, NOS
BCL-81	chrX	74741304	74741304	T	A	exonic	KIAA2022	9	2	0.1818	Nonsense_Mutation	p.R1085X	Non-Targeted Panel Gene	Adult HGBCL, NOS
BCL-81	chr12	123210084	123210084	A	T	exonic	MPHOSPH9	8	2	0.2	Nonsense_Mutation	p.L389X	Non-Targeted Panel Gene	Adult HGBCL, NOS
BCL-81	chr14	92809518	92809518	C	T	exonic	GOLGA5	2	7	0.7778	Nonsense_Mutation	p.R331X	Non-Targeted Panel Gene	Adult HGBCL, NOS
BCL-81	chr12	92421012	92421012	A	T	exonic	CLLU1OS	16	2	0.1111	Nonstop_Mutation	p.X102K	Non-Targeted Panel Gene	Adult HGBCL, NOS
BCL-82	chr14	30935161	30935161	A	G	splicing	STRN3	20	9	0.3103	Splice_Site	.	Non-Targeted Panel Gene	Adult HGBCL, NOS
BCL-82	chr2	137115123	137115123	G	A	splicing	THSD7B	13	6	0.3158	Splice_Site	.	Non-Targeted Panel Gene	Adult HGBCL, NOS
BCL-82	chr2	1484595	1484595	G	A	splicing	TPO	21	18	0.4615	Splice_Site	.	Non-Targeted Panel Gene	Adult HGBCL, NOS
BCL-82	chr12	11869423	11869423	G	C	splicing	ETV6	8	20	0.7143	Splice_Site	.	Non-Targeted Panel Gene	Adult HGBCL, NOS
BCL-82	chr10	89013348	89013349	AG	-	exonic	FAS	23	7	0.2333	Frame_Shift_Del	p.T219fs	Targerted Panel Gene	Adult HGBCL, NOS
BCL-82	chr1	23559232	23559236	GCTAA	-	exonic	ID3	233	182	0.4386	Frame_Shift_Del	p.L64fs	Targerted Panel Gene	Adult HGBCL, NOS
BCL-82	chr12	132621878	132621888	TCTCTGCCCCT	-	exonic	P2RX2	80	40	0.3333	Frame_Shift_Del	p.I441fs	Non-Targeted Panel Gene	Adult HGBCL, NOS
BCL-82	chr3	49660806	49660807	AG	-	exonic	BSN	39	24	0.381	Frame_Shift_Del	p.K2987fs	Non-Targeted Panel Gene	Adult HGBCL, NOS
BCL-82	chr17	48037710	48037710	C	-	exonic	COPZ2	109	86	0.4388	Frame_Shift_Del	p.G23fs	Non-Targeted Panel Gene	Adult HGBCL, NOS
BCL-82	chr10	79946526	79946527	CT	-	exonic	SFTPD	13	11	0.4583	Frame_Shift_Del	p.S45fs	Non-Targeted Panel Gene	Adult HGBCL, NOS
BCL-82	chr8	112666394	112666394	-	A	exonic	CSMD3	7	4	0.3636	Frame_Shift_Ins	p.S860fs	Targerted Panel Gene	Adult HGBCL, NOS
BCL-82	chr6	129313052	129313052	-	A	exonic	LAMA2	16	4	0.2	Frame_Shift_Ins	p.T1122fs	Non-Targeted Panel Gene	Adult HGBCL, NOS
BCL-82	chr7	99666680	99666680	-	G	exonic	CYP3A5	18	5	0.2174	Frame_Shift_Ins	p.I138fs	Non-Targeted Panel Gene	Adult HGBCL, NOS
BCL-82	chr11	66744820	66744822	GGC	-	exonic	C11orf80	320	17	0.05	In_Frame_Del	p.26_27del	Non-Targeted Panel Gene	Adult HGBCL, NOS
BCL-82	chr19	5867629	5867631	AGG	-	exonic	FUT5	42	8	0.16	In_Frame_Del	p.32_33del	Non-Targeted Panel Gene	Adult HGBCL, NOS
BCL-82	chr5	150656743	150656748	CCGCCC	-	exonic	SYNPO	70	17	0.1954	In_Frame_Del	p.790_791del	Non-Targeted Panel Gene	Adult HGBCL, NOS
BCL-82	chr2	74464556	74464558	GAC	-	exonic	MOGS	37	25	0.4032	In_Frame_Del	p.67_67del	Non-Targeted Panel Gene	Adult HGBCL, NOS
BCL-82	chrX	17375962	17375964	CCG	-	exonic	NHS	5	39	0.8864	In_Frame_Del	p.69_69del	Non-Targeted Panel Gene	Adult HGBCL, NOS
BCL-82	chr10	117134320	117134320	-	GCGGCGGCT	exonic	VAX1	97	37	0.2517	In_Frame_Ins	p.A231delinsAAAA	Non-Targeted Panel Gene	Adult HGBCL, NOS
BCL-82	chr13	27924562	27924562	-	GCC	exonic	PDX1	63	51	0.4397	In_Frame_Ins	p.L238delinsLP	Non-Targeted Panel Gene	Adult HGBCL, NOS
BCL-82	chr19	23361261	23361261	A	G	exonic	ZNF91	78	4	0.0488	Missense_Mutation	p.L573P	Targerted Panel Gene	Adult HGBCL, NOS
BCL-82	chr19	23361479	23361479	C	G	exonic	ZNF91	93	5	0.051	Missense_Mutation	p.R500S	Targerted Panel Gene	Adult HGBCL, NOS
BCL-82	chr19	24127130	24127130	G	C	exonic	ZNF254	56	4	0.0667	Missense_Mutation	p.R336P	Targerted Panel Gene	Adult HGBCL, NOS
BCL-82	chr19	23361838	23361838	T	C	exonic	ZNF91	84	6	0.0667	Missense_Mutation	p.K381E	Targerted Panel Gene	Adult HGBCL, NOS
BCL-82	chr10	128104952	128104952	T	A	exonic	MKI67	57	8	0.1231	Missense_Mutation	p.L2296F	Targerted Panel Gene	Adult HGBCL, NOS
BCL-82	chr6	27146476	27146476	G	A	exonic	HIST1H2BK	243	71	0.2261	Missense_Mutation	p.A108V	Targerted Panel Gene	Adult HGBCL, NOS
BCL-82	chr1	26766493	26766493	A	G	exonic	ARID1A	15	5	0.2381	Missense_Mutation	p.D972G	Targerted Panel Gene	Adult HGBCL, NOS
BCL-82	chr6	394888	394888	C	A	exonic	IRF4	25	11	0.3056	Missense_Mutation	p.T95K	Targerted Panel Gene	Adult HGBCL, NOS
BCL-82	chr3	183555373	183555373	C	G	exonic	KLHL6	60	29	0.3258	Missense_Mutation	p.S94T	Targerted Panel Gene	Adult HGBCL, NOS
BCL-82	chr2	214934104	214934104	A	C	exonic	ABCA12	10	5	0.3333	Missense_Mutation	p.L2552V	Targerted Panel Gene	Adult HGBCL, NOS
BCL-82	chr16	2080189	2080189	C	T	exonic	TSC2	42	21	0.3333	Missense_Mutation	p.A1141V	Targerted Panel Gene	Adult HGBCL, NOS
BCL-82	chr2	27231542	27231542	G	A	exonic	CAD	21	11	0.3438	Missense_Mutation	p.V788M	Targerted Panel Gene	Adult HGBCL, NOS

BCL-82	chr7	2938031	2938031	T	G	exonic	CARD11	53	28	0.3457	Missense_Mutation	p.Y340S	Targerted Panel Gene	Adult HGBCL, NOS
BCL-82	chr19	24127814	24127814	A	C	exonic	ZNF254	31	19	0.38	Missense_Mutation	p.E564A	Targerted Panel Gene	Adult HGBCL, NOS
BCL-82	chr6	117357857	117357857	C	A	exonic	ROS1	8	5	0.3846	Missense_Mutation	p.K1267N	Targerted Panel Gene	Adult HGBCL, NOS
BCL-82	chr3	177038385	177038385	A	C	exonic	TBL1XR1	23	16	0.4103	Missense_Mutation	p.C325W	Targerted Panel Gene	Adult HGBCL, NOS
BCL-82	chr20	58853765	58853765	A	G	exonic	GNAS	37	32	0.4638	Missense_Mutation	p.D167G	Targerted Panel Gene	Adult HGBCL, NOS
BCL-82	chr12	49031704	49031704	G	A	exonic	KMT2D	12	12	0.5	Missense_Mutation	p.A4334V	Targerted Panel Gene	Adult HGBCL, NOS
BCL-82	chr19	41879574	41879574	C	A	exonic	CD79A	44	46	0.5111	Missense_Mutation	p.T102N	Targerted Panel Gene	Adult HGBCL, NOS
BCL-82	chr12	49022087	49022087	T	C	exonic	KMT2D	11	14	0.56	Missense_Mutation	p.K5493E	Targerted Panel Gene	Adult HGBCL, NOS
BCL-82	chr7	2917352	2917352	T	C	exonic	CARD11	9	12	0.5714	Missense_Mutation	p.S881G	Targerted Panel Gene	Adult HGBCL, NOS
BCL-82	chr12	49022085	49022085	T	G	exonic	KMT2D	11	15	0.5769	Missense_Mutation	p.K5493N	Targerted Panel Gene	Adult HGBCL, NOS
BCL-82	chr15	44711581	44711581	T	C	exonic	B2M	82	115	0.5838	Missense_Mutation	p.L12P	Targerted Panel Gene	Adult HGBCL, NOS
BCL-82	chrX	53604784	53604784	G	T	exonic	HUWE1	4	10	0.7143	Missense_Mutation	p.D849E	Targerted Panel Gene	Adult HGBCL, NOS
BCL-82	chr5	140796453	140796453	C	T	exonic	PCDHA2	156	13	0.0769	Missense_Mutation	p.R497W	Non-Targeted Panel Gene	Adult HGBCL, NOS
BCL-82	chr19	53069049	53069049	T	G	exonic	ZNF160	48	4	0.0769	Missense_Mutation	p.Q459H	Non-Targeted Panel Gene	Adult HGBCL, NOS
BCL-82	chr19	36877183	36877183	T	A	exonic	ZNF345	35	3	0.0789	Missense_Mutation	p.F118Y	Non-Targeted Panel Gene	Adult HGBCL, NOS
BCL-82	chr12	2801255	2801255	A	G	exonic	FKBP4	22	2	0.0833	Missense_Mutation	p.T391A	Non-Targeted Panel Gene	Adult HGBCL, NOS
BCL-82	chr19	20197599	20197599	A	G	exonic	ZNF486	43	4	0.0833	Missense_Mutation	p.K297E	Non-Targeted Panel Gene	Adult HGBCL, NOS
BCL-82	chr19	53381606	53381606	G	A	exonic	ZNF525	55	5	0.0833	Missense_Mutation	p.A307T	Non-Targeted Panel Gene	Adult HGBCL, NOS
BCL-82	chr19	52582369	52582369	A	G	exonic	ZNF701	43	4	0.0851	Missense_Mutation	p.N170D	Non-Targeted Panel Gene	Adult HGBCL, NOS
BCL-82	chr19	20949634	20949634	A	G	exonic	ZNF85	42	4	0.087	Missense_Mutation	p.K404E	Non-Targeted Panel Gene	Adult HGBCL, NOS
BCL-82	chr19	11949256	11949256	T	C	exonic	ZNF700	62	6	0.0882	Missense_Mutation	p.I411T	Non-Targeted Panel Gene	Adult HGBCL, NOS
BCL-82	chr2	165389723	165389723	C	G	exonic	SCN2A	18	2	0.1	Missense_Mutation	p.P1973A	Non-Targeted Panel Gene	Adult HGBCL, NOS
BCL-82	chr1	167126395	167126395	G	A	exonic	DUSP27	135	16	0.106	Missense_Mutation	p.D422N	Non-Targeted Panel Gene	Adult HGBCL, NOS
BCL-82	chr19	20197609	20197609	A	G	exonic	ZNF486	41	5	0.1087	Missense_Mutation	p.D300G	Non-Targeted Panel Gene	Adult HGBCL, NOS
BCL-82	chr19	21294034	21294034	C	T	exonic	ZNF708	79	10	0.1124	Missense_Mutation	p.G311E	Non-Targeted Panel Gene	Adult HGBCL, NOS
BCL-82	chr7	64978672	64978672	C	G	exonic	ERV3-1-ZNF117;ZN	52	7	0.1186	Missense_Mutation	p.S300T	Non-Targeted Panel Gene	Adult HGBCL, NOS
BCL-82	chr19	53352240	53352240	T	C	exonic	ZNF845	56	8	0.125	Missense_Mutation	p.L522P	Non-Targeted Panel Gene	Adult HGBCL, NOS
BCL-82	chr2	217818514	217818514	G	T	exonic	TNS1	26	4	0.1333	Missense_Mutation	p.A1169D	Non-Targeted Panel Gene	Adult HGBCL, NOS
BCL-82	chr7	64832089	64832089	C	G	exonic	ZNF138	25	4	0.1379	Missense_Mutation	p.Q283E	Non-Targeted Panel Gene	Adult HGBCL, NOS
BCL-82	chr11	57796910	57796910	G	C	exonic	CTNND1	18	4	0.1818	Missense_Mutation	p.D292H	Non-Targeted Panel Gene	Adult HGBCL, NOS
BCL-82	chr6	41090222	41090222	G	C	exonic	NFYA	9	2	0.1818	Missense_Mutation	p.V154L	Non-Targeted Panel Gene	Adult HGBCL, NOS
BCL-82	chr17	7043518	7043518	G	A	exonic	SLC16A11	414	102	0.1977	Missense_Mutation	p.T23M	Non-Targeted Panel Gene	Adult HGBCL, NOS
BCL-82	chr7	33109264	33109264	G	T	exonic	RP9	74	20	0.2128	Missense_Mutation	p.H37N	Non-Targeted Panel Gene	Adult HGBCL, NOS
BCL-82	chr11	94864785	94864785	C	T	exonic	AMOTL1	13	4	0.2353	Missense_Mutation	p.S729L	Non-Targeted Panel Gene	Adult HGBCL, NOS
BCL-82	chr3	121716917	121716917	C	T	exonic	GOLGB1	25	8	0.2353	Missense_Mutation	p.A365T	Non-Targeted Panel Gene	Adult HGBCL, NOS
BCL-82	chr4	6863341	6863341	A	G	exonic	KIAA0232	13	4	0.2353	Missense_Mutation	p.R987G	Non-Targeted Panel Gene	Adult HGBCL, NOS
BCL-82	chr17	28919215	28919215	A	T	exonic	PHF12	24	8	0.25	Missense_Mutation	p.D299E	Non-Targeted Panel Gene	Adult HGBCL, NOS
BCL-82	chr7	83961562	83961562	G	A	exonic	SEMA3A	14	5	0.2632	Missense_Mutation	p.L709F	Non-Targeted Panel Gene	Adult HGBCL, NOS
BCL-82	chr1	66682130	66682130	G	T	exonic	SGIP1	14	5	0.2632	Missense_Mutation	p.G359V	Non-Targeted Panel Gene	Adult HGBCL, NOS
BCL-82	chr20	35000589	35000589	A	T	exonic	MYH7B	109	39	0.2635	Missense_Mutation	p.E1735V	Non-Targeted Panel Gene	Adult HGBCL, NOS
BCL-82	chr4	17709488	17709488	G	A	exonic	FAM184B	25	9	0.2647	Missense_Mutation	p.R100C	Non-Targeted Panel Gene	Adult HGBCL, NOS
BCL-82	chr19	40236264	40236264	G	A	exonic	AKT2	16	6	0.2727	Missense_Mutation	p.A318V	Non-Targeted Panel Gene	Adult HGBCL, NOS
BCL-82	chr4	13602014	13602014	T	G	exonic	BOD1L1	40	15	0.2727	Missense_Mutation	p.D1629A	Non-Targeted Panel Gene	Adult HGBCL, NOS
BCL-82	chr7	120275485	120275485	G	A	exonic	KCND2	21	8	0.2759	Missense_Mutation	p.V285I	Non-Targeted Panel Gene	Adult HGBCL, NOS
BCL-82	chr1	6625258	6625258	T	G	exonic	THAP3	118	47	0.2831	Missense_Mutation	p.Y14D	Non-Targeted Panel Gene	Adult HGBCL, NOS
BCL-82	chr1	70038408	70038408	G	C	exonic	LRRC7	10	4	0.2857	Missense_Mutation	p.E829Q	Non-Targeted Panel Gene	Adult HGBCL, NOS
BCL-82	chr7	905089	905089	C	G	exonic	ADAP1	61	25	0.2907	Missense_Mutation	p.G158R	Non-Targeted Panel Gene	Adult HGBCL, NOS
BCL-82	chr18	7080375	7080375	C	T	exonic	LAMA1	16	7	0.3043	Missense_Mutation	p.M48I	Non-Targeted Panel Gene	Adult HGBCL, NOS
BCL-82	chr5	179079980	179079980	A	T	exonic	ZNF354C	18	8	0.3077	Missense_Mutation	p.K516N	Non-Targeted Panel Gene	Adult HGBCL, NOS
BCL-82	chr4	184631079	184631079	G	T	exonic	CASP3	22	10	0.3125	Missense_Mutation	p.P188T	Non-Targeted Panel Gene	Adult HGBCL, NOS
BCL-82	chr3	51662515	51662515	C	T	exonic	RAD54L2	34	16	0.32	Missense_Mutation	p.P1167S	Non-Targeted Panel Gene	Adult HGBCL, NOS
BCL-82	chr14	69485096	69485096	C	T	exonic	PLEKHD1	105	50	0.3226	Missense_Mutation	p.S44L	Non-Targeted Panel Gene	Adult HGBCL, NOS
BCL-82	chr8	38146364	38146364	C	A	exonic	STAR	23	11	0.3235	Missense_Mutation	p.M130I	Non-Targeted Panel Gene	Adult HGBCL, NOS
BCL-82	chr17	82750525	82750525	G	A	exonic	FN3K	62	30	0.3261	Missense_Mutation	p.D234N	Non-Targeted Panel Gene	Adult HGBCL, NOS
BCL-82	chr1	152109438	152109438	T	G	exonic	TCHH	45	22	0.3284	Missense_Mutation	p.D1260A	Non-Targeted Panel Gene	Adult HGBCL, NOS
BCL-82	chr7	16421076	16421076	C	A	exonic	ISPD	343	170	0.3307	Missense_Mutation	p.A83S	Non-Targeted Panel Gene	Adult HGBCL, NOS
BCL-82	chr5	90683668	90683668	C	T	exonic	ADGRV1	16	8	0.3333	Missense_Mutation	p.T1916I	Non-Targeted Panel Gene	Adult HGBCL, NOS
BCL-82	chr13	66304780	66304780	G	T	exonic	PCDH9	14	7	0.3333	Missense_Mutation	p.Q1197K	Non-Targeted Panel Gene	Adult HGBCL, NOS
BCL-82	chr11	8168899	8168899	C	T	exonic	RIC3	50	25	0.3333	Missense_Mutation	p.G31R	Non-Targeted Panel Gene	Adult HGBCL, NOS
BCL-82	chr19	16325217	16325217	G	A	exonic	KLF2	370	186	0.3345	Missense_Mutation	p.R26H	Non-Targeted Panel Gene	Adult HGBCL, NOS
BCL-82	chr11	47349898	47349898	C	T	exonic	MYBPC3	72	37	0.3394	Missense_Mutation	p.R177H	Non-Targeted Panel Gene	Adult HGBCL, NOS
BCL-82	chr11	79069902	79069902	G	A	exonic	TENM4	106	56	0.3457	Missense_Mutation	p.R15C	Non-Targeted Panel Gene	Adult HGBCL, NOS
BCL-82	chr6	7565401	7565401	A	G	exonic	DSP	15	8	0.3478	Missense_Mutation	p.N274D	Non-Targeted Panel Gene	Adult HGBCL, NOS
BCL-82	chr3	52221872	52221872	G	A	exonic	TLR9	75	40	0.3478	Missense_Mutation	p.S815F	Non-Targeted Panel Gene	Adult HGBCL, NOS
BCL-82	chr2	184937844	184937844	A	C	exonic	ZNF804A	13	7	0.35	Missense_Mutation	p.R816S	Non-Targeted Panel Gene	Adult HGBCL, NOS
BCL-82	chr22	39661203	39661203	C	A	exonic	CACNA1I	22	12	0.3529	Missense_Mutation	p.P932T	Non-Targeted Panel Gene	Adult HGBCL, NOS
BCL-82	chr15	89667753	89667753	G	A	exonic	PLIN1	77	42	0.3529	Missense_Mutation	p.A271V	Non-Targeted Panel Gene	Adult HGBCL, NOS
BCL-82	chr11	8925688	8925688	C	A	exonic	C11orf16	25	14	0.359	Missense_Mutation	p.A327S	Non-Targeted Panel Gene	Adult HGBCL, NOS
BCL-82	chr12	76348172	76348172	G	A	exonic	BBS10	23	13	0.3611	Missense_Mutation	p.P63S	Non-Targeted Panel Gene	Adult HGBCL, NOS
BCL-82	chr14	24332535	24332535	G	C	exonic	ADCY4	70	40	0.3636	Missense_Mutation	p.A169G	Non-Targeted Panel Gene	Adult HGBCL, NOS
BCL-82	chr11	134203765	134203765	A	G	exonic	NCAPD3	14	8	0.3636	Missense_Mutation	p.C453R	Non-Targeted Panel Gene	Adult HGBCL, NOS
BCL-82	chr6	35500100	35500100	A	G	exonic	TULP1	14	8	0.3636	Missense_Mutation	p.I459T	Non-Targeted Panel Gene	Adult HGBCL, NOS
BCL-82	chr21	28879881	28879881	G	A	exonic	N6AMT1	12	7	0.3684	Missense_Mutation	p.P129S	Non-Targeted Panel Gene	Adult HGBCL, NOS
BCL-82	chr4	1718475	1718475	C	G	exonic	TMEM129	34	20	0.3704	Missense_Mutation	p.W119C	Non-Targeted Panel Gene	Adult HGBCL, NOS
BCL-82	chr16	89530702	89530702	G	A	exonic	SPG7	10	6	0.375	Missense_Mutation	p.R294H	Non-Targeted Panel Gene	Adult HGBCL, NOS
BCL-82	chr22	37621626	37621626	G	A	exonic	GGA1	26	16	0.381	Missense_Mutation	p.R107H	Non-Targeted Panel Gene	Adult HGBCL, NOS
BCL-82	chr3	113657882	113657882	G	A	exonic	USF3	13	8	0.381	Missense_Mutation	p.T1267I	Non-Targeted Panel Gene	Adult HGBCL, NOS
BCL-82	chr14	26448050	26448050	G	A	exonic	NOVA1	16	10	0.3846	Missense_Mutation	p.P478L	Non-Targeted Panel Gene	Adult HGBCL, NOS
BCL-82	chr11	36576092	36576092	A	G	exonic	RAG1	11	7	0.3889	Missense_Mutation	p.R930G	Non-Targeted Panel Gene	Adult HGBCL, NOS
BCL-82	chr2	185813581	185813581	C	A	exonic	FSIP2	17	11	0.3929	Missense_Mutation	p.P6622T	Non-Targeted Panel Gene	Adult HGBCL, NOS
BCL-82	chr10	49470676	49470676	G	C	exonic	ERCC6	20	13	0.3939	Missense_Mutation	p.P1095R	Non-Targeted Panel Gene	Adult HGBCL, NOS
BCL-82	chr19	56222030	56222030	C	A	exonic	ZSCAN5A	29	19	0.3958	Missense_Mutation	p.D346Y	Non-Targeted Panel Gene	Adult HGBCL, NOS

BCL-82	chr4	904698	904698	G	A	exonic	GAK	21	14	0.4	Missense_Mutation	p.T155M	Non-Targeted Panel Gene	Adult HGBCL, NOS
BCL-82	chr18	75285480	75285480	G	A	exonic	TSHZ1	9	6	0.4	Missense_Mutation	p.E25K	Non-Targeted Panel Gene	Adult HGBCL, NOS
BCL-82	chr16	13948175	13948175	C	A	exonic	ERCC4	10	7	0.4118	Missense_Mutation	p.A860D	Non-Targeted Panel Gene	Adult HGBCL, NOS
BCL-82	chr5	127396657	127396657	G	T	exonic	MEGF10	20	14	0.4118	Missense_Mutation	p.D180Y	Non-Targeted Panel Gene	Adult HGBCL, NOS
BCL-82	chr17	46024032	46024032	C	T	exonic	MAPT	24	17	0.4146	Missense_Mutation	p.S731L	Non-Targeted Panel Gene	Adult HGBCL, NOS
BCL-82	chr4	161455064	161455064	T	A	exonic	FSTL5	7	5	0.4167	Missense_Mutation	p.Q594L	Non-Targeted Panel Gene	Adult HGBCL, NOS
BCL-82	chr12	113207722	113207722	T	C	exonic	IQCD	28	20	0.4167	Missense_Mutation	p.K149E	Non-Targeted Panel Gene	Adult HGBCL, NOS
BCL-82	chr5	141478209	141478209	T	A	exonic	PCDHGC3	33	24	0.4211	Missense_Mutation	p.L698Q	Non-Targeted Panel Gene	Adult HGBCL, NOS
BCL-82	chr4	141132582	141132582	C	T	exonic	RNF150	222	165	0.4264	Missense_Mutation	p.R76H	Non-Targeted Panel Gene	Adult HGBCL, NOS
BCL-82	chr5	90692732	90692732	G	A	exonic	ADGRV1	8	6	0.4286	Missense_Mutation	p.R2360H	Non-Targeted Panel Gene	Adult HGBCL, NOS
BCL-82	chr4	76355366	76355366	G	C	exonic	CCDC158	12	9	0.4286	Missense_Mutation	p.S748R	Non-Targeted Panel Gene	Adult HGBCL, NOS
BCL-82	chr1	66777352	66777352	C	A	exonic	TCTEX1D1	18	14	0.4375	Missense_Mutation	p.S146R	Non-Targeted Panel Gene	Adult HGBCL, NOS
BCL-82	chr8	68331092	68331092	G	A	exonic	C8orf34	118	95	0.446	Missense_Mutation	p.R27H	Non-Targeted Panel Gene	Adult HGBCL, NOS
BCL-82	chr13	46553533	46553533	G	A	exonic	LRCH1	338	275	0.4486	Missense_Mutation	p.G46D	Non-Targeted Panel Gene	Adult HGBCL, NOS
BCL-82	chr6	138814569	138814569	G	A	exonic	ECT2L	22	19	0.4524	Missense_Mutation	p.A49T	Non-Targeted Panel Gene	Adult HGBCL, NOS
BCL-82	chr8	143816630	143816630	C	G	exonic	PUF60	59	49	0.4537	Missense_Mutation	p.E524Q	Non-Targeted Panel Gene	Adult HGBCL, NOS
BCL-82	chr2	38798372	38798372	C	T	exonic	DHX57	12	10	0.4545	Missense_Mutation	p.S1363N	Non-Targeted Panel Gene	Adult HGBCL, NOS
BCL-82	chr17	10631669	10631669	T	C	exonic	MYH3	12	10	0.4545	Missense_Mutation	p.E1743G	Non-Targeted Panel Gene	Adult HGBCL, NOS
BCL-82	chr19	46787862	46787862	G	C	exonic	SLC1A5	294	246	0.4556	Missense_Mutation	p.A35G	Non-Targeted Panel Gene	Adult HGBCL, NOS
BCL-82	chr1	40202488	40202488	C	G	exonic	RLF	19	16	0.4571	Missense_Mutation	p.I228M	Non-Targeted Panel Gene	Adult HGBCL, NOS
BCL-82	chr14	21301003	21301003	C	T	exonic	RPGRIP1	142	120	0.458	Missense_Mutation	p.R86W	Non-Targeted Panel Gene	Adult HGBCL, NOS
BCL-82	chr13	113835561	113835561	G	A	exonic	GAS6	39	33	0.4583	Missense_Mutation	p.L222F	Non-Targeted Panel Gene	Adult HGBCL, NOS
BCL-82	chr13	39600870	39600870	G	T	exonic	LHFP	13	11	0.4583	Missense_Mutation	p.T116K	Non-Targeted Panel Gene	Adult HGBCL, NOS
BCL-82	chr12	39370145	39370145	A	G	exonic	KIF21A	20	17	0.4595	Missense_Mutation	p.V54A	Non-Targeted Panel Gene	Adult HGBCL, NOS
BCL-82	chr19	10577307	10577307	G	C	exonic	AP1M2	14	12	0.4615	Missense_Mutation	p.S313C	Non-Targeted Panel Gene	Adult HGBCL, NOS
BCL-82	chr4	99948520	99948520	A	C	exonic	H2AFZ	14	12	0.4615	Missense_Mutation	p.V110G	Non-Targeted Panel Gene	Adult HGBCL, NOS
BCL-82	chr14	37680245	37680245	C	T	exonic	TTC6	7	6	0.4615	Missense_Mutation	p.A361V	Non-Targeted Panel Gene	Adult HGBCL, NOS
BCL-82	chr19	38407027	38407027	C	G	exonic	FAM98C	15	13	0.4643	Missense_Mutation	p.P290A	Non-Targeted Panel Gene	Adult HGBCL, NOS
BCL-82	chr14	88620913	88620913	T	C	exonic	EML5	8	7	0.4667	Missense_Mutation	p.K1739R	Non-Targeted Panel Gene	Adult HGBCL, NOS
BCL-82	chr5	146013973	146013973	G	T	exonic	SH3RF2	16	14	0.4667	Missense_Mutation	p.S324I	Non-Targeted Panel Gene	Adult HGBCL, NOS
BCL-82	chr20	38926911	38926911	C	G	exonic	FAM83D	160	142	0.4702	Missense_Mutation	p.R157G	Non-Targeted Panel Gene	Adult HGBCL, NOS
BCL-82	chr9	115046550	115046550	T	C	exonic	TNC	9	8	0.4706	Missense_Mutation	p.E1662G	Non-Targeted Panel Gene	Adult HGBCL, NOS
BCL-82	chr17	14206977	14206977	G	T	exonic	COX10	248	229	0.4791	Missense_Mutation	p.V366L	Non-Targeted Panel Gene	Adult HGBCL, NOS
BCL-82	chr10	60073166	60073166	A	G	exonic	ANK3	27	25	0.4808	Missense_Mutation	p.I2572T	Non-Targeted Panel Gene	Adult HGBCL, NOS
BCL-82	chr20	51004466	51004466	C	T	exonic	KCNG1	114	107	0.4842	Missense_Mutation	p.R372H	Non-Targeted Panel Gene	Adult HGBCL, NOS
BCL-82	chr19	45804347	45804347	A	G	exonic	RSPH6A	34	32	0.4848	Missense_Mutation	p.Y520H	Non-Targeted Panel Gene	Adult HGBCL, NOS
BCL-82	chr16	1392941	1392941	C	T	exonic	UNKL	17	16	0.4848	Missense_Mutation	p.D325N	Non-Targeted Panel Gene	Adult HGBCL, NOS
BCL-82	chr16	29694769	29694769	G	A	exonic	QPRT	136	134	0.4963	Missense_Mutation	p.G40D	Non-Targeted Panel Gene	Adult HGBCL, NOS
BCL-82	chr5	96896846	96896846	T	C	exonic	ERAP2	16	16	0.5	Missense_Mutation	p.W496R	Non-Targeted Panel Gene	Adult HGBCL, NOS
BCL-82	chr11	69775026	69775026	A	G	exonic	FGF4	49	49	0.5	Missense_Mutation	p.L20P	Non-Targeted Panel Gene	Adult HGBCL, NOS
BCL-82	chr6	85547548	85547548	A	C	exonic	SNX14	6	6	0.5	Missense_Mutation	p.D311E	Non-Targeted Panel Gene	Adult HGBCL, NOS
BCL-82	chr17	50538930	50538930	T	C	exonic	EPN3	20	21	0.5122	Missense_Mutation	p.L243P	Non-Targeted Panel Gene	Adult HGBCL, NOS
BCL-82	chr17	50669479	50669479	C	T	exonic	ABCC3	18	19	0.5135	Missense_Mutation	p.A731V	Non-Targeted Panel Gene	Adult HGBCL, NOS
BCL-82	chr12	120994322	120994322	C	G	exonic	HNF1A	80	89	0.5145	Missense_Mutation	p.P291R	Non-Targeted Panel Gene	Adult HGBCL, NOS
BCL-82	chr11	126273351	126273351	G	A	exonic	FOXRED1	14	15	0.5172	Missense_Mutation	p.V145I	Non-Targeted Panel Gene	Adult HGBCL, NOS
BCL-82	chr12	50887342	50887342	T	G	exonic	TMPRSS12	28	30	0.5172	Missense_Mutation	p.H292Q	Non-Targeted Panel Gene	Adult HGBCL, NOS
BCL-82	chr13	113549461	113549461	C	T	exonic	TMCO3	12	13	0.52	Missense_Mutation	p.L653F	Non-Targeted Panel Gene	Adult HGBCL, NOS
BCL-82	chr2	43574826	43574826	G	C	exonic	THADA	10	11	0.5238	Missense_Mutation	p.I413M	Non-Targeted Panel Gene	Adult HGBCL, NOS
BCL-82	chr2	237371855	237371855	C	T	exonic	COL6A3	9	10	0.5263	Missense_Mutation	p.V1388M	Non-Targeted Panel Gene	Adult HGBCL, NOS
BCL-82	chr2	222232164	222232164	G	A	exonic	PAX3	7	8	0.5333	Missense_Mutation	p.R236C	Non-Targeted Panel Gene	Adult HGBCL, NOS
BCL-82	chr21	46401669	46401669	C	T	exonic	PCNT	11	13	0.5417	Missense_Mutation	p.P1637L	Non-Targeted Panel Gene	Adult HGBCL, NOS
BCL-82	chr1	32949572	32949572	G	C	exonic	RNF19B	14	17	0.5484	Missense_Mutation	p.P280A	Non-Targeted Panel Gene	Adult HGBCL, NOS
BCL-82	chr14	64052510	64052510	C	T	exonic	SYNE2	9	11	0.55	Missense_Mutation	p.T2866M	Non-Targeted Panel Gene	Adult HGBCL, NOS
BCL-82	chr3	14164853	14164853	A	C	exonic	XPC	9	11	0.55	Missense_Mutation	p.F287C	Non-Targeted Panel Gene	Adult HGBCL, NOS
BCL-82	chr7	150957380	150957380	G	A	exonic	KCNH2	26	32	0.5517	Missense_Mutation	p.P347S	Non-Targeted Panel Gene	Adult HGBCL, NOS
BCL-82	chr14	50752602	50752602	T	G	exonic	NIN	13	18	0.5625	Missense_Mutation	p.E1622D	Non-Targeted Panel Gene	Adult HGBCL, NOS
BCL-82	chr1	65148532	65148532	A	C	exonic	AK4	39	52	0.5714	Missense_Mutation	p.E42A	Non-Targeted Panel Gene	Adult HGBCL, NOS
BCL-82	chr1	35850897	35850897	A	C	exonic	AGO4	5	7	0.5833	Missense_Mutation	p.N774T	Non-Targeted Panel Gene	Adult HGBCL, NOS
BCL-82	chr19	48724870	48724870	T	C	exonic	RASIP1	25	35	0.5833	Missense_Mutation	p.M740V	Non-Targeted Panel Gene	Adult HGBCL, NOS
BCL-82	chr5	16475100	16475100	G	C	exonic	RETREG1	12	17	0.5862	Missense_Mutation	p.Q379E	Non-Targeted Panel Gene	Adult HGBCL, NOS
BCL-82	chr15	23647360	23647360	A	G	exonic	MAGEL2	34	49	0.5904	Missense_Mutation	p.L128P	Non-Targeted Panel Gene	Adult HGBCL, NOS
BCL-82	chr5	33881280	33881280	G	C	exonic	ADAMTS12	8	12	0.6	Missense_Mutation	p.Q110E	Non-Targeted Panel Gene	Adult HGBCL, NOS
BCL-82	chr16	13935420	13935420	A	T	exonic	ERCC4	6	9	0.6	Missense_Mutation	p.Q496H	Non-Targeted Panel Gene	Adult HGBCL, NOS
BCL-82	chr3	151446847	151446847	C	A	exonic	IGSF10	8	12	0.6	Missense_Mutation	p.G1045V	Non-Targeted Panel Gene	Adult HGBCL, NOS
BCL-82	chr17	18148924	18148924	C	T	exonic	MYO15A	10	15	0.6	Missense_Mutation	p.P2310S	Non-Targeted Panel Gene	Adult HGBCL, NOS
BCL-82	chr2	115836731	115836731	C	A	exonic	DPP10	5	8	0.6154	Missense_Mutation	p.H673N	Non-Targeted Panel Gene	Adult HGBCL, NOS
BCL-82	chr1	23796722	23796722	T	C	exonic	GALE	18	30	0.625	Missense_Mutation	p.K257R	Non-Targeted Panel Gene	Adult HGBCL, NOS
BCL-82	chr1	26343890	26343890	G	A	exonic	CRYBG2	12	22	0.6471	Missense_Mutation	p.P923L	Non-Targeted Panel Gene	Adult HGBCL, NOS
BCL-82	chr15	43002648	43002648	T	A	exonic	UBR1	3	6	0.6667	Missense_Mutation	p.D1189V	Non-Targeted Panel Gene	Adult HGBCL, NOS
BCL-82	chr5	128408776	128408776	G	A	exonic	FBN2	5	12	0.7059	Missense_Mutation	p.P326S	Non-Targeted Panel Gene	Adult HGBCL, NOS
BCL-82	chr1	156294074	156294074	G	A	exonic	GLMP	9	22	0.7097	Missense_Mutation	p.P248S	Non-Targeted Panel Gene	Adult HGBCL, NOS
BCL-82	chr16	3243896	3243896	C	T	exonic	MEFV	4	10	0.7143	Missense_Mutation	p.R433H	Non-Targeted Panel Gene	Adult HGBCL, NOS
BCL-82	chrX	126820844	126820844	A	C	exonic	PRR32	4	10	0.7143	Missense_Mutation	p.Q69P	Non-Targeted Panel Gene	Adult HGBCL, NOS
BCL-82	chr14	64052936	64052936	T	C	exonic	SYNE2	8	20	0.7143	Missense_Mutation	p.I3008T	Non-Targeted Panel Gene	Adult HGBCL, NOS
BCL-82	chrX	153803957	153803957	C	T	exonic	PDZD4	51	135	0.7258	Missense_Mutation	p.R473Q	Non-Targeted Panel Gene	Adult HGBCL, NOS
BCL-82	chrX	47565250	47565250	A	G	exonic;splicing	ARAF;ARAF	8	22	0.7333	Missense_Mutation	p.R156G	Non-Targeted Panel Gene	Adult HGBCL, NOS
BCL-82	chr2	219425952	219425952	G	A	exonic	DES	7	22	0.7586	Missense_Mutation	p.V459I	Non-Targeted Panel Gene	Adult HGBCL, NOS
BCL-82	chrX	80443318	80443318	G	T	exonic	FAM46D	3	11	0.7857	Missense_Mutation	p.R260L	Non-Targeted Panel Gene	Adult HGBCL, NOS
BCL-82	chr1	114405480	114405480	A	C	exonic	TRIM33	2	13	0.8667	Missense_Mutation	p.L900V	Non-Targeted Panel Gene	Adult HGBCL, NOS
BCL-82	chr1	197146215	197146215	C	T	exonic	ASPM	48	478	0.9087	Missense_Mutation	p.A75T	Non-Targeted Panel Gene	Adult HGBCL, NOS
BCL-82	chrX	54928890	54928890	C	A	exonic	TRO	1	10	0.9091	Missense_Mutation	p.N722K	Non-Targeted Panel Gene	Adult HGBCL, NOS
BCL-82	chrX	12717912	12717912	G	A	exonic	FRMPD4	0	23	1	Missense_Mutation	p.S1029N	Non-Targeted Panel Gene	Adult HGBCL, NOS

BCL-82	chr10	87933048	87933048	C	T	exonic	PTEN	26	16	0.381	Nonsense_Mutation	p.Q270X	Targerted Panel Gene	Adult HGBCL, NOS
BCL-82	chr16	69647357	69647357	G	T	exonic	NFAT5	17	2	0.1053	Nonsense_Mutation	p.E101X	Non-Targeted Panel Gene	Adult HGBCL, NOS
BCL-82	chr4	41676398	41676398	G	A	exonic	LIMCH1	8	3	0.2727	Nonsense_Mutation	p.W1152X	Non-Targeted Panel Gene	Adult HGBCL, NOS
BCL-83	chr19	38616260	38616260	C	T	splicing	MAP4K1	29	7	0.1944	Splice_Site	.	Targerted Panel Gene	Adult HGBCL, NOS
BCL-83	chr8	144054578	144054578	C	-	exonic	OPLAH	92	5	0.0515	Frame_Shift_Del	p.G890fs	Non-Targeted Panel Gene	Adult HGBCL, NOS
BCL-83	chr14	74798006	74798006	-	AA	exonic	YLPM1	25	8	0.2424	Frame_Shift_Ins	p.S903fs	Non-Targeted Panel Gene	Adult HGBCL, NOS
BCL-83	chr10	23192952	23192952	-	GAGCG	exonic	PTF1A	63	35	0.35	Frame_Shift_Ins	p.L141fs	Non-Targeted Panel Gene	Adult HGBCL, NOS
BCL-83	chr12	88077281	88077281	-	T	exonic	CEP290	23	35	0.6034	Frame_Shift_Ins	p.L1884fs	Non-Targeted Panel Gene	Adult HGBCL, NOS
BCL-83	chr19	50482215	50482238	GGTGGGGGTGGG[illegible]	-	exonic	EMC10	131	80	0.3791	In_Frame_Del	p.249_256del	Non-Targeted Panel Gene	Adult HGBCL, NOS
BCL-83	chr11	801588	801590	AGG	-	exonic	PIDD1	72	56	0.4375	In_Frame_Del	p.446_447del	Non-Targeted Panel Gene	Adult HGBCL, NOS
BCL-83	chr6	37170443	37170443	C	G	exonic	PIM1	219	86	0.281	Missense_Mutation	p.S47R	Targerted Panel Gene	Adult HGBCL, NOS
BCL-83	chr2	140495594	140495594	T	A	exonic	LRP1B	27	14	0.3415	Missense_Mutation	p.D3002V	Targerted Panel Gene	Adult HGBCL, NOS
BCL-83	chr2	96145156	96145156	G	C	exonic	DUSP2	65	34	0.3434	Missense_Mutation	p.L67V	Targerted Panel Gene	Adult HGBCL, NOS
BCL-83	chr17	63929438	63929438	T	C	exonic	CD79B	42	23	0.3538	Missense_Mutation	p.Y197C	Targerted Panel Gene	Adult HGBCL, NOS
BCL-83	chr4	105236541	105236541	T	C	exonic	TET2	27	24	0.4706	Missense_Mutation	p.Y867H	Targerted Panel Gene	Adult HGBCL, NOS
BCL-83	chr1	215934786	215934786	T	C	exonic	USH2A	12	12	0.5	Missense_Mutation	p.N2377S	Targerted Panel Gene	Adult HGBCL, NOS
BCL-83	chr6	72400574	72400574	G	A	exonic	RIMS1	64	3	0.0448	Missense_Mutation	p.D1647N	Non-Targeted Panel Gene	Adult HGBCL, NOS
BCL-83	chr19	21809162	21809162	G	C	exonic	ZNF43	48	5	0.0943	Missense_Mutation	p.A286G	Non-Targeted Panel Gene	Adult HGBCL, NOS
BCL-83	chr5	79080716	79080716	C	G	exonic	BHMT2	14	2	0.125	Missense_Mutation	p.D96E	Non-Targeted Panel Gene	Adult HGBCL, NOS
BCL-83	chr9	129059235	129059235	T	C	exonic	MIGA2	27	4	0.129	Missense_Mutation	p.S317P	Non-Targeted Panel Gene	Adult HGBCL, NOS
BCL-83	chr6	26368189	26368189	A	G	exonic	BTN3A2	120	20	0.1429	Missense_Mutation	p.M3V	Non-Targeted Panel Gene	Adult HGBCL, NOS
BCL-83	chr6	38929624	38929624	T	A	exonic	DNAH8	29	6	0.1714	Missense_Mutation	p.N3744K	Non-Targeted Panel Gene	Adult HGBCL, NOS
BCL-83	chr12	120174136	120174136	C	A	exonic	GCN1	29	6	0.1714	Missense_Mutation	p.G376V	Non-Targeted Panel Gene	Adult HGBCL, NOS
BCL-83	chr7	88282937	88282937	C	T	exonic	STEAP4	34	9	0.2093	Missense_Mutation	p.E230K	Non-Targeted Panel Gene	Adult HGBCL, NOS
BCL-83	chr3	125232454	125232454	G	A	exonic	ZNF148	66	20	0.2326	Missense_Mutation	p.R758W	Non-Targeted Panel Gene	Adult HGBCL, NOS
BCL-83	chr3	48417984	48417984	C	T	exonic	PLXNB1	110	36	0.2466	Missense_Mutation	p.D1101N	Non-Targeted Panel Gene	Adult HGBCL, NOS
BCL-83	chr12	52007057	52007057	A	G	exonic	GRASP	89	31	0.2583	Missense_Mutation	p.E13G	Non-Targeted Panel Gene	Adult HGBCL, NOS
BCL-83	chr12	54463180	54463180	G	T	exonic	GTSF1	31	11	0.2619	Missense_Mutation	p.Q79K	Non-Targeted Panel Gene	Adult HGBCL, NOS
BCL-83	chr11	126408420	126408420	A	G	exonic	ST3GAL4	51	19	0.2714	Missense_Mutation	p.D205G	Non-Targeted Panel Gene	Adult HGBCL, NOS
BCL-83	chr14	50188639	50188639	G	C	exonic	SOS2	15	6	0.2857	Missense_Mutation	p.P191R	Non-Targeted Panel Gene	Adult HGBCL, NOS
BCL-83	chr8	144108397	144108397	C	T	exonic	WDR97	105	44	0.2953	Missense_Mutation	p.R111C	Non-Targeted Panel Gene	Adult HGBCL, NOS
BCL-83	chr2	95374869	95374869	G	T	exonic	KCNIP3	89	38	0.2992	Missense_Mutation	p.D110Y	Non-Targeted Panel Gene	Adult HGBCL, NOS
BCL-83	chr7	107929489	107929489	T	G	exonic	LAMB1	44	20	0.3125	Missense_Mutation	p.Q1556H	Non-Targeted Panel Gene	Adult HGBCL, NOS
BCL-83	chr12	113157611	113157611	T	C	exonic	CFAP73	100	47	0.3197	Missense_Mutation	p.F287L	Non-Targeted Panel Gene	Adult HGBCL, NOS
BCL-83	chr6	116809357	116809357	G	C	exonic	GPRC6A	25	12	0.3243	Missense_Mutation	p.T152S	Non-Targeted Panel Gene	Adult HGBCL, NOS
BCL-83	chr22	17120358	17120358	G	A	exonic	CECR6	150	76	0.3363	Missense_Mutation	p.A257V	Non-Targeted Panel Gene	Adult HGBCL, NOS
BCL-83	chr20	35434442	35434442	G	A	exonic	GDF5;GDF5OS	145	74	0.3379	Missense_Mutation	p.R325C	Non-Targeted Panel Gene	Adult HGBCL, NOS
BCL-83	chr16	57920480	57920480	C	T	exonic	CNGB1	36	19	0.3455	Missense_Mutation	p.E570K	Non-Targeted Panel Gene	Adult HGBCL, NOS
BCL-83	chr1	151289756	151289756	C	T	exonic	ZNF687	34	18	0.3462	Missense_Mutation	p.P905S	Non-Targeted Panel Gene	Adult HGBCL, NOS
BCL-83	chr20	38724828	38724828	C	T	exonic	SLC32A1	141	75	0.3472	Missense_Mutation	p.A35V	Non-Targeted Panel Gene	Adult HGBCL, NOS
BCL-83	chr11	3228166	3228166	G	T	exonic	MRGPRE	76	43	0.3613	Missense_Mutation	p.P212T	Non-Targeted Panel Gene	Adult HGBCL, NOS
BCL-83	chr4	13605071	13605071	T	C	exonic	BOD1L1	19	11	0.3667	Missense_Mutation	p.E610G	Non-Targeted Panel Gene	Adult HGBCL, NOS
BCL-83	chr22	25029141	25029141	A	G	exonic	KIAA1671	30	18	0.375	Missense_Mutation	p.N381S	Non-Targeted Panel Gene	Adult HGBCL, NOS
BCL-83	chr1	149945018	149945018	C	A	exonic	OTUD7B	48	30	0.3846	Missense_Mutation	p.E457D	Non-Targeted Panel Gene	Adult HGBCL, NOS
BCL-83	chr16	55567414	55567414	T	C	exonic	CAPNS2	28	18	0.3913	Missense_Mutation	p.F220L	Non-Targeted Panel Gene	Adult HGBCL, NOS
BCL-83	chr13	46372363	46372363	G	A	exonic	RUBCNL	26	17	0.3953	Missense_Mutation	p.P38L	Non-Targeted Panel Gene	Adult HGBCL, NOS
BCL-83	chr17	60623662	60623662	A	T	exonic	PPM1D	18	12	0.4	Missense_Mutation	p.D205V	Non-Targeted Panel Gene	Adult HGBCL, NOS
BCL-83	chr6	132783939	132783939	T	G	exonic	SLC18B1	12	8	0.4	Missense_Mutation	p.N218H	Non-Targeted Panel Gene	Adult HGBCL, NOS
BCL-83	chr19	17281937	17281937	G	A	exonic	ANKLE1	68	46	0.4035	Missense_Mutation	p.R60H	Non-Targeted Panel Gene	Adult HGBCL, NOS
BCL-83	chr2	184936300	184936300	G	C	exonic	ZNF804A	19	13	0.4062	Missense_Mutation	p.E302Q	Non-Targeted Panel Gene	Adult HGBCL, NOS
BCL-83	chr6	147339208	147339208	G	C	exonic	STXBP5	17	12	0.4138	Missense_Mutation	p.E726Q	Non-Targeted Panel Gene	Adult HGBCL, NOS
BCL-83	chr8	144255636	144255636	G	A	exonic	MROH1	67	51	0.4322	Missense_Mutation	p.R1241Q	Non-Targeted Panel Gene	Adult HGBCL, NOS
BCL-83	chr5	141394293	141394293	G	A	exonic	PCDHGA8	114	88	0.4356	Missense_Mutation	p.D494N	Non-Targeted Panel Gene	Adult HGBCL, NOS
BCL-83	chr4	153781503	153781503	C	T	exonic	SFRP2	36	28	0.4375	Missense_Mutation	p.G279E	Non-Targeted Panel Gene	Adult HGBCL, NOS
BCL-83	chr3	124973879	124973879	A	C	exonic	HEG1	23	18	0.439	Missense_Mutation	p.L1283R	Non-Targeted Panel Gene	Adult HGBCL, NOS
BCL-83	chr1	167918357	167918357	T	G	exonic	MPC2	19	15	0.4412	Missense_Mutation	p.Y117S	Non-Targeted Panel Gene	Adult HGBCL, NOS
BCL-83	chr19	55955295	55955295	G	A	exonic	NLRP8	19	15	0.4412	Missense_Mutation	p.G413R	Non-Targeted Panel Gene	Adult HGBCL, NOS
BCL-83	chr10	49655142	49655142	G	A	exonic	CHAT	46	37	0.4458	Missense_Mutation	p.R443Q	Non-Targeted Panel Gene	Adult HGBCL, NOS
BCL-83	chr15	28014912	28014912	C	A	exonic	OCA2	27	23	0.46	Missense_Mutation	p.S303I	Non-Targeted Panel Gene	Adult HGBCL, NOS
BCL-83	chr3	195579407	195579407	C	T	exonic	APOD	30	26	0.4643	Missense_Mutation	p.E19K	Non-Targeted Panel Gene	Adult HGBCL, NOS
BCL-83	chr15	47770668	47770668	A	G	exonic	SEMA6D	15	13	0.4643	Missense_Mutation	p.E640G	Non-Targeted Panel Gene	Adult HGBCL, NOS
BCL-83	chr5	90622613	90622613	T	C	exonic	ADGRV1	17	15	0.4688	Missense_Mutation	p.V157A	Non-Targeted Panel Gene	Adult HGBCL, NOS
BCL-83	chr3	39143393	39143393	G	A	exonic	CSRNP1	21	20	0.4878	Missense_Mutation	p.L478F	Non-Targeted Panel Gene	Adult HGBCL, NOS
BCL-83	chr8	144116132	144116132	G	T	exonic	WDR97	117	115	0.4957	Missense_Mutation	p.G1570C	Non-Targeted Panel Gene	Adult HGBCL, NOS
BCL-83	chr2	165127711	165127711	T	C	exonic	SCN3A	11	11	0.5	Missense_Mutation	p.I1105V	Non-Targeted Panel Gene	Adult HGBCL, NOS
BCL-83	chr20	33443412	33443412	G	T	exonic	SNTA1	15	15	0.5	Missense_Mutation	p.P70Q	Non-Targeted Panel Gene	Adult HGBCL, NOS
BCL-83	chr11	94821715	94821715	C	A	exonic	AMOTL1	88	89	0.5028	Missense_Mutation	p.A436E	Non-Targeted Panel Gene	Adult HGBCL, NOS
BCL-83	chr11	134168157	134168157	C	T	exonic	NCAPD3	21	22	0.5116	Missense_Mutation	p.A1138T	Non-Targeted Panel Gene	Adult HGBCL, NOS
BCL-83	chr10	132331501	132331501	T	A	exonic	STK32C	53	56	0.5138	Missense_Mutation	p.Q79L	Non-Targeted Panel Gene	Adult HGBCL, NOS
BCL-83	chr7	74254347	74254347	C	T	exonic	RFC2	99	106	0.5171	Missense_Mutation	p.V13M	Non-Targeted Panel Gene	Adult HGBCL, NOS
BCL-83	chr14	57282167	57282167	C	T	exonic	AP5M1	12	13	0.52	Missense_Mutation	p.L343F	Non-Targeted Panel Gene	Adult HGBCL, NOS
BCL-83	chr16	68822063	68822063	G	A	exonic	CDH1	16	18	0.5294	Missense_Mutation	p.A592T	Non-Targeted Panel Gene	Adult HGBCL, NOS
BCL-83	chr4	154828039	154828039	G	C	exonic	RBM46	21	25	0.5435	Missense_Mutation	p.C525S	Non-Targeted Panel Gene	Adult HGBCL, NOS
BCL-83	chr13	101579359	101579359	G	A	exonic	ITGBL1	10	12	0.5455	Missense_Mutation	p.C220Y	Non-Targeted Panel Gene	Adult HGBCL, NOS
BCL-83	chr1	20684810	20684810	G	T	exonic	KIF17	43	52	0.5474	Missense_Mutation	p.R744S	Non-Targeted Panel Gene	Adult HGBCL, NOS
BCL-83	chr8	144931565	144931565	T	C	exonic	ZNF16	30	37	0.5522	Missense_Mutation	p.N408D	Non-Targeted Panel Gene	Adult HGBCL, NOS
BCL-83	chr15	41760390	41760390	C	T	exonic	MGA	11	14	0.56	Missense_Mutation	p.A2420V	Non-Targeted Panel Gene	Adult HGBCL, NOS
BCL-83	chr5	128408776	128408776	G	A	exonic	FBN2	24	31	0.5636	Missense_Mutation	p.P326S	Non-Targeted Panel Gene	Adult HGBCL, NOS
BCL-83	chr2	157766106	157766106	T	C	exonic	ACVR1	21	28	0.5714	Missense_Mutation	p.Y294C	Non-Targeted Panel Gene	Adult HGBCL, NOS
BCL-83	chr10	100463232	100463232	A	G	exonic	WNT8B	14	19	0.5758	Missense_Mutation	p.S22G	Non-Targeted Panel Gene	Adult HGBCL, NOS
BCL-83	chr2	202555905	202555905	A	C	exonic	BMPR2	13	18	0.5806	Missense_Mutation	p.K747T	Non-Targeted Panel Gene	Adult HGBCL, NOS
BCL-83	chr2	240011654	240011654	C	T	exonic	NDUFA10	19	27	0.587	Missense_Mutation	p.E238K	Non-Targeted Panel Gene	Adult HGBCL, NOS

BCL-83	chr3	50345533	50345533	A	C	exonic	ZMYND10	190	289	0.6033	Missense_Mutation	p.V16G	Non-Targeted Panel Gene	Adult HGBCL, NOS
BCL-83	chr15	34090319	34090319	T	C	exonic	EMC7	12	20	0.625	Missense_Mutation	p.M165V	Non-Targeted Panel Gene	Adult HGBCL, NOS
BCL-83	chr8	50502807	50502807	A	C	exonic	SNTG1	9	15	0.625	Missense_Mutation	p.E131D	Non-Targeted Panel Gene	Adult HGBCL, NOS
BCL-83	chrX	1466443	1466443	G	A	exonic	P2RY8	22	41	0.6508	Missense_Mutation	p.P39L	Non-Targeted Panel Gene	Adult HGBCL, NOS
BCL-83	chr4	65348166	65348166	G	A	exonic	EPHA5	9	17	0.6538	Missense_Mutation	p.A849V	Non-Targeted Panel Gene	Adult HGBCL, NOS
BCL-83	chr6	27401281	27401281	C	T	exonic	ZNF391	12	32	0.7273	Missense_Mutation	p.P304L	Non-Targeted Panel Gene	Adult HGBCL, NOS
BCL-83	chr1	170166697	170166697	G	A	exonic	METTL11B	9	25	0.7353	Missense_Mutation	p.E176K	Non-Targeted Panel Gene	Adult HGBCL, NOS
BCL-83	chrX	77875487	77875487	A	C	exonic	MAGT1	2	10	0.8333	Missense_Mutation	p.N103K	Non-Targeted Panel Gene	Adult HGBCL, NOS
BCL-83	chr6	7542003	7542003	G	A	exonic	DSP	31	319	0.9114	Missense_Mutation	p.V30M	Non-Targeted Panel Gene	Adult HGBCL, NOS
BCL-83	chrX	106827219	106827219	G	A	exonic	TBC1D8B	0	24	1	Missense_Mutation	p.S362N	Non-Targeted Panel Gene	Adult HGBCL, NOS
BCL-83	chr12	8829731	8829731	T	A	exonic	A2ML1	47	13	0.2167	Nonsense_Mutation	p.Y138X	Non-Targeted Panel Gene	Adult HGBCL, NOS
BCL-83	chr2	67403250	67403250	G	T	exonic	ETAA1	13	6	0.3158	Nonsense_Mutation	p.E190X	Non-Targeted Panel Gene	Adult HGBCL, NOS
BCL-83	chr1	37483164	37483164	G	A	exonic	ZC3H12A	138	67	0.3252	Nonsense_Mutation	p.W451X	Non-Targeted Panel Gene	Adult HGBCL, NOS
BCL-83	chr15	62835948	62835948	C	T	exonic	TLN2	104	53	0.3376	Nonsense_Mutation	p.Q2417X	Non-Targeted Panel Gene	Adult HGBCL, NOS
BCL-83	chr5	122423425	122423425	A	T	exonic	SNCAIP	24	16	0.4	Nonsense_Mutation	p.K277X	Non-Targeted Panel Gene	Adult HGBCL, NOS
BCL-83	chr15	74956145	74956145	G	A	exonic	RPP25	156	111	0.4157	Nonsense_Mutation	p.R147X	Non-Targeted Panel Gene	Adult HGBCL, NOS
BCL-83	chr9	5164200	5164200	-	A	exonic	INSL6	6	27	0.7714	Nonsense_Mutation	p.K119_K120delins	Non-Targeted Panel Gene	Adult HGBCL, NOS
BCL-84	chr4	154328193	154328193	C	G	splicing	DCHS2	33	27	0.45	Splice_Site	.	Targerted Panel Gene	Adult HGBCL, NOS
BCL-84	chr19	4028929	4028929	A	G	splicing	PIAS4	73	40	0.354	Splice_Site	.	Non-Targeted Panel Gene	Adult HGBCL, NOS
BCL-84	chr6	105162161	105162161	C	T	splicing	POPDC3	4	12	0.75	Splice_Site	.	Non-Targeted Panel Gene	Adult HGBCL, NOS
BCL-84	chr22	41168753	41168757	TTCCA	-	exonic	EP300	65	46	0.4144	Frame_Shift_Del	p.F1353fs	Targerted Panel Gene	Adult HGBCL, NOS
BCL-84	chr10	89013348	89013349	AG	-	exonic	FAS	29	26	0.4727	Frame_Shift_Del	p.T219fs	Targerted Panel Gene	Adult HGBCL, NOS
BCL-84	chr9	4662593	4662593	T	-	exonic	PLPP6	64	4	0.0588	Frame_Shift_Del	p.L73fs	Non-Targeted Panel Gene	Adult HGBCL, NOS
BCL-84	chr4	68314236	68314236	G	-	exonic	YTHDC1	80	57	0.4161	Frame_Shift_Del	p.R691fs	Non-Targeted Panel Gene	Adult HGBCL, NOS
BCL-84	chr11	63228554	63228554	A	-	exonic	SLC22A25	32	25	0.4386	Frame_Shift_Del	p.V138fs	Non-Targeted Panel Gene	Adult HGBCL, NOS
BCL-84	chr11	121612758	121612758	T	-	exonic	SORL1	25	29	0.537	Frame_Shift_Del	p.L1782fs	Non-Targeted Panel Gene	Adult HGBCL, NOS
BCL-84	chr16	11047301	11047301	-	T	exonic	CLEC16A	38	14	0.2692	Frame_Shift_Ins	p.I609fs	Non-Targeted Panel Gene	Adult HGBCL, NOS
BCL-84	chr12	71583778	71583778	-	C	exonic	LGR5	49	20	0.2857	Frame_Shift_Ins	p.S590fs	Non-Targeted Panel Gene	Adult HGBCL, NOS
BCL-84	chr2	21142286	21142286	-	T	exonic	TDRD15	43	23	0.3433	Frame_Shift_Ins	p.V1607fs	Non-Targeted Panel Gene	Adult HGBCL, NOS
BCL-84	chr19	8896820	8896820	-	GGGGA	exonic	MUC16	34	18	0.3462	Frame_Shift_Ins	p.S13158fs	Non-Targeted Panel Gene	Adult HGBCL, NOS
BCL-84	chr6	31356201	31356201	-	TA	exonic	HLA-B	75	99	0.5625	Frame_Shift_Ins	p.Y195fs	Non-Targeted Panel Gene	Adult HGBCL, NOS
BCL-84	chr3	50257024	50257026	AAG	-	exonic	GNAI2	52	18	0.2571	In_Frame_Del	p.255_255del	Targerted Panel Gene	Adult HGBCL, NOS
BCL-84	chr2	168247287	168247292	GCCGGG	-	exonic	STK39	46	20	0.303	In_Frame_Del	p.48_50del	Non-Targeted Panel Gene	Adult HGBCL, NOS
BCL-84	chr3	32541167	32541169	TCT	-	exonic	DYNC1LI1	24	15	0.3846	In_Frame_Del	p.202_203del	Non-Targeted Panel Gene	Adult HGBCL, NOS
BCL-84	chr12	49039322	49039322	G	C	exonic	KMT2D	71	18	0.2022	Missense_Mutation	p.L2756V	Targerted Panel Gene	Adult HGBCL, NOS
BCL-84	chr15	33835068	33835068	C	G	exonic	RYR3	52	24	0.3158	Missense_Mutation	p.S3855C	Targerted Panel Gene	Adult HGBCL, NOS
BCL-84	chr7	100953706	100953706	A	C	exonic	MUC3A	10	5	0.3333	Missense_Mutation	p.T643P	Targerted Panel Gene	Adult HGBCL, NOS
BCL-84	chr3	78746786	78746786	T	A	exonic	ROBO1	17	9	0.3462	Missense_Mutation	p.K205M	Targerted Panel Gene	Adult HGBCL, NOS
BCL-84	chr16	72787661	72787661	A	C	exonic	ZFHX3	128	81	0.3876	Missense_Mutation	p.C3539G	Targerted Panel Gene	Adult HGBCL, NOS
BCL-84	chr4	78400798	78400798	A	G	exonic	FRAS1	25	17	0.4048	Missense_Mutation	p.Q1347R	Targerted Panel Gene	Adult HGBCL, NOS
BCL-84	chr19	621132	621132	G	A	exonic	POLRMT	221	190	0.4612	Missense_Mutation	p.P856S	Targerted Panel Gene	Adult HGBCL, NOS
BCL-84	chr11	6520269	6520269	C	T	exonic	DNHD1	37	33	0.4714	Missense_Mutation	p.S606L	Targerted Panel Gene	Adult HGBCL, NOS
BCL-84	chr16	3729114	3729114	T	C	exonic	CREBBP	71	65	0.4779	Missense_Mutation	p.N1978S	Targerted Panel Gene	Adult HGBCL, NOS
BCL-84	chr6	41935969	41935969	G	A	exonic	CCND3	37	39	0.5132	Missense_Mutation	p.P284S	Targerted Panel Gene	Adult HGBCL, NOS
BCL-84	chr12	49040100	49040100	G	A	exonic	KMT2D	29	35	0.5469	Missense_Mutation	p.P2557L	Targerted Panel Gene	Adult HGBCL, NOS
BCL-84	chr6	112349944	112349944	G	A	exonic	RFPL4B	73	3	0.0395	Missense_Mutation	p.R79Q	Non-Targeted Panel Gene	Adult HGBCL, NOS
BCL-84	chrX	101102330	101102330	G	A	exonic	CENPI	48	2	0.04	Missense_Mutation	p.V95M	Non-Targeted Panel Gene	Adult HGBCL, NOS
BCL-84	chr6	138432094	138432094	C	T	exonic	NHSL1	72	3	0.04	Missense_Mutation	p.A751T	Non-Targeted Panel Gene	Adult HGBCL, NOS
BCL-84	chr11	111836287	111836287	G	A	exonic	ALG9	46	2	0.0417	Missense_Mutation	p.L494F	Non-Targeted Panel Gene	Adult HGBCL, NOS
BCL-84	chr8	132077846	132077846	G	A	exonic	HHLA1	47	3	0.06	Missense_Mutation	p.R351C	Non-Targeted Panel Gene	Adult HGBCL, NOS
BCL-84	chr4	2341480	2341480	C	T	exonic	ZFYVE28	46	3	0.0612	Missense_Mutation	p.E106K	Non-Targeted Panel Gene	Adult HGBCL, NOS
BCL-84	chr15	65077221	65077221	C	G	exonic	KBTBD13	190	13	0.064	Missense_Mutation	p.L136V	Non-Targeted Panel Gene	Adult HGBCL, NOS
BCL-84	chr17	30858290	30858290	C	A	exonic	ATAD5	43	3	0.0652	Missense_Mutation	p.Q975K	Non-Targeted Panel Gene	Adult HGBCL, NOS
BCL-84	chr22	31618347	31618347	C	T	exonic	SFI1	42	3	0.0667	Missense_Mutation	p.L1220F	Non-Targeted Panel Gene	Adult HGBCL, NOS
BCL-84	chrX	12810082	12810082	C	T	exonic	PRPS2	40	3	0.0698	Missense_Mutation	p.R156W	Non-Targeted Panel Gene	Adult HGBCL, NOS
BCL-84	chr5	52788384	52788384	G	C	exonic	ITGA1	52	4	0.0714	Missense_Mutation	p.V11L	Non-Targeted Panel Gene	Adult HGBCL, NOS
BCL-84	chrX	100296508	100296508	C	G	exonic	PCDH19	23	2	0.08	Missense_Mutation	p.K1072N	Non-Targeted Panel Gene	Adult HGBCL, NOS
BCL-84	chr5	133101783	133101783	C	T	exonic	HSPA4	66	6	0.0833	Missense_Mutation	p.R688C	Non-Targeted Panel Gene	Adult HGBCL, NOS
BCL-84	chr15	58771825	58771825	A	G	exonic	MINDY2	114	14	0.1094	Missense_Mutation	p.T144A	Non-Targeted Panel Gene	Adult HGBCL, NOS
BCL-84	chr2	63056179	63056179	G	T	exonic	OTX1	106	20	0.1587	Missense_Mutation	p.A310S	Non-Targeted Panel Gene	Adult HGBCL, NOS
BCL-84	chr9	99036323	99036323	G	T	exonic	COL15A1	40	10	0.2	Missense_Mutation	p.G779V	Non-Targeted Panel Gene	Adult HGBCL, NOS
BCL-84	chr12	88083908	88083908	A	C	exonic	CEP290	27	9	0.25	Missense_Mutation	p.L1584R	Non-Targeted Panel Gene	Adult HGBCL, NOS
BCL-84	chr1	154551483	154551483	T	A	exonic	UBE2Q1	27	9	0.25	Missense_Mutation	p.S362C	Non-Targeted Panel Gene	Adult HGBCL, NOS
BCL-84	chr4	168225604	168225604	A	C	exonic	DDX60	29	10	0.2564	Missense_Mutation	p.F1536V	Non-Targeted Panel Gene	Adult HGBCL, NOS
BCL-84	chr8	132141213	132141213	G	T	exonic	KCNQ3	35	15	0.3	Missense_Mutation	p.P461T	Non-Targeted Panel Gene	Adult HGBCL, NOS
BCL-84	chr6	50828674	50828674	A	C	exonic	TFAP2B	37	16	0.3019	Missense_Mutation	p.K199T	Non-Targeted Panel Gene	Adult HGBCL, NOS
BCL-84	chr4	2931898	2931898	G	A	exonic	MFSD10	73	32	0.3048	Missense_Mutation	p.L281F	Non-Targeted Panel Gene	Adult HGBCL, NOS
BCL-84	chr6	43780743	43780743	G	A	exonic	VEGFA	17	8	0.32	Missense_Mutation	p.R325Q	Non-Targeted Panel Gene	Adult HGBCL, NOS
BCL-84	chr1	153757677	153757677	G	A	exonic	INTS3	35	17	0.3269	Missense_Mutation	p.G355R	Non-Targeted Panel Gene	Adult HGBCL, NOS
BCL-84	chr4	185370785	185370785	A	T	exonic	LRP2BP	28	14	0.3333	Missense_Mutation	p.I278N	Non-Targeted Panel Gene	Adult HGBCL, NOS
BCL-84	chr1	248239629	248239629	G	A	exonic	OR2M4	34	17	0.3333	Missense_Mutation	p.R234H	Non-Targeted Panel Gene	Adult HGBCL, NOS
BCL-84	chr18	25226042	25226042	C	T	exonic	ZNF521	46	24	0.3429	Missense_Mutation	p.A626T	Non-Targeted Panel Gene	Adult HGBCL, NOS
BCL-84	chr19	56423115	56423115	G	T	exonic	ZNF583	57	30	0.3448	Missense_Mutation	p.V153F	Non-Targeted Panel Gene	Adult HGBCL, NOS
BCL-84	chr2	208142825	208142825	T	C	exonic	CRYGB	26	14	0.35	Missense_Mutation	p.Q114R	Non-Targeted Panel Gene	Adult HGBCL, NOS
BCL-84	chr17	1280107	1280107	A	G	exonic	TUSC5	65	35	0.35	Missense_Mutation	p.K36E	Non-Targeted Panel Gene	Adult HGBCL, NOS
BCL-84	chr21	36211980	36211980	A	G	exonic	DOPEY2	33	18	0.3529	Missense_Mutation	p.N263D	Non-Targeted Panel Gene	Adult HGBCL, NOS
BCL-84	chr11	132314683	132314683	A	T	exonic	NTM	31	17	0.3542	Missense_Mutation	p.N305I	Non-Targeted Panel Gene	Adult HGBCL, NOS
BCL-84	chr4	70196716	70196716	G	A	exonic	ODAM	48	27	0.36	Missense_Mutation	p.A26T	Non-Targeted Panel Gene	Adult HGBCL, NOS
BCL-84	chr16	84237423	84237423	G	T	exonic	KCNG4	51	29	0.3625	Missense_Mutation	p.S21R	Non-Targeted Panel Gene	Adult HGBCL, NOS
BCL-84	chr10	89339962	89339962	A	G	exonic	IFIT3	35	20	0.3636	Missense_Mutation	p.K384R	Non-Targeted Panel Gene	Adult HGBCL, NOS
BCL-84	chr1	24084591	24084591	G	T	exonic	MYOM3	40	23	0.3651	Missense_Mutation	p.T616N	Non-Targeted Panel Gene	Adult HGBCL, NOS
BCL-84	chr17	78164358	78164358	C	T	exonic	C17orf99	94	55	0.3691	Missense_Mutation	p.P212S	Non-Targeted Panel Gene	Adult HGBCL, NOS

BCL-84	chr6	54926452	54926452	G	C	exonic	FAM83B	32	19	0.3725	Missense_Mutation	p.V176L	Non-Targeted Panel Gene	Adult HGBCL, NOS
BCL-84	chr22	38867041	38867041	C	T	exonic	CBX6	114	68	0.3736	Missense_Mutation	p.R136H	Non-Targeted Panel Gene	Adult HGBCL, NOS
BCL-84	chr6	131808117	131808117	G	A	exonic	ENPP1	10	6	0.375	Missense_Mutation	p.G28S	Non-Targeted Panel Gene	Adult HGBCL, NOS
BCL-84	chr1	32668847	32668847	A	G	exonic	RBBP4	28	17	0.3778	Missense_Mutation	p.D198G	Non-Targeted Panel Gene	Adult HGBCL, NOS
BCL-84	chr6	138263348	138263348	C	T	exonic	ARFGEF3	53	33	0.3837	Missense_Mutation	p.S622L	Non-Targeted Panel Gene	Adult HGBCL, NOS
BCL-84	chr3	53718725	53718725	G	T	exonic	CACNA1D	33	21	0.3889	Missense_Mutation	p.G508W	Non-Targeted Panel Gene	Adult HGBCL, NOS
BCL-84	chr8	37965340	37965340	G	A	exonic	ADRB3	51	33	0.3929	Missense_Mutation	p.P377L	Non-Targeted Panel Gene	Adult HGBCL, NOS
BCL-84	chr8	105801714	105801714	G	A	exonic	ZFPM2	26	17	0.3953	Missense_Mutation	p.M544I	Non-Targeted Panel Gene	Adult HGBCL, NOS
BCL-84	chr18	26856382	26856382	G	T	exonic	AQP4	44	29	0.3973	Missense_Mutation	p.S267R	Non-Targeted Panel Gene	Adult HGBCL, NOS
BCL-84	chr20	63964603	63964603	T	G	exonic	ZNF512B	44	29	0.3973	Missense_Mutation	p.D383A	Non-Targeted Panel Gene	Adult HGBCL, NOS
BCL-84	chr17	40835174	40835174	G	A	exonic	TMEM99	40	27	0.403	Missense_Mutation	p.G220S	Non-Targeted Panel Gene	Adult HGBCL, NOS
BCL-84	chr16	88859950	88859950	C	T	exonic	TRAPPC2L	115	78	0.4041	Missense_Mutation	p.P118S	Non-Targeted Panel Gene	Adult HGBCL, NOS
BCL-84	chr15	54338376	54338376	A	G	exonic	UNC13C	39	27	0.4091	Missense_Mutation	p.S1532G	Non-Targeted Panel Gene	Adult HGBCL, NOS
BCL-84	chr12	20369297	20369297	G	A	exonic	PDE3A	20	14	0.4118	Missense_Mutation	p.G5S	Non-Targeted Panel Gene	Adult HGBCL, NOS
BCL-84	chr9	38068315	38068315	G	A	exonic	SHB	40	28	0.4118	Missense_Mutation	p.R111C	Non-Targeted Panel Gene	Adult HGBCL, NOS
BCL-84	chr8	9011627	9011627	A	T	exonic	ERI1	34	24	0.4138	Missense_Mutation	p.S125C	Non-Targeted Panel Gene	Adult HGBCL, NOS
BCL-84	chr2	73448281	73448281	A	C	exonic	ALMS1	53	39	0.4239	Missense_Mutation	p.Y585S	Non-Targeted Panel Gene	Adult HGBCL, NOS
BCL-84	chr3	57428372	57428372	T	G	exonic	DNAH12	38	28	0.4242	Missense_Mutation	p.K1777Q	Non-Targeted Panel Gene	Adult HGBCL, NOS
BCL-84	chr13	99982192	99982192	C	G	exonic	ZIC2	137	101	0.4244	Missense_Mutation	p.A43G	Non-Targeted Panel Gene	Adult HGBCL, NOS
BCL-84	chr12	41188745	41188745	T	A	exonic	PDZRN4	23	17	0.425	Missense_Mutation	p.L97Q	Non-Targeted Panel Gene	Adult HGBCL, NOS
BCL-84	chr9	116187318	116187318	G	T	exonic	PAPPA	41	31	0.4306	Missense_Mutation	p.G194C	Non-Targeted Panel Gene	Adult HGBCL, NOS
BCL-84	chr13	87675735	87675735	T	G	exonic	SLITRK5	41	31	0.4306	Missense_Mutation	p.V116G	Non-Targeted Panel Gene	Adult HGBCL, NOS
BCL-84	chr1	205774841	205774841	G	A	exonic	RAB29	59	45	0.4327	Missense_Mutation	p.T39M	Non-Targeted Panel Gene	Adult HGBCL, NOS
BCL-84	chr17	1480603	1480603	G	A	exonic	MYO1C	55	42	0.433	Missense_Mutation	p.S242F	Non-Targeted Panel Gene	Adult HGBCL, NOS
BCL-84	chr8	42322484	42322484	A	G	exonic	IKBKB	17	13	0.4333	Missense_Mutation	p.K659R	Non-Targeted Panel Gene	Adult HGBCL, NOS
BCL-84	chr16	1747195	1747195	A	G	exonic	MAPK8IP3	64	49	0.4336	Missense_Mutation	p.N304S	Non-Targeted Panel Gene	Adult HGBCL, NOS
BCL-84	chr13	95833047	95833047	T	G	exonic	UGGT2	26	20	0.4348	Missense_Mutation	p.N1470H	Non-Targeted Panel Gene	Adult HGBCL, NOS
BCL-84	chr2	61872241	61872241	A	T	exonic	CCT4	31	24	0.4364	Missense_Mutation	p.S444R	Non-Targeted Panel Gene	Adult HGBCL, NOS
BCL-84	chr11	125983979	125983979	T	C	exonic	CDON	19	15	0.4412	Missense_Mutation	p.D963G	Non-Targeted Panel Gene	Adult HGBCL, NOS
BCL-84	chr1	1048126	1048126	C	T	exonic	AGRN	44	35	0.443	Missense_Mutation	p.P1289L	Non-Targeted Panel Gene	Adult HGBCL, NOS
BCL-84	chr11	130449052	130449052	G	A	exonic	ADAMTS15	57	46	0.4466	Missense_Mutation	p.V27I	Non-Targeted Panel Gene	Adult HGBCL, NOS
BCL-84	chr13	95833037	95833037	G	C	exonic	UGGT2	26	21	0.4468	Missense_Mutation	p.T1473R	Non-Targeted Panel Gene	Adult HGBCL, NOS
BCL-84	chr11	47282945	47282945	C	G	exonic	MADD	37	30	0.4478	Missense_Mutation	p.S613C	Non-Targeted Panel Gene	Adult HGBCL, NOS
BCL-84	chr1	161048380	161048380	G	C	exonic	ARHGAP30	40	33	0.4521	Missense_Mutation	p.H881D	Non-Targeted Panel Gene	Adult HGBCL, NOS
BCL-84	chr19	42095449	42095449	G	A	exonic	POU2F2	98	81	0.4525	Missense_Mutation	p.T307I	Non-Targeted Panel Gene	Adult HGBCL, NOS
BCL-84	chr21	21477331	21477331	G	A	exonic	NCAM2	36	30	0.4545	Missense_Mutation	p.G646E	Non-Targeted Panel Gene	Adult HGBCL, NOS
BCL-84	chr20	59880407	59880407	T	C	exonic	SYCP2	30	25	0.4545	Missense_Mutation	p.D946G	Non-Targeted Panel Gene	Adult HGBCL, NOS
BCL-84	chr1	94007731	94007731	G	A	exonic	ABCA4	27	23	0.46	Missense_Mutation	p.L1970F	Non-Targeted Panel Gene	Adult HGBCL, NOS
BCL-84	chr2	85553369	85553369	T	A	exonic	GGCX	32	28	0.4667	Missense_Mutation	p.S340C	Non-Targeted Panel Gene	Adult HGBCL, NOS
BCL-84	chr11	101944389	101944389	C	G	exonic	CEP126	27	24	0.4706	Missense_Mutation	p.L125V	Non-Targeted Panel Gene	Adult HGBCL, NOS
BCL-84	chr11	19234518	19234518	G	A	exonic	E2F8	36	32	0.4706	Missense_Mutation	p.S257F	Non-Targeted Panel Gene	Adult HGBCL, NOS
BCL-84	chr20	62318619	62318619	G	C	exonic	LAMA5	56	50	0.4717	Missense_Mutation	p.S2358R	Non-Targeted Panel Gene	Adult HGBCL, NOS
BCL-84	chr2	178541317	178541317	C	T	exonic	TTN	28	26	0.4815	Missense_Mutation	p.R32587H	Non-Targeted Panel Gene	Adult HGBCL, NOS
BCL-84	chr17	65534045	65534045	C	T	exonic	AXIN2	31	29	0.4833	Missense_Mutation	p.A758T	Non-Targeted Panel Gene	Adult HGBCL, NOS
BCL-84	chr11	101062632	101062632	G	A	exonic	PGR	35	33	0.4853	Missense_Mutation	p.S82L	Non-Targeted Panel Gene	Adult HGBCL, NOS
BCL-84	chr1	173751813	173751813	A	G	exonic	KLHL20	19	18	0.4865	Missense_Mutation	p.D216G	Non-Targeted Panel Gene	Adult HGBCL, NOS
BCL-84	chr22	28107929	28107929	T	C	exonic	TTC28	38	36	0.4865	Missense_Mutation	p.N639S	Non-Targeted Panel Gene	Adult HGBCL, NOS
BCL-84	chr19	7862488	7862488	C	T	exonic	EVI5L	94	90	0.4891	Missense_Mutation	p.T623M	Non-Targeted Panel Gene	Adult HGBCL, NOS
BCL-84	chr4	667977	667977	A	C	exonic	PDE6B	24	24	0.4898	Missense_Mutation	p.E825A	Non-Targeted Panel Gene	Adult HGBCL, NOS
BCL-84	chr3	30672342	30672342	G	A	exonic	TGFBR2	59	57	0.4914	Missense_Mutation	p.V412M	Non-Targeted Panel Gene	Adult HGBCL, NOS
BCL-84	chr15	41027646	41027646	G	A	exonic	INO80	29	29	0.5	Missense_Mutation	p.R1000C	Non-Targeted Panel Gene	Adult HGBCL, NOS
BCL-84	chr22	44888093	44888093	G	A	exonic	PHF21B	5	5	0.5	Missense_Mutation	p.A152V	Non-Targeted Panel Gene	Adult HGBCL, NOS
BCL-84	chr16	30756449	30756449	A	G	exonic	PHKG2	72	72	0.5	Missense_Mutation	p.M244V	Non-Targeted Panel Gene	Adult HGBCL, NOS
BCL-84	chr8	89971232	89971232	G	A	exonic	NBN	38	40	0.5128	Missense_Mutation	p.R133W	Non-Targeted Panel Gene	Adult HGBCL, NOS
BCL-84	chr16	30724027	30724027	C	G	exonic	SRCAP	116	123	0.5146	Missense_Mutation	p.P1535A	Non-Targeted Panel Gene	Adult HGBCL, NOS
BCL-84	chr2	184938847	184938847	G	A	exonic	ZNF804A	45	48	0.5161	Missense_Mutation	p.V1151I	Non-Targeted Panel Gene	Adult HGBCL, NOS
BCL-84	chr11	15245966	15245966	T	C	exonic	INSC	39	43	0.5244	Missense_Mutation	p.F544L	Non-Targeted Panel Gene	Adult HGBCL, NOS
BCL-84	chr11	94545591	94545591	C	A	exonic	FUT4	107	120	0.5263	Missense_Mutation	p.F486L	Non-Targeted Panel Gene	Adult HGBCL, NOS
BCL-84	chr11	55820314	55820314	A	G	exonic	OR5D18	26	29	0.5273	Missense_Mutation	p.M229V	Non-Targeted Panel Gene	Adult HGBCL, NOS
BCL-84	chr4	88697554	88697554	A	T	exonic	NAP1L5	59	67	0.5317	Missense_Mutation	p.N67K	Non-Targeted Panel Gene	Adult HGBCL, NOS
BCL-84	chr12	6937198	6937198	C	A	exonic	ATN1	85	97	0.533	Missense_Mutation	p.P644Q	Non-Targeted Panel Gene	Adult HGBCL, NOS
BCL-84	chr3	134608706	134608706	C	T	exonic	KY	36	42	0.5385	Missense_Mutation	p.E345K	Non-Targeted Panel Gene	Adult HGBCL, NOS
BCL-84	chr20	25079507	25079507	G	C	exonic	VSX1	14	17	0.5484	Missense_Mutation	p.D144E	Non-Targeted Panel Gene	Adult HGBCL, NOS
BCL-84	chr10	116674941	116674941	G	A	exonic	HSPA12A	42	54	0.5567	Missense_Mutation	p.T640M	Non-Targeted Panel Gene	Adult HGBCL, NOS
BCL-84	chr15	100341197	100341197	G	A	exonic	ADAMTS17	27	34	0.5574	Missense_Mutation	p.R98C	Non-Targeted Panel Gene	Adult HGBCL, NOS
BCL-84	chr16	88864265	88864265	G	A	exonic	PABPN1L	28	39	0.5821	Missense_Mutation	p.P257S	Non-Targeted Panel Gene	Adult HGBCL, NOS
BCL-84	chr4	57110314	57110314	G	A	exonic	IGFBP7	15	23	0.6053	Missense_Mutation	p.A13V	Non-Targeted Panel Gene	Adult HGBCL, NOS
BCL-84	chr19	58294279	58294279	G	T	exonic	ZNF8	31	56	0.6437	Missense_Mutation	p.K157N	Non-Targeted Panel Gene	Adult HGBCL, NOS
BCL-84	chr9	101476957	101476957	G	A	exonic	TMEM246	8	21	0.7241	Missense_Mutation	p.L46F	Non-Targeted Panel Gene	Adult HGBCL, NOS
BCL-84	chr5	134359941	134359941	G	A	exonic	CDKL3	13	40	0.7547	Missense_Mutation	p.L106F	Non-Targeted Panel Gene	Adult HGBCL, NOS
BCL-84	chr5	109817415	109817415	A	T	exonic	MAN2A1	15	48	0.7619	Missense_Mutation	p.T696S	Non-Targeted Panel Gene	Adult HGBCL, NOS
BCL-84	chr5	170234152	170234152	A	G	exonic	C5orf58	10	43	0.8113	Missense_Mutation	p.E6G	Non-Targeted Panel Gene	Adult HGBCL, NOS
BCL-84	chr5	135578504	135578504	T	A	exonic	CXCL14	7	48	0.8727	Missense_Mutation	p.I46F	Non-Targeted Panel Gene	Adult HGBCL, NOS
BCL-84	chr14	104701648	104701648	G	A	exonic	INF2	14	113	0.8898	Missense_Mutation	p.A95T	Non-Targeted Panel Gene	Adult HGBCL, NOS
BCL-84	chr19	38141326	38141326	T	G	exonic	SIPA1L3	5	44	0.898	Missense_Mutation	p.S1096A	Non-Targeted Panel Gene	Adult HGBCL, NOS
BCL-84	chr3	169116394	169116394	C	G	exonic	MECOM	4	48	0.9231	Missense_Mutation	p.G493A	Non-Targeted Panel Gene	Adult HGBCL, NOS
BCL-84	chr5	58459940	58459940	A	G	exonic	PLK2	5	62	0.9254	Missense_Mutation	p.I7T	Non-Targeted Panel Gene	Adult HGBCL, NOS
BCL-84	chr1	158653323	158653323	G	A	exonic	SPTA1	36	20	0.3571	Nonsense_Mutation	p.R1047X	Non-Targeted Panel Gene	Adult HGBCL, NOS
BCL-84	chr11	121606868	121606868	C	T	exonic	SORL1	41	30	0.4225	Nonsense_Mutation	p.Q1658X	Non-Targeted Panel Gene	Adult HGBCL, NOS
BCL-84	chr3	189789831	189789831	C	T	exonic	TP63	16	12	0.4286	Nonsense_Mutation	p.Q11X	Non-Targeted Panel Gene	Adult HGBCL, NOS
BCL-84	chr19	8856053	8856053	A	C	exonic	MUC16	34	28	0.4516	Nonsense_Mutation	p.Y14408X	Non-Targeted Panel Gene	Adult HGBCL, NOS
BCL-84	chr4	76897588	76897588	G	A	exonic	SOWAHB	73	62	0.4593	Nonsense_Mutation	p.R88X	Non-Targeted Panel Gene	Adult HGBCL, NOS

BCL-84	chr16	4694185	4694185	C	T	exonic	NUDT16L1	85	90	0.5143	Nonsense_Mutation	p.Q121X	Non-Targeted Panel Gene	Adult HGBCL, NOS
BCL-85	chr7	124929793	124929793	C	T	splicing	POT1	34	19	0.3585	Splice_Site	.	Targerted Panel Gene	Adult HGBCL, NOS
BCL-85	chr3	53745877	53745879	TGA	-	splicing	CACNA1D	37	17	0.3148	Splice_Site	.	Non-Targeted Panel Gene	Adult HGBCL, NOS
BCL-85	chr7	127701161	127701161	A	G	splicing	SND1	48	24	0.3333	Splice_Site	.	Non-Targeted Panel Gene	Adult HGBCL, NOS
BCL-85	chr2	232815636	232815636	G	A	splicing	GIGYF2	44	28	0.3889	Splice_Site	.	Non-Targeted Panel Gene	Adult HGBCL, NOS
BCL-85	chr11	34134511	34134511	G	A	splicing	NAT10	79	56	0.4148	Splice_Site	.	Non-Targeted Panel Gene	Adult HGBCL, NOS
BCL-85	chr15	80122802	80122802	T	G	splicing	ZFAND6	39	34	0.4658	Splice_Site	.	Non-Targeted Panel Gene	Adult HGBCL, NOS
BCL-85	chr15	42811686	42811686	A	G	splicing	TTBK2	14	20	0.5882	Splice_Site	.	Non-Targeted Panel Gene	Adult HGBCL, NOS
BCL-85	chr3	186785882	186785882	G	C	splicing	EIF4A2	7	11	0.6111	Splice_Site	.	Non-Targeted Panel Gene	Adult HGBCL, NOS
BCL-85	chr8	102257714	102257739	TCTCCTAAGAATGA	-	exonic	UBR5	23	19	0.4524	Frame_Shift_Del	p.G2701fs	Targerted Panel Gene	Adult HGBCL, NOS
BCL-85	chr1	37587333	37587349	CCAAATCACGATCC	-	exonic	GNL2	47	26	0.3562	Frame_Shift_Del	p.G177fs	Non-Targeted Panel Gene	Adult HGBCL, NOS
BCL-85	chr1	153958406	153958406	T	-	exonic	CRTC2	141	104	0.4245	Frame_Shift_Del	p.Q31fs	Non-Targeted Panel Gene	Adult HGBCL, NOS
BCL-85	chr20	62812951	62812951	C	-	exonic	OGFR	117	87	0.4265	Frame_Shift_Del	p.P446fs	Non-Targeted Panel Gene	Adult HGBCL, NOS
BCL-85	chrX	1466425	1466426	AG	-	exonic	P2RY8	29	73	0.7157	Frame_Shift_Del	p.L45fs	Non-Targeted Panel Gene	Adult HGBCL, NOS
BCL-85	chr17	21535314	21535316	AAG	-	exonic	C17orf51	37	21	0.3621	In_Frame_Del	p.217_218del	Non-Targeted Panel Gene	Adult HGBCL, NOS
BCL-85	chr17	79795290	79795301	CGCTCCCTCTCT	-	exonic	CBX8	177	116	0.3959	In_Frame_Del	p.168_172del	Non-Targeted Panel Gene	Adult HGBCL, NOS
BCL-85	chr18	26548345	26548345	-	TCCTCCTCCTCCTC	exonic	KCTD1	70	19	0.2088	In_Frame_Ins	p.D64delinsEEEEE	Non-Targeted Panel Gene	Adult HGBCL, NOS
BCL-85	chr5	71509940	71509940	C	G	exonic	BDP1	65	4	0.058	Missense_Mutation	p.L950V	Targerted Panel Gene	Adult HGBCL, NOS
BCL-85	chr5	71509949	71509949	G	A	exonic	BDP1	61	5	0.0758	Missense_Mutation	p.V953M	Targerted Panel Gene	Adult HGBCL, NOS
BCL-85	chr4	84794587	84794587	G	T	exonic	WDFY3	15	6	0.2857	Missense_Mutation	p.A1140E	Targerted Panel Gene	Adult HGBCL, NOS
BCL-85	chr17	39777710	39777710	A	G	exonic	IKZF3	41	34	0.4533	Missense_Mutation	p.L9P	Targerted Panel Gene	Adult HGBCL, NOS
BCL-85	chr6	41935950	41935950	A	T	exonic	CCND3	111	98	0.4689	Missense_Mutation	p.I290K	Targerted Panel Gene	Adult HGBCL, NOS
BCL-85	chr4	125319449	125319449	C	A	exonic	FAT4	22	23	0.5111	Missense_Mutation	p.S1013Y	Targerted Panel Gene	Adult HGBCL, NOS
BCL-85	chr7	111834674	111834674	C	T	exonic	DOCK4	10	11	0.5238	Missense_Mutation	p.A917T	Targerted Panel Gene	Adult HGBCL, NOS
BCL-85	chr15	42693121	42693121	C	T	exonic	STARD9	66	78	0.5417	Missense_Mutation	p.P3848L	Targerted Panel Gene	Adult HGBCL, NOS
BCL-85	chr22	22888214	22888214	C	T	exonic	IGLL5	45	56	0.5545	Missense_Mutation	p.P19S	Targerted Panel Gene	Adult HGBCL, NOS
BCL-85	chr5	154901386	154901386	G	T	exonic	GEMIN5	39	2	0.0488	Missense_Mutation	p.H989Q	Non-Targeted Panel Gene	Adult HGBCL, NOS
BCL-85	chr19	21971195	21971195	A	G	exonic	ZNF208	75	4	0.0506	Missense_Mutation	p.L1280P	Non-Targeted Panel Gene	Adult HGBCL, NOS
BCL-85	chr6	131621976	131621976	C	A	exonic	MED23	12	2	0.1429	Missense_Mutation	p.V134F	Non-Targeted Panel Gene	Adult HGBCL, NOS
BCL-85	chr5	132660276	132660276	C	A	exonic	IL13	156	27	0.1475	Missense_Mutation	p.F145L	Non-Targeted Panel Gene	Adult HGBCL, NOS
BCL-85	chr8	139988817	139988817	G	A	exonic	TRAPPC9	34	7	0.1707	Missense_Mutation	p.L1005F	Non-Targeted Panel Gene	Adult HGBCL, NOS
BCL-85	chr2	231037987	231037987	C	T	exonic	C2orf72	9	2	0.1818	Missense_Mutation	p.A141V	Non-Targeted Panel Gene	Adult HGBCL, NOS
BCL-85	chr1	47438839	47438839	C	T	exonic	FOXD2	119	36	0.2308	Missense_Mutation	p.P235L	Non-Targeted Panel Gene	Adult HGBCL, NOS
BCL-85	chr1	171064256	171064256	G	T	exonic	MROH9	30	11	0.2683	Missense_Mutation	p.K834N	Non-Targeted Panel Gene	Adult HGBCL, NOS
BCL-85	chr17	64527198	64527198	A	C	exonic	CEP95	22	9	0.2903	Missense_Mutation	p.T414P	Non-Targeted Panel Gene	Adult HGBCL, NOS
BCL-85	chr17	81642311	81642311	G	A	exonic	TSPAN10	64	30	0.3191	Missense_Mutation	p.A5T	Non-Targeted Panel Gene	Adult HGBCL, NOS
BCL-85	chr18	21440308	21440308	G	A	exonic	GREB1L	45	23	0.3382	Missense_Mutation	p.R330Q	Non-Targeted Panel Gene	Adult HGBCL, NOS
BCL-85	chr4	25862739	25862739	C	T	exonic	SEL1L3	37	19	0.3393	Missense_Mutation	p.S33N	Non-Targeted Panel Gene	Adult HGBCL, NOS
BCL-85	chr3	129586179	129586179	C	G	exonic	PLXND1	146	75	0.3394	Missense_Mutation	p.E572Q	Non-Targeted Panel Gene	Adult HGBCL, NOS
BCL-85	chr12	8936886	8936886	G	A	exonic	PHC1	98	54	0.3553	Missense_Mutation	p.C800Y	Non-Targeted Panel Gene	Adult HGBCL, NOS
BCL-85	chr10	65988789	65988789	C	G	exonic	CTNNA3	37	21	0.3621	Missense_Mutation	p.G723A	Non-Targeted Panel Gene	Adult HGBCL, NOS
BCL-85	chr4	84604246	84604246	A	G	exonic	CDS1	19	11	0.3667	Missense_Mutation	p.T41A	Non-Targeted Panel Gene	Adult HGBCL, NOS
BCL-85	chr15	64816744	64816744	C	T	exonic	PIF1	106	68	0.3886	Missense_Mutation	p.E566K	Non-Targeted Panel Gene	Adult HGBCL, NOS
BCL-85	chr1	108659876	108659876	T	G	exonic	HENMT1	50	32	0.3902	Missense_Mutation	p.E3D	Non-Targeted Panel Gene	Adult HGBCL, NOS
BCL-85	chr6	31355407	31355407	C	T	exonic	HLA-B	98	64	0.3951	Missense_Mutation	p.A269T	Non-Targeted Panel Gene	Adult HGBCL, NOS
BCL-85	chr2	64636022	64636022	C	G	exonic	SERTAD2	87	57	0.3958	Missense_Mutation	p.A284P	Non-Targeted Panel Gene	Adult HGBCL, NOS
BCL-85	chr12	6952156	6952156	G	A	exonic	PTPN6	201	132	0.3964	Missense_Mutation	p.C102Y	Non-Targeted Panel Gene	Adult HGBCL, NOS
BCL-85	chr6	33069734	33069734	A	C	exonic	HLA-DPA1	59	39	0.398	Missense_Mutation	p.F85V	Non-Targeted Panel Gene	Adult HGBCL, NOS
BCL-85	chr17	42323307	42323307	A	T	exonic	STAT3	71	48	0.4034	Missense_Mutation	p.N567K	Non-Targeted Panel Gene	Adult HGBCL, NOS
BCL-85	chr17	78050633	78050633	G	T	exonic	TNRC6C	133	90	0.4036	Missense_Mutation	p.G524V	Non-Targeted Panel Gene	Adult HGBCL, NOS
BCL-85	chr17	75830142	75830142	T	G	exonic	UNC13D	86	59	0.4069	Missense_Mutation	p.D947A	Non-Targeted Panel Gene	Adult HGBCL, NOS
BCL-85	chr19	2050837	2050837	T	G	exonic	MKNK2	496	342	0.4076	Missense_Mutation	p.K5N	Non-Targeted Panel Gene	Adult HGBCL, NOS
BCL-85	chr12	6960204	6960204	A	G	exonic	PTPN6	155	107	0.4084	Missense_Mutation	p.I516V	Non-Targeted Panel Gene	Adult HGBCL, NOS
BCL-85	chr12	6960163	6960163	A	T	exonic	PTPN6	177	124	0.412	Missense_Mutation	p.E502V	Non-Targeted Panel Gene	Adult HGBCL, NOS
BCL-85	chr12	6960344	6960344	T	C	exonic	PTPN6	217	154	0.4151	Missense_Mutation	p.S528P	Non-Targeted Panel Gene	Adult HGBCL, NOS
BCL-85	chr10	43106556	43106556	A	G	exonic	RET	112	80	0.4167	Missense_Mutation	p.T350A	Non-Targeted Panel Gene	Adult HGBCL, NOS
BCL-85	chr19	10224427	10224427	A	G	exonic	S1PR2	175	125	0.4167	Missense_Mutation	p.L160P	Non-Targeted Panel Gene	Adult HGBCL, NOS
BCL-85	chr12	6960171	6960171	T	A	exonic	PTPN6	172	124	0.4189	Missense_Mutation	p.Y505N	Non-Targeted Panel Gene	Adult HGBCL, NOS
BCL-85	chr19	8511494	8511494	G	A	exonic	ZNF414	161	119	0.425	Missense_Mutation	p.A306V	Non-Targeted Panel Gene	Adult HGBCL, NOS
BCL-85	chr7	148760428	148760428	G	C	exonic	CUL1	31	23	0.4259	Missense_Mutation	p.E241Q	Non-Targeted Panel Gene	Adult HGBCL, NOS
BCL-85	chr17	45137740	45137740	C	T	exonic	ACBD4	99	74	0.4277	Missense_Mutation	p.T174I	Non-Targeted Panel Gene	Adult HGBCL, NOS
BCL-85	chr1	32275649	32275649	T	C	exonic	LCK	125	95	0.4318	Missense_Mutation	p.I153T	Non-Targeted Panel Gene	Adult HGBCL, NOS
BCL-85	chr16	5025614	5025614	A	G	exonic	NAGPA	82	63	0.4345	Missense_Mutation	p.L471S	Non-Targeted Panel Gene	Adult HGBCL, NOS
BCL-85	chr3	156465684	156465684	A	G	exonic	KCNAB1	22	17	0.4359	Missense_Mutation	p.E190G	Non-Targeted Panel Gene	Adult HGBCL, NOS
BCL-85	chr12	6960224	6960224	G	C	exonic	PTPN6	146	114	0.4368	Missense_Mutation	p.K522N	Non-Targeted Panel Gene	Adult HGBCL, NOS
BCL-85	chr20	52152310	52152310	C	G	exonic	ZFP64	131	102	0.4378	Missense_Mutation	p.V626L	Non-Targeted Panel Gene	Adult HGBCL, NOS
BCL-85	chr19	4719877	4719877	G	T	exonic	DPP9	218	170	0.4381	Missense_Mutation	p.D10E	Non-Targeted Panel Gene	Adult HGBCL, NOS
BCL-85	chr4	144646882	144646882	C	G	exonic	HHIP	121	95	0.4398	Missense_Mutation	p.C69W	Non-Targeted Panel Gene	Adult HGBCL, NOS
BCL-85	chr19	56814405	56814405	A	G	exonic	PEG3	34	27	0.4426	Missense_Mutation	p.V1346A	Non-Targeted Panel Gene	Adult HGBCL, NOS
BCL-85	chr12	53713756	53713756	G	T	exonic	CALCOCO1	126	101	0.443	Missense_Mutation	p.P579Q	Non-Targeted Panel Gene	Adult HGBCL, NOS
BCL-85	chr21	32347154	32347154	C	A	exonic	URB1	94	75	0.4438	Missense_Mutation	p.A1224S	Non-Targeted Panel Gene	Adult HGBCL, NOS
BCL-85	chr9	103005318	103005318	T	A	exonic	CYLC2	16	13	0.4483	Missense_Mutation	p.D229E	Non-Targeted Panel Gene	Adult HGBCL, NOS
BCL-85	chr3	14468134	14468134	T	C	exonic	SLC6A6	28	23	0.451	Missense_Mutation	p.S340P	Non-Targeted Panel Gene	Adult HGBCL, NOS
BCL-85	chr14	102086227	102086227	T	A	exonic	HSP90AA1	63	53	0.4569	Missense_Mutation	p.N173I	Non-Targeted Panel Gene	Adult HGBCL, NOS
BCL-85	chr12	6912889	6912889	C	A	exonic	LRRC23	162	141	0.4653	Missense_Mutation	p.F306L	Non-Targeted Panel Gene	Adult HGBCL, NOS
BCL-85	chr2	38986117	38986117	G	C	exonic	SOS1	47	41	0.4659	Missense_Mutation	p.P1237A	Non-Targeted Panel Gene	Adult HGBCL, NOS
BCL-85	chr5	180119355	180119355	C	G	exonic	RASGEF1C	90	79	0.4675	Missense_Mutation	p.A300P	Non-Targeted Panel Gene	Adult HGBCL, NOS
BCL-85	chr15	55359534	55359534	C	T	exonic	CCPG1	46	41	0.4713	Missense_Mutation	p.V747I	Non-Targeted Panel Gene	Adult HGBCL, NOS
BCL-85	chr1	161819701	161819701	G	T	exonic	ATF6	19	17	0.4722	Missense_Mutation	p.K326N	Non-Targeted Panel Gene	Adult HGBCL, NOS
BCL-85	chr17	31326019	31326019	A	G	exonic	NF1	49	44	0.4731	Missense_Mutation	p.I1679V	Non-Targeted Panel Gene	Adult HGBCL, NOS
BCL-85	chr9	131519447	131519447	C	G	exonic	POMT1	235	213	0.4744	Missense_Mutation	p.S537R	Non-Targeted Panel Gene	Adult HGBCL, NOS
BCL-85	chr4	443895	443895	T	C	exonic	ZNF721	62	56	0.4746	Missense_Mutation	p.K191R	Non-Targeted Panel Gene	Adult HGBCL, NOS

BCL-85	chr19	35009229	35009229	T	G	exonic	GRAMD1A	166	150	0.4747	Missense_Mutation	p.M127R	Non-Targeted Panel Gene	Adult HGBCL, NOS
BCL-85	chr19	45409103	45409103	G	A	exonic	CD3EAP	73	66	0.4748	Missense_Mutation	p.A379T	Non-Targeted Panel Gene	Adult HGBCL, NOS
BCL-85	chr15	40620775	40620775	A	G	exonic	KNL1	74	68	0.4789	Missense_Mutation	p.S197G	Non-Targeted Panel Gene	Adult HGBCL, NOS
BCL-85	chr12	6959935	6959935	T	C	exonic	PTPN6	89	82	0.4795	Missense_Mutation	p.I457T	Non-Targeted Panel Gene	Adult HGBCL, NOS
BCL-85	chr1	46034251	46034251	G	A	exonic	MAST2	245	227	0.4809	Missense_Mutation	p.D1292N	Non-Targeted Panel Gene	Adult HGBCL, NOS
BCL-85	chr15	64387933	64387933	T	C	exonic	TRIP4	85	79	0.4817	Missense_Mutation	p.F24L	Non-Targeted Panel Gene	Adult HGBCL, NOS
BCL-85	chr1	247815568	247815568	G	T	exonic	OR14A16	16	15	0.4839	Missense_Mutation	p.H54Q	Non-Targeted Panel Gene	Adult HGBCL, NOS
BCL-85	chr3	121808954	121808954	A	T	exonic	IQCB1	16	16	0.4848	Missense_Mutation	p.F150Y	Non-Targeted Panel Gene	Adult HGBCL, NOS
BCL-85	chr2	185800190	185800190	T	G	exonic	FSIP2	20	19	0.4872	Missense_Mutation	p.N3628K	Non-Targeted Panel Gene	Adult HGBCL, NOS
BCL-85	chr4	183282897	183282897	A	G	exonic	WWC2	23	22	0.4889	Missense_Mutation	p.I958M	Non-Targeted Panel Gene	Adult HGBCL, NOS
BCL-85	chr12	53954982	53954982	T	G	exonic	HOXC12	259	252	0.4932	Missense_Mutation	p.I18S	Non-Targeted Panel Gene	Adult HGBCL, NOS
BCL-85	chr11	61962709	61962709	A	C	exonic	BEST1	52	51	0.4951	Missense_Mutation	p.S519R	Non-Targeted Panel Gene	Adult HGBCL, NOS
BCL-85	chr11	61345484	61345484	C	T	exonic	TKFC	159	158	0.4984	Missense_Mutation	p.A457V	Non-Targeted Panel Gene	Adult HGBCL, NOS
BCL-85	chr4	42616053	42616053	A	C	exonic	ATP8A1	27	27	0.5	Missense_Mutation	p.V130G	Non-Targeted Panel Gene	Adult HGBCL, NOS
BCL-85	chr11	55968435	55968435	T	A	exonic	OR10AG1	17	17	0.5	Missense_Mutation	p.D10V	Non-Targeted Panel Gene	Adult HGBCL, NOS
BCL-85	chr19	53238013	53238013	A	C	exonic	ZNF677	35	35	0.5	Missense_Mutation	p.N238K	Non-Targeted Panel Gene	Adult HGBCL, NOS
BCL-85	chr8	23682682	23682682	C	G	exonic	NKX3-1	85	87	0.5058	Missense_Mutation	p.G70R	Non-Targeted Panel Gene	Adult HGBCL, NOS
BCL-85	chr3	44450456	44450456	C	T	exonic	ZNF445	23	24	0.5106	Missense_Mutation	p.A271T	Non-Targeted Panel Gene	Adult HGBCL, NOS
BCL-85	chr19	46411469	46411469	C	G	exonic	CCDC8	192	201	0.5115	Missense_Mutation	p.A448P	Non-Targeted Panel Gene	Adult HGBCL, NOS
BCL-85	chr16	67435754	67435754	G	A	exonic	HSD11B2	177	202	0.533	Missense_Mutation	p.R131H	Non-Targeted Panel Gene	Adult HGBCL, NOS
BCL-85	chr1	145838082	145838082	T	G	exonic	POLR3C	28	32	0.5333	Missense_Mutation	p.F366C	Non-Targeted Panel Gene	Adult HGBCL, NOS
BCL-85	chr17	66735559	66735559	T	C	exonic	PRKCA	31	36	0.5373	Missense_Mutation	p.I376T	Non-Targeted Panel Gene	Adult HGBCL, NOS
BCL-85	chr7	7969402	7969402	C	G	exonic	GLCCI1	115	134	0.5382	Missense_Mutation	p.P18A	Non-Targeted Panel Gene	Adult HGBCL, NOS
BCL-85	chr2	111142390	111142390	A	C	exonic	BCL2L11	15	18	0.5455	Missense_Mutation	p.S98R	Non-Targeted Panel Gene	Adult HGBCL, NOS
BCL-85	chr19	43750671	43750671	C	G	exonic	SMG9	43	53	0.5521	Missense_Mutation	p.G24A	Non-Targeted Panel Gene	Adult HGBCL, NOS
BCL-85	chr12	110647752	110647752	A	G	exonic	TCTN1	64	79	0.5524	Missense_Mutation	p.N364D	Non-Targeted Panel Gene	Adult HGBCL, NOS
BCL-85	chr4	137530174	137530174	G	A	exonic	PCDH18	33	41	0.5541	Missense_Mutation	p.H639Y	Non-Targeted Panel Gene	Adult HGBCL, NOS
BCL-85	chr3	109308143	109308143	C	T	exonic	DPPA2	23	31	0.5741	Missense_Mutation	p.G183R	Non-Targeted Panel Gene	Adult HGBCL, NOS
BCL-85	chr3	130455618	130455618	A	G	exonic	COL6A5	5	7	0.5833	Missense_Mutation	p.N2248D	Non-Targeted Panel Gene	Adult HGBCL, NOS
BCL-85	chr4	8587196	8587196	G	T	exonic	GPR78	152	214	0.5847	Missense_Mutation	p.V309L	Non-Targeted Panel Gene	Adult HGBCL, NOS
BCL-85	chr1	2518201	2518201	G	A	exonic	PANK4	32	126	0.7975	Missense_Mutation	p.P402L	Non-Targeted Panel Gene	Adult HGBCL, NOS
BCL-85	chrX	104114968	104114968	A	G	exonic	ZCCHC18	9	36	0.8	Missense_Mutation	p.Y286C	Non-Targeted Panel Gene	Adult HGBCL, NOS
BCL-85	chr6	32442535	32442535	T	C	exonic	HLA-DRA	6	39	0.8667	Missense_Mutation	p.F57S	Non-Targeted Panel Gene	Adult HGBCL, NOS
BCL-85	chr6	32665037	32665037	C	T	exonic	HLA-DQB1	9	75	0.8929	Missense_Mutation	p.C47Y	Non-Targeted Panel Gene	Adult HGBCL, NOS
BCL-85	chr6	142370297	142370297	A	C	exonic	ADGRG6	3	36	0.9231	Missense_Mutation	p.K191N	Non-Targeted Panel Gene	Adult HGBCL, NOS
BCL-85	chr4	93110873	93110873	G	T	exonic	GRID2	30	6	0.1667	Nonsense_Mutation	p.E219X	Targerted Panel Gene	Adult HGBCL, NOS
BCL-86	chr4	42444576	42444577	AC	-	splicing	ATP8A1	47	2	0.0408	Splice_Site	.	Non-Targeted Panel Gene	Adult HGBCL, NOS
BCL-86	chr12	8839112	8839112	G	A	splicing	A2ML1	18	6	0.25	Splice_Site	.	Non-Targeted Panel Gene	Adult HGBCL, NOS
BCL-86	chr7	89218153	89218153	A	G	splicing	ZNF804B	17	12	0.4138	Splice_Site	.	Non-Targeted Panel Gene	Adult HGBCL, NOS
BCL-86	chr12	55726698	55726698	A	C	splicing	CD63	12	17	0.5862	Splice_Site	.	Non-Targeted Panel Gene	Adult HGBCL, NOS
BCL-86	chr1	11114841	11114841	A	-	exonic	MTOR	27	2	0.069	Frame_Shift_Del	p.L2379fs	Targerted Panel Gene	Adult HGBCL, NOS
BCL-86	chr2	174759355	174759356	CA	-	exonic	CHRNA1	49	2	0.0392	Frame_Shift_Del	p.V70fs	Non-Targeted Panel Gene	Adult HGBCL, NOS
BCL-86	chr1	173873492	173873493	CC	-	exonic	ZBTB37	48	2	0.04	Frame_Shift_Del	p.P317fs	Non-Targeted Panel Gene	Adult HGBCL, NOS
BCL-86	chr5	10433631	10433631	A	-	exonic	MARCHF6	34	2	0.0556	Frame_Shift_Del	p.K894fs	Non-Targeted Panel Gene	Adult HGBCL, NOS
BCL-86	chr2	197470077	197470077	G	-	exonic	COQ10B	32	2	0.0588	Frame_Shift_Del	p.C152fs	Non-Targeted Panel Gene	Adult HGBCL, NOS
BCL-86	chr1	61769360	61769360	T	-	exonic	PATJ	22	2	0.0833	Frame_Shift_Del	p.S154fs	Non-Targeted Panel Gene	Adult HGBCL, NOS
BCL-86	chr13	48512757	48512757	T	-	exonic	RCBTB2	20	2	0.0909	Frame_Shift_Del	p.H163fs	Non-Targeted Panel Gene	Adult HGBCL, NOS
BCL-86	chr6	44406340	44406341	AT	-	exonic	CDC5L	16	6	0.2727	Frame_Shift_Del	p.D259fs	Non-Targeted Panel Gene	Adult HGBCL, NOS
BCL-86	chr3	105867420	105867420	A	-	exonic	CBLB	20	8	0.2857	Frame_Shift_Del	p.M53fs	Non-Targeted Panel Gene	Adult HGBCL, NOS
BCL-86	chr9	76703791	76703795	CTTTT	-	exonic	PRUNE2	15	16	0.5161	Frame_Shift_Del	p.R2606fs	Non-Targeted Panel Gene	Adult HGBCL, NOS
BCL-86	chr1	235160560	235160560	-	T	exonic	RBM34	16	27	0.6136	Frame_Shift_Ins	p.V106fs	Non-Targeted Panel Gene	Adult HGBCL, NOS
BCL-86	chr9	70863063	70863068	TACTGG	-	exonic	TRPM3	32	2	0.0588	In_Frame_Del	p.101_103del	Non-Targeted Panel Gene	Adult HGBCL, NOS
BCL-86	chr1	28274156	28274156	-	GGA	exonic	SESN2	70	4	0.0541	In_Frame_Ins	p.Q340delinsRK	Non-Targeted Panel Gene	Adult HGBCL, NOS
BCL-86	chr3	38140541	38140541	C	G	exonic	MYD88	58	4	0.0645	Missense_Mutation	p.S219C	Targerted Panel Gene	Adult HGBCL, NOS
BCL-86	chr19	23361838	23361838	T	C	exonic	ZNF91	60	4	0.0625	Missense_Mutation	p.K381E	Targerted Panel Gene	Adult HGBCL, NOS
BCL-86	chr1	11114842	11114842	G	T	exonic	MTOR	27	2	0.069	Missense_Mutation	p.L2379I	Targerted Panel Gene	Adult HGBCL, NOS
BCL-86	chr11	117432393	117432393	G	C	exonic	DSCAML1	25	2	0.0741	Missense_Mutation	p.T1773R	Targerted Panel Gene	Adult HGBCL, NOS
BCL-86	chr6	128089759	128089759	T	G	exonic	PTPRK	46	11	0.193	Missense_Mutation	p.K466Q	Targerted Panel Gene	Adult HGBCL, NOS
BCL-86	chr19	30445130	30445130	C	T	exonic	ZNF536	69	29	0.2959	Missense_Mutation	p.A523V	Targerted Panel Gene	Adult HGBCL, NOS
BCL-86	chr18	67511241	67511241	T	G	exonic	DSEL	20	9	0.3103	Missense_Mutation	p.Q1133P	Targerted Panel Gene	Adult HGBCL, NOS
BCL-86	chr1	155337725	155337725	C	T	exonic	ASH1L	28	19	0.4043	Missense_Mutation	p.E2944K	Targerted Panel Gene	Adult HGBCL, NOS
BCL-86	chr9	127508457	127508457	T	G	exonic	FAM129B	100	108	0.5192	Missense_Mutation	p.K467Q	Targerted Panel Gene	Adult HGBCL, NOS
BCL-86	chr20	32436647	32436647	C	T	exonic	ASXL1	22	31	0.5849	Missense_Mutation	p.A1312V	Targerted Panel Gene	Adult HGBCL, NOS
BCL-86	chrX	53076832	53076832	C	T	exonic	GPR173	97	2	0.0202	Missense_Mutation	p.R71C	Non-Targeted Panel Gene	Adult HGBCL, NOS
BCL-86	chr2	80303049	80303049	G	T	exonic	LRRTM1	105	5	0.0455	Missense_Mutation	p.N257K	Non-Targeted Panel Gene	Adult HGBCL, NOS
BCL-86	chrX	13767234	13767234	G	A	exonic	OFD1	42	2	0.0455	Missense_Mutation	p.E763K	Non-Targeted Panel Gene	Adult HGBCL, NOS
BCL-86	chr9	129868898	129868898	G	T	exonic	USP20	41	2	0.0465	Missense_Mutation	p.R391L	Non-Targeted Panel Gene	Adult HGBCL, NOS
BCL-86	chr7	122011964	122011964	A	C	exonic	PTPRZ1	92	5	0.051	Missense_Mutation	p.K973T	Non-Targeted Panel Gene	Adult HGBCL, NOS
BCL-86	chr19	18146994	18146994	C	A	exonic	MAST3	55	3	0.0517	Missense_Mutation	p.S1063R	Non-Targeted Panel Gene	Adult HGBCL, NOS
BCL-86	chrX	125321791	125321791	A	G	exonic	TEX13C	127	7	0.0522	Missense_Mutation	p.I558V	Non-Targeted Panel Gene	Adult HGBCL, NOS
BCL-86	chr11	124987931	124987931	G	C	exonic	CCDC15	35	2	0.0526	Missense_Mutation	p.V569L	Non-Targeted Panel Gene	Adult HGBCL, NOS
BCL-86	chr1	151133990	151133990	G	C	exonic	SEMA6C	36	2	0.0526	Missense_Mutation	p.Q609E	Non-Targeted Panel Gene	Adult HGBCL, NOS
BCL-86	chr5	10433632	10433632	A	G	exonic	MARCHF6	34	2	0.0556	Missense_Mutation	p.K894R	Non-Targeted Panel Gene	Adult HGBCL, NOS
BCL-86	chr19	21294034	21294034	C	T	exonic	ZNF708	98	6	0.0577	Missense_Mutation	p.G311E	Non-Targeted Panel Gene	Adult HGBCL, NOS
BCL-86	chrX	1466057	1466057	G	T	exonic	P2RY8	248	16	0.0606	Missense_Mutation	p.H168N	Non-Targeted Panel Gene	Adult HGBCL, NOS
BCL-86	chr20	3046109	3046109	G	A	exonic	MRPS26	281	19	0.0633	Missense_Mutation	p.C14Y	Non-Targeted Panel Gene	Adult HGBCL, NOS
BCL-86	chr19	10224812	10224812	A	G	exonic	S1PR2	141	10	0.0662	Missense_Mutation	p.S32P	Non-Targeted Panel Gene	Adult HGBCL, NOS
BCL-86	chr6	22569820	22569820	T	G	exonic	HDGFL1	257	19	0.0688	Missense_Mutation	p.I82S	Non-Targeted Panel Gene	Adult HGBCL, NOS
BCL-86	chr19	10224884	10224884	A	G	exonic	S1PR2	94	7	0.0693	Missense_Mutation	p.Y8H	Non-Targeted Panel Gene	Adult HGBCL, NOS
BCL-86	chr3	190409977	190409977	T	C	exonic	CLDN16	49	4	0.0755	Missense_Mutation	p.S287P	Non-Targeted Panel Gene	Adult HGBCL, NOS
BCL-86	chr11	62531039	62531039	T	C	exonic	AHNAK	58	5	0.0794	Missense_Mutation	p.I1126M	Non-Targeted Panel Gene	Adult HGBCL, NOS
BCL-86	chr1	61769361	61769361	C	A	exonic	PATJ	22	2	0.08	Missense_Mutation	p.Q155K	Non-Targeted Panel Gene	Adult HGBCL, NOS

BCL-86	chr2	209849520	209849520	T	C	exonic	UNC80	46	4	0.08	Missense_Mutation	p.L1172P	Non-Targeted Panel Gene	Adult HGBCL, NOS
BCL-86	chr19	48881121	48881121	G	T	exonic	TULP2	34	3	0.0811	Missense_Mutation	p.H485N	Non-Targeted Panel Gene	Adult HGBCL, NOS
BCL-86	chr8	123506547	123506547	C	A	exonic	FBXO32	86	8	0.0851	Missense_Mutation	p.D227Y	Non-Targeted Panel Gene	Adult HGBCL, NOS
BCL-86	chr11	30911391	30911391	A	C	exonic	DCDC1	30	3	0.0909	Missense_Mutation	p.L847R	Non-Targeted Panel Gene	Adult HGBCL, NOS
BCL-86	chr13	48512758	48512758	G	A	exonic	RCBTB2	20	2	0.0909	Missense_Mutation	p.H163Y	Non-Targeted Panel Gene	Adult HGBCL, NOS
BCL-86	chr1	247949641	247949641	A	G	exonic	OR2L8	59	6	0.0923	Missense_Mutation	p.R262G	Non-Targeted Panel Gene	Adult HGBCL, NOS
BCL-86	chr12	77055944	77055944	C	T	exonic	E2F7	39	4	0.093	Missense_Mutation	p.A94T	Non-Targeted Panel Gene	Adult HGBCL, NOS
BCL-86	chr1	109281995	109281995	T	C	exonic	PSRC1	87	9	0.0938	Missense_Mutation	p.H48R	Non-Targeted Panel Gene	Adult HGBCL, NOS
BCL-86	chr19	15680518	15680518	T	G	exonic	CYP4F12	57	6	0.0952	Missense_Mutation	p.L175R	Non-Targeted Panel Gene	Adult HGBCL, NOS
BCL-86	chr3	73384123	73384123	G	A	exonic	PDZRN3	94	10	0.0962	Missense_Mutation	p.P815S	Non-Targeted Panel Gene	Adult HGBCL, NOS
BCL-86	chr8	120227281	120227281	A	T	exonic	COL14A1	18	2	0.1	Missense_Mutation	p.Y689F	Non-Targeted Panel Gene	Adult HGBCL, NOS
BCL-86	chr9	5810145	5810145	C	A	exonic	ERMP1	27	3	0.1	Missense_Mutation	p.A472S	Non-Targeted Panel Gene	Adult HGBCL, NOS
BCL-86	chrX	55452792	55452792	T	G	exonic	MAGEH1	53	6	0.1017	Missense_Mutation	p.F140V	Non-Targeted Panel Gene	Adult HGBCL, NOS
BCL-86	chr1	28987525	28987525	C	G	exonic	EPB41	26	3	0.1034	Missense_Mutation	p.Q30E	Non-Targeted Panel Gene	Adult HGBCL, NOS
BCL-86	chr13	48411484	48411484	A	C	exonic	LPAR6	64	8	0.1096	Missense_Mutation	p.F314V	Non-Targeted Panel Gene	Adult HGBCL, NOS
BCL-86	chr8	129879403	129879403	A	G	exonic	FAM49B	31	4	0.1143	Missense_Mutation	p.F20S	Non-Targeted Panel Gene	Adult HGBCL, NOS
BCL-86	chr6	70102183	70102183	A	T	exonic	COL19A1	15	2	0.1176	Missense_Mutation	p.K413N	Non-Targeted Panel Gene	Adult HGBCL, NOS
BCL-86	chr8	87237090	87237090	A	G	exonic	CNBD1	35	5	0.125	Missense_Mutation	p.Y250C	Non-Targeted Panel Gene	Adult HGBCL, NOS
BCL-86	chr12	124960409	124960409	G	A	exonic	DHX37	14	2	0.125	Missense_Mutation	p.A687V	Non-Targeted Panel Gene	Adult HGBCL, NOS
BCL-86	chrX	79171188	79171188	T	G	exonic	GPR174	49	7	0.125	Missense_Mutation	p.L61V	Non-Targeted Panel Gene	Adult HGBCL, NOS
BCL-86	chr11	56275861	56275861	C	A	exonic	OR5T1	49	7	0.125	Missense_Mutation	p.L75I	Non-Targeted Panel Gene	Adult HGBCL, NOS
BCL-86	chr7	117592085	117592085	T	G	exonic	CFTR	76	11	0.1264	Missense_Mutation	p.F640V	Non-Targeted Panel Gene	Adult HGBCL, NOS
BCL-86	chr3	186584531	186584531	G	T	exonic	DNAJB11	23	4	0.1481	Missense_Mutation	p.L318F	Non-Targeted Panel Gene	Adult HGBCL, NOS
BCL-86	chrX	43780357	43780357	A	G	exonic	MAOB	11	2	0.1538	Missense_Mutation	p.L355P	Non-Targeted Panel Gene	Adult HGBCL, NOS
BCL-86	chr7	89334877	89334877	C	T	exonic	ZNF804B	44	8	0.1538	Missense_Mutation	p.S632F	Non-Targeted Panel Gene	Adult HGBCL, NOS
BCL-86	chr5	141303030	141303030	G	A	exonic	SLC25A2	10	2	0.1667	Missense_Mutation	p.A279V	Non-Targeted Panel Gene	Adult HGBCL, NOS
BCL-86	chr7	90236919	90236919	C	A	exonic	STEAP2	110	22	0.1667	Missense_Mutation	p.D454E	Non-Targeted Panel Gene	Adult HGBCL, NOS
BCL-86	chrX	88753698	88753698	C	T	exonic	CPXCR1	28	7	0.2	Missense_Mutation	p.P95L	Non-Targeted Panel Gene	Adult HGBCL, NOS
BCL-86	chrX	139561625	139561625	C	A	exonic	F9	44	15	0.2542	Missense_Mutation	p.H314N	Non-Targeted Panel Gene	Adult HGBCL, NOS
BCL-86	chr7	150998438	150998438	G	A	exonic	NOS3	119	41	0.2562	Missense_Mutation	p.G222S	Non-Targeted Panel Gene	Adult HGBCL, NOS
BCL-86	chr6	147509013	147509013	G	A	exonic	SAMD5	329	130	0.2832	Missense_Mutation	p.D29N	Non-Targeted Panel Gene	Adult HGBCL, NOS
BCL-86	chr6	127719801	127719801	T	G	exonic	THEMIS	29	12	0.2927	Missense_Mutation	p.K497T	Non-Targeted Panel Gene	Adult HGBCL, NOS
BCL-86	chr11	55812333	55812333	C	G	exonic	OR5L1	47	20	0.2985	Missense_Mutation	p.I289M	Non-Targeted Panel Gene	Adult HGBCL, NOS
BCL-86	chr13	110484935	110484935	C	T	exonic	COL4A2	16	7	0.3043	Missense_Mutation	p.P978L	Non-Targeted Panel Gene	Adult HGBCL, NOS
BCL-86	chr4	112562425	112562425	A	T	exonic	ZGRF1	16	7	0.3043	Missense_Mutation	p.I1548N	Non-Targeted Panel Gene	Adult HGBCL, NOS
BCL-86	chr11	88653263	88653263	C	T	exonic	GRM5	27	12	0.3077	Missense_Mutation	p.R351Q	Non-Targeted Panel Gene	Adult HGBCL, NOS
BCL-86	chr19	49497281	49497281	C	T	exonic	RPS11	42	19	0.3115	Missense_Mutation	p.R35W	Non-Targeted Panel Gene	Adult HGBCL, NOS
BCL-86	chr16	89749877	89749877	T	G	exonic	FANCA	11	5	0.3125	Missense_Mutation	p.Q1031P	Non-Targeted Panel Gene	Adult HGBCL, NOS
BCL-86	chr17	17175333	17175333	G	A	exonic	MPRIP	22	10	0.3125	Missense_Mutation	p.R877Q	Non-Targeted Panel Gene	Adult HGBCL, NOS
BCL-86	chr4	186076927	186076927	T	G	exonic	TLR3	35	16	0.3137	Missense_Mutation	p.V103G	Non-Targeted Panel Gene	Adult HGBCL, NOS
BCL-86	chr6	43132642	43132642	A	C	exonic	PTK7	42	20	0.3226	Missense_Mutation	p.N395H	Non-Targeted Panel Gene	Adult HGBCL, NOS
BCL-86	chr18	47033263	47033263	C	T	exonic	ELOA2	49	24	0.3288	Missense_Mutation	p.E668K	Non-Targeted Panel Gene	Adult HGBCL, NOS
BCL-86	chr1	81943304	81943304	A	G	exonic	ADGRL2	31	16	0.3404	Missense_Mutation	p.I249V	Non-Targeted Panel Gene	Adult HGBCL, NOS
BCL-86	chr7	137572835	137572835	C	T	exonic	DGKI	25	13	0.3421	Missense_Mutation	p.D289N	Non-Targeted Panel Gene	Adult HGBCL, NOS
BCL-86	chr3	171139474	171139474	A	G	exonic	TNIK	15	8	0.3478	Missense_Mutation	p.L472P	Non-Targeted Panel Gene	Adult HGBCL, NOS
BCL-86	chr19	47094417	47094417	G	A	exonic	ZC3H4	39	21	0.35	Missense_Mutation	p.S118L	Non-Targeted Panel Gene	Adult HGBCL, NOS
BCL-86	chr6	125881183	125881183	G	T	exonic	NCOA7	14	8	0.3636	Missense_Mutation	p.A185S	Non-Targeted Panel Gene	Adult HGBCL, NOS
BCL-86	chr2	120990613	120990613	G	A	exonic	GLI2	60	35	0.3684	Missense_Mutation	p.D1567N	Non-Targeted Panel Gene	Adult HGBCL, NOS
BCL-86	chr17	16299976	16299976	C	A	exonic	PIGL	20	12	0.375	Missense_Mutation	p.L142M	Non-Targeted Panel Gene	Adult HGBCL, NOS
BCL-86	chr5	31267579	31267579	A	G	exonic	CDH6	33	20	0.3774	Missense_Mutation	p.R36G	Non-Targeted Panel Gene	Adult HGBCL, NOS
BCL-86	chr12	49648213	49648213	G	A	exonic	FMNL3	28	17	0.3778	Missense_Mutation	p.R886W	Non-Targeted Panel Gene	Adult HGBCL, NOS
BCL-86	chr2	5693733	5693733	T	C	exonic	SOX11	591	379	0.3907	Missense_Mutation	p.S338P	Non-Targeted Panel Gene	Adult HGBCL, NOS
BCL-86	chr14	31326337	31326337	T	C	exonic	HEATR5A	21	15	0.4167	Missense_Mutation	p.N1125D	Non-Targeted Panel Gene	Adult HGBCL, NOS
BCL-86	chr13	105472655	105472655	G	T	exonic	DAOA	19	14	0.4242	Missense_Mutation	p.W13L	Non-Targeted Panel Gene	Adult HGBCL, NOS
BCL-86	chr2	178546480	178546480	A	T	exonic	TTN	28	21	0.4286	Missense_Mutation	p.D31617E	Non-Targeted Panel Gene	Adult HGBCL, NOS
BCL-86	chr8	31080997	31080997	A	G	exonic	WRN	41	31	0.4306	Missense_Mutation	p.T324A	Non-Targeted Panel Gene	Adult HGBCL, NOS
BCL-86	chr14	81144162	81144162	C	G	exonic	TSHR	42	32	0.4324	Missense_Mutation	p.Q702E	Non-Targeted Panel Gene	Adult HGBCL, NOS
BCL-86	chr1	248811934	248811934	C	T	exonic	SH3BP5L	166	137	0.4507	Missense_Mutation	p.R264H	Non-Targeted Panel Gene	Adult HGBCL, NOS
BCL-86	chr2	48479993	48479993	A	G	exonic	PPP1R21	20	17	0.4595	Missense_Mutation	p.H432R	Non-Targeted Panel Gene	Adult HGBCL, NOS
BCL-86	chr19	57291722	57291722	G	C	exonic	ZNF460	15	13	0.4643	Missense_Mutation	p.C394S	Non-Targeted Panel Gene	Adult HGBCL, NOS
BCL-86	chr6	38842441	38842441	G	C	exonic	DNAH8	16	14	0.4667	Missense_Mutation	p.E1514Q	Non-Targeted Panel Gene	Adult HGBCL, NOS
BCL-86	chr5	141639595	141639595	A	T	exonic	RELL2	18	16	0.4706	Missense_Mutation	p.K150M	Non-Targeted Panel Gene	Adult HGBCL, NOS
BCL-86	chr4	87662640	87662640	A	G	exonic	DMP1	12	11	0.4783	Missense_Mutation	p.M288V	Non-Targeted Panel Gene	Adult HGBCL, NOS
BCL-86	chr16	68684685	68684685	G	A	exonic	CDH3	27	25	0.4808	Missense_Mutation	p.V429I	Non-Targeted Panel Gene	Adult HGBCL, NOS
BCL-86	chr3	49662367	49662367	G	C	exonic	BSN	32	31	0.4844	Missense_Mutation	p.V3508L	Non-Targeted Panel Gene	Adult HGBCL, NOS
BCL-86	chr13	109785353	109785353	T	C	exonic	IRS2	229	219	0.4888	Missense_Mutation	p.E234G	Non-Targeted Panel Gene	Adult HGBCL, NOS
BCL-86	chr14	94619305	94619305	C	G	exonic	SERPINA3	26	25	0.4902	Missense_Mutation	p.P252A	Non-Targeted Panel Gene	Adult HGBCL, NOS
BCL-86	chr16	2528583	2528583	C	T	exonic	AMDHD2	98	98	0.5	Missense_Mutation	p.P332S	Non-Targeted Panel Gene	Adult HGBCL, NOS
BCL-86	chr12	121849726	121849726	T	C	exonic	HPD	14	14	0.5	Missense_Mutation	p.Y121C	Non-Targeted Panel Gene	Adult HGBCL, NOS
BCL-86	chr1	205931957	205931957	T	C	exonic	SLC26A9	26	26	0.5	Missense_Mutation	p.N152S	Non-Targeted Panel Gene	Adult HGBCL, NOS
BCL-86	chrX	1466522	1466522	T	C	exonic	P2RY8	50	51	0.505	Missense_Mutation	p.T13A	Non-Targeted Panel Gene	Adult HGBCL, NOS
BCL-86	chr5	67166458	67166458	C	T	exonic	MAST4	26	27	0.5094	Missense_Mutation	p.P2427S	Non-Targeted Panel Gene	Adult HGBCL, NOS
BCL-86	chr2	196726048	196726048	T	G	exonic	CCDC150	15	16	0.5161	Missense_Mutation	p.F835L	Non-Targeted Panel Gene	Adult HGBCL, NOS
BCL-86	chr13	113184379	113184379	G	A	exonic	PCID2	23	25	0.5208	Missense_Mutation	p.R218C	Non-Targeted Panel Gene	Adult HGBCL, NOS
BCL-86	chr15	24676826	24676826	C	G	exonic	NPAP1	33	36	0.5217	Missense_Mutation	p.A320G	Non-Targeted Panel Gene	Adult HGBCL, NOS
BCL-86	chr7	11636650	11636650	G	A	exonic	THSD7A	21	23	0.5227	Missense_Mutation	p.P168S	Non-Targeted Panel Gene	Adult HGBCL, NOS
BCL-86	chr21	46432135	46432135	G	A	exonic	PCNT	8	9	0.5294	Missense_Mutation	p.A2891T	Non-Targeted Panel Gene	Adult HGBCL, NOS
BCL-86	chr3	27304769	27304769	G	C	exonic	NEK10	15	17	0.5312	Missense_Mutation	p.Q336E	Non-Targeted Panel Gene	Adult HGBCL, NOS
BCL-86	chr12	56142876	56142876	T	C	exonic	ESYT1	36	41	0.5325	Missense_Mutation	p.L977P	Non-Targeted Panel Gene	Adult HGBCL, NOS
BCL-86	chr1	32893832	32893832	G	T	exonic	HPCA	60	80	0.5714	Missense_Mutation	p.Q184H	Non-Targeted Panel Gene	Adult HGBCL, NOS
BCL-86	chr11	119092975	119092975	A	C	exonic	HMBS	24	37	0.6066	Missense_Mutation	p.D272A	Non-Targeted Panel Gene	Adult HGBCL, NOS
BCL-86	chr5	74730268	74730268	C	T	exonic	GFM2	12	24	0.6667	Missense_Mutation	p.R605H	Non-Targeted Panel Gene	Adult HGBCL, NOS

BCL-86	chrX	2490455	2490455	C	A	exonic	ZBED1	53	186	0.7782	Missense_Mutation	p.A89S	Non-Targeted Panel Gene	Adult HGBCL, NOS
BCL-86	chr6	75265260	75265260	G	A	exonic	TMEM30A	13	10	0.4348	Nonsense_Mutation	p.Q142X	Targerted Panel Gene	Adult HGBCL, NOS
BCL-86	chr10	30029353	30029353	G	T	exonic	JCAD	47	2	0.04	Nonsense_Mutation	p.C265X	Non-Targeted Panel Gene	Adult HGBCL, NOS
BCL-86	chr14	30919037	30919037	G	-	exonic	STRN3	31	2	0.0606	Nonsense_Mutation	p.S390X	Non-Targeted Panel Gene	Adult HGBCL, NOS
BCL-86	chr12	103740737	103740737	C	A	exonic	STAB2	46	3	0.0612	Nonsense_Mutation	p.Y1954X	Non-Targeted Panel Gene	Adult HGBCL, NOS
BCL-86	chr14	58260956	58260956	T	A	exonic	PSMA3	43	3	0.0652	Nonsense_Mutation	p.L138X	Non-Targeted Panel Gene	Adult HGBCL, NOS
BCL-86	chr12	49642960	49642960	C	T	exonic	PRPF40B	28	2	0.0667	Nonsense_Mutation	p.R717X	Non-Targeted Panel Gene	Adult HGBCL, NOS
BCL-86	chr16	80997323	80997323	A	T	exonic	CMC2	27	2	0.069	Nonsense_Mutation	p.C24X	Non-Targeted Panel Gene	Adult HGBCL, NOS
BCL-86	chr6	48009075	48009075	C	-	exonic	PTCHD4	46	4	0.08	Nonsense_Mutation	p.V156X	Non-Targeted Panel Gene	Adult HGBCL, NOS
BCL-86	chr1	70355183	70355183	A	T	exonic	HHLA3	33	3	0.0833	Nonsense_Mutation	p.K78X	Non-Targeted Panel Gene	Adult HGBCL, NOS
BCL-86	chr11	65130804	65130804	G	A	exonic	SYVN1	127	33	0.2062	Nonsense_Mutation	p.Q321X	Non-Targeted Panel Gene	Adult HGBCL, NOS
BCL-86	chrX	68511879	68511879	C	T	exonic	YIPF6	38	18	0.3214	Nonsense_Mutation	p.Q30X	Non-Targeted Panel Gene	Adult HGBCL, NOS
BCL-86	chr11	129876877	129876877	G	A	exonic	NFRKB	8	16	0.6667	Nonsense_Mutation	p.Q531X	Non-Targeted Panel Gene	Adult HGBCL, NOS
BCL-87	chr6	134174509	134174509	A	C	splicing	SGK1	71	13	0.1548	Splice_Site	.	Targerted Panel Gene	Adult HGBCL, NOS
BCL-87	chr9	78304941	78304941	G	A	splicing	PSAT1	59	10	0.1449	Splice_Site	.	Non-Targeted Panel Gene	Adult HGBCL, NOS
BCL-87	chr3	183945849	183945849	C	T	splicing	ABCC5	33	8	0.1951	Splice_Site	.	Non-Targeted Panel Gene	Adult HGBCL, NOS
BCL-87	chr1	44758142	44758142	T	C	splicing	KIF2C	55	14	0.2029	Splice_Site	.	Non-Targeted Panel Gene	Adult HGBCL, NOS
BCL-87	chr19	808763	808763	G	A	splicing	PTBP1	29	11	0.275	Splice_Site	.	Non-Targeted Panel Gene	Adult HGBCL, NOS
BCL-87	chr13	71865921	71865921	C	G	splicing	DACH1	45	23	0.3382	Splice_Site	.	Non-Targeted Panel Gene	Adult HGBCL, NOS
BCL-87	chr9	133636712	133636712	T	C	splicing	DBH	29	30	0.5085	Splice_Site	.	Non-Targeted Panel Gene	Adult HGBCL, NOS
BCL-87	chrX	12976245	12976245	G	C	splicing	TMSB4X	12	15	0.5556	Splice_Site	.	Non-Targeted Panel Gene	Adult HGBCL, NOS
BCL-87	chr9	19082871	19082871	A	G	splicing	HAUS6	13	19	0.5938	Splice_Site	.	Non-Targeted Panel Gene	Adult HGBCL, NOS
BCL-87	chr3	49360230	49360230	T	-	exonic	RHOA	86	22	0.2037	Frame_Shift_Del	p.K187fs	Targerted Panel Gene	Adult HGBCL, NOS
BCL-87	chr6	41935984	41935991	TGGGCCCT	-	exonic	CCND3	28	9	0.2432	Frame_Shift_Del	p.Q276fs	Targerted Panel Gene	Adult HGBCL, NOS
BCL-87	chr8	60856660	60856663	TCTT	-	exonic	CHD7	30	10	0.25	Frame_Shift_Del	p.S2460fs	Targerted Panel Gene	Adult HGBCL, NOS
BCL-87	chr4	121036315	121036315	C	-	exonic	NDNF	81	9	0.1	Frame_Shift_Del	p.G552fs	Non-Targeted Panel Gene	Adult HGBCL, NOS
BCL-87	chr1	154154995	154154996	AA	-	exonic	NUP210L	50	9	0.1525	Frame_Shift_Del	p.F17fs	Non-Targeted Panel Gene	Adult HGBCL, NOS
BCL-87	chr1	247443989	247443989	T	-	exonic	NLRP3	35	14	0.2857	Frame_Shift_Del	p.L896fs	Non-Targeted Panel Gene	Adult HGBCL, NOS
BCL-87	chr12	26122479	26122494	GGGCCGCGGCGGC	-	exonic	BHLHE41	14	6	0.3	Frame_Shift_Del	p.A341fs	Non-Targeted Panel Gene	Adult HGBCL, NOS
BCL-87	chr9	37020771	37020771	-	CC	exonic	PAX5	40	11	0.2157	Frame_Shift_Ins	p.V26fs	Targerted Panel Gene	Adult HGBCL, NOS
BCL-87	chr6	75284457	75284457	-	AG	exonic	TMEM30A	53	24	0.3117	Frame_Shift_Ins	p.F61fs	Targerted Panel Gene	Adult HGBCL, NOS
BCL-87	chr11	128521923	128521923	-	AA	exonic	ETS1	34	17	0.3269	Frame_Shift_Ins	p.F24fs	Targerted Panel Gene	Adult HGBCL, NOS
BCL-87	chr5	35670246	35670246	-	A	exonic	SPEF2	46	18	0.2812	Frame_Shift_Ins	p.X515delinsX	Non-Targeted Panel Gene	Adult HGBCL, NOS
BCL-87	chr6	33201893	33201893	-	C	exonic	SLC39A7	72	30	0.2941	Frame_Shift_Ins	p.H62fs	Non-Targeted Panel Gene	Adult HGBCL, NOS
BCL-87	chr2	38017215	38017217	AAT	-	exonic	RMDN2	44	13	0.2281	In_Frame_Del	p.403_404del	Non-Targeted Panel Gene	Adult HGBCL, NOS
BCL-87	chr1	108849542	108849544	AGA	-	exonic	AKNAD1	30	14	0.3182	In_Frame_Del	p.342_343del	Non-Targeted Panel Gene	Adult HGBCL, NOS
BCL-87	chr6	134174516	134174516	G	C	exonic	SGK1	72	13	0.1529	Missense_Mutation	p.C144W	Targerted Panel Gene	Adult HGBCL, NOS
BCL-87	chr4	154236985	154236985	A	C	exonic	DCHS2	59	12	0.169	Missense_Mutation	p.L2101R	Targerted Panel Gene	Adult HGBCL, NOS
BCL-87	chr2	96144861	96144861	C	A	exonic	DUSP2	43	10	0.1887	Missense_Mutation	p.G137V	Targerted Panel Gene	Adult HGBCL, NOS
BCL-87	chr7	82952903	82952903	T	G	exonic	PCLO	64	15	0.1899	Missense_Mutation	p.S2684R	Targerted Panel Gene	Adult HGBCL, NOS
BCL-87	chr4	84829016	84829016	T	G	exonic	WDFY3	25	6	0.1935	Missense_Mutation	p.D315A	Targerted Panel Gene	Adult HGBCL, NOS
BCL-87	chr22	22888211	22888211	G	A	exonic	IGLL5	15	4	0.2105	Missense_Mutation	p.E18K	Targerted Panel Gene	Adult HGBCL, NOS
BCL-87	chr4	84829007	84829007	A	C	exonic	WDFY3	22	6	0.2143	Missense_Mutation	p.L318R	Targerted Panel Gene	Adult HGBCL, NOS
BCL-87	chr8	112281311	112281311	T	A	exonic	CSMD3	43	13	0.2321	Missense_Mutation	p.K3084I	Targerted Panel Gene	Adult HGBCL, NOS
BCL-87	chr9	90888596	90888596	T	G	exonic	SYK	29	9	0.2368	Missense_Mutation	p.Y602D	Targerted Panel Gene	Adult HGBCL, NOS
BCL-87	chr1	103074690	103074690	A	T	exonic	COL11A1	28	9	0.2432	Missense_Mutation	p.S193R	Targerted Panel Gene	Adult HGBCL, NOS
BCL-87	chr6	27132534	27132534	G	T	exonic	HIST1H2BJ	119	39	0.2468	Missense_Mutation	p.R73S	Targerted Panel Gene	Adult HGBCL, NOS
BCL-87	chr6	26056260	26056260	C	G	exonic	HIST1H1C	63	22	0.2588	Missense_Mutation	p.V57L	Targerted Panel Gene	Adult HGBCL, NOS
BCL-87	chr3	79018440	79018440	A	G	exonic	ROBO1	17	7	0.2917	Missense_Mutation	p.F8L	Targerted Panel Gene	Adult HGBCL, NOS
BCL-87	chr6	134174568	134174568	C	G	exonic	SGK1	62	26	0.2921	Missense_Mutation	p.R127T	Targerted Panel Gene	Adult HGBCL, NOS
BCL-87	chr22	22888237	22888237	A	C	exonic	IGLL5	12	5	0.2941	Missense_Mutation	p.S62R	Targerted Panel Gene	Adult HGBCL, NOS
BCL-87	chr19	11252855	11252855	G	T	exonic	DOCK6	30	13	0.3023	Missense_Mutation	p.P79Q	Targerted Panel Gene	Adult HGBCL, NOS
BCL-87	chr7	111728687	111728687	C	T	exonic	DOCK4	38	17	0.3091	Missense_Mutation	p.E1830K	Targerted Panel Gene	Adult HGBCL, NOS
BCL-87	chr22	22888138	22888138	C	G	exonic	IGLL5	15	9	0.375	Missense_Mutation	p.L29V	Targerted Panel Gene	Adult HGBCL, NOS
BCL-87	chr12	25245321	25245321	G	T	exonic	KRAS	41	27	0.3971	Missense_Mutation	p.Q22K	Targerted Panel Gene	Adult HGBCL, NOS
BCL-87	chr19	38525492	38525492	G	A	exonic	RYR1	18	12	0.4	Missense_Mutation	p.R3539H	Targerted Panel Gene	Adult HGBCL, NOS
BCL-87	chr1	201207483	201207483	G	A	exonic	IGFN1	16	13	0.4483	Missense_Mutation	p.G864R	Targerted Panel Gene	Adult HGBCL, NOS
BCL-87	chr6	134174586	134174586	G	A	exonic	SGK1	45	44	0.4944	Missense_Mutation	p.A121V	Targerted Panel Gene	Adult HGBCL, NOS
BCL-87	chr10	71777885	71777885	G	A	exonic	CDH23	11	18	0.6207	Missense_Mutation	p.R1684H	Targerted Panel Gene	Adult HGBCL, NOS
BCL-87	chr2	241065496	241065496	G	C	exonic	SNED1	48	2	0.04	Missense_Mutation	p.A971P	Non-Targeted Panel Gene	Adult HGBCL, NOS
BCL-87	chr1	58534166	58534166	C	T	exonic	OMA1	78	4	0.0488	Missense_Mutation	p.V299M	Non-Targeted Panel Gene	Adult HGBCL, NOS
BCL-87	chr7	100430832	100430832	C	A	exonic	MEPCE	96	5	0.0495	Missense_Mutation	p.Q272K	Non-Targeted Panel Gene	Adult HGBCL, NOS
BCL-87	chr9	112659326	112659326	G	A	exonic	KIAA1958	62	4	0.0606	Missense_Mutation	p.G498S	Non-Targeted Panel Gene	Adult HGBCL, NOS
BCL-87	chr1	44777715	44777715	G	A	exonic	RPS8	39	3	0.0714	Missense_Mutation	p.D105N	Non-Targeted Panel Gene	Adult HGBCL, NOS
BCL-87	chr16	67648441	67648441	C	T	exonic	CARMIL2	34	3	0.0811	Missense_Mutation	p.R424C	Non-Targeted Panel Gene	Adult HGBCL, NOS
BCL-87	chr14	55007336	55007336	C	T	exonic	WDHD1	56	5	0.082	Missense_Mutation	p.D182N	Non-Targeted Panel Gene	Adult HGBCL, NOS
BCL-87	chr18	50274296	50274296	G	A	exonic	MBD1	30	3	0.0909	Missense_Mutation	p.R202W	Non-Targeted Panel Gene	Adult HGBCL, NOS
BCL-87	chr1	153150085	153150085	T	G	exonic	SPRR2G	26	3	0.1034	Missense_Mutation	p.K9T	Non-Targeted Panel Gene	Adult HGBCL, NOS
BCL-87	chr6	138424407	138424407	G	A	exonic	NHSL1	53	7	0.1167	Missense_Mutation	p.R1499C	Non-Targeted Panel Gene	Adult HGBCL, NOS
BCL-87	chr11	65387121	65387121	G	C	exonic	FRMD8	46	7	0.1321	Missense_Mutation	p.A29P	Non-Targeted Panel Gene	Adult HGBCL, NOS
BCL-87	chr10	116089850	116089850	G	T	exonic	GFRA1	26	4	0.1333	Missense_Mutation	p.T363N	Non-Targeted Panel Gene	Adult HGBCL, NOS
BCL-87	chr3	138698942	138698942	A	C	exonic	PIK3CB	45	7	0.1346	Missense_Mutation	p.S579A	Non-Targeted Panel Gene	Adult HGBCL, NOS
BCL-87	chr14	75959240	75959240	G	C	exonic	TGFB3	47	8	0.1455	Missense_Mutation	p.P396A	Non-Targeted Panel Gene	Adult HGBCL, NOS
BCL-87	chr9	97317209	97317209	G	A	exonic	CCDC180	28	5	0.1515	Missense_Mutation	p.A355T	Non-Targeted Panel Gene	Adult HGBCL, NOS
BCL-87	chr19	10252303	10252303	G	C	exonic	MRPL4	33	6	0.1538	Missense_Mutation	p.G17A	Non-Targeted Panel Gene	Adult HGBCL, NOS
BCL-87	chr1	244863950	244863950	G	A	exonic	HNRNPU	72	14	0.1628	Missense_Mutation	p.P120S	Non-Targeted Panel Gene	Adult HGBCL, NOS
BCL-87	chr9	111929664	111929664	A	C	exonic	UGCG	54	11	0.1667	Missense_Mutation	p.K241N	Non-Targeted Panel Gene	Adult HGBCL, NOS
BCL-87	chr5	80080043	80080043	T	G	exonic	THBS4	38	8	0.1739	Missense_Mutation	p.F793V	Non-Targeted Panel Gene	Adult HGBCL, NOS
BCL-87	chr17	82083287	82083287	T	C	exonic	FASN	66	14	0.175	Missense_Mutation	p.K1827R	Non-Targeted Panel Gene	Adult HGBCL, NOS
BCL-87	chr8	32764010	32764010	A	C	exonic	NRG1	84	18	0.1765	Missense_Mutation	p.S516R	Non-Targeted Panel Gene	Adult HGBCL, NOS
BCL-87	chr9	99828068	99828068	G	A	exonic	NR4A3	72	16	0.1818	Missense_Mutation	p.S20N	Non-Targeted Panel Gene	Adult HGBCL, NOS
BCL-87	chr16	66550059	66550059	C	T	exonic	TK2	27	6	0.1818	Missense_Mutation	p.M1I	Non-Targeted Panel Gene	Adult HGBCL, NOS

BCL-87	chr12	89350959	89350959	C	T	exonic	DUSP6	67	15	0.1829	Missense_Mutation	p.S156N	Non-Targeted Panel Gene	Adult HGBCL, NOS
BCL-87	chr17	3397885	3397885	G	A	exonic	OR1E1	57	13	0.1857	Missense_Mutation	p.H176Y	Non-Targeted Panel Gene	Adult HGBCL, NOS
BCL-87	chr13	51774021	51774021	G	A	exonic	DHRS12	47	11	0.1897	Missense_Mutation	p.S175L	Non-Targeted Panel Gene	Adult HGBCL, NOS
BCL-87	chr6	26031717	26031717	G	C	exonic	HIST1H3B	47	11	0.1897	Missense_Mutation	p.A115G	Non-Targeted Panel Gene	Adult HGBCL, NOS
BCL-87	chr4	154586270	154586270	G	T	exonic	FGA	102	24	0.1905	Missense_Mutation	p.H387N	Non-Targeted Panel Gene	Adult HGBCL, NOS
BCL-87	chr8	63064239	63064239	G	C	exonic	TTPA	51	12	0.1905	Missense_Mutation	p.I210M	Non-Targeted Panel Gene	Adult HGBCL, NOS
BCL-87	chr1	153991571	153991571	T	A	exonic	RPS27	106	26	0.197	Missense_Mutation	p.Y9N	Non-Targeted Panel Gene	Adult HGBCL, NOS
BCL-87	chr12	47791984	47791984	T	C	exonic	HDAC7	24	6	0.2	Missense_Mutation	p.M567V	Non-Targeted Panel Gene	Adult HGBCL, NOS
BCL-87	chr17	74952342	74952342	T	C	exonic	HID1	28	7	0.2	Missense_Mutation	p.K691E	Non-Targeted Panel Gene	Adult HGBCL, NOS
BCL-87	chr17	38327442	38327442	C	T	exonic	GPR179	90	23	0.2035	Missense_Mutation	p.E2043K	Non-Targeted Panel Gene	Adult HGBCL, NOS
BCL-87	chr1	32100495	32100495	A	G	exonic	TMEM39B	39	10	0.2041	Missense_Mutation	p.N390S	Non-Targeted Panel Gene	Adult HGBCL, NOS
BCL-87	chr9	69516822	69516822	G	A	exonic	APBA1	50	13	0.2063	Missense_Mutation	p.T130M	Non-Targeted Panel Gene	Adult HGBCL, NOS
BCL-87	chr7	41689847	41689847	A	G	exonic	INHBA	61	16	0.2078	Missense_Mutation	p.S362P	Non-Targeted Panel Gene	Adult HGBCL, NOS
BCL-87	chr1	236686710	236686710	G	A	exonic	ACTN2	45	12	0.2105	Missense_Mutation	p.V13M	Non-Targeted Panel Gene	Adult HGBCL, NOS
BCL-87	chr3	15720953	15720953	C	T	exonic	ANKRD28	45	12	0.2105	Missense_Mutation	p.E287K	Non-Targeted Panel Gene	Adult HGBCL, NOS
BCL-87	chr2	191064871	191064871	G	A	exonic	STAT4	58	16	0.2162	Missense_Mutation	p.R240W	Non-Targeted Panel Gene	Adult HGBCL, NOS
BCL-87	chr7	13977444	13977444	T	C	exonic	ETV1	36	10	0.2174	Missense_Mutation	p.D73G	Non-Targeted Panel Gene	Adult HGBCL, NOS
BCL-87	chr19	6744908	6744908	C	G	exonic	TRIP10	45	13	0.2241	Missense_Mutation	p.L300V	Non-Targeted Panel Gene	Adult HGBCL, NOS
BCL-87	chr6	33711655	33711655	T	C	exonic	UQCC2	45	13	0.2241	Missense_Mutation	p.K11R	Non-Targeted Panel Gene	Adult HGBCL, NOS
BCL-87	chr12	57463753	57463753	C	A	exonic	GLI1	48	14	0.2258	Missense_Mutation	p.P21H	Non-Targeted Panel Gene	Adult HGBCL, NOS
BCL-87	chr2	119441569	119441569	G	A	exonic	SCTR	24	7	0.2258	Missense_Mutation	p.L391F	Non-Targeted Panel Gene	Adult HGBCL, NOS
BCL-87	chr2	169749546	169749546	G	C	exonic	KLHL23;PHOSPHO2	41	12	0.2264	Missense_Mutation	p.R497S	Non-Targeted Panel Gene	Adult HGBCL, NOS
BCL-87	chr5	52910316	52910316	G	T	exonic	ITGA1	17	5	0.2273	Missense_Mutation	p.G585V	Non-Targeted Panel Gene	Adult HGBCL, NOS
BCL-87	chr2	181919307	181919307	A	G	exonic	SSFA2	61	18	0.2278	Missense_Mutation	p.D1002G	Non-Targeted Panel Gene	Adult HGBCL, NOS
BCL-87	chr2	8682874	8682874	A	G	exonic	ID2	67	20	0.2299	Missense_Mutation	p.N127S	Non-Targeted Panel Gene	Adult HGBCL, NOS
BCL-87	chr7	122113686	122113686	C	T	exonic	AASS	80	24	0.2308	Missense_Mutation	p.G360R	Non-Targeted Panel Gene	Adult HGBCL, NOS
BCL-87	chr1	145871998	145871998	C	G	exonic	ANKRD35	20	6	0.2308	Missense_Mutation	p.R924P	Non-Targeted Panel Gene	Adult HGBCL, NOS
BCL-87	chr2	166432325	166432325	C	T	exonic	SCN7A	40	12	0.2308	Missense_Mutation	p.S862N	Non-Targeted Panel Gene	Adult HGBCL, NOS
BCL-87	chr18	580529	580529	G	A	exonic	CETN1	59	18	0.2338	Missense_Mutation	p.D41N	Non-Targeted Panel Gene	Adult HGBCL, NOS
BCL-87	chr3	52693214	52693214	T	G	exonic	GNL3	57	18	0.24	Missense_Mutation	p.Y346D	Non-Targeted Panel Gene	Adult HGBCL, NOS
BCL-87	chr5	1076795	1076795	C	G	exonic	SLC12A7	19	6	0.24	Missense_Mutation	p.K549N	Non-Targeted Panel Gene	Adult HGBCL, NOS
BCL-87	chr2	189706939	189706939	A	C	exonic	ANKAR	44	14	0.2414	Missense_Mutation	p.N638H	Non-Targeted Panel Gene	Adult HGBCL, NOS
BCL-87	chr19	52116940	52116940	T	C	exonic	ZNF616	103	33	0.2426	Missense_Mutation	p.E75G	Non-Targeted Panel Gene	Adult HGBCL, NOS
BCL-87	chr3	167449392	167449392	T	G	exonic	SERPINI2	31	10	0.2439	Missense_Mutation	p.E335D	Non-Targeted Panel Gene	Adult HGBCL, NOS
BCL-87	chr13	96986774	96986774	T	C	exonic	OXGR1	55	18	0.2466	Missense_Mutation	p.K329R	Non-Targeted Panel Gene	Adult HGBCL, NOS
BCL-87	chr11	101128179	101128179	T	A	exonic	PGR	51	17	0.25	Missense_Mutation	p.T134S	Non-Targeted Panel Gene	Adult HGBCL, NOS
BCL-87	chr10	113567513	113567513	A	C	exonic	HABP2	53	18	0.2535	Missense_Mutation	p.S32R	Non-Targeted Panel Gene	Adult HGBCL, NOS
BCL-87	chr22	37244118	37244118	C	T	exonic	RAC2	53	18	0.2535	Missense_Mutation	p.D11N	Non-Targeted Panel Gene	Adult HGBCL, NOS
BCL-87	chr19	36877025	36877025	G	C	exonic	ZNF345	79	27	0.2547	Missense_Mutation	p.K65N	Non-Targeted Panel Gene	Adult HGBCL, NOS
BCL-87	chr17	49222224	49222224	A	C	exonic	ABI3	26	9	0.2571	Missense_Mutation	p.K312N	Non-Targeted Panel Gene	Adult HGBCL, NOS
BCL-87	chr5	13714538	13714538	T	G	exonic	DNAH5	23	8	0.2581	Missense_Mutation	p.D4331A	Non-Targeted Panel Gene	Adult HGBCL, NOS
BCL-87	chr17	50353729	50353729	G	A	exonic	XYLT2	20	7	0.2593	Missense_Mutation	p.G79R	Non-Targeted Panel Gene	Adult HGBCL, NOS
BCL-87	chr19	19642014	19642014	C	G	exonic	GMIP	47	17	0.2656	Missense_Mutation	p.E48Q	Non-Targeted Panel Gene	Adult HGBCL, NOS
BCL-87	chr4	124710280	124710280	C	T	exonic	ANKRD50	44	16	0.2667	Missense_Mutation	p.G78R	Non-Targeted Panel Gene	Adult HGBCL, NOS
BCL-87	chr3	149751404	149751404	A	G	exonic	COMMD2	44	16	0.2667	Missense_Mutation	p.M76T	Non-Targeted Panel Gene	Adult HGBCL, NOS
BCL-87	chr16	50094747	50094747	C	A	exonic	HEATR3	44	16	0.2667	Missense_Mutation	p.A401D	Non-Targeted Panel Gene	Adult HGBCL, NOS
BCL-87	chr6	27453074	27453074	T	G	exonic	ZNF184	74	27	0.2673	Missense_Mutation	p.K162T	Non-Targeted Panel Gene	Adult HGBCL, NOS
BCL-87	chr1	38857060	38857060	G	C	exonic	RRAGC	30	11	0.2683	Missense_Mutation	p.P87R	Non-Targeted Panel Gene	Adult HGBCL, NOS
BCL-87	chr11	30012251	30012251	C	T	exonic	KCNA4	19	7	0.2692	Missense_Mutation	p.S143N	Non-Targeted Panel Gene	Adult HGBCL, NOS
BCL-87	chr5	102459699	102459699	C	A	exonic	SLCO6A1	38	14	0.2692	Missense_Mutation	p.W326C	Non-Targeted Panel Gene	Adult HGBCL, NOS
BCL-87	chr15	74134065	74134065	G	C	exonic	ISLR2	84	31	0.2696	Missense_Mutation	p.E437D	Non-Targeted Panel Gene	Adult HGBCL, NOS
BCL-87	chr14	50591635	50591635	T	C	exonic	ATL1	121	45	0.2711	Missense_Mutation	p.I173T	Non-Targeted Panel Gene	Adult HGBCL, NOS
BCL-87	chr11	64768276	64768276	G	A	exonic	SF1	51	19	0.2714	Missense_Mutation	p.P300S	Non-Targeted Panel Gene	Adult HGBCL, NOS
BCL-87	chr3	58870192	58870192	T	G	exonic	C3orf67	42	16	0.2759	Missense_Mutation	p.N153T	Non-Targeted Panel Gene	Adult HGBCL, NOS
BCL-87	chr13	108286841	108286841	A	C	exonic	TNFSF13B	15	6	0.2857	Missense_Mutation	p.T155P	Non-Targeted Panel Gene	Adult HGBCL, NOS
BCL-87	chr3	107377694	107377694	A	C	exonic	CCDC54	67	27	0.2872	Missense_Mutation	p.K36T	Non-Targeted Panel Gene	Adult HGBCL, NOS
BCL-87	chr12	43425643	43425643	A	C	exonic	ADAMTS20	42	17	0.2881	Missense_Mutation	p.C1385W	Non-Targeted Panel Gene	Adult HGBCL, NOS
BCL-87	chr1	158654646	158654646	C	A	exonic	SPTA1	27	11	0.2895	Missense_Mutation	p.G1001C	Non-Targeted Panel Gene	Adult HGBCL, NOS
BCL-87	chr1	27616962	27616962	C	A	exonic	FGR	44	18	0.2903	Missense_Mutation	p.D193Y	Non-Targeted Panel Gene	Adult HGBCL, NOS
BCL-87	chr6	85450158	85450158	C	T	exonic	NT5E	17	7	0.2917	Missense_Mutation	p.R7W	Non-Targeted Panel Gene	Adult HGBCL, NOS
BCL-87	chr11	6169900	6169900	C	T	exonic	OR52B2	34	14	0.2917	Missense_Mutation	p.V143M	Non-Targeted Panel Gene	Adult HGBCL, NOS
BCL-87	chr2	31202183	31202183	G	A	exonic	CAPN14	21	9	0.3	Missense_Mutation	p.P122L	Non-Targeted Panel Gene	Adult HGBCL, NOS
BCL-87	chr11	30012395	30012395	G	T	exonic	KCNA4	21	9	0.3	Missense_Mutation	p.A95D	Non-Targeted Panel Gene	Adult HGBCL, NOS
BCL-87	chr8	2073479	2073479	A	C	exonic	MYOM2	14	6	0.3	Missense_Mutation	p.S367R	Non-Targeted Panel Gene	Adult HGBCL, NOS
BCL-87	chr11	9003794	9003794	C	T	exonic	NRIP3	7	3	0.3	Missense_Mutation	p.G48S	Non-Targeted Panel Gene	Adult HGBCL, NOS
BCL-87	chr9	89339928	89339928	A	C	exonic	SECISBP2	28	12	0.3	Missense_Mutation	p.K426T	Non-Targeted Panel Gene	Adult HGBCL, NOS
BCL-87	chr17	78698845	78698845	C	G	exonic	CYTH1	23	10	0.303	Missense_Mutation	p.G225A	Non-Targeted Panel Gene	Adult HGBCL, NOS
BCL-87	chr3	13570674	13570674	A	G	exonic	FBLN2	29	13	0.3095	Missense_Mutation	p.I107V	Non-Targeted Panel Gene	Adult HGBCL, NOS
BCL-87	chr8	71299187	71299187	T	A	exonic	EYA1	20	9	0.3103	Missense_Mutation	p.Y107F	Non-Targeted Panel Gene	Adult HGBCL, NOS
BCL-87	chr2	168902456	168902456	G	A	exonic	G6PC2	26	12	0.3158	Missense_Mutation	p.G77D	Non-Targeted Panel Gene	Adult HGBCL, NOS
BCL-87	chr12	41189071	41189071	T	G	exonic	PDZRN4	42	20	0.3175	Missense_Mutation	p.F206V	Non-Targeted Panel Gene	Adult HGBCL, NOS
BCL-87	chr10	89307290	89307290	A	G	exonic	IFIT2	30	14	0.3182	Missense_Mutation	p.K445R	Non-Targeted Panel Gene	Adult HGBCL, NOS
BCL-87	chr10	132194991	132194991	G	A	exonic	DPYSL4	17	8	0.32	Missense_Mutation	p.A154T	Non-Targeted Panel Gene	Adult HGBCL, NOS
BCL-87	chr10	25596662	25596662	A	C	exonic	GPR158	19	9	0.3214	Missense_Mutation	p.N673T	Non-Targeted Panel Gene	Adult HGBCL, NOS
BCL-87	chr12	107688706	107688706	C	T	exonic	PWP1	35	17	0.3269	Missense_Mutation	p.P75S	Non-Targeted Panel Gene	Adult HGBCL, NOS
BCL-87	chr4	30722553	30722553	G	C	exonic	PCDH7	69	34	0.3301	Missense_Mutation	p.E377D	Non-Targeted Panel Gene	Adult HGBCL, NOS
BCL-87	chr7	99030670	99030670	C	T	exonic	SMURF1	36	18	0.3333	Missense_Mutation	p.D730N	Non-Targeted Panel Gene	Adult HGBCL, NOS
BCL-87	chr17	75991510	75991510	C	T	exonic	TEN1	29	15	0.3409	Missense_Mutation	p.A46V	Non-Targeted Panel Gene	Adult HGBCL, NOS
BCL-87	chr6	12163353	12163353	C	A	exonic	HIVEP1	48	25	0.3425	Missense_Mutation	p.S2350Y	Non-Targeted Panel Gene	Adult HGBCL, NOS
BCL-87	chr6	54121559	54121559	T	G	exonic	MLIP	17	9	0.3462	Missense_Mutation	p.I59S	Non-Targeted Panel Gene	Adult HGBCL, NOS
BCL-87	chr17	67056114	67056114	C	A	exonic	CACNG1	30	16	0.3478	Missense_Mutation	p.T171N	Non-Targeted Panel Gene	Adult HGBCL, NOS
BCL-87	chr11	5737243	5737243	G	T	exonic	OR56B1	44	24	0.3529	Missense_Mutation	p.A243S	Non-Targeted Panel Gene	Adult HGBCL, NOS

BCL-87	chr8	119562886	119562886	G	T	exonic	ENPP2	25	14	0.359	Missense_Mutation	p.H850N	Non-Targeted Panel Gene	Adult HGBCL, NOS
BCL-87	chr22	36467843	36467843	C	A	exonic	TXN2	25	14	0.359	Missense_Mutation	p.E154D	Non-Targeted Panel Gene	Adult HGBCL, NOS
BCL-87	chr4	81110458	81110458	G	T	exonic	PRKG2	23	13	0.3611	Missense_Mutation	p.L644I	Non-Targeted Panel Gene	Adult HGBCL, NOS
BCL-87	chr5	134718094	134718094	C	T	exonic	SEC24A	27	16	0.3721	Missense_Mutation	p.T964I	Non-Targeted Panel Gene	Adult HGBCL, NOS
BCL-87	chr5	128349467	128349467	T	A	exonic	FBN2	13	8	0.381	Missense_Mutation	p.N957Y	Non-Targeted Panel Gene	Adult HGBCL, NOS
BCL-87	chr2	28241340	28241340	A	C	exonic	BABAM2	33	21	0.3889	Missense_Mutation	p.Q266H	Non-Targeted Panel Gene	Adult HGBCL, NOS
BCL-87	chr22	46261396	46261396	A	G	exonic	PKDREJ	33	21	0.3889	Missense_Mutation	p.Y643H	Non-Targeted Panel Gene	Adult HGBCL, NOS
BCL-87	chr11	22343313	22343313	A	T	exonic	SLC17A6	41	27	0.3971	Missense_Mutation	p.I136F	Non-Targeted Panel Gene	Adult HGBCL, NOS
BCL-87	chr7	77940241	77940241	G	A	exonic	PHTF2	44	29	0.3973	Missense_Mutation	p.R522H	Non-Targeted Panel Gene	Adult HGBCL, NOS
BCL-87	chr3	52388815	52388815	G	A	exonic	DNAH1	18	12	0.4	Missense_Mutation	p.G3125R	Non-Targeted Panel Gene	Adult HGBCL, NOS
BCL-87	chr14	74239734	74239734	C	T	exonic	VSX2	15	10	0.4	Missense_Mutation	p.P58L	Non-Targeted Panel Gene	Adult HGBCL, NOS
BCL-87	chr4	25390176	25390176	G	C	exonic	ANAPC4	25	17	0.4048	Missense_Mutation	p.E186Q	Non-Targeted Panel Gene	Adult HGBCL, NOS
BCL-87	chr14	64210033	64210033	C	T	exonic	SYNE2	30	21	0.4118	Missense_Mutation	p.T6211M	Non-Targeted Panel Gene	Adult HGBCL, NOS
BCL-87	chr8	94529198	94529198	T	A	exonic	VIRMA	47	33	0.4125	Missense_Mutation	p.D251V	Non-Targeted Panel Gene	Adult HGBCL, NOS
BCL-87	chr12	123334198	123334198	C	T	exonic	SBNO1	17	12	0.4138	Missense_Mutation	p.R254H	Non-Targeted Panel Gene	Adult HGBCL, NOS
BCL-87	chr14	94378529	94378529	G	A	exonic	SERPINA1	35	26	0.4194	Missense_Mutation	p.P393S	Non-Targeted Panel Gene	Adult HGBCL, NOS
BCL-87	chr13	75312867	75312867	T	C	exonic	TBC1D4	22	16	0.4211	Missense_Mutation	p.T752A	Non-Targeted Panel Gene	Adult HGBCL, NOS
BCL-87	chr8	18011819	18011819	G	A	exonic	PCM1	28	21	0.4286	Missense_Mutation	p.D1835N	Non-Targeted Panel Gene	Adult HGBCL, NOS
BCL-87	chr22	24868427	24868427	C	T	exonic	SGSM1	16	12	0.4286	Missense_Mutation	p.P349L	Non-Targeted Panel Gene	Adult HGBCL, NOS
BCL-87	chr8	23437036	23437036	C	G	exonic	ENTPD4	33	25	0.431	Missense_Mutation	p.Q424H	Non-Targeted Panel Gene	Adult HGBCL, NOS
BCL-87	chr17	39723332	39723332	A	G	exonic	ERBB2	17	13	0.4333	Missense_Mutation	p.I639V	Non-Targeted Panel Gene	Adult HGBCL, NOS
BCL-87	chr22	20405610	20405610	G	A	exonic	ZNF74	18	14	0.4375	Missense_Mutation	p.E193K	Non-Targeted Panel Gene	Adult HGBCL, NOS
BCL-87	chrX	55017533	55017533	G	T	exonic	ALAS2	28	22	0.44	Missense_Mutation	p.P306H	Non-Targeted Panel Gene	Adult HGBCL, NOS
BCL-87	chr14	96328703	96328703	A	G	exonic	ATG2B	24	19	0.4419	Missense_Mutation	p.Y649H	Non-Targeted Panel Gene	Adult HGBCL, NOS
BCL-87	chr5	110605225	110605225	T	C	exonic	TMEM232	31	25	0.4464	Missense_Mutation	p.H387R	Non-Targeted Panel Gene	Adult HGBCL, NOS
BCL-87	chr2	152657958	152657958	T	G	exonic	PRPF40A	35	30	0.4615	Missense_Mutation	p.E877D	Non-Targeted Panel Gene	Adult HGBCL, NOS
BCL-87	chr5	129023699	129023699	A	G	exonic	SLC27A6	22	19	0.4634	Missense_Mutation	p.H415R	Non-Targeted Panel Gene	Adult HGBCL, NOS
BCL-87	chr4	36121264	36121264	G	A	exonic	ARAP2	63	55	0.4661	Missense_Mutation	p.S1270F	Non-Targeted Panel Gene	Adult HGBCL, NOS
BCL-87	chr19	35450309	35450309	T	C	exonic	FFAR2	57	51	0.4722	Missense_Mutation	p.Y199H	Non-Targeted Panel Gene	Adult HGBCL, NOS
BCL-87	chr4	13601029	13601029	A	T	exonic	BOD1L1	66	61	0.4803	Missense_Mutation	p.S1957R	Non-Targeted Panel Gene	Adult HGBCL, NOS
BCL-87	chr19	42272993	42272993	C	T	exonic	CIC	33	31	0.4844	Missense_Mutation	p.H404Y	Non-Targeted Panel Gene	Adult HGBCL, NOS
BCL-87	chr15	40335481	40335481	C	T	exonic	C15orf52	15	15	0.5	Missense_Mutation	p.G428S	Non-Targeted Panel Gene	Adult HGBCL, NOS
BCL-87	chr4	25268566	25268566	A	G	exonic	PI4K2B	16	16	0.5	Missense_Mutation	p.E401G	Non-Targeted Panel Gene	Adult HGBCL, NOS
BCL-87	chr4	184762445	184762445	C	A	exonic	ACSL1	16	17	0.5152	Missense_Mutation	p.G363C	Non-Targeted Panel Gene	Adult HGBCL, NOS
BCL-87	chrX	36073146	36073146	A	T	exonic	CFAP47	11	12	0.5217	Missense_Mutation	p.E1491D	Non-Targeted Panel Gene	Adult HGBCL, NOS
BCL-87	chr7	1898388	1898388	G	C	exonic	MAD1L1	25	28	0.5283	Missense_Mutation	p.L604V	Non-Targeted Panel Gene	Adult HGBCL, NOS
BCL-87	chr1	46189973	46189973	C	T	exonic	POMGNT1	7	8	0.5333	Missense_Mutation	p.D556N	Non-Targeted Panel Gene	Adult HGBCL, NOS
BCL-87	chr7	37342632	37342632	T	C	exonic	ELMO1	13	15	0.5357	Missense_Mutation	p.K20R	Non-Targeted Panel Gene	Adult HGBCL, NOS
BCL-87	chr20	59194323	59194323	G	A	exonic	ZNF831	37	44	0.5432	Missense_Mutation	p.E1102K	Non-Targeted Panel Gene	Adult HGBCL, NOS
BCL-87	chr2	55558822	55558822	A	T	exonic	PPP4R3B	27	33	0.55	Missense_Mutation	p.S803T	Non-Targeted Panel Gene	Adult HGBCL, NOS
BCL-87	chr7	86839045	86839045	G	T	exonic	GRM3	28	35	0.5556	Missense_Mutation	p.D511Y	Non-Targeted Panel Gene	Adult HGBCL, NOS
BCL-87	chr17	50189387	50189387	T	A	exonic	COL1A1	40	52	0.5652	Missense_Mutation	p.D940V	Non-Targeted Panel Gene	Adult HGBCL, NOS
BCL-87	chr2	240999009	240999009	G	C	exonic	SNED1	9	12	0.5714	Missense_Mutation	p.V58L	Non-Targeted Panel Gene	Adult HGBCL, NOS
BCL-87	chr1	37568301	37568301	G	A	exonic	GNL2	20	29	0.5918	Missense_Mutation	p.T710I	Non-Targeted Panel Gene	Adult HGBCL, NOS
BCL-87	chr1	197104066	197104066	G	A	exonic	ASPM	37	54	0.5934	Missense_Mutation	p.R1729W	Non-Targeted Panel Gene	Adult HGBCL, NOS
BCL-87	chr5	36049001	36049001	G	A	exonic	UGT3A2	13	20	0.6061	Missense_Mutation	p.A244V	Non-Targeted Panel Gene	Adult HGBCL, NOS
BCL-87	chr19	16127478	16127478	G	C	exonic	RAB8A	25	39	0.6094	Missense_Mutation	p.K122N	Non-Targeted Panel Gene	Adult HGBCL, NOS
BCL-87	chr14	23390247	23390247	T	A	exonic	MYH6	8	13	0.619	Missense_Mutation	p.E1181V	Non-Targeted Panel Gene	Adult HGBCL, NOS
BCL-87	chr6	35243050	35243050	C	T	exonic	SCUBE3	45	2	0.0426	Nonsense_Mutation	p.R575X	Non-Targeted Panel Gene	Adult HGBCL, NOS
BCL-87	chr4	158868182	158868182	C	T	exonic	FNIP2	72	4	0.0526	Nonsense_Mutation	p.R516X	Non-Targeted Panel Gene	Adult HGBCL, NOS
BCL-87	chr2	179950892	179950892	G	A	exonic	CWC22	51	3	0.0556	Nonsense_Mutation	p.R618X	Non-Targeted Panel Gene	Adult HGBCL, NOS
BCL-87	chr16	15720279	15720279	G	A	exonic	MYH11	40	3	0.0698	Nonsense_Mutation	p.R1616X	Non-Targeted Panel Gene	Adult HGBCL, NOS
BCL-87	chr2	241114992	241114992	C	T	exonic	PASK	61	18	0.2278	Nonsense_Mutation	p.W1128X	Non-Targeted Panel Gene	Adult HGBCL, NOS
BCL-87	chr19	45152702	45152702	G	A	exonic	NKPD1	17	17	0.5	Nonsense_Mutation	p.Q579X	Non-Targeted Panel Gene	Adult HGBCL, NOS
BCL-88	chr8	132839386	132839386	G	T	splicing	PHF20L1	11	2	0.1538	Splice_Site	.	Non-Targeted Panel Gene	Adult HGBCL, NOS
BCL-88	chrX	45078506	45078506	G	A	splicing	KDM6A	47	10	0.1754	Splice_Site	.	Non-Targeted Panel Gene	Adult HGBCL, NOS
BCL-88	chr11	77199601	77199604	GGCA	-	exonic	MYO7A	46	2	0.0417	Frame_Shift_Del	p.W1545fs	Non-Targeted Panel Gene	Adult HGBCL, NOS
BCL-88	chr11	9033766	9033766	C	-	exonic	SCUBE2	26	2	0.0714	Frame_Shift_Del	p.G649fs	Non-Targeted Panel Gene	Adult HGBCL, NOS
BCL-88	chr13	46895683	46895683	T	-	exonic	HTR2A	48	5	0.0943	Frame_Shift_Del	p.N75fs	Non-Targeted Panel Gene	Adult HGBCL, NOS
BCL-88	chr3	56613725	56613726	AC	-	exonic	CCDC66	10	2	0.1667	Frame_Shift_Del	p.D514fs	Non-Targeted Panel Gene	Adult HGBCL, NOS
BCL-88	chr17	42115765	42115765	-	AGGAAGT	exonic	KAT2A	35	2	0.0541	Frame_Shift_Ins	p.L611fs	Non-Targeted Panel Gene	Adult HGBCL, NOS
BCL-88	chr19	20545299	20545299	-	A	exonic	ZNF737	42	29	0.3919	Frame_Shift_Ins	p.A302fs	Non-Targeted Panel Gene	Adult HGBCL, NOS
BCL-88	chr9	128947302	128947302	-	T	exonic	DOLK	22	21	0.4884	Frame_Shift_Ins	p.M1fs	Non-Targeted Panel Gene	Adult HGBCL, NOS
BCL-88	chr10	84372276	84372281	TGATTT	-	exonic	CCSER2	51	2	0.0377	In_Frame_Del	p.408_410del	Non-Targeted Panel Gene	Adult HGBCL, NOS
BCL-88	chr9	128999780	128999782	CCC	-	exonic	NUP188	49	2	0.0392	In_Frame_Del	p.1273_1274del	Non-Targeted Panel Gene	Adult HGBCL, NOS
BCL-88	chr12	96708756	96708803	AGACAGTAATTAC	-	exonic	CFAP54	40	2	0.0476	In_Frame_Del	p.2226_2242del	Non-Targeted Panel Gene	Adult HGBCL, NOS
BCL-88	chr17	7885064	7885064	-	CCGCCGCCC	exonic	CHD3	15	7	0.3043	In_Frame_Ins	p.P86delinsPPPP	Non-Targeted Panel Gene	Adult HGBCL, NOS
BCL-88	chr11	70206450	70206450	G	A	exonic	FADD	56	3	0.0508	Missense_Mutation	p.A202T	Targerted Panel Gene	Adult HGBCL, NOS
BCL-88	chr1	228303815	228303815	T	C	exonic	OBSCN	61	4	0.0615	Missense_Mutation	p.Y4627H	Targerted Panel Gene	Adult HGBCL, NOS
BCL-88	chr16	10903758	10903758	C	A	exonic	CIITA	52	18	0.2571	Missense_Mutation	p.P268H	Targerted Panel Gene	Adult HGBCL, NOS
BCL-88	chr2	96144820	96144820	G	C	exonic	DUSP2	37	19	0.3393	Missense_Mutation	p.L151V	Targerted Panel Gene	Adult HGBCL, NOS
BCL-88	chr12	121823039	121823039	C	A	exonic	SETD1B	88	50	0.3623	Missense_Mutation	p.P1444H	Targerted Panel Gene	Adult HGBCL, NOS
BCL-88	chr6	137880103	137880103	A	C	exonic	TNFAIP3	29	25	0.463	Missense_Mutation	p.T647P	Targerted Panel Gene	Adult HGBCL, NOS
BCL-88	chr15	33696332	33696332	G	A	exonic	RYR3	32	29	0.4754	Missense_Mutation	p.S1992N	Targerted Panel Gene	Adult HGBCL, NOS
BCL-88	chr1	119925578	119925578	A	T	exonic	NOTCH2	25	32	0.5614	Missense_Mutation	p.L1413H	Targerted Panel Gene	Adult HGBCL, NOS
BCL-88	chr6	41935971	41935971	G	A	exonic	CCND3	42	60	0.5882	Missense_Mutation	p.T283I	Targerted Panel Gene	Adult HGBCL, NOS
BCL-88	chr17	69313107	69313107	C	A	exonic	ABCA5	52	2	0.037	Missense_Mutation	p.D98Y	Non-Targeted Panel Gene	Adult HGBCL, NOS
BCL-88	chr10	102141957	102141957	G	A	exonic	PPRC1	76	3	0.038	Missense_Mutation	p.R1150H	Non-Targeted Panel Gene	Adult HGBCL, NOS
BCL-88	chr9	84290222	84290222	G	T	exonic	SLC28A3	50	2	0.0385	Missense_Mutation	p.H361N	Non-Targeted Panel Gene	Adult HGBCL, NOS
BCL-88	chr21	46658898	46658898	G	A	exonic	PRMT2	56	3	0.0508	Missense_Mutation	p.E270K	Non-Targeted Panel Gene	Adult HGBCL, NOS
BCL-88	chr11	120905345	120905345	G	A	exonic	GRIK4	74	4	0.0513	Missense_Mutation	p.R443H	Non-Targeted Panel Gene	Adult HGBCL, NOS
BCL-88	chr15	58913555	58913555	C	T	exonic	SLTM	37	2	0.0513	Missense_Mutation	p.E153K	Non-Targeted Panel Gene	Adult HGBCL, NOS

BCL-88	chr11	720090	720090	G	A	exonic	EPS8L2	126	8	0.0597	Missense_Mutation	p.S65N	Non-Targeted Panel Gene	Adult HGBCL, NOS
BCL-88	chr10	97572733	97572733	G	A	exonic	ANKRD2	26	2	0.0714	Missense_Mutation	p.G9E	Non-Targeted Panel Gene	Adult HGBCL, NOS
BCL-88	chr17	3469291	3469291	C	A	exonic	SPATA22	29	3	0.0938	Missense_Mutation	p.S12I	Non-Targeted Panel Gene	Adult HGBCL, NOS
BCL-88	chr18	69564551	69564551	A	G	exonic	DOK6	69	10	0.1266	Missense_Mutation	p.K44R	Non-Targeted Panel Gene	Adult HGBCL, NOS
BCL-88	chr18	25225269	25225269	C	A	exonic	ZNF521	119	20	0.1439	Missense_Mutation	p.E883D	Non-Targeted Panel Gene	Adult HGBCL, NOS
BCL-88	chr2	218685155	218685155	C	T	exonic	STK36	46	9	0.1636	Missense_Mutation	p.A436V	Non-Targeted Panel Gene	Adult HGBCL, NOS
BCL-88	chr11	77203068	77203068	C	T	exonic	MYO7A	10	2	0.1667	Missense_Mutation	p.P1688L	Non-Targeted Panel Gene	Adult HGBCL, NOS
BCL-88	chr16	55327630	55327630	C	T	exonic	IRX6	19	4	0.1739	Missense_Mutation	p.A153V	Non-Targeted Panel Gene	Adult HGBCL, NOS
BCL-88	chr16	48187294	48187294	A	G	exonic	ABCC11	58	14	0.1944	Missense_Mutation	p.M947T	Non-Targeted Panel Gene	Adult HGBCL, NOS
BCL-88	chr5	45645566	45645566	A	T	exonic	HCN1	28	10	0.2632	Missense_Mutation	p.N156K	Non-Targeted Panel Gene	Adult HGBCL, NOS
BCL-88	chr16	70474689	70474689	T	G	exonic	FUK	53	19	0.2639	Missense_Mutation	p.F717C	Non-Targeted Panel Gene	Adult HGBCL, NOS
BCL-88	chr16	724734	724734	T	G	exonic	CCDC78	68	25	0.2688	Missense_Mutation	p.K238Q	Non-Targeted Panel Gene	Adult HGBCL, NOS
BCL-88	chr15	71893735	71893735	T	C	exonic	MYO9A	28	12	0.3	Missense_Mutation	p.K1696E	Non-Targeted Panel Gene	Adult HGBCL, NOS
BCL-88	chr3	122680894	122680894	C	T	exonic	PARP14	145	66	0.3128	Missense_Mutation	p.P4L	Non-Targeted Panel Gene	Adult HGBCL, NOS
BCL-88	chr6	165301643	165301643	C	T	exonic	C6orf118	52	25	0.3205	Missense_Mutation	p.A227T	Non-Targeted Panel Gene	Adult HGBCL, NOS
BCL-88	chr5	116504889	116504889	C	G	exonic	SEMA6A	21	10	0.3226	Missense_Mutation	p.G19A	Non-Targeted Panel Gene	Adult HGBCL, NOS
BCL-88	chr10	100352402	100352402	C	T	exonic	SCD	20	10	0.3333	Missense_Mutation	p.T116I	Non-Targeted Panel Gene	Adult HGBCL, NOS
BCL-88	chr11	34916394	34916394	G	A	exonic	PDHX	45	23	0.3382	Missense_Mutation	p.G8D	Non-Targeted Panel Gene	Adult HGBCL, NOS
BCL-88	chr14	81204005	81204005	G	T	exonic	GTF2A1	31	16	0.3404	Missense_Mutation	p.Q28K	Non-Targeted Panel Gene	Adult HGBCL, NOS
BCL-88	chr3	196229921	196229921	G	A	exonic	SLC51A	62	32	0.3404	Missense_Mutation	p.E214K	Non-Targeted Panel Gene	Adult HGBCL, NOS
BCL-88	chr19	18903357	18903357	G	T	exonic	COPE	43	24	0.3582	Missense_Mutation	p.L239M	Non-Targeted Panel Gene	Adult HGBCL, NOS
BCL-88	chr3	72848198	72848198	C	G	exonic	SHQ1	169	98	0.3657	Missense_Mutation	p.R48T	Non-Targeted Panel Gene	Adult HGBCL, NOS
BCL-88	chr4	155854182	155854182	A	T	exonic	ASIC5	31	18	0.3673	Missense_Mutation	p.S160R	Non-Targeted Panel Gene	Adult HGBCL, NOS
BCL-88	chr6	43451072	43451072	G	A	exonic	DLK2	93	54	0.3673	Missense_Mutation	p.R207C	Non-Targeted Panel Gene	Adult HGBCL, NOS
BCL-88	chr19	35527282	35527282	G	T	exonic	SBSN	123	72	0.3692	Missense_Mutation	p.Q334K	Non-Targeted Panel Gene	Adult HGBCL, NOS
BCL-88	chr8	37841112	37841112	T	C	exonic	ADGRA2	73	46	0.3866	Missense_Mutation	p.L925P	Non-Targeted Panel Gene	Adult HGBCL, NOS
BCL-88	chr4	55118792	55118792	C	G	exonic	KDR	15	10	0.4	Missense_Mutation	p.R57T	Non-Targeted Panel Gene	Adult HGBCL, NOS
BCL-88	chr1	92837557	92837557	A	G	exonic	RPL5	21	14	0.4	Missense_Mutation	p.Y210C	Non-Targeted Panel Gene	Adult HGBCL, NOS
BCL-88	chr15	72350584	72350584	G	A	exonic	HEXA	19	13	0.4062	Missense_Mutation	p.R258W	Non-Targeted Panel Gene	Adult HGBCL, NOS
BCL-88	chr2	178528529	178528529	G	C	exonic	TTN	42	29	0.4085	Missense_Mutation	p.P35741R	Non-Targeted Panel Gene	Adult HGBCL, NOS
BCL-88	chr11	75200341	75200341	G	A	exonic	SLCO2B1	49	34	0.4096	Missense_Mutation	p.A573T	Non-Targeted Panel Gene	Adult HGBCL, NOS
BCL-88	chr21	46397333	46397333	C	T	exonic	PCNT	44	32	0.4211	Missense_Mutation	p.R1429C	Non-Targeted Panel Gene	Adult HGBCL, NOS
BCL-88	chr1	44649756	44649756	G	C	exonic	RNF220	48	35	0.4217	Missense_Mutation	p.C514S	Non-Targeted Panel Gene	Adult HGBCL, NOS
BCL-88	chr11	93793820	93793820	G	C	exonic	MED17	15	11	0.4231	Missense_Mutation	p.D244H	Non-Targeted Panel Gene	Adult HGBCL, NOS
BCL-88	chr1	22511994	22511994	C	T	exonic	ZBTB40	13	10	0.4348	Missense_Mutation	p.T774I	Non-Targeted Panel Gene	Adult HGBCL, NOS
BCL-88	chr9	132542971	132542971	C	T	exonic	CFAP77	36	29	0.4462	Missense_Mutation	p.T255I	Non-Targeted Panel Gene	Adult HGBCL, NOS
BCL-88	chr15	60005147	60005147	C	T	exonic	FOXB1	60	49	0.4495	Missense_Mutation	p.R62C	Non-Targeted Panel Gene	Adult HGBCL, NOS
BCL-88	chr13	95433873	95433873	G	A	exonic	CLDN10	39	32	0.4507	Missense_Mutation	p.G14R	Non-Targeted Panel Gene	Adult HGBCL, NOS
BCL-88	chr14	57480714	57480714	C	A	exonic	C14orf105	28	23	0.451	Missense_Mutation	p.R179I	Non-Targeted Panel Gene	Adult HGBCL, NOS
BCL-88	chr17	1125305	1125305	C	T	exonic	ABR	117	102	0.4658	Missense_Mutation	p.E42K	Non-Targeted Panel Gene	Adult HGBCL, NOS
BCL-88	chr19	7489006	7489006	G	A	exonic	PEX11G	28	26	0.4815	Missense_Mutation	p.A2V	Non-Targeted Panel Gene	Adult HGBCL, NOS
BCL-88	chr15	74023051	74023051	G	A	exonic	PML	50	47	0.4845	Missense_Mutation	p.E276K	Non-Targeted Panel Gene	Adult HGBCL, NOS
BCL-88	chr1	157125602	157125602	T	C	exonic	ETV3	50	48	0.4898	Missense_Mutation	p.N260D	Non-Targeted Panel Gene	Adult HGBCL, NOS
BCL-88	chr9	84290230	84290230	G	C	exonic	SLC28A3	26	25	0.4902	Missense_Mutation	p.S358C	Non-Targeted Panel Gene	Adult HGBCL, NOS
BCL-88	chr1	114726337	114726337	G	A	exonic	CSDE1	31	30	0.4918	Missense_Mutation	p.T551I	Non-Targeted Panel Gene	Adult HGBCL, NOS
BCL-88	chr8	144266837	144266837	G	A	exonic	SCX	46	45	0.4945	Missense_Mutation	p.R75Q	Non-Targeted Panel Gene	Adult HGBCL, NOS
BCL-88	chr10	113589059	113589059	C	A	exonic	NRAP	23	23	0.5	Missense_Mutation	p.E1703D	Non-Targeted Panel Gene	Adult HGBCL, NOS
BCL-88	chr4	70593348	70593348	G	T	exonic	AMBN	38	39	0.5065	Missense_Mutation	p.D13Y	Non-Targeted Panel Gene	Adult HGBCL, NOS
BCL-88	chr17	39634017	39634017	G	A	exonic	PPP1R1B	26	27	0.5094	Missense_Mutation	p.E90K	Non-Targeted Panel Gene	Adult HGBCL, NOS
BCL-88	chr1	42735842	42735842	G	T	exonic	CLDN19	22	24	0.5217	Missense_Mutation	p.H193N	Non-Targeted Panel Gene	Adult HGBCL, NOS
BCL-88	chr6	160079721	160079721	C	G	exonic	IGF2R	26	29	0.5273	Missense_Mutation	p.L1874V	Non-Targeted Panel Gene	Adult HGBCL, NOS
BCL-88	chr1	160799753	160799753	G	T	exonic	LY9	16	18	0.5294	Missense_Mutation	p.G42V	Non-Targeted Panel Gene	Adult HGBCL, NOS
BCL-88	chr14	50628188	50628188	A	G	exonic	ATL1	25	29	0.537	Missense_Mutation	p.D426G	Non-Targeted Panel Gene	Adult HGBCL, NOS
BCL-88	chr17	80960133	80960133	G	A	exonic	RPTOR	33	39	0.5417	Missense_Mutation	p.R1178H	Non-Targeted Panel Gene	Adult HGBCL, NOS
BCL-88	chr17	59197559	59197559	T	A	exonic	PRR11	20	24	0.5455	Missense_Mutation	p.S291R	Non-Targeted Panel Gene	Adult HGBCL, NOS
BCL-88	chr9	5126715	5126715	A	G	exonic	JAK2	26	32	0.5517	Missense_Mutation	p.N1108S	Non-Targeted Panel Gene	Adult HGBCL, NOS
BCL-88	chr10	102066135	102066135	C	A	exonic	HPS6	60	77	0.562	Missense_Mutation	p.P221T	Non-Targeted Panel Gene	Adult HGBCL, NOS
BCL-88	chr5	148203154	148203154	G	A	exonic	SPINK6	19	25	0.5682	Missense_Mutation	p.G20S	Non-Targeted Panel Gene	Adult HGBCL, NOS
BCL-88	chr1	173982891	173982891	C	T	exonic	RC3H1	16	23	0.5897	Missense_Mutation	p.E202K	Non-Targeted Panel Gene	Adult HGBCL, NOS
BCL-88	chr8	17210452	17210452	C	A	exonic	ZDHHC2	20	30	0.6	Missense_Mutation	p.P308T	Non-Targeted Panel Gene	Adult HGBCL, NOS
BCL-88	chr8	1780475	1780475	C	A	exonic	CLN8	26	41	0.6119	Missense_Mutation	p.L257I	Non-Targeted Panel Gene	Adult HGBCL, NOS
BCL-88	chr2	31383784	31383784	T	C	exonic	XDH	16	26	0.619	Missense_Mutation	p.E286G	Non-Targeted Panel Gene	Adult HGBCL, NOS
BCL-88	chr17	75209832	75209832	A	G	exonic	NUP85	5	9	0.6429	Missense_Mutation	p.E46G	Non-Targeted Panel Gene	Adult HGBCL, NOS
BCL-88	chr16	28477623	28477623	G	T	exonic	CLN3	30	66	0.6875	Missense_Mutation	p.H404N	Non-Targeted Panel Gene	Adult HGBCL, NOS
BCL-88	chr18	10789342	10789342	C	G	exonic	PIEZO2	5	31	0.8611	Missense_Mutation	p.V636L	Non-Targeted Panel Gene	Adult HGBCL, NOS
BCL-88	chrX	108601436	108601436	G	T	exonic	COL4A5	2	14	0.875	Missense_Mutation	p.K664N	Non-Targeted Panel Gene	Adult HGBCL, NOS
BCL-88	chr15	58913551	58913551	A	-	exonic	SLTM	36	2	0.0526	Nonsense_Mutation	p.L154X	Non-Targeted Panel Gene	Adult HGBCL, NOS
BCL-88	chr11	9033767	9033767	C	A	exonic	SCUBE2	26	2	0.0714	Nonsense_Mutation	p.G649X	Non-Targeted Panel Gene	Adult HGBCL, NOS
BCL-88	chr10	119042279	119042279	G	A	exonic	EIF3A	86	8	0.0851	Nonsense_Mutation	p.R1081X	Non-Targeted Panel Gene	Adult HGBCL, NOS
BCL-88	chr11	30903515	30903515	C	A	exonic	DCDC1	12	2	0.1429	Nonsense_Mutation	p.G1112X	Non-Targeted Panel Gene	Adult HGBCL, NOS
BCL-88	chr2	218812730	218812730	G	A	exonic	CYP27A1	40	36	0.4737	Nonsense_Mutation	p.W275X	Non-Targeted Panel Gene	Adult HGBCL, NOS
BCL-88	chr16	68166892	68166892	C	T	exonic	NFATC3	19	32	0.6275	Nonsense_Mutation	p.R551X	Non-Targeted Panel Gene	Adult HGBCL, NOS
BCL-89	chr6	41936041	41936042	GA	-	exonic	CCND3	181	122	0.4026	Frame_Shift_Del	p.S259fs	Targerted Panel Gene	Adult HGBCL, NOS
BCL-89	chrX	41343280	41343280	-	A	exonic	DDX3X	30	169	0.8492	Frame_Shift_Ins	p.P203fs	Targerted Panel Gene	Adult HGBCL, NOS
BCL-89	chr1	23559258	23559258	-	GG	exonic	ID3	213	136	0.3886	Frame_Shift_Ins	p.G57fs	Targerted Panel Gene	Adult HGBCL, NOS
BCL-89	chr8	127738431	127738431	C	T	exonic	MYC	153	179	0.5392	Missense_Mutation	p.P72S	Targerted Panel Gene	Adult HGBCL, NOS
BCL-89	chr8	127738432	127738432	C	A	exonic	MYC	153	176	0.535	Missense_Mutation	p.P72H	Targerted Panel Gene	Adult HGBCL, NOS
BCL-89	chr8	127738723	127738723	C	G	exonic	MYC	155	153	0.4968	Missense_Mutation	p.A169G	Targerted Panel Gene	Adult HGBCL, NOS
BCL-89	chr9	136504743	136504743	C	T	exonic	NOTCH1	103	61	0.372	Missense_Mutation	p.A1650T	Targerted Panel Gene	Adult HGBCL, NOS
BCL-89	chr10	87864492	87864492	T	A	exonic	PTEN	235	228	0.4924	Missense_Mutation	p.I181N	Targerted Panel Gene	Adult HGBCL, NOS
BCL-89	chr6	117319983	117319983	C	A	exonic	ROS1	235	220	0.4835	Missense_Mutation	p.R1942L	Targerted Panel Gene	Adult HGBCL, NOS
BCL-89	chr14	64786746	64786746	G	C	exonic	SPTB	111	108	0.4909	Missense_Mutation	p.F1073L	Targerted Panel Gene	Adult HGBCL, NOS

BCL-89	chr15	42687414	42687414	T	C	exonic	STARD9	293	251	0.4614	Missense_Mutation	p.S1946P	Targerted Panel Gene	Adult HGBCL, NOS
BCL-89	chr17	7674241	7674241	G	C	exonic	TP53	10	192	0.9505	Missense_Mutation	p.S202C	Targerted Panel Gene	Adult HGBCL, NOS
BCL-89	chr6	128067597	128067597	G	T	exonic;splicing	PTPRK;LOC101928	273	242	0.4681	Nonsense_Mutation	p.Y693X	Targerted Panel Gene	Adult HGBCL, NOS
BCL-89	chr4	105236065	105236065	C	G	exonic	TET2	414	364	0.4673	Nonsense_Mutation	p.S708X	Targerted Panel Gene	Adult HGBCL, NOS
BCL-9	chr3	193643445	193643445	-	T	splicing	OPA1	111	53	0.3232	Splice_Site	.	Non-Targeted Panel Gene	Adult HGBCL, NOS
BCL-9	chr11	123653827	123653827	C	T	splicing	SCN3B	64	41	0.3905	Splice_Site	.	Non-Targeted Panel Gene	Adult HGBCL, NOS
BCL-9	chr12	11650161	11650161	G	A	splicing	ETV6	180	127	0.4137	Splice_Site	.	Non-Targeted Panel Gene	Adult HGBCL, NOS
BCL-9	chr14	74480701	74480701	C	T	splicing	NPC2	234	193	0.4499	Splice_Site	.	Non-Targeted Panel Gene	Adult HGBCL, NOS
BCL-9	chrX	54958363	54958363	C	G	splicing	PFKFB1	5	101	0.9528	Splice_Site	.	Non-Targeted Panel Gene	Adult HGBCL, NOS
BCL-9	chr20	34413878	34413881	AAGT	-	exonic	ITCH	152	69	0.3122	Frame_Shift_Del	p.E158fs	Targerted Panel Gene	Adult HGBCL, NOS
BCL-9	chr16	10895383	10895387	GACCA	-	exonic	CIITA	168	100	0.3731	Frame_Shift_Del	p.D52fs	Targerted Panel Gene	Adult HGBCL, NOS
BCL-9	chr17	65056348	65056349	CT	-	exonic	GNA13	84	55	0.3957	Frame_Shift_Del	p.E82fs	Targerted Panel Gene	Adult HGBCL, NOS
BCL-9	chr2	74534570	74534570	T	-	exonic	LOXL3	287	181	0.3868	Frame_Shift_Del	p.K595fs	Non-Targeted Panel Gene	Adult HGBCL, NOS
BCL-9	chr18	31546259	31546260	CA	-	exonic	DSG2	192	149	0.4357	Frame_Shift_Del	p.P958fs	Non-Targeted Panel Gene	Adult HGBCL, NOS
BCL-9	chrX	119837970	119837973	TCTT	-	exonic	UPF3B	50	101	0.6689	Frame_Shift_Del	p.E362fs	Non-Targeted Panel Gene	Adult HGBCL, NOS
BCL-9	chr16	10909127	10909127	-	T	exonic	CIITA	134	99	0.4195	Frame_Shift_Ins	p.P920fs	Targerted Panel Gene	Adult HGBCL, NOS
BCL-9	chr9	130347329	130347329	-	A	exonic	HMCN2	37	17	0.3148	Frame_Shift_Ins	p.S1331fs	Non-Targeted Panel Gene	Adult HGBCL, NOS
BCL-9	chr5	179825183	179825185	GAA	-	exonic	SQSTM1	106	62	0.369	In_Frame_Del	p.153_154del	Non-Targeted Panel Gene	Adult HGBCL, NOS
BCL-9	chr7	100954135	100954135	G	A	exonic	MUC3A	914	52	0.0538	Missense_Mutation	p.A786T	Targerted Panel Gene	Adult HGBCL, NOS
BCL-9	chr22	22888214	22888214	C	T	exonic	IGLL5	78	29	0.271	Missense_Mutation	p.P19S	Targerted Panel Gene	Adult HGBCL, NOS
BCL-9	chr22	22888180	22888180	G	T	exonic	IGLL5	96	48	0.3333	Missense_Mutation	p.W7C	Targerted Panel Gene	Adult HGBCL, NOS
BCL-9	chr19	11215881	11215881	G	T	exonic	DOCK6	141	74	0.3442	Missense_Mutation	p.S1314Y	Targerted Panel Gene	Adult HGBCL, NOS
BCL-9	chr5	138465955	138465955	G	A	exonic	EGR1	122	65	0.3476	Missense_Mutation	p.S65N	Targerted Panel Gene	Adult HGBCL, NOS
BCL-9	chr1	203305634	203305634	C	T	exonic	BTG2	169	95	0.3598	Missense_Mutation	p.L10F	Targerted Panel Gene	Adult HGBCL, NOS
BCL-9	chr2	51027658	51027658	C	T	exonic	NRXN1	209	121	0.3667	Missense_Mutation	p.D206N	Targerted Panel Gene	Adult HGBCL, NOS
BCL-9	chr5	138465951	138465951	A	T	exonic	EGR1	124	73	0.3706	Missense_Mutation	p.S64C	Targerted Panel Gene	Adult HGBCL, NOS
BCL-9	chr6	56555479	56555479	T	G	exonic	DST	259	160	0.3819	Missense_Mutation	p.K2882T	Targerted Panel Gene	Adult HGBCL, NOS
BCL-9	chr15	33530634	33530634	G	A	exonic	RYR3	98	64	0.3951	Missense_Mutation	p.A108T	Targerted Panel Gene	Adult HGBCL, NOS
BCL-9	chr8	3181110	3181110	T	G	exonic	CSMD1	81	54	0.3971	Missense_Mutation	p.T1909P	Targerted Panel Gene	Adult HGBCL, NOS
BCL-9	chr4	125463662	125463662	C	T	exonic	FAT4	147	97	0.3975	Missense_Mutation	p.P3965L	Targerted Panel Gene	Adult HGBCL, NOS
BCL-9	chr10	52293863	52293863	C	A	exonic	PRKG1	150	102	0.4048	Missense_Mutation	p.P675Q	Targerted Panel Gene	Adult HGBCL, NOS
BCL-9	chr12	113058291	113058291	G	C	exonic	DTX1	107	73	0.4056	Missense_Mutation	p.E33D	Targerted Panel Gene	Adult HGBCL, NOS
BCL-9	chr19	24127079	24127079	G	T	exonic	ZNF254	348	241	0.4092	Missense_Mutation	p.S319I	Targerted Panel Gene	Adult HGBCL, NOS
BCL-9	chr3	7578942	7578942	G	A	exonic	GRM7	140	99	0.4142	Missense_Mutation	p.R679Q	Targerted Panel Gene	Adult HGBCL, NOS
BCL-9	chr6	27866938	27866938	G	A	exonic	HIST1H1B	114	84	0.4221	Missense_Mutation	p.P198S	Targerted Panel Gene	Adult HGBCL, NOS
BCL-9	chr22	22895671	22895671	G	A	exonic	IGLL5	319	234	0.4231	Missense_Mutation	p.V208M	Targerted Panel Gene	Adult HGBCL, NOS
BCL-9	chr12	49031191	49031191	G	T	exonic	KMT2D	325	247	0.4318	Missense_Mutation	p.T4505N	Targerted Panel Gene	Adult HGBCL, NOS
BCL-9	chr17	63929438	63929438	T	C	exonic	CD79B	138	105	0.4321	Missense_Mutation	p.Y197C	Targerted Panel Gene	Adult HGBCL, NOS
BCL-9	chr22	22893792	22893792	G	A	exonic	IGLL5	466	369	0.4419	Missense_Mutation	p.G100E	Targerted Panel Gene	Adult HGBCL, NOS
BCL-9	chr17	39777678	39777678	T	C	exonic	IKZF3	363	288	0.4424	Missense_Mutation	p.K20E	Targerted Panel Gene	Adult HGBCL, NOS
BCL-9	chr1	26697008	26697008	C	G	exonic	ARID1A	335	282	0.4571	Missense_Mutation	p.S202C	Targerted Panel Gene	Adult HGBCL, NOS
BCL-9	chr22	22888194	22888194	C	G	exonic	IGLL5	67	63	0.4846	Missense_Mutation	p.A12G	Targerted Panel Gene	Adult HGBCL, NOS
BCL-9	chr22	22888126	22888126	C	G	exonic	IGLL5	104	102	0.4951	Missense_Mutation	p.P25A	Targerted Panel Gene	Adult HGBCL, NOS
BCL-9	chr4	105237193	105237193	A	C	exonic	TET2	110	111	0.5023	Missense_Mutation	p.Q1084P	Targerted Panel Gene	Adult HGBCL, NOS
BCL-9	chrX	101358645	101358645	T	C	exonic	BTK	29	113	0.7958	Missense_Mutation	p.T316A	Targerted Panel Gene	Adult HGBCL, NOS
BCL-9	chrX	31679375	31679375	C	A	exonic	DMD	11	43	0.7963	Missense_Mutation	p.K2501N	Targerted Panel Gene	Adult HGBCL, NOS
BCL-9	chr3	177047310	177047310	C	A	exonic	TBL1XR1	88	538	0.8594	Missense_Mutation	p.G285V	Targerted Panel Gene	Adult HGBCL, NOS
BCL-9	chr19	1581269	1581269	C	A	exonic	MBD3	88	5	0.0538	Missense_Mutation	p.G135V	Non-Targeted Panel Gene	Adult HGBCL, NOS
BCL-9	chrX	153647970	153647970	G	A	exonic	DUSP9	68	4	0.0556	Missense_Mutation	p.R6H	Non-Targeted Panel Gene	Adult HGBCL, NOS
BCL-9	chr6	32761713	32761713	T	G	exonic	HLA-DQB2	112	7	0.0588	Missense_Mutation	p.K104T	Non-Targeted Panel Gene	Adult HGBCL, NOS
BCL-9	chr1	151707851	151707851	A	C	exonic	CELF3	77	6	0.0723	Missense_Mutation	p.L191V	Non-Targeted Panel Gene	Adult HGBCL, NOS
BCL-9	chr11	1016937	1016937	G	C	exonic	MUC6	776	67	0.0794	Missense_Mutation	p.T1955S	Non-Targeted Panel Gene	Adult HGBCL, NOS
BCL-9	chr6	31630699	31630699	A	G	exonic	PRRC2A	156	18	0.1034	Missense_Mutation	p.K788R	Non-Targeted Panel Gene	Adult HGBCL, NOS
BCL-9	chr6	152329783	152329783	C	T	exonic	SYNE1	103	20	0.1626	Missense_Mutation	p.A4968T	Non-Targeted Panel Gene	Adult HGBCL, NOS
BCL-9	chr6	31271318	31271318	C	T	exonic	HLA-C	105	40	0.2759	Missense_Mutation	p.C125Y	Non-Targeted Panel Gene	Adult HGBCL, NOS
BCL-9	chr15	41815624	41815624	G	A	exonic	MAPKBP1	33	14	0.2979	Missense_Mutation	p.D446N	Non-Targeted Panel Gene	Adult HGBCL, NOS
BCL-9	chr16	67936251	67936251	C	T	exonic	PSMB10	204	90	0.3061	Missense_Mutation	p.S69N	Non-Targeted Panel Gene	Adult HGBCL, NOS
BCL-9	chr2	206017777	206017777	C	A	exonic	INO80D	148	71	0.3242	Missense_Mutation	p.S482I	Non-Targeted Panel Gene	Adult HGBCL, NOS
BCL-9	chr19	35250659	35250659	A	C	exonic	LSR	27	13	0.325	Missense_Mutation	p.N200H	Non-Targeted Panel Gene	Adult HGBCL, NOS
BCL-9	chr3	31980902	31980902	T	C	exonic	OSBPL10	31	15	0.3261	Missense_Mutation	p.N93S	Non-Targeted Panel Gene	Adult HGBCL, NOS
BCL-9	chr2	119681194	119681194	T	A	exonic	TMEM177	156	79	0.3362	Missense_Mutation	p.L114Q	Non-Targeted Panel Gene	Adult HGBCL, NOS
BCL-9	chr19	45940381	45940381	C	T	exonic	NOVA2	31	16	0.3404	Missense_Mutation	p.A321T	Non-Targeted Panel Gene	Adult HGBCL, NOS
BCL-9	chr12	11839248	11839248	G	A	exonic	ETV6	214	111	0.3415	Missense_Mutation	p.G91D	Non-Targeted Panel Gene	Adult HGBCL, NOS
BCL-9	chr11	128988059	128988059	C	T	exonic	ARHGAP32	143	75	0.344	Missense_Mutation	p.R407H	Non-Targeted Panel Gene	Adult HGBCL, NOS
BCL-9	chr2	119681192	119681192	C	G	exonic	TMEM177	155	82	0.346	Missense_Mutation	p.D113E	Non-Targeted Panel Gene	Adult HGBCL, NOS
BCL-9	chr22	21634090	21634090	C	G	exonic	CCDC116	94	51	0.3517	Missense_Mutation	p.H47Q	Non-Targeted Panel Gene	Adult HGBCL, NOS
BCL-9	chr2	176151676	176151676	C	A	exonic	HOXD4	66	36	0.3529	Missense_Mutation	p.P15T	Non-Targeted Panel Gene	Adult HGBCL, NOS
BCL-9	chr7	127694909	127694909	C	T	exonic	SND1	88	48	0.3529	Missense_Mutation	p.P104S	Non-Targeted Panel Gene	Adult HGBCL, NOS
BCL-9	chr6	47878584	47878584	C	T	exonic	PTCHD4	184	102	0.3566	Missense_Mutation	p.D754N	Non-Targeted Panel Gene	Adult HGBCL, NOS
BCL-9	chr4	7718070	7718070	G	A	exonic	SORCS2	89	50	0.3597	Missense_Mutation	p.V771M	Non-Targeted Panel Gene	Adult HGBCL, NOS
BCL-9	chr8	119569259	119569259	A	G	exonic	ENPP2	185	105	0.3621	Missense_Mutation	p.S729P	Non-Targeted Panel Gene	Adult HGBCL, NOS
BCL-9	chr11	77258405	77258405	T	C	exonic	GDPD4	80	46	0.3651	Missense_Mutation	p.K282R	Non-Targeted Panel Gene	Adult HGBCL, NOS
BCL-9	chr1	27334025	27334025	G	A	exonic	TMEM222	77	46	0.374	Missense_Mutation	p.D127N	Non-Targeted Panel Gene	Adult HGBCL, NOS
BCL-9	chr10	53840419	53840419	C	T	exonic	PCDH15	184	111	0.3763	Missense_Mutation	p.G1300E	Non-Targeted Panel Gene	Adult HGBCL, NOS
BCL-9	chr15	73116806	73116806	G	T	exonic	NEO1	152	92	0.377	Missense_Mutation	p.V133F	Non-Targeted Panel Gene	Adult HGBCL, NOS
BCL-9	chr10	61902183	61902183	C	T	exonic	ARID5B	89	54	0.3776	Missense_Mutation	p.H16Y	Non-Targeted Panel Gene	Adult HGBCL, NOS
BCL-9	chr3	165188320	165188320	G	T	exonic	SLITRK3	125	76	0.3781	Missense_Mutation	p.H837Q	Non-Targeted Panel Gene	Adult HGBCL, NOS
BCL-9	chr1	99881146	99881146	C	G	exonic	AGL	134	82	0.3796	Missense_Mutation	p.T641R	Non-Targeted Panel Gene	Adult HGBCL, NOS
BCL-9	chr19	45762139	45762139	C	A	exonic	BHMG1	83	52	0.3852	Missense_Mutation	p.N545K	Non-Targeted Panel Gene	Adult HGBCL, NOS
BCL-9	chr10	53822749	53822749	G	T	exonic	PCDH15	1019	639	0.3852	Missense_Mutation	p.D1666E	Non-Targeted Panel Gene	Adult HGBCL, NOS
BCL-9	chr5	83521718	83521718	C	A	exonic	VCAN	141	89	0.3853	Missense_Mutation	p.Q1138K	Non-Targeted Panel Gene	Adult HGBCL, NOS
BCL-9	chr11	4860272	4860272	G	C	exonic	OR51H1	194	123	0.388	Missense_Mutation	p.A98G	Non-Targeted Panel Gene	Adult HGBCL, NOS

BCL-9	chr19	52065957	52065957	C	T	exonic	ZNF841	375	239	0.3893	Missense_Mutation	p.R642H	Non-Targeted Panel Gene	Adult HGBCL, NOS
BCL-9	chr10	11869912	11869912	T	A	exonic	PROSER2	247	158	0.3901	Missense_Mutation	p.S272T	Non-Targeted Panel Gene	Adult HGBCL, NOS
BCL-9	chr5	36049383	36049383	A	C	exonic	UGT3A2	111	71	0.3901	Missense_Mutation	p.L117V	Non-Targeted Panel Gene	Adult HGBCL, NOS
BCL-9	chr3	36715438	36715438	T	A	exonic	DCLK3	214	137	0.3903	Missense_Mutation	p.I613F	Non-Targeted Panel Gene	Adult HGBCL, NOS
BCL-9	chr10	6498502	6498502	G	A	exonic	PRKCQ	128	82	0.3905	Missense_Mutation	p.R146W	Non-Targeted Panel Gene	Adult HGBCL, NOS
BCL-9	chr1	184051876	184051876	G	T	exonic	TSEN15	115	74	0.3915	Missense_Mutation	p.G41C	Non-Targeted Panel Gene	Adult HGBCL, NOS
BCL-9	chr12	123696492	123696492	T	C	exonic	TCTN2	226	147	0.393	Missense_Mutation	p.S464P	Non-Targeted Panel Gene	Adult HGBCL, NOS
BCL-9	chr5	181230745	181230745	C	T	exonic	TRIM41	123	80	0.3941	Missense_Mutation	p.A272V	Non-Targeted Panel Gene	Adult HGBCL, NOS
BCL-9	chr5	44809548	44809548	G	A	exonic	MRPS30	66	43	0.3945	Missense_Mutation	p.A196T	Non-Targeted Panel Gene	Adult HGBCL, NOS
BCL-9	chr3	170128956	170128956	A	G	exonic	PHC3	183	120	0.3947	Missense_Mutation	p.S506P	Non-Targeted Panel Gene	Adult HGBCL, NOS
BCL-9	chr2	61314723	61314723	T	C	exonic	USP34	205	135	0.3971	Missense_Mutation	p.E1135G	Non-Targeted Panel Gene	Adult HGBCL, NOS
BCL-9	chr12	6587816	6587816	C	T	exonic	CHD4	159	105	0.3977	Missense_Mutation	p.R1200Q	Non-Targeted Panel Gene	Adult HGBCL, NOS
BCL-9	chr19	1073982	1073982	G	A	exonic	ARHGAP45	68	45	0.3982	Missense_Mutation	p.G253D	Non-Targeted Panel Gene	Adult HGBCL, NOS
BCL-9	chr18	31025850	31025850	T	G	exonic	DSC3	127	85	0.4009	Missense_Mutation	p.K180N	Non-Targeted Panel Gene	Adult HGBCL, NOS
BCL-9	chr8	99511141	99511141	A	G	exonic	VPS13B	109	73	0.4011	Missense_Mutation	p.H1421R	Non-Targeted Panel Gene	Adult HGBCL, NOS
BCL-9	chr1	68444632	68444632	C	T	exonic	RPE65	173	116	0.4014	Missense_Mutation	p.A132T	Non-Targeted Panel Gene	Adult HGBCL, NOS
BCL-9	chr1	11087551	11087551	A	C	exonic	EXOSC10	147	99	0.4024	Missense_Mutation	p.M329R	Non-Targeted Panel Gene	Adult HGBCL, NOS
BCL-9	chr7	8221311	8221311	G	A	exonic	ICA1	92	62	0.4026	Missense_Mutation	p.A41V	Non-Targeted Panel Gene	Adult HGBCL, NOS
BCL-9	chr14	24103525	24103525	A	G	exonic	PCK2	102	69	0.4035	Missense_Mutation	p.H495R	Non-Targeted Panel Gene	Adult HGBCL, NOS
BCL-9	chr20	23048898	23048898	C	A	exonic	THBD	31	21	0.4038	Missense_Mutation	p.D203Y	Non-Targeted Panel Gene	Adult HGBCL, NOS
BCL-9	chr11	66564604	66564604	G	T	exonic	CTSF	121	82	0.4039	Missense_Mutation	p.S425R	Non-Targeted Panel Gene	Adult HGBCL, NOS
BCL-9	chr17	47361460	47361460	C	G	exonic	EFCAB13	168	114	0.4043	Missense_Mutation	p.S248R	Non-Targeted Panel Gene	Adult HGBCL, NOS
BCL-9	chr7	117592329	117592329	T	A	exonic	CFTR	772	527	0.4057	Missense_Mutation	p.M721K	Non-Targeted Panel Gene	Adult HGBCL, NOS
BCL-9	chr7	117735370	117735370	A	T	exonic	CTTNBP2	54	37	0.4066	Missense_Mutation	p.I1196N	Non-Targeted Panel Gene	Adult HGBCL, NOS
BCL-9	chr5	141398586	141398586	A	G	exonic	PCDHGB5	156	107	0.4068	Missense_Mutation	p.I153M	Non-Targeted Panel Gene	Adult HGBCL, NOS
BCL-9	chr1	155725974	155725974	C	A	exonic	DAP3	139	96	0.4085	Missense_Mutation	p.H143N	Non-Targeted Panel Gene	Adult HGBCL, NOS
BCL-9	chr1	153991570	153991570	C	G	exonic	RPS27	146	101	0.4089	Missense_Mutation	p.C8W	Non-Targeted Panel Gene	Adult HGBCL, NOS
BCL-9	chr12	118095619	118095619	G	A	exonic	VSIG10	272	189	0.4091	Missense_Mutation	p.S92L	Non-Targeted Panel Gene	Adult HGBCL, NOS
BCL-9	chr5	116467733	116467733	G	A	exonic	SEMA6A	187	132	0.4138	Missense_Mutation	p.L599F	Non-Targeted Panel Gene	Adult HGBCL, NOS
BCL-9	chr5	140822338	140822338	C	T	exonic	PCDHA5	181	128	0.4142	Missense_Mutation	p.T188M	Non-Targeted Panel Gene	Adult HGBCL, NOS
BCL-9	chr17	44904248	44904248	T	C	exonic	FAM187A	212	150	0.4144	Missense_Mutation	p.I140T	Non-Targeted Panel Gene	Adult HGBCL, NOS
BCL-9	chr5	22078525	22078525	C	T	exonic	CDH12	169	121	0.4172	Missense_Mutation	p.R51H	Non-Targeted Panel Gene	Adult HGBCL, NOS
BCL-9	chr2	61109205	61109205	G	C	exonic	KIAA1841	74	53	0.4173	Missense_Mutation	p.Q551H	Non-Targeted Panel Gene	Adult HGBCL, NOS
BCL-9	chr19	15265565	15265565	G	A	exonic	BRD4	183	134	0.4227	Missense_Mutation	p.P213L	Non-Targeted Panel Gene	Adult HGBCL, NOS
BCL-9	chr4	117085083	117085083	T	G	exonic	TRAM1L1	195	144	0.4248	Missense_Mutation	p.K104T	Non-Targeted Panel Gene	Adult HGBCL, NOS
BCL-9	chr13	105489989	105489989	C	A	exonic	DAOA	138	103	0.4274	Missense_Mutation	p.Q53K	Non-Targeted Panel Gene	Adult HGBCL, NOS
BCL-9	chr4	151586692	151586692	G	A	exonic	FAM160A1	105	79	0.4293	Missense_Mutation	p.E262K	Non-Targeted Panel Gene	Adult HGBCL, NOS
BCL-9	chr17	7852597	7852597	C	T	exonic	KDM6B	118	89	0.43	Missense_Mutation	p.T1524M	Non-Targeted Panel Gene	Adult HGBCL, NOS
BCL-9	chr8	86431412	86431412	G	A	exonic	WWP1	41	31	0.4306	Missense_Mutation	p.R465K	Non-Targeted Panel Gene	Adult HGBCL, NOS
BCL-9	chr9	127108476	127108476	T	C	exonic	ANGPTL2	51	39	0.4333	Missense_Mutation	p.K86E	Non-Targeted Panel Gene	Adult HGBCL, NOS
BCL-9	chr5	148086502	148086502	A	G	exonic	SPINK5	121	93	0.4346	Missense_Mutation	p.D127G	Non-Targeted Panel Gene	Adult HGBCL, NOS
BCL-9	chr1	69760321	69760321	G	T	exonic	LRRC7	128	99	0.4361	Missense_Mutation	p.K44N	Non-Targeted Panel Gene	Adult HGBCL, NOS
BCL-9	chr3	42686276	42686276	G	A	exonic	KLHL40	27	21	0.4375	Missense_Mutation	p.V220I	Non-Targeted Panel Gene	Adult HGBCL, NOS
BCL-9	chr12	6057850	6057850	C	A	exonic	VWF	90	70	0.4375	Missense_Mutation	p.M576I	Non-Targeted Panel Gene	Adult HGBCL, NOS
BCL-9	chr14	70733133	70733133	G	A	exonic	MAP3K9	77	60	0.438	Missense_Mutation	p.H760Y	Non-Targeted Panel Gene	Adult HGBCL, NOS
BCL-9	chr5	2748755	2748755	G	A	exonic	IRX2	23	18	0.439	Missense_Mutation	p.P318L	Non-Targeted Panel Gene	Adult HGBCL, NOS
BCL-9	chr4	169707072	169707072	C	T	exonic	CLCN3	268	210	0.4393	Missense_Mutation	p.T652I	Non-Targeted Panel Gene	Adult HGBCL, NOS
BCL-9	chr18	70166070	70166070	T	C	exonic	RTTN	116	91	0.4396	Missense_Mutation	p.I641V	Non-Targeted Panel Gene	Adult HGBCL, NOS
BCL-9	chr2	166015636	166015636	G	C	exonic	SCN1A	192	151	0.4402	Missense_Mutation	p.T1174S	Non-Targeted Panel Gene	Adult HGBCL, NOS
BCL-9	chr10	19205182	19205182	G	A	exonic	MALRD1	137	108	0.4408	Missense_Mutation	p.R832H	Non-Targeted Panel Gene	Adult HGBCL, NOS
BCL-9	chr2	178541317	178541317	C	T	exonic	TTN	119	94	0.4413	Missense_Mutation	p.R32587H	Non-Targeted Panel Gene	Adult HGBCL, NOS
BCL-9	chr6	43645246	43645246	A	C	exonic	RSPH9	118	95	0.446	Missense_Mutation	p.I50L	Non-Targeted Panel Gene	Adult HGBCL, NOS
BCL-9	chr8	144931649	144931649	C	T	exonic	ZNF16	147	119	0.4474	Missense_Mutation	p.G380S	Non-Targeted Panel Gene	Adult HGBCL, NOS
BCL-9	chr3	164982401	164982401	T	C	exonic	SI	100	81	0.4475	Missense_Mutation	p.T1753A	Non-Targeted Panel Gene	Adult HGBCL, NOS
BCL-9	chr5	180850815	180850815	C	A	exonic	ZFP62	279	226	0.4475	Missense_Mutation	p.C194F	Non-Targeted Panel Gene	Adult HGBCL, NOS
BCL-9	chr10	25175789	25175789	C	G	exonic	GPR158	107	87	0.4485	Missense_Mutation	p.H123Q	Non-Targeted Panel Gene	Adult HGBCL, NOS
BCL-9	chr1	1927021	1927021	C	T	exonic	CFAP74	45	37	0.4512	Missense_Mutation	p.E1179K	Non-Targeted Panel Gene	Adult HGBCL, NOS
BCL-9	chr8	23103155	23103155	G	A	exonic	TNFRSF10C	197	162	0.4513	Missense_Mutation	p.V12I	Non-Targeted Panel Gene	Adult HGBCL, NOS
BCL-9	chr3	8567576	8567576	G	A	exonic	LMCD1	51	42	0.4516	Missense_Mutation	p.C359Y	Non-Targeted Panel Gene	Adult HGBCL, NOS
BCL-9	chr13	78602307	78602307	G	A	exonic	POU4F1	46	38	0.4524	Missense_Mutation	p.P123L	Non-Targeted Panel Gene	Adult HGBCL, NOS
BCL-9	chr9	127711350	127711350	C	A	exonic	CFAP157	81	67	0.4527	Missense_Mutation	p.Q237K	Non-Targeted Panel Gene	Adult HGBCL, NOS
BCL-9	chr18	24159726	24159726	A	C	exonic	CABYR	171	142	0.4537	Missense_Mutation	p.N266H	Non-Targeted Panel Gene	Adult HGBCL, NOS
BCL-9	chr5	5457413	5457413	G	C	exonic	ICE1	108	90	0.4545	Missense_Mutation	p.R258T	Non-Targeted Panel Gene	Adult HGBCL, NOS
BCL-9	chr9	131085639	131085639	G	C	exonic	LAMC3	101	85	0.457	Missense_Mutation	p.R1382S	Non-Targeted Panel Gene	Adult HGBCL, NOS
BCL-9	chr13	49145875	49145875	A	G	exonic	FNDC3A	195	165	0.4583	Missense_Mutation	p.Y306C	Non-Targeted Panel Gene	Adult HGBCL, NOS
BCL-9	chr15	44490789	44490789	T	C	exonic	CTDSPL2	41	35	0.4605	Missense_Mutation	p.S161P	Non-Targeted Panel Gene	Adult HGBCL, NOS
BCL-9	chr7	142876546	142876546	G	T	exonic	TRPV6	107	92	0.4623	Missense_Mutation	p.N248K	Non-Targeted Panel Gene	Adult HGBCL, NOS
BCL-9	chr5	140674776	140674776	T	G	exonic	HARS	224	196	0.4645	Missense_Mutation	p.Y454S	Non-Targeted Panel Gene	Adult HGBCL, NOS
BCL-9	chr3	134650875	134650875	G	A	exonic	KY	113	99	0.4648	Missense_Mutation	p.T29M	Non-Targeted Panel Gene	Adult HGBCL, NOS
BCL-9	chr17	10324263	10324263	T	G	exonic	MYH13	122	106	0.4649	Missense_Mutation	p.E898A	Non-Targeted Panel Gene	Adult HGBCL, NOS
BCL-9	chr20	21705753	21705753	T	G	exonic	PAX1	42	38	0.4691	Missense_Mutation	p.V14G	Non-Targeted Panel Gene	Adult HGBCL, NOS
BCL-9	chr19	33205753	33205753	G	A	exonic	LRP3	58	52	0.4727	Missense_Mutation	p.R328H	Non-Targeted Panel Gene	Adult HGBCL, NOS
BCL-9	chr19	17808016	17808016	A	T	exonic	B3GNT3	98	89	0.4734	Missense_Mutation	p.H70L	Non-Targeted Panel Gene	Adult HGBCL, NOS
BCL-9	chr18	31006949	31006949	A	C	exonic	DSC3	70	63	0.4737	Missense_Mutation	p.S616A	Non-Targeted Panel Gene	Adult HGBCL, NOS
BCL-9	chr2	106843407	106843407	C	A	exonic	ST6GAL2	93	84	0.4746	Missense_Mutation	p.D191Y	Non-Targeted Panel Gene	Adult HGBCL, NOS
BCL-9	chr11	124870719	124870719	A	G	exonic	ROBO3	99	90	0.4762	Missense_Mutation	p.S342G	Non-Targeted Panel Gene	Adult HGBCL, NOS
BCL-9	chr10	105211198	105211198	G	A	exonic	SORCS3	96	88	0.4783	Missense_Mutation	p.A775T	Non-Targeted Panel Gene	Adult HGBCL, NOS
BCL-9	chr6	116462092	116462092	T	A	exonic	FAM26F	30	28	0.4828	Missense_Mutation	p.F55I	Non-Targeted Panel Gene	Adult HGBCL, NOS
BCL-9	chr2	108470912	108470912	C	A	exonic	GCC2	124	116	0.4833	Missense_Mutation	p.T528N	Non-Targeted Panel Gene	Adult HGBCL, NOS
BCL-9	chr15	31483557	31483557	T	C	exonic	OTUD7A	52	49	0.4851	Missense_Mutation	p.T840A	Non-Targeted Panel Gene	Adult HGBCL, NOS
BCL-9	chr15	59224756	59224756	T	A	exonic	MYO1E	62	60	0.4918	Missense_Mutation	p.Y237F	Non-Targeted Panel Gene	Adult HGBCL, NOS
BCL-9	chr4	168276160	168276160	C	T	exonic	DDX60	46	45	0.4945	Missense_Mutation	p.S667N	Non-Targeted Panel Gene	Adult HGBCL, NOS

BCL-9	chr14	35121959	35121959	T	C	exonic	PPP2R3C	145	142	0.4948	Missense_Mutation	p.M1V	Non-Targeted Panel Gene	Adult HGBCL, NOS
BCL-9	chr11	7042687	7042687	A	G	exonic	NLRP14	119	121	0.5042	Missense_Mutation	p.I221V	Non-Targeted Panel Gene	Adult HGBCL, NOS
BCL-9	chr19	7900879	7900879	C	T	exonic	LRRC8E	35	36	0.507	Missense_Mutation	p.P786L	Non-Targeted Panel Gene	Adult HGBCL, NOS
BCL-9	chr20	33083656	33083656	G	T	exonic	BPIFB4	55	58	0.5133	Missense_Mutation	p.Q153H	Non-Targeted Panel Gene	Adult HGBCL, NOS
BCL-9	chr16	68684685	68684685	G	A	exonic	CDH3	134	147	0.5231	Missense_Mutation	p.V429I	Non-Targeted Panel Gene	Adult HGBCL, NOS
BCL-9	chr16	31435997	31435997	C	T	exonic	ZNF843	35	40	0.5333	Missense_Mutation	p.A285T	Non-Targeted Panel Gene	Adult HGBCL, NOS
BCL-9	chr1	45621356	45621356	A	G	exonic	CCDC17	55	68	0.5528	Missense_Mutation	p.L429P	Non-Targeted Panel Gene	Adult HGBCL, NOS
BCL-9	chr5	180071524	180071524	C	T	exonic	RNF130	45	57	0.5588	Missense_Mutation	p.R60H	Non-Targeted Panel Gene	Adult HGBCL, NOS
BCL-9	chr16	23700670	23700670	G	T	exonic	ERN2	25	33	0.569	Missense_Mutation	p.T465N	Non-Targeted Panel Gene	Adult HGBCL, NOS
BCL-9	chr11	33700529	33700529	G	A	exonic	C11orf91	9	16	0.64	Missense_Mutation	p.A71V	Non-Targeted Panel Gene	Adult HGBCL, NOS
BCL-9	chrX	24211248	24211248	C	T	exonic	ZFX	87	449	0.8377	Missense_Mutation	p.R803W	Non-Targeted Panel Gene	Adult HGBCL, NOS
BCL-9	chrX	48917790	48917790	C	G	exonic	PIM2	20	111	0.8473	Missense_Mutation	p.W71C	Non-Targeted Panel Gene	Adult HGBCL, NOS
BCL-9	chr3	194398224	194398224	G	A	exonic	GP5	22	133	0.8581	Missense_Mutation	p.P20L	Non-Targeted Panel Gene	Adult HGBCL, NOS
BCL-9	chr6	31356273	31356273	C	T	exonic	HLA-B	115	47	0.2901	Nonsense_Mutation	p.W171X	Non-Targeted Panel Gene	Adult HGBCL, NOS
BCL-9	chr17	50079162	50079162	G	A	exonic	ITGA3	179	129	0.4188	Nonsense_Mutation	p.W829X	Non-Targeted Panel Gene	Adult HGBCL, NOS
BCL-9	chr1	179393957	179393957	C	A	exonic	AXDND1	173	139	0.4441	Nonsense_Mutation	p.Y306X	Non-Targeted Panel Gene	Adult HGBCL, NOS
BCL-9	chr9	32542480	32542480	G	T	exonic	TOPORS	242	211	0.4658	Nonsense_Mutation	p.S682X	Non-Targeted Panel Gene	Adult HGBCL, NOS
BCL-9	chr16	1602499	1602499	C	T	exonic	IFT140	94	88	0.4835	Nonsense_Mutation	p.W80X	Non-Targeted Panel Gene	Adult HGBCL, NOS
BCL-9	chrX	12976785	12976785	C	T	exonic	TMSB4X	36	310	0.896	Nonsense_Mutation	p.Q37X	Non-Targeted Panel Gene	Adult HGBCL, NOS
BCL-9	chr3	38141150	38141150	T	C	exonic	MYD88	164	101	0.3811	Nonstop_Mutation	p.L265P	Targerted Panel Gene	Adult HGBCL, NOS
BCL-90	chr3	187725578	187725578	G	T	exonic	BCL6	64	60	0.4839	Missense_Mutation	p.A587D	Targerted Panel Gene	Adult HGBCL, NOS
BCL-90	chr1	186087262	186087262	G	A	exonic	HMCN1	70	46	0.3966	Missense_Mutation	p.G3031D	Targerted Panel Gene	Adult HGBCL, NOS
BCL-90	chr8	127736597	127736597	G	C	exonic	MYC	112	90	0.4455	Missense_Mutation	p.D2H	Targerted Panel Gene	Adult HGBCL, NOS
BCL-90	chr1	228372823	228372823	C	T	exonic	OBSCN	48	22	0.3143	Missense_Mutation	p.P8306S	Targerted Panel Gene	Adult HGBCL, NOS
BCL-90	chr17	7675085	7675085	C	T	exonic	TP53	42	31	0.4247	Missense_Mutation	p.C137Y	Targerted Panel Gene	Adult HGBCL, NOS
BCL-90	chr1	215867100	215867100	C	T	exonic	USH2A	53	39	0.4239	Missense_Mutation	p.V2918M	Targerted Panel Gene	Adult HGBCL, NOS
BCL-90	chr8	127738435	127738435	C	T	exonic	MYC	57	58	0.5043	Missense_Mutation	p.T73I	Targerted Panel Gene	Adult HGBCL, NOS
BCL-35	chr2	151678189	151678189	T	C	splicing	NEB	234	145	0.3816	Splice_Site	.	Targerted Panel Gene	Pediatric non-nmBL
BCL-35	chr17	40389548	40389548	C	-	exonic	TOP2A	363	292	0.4458	Frame_Shift_Del	p.E1523fs	Targerted Panel Gene	Pediatric non-nmBL
BCL-35	chr5	112841945	112841945	-	GCT	exonic	APC	381	232	0.3785	In_Frame_Ins	p.Q2117delinsQA	Targerted Panel Gene	Pediatric non-nmBL
BCL-35	chr6	41935950	41935950	A	T	exonic	CCND3	208	145	0.4108	Missense_Mutation	p.I290K	Targerted Panel Gene	Pediatric non-nmBL
BCL-35	chr13	40666151	40666151	C	T	exonic	FOXO1	47	37	0.4405	Missense_Mutation	p.R21H	Targerted Panel Gene	Pediatric non-nmBL
BCL-35	chr1	228377108	228377108	C	G	exonic	OBSCN	71	65	0.4779	Missense_Mutation	p.A8656G	Targerted Panel Gene	Pediatric non-nmBL
BCL-35	chr19	11033486	11033486	A	C	exonic	SMARCA4	148	113	0.433	Missense_Mutation	p.Q1248P	Targerted Panel Gene	Pediatric non-nmBL
BCL-35	chr19	1612350	1612350	A	T	exonic	TCF3	165	136	0.4503	Missense_Mutation	p.V557E	Targerted Panel Gene	Pediatric non-nmBL
BCL-35	chr19	1612337	1612337	A	C	exonic	TCF3	152	139	0.4777	Missense_Mutation	p.D561E	Targerted Panel Gene	Pediatric non-nmBL
BCL-35	chr19	1612399	1612399	C	T	exonic	TCF3	157	113	0.4185	Missense_Mutation	p.D541N	Targerted Panel Gene	Pediatric non-nmBL
BCL-35	chr1	215628969	215628969	A	G	exonic	USH2A	219	214	0.492	Missense_Mutation	p.C5122R	Targerted Panel Gene	Pediatric non-nmBL
BCL-37	chr6	393096	393096	A	T	splicing	IRF4	62	19	0.2346	Splice_Site	.	Targerted Panel Gene	Pediatric non-nmBL
BCL-37	chr6	134174588	134174588	T	A	splicing	SGK1	220	78	0.2617	Splice_Site	.	Targerted Panel Gene	Pediatric non-nmBL
BCL-37	chr17	63929792	63929792	G	-	exonic	CD79B	161	105	0.3947	Frame_Shift_Del	p.P177fs	Targerted Panel Gene	Pediatric non-nmBL
BCL-37	chr3	50257024	50257026	AAG	-	exonic	GNAI2	208	108	0.3418	In_Frame_Del	p.255_255del	Targerted Panel Gene	Pediatric non-nmBL
BCL-37	chrX	77521407	77521407	G	A	exonic	ATRX	220	63	0.2226	Missense_Mutation	p.A2356V	Targerted Panel Gene	Pediatric non-nmBL
BCL-37	chr6	41935962	41935962	T	C	exonic	CCND3	175	135	0.4355	Missense_Mutation	p.D286G	Targerted Panel Gene	Pediatric non-nmBL
BCL-37	chr8	13100131	13100131	T	C	exonic	DLC1	205	117	0.3634	Missense_Mutation	p.N736D	Targerted Panel Gene	Pediatric non-nmBL
BCL-37	chr11	103245300	103245300	C	G	exonic	DYNC2H1	224	712	0.7607	Missense_Mutation	p.T3330S	Targerted Panel Gene	Pediatric non-nmBL
BCL-37	chr1	240092797	240092797	G	A	exonic	FMN2	78	70	0.473	Missense_Mutation	p.E230K	Targerted Panel Gene	Pediatric non-nmBL
BCL-37	chr22	22888210	22888210	G	A	exonic	IGLL5	96	86	0.4725	Missense_Mutation	p.G53R	Targerted Panel Gene	Pediatric non-nmBL
BCL-37	chr6	393298	393298	T	C	exonic	IRF4	200	76	0.2754	Missense_Mutation	p.I49T	Targerted Panel Gene	Pediatric non-nmBL
BCL-37	chr6	393260	393260	G	T	exonic	IRF4	217	76	0.2594	Missense_Mutation	p.K36N	Targerted Panel Gene	Pediatric non-nmBL
BCL-37	chr6	393332	393332	G	T	exonic	IRF4	162	55	0.2535	Missense_Mutation	p.Q60H	Targerted Panel Gene	Pediatric non-nmBL
BCL-37	chr1	75867578	75867578	A	G	exonic	MSH4	332	198	0.3736	Missense_Mutation	p.D432G	Targerted Panel Gene	Pediatric non-nmBL
BCL-37	chr1	228307430	228307430	C	A	exonic	OBSCN	214	136	0.3875	Missense_Mutation	p.A5079D	Targerted Panel Gene	Pediatric non-nmBL
BCL-37	chr17	7675088	7675088	C	T	exonic	TP53	67	121	0.6436	Missense_Mutation	p.R136H	Targerted Panel Gene	Pediatric non-nmBL
BCL-37	chr1	237793996	237793996	C	T	exonic	RYR2	277	161	0.3676	Nonsense_Mutation	p.Q4638X	Targerted Panel Gene	Pediatric non-nmBL
BCL-38	chr1	23559126	23559126	C	T	splicing	ID3	280	172	0.3797	Splice_Site	.	Targerted Panel Gene	Pediatric non-nmBL
BCL-38	chr7	140781617	140781617	C	A	exonic	BRAF	508	271	0.3479	Missense_Mutation	p.G464V	Targerted Panel Gene	Pediatric non-nmBL
BCL-38	chr13	40560535	40560535	G	A	exonic	FOXO1	703	301	0.2992	Missense_Mutation	p.S319L	Targerted Panel Gene	Pediatric non-nmBL
BCL-38	chr3	50257602	50257602	C	A	exonic	GNAI2	502	81	0.1389	Missense_Mutation	p.A311D	Targerted Panel Gene	Pediatric non-nmBL
BCL-38	chr1	23559197	23559197	A	G	exonic	ID3	302	200	0.3976	Missense_Mutation	p.I77T	Targerted Panel Gene	Pediatric non-nmBL
BCL-38	chr8	127736597	127736597	G	C	exonic	MYC	409	261	0.389	Missense_Mutation	p.D2H	Targerted Panel Gene	Pediatric non-nmBL
BCL-38	chr1	26762186	26762186	C	A	exonic	ARID1A	383	225	0.3701	Nonsense_Mutation	p.Y762X	Targerted Panel Gene	Pediatric non-nmBL
BCL-38	chrX	41345250	41345250	G	T	exonic	DDX3X	63	289	0.821	Nonsense_Mutation	p.E366X	Targerted Panel Gene	Pediatric non-nmBL
BCL-39	chr22	23181061	23181061	A	G	exonic	BCR	161	53	0.2477	Missense_Mutation	p.E34G	Targerted Panel Gene	Pediatric non-nmBL
BCL-39	chr6	41935972	41935972	T	C	exonic	CCND3	364	167	0.3145	Missense_Mutation	p.T283A	Targerted Panel Gene	Pediatric non-nmBL
BCL-39	chr6	393262	393262	A	T	exonic	IRF4	340	130	0.2766	Missense_Mutation	p.Y37F	Targerted Panel Gene	Pediatric non-nmBL
BCL-39	chr6	393251	393251	C	A	exonic	IRF4	335	123	0.2668	Missense_Mutation	p.D33E	Targerted Panel Gene	Pediatric non-nmBL
BCL-39	chr6	393204	393204	A	C	exonic	IRF4	316	95	0.2311	Missense_Mutation	p.S18R	Targerted Panel Gene	Pediatric non-nmBL
BCL-39	chr15	42693021	42693021	C	T	exonic	STARD9	475	450	0.4865	Missense_Mutation	p.P3815S	Targerted Panel Gene	Pediatric non-nmBL
BCL-40	chr6	27867429	27867429	G	C	exonic	HIST1H1B	126	106	0.453	Missense_Mutation	p.A34G	Targerted Panel Gene	Pediatric non-nmBL
BCL-40	chr1	232986410	232986410	G	T	exonic	PCNXL2	118	96	0.4486	Missense_Mutation	p.H1974Q	Targerted Panel Gene	Pediatric non-nmBL
BCL-40	chr15	41881988	41881988	C	T	exonic	SPTBN5	50	40	0.4444	Missense_Mutation	p.R802Q	Targerted Panel Gene	Pediatric non-nmBL
BCL-41	chr6	156778916	156778924	AGGAGCAGG	-	exonic	ARID1B	103	40	0.2797	In_Frame_Del	p.329_332del	Targerted Panel Gene	Pediatric non-nmBL
BCL-41	chr18	34818283	34818283	G	A	exonic	DTNA	434	382	0.4664	Missense_Mutation	p.G277S	Targerted Panel Gene	Pediatric non-nmBL
BCL-41	chr15	42718092	42718092	G	C	exonic	STARD9	333	322	0.4894	Missense_Mutation	p.V4559L	Targerted Panel Gene	Pediatric non-nmBL
BCL-41	chr1	15219486	15219486	G	T	exonic	TMEM51	714	152	0.1753	Nonsense_Mutation	p.E169X	Targerted Panel Gene	Pediatric non-nmBL
BCL-42	chr1	26731445	26731445	-	C	exonic	ARID1A	299	158	0.3457	Frame_Shift_Ins	p.Q548fs	Targerted Panel Gene	Pediatric non-nmBL
BCL-42	chrX	41345294	41345294	-	TT	exonic	DDX3X	49	207	0.7992	Frame_Shift_Ins	p.M380fs	Targerted Panel Gene	Pediatric non-nmBL
BCL-42	chr1	155479295	155479295	T	C	exonic	ASH1L	520	269	0.3409	Missense_Mutation	p.H1192R	Targerted Panel Gene	Pediatric non-nmBL
BCL-42	chr1	23559127	23559127	C	G	exonic	ID3	228	119	0.3429	Missense_Mutation	p.Q100H	Targerted Panel Gene	Pediatric non-nmBL
BCL-42	chr1	23559285	23559285	A	G	exonic	ID3	191	139	0.4212	Missense_Mutation	p.Y48H	Targerted Panel Gene	Pediatric non-nmBL
BCL-42	chr6	89696528	89696528	T	G	exonic	MDN1	388	47	0.108	Missense_Mutation	p.E3072A	Targerted Panel Gene	Pediatric non-nmBL

BCL-42	chr8	127736598	127736598	A	G	exonic	MYC	355	150	0.297	Missense_Mutation	p.D2G	Targerted Panel Gene	Pediatric non-nmBL
BCL-42	chr9	90877689	90877689	C	T	exonic	SYK	300	194	0.3911	Missense_Mutation	p.R434W	Targerted Panel Gene	Pediatric non-nmBL
BCL-43	chr1	228376016	228376016	T	C	exonic	OBSCN	90	99	0.5238	Missense_Mutation	p.W8556R	Targerted Panel Gene	Pediatric non-nmBL
BCL-43	chr1	23559129	23559129	G	A	exonic	ID3	226	33	0.1274	Nonsense_Mutation	p.Q100X	Targerted Panel Gene	Pediatric non-nmBL
mBL_11	chr1	23559126	23559126	C	T	splicing	ID3	264	204	0.4359	Splice_Site	.	Targerted Panel Gene	Adult mBL
mBL_11	chr3	50257024	50257026	AAG	-	exonic	GNAI2	340	213	0.3852	In_Frame_Del	p.255_255del	Targerted Panel Gene	Adult mBL
mBL_11	chr3	48581734	48581734	C	T	exonic	COL7A1	392	361	0.4794	Missense_Mutation	p.R1565K	Targerted Panel Gene	Adult mBL
mBL_11	chr13	40666142	40666142	G	A	exonic	FOXO1	66	35	0.3465	Missense_Mutation	p.T24I	Targerted Panel Gene	Adult mBL
mBL_11	chr22	22888178	22888178	T	G	exonic	IGLL5	183	138	0.4286	Missense_Mutation	p.W7G	Targerted Panel Gene	Adult mBL
mBL_11	chr8	127736612	127736612	G	T	exonic	MYC	297	155	0.3429	Missense_Mutation	p.V7L	Targerted Panel Gene	Adult mBL
mBL_11	chr13	35156164	35156164	C	A	exonic	NBEA	177	109	0.3811	Missense_Mutation	p.S870Y	Targerted Panel Gene	Adult mBL
mBL_11	chr2	51027770	51027770	C	G	exonic	NRXN1	207	197	0.4876	Missense_Mutation	p.K168N	Targerted Panel Gene	Adult mBL
mBL_11	chr6	128089929	128089929	T	C	exonic	PTPRK	283	210	0.4251	Missense_Mutation	p.D409G	Targerted Panel Gene	Adult mBL
mBL_11	chr1	237548447	237548447	G	A	exonic	RYR2	245	188	0.4342	Missense_Mutation	p.G975R	Targerted Panel Gene	Adult mBL
mBL_11	chr17	7673803	7673803	G	A	exonic	TP53	250	242	0.4919	Missense_Mutation	p.R234C	Targerted Panel Gene	Adult mBL
mBL_11	chr1	23559286	23559286	G	T	exonic	ID3	283	228	0.4462	Nonsense_Mutation	p.C47X	Targerted Panel Gene	Adult mBL
mBL_11	chr1	15219486	15219486	G	T	exonic	TMEM51	541	82	0.1316	Nonsense_Mutation	p.E169X	Targerted Panel Gene	Adult mBL
mBL_12	chr1	26779440	26779440	G	-	exonic	ARID1A	567	361	0.3878	Frame_Shift_Del	p.G1631fs	Targerted Panel Gene	Adult mBL
mBL_12	chr16	85921275	85921275	C	-	exonic	IRF8	586	390	0.3996	Frame_Shift_Del	p.T425fs	Targerted Panel Gene	Adult mBL
mBL_12	chr18	63318560	63318560	C	T	exonic	BCL2	324	226	0.4109	Missense_Mutation	p.G36D	Targerted Panel Gene	Adult mBL
mBL_12	chr18	63318569	63318569	C	G	exonic	BCL2	349	219	0.3856	Missense_Mutation	p.G33A	Targerted Panel Gene	Adult mBL
mBL_12	chrX	80696808	80696808	A	C	exonic	BRWD3	594	194	0.2462	Missense_Mutation	p.L1000R	Targerted Panel Gene	Adult mBL
mBL_12	chr6	41941465	41941465	T	G	exonic	CCND3	277	180	0.3939	Missense_Mutation	p.Y62S	Targerted Panel Gene	Adult mBL
mBL_12	chr4	125446303	125446303	A	T	exonic	FAT4	156	292	0.6518	Missense_Mutation	p.I2402F	Targerted Panel Gene	Adult mBL
mBL_12	chr12	49046167	49046167	G	A	exonic	KMT2D	442	281	0.3887	Missense_Mutation	p.H1531Y	Targerted Panel Gene	Adult mBL
mBL_12	chr6	134174031	134174031	G	A	exonic	SGK1	399	257	0.3918	Missense_Mutation	p.L163F	Targerted Panel Gene	Adult mBL
mBL_12	chr17	7674259	7674259	T	A	exonic	TP53	78	208	0.7273	Missense_Mutation	p.N196I	Targerted Panel Gene	Adult mBL
mBL_12	chr17	7674256	7674256	T	C	exonic	TP53	80	216	0.7297	Missense_Mutation	p.Y197C	Targerted Panel Gene	Adult mBL
mBL_12	chr19	22088126	22088126	T	C	exonic	ZNF257	55	10	0.1538	Missense_Mutation	p.C50R	Targerted Panel Gene	Adult mBL
mBL_12	chr19	22088127	22088127	G	A	exonic	ZNF257	55	10	0.1538	Missense_Mutation	p.C50Y	Targerted Panel Gene	Adult mBL
mBL_12	chrX	40073744	40073744	C	T	exonic	BCOR	966	349	0.2654	Nonsense_Mutation	p.W534X	Targerted Panel Gene	Adult mBL
mBL_12	chr12	49022742	49022742	T	A	exonic	KMT2D	634	405	0.3898	Nonsense_Mutation	p.K5396X	Targerted Panel Gene	Adult mBL
mBL_13	chr13	48463797	48463800	GTAA	-	exonic	RB1	34	80	0.7018	Frame_Shift_Del	p.V725fs	Targerted Panel Gene	Adult mBL
mBL_13	chr12	49051555	49051555	-	T	exonic	KMT2D	310	203	0.3957	Frame_Shift_Ins	p.P710fs	Targerted Panel Gene	Adult mBL
mBL_13	chr18	63318060	63318060	T	A	exonic	BCL2	700	262	0.2723	Missense_Mutation	p.S203C	Targerted Panel Gene	Adult mBL
mBL_13	chr18	63318380	63318380	G	A	exonic	BCL2	388	132	0.2538	Missense_Mutation	p.T96I	Targerted Panel Gene	Adult mBL
mBL_13	chr12	122022093	122022093	T	A	exonic	BCL7A	198	275	0.5814	Missense_Mutation	p.M1K	Targerted Panel Gene	Adult mBL
mBL_13	chr22	23181166	23181166	G	C	exonic	BCR	127	75	0.3659	Missense_Mutation	p.S69T	Targerted Panel Gene	Adult mBL
mBL_13	chr22	23181963	23181963	A	G	exonic	BCR	328	227	0.4083	Missense_Mutation	p.N335D	Targerted Panel Gene	Adult mBL
mBL_13	chr22	23181817	23181817	G	T	exonic	BCR	297	199	0.4004	Missense_Mutation	p.G286V	Targerted Panel Gene	Adult mBL
mBL_13	chr22	23180997	23180997	G	A	exonic	BCR	94	62	0.3974	Missense_Mutation	p.A13T	Targerted Panel Gene	Adult mBL
mBL_13	chr22	23181608	23181608	G	C	exonic	BCR	266	170	0.3881	Missense_Mutation	p.Q216H	Targerted Panel Gene	Adult mBL
mBL_13	chr22	23181163	23181163	A	G	exonic	BCR	130	75	0.3659	Missense_Mutation	p.K68R	Targerted Panel Gene	Adult mBL
mBL_13	chr12	92145408	92145408	C	T	exonic	BTG1	221	86	0.2801	Missense_Mutation	p.S43N	Targerted Panel Gene	Adult mBL
mBL_13	chr8	3408162	3408162	T	C	exonic	CSMD1	355	218	0.3805	Missense_Mutation	p.E603G	Targerted Panel Gene	Adult mBL
mBL_13	chr9	87706876	87706876	A	G	exonic	DAPK1	292	213	0.4218	Missense_Mutation	p.T1269A	Targerted Panel Gene	Adult mBL
mBL_13	chr4	186611449	186611449	C	T	exonic	FAT1	328	200	0.3788	Missense_Mutation	p.E3264K	Targerted Panel Gene	Adult mBL
mBL_13	chr4	186618670	186618670	G	T	exonic	FAT1	403	232	0.3654	Missense_Mutation	p.S2639Y	Targerted Panel Gene	Adult mBL
mBL_13	chr13	40666148	40666148	G	C	exonic	FOXO1	69	74	0.5175	Missense_Mutation	p.S22W	Targerted Panel Gene	Adult mBL
mBL_13	chr11	119095144	119095144	A	T	exonic	H2AFX	53	23	0.3026	Missense_Mutation	p.L84Q	Targerted Panel Gene	Adult mBL
mBL_13	chr6	26199668	26199668	G	C	exonic	HIST1H2BF	94	62	0.3974	Missense_Mutation	p.S37T	Targerted Panel Gene	Adult mBL
mBL_13	chr6	27146689	27146689	C	G	exonic	HIST1H2BK	366	156	0.2983	Missense_Mutation	p.S37T	Targerted Panel Gene	Adult mBL
mBL_13	chr1	21887639	21887639	G	C	exonic	HSPG2	278	191	0.4072	Missense_Mutation	p.L247V	Targerted Panel Gene	Adult mBL
mBL_13	chr16	85903082	85903082	T	C	exonic	IRF8	375	289	0.4352	Missense_Mutation	p.Y23H	Targerted Panel Gene	Adult mBL
mBL_13	chr9	13168486	13168486	T	C	exonic	MPDZ	291	213	0.4226	Missense_Mutation	p.D1045G	Targerted Panel Gene	Adult mBL
mBL_13	chr16	11255147	11255147	C	T	exonic	SOCS1	163	117	0.4179	Missense_Mutation	p.C111Y	Targerted Panel Gene	Adult mBL
mBL_13	chr19	1612367	1612367	A	T	exonic	TCF3	216	176	0.4467	Missense_Mutation	p.N551K	Targerted Panel Gene	Adult mBL
mBL_13	chr7	98955246	98955246	A	C	exonic	TRRAP	202	136	0.4024	Missense_Mutation	p.E1953A	Targerted Panel Gene	Adult mBL
mBL_13	chr8	102311422	102311422	C	A	exonic	UBR5	354	231	0.3949	Missense_Mutation	p.R831S	Targerted Panel Gene	Adult mBL
mBL_13	chr1	215900807	215900807	G	A	exonic	USH2A	494	166	0.2515	Missense_Mutation	p.P2467S	Targerted Panel Gene	Adult mBL
mBL_13	chr15	44711548	44711548	T	G	exonic	B2M	69	171	0.7125	Missense_Mutation	p.M1R	Targerted Panel Gene	Adult mBL
mBL_13	chr6	27892837	27892837	G	A	exonic	HIST1H2AM	130	77	0.372	Nonsense_Mutation	p.Q105X	Targerted Panel Gene	Adult mBL
mBL_13	chr16	11255287	11255287	G	T	exonic	SOCS1	134	86	0.3891	Nonsense_Mutation	p.Y64X	Targerted Panel Gene	Adult mBL
mBL_15	chr12	132062590	132062590	-	CAA	exonic	EP400	199	60	0.2239	In_Frame_Ins	p.Q2741delinsQQ	Targerted Panel Gene	Adult mBL
mBL_15	chr6	157206704	157206704	A	G	exonic	ARID1B	535	442	0.4524	Missense_Mutation	p.R1855G	Targerted Panel Gene	Adult mBL
mBL_15	chr1	155354561	155354561	T	G	exonic	ASH1L	473	228	0.3252	Missense_Mutation	p.E2375D	Targerted Panel Gene	Adult mBL
mBL_15	chrX	41346593	41346593	T	C	exonic	DDX3X	87	200	0.6969	Missense_Mutation	p.I529T	Targerted Panel Gene	Adult mBL
mBL_15	chr22	41176791	41176791	A	G	exonic	EP300	525	208	0.2834	Missense_Mutation	p.T1694A	Targerted Panel Gene	Adult mBL
mBL_15	chr17	65056513	65056513	C	G	exonic	GNA13	310	159	0.339	Missense_Mutation	p.Q27H	Targerted Panel Gene	Adult mBL
mBL_15	chr17	65056486	65056486	T	G	exonic	GNA13	351	183	0.3427	Missense_Mutation	p.K36N	Targerted Panel Gene	Adult mBL
mBL_15	chr22	22888214	22888214	C	A	exonic	IGLL5	225	120	0.3478	Missense_Mutation	p.P19T	Targerted Panel Gene	Adult mBL
mBL_15	chr22	22888073	22888073	A	T	exonic	IGLL5	365	173	0.3216	Missense_Mutation	p.Q7L	Targerted Panel Gene	Adult mBL
mBL_15	chr22	22888119	22888119	G	C	exonic	IGLL5	312	146	0.3188	Missense_Mutation	p.Q22H	Targerted Panel Gene	Adult mBL
mBL_15	chr22	22888179	22888179	G	T	exonic	IGLL5	279	113	0.2883	Missense_Mutation	p.W7L	Targerted Panel Gene	Adult mBL
mBL_15	chr8	127738287	127738287	A	T	exonic	MYC	577	219	0.2744	Missense_Mutation	p.N24Y	Targerted Panel Gene	Adult mBL
mBL_15	chr8	127738707	127738707	C	G	exonic	MYC	418	224	0.3489	Missense_Mutation	p.L164V	Targerted Panel Gene	Adult mBL
mBL_15	chr8	127738786	127738786	G	A	exonic	MYC	390	172	0.306	Missense_Mutation	p.S190N	Targerted Panel Gene	Adult mBL
mBL_15	chr8	127738379	127738379	G	C	exonic	MYC	493	138	0.2187	Missense_Mutation	p.E54D	Targerted Panel Gene	Adult mBL
mBL_15	chr8	127738768	127738768	G	C	exonic	MYC	395	177	0.3094	Missense_Mutation	p.S184T	Targerted Panel Gene	Adult mBL
mBL_15	chr8	127738610	127738610	C	G	exonic	MYC	527	225	0.2992	Missense_Mutation	p.I131M	Targerted Panel Gene	Adult mBL
mBL_15	chr8	127738799	127738799	G	C	exonic	MYC	416	170	0.2901	Missense_Mutation	p.Q194H	Targerted Panel Gene	Adult mBL
mBL_15	chr8	127738266	127738266	C	G	exonic	MYC	601	238	0.283	Missense_Mutation	p.P17A	Targerted Panel Gene	Adult mBL

mBL_15	chr8	127738556	127738556	G	C	exonic	MYC	498	185	0.2705	Missense_Mutation	p.Q113H	Targerted Panel Gene	Adult mBL
mBL_15	chr8	127738367	127738367	G	C	exonic	MYC	502	157	0.2379	Missense_Mutation	p.Q50H	Targerted Panel Gene	Adult mBL
mBL_15	chr8	127738364	127738364	G	C	exonic	MYC	506	154	0.2312	Missense_Mutation	p.Q49H	Targerted Panel Gene	Adult mBL
mBL_15	chr8	127738482	127738482	T	A	exonic	MYC	425	117	0.2159	Missense_Mutation	p.Y89N	Targerted Panel Gene	Adult mBL
mBL_15	chr7	82955880	82955880	C	A	exonic	PCLO	460	157	0.2545	Missense_Mutation	p.L1691F	Targerted Panel Gene	Adult mBL
mBL_15	chr18	55229007	55229007	G	C	exonic	TCF4	602	230	0.2764	Missense_Mutation	p.N675K	Targerted Panel Gene	Adult mBL
mBL_15	chr7	82956125	82956125	G	A	exonic	PCLO	677	302	0.3075	Nonsense_Mutation	p.R1610X	Targerted Panel Gene	Adult mBL
mBL_15	chr1	15219486	15219486	G	T	exonic	TMEM51	755	72	0.0869	Nonsense_Mutation	p.E169X	Targerted Panel Gene	Adult mBL
mBL_16	chr16	3736766	3736766	A	T	exonic	CREBBP	188	512	0.7314	Missense_Mutation	p.Y1482N	Targerted Panel Gene	Adult mBL
mBL_16	chr1	33605440	33605440	T	C	exonic	CSMD2	606	122	0.1676	Missense_Mutation	p.E2127G	Targerted Panel Gene	Adult mBL
mBL_16	chr8	113278679	113278679	G	T	exonic	CSMD3	451	430	0.4881	Missense_Mutation	p.P103T	Targerted Panel Gene	Adult mBL
mBL_16	chr13	72772725	72772725	G	C	exonic	DIS3	944	149	0.1363	Missense_Mutation	p.L452V	Targerted Panel Gene	Adult mBL
mBL_16	chr6	56640213	56640213	T	G	exonic	DST	540	485	0.4727	Missense_Mutation	p.Q774P	Targerted Panel Gene	Adult mBL
mBL_16	chr6	56616285	56616285	A	C	exonic	DST	798	257	0.2436	Missense_Mutation	p.D2394E	Targerted Panel Gene	Adult mBL
mBL_16	chr6	26234933	26234933	T	G	exonic	HIST1H1D	145	498	0.7745	Missense_Mutation	p.M1L	Targerted Panel Gene	Adult mBL
mBL_16	chr13	110719733	110719733	T	C	exonic	ING1	31	58	0.6517	Missense_Mutation	p.L214P	Targerted Panel Gene	Adult mBL
mBL_16	chr8	127738291	127738291	G	T	exonic	MYC	1129	258	0.186	Missense_Mutation	p.R25M	Targerted Panel Gene	Adult mBL
mBL_16	chr4	133151504	133151504	A	T	exonic	PCDH10	352	180	0.3383	Missense_Mutation	p.D455V	Targerted Panel Gene	Adult mBL
mBL_16	chr6	52053181	52053181	C	A	exonic	PKHD1	186	445	0.7052	Missense_Mutation	p.A679S	Targerted Panel Gene	Adult mBL
mBL_16	chr12	70587206	70587206	C	T	exonic	PTPRB	432	237	0.3543	Missense_Mutation	p.M704I	Targerted Panel Gene	Adult mBL
mBL_16	chr12	57102878	57102878	T	C	exonic	STAT6	330	175	0.3465	Missense_Mutation	p.D419G	Targerted Panel Gene	Adult mBL
mBL_16	chr1	2556667	2556667	G	A	exonic	TNFRSF14	103	318	0.7553	Missense_Mutation	p.M1I	Targerted Panel Gene	Adult mBL
mBL_16	chr17	7675076	7675076	T	C	exonic	TP53	89	294	0.7676	Missense_Mutation	p.H140R	Targerted Panel Gene	Adult mBL
mBL_16	chr11	128463613	128463613	G	A	exonic	ETS1	586	213	0.2656	Nonsense_Mutation	p.Q380X	Targerted Panel Gene	Adult mBL
mBL_2	chr18	63318953	63318953	C	G	splicing	BCL2	359	210	0.3691	Splice_Site	.	Targerted Panel Gene	Adult mBL
mBL_2	chr1	23559376	23559379	CAGG	-	exonic	ID3	366	297	0.448	Frame_Shift_Del	p.C16fs	Targerted Panel Gene	Adult mBL
mBL_2	chr22	22888214	22888214	-	CGGGAGT	exonic	IGLL5	230	122	0.3417	Frame_Shift_Ins	p.P19fs	Targerted Panel Gene	Adult mBL
mBL_2	chr18	63318504	63318509	GCCCGG	-	exonic	BCL2	218	172	0.441	In_Frame_Del	p.53_55del	Targerted Panel Gene	Adult mBL
mBL_2	chr1	26696649	26696649	-	GGC	exonic	ARID1A	133	80	0.367	In_Frame_Ins	p.G82delinsGG	Targerted Panel Gene	Adult mBL
mBL_2	chr18	63318470	63318470	A	G	exonic	BCL2	192	195	0.5039	Missense_Mutation	p.V66A	Targerted Panel Gene	Adult mBL
mBL_2	chr18	63318500	63318500	G	A	exonic	BCL2	201	184	0.4779	Missense_Mutation	p.T56M	Targerted Panel Gene	Adult mBL
mBL_2	chr18	63318645	63318645	C	T	exonic	BCL2	406	266	0.3958	Missense_Mutation	p.G8R	Targerted Panel Gene	Adult mBL
mBL_2	chr7	2944513	2944513	G	A	exonic	CARD11	224	179	0.4442	Missense_Mutation	p.T128M	Targerted Panel Gene	Adult mBL
mBL_2	chr1	149886381	149886381	C	T	exonic	HIST2H2BE	112	117	0.5087	Missense_Mutation	p.R87H	Targerted Panel Gene	Adult mBL
mBL_2	chr22	22888117	22888117	C	G	exonic	IGLL5	261	243	0.4793	Missense_Mutation	p.Q22E	Targerted Panel Gene	Adult mBL
mBL_2	chr22	22888241	22888241	T	C	exonic	IGLL5	146	128	0.4655	Missense_Mutation	p.C28R	Targerted Panel Gene	Adult mBL
mBL_2	chr22	22888238	22888238	G	A	exonic	IGLL5	153	130	0.4594	Missense_Mutation	p.A27T	Targerted Panel Gene	Adult mBL
mBL_2	chr13	110719761	110719761	G	T	exonic	ING1	116	77	0.399	Missense_Mutation	p.M223I	Targerted Panel Gene	Adult mBL
mBL_2	chr10	87933145	87933145	G	A	exonic	PTEN	30	332	0.9171	Missense_Mutation	p.G302E	Targerted Panel Gene	Adult mBL
mBL_2	chr15	41876925	41876925	G	T	exonic	SPTBN5	111	104	0.4837	Missense_Mutation	p.S1245R	Targerted Panel Gene	Adult mBL
mBL_2	chr9	90887782	90887782	T	C	exonic	SYK	389	315	0.4474	Missense_Mutation	p.Y539H	Targerted Panel Gene	Adult mBL
mBL_2	chr3	183555413	183555413	G	A	exonic	KLHL6	400	334	0.4526	Nonsense_Mutation	p.Q81X	Targerted Panel Gene	Adult mBL
mBL_39	chr17	7687376	7687376	C	T	splicing	TP53	15	330	0.9565	Splice_Site	.	Targerted Panel Gene	Adult mBL
mBL_39	chr3	177046136	177046138	AAA	-	exonic	TBL1XR1	121	340	0.7375	In_Frame_Del	p.306_306del	Targerted Panel Gene	Adult mBL
mBL_39	chr12	132062548	132062548	-	CAGCAGCAGCAG	exonic	EP400	208	36	0.1401	In_Frame_Ins	p.Q2727delinsQQ[illegible]	Targerted Panel Gene	Adult mBL
mBL_39	chr2	21035624	21035624	T	A	exonic	APOB	290	254	0.4669	Missense_Mutation	p.I260F	Targerted Panel Gene	Adult mBL
mBL_39	chr1	26696728	26696728	C	T	exonic	ARID1A	148	129	0.4657	Missense_Mutation	p.P109S	Targerted Panel Gene	Adult mBL
mBL_39	chr12	85056785	85056785	G	A	exonic	LRRIQ1	282	229	0.4481	Missense_Mutation	p.M664I	Targerted Panel Gene	Adult mBL
mBL_39	chr9	74887238	74887238	G	T	exonic	TRPM6	117	104	0.4706	Missense_Mutation	p.P4Q	Targerted Panel Gene	Adult mBL
mBL_39	chr3	38141150	38141150	T	C	exonic	MYD88	25	294	0.9216	Nonstop_Mutation	p.L265P	Targerted Panel Gene	Adult mBL
mBL_41	chr1	23559225	23559228	CCAC	-	exonic	ID3	356	310	0.4655	Frame_Shift_Del	p.V67fs	Targerted Panel Gene	Adult mBL
mBL_41	chr6	41936007	41936007	-	G	exonic	CCND3	370	275	0.4237	Frame_Shift_Ins	p.R271fs	Targerted Panel Gene	Adult mBL
mBL_41	chr13	40666137	40666137	-	CCAGGTGCA	exonic	FOXO1	90	42	0.3182	In_Frame_Ins	p.P26delinsCTWP	Targerted Panel Gene	Adult mBL
mBL_41	chr8	112587142	112587142	C	A	exonic	CSMD3	275	179	0.3943	Missense_Mutation	p.S1230I	Targerted Panel Gene	Adult mBL
mBL_41	chr13	40666212	40666212	T	C	exonic	FOXO1	113	87	0.435	Missense_Mutation	p.M1V	Targerted Panel Gene	Adult mBL
mBL_41	chr6	27810416	27810416	G	A	exonic	HIST1H3H	74	62	0.4559	Missense_Mutation	p.A115T	Targerted Panel Gene	Adult mBL
mBL_41	chr1	23559197	23559197	A	C	exonic	ID3	377	285	0.4305	Missense_Mutation	p.I77S	Targerted Panel Gene	Adult mBL
mBL_41	chr8	127738674	127738674	T	G	exonic	MYC	354	365	0.5069	Missense_Mutation	p.F153V	Targerted Panel Gene	Adult mBL
mBL_41	chr8	127736612	127736612	G	C	exonic	MYC	371	294	0.4421	Missense_Mutation	p.V7L	Targerted Panel Gene	Adult mBL
mBL_41	chr10	87960912	87960912	T	G	exonic	PTEN	69	65	0.4851	Missense_Mutation	p.W447G	Targerted Panel Gene	Adult mBL
mBL_41	chr1	178445642	178445642	A	G	exonic	RASAL2	355	267	0.4293	Missense_Mutation	p.Q536R	Targerted Panel Gene	Adult mBL
mBL_41	chr17	7675136	7675136	G	A	exonic	TP53	363	346	0.488	Missense_Mutation	p.A120V	Targerted Panel Gene	Adult mBL
mBL_41	chr19	22088126	22088126	T	C	exonic	ZNF257	54	7	0.1148	Missense_Mutation	p.C50R	Targerted Panel Gene	Adult mBL
mBL_41	chr19	22088127	22088127	G	A	exonic	ZNF257	52	8	0.1333	Missense_Mutation	p.C50Y	Targerted Panel Gene	Adult mBL
mBL_41	chr19	22088132	22088132	G	C	exonic	ZNF257	52	8	0.1333	Missense_Mutation	p.G52R	Targerted Panel Gene	Adult mBL
mBL_41	chr19	22088133	22088133	G	A	exonic	ZNF257	54	8	0.129	Missense_Mutation	p.G52E	Targerted Panel Gene	Adult mBL
mBL_42	chr17	7674281	7674301	CAGAGCCAACCTA[illegible]	-	exonic	TP53	64	151	0.7023	Frame_Shift_Del	p.V186fs	Targerted Panel Gene	Adult mBL
mBL_42	chr13	48456342	48456342	-	A	exonic	RB1	51	235	0.8217	Frame_Shift_Ins	p.Y651fs	Targerted Panel Gene	Adult mBL
mBL_42	chr16	3731323	3731325	AGG	-	exonic	CREBBP	100	508	0.8355	In_Frame_Del	p.1680_1681del	Targerted Panel Gene	Adult mBL
mBL_42	chr18	63318485	63318485	G	A	exonic	BCL2	240	241	0.501	Missense_Mutation	p.A61V	Targerted Panel Gene	Adult mBL
mBL_42	chr12	122022118	122022118	G	C	exonic	BCL7A	269	229	0.4598	Missense_Mutation	p.E9D	Targerted Panel Gene	Adult mBL
mBL_42	chr12	122022092	122022092	A	T	exonic	BCL7A	240	199	0.4533	Missense_Mutation	p.M1L	Targerted Panel Gene	Adult mBL
mBL_42	chr8	143871968	143871968	T	C	exonic	EPPK1	10	202	0.9528	Missense_Mutation	p.Q429R	Targerted Panel Gene	Adult mBL
mBL_42	chr3	46995737	46995737	G	A	exonic	NBEAL2	540	469	0.4639	Missense_Mutation	p.G641E	Targerted Panel Gene	Adult mBL
mBL_42	chr12	57102878	57102878	T	C	exonic	STAT6	273	228	0.4551	Missense_Mutation	p.D419G	Targerted Panel Gene	Adult mBL
mBL_42	chr14	23533986	23533986	C	T	exonic	ZFHX2	48	43	0.4725	Missense_Mutation	p.R447H	Targerted Panel Gene	Adult mBL
mBL_42	chr19	22088126	22088126	T	C	exonic	ZNF257	63	8	0.1127	Missense_Mutation	p.C50R	Targerted Panel Gene	Adult mBL
mBL_42	chr19	22088127	22088127	G	A	exonic	ZNF257	60	9	0.1304	Missense_Mutation	p.C50Y	Targerted Panel Gene	Adult mBL
mBL_42	chr19	22088132	22088132	G	C	exonic	ZNF257	55	9	0.1406	Missense_Mutation	p.G52R	Targerted Panel Gene	Adult mBL
mBL_42	chr19	22088133	22088133	G	A	exonic	ZNF257	55	9	0.1406	Missense_Mutation	p.G52E	Targerted Panel Gene	Adult mBL
mBL_42	chr1	2560713	2560713	A	T	exonic	TNFRSF14	10	376	0.9741	Nonsense_Mutation	p.K184X	Targerted Panel Gene	Adult mBL
mBL_43	chr6	41935968	41935968	G	A	exonic	CCND3	58	692	0.9214	Missense_Mutation	p.P284L	Targerted Panel Gene	Adult mBL

mBL_43	chr1	26762304	26762304	C	T	exonic	ARID1A	349	276	0.4416	Nonsense_Mutation	p.Q802X	Targerted Panel Gene	Adult mBL
mBL_43	chr1	23559186	23559186	G	A	exonic	ID3	69	610	0.8984	Nonsense_Mutation	p.Q81X	Targerted Panel Gene	Adult mBL
mBL_44	chr15	93024543	93024543	G	T	exonic	CHD2	467	443	0.4863	Missense_Mutation	p.Q1775H	Targerted Panel Gene	Adult mBL
mBL_44	chr6	27146610	27146610	C	G	exonic	HIST1H2BK	264	122	0.3161	Missense_Mutation	p.M63I	Targerted Panel Gene	Adult mBL
mBL_44	chr22	22888169	22888169	T	C	exonic	IGLL5	278	143	0.3397	Missense_Mutation	p.C4R	Targerted Panel Gene	Adult mBL
mBL_44	chr7	103566374	103566374	T	G	exonic	RELN	154	136	0.469	Missense_Mutation	p.I1596L	Targerted Panel Gene	Adult mBL
mBL_44	chr3	49368513	49368513	T	G	exonic	RHOA	533	221	0.2931	Missense_Mutation	p.E64D	Targerted Panel Gene	Adult mBL
mBL_5	chr1	85270618	85270618	-	T	exonic	BCL10	218	159	0.4195	Frame_Shift_Ins	p.G116fs	Targerted Panel Gene	Adult mBL
mBL_5	chr22	22888233	22888233	A	G	exonic	IGLL5	137	67	0.3284	Missense_Mutation	p.D25G	Targerted Panel Gene	Adult mBL
mBL_5	chr22	22888139	22888139	T	G	exonic	IGLL5	189	153	0.4474	Missense_Mutation	p.L29R	Targerted Panel Gene	Adult mBL
mBL_5	chr22	22888100	22888100	T	C	exonic	IGLL5	241	175	0.4207	Missense_Mutation	p.L16P	Targerted Panel Gene	Adult mBL
mBL_5	chr10	94246248	94246248	T	G	exonic	PLCE1	436	347	0.4432	Missense_Mutation	p.L908R	Targerted Panel Gene	Adult mBL
mBL_5	chr3	78657233	78657233	T	A	exonic	ROBO1	366	240	0.396	Missense_Mutation	p.N827Y	Targerted Panel Gene	Adult mBL
mBL_5	chr4	105259708	105259708	G	A	exonic	TET2	104	438	0.8081	Missense_Mutation	p.C1298Y	Targerted Panel Gene	Adult mBL
mBL_58	chr16	3774567	3774567	A	T	splicing	CREBBP	274	104	0.2751	Splice_Site	.	Targerted Panel Gene	Adult mBL
mBL_58	chr3	50252121	50252121	G	A	exonic	GNAI2	205	158	0.4353	Missense_Mutation	p.S31N	Targerted Panel Gene	Adult mBL
mBL_58	chr1	23559204	23559204	C	A	exonic	ID3	409	52	0.1128	Missense_Mutation	p.D75Y	Targerted Panel Gene	Adult mBL
mBL_58	chr2	197400849	197400849	C	T	exonic	SF3B1	380	288	0.4286	Missense_Mutation	p.E862K	Targerted Panel Gene	Adult mBL
mBL_58	chr19	1612338	1612338	T	A	exonic	TCF3	62	342	0.8403	Missense_Mutation	p.D561V	Targerted Panel Gene	Adult mBL
mBL_58	chr17	7674885	7674885	C	T	exonic	TP53	402	163	0.288	Missense_Mutation	p.V177M	Targerted Panel Gene	Adult mBL
mBL_59	chr1	23559126	23559126	C	T	splicing	ID3	340	245	0.4188	Splice_Site	.	Targerted Panel Gene	Adult mBL
mBL_59	chr5	71489485	71489485	A	G	exonic	BDP1	357	311	0.4656	Missense_Mutation	p.Q432R	Targerted Panel Gene	Adult mBL
mBL_59	chr10	16876917	16876917	T	G	exonic	CUBN	519	78	0.1307	Missense_Mutation	p.K3029T	Targerted Panel Gene	Adult mBL
mBL_59	chr11	103245331	103245331	A	T	exonic	DYNC2H1	231	198	0.4615	Missense_Mutation	p.E3340D	Targerted Panel Gene	Adult mBL
mBL_59	chr1	186128208	186128208	G	C	exonic	HMCN1	340	345	0.5022	Missense_Mutation	p.G4274A	Targerted Panel Gene	Adult mBL
mBL_59	chr11	118474298	118474298	G	A	exonic	KMT2A	422	379	0.4732	Missense_Mutation	p.D1047N	Targerted Panel Gene	Adult mBL
mBL_59	chr6	89756351	89756351	T	G	exonic	MDN1	273	230	0.4573	Missense_Mutation	p.E914D	Targerted Panel Gene	Adult mBL
mBL_59	chr3	177047542	177047542	C	T	exonic	TBL1XR1	371	47	0.1124	Missense_Mutation	p.G237D	Targerted Panel Gene	Adult mBL
mBL_59	chrX	41344305	41344305	C	T	exonic	DDX3X	34	339	0.9088	Nonsense_Mutation	p.R311X	Targerted Panel Gene	Adult mBL
mBL_59	chr1	23559186	23559186	G	A	exonic	ID3	318	299	0.4838	Nonsense_Mutation	p.Q81X	Targerted Panel Gene	Adult mBL
mBL_60	chrX	141906190	141906190	T	-	exonic	MAGEC1	199	191	0.4897	Frame_Shift_Del	p.G262fs	Targerted Panel Gene	Adult mBL
mBL_60	chr18	63318333	63318333	A	G	exonic	BCL2	488	422	0.4637	Missense_Mutation	p.F112L	Targerted Panel Gene	Adult mBL
mBL_60	chr5	168244615	168244615	C	T	exonic	TENM2	248	230	0.4812	Missense_Mutation	p.R1897C	Targerted Panel Gene	Adult mBL
mBL_60	chr17	7674872	7674872	T	C	exonic	TP53	28	284	0.9103	Missense_Mutation	p.Y181C	Targerted Panel Gene	Adult mBL
mBL_60	chr12	49026536	49026536	G	A	exonic	KMT2D	377	298	0.4408	Nonsense_Mutation	p.Q5144X	Targerted Panel Gene	Adult mBL
mBL_61	chr22	41160723	41160723	G	A	splicing	EP300	288	213	0.4251	Splice_Site	.	Targerted Panel Gene	Adult mBL
mBL_61	chr12	49041915	49041915	A	G	splicing	KMT2D	200	152	0.4318	Splice_Site	.	Targerted Panel Gene	Adult mBL
mBL_61	chr18	63318210	63318210	A	G	exonic	BCL2	348	259	0.4267	Missense_Mutation	p.F153L	Targerted Panel Gene	Adult mBL
mBL_61	chr19	41877338	41877338	C	T	exonic	CD79A	287	203	0.4143	Missense_Mutation	p.P12S	Targerted Panel Gene	Adult mBL
mBL_61	chr3	48593580	48593580	T	A	exonic	COL7A1	295	204	0.4088	Missense_Mutation	p.D128V	Targerted Panel Gene	Adult mBL
mBL_61	chr19	10802345	10802345	A	G	exonic	DNM2	266	177	0.3995	Missense_Mutation	p.I494V	Targerted Panel Gene	Adult mBL
mBL_61	chr8	25332307	25332307	C	G	exonic	DOCK5	620	343	0.3554	Missense_Mutation	p.L654V	Targerted Panel Gene	Adult mBL
mBL_61	chr6	56470180	56470180	G	C	exonic	DST	371	262	0.4132	Missense_Mutation	p.P5356R	Targerted Panel Gene	Adult mBL
mBL_61	chr7	148811635	148811635	T	A	exonic	EZH2	450	195	0.3019	Missense_Mutation	p.Y646F	Targerted Panel Gene	Adult mBL
mBL_61	chr6	26156683	26156683	G	A	exonic	HIST1H1E	361	265	0.4233	Missense_Mutation	p.G98D	Targerted Panel Gene	Adult mBL
mBL_61	chr6	26123549	26123549	A	G	exonic	HIST1H2BC	270	178	0.3973	Missense_Mutation	p.V119A	Targerted Panel Gene	Adult mBL
mBL_61	chr1	21841102	21841102	T	G	exonic	HSPG2	128	78	0.3786	Missense_Mutation	p.Q3171P	Targerted Panel Gene	Adult mBL
mBL_61	chrX	53547777	53547777	A	G	exonic	HUWE1	32	284	0.8959	Missense_Mutation	p.V3511A	Targerted Panel Gene	Adult mBL
mBL_61	chr22	22888159	22888159	C	T	exonic	IGLL5	168	125	0.4266	Missense_Mutation	p.H36Y	Targerted Panel Gene	Adult mBL
mBL_61	chr22	22888221	22888221	T	G	exonic	IGLL5	147	126	0.4615	Missense_Mutation	p.L21W	Targerted Panel Gene	Adult mBL
mBL_61	chr22	22888058	22888058	G	C	exonic	IGLL5	226	178	0.4406	Missense_Mutation	p.R2T	Targerted Panel Gene	Adult mBL
mBL_61	chr22	22888121	22888121	G	C	exonic	IGLL5	196	155	0.4391	Missense_Mutation	p.R23P	Targerted Panel Gene	Adult mBL
mBL_61	chr8	127738431	127738431	C	A	exonic	MYC	496	237	0.3229	Missense_Mutation	p.P72T	Targerted Panel Gene	Adult mBL
mBL_61	chr16	11255430	11255430	C	T	exonic	SOCS1	138	103	0.4274	Missense_Mutation	p.A17T	Targerted Panel Gene	Adult mBL
mBL_61	chr9	32630460	32630460	C	T	exonic	TAF1L	228	205	0.4724	Missense_Mutation	p.G1707D	Targerted Panel Gene	Adult mBL
mBL_61	chr12	49022063	49022063	G	A	exonic	KMT2D	319	269	0.4567	Nonsense_Mutation	p.R5501X	Targerted Panel Gene	Adult mBL
mBL_62	chr12	122022140	122022147	GATATCAA	-	exonic	BCL7A	188	104	0.3549	Frame_Shift_Del	p.D17fs	Targerted Panel Gene	Adult mBL
mBL_62	chr8	127738485	127738490	GTTGCG	-	exonic	MYC	321	176	0.3541	In_Frame_Del	p.90_91del	Targerted Panel Gene	Adult mBL
mBL_62	chr18	63318663	63318663	C	T	exonic	BCL2	384	289	0.4294	Missense_Mutation	p.A2T	Targerted Panel Gene	Adult mBL
mBL_62	chr18	63318344	63318344	T	C	exonic	BCL2	185	149	0.4461	Missense_Mutation	p.Y108C	Targerted Panel Gene	Adult mBL
mBL_62	chr13	40666142	40666142	G	A	exonic	FOXO1	53	43	0.4479	Missense_Mutation	p.T24I	Targerted Panel Gene	Adult mBL
mBL_62	chr13	40665739	40665739	G	C	exonic	FOXO1	103	62	0.3758	Missense_Mutation	p.N158K	Targerted Panel Gene	Adult mBL
mBL_62	chr1	155784001	155784001	G	T	exonic	GON4L	310	263	0.4582	Missense_Mutation	p.T626N	Targerted Panel Gene	Adult mBL
mBL_62	chr12	49026760	49026760	A	T	exonic	KMT2D	300	187	0.384	Missense_Mutation	p.V5069E	Targerted Panel Gene	Adult mBL
mBL_62	chr8	127736594	127736594	C	G	exonic	MYC	418	235	0.3599	Missense_Mutation	p.L1V	Targerted Panel Gene	Adult mBL
mBL_62	chr8	127740554	127740554	C	G	exonic	MYC	336	254	0.4298	Missense_Mutation	p.Q321E	Targerted Panel Gene	Adult mBL
mBL_62	chr8	127738379	127738379	G	T	exonic	MYC	297	208	0.4119	Missense_Mutation	p.E54D	Targerted Panel Gene	Adult mBL
mBL_62	chr8	127738591	127738591	T	A	exonic	MYC	360	236	0.396	Missense_Mutation	p.M125K	Targerted Panel Gene	Adult mBL
mBL_62	chr2	50236829	50236829	T	C	exonic	NRXN1	419	264	0.3865	Missense_Mutation	p.D1209G	Targerted Panel Gene	Adult mBL
mBL_62	chr1	215634475	215634475	G	A	exonic	USH2A	241	285	0.5408	Missense_Mutation	p.P5094L	Targerted Panel Gene	Adult mBL
mBL_62	chr6	41935993	41935993	G	A	exonic	CCND3	242	297	0.55	Nonsense_Mutation	p.Q276X	Targerted Panel Gene	Adult mBL
mBL_72	chrX	41346590	41346590	G	A	exonic	DDX3X	7	235	0.9711	Missense_Mutation	p.R528H	Targerted Panel Gene	Adult mBL
mBL_72	chrX	31820072	31820072	T	G	exonic	DMD	180	40	0.1818	Missense_Mutation	p.E2281D	Targerted Panel Gene	Adult mBL
mBL_72	chr1	23559188	23559188	A	T	exonic	ID3	29	213	0.8802	Missense_Mutation	p.L80Q	Targerted Panel Gene	Adult mBL
mBL_72	chr22	22893809	22893809	A	T	exonic	IGLL5	247	54	0.1794	Missense_Mutation	p.T106S	Targerted Panel Gene	Adult mBL
mBL_72	chr1	11133150	11133150	T	C	exonic	MTOR	269	247	0.4787	Missense_Mutation	p.N1765S	Targerted Panel Gene	Adult mBL
mBL_72	chr8	127738525	127738525	G	T	exonic	MYC	243	227	0.483	Missense_Mutation	p.G103V	Targerted Panel Gene	Adult mBL
mBL_72	chr13	35196066	35196066	A	T	exonic	NBEA	325	311	0.489	Missense_Mutation	p.E1710D	Targerted Panel Gene	Adult mBL
mBL_72	chr6	117389814	117389814	T	G	exonic	ROS1	284	274	0.491	Missense_Mutation	p.Y432S	Targerted Panel Gene	Adult mBL
mBL_72	chr19	11021944	11021944	C	T	exonic	SMARCA4	256	228	0.4711	Missense_Mutation	p.P946S	Targerted Panel Gene	Adult mBL
mBL_72	chr17	7674220	7674220	C	T	exonic	TP53	220	189	0.4621	Missense_Mutation	p.R209Q	Targerted Panel Gene	Adult mBL
mBL_72	chr8	127738435	127738435	C	T	exonic	MYC	260	246	0.4862	Missense_Mutation	p.T73I	Targerted Panel Gene	Adult mBL

mBL_72	chr1	26697194	26697194	C	A	exonic	ARID1A	122	123	0.502	Nonsense_Mutation	p.S264X	Targerted Panel Gene	Adult mBL
mBL_73	chr10	87933012	87933012	G	A	splicing	PTEN	212	141	0.3994	Splice_Site	.	Targerted Panel Gene	Adult mBL
mBL_73	chrX	41346605	41346605	G	C	exonic	DDX3X	40	180	0.8182	Missense_Mutation	p.G533A	Targerted Panel Gene	Adult mBL
mBL_73	chr13	40666148	40666148	G	C	exonic	FOXO1	49	55	0.5238	Missense_Mutation	p.S22W	Targerted Panel Gene	Adult mBL
mBL_73	chr1	23559201	23559201	A	C	exonic	ID3	262	217	0.453	Missense_Mutation	p.Y76D	Targerted Panel Gene	Adult mBL
mBL_73	chr1	23559285	23559285	A	T	exonic	ID3	278	190	0.406	Missense_Mutation	p.Y48N	Targerted Panel Gene	Adult mBL
mBL_73	chr12	85127925	85127925	A	C	exonic	LRRIQ1	204	268	0.5678	Missense_Mutation	p.E1367D	Targerted Panel Gene	Adult mBL
mBL_73	chr1	11114329	11114329	C	A	exonic	MTOR	213	109	0.3375	Missense_Mutation	p.R2430M	Targerted Panel Gene	Adult mBL
mBL_73	chr8	127738350	127738350	A	C	exonic	MYC	372	232	0.3841	Missense_Mutation	p.N45H	Targerted Panel Gene	Adult mBL
mBL_73	chr9	8319875	8319875	C	A	exonic	PTPRD	257	159	0.3822	Missense_Mutation	p.V1876F	Targerted Panel Gene	Adult mBL
mBL_73	chr1	237640941	237640941	C	T	exonic	RYR2	205	127	0.3825	Missense_Mutation	p.A2387V	Targerted Panel Gene	Adult mBL
mBL_73	chr19	11033317	11033317	C	T	exonic	SMARCA4	247	54	0.1794	Missense_Mutation	p.R1192C	Targerted Panel Gene	Adult mBL
mBL_73	chr19	1612350	1612350	A	T	exonic	TCF3	72	377	0.8396	Missense_Mutation	p.V557E	Targerted Panel Gene	Adult mBL
mBL_73	chr1	215934786	215934786	T	C	exonic	USH2A	104	119	0.5336	Missense_Mutation	p.N2377S	Targerted Panel Gene	Adult mBL
mBL_73	chr3	49375465	49375465	T	C	exonic	RHOA	314	168	0.3485	Missense_Mutation	p.Y42C	Targerted Panel Gene	Adult mBL
mBL_74	chr16	3780731	3780731	C	T	splicing	CREBBP	263	181	0.4077	Splice_Site	.	Targerted Panel Gene	Adult mBL
mBL_74	chr12	49041416	49041416	G	-	exonic	KMT2D	218	142	0.3944	Frame_Shift_Del	p.P2118fs	Targerted Panel Gene	Adult mBL
mBL_74	chr22	41178507	41178509	CAG	-	exonic	EP300	412	314	0.4325	In_Frame_Del	p.2266_2266del	Targerted Panel Gene	Adult mBL
mBL_74	chr18	63318068	63318068	C	G	exonic	BCL2	428	266	0.3816	Missense_Mutation	p.G200A	Targerted Panel Gene	Adult mBL
mBL_74	chr12	122022134	122022134	A	G	exonic	BCL7A	77	266	0.7755	Missense_Mutation	p.K15E	Targerted Panel Gene	Adult mBL
mBL_74	chr5	79729831	79729831	C	G	exonic	CMYA5	372	379	0.5047	Missense_Mutation	p.Q356E	Targerted Panel Gene	Adult mBL
mBL_74	chr7	148811635	148811635	T	G	exonic	EZH2	202	127	0.3848	Missense_Mutation	p.Y646S	Targerted Panel Gene	Adult mBL
mBL_74	chr10	89014257	89014257	A	C	exonic	FAS	422	242	0.3645	Missense_Mutation	p.E272A	Targerted Panel Gene	Adult mBL
mBL_74	chr4	93085178	93085178	C	A	exonic	GRID2	294	200	0.4049	Missense_Mutation	p.T143N	Targerted Panel Gene	Adult mBL
mBL_74	chr1	21873934	21873934	T	C	exonic	HSPG2	133	100	0.4292	Missense_Mutation	p.H1245R	Targerted Panel Gene	Adult mBL
mBL_74	chr22	22888214	22888214	C	T	exonic	IGLL5	42	93	0.6889	Missense_Mutation	p.P19S	Targerted Panel Gene	Adult mBL
mBL_74	chr9	37020761	37020761	A	C	exonic	PAX5	473	295	0.3841	Missense_Mutation	p.N29K	Targerted Panel Gene	Adult mBL
mBL_74	chr13	48463740	48463740	T	C	exonic	RB1	36	103	0.741	Missense_Mutation	p.C706R	Targerted Panel Gene	Adult mBL
mBL_74	chr19	38500664	38500664	T	C	exonic	RYR1	189	175	0.4795	Missense_Mutation	p.V2461A	Targerted Panel Gene	Adult mBL
mBL_74	chr1	2556707	2556707	A	G	exonic	TNFRSF14	87	266	0.7535	Missense_Mutation	p.T15A	Targerted Panel Gene	Adult mBL
mBL_74	chr1	215671270	215671270	C	A	exonic	USH2A	271	146	0.3501	Missense_Mutation	p.C4612F	Targerted Panel Gene	Adult mBL
mBL_74	chr16	3738616	3738616	C	T	exonic	CREBBP	284	229	0.4464	Missense_Mutation	p.R1446H	Targerted Panel Gene	Adult mBL
mBL_74	chr8	127738435	127738435	C	T	exonic	MYC	311	175	0.3601	Missense_Mutation	p.T73I	Targerted Panel Gene	Adult mBL
mBL_74	chr1	2556677	2556677	G	T	exonic	TNFRSF14	84	275	0.766	Nonsense_Mutation	p.G5X	Targerted Panel Gene	Adult mBL
mBL_9	chr17	65014590	65014591	CT	-	exonic	GNA13	317	324	0.5055	Frame_Shift_Del	p.E267fs	Targerted Panel Gene	Adult mBL
mBL_9	chr10	121551381	121551381	C	A	exonic	FGFR2	298	132	0.307	Missense_Mutation	p.R178L	Targerted Panel Gene	Adult mBL
mBL_9	chr22	22888076	22888076	T	C	exonic	IGLL5	296	137	0.3164	Missense_Mutation	p.V8A	Targerted Panel Gene	Adult mBL
mBL_9	chr1	236852530	236852530	C	G	exonic	MTR	224	101	0.3108	Missense_Mutation	p.P569A	Targerted Panel Gene	Adult mBL
mBL_9	chr8	127738925	127738925	T	G	exonic	MYC	338	140	0.2923	Missense_Mutation	p.D236E	Targerted Panel Gene	Adult mBL
mBL_9	chr8	127738440	127738440	C	A	exonic	MYC	326	129	0.2835	Missense_Mutation	p.P75T	Targerted Panel Gene	Adult mBL
mBL_9	chr8	127738539	127738539	T	A	exonic	MYC	317	161	0.3368	Missense_Mutation	p.F108I	Targerted Panel Gene	Adult mBL
mBL_9	chr8	127739002	127739002	C	T	exonic	MYC	389	154	0.2836	Missense_Mutation	p.T262I	Targerted Panel Gene	Adult mBL
mBL_9	chr8	127738947	127738947	T	C	exonic	MYC	355	130	0.268	Missense_Mutation	p.S244P	Targerted Panel Gene	Adult mBL
mBL_9	chr2	151654003	151654003	T	C	exonic	NEB	147	163	0.5258	Missense_Mutation	p.I2302V	Targerted Panel Gene	Adult mBL
mBL_9	chr6	17626024	17626024	C	T	exonic	NUP153	326	310	0.4867	Missense_Mutation	p.G1260R	Targerted Panel Gene	Adult mBL
mBL_9	chr9	32633647	32633647	C	T	exonic	TAF1L	406	177	0.3036	Missense_Mutation	p.G645S	Targerted Panel Gene	Adult mBL
mBL_9	chr19	1612367	1612367	A	C	exonic	TCF3	266	117	0.3055	Missense_Mutation	p.N551K	Targerted Panel Gene	Adult mBL
mBL_9	chr8	102298933	102298933	G	A	exonic	UBR5	324	122	0.2735	Nonsense_Mutation	p.R1081X	Targerted Panel Gene	Adult mBL
mBL_20	chrX	41339085	41339085	T	C	splicing	DDX3X	201	199	0.4975	Splice_Site	.	Targerted Panel Gene	Pediatric mBL
mBL_20	chr1	23559126	23559126	C	T	splicing	ID3	169	478	0.7388	Splice_Site	.	Targerted Panel Gene	Pediatric mBL
mBL_20	chr8	143870942	143870942	T	C	exonic	EPPK1	381	368	0.4913	Missense_Mutation	p.N771S	Targerted Panel Gene	Pediatric mBL
mBL_20	chr1	228457443	228457443	G	C	exonic	HIST3H2A	50	41	0.4505	Missense_Mutation	p.H125Q	Targerted Panel Gene	Pediatric mBL
mBL_20	chr8	127738440	127738440	C	A	exonic	MYC	495	126	0.2029	Missense_Mutation	p.P75T	Targerted Panel Gene	Pediatric mBL
mBL_20	chr8	127738797	127738797	C	G	exonic	MYC	328	209	0.3892	Missense_Mutation	p.Q194E	Targerted Panel Gene	Pediatric mBL
mBL_20	chr8	127738392	127738392	G	A	exonic	MYC	403	241	0.3736	Missense_Mutation	p.A59T	Targerted Panel Gene	Pediatric mBL
mBL_20	chr10	94032053	94032053	T	G	exonic	PLCE1	661	240	0.2664	Missense_Mutation	p.V336G	Targerted Panel Gene	Pediatric mBL
mBL_20	chr1	237491853	237491853	C	G	exonic	RYR2	566	57	0.0915	Missense_Mutation	p.L586V	Targerted Panel Gene	Pediatric mBL
mBL_20	chr17	7674223	7674223	T	A	exonic	TP53	210	117	0.3578	Missense_Mutation	p.N208I	Targerted Panel Gene	Pediatric mBL
mBL_20	chr17	7673824	7673824	C	T	exonic	TP53	324	60	0.1562	Missense_Mutation	p.G227R	Targerted Panel Gene	Pediatric mBL
mBL_20	chr13	44576001	44576001	G	A	exonic	TSC22D1	249	314	0.5577	Missense_Mutation	p.P25L	Targerted Panel Gene	Pediatric mBL
mBL_20	chr3	65357029	65357029	G	C	exonic	MAGI1	372	296	0.4431	Nonsense_Mutation	p.Y1246X	Targerted Panel Gene	Pediatric mBL
mBL_22	chr12	122022184	122022184	G	A	splicing	BCL7A	155	93	0.375	Splice_Site	.	Targerted Panel Gene	Pediatric mBL
mBL_22	chr12	122022146	122022146	A	C	exonic	BCL7A	216	117	0.3514	Missense_Mutation	p.K19Q	Targerted Panel Gene	Pediatric mBL
mBL_22	chr8	112954725	112954725	T	G	exonic	CSMD3	405	281	0.4096	Missense_Mutation	p.K420T	Targerted Panel Gene	Pediatric mBL
mBL_22	chr13	40666136	40666136	G	A	exonic	FOXO1	56	44	0.44	Missense_Mutation	p.P26L	Targerted Panel Gene	Pediatric mBL
mBL_22	chr17	65014564	65014564	A	C	exonic	GNA13	641	505	0.4407	Missense_Mutation	p.V276G	Targerted Panel Gene	Pediatric mBL
mBL_22	chr6	26183977	26183977	G	A	exonic	HIST1H2BE	182	108	0.3711	Missense_Mutation	p.G61E	Targerted Panel Gene	Pediatric mBL
mBL_22	chr6	26250271	26250271	G	A	exonic	HIST1H3F	117	78	0.4	Missense_Mutation	p.A112V	Targerted Panel Gene	Pediatric mBL
mBL_22	chr1	23559237	23559237	G	A	exonic	ID3	211	265	0.5544	Missense_Mutation	p.L64F	Targerted Panel Gene	Pediatric mBL
mBL_22	chr1	23558973	23558973	C	T	exonic	ID3	325	236	0.4207	Missense_Mutation	p.S116N	Targerted Panel Gene	Pediatric mBL
mBL_22	chr7	152182036	152182036	T	C	exonic	KMT2C	579	493	0.459	Missense_Mutation	p.T1942A	Targerted Panel Gene	Pediatric mBL
mBL_22	chr8	127738786	127738786	G	A	exonic	MYC	438	84	0.1609	Missense_Mutation	p.S190N	Targerted Panel Gene	Pediatric mBL
mBL_22	chr8	127738292	127738292	G	C	exonic	MYC	499	351	0.4086	Missense_Mutation	p.R25S	Targerted Panel Gene	Pediatric mBL
mBL_22	chr8	127738317	127738317	G	T	exonic	MYC	467	304	0.3943	Missense_Mutation	p.V34L	Targerted Panel Gene	Pediatric mBL
mBL_22	chr13	101068773	101068773	C	T	exonic	NALCN	405	414	0.5036	Missense_Mutation	p.G1418R	Targerted Panel Gene	Pediatric mBL
mBL_22	chr10	46584613	46584613	T	C	exonic	SYT15	496	132	0.2102	Missense_Mutation	p.S315G	Targerted Panel Gene	Pediatric mBL
mBL_22	chr19	1612338	1612338	T	G	exonic	TCF3	349	244	0.4115	Missense_Mutation	p.D561A	Targerted Panel Gene	Pediatric mBL
mBL_22	chr19	1612350	1612350	A	G	exonic	TCF3	359	224	0.3842	Missense_Mutation	p.V557A	Targerted Panel Gene	Pediatric mBL
mBL_22	chr6	75284472	75284472	A	G	exonic	TMEM30A	417	245	0.3701	Missense_Mutation	p.I56T	Targerted Panel Gene	Pediatric mBL
mBL_22	chr13	44573298	44573298	T	C	exonic	TSC22D1	448	388	0.4614	Missense_Mutation	p.Q926R	Targerted Panel Gene	Pediatric mBL
mBL_22	chrX	71252958	71252958	C	A	exonic	ZMYM3	55	312	0.8501	Nonsense_Mutation	p.E100X	Targerted Panel Gene	Pediatric mBL
mBL_23	chrX	41343743	41343743	-	A	exonic	DDX3X	47	380	0.8879	Frame_Shift_Ins	p.G229fs	Targerted Panel Gene	Pediatric mBL

mBL_23	chr17	65014418	65014418	-	T	exonic	GNA13	483	247	0.3384	Frame_Shift_Ins	p.F325fs	Targerted Panel Gene	Pediatric mBL
mBL_23	chr12	132062548	132062548	-	CAGCAGCAGCAG	exonic	EP400	148	30	0.1639	In_Frame_Ins	p.Q2727delinsQQ	Targerted Panel Gene	Pediatric mBL
mBL_23	chr6	41936027	41936027	G	T	exonic	CCND3	187	60	0.2419	Missense_Mutation	p.S264R	Targerted Panel Gene	Pediatric mBL
mBL_23	chr3	62370136	62370136	C	G	exonic	FEZF2	43	46	0.5169	Missense_Mutation	p.V443L	Targerted Panel Gene	Pediatric mBL
mBL_23	chr1	23559237	23559237	G	A	exonic	ID3	237	170	0.4177	Missense_Mutation	p.L64F	Targerted Panel Gene	Pediatric mBL
mBL_23	chr1	23559203	23559203	T	C	exonic	ID3	237	150	0.3876	Missense_Mutation	p.D75G	Targerted Panel Gene	Pediatric mBL
mBL_23	chr1	201224701	201224701	C	A	exonic	IGFN1	178	153	0.4622	Missense_Mutation	p.T3438N	Targerted Panel Gene	Pediatric mBL
mBL_23	chr19	11033441	11033441	T	C	exonic	SMARCA4	227	140	0.3804	Missense_Mutation	p.M1233T	Targerted Panel Gene	Pediatric mBL
mBL_23	chr17	7674220	7674220	C	T	exonic	TP53	206	137	0.3994	Missense_Mutation	p.R209Q	Targerted Panel Gene	Pediatric mBL
mBL_25	chr18	79396352	79396352	G	A	splicing	NFATC1	105	74	0.4134	Splice_Site	.	Targerted Panel Gene	Pediatric mBL
mBL_25	chr1	23559319	23559319	C	-	exonic	ID3	107	190	0.6397	Frame_Shift_Del	p.E36fs	Targerted Panel Gene	Pediatric mBL
mBL_25	chr6	156778889	156778889	-	GGA	exonic	ARID1B	109	37	0.2434	In_Frame_Ins	p.S320delinsSG	Targerted Panel Gene	Pediatric mBL
mBL_25	chr16	77367584	77367584	T	C	exonic	ADAMTS18	393	361	0.4781	Missense_Mutation	p.Q212R	Targerted Panel Gene	Pediatric mBL
mBL_25	chr19	10759775	10759775	C	T	exonic	DNM2	380	219	0.365	Missense_Mutation	p.R67W	Targerted Panel Gene	Pediatric mBL
mBL_25	chr17	65056415	65056415	C	G	exonic	GNA13	196	246	0.5541	Missense_Mutation	p.G60A	Targerted Panel Gene	Pediatric mBL
mBL_25	chr13	101107566	101107566	C	T	exonic	NALCN	427	261	0.3777	Missense_Mutation	p.D834N	Targerted Panel Gene	Pediatric mBL
mBL_25	chr1	183538394	183538394	G	A	exonic	SMG7	260	127	0.3282	Missense_Mutation	p.E417K	Targerted Panel Gene	Pediatric mBL
mBL_25	chr1	26774532	26774532	T	G	exonic	ARID1A	414	188	0.3123	Nonsense_Mutation	p.Y1435X	Targerted Panel Gene	Pediatric mBL
mBL_26	chr1	228215003	228215003	A	C	exonic	OBSCN	404	350	0.4642	Missense_Mutation	p.D578A	Targerted Panel Gene	Pediatric mBL
mBL_27	chr6	41936007	41936007	-	G	exonic	CCND3	285	178	0.3836	Frame_Shift_Ins	p.R271fs	Targerted Panel Gene	Pediatric mBL
mBL_27	chrX	41346239	41346239	-	CTGAC	exonic	DDX3X	50	256	0.8339	Frame_Shift_Ins	p.S442fs	Targerted Panel Gene	Pediatric mBL
mBL_27	chr3	187725578	187725578	G	T	exonic	BCL6	491	452	0.4788	Missense_Mutation	p.A587D	Targerted Panel Gene	Pediatric mBL
mBL_27	chr5	79731143	79731143	C	T	exonic	CMYA5	466	417	0.4712	Missense_Mutation	p.P793L	Targerted Panel Gene	Pediatric mBL
mBL_27	chr7	137915842	137915842	T	G	exonic	CREB3L2	208	332	0.6137	Missense_Mutation	p.T164P	Targerted Panel Gene	Pediatric mBL
mBL_27	chr16	3728423	3728423	T	G	exonic	CREBBP	168	197	0.5397	Missense_Mutation	p.Q2208H	Targerted Panel Gene	Pediatric mBL
mBL_27	chr13	40666157	40666157	C	T	exonic	FOXO1	104	14	0.1176	Missense_Mutation	p.R19Q	Targerted Panel Gene	Pediatric mBL
mBL_27	chr4	93085130	93085130	C	A	exonic	GRID2	264	211	0.4442	Missense_Mutation	p.P127Q	Targerted Panel Gene	Pediatric mBL
mBL_27	chr8	127736597	127736597	G	A	exonic	MYC	691	78	0.1014	Missense_Mutation	p.D2N	Targerted Panel Gene	Pediatric mBL
mBL_27	chr6	51619140	51619140	C	T	exonic	PKHD1	287	256	0.4715	Missense_Mutation	p.A4056T	Targerted Panel Gene	Pediatric mBL
mBL_27	chr19	10994970	10994970	G	C	exonic	SMARCA4	349	124	0.2616	Missense_Mutation	p.R521P	Targerted Panel Gene	Pediatric mBL
mBL_27	chr19	1612367	1612367	A	T	exonic	TCF3	291	267	0.4785	Missense_Mutation	p.N551K	Targerted Panel Gene	Pediatric mBL
mBL_28	chr6	41935950	41935950	A	G	exonic	CCND3	200	163	0.4478	Missense_Mutation	p.I290T	Targerted Panel Gene	Pediatric mBL
mBL_28	chr1	23559261	23559261	G	A	exonic	ID3	211	158	0.427	Missense_Mutation	p.P56S	Targerted Panel Gene	Pediatric mBL
mBL_28	chr1	23559127	23559127	C	G	exonic	ID3	178	164	0.4795	Missense_Mutation	p.Q100H	Targerted Panel Gene	Pediatric mBL
mBL_28	chr12	49031906	49031906	G	A	exonic	KMT2D	183	201	0.5234	Missense_Mutation	p.P4267S	Targerted Panel Gene	Pediatric mBL
mBL_28	chr8	127738373	127738373	G	C	exonic	MYC	195	175	0.4704	Missense_Mutation	p.Q52H	Targerted Panel Gene	Pediatric mBL
mBL_28	chr8	127738675	127738675	T	C	exonic	MYC	208	178	0.4611	Missense_Mutation	p.F153S	Targerted Panel Gene	Pediatric mBL
mBL_28	chr20	41159662	41159662	C	T	exonic	PLCG1	257	236	0.4787	Missense_Mutation	p.R92C	Targerted Panel Gene	Pediatric mBL
mBL_28	chr19	38525492	38525492	G	A	exonic	RYR1	102	116	0.5321	Missense_Mutation	p.R3539H	Targerted Panel Gene	Pediatric mBL
mBL_28	chr17	7674275	7674275	T	C	exonic	TP53	146	118	0.447	Missense_Mutation	p.T191A	Targerted Panel Gene	Pediatric mBL
mBL_28	chr17	7674248	7674248	T	C	exonic	TP53	150	116	0.4361	Missense_Mutation	p.N200D	Targerted Panel Gene	Pediatric mBL
mBL_28	chr17	7674225	7674225	C	T	exonic	TP53	161	134	0.4527	Missense_Mutation	p.M207I	Targerted Panel Gene	Pediatric mBL
mBL_28	chr1	26779185	26779185	G	T	exonic	ARID1A	207	170	0.4509	Nonsense_Mutation	p.E1546X	Targerted Panel Gene	Pediatric mBL
mBL_28	chr3	177033096	177033096	G	A	exonic	TBL1XR1	219	175	0.443	Nonsense_Mutation	p.R431X	Targerted Panel Gene	Pediatric mBL
mBL_28	chr1	15219486	15219486	G	T	exonic	TMEM51	419	52	0.1104	Nonsense_Mutation	p.E169X	Targerted Panel Gene	Pediatric mBL
mBL_29	chr1	33557743	33557743	G	C	exonic	CSMD2	112	54	0.3253	Missense_Mutation	p.Q2912E	Targerted Panel Gene	Pediatric mBL
mBL_29	chr8	112380354	112380354	G	T	exonic	CSMD3	117	77	0.3969	Missense_Mutation	p.T2005K	Targerted Panel Gene	Pediatric mBL
mBL_29	chr1	23559236	23559236	A	C	exonic	ID3	54	229	0.8092	Missense_Mutation	p.L64R	Targerted Panel Gene	Pediatric mBL
mBL_29	chr3	49368499	49368499	A	C	exonic	RHOA	196	166	0.4586	Missense_Mutation	p.L69R	Targerted Panel Gene	Pediatric mBL
mBL_29	chr2	228018354	228018354	A	G	exonic	SPHKAP	186	205	0.523	Missense_Mutation	p.Y834H	Targerted Panel Gene	Pediatric mBL
mBL_29	chr11	103243696	103243696	C	T	exonic	DYNC2H1	184	152	0.4524	Nonsense_Mutation	p.R3282X	Targerted Panel Gene	Pediatric mBL
mBL_29	chr1	23559270	23559270	C	A	exonic	ID3	261	36	0.1212	Nonsense_Mutation	p.E53X	Targerted Panel Gene	Pediatric mBL
mBL_30	chr17	65018251	65018251	A	T	splicing	GNA13	272	171	0.386	Splice_Site	.	Targerted Panel Gene	Pediatric mBL
mBL_30	chr10	52133902	52133902	G	T	exonic	PRKG1	133	61	0.3144	Missense_Mutation	p.R333I	Targerted Panel Gene	Pediatric mBL
mBL_30	chr19	38579989	38579989	T	G	exonic	RYR1	133	117	0.468	Missense_Mutation	p.L4791R	Targerted Panel Gene	Pediatric mBL
mBL_30	chr17	7674220	7674220	C	T	exonic	TP53	205	73	0.2626	Missense_Mutation	p.R209Q	Targerted Panel Gene	Pediatric mBL
mBL_30	chrX	41334295	41334295	C	T	exonic	DDX3X	25	133	0.8418	Nonsense_Mutation	p.Q15X	Targerted Panel Gene	Pediatric mBL
mBL_30	chr17	65056515	65056515	G	A	exonic	GNA13	195	115	0.371	Nonsense_Mutation	p.Q27X	Targerted Panel Gene	Pediatric mBL
mBL_31	chrX	41343735	41343735	A	G	splicing	DDX3X	20	198	0.9083	Splice_Site	.	Targerted Panel Gene	Pediatric mBL
mBL_31	chr17	65014660	65014660	A	G	exonic	GNA13	579	371	0.3905	Missense_Mutation	p.L244P	Targerted Panel Gene	Pediatric mBL
mBL_31	chr1	23559237	23559237	G	A	exonic	ID3	416	286	0.4074	Missense_Mutation	p.L64F	Targerted Panel Gene	Pediatric mBL
mBL_31	chr1	23559261	23559261	G	A	exonic	ID3	375	294	0.4395	Missense_Mutation	p.P56S	Targerted Panel Gene	Pediatric mBL
mBL_31	chr12	53471660	53471660	G	A	exonic	PCBP2	78	69	0.4694	Missense_Mutation	p.R302H	Targerted Panel Gene	Pediatric mBL
mBL_31	chr7	83135423	83135423	T	G	exonic	PCLO	661	473	0.4171	Missense_Mutation	p.Q709H	Targerted Panel Gene	Pediatric mBL
mBL_31	chr21	41847167	41847167	G	T	exonic	PRDM15	203	156	0.4345	Missense_Mutation	p.A554D	Targerted Panel Gene	Pediatric mBL
mBL_31	chr19	11033323	11033323	G	A	exonic	SMARCA4	236	166	0.4129	Missense_Mutation	p.G1194R	Targerted Panel Gene	Pediatric mBL
mBL_31	chr3	177051705	177051705	G	A	exonic	TBL1XR1	215	201	0.4832	Nonsense_Mutation	p.R76X	Targerted Panel Gene	Pediatric mBL
mBL_32	chr11	118501690	118501690	-	T	exonic	KMT2A	1199	438	0.2672	Frame_Shift_Ins	p.S2110fs	Targerted Panel Gene	Pediatric mBL
mBL_32	chr12	132062548	132062548	-	CAGCAGCAG	exonic	EP400	413	53	0.1106	In_Frame_Ins	p.Q2727delinsQQ	Targerted Panel Gene	Pediatric mBL
mBL_32	chr7	2944252	2944252	T	G	exonic	CARD11	744	277	0.2713	Missense_Mutation	p.K215T	Targerted Panel Gene	Pediatric mBL
mBL_32	chr6	41935972	41935972	T	C	exonic	CCND3	387	231	0.3738	Missense_Mutation	p.T283A	Targerted Panel Gene	Pediatric mBL
mBL_32	chr16	88806562	88806562	T	C	exonic	CDT1	219	177	0.447	Missense_Mutation	p.V337A	Targerted Panel Gene	Pediatric mBL
mBL_32	chr1	23559200	23559200	T	G	exonic	ID3	480	288	0.375	Missense_Mutation	p.Y76S	Targerted Panel Gene	Pediatric mBL
mBL_32	chr2	169226481	169226481	T	C	exonic	LRP2	643	279	0.3026	Missense_Mutation	p.N1779D	Targerted Panel Gene	Pediatric mBL
mBL_32	chr8	127738925	127738925	T	G	exonic	MYC	446	248	0.3573	Missense_Mutation	p.D236E	Targerted Panel Gene	Pediatric mBL
mBL_32	chr8	127738491	127738491	G	A	exonic	MYC	463	278	0.3752	Missense_Mutation	p.V92I	Targerted Panel Gene	Pediatric mBL
mBL_32	chr8	127738279	127738279	G	A	exonic	MYC	563	332	0.3705	Missense_Mutation	p.S21N	Targerted Panel Gene	Pediatric mBL
mBL_32	chr8	127738428	127738428	C	A	exonic	MYC	477	274	0.3648	Missense_Mutation	p.L71M	Targerted Panel Gene	Pediatric mBL
mBL_32	chr8	127738920	127738920	T	C	exonic	MYC	440	235	0.3476	Missense_Mutation	p.S235P	Targerted Panel Gene	Pediatric mBL
mBL_32	chr8	127738992	127738992	A	T	exonic	MYC	526	280	0.347	Missense_Mutation	p.T259S	Targerted Panel Gene	Pediatric mBL
mBL_32	chr8	127738266	127738266	C	T	exonic	MYC	769	121	0.136	Missense_Mutation	p.P17S	Targerted Panel Gene	Pediatric mBL
mBL_32	chr9	137522853	137522853	T	C	exonic	PNPLA7	342	326	0.488	Missense_Mutation	p.H251R	Targerted Panel Gene	Pediatric mBL

mBL_32	chr10	87864498	87864498	G	A	exonic	PTEN	810	504	0.383	Missense_Mutation	p.S183N	Targerted Panel Gene	Pediatric mBL
mBL_32	chr7	98906195	98906195	A	T	exonic	TRRAP	591	344	0.3679	Missense_Mutation	p.D352V	Targerted Panel Gene	Pediatric mBL
mBL_33	chr1	23559222	23559222	T	-	exonic	ID3	512	360	0.4128	Frame_Shift_Del	p.I69fs	Targerted Panel Gene	Pediatric mBL
mBL_33	chr17	65014809	65014811	TTG	-	exonic	GNA13	444	476	0.5163	In_Frame_Del	p.194_194del	Targerted Panel Gene	Pediatric mBL
mBL_33	chr8	60865256	60865256	A	G	exonic	CHD7	471	444	0.4852	Missense_Mutation	p.M2773V	Targerted Panel Gene	Pediatric mBL
mBL_33	chr2	96145103	96145103	C	G	exonic	DUSP2	38	36	0.4865	Missense_Mutation	p.E84D	Targerted Panel Gene	Pediatric mBL
mBL_33	chr17	65056415	65056415	C	T	exonic	GNA13	508	132	0.2056	Missense_Mutation	p.G60D	Targerted Panel Gene	Pediatric mBL
mBL_33	chr6	26055981	26055981	T	G	exonic	HIST1H1C	660	478	0.4197	Missense_Mutation	p.S150R	Targerted Panel Gene	Pediatric mBL
mBL_33	chr1	23559228	23559228	C	A	exonic	ID3	468	382	0.4494	Missense_Mutation	p.V67L	Targerted Panel Gene	Pediatric mBL
mBL_33	chr1	23559236	23559236	A	T	exonic	ID3	499	372	0.4271	Missense_Mutation	p.L64H	Targerted Panel Gene	Pediatric mBL
mBL_33	chr8	127738431	127738431	C	T	exonic	MYC	420	296	0.4134	Missense_Mutation	p.P72S	Targerted Panel Gene	Pediatric mBL
mBL_33	chr8	127738432	127738432	C	G	exonic	MYC	425	290	0.4056	Missense_Mutation	p.P72R	Targerted Panel Gene	Pediatric mBL
mBL_33	chr8	127739012	127739012	C	G	exonic	MYC	504	398	0.4398	Missense_Mutation	p.S265R	Targerted Panel Gene	Pediatric mBL
mBL_33	chr8	127738486	127738486	T	C	exonic	MYC	440	342	0.4373	Missense_Mutation	p.V90A	Targerted Panel Gene	Pediatric mBL
mBL_33	chr21	41836584	41836584	C	A	exonic	PRDM15	517	450	0.4654	Missense_Mutation	p.G722V	Targerted Panel Gene	Pediatric mBL
mBL_33	chr1	215888472	215888472	C	T	exonic	USH2A	390	685	0.6372	Missense_Mutation	p.G2726E	Targerted Panel Gene	Pediatric mBL
mBL_33	chr14	100277552	100277552	C	A	exonic	YY1	685	561	0.4502	Missense_Mutation	p.N399K	Targerted Panel Gene	Pediatric mBL
mBL_33	chr19	22088126	22088126	T	C	exonic	ZNF257	82	12	0.1277	Missense_Mutation	p.C50R	Targerted Panel Gene	Pediatric mBL
mBL_33	chr19	22088127	22088127	G	A	exonic	ZNF257	79	12	0.1319	Missense_Mutation	p.C50Y	Targerted Panel Gene	Pediatric mBL
mBL_33	chr19	22088132	22088132	G	C	exonic	ZNF257	75	12	0.1379	Missense_Mutation	p.G52R	Targerted Panel Gene	Pediatric mBL
mBL_33	chr19	22088133	22088133	G	A	exonic	ZNF257	76	12	0.1364	Missense_Mutation	p.G52E	Targerted Panel Gene	Pediatric mBL
mBL_33	chr1	15219486	15219486	G	T	exonic	TMEM51	948	120	0.1124	Nonsense_Mutation	p.E169X	Targerted Panel Gene	Pediatric mBL
mBL_34	chr6	26273108	26273108	G	T	exonic	HIST1H2BI	74	20	0.2128	Missense_Mutation	p.V45L	Targerted Panel Gene	Pediatric mBL
mBL_34	chr8	127736619	127736619	A	G	exonic	MYC	148	150	0.5034	Missense_Mutation	p.N9S	Targerted Panel Gene	Pediatric mBL
mBL_34	chr19	1612338	1612338	T	G	exonic	TCF3	92	79	0.462	Missense_Mutation	p.D561A	Targerted Panel Gene	Pediatric mBL
mBL_34	chr6	41936041	41936041	G	A	exonic	CCND3	74	88	0.5432	Nonsense_Mutation	p.Q260X	Targerted Panel Gene	Pediatric mBL
mBL_34	chr17	7676059	7676059	G	A	exonic	TP53	14	196	0.9289	Nonsense_Mutation	p.Q65X	Targerted Panel Gene	Pediatric mBL
mBL_35	chr1	23559126	23559126	C	G	splicing	ID3	214	194	0.4743	Splice_Site	.	Targerted Panel Gene	Pediatric mBL
mBL_35	chr12	124426641	124426642	TC	-	exonic	NCOR2	175	110	0.386	Frame_Shift_Del	p.E436fs	Targerted Panel Gene	Pediatric mBL
mBL_35	chr6	41935971	41935971	G	A	exonic	CCND3	220	190	0.4634	Missense_Mutation	p.T283I	Targerted Panel Gene	Pediatric mBL
mBL_35	chr3	50256263	50256263	G	A	exonic	GNAI2	229	197	0.4614	Missense_Mutation	p.R163H	Targerted Panel Gene	Pediatric mBL
mBL_35	chr8	127736597	127736597	G	A	exonic	MYC	315	286	0.4735	Missense_Mutation	p.D2N	Targerted Panel Gene	Pediatric mBL
mBL_35	chr12	124426646	124426646	T	G	exonic	NCOR2	174	115	0.3979	Missense_Mutation	p.Q435P	Targerted Panel Gene	Pediatric mBL
mBL_35	chr19	11033318	11033318	G	A	exonic	SMARCA4	140	103	0.4221	Missense_Mutation	p.R1192H	Targerted Panel Gene	Pediatric mBL
mBL_35	chr17	7675085	7675085	C	A	exonic	TP53	16	157	0.9075	Missense_Mutation	p.C137F	Targerted Panel Gene	Pediatric mBL
mBL_35	chr1	23559186	23559186	G	A	exonic	ID3	235	185	0.4405	Nonsense_Mutation	p.Q81X	Targerted Panel Gene	Pediatric mBL
mBL_35	chr1	15219486	15219486	G	T	exonic	TMEM51	521	63	0.1075	Nonsense_Mutation	p.E169X	Targerted Panel Gene	Pediatric mBL
mBL_36	chr1	185994858	185994858	A	C	exonic	HMCN1	211	155	0.4235	Missense_Mutation	p.Q1183H	Targerted Panel Gene	Pediatric mBL
mBL_36	chr8	127738707	127738707	C	G	exonic	MYC	118	112	0.487	Missense_Mutation	p.L164V	Targerted Panel Gene	Pediatric mBL
mBL_36	chr9	131627916	131627916	G	C	exonic	RAPGEF1	95	80	0.4571	Missense_Mutation	p.L401V	Targerted Panel Gene	Pediatric mBL
mBL_36	chr1	237491815	237491815	A	G	exonic	RYR2	133	116	0.4659	Missense_Mutation	p.E573G	Targerted Panel Gene	Pediatric mBL
mBL_36	chr19	11021837	11021837	C	T	exonic	SMARCA4	148	99	0.4008	Missense_Mutation	p.T910M	Targerted Panel Gene	Pediatric mBL
mBL_36	chr5	168062087	168062087	T	C	exonic	TENM2	136	106	0.4327	Missense_Mutation	p.I446T	Targerted Panel Gene	Pediatric mBL
mBL_36	chr17	7675216	7675216	C	G	exonic	TP53	177	142	0.4451	Missense_Mutation	p.K93N	Targerted Panel Gene	Pediatric mBL
mBL_36	chr17	58308753	58308753	G	A	exonic	BZRAP1	90	84	0.4828	Nonsense_Mutation	p.Q1507X	Targerted Panel Gene	Pediatric mBL
mBL_36	chr17	7673789	7673789	A	T	exonic	TP53	164	114	0.4101	Nonsense_Mutation	p.C238X	Targerted Panel Gene	Pediatric mBL
mBL_37	chr1	23559125	23559125	A	G	splicing	ID3	209	162	0.4355	Splice_Site	.	Targerted Panel Gene	Pediatric mBL
mBL_37	chr17	65053653	65053653	-	A	exonic	GNA13	405	336	0.4534	Frame_Shift_Ins	p.D120fs	Targerted Panel Gene	Pediatric mBL
mBL_37	chr17	58307710	58307710	C	A	exonic	BZRAP1	258	256	0.4981	Missense_Mutation	p.R1628S	Targerted Panel Gene	Pediatric mBL
mBL_37	chrX	41346262	41346262	C	T	exonic	DDX3X	21	295	0.9335	Missense_Mutation	p.T450I	Targerted Panel Gene	Pediatric mBL
mBL_37	chr13	40666148	40666148	G	C	exonic	FOXO1	45	41	0.4767	Missense_Mutation	p.S22W	Targerted Panel Gene	Pediatric mBL
mBL_37	chr1	23559237	23559237	G	A	exonic	ID3	217	193	0.4707	Missense_Mutation	p.L64F	Targerted Panel Gene	Pediatric mBL
mBL_37	chr8	127736594	127736594	C	G	exonic	MYC	327	304	0.4818	Missense_Mutation	p.L1V	Targerted Panel Gene	Pediatric mBL
mBL_37	chr8	127738434	127738434	A	G	exonic	MYC	218	176	0.4467	Missense_Mutation	p.T73A	Targerted Panel Gene	Pediatric mBL
mBL_37	chr3	77568379	77568379	T	C	exonic	ROBO2	255	188	0.4244	Missense_Mutation	p.V46A	Targerted Panel Gene	Pediatric mBL
mBL_37	chr1	26729750	26729750	C	T	exonic	ARID1A	289	235	0.4476	Nonsense_Mutation	p.Q413X	Targerted Panel Gene	Pediatric mBL
mBL_37	chr17	65014688	65014688	C	A	exonic	GNA13	471	398	0.458	Nonsense_Mutation	p.E235X	Targerted Panel Gene	Pediatric mBL
mBL_38	chr3	50257024	50257026	AAG	-	exonic	GNAI2	484	324	0.401	In_Frame_Del	p.255_255del	Targerted Panel Gene	Pediatric mBL
mBL_38	chr1	26696448	26696448	-	CCGCCG	exonic	ARID1A	56	18	0.2368	In_Frame_Ins	p.N15delinsNPP	Targerted Panel Gene	Pediatric mBL
mBL_38	chr22	41178373	41178373	C	T	exonic	EP300	397	350	0.4685	Missense_Mutation	p.P2221L	Targerted Panel Gene	Pediatric mBL
mBL_38	chr3	183555400	183555400	C	A	exonic	KLHL6	603	208	0.2565	Missense_Mutation	p.C85F	Targerted Panel Gene	Pediatric mBL
mBL_38	chr2	140813695	140813695	T	G	exonic	LRP1B	374	353	0.4856	Missense_Mutation	p.K1774T	Targerted Panel Gene	Pediatric mBL
mBL_38	chr3	38140768	38140768	T	C	exonic	MYD88	356	410	0.5352	Missense_Mutation	p.M232T	Targerted Panel Gene	Pediatric mBL
mBL_48	chr14	32600785	32600785	G	T	exonic	AKAP6	207	161	0.4375	Missense_Mutation	p.S908I	Targerted Panel Gene	Pediatric mBL
mBL_48	chr6	41935950	41935950	A	G	exonic	CCND3	236	182	0.4354	Missense_Mutation	p.I290T	Targerted Panel Gene	Pediatric mBL
mBL_48	chr5	79689959	79689959	G	A	exonic	CMYA5	87	67	0.4351	Missense_Mutation	p.G18R	Targerted Panel Gene	Pediatric mBL
mBL_48	chr8	127738296	127738296	T	A	exonic	MYC	251	217	0.4627	Missense_Mutation	p.Y27N	Targerted Panel Gene	Pediatric mBL
mBL_48	chr19	11033300	11033300	C	A	exonic	SMARCA4	104	98	0.4851	Missense_Mutation	p.A1186E	Targerted Panel Gene	Pediatric mBL
mBL_48	chr17	7673775	7673775	C	G	exonic	TP53	235	171	0.4181	Missense_Mutation	p.R243P	Targerted Panel Gene	Pediatric mBL
mBL_48	chr19	23360674	23360674	G	C	exonic	ZNF91	210	211	0.5	Missense_Mutation	p.P769A	Targerted Panel Gene	Pediatric mBL
mBL_48	chrX	41339068	41339068	C	T	exonic	DDX3X	29	209	0.8745	Nonsense_Mutation	p.R46X	Targerted Panel Gene	Pediatric mBL
mBL_49	chr16	3767801	3767801	-	T	exonic	CREBBP	664	328	0.33	Frame_Shift_Ins	p.V1057fs	Targerted Panel Gene	Pediatric mBL
mBL_49	chr1	116519267	116519267	C	T	exonic	CD58	378	347	0.478	Missense_Mutation	p.G236D	Targerted Panel Gene	Pediatric mBL
mBL_49	chr8	4637390	4637390	A	G	exonic	CSMD1	351	364	0.5091	Missense_Mutation	p.I85T	Targerted Panel Gene	Pediatric mBL
mBL_49	chrX	41346375	41346375	C	T	exonic	DDX3X	147	318	0.6824	Missense_Mutation	p.R488C	Targerted Panel Gene	Pediatric mBL
mBL_49	chr13	40666158	40666158	G	A	exonic	FOXO1	69	35	0.3333	Missense_Mutation	p.R19W	Targerted Panel Gene	Pediatric mBL
mBL_49	chr10	128106059	128106059	T	A	exonic	MKI67	484	484	0.5	Missense_Mutation	p.K1927N	Targerted Panel Gene	Pediatric mBL
mBL_49	chr9	8404560	8404560	C	A	exonic	PTPRD	538	118	0.1799	Missense_Mutation	p.R1396L	Targerted Panel Gene	Pediatric mBL
mBL_49	chr15	33629944	33629944	G	C	exonic	RYR3	310	323	0.5095	Missense_Mutation	p.R895P	Targerted Panel Gene	Pediatric mBL
mBL_49	chr2	197401515	197401515	T	C	exonic	SF3B1	532	253	0.3223	Missense_Mutation	p.Q794R	Targerted Panel Gene	Pediatric mBL
mBL_49	chr1	23559283	23559283	G	C	exonic	ID3	149	355	0.703	Nonsense_Mutation	p.Y48X	Targerted Panel Gene	Pediatric mBL
mBL_49	chr1	15219486	15219486	G	T	exonic	TMEM51	517	188	0.2659	Nonsense_Mutation	p.E169X	Targerted Panel Gene	Pediatric mBL

mBL_50	chr2	32505082	32505082	C	T	exonic	BIRC6	301	208	0.4078	Missense_Mutation	p.R3193C	Targerted Panel Gene	Pediatric mBL
mBL_50	chr19	17011603	17011603	G	A	exonic	CPAMD8	434	181	0.2938	Missense_Mutation	p.P188L	Targerted Panel Gene	Pediatric mBL
mBL_50	chr8	113436732	113436732	T	A	exonic	CSMD3	555	347	0.3843	Missense_Mutation	p.K41N	Targerted Panel Gene	Pediatric mBL
mBL_50	chrX	41343297	41343297	C	G	exonic	DDX3X	99	183	0.6466	Missense_Mutation	p.H209D	Targerted Panel Gene	Pediatric mBL
mBL_50	chr11	6545002	6545002	C	G	exonic	DNHD1	385	324	0.4551	Missense_Mutation	p.H1355D	Targerted Panel Gene	Pediatric mBL
mBL_50	chr7	55201293	55201293	A	G	exonic	EGFR	357	268	0.4288	Missense_Mutation	p.I1018V	Targerted Panel Gene	Pediatric mBL
mBL_50	chr13	40666142	40666142	G	A	exonic	FOXO1	65	44	0.4037	Missense_Mutation	p.T24I	Targerted Panel Gene	Pediatric mBL
mBL_50	chr6	27867249	27867249	C	A	exonic	HIST1H1B	201	760	0.79	Missense_Mutation	p.G94V	Targerted Panel Gene	Pediatric mBL
mBL_50	chr1	228321572	228321572	G	T	exonic	OBSCN	353	337	0.487	Missense_Mutation	p.V5868L	Targerted Panel Gene	Pediatric mBL
mBL_50	chr1	233259267	233259267	C	A	exonic	PCNXL2	491	326	0.399	Missense_Mutation	p.A199S	Targerted Panel Gene	Pediatric mBL
mBL_50	chr9	8389351	8389351	T	G	exonic	PTPRD	534	216	0.288	Missense_Mutation	p.N1423H	Targerted Panel Gene	Pediatric mBL
mBL_50	chr19	38499241	38499241	A	G	exonic	RYR1	365	239	0.3957	Missense_Mutation	p.N2342S	Targerted Panel Gene	Pediatric mBL
mBL_50	chr19	1612367	1612367	A	T	exonic	TCF3	304	305	0.4992	Missense_Mutation	p.N551K	Targerted Panel Gene	Pediatric mBL
mBL_51	chr22	23287208	23287208	C	G	exonic	BCR	150	141	0.4829	Missense_Mutation	p.S819W	Targerted Panel Gene	Pediatric mBL
mBL_51	chr6	41935968	41935968	G	C	exonic	CCND3	215	174	0.4462	Missense_Mutation	p.P284R	Targerted Panel Gene	Pediatric mBL
mBL_51	chr12	113058333	113058333	C	G	exonic	DTX1	180	174	0.4915	Missense_Mutation	p.H47Q	Targerted Panel Gene	Pediatric mBL
mBL_51	chr13	40666210	40666210	C	T	exonic	FOXO1	48	44	0.4783	Missense_Mutation	p.M1I	Targerted Panel Gene	Pediatric mBL
mBL_51	chr8	127738431	127738431	C	T	exonic	MYC	204	152	0.427	Missense_Mutation	p.P72S	Targerted Panel Gene	Pediatric mBL
mBL_51	chr8	127738432	127738432	C	G	exonic	MYC	206	151	0.4206	Missense_Mutation	p.P72R	Targerted Panel Gene	Pediatric mBL
mBL_51	chr8	127738363	127738363	A	T	exonic	MYC	217	172	0.4422	Missense_Mutation	p.Q49L	Targerted Panel Gene	Pediatric mBL
mBL_51	chr3	47002269	47002269	G	A	exonic	NBEAL2	194	84	0.3022	Missense_Mutation	p.R1711H	Targerted Panel Gene	Pediatric mBL
mBL_51	chr3	47002273	47002273	C	A	exonic	NBEAL2	195	85	0.3036	Missense_Mutation	p.H1712Q	Targerted Panel Gene	Pediatric mBL
mBL_51	chr3	47002271	47002271	C	A	exonic	NBEAL2	195	84	0.3011	Missense_Mutation	p.H1712N	Targerted Panel Gene	Pediatric mBL
mBL_51	chr3	47002275	47002275	T	A	exonic	NBEAL2	197	84	0.2989	Missense_Mutation	p.F1713Y	Targerted Panel Gene	Pediatric mBL
mBL_51	chr3	47002274	47002274	T	A	exonic	NBEAL2	197	83	0.2964	Missense_Mutation	p.F1713I	Targerted Panel Gene	Pediatric mBL
mBL_51	chr19	11030875	11030875	C	G	exonic	SMARCA4	159	133	0.4539	Missense_Mutation	p.S1176R	Targerted Panel Gene	Pediatric mBL
mBL_51	chr17	7673802	7673802	C	T	exonic	TP53	257	203	0.4413	Missense_Mutation	p.R234H	Targerted Panel Gene	Pediatric mBL
mBL_51	chr17	7675995	7675995	G	A	exonic	TP53	189	136	0.4185	Missense_Mutation	p.T86M	Targerted Panel Gene	Pediatric mBL
mBL_51	chr17	65056512	65056512	G	A	exonic	GNA13	136	174	0.5577	Nonsense_Mutation	p.Q28X	Targerted Panel Gene	Pediatric mBL
mBL_52	chrX	71375165	71375165	A	C	splicing	TAF1	129	85	0.3953	Splice_Site	.	Targerted Panel Gene	Pediatric mBL
mBL_52	chr8	127738423	127738423	-	GCT	exonic	MYC	109	100	0.4785	In_Frame_Ins	p.E69delinsEL	Targerted Panel Gene	Pediatric mBL
mBL_52	chr11	108330396	108330396	T	G	exonic	ATM	237	95	0.2861	Missense_Mutation	p.V2497G	Targerted Panel Gene	Pediatric mBL
mBL_52	chr6	41935950	41935950	A	T	exonic	CCND3	141	105	0.4268	Missense_Mutation	p.I290K	Targerted Panel Gene	Pediatric mBL
mBL_52	chr2	124798113	124798113	G	C	exonic	CNTNAP5	109	97	0.4686	Missense_Mutation	p.E1003Q	Targerted Panel Gene	Pediatric mBL
mBL_52	chr8	25400970	25400970	A	C	exonic	DOCK5	141	93	0.3974	Missense_Mutation	p.K1610N	Targerted Panel Gene	Pediatric mBL
mBL_52	chr7	148811636	148811636	A	T	exonic	EZH2	131	105	0.4449	Missense_Mutation	p.Y646N	Targerted Panel Gene	Pediatric mBL
mBL_52	chr11	92800077	92800077	A	C	exonic	FAT3	284	127	0.3083	Missense_Mutation	p.E2355A	Targerted Panel Gene	Pediatric mBL
mBL_52	chr6	26198985	26198985	C	T	exonic	HIST1H2AD	121	133	0.5236	Missense_Mutation	p.A87T	Targerted Panel Gene	Pediatric mBL
mBL_52	chr7	111123174	111123174	A	C	exonic	LRRN3	211	154	0.4219	Missense_Mutation	p.K134N	Targerted Panel Gene	Pediatric mBL
mBL_52	chr9	8484147	8484147	G	A	exonic	PTPRD	141	117	0.4535	Missense_Mutation	p.P1129S	Targerted Panel Gene	Pediatric mBL
mBL_52	chr3	77607817	77607817	G	T	exonic	ROBO2	122	98	0.4455	Missense_Mutation	p.K459N	Targerted Panel Gene	Pediatric mBL
mBL_52	chr19	38446719	38446719	G	A	exonic	RYR1	59	72	0.5496	Missense_Mutation	p.V251M	Targerted Panel Gene	Pediatric mBL
mBL_52	chr17	7674918	7674918	A	C	exonic	TP53	42	305	0.879	Missense_Mutation	p.Y166D	Targerted Panel Gene	Pediatric mBL
mBL_52	chr8	127738393	127738393	C	T	exonic	MYC	89	106	0.5408	Missense_Mutation	p.A59V	Targerted Panel Gene	Pediatric mBL
mBL_53	chr16	72958825	72958827	CTT	-	exonic	ZFHX3	297	99	0.2487	In_Frame_Del	p.440_441del	Targerted Panel Gene	Pediatric mBL
mBL_53	chr2	214950937	214950937	A	C	exonic	ABCA12	303	412	0.5754	Missense_Mutation	p.L2265R	Targerted Panel Gene	Pediatric mBL
mBL_53	chr3	187725578	187725578	G	T	exonic	BCL6	570	520	0.4771	Missense_Mutation	p.A587D	Targerted Panel Gene	Pediatric mBL
mBL_53	chr12	111295345	111295345	A	C	exonic	CUX2	305	143	0.3192	Missense_Mutation	p.E191D	Targerted Panel Gene	Pediatric mBL
mBL_53	chrX	41345191	41345191	T	C	exonic	DDX3X	129	474	0.7822	Missense_Mutation	p.L346S	Targerted Panel Gene	Pediatric mBL
mBL_53	chr11	92800876	92800876	A	C	exonic	FAT3	538	172	0.2423	Missense_Mutation	p.Q2621H	Targerted Panel Gene	Pediatric mBL
mBL_53	chr6	26123577	26123577	G	A	exonic	HIST1H2BC	330	115	0.2584	Missense_Mutation	p.H110Y	Targerted Panel Gene	Pediatric mBL
mBL_53	chr1	235801017	235801017	C	T	exonic	LYST	424	380	0.4703	Missense_Mutation	p.E1265K	Targerted Panel Gene	Pediatric mBL
mBL_53	chr7	82822582	82822582	T	A	exonic	PCLO	677	203	0.2307	Missense_Mutation	p.T4902S	Targerted Panel Gene	Pediatric mBL
mBL_53	chr1	237548556	237548556	G	A	exonic	RYR2	445	199	0.309	Missense_Mutation	p.R1011Q	Targerted Panel Gene	Pediatric mBL
mBL_53	chr17	7675223	7675223	A	G	exonic	TP53	255	393	0.5973	Missense_Mutation	p.L91P	Targerted Panel Gene	Pediatric mBL
mBL_53	chr8	127738435	127738435	C	T	exonic	MYC	288	147	0.3379	Missense_Mutation	p.T73I	Targerted Panel Gene	Pediatric mBL
mBL_53	chr1	15219486	15219486	G	T	exonic	TMEM51	482	142	0.2276	Nonsense_Mutation	p.E169X	Targerted Panel Gene	Pediatric mBL
mBL_55	chr6	41936007	41936007	-	G	exonic	CCND3	261	148	0.3601	Frame_Shift_Ins	p.R271fs	Targerted Panel Gene	Pediatric mBL
mBL_55	chr1	23559236	23559236	-	G	exonic	ID3	301	191	0.3882	Frame_Shift_Ins	p.L64fs	Targerted Panel Gene	Pediatric mBL
mBL_55	chr1	26696448	26696448	-	CCGCCG	exonic	ARID1A	46	20	0.2899	In_Frame_Ins	p.N15delinsNPP	Targerted Panel Gene	Pediatric mBL
mBL_55	chr8	127738707	127738707	C	G	exonic	MYC	240	188	0.4393	Missense_Mutation	p.L164V	Targerted Panel Gene	Pediatric mBL
mBL_55	chr1	228243396	228243396	A	T	exonic	OBSCN	229	289	0.5579	Missense_Mutation	p.D1140V	Targerted Panel Gene	Pediatric mBL
mBL_55	chr10	87952221	87952221	T	C	exonic	PTEN	113	117	0.5087	Missense_Mutation	p.M372T	Targerted Panel Gene	Pediatric mBL
mBL_55	chr19	11023576	11023576	G	T	exonic	SMARCA4	222	194	0.4641	Missense_Mutation	p.R973L	Targerted Panel Gene	Pediatric mBL
mBL_56	chr17	7673782	7673782	T	C	exonic	TP53	57	815	0.9293	Missense_Mutation	p.R241G	Targerted Panel Gene	Pediatric mBL
mBL_56	chr16	3731210	3731210	C	T	exonic	CREBBP	320	249	0.4376	Nonsense_Mutation	p.W1718X	Targerted Panel Gene	Pediatric mBL
mBL_64	chr1	39254298	39254298	C	-	exonic	MACF1	85	62	0.4218	Frame_Shift_Del	p.P125fs	Targerted Panel Gene	Pediatric mBL
mBL_64	chr3	50257024	50257026	AAG	-	exonic	GNAI2	165	120	0.4211	In_Frame_Del	p.255_255del	Targerted Panel Gene	Pediatric mBL
mBL_64	chr12	49032935	49032970	GTTGCTGTTGTAGC	-	exonic	KMT2D	73	21	0.2234	In_Frame_Del	p.3912_3924del	Targerted Panel Gene	Pediatric mBL
mBL_64	chr1	23559260	23559260	G	A	exonic	ID3	148	119	0.4457	Missense_Mutation	p.P56L	Targerted Panel Gene	Pediatric mBL
mBL_64	chr1	23559270	23559270	C	T	exonic	ID3	125	126	0.502	Missense_Mutation	p.E53K	Targerted Panel Gene	Pediatric mBL
mBL_64	chr3	47409666	47409666	C	T	exonic	PTPN23	94	78	0.4535	Missense_Mutation	p.T654M	Targerted Panel Gene	Pediatric mBL
mBL_64	chr19	1612350	1612350	A	T	exonic	TCF3	12	213	0.9425	Missense_Mutation	p.V557E	Targerted Panel Gene	Pediatric mBL
mBL_64	chr8	127738435	127738435	C	T	exonic	MYC	128	105	0.4506	Missense_Mutation	p.T73I	Targerted Panel Gene	Pediatric mBL
mBL_64	chr1	26697069	26697069	C	A	exonic	ARID1A	134	114	0.4597	Nonsense_Mutation	p.Y222X	Targerted Panel Gene	Pediatric mBL
mBL_64	chr1	23559240	23559240	G	A	exonic	ID3	123	132	0.5176	Nonsense_Mutation	p.Q63X	Targerted Panel Gene	Pediatric mBL
mBL_65	chr1	23559125	23559125	A	T	splicing	ID3	261	265	0.5038	Splice_Site	.	Targerted Panel Gene	Pediatric mBL
mBL_65	chr22	22888077	22888077	G	-	exonic	IGLL5	259	240	0.479	Frame_Shift_Del	p.V8fs	Targerted Panel Gene	Pediatric mBL
mBL_65	chr10	71712749	71712749	T	C	exonic	CDH23	241	198	0.451	Missense_Mutation	p.F1102S	Targerted Panel Gene	Pediatric mBL
mBL_65	chr4	154259549	154259549	A	G	exonic	DCHS2	281	285	0.5026	Missense_Mutation	p.I1807T	Targerted Panel Gene	Pediatric mBL
mBL_65	chrX	41341535	41341535	C	T	exonic	DDX3X	514	378	0.4233	Missense_Mutation	p.A68V	Targerted Panel Gene	Pediatric mBL
mBL_65	chr1	23559261	23559261	G	A	exonic	ID3	307	253	0.4518	Missense_Mutation	p.P56S	Targerted Panel Gene	Pediatric mBL

mBL_65	chr8	127739008	127739008	G	A	exonic	MYC	290	328	0.5307	Missense_Mutation	p.S264N	Targerted Panel Gene	Pediatric mBL
mBL_65	chr19	11033317	11033317	C	T	exonic	SMARCA4	186	183	0.4946	Missense_Mutation	p.R1192C	Targerted Panel Gene	Pediatric mBL
mBL_65	chr10	46584586	46584586	G	A	exonic	SYT15	529	159	0.2311	Missense_Mutation	p.R306W	Targerted Panel Gene	Pediatric mBL
mBL_65	chr8	127738435	127738435	C	T	exonic	MYC	362	157	0.3025	Missense_Mutation	p.T73I	Targerted Panel Gene	Pediatric mBL
mBL_65	chr3	177050590	177050590	C	A	exonic	TBL1XR1	670	242	0.2651	Nonsense_Mutation	p.E150X	Targerted Panel Gene	Pediatric mBL
mBL_65	chr3	177064920	177064920	C	A	exonic	TBL1XR1	444	56	0.112	Nonsense_Mutation	p.G20X	Targerted Panel Gene	Pediatric mBL
mBL_65	chr1	15219486	15219486	G	T	exonic	TMEM51	466	202	0.3015	Nonsense_Mutation	p.E169X	Targerted Panel Gene	Pediatric mBL
mBL_66	chr11	6498252	6498252	G	-	exonic	DNHD1	143	161	0.5296	Frame_Shift_Del	p.D13fs	Targerted Panel Gene	Pediatric mBL
mBL_66	chr19	11035053	11035055	AGA	-	exonic	SMARCA4	82	39	0.3223	In_Frame_Del	p.1364_1365del	Targerted Panel Gene	Pediatric mBL
mBL_66	chr8	143872500	143872500	T	C	exonic	EPPK1	60	84	0.5833	Missense_Mutation	p.I252V	Targerted Panel Gene	Pediatric mBL
mBL_66	chr6	27838927	27838927	C	T	exonic	HIST1H2BN	44	24	0.3529	Missense_Mutation	p.T89I	Targerted Panel Gene	Pediatric mBL
mBL_66	chr8	127738431	127738431	C	T	exonic	MYC	162	78	0.325	Missense_Mutation	p.P72S	Targerted Panel Gene	Pediatric mBL
mBL_66	chr8	127738432	127738432	C	T	exonic	MYC	159	79	0.3292	Missense_Mutation	p.P72L	Targerted Panel Gene	Pediatric mBL
mBL_66	chr15	42693264	42693264	A	G	exonic	STARD9	208	163	0.4382	Missense_Mutation	p.R3896G	Targerted Panel Gene	Pediatric mBL
mBL_66	chr19	1612350	1612350	A	G	exonic	TCF3	147	83	0.3609	Missense_Mutation	p.V557A	Targerted Panel Gene	Pediatric mBL
mBL_66	chr17	65056515	65056515	G	A	exonic	GNA13	54	147	0.7313	Nonsense_Mutation	p.Q27X	Targerted Panel Gene	Pediatric mBL
mBL_68	chr1	26773628	26773628	A	-	exonic	ARID1A	236	82	0.2579	Frame_Shift_Del	p.P1305fs	Targerted Panel Gene	Pediatric mBL
mBL_68	chr6	26225464	26225464	-	TT	exonic	HIST1H3E	25	28	0.5283	Frame_Shift_Ins	p.L104fs	Targerted Panel Gene	Pediatric mBL
mBL_68	chr17	7674254	7674254	-	GTA	exonic	TP53	23	164	0.877	In_Frame_Ins	p.M198delinsYM	Targerted Panel Gene	Pediatric mBL
mBL_68	chr10	71807399	71807399	C	A	exonic	CDH23	70	69	0.4964	Missense_Mutation	p.F2767L	Targerted Panel Gene	Pediatric mBL
mBL_68	chr3	48575428	48575428	C	T	exonic	COL7A1	105	32	0.2336	Missense_Mutation	p.G2031S	Targerted Panel Gene	Pediatric mBL
mBL_68	chr2	169241124	169241124	C	G	exonic	LRP2	176	201	0.5332	Missense_Mutation	p.K1303N	Targerted Panel Gene	Pediatric mBL
mBL_68	chr8	127736618	127736618	A	G	exonic	MYC	211	159	0.4297	Missense_Mutation	p.N9D	Targerted Panel Gene	Pediatric mBL
mBL_68	chr4	133151823	133151823	C	A	exonic	PCDH10	94	80	0.4598	Missense_Mutation	p.N561K	Targerted Panel Gene	Pediatric mBL
mBL_68	chr19	38525492	38525492	G	A	exonic	RYR1	63	67	0.5154	Missense_Mutation	p.R3539H	Targerted Panel Gene	Pediatric mBL
mBL_68	chr6	41935993	41935993	G	A	exonic	CCND3	98	118	0.5463	Nonsense_Mutation	p.Q276X	Targerted Panel Gene	Pediatric mBL
mBL_68	chr1	23559129	23559129	G	A	exonic	ID3	127	49	0.2784	Nonsense_Mutation	p.Q100X	Targerted Panel Gene	Pediatric mBL
mBL_68	chr1	23559382	23559382	G	T	exonic	ID3	59	116	0.6629	Nonsense_Mutation	p.C15X	Targerted Panel Gene	Pediatric mBL
mBL_69	chr1	11124516	11124516	G	A	exonic	MTOR	368	226	0.3805	Missense_Mutation	p.S2215F	Targerted Panel Gene	Pediatric mBL
mBL_71	chr11	108284332	108284332	A	-	exonic	ATM	296	265	0.4724	Frame_Shift_Del	p.T1284fs	Targerted Panel Gene	Pediatric mBL
mBL_71	chr10	71799521	71799538	CACAATCATCCTGA	-	exonic	CDH23	101	48	0.3221	In_Frame_Del	p.2418_2424del	Targerted Panel Gene	Pediatric mBL
mBL_71	chr22	41178507	41178509	CAG	-	exonic	EP300	348	243	0.4112	In_Frame_Del	p.2266_2266del	Targerted Panel Gene	Pediatric mBL
mBL_71	chr3	50257024	50257026	AAG	-	exonic	GNAI2	283	166	0.3697	In_Frame_Del	p.255_255del	Targerted Panel Gene	Pediatric mBL
mBL_71	chr12	132062548	132062548	-	CAGCAGCAG	exonic	EP400	173	37	0.1659	In_Frame_Ins	p.Q2727delinsQQ(	Targerted Panel Gene	Pediatric mBL
mBL_71	chrX	41346590	41346590	G	A	exonic	DDX3X	36	281	0.8864	Missense_Mutation	p.R528H	Targerted Panel Gene	Pediatric mBL
mBL_71	chr13	40666137	40666137	G	A	exonic	FOXO1	44	35	0.443	Missense_Mutation	p.P26S	Targerted Panel Gene	Pediatric mBL
mBL_71	chr22	22888138	22888138	C	A	exonic	IGLL5	33	138	0.807	Missense_Mutation	p.L29M	Targerted Panel Gene	Pediatric mBL
mBL_71	chr8	127736619	127736619	A	G	exonic	MYC	314	217	0.4087	Missense_Mutation	p.N9S	Targerted Panel Gene	Pediatric mBL
mBL_71	chr8	127736620	127736620	C	G	exonic	MYC	317	217	0.4064	Missense_Mutation	p.N9K	Targerted Panel Gene	Pediatric mBL
mBL_71	chr8	109466656	109466656	T	C	exonic	PKHD1L1	267	234	0.4661	Missense_Mutation	p.I2831T	Targerted Panel Gene	Pediatric mBL
mBL_71	chr19	11023554	11023554	C	T	exonic	SMARCA4	197	128	0.3938	Missense_Mutation	p.R966W	Targerted Panel Gene	Pediatric mBL
mBL_71	chr19	1612296	1612296	A	T	exonic	TCF3	178	150	0.4573	Missense_Mutation	p.M575K	Targerted Panel Gene	Pediatric mBL
mBL_71	chr19	1612337	1612337	A	T	exonic	TCF3	196	158	0.4463	Missense_Mutation	p.D561E	Targerted Panel Gene	Pediatric mBL
mBL_71	chr17	7674220	7674220	C	T	exonic	TP53	46	289	0.8601	Missense_Mutation	p.R209Q	Targerted Panel Gene	Pediatric mBL
ABC-1	chr3	187745408	187745408	A	T	splicing	BCL6	479	74	0.1338	Splice_Site	.	Targerted Panel Gene	ABC-DLBCL
ABC-1	chr8	112829790	112829790	C	A	splicing	CSMD3	339	49	0.1263	Splice_Site	.	Targerted Panel Gene	ABC-DLBCL
ABC-1	chr1	1455863	1455863	A	G	exonic	ATAD3C	60	54	0.4737	Missense_Mutation	p.R171G	Targerted Panel Gene	ABC-DLBCL
ABC-1	chr19	6586139	6586139	A	G	exonic	CD70	466	149	0.2415	Missense_Mutation	p.S155P	Targerted Panel Gene	ABC-DLBCL
ABC-1	chr6	56630256	56630256	T	G	exonic	DST	554	95	0.1464	Missense_Mutation	p.S1391R	Targerted Panel Gene	ABC-DLBCL
ABC-1	chr4	186595793	186595793	T	G	exonic	FAT1	306	42	0.1207	Missense_Mutation	p.K4345T	Targerted Panel Gene	ABC-DLBCL
ABC-1	chr6	26217229	26217229	G	C	exonic	HIST1H2AE	61	8	0.1159	Missense_Mutation	p.Q85H	Targerted Panel Gene	ABC-DLBCL
ABC-1	chr4	54727537	54727537	G	A	exonic	KIT	568	92	0.1394	Missense_Mutation	p.S586N	Targerted Panel Gene	ABC-DLBCL
ABC-1	chr10	94171257	94171257	G	T	exonic	PLCE1	458	76	0.1423	Missense_Mutation	p.D524Y	Targerted Panel Gene	ABC-DLBCL
ABC-1	chr3	77098044	77098044	C	T	exonic	ROBO2	408	58	0.1245	Missense_Mutation	p.P31L	Targerted Panel Gene	ABC-DLBCL
ABC-1	chr15	33859662	33859662	A	G	exonic	RYR3	284	477	0.6268	Missense_Mutation	p.M4744V	Targerted Panel Gene	ABC-DLBCL
ABC-1	chr16	11254996	11254996	C	T	exonic	SOCS1	218	27	0.1102	Missense_Mutation	p.M161I	Targerted Panel Gene	ABC-DLBCL
ABC-1	chr15	44711548	44711548	T	G	exonic	B2M	214	56	0.2066	Missense_Mutation	p.M1R	Targerted Panel Gene	ABC-DLBCL
ABC-10	chr6	37171124	37171124	G	C	splicing	PIM1	677	175	0.2052	Splice_Site	.	Targerted Panel Gene	ABC-DLBCL
ABC-10	chr18	63318531	63318531	G	A	exonic	BCL2	676	74	0.0985	Missense_Mutation	p.P46S	Targerted Panel Gene	ABC-DLBCL
ABC-10	chr5	71510981	71510981	G	A	exonic	BDP1	1324	290	0.1795	Missense_Mutation	p.E1297K	Targerted Panel Gene	ABC-DLBCL
ABC-10	chr17	63929439	63929439	A	G	exonic	CD79B	242	146	0.3763	Missense_Mutation	p.Y197H	Targerted Panel Gene	ABC-DLBCL
ABC-10	chr17	63929828	63929828	A	T	exonic	CD79B	353	69	0.1635	Missense_Mutation	p.I165N	Targerted Panel Gene	ABC-DLBCL
ABC-10	chr18	67512238	67512238	A	G	exonic	DSEL	1953	306	0.1355	Missense_Mutation	p.W801R	Targerted Panel Gene	ABC-DLBCL
ABC-10	chr6	56552538	56552538	C	G	exonic	DST	1194	308	0.2048	Missense_Mutation	p.L3299F	Targerted Panel Gene	ABC-DLBCL
ABC-10	chr11	92352165	92352165	T	C	exonic	FAT3	759	179	0.1908	Missense_Mutation	p.L18P	Targerted Panel Gene	ABC-DLBCL
ABC-10	chr4	78450270	78450270	C	T	exonic	FRAS1	815	218	0.211	Missense_Mutation	p.H2132Y	Targerted Panel Gene	ABC-DLBCL
ABC-10	chr6	27892845	27892845	G	A	exonic	HIST1H2AM	317	42	0.117	Missense_Mutation	p.T102I	Targerted Panel Gene	ABC-DLBCL
ABC-10	chr22	22888259	22888259	G	C	exonic	IGLL5	142	46	0.2434	Missense_Mutation	p.G34R	Targerted Panel Gene	ABC-DLBCL
ABC-10	chr22	22893792	22893792	G	A	exonic	IGLL5	526	112	0.1753	Missense_Mutation	p.G100E	Targerted Panel Gene	ABC-DLBCL
ABC-10	chr22	22888059	22888059	A	C	exonic	IGLL5	350	79	0.1841	Missense_Mutation	p.R2S	Targerted Panel Gene	ABC-DLBCL
ABC-10	chr6	393360	393360	C	G	exonic	IRF4	343	58	0.1446	Missense_Mutation	p.L70V	Targerted Panel Gene	ABC-DLBCL
ABC-10	chr6	37171428	37171428	C	G	exonic	PIM1	387	191	0.3304	Missense_Mutation	p.L182V	Targerted Panel Gene	ABC-DLBCL
ABC-10	chr6	37170514	37170514	G	A	exonic	PIM1	201	27	0.1184	Missense_Mutation	p.G71D	Targerted Panel Gene	ABC-DLBCL
ABC-10	chr6	106088449	106088449	G	C	exonic	PRDM1	669	200	0.2301	Missense_Mutation	p.E97D	Targerted Panel Gene	ABC-DLBCL
ABC-10	chr1	237569280	237569280	G	A	exonic	RYR2	856	108	0.112	Missense_Mutation	p.G1187S	Targerted Panel Gene	ABC-DLBCL
ABC-10	chr16	11254858	11254858	G	T	exonic	SOCS1	627	149	0.192	Missense_Mutation	p.F207L	Targerted Panel Gene	ABC-DLBCL
ABC-10	chr3	177034264	177034264	T	C	exonic	TBL1XR1	939	258	0.2155	Missense_Mutation	p.Y395C	Targerted Panel Gene	ABC-DLBCL
ABC-10	chr4	84740389	84740389	G	T	exonic	WDFY3	847	248	0.2265	Missense_Mutation	p.L2088M	Targerted Panel Gene	ABC-DLBCL
ABC-10	chr2	167247230	167247230	A	C	exonic	XIRP2	1063	219	0.1708	Missense_Mutation	p.K1946N	Targerted Panel Gene	ABC-DLBCL
ABC-10	chr19	22088126	22088126	T	C	exonic	ZNF257	165	17	0.0934	Missense_Mutation	p.C50R	Targerted Panel Gene	ABC-DLBCL
ABC-10	chr19	22088127	22088127	G	A	exonic	ZNF257	164	17	0.0939	Missense_Mutation	p.C50Y	Targerted Panel Gene	ABC-DLBCL
ABC-10	chr19	22088132	22088132	G	C	exonic	ZNF257	162	16	0.0894	Missense_Mutation	p.G52R	Targerted Panel Gene	ABC-DLBCL

ABC-10	chr19	22088133	22088133	G	A	exonic	ZNF257	163	16	0.0894	Missense_Mutation	p.G52E	Targerted Panel Gene	ABC-DLBCL
ABC-10	chr8	112947872	112947872	C	A	exonic	CSMD3	734	153	0.1725	Nonsense_Mutation	p.E436X	Targerted Panel Gene	ABC-DLBCL
ABC-10	chr12	40238038	40238038	C	A	exonic	LRRK2	714	192	0.2115	Nonsense_Mutation	p.S169X	Targerted Panel Gene	ABC-DLBCL
ABC-10	chr6	37171263	37171263	C	T	exonic	PIM1	669	153	0.1857	Nonsense_Mutation	p.Q127X	Targerted Panel Gene	ABC-DLBCL
ABC-10	chr3	38141150	38141150	T	C	exonic	MYD88	510	253	0.3303	Nonstop_Mutation	p.L265P	Targerted Panel Gene	ABC-DLBCL
ABC-11	chr3	177038435	177038435	C	A	splicing	TBL1XR1	419	85	0.1687	Splice_Site	.	Targerted Panel Gene	ABC-DLBCL
ABC-11	chr5	71464117	71464120	AGTA	-	exonic	BDP1	164	105	0.3903	Frame_Shift_Del	p.E220fs	Targerted Panel Gene	ABC-DLBCL
ABC-11	chr13	40666158	40666158	G	A	exonic	FOXO1	45	9	0.1667	Missense_Mutation	p.R19W	Targerted Panel Gene	ABC-DLBCL
ABC-11	chr7	82822486	82822486	G	A	exonic	PCLO	977	125	0.1133	Missense_Mutation	p.R4934C	Targerted Panel Gene	ABC-DLBCL
ABC-12	chr15	44711583	44711584	CT	-	exonic	B2M	78	70	0.473	Frame_Shift_Del	p.L13fs	Targerted Panel Gene	ABC-DLBCL
ABC-12	chr18	63318399	63318399	G	A	exonic	BCL2	99	30	0.2326	Missense_Mutation	p.P90S	Targerted Panel Gene	ABC-DLBCL
ABC-12	chr1	203305634	203305634	C	T	exonic	BTG2	99	45	0.3103	Missense_Mutation	p.L10F	Targerted Panel Gene	ABC-DLBCL
ABC-12	chr19	6586258	6586258	C	A	exonic	CD70	132	197	0.5988	Missense_Mutation	p.C115F	Targerted Panel Gene	ABC-DLBCL
ABC-12	chr16	10923269	10923269	T	G	exonic	CIITA	113	50	0.3067	Missense_Mutation	p.L1121R	Targerted Panel Gene	ABC-DLBCL
ABC-12	chr16	10906801	10906801	T	C	exonic	CIITA	198	70	0.2612	Missense_Mutation	p.W438R	Targerted Panel Gene	ABC-DLBCL
ABC-12	chr3	7461588	7461588	G	T	exonic	GRM7	247	93	0.2735	Missense_Mutation	p.A461S	Targerted Panel Gene	ABC-DLBCL
ABC-12	chr1	21831018	21831018	G	A	exonic	HSPG2	63	23	0.2674	Missense_Mutation	p.R3879C	Targerted Panel Gene	ABC-DLBCL
ABC-12	chr22	22895482	22895482	G	A	exonic	IGLL5	71	27	0.2755	Missense_Mutation	p.A145T	Targerted Panel Gene	ABC-DLBCL
ABC-12	chr22	22893753	22893753	G	A	exonic	IGLL5	176	92	0.3433	Missense_Mutation	p.G87E	Targerted Panel Gene	ABC-DLBCL
ABC-12	chr22	22895441	22895441	A	G	exonic	IGLL5	71	34	0.3238	Missense_Mutation	p.K131R	Targerted Panel Gene	ABC-DLBCL
ABC-12	chr22	22888191	22888191	A	T	exonic	IGLL5	102	35	0.2555	Missense_Mutation	p.K11I	Targerted Panel Gene	ABC-DLBCL
ABC-12	chr3	78600133	78600133	C	T	exonic	ROBO1	499	125	0.2	Missense_Mutation	p.D1641N	Targerted Panel Gene	ABC-DLBCL
ABC-12	chr12	57105579	57105579	A	T	exonic	STAT6	181	67	0.2702	Missense_Mutation	p.I234N	Targerted Panel Gene	ABC-DLBCL
ABC-12	chr1	216200073	216200073	G	A	exonic	USH2A	240	216	0.4737	Missense_Mutation	p.S1122F	Targerted Panel Gene	ABC-DLBCL
ABC-12	chr8	36935820	36935820	C	A	exonic	KCNU1	659	177	0.2117	Nonsense_Mutation	p.S1117X	Targerted Panel Gene	ABC-DLBCL
ABC-13	chr11	6545432	6545432	A	-	exonic	DNHD1	212	166	0.4392	Frame_Shift_Del	p.H1498fs	Targerted Panel Gene	ABC-DLBCL
ABC-13	chr1	85270791	85270791	C	T	exonic	BCL10	518	130	0.2003	Missense_Mutation	p.R58Q	Targerted Panel Gene	ABC-DLBCL
ABC-13	chr11	117431596	117431596	G	T	exonic	DSCAML1	93	95	0.5053	Missense_Mutation	p.S1831Y	Targerted Panel Gene	ABC-DLBCL
ABC-13	chr13	24455025	24455025	A	G	exonic	PARP4	119	109	0.4781	Missense_Mutation	p.F917S	Targerted Panel Gene	ABC-DLBCL
ABC-13	chr6	56494150	56494150	C	A	exonic	DST	195	167	0.4613	Nonsense_Mutation	p.E4633X	Targerted Panel Gene	ABC-DLBCL
ABC-13	chr1	216073136	216073136	C	A	exonic	USH2A	225	173	0.4336	Nonsense_Mutation	p.E1913X	Targerted Panel Gene	ABC-DLBCL
ABC-14	chr12	49033069	49033070	CT	-	exonic	KMT2D	115	65	0.3611	Frame_Shift_Del	p.S3879fs	Targerted Panel Gene	ABC-DLBCL
ABC-14	chr7	2937948	2937948	C	A	exonic	CARD11	145	101	0.4089	Missense_Mutation	p.V368F	Targerted Panel Gene	ABC-DLBCL
ABC-14	chr1	185928632	185928632	G	C	exonic	HMCN1	337	284	0.4566	Missense_Mutation	p.G506A	Targerted Panel Gene	ABC-DLBCL
ABC-14	chr22	22888147	22888147	G	A	exonic	IGLL5	144	107	0.4263	Missense_Mutation	p.A32T	Targerted Panel Gene	ABC-DLBCL
ABC-14	chr22	22888067	22888067	C	T	exonic	IGLL5	186	125	0.4019	Missense_Mutation	p.T5I	Targerted Panel Gene	ABC-DLBCL
ABC-14	chr22	22888252	22888252	T	C	exonic	IGLL5	98	57	0.3677	Missense_Mutation	p.W67R	Targerted Panel Gene	ABC-DLBCL
ABC-14	chr3	183492634	183492634	C	T	exonic	KLHL6	394	535	0.5753	Missense_Mutation	p.G475E	Targerted Panel Gene	ABC-DLBCL
ABC-14	chr12	49026430	49026430	C	T	exonic	KMT2D	351	220	0.3846	Missense_Mutation	p.R5179H	Targerted Panel Gene	ABC-DLBCL
ABC-14	chr7	30436013	30436013	G	A	exonic	NOD1	256	163	0.389	Missense_Mutation	p.S869F	Targerted Panel Gene	ABC-DLBCL
ABC-14	chr9	8486156	8486156	C	A	exonic	PTPRD	537	488	0.4761	Missense_Mutation	p.K887N	Targerted Panel Gene	ABC-DLBCL
ABC-14	chr4	84860555	84860555	G	T	exonic	WDFY3	331	221	0.4004	Missense_Mutation	p.Q13K	Targerted Panel Gene	ABC-DLBCL
ABC-14	chr13	48362847	48362847	C	T	exonic	RB1	144	277	0.658	Nonsense_Mutation	p.R251X	Targerted Panel Gene	ABC-DLBCL
ABC-15	chr19	38464708	38464708	A	-	exonic	RYR1	90	66	0.4204	Frame_Shift_Del	p.T952fs	Targerted Panel Gene	ABC-DLBCL
ABC-15	chr1	155347882	155347882	G	A	exonic	ASH1L	160	127	0.4425	Missense_Mutation	p.P2526L	Targerted Panel Gene	ABC-DLBCL
ABC-15	chr15	44711549	44711549	G	C	exonic	B2M	103	15	0.1271	Missense_Mutation	p.M1I	Targerted Panel Gene	ABC-DLBCL
ABC-15	chr15	33742422	33742422	A	G	exonic	RYR3	122	105	0.4605	Missense_Mutation	p.H2626R	Targerted Panel Gene	ABC-DLBCL
ABC-15	chr14	102875678	102875678	C	T	exonic	TRAF3	72	81	0.5294	Missense_Mutation	p.R118W	Targerted Panel Gene	ABC-DLBCL
ABC-16	chr15	42684868	42684869	AC	-	exonic	STARD9	348	348	0.4993	Frame_Shift_Del	p.D1097fs	Targerted Panel Gene	ABC-DLBCL
ABC-16	chr6	44262654	44262654	-	G	exonic	NFKBIE	185	78	0.2966	Frame_Shift_Ins	p.H264fs	Targerted Panel Gene	ABC-DLBCL
ABC-16	chr17	7676152	7676152	-	G	exonic	TP53	165	107	0.3891	Frame_Shift_Ins	p.V34fs	Targerted Panel Gene	ABC-DLBCL
ABC-16	chr13	35593346	35593346	T	C	exonic	NBEA	443	58	0.1153	Missense_Mutation	p.F2399L	Targerted Panel Gene	ABC-DLBCL
ABC-16	chr1	119919428	119919428	G	A	exonic	NOTCH2	167	140	0.456	Missense_Mutation	p.R1889W	Targerted Panel Gene	ABC-DLBCL
ABC-16	chr2	51028173	51028173	G	A	exonic	NRXN1	189	24	0.1127	Missense_Mutation	p.P34L	Targerted Panel Gene	ABC-DLBCL
ABC-16	chr6	52082521	52082521	T	C	exonic	PKHD1	482	404	0.456	Missense_Mutation	p.Y51C	Targerted Panel Gene	ABC-DLBCL
ABC-16	chr19	22088126	22088126	T	C	exonic	ZNF257	69	7	0.0921	Missense_Mutation	p.C50R	Targerted Panel Gene	ABC-DLBCL
ABC-16	chr19	22088127	22088127	G	A	exonic	ZNF257	67	7	0.0933	Missense_Mutation	p.C50Y	Targerted Panel Gene	ABC-DLBCL
ABC-17	chr6	137878564	137878564	G	-	exonic	TNFAIP3	355	268	0.4302	Frame_Shift_Del	p.M373fs	Targerted Panel Gene	ABC-DLBCL
ABC-17	chr8	39788676	39788676	G	T	exonic	ADAM2	291	191	0.3954	Missense_Mutation	p.T212K	Targerted Panel Gene	ABC-DLBCL
ABC-17	chr8	39788677	39788677	T	G	exonic	ADAM2	290	189	0.3938	Missense_Mutation	p.T212P	Targerted Panel Gene	ABC-DLBCL
ABC-17	chrX	40074463	40074463	G	A	exonic	BCOR	311	176	0.3607	Missense_Mutation	p.P295S	Targerted Panel Gene	ABC-DLBCL
ABC-17	chr6	56611574	56611574	G	A	exonic	DST	223	196	0.4678	Missense_Mutation	p.T1661I	Targerted Panel Gene	ABC-DLBCL
ABC-17	chr12	113096878	113096878	T	C	exonic	DTX1	237	130	0.3542	Missense_Mutation	p.L601P	Targerted Panel Gene	ABC-DLBCL
ABC-17	chr19	39870304	39870304	C	T	exonic	FCGBP	125	145	0.537	Missense_Mutation	p.C5155Y	Targerted Panel Gene	ABC-DLBCL
ABC-17	chr6	26156757	26156757	G	T	exonic	HIST1H1E	295	149	0.3356	Missense_Mutation	p.A123S	Targerted Panel Gene	ABC-DLBCL
ABC-17	chr22	22888207	22888207	C	T	exonic	IGLL5	91	72	0.4417	Missense_Mutation	p.P52S	Targerted Panel Gene	ABC-DLBCL
ABC-17	chr22	22888182	22888182	T	C	exonic	IGLL5	111	78	0.4127	Missense_Mutation	p.L8S	Targerted Panel Gene	ABC-DLBCL
ABC-17	chr22	22888239	22888239	C	G	exonic	IGLL5	75	48	0.3902	Missense_Mutation	p.A27G	Targerted Panel Gene	ABC-DLBCL
ABC-17	chr12	25245347	25245347	C	T	exonic	KRAS	193	118	0.3782	Missense_Mutation	p.G13D	Targerted Panel Gene	ABC-DLBCL
ABC-17	chr2	140485418	140485418	T	C	exonic	LRP1B	387	254	0.3963	Missense_Mutation	p.N3117S	Targerted Panel Gene	ABC-DLBCL
ABC-17	chr6	89719141	89719141	C	T	exonic	MDN1	315	171	0.3519	Missense_Mutation	p.V2018I	Targerted Panel Gene	ABC-DLBCL
ABC-17	chr7	103593737	103593737	C	T	exonic	RELN	408	257	0.3865	Missense_Mutation	p.R1286Q	Targerted Panel Gene	ABC-DLBCL
ABC-17	chr19	38528675	38528675	C	T	exonic	RYR1	154	149	0.4917	Missense_Mutation	p.R3672C	Targerted Panel Gene	ABC-DLBCL
ABC-17	chr1	237718479	237718479	A	T	exonic	RYR2	229	139	0.3777	Missense_Mutation	p.E3504D	Targerted Panel Gene	ABC-DLBCL
ABC-17	chr3	177038161	177038161	A	T	exonic	TBL1XR1	333	461	0.5806	Missense_Mutation	p.N353K	Targerted Panel Gene	ABC-DLBCL
ABC-17	chr6	56463687	56463687	G	A	exonic	DST	247	145	0.3699	Nonsense_Mutation	p.R5470X	Targerted Panel Gene	ABC-DLBCL
ABC-17	chr1	119915524	119915524	G	A	exonic	NOTCH2	320	222	0.4096	Nonsense_Mutation	p.R2400X	Targerted Panel Gene	ABC-DLBCL
ABC-17	chr1	15928434	15928434	C	T	exonic	SPEN	277	202	0.4208	Nonsense_Mutation	p.R732X	Targerted Panel Gene	ABC-DLBCL
ABC-17	chr3	38141150	38141150	T	C	exonic	MYD88	231	177	0.4338	Nonstop_Mutation	p.L265P	Targerted Panel Gene	ABC-DLBCL
ABC-18	chr17	7670685	7670685	G	-	exonic	TP53	147	80	0.3524	Frame_Shift_Del	p.R303fs	Targerted Panel Gene	ABC-DLBCL
ABC-18	chr11	108259048	108259048	T	A	exonic	ATM	3402	437	0.1138	Missense_Mutation	p.N813K	Targerted Panel Gene	ABC-DLBCL
ABC-18	chrX	32441226	32441226	G	A	exonic	DMD	89	249	0.7367	Missense_Mutation	p.T1169I	Targerted Panel Gene	ABC-DLBCL

ABC-18	chr4	186618284	186618284	G	T	exonic	FAT1	504	241	0.3235	Missense_Mutation	p.H2768N	Targerted Panel Gene	ABC-DLBCL
ABC-18	chr3	78635822	78635822	T	G	exonic	ROBO1	457	245	0.349	Missense_Mutation	p.E1108D	Targerted Panel Gene	ABC-DLBCL
ABC-18	chr17	7675088	7675088	C	T	exonic	TP53	234	159	0.4025	Missense_Mutation	p.R136H	Targerted Panel Gene	ABC-DLBCL
ABC-19	chr6	137874947	137874947	-	A	exonic	TNFAIP3	214	277	0.5642	Frame_Shift_Ins	p.T133fs	Targerted Panel Gene	ABC-DLBCL
ABC-19	chr8	3096974	3096974	G	A	exonic	CSMD1	411	143	0.2581	Missense_Mutation	p.P2338L	Targerted Panel Gene	ABC-DLBCL
ABC-19	chr13	72771818	72771818	T	A	exonic	DIS3	290	178	0.3803	Missense_Mutation	p.T528S	Targerted Panel Gene	ABC-DLBCL
ABC-19	chr22	22888064	22888064	A	G	exonic	IGLL5	206	91	0.3064	Missense_Mutation	p.K4R	Targerted Panel Gene	ABC-DLBCL
ABC-19	chr2	140701745	140701745	T	C	exonic	LRP1B	238	211	0.4699	Missense_Mutation	p.I2135V	Targerted Panel Gene	ABC-DLBCL
ABC-19	chr16	88726410	88726410	T	C	exonic	PIEZO1	96	92	0.4894	Missense_Mutation	p.E1281G	Targerted Panel Gene	ABC-DLBCL
ABC-19	chr19	621284	621284	C	T	exonic	POLRMT	68	75	0.5245	Missense_Mutation	p.R805H	Targerted Panel Gene	ABC-DLBCL
ABC-19	chr3	177047364	177047364	C	A	exonic	TBL1XR1	455	114	0.2004	Missense_Mutation	p.G267V	Targerted Panel Gene	ABC-DLBCL
ABC-19	chr15	44715675	44715675	T	A	exonic	B2M	188	157	0.4551	Nonsense_Mutation	p.L107X	Targerted Panel Gene	ABC-DLBCL
ABC-19	chr15	44715649	44715649	T	A	exonic	B2M	230	177	0.4349	Nonsense_Mutation	p.Y98X	Targerted Panel Gene	ABC-DLBCL
ABC-19	chr4	105234687	105234687	A	-	exonic	TET2	556	204	0.2684	Nonsense_Mutation	p.I249X	Targerted Panel Gene	ABC-DLBCL
ABC-19	chr4	105243610	105243610	T	A	exonic	TET2	211	71	0.2518	Nonsense_Mutation	p.L1212X	Targerted Panel Gene	ABC-DLBCL
ABC-19	chr6	75258804	75258804	G	A	exonic	TMEM30A	211	268	0.5595	Nonsense_Mutation	p.R290X	Targerted Panel Gene	ABC-DLBCL
ABC-2	chr3	187745408	187745408	A	T	splicing	BCL6	309	206	0.4	Splice_Site	.	Targerted Panel Gene	ABC-DLBCL
ABC-2	chr6	37170658	37170658	G	T	splicing	PIM1	91	64	0.4129	Splice_Site	.	Targerted Panel Gene	ABC-DLBCL
ABC-2	chr2	96144982	96144996	CGGCAGTGGGCCC	-	exonic	DUSP2	40	17	0.2982	In_Frame_Del	p.120_125del	Targerted Panel Gene	ABC-DLBCL
ABC-2	chr3	50252100	50252100	-	TGC	exonic	GNAI2	145	88	0.3761	In_Frame_Ins	p.G24delinsGA	Targerted Panel Gene	ABC-DLBCL
ABC-2	chr1	203307161	203307161	G	A	exonic	BTG2	187	278	0.5978	Missense_Mutation	p.C67Y	Targerted Panel Gene	ABC-DLBCL
ABC-2	chr1	203305676	203305676	C	G	exonic	BTG2	217	81	0.2709	Missense_Mutation	p.L24V	Targerted Panel Gene	ABC-DLBCL
ABC-2	chr17	63929439	63929439	A	T	exonic	CD79B	145	72	0.3318	Missense_Mutation	p.Y197N	Targerted Panel Gene	ABC-DLBCL
ABC-2	chr17	63930149	63930149	T	C	exonic	CD79B	260	204	0.4397	Missense_Mutation	p.I120V	Targerted Panel Gene	ABC-DLBCL
ABC-2	chr17	63930121	63930121	T	C	exonic	CD79B	214	147	0.4072	Missense_Mutation	p.N129S	Targerted Panel Gene	ABC-DLBCL
ABC-2	chr17	63930119	63930119	T	A	exonic	CD79B	212	145	0.4062	Missense_Mutation	p.T130S	Targerted Panel Gene	ABC-DLBCL
ABC-2	chr17	63929852	63929852	G	C	exonic	CD79B	149	89	0.3739	Missense_Mutation	p.T157R	Targerted Panel Gene	ABC-DLBCL
ABC-2	chr19	11214288	11214288	G	A	exonic	DOCK6	121	123	0.5041	Missense_Mutation	p.A1442V	Targerted Panel Gene	ABC-DLBCL
ABC-2	chr12	113058364	113058364	G	C	exonic	DTX1	184	96	0.3416	Missense_Mutation	p.G58R	Targerted Panel Gene	ABC-DLBCL
ABC-2	chr2	96144817	96144817	G	T	exonic	DUSP2	80	47	0.3701	Missense_Mutation	p.P152T	Targerted Panel Gene	ABC-DLBCL
ABC-2	chr4	125487417	125487417	G	A	exonic	FAT4	349	215	0.3812	Missense_Mutation	p.V4297I	Targerted Panel Gene	ABC-DLBCL
ABC-2	chr12	49044262	49044262	C	T	exonic	KMT2D	221	138	0.3844	Missense_Mutation	p.R1709H	Targerted Panel Gene	ABC-DLBCL
ABC-2	chr7	111123591	111123591	T	G	exonic	LRRN3	529	344	0.394	Missense_Mutation	p.N273K	Targerted Panel Gene	ABC-DLBCL
ABC-2	chr1	228367031	228367031	C	T	exonic	OBSCN	263	365	0.5812	Missense_Mutation	p.A7452V	Targerted Panel Gene	ABC-DLBCL
ABC-2	chr6	37170657	37170657	G	A	exonic	PIM1	91	64	0.4129	Missense_Mutation	p.G28S	Targerted Panel Gene	ABC-DLBCL
ABC-2	chr6	37171321	37171321	G	A	exonic	PIM1	238	176	0.4251	Missense_Mutation	p.S146N	Targerted Panel Gene	ABC-DLBCL
ABC-2	chr6	37170593	37170593	C	G	exonic	PIM1	117	74	0.3874	Missense_Mutation	p.I6M	Targerted Panel Gene	ABC-DLBCL
ABC-2	chr6	37171287	37171287	G	T	exonic	PIM1	291	176	0.3753	Nonsense_Mutation	p.E135X	Targerted Panel Gene	ABC-DLBCL
ABC-2	chr9	12698601	12698601	C	T	exonic	TYRP1	422	293	0.4098	Nonsense_Mutation	p.R287X	Targerted Panel Gene	ABC-DLBCL
ABC-20	chr6	156778889	156778889	-	GGA	exonic	ARID1B	133	57	0.2923	In_Frame_Ins	p.S320delinsSG	Targerted Panel Gene	ABC-DLBCL
ABC-20	chr19	1044712	1044712	C	T	exonic	ABCA7	160	141	0.4684	Missense_Mutation	p.H395Y	Targerted Panel Gene	ABC-DLBCL
ABC-20	chr7	2937177	2937177	C	T	exonic	CARD11	681	73	0.0967	Missense_Mutation	p.D401N	Targerted Panel Gene	ABC-DLBCL
ABC-20	chr6	89754141	89754141	C	A	exonic	MDN1	320	308	0.4897	Missense_Mutation	p.R969L	Targerted Panel Gene	ABC-DLBCL
ABC-20	chr16	11255472	11255472	C	A	exonic	SOCS1	230	27	0.1051	Missense_Mutation	p.A3S	Targerted Panel Gene	ABC-DLBCL
ABC-20	chr16	85921175	85921175	C	T	exonic	IRF8	644	112	0.1481	Nonsense_Mutation	p.Q392X	Targerted Panel Gene	ABC-DLBCL
ABC-20	chr9	136496409	136496409	G	A	exonic	NOTCH1	351	43	0.1091	Nonsense_Mutation	p.Q2444X	Targerted Panel Gene	ABC-DLBCL
ABC-20	chr4	105275394	105275394	T	G	exonic	TET2	1410	173	0.1093	Nonsense_Mutation	p.Y1628X	Targerted Panel Gene	ABC-DLBCL
ABC-21	chr2	214974015	214974015	T	G	exonic	ABCA12	326	65	0.1662	Missense_Mutation	p.E1832D	Targerted Panel Gene	ABC-DLBCL
ABC-21	chr1	186087589	186087589	T	A	exonic	HMCN1	292	58	0.1657	Missense_Mutation	p.S3103T	Targerted Panel Gene	ABC-DLBCL
ABC-21	chr6	89718798	89718798	T	C	exonic	MDN1	130	86	0.3981	Missense_Mutation	p.D2097G	Targerted Panel Gene	ABC-DLBCL
ABC-21	chr1	233198968	233198968	G	A	exonic	PCNXL2	54	70	0.5645	Missense_Mutation	p.L1013F	Targerted Panel Gene	ABC-DLBCL
ABC-21	chr3	177038393	177038393	C	G	exonic	TBL1XR1	273	29	0.096	Missense_Mutation	p.A323P	Targerted Panel Gene	ABC-DLBCL
ABC-21	chr7	148809375	148809375	G	C	exonic	EZH2	233	26	0.1004	Missense_Mutation	p.A682G	Targerted Panel Gene	ABC-DLBCL
ABC-22	chr16	2317403	2317403	C	T	exonic	ABCA3	73	18	0.1978	Missense_Mutation	p.V331M	Targerted Panel Gene	ABC-DLBCL
ABC-22	chr1	203305612	203305612	C	G	exonic	BTG2	155	40	0.2051	Missense_Mutation	p.S2R	Targerted Panel Gene	ABC-DLBCL
ABC-22	chr1	203305727	203305727	G	A	exonic	BTG2	90	18	0.1667	Missense_Mutation	p.A41T	Targerted Panel Gene	ABC-DLBCL
ABC-22	chr17	63929438	63929438	T	G	exonic	CD79B	118	34	0.2237	Missense_Mutation	p.Y197S	Targerted Panel Gene	ABC-DLBCL
ABC-22	chr10	71813280	71813280	C	T	exonic	CDH23	107	97	0.4755	Missense_Mutation	p.R3224W	Targerted Panel Gene	ABC-DLBCL
ABC-22	chr5	79733597	79733597	A	G	exonic	CMYA5	662	147	0.1817	Missense_Mutation	p.Q1611R	Targerted Panel Gene	ABC-DLBCL
ABC-22	chr8	3307803	3307803	A	T	exonic	CSMD1	288	72	0.2	Missense_Mutation	p.I1281N	Targerted Panel Gene	ABC-DLBCL
ABC-22	chr8	112954704	112954704	T	C	exonic	CSMD3	189	147	0.4375	Missense_Mutation	p.N427S	Targerted Panel Gene	ABC-DLBCL
ABC-22	chr4	154235245	154235245	G	A	exonic	DCHS2	242	247	0.5041	Missense_Mutation	p.P2681L	Targerted Panel Gene	ABC-DLBCL
ABC-22	chr7	154794144	154794144	G	A	exonic	DPP6	98	31	0.2403	Missense_Mutation	p.R401Q	Targerted Panel Gene	ABC-DLBCL
ABC-22	chr12	113058435	113058435	G	C	exonic	DTX1	152	49	0.2438	Missense_Mutation	p.Q81H	Targerted Panel Gene	ABC-DLBCL
ABC-22	chr12	113058436	113058436	T	G	exonic	DTX1	156	41	0.2081	Missense_Mutation	p.F82V	Targerted Panel Gene	ABC-DLBCL
ABC-22	chr12	113058430	113058430	C	A	exonic	DTX1	166	43	0.2057	Missense_Mutation	p.H80N	Targerted Panel Gene	ABC-DLBCL
ABC-22	chr12	113058269	113058269	T	C	exonic	DTX1	240	48	0.1667	Missense_Mutation	p.V26A	Targerted Panel Gene	ABC-DLBCL
ABC-22	chr12	113058350	113058350	A	G	exonic	DTX1	233	45	0.1619	Missense_Mutation	p.K53R	Targerted Panel Gene	ABC-DLBCL
ABC-22	chr7	148811636	148811636	A	G	exonic	EZH2	228	86	0.2739	Missense_Mutation	p.Y646H	Targerted Panel Gene	ABC-DLBCL
ABC-22	chr4	186628295	186628295	C	T	exonic	FAT1	323	72	0.1823	Missense_Mutation	p.D1557N	Targerted Panel Gene	ABC-DLBCL
ABC-22	chr19	39915469	39915469	C	T	exonic	FCGBP	136	46	0.2527	Missense_Mutation	p.V849I	Targerted Panel Gene	ABC-DLBCL
ABC-22	chr17	65056413	65056413	T	C	exonic	GNA13	169	32	0.1592	Missense_Mutation	p.K61E	Targerted Panel Gene	ABC-DLBCL
ABC-22	chr6	27867520	27867520	T	C	exonic	HIST1H1B	188	78	0.2932	Missense_Mutation	p.T4A	Targerted Panel Gene	ABC-DLBCL
ABC-22	chr1	149886409	149886409	C	T	exonic	HIST2H2BE	84	18	0.1765	Missense_Mutation	p.A78T	Targerted Panel Gene	ABC-DLBCL
ABC-22	chr1	23559260	23559260	G	A	exonic	ID3	207	63	0.2333	Missense_Mutation	p.P56L	Targerted Panel Gene	ABC-DLBCL
ABC-22	chr22	22888210	22888210	G	A	exonic	IGLL5	107	15	0.123	Missense_Mutation	p.G53R	Targerted Panel Gene	ABC-DLBCL
ABC-22	chr22	22888207	22888207	C	T	exonic	IGLL5	105	14	0.1176	Missense_Mutation	p.P52S	Targerted Panel Gene	ABC-DLBCL
ABC-22	chr22	22888214	22888214	C	A	exonic	IGLL5	109	14	0.1138	Missense_Mutation	p.P19T	Targerted Panel Gene	ABC-DLBCL
ABC-22	chr22	22888054	22888054	A	T	exonic	IGLL5	163	43	0.2087	Missense_Mutation	p.M1L	Targerted Panel Gene	ABC-DLBCL
ABC-22	chr22	22888178	22888178	T	G	exonic	IGLL5	121	16	0.1168	Missense_Mutation	p.W7G	Targerted Panel Gene	ABC-DLBCL
ABC-22	chr22	22888064	22888064	A	G	exonic	IGLL5	162	49	0.2311	Missense_Mutation	p.K4R	Targerted Panel Gene	ABC-DLBCL
ABC-22	chr22	22888220	22888220	T	G	exonic	IGLL5	102	16	0.1356	Missense_Mutation	p.L21V	Targerted Panel Gene	ABC-DLBCL

ABC-22	chr2	140514736	140514736	G	A	exonic	LRP1B	230	227	0.4967	Missense_Mutation	p.A2729V	Targerted Panel Gene	ABC-DLBCL
ABC-22	chr13	101062105	101062105	G	A	exonic	NALCN	115	26	0.1844	Missense_Mutation	p.R1540W	Targerted Panel Gene	ABC-DLBCL
ABC-22	chr9	37034010	37034010	G	C	exonic	PAX5	228	57	0.1979	Missense_Mutation	p.P8A	Targerted Panel Gene	ABC-DLBCL
ABC-22	chr4	133154991	133154991	A	T	exonic	PCDH10	339	87	0.2042	Missense_Mutation	p.D922V	Targerted Panel Gene	ABC-DLBCL
ABC-22	chr6	37171434	37171434	C	T	exonic	PIM1	131	98	0.363	Missense_Mutation	p.L184F	Targerted Panel Gene	ABC-DLBCL
ABC-22	chr6	37170520	37170520	G	A	exonic	PIM1	52	64	0.5517	Missense_Mutation	p.G73D	Targerted Panel Gene	ABC-DLBCL
ABC-22	chr6	37170413	37170413	C	G	exonic	PIM1	37	22	0.3729	Missense_Mutation	p.D37E	Targerted Panel Gene	ABC-DLBCL
ABC-22	chr6	37170784	37170784	G	C	exonic	PIM1	82	42	0.3387	Missense_Mutation	p.E32Q	Targerted Panel Gene	ABC-DLBCL
ABC-22	chr6	37171198	37171198	G	C	exonic	PIM1	262	133	0.3367	Missense_Mutation	p.R105T	Targerted Panel Gene	ABC-DLBCL
ABC-22	chr6	37173082	37173082	C	G	exonic	PIM1	377	85	0.1836	Missense_Mutation	p.L232V	Targerted Panel Gene	ABC-DLBCL
ABC-22	chr6	37171228	37171228	G	A	exonic	PIM1	354	43	0.108	Missense_Mutation	p.S115N	Targerted Panel Gene	ABC-DLBCL
ABC-22	chr2	1660881	1660881	C	T	exonic	PXDN	240	46	0.1608	Missense_Mutation	p.V613I	Targerted Panel Gene	ABC-DLBCL
ABC-22	chr16	11255247	11255247	C	T	exonic	SOCS1	65	23	0.2614	Missense_Mutation	p.G78R	Targerted Panel Gene	ABC-DLBCL
ABC-22	chr16	11255468	11255468	T	C	exonic	SOCS1	139	32	0.1871	Missense_Mutation	p.H4R	Targerted Panel Gene	ABC-DLBCL
ABC-22	chr3	177032999	177032999	T	C	exonic	TBL1XR1	351	94	0.2112	Missense_Mutation	p.D463G	Targerted Panel Gene	ABC-DLBCL
ABC-22	chr16	3793494	3793494	G	A	exonic	CREBBP	353	104	0.2276	Nonsense_Mutation	p.R370X	Targerted Panel Gene	ABC-DLBCL
ABC-22	chr22	41177696	41177696	G	A	exonic	EP300	319	93	0.2252	Nonsense_Mutation	p.W1995X	Targerted Panel Gene	ABC-DLBCL
ABC-22	chr6	37173033	37173033	C	G	exonic	PIM1	265	144	0.3521	Nonsense_Mutation	p.Y215X	Targerted Panel Gene	ABC-DLBCL
ABC-22	chr6	37171211	37171211	G	A	exonic	PIM1	373	35	0.0856	Nonsense_Mutation	p.W109X	Targerted Panel Gene	ABC-DLBCL
ABC-22	chr3	38141150	38141150	T	C	exonic	MYD88	199	146	0.4232	Nonstop_Mutation	p.L265P	Targerted Panel Gene	ABC-DLBCL
ABC-23	chr2	21012516	21012516	C	T	exonic	APOB	499	455	0.4764	Missense_Mutation	p.G1451D	Targerted Panel Gene	ABC-DLBCL
ABC-23	chr11	103155385	103155385	T	G	exonic	DYNC2H1	637	92	0.1262	Missense_Mutation	p.W1210G	Targerted Panel Gene	ABC-DLBCL
ABC-23	chr19	4504680	4504680	G	T	exonic	PLIN4	75	63	0.4565	Missense_Mutation	p.Q1285K	Targerted Panel Gene	ABC-DLBCL
ABC-23	chr3	52502088	52502088	C	A	exonic	STAB1	193	137	0.4152	Nonsense_Mutation	p.C138X	Targerted Panel Gene	ABC-DLBCL
ABC-24	chr15	44711581	44711581	T	C	exonic	B2M	214	86	0.2867	Missense_Mutation	p.L12P	Targerted Panel Gene	ABC-DLBCL
ABC-24	chr22	23181018	23181018	C	T	exonic	BCR	123	24	0.1633	Missense_Mutation	p.P20S	Targerted Panel Gene	ABC-DLBCL
ABC-24	chr6	393325	393325	G	A	exonic	IRF4	295	43	0.1268	Missense_Mutation	p.G58D	Targerted Panel Gene	ABC-DLBCL
ABC-24	chr6	393324	393324	G	A	exonic	IRF4	301	43	0.125	Missense_Mutation	p.G58S	Targerted Panel Gene	ABC-DLBCL
ABC-24	chr2	50531344	50531344	A	C	exonic	NRXN1	617	83	0.1182	Missense_Mutation	p.L784V	Targerted Panel Gene	ABC-DLBCL
ABC-24	chr6	75267713	75267713	A	C	exonic	TMEM30A	572	96	0.1435	Missense_Mutation	p.C91W	Targerted Panel Gene	ABC-DLBCL
ABC-24	chr19	22088126	22088126	T	C	exonic	ZNF257	103	12	0.1043	Missense_Mutation	p.C50R	Targerted Panel Gene	ABC-DLBCL
ABC-24	chr19	22088133	22088133	G	A	exonic	ZNF257	94	12	0.1132	Missense_Mutation	p.G52E	Targerted Panel Gene	ABC-DLBCL
ABC-24	chr19	22088132	22088132	G	C	exonic	ZNF257	97	12	0.1101	Missense_Mutation	p.G52R	Targerted Panel Gene	ABC-DLBCL
ABC-24	chr19	22088163	22088163	T	C	exonic	ZNF257	85	15	0.15	Missense_Mutation	p.I62T	Targerted Panel Gene	ABC-DLBCL
ABC-24	chr19	22088156	22088156	T	C	exonic	ZNF257	91	15	0.1415	Missense_Mutation	p.C60R	Targerted Panel Gene	ABC-DLBCL
ABC-24	chr19	22088157	22088157	G	A	exonic	ZNF257	91	15	0.1415	Missense_Mutation	p.C60Y	Targerted Panel Gene	ABC-DLBCL
ABC-24	chr16	3728776	3728776	T	A	exonic	CREBBP	372	40	0.0971	Nonsense_Mutation	p.K2091X	Targerted Panel Gene	ABC-DLBCL
ABC-25	chrX	40055479	40055479	C	T	exonic	BCOR	37	129	0.7771	Missense_Mutation	p.E1510K	Targerted Panel Gene	ABC-DLBCL
ABC-25	chrX	40053964	40053964	T	G	exonic	BCOR	62	185	0.749	Missense_Mutation	p.Y1599S	Targerted Panel Gene	ABC-DLBCL
ABC-25	chrX	40052297	40052297	C	T	exonic	BCOR	74	131	0.639	Missense_Mutation	p.E1660K	Targerted Panel Gene	ABC-DLBCL
ABC-25	chr17	63929830	63929830	C	T	exonic	CD79B	126	75	0.3731	Missense_Mutation	p.M164I	Targerted Panel Gene	ABC-DLBCL
ABC-25	chr11	6533104	6533104	A	G	exonic	DNHD1	158	183	0.5367	Missense_Mutation	p.M809V	Targerted Panel Gene	ABC-DLBCL
ABC-25	chr11	92353380	92353380	A	G	exonic	FAT3	464	259	0.3582	Missense_Mutation	p.N423S	Targerted Panel Gene	ABC-DLBCL
ABC-25	chr13	40665716	40665716	G	A	exonic	FOXO1	30	128	0.8101	Missense_Mutation	p.A166V	Targerted Panel Gene	ABC-DLBCL
ABC-25	chr22	22888239	22888239	C	A	exonic	IGLL5	70	34	0.3269	Missense_Mutation	p.A27D	Targerted Panel Gene	ABC-DLBCL
ABC-25	chr7	152182587	152182587	C	T	exonic	KMT2C	236	126	0.3481	Missense_Mutation	p.R1758H	Targerted Panel Gene	ABC-DLBCL
ABC-25	chr3	38140768	38140768	T	C	exonic	MYD88	328	219	0.4004	Missense_Mutation	p.M232T	Targerted Panel Gene	ABC-DLBCL
ABC-25	chr1	237680532	237680532	G	A	exonic	RYR2	200	113	0.361	Missense_Mutation	p.C2991Y	Targerted Panel Gene	ABC-DLBCL
ABC-25	chr16	11255317	11255317	G	C	exonic	SOCS1	58	38	0.3958	Missense_Mutation	p.H54Q	Targerted Panel Gene	ABC-DLBCL
ABC-25	chr16	72794458	72794458	C	G	exonic	ZFHX3	296	253	0.46	Missense_Mutation	p.G2742R	Targerted Panel Gene	ABC-DLBCL
ABC-25	chr22	22888221	22888221	T	A	exonic	IGLL5	64	47	0.4234	Nonsense_Mutation	p.L21X	Targerted Panel Gene	ABC-DLBCL
ABC-25	chr1	15215358	15215358	C	T	exonic	TMEM51	86	99	0.5351	Nonsense_Mutation	p.Q91X	Targerted Panel Gene	ABC-DLBCL
ABC-26	chr2	32518266	32518266	C	G	exonic	BIRC6	241	237	0.4958	Missense_Mutation	p.L3788V	Targerted Panel Gene	ABC-DLBCL
ABC-26	chr22	41170517	41170517	G	C	exonic	EP300	351	159	0.3118	Missense_Mutation	p.W1466C	Targerted Panel Gene	ABC-DLBCL
ABC-26	chr6	37171254	37171254	G	A	exonic	PIM1	428	143	0.2496	Missense_Mutation	p.E124K	Targerted Panel Gene	ABC-DLBCL
ABC-26	chr3	77607870	77607870	C	A	exonic	ROBO2	537	143	0.2103	Missense_Mutation	p.A477D	Targerted Panel Gene	ABC-DLBCL
ABC-26	chr1	215680234	215680234	C	G	exonic	USH2A	321	315	0.4945	Missense_Mutation	p.R4070T	Targerted Panel Gene	ABC-DLBCL
ABC-26	chr1	116521964	116521964	A	C	exonic	CD58	185	95	0.3393	Nonsense_Mutation	p.Y216X	Targerted Panel Gene	ABC-DLBCL
ABC-26	chr3	38141150	38141150	T	C	exonic	MYD88	244	225	0.4787	Nonstop_Mutation	p.L265P	Targerted Panel Gene	ABC-DLBCL
ABC-27	chr7	140794408	140794408	C	T	exonic	BRAF	435	431	0.4971	Missense_Mutation	p.R347Q	Targerted Panel Gene	ABC-DLBCL
ABC-27	chr6	127995502	127995502	T	A	exonic	PTPRK	261	99	0.275	Missense_Mutation	p.D951V	Targerted Panel Gene	ABC-DLBCL
ABC-27	chr4	20524106	20524106	G	A	exonic	SLIT2	575	230	0.2854	Missense_Mutation	p.R456H	Targerted Panel Gene	ABC-DLBCL
ABC-27	chr2	230238381	230238381	G	A	exonic	SP140	327	148	0.3109	Missense_Mutation	p.V136I	Targerted Panel Gene	ABC-DLBCL
ABC-28	chrX	101356818	101356818	C	T	exonic	BTK	38	275	0.8786	Missense_Mutation	p.E473K	Targerted Panel Gene	ABC-DLBCL
ABC-28	chr17	63931343	63931343	T	G	exonic	CD79B	177	127	0.4178	Missense_Mutation	p.N37T	Targerted Panel Gene	ABC-DLBCL
ABC-28	chr2	96144852	96144852	G	A	exonic	DUSP2	70	64	0.4776	Missense_Mutation	p.P140L	Targerted Panel Gene	ABC-DLBCL
ABC-28	chr22	41170515	41170515	T	C	exonic	EP300	364	232	0.3893	Missense_Mutation	p.W1466R	Targerted Panel Gene	ABC-DLBCL
ABC-28	chr20	58854442	58854442	A	G	exonic	GNAS	120	113	0.4829	Missense_Mutation	p.R393G	Targerted Panel Gene	ABC-DLBCL
ABC-28	chr6	26234292	26234292	C	G	exonic	HIST1H1D	433	337	0.4377	Missense_Mutation	p.K214N	Targerted Panel Gene	ABC-DLBCL
ABC-28	chr6	26234630	26234630	C	T	exonic	HIST1H1D	395	298	0.43	Missense_Mutation	p.A102T	Targerted Panel Gene	ABC-DLBCL
ABC-28	chr6	26124345	26124345	G	A	exonic	HIST1H2AC	184	141	0.4338	Missense_Mutation	p.G38D	Targerted Panel Gene	ABC-DLBCL
ABC-28	chr6	26251730	26251730	G	A	exonic	HIST1H2BH	99	55	0.3571	Missense_Mutation	p.G27D	Targerted Panel Gene	ABC-DLBCL
ABC-28	chr22	22888064	22888064	A	C	exonic	IGLL5	165	101	0.3797	Missense_Mutation	p.K4T	Targerted Panel Gene	ABC-DLBCL
ABC-28	chr3	183555569	183555569	C	T	exonic	KLHL6	429	322	0.4282	Missense_Mutation	p.D29N	Targerted Panel Gene	ABC-DLBCL
ABC-28	chr19	19149377	19149377	C	T	exonic	MEF2B,MEF2BNB-	234	155	0.3985	Missense_Mutation	p.S36N	Targerted Panel Gene	ABC-DLBCL
ABC-28	chr6	37170437	37170437	C	A	exonic	PIM1	54	19	0.2603	Missense_Mutation	p.S45R	Targerted Panel Gene	ABC-DLBCL
ABC-28	chr8	109526802	109526802	A	C	exonic	PKHD1L1	254	182	0.4174	Missense_Mutation	p.E4168A	Targerted Panel Gene	ABC-DLBCL
ABC-28	chr15	33633098	33633098	G	A	exonic	RYR3	259	188	0.4196	Missense_Mutation	p.G1006D	Targerted Panel Gene	ABC-DLBCL
ABC-28	chr18	55228979	55228979	C	T	exonic	TCF4	551	225	0.2899	Missense_Mutation	p.D685N	Targerted Panel Gene	ABC-DLBCL
ABC-28	chr9	74762031	74762031	T	A	exonic	TRPM6	90	238	0.7256	Missense_Mutation	p.E1547V	Targerted Panel Gene	ABC-DLBCL
ABC-28	chr6	37171263	37171263	C	T	exonic	PIM1	182	215	0.5416	Nonsense_Mutation	p.Q127X	Targerted Panel Gene	ABC-DLBCL
ABC-28	chr3	38141150	38141150	T	C	exonic	MYD88	58	379	0.8673	Nonstop_Mutation	p.L265P	Targerted Panel Gene	ABC-DLBCL

ABC-29	chr7	2937980	2937980	T	A	exonic	CARD11	271	30	0.0997	Missense_Mutation	p.D357V	Targerted Panel Gene	ABC-DLBCL
ABC-29	chr7	2939865	2939865	A	G	exonic	CARD11	716	70	0.0891	Missense_Mutation	p.S250P	Targerted Panel Gene	ABC-DLBCL
ABC-29	chr16	3736714	3736714	A	T	exonic	CREBBP	651	94	0.1262	Missense_Mutation	p.L1499Q	Targerted Panel Gene	ABC-DLBCL
ABC-29	chr6	26055793	26055793	C	G	exonic	HIST1H1C	255	251	0.496	Missense_Mutation	p.K212N	Targerted Panel Gene	ABC-DLBCL
ABC-29	chr1	228303866	228303866	G	A	exonic	OBSCN	694	80	0.1032	Missense_Mutation	p.A4644T	Targerted Panel Gene	ABC-DLBCL
ABC-29	chr1	2558409	2558409	C	T	exonic	TNFRSF14	250	36	0.1259	Missense_Mutation	p.T82I	Targerted Panel Gene	ABC-DLBCL
ABC-3	chr3	187745408	187745408	A	G	splicing	BCL6	227	113	0.3324	Splice_Site	.	Targerted Panel Gene	ABC-DLBCL
ABC-3	chr19	6590102	6590102	C	T	splicing	CD70	42	188	0.8174	Splice_Site	.	Targerted Panel Gene	ABC-DLBCL
ABC-3	chr22	22888260	22888260	G	C	splicing	IGLL5	57	38	0.4	Splice_Site	.	Targerted Panel Gene	ABC-DLBCL
ABC-3	chr6	106106460	106106460	-	T	exonic	PRDM1	90	242	0.7289	Frame_Shift_Ins	p.H621fs	Targerted Panel Gene	ABC-DLBCL
ABC-3	chr17	63929439	63929439	A	C	exonic	CD79B	85	65	0.4333	Missense_Mutation	p.Y197D	Targerted Panel Gene	ABC-DLBCL
ABC-3	chr8	112244464	112244464	T	G	exonic	CSMD3	400	129	0.2439	Missense_Mutation	p.L3404F	Targerted Panel Gene	ABC-DLBCL
ABC-3	chr12	113058292	113058292	C	T	exonic	DTX1	134	70	0.3415	Missense_Mutation	p.H34Y	Targerted Panel Gene	ABC-DLBCL
ABC-3	chr22	22888181	22888181	T	C	exonic	IGLL5	95	58	0.3791	Missense_Mutation	p.V43A	Targerted Panel Gene	ABC-DLBCL
ABC-3	chr22	22888133	22888133	T	C	exonic	IGLL5	109	67	0.3807	Missense_Mutation	p.L27P	Targerted Panel Gene	ABC-DLBCL
ABC-3	chr22	22893780	22893780	G	A	exonic	IGLL5	221	130	0.3704	Missense_Mutation	p.C96Y	Targerted Panel Gene	ABC-DLBCL
ABC-3	chr22	22888249	22888249	C	G	exonic	IGLL5	64	43	0.4019	Missense_Mutation	p.L66V	Targerted Panel Gene	ABC-DLBCL
ABC-3	chr7	82950540	82950540	A	C	exonic	PCLO	282	147	0.3427	Missense_Mutation	p.W3350G	Targerted Panel Gene	ABC-DLBCL
ABC-3	chr6	37170532	37170532	G	A	exonic	PIM1	66	39	0.3714	Missense_Mutation	p.S77N	Targerted Panel Gene	ABC-DLBCL
ABC-3	chr6	37170839	37170839	G	A	exonic	PIM1	105	54	0.3396	Missense_Mutation	p.G50D	Targerted Panel Gene	ABC-DLBCL
ABC-3	chr6	51830947	51830947	G	C	exonic	PKHD1	120	369	0.7546	Missense_Mutation	p.P2739R	Targerted Panel Gene	ABC-DLBCL
ABC-3	chr1	237631530	237631530	G	C	exonic	RYR2	216	202	0.481	Missense_Mutation	p.G2182R	Targerted Panel Gene	ABC-DLBCL
ABC-3	chr17	7673823	7673823	C	T	exonic	TP53	163	98	0.3755	Missense_Mutation	p.G227E	Targerted Panel Gene	ABC-DLBCL
ABC-3	chr1	3727185	3727185	G	T	exonic	TP73	151	91	0.376	Missense_Mutation	p.R268L	Targerted Panel Gene	ABC-DLBCL
ABC-3	chr4	1974878	1974878	G	A	exonic	WHSC1	242	153	0.3873	Missense_Mutation	p.A1130T	Targerted Panel Gene	ABC-DLBCL
ABC-3	chr19	22088126	22088126	T	C	exonic	ZNF257	61	12	0.1644	Missense_Mutation	p.C50R	Targerted Panel Gene	ABC-DLBCL
ABC-3	chr19	22088127	22088127	G	A	exonic	ZNF257	59	12	0.169	Missense_Mutation	p.C50Y	Targerted Panel Gene	ABC-DLBCL
ABC-3	chr19	22088132	22088132	G	C	exonic	ZNF257	59	11	0.1571	Missense_Mutation	p.G52R	Targerted Panel Gene	ABC-DLBCL
ABC-3	chr19	22088133	22088133	G	A	exonic	ZNF257	59	11	0.1571	Missense_Mutation	p.G52E	Targerted Panel Gene	ABC-DLBCL
ABC-3	chr6	41935993	41935993	G	A	exonic	CCND3	51	200	0.7968	Nonsense_Mutation	p.Q276X	Targerted Panel Gene	ABC-DLBCL
ABC-3	chr22	22895605	22895605	C	T	exonic	IGLL5	131	65	0.3316	Nonsense_Mutation	p.Q186X	Targerted Panel Gene	ABC-DLBCL
ABC-3	chr1	119915641	119915641	G	A	exonic	NOTCH2	79	136	0.6326	Nonsense_Mutation	p.Q2361X	Targerted Panel Gene	ABC-DLBCL
ABC-30	chr1	116544542	116544542	C	A	exonic	CD58	360	340	0.4857	Missense_Mutation	p.V45L	Targerted Panel Gene	ABC-DLBCL
ABC-30	chr18	34794120	34794120	G	A	exonic	DTNA	415	392	0.4857	Missense_Mutation	p.V78M	Targerted Panel Gene	ABC-DLBCL
ABC-30	chr2	169225392	169225392	A	G	exonic	LRP2	328	345	0.5126	Missense_Mutation	p.M1819T	Targerted Panel Gene	ABC-DLBCL
ABC-30	chr12	40354384	40354384	C	T	exonic	LRRK2	335	359	0.5173	Missense_Mutation	p.S2221F	Targerted Panel Gene	ABC-DLBCL
ABC-30	chr19	22088126	22088126	T	C	exonic	ZNF257	79	13	0.1413	Missense_Mutation	p.C50R	Targerted Panel Gene	ABC-DLBCL
ABC-30	chr19	22088127	22088127	G	A	exonic	ZNF257	77	13	0.1444	Missense_Mutation	p.C50Y	Targerted Panel Gene	ABC-DLBCL
ABC-30	chr19	22088132	22088132	G	C	exonic	ZNF257	79	13	0.1413	Missense_Mutation	p.G52R	Targerted Panel Gene	ABC-DLBCL
ABC-30	chr19	22088133	22088133	G	A	exonic	ZNF257	79	12	0.1319	Missense_Mutation	p.G52E	Targerted Panel Gene	ABC-DLBCL
ABC-30	chr19	21423013	21423013	G	C	exonic	ZNF493	35	32	0.4776	Missense_Mutation	p.K118N	Targerted Panel Gene	ABC-DLBCL
ABC-31	chr5	79731167	79731167	A	G	exonic	CMYA5	402	394	0.495	Missense_Mutation	p.Y801C	Targerted Panel Gene	ABC-DLBCL
ABC-31	chr2	169173996	169173996	C	T	exonic	LRP2	192	185	0.4907	Missense_Mutation	p.R3646H	Targerted Panel Gene	ABC-DLBCL
ABC-31	chr2	151553971	151553971	C	A	exonic	NEB	303	315	0.5097	Missense_Mutation	p.D6495Y	Targerted Panel Gene	ABC-DLBCL
ABC-32	chr12	113058451	113058451	G	A	exonic	DTX1	211	32	0.1317	Missense_Mutation	p.G87S	Targerted Panel Gene	ABC-DLBCL
ABC-32	chr4	186621149	186621149	C	T	exonic	FAT1	428	395	0.48	Missense_Mutation	p.E1813K	Targerted Panel Gene	ABC-DLBCL
ABC-32	chr13	101082818	101082818	A	T	exonic	NALCN	217	195	0.4733	Missense_Mutation	p.F1252L	Targerted Panel Gene	ABC-DLBCL
ABC-32	chr3	47005056	47005056	G	A	exonic	NBEAL2	245	249	0.503	Missense_Mutation	p.G2127S	Targerted Panel Gene	ABC-DLBCL
ABC-32	chr1	228360237	228360237	C	G	exonic	OBSCN	250	219	0.466	Missense_Mutation	p.L6449V	Targerted Panel Gene	ABC-DLBCL
ABC-32	chr8	109476530	109476530	C	T	exonic	PKHD1L1	275	216	0.4399	Missense_Mutation	p.S2927L	Targerted Panel Gene	ABC-DLBCL
ABC-32	chr1	15931279	15931279	C	G	exonic	SPEN	573	64	0.1005	Nonsense_Mutation	p.S1680X	Targerted Panel Gene	ABC-DLBCL
ABC-33	chr1	240093601	240093601	G	A	exonic	FMN2	35	33	0.4853	Missense_Mutation	p.G498S	Targerted Panel Gene	ABC-DLBCL
ABC-33	chr6	394899	394899	T	C	exonic	IRF4	299	38	0.1124	Missense_Mutation	p.C99R	Targerted Panel Gene	ABC-DLBCL
ABC-33	chrX	141881542	141881542	A	T	exonic	MAGEC3	48	18	0.2727	Missense_Mutation	p.T219S	Targerted Panel Gene	ABC-DLBCL
ABC-33	chr1	228282026	228282026	C	G	exonic	OBSCN	145	101	0.4089	Missense_Mutation	p.A3193G	Targerted Panel Gene	ABC-DLBCL
ABC-33	chr1	15934599	15934599	C	G	exonic	SPEN	188	176	0.4835	Missense_Mutation	p.P2787A	Targerted Panel Gene	ABC-DLBCL
ABC-33	chr3	52510467	52510467	G	A	exonic	STAB1	126	17	0.1189	Missense_Mutation	p.C916Y	Targerted Panel Gene	ABC-DLBCL
ABC-34	chr6	26056298	26056298	A	G	exonic	HIST1H1C	248	84	0.253	Missense_Mutation	p.I44T	Targerted Panel Gene	ABC-DLBCL
ABC-34	chr22	22888230	22888230	C	A	exonic	IGLL5	95	13	0.1204	Missense_Mutation	p.A24D	Targerted Panel Gene	ABC-DLBCL
ABC-34	chr22	22893768	22893768	C	T	exonic	IGLL5	268	33	0.1096	Missense_Mutation	p.P92L	Targerted Panel Gene	ABC-DLBCL
ABC-34	chrX	71137582	71137582	T	A	exonic	MED12	154	39	0.2021	Missense_Mutation	p.S1925T	Targerted Panel Gene	ABC-DLBCL
ABC-34	chr6	37171269	37171269	C	T	exonic	PIM1	288	58	0.1676	Missense_Mutation	p.L129F	Targerted Panel Gene	ABC-DLBCL
ABC-34	chr6	37170596	37170596	C	G	exonic	PIM1	123	15	0.1087	Missense_Mutation	p.N7K	Targerted Panel Gene	ABC-DLBCL
ABC-34	chr7	98977026	98977026	T	A	exonic	TRRAP	298	63	0.1745	Missense_Mutation	p.S2772T	Targerted Panel Gene	ABC-DLBCL
ABC-34	chr4	84733446	84733446	C	T	exonic	WDFY3	413	81	0.164	Missense_Mutation	p.R2386Q	Targerted Panel Gene	ABC-DLBCL
ABC-34	chr3	38141150	38141150	T	C	exonic	MYD88	258	72	0.2182	Nonstop_Mutation	p.L265P	Targerted Panel Gene	ABC-DLBCL
ABC-35	chr6	393360	393360	C	G	exonic	IRF4	210	48	0.186	Missense_Mutation	p.L70V	Targerted Panel Gene	ABC-DLBCL
ABC-35	chr2	151678161	151678161	C	A	exonic	NEB	647	134	0.1711	Missense_Mutation	p.K1094N	Targerted Panel Gene	ABC-DLBCL
ABC-35	chr14	79697789	79697789	G	T	exonic	NRXN3	848	342	0.2874	Missense_Mutation	p.S886I	Targerted Panel Gene	ABC-DLBCL
ABC-35	chr1	237784338	237784338	C	T	exonic	RYR2	603	134	0.1816	Missense_Mutation	p.S4209L	Targerted Panel Gene	ABC-DLBCL
ABC-35	chrX	71424173	71424173	A	G	exonic	TAF1	467	130	0.2178	Missense_Mutation	p.Y1562C	Targerted Panel Gene	ABC-DLBCL
ABC-35	chr1	215970690	215970690	G	A	exonic	USH2A	479	90	0.1582	Missense_Mutation	p.P2298S	Targerted Panel Gene	ABC-DLBCL
ABC-36	chr7	111123017	111123017	C	A	exonic	LRRN3	808	143	0.1501	Missense_Mutation	p.A82E	Targerted Panel Gene	ABC-DLBCL
ABC-36	chr9	13122107	13122107	C	G	exonic	MPDZ	283	188	0.3992	Missense_Mutation	p.A1673P	Targerted Panel Gene	ABC-DLBCL
ABC-36	chr17	7675137	7675137	C	G	exonic	TP53	172	28	0.14	Missense_Mutation	p.A120P	Targerted Panel Gene	ABC-DLBCL
ABC-36	chr1	215879002	215879002	C	T	exonic	USH2A	385	350	0.4755	Missense_Mutation	p.A2774T	Targerted Panel Gene	ABC-DLBCL
ABC-37	chr20	41166288	41166288	C	G	exonic	PLCG1	160	148	0.4805	Missense_Mutation	p.L632V	Targerted Panel Gene	ABC-DLBCL
ABC-37	chr19	24126522	24126522	A	G	exonic	ZNF254	69	86	0.5548	Missense_Mutation	p.I133M	Targerted Panel Gene	ABC-DLBCL
ABC-4	chr3	187745409	187745409	C	T	splicing	BCL6	284	126	0.3073	Splice_Site	.	Targerted Panel Gene	ABC-DLBCL
ABC-4	chr1	116544500	116544500	-	T	exonic	CD58	255	426	0.6237	Frame_Shift_Ins	p.Q59fs	Targerted Panel Gene	ABC-DLBCL
ABC-4	chr17	63929438	63929452	TAGGTGTGATCTTC	-	exonic	CD79B	157	55	0.2594	In_Frame_Del	p.192_197del	Targerted Panel Gene	ABC-DLBCL
ABC-4	chr5	79730809	79730814	GACGAG	-	exonic	CMYA5	359	263	0.4228	In_Frame_Del	p.682_683del	Targerted Panel Gene	ABC-DLBCL

ABC-4	chr2	32468537	32468537	G	T	exonic	BIRC6	411	398	0.492	Missense_Mutation	p.D1961Y	Targerted Panel Gene	ABC-DLBCL
ABC-4	chr12	92145472	92145472	T	C	exonic	BTG1	74	93	0.5569	Missense_Mutation	p.I22V	Targerted Panel Gene	ABC-DLBCL
ABC-4	chr1	203305659	203305659	G	A	exonic	BTG2	114	53	0.3174	Missense_Mutation	p.G18D	Targerted Panel Gene	ABC-DLBCL
ABC-4	chrX	101375266	101375266	C	T	exonic	BTK	117	95	0.4481	Missense_Mutation	p.E7K	Targerted Panel Gene	ABC-DLBCL
ABC-4	chr7	2939925	2939925	C	T	exonic	CARD11	302	143	0.3206	Missense_Mutation	p.D230N	Targerted Panel Gene	ABC-DLBCL
ABC-4	chr16	88881390	88881390	C	T	exonic	CBFA2T3	42	37	0.4684	Missense_Mutation	p.A435T	Targerted Panel Gene	ABC-DLBCL
ABC-4	chr17	63929777	63929777	A	T	exonic	CD79B	82	101	0.5519	Missense_Mutation	p.L182Q	Targerted Panel Gene	ABC-DLBCL
ABC-4	chr1	102914807	102914807	G	C	exonic	COL11A1	81	92	0.5287	Missense_Mutation	p.P1286R	Targerted Panel Gene	ABC-DLBCL
ABC-4	chr6	27132519	27132519	C	T	exonic	HIST1H2BJ	57	17	0.2297	Missense_Mutation	p.A78T	Targerted Panel Gene	ABC-DLBCL
ABC-4	chr22	22888188	22888188	G	A	exonic	IGLL5	108	56	0.3415	Missense_Mutation	p.R10H	Targerted Panel Gene	ABC-DLBCL
ABC-4	chr22	22888161	22888161	T	G	exonic	IGLL5	113	51	0.311	Missense_Mutation	p.M1R	Targerted Panel Gene	ABC-DLBCL
ABC-4	chr4	142082142	142082142	C	A	exonic	INPP4B	300	123	0.2908	Missense_Mutation	p.S844I	Targerted Panel Gene	ABC-DLBCL
ABC-4	chr11	118474134	118474134	C	T	exonic	KMT2A	485	228	0.3189	Missense_Mutation	p.S992F	Targerted Panel Gene	ABC-DLBCL
ABC-4	chr3	38140801	38140801	G	A	exonic	MYD88	235	148	0.3864	Missense_Mutation	p.S243N	Targerted Panel Gene	ABC-DLBCL
ABC-4	chr6	37170621	37170621	C	T	exonic	PIM1	96	55	0.3642	Missense_Mutation	p.P16S	Targerted Panel Gene	ABC-DLBCL
ABC-4	chr6	106106421	106106421	C	G	exonic	PRDM1	150	113	0.428	Missense_Mutation	p.C608W	Targerted Panel Gene	ABC-DLBCL
ABC-4	chr3	52513793	52513793	A	G	exonic	STAB1	100	94	0.4845	Missense_Mutation	p.Q1116R	Targerted Panel Gene	ABC-DLBCL
ABC-4	chr19	1625593	1625593	G	A	exonic	TCF3	72	34	0.3208	Missense_Mutation	p.A161V	Targerted Panel Gene	ABC-DLBCL
ABC-4	chr2	167244799	167244799	T	C	exonic	XIRP2	462	176	0.2759	Missense_Mutation	p.L1136P	Targerted Panel Gene	ABC-DLBCL
ABC-4	chr14	32823802	32823802	C	T	exonic	AKAP6	380	173	0.3128	Nonsense_Mutation	p.Q1997X	Targerted Panel Gene	ABC-DLBCL
ABC-4	chr6	41935981	41935981	G	A	exonic	CCND3	221	115	0.3423	Nonsense_Mutation	p.Q280X	Targerted Panel Gene	ABC-DLBCL
ABC-4	chr2	140334516	140334516	C	A	exonic	LRP1B	375	139	0.2699	Nonsense_Mutation	p.E4054X	Targerted Panel Gene	ABC-DLBCL
ABC-4	chr6	137877081	137877081	C	T	exonic	TNFAIP3	177	146	0.452	Nonsense_Mutation	p.R271X	Targerted Panel Gene	ABC-DLBCL
ABC-4	chr1	3682422	3682422	G	A	exonic	TP73	32	57	0.6404	Nonsense_Mutation	p.W19X	Targerted Panel Gene	ABC-DLBCL
ABC-5	chr8	4994331	4994331	C	T	splicing	CSMD1	153	34	0.1818	Splice_Site	.	Targerted Panel Gene	ABC-DLBCL
ABC-5	chr22	41093099	41093099	G	C	splicing	EP300	286	76	0.2099	Splice_Site	.	Targerted Panel Gene	ABC-DLBCL
ABC-5	chr6	41936007	41936007	-	G	exonic	CCND3	320	111	0.2569	Frame_Shift_Ins	p.R271fs	Targerted Panel Gene	ABC-DLBCL
ABC-5	chr1	203305739	203305739	G	A	exonic	BTG2	130	34	0.2073	Missense_Mutation	p.A45T	Targerted Panel Gene	ABC-DLBCL
ABC-5	chr17	63929438	63929438	T	G	exonic	CD79B	180	178	0.4972	Missense_Mutation	p.Y197S	Targerted Panel Gene	ABC-DLBCL
ABC-5	chr10	17088280	17088280	G	A	exonic	CUBN	451	486	0.5176	Missense_Mutation	p.P611S	Targerted Panel Gene	ABC-DLBCL
ABC-5	chr7	150477256	150477256	G	T	exonic	GIMAP8	730	203	0.2176	Missense_Mutation	p.V492L	Targerted Panel Gene	ABC-DLBCL
ABC-5	chr22	22888178	22888178	T	G	exonic	IGLL5	230	80	0.2581	Missense_Mutation	p.W7G	Targerted Panel Gene	ABC-DLBCL
ABC-5	chr22	22895621	22895621	G	C	exonic	IGLL5	291	124	0.2988	Missense_Mutation	p.R191T	Targerted Panel Gene	ABC-DLBCL
ABC-5	chr6	393217	393217	G	T	exonic	IRF4	279	91	0.2459	Missense_Mutation	p.G22V	Targerted Panel Gene	ABC-DLBCL
ABC-5	chr19	19149372	19149372	G	A	exonic	MEF2B,MEF2BNB-I	351	106	0.2319	Missense_Mutation	p.L38F	Targerted Panel Gene	ABC-DLBCL
ABC-5	chr4	133151986	133151986	C	A	exonic	PCDH10	225	64	0.2215	Missense_Mutation	p.L616I	Targerted Panel Gene	ABC-DLBCL
ABC-5	chr15	44715613	44715613	C	G	exonic	B2M	611	221	0.2653	Nonsense_Mutation	p.Y86X	Targerted Panel Gene	ABC-DLBCL
ABC-6	chr11	128463506	128463507	TA	-	splicing	ETS1	335	516	0.6063	Splice_Site	.	Targerted Panel Gene	ABC-DLBCL
ABC-6	chr6	106088301	106088302	TG	-	exonic	PRDM1	186	266	0.5885	Frame_Shift_Del	p.L48fs	Targerted Panel Gene	ABC-DLBCL
ABC-6	chr11	108326070	108326070	G	A	exonic	ATM	215	551	0.7193	Missense_Mutation	p.A2274T	Targerted Panel Gene	ABC-DLBCL
ABC-6	chr1	203305688	203305688	G	A	exonic	BTG2	127	85	0.4009	Missense_Mutation	p.G28S	Targerted Panel Gene	ABC-DLBCL
ABC-6	chr15	92997052	92997052	A	G	exonic	CHD2	320	292	0.4771	Missense_Mutation	p.M1231V	Targerted Panel Gene	ABC-DLBCL
ABC-6	chr22	22888257	22888257	C	T	exonic	IGLL5	84	63	0.4286	Missense_Mutation	p.A33V	Targerted Panel Gene	ABC-DLBCL
ABC-6	chr7	82915945	82915945	C	A	exonic	PCLO	583	364	0.3844	Missense_Mutation	p.G4014V	Targerted Panel Gene	ABC-DLBCL
ABC-6	chr6	37170990	37170990	A	G	exonic	PIM1	297	200	0.4024	Missense_Mutation	p.K67E	Targerted Panel Gene	ABC-DLBCL
ABC-6	chr17	7673802	7673802	C	T	exonic	TP53	96	155	0.6175	Missense_Mutation	p.R234H	Targerted Panel Gene	ABC-DLBCL
ABC-6	chr1	215998919	215998919	A	C	exonic	USH2A	499	246	0.3302	Missense_Mutation	p.L2209V	Targerted Panel Gene	ABC-DLBCL
ABC-6	chr5	79732312	79732312	G	T	exonic	CMYA5	407	399	0.4944	Nonsense_Mutation	p.E1183X	Targerted Panel Gene	ABC-DLBCL
ABC-7	chr11	70206132	70206132	G	T	splicing	FADD	169	48	0.2212	Splice_Site	.	Targerted Panel Gene	ABC-DLBCL
ABC-7	chr22	41162779	41162780	TG	-	exonic	EP300	191	62	0.2451	Frame_Shift_Del	p.L1243fs	Targerted Panel Gene	ABC-DLBCL
ABC-7	chr1	26780139	26780139	T	C	exonic	ARID1A	303	86	0.2211	Missense_Mutation	p.C1864R	Targerted Panel Gene	ABC-DLBCL
ABC-7	chr19	6586339	6586339	C	T	exonic	CD70	109	86	0.441	Missense_Mutation	p.G88E	Targerted Panel Gene	ABC-DLBCL
ABC-7	chr5	79731955	79731955	C	A	exonic	CMYA5	176	156	0.4699	Missense_Mutation	p.Q1064K	Targerted Panel Gene	ABC-DLBCL
ABC-7	chr1	102939084	102939084	T	C	exonic	COL11A1	209	72	0.2562	Missense_Mutation	p.E1142G	Targerted Panel Gene	ABC-DLBCL
ABC-7	chr8	112247109	112247109	T	A	exonic	CSMD3	214	75	0.2595	Missense_Mutation	p.H3338L	Targerted Panel Gene	ABC-DLBCL
ABC-7	chr8	112682559	112682559	T	G	exonic	CSMD3	315	103	0.2464	Missense_Mutation	p.S814R	Targerted Panel Gene	ABC-DLBCL
ABC-7	chr12	113058247	113058247	C	A	exonic	DTX1	114	65	0.3631	Missense_Mutation	p.P19T	Targerted Panel Gene	ABC-DLBCL
ABC-7	chr12	113058383	113058383	A	G	exonic	DTX1	118	41	0.2579	Missense_Mutation	p.Q64R	Targerted Panel Gene	ABC-DLBCL
ABC-7	chr4	125416452	125416452	T	C	exonic	FAT4	180	55	0.234	Missense_Mutation	p.V2283A	Targerted Panel Gene	ABC-DLBCL
ABC-7	chr22	22888147	22888147	G	A	exonic	IGLL5	91	18	0.1651	Missense_Mutation	p.A32T	Targerted Panel Gene	ABC-DLBCL
ABC-7	chr11	118472439	118472439	G	A	exonic	KMT2A	303	100	0.2481	Missense_Mutation	p.R427Q	Targerted Panel Gene	ABC-DLBCL
ABC-7	chr2	140813773	140813773	T	C	exonic	LRP1B	296	86	0.2251	Missense_Mutation	p.Y1748C	Targerted Panel Gene	ABC-DLBCL
ABC-7	chr3	38140768	38140768	T	C	exonic	MYD88	156	33	0.1746	Missense_Mutation	p.M232T	Targerted Panel Gene	ABC-DLBCL
ABC-7	chr12	124341987	124341987	T	C	exonic	NCOR2	137	44	0.2431	Missense_Mutation	p.D1675G	Targerted Panel Gene	ABC-DLBCL
ABC-7	chr2	151633751	151633751	C	T	exonic	NEB	160	142	0.4702	Missense_Mutation	p.S3106N	Targerted Panel Gene	ABC-DLBCL
ABC-7	chr7	82955856	82955856	C	A	exonic	PCLO	305	94	0.2356	Missense_Mutation	p.L1699F	Targerted Panel Gene	ABC-DLBCL
ABC-7	chr9	131621998	131621998	A	T	exonic	RAPGEF1	101	19	0.1583	Missense_Mutation	p.M569K	Targerted Panel Gene	ABC-DLBCL
ABC-7	chr3	49375502	49375502	A	T	exonic	RHOA	152	63	0.293	Missense_Mutation	p.F30I	Targerted Panel Gene	ABC-DLBCL
ABC-7	chr3	49375466	49375466	A	G	exonic	RHOA	147	60	0.2899	Missense_Mutation	p.Y42H	Targerted Panel Gene	ABC-DLBCL
ABC-7	chr1	216097111	216097111	C	T	exonic	USH2A	173	101	0.3686	Missense_Mutation	p.G1577E	Targerted Panel Gene	ABC-DLBCL
ABC-7	chr1	119915497	119915497	G	A	exonic	NOTCH2	203	71	0.2572	Nonsense_Mutation	p.Q2409X	Targerted Panel Gene	ABC-DLBCL
ABC-7	chr1	15930648	15930648	C	T	exonic	SPEN	340	113	0.2494	Nonsense_Mutation	p.R1470X	Targerted Panel Gene	ABC-DLBCL
ABC-8	chr15	48441720	48441720	C	T	splicing	FBN1	346	194	0.3593	Splice_Site	.	Targerted Panel Gene	ABC-DLBCL
ABC-8	chr6	106106500	106106500	G	A	splicing	PRDM1	72	147	0.6682	Splice_Site	.	Targerted Panel Gene	ABC-DLBCL
ABC-8	chr6	27133351	27133352	CT	-	exonic	HIST1H2AG	50	30	0.375	Frame_Shift_Del	p.L94fs	Targerted Panel Gene	ABC-DLBCL
ABC-8	chr8	55952114	55952117	AAGT	-	exonic	LYN	219	298	0.5764	Frame_Shift_Del	p.Q212fs	Targerted Panel Gene	ABC-DLBCL
ABC-8	chr22	41178507	41178509	CAG	-	exonic	EP300	279	222	0.4431	In_Frame_Del	p.2266_2266del	Targerted Panel Gene	ABC-DLBCL
ABC-8	chr8	143866179	143866179	C	T	exonic	EPPK1	1043	117	0.1009	Missense_Mutation	p.V2359M	Targerted Panel Gene	ABC-DLBCL
ABC-8	chr8	143866113	143866113	C	T	exonic	EPPK1	909	87	0.0873	Missense_Mutation	p.D2381N	Targerted Panel Gene	ABC-DLBCL
ABC-8	chr10	89014221	89014221	A	G	exonic	FAS	387	218	0.3603	Missense_Mutation	p.D260G	Targerted Panel Gene	ABC-DLBCL
ABC-8	chr6	26156930	26156930	G	T	exonic	HIST1H1E	315	190	0.3762	Missense_Mutation	p.K180N	Targerted Panel Gene	ABC-DLBCL
ABC-8	chr6	27865557	27865557	G	A	exonic	HIST1H2AL	40	28	0.4118	Missense_Mutation	p.G68D	Targerted Panel Gene	ABC-DLBCL

ABC-8	chr1	201207742	201207742	G	T	exonic	IGFN1	432	392	0.4757	Missense_Mutation	p.R950I	Targerted Panel Gene	ABC-DLBCL
ABC-8	chr11	118519788	118519788	C	T	exonic	KMT2A	263	222	0.4568	Missense_Mutation	p.L3770F	Targerted Panel Gene	ABC-DLBCL
ABC-8	chr3	38140801	38140801	G	A	exonic	MYD88	250	146	0.3687	Missense_Mutation	p.S243N	Targerted Panel Gene	ABC-DLBCL
ABC-8	chr9	137542700	137542700	G	A	exonic	PNPLA7	103	133	0.5636	Missense_Mutation	p.P203L	Targerted Panel Gene	ABC-DLBCL
ABC-8	chr1	237589793	237589793	G	A	exonic	RYR2	364	229	0.3855	Missense_Mutation	p.G1200E	Targerted Panel Gene	ABC-DLBCL
ABC-8	chr15	44711548	44711548	T	C	exonic	B2M	67	123	0.6474	Missense_Mutation	p.M1T	Targerted Panel Gene	ABC-DLBCL
ABC-9	chr22	22888260	22888260	G	C	splicing	IGLL5	67	13	0.1605	Splice_Site	.	Targerted Panel Gene	ABC-DLBCL
ABC-9	chr6	137871301	137871301	C	-	exonic	TNFAIP3	217	72	0.2483	Frame_Shift_Del	p.T25fs	Targerted Panel Gene	ABC-DLBCL
ABC-9	chr19	6590918	6590918	-	CGCG	exonic	CD70	60	63	0.5122	Frame_Shift_Ins	p.G29fs	Targerted Panel Gene	ABC-DLBCL
ABC-9	chr5	112839054	112839054	G	A	exonic	APC	468	139	0.229	Missense_Mutation	p.E1154K	Targerted Panel Gene	ABC-DLBCL
ABC-9	chr1	203307377	203307377	A	G	exonic	BTG2	70	29	0.2929	Missense_Mutation	p.N139S	Targerted Panel Gene	ABC-DLBCL
ABC-9	chr1	203305658	203305658	G	A	exonic	BTG2	57	19	0.25	Missense_Mutation	p.G18S	Targerted Panel Gene	ABC-DLBCL
ABC-9	chr1	203305671	203305671	G	C	exonic	BTG2	56	18	0.2432	Missense_Mutation	p.S22T	Targerted Panel Gene	ABC-DLBCL
ABC-9	chr19	10797399	10797399	G	A	exonic	DNM2	219	74	0.2526	Missense_Mutation	p.D406N	Targerted Panel Gene	ABC-DLBCL
ABC-9	chr18	67514318	67514318	C	T	exonic	DSEL	274	140	0.3382	Missense_Mutation	p.M107I	Targerted Panel Gene	ABC-DLBCL
ABC-9	chr1	186061913	186061913	G	T	exonic	HMCN1	238	59	0.1987	Missense_Mutation	p.V2459L	Targerted Panel Gene	ABC-DLBCL
ABC-9	chr22	22888234	22888234	T	C	exonic	IGLL5	83	17	0.17	Missense_Mutation	p.S61P	Targerted Panel Gene	ABC-DLBCL
ABC-9	chr6	393318	393318	C	T	exonic	IRF4	84	37	0.3058	Missense_Mutation	p.H56Y	Targerted Panel Gene	ABC-DLBCL
ABC-9	chr7	111124549	111124549	A	C	exonic	LRRN3	389	123	0.2402	Missense_Mutation	p.I593L	Targerted Panel Gene	ABC-DLBCL
ABC-9	chr19	38617408	38617408	A	T	exonic	MAP4K1	118	28	0.1918	Missense_Mutation	p.I65K	Targerted Panel Gene	ABC-DLBCL
ABC-9	chr19	19149383	19149383	T	G	exonic	MEF2B,MEF2BNB-[illegible]	200	133	0.3994	Missense_Mutation	p.E34A	Targerted Panel Gene	ABC-DLBCL
ABC-9	chr19	19149425	19149425	G	A	exonic	MEF2B,MEF2BNB-[illegible]	192	44	0.1857	Missense_Mutation	p.T20M	Targerted Panel Gene	ABC-DLBCL
ABC-9	chr12	78168846	78168846	G	T	exonic	NAV3	278	67	0.1942	Missense_Mutation	p.G1654V	Targerted Panel Gene	ABC-DLBCL
ABC-9	chr1	237773574	237773574	C	A	exonic	RYR2	327	118	0.2652	Missense_Mutation	p.Q3901K	Targerted Panel Gene	ABC-DLBCL
ABC-9	chr1	237781637	237781637	A	G	exonic	RYR2	305	68	0.1823	Missense_Mutation	p.M3985V	Targerted Panel Gene	ABC-DLBCL
ABC-9	chr4	20617626	20617626	T	A	exonic	SLIT2	161	62	0.2743	Missense_Mutation	p.Y1442N	Targerted Panel Gene	ABC-DLBCL
ABC-9	chr2	228019815	228019815	A	T	exonic	SPHKAP	527	159	0.2314	Missense_Mutation	p.S347T	Targerted Panel Gene	ABC-DLBCL
ABC-9	chr1	3722043	3722043	T	A	exonic	TP73	271	86	0.2409	Missense_Mutation	p.L151H	Targerted Panel Gene	ABC-DLBCL
ABC-9	chr22	41150133	41150133	C	T	exonic	EP300	223	83	0.2712	Nonsense_Mutation	p.Q918X	Targerted Panel Gene	ABC-DLBCL
ABC-9	chr6	137871390	137871390	C	T	exonic	TNFAIP3	253	79	0.238	Nonsense_Mutation	p.Q55X	Targerted Panel Gene	ABC-DLBCL
GCB-1	chr19	1047026	1047026	T	C	splicing	ABCA7	134	57	0.2984	Splice_Site	.	Targerted Panel Gene	GCB-DLBCL
GCB-1	chr19	1062216	1062216	G	T	exonic	ABCA7	103	86	0.455	Missense_Mutation	p.C1872F	Targerted Panel Gene	GCB-DLBCL
GCB-1	chr2	124609872	124609872	G	C	exonic	CNTNAP5	253	140	0.3553	Missense_Mutation	p.D609H	Targerted Panel Gene	GCB-DLBCL
GCB-1	chr12	113058368	113058368	C	T	exonic	DTX1	235	120	0.338	Missense_Mutation	p.S59F	Targerted Panel Gene	GCB-DLBCL
GCB-1	chr22	22888214	22888214	C	T	exonic	IGLL5	157	55	0.2594	Missense_Mutation	p.P19S	Targerted Panel Gene	GCB-DLBCL
GCB-1	chr22	22888210	22888210	G	A	exonic	IGLL5	153	55	0.2632	Missense_Mutation	p.G53R	Targerted Panel Gene	GCB-DLBCL
GCB-1	chrX	71119380	71119380	T	C	exonic	MED12	39	100	0.7194	Missense_Mutation	p.L36P	Targerted Panel Gene	GCB-DLBCL
GCB-1	chr20	8789538	8789538	G	A	exonic	PLCB1	353	161	0.3132	Missense_Mutation	p.R1100Q	Targerted Panel Gene	GCB-DLBCL
GCB-1	chr3	16377739	16377739	G	A	exonic	RFTN1	338	166	0.3294	Missense_Mutation	p.H269Y	Targerted Panel Gene	GCB-DLBCL
GCB-1	chr4	105276151	105276151	C	T	exonic	TET2	289	570	0.6636	Missense_Mutation	p.H1881Y	Targerted Panel Gene	GCB-DLBCL
GCB-1	chr1	2556667	2556667	G	A	exonic	TNFRSF14	111	259	0.7	Missense_Mutation	p.M1I	Targerted Panel Gene	GCB-DLBCL
GCB-1	chr16	72797554	72797554	C	T	exonic	ZFHX3	460	233	0.3362	Missense_Mutation	p.A1710T	Targerted Panel Gene	GCB-DLBCL
GCB-1	chr1	26731344	26731344	C	T	exonic	ARID1A	754	71	0.0861	Nonsense_Mutation	p.Q515X	Targerted Panel Gene	GCB-DLBCL
GCB-10	chrX	33128145	33128145	A	C	splicing	DMD	184	54	0.2269	Splice_Site	.	Targerted Panel Gene	GCB-DLBCL
GCB-10	chr17	39777768	39777768	C	G	splicing	IKZF3	418	66	0.1361	Splice_Site	.	Targerted Panel Gene	GCB-DLBCL
GCB-10	chr9	37033985	37033985	C	T	splicing	PAX5	408	62	0.1319	Splice_Site	.	Targerted Panel Gene	GCB-DLBCL
GCB-10	chr6	134174587	134174587	C	G	splicing	SGK1	245	46	0.1581	Splice_Site	.	Targerted Panel Gene	GCB-DLBCL
GCB-10	chr6	134174509	134174509	A	G	splicing	SGK1	278	52	0.1576	Splice_Site	.	Targerted Panel Gene	GCB-DLBCL
GCB-10	chr6	137871524	137871524	T	C	splicing	TNFAIP3	395	78	0.1649	Splice_Site	.	Targerted Panel Gene	GCB-DLBCL
GCB-10	chr2	214987788	214987788	A	G	exonic	ABCA12	230	210	0.4773	Missense_Mutation	p.Y1279H	Targerted Panel Gene	GCB-DLBCL
GCB-10	chr19	1064202	1064202	T	C	exonic	ABCA7	45	29	0.3919	Missense_Mutation	p.M1998T	Targerted Panel Gene	GCB-DLBCL
GCB-10	chr14	32546978	32546978	A	C	exonic	AKAP6	397	64	0.1388	Missense_Mutation	p.E775D	Targerted Panel Gene	GCB-DLBCL
GCB-10	chr15	44711569	44711569	C	T	exonic	B2M	236	42	0.1511	Missense_Mutation	p.A8V	Targerted Panel Gene	GCB-DLBCL
GCB-10	chr12	92145424	92145424	G	C	exonic	BTG1	131	28	0.1761	Missense_Mutation	p.Q38E	Targerted Panel Gene	GCB-DLBCL
GCB-10	chr12	92145433	92145433	G	C	exonic	BTG1	155	20	0.1143	Missense_Mutation	p.R35G	Targerted Panel Gene	GCB-DLBCL
GCB-10	chrX	33128153	33128153	A	T	exonic	DMD	201	52	0.2055	Missense_Mutation	p.S5T	Targerted Panel Gene	GCB-DLBCL
GCB-10	chr12	113058221	113058221	T	A	exonic	DTX1	259	39	0.1309	Missense_Mutation	p.M10K	Targerted Panel Gene	GCB-DLBCL
GCB-10	chr12	113058406	113058406	T	G	exonic	DTX1	168	68	0.2881	Missense_Mutation	p.Y72D	Targerted Panel Gene	GCB-DLBCL
GCB-10	chr12	113058269	113058269	T	G	exonic	DTX1	229	38	0.1423	Missense_Mutation	p.V26G	Targerted Panel Gene	GCB-DLBCL
GCB-10	chr12	113058437	113058437	T	C	exonic	DTX1	183	22	0.1073	Missense_Mutation	p.F82S	Targerted Panel Gene	GCB-DLBCL
GCB-10	chr12	113058436	113058436	T	C	exonic	DTX1	184	22	0.1068	Missense_Mutation	p.F82L	Targerted Panel Gene	GCB-DLBCL
GCB-10	chr2	96144211	96144211	C	T	exonic	DUSP2	297	56	0.1582	Missense_Mutation	p.E225K	Targerted Panel Gene	GCB-DLBCL
GCB-10	chr2	96144828	96144828	G	C	exonic	DUSP2	100	17	0.1453	Missense_Mutation	p.A148G	Targerted Panel Gene	GCB-DLBCL
GCB-10	chr10	62813925	62813925	A	C	exonic	EGR2	618	92	0.1296	Missense_Mutation	p.L238R	Targerted Panel Gene	GCB-DLBCL
GCB-10	chr11	70206187	70206187	G	A	exonic	FADD	255	203	0.4423	Missense_Mutation	p.R114K	Targerted Panel Gene	GCB-DLBCL
GCB-10	chr4	125321098	125321098	A	G	exonic	FAT4	490	79	0.1388	Missense_Mutation	p.I1563V	Targerted Panel Gene	GCB-DLBCL
GCB-10	chr6	27867213	27867213	C	T	exonic	HIST1H1B	502	79	0.136	Missense_Mutation	p.G106D	Targerted Panel Gene	GCB-DLBCL
GCB-10	chr6	26056110	26056110	G	A	exonic	HIST1H1C	534	90	0.1442	Missense_Mutation	p.L107F	Targerted Panel Gene	GCB-DLBCL
GCB-10	chr6	26056114	26056114	A	T	exonic	HIST1H1C	528	87	0.1415	Missense_Mutation	p.F105L	Targerted Panel Gene	GCB-DLBCL
GCB-10	chr6	26217169	26217169	G	C	exonic	HIST1H2AE	44	11	0.2	Missense_Mutation	p.E65D	Targerted Panel Gene	GCB-DLBCL
GCB-10	chr6	26123673	26123673	C	G	exonic	HIST1H2BC	318	51	0.1371	Missense_Mutation	p.A78P	Targerted Panel Gene	GCB-DLBCL
GCB-10	chr6	26158261	26158261	A	G	exonic	HIST1H2BD	369	52	0.1235	Missense_Mutation	p.K31R	Targerted Panel Gene	GCB-DLBCL
GCB-10	chr6	26197049	26197049	A	G	exonic	HIST1H3D	157	28	0.1514	Missense_Mutation	p.F68L	Targerted Panel Gene	GCB-DLBCL
GCB-10	chr22	22888169	22888169	T	C	exonic	IGLL5	112	27	0.1942	Missense_Mutation	p.C4R	Targerted Panel Gene	GCB-DLBCL
GCB-10	chr22	22888061	22888061	C	G	exonic	IGLL5	210	26	0.1102	Missense_Mutation	p.P3R	Targerted Panel Gene	GCB-DLBCL
GCB-10	chr22	22888229	22888229	G	A	exonic	IGLL5	121	16	0.1168	Missense_Mutation	p.A24T	Targerted Panel Gene	GCB-DLBCL
GCB-10	chr22	22888064	22888064	A	T	exonic	IGLL5	210	26	0.1102	Missense_Mutation	p.K4M	Targerted Panel Gene	GCB-DLBCL
GCB-10	chr17	39792699	39792699	A	G	exonic	IKZF3	400	70	0.1489	Missense_Mutation	p.L133S	Targerted Panel Gene	GCB-DLBCL
GCB-10	chr16	27362290	27362290	T	G	exonic	IL4R	317	135	0.2987	Missense_Mutation	p.F298C	Targerted Panel Gene	GCB-DLBCL
GCB-10	chr2	151537906	151537906	A	G	exonic	NEB	304	57	0.1579	Missense_Mutation	p.F7023S	Targerted Panel Gene	GCB-DLBCL
GCB-10	chr7	82915865	82915865	G	A	exonic	PCLO	362	361	0.4986	Missense_Mutation	p.H4041Y	Targerted Panel Gene	GCB-DLBCL
GCB-10	chr1	204425735	204425735	G	A	exonic	PIK3C2B	526	89	0.1445	Missense_Mutation	p.L1532F	Targerted Panel Gene	GCB-DLBCL

GCB-10	chr22	49961776	49961776	C	T	exonic	PIM3	50	11	0.1803	Missense_Mutation	p.A194V	Targerted Panel Gene	GCB-DLBCL
GCB-10	chr8	109430037	109430037	G	A	exonic	PKHD1L1	376	61	0.1396	Missense_Mutation	p.G1077R	Targerted Panel Gene	GCB-DLBCL
GCB-10	chr10	87864537	87864537	T	C	exonic	PTEN	426	76	0.1514	Missense_Mutation	p.L196S	Targerted Panel Gene	GCB-DLBCL
GCB-10	chr3	16377873	16377873	C	T	exonic	RFTN1	401	72	0.1522	Missense_Mutation	p.S224N	Targerted Panel Gene	GCB-DLBCL
GCB-10	chr19	38458260	38458260	C	T	exonic	RYR1	259	54	0.1725	Missense_Mutation	p.S712F	Targerted Panel Gene	GCB-DLBCL
GCB-10	chr6	134174512	134174512	G	A	exonic	SGK1	307	28	0.0836	Missense_Mutation	p.H146Y	Targerted Panel Gene	GCB-DLBCL
GCB-10	chr6	134174586	134174586	G	A	exonic	SGK1	248	46	0.1565	Missense_Mutation	p.A121V	Targerted Panel Gene	GCB-DLBCL
GCB-10	chr6	134174576	134174576	C	G	exonic	SGK1	275	43	0.1348	Missense_Mutation	p.K124N	Targerted Panel Gene	GCB-DLBCL
GCB-10	chr6	134173517	134173517	G	A	exonic	SGK1	319	60	0.1583	Missense_Mutation	p.A188V	Targerted Panel Gene	GCB-DLBCL
GCB-10	chr6	134174581	134174581	T	C	exonic	SGK1	256	39	0.1322	Missense_Mutation	p.M123V	Targerted Panel Gene	GCB-DLBCL
GCB-10	chr6	134174026	134174026	C	T	exonic	SGK1	393	50	0.1129	Missense_Mutation	p.M164I	Targerted Panel Gene	GCB-DLBCL
GCB-10	chr16	11255429	11255429	G	A	exonic	SOCS1	148	27	0.1534	Missense_Mutation	p.A17V	Targerted Panel Gene	GCB-DLBCL
GCB-10	chr16	11255453	11255453	G	A	exonic	SOCS1	155	28	0.153	Missense_Mutation	p.A9V	Targerted Panel Gene	GCB-DLBCL
GCB-10	chr16	11255475	11255475	C	T	exonic	SOCS1	145	23	0.1369	Missense_Mutation	p.V2I	Targerted Panel Gene	GCB-DLBCL
GCB-10	chr16	11255039	11255039	A	T	exonic	SOCS1	139	19	0.1203	Missense_Mutation	p.L147H	Targerted Panel Gene	GCB-DLBCL
GCB-10	chr1	15848111	15848111	A	C	exonic	SPEN	173	20	0.1036	Missense_Mutation	p.E15A	Targerted Panel Gene	GCB-DLBCL
GCB-10	chr2	228027527	228027527	A	T	exonic	SPHKAP	368	54	0.128	Missense_Mutation	p.L88H	Targerted Panel Gene	GCB-DLBCL
GCB-10	chr6	137871474	137871474	C	G	exonic	TNFAIP3	579	128	0.181	Missense_Mutation	p.L83V	Targerted Panel Gene	GCB-DLBCL
GCB-10	chr17	59838329	59838329	C	G	exonic	VMP1	256	41	0.138	Missense_Mutation	p.P337A	Targerted Panel Gene	GCB-DLBCL
GCB-10	chr2	167251567	167251567	A	T	exonic	XIRP2	602	77	0.1134	Missense_Mutation	p.K3392I	Targerted Panel Gene	GCB-DLBCL
GCB-10	chr2	61492337	61492337	C	T	exonic	XPO1	569	74	0.1151	Missense_Mutation	p.E571K	Targerted Panel Gene	GCB-DLBCL
GCB-10	chr1	26731581	26731581	C	T	exonic	ARID1A	253	41	0.1395	Nonsense_Mutation	p.Q594X	Targerted Panel Gene	GCB-DLBCL
GCB-10	chr17	65056512	65056512	G	A	exonic	GNA13	186	22	0.1058	Nonsense_Mutation	p.Q28X	Targerted Panel Gene	GCB-DLBCL
GCB-10	chr6	26158272	26158272	A	T	exonic	HIST1H2BD	342	50	0.1276	Nonsense_Mutation	p.K35X	Targerted Panel Gene	GCB-DLBCL
GCB-10	chr1	15928770	15928770	C	T	exonic	SPEN	545	122	0.1829	Nonsense_Mutation	p.R844X	Targerted Panel Gene	GCB-DLBCL
GCB-10	chr6	137878789	137878789	G	A	exonic	TNFAIP3	351	60	0.146	Nonsense_Mutation	p.W448X	Targerted Panel Gene	GCB-DLBCL
GCB-11	chr6	73520999	73520999	C	G	splicing	EEF1A1	325	177	0.3512	Splice_Site	.	Targerted Panel Gene	GCB-DLBCL
GCB-11	chr6	134174587	134174587	C	G	splicing	SGK1	190	136	0.4159	Splice_Site	.	Targerted Panel Gene	GCB-DLBCL
GCB-11	chr18	63318395	63318395	G	A	exonic	BCL2	159	92	0.3651	Missense_Mutation	p.P91L	Targerted Panel Gene	GCB-DLBCL
GCB-11	chrX	101353936	101353936	G	A	exonic	BTK	41	428	0.9126	Missense_Mutation	p.R596W	Targerted Panel Gene	GCB-DLBCL
GCB-11	chr12	113058221	113058221	T	A	exonic	DTX1	198	104	0.3432	Missense_Mutation	p.M10K	Targerted Panel Gene	GCB-DLBCL
GCB-11	chr6	26156610	26156610	G	A	exonic	HIST1H1E	278	169	0.3781	Missense_Mutation	p.E74K	Targerted Panel Gene	GCB-DLBCL
GCB-11	chr1	185977840	185977840	A	T	exonic	HMCN1	130	572	0.8148	Missense_Mutation	p.N809Y	Targerted Panel Gene	GCB-DLBCL
GCB-11	chr12	40235634	40235634	T	C	exonic	LRRK2	235	190	0.4471	Missense_Mutation	p.L119P	Targerted Panel Gene	GCB-DLBCL
GCB-11	chr7	111124052	111124052	G	C	exonic	LRRN3	486	392	0.4465	Missense_Mutation	p.S427T	Targerted Panel Gene	GCB-DLBCL
GCB-11	chr7	111124354	111124354	A	G	exonic	LRRN3	534	369	0.4082	Missense_Mutation	p.K528E	Targerted Panel Gene	GCB-DLBCL
GCB-11	chr8	127738707	127738707	C	G	exonic	MYC	275	136	0.3309	Missense_Mutation	p.L164V	Targerted Panel Gene	GCB-DLBCL
GCB-11	chr6	37171322	37171322	C	G	exonic	PIM1	274	157	0.3643	Missense_Mutation	p.S146R	Targerted Panel Gene	GCB-DLBCL
GCB-11	chr6	134174586	134174586	G	C	exonic	SGK1	212	121	0.3623	Missense_Mutation	p.A121G	Targerted Panel Gene	GCB-DLBCL
GCB-11	chr6	134173045	134173045	A	G	exonic	SGK1	317	228	0.4183	Missense_Mutation	p.L271P	Targerted Panel Gene	GCB-DLBCL
GCB-11	chr6	134174547	134174547	A	G	exonic	SGK1	247	137	0.3568	Missense_Mutation	p.I134T	Targerted Panel Gene	GCB-DLBCL
GCB-11	chr17	7674241	7674241	G	A	exonic	TP53	66	127	0.658	Missense_Mutation	p.S202F	Targerted Panel Gene	GCB-DLBCL
GCB-11	chr7	98953291	98953291	G	A	exonic	TRRAP	217	123	0.3618	Missense_Mutation	p.S1856N	Targerted Panel Gene	GCB-DLBCL
GCB-11	chr10	63215594	63215594	G	A	exonic	JMJD1C	362	236	0.3933	Nonsense_Mutation	p.R261X	Targerted Panel Gene	GCB-DLBCL
GCB-11	chr12	49037359	49037359	G	A	exonic	KMT2D	276	168	0.3784	Nonsense_Mutation	p.Q3333X	Targerted Panel Gene	GCB-DLBCL
GCB-12	chr22	41169618	41169618	T	C	splicing	EP300	225	73	0.2441	Splice_Site	.	Targerted Panel Gene	GCB-DLBCL
GCB-12	chr4	105275863	105275863	A	-	exonic	TET2	97	377	0.7954	Frame_Shift_Del	p.K1785fs	Targerted Panel Gene	GCB-DLBCL
GCB-12	chr2	21022934	21022934	A	C	exonic	APOB	241	291	0.546	Missense_Mutation	p.F905V	Targerted Panel Gene	GCB-DLBCL
GCB-12	chr1	116544433	116544433	A	T	exonic	CD58	74	380	0.837	Missense_Mutation	p.V81D	Targerted Panel Gene	GCB-DLBCL
GCB-12	chr18	67511955	67511955	G	A	exonic	DSEL	241	334	0.5809	Missense_Mutation	p.T895I	Targerted Panel Gene	GCB-DLBCL
GCB-12	chr1	117623364	117623364	G	C	exonic	FAM46C	421	49	0.1043	Missense_Mutation	p.E166Q	Targerted Panel Gene	GCB-DLBCL
GCB-12	chr6	27867296	27867296	C	G	exonic	HIST1H1B	284	110	0.2792	Missense_Mutation	p.K78N	Targerted Panel Gene	GCB-DLBCL
GCB-12	chr22	22888069	22888069	G	A	exonic	IGLL5	79	104	0.5683	Missense_Mutation	p.G6S	Targerted Panel Gene	GCB-DLBCL
GCB-12	chr12	40251273	40251273	G	A	exonic	LRRK2	207	120	0.367	Missense_Mutation	p.E334K	Targerted Panel Gene	GCB-DLBCL
GCB-12	chr16	81895860	81895860	C	T	exonic	PLCG2	237	57	0.1939	Missense_Mutation	p.R376W	Targerted Panel Gene	GCB-DLBCL
GCB-12	chr19	22088126	22088126	T	C	exonic	ZNF257	65	11	0.1447	Missense_Mutation	p.C50R	Targerted Panel Gene	GCB-DLBCL
GCB-12	chr19	22088127	22088127	G	A	exonic	ZNF257	63	11	0.1486	Missense_Mutation	p.C50Y	Targerted Panel Gene	GCB-DLBCL
GCB-12	chr19	22088132	22088132	G	C	exonic	ZNF257	63	10	0.137	Missense_Mutation	p.G52R	Targerted Panel Gene	GCB-DLBCL
GCB-12	chr19	22088133	22088133	G	A	exonic	ZNF257	63	10	0.137	Missense_Mutation	p.G52E	Targerted Panel Gene	GCB-DLBCL
GCB-12	chr1	15931035	15931035	G	T	exonic	SPEN	53	272	0.8369	Nonsense_Mutation	p.E1599X	Targerted Panel Gene	GCB-DLBCL
GCB-13	chr4	186621776	186621776	C	T	splicing	FAT1	413	210	0.3365	Splice_Site	.	Targerted Panel Gene	GCB-DLBCL
GCB-13	chr6	134174510	134174510	C	T	splicing	SGK1	128	56	0.3027	Splice_Site	.	Targerted Panel Gene	GCB-DLBCL
GCB-13	chr4	105243568	105243568	A	-	splicing	TET2	151	95	0.3846	Splice_Site	.	Targerted Panel Gene	GCB-DLBCL
GCB-13	chr4	105235756	105235756	A	-	exonic	TET2	562	345	0.3804	Frame_Shift_Del	p.Y605fs	Targerted Panel Gene	GCB-DLBCL
GCB-13	chr6	134173312	134173312	-	GACTGCA	exonic	SGK1	394	137	0.258	Frame_Shift_Ins	p.K222fs	Targerted Panel Gene	GCB-DLBCL
GCB-13	chr3	187729365	187729365	G	A	exonic	BCL6	504	258	0.3377	Missense_Mutation	p.S347F	Targerted Panel Gene	GCB-DLBCL
GCB-13	chr8	60855993	60855993	C	T	exonic	CHD7	108	188	0.6351	Missense_Mutation	p.R2319C	Targerted Panel Gene	GCB-DLBCL
GCB-13	chr2	96144842	96144842	G	C	exonic	DUSP2	86	74	0.4625	Missense_Mutation	p.C143W	Targerted Panel Gene	GCB-DLBCL
GCB-13	chr2	96144990	96144990	G	C	exonic	DUSP2	42	35	0.4545	Missense_Mutation	p.P122R	Targerted Panel Gene	GCB-DLBCL
GCB-13	chr2	96144165	96144165	A	T	exonic	DUSP2	241	144	0.3731	Missense_Mutation	p.I240K	Targerted Panel Gene	GCB-DLBCL
GCB-13	chr16	85909042	85909042	C	T	exonic	IRF8	550	299	0.3522	Missense_Mutation	p.A76V	Targerted Panel Gene	GCB-DLBCL
GCB-13	chr1	114716123	114716123	C	T	exonic	NRAS	510	328	0.3909	Missense_Mutation	p.G13D	Targerted Panel Gene	GCB-DLBCL
GCB-13	chr1	114716126	114716126	C	G	exonic	NRAS	518	328	0.3877	Missense_Mutation	p.G12A	Targerted Panel Gene	GCB-DLBCL
GCB-13	chr9	37034027	37034027	T	G	exonic	PAX5	278	204	0.4232	Missense_Mutation	p.D2A	Targerted Panel Gene	GCB-DLBCL
GCB-13	chr6	128219079	128219079	A	C	exonic	PTPRK	421	216	0.3391	Missense_Mutation	p.D237E	Targerted Panel Gene	GCB-DLBCL
GCB-13	chr6	134174538	134174538	A	C	exonic	SGK1	111	81	0.4133	Missense_Mutation	p.I137S	Targerted Panel Gene	GCB-DLBCL
GCB-13	chr6	134174789	134174789	C	T	exonic	SGK1	120	231	0.6581	Missense_Mutation	p.A7T	Targerted Panel Gene	GCB-DLBCL
GCB-13	chr6	134174768	134174768	A	C	exonic	SGK1	116	224	0.6588	Missense_Mutation	p.Y14D	Targerted Panel Gene	GCB-DLBCL
GCB-13	chr6	134174576	134174576	C	G	exonic	SGK1	90	86	0.4886	Missense_Mutation	p.K124N	Targerted Panel Gene	GCB-DLBCL
GCB-13	chr6	134174586	134174586	G	A	exonic	SGK1	86	81	0.485	Missense_Mutation	p.A121V	Targerted Panel Gene	GCB-DLBCL
GCB-13	chr6	134174547	134174547	A	T	exonic	SGK1	111	87	0.4394	Missense_Mutation	p.I134N	Targerted Panel Gene	GCB-DLBCL
GCB-13	chr6	134174034	134174034	C	G	exonic	SGK1	177	144	0.4486	Missense_Mutation	p.E162Q	Targerted Panel Gene	GCB-DLBCL

GCB-13	chr6	134174571	134174571	C	G	exonic	SGK1	107	71	0.3989	Missense_Mutation	p.R126T	Targerted Panel Gene	GCB-DLBCL
GCB-13	chr6	134173322	134173322	G	C	exonic	SGK1	295	185	0.3838	Missense_Mutation	p.F218L	Targerted Panel Gene	GCB-DLBCL
GCB-13	chr6	134173139	134173139	A	G	exonic	SGK1	406	249	0.3796	Missense_Mutation	p.S240P	Targerted Panel Gene	GCB-DLBCL
GCB-13	chr6	134174520	134174520	G	T	exonic	SGK1	166	19	0.1022	Missense_Mutation	p.A143E	Targerted Panel Gene	GCB-DLBCL
GCB-13	chr19	18846112	18846112	G	T	exonic	UPF1	126	120	0.4878	Missense_Mutation	p.A122S	Targerted Panel Gene	GCB-DLBCL
GCB-13	chr17	65056395	65056395	G	A	exonic	GNA13	177	138	0.4381	Nonsense_Mutation	p.Q67X	Targerted Panel Gene	GCB-DLBCL
GCB-14	chr4	142429219	142429219	T	C	splicing	INPP4B	126	126	0.5	Splice_Site	.	Targerted Panel Gene	GCB-DLBCL
GCB-14	chr6	56624591	56624591	G	C	exonic	DST	137	143	0.5089	Missense_Mutation	p.T1590S	Targerted Panel Gene	GCB-DLBCL
GCB-14	chr7	148811636	148811636	A	T	exonic	EZH2	172	121	0.413	Missense_Mutation	p.Y646N	Targerted Panel Gene	GCB-DLBCL
GCB-14	chr16	27355862	27355862	T	A	exonic	IL4R	93	65	0.4114	Missense_Mutation	p.I227N	Targerted Panel Gene	GCB-DLBCL
GCB-14	chr16	85903082	85903082	T	C	exonic	IRF8	275	172	0.3848	Missense_Mutation	p.Y23H	Targerted Panel Gene	GCB-DLBCL
GCB-14	chr16	85903075	85903075	T	A	exonic	IRF8	272	169	0.3832	Missense_Mutation	p.S20R	Targerted Panel Gene	GCB-DLBCL
GCB-14	chr12	57200524	57200524	C	T	exonic	LRP1	147	163	0.5258	Missense_Mutation	p.P3366L	Targerted Panel Gene	GCB-DLBCL
GCB-14	chr2	169206385	169206385	C	A	exonic	LRP2	297	223	0.4288	Missense_Mutation	p.Q2445H	Targerted Panel Gene	GCB-DLBCL
GCB-14	chr4	133152161	133152161	T	A	exonic	PCDH10	88	62	0.4106	Missense_Mutation	p.L674Q	Targerted Panel Gene	GCB-DLBCL
GCB-14	chr15	33831014	33831014	G	A	exonic	RYR3	228	148	0.3936	Missense_Mutation	p.E3796K	Targerted Panel Gene	GCB-DLBCL
GCB-14	chr18	45063484	45063484	C	A	exonic	SETBP1	40	42	0.5122	Missense_Mutation	p.P1526Q	Targerted Panel Gene	GCB-DLBCL
GCB-14	chr11	108304822	108304822	C	T	exonic	ATM	264	145	0.3545	Nonsense_Mutation	p.R1882X	Targerted Panel Gene	GCB-DLBCL
GCB-15	chr12	49053474	49053474	A	G	splicing	KMT2D	159	115	0.4182	Splice_Site	.	Targerted Panel Gene	GCB-DLBCL
GCB-15	chr12	49034668	49034668	T	C	splicing	KMT2D	137	96	0.412	Splice_Site	.	Targerted Panel Gene	GCB-DLBCL
GCB-15	chr6	134174510	134174510	C	T	splicing	SGK1	89	168	0.6537	Splice_Site	.	Targerted Panel Gene	GCB-DLBCL
GCB-15	chr1	216072935	216072935	T	-	exonic	USH2A	236	293	0.5539	Frame_Shift_Del	p.G1937fs	Targerted Panel Gene	GCB-DLBCL
GCB-15	chr17	63482524	63482524	G	T	exonic	ACE	158	104	0.3969	Missense_Mutation	p.G393C	Targerted Panel Gene	GCB-DLBCL
GCB-15	chr22	23181124	23181124	G	A	exonic	BCR	97	69	0.4157	Missense_Mutation	p.R55H	Targerted Panel Gene	GCB-DLBCL
GCB-15	chr22	23181633	23181633	G	A	exonic	BCR	140	95	0.4025	Missense_Mutation	p.D225N	Targerted Panel Gene	GCB-DLBCL
GCB-15	chr7	2944513	2944513	G	A	exonic	CARD11	17	250	0.9363	Missense_Mutation	p.T128M	Targerted Panel Gene	GCB-DLBCL
GCB-15	chr16	3736766	3736766	A	G	exonic	CREBBP	75	428	0.8492	Missense_Mutation	p.Y1482H	Targerted Panel Gene	GCB-DLBCL
GCB-15	chr4	154305202	154305202	T	C	exonic	DCHS2	516	163	0.2401	Missense_Mutation	p.M1309V	Targerted Panel Gene	GCB-DLBCL
GCB-15	chr12	113058327	113058327	C	G	exonic	DTX1	153	122	0.4436	Missense_Mutation	p.C45W	Targerted Panel Gene	GCB-DLBCL
GCB-15	chr6	26216493	26216493	G	A	exonic	HIST1H2BG	89	92	0.5083	Missense_Mutation	p.P51S	Targerted Panel Gene	GCB-DLBCL
GCB-15	chr6	26216494	26216494	G	T	exonic	HIST1H2BG	92	91	0.4946	Missense_Mutation	p.H50Q	Targerted Panel Gene	GCB-DLBCL
GCB-15	chr1	149886409	149886409	C	A	exonic	HIST2H2BE	78	70	0.473	Missense_Mutation	p.A78S	Targerted Panel Gene	GCB-DLBCL
GCB-15	chr16	85909086	85909086	G	A	exonic	IRF8	515	376	0.4215	Missense_Mutation	p.D91N	Targerted Panel Gene	GCB-DLBCL
GCB-15	chr19	19149236	19149236	T	A	exonic	MEF2B,MEF2BNB-I	174	117	0.4021	Missense_Mutation	p.D83V	Targerted Panel Gene	GCB-DLBCL
GCB-15	chr11	111379165	111379165	T	C	exonic	POU2AF1	630	161	0.2035	Missense_Mutation	p.K5E	Targerted Panel Gene	GCB-DLBCL
GCB-15	chr21	41836603	41836603	T	A	exonic	PRDM15	260	171	0.3968	Missense_Mutation	p.I716F	Targerted Panel Gene	GCB-DLBCL
GCB-15	chr21	41836611	41836611	C	A	exonic	PRDM15	263	169	0.3912	Missense_Mutation	p.R713M	Targerted Panel Gene	GCB-DLBCL
GCB-15	chr6	134174576	134174576	C	G	exonic	SGK1	91	154	0.6286	Missense_Mutation	p.K124N	Targerted Panel Gene	GCB-DLBCL
GCB-15	chr2	167244820	167244820	C	A	exonic	XIRP2	326	338	0.509	Missense_Mutation	p.S1143Y	Targerted Panel Gene	GCB-DLBCL
GCB-15	chr19	30445702	30445702	A	G	exonic	ZNF536	55	37	0.4022	Missense_Mutation	p.S714G	Targerted Panel Gene	GCB-DLBCL
GCB-16	chr12	85128034	85128034	G	A	splicing	LRRIQ1	401	68	0.145	Splice_Site	.	Targerted Panel Gene	GCB-DLBCL
GCB-16	chr15	44711581	44711581	T	A	exonic	B2M	134	43	0.2429	Missense_Mutation	p.L12Q	Targerted Panel Gene	GCB-DLBCL
GCB-16	chr15	44711548	44711548	T	A	exonic	B2M	157	31	0.1649	Missense_Mutation	p.M1K	Targerted Panel Gene	GCB-DLBCL
GCB-16	chr7	140753336	140753336	A	T	exonic	BRAF	438	93	0.1751	Missense_Mutation	p.V600E	Targerted Panel Gene	GCB-DLBCL
GCB-16	chr1	203305739	203305739	G	A	exonic	BTG2	85	17	0.1667	Missense_Mutation	p.A45T	Targerted Panel Gene	GCB-DLBCL
GCB-16	chr7	82170593	82170593	G	A	exonic	CACNA2D1	288	57	0.1652	Missense_Mutation	p.A104V	Targerted Panel Gene	GCB-DLBCL
GCB-16	chr7	2944529	2944529	C	T	exonic	CARD11	123	24	0.1633	Missense_Mutation	p.G123S	Targerted Panel Gene	GCB-DLBCL
GCB-16	chrX	41343285	41343285	C	G	exonic	DDX3X	312	67	0.1768	Missense_Mutation	p.P205A	Targerted Panel Gene	GCB-DLBCL
GCB-16	chr2	96144336	96144336	C	T	exonic	DUSP2	211	22	0.0944	Missense_Mutation	p.G183D	Targerted Panel Gene	GCB-DLBCL
GCB-16	chr4	125415777	125415777	T	G	exonic	FAT4	397	69	0.1481	Missense_Mutation	p.L2272V	Targerted Panel Gene	GCB-DLBCL
GCB-16	chr6	26123723	26123723	C	T	exonic	HIST1H2BC	294	59	0.1671	Missense_Mutation	p.G61D	Targerted Panel Gene	GCB-DLBCL
GCB-16	chr6	26273108	26273108	G	A	exonic	HIST1H2BI	96	25	0.2066	Missense_Mutation	p.V45M	Targerted Panel Gene	GCB-DLBCL
GCB-16	chr6	27132640	27132640	G	C	exonic	HIST1H2BJ	102	20	0.1639	Missense_Mutation	p.S37R	Targerted Panel Gene	GCB-DLBCL
GCB-16	chr22	22888174	22888174	C	T	exonic	IGLL5	139	30	0.1775	Missense_Mutation	p.P41S	Targerted Panel Gene	GCB-DLBCL
GCB-16	chr3	183492091	183492091	C	T	exonic	KLHL6	234	49	0.1725	Missense_Mutation	p.E568K	Targerted Panel Gene	GCB-DLBCL
GCB-16	chr12	25245345	25245345	C	T	exonic	KRAS	196	51	0.2065	Missense_Mutation	p.V14I	Targerted Panel Gene	GCB-DLBCL
GCB-16	chr16	88725505	88725505	C	T	exonic	PIEZO1	119	29	0.1959	Missense_Mutation	p.R1358H	Targerted Panel Gene	GCB-DLBCL
GCB-16	chr6	134174073	134174073	C	T	exonic	SGK1	257	49	0.1601	Missense_Mutation	p.V149I	Targerted Panel Gene	GCB-DLBCL
GCB-16	chr6	134175655	134175655	C	G	exonic	SGK1	172	36	0.1731	Missense_Mutation	p.E3Q	Targerted Panel Gene	GCB-DLBCL
GCB-16	chr6	134173150	134173150	T	C	exonic	SGK1	453	85	0.158	Missense_Mutation	p.K236R	Targerted Panel Gene	GCB-DLBCL
GCB-16	chr6	134173326	134173326	A	T	exonic	SGK1	340	62	0.1542	Missense_Mutation	p.V217E	Targerted Panel Gene	GCB-DLBCL
GCB-16	chr6	134173566	134173566	G	A	exonic	SGK1	183	31	0.1435	Missense_Mutation	p.P172S	Targerted Panel Gene	GCB-DLBCL
GCB-16	chr6	134174078	134174078	G	A	exonic	SGK1	258	40	0.1342	Missense_Mutation	p.P147L	Targerted Panel Gene	GCB-DLBCL
GCB-16	chr6	134174080	134174080	G	T	exonic	SGK1	254	38	0.1293	Missense_Mutation	p.H146Q	Targerted Panel Gene	GCB-DLBCL
GCB-16	chr6	134174752	134174752	C	A	exonic	SGK1	259	36	0.122	Missense_Mutation	p.G19V	Targerted Panel Gene	GCB-DLBCL
GCB-16	chr6	134173472	134173472	C	G	exonic	SGK1	307	37	0.1076	Missense_Mutation	p.S203T	Targerted Panel Gene	GCB-DLBCL
GCB-16	chrX	71367527	71367527	G	A	exonic	TAF1	235	46	0.1637	Missense_Mutation	p.G70E	Targerted Panel Gene	GCB-DLBCL
GCB-16	chr6	56466174	56466174	G	A	exonic	DST	335	59	0.1497	Nonsense_Mutation	p.R5412X	Targerted Panel Gene	GCB-DLBCL
GCB-17	chr2	51026369	51026369	A	C	splicing	NRXN1	536	61	0.1022	Splice_Site	.	Targerted Panel Gene	GCB-DLBCL
GCB-17	chr16	30979919	30979919	-	CAG	exonic	SETD1A	93	69	0.4157	In_Frame_Ins	p.D1378delinsDS	Targerted Panel Gene	GCB-DLBCL
GCB-17	chr2	215010374	215010374	A	G	exonic	ABCA12	679	83	0.1089	Missense_Mutation	p.L810S	Targerted Panel Gene	GCB-DLBCL
GCB-17	chr20	32436739	32436739	A	T	exonic	ASXL1	567	75	0.1161	Missense_Mutation	p.N1343Y	Targerted Panel Gene	GCB-DLBCL
GCB-17	chr15	44711581	44711581	T	A	exonic	B2M	206	31	0.1308	Missense_Mutation	p.L12Q	Targerted Panel Gene	GCB-DLBCL
GCB-17	chr12	122022122	122022122	A	G	exonic	BCL7A	198	37	0.1574	Missense_Mutation	p.R11G	Targerted Panel Gene	GCB-DLBCL
GCB-17	chr10	71805842	71805842	G	A	exonic	CDH23	280	40	0.125	Missense_Mutation	p.A2637T	Targerted Panel Gene	GCB-DLBCL
GCB-17	chr8	4637400	4637400	C	T	exonic	CSMD1	518	91	0.1494	Missense_Mutation	p.D82N	Targerted Panel Gene	GCB-DLBCL
GCB-17	chrX	32217018	32217018	A	C	exonic	DMD	335	151	0.3107	Missense_Mutation	p.D1989E	Targerted Panel Gene	GCB-DLBCL
GCB-17	chrX	32348409	32348409	A	T	exonic	DMD	359	83	0.1878	Missense_Mutation	p.D1692E	Targerted Panel Gene	GCB-DLBCL
GCB-17	chr2	96144852	96144852	G	A	exonic	DUSP2	94	22	0.1897	Missense_Mutation	p.P140L	Targerted Panel Gene	GCB-DLBCL
GCB-17	chr11	70206196	70206196	G	A	exonic	FADD	472	146	0.2362	Missense_Mutation	p.R117H	Targerted Panel Gene	GCB-DLBCL
GCB-17	chr6	159232932	159232932	C	T	exonic	FNDC1	387	39	0.0915	Missense_Mutation	p.T807M	Targerted Panel Gene	GCB-DLBCL
GCB-17	chr3	50257034	50257034	T	C	exonic	GNAI2	354	54	0.1324	Missense_Mutation	p.L258P	Targerted Panel Gene	GCB-DLBCL

GCB-17	chr6	27867162	27867162	G	A	exonic	HIST1H1B	544	102	0.1579	Missense_Mutation	p.A123V	Targerted Panel Gene	GCB-DLBCL
GCB-17	chr6	27865694	27865694	G	A	exonic	HIST1H2AL	97	22	0.1833	Missense_Mutation	p.A114T	Targerted Panel Gene	GCB-DLBCL
GCB-17	chr6	26216533	26216533	G	C	exonic	HIST1H2BG	102	11	0.0973	Missense_Mutation	p.S37R	Targerted Panel Gene	GCB-DLBCL
GCB-17	chr22	22888141	22888141	G	C	exonic	IGLL5	178	32	0.1524	Missense_Mutation	p.G30R	Targerted Panel Gene	GCB-DLBCL
GCB-17	chr3	183555604	183555604	T	G	exonic	KLHL6	644	98	0.1321	Missense_Mutation	p.E17A	Targerted Panel Gene	GCB-DLBCL
GCB-17	chr3	183555646	183555646	A	C	exonic	KLHL6	572	83	0.1267	Missense_Mutation	p.M3R	Targerted Panel Gene	GCB-DLBCL
GCB-17	chr2	169242989	169242989	C	A	exonic	LRP2	612	108	0.1496	Missense_Mutation	p.D1212Y	Targerted Panel Gene	GCB-DLBCL
GCB-17	chr2	169138588	169138588	A	G	exonic	LRP2	460	61	0.1171	Missense_Mutation	p.S4503P	Targerted Panel Gene	GCB-DLBCL
GCB-17	chr12	40284086	40284086	T	G	exonic	LRRK2	645	189	0.2266	Missense_Mutation	p.L818R	Targerted Panel Gene	GCB-DLBCL
GCB-17	chr17	16070216	16070216	A	T	exonic	NCOR1	496	84	0.1448	Missense_Mutation	p.Y1488N	Targerted Panel Gene	GCB-DLBCL
GCB-17	chr2	51026393	51026393	A	C	exonic	NRXN1	559	66	0.1054	Missense_Mutation	p.N283K	Targerted Panel Gene	GCB-DLBCL
GCB-17	chr14	79280419	79280419	C	T	exonic	NRXN3	698	80	0.1027	Missense_Mutation	p.S43F	Targerted Panel Gene	GCB-DLBCL
GCB-17	chr6	37170481	37170481	A	G	exonic	PIM1	114	16	0.1231	Missense_Mutation	p.H60R	Targerted Panel Gene	GCB-DLBCL
GCB-17	chr9	8636745	8636745	A	T	exonic	PTPRD	269	42	0.135	Missense_Mutation	p.I55N	Targerted Panel Gene	GCB-DLBCL
GCB-17	chr2	1660904	1660904	A	T	exonic	PXDN	409	67	0.1408	Missense_Mutation	p.V605E	Targerted Panel Gene	GCB-DLBCL
GCB-17	chr19	38460511	38460511	C	G	exonic	RYR1	251	32	0.1131	Missense_Mutation	p.P833A	Targerted Panel Gene	GCB-DLBCL
GCB-17	chr18	44950640	44950640	G	A	exonic	SETBP1	751	110	0.1278	Missense_Mutation	p.E434K	Targerted Panel Gene	GCB-DLBCL
GCB-17	chr6	134174076	134174076	C	G	exonic	SGK1	388	48	0.1101	Missense_Mutation	p.E148Q	Targerted Panel Gene	GCB-DLBCL
GCB-17	chr12	57102879	57102879	C	G	exonic	STAT6	339	89	0.2079	Missense_Mutation	p.D419H	Targerted Panel Gene	GCB-DLBCL
GCB-17	chr5	168124958	168124958	G	A	exonic	TENM2	280	47	0.1433	Missense_Mutation	p.C706Y	Targerted Panel Gene	GCB-DLBCL
GCB-17	chr1	215799038	215799038	G	A	exonic	USH2A	655	83	0.1125	Missense_Mutation	p.S3276L	Targerted Panel Gene	GCB-DLBCL
GCB-17	chr16	72793537	72793537	T	C	exonic	ZFHX3	885	124	0.1229	Missense_Mutation	p.I3049V	Targerted Panel Gene	GCB-DLBCL
GCB-17	chr19	30444140	30444140	G	A	exonic	ZNF536	196	22	0.1009	Missense_Mutation	p.R193H	Targerted Panel Gene	GCB-DLBCL
GCB-17	chr1	116544500	116544500	G	A	exonic	CD58	743	106	0.1247	Nonsense_Mutation	p.Q59X	Targerted Panel Gene	GCB-DLBCL
GCB-17	chr5	138338020	138338020	C	A	exonic	CDC25C	540	71	0.1162	Nonsense_Mutation	p.E46X	Targerted Panel Gene	GCB-DLBCL
GCB-17	chr3	183555529	183555529	A	C	exonic	KLHL6	732	113	0.1337	Nonsense_Mutation	p.L42X	Targerted Panel Gene	GCB-DLBCL
GCB-17	chr6	44265304	44265304	G	A	exonic	NFKBIE	167	32	0.1608	Nonsense_Mutation	p.Q154X	Targerted Panel Gene	GCB-DLBCL
GCB-17	chr1	2561682	2561682	G	A	exonic	TNFRSF14	226	96	0.2981	Nonsense_Mutation	p.W187X	Targerted Panel Gene	GCB-DLBCL
GCB-18	chr1	228316985	228316985	G	A	splicing	OBSCN	62	39	0.3861	Splice_Site	.	Targerted Panel Gene	GCB-DLBCL
GCB-18	chr11	108343279	108343279	A	T	exonic	ATM	777	103	0.1169	Missense_Mutation	p.I2776F	Targerted Panel Gene	GCB-DLBCL
GCB-18	chr1	102935113	102935113	C	T	exonic	COL11A1	239	38	0.1372	Missense_Mutation	p.G1159S	Targerted Panel Gene	GCB-DLBCL
GCB-18	chr5	138465998	138465998	C	A	exonic	EGR1	109	46	0.2968	Missense_Mutation	p.S79R	Targerted Panel Gene	GCB-DLBCL
GCB-18	chr7	148811636	148811636	A	T	exonic	EZH2	469	89	0.1595	Missense_Mutation	p.Y646N	Targerted Panel Gene	GCB-DLBCL
GCB-18	chr4	125448660	125448660	G	T	exonic	FAT4	802	136	0.1448	Missense_Mutation	p.K2548N	Targerted Panel Gene	GCB-DLBCL
GCB-18	chr3	65361286	65361286	G	A	exonic	MAGI1	670	99	0.1287	Missense_Mutation	p.H1211Y	Targerted Panel Gene	GCB-DLBCL
GCB-18	chr1	75898028	75898028	C	T	exonic	MSH4	517	77	0.1296	Missense_Mutation	p.A826V	Targerted Panel Gene	GCB-DLBCL
GCB-18	chr8	127738602	127738602	A	G	exonic	MYC	499	72	0.1261	Missense_Mutation	p.S129G	Targerted Panel Gene	GCB-DLBCL
GCB-18	chr20	8774611	8774611	G	T	exonic	PLCB1	447	136	0.2333	Missense_Mutation	p.M1001I	Targerted Panel Gene	GCB-DLBCL
GCB-18	chr10	87864153	87864153	C	G	exonic	PTEN	100	14	0.1217	Missense_Mutation	p.A68G	Targerted Panel Gene	GCB-DLBCL
GCB-18	chr10	87864211	87864211	T	A	exonic	PTEN	103	14	0.1197	Missense_Mutation	p.D87E	Targerted Panel Gene	GCB-DLBCL
GCB-18	chr10	87864143	87864143	T	G	exonic	PTEN	108	14	0.1148	Missense_Mutation	p.C65G	Targerted Panel Gene	GCB-DLBCL
GCB-18	chr9	8437229	8437229	A	T	exonic	PTPRD	186	24	0.1143	Missense_Mutation	p.D912E	Targerted Panel Gene	GCB-DLBCL
GCB-18	chr9	8437209	8437209	A	C	exonic	PTPRD	204	26	0.1126	Missense_Mutation	p.L919R	Targerted Panel Gene	GCB-DLBCL
GCB-18	chr7	96277238	96277238	G	A	exonic	SLC25A13	823	159	0.1619	Missense_Mutation	p.T57I	Targerted Panel Gene	GCB-DLBCL
GCB-18	chr4	20523887	20523887	C	T	exonic	SLIT2	798	116	0.1269	Missense_Mutation	p.R420W	Targerted Panel Gene	GCB-DLBCL
GCB-18	chr2	230269854	230269854	T	A	exonic	SP140	398	41	0.0934	Missense_Mutation	p.Y449N	Targerted Panel Gene	GCB-DLBCL
GCB-18	chr15	42684277	42684277	A	G	exonic	STARD9	171	105	0.3804	Missense_Mutation	p.H900R	Targerted Panel Gene	GCB-DLBCL
GCB-18	chr15	44711548	44711548	T	G	exonic	B2M	159	35	0.1549	Missense_Mutation	p.M1R	Targerted Panel Gene	GCB-DLBCL
GCB-18	chr17	31993873	31993873	T	G	exonic	SUZ12	481	75	0.1349	Nonsense_Mutation	p.Y434X	Targerted Panel Gene	GCB-DLBCL
GCB-18	chr17	31993954	31993954	T	G	exonic	SUZ12	640	85	0.1172	Nonsense_Mutation	p.Y461X	Targerted Panel Gene	GCB-DLBCL
GCB-18	chr1	2560707	2560707	C	T	exonic	TNFRSF14	95	56	0.3709	Nonsense_Mutation	p.Q182X	Targerted Panel Gene	GCB-DLBCL
GCB-19	chr7	82847248	82847248	C	A	splicing	PCLO	173	32	0.1561	Splice_Site	.	Targerted Panel Gene	GCB-DLBCL
GCB-19	chr1	215743336	215743336	C	A	splicing	USH2A	328	47	0.1253	Splice_Site	.	Targerted Panel Gene	GCB-DLBCL
GCB-19	chr6	157196306	157196306	A	G	exonic	ARID1B	335	59	0.1497	Missense_Mutation	p.Y1335C	Targerted Panel Gene	GCB-DLBCL
GCB-19	chr3	187722574	187722574	G	A	exonic	BCL6	568	100	0.1495	Missense_Mutation	p.H669Y	Targerted Panel Gene	GCB-DLBCL
GCB-19	chr12	122022161	122022161	G	C	exonic	BCL7A	149	23	0.1337	Missense_Mutation	p.A24P	Targerted Panel Gene	GCB-DLBCL
GCB-19	chr5	71522333	71522333	G	C	exonic	BDP1	567	88	0.1344	Missense_Mutation	p.R1679T	Targerted Panel Gene	GCB-DLBCL
GCB-19	chr7	81965611	81965611	C	T	exonic	CACNA2D1	542	99	0.1542	Missense_Mutation	p.D853N	Targerted Panel Gene	GCB-DLBCL
GCB-19	chr1	116570971	116570971	A	G	exonic	CD58	77	16	0.172	Missense_Mutation	p.M1T	Targerted Panel Gene	GCB-DLBCL
GCB-19	chr1	103002472	103002472	C	A	exonic	COL11A1	362	75	0.1716	Missense_Mutation	p.G697W	Targerted Panel Gene	GCB-DLBCL
GCB-19	chr4	154490362	154490362	G	A	exonic	DCHS2	97	18	0.1565	Missense_Mutation	p.R332C	Targerted Panel Gene	GCB-DLBCL
GCB-19	chrX	31507386	31507386	A	T	exonic	DMD	254	86	0.2529	Missense_Mutation	p.I2639N	Targerted Panel Gene	GCB-DLBCL
GCB-19	chr3	135132986	135132986	G	A	exonic	EPHB1	305	321	0.5128	Missense_Mutation	p.V412I	Targerted Panel Gene	GCB-DLBCL
GCB-19	chr11	92799591	92799591	C	T	exonic	FAT3	958	137	0.1251	Missense_Mutation	p.A2193V	Targerted Panel Gene	GCB-DLBCL
GCB-19	chr4	93769283	93769283	G	A	exonic	GRID2	364	83	0.1849	Missense_Mutation	p.D812N	Targerted Panel Gene	GCB-DLBCL
GCB-19	chr6	26156881	26156881	C	G	exonic	HIST1H1E	466	82	0.1496	Missense_Mutation	p.A164G	Targerted Panel Gene	GCB-DLBCL
GCB-19	chr6	26156563	26156563	C	T	exonic	HIST1H1E	377	53	0.1233	Missense_Mutation	p.S58F	Targerted Panel Gene	GCB-DLBCL
GCB-19	chr1	186130031	186130031	G	T	exonic	HMCN1	694	116	0.1432	Missense_Mutation	p.G4324C	Targerted Panel Gene	GCB-DLBCL
GCB-19	chrX	53603384	53603384	G	C	exonic	HUWE1	224	88	0.2812	Missense_Mutation	p.L954V	Targerted Panel Gene	GCB-DLBCL
GCB-19	chr11	118488639	118488639	A	T	exonic	KMT2A	618	231	0.2718	Missense_Mutation	p.E1453V	Targerted Panel Gene	GCB-DLBCL
GCB-19	chr12	85038199	85038199	T	C	exonic	LRRIQ1	485	66	0.1198	Missense_Mutation	p.L8P	Targerted Panel Gene	GCB-DLBCL
GCB-19	chr1	235788844	235788844	C	A	exonic	LYST	531	78	0.1281	Missense_Mutation	p.E1515D	Targerted Panel Gene	GCB-DLBCL
GCB-19	chr10	128105906	128105906	T	G	exonic	MKI67	1203	193	0.1382	Missense_Mutation	p.E1978D	Targerted Panel Gene	GCB-DLBCL
GCB-19	chr13	101107719	101107719	T	C	exonic	NALCN	731	95	0.115	Missense_Mutation	p.K812R	Targerted Panel Gene	GCB-DLBCL
GCB-19	chr12	78159215	78159215	G	T	exonic	NAV3	436	75	0.1468	Missense_Mutation	p.A1600S	Targerted Panel Gene	GCB-DLBCL
GCB-19	chr18	79486640	79486640	C	T	exonic	NFATC1	136	45	0.2486	Missense_Mutation	p.P816S	Targerted Panel Gene	GCB-DLBCL
GCB-19	chr6	17629500	17629500	G	T	exonic	NUP153	705	116	0.1413	Missense_Mutation	p.S931Y	Targerted Panel Gene	GCB-DLBCL
GCB-19	chr7	83135009	83135009	G	T	exonic	PCLO	1088	189	0.148	Missense_Mutation	p.D847E	Targerted Panel Gene	GCB-DLBCL
GCB-19	chr1	233208535	233208535	G	A	exonic	PCNXL2	600	76	0.1124	Missense_Mutation	p.A949V	Targerted Panel Gene	GCB-DLBCL
GCB-19	chr16	88721249	88721249	C	A	exonic	PIEZO1	551	73	0.1166	Missense_Mutation	p.R1862M	Targerted Panel Gene	GCB-DLBCL
GCB-19	chr1	9720773	9720773	G	A	exonic	PIK3CD	64	81	0.5586	Missense_Mutation	p.R518Q	Targerted Panel Gene	GCB-DLBCL
GCB-19	chr8	109396120	109396120	T	C	exonic	PKHD1L1	493	86	0.1485	Missense_Mutation	p.V302A	Targerted Panel Gene	GCB-DLBCL

GCB-19	chr10	94270583	94270583	A	G	exonic	PLCE1	342	48	0.1231	Missense_Mutation	p.K1496R	Targerted Panel Gene	GCB-DLBCL
GCB-19	chr1	237649959	237649959	C	A	exonic	RYR2	700	87	0.1104	Missense_Mutation	p.A2532D	Targerted Panel Gene	GCB-DLBCL
GCB-19	chr16	11255111	11255111	G	A	exonic	SOCS1	156	25	0.1381	Missense_Mutation	p.P123L	Targerted Panel Gene	GCB-DLBCL
GCB-19	chr16	11254950	11254950	G	C	exonic	SOCS1	168	35	0.1724	Missense_Mutation	p.L177V	Targerted Panel Gene	GCB-DLBCL
GCB-19	chr16	11255063	11255063	C	T	exonic	SOCS1	180	28	0.1346	Missense_Mutation	p.G139D	Targerted Panel Gene	GCB-DLBCL
GCB-19	chr16	11255460	11255460	C	T	exonic	SOCS1	114	17	0.1298	Missense_Mutation	p.V7M	Targerted Panel Gene	GCB-DLBCL
GCB-19	chr16	11255199	11255199	G	C	exonic	SOCS1	107	26	0.1955	Missense_Mutation	p.R94G	Targerted Panel Gene	GCB-DLBCL
GCB-19	chr16	11255147	11255147	C	G	exonic	SOCS1	124	30	0.1948	Missense_Mutation	p.C111S	Targerted Panel Gene	GCB-DLBCL
GCB-19	chr16	11254910	11254910	T	G	exonic	SOCS1	200	31	0.1342	Missense_Mutation	p.N190T	Targerted Panel Gene	GCB-DLBCL
GCB-19	chr16	11255104	11255104	G	C	exonic	SOCS1	164	24	0.127	Missense_Mutation	p.S125R	Targerted Panel Gene	GCB-DLBCL
GCB-19	chr19	55669165	55669165	C	T	exonic	U2AF2	110	20	0.1538	Missense_Mutation	p.T343M	Targerted Panel Gene	GCB-DLBCL
GCB-19	chr1	216084786	216084786	C	T	exonic	USH2A	579	100	0.1473	Nonsense_Mutation	p.W1693X	Targerted Panel Gene	GCB-DLBCL
GCB-19	chr6	27865745	27865745	T	G	exonic	HIST1H2AL	79	23	0.2255	Nonstop_Mutation	p.X131E	Targerted Panel Gene	GCB-DLBCL
GCB-2	chr18	63319172	63319172	A	T	splicing	BCL2	150	78	0.3421	Splice_Site	.	Targerted Panel Gene	GCB-DLBCL
GCB-2	chr12	49054306	49054306	C	G	splicing	KMT2D	246	41	0.1429	Splice_Site	.	Targerted Panel Gene	GCB-DLBCL
GCB-2	chr18	63318275	63318275	G	A	exonic	BCL2	174	56	0.2435	Missense_Mutation	p.A131V	Targerted Panel Gene	GCB-DLBCL
GCB-2	chr18	63318281	63318281	C	T	exonic	BCL2	174	56	0.2435	Missense_Mutation	p.R129H	Targerted Panel Gene	GCB-DLBCL
GCB-2	chr18	63318060	63318060	T	A	exonic	BCL2	252	69	0.215	Missense_Mutation	p.S203C	Targerted Panel Gene	GCB-DLBCL
GCB-2	chr18	63318653	63318653	C	T	exonic	BCL2	264	82	0.237	Missense_Mutation	p.G5E	Targerted Panel Gene	GCB-DLBCL
GCB-2	chr18	63318267	63318267	C	G	exonic	BCL2	196	54	0.216	Missense_Mutation	p.V134L	Targerted Panel Gene	GCB-DLBCL
GCB-2	chr18	63318053	63318053	C	T	exonic	BCL2	258	68	0.2086	Missense_Mutation	p.G205D	Targerted Panel Gene	GCB-DLBCL
GCB-2	chr18	63318130	63318130	C	G	exonic	BCL2	273	67	0.1971	Missense_Mutation	p.E179D	Targerted Panel Gene	GCB-DLBCL
GCB-2	chr12	122022124	122022124	G	T	exonic	BCL7A	181	49	0.213	Missense_Mutation	p.R11S	Targerted Panel Gene	GCB-DLBCL
GCB-2	chr12	122022173	122022173	G	T	exonic	BCL7A	167	35	0.1733	Missense_Mutation	p.V28L	Targerted Panel Gene	GCB-DLBCL
GCB-2	chr22	23181066	23181066	G	C	exonic	BCR	142	16	0.1013	Missense_Mutation	p.E36Q	Targerted Panel Gene	GCB-DLBCL
GCB-2	chr16	3757897	3757897	A	C	exonic	CREBBP	333	55	0.1418	Missense_Mutation	p.V1174G	Targerted Panel Gene	GCB-DLBCL
GCB-2	chr4	154334985	154334985	T	C	exonic	DCHS2	423	126	0.2291	Missense_Mutation	p.I367V	Targerted Panel Gene	GCB-DLBCL
GCB-2	chr12	113058403	113058403	C	G	exonic	DTX1	169	38	0.1836	Missense_Mutation	p.P71A	Targerted Panel Gene	GCB-DLBCL
GCB-2	chr5	138465958	138465958	G	T	exonic	EGR1	75	39	0.3421	Missense_Mutation	p.S66I	Targerted Panel Gene	GCB-DLBCL
GCB-2	chr12	50351365	50351365	C	T	exonic	FAM186A	359	70	0.1632	Missense_Mutation	p.G1823R	Targerted Panel Gene	GCB-DLBCL
GCB-2	chr6	26156862	26156862	G	A	exonic	HIST1H1E	194	134	0.4085	Missense_Mutation	p.A158T	Targerted Panel Gene	GCB-DLBCL
GCB-2	chr6	26156991	26156991	C	T	exonic	HIST1H1E	292	100	0.2545	Missense_Mutation	p.P201S	Targerted Panel Gene	GCB-DLBCL
GCB-2	chr6	26123833	26123833	C	G	exonic	HIST1H2BC	136	28	0.1697	Missense_Mutation	p.K24N	Targerted Panel Gene	GCB-DLBCL
GCB-2	chr6	27146609	27146609	T	C	exonic	HIST1H2BK	93	83	0.4716	Missense_Mutation	p.N64D	Targerted Panel Gene	GCB-DLBCL
GCB-2	chr6	27146615	27146615	T	A	exonic	HIST1H2BK	97	80	0.452	Missense_Mutation	p.I62F	Targerted Panel Gene	GCB-DLBCL
GCB-2	chr12	49026292	49026292	C	G	exonic	KMT2D	405	65	0.138	Missense_Mutation	p.R5225P	Targerted Panel Gene	GCB-DLBCL
GCB-2	chr7	111124351	111124351	A	T	exonic	LRRN3	614	73	0.1063	Missense_Mutation	p.I527F	Targerted Panel Gene	GCB-DLBCL
GCB-2	chr19	19149236	19149236	T	A	exonic	MEF2B,MEF2BNB-I	274	57	0.1722	Missense_Mutation	p.D83V	Targerted Panel Gene	GCB-DLBCL
GCB-2	chr2	151519680	151519680	T	C	exonic	NEB	173	145	0.4545	Missense_Mutation	p.D7558G	Targerted Panel Gene	GCB-DLBCL
GCB-2	chr7	30446189	30446189	T	C	exonic	NOD1	304	96	0.24	Missense_Mutation	p.K802R	Targerted Panel Gene	GCB-DLBCL
GCB-2	chr1	228280776	228280776	G	A	exonic	OBSCN	162	23	0.1243	Missense_Mutation	p.R3155Q	Targerted Panel Gene	GCB-DLBCL
GCB-2	chr5	141491755	141491755	G	T	exonic	PCDHGC5	260	82	0.2398	Missense_Mutation	p.A839S	Targerted Panel Gene	GCB-DLBCL
GCB-2	chr1	9724421	9724421	G	A	exonic	PIK3CD	195	36	0.1558	Missense_Mutation	p.R955Q	Targerted Panel Gene	GCB-DLBCL
GCB-2	chr7	103603468	103603468	T	C	exonic	RELN	378	306	0.4467	Missense_Mutation	p.T1057A	Targerted Panel Gene	GCB-DLBCL
GCB-2	chr3	49368502	49368502	C	T	exonic	RHOA	296	61	0.1709	Missense_Mutation	p.R68H	Targerted Panel Gene	GCB-DLBCL
GCB-2	chr6	134173031	134173031	C	A	exonic	SGK1	245	75	0.2336	Missense_Mutation	p.G276C	Targerted Panel Gene	GCB-DLBCL
GCB-2	chr3	177050130	177050130	T	A	exonic	TBL1XR1	232	107	0.3156	Missense_Mutation	p.D190V	Targerted Panel Gene	GCB-DLBCL
GCB-2	chr19	30445380	30445380	T	G	exonic	ZNF536	508	143	0.2193	Missense_Mutation	p.D606E	Targerted Panel Gene	GCB-DLBCL
GCB-2	chr19	23361709	23361709	G	A	exonic	ZNF91	160	112	0.4118	Missense_Mutation	p.H424Y	Targerted Panel Gene	GCB-DLBCL
GCB-2	chr8	36784442	36784442	G	A	exonic	KCNU1	632	153	0.1949	Nonsense_Mutation	p.W11X	Targerted Panel Gene	GCB-DLBCL
GCB-20	chr6	134174587	134174587	C	G	splicing	SGK1	255	66	0.2056	Splice_Site	.	Targerted Panel Gene	GCB-DLBCL
GCB-20	chr14	32823190	32823190	T	C	exonic	AKAP6	447	114	0.2032	Missense_Mutation	p.S1793P	Targerted Panel Gene	GCB-DLBCL
GCB-20	chr3	187724969	187724969	C	T	exonic	BCL6	295	222	0.4294	Missense_Mutation	p.R650Q	Targerted Panel Gene	GCB-DLBCL
GCB-20	chr7	2945834	2945834	A	G	exonic	CARD11	444	103	0.188	Missense_Mutation	p.F115L	Targerted Panel Gene	GCB-DLBCL
GCB-20	chr6	41935969	41935969	G	C	exonic	CCND3	272	62	0.1856	Missense_Mutation	p.P284A	Targerted Panel Gene	GCB-DLBCL
GCB-20	chr16	10877356	10877356	C	G	exonic	CIITA	285	63	0.181	Missense_Mutation	p.A9G	Targerted Panel Gene	GCB-DLBCL
GCB-20	chr16	10877365	10877365	A	T	exonic	CIITA	272	60	0.1802	Missense_Mutation	p.Y12F	Targerted Panel Gene	GCB-DLBCL
GCB-20	chr16	10918463	10918463	A	T	exonic	CIITA	165	30	0.1538	Missense_Mutation	p.D1030V	Targerted Panel Gene	GCB-DLBCL
GCB-20	chr10	89014178	89014178	A	G	exonic	FAS	441	113	0.2036	Missense_Mutation	p.K246E	Targerted Panel Gene	GCB-DLBCL
GCB-20	chr10	89014148	89014148	A	C	exonic	FAS	386	94	0.1958	Missense_Mutation	p.I236L	Targerted Panel Gene	GCB-DLBCL
GCB-20	chr3	50256730	50256730	G	T	exonic	GNAI2	200	136	0.4048	Missense_Mutation	p.D185Y	Targerted Panel Gene	GCB-DLBCL
GCB-20	chr3	50252414	50252414	G	A	exonic	GNAI2	206	117	0.3622	Missense_Mutation	p.G44D	Targerted Panel Gene	GCB-DLBCL
GCB-20	chr6	27867238	27867238	G	C	exonic	HIST1H1B	445	105	0.1899	Missense_Mutation	p.Q98E	Targerted Panel Gene	GCB-DLBCL
GCB-20	chr6	27892845	27892845	G	A	exonic	HIST1H2AM	157	34	0.178	Missense_Mutation	p.T102I	Targerted Panel Gene	GCB-DLBCL
GCB-20	chr6	26123645	26123645	C	T	exonic	HIST1H2BC	289	74	0.2033	Missense_Mutation	p.R87H	Targerted Panel Gene	GCB-DLBCL
GCB-20	chr1	201213378	201213378	G	A	exonic	IGFN1	407	77	0.1588	Missense_Mutation	p.G2829R	Targerted Panel Gene	GCB-DLBCL
GCB-20	chr22	22888259	22888259	G	C	exonic	IGLL5	66	29	0.3053	Missense_Mutation	p.G34R	Targerted Panel Gene	GCB-DLBCL
GCB-20	chr22	22888182	22888182	T	C	exonic	IGLL5	124	35	0.2201	Missense_Mutation	p.L8S	Targerted Panel Gene	GCB-DLBCL
GCB-20	chr9	13221422	13221422	C	G	exonic	MPDZ	202	403	0.665	Missense_Mutation	p.A276P	Targerted Panel Gene	GCB-DLBCL
GCB-20	chr6	37171132	37171132	G	C	exonic	PIM1	357	81	0.1845	Missense_Mutation	p.G83A	Targerted Panel Gene	GCB-DLBCL
GCB-20	chr15	33707015	33707015	C	T	exonic	RYR3	240	86	0.2638	Missense_Mutation	p.R2194C	Targerted Panel Gene	GCB-DLBCL
GCB-20	chr16	11255013	11255013	C	A	exonic	SOCS1	152	31	0.1694	Missense_Mutation	p.A156S	Targerted Panel Gene	GCB-DLBCL
GCB-20	chr9	32633265	32633265	T	C	exonic	TAF1L	477	195	0.2902	Missense_Mutation	p.Y772C	Targerted Panel Gene	GCB-DLBCL
GCB-20	chr1	2556666	2556666	T	A	exonic	TNFRSF14	138	79	0.3641	Missense_Mutation	p.M1K	Targerted Panel Gene	GCB-DLBCL
GCB-20	chr17	7674268	7674268	A	G	exonic	TP53	237	49	0.1713	Missense_Mutation	p.I193T	Targerted Panel Gene	GCB-DLBCL
GCB-20	chr3	126508006	126508006	G	T	exonic	UROC1	177	33	0.1564	Missense_Mutation	p.S167R	Targerted Panel Gene	GCB-DLBCL
GCB-20	chr19	30444640	30444640	C	T	exonic	ZNF536	243	40	0.1413	Missense_Mutation	p.L360F	Targerted Panel Gene	GCB-DLBCL
GCB-20	chr2	124647898	124647898	G	T	exonic	CNTNAP5	157	38	0.1949	Nonsense_Mutation	p.E672X	Targerted Panel Gene	GCB-DLBCL
GCB-20	chrX	41342617	41342617	G	A	exonic	DDX3X	531	133	0.2003	Nonsense_Mutation	p.W136X	Targerted Panel Gene	GCB-DLBCL
GCB-20	chr10	89014138	89014138	T	A	exonic	FAS	365	95	0.2065	Nonsense_Mutation	p.Y232X	Targerted Panel Gene	GCB-DLBCL
GCB-20	chr7	124863447	124863447	A	C	exonic	POT1	518	126	0.1953	Nonsense_Mutation	p.L150X	Targerted Panel Gene	GCB-DLBCL
GCB-20	chr4	105235468	105235468	C	A	exonic	TET2	590	137	0.1882	Nonsense_Mutation	p.S509X	Targerted Panel Gene	GCB-DLBCL

GCB-21	chr6	134174510	134174510	C	T	splicing	SGK1	371	75	0.1682	Splice_Site	.	Targerted Panel Gene	GCB-DLBCL
GCB-21	chrX	31121884	31121896	TCTGCCCAAATCA	-	exonic	DMD	73	164	0.692	Frame_Shift_Del	p.D1223fs	Targerted Panel Gene	GCB-DLBCL
GCB-21	chr2	21006037	21006037	G	C	exonic	APOB	645	96	0.1292	Missense_Mutation	p.H3611D	Targerted Panel Gene	GCB-DLBCL
GCB-21	chr1	203305709	203305709	C	T	exonic	BTG2	195	38	0.1631	Missense_Mutation	p.L35F	Targerted Panel Gene	GCB-DLBCL
GCB-21	chr16	10904755	10904755	T	C	exonic	CIITA	314	57	0.1532	Missense_Mutation	p.S318P	Targerted Panel Gene	GCB-DLBCL
GCB-21	chr5	79738568	79738568	G	A	exonic	CMYA5	503	181	0.2646	Missense_Mutation	p.G3268D	Targerted Panel Gene	GCB-DLBCL
GCB-21	chr12	111312112	111312112	C	T	exonic	CUX2	73	23	0.2396	Missense_Mutation	p.P638L	Targerted Panel Gene	GCB-DLBCL
GCB-21	chr2	96144861	96144861	C	T	exonic	DUSP2	116	18	0.1343	Missense_Mutation	p.G137D	Targerted Panel Gene	GCB-DLBCL
GCB-21	chr2	96144984	96144984	G	A	exonic	DUSP2	51	13	0.2031	Missense_Mutation	p.A124V	Targerted Panel Gene	GCB-DLBCL
GCB-21	chr2	96144870	96144870	C	A	exonic	DUSP2	92	37	0.2868	Missense_Mutation	p.G134V	Targerted Panel Gene	GCB-DLBCL
GCB-21	chr2	96144220	96144220	T	C	exonic	DUSP2	353	83	0.1904	Missense_Mutation	p.I222V	Targerted Panel Gene	GCB-DLBCL
GCB-21	chr7	55156541	55156541	G	A	exonic	EGFR	520	72	0.1214	Missense_Mutation	p.G339R	Targerted Panel Gene	GCB-DLBCL
GCB-21	chr4	186633817	186633817	T	G	exonic	FAT1	391	66	0.1444	Missense_Mutation	p.N1397T	Targerted Panel Gene	GCB-DLBCL
GCB-21	chr4	125316900	125316900	C	A	exonic	FAT4	385	68	0.1501	Missense_Mutation	p.D163E	Targerted Panel Gene	GCB-DLBCL
GCB-21	chr4	125317328	125317328	C	T	exonic	FAT4	343	57	0.1425	Missense_Mutation	p.T306M	Targerted Panel Gene	GCB-DLBCL
GCB-21	chr13	40666143	40666143	T	G	exonic	FOXO1	51	9	0.15	Missense_Mutation	p.T24P	Targerted Panel Gene	GCB-DLBCL
GCB-21	chr6	26056289	26056289	G	C	exonic	HIST1H1C	538	73	0.1193	Missense_Mutation	p.A47G	Targerted Panel Gene	GCB-DLBCL
GCB-21	chr6	26156557	26156557	G	A	exonic	HIST1H1E	436	80	0.155	Missense_Mutation	p.G56D	Targerted Panel Gene	GCB-DLBCL
GCB-21	chr6	27146540	27146540	G	A	exonic	HIST1H2BK	258	54	0.1731	Missense_Mutation	p.R87C	Targerted Panel Gene	GCB-DLBCL
GCB-21	chr6	27146481	27146481	C	G	exonic	HIST1H2BK	301	71	0.1909	Missense_Mutation	p.E106D	Targerted Panel Gene	GCB-DLBCL
GCB-21	chr6	27146586	27146586	G	C	exonic	HIST1H2BK	262	47	0.1521	Missense_Mutation	p.F71L	Targerted Panel Gene	GCB-DLBCL
GCB-21	chr22	22888151	22888151	T	C	exonic	IGLL5	146	35	0.1934	Missense_Mutation	p.M33T	Targerted Panel Gene	GCB-DLBCL
GCB-21	chr22	22888227	22888227	C	G	exonic	IGLL5	117	17	0.1269	Missense_Mutation	p.A23G	Targerted Panel Gene	GCB-DLBCL
GCB-21	chr22	22888210	22888210	G	A	exonic	IGLL5	122	22	0.1528	Missense_Mutation	p.G53R	Targerted Panel Gene	GCB-DLBCL
GCB-21	chr22	22888206	22888206	C	T	exonic	IGLL5	120	22	0.1549	Missense_Mutation	p.T16I	Targerted Panel Gene	GCB-DLBCL
GCB-21	chr19	38612671	38612671	C	T	exonic	MAP4K1	146	29	0.1657	Missense_Mutation	p.G202D	Targerted Panel Gene	GCB-DLBCL
GCB-21	chr4	102537940	102537940	C	G	exonic	NFKB1	219	198	0.4748	Missense_Mutation	p.S81C	Targerted Panel Gene	GCB-DLBCL
GCB-21	chr2	50472431	50472431	T	G	exonic	NRXN1	386	65	0.1438	Missense_Mutation	p.K1077N	Targerted Panel Gene	GCB-DLBCL
GCB-21	chr4	133152496	133152496	G	C	exonic	PCDH10	374	60	0.1379	Missense_Mutation	p.D786H	Targerted Panel Gene	GCB-DLBCL
GCB-21	chr6	117383359	117383359	A	C	exonic	ROS1	561	95	0.1448	Missense_Mutation	p.S818R	Targerted Panel Gene	GCB-DLBCL
GCB-21	chr4	20549124	20549124	T	G	exonic	SLIT2	321	48	0.1301	Missense_Mutation	p.L829V	Targerted Panel Gene	GCB-DLBCL
GCB-21	chr16	11255063	11255063	C	T	exonic	SOCS1	185	50	0.2128	Missense_Mutation	p.G139D	Targerted Panel Gene	GCB-DLBCL
GCB-21	chr16	11255460	11255460	C	T	exonic	SOCS1	161	36	0.1827	Missense_Mutation	p.V7M	Targerted Panel Gene	GCB-DLBCL
GCB-21	chr16	11255343	11255343	G	C	exonic	SOCS1	105	24	0.186	Missense_Mutation	p.P46A	Targerted Panel Gene	GCB-DLBCL
GCB-21	chr16	11255120	11255120	G	C	exonic	SOCS1	226	37	0.1407	Missense_Mutation	p.A120G	Targerted Panel Gene	GCB-DLBCL
GCB-21	chr9	12709153	12709153	C	A	exonic	TYRP1	574	91	0.1368	Missense_Mutation	p.L529I	Targerted Panel Gene	GCB-DLBCL
GCB-21	chr1	215680252	215680252	G	T	exonic	USH2A	530	77	0.1269	Missense_Mutation	p.S4064Y	Targerted Panel Gene	GCB-DLBCL
GCB-21	chr1	215674505	215674505	T	G	exonic	USH2A	659	94	0.1247	Missense_Mutation	p.N4469T	Targerted Panel Gene	GCB-DLBCL
GCB-21	chr1	216324285	216324285	T	C	exonic	USH2A	639	80	0.1113	Missense_Mutation	p.E404G	Targerted Panel Gene	GCB-DLBCL
GCB-21	chr16	11255146	11255146	G	T	exonic	SOCS1	200	37	0.1561	Nonsense_Mutation	p.C111X	Targerted Panel Gene	GCB-DLBCL
GCB-21	chr1	2561724	2561724	G	A	exonic	TNFRSF14	384	64	0.1429	Nonsense_Mutation	p.W201X	Targerted Panel Gene	GCB-DLBCL
GCB-21	chr16	11254844	11254844	C	G	exonic	SOCS1	368	97	0.2086	Nonstop_Mutation	p.X212S	Targerted Panel Gene	GCB-DLBCL
GCB-22	chr6	134174510	134174510	C	T	splicing	SGK1	159	84	0.3457	Splice_Site	.	Targerted Panel Gene	GCB-DLBCL
GCB-22	chr12	122022173	122022173	G	C	exonic	BCL7A	125	123	0.496	Missense_Mutation	p.V28L	Targerted Panel Gene	GCB-DLBCL
GCB-22	chr22	23181431	23181431	C	G	exonic	BCR	100	53	0.3464	Missense_Mutation	p.N157K	Targerted Panel Gene	GCB-DLBCL
GCB-22	chr22	23181442	23181442	T	G	exonic	BCR	111	56	0.3353	Missense_Mutation	p.I161S	Targerted Panel Gene	GCB-DLBCL
GCB-22	chr7	148172308	148172308	T	C	exonic	CNTNAP2	312	134	0.3004	Missense_Mutation	p.L947P	Targerted Panel Gene	GCB-DLBCL
GCB-22	chr2	124798173	124798173	A	C	exonic	CNTNAP5	541	230	0.2979	Missense_Mutation	p.S1023R	Targerted Panel Gene	GCB-DLBCL
GCB-22	chr2	136118051	136118051	T	C	exonic	CXCR4	290	116	0.2857	Missense_Mutation	p.I4V	Targerted Panel Gene	GCB-DLBCL
GCB-22	chr11	92801422	92801422	G	C	exonic	FAT3	405	385	0.4873	Missense_Mutation	p.K2803N	Targerted Panel Gene	GCB-DLBCL
GCB-22	chr13	40666149	40666149	A	G	exonic	FOXO1	54	21	0.28	Missense_Mutation	p.S22P	Targerted Panel Gene	GCB-DLBCL
GCB-22	chr6	26124531	26124531	G	T	exonic	HIST1H2AC	324	147	0.3121	Missense_Mutation	p.R100L	Targerted Panel Gene	GCB-DLBCL
GCB-22	chr6	26158532	26158532	G	C	exonic	HIST1H2BD	443	154	0.2571	Missense_Mutation	p.K121N	Targerted Panel Gene	GCB-DLBCL
GCB-22	chr6	27893604	27893604	C	G	exonic	HIST1H2BO	252	116	0.3152	Missense_Mutation	p.Q48E	Targerted Panel Gene	GCB-DLBCL
GCB-22	chr22	22888259	22888259	G	T	exonic	IGLL5	101	46	0.3129	Missense_Mutation	p.G34C	Targerted Panel Gene	GCB-DLBCL
GCB-22	chr22	22888169	22888169	T	C	exonic	IGLL5	148	66	0.3084	Missense_Mutation	p.C4R	Targerted Panel Gene	GCB-DLBCL
GCB-22	chr22	22888157	22888157	C	G	exonic	IGLL5	145	85	0.3696	Missense_Mutation	p.A35G	Targerted Panel Gene	GCB-DLBCL
GCB-22	chr16	85911581	85911581	G	C	exonic	IRF8	198	154	0.4375	Missense_Mutation	p.V124L	Targerted Panel Gene	GCB-DLBCL
GCB-22	chr3	183555449	183555449	T	C	exonic	KLHL6	454	211	0.3173	Missense_Mutation	p.N69D	Targerted Panel Gene	GCB-DLBCL
GCB-22	chr3	183555414	183555414	A	C	exonic	KLHL6	370	162	0.3045	Missense_Mutation	p.I80M	Targerted Panel Gene	GCB-DLBCL
GCB-22	chr3	183555437	183555437	C	G	exonic	KLHL6	426	186	0.3039	Missense_Mutation	p.D73H	Targerted Panel Gene	GCB-DLBCL
GCB-22	chr1	235709120	235709120	C	T	exonic	LYST	433	194	0.3089	Missense_Mutation	p.V3372I	Targerted Panel Gene	GCB-DLBCL
GCB-22	chr6	37170642	37170642	A	G	exonic	PIM1	105	59	0.3598	Missense_Mutation	p.T23A	Targerted Panel Gene	GCB-DLBCL
GCB-22	chr3	16377769	16377769	C	G	exonic	RFTN1	400	179	0.3086	Missense_Mutation	p.D259H	Targerted Panel Gene	GCB-DLBCL
GCB-22	chr6	134174794	134174794	G	A	exonic	SGK1	165	126	0.433	Missense_Mutation	p.T5I	Targerted Panel Gene	GCB-DLBCL
GCB-22	chr6	134174520	134174520	G	C	exonic	SGK1	164	91	0.3555	Missense_Mutation	p.A143G	Targerted Panel Gene	GCB-DLBCL
GCB-22	chr6	134174741	134174741	T	G	exonic	SGK1	155	119	0.4343	Missense_Mutation	p.I23L	Targerted Panel Gene	GCB-DLBCL
GCB-22	chr17	7676046	7676046	C	T	exonic	TP53	209	111	0.3458	Missense_Mutation	p.G69D	Targerted Panel Gene	GCB-DLBCL
GCB-22	chrX	101353338	101353338	C	T	exonic	BTK	107	150	0.5837	Nonsense_Mutation	p.W622X	Targerted Panel Gene	GCB-DLBCL
GCB-23	chr6	134174587	134174587	C	G	splicing	SGK1	150	136	0.4739	Splice_Site	.	Targerted Panel Gene	GCB-DLBCL
GCB-23	chr20	32434571	32434571	G	C	exonic	ASXL1	143	128	0.4723	Missense_Mutation	p.R620P	Targerted Panel Gene	GCB-DLBCL
GCB-23	chrX	101353884	101353884	T	C	exonic	BTK	56	182	0.7615	Missense_Mutation	p.D613G	Targerted Panel Gene	GCB-DLBCL
GCB-23	chr7	2937972	2937972	T	C	exonic	CARD11	159	125	0.4401	Missense_Mutation	p.M360V	Targerted Panel Gene	GCB-DLBCL
GCB-23	chr10	71807401	71807401	T	A	exonic	CDH23	93	93	0.5	Missense_Mutation	p.I2768N	Targerted Panel Gene	GCB-DLBCL
GCB-23	chr2	224770220	224770220	C	T	exonic	DOCK10	176	131	0.4267	Missense_Mutation	p.M2145I	Targerted Panel Gene	GCB-DLBCL
GCB-23	chr7	111760199	111760199	A	C	exonic	DOCK4	216	142	0.3966	Missense_Mutation	p.F1373V	Targerted Panel Gene	GCB-DLBCL
GCB-23	chr3	135192788	135192788	T	G	exonic	EPHB1	270	208	0.4351	Missense_Mutation	p.F699V	Targerted Panel Gene	GCB-DLBCL
GCB-23	chr13	40666151	40666151	C	G	exonic	FOXO1	38	22	0.3667	Missense_Mutation	p.R21P	Targerted Panel Gene	GCB-DLBCL
GCB-23	chr20	58854189	58854189	G	A	exonic	GNAS	177	110	0.3833	Missense_Mutation	p.R246H	Targerted Panel Gene	GCB-DLBCL
GCB-23	chr6	26156621	26156621	C	A	exonic	HIST1H1E	93	184	0.6643	Missense_Mutation	p.N77K	Targerted Panel Gene	GCB-DLBCL
GCB-23	chr6	26124362	26124362	G	A	exonic	HIST1H2AC	36	105	0.7447	Missense_Mutation	p.V44I	Targerted Panel Gene	GCB-DLBCL
GCB-23	chr6	27892770	27892770	G	A	exonic	HIST1H2AM	15	59	0.7973	Missense_Mutation	p.A127V	Targerted Panel Gene	GCB-DLBCL

GCB-23	chr22	22888181	22888181	T	C	exonic	IGLL5	25	71	0.7396	Missense_Mutation	p.V43A	Targerted Panel Gene	GCB-DLBCL
GCB-23	chr22	22888223	22888223	G	C	exonic	IGLL5	22	61	0.7349	Missense_Mutation	p.E22Q	Targerted Panel Gene	GCB-DLBCL
GCB-23	chr10	63183513	63183513	A	C	exonic	JMJD1C	338	311	0.4792	Missense_Mutation	p.S2340A	Targerted Panel Gene	GCB-DLBCL
GCB-23	chr7	111124274	111124274	T	A	exonic	LRRN3	451	383	0.4592	Missense_Mutation	p.L501Q	Targerted Panel Gene	GCB-DLBCL
GCB-23	chr7	111123027	111123027	A	T	exonic	LRRN3	528	402	0.4323	Missense_Mutation	p.E85D	Targerted Panel Gene	GCB-DLBCL
GCB-23	chr7	111123052	111123052	C	A	exonic	LRRN3	518	393	0.4314	Missense_Mutation	p.L94I	Targerted Panel Gene	GCB-DLBCL
GCB-23	chr7	111124409	111124409	A	G	exonic	LRRN3	465	317	0.4054	Missense_Mutation	p.K546R	Targerted Panel Gene	GCB-DLBCL
GCB-23	chr7	111124774	111124774	A	G	exonic	LRRN3	476	314	0.397	Missense_Mutation	p.K668E	Targerted Panel Gene	GCB-DLBCL
GCB-23	chr7	111124881	111124881	A	T	exonic	LRRN3	364	216	0.3724	Missense_Mutation	p.L703F	Targerted Panel Gene	GCB-DLBCL
GCB-23	chr3	16377741	16377741	A	T	exonic	RFTN1	263	186	0.4143	Missense_Mutation	p.V268E	Targerted Panel Gene	GCB-DLBCL
GCB-23	chr1	237591776	237591776	A	C	exonic	RYR2	208	171	0.4512	Missense_Mutation	p.S1400R	Targerted Panel Gene	GCB-DLBCL
GCB-23	chr6	134174586	134174586	G	A	exonic	SGK1	195	95	0.3265	Missense_Mutation	p.A121V	Targerted Panel Gene	GCB-DLBCL
GCB-23	chr6	134175544	134175544	C	T	exonic	SGK1	71	33	0.3173	Missense_Mutation	p.A40T	Targerted Panel Gene	GCB-DLBCL
GCB-23	chr6	134174568	134174568	C	G	exonic	SGK1	154	157	0.5032	Missense_Mutation	p.R127T	Targerted Panel Gene	GCB-DLBCL
GCB-23	chr6	134174786	134174786	C	T	exonic	SGK1	237	179	0.4303	Missense_Mutation	p.A8T	Targerted Panel Gene	GCB-DLBCL
GCB-23	chr6	134174009	134174009	G	T	exonic	SGK1	241	119	0.3296	Missense_Mutation	p.P170H	Targerted Panel Gene	GCB-DLBCL
GCB-23	chr6	134174563	134174563	C	T	exonic	SGK1	230	86	0.2722	Missense_Mutation	p.G129S	Targerted Panel Gene	GCB-DLBCL
GCB-23	chr6	134174511	134174511	T	C	exonic	SGK1	233	71	0.2336	Missense_Mutation	p.H146R	Targerted Panel Gene	GCB-DLBCL
GCB-23	chr4	20257909	20257909	C	A	exonic	SLIT2	306	187	0.3793	Missense_Mutation	p.A98E	Targerted Panel Gene	GCB-DLBCL
GCB-23	chr16	11254946	11254946	C	G	exonic	SOCS1	138	106	0.4344	Missense_Mutation	p.C178S	Targerted Panel Gene	GCB-DLBCL
GCB-23	chr16	11255477	11255477	A	T	exonic	SOCS1	126	89	0.412	Missense_Mutation	p.M1K	Targerted Panel Gene	GCB-DLBCL
GCB-23	chr16	11254929	11254929	C	G	exonic	SOCS1	149	100	0.4016	Missense_Mutation	p.A184P	Targerted Panel Gene	GCB-DLBCL
GCB-23	chr13	40666197	40666197	G	A	exonic	FOXO1	27	39	0.5909	Nonsense_Mutation	p.Q6X	Targerted Panel Gene	GCB-DLBCL
GCB-23	chr17	65056515	65056515	G	A	exonic	GNA13	25	211	0.8941	Nonsense_Mutation	p.Q27X	Targerted Panel Gene	GCB-DLBCL
GCB-23	chr16	11255088	11255088	G	A	exonic	SOCS1	117	108	0.48	Nonsense_Mutation	p.Q131X	Targerted Panel Gene	GCB-DLBCL
GCB-23	chr1	15928416	15928416	C	T	exonic	SPEN	345	279	0.4464	Nonsense_Mutation	p.Q726X	Targerted Panel Gene	GCB-DLBCL
GCB-24	chr6	134174510	134174510	C	T	splicing	SGK1	405	44	0.098	Splice_Site	.	Targerted Panel Gene	GCB-DLBCL
GCB-24	chr14	32823399	32823399	T	A	exonic	AKAP6	802	104	0.1148	Missense_Mutation	p.N1862K	Targerted Panel Gene	GCB-DLBCL
GCB-24	chr18	63318275	63318275	G	A	exonic	BCL2	535	70	0.1157	Missense_Mutation	p.A131V	Targerted Panel Gene	GCB-DLBCL
GCB-24	chr18	63318411	63318411	G	A	exonic	BCL2	221	35	0.1367	Missense_Mutation	p.L86F	Targerted Panel Gene	GCB-DLBCL
GCB-24	chr18	63318407	63318407	C	G	exonic	BCL2	217	34	0.1349	Missense_Mutation	p.S87T	Targerted Panel Gene	GCB-DLBCL
GCB-24	chr18	63318293	63318293	G	T	exonic	BCL2	493	62	0.1117	Missense_Mutation	p.T125N	Targerted Panel Gene	GCB-DLBCL
GCB-24	chr18	63318539	63318539	G	A	exonic	BCL2	331	35	0.0954	Missense_Mutation	p.A43V	Targerted Panel Gene	GCB-DLBCL
GCB-24	chr1	203305689	203305689	G	C	exonic	BTG2	204	49	0.1937	Missense_Mutation	p.G28A	Targerted Panel Gene	GCB-DLBCL
GCB-24	chr1	203307152	203307152	G	A	exonic	BTG2	368	39	0.0958	Missense_Mutation	p.G64D	Targerted Panel Gene	GCB-DLBCL
GCB-24	chr6	26199719	26199719	G	A	exonic	HIST1H2BF	149	32	0.1768	Missense_Mutation	p.G54D	Targerted Panel Gene	GCB-DLBCL
GCB-24	chr6	27872048	27872048	C	A	exonic	HIST1H3I	44	9	0.1698	Missense_Mutation	p.V90L	Targerted Panel Gene	GCB-DLBCL
GCB-24	chr7	124841171	124841171	C	A	exonic	POT1	558	69	0.11	Missense_Mutation	p.V391F	Targerted Panel Gene	GCB-DLBCL
GCB-24	chr3	16377731	16377731	C	G	exonic	RFTN1	410	54	0.1164	Missense_Mutation	p.E271D	Targerted Panel Gene	GCB-DLBCL
GCB-24	chr16	11255208	11255208	C	G	exonic	SOCS1	132	18	0.12	Missense_Mutation	p.E91Q	Targerted Panel Gene	GCB-DLBCL
GCB-24	chr16	11255430	11255430	C	A	exonic	SOCS1	219	29	0.1169	Missense_Mutation	p.A17S	Targerted Panel Gene	GCB-DLBCL
GCB-24	chr16	11255154	11255154	G	C	exonic	SOCS1	184	22	0.1068	Missense_Mutation	p.R109G	Targerted Panel Gene	GCB-DLBCL
GCB-25	chr6	134171733	134171733	C	G	splicing	SGK1	217	25	0.1029	Splice_Site	.	Targerted Panel Gene	GCB-DLBCL
GCB-25	chr1	203305709	203305709	C	G	exonic	BTG2	133	19	0.125	Missense_Mutation	p.L35V	Targerted Panel Gene	GCB-DLBCL
GCB-25	chr8	4032075	4032075	T	G	exonic	CSMD1	427	61	0.1247	Missense_Mutation	p.N147T	Targerted Panel Gene	GCB-DLBCL
GCB-25	chr12	113058429	113058429	G	A	exonic	DTX1	203	56	0.2162	Missense_Mutation	p.M79I	Targerted Panel Gene	GCB-DLBCL
GCB-25	chr19	39875484	39875484	G	A	exonic	FCGBP	255	31	0.1084	Missense_Mutation	p.H4704Y	Targerted Panel Gene	GCB-DLBCL
GCB-25	chr6	27146472	27146472	C	G	exonic	HIST1H2BK	299	36	0.1071	Missense_Mutation	p.K109N	Targerted Panel Gene	GCB-DLBCL
GCB-25	chr22	22893804	22893804	A	C	exonic	IGLL5	371	49	0.1167	Missense_Mutation	p.K104T	Targerted Panel Gene	GCB-DLBCL
GCB-25	chr22	22888216	22888216	T	C	exonic	IGLL5	151	18	0.1065	Missense_Mutation	p.S55P	Targerted Panel Gene	GCB-DLBCL
GCB-25	chr12	25245351	25245351	C	T	exonic	KRAS	542	146	0.2116	Missense_Mutation	p.G12S	Targerted Panel Gene	GCB-DLBCL
GCB-25	chr12	53471651	53471651	T	G	exonic	PCBP2	50	7	0.1228	Missense_Mutation	p.I299R	Targerted Panel Gene	GCB-DLBCL
GCB-25	chr12	121827958	121827958	G	A	exonic	SETD1B	38	12	0.24	Missense_Mutation	p.R1829H	Targerted Panel Gene	GCB-DLBCL
GCB-25	chr6	134171666	134171666	C	T	exonic	SGK1	291	46	0.1365	Missense_Mutation	p.A380T	Targerted Panel Gene	GCB-DLBCL
GCB-25	chr6	134317457	134317457	C	T	exonic	SGK1	704	86	0.1089	Missense_Mutation	p.V2I	Targerted Panel Gene	GCB-DLBCL
GCB-25	chr1	15935965	15935965	C	T	exonic	SPEN	164	19	0.1038	Missense_Mutation	p.A3242V	Targerted Panel Gene	GCB-DLBCL
GCB-25	chr4	105259712	105259712	G	C	exonic	TET2	481	181	0.2734	Missense_Mutation	p.K1299N	Targerted Panel Gene	GCB-DLBCL
GCB-26	chr6	134175543	134175543	C	T	splicing	SGK1	42	17	0.2881	Splice_Site	.	Targerted Panel Gene	GCB-DLBCL
GCB-26	chr15	44715690	44715693	TAGT	-	exonic	B2M	229	95	0.2932	Frame_Shift_Del	p.I112fs	Targerted Panel Gene	GCB-DLBCL
GCB-26	chr22	22888190	22888190	A	-	exonic	IGLL5	70	42	0.375	Frame_Shift_Del	p.K11fs	Targerted Panel Gene	GCB-DLBCL
GCB-26	chr2	32549406	32549406	C	T	exonic	BIRC6	349	116	0.2495	Missense_Mutation	p.P4357S	Targerted Panel Gene	GCB-DLBCL
GCB-26	chr12	92145472	92145472	T	G	exonic	BTG1	114	41	0.2645	Missense_Mutation	p.I22L	Targerted Panel Gene	GCB-DLBCL
GCB-26	chr12	92145447	92145447	C	T	exonic	BTG1	108	38	0.2603	Missense_Mutation	p.G30E	Targerted Panel Gene	GCB-DLBCL
GCB-26	chr1	203305739	203305739	G	C	exonic	BTG2	70	17	0.1954	Missense_Mutation	p.A45P	Targerted Panel Gene	GCB-DLBCL
GCB-26	chr1	203305707	203305707	G	A	exonic	BTG2	105	21	0.1667	Missense_Mutation	p.R34K	Targerted Panel Gene	GCB-DLBCL
GCB-26	chr7	2944519	2944519	C	T	exonic	CARD11	144	62	0.301	Missense_Mutation	p.G126D	Targerted Panel Gene	GCB-DLBCL
GCB-26	chr7	2944252	2944252	T	A	exonic	CARD11	336	166	0.3307	Missense_Mutation	p.K215M	Targerted Panel Gene	GCB-DLBCL
GCB-26	chr5	138292080	138292080	G	C	exonic	CDC25C	357	355	0.4972	Missense_Mutation	p.P218A	Targerted Panel Gene	GCB-DLBCL
GCB-26	chr16	10907399	10907399	C	T	exonic	CIITA	118	57	0.3257	Missense_Mutation	p.T637M	Targerted Panel Gene	GCB-DLBCL
GCB-26	chr8	3396346	3396346	A	T	exonic	CSMD1	309	125	0.288	Missense_Mutation	p.V814D	Targerted Panel Gene	GCB-DLBCL
GCB-26	chr6	56511398	56511398	T	G	exonic	DST	328	95	0.2246	Missense_Mutation	p.Q4074H	Targerted Panel Gene	GCB-DLBCL
GCB-26	chr5	138465878	138465878	G	A	exonic	EGR1	220	75	0.2542	Missense_Mutation	p.M39I	Targerted Panel Gene	GCB-DLBCL
GCB-26	chr22	41093006	41093006	T	A	exonic	EP300	340	128	0.2735	Missense_Mutation	p.M1K	Targerted Panel Gene	GCB-DLBCL
GCB-26	chr13	40666142	40666142	G	A	exonic	FOXO1	62	13	0.1733	Missense_Mutation	p.T24I	Targerted Panel Gene	GCB-DLBCL
GCB-26	chr13	40666106	40666106	T	A	exonic	FOXO1	51	13	0.2031	Missense_Mutation	p.N36I	Targerted Panel Gene	GCB-DLBCL
GCB-26	chr13	40666143	40666143	T	C	exonic	FOXO1	62	14	0.1842	Missense_Mutation	p.T24A	Targerted Panel Gene	GCB-DLBCL
GCB-26	chr13	40665599	40665599	C	T	exonic	FOXO1	194	42	0.178	Missense_Mutation	p.S205N	Targerted Panel Gene	GCB-DLBCL
GCB-26	chr9	37033995	37033995	T	C	exonic	PAX5	188	65	0.2569	Missense_Mutation	p.S13G	Targerted Panel Gene	GCB-DLBCL
GCB-26	chr9	37033994	37033994	C	G	exonic	PAX5	188	66	0.2598	Missense_Mutation	p.S13T	Targerted Panel Gene	GCB-DLBCL
GCB-26	chr4	133151053	133151053	C	G	exonic	PCDH10	208	79	0.2753	Missense_Mutation	p.P305A	Targerted Panel Gene	GCB-DLBCL
GCB-26	chr4	133151030	133151030	C	T	exonic	PCDH10	197	73	0.2704	Missense_Mutation	p.A297V	Targerted Panel Gene	GCB-DLBCL
GCB-26	chr6	37171170	37171170	G	A	exonic	PIM1	402	44	0.0987	Missense_Mutation	p.V96M	Targerted Panel Gene	GCB-DLBCL

GCB-26	chr9	8449782	8449782	G	A	exonic	PTPRD	590	202	0.2551	Missense_Mutation	p.P1311S	Targerted Panel Gene	GCB-DLBCL
GCB-26	chr2	197405412	197405412	T	C	exonic	SF3B1	329	138	0.2942	Missense_Mutation	p.T434A	Targerted Panel Gene	GCB-DLBCL
GCB-26	chr6	134173523	134173523	G	A	exonic	SGK1	280	60	0.1765	Missense_Mutation	p.P186L	Targerted Panel Gene	GCB-DLBCL
GCB-26	chr6	134174512	134174512	G	A	exonic	SGK1	229	28	0.1089	Missense_Mutation	p.H146Y	Targerted Panel Gene	GCB-DLBCL
GCB-26	chr6	134174031	134174031	G	A	exonic	SGK1	242	66	0.2143	Missense_Mutation	p.L163F	Targerted Panel Gene	GCB-DLBCL
GCB-26	chr6	134175544	134175544	C	T	exonic	SGK1	44	16	0.2667	Missense_Mutation	p.A40T	Targerted Panel Gene	GCB-DLBCL
GCB-26	chr6	134173517	134173517	G	A	exonic	SGK1	283	67	0.1914	Missense_Mutation	p.A188V	Targerted Panel Gene	GCB-DLBCL
GCB-26	chr6	134174545	134174545	G	C	exonic	SGK1	211	70	0.2491	Missense_Mutation	p.Q135E	Targerted Panel Gene	GCB-DLBCL
GCB-26	chr6	134174571	134174571	C	T	exonic	SGK1	209	60	0.223	Missense_Mutation	p.R126K	Targerted Panel Gene	GCB-DLBCL
GCB-26	chr6	134173142	134173142	T	A	exonic	SGK1	357	95	0.2097	Missense_Mutation	p.M239L	Targerted Panel Gene	GCB-DLBCL
GCB-26	chr6	134174575	134174575	G	T	exonic	SGK1	212	45	0.1692	Missense_Mutation	p.Q125K	Targerted Panel Gene	GCB-DLBCL
GCB-26	chr6	134174016	134174016	G	A	exonic	SGK1	254	51	0.1661	Missense_Mutation	p.P168S	Targerted Panel Gene	GCB-DLBCL
GCB-26	chr16	11255472	11255472	C	A	exonic	SOCS1	127	33	0.2062	Missense_Mutation	p.A3S	Targerted Panel Gene	GCB-DLBCL
GCB-26	chr16	10907005	10907005	G	T	exonic	CIITA	177	58	0.2468	Nonsense_Mutation	p.E506X	Targerted Panel Gene	GCB-DLBCL
GCB-26	chr6	27838087	27838087	G	A	exonic	HIST1H2AK	44	12	0.2143	Nonsense_Mutation	p.Q85X	Targerted Panel Gene	GCB-DLBCL
GCB-27	chr17	7676273	7676273	C	T	splicing	TP53	65	82	0.5578	Splice_Site	.	Targerted Panel Gene	GCB-DLBCL
GCB-27	chr1	236897007	236897007	C	-	exonic	MTR	137	144	0.5125	Frame_Shift_Del	p.G1200fs	Targerted Panel Gene	GCB-DLBCL
GCB-27	chr1	228246766	228246766	T	-	exonic	OBSCN	132	95	0.4167	Frame_Shift_Del	p.A1424fs	Targerted Panel Gene	GCB-DLBCL
GCB-27	chr7	2945827	2945827	G	T	exonic	CARD11	212	156	0.4228	Missense_Mutation	p.T117N	Targerted Panel Gene	GCB-DLBCL
GCB-27	chr10	17100226	17100226	G	C	exonic	CUBN	268	145	0.3511	Missense_Mutation	p.T515S	Targerted Panel Gene	GCB-DLBCL
GCB-27	chr9	87703210	87703210	C	G	exonic	DAPK1	62	22	0.2619	Missense_Mutation	p.T1018R	Targerted Panel Gene	GCB-DLBCL
GCB-27	chr22	22888243	22888243	C	G	exonic	IGLL5	53	36	0.4	Missense_Mutation	p.C28W	Targerted Panel Gene	GCB-DLBCL
GCB-27	chr12	25245347	25245347	C	T	exonic	KRAS	152	166	0.522	Missense_Mutation	p.G13D	Targerted Panel Gene	GCB-DLBCL
GCB-27	chr1	11199271	11199271	C	T	exonic	MTOR	73	204	0.7365	Missense_Mutation	p.E1414K	Targerted Panel Gene	GCB-DLBCL
GCB-27	chr16	88717023	88717023	C	G	exonic	PIEZO1	175	149	0.4599	Missense_Mutation	p.E2220D	Targerted Panel Gene	GCB-DLBCL
GCB-27	chr3	16378057	16378057	G	A	exonic	RFTN1	197	102	0.3411	Missense_Mutation	p.P163S	Targerted Panel Gene	GCB-DLBCL
GCB-27	chr6	134174031	134174031	G	A	exonic	SGK1	204	83	0.2892	Missense_Mutation	p.L163F	Targerted Panel Gene	GCB-DLBCL
GCB-27	chr4	105259686	105259686	T	G	exonic	TET2	292	146	0.3333	Missense_Mutation	p.W1291G	Targerted Panel Gene	GCB-DLBCL
GCB-27	chr6	137876155	137876155	A	T	exonic	TNFAIP3	241	145	0.3756	Missense_Mutation	p.D265V	Targerted Panel Gene	GCB-DLBCL
GCB-27	chr1	216084781	216084781	C	T	exonic	USH2A	263	168	0.3898	Missense_Mutation	p.S1695N	Targerted Panel Gene	GCB-DLBCL
GCB-27	chr10	63465551	63465551	G	A	exonic	JMJD1C	44	46	0.5111	Nonsense_Mutation	p.R38X	Targerted Panel Gene	GCB-DLBCL
GCB-27	chr12	49033457	49033457	G	A	exonic	KMT2D	191	60	0.239	Nonsense_Mutation	p.Q3750X	Targerted Panel Gene	GCB-DLBCL
GCB-27	chr1	2561676	2561676	C	A	exonic	TNFRSF14	41	118	0.7421	Nonsense_Mutation	p.C185X	Targerted Panel Gene	GCB-DLBCL
GCB-28	chr16	3744907	3744911	TTTGT	-	exonic	CREBBP	318	117	0.269	Frame_Shift_Del	p.N1322fs	Targerted Panel Gene	GCB-DLBCL
GCB-28	chr12	49031665	49031665	T	-	exonic	KMT2D	332	317	0.4877	Frame_Shift_Del	p.Q4347fs	Targerted Panel Gene	GCB-DLBCL
GCB-28	chrX	154432317	154432317	T	A	exonic	ATP6AP1	200	102	0.3377	Missense_Mutation	p.Y139N	Targerted Panel Gene	GCB-DLBCL
GCB-28	chr1	103002430	103002430	G	T	exonic	COL11A1	356	147	0.2922	Missense_Mutation	p.Q711K	Targerted Panel Gene	GCB-DLBCL
GCB-28	chr8	112689999	112689999	C	G	exonic	CSMD3	619	314	0.3362	Missense_Mutation	p.R635T	Targerted Panel Gene	GCB-DLBCL
GCB-28	chrX	41346372	41346372	T	A	exonic	DDX3X	491	168	0.2549	Missense_Mutation	p.F487I	Targerted Panel Gene	GCB-DLBCL
GCB-28	chr22	41170518	41170518	T	C	exonic	EP300	356	162	0.3121	Missense_Mutation	p.Y1467H	Targerted Panel Gene	GCB-DLBCL
GCB-28	chr11	128480385	128480385	T	A	exonic	ETS1	267	244	0.4775	Missense_Mutation	p.Q310L	Targerted Panel Gene	GCB-DLBCL
GCB-28	chr7	148811635	148811635	T	A	exonic	EZH2	389	111	0.222	Missense_Mutation	p.Y646F	Targerted Panel Gene	GCB-DLBCL
GCB-28	chr17	65014660	65014660	A	C	exonic	GNA13	717	218	0.2332	Missense_Mutation	p.L244R	Targerted Panel Gene	GCB-DLBCL
GCB-28	chr10	87933163	87933163	T	G	exonic	PTEN	244	275	0.5299	Missense_Mutation	p.I308R	Targerted Panel Gene	GCB-DLBCL
GCB-28	chr13	48347826	48347826	A	C	exonic	RB1	159	145	0.477	Missense_Mutation	p.T168P	Targerted Panel Gene	GCB-DLBCL
GCB-28	chr19	11030832	11030832	G	A	exonic	SMARCA4	190	127	0.3981	Missense_Mutation	p.G1162D	Targerted Panel Gene	GCB-DLBCL
GCB-28	chr19	10984267	10984267	C	T	exonic	SMARCA4	150	82	0.3534	Missense_Mutation	p.A39V	Targerted Panel Gene	GCB-DLBCL
GCB-28	chr17	65053683	65053683	C	T	exonic	GNA13	482	180	0.2715	Nonsense_Mutation	p.W110X	Targerted Panel Gene	GCB-DLBCL
GCB-28	chr12	49040506	49040506	G	A	exonic	KMT2D	597	184	0.2353	Nonsense_Mutation	p.Q2422X	Targerted Panel Gene	GCB-DLBCL
GCB-29	chr12	49041466	49041466	G	-	exonic	KMT2D	161	105	0.3933	Frame_Shift_Del	p.L2102fs	Targerted Panel Gene	GCB-DLBCL
GCB-29	chr12	113094874	113094882	TGGGCCGCC	-	exonic	DTX1	83	34	0.2906	In_Frame_Del	p.438_441del	Targerted Panel Gene	GCB-DLBCL
GCB-29	chr15	44711547	44711547	A	C	exonic	B2M	55	86	0.6099	Missense_Mutation	p.M1L	Targerted Panel Gene	GCB-DLBCL
GCB-29	chr18	63318575	63318575	T	C	exonic	BCL2	402	136	0.2528	Missense_Mutation	p.D31G	Targerted Panel Gene	GCB-DLBCL
GCB-29	chrX	101370055	101370055	A	T	exonic	BTK	253	148	0.3691	Missense_Mutation	p.Y112N	Targerted Panel Gene	GCB-DLBCL
GCB-29	chr16	3736766	3736766	A	T	exonic	CREBBP	432	177	0.2906	Missense_Mutation	p.Y1482N	Targerted Panel Gene	GCB-DLBCL
GCB-29	chrX	31209582	31209582	C	T	exonic	DMD	211	34	0.1388	Missense_Mutation	p.R3037H	Targerted Panel Gene	GCB-DLBCL
GCB-29	chr11	117532453	117532453	G	A	exonic	DSCAML1	244	351	0.5899	Missense_Mutation	p.S254F	Targerted Panel Gene	GCB-DLBCL
GCB-29	chr1	2556667	2556667	G	A	exonic	TNFRSF14	120	43	0.2638	Missense_Mutation	p.M1I	Targerted Panel Gene	GCB-DLBCL
GCB-29	chr12	49022320	49022320	C	A	exonic	KMT2D	168	130	0.4362	Nonsense_Mutation	p.E5458X	Targerted Panel Gene	GCB-DLBCL
GCB-3	chr3	187745408	187745408	A	T	splicing	BCL6	435	68	0.1352	Splice_Site	.	Targerted Panel Gene	GCB-DLBCL
GCB-3	chr4	102612432	102612432	A	C	splicing	NFKB1	256	82	0.2426	Splice_Site	.	Targerted Panel Gene	GCB-DLBCL
GCB-3	chr19	6590918	6590918	-	A	exonic	CD70	155	53	0.2548	Frame_Shift_Ins	p.G29fs	Targerted Panel Gene	GCB-DLBCL
GCB-3	chr15	44711581	44711581	T	C	exonic	B2M	151	84	0.3574	Missense_Mutation	p.L12P	Targerted Panel Gene	GCB-DLBCL
GCB-3	chr11	69643844	69643844	C	T	exonic	CCND1	191	144	0.4299	Missense_Mutation	p.L143F	Targerted Panel Gene	GCB-DLBCL
GCB-3	chr16	10906772	10906772	G	C	exonic	CIITA	264	86	0.2457	Missense_Mutation	p.S428T	Targerted Panel Gene	GCB-DLBCL
GCB-3	chr8	25325375	25325375	A	T	exonic	DOCK5	387	99	0.2037	Missense_Mutation	p.K577N	Targerted Panel Gene	GCB-DLBCL
GCB-3	chr4	125451913	125451913	T	G	exonic	FAT4	519	153	0.2277	Missense_Mutation	p.L3633V	Targerted Panel Gene	GCB-DLBCL
GCB-3	chr4	125318113	125318113	C	A	exonic	FAT4	521	116	0.1821	Missense_Mutation	p.L568I	Targerted Panel Gene	GCB-DLBCL
GCB-3	chr13	40666142	40666142	G	T	exonic	FOXO1	45	16	0.2623	Missense_Mutation	p.T24N	Targerted Panel Gene	GCB-DLBCL
GCB-3	chr13	40666144	40666144	G	C	exonic	FOXO1	45	15	0.25	Missense_Mutation	p.C23W	Targerted Panel Gene	GCB-DLBCL
GCB-3	chr3	183555421	183555421	A	T	exonic	KLHL6	343	144	0.2957	Missense_Mutation	p.V78E	Targerted Panel Gene	GCB-DLBCL
GCB-3	chrX	141907256	141907256	G	T	exonic	MAGEC1	124	128	0.5079	Missense_Mutation	p.G618W	Targerted Panel Gene	GCB-DLBCL
GCB-3	chr17	7673803	7673803	G	C	exonic	TP53	221	103	0.3179	Missense_Mutation	p.R234G	Targerted Panel Gene	GCB-DLBCL
GCB-3	chr8	102295438	102295438	T	C	exonic	UBR5	514	113	0.1802	Missense_Mutation	p.N1303D	Targerted Panel Gene	GCB-DLBCL
GCB-3	chr17	59838365	59838365	C	T	exonic	VMP1	289	45	0.1347	Missense_Mutation	p.L349F	Targerted Panel Gene	GCB-DLBCL
GCB-3	chr3	38141236	38141236	A	C	exonic	MYD88	474	200	0.2963	Missense_Mutation	p.T294P	Targerted Panel Gene	GCB-DLBCL
GCB-3	chr1	119915497	119915497	G	A	exonic	NOTCH2	418	142	0.2536	Nonsense_Mutation	p.Q2409X	Targerted Panel Gene	GCB-DLBCL
GCB-3	chr6	75256269	75256269	G	A	exonic	TMEM30A	325	162	0.3326	Nonsense_Mutation	p.R307X	Targerted Panel Gene	GCB-DLBCL
GCB-30	chr12	49041175	49041175	A	-	exonic	KMT2D	182	107	0.3677	Frame_Shift_Del	p.Y2199fs	Targerted Panel Gene	GCB-DLBCL
GCB-30	chr12	18496122	18496122	C	-	exonic	PIK3C2G	273	213	0.4383	Frame_Shift_Del	p.P730fs	Targerted Panel Gene	GCB-DLBCL
GCB-30	chr3	46672023	46672023	C	A	exonic	ALS2CL	151	132	0.4664	Missense_Mutation	p.D849Y	Targerted Panel Gene	GCB-DLBCL
GCB-30	chrX	101353872	101353872	A	G	exonic	BTK	121	245	0.6676	Missense_Mutation	p.F617S	Targerted Panel Gene	GCB-DLBCL

GCB-30	chr8	143869167	143869167	C	T	exonic	EPPK1	248	80	0.2439	Missense_Mutation	p.G1363R	Targerted Panel Gene	GCB-DLBCL
GCB-30	chr13	40665635	40665635	C	T	exonic	FOXO1	55	44	0.4444	Missense_Mutation	p.S193N	Targerted Panel Gene	GCB-DLBCL
GCB-30	chr2	141254522	141254522	C	A	exonic	LRP1B	393	224	0.363	Missense_Mutation	p.D155Y	Targerted Panel Gene	GCB-DLBCL
GCB-30	chr3	46998079	46998079	G	T	exonic	NBEAL2	127	65	0.3368	Missense_Mutation	p.A991S	Targerted Panel Gene	GCB-DLBCL
GCB-30	chr9	136507350	136507350	C	T	exonic	NOTCH1	149	70	0.3196	Missense_Mutation	p.D1200N	Targerted Panel Gene	GCB-DLBCL
GCB-30	chr17	7674220	7674220	C	T	exonic	TP53	86	154	0.6417	Missense_Mutation	p.R209Q	Targerted Panel Gene	GCB-DLBCL
GCB-30	chr19	22088126	22088126	T	C	exonic	ZNF257	49	4	0.0755	Missense_Mutation	p.C50R	Targerted Panel Gene	GCB-DLBCL
GCB-30	chr12	49037722	49037722	-	TA	exonic	KMT2D	361	157	0.3025	Nonsense_Mutation	p.K3212_F3213del	Targerted Panel Gene	GCB-DLBCL
GCB-31	chr19	19146293	19146293	G	-	exonic	MEF2B	39	19	0.3276	Frame_Shift_Del	p.P287fs	Targerted Panel Gene	GCB-DLBCL
GCB-31	chr2	32607477	32607477	T	C	exonic	BIRC6	528	175	0.2482	Missense_Mutation	p.L4698P	Targerted Panel Gene	GCB-DLBCL
GCB-31	chr1	203305709	203305709	C	T	exonic	BTG2	100	44	0.3056	Missense_Mutation	p.L35F	Targerted Panel Gene	GCB-DLBCL
GCB-31	chr4	186708155	186708155	G	T	exonic	FAT1	628	203	0.2443	Missense_Mutation	p.T558N	Targerted Panel Gene	GCB-DLBCL
GCB-31	chr7	111124267	111124267	A	G	exonic	LRRN3	642	196	0.2339	Missense_Mutation	p.T499A	Targerted Panel Gene	GCB-DLBCL
GCB-31	chr7	111124261	111124261	A	G	exonic	LRRN3	623	187	0.2309	Missense_Mutation	p.I497V	Targerted Panel Gene	GCB-DLBCL
GCB-31	chr10	87864541	87864541	C	G	exonic	PTEN	392	139	0.2618	Missense_Mutation	p.D197E	Targerted Panel Gene	GCB-DLBCL
GCB-31	chr3	16378057	16378057	G	A	exonic	RFTN1	316	99	0.2386	Missense_Mutation	p.P163S	Targerted Panel Gene	GCB-DLBCL
GCB-31	chr3	16378086	16378086	C	T	exonic	RFTN1	250	84	0.2515	Missense_Mutation	p.S153N	Targerted Panel Gene	GCB-DLBCL
GCB-31	chr3	49368509	49368509	A	T	exonic	RHOA	383	136	0.2615	Missense_Mutation	p.Y66N	Targerted Panel Gene	GCB-DLBCL
GCB-31	chr16	30964835	30964835	A	C	exonic	SETD1A	537	180	0.251	Missense_Mutation	p.N365H	Targerted Panel Gene	GCB-DLBCL
GCB-31	chr16	11255477	11255477	A	G	exonic	SOCS1	175	68	0.2787	Missense_Mutation	p.M1T	Targerted Panel Gene	GCB-DLBCL
GCB-31	chr16	11255141	11255141	A	G	exonic	SOCS1	150	45	0.2308	Missense_Mutation	p.F113S	Targerted Panel Gene	GCB-DLBCL
GCB-31	chr16	11255279	11255279	A	T	exonic	SOCS1	93	27	0.225	Missense_Mutation	p.I67N	Targerted Panel Gene	GCB-DLBCL
GCB-31	chr12	57099034	57099034	T	G	exonic	STAT6	550	201	0.2673	Missense_Mutation	p.I646L	Targerted Panel Gene	GCB-DLBCL
GCB-31	chr19	22088126	22088126	T	C	exonic	ZNF257	117	16	0.1203	Missense_Mutation	p.C50R	Targerted Panel Gene	GCB-DLBCL
GCB-31	chr19	22088127	22088127	G	A	exonic	ZNF257	117	16	0.1203	Missense_Mutation	p.C50Y	Targerted Panel Gene	GCB-DLBCL
GCB-31	chr19	22088133	22088133	G	A	exonic	ZNF257	121	16	0.1168	Missense_Mutation	p.G52E	Targerted Panel Gene	GCB-DLBCL
GCB-31	chr19	22088132	22088132	G	C	exonic	ZNF257	122	16	0.1159	Missense_Mutation	p.G52R	Targerted Panel Gene	GCB-DLBCL
GCB-32	chr6	44265696	44265696	T	-	exonic	NFKBIE	123	146	0.5428	Frame_Shift_Del	p.K23fs	Targerted Panel Gene	GCB-DLBCL
GCB-32	chr14	100276495	100276496	CA	-	exonic	YY1	327	211	0.3915	Frame_Shift_Del	p.C303fs	Targerted Panel Gene	GCB-DLBCL
GCB-32	chr19	39875632	39875632	G	C	exonic	FCGBP	251	256	0.5049	Missense_Mutation	p.C4654W	Targerted Panel Gene	GCB-DLBCL
GCB-32	chr16	85903178	85903178	T	G	exonic	IRF8	75	376	0.8337	Missense_Mutation	p.S55A	Targerted Panel Gene	GCB-DLBCL
GCB-32	chr6	44261763	44261763	T	A	exonic	NFKBIE	139	74	0.3474	Missense_Mutation	p.D324V	Targerted Panel Gene	GCB-DLBCL
GCB-32	chr19	38565458	38565458	C	G	exonic	RYR1	36	40	0.5263	Missense_Mutation	p.T4375R	Targerted Panel Gene	GCB-DLBCL
GCB-32	chr6	137875998	137875998	A	T	exonic	TNFAIP3	404	303	0.4286	Nonsense_Mutation	p.K213X	Targerted Panel Gene	GCB-DLBCL
GCB-33	chr12	18420941	18420941	T	-	exonic	PIK3C2G	426	171	0.2855	Frame_Shift_Del	p.S550fs	Targerted Panel Gene	GCB-DLBCL
GCB-33	chr15	44715554	44715554	-	A	exonic	B2M	365	550	0.5998	Frame_Shift_Ins	p.E67fs	Targerted Panel Gene	GCB-DLBCL
GCB-33	chr12	40320083	40320083	T	G	exonic	LRRK2	502	209	0.2935	Missense_Mutation	p.F1641L	Targerted Panel Gene	GCB-DLBCL
GCB-33	chr12	40249829	40249829	A	T	exonic	LRRK2	364	133	0.2671	Missense_Mutation	p.N281I	Targerted Panel Gene	GCB-DLBCL
GCB-33	chrX	141907038	141907038	C	T	exonic	MAGEC1	60	159	0.726	Missense_Mutation	p.P545L	Targerted Panel Gene	GCB-DLBCL
GCB-33	chr7	82822489	82822489	T	C	exonic	PCLO	855	290	0.2531	Missense_Mutation	p.R4933G	Targerted Panel Gene	GCB-DLBCL
GCB-33	chr19	4511501	4511501	G	C	exonic	PLIN4	1431	140	0.0891	Missense_Mutation	p.T806R	Targerted Panel Gene	GCB-DLBCL
GCB-33	chr17	7674888	7674888	T	G	exonic	TP53	222	204	0.4789	Missense_Mutation	p.S176R	Targerted Panel Gene	GCB-DLBCL
GCB-33	chr8	102311869	102311869	T	A	exonic	UBR5	352	196	0.3577	Missense_Mutation	p.T762S	Targerted Panel Gene	GCB-DLBCL
GCB-33	chr8	102281383	102281383	T	C	exonic	UBR5	489	223	0.3132	Missense_Mutation	p.K1945E	Targerted Panel Gene	GCB-DLBCL
GCB-33	chr16	72797998	72797998	C	T	exonic	ZFHX3	825	412	0.3331	Missense_Mutation	p.V1562I	Targerted Panel Gene	GCB-DLBCL
GCB-33	chr12	49033106	49033106	G	A	exonic	KMT2D	142	77	0.3516	Nonsense_Mutation	p.Q3867X	Targerted Panel Gene	GCB-DLBCL
GCB-33	chr12	49050467	49050467	G	A	exonic	KMT2D	562	292	0.3407	Nonsense_Mutation	p.Q1041X	Targerted Panel Gene	GCB-DLBCL
GCB-33	chr1	2559875	2559875	T	A	exonic	TNFRSF14	60	96	0.6154	Nonsense_Mutation	p.C119X	Targerted Panel Gene	GCB-DLBCL
GCB-34	chr6	52026044	52026044	G	-	exonic	PKHD1	594	400	0.402	Frame_Shift_Del	p.Q1256fs	Targerted Panel Gene	GCB-DLBCL
GCB-34	chr9	136505560	136505560	C	T	exonic	NOTCH1	251	240	0.4888	Missense_Mutation	p.E1446K	Targerted Panel Gene	GCB-DLBCL
GCB-34	chr1	228362749	228362749	G	A	exonic	OBSCN	233	193	0.4531	Missense_Mutation	p.A7236T	Targerted Panel Gene	GCB-DLBCL
GCB-34	chr1	216070175	216070175	T	C	exonic	USH2A	628	602	0.4894	Missense_Mutation	p.Y1992C	Targerted Panel Gene	GCB-DLBCL
GCB-35	chr6	137874862	137874863	AT	-	exonic	TNFAIP3	232	90	0.2795	Frame_Shift_Del	p.M105fs	Targerted Panel Gene	GCB-DLBCL
GCB-35	chr1	2556660	2556667	GAGGCATG	-	exonic	TNFRSF14	98	39	0.2847	Frame_Shift_Del	wholegene	Targerted Panel Gene	GCB-DLBCL
GCB-35	chr1	85267912	85267912	A	T	exonic	BCL10	426	153	0.2642	Missense_Mutation	p.D139E	Targerted Panel Gene	GCB-DLBCL
GCB-35	chr2	124527361	124527361	T	G	exonic	CNTNAP5	792	232	0.2266	Missense_Mutation	p.F517L	Targerted Panel Gene	GCB-DLBCL
GCB-35	chr1	102883262	102883262	T	G	exonic	COL11A1	390	145	0.271	Missense_Mutation	p.K1648N	Targerted Panel Gene	GCB-DLBCL
GCB-35	chrX	41346330	41346330	G	A	exonic	DDX3X	221	180	0.4489	Missense_Mutation	p.G473R	Targerted Panel Gene	GCB-DLBCL
GCB-35	chr3	65439986	65439986	T	C	exonic	MAGI1	333	99	0.2292	Missense_Mutation	p.E388G	Targerted Panel Gene	GCB-DLBCL
GCB-35	chr1	228316068	228316068	G	T	exonic	OBSCN	179	194	0.5201	Missense_Mutation	p.V5369L	Targerted Panel Gene	GCB-DLBCL
GCB-35	chr8	100718194	100718194	G	A	exonic	PABPC1	395	96	0.1955	Missense_Mutation	p.R94C	Targerted Panel Gene	GCB-DLBCL
GCB-35	chr7	82908881	82908881	T	C	exonic	PCLO	278	265	0.488	Missense_Mutation	p.E4478G	Targerted Panel Gene	GCB-DLBCL
GCB-35	chr16	88722898	88722898	T	G	exonic	PIEZO1	58	62	0.5167	Missense_Mutation	p.H1536P	Targerted Panel Gene	GCB-DLBCL
GCB-35	chr1	237590854	237590854	C	G	exonic	RYR2	669	207	0.2363	Missense_Mutation	p.S1341C	Targerted Panel Gene	GCB-DLBCL
GCB-35	chr1	2556675	2556675	C	G	exonic	TNFRSF14	91	45	0.3309	Missense_Mutation	p.P4R	Targerted Panel Gene	GCB-DLBCL
GCB-35	chr1	85267911	85267911	C	A	exonic	BCL10	426	153	0.2638	Nonsense_Mutation	p.E140X	Targerted Panel Gene	GCB-DLBCL
GCB-35	chr22	41127646	41127646	C	T	exonic	EP300	308	99	0.2432	Nonsense_Mutation	p.Q356X	Targerted Panel Gene	GCB-DLBCL
GCB-36	chr6	137874839	137874857	CCTCAGGTGACGG	-	exonic	TNFAIP3	173	75	0.3024	Frame_Shift_Del	p.G99fs	Targerted Panel Gene	GCB-DLBCL
GCB-36	chr16	85903030	85903032	TGG	-	exonic	IRF8	269	205	0.4316	In_Frame_Del	p.5_6del	Targerted Panel Gene	GCB-DLBCL
GCB-36	chr12	92145532	92145532	G	T	exonic	BTG1	165	32	0.1624	Missense_Mutation	p.H2N	Targerted Panel Gene	GCB-DLBCL
GCB-36	chr12	92145475	92145475	A	G	exonic	BTG1	162	27	0.1429	Missense_Mutation	p.F21L	Targerted Panel Gene	GCB-DLBCL
GCB-36	chr12	92145463	92145463	A	T	exonic	BTG1	156	25	0.1381	Missense_Mutation	p.F25I	Targerted Panel Gene	GCB-DLBCL
GCB-36	chr19	6586304	6586304	G	A	exonic	CD70	290	114	0.2822	Missense_Mutation	p.R100C	Targerted Panel Gene	GCB-DLBCL
GCB-36	chr5	138329610	138329610	T	C	exonic	CDC25C	279	155	0.3571	Missense_Mutation	p.S78G	Targerted Panel Gene	GCB-DLBCL
GCB-36	chr8	3343314	3343314	C	A	exonic	CSMD1	505	201	0.2847	Missense_Mutation	p.G1204V	Targerted Panel Gene	GCB-DLBCL
GCB-36	chr11	128521928	128521928	G	A	exonic	ETS1	226	320	0.5861	Missense_Mutation	p.L23F	Targerted Panel Gene	GCB-DLBCL
GCB-36	chr10	89014227	89014227	T	A	exonic	FAS	374	202	0.3507	Missense_Mutation	p.I262N	Targerted Panel Gene	GCB-DLBCL
GCB-36	chr6	26250200	26250200	C	T	exonic	HIST1H3F	50	36	0.4186	Missense_Mutation	p.A136T	Targerted Panel Gene	GCB-DLBCL
GCB-36	chr4	142429193	142429193	G	A	exonic	INPP4B	297	52	0.149	Missense_Mutation	p.P39L	Targerted Panel Gene	GCB-DLBCL
GCB-36	chr11	118504078	118504078	G	A	exonic	KMT2A	849	269	0.2406	Missense_Mutation	p.S2726N	Targerted Panel Gene	GCB-DLBCL
GCB-36	chr12	49029165	49029165	C	T	exonic	KMT2D	289	44	0.1321	Missense_Mutation	p.G4716E	Targerted Panel Gene	GCB-DLBCL
GCB-36	chr9	13119594	13119594	C	T	exonic	MPDZ	444	232	0.3432	Missense_Mutation	p.D1763N	Targerted Panel Gene	GCB-DLBCL

GCB-36	chr6	37171125	37171125	C	T	exonic	PIM1	288	193	0.4012	Missense_Mutation	p.P81S	Targerted Panel Gene	GCB-DLBCL
GCB-36	chr10	87864498	87864498	G	A	exonic	PTEN	376	73	0.1626	Missense_Mutation	p.S183N	Targerted Panel Gene	GCB-DLBCL
GCB-36	chr10	87864548	87864548	T	A	exonic	PTEN	294	121	0.2916	Missense_Mutation	p.Y200N	Targerted Panel Gene	GCB-DLBCL
GCB-36	chr6	134174512	134174512	G	A	exonic	SGK1	325	59	0.1536	Missense_Mutation	p.H146Y	Targerted Panel Gene	GCB-DLBCL
GCB-36	chr6	134174576	134174576	C	G	exonic	SGK1	263	107	0.2892	Missense_Mutation	p.K124N	Targerted Panel Gene	GCB-DLBCL
GCB-36	chr6	134174586	134174586	G	A	exonic	SGK1	313	45	0.1253	Missense_Mutation	p.A121V	Targerted Panel Gene	GCB-DLBCL
GCB-36	chr6	134174557	134174557	T	G	exonic	SGK1	278	112	0.2872	Missense_Mutation	p.N131H	Targerted Panel Gene	GCB-DLBCL
GCB-36	chr16	11255183	11255183	C	T	exonic	SOCS1	96	43	0.3094	Missense_Mutation	p.G99D	Targerted Panel Gene	GCB-DLBCL
GCB-36	chr16	11254953	11254953	C	T	exonic	SOCS1	168	60	0.2632	Missense_Mutation	p.E176K	Targerted Panel Gene	GCB-DLBCL
GCB-36	chr9	90877732	90877732	T	C	exonic	SYK	321	168	0.3436	Missense_Mutation	p.M448T	Targerted Panel Gene	GCB-DLBCL
GCB-36	chr17	7674229	7674229	C	T	exonic	TP53	224	88	0.2821	Missense_Mutation	p.G206D	Targerted Panel Gene	GCB-DLBCL
GCB-36	chr17	59765061	59765061	C	G	exonic	VMP1	429	358	0.4549	Missense_Mutation	p.P169A	Targerted Panel Gene	GCB-DLBCL
GCB-36	chr19	24127433	24127433	C	G	exonic	ZNF254	155	60	0.2791	Missense_Mutation	p.T437S	Targerted Panel Gene	GCB-DLBCL
GCB-36	chr19	30444541	30444541	G	A	exonic	ZNF536	152	66	0.3028	Missense_Mutation	p.E327K	Targerted Panel Gene	GCB-DLBCL
GCB-36	chr1	203307379	203307379	C	T	exonic	BTG2	285	34	0.1066	Nonsense_Mutation	p.Q140X	Targerted Panel Gene	GCB-DLBCL
GCB-36	chr14	100239686	100239686	C	T	exonic	YY1	164	52	0.2407	Nonsense_Mutation	p.Q148X	Targerted Panel Gene	GCB-DLBCL
GCB-37	chr5	79733007	79733009	AGA	-	exonic	CMYA5	422	302	0.4154	In_Frame_Del	p.1414_1415del	Targerted Panel Gene	GCB-DLBCL
GCB-37	chr11	6544841	6544841	T	A	exonic	DNHD1	212	213	0.5012	Missense_Mutation	p.I1301N	Targerted Panel Gene	GCB-DLBCL
GCB-37	chr8	143871361	143871361	G	C	exonic	EPPK1	121	155	0.5616	Missense_Mutation	p.C631W	Targerted Panel Gene	GCB-DLBCL
GCB-37	chr4	84801741	84801741	C	A	exonic	WDFY3	177	185	0.511	Missense_Mutation	p.A911S	Targerted Panel Gene	GCB-DLBCL
GCB-38	chr19	19149234	19149239	TGTCAG	-	exonic	MEF2B,MEF2BNB-(	336	108	0.2432	In_Frame_Del	p.82_84del	Targerted Panel Gene	GCB-DLBCL
GCB-38	chr5	112780865	112780865	C	G	exonic	APC	430	389	0.475	Missense_Mutation	p.Q203E	Targerted Panel Gene	GCB-DLBCL
GCB-38	chr18	63318565	63318565	A	T	exonic	BCL2	286	191	0.3996	Missense_Mutation	p.D34E	Targerted Panel Gene	GCB-DLBCL
GCB-38	chr18	63128740	63128740	T	A	exonic	BCL2	336	169	0.334	Missense_Mutation	p.Y202F	Targerted Panel Gene	GCB-DLBCL
GCB-38	chr12	122022108	122022108	T	A	exonic	BCL7A	155	66	0.2986	Missense_Mutation	p.V6D	Targerted Panel Gene	GCB-DLBCL
GCB-38	chr7	2945834	2945834	A	T	exonic	CARD11	485	233	0.3245	Missense_Mutation	p.F115I	Targerted Panel Gene	GCB-DLBCL
GCB-38	chr2	124647769	124647769	T	A	exonic	CNTNAP5	133	109	0.4504	Missense_Mutation	p.W629R	Targerted Panel Gene	GCB-DLBCL
GCB-38	chr8	112287157	112287157	A	C	exonic	CSMD3	274	507	0.6492	Missense_Mutation	p.Y3040D	Targerted Panel Gene	GCB-DLBCL
GCB-38	chr2	136115894	136115894	A	T	exonic	CXCR4	726	198	0.2141	Missense_Mutation	p.Y12N	Targerted Panel Gene	GCB-DLBCL
GCB-38	chr5	138465874	138465874	T	A	exonic	EGR1	269	130	0.3258	Missense_Mutation	p.M38K	Targerted Panel Gene	GCB-DLBCL
GCB-38	chr5	138465831	138465831	C	T	exonic	EGR1	286	132	0.3158	Missense_Mutation	p.P24S	Targerted Panel Gene	GCB-DLBCL
GCB-38	chr22	41170518	41170518	T	G	exonic	EP300	465	88	0.1591	Missense_Mutation	p.Y1467D	Targerted Panel Gene	GCB-DLBCL
GCB-38	chr7	148809345	148809345	G	A	exonic	EZH2	444	228	0.3388	Missense_Mutation	p.A692V	Targerted Panel Gene	GCB-DLBCL
GCB-38	chr3	50256953	50256953	G	T	exonic	GNAI2	339	209	0.3814	Missense_Mutation	p.S231I	Targerted Panel Gene	GCB-DLBCL
GCB-38	chr3	50256262	50256262	C	T	exonic	GNAI2	283	158	0.3583	Missense_Mutation	p.R163C	Targerted Panel Gene	GCB-DLBCL
GCB-38	chr7	82953703	82953703	G	T	exonic	PCLO	229	125	0.3531	Missense_Mutation	p.P2417H	Targerted Panel Gene	GCB-DLBCL
GCB-38	chr3	49368544	49368544	T	C	exonic	RHOA	381	193	0.3362	Missense_Mutation	p.E54G	Targerted Panel Gene	GCB-DLBCL
GCB-38	chr4	105235773	105235773	A	T	exonic	TET2	645	193	0.2303	Missense_Mutation	p.M611L	Targerted Panel Gene	GCB-DLBCL
GCB-38	chr2	167247857	167247857	T	G	exonic	XIRP2	706	74	0.0949	Missense_Mutation	p.D2155E	Targerted Panel Gene	GCB-DLBCL
GCB-38	chr19	30548501	30548501	G	C	exonic	ZNF536	611	342	0.3585	Missense_Mutation	p.G961A	Targerted Panel Gene	GCB-DLBCL
GCB-38	chr15	44711566	44711566	T	A	exonic	B2M	93	102	0.5231	Nonsense_Mutation	p.L7X	Targerted Panel Gene	GCB-DLBCL
GCB-39	chr12	124402513	124402533	GCTGCTGCTGCTG(	-	exonic	NCOR2	59	31	0.3298	In_Frame_Del	p.504_511del	Targerted Panel Gene	GCB-DLBCL
GCB-39	chrX	32365035	32365035	C	A	exonic	DMD	299	267	0.4717	Missense_Mutation	p.W1547C	Targerted Panel Gene	GCB-DLBCL
GCB-39	chr4	186636112	186636112	T	C	exonic	FAT1	257	233	0.4755	Missense_Mutation	p.T1366A	Targerted Panel Gene	GCB-DLBCL
GCB-39	chr1	233262107	233262107	G	A	exonic	PCNXL2	208	175	0.4569	Missense_Mutation	p.P140S	Targerted Panel Gene	GCB-DLBCL
GCB-4	chr12	122022184	122022184	G	A	splicing	BCL7A	109	35	0.2431	Splice_Site	.	Targerted Panel Gene	GCB-DLBCL
GCB-4	chr16	3879831	3879831	C	T	splicing	CREBBP	205	30	0.1277	Splice_Site	.	Targerted Panel Gene	GCB-DLBCL
GCB-4	chr2	50925937	50925937	C	T	splicing	NRXN1	412	52	0.1121	Splice_Site	.	Targerted Panel Gene	GCB-DLBCL
GCB-4	chr16	11255529	11255529	C	G	splicing	SOCS1	154	24	0.1348	Splice_Site	.	Targerted Panel Gene	GCB-DLBCL
GCB-4	chr12	92145415	92145415	T	G	exonic	BTG1	134	19	0.1242	Missense_Mutation	p.S41R	Targerted Panel Gene	GCB-DLBCL
GCB-4	chrX	101353974	101353974	T	C	exonic	BTK	358	53	0.129	Missense_Mutation	p.D583G	Targerted Panel Gene	GCB-DLBCL
GCB-4	chr7	2937980	2937980	T	A	exonic	CARD11	249	27	0.0978	Missense_Mutation	p.D357V	Targerted Panel Gene	GCB-DLBCL
GCB-4	chr7	148811635	148811635	T	A	exonic	EZH2	328	34	0.0939	Missense_Mutation	p.Y646F	Targerted Panel Gene	GCB-DLBCL
GCB-4	chr13	40666151	40666151	C	T	exonic	FOXO1	48	9	0.1579	Missense_Mutation	p.R21H	Targerted Panel Gene	GCB-DLBCL
GCB-4	chr17	65014696	65014696	C	T	exonic	GNA13	827	143	0.1474	Missense_Mutation	p.R232H	Targerted Panel Gene	GCB-DLBCL
GCB-4	chr22	22888234	22888234	T	G	exonic	IGLL5	143	23	0.1386	Missense_Mutation	p.D25E	Targerted Panel Gene	GCB-DLBCL
GCB-4	chr22	22888182	22888182	T	C	exonic	IGLL5	181	23	0.1127	Missense_Mutation	p.L8S	Targerted Panel Gene	GCB-DLBCL
GCB-4	chr22	22888157	22888157	C	T	exonic	IGLL5	171	22	0.114	Missense_Mutation	p.A35V	Targerted Panel Gene	GCB-DLBCL
GCB-4	chr16	85903082	85903082	T	C	exonic	IRF8	500	69	0.1213	Missense_Mutation	p.Y23H	Targerted Panel Gene	GCB-DLBCL
GCB-4	chr11	118498406	118498406	G	T	exonic	KMT2A	247	205	0.4535	Missense_Mutation	p.G1944C	Targerted Panel Gene	GCB-DLBCL
GCB-4	chr7	152138797	152138797	T	A	exonic	KMT2C	362	50	0.1214	Missense_Mutation	p.E4881V	Targerted Panel Gene	GCB-DLBCL
GCB-4	chr19	38485882	38485882	G	A	exonic	RYR1	218	27	0.1102	Missense_Mutation	p.A1743T	Targerted Panel Gene	GCB-DLBCL
GCB-4	chr6	134174066	134174066	G	A	exonic	SGK1	339	32	0.086	Missense_Mutation	p.S151F	Targerted Panel Gene	GCB-DLBCL
GCB-4	chr4	84708956	84708956	T	A	exonic	WDFY3	329	44	0.118	Missense_Mutation	p.M2724L	Targerted Panel Gene	GCB-DLBCL
GCB-4	chr19	41880735	41880735	T	A	exonic	CD79A	333	45	0.1187	Nonsense_Mutation	p.Y150X	Targerted Panel Gene	GCB-DLBCL
GCB-4	chr1	3858750	3858750	C	G	exonic	DFFB	133	89	0.3991	Nonsense_Mutation	p.Y49X	Targerted Panel Gene	GCB-DLBCL
GCB-40	chr17	63478005	63478005	C	G	exonic	ACE	170	43	0.2019	Missense_Mutation	p.I108M	Targerted Panel Gene	GCB-DLBCL
GCB-40	chr17	47181582	47181582	G	A	exonic	CDC27	117	34	0.2252	Missense_Mutation	p.A28V	Targerted Panel Gene	GCB-DLBCL
GCB-40	chr3	48581285	48581285	C	T	exonic	COL7A1	290	82	0.2204	Missense_Mutation	p.G1625E	Targerted Panel Gene	GCB-DLBCL
GCB-40	chr19	16903770	16903770	T	C	exonic	CPAMD8	187	165	0.4688	Missense_Mutation	p.T1494A	Targerted Panel Gene	GCB-DLBCL
GCB-40	chr4	93455873	93455873	T	A	exonic	GRID2	768	144	0.1579	Missense_Mutation	p.L586H	Targerted Panel Gene	GCB-DLBCL
GCB-40	chr6	27147356	27147356	G	C	exonic	HIST1H2AH	48	12	0.2	Missense_Mutation	p.K76N	Targerted Panel Gene	GCB-DLBCL
GCB-40	chr6	26197244	26197244	G	C	exonic	HIST1H3D	164	33	0.1675	Missense_Mutation	p.R3G	Targerted Panel Gene	GCB-DLBCL
GCB-40	chr13	35628126	35628126	G	A	exonic	NBEA	455	83	0.154	Missense_Mutation	p.D2499N	Targerted Panel Gene	GCB-DLBCL
GCB-40	chr6	117403217	117403217	G	C	exonic	ROS1	196	147	0.4273	Missense_Mutation	p.R167G	Targerted Panel Gene	GCB-DLBCL
GCB-40	chr19	11019630	11019630	C	T	exonic	SMARCA4	243	58	0.1927	Missense_Mutation	p.R849W	Targerted Panel Gene	GCB-DLBCL
GCB-40	chr10	62814143	62814143	G	C	exonic	EGR2	534	117	0.1797	Nonsense_Mutation	p.Y165X	Targerted Panel Gene	GCB-DLBCL
GCB-41	chr15	44711581	44711581	T	A	exonic	B2M	77	80	0.5096	Missense_Mutation	p.L12Q	Targerted Panel Gene	GCB-DLBCL
GCB-41	chr7	2944405	2944405	T	C	exonic	CARD11	271	160	0.3712	Missense_Mutation	p.K164R	Targerted Panel Gene	GCB-DLBCL
GCB-41	chr7	2944253	2944253	T	C	exonic	CARD11	306	163	0.3475	Missense_Mutation	p.K215E	Targerted Panel Gene	GCB-DLBCL
GCB-41	chr11	92844316	92844316	C	T	exonic	FAT3	240	386	0.6166	Missense_Mutation	p.T3650I	Targerted Panel Gene	GCB-DLBCL
GCB-41	chr7	150474060	150474060	G	C	exonic	GIMAP8	383	222	0.3669	Missense_Mutation	p.G244A	Targerted Panel Gene	GCB-DLBCL

GCB-41	chr6	27867264	27867264	C	G	exonic	HIST1H1B	247	170	0.4077	Missense_Mutation	p.S89T	Targerted Panel Gene	GCB-DLBCL
GCB-41	chr16	85921271	85921271	A	T	exonic	IRF8	407	252	0.3824	Missense_Mutation	p.I424F	Targerted Panel Gene	GCB-DLBCL
GCB-41	chr12	40235634	40235634	T	C	exonic	LRRK2	287	238	0.4533	Missense_Mutation	p.L119P	Targerted Panel Gene	GCB-DLBCL
GCB-41	chr7	111123502	111123502	T	A	exonic	LRRN3	622	317	0.3376	Missense_Mutation	p.Y244N	Targerted Panel Gene	GCB-DLBCL
GCB-41	chr13	35173593	35173593	A	T	exonic	NBEA	194	97	0.3333	Missense_Mutation	p.K1518M	Targerted Panel Gene	GCB-DLBCL
GCB-41	chr13	35550975	35550975	T	C	exonic	NBEA	199	79	0.2842	Missense_Mutation	p.V2250A	Targerted Panel Gene	GCB-DLBCL
GCB-41	chr7	82915258	82915258	A	G	exonic	PCLO	559	299	0.3485	Missense_Mutation	p.F4243S	Targerted Panel Gene	GCB-DLBCL
GCB-41	chr15	41882694	41882694	C	T	exonic	SPTBN5	34	55	0.618	Missense_Mutation	p.R646H	Targerted Panel Gene	GCB-DLBCL
GCB-41	chr4	105236554	105236554	A	C	exonic	TET2	354	288	0.4479	Missense_Mutation	p.N871T	Targerted Panel Gene	GCB-DLBCL
GCB-41	chr4	105236698	105236698	A	T	exonic	TET2	334	234	0.4112	Missense_Mutation	p.Y919F	Targerted Panel Gene	GCB-DLBCL
GCB-41	chr1	2556665	2556665	A	G	exonic	TNFRSF14	75	117	0.6094	Missense_Mutation	p.M1V	Targerted Panel Gene	GCB-DLBCL
GCB-41	chr1	216073201	216073201	C	T	exonic	USH2A	370	199	0.3497	Missense_Mutation	p.C1891Y	Targerted Panel Gene	GCB-DLBCL
GCB-41	chr1	116536094	116536094	T	A	exonic	CD58	380	328	0.4626	Nonsense_Mutation	p.K167X	Targerted Panel Gene	GCB-DLBCL
GCB-41	chr1	116544415	116544415	G	C	exonic	CD58	499	131	0.2076	Nonsense_Mutation	p.S87X	Targerted Panel Gene	GCB-DLBCL
GCB-42	chr3	187725614	187725614	C	T	exonic	BCL6	419	431	0.5071	Missense_Mutation	p.R575H	Targerted Panel Gene	GCB-DLBCL
GCB-42	chr7	148811636	148811636	A	T	exonic	EZH2	277	107	0.2786	Missense_Mutation	p.Y646N	Targerted Panel Gene	GCB-DLBCL
GCB-42	chr16	27362276	27362276	G	C	exonic	IL4R	376	150	0.2841	Missense_Mutation	p.K293N	Targerted Panel Gene	GCB-DLBCL
GCB-42	chr10	63177789	63177789	A	T	exonic	JMJD1C	522	208	0.2849	Missense_Mutation	p.S2384R	Targerted Panel Gene	GCB-DLBCL
GCB-42	chr2	140371268	140371268	A	G	exonic	LRP1B	180	183	0.5014	Missense_Mutation	p.S3596P	Targerted Panel Gene	GCB-DLBCL
GCB-42	chr2	169206663	169206663	A	G	exonic	LRP2	623	264	0.2973	Missense_Mutation	p.C2353R	Targerted Panel Gene	GCB-DLBCL
GCB-42	chr16	11255161	11255161	G	T	exonic	SOCS1	128	55	0.2989	Missense_Mutation	p.S106R	Targerted Panel Gene	GCB-DLBCL
GCB-42	chr12	57099042	57099042	G	A	exonic	STAT6	527	386	0.4228	Missense_Mutation	p.P643L	Targerted Panel Gene	GCB-DLBCL
GCB-43	chr3	187726764	187726764	C	G	exonic	BCL6	426	591	0.58	Missense_Mutation	p.G559R	Targerted Panel Gene	GCB-DLBCL
GCB-43	chr22	22888142	22888142	G	A	exonic	IGLL5	18	86	0.8269	Missense_Mutation	p.G30D	Targerted Panel Gene	GCB-DLBCL
GCB-43	chr7	111123122	111123122	A	T	exonic	LRRN3	530	386	0.4214	Missense_Mutation	p.Q117L	Targerted Panel Gene	GCB-DLBCL
GCB-43	chr9	37034010	37034010	G	A	exonic	PAX5	166	142	0.461	Missense_Mutation	p.P8S	Targerted Panel Gene	GCB-DLBCL
GCB-43	chr19	4510520	4510520	G	A	exonic	PLIN4	179	137	0.4335	Missense_Mutation	p.P1133L	Targerted Panel Gene	GCB-DLBCL
GCB-43	chr6	128219086	128219086	C	T	exonic	PTPRK	348	210	0.3757	Missense_Mutation	p.G235E	Targerted Panel Gene	GCB-DLBCL
GCB-43	chr9	4844595	4844595	T	G	exonic	RCL1	245	166	0.4029	Missense_Mutation	p.S261A	Targerted Panel Gene	GCB-DLBCL
GCB-43	chr3	16377780	16377780	C	T	exonic	RFTN1	681	77	0.1016	Missense_Mutation	p.S255N	Targerted Panel Gene	GCB-DLBCL
GCB-43	chr17	7674230	7674230	C	T	exonic	TP53	36	145	0.8011	Missense_Mutation	p.G206S	Targerted Panel Gene	GCB-DLBCL
GCB-43	chr8	112550673	112550673	G	T	exonic	CSMD3	241	169	0.4122	Nonsense_Mutation	p.S1481X	Targerted Panel Gene	GCB-DLBCL
GCB-44	chr12	122022131	122022131	G	A	exonic	BCL7A	102	72	0.4138	Missense_Mutation	p.A14T	Targerted Panel Gene	GCB-DLBCL
GCB-44	chr2	136115883	136115883	T	A	exonic	CXCR4	198	327	0.6229	Missense_Mutation	p.E15D	Targerted Panel Gene	GCB-DLBCL
GCB-44	chr22	41127729	41127729	G	C	exonic	EP300	200	116	0.3659	Missense_Mutation	p.Q383H	Targerted Panel Gene	GCB-DLBCL
GCB-44	chr1	235806035	235806035	A	G	exonic	LYST	548	953	0.6345	Missense_Mutation	p.M1034T	Targerted Panel Gene	GCB-DLBCL
GCB-44	chr10	94324908	94324908	A	T	exonic	PLCE1	505	120	0.192	Missense_Mutation	p.K2246I	Targerted Panel Gene	GCB-DLBCL
GCB-44	chr3	77493349	77493349	G	T	exonic	ROBO2	443	249	0.3598	Missense_Mutation	p.W258L	Targerted Panel Gene	GCB-DLBCL
GCB-44	chr2	61493010	61493010	A	C	exonic	XPO1	471	300	0.3891	Missense_Mutation	p.V430G	Targerted Panel Gene	GCB-DLBCL
GCB-44	chr16	72958882	72958882	G	A	exonic	ZFHX3	226	156	0.4084	Missense_Mutation	p.P422S	Targerted Panel Gene	GCB-DLBCL
GCB-44	chr12	49030376	49030376	G	A	exonic	KMT2D	62	50	0.4464	Nonsense_Mutation	p.Q4635X	Targerted Panel Gene	GCB-DLBCL
GCB-44	chr15	44716341	44716341	A	T	exonic	B2M	84	175	0.6757	Nonstop_Mutation	p.X120L	Targerted Panel Gene	GCB-DLBCL
GCB-45	chr11	102324747	102324747	G	A	exonic	BIRC3	1166	288	0.1978	Missense_Mutation	p.D80N	Targerted Panel Gene	GCB-DLBCL
GCB-45	chr1	203305735	203305735	G	C	exonic	BTG2	98	40	0.2899	Missense_Mutation	p.Q43H	Targerted Panel Gene	GCB-DLBCL
GCB-45	chr7	82005767	82005767	T	C	exonic	CACNA2D1	506	161	0.241	Missense_Mutation	p.T505A	Targerted Panel Gene	GCB-DLBCL
GCB-45	chr2	124434603	124434603	G	T	exonic	CNTNAP5	291	165	0.3611	Missense_Mutation	p.V217F	Targerted Panel Gene	GCB-DLBCL
GCB-45	chr16	3736799	3736799	T	C	exonic	CREBBP	290	128	0.3062	Missense_Mutation	p.I1471V	Targerted Panel Gene	GCB-DLBCL
GCB-45	chr8	112351237	112351237	G	A	exonic	CSMD3	426	118	0.2161	Missense_Mutation	p.S2048F	Targerted Panel Gene	GCB-DLBCL
GCB-45	chr18	36129935	36129935	T	A	exonic	ELP2	221	197	0.4713	Missense_Mutation	p.M1K	Targerted Panel Gene	GCB-DLBCL
GCB-45	chr11	92524825	92524825	C	A	exonic	FAT3	371	595	0.6159	Missense_Mutation	p.P1162T	Targerted Panel Gene	GCB-DLBCL
GCB-45	chr7	82822668	82822668	T	G	exonic	PCLO	492	162	0.2477	Missense_Mutation	p.E4873A	Targerted Panel Gene	GCB-DLBCL
GCB-45	chr6	51911828	51911828	A	G	exonic	PKHD1	225	188	0.4552	Missense_Mutation	p.V2154A	Targerted Panel Gene	GCB-DLBCL
GCB-45	chr3	16377731	16377731	C	G	exonic	RFTN1	205	126	0.3795	Missense_Mutation	p.E271D	Targerted Panel Gene	GCB-DLBCL
GCB-45	chr15	44711548	44711548	T	G	exonic	B2M	68	78	0.5342	Missense_Mutation	p.M1R	Targerted Panel Gene	GCB-DLBCL
GCB-45	chr16	3758871	3758871	G	A	exonic	CREBBP	305	176	0.3651	Nonsense_Mutation	p.Q1118X	Targerted Panel Gene	GCB-DLBCL
GCB-46	chr7	2937176	2937176	T	A	exonic	CARD11	314	55	0.1486	Missense_Mutation	p.D401V	Targerted Panel Gene	GCB-DLBCL
GCB-46	chr8	112650201	112650201	T	G	exonic	CSMD3	402	44	0.0987	Missense_Mutation	p.E1011D	Targerted Panel Gene	GCB-DLBCL
GCB-46	chr4	186708273	186708273	C	T	exonic	FAT1	571	63	0.0994	Missense_Mutation	p.V519M	Targerted Panel Gene	GCB-DLBCL
GCB-46	chr4	78472319	78472319	C	A	exonic	FRAS1	224	29	0.1146	Missense_Mutation	p.T2504N	Targerted Panel Gene	GCB-DLBCL
GCB-46	chr1	21847786	21847786	T	A	exonic	HSPG2	91	27	0.2288	Missense_Mutation	p.E2643V	Targerted Panel Gene	GCB-DLBCL
GCB-46	chr22	22895666	22895666	A	G	exonic	IGLL5	309	29	0.0858	Missense_Mutation	p.K206R	Targerted Panel Gene	GCB-DLBCL
GCB-46	chr19	38595980	38595980	A	C	exonic	MAP4K1	311	66	0.1751	Missense_Mutation	p.V713G	Targerted Panel Gene	GCB-DLBCL
GCB-46	chr9	13147591	13147591	T	C	exonic	MPDZ	431	43	0.0907	Missense_Mutation	p.N1233S	Targerted Panel Gene	GCB-DLBCL
GCB-46	chr8	100709694	100709694	A	G	exonic	PABPC1	304	30	0.0896	Missense_Mutation	p.F337S	Targerted Panel Gene	GCB-DLBCL
GCB-46	chr10	87894069	87894069	C	T	exonic	PTEN	318	46	0.1264	Missense_Mutation	p.L215F	Targerted Panel Gene	GCB-DLBCL
GCB-46	chr1	215675568	215675568	G	A	exonic	USH2A	248	258	0.5099	Missense_Mutation	p.R4115C	Targerted Panel Gene	GCB-DLBCL
GCB-46	chr14	100277461	100277461	A	G	exonic	YY1	499	64	0.1137	Missense_Mutation	p.N369S	Targerted Panel Gene	GCB-DLBCL
GCB-46	chr4	105236328	105236328	G	T	exonic	TET2	565	69	0.1088	Nonsense_Mutation	p.E796X	Targerted Panel Gene	GCB-DLBCL
GCB-46	chr4	105259684	105259684	C	G	exonic	TET2	454	46	0.0918	Nonsense_Mutation	p.S1290X	Targerted Panel Gene	GCB-DLBCL
GCB-5	chr12	122022184	122022184	G	A	splicing	BCL7A	117	71	0.3777	Splice_Site	.	Targerted Panel Gene	GCB-DLBCL
GCB-5	chr1	2556735	2556735	T	C	splicing	TNFRSF14	118	33	0.2185	Splice_Site	.	Targerted Panel Gene	GCB-DLBCL
GCB-5	chr1	26696975	26696975	G	T	exonic	ARID1A	116	110	0.4867	Missense_Mutation	p.G191V	Targerted Panel Gene	GCB-DLBCL
GCB-5	chr15	44711547	44711547	A	C	exonic	B2M	118	44	0.2716	Missense_Mutation	p.M1L	Targerted Panel Gene	GCB-DLBCL
GCB-5	chr18	63318527	63318527	C	T	exonic	BCL2	158	67	0.2978	Missense_Mutation	p.G47D	Targerted Panel Gene	GCB-DLBCL
GCB-5	chr18	63318074	63318074	G	A	exonic	BCL2	407	168	0.2922	Missense_Mutation	p.A198V	Targerted Panel Gene	GCB-DLBCL
GCB-5	chr18	63318062	63318062	A	C	exonic	BCL2	409	141	0.2564	Missense_Mutation	p.V202G	Targerted Panel Gene	GCB-DLBCL
GCB-5	chr18	63318587	63318587	C	T	exonic	BCL2	299	92	0.2353	Missense_Mutation	p.G27D	Targerted Panel Gene	GCB-DLBCL
GCB-5	chr18	63318173	63318173	T	C	exonic	BCL2	524	211	0.2867	Missense_Mutation	p.E165G	Targerted Panel Gene	GCB-DLBCL
GCB-5	chr18	63318600	63318600	G	C	exonic	BCL2	315	103	0.2447	Missense_Mutation	p.L23V	Targerted Panel Gene	GCB-DLBCL
GCB-5	chr22	23288139	23288139	T	A	exonic	BCR	319	152	0.322	Missense_Mutation	p.W857R	Targerted Panel Gene	GCB-DLBCL
GCB-5	chr7	140753332	140753332	T	G	exonic	BRAF	642	171	0.2103	Missense_Mutation	p.K601N	Targerted Panel Gene	GCB-DLBCL
GCB-5	chr1	203305633	203305633	G	A	exonic	BTG2	165	60	0.2667	Missense_Mutation	p.M9I	Targerted Panel Gene	GCB-DLBCL

GCB-5	chr16	3770715	3770715	G	A	exonic	CREBBP	217	230	0.5145	Missense_Mutation	p.T912I	Targerted Panel Gene	GCB-DLBCL
GCB-5	chr16	3751787	3751787	A	C	exonic	CREBBP	424	168	0.2838	Missense_Mutation	p.C1240G	Targerted Panel Gene	GCB-DLBCL
GCB-5	chr20	58853359	58853359	G	C	exonic	GNAS	285	269	0.4856	Missense_Mutation	p.E32Q	Targerted Panel Gene	GCB-DLBCL
GCB-5	chr12	78198633	78198633	G	A	exonic	NAV3	183	177	0.4917	Missense_Mutation	p.G2159R	Targerted Panel Gene	GCB-DLBCL
GCB-5	chr2	70087957	70087957	T	G	exonic	PCBP1	286	131	0.3134	Missense_Mutation	p.F72V	Targerted Panel Gene	GCB-DLBCL
GCB-5	chr3	49375459	49375459	G	A	exonic;splicing	RHOA;RHOA	294	140	0.3226	Missense_Mutation	p.A44V	Targerted Panel Gene	GCB-DLBCL
GCB-5	chr16	11254872	11254872	A	G	exonic	SOCS1	222	96	0.3019	Missense_Mutation	p.Y203H	Targerted Panel Gene	GCB-DLBCL
GCB-5	chr6	21594985	21594985	C	T	exonic	SOX4	83	43	0.3413	Missense_Mutation	p.P151S	Targerted Panel Gene	GCB-DLBCL
GCB-5	chr19	30548029	30548029	G	A	exonic	ZNF536	181	205	0.5297	Missense_Mutation	p.E804K	Targerted Panel Gene	GCB-DLBCL
GCB-5	chr8	60743077	60743077	C	T	exonic	CHD7	385	154	0.2857	Nonsense_Mutation	p.Q549X	Targerted Panel Gene	GCB-DLBCL
GCB-5	chr17	65056515	65056515	G	A	exonic	GNA13	125	59	0.3207	Nonsense_Mutation	p.Q27X	Targerted Panel Gene	GCB-DLBCL
GCB-5	chr10	87864500	87864500	A	T	exonic	PTEN	389	105	0.2126	Nonsense_Mutation	p.R184X	Targerted Panel Gene	GCB-DLBCL
GCB-5	chr1	2557822	2557822	A	T	exonic	TNFRSF14	85	27	0.2411	Nonsense_Mutation	p.K56X	Targerted Panel Gene	GCB-DLBCL
GCB-6	chr1	102887057	102887057	C	A	splicing	COL11A1	630	108	0.1463	Splice_Site	.	Targerted Panel Gene	GCB-DLBCL
GCB-6	chr4	108899181	108899181	C	T	splicing	COL25A1	633	112	0.1489	Splice_Site	.	Targerted Panel Gene	GCB-DLBCL
GCB-6	chr16	81900611	81900611	G	A	splicing	PLCG2	136	27	0.1656	Splice_Site	.	Targerted Panel Gene	GCB-DLBCL
GCB-6	chr1	116536040	116536040	T	-	exonic	CD58	682	265	0.2798	Frame_Shift_Del	p.I185fs	Targerted Panel Gene	GCB-DLBCL
GCB-6	chr1	103078819	103078819	T	-	exonic	COL11A1	650	181	0.217	Frame_Shift_Del	p.K109fs	Targerted Panel Gene	GCB-DLBCL
GCB-6	chr14	32822935	32822935	A	G	exonic	AKAP6	597	100	0.1435	Missense_Mutation	p.K1708E	Targerted Panel Gene	GCB-DLBCL
GCB-6	chr11	108293376	108293376	A	G	exonic	ATM	781	120	0.1332	Missense_Mutation	p.I1559V	Targerted Panel Gene	GCB-DLBCL
GCB-6	chrX	77521416	77521416	A	G	exonic	ATRX	225	83	0.2695	Missense_Mutation	p.I2353T	Targerted Panel Gene	GCB-DLBCL
GCB-6	chr1	7744908	7744908	C	A	exonic	CAMTA1	347	288	0.4528	Missense_Mutation	p.P1419H	Targerted Panel Gene	GCB-DLBCL
GCB-6	chr7	2939879	2939879	A	G	exonic	CARD11	625	188	0.2312	Missense_Mutation	p.L245P	Targerted Panel Gene	GCB-DLBCL
GCB-6	chr5	635551	635551	C	A	exonic	CEP72	245	61	0.1993	Missense_Mutation	p.P291T	Targerted Panel Gene	GCB-DLBCL
GCB-6	chr16	3740485	3740485	C	A	exonic	CREBBP	608	97	0.1376	Missense_Mutation	p.Q1349H	Targerted Panel Gene	GCB-DLBCL
GCB-6	chr16	3782705	3782705	T	C	exonic	CREBBP	349	43	0.1097	Missense_Mutation	p.M518V	Targerted Panel Gene	GCB-DLBCL
GCB-6	chr8	3187890	3187890	G	T	exonic	CSMD1	354	73	0.1706	Missense_Mutation	p.P1867T	Targerted Panel Gene	GCB-DLBCL
GCB-6	chr8	3493666	3493666	C	A	exonic	CSMD1	479	57	0.1063	Missense_Mutation	p.G469C	Targerted Panel Gene	GCB-DLBCL
GCB-6	chr4	154366312	154366312	T	C	exonic	DCHS2	690	118	0.146	Missense_Mutation	p.N198D	Targerted Panel Gene	GCB-DLBCL
GCB-6	chrX	41344073	41344073	T	C	exonic	DDX3X	266	105	0.283	Missense_Mutation	p.L270S	Targerted Panel Gene	GCB-DLBCL
GCB-6	chr6	56536831	56536831	A	G	exonic	DST	625	94	0.1307	Missense_Mutation	p.L3454S	Targerted Panel Gene	GCB-DLBCL
GCB-6	chr6	93269636	93269636	T	C	exonic	EPHA7	844	125	0.129	Missense_Mutation	p.T492A	Targerted Panel Gene	GCB-DLBCL
GCB-6	chr10	89007804	89007804	T	C	exonic	FAS	414	80	0.1619	Missense_Mutation	p.C101R	Targerted Panel Gene	GCB-DLBCL
GCB-6	chr4	125416604	125416604	A	T	exonic	FAT4	513	87	0.1448	Missense_Mutation	p.I2334F	Targerted Panel Gene	GCB-DLBCL
GCB-6	chr19	39924484	39924484	C	T	exonic	FCGBP	304	49	0.138	Missense_Mutation	p.E518K	Targerted Panel Gene	GCB-DLBCL
GCB-6	chr1	240093551	240093551	G	A	exonic	FMN2	119	20	0.1439	Missense_Mutation	p.R481H	Targerted Panel Gene	GCB-DLBCL
GCB-6	chr4	78245306	78245306	A	G	exonic	FRAS1	418	71	0.1452	Missense_Mutation	p.H97R	Targerted Panel Gene	GCB-DLBCL
GCB-6	chr16	75641655	75641655	C	A	exonic	KARS	446	81	0.1537	Missense_Mutation	p.S72I	Targerted Panel Gene	GCB-DLBCL
GCB-6	chr12	49044767	49044767	G	A	exonic	KMT2D	312	59	0.159	Missense_Mutation	p.T1647I	Targerted Panel Gene	GCB-DLBCL
GCB-6	chr1	235746409	235746409	T	G	exonic	LYST	667	118	0.1503	Missense_Mutation	p.Q2633H	Targerted Panel Gene	GCB-DLBCL
GCB-6	chr1	39293548	39293548	G	A	exonic	MACF1	400	65	0.1392	Missense_Mutation	p.E700K	Targerted Panel Gene	GCB-DLBCL
GCB-6	chrX	141906925	141906925	G	T	exonic	MAGEC1	260	97	0.2717	Missense_Mutation	p.E507D	Targerted Panel Gene	GCB-DLBCL
GCB-6	chr12	124330886	124330886	G	T	exonic	NCOR2	334	65	0.1629	Missense_Mutation	p.P2306H	Targerted Panel Gene	GCB-DLBCL
GCB-6	chr1	248203269	248203269	C	G	exonic	OR2M3	96	17	0.1504	Missense_Mutation	p.L68V	Targerted Panel Gene	GCB-DLBCL
GCB-6	chr7	82914918	82914918	C	A	exonic	PCLO	794	107	0.1185	Missense_Mutation	p.Q4356H	Targerted Panel Gene	GCB-DLBCL
GCB-6	chr1	204429967	204429967	T	C	exonic	PIK3C2B	171	18	0.0952	Missense_Mutation	p.N1451S	Targerted Panel Gene	GCB-DLBCL
GCB-6	chr6	51627060	51627060	G	T	exonic	PKHD1	664	99	0.1298	Missense_Mutation	p.H3908N	Targerted Panel Gene	GCB-DLBCL
GCB-6	chr8	109518226	109518226	T	C	exonic	PKHD1L1	795	128	0.1387	Missense_Mutation	p.Y3917H	Targerted Panel Gene	GCB-DLBCL
GCB-6	chr6	127981232	127981232	G	A	exonic	PTPRK	363	55	0.1313	Missense_Mutation	p.P1221S	Targerted Panel Gene	GCB-DLBCL
GCB-6	chr1	237674159	237674159	A	T	exonic	RYR2	548	91	0.1424	Missense_Mutation	p.D2885V	Targerted Panel Gene	GCB-DLBCL
GCB-6	chr19	11023525	11023525	T	C	exonic	SMARCA4	256	38	0.1288	Missense_Mutation	p.L956P	Targerted Panel Gene	GCB-DLBCL
GCB-6	chr15	41878510	41878510	G	T	exonic	SPTBN5	151	24	0.1371	Missense_Mutation	p.T1101N	Targerted Panel Gene	GCB-DLBCL
GCB-6	chr4	1955747	1955747	T	C	exonic	WHSC1	263	58	0.1807	Missense_Mutation	p.L858P	Targerted Panel Gene	GCB-DLBCL
GCB-6	chr16	72788435	72788435	A	G	exonic	ZFHX3	540	87	0.1385	Missense_Mutation	p.Y3281H	Targerted Panel Gene	GCB-DLBCL
GCB-6	chr16	3736690	3736690	A	G	exonic	CREBBP	696	117	0.1439	Missense_Mutation	p.L1507P	Targerted Panel Gene	GCB-DLBCL
GCB-6	chr8	109461786	109461786	C	T	exonic	PKHD1L1	529	98	0.1563	Nonsense_Mutation	p.Q2421X	Targerted Panel Gene	GCB-DLBCL
GCB-6	chr3	77622281	77622281	T	A	exonic	ROBO2	634	90	0.1243	Nonsense_Mutation	p.Y610X	Targerted Panel Gene	GCB-DLBCL
GCB-7	chr16	3774694	3774694	C	T	splicing	CREBBP	243	105	0.3017	Splice_Site	.	Targerted Panel Gene	GCB-DLBCL
GCB-7	chr1	7663893	7663893	G	A	exonic	CAMTA1	241	199	0.4523	Missense_Mutation	p.S449N	Targerted Panel Gene	GCB-DLBCL
GCB-7	chr7	148147607	148147607	G	T	exonic	CNTNAP2	164	273	0.6247	Missense_Mutation	p.V891F	Targerted Panel Gene	GCB-DLBCL
GCB-7	chr22	41169528	41169528	A	T	exonic	EP300	129	99	0.4342	Missense_Mutation	p.S1400C	Targerted Panel Gene	GCB-DLBCL
GCB-7	chr13	35159791	35159791	A	G	exonic	NBEA	394	80	0.1688	Missense_Mutation	p.E1207G	Targerted Panel Gene	GCB-DLBCL
GCB-7	chr1	233160370	233160370	G	T	exonic	PCNXL2	206	85	0.2921	Missense_Mutation	p.L1144I	Targerted Panel Gene	GCB-DLBCL
GCB-7	chr12	57102878	57102878	T	C	exonic	STAT6	122	114	0.4831	Missense_Mutation	p.D419G	Targerted Panel Gene	GCB-DLBCL
GCB-7	chr17	7673820	7673820	C	G	exonic	TP53	22	83	0.783	Missense_Mutation	p.R228P	Targerted Panel Gene	GCB-DLBCL
GCB-7	chr2	167258027	167258027	C	T	exonic	XIRP2	528	139	0.2084	Missense_Mutation	p.P527L	Targerted Panel Gene	GCB-DLBCL
GCB-7	chr12	49026887	49026887	G	A	exonic	KMT2D	397	137	0.2566	Nonsense_Mutation	p.R5027X	Targerted Panel Gene	GCB-DLBCL
GCB-8	chr8	2957809	2957809	T	C	splicing	CSMD1	182	139	0.4317	Splice_Site	.	Targerted Panel Gene	GCB-DLBCL
GCB-8	chr12	122022182	122022182	T	G	exonic	BCL7A	93	46	0.3309	Missense_Mutation	p.W31G	Targerted Panel Gene	GCB-DLBCL
GCB-8	chr7	81997205	81997205	C	T	exonic	CACNA2D1	334	126	0.2739	Missense_Mutation	p.E546K	Targerted Panel Gene	GCB-DLBCL
GCB-8	chr3	48570165	48570165	C	A	exonic	COL7A1	191	103	0.3503	Missense_Mutation	p.R2485L	Targerted Panel Gene	GCB-DLBCL
GCB-8	chr2	96144778	96144778	C	T	exonic	DUSP2	43	81	0.6532	Missense_Mutation	p.A165T	Targerted Panel Gene	GCB-DLBCL
GCB-8	chr4	78466397	78466397	A	T	exonic	FRAS1	229	250	0.5219	Missense_Mutation	p.T2407S	Targerted Panel Gene	GCB-DLBCL
GCB-8	chr14	79280508	79280508	C	T	exonic	NRXN3	320	130	0.2889	Missense_Mutation	p.R73C	Targerted Panel Gene	GCB-DLBCL
GCB-8	chr9	137464387	137464387	C	A	exonic	PNPLA7	181	82	0.3118	Missense_Mutation	p.G1037C	Targerted Panel Gene	GCB-DLBCL
GCB-8	chr1	15937562	15937562	A	G	exonic	SPEN	351	114	0.2446	Missense_Mutation	p.T3476A	Targerted Panel Gene	GCB-DLBCL
GCB-8	chr10	46582121	46582121	G	A	exonic	SYT15	678	118	0.1475	Missense_Mutation	p.R195C	Targerted Panel Gene	GCB-DLBCL
GCB-8	chr4	105276125	105276125	T	C	exonic	TET2	351	161	0.3138	Missense_Mutation	p.L1872P	Targerted Panel Gene	GCB-DLBCL
GCB-8	chr1	216422170	216422170	G	C	exonic	USH2A	329	491	0.5981	Missense_Mutation	p.A56G	Targerted Panel Gene	GCB-DLBCL
GCB-9	chrX	33339257	33339257	A	C	splicing	DMD	418	120	0.223	Splice_Site	.	Targerted Panel Gene	GCB-DLBCL
GCB-9	chr4	93216912	93216912	G	A	splicing	GRID2	466	88	0.1583	Splice_Site	.	Targerted Panel Gene	GCB-DLBCL
GCB-9	chr3	78646147	78646147	C	T	splicing	ROBO1	329	82	0.1995	Splice_Site	.	Targerted Panel Gene	GCB-DLBCL

GCB-9	chr6	134174587	134174587	C	G	splicing	SGK1	323	106	0.2471	Splice_Site	.	Targerted Panel Gene	GCB-DLBCL
GCB-9	chr6	134174510	134174510	C	G	splicing	SGK1	307	99	0.2432	Splice_Site	.	Targerted Panel Gene	GCB-DLBCL
GCB-9	chr1	116544406	116544406	A	C	exonic	CD58	472	199	0.2957	Missense_Mutation	p.L90R	Targerted Panel Gene	GCB-DLBCL
GCB-9	chrX	41346607	41346607	C	T	exonic	DDX3X	585	174	0.2292	Missense_Mutation	p.R534C	Targerted Panel Gene	GCB-DLBCL
GCB-9	chr8	25389175	25389175	G	A	exonic	DOCK5	311	113	0.2665	Missense_Mutation	p.E1406K	Targerted Panel Gene	GCB-DLBCL
GCB-9	chr6	27867138	27867138	G	C	exonic	HIST1H1B	538	179	0.249	Missense_Mutation	p.A131G	Targerted Panel Gene	GCB-DLBCL
GCB-9	chr1	201211972	201211972	A	T	exonic	IGFN1	723	438	0.3766	Missense_Mutation	p.D2360V	Targerted Panel Gene	GCB-DLBCL
GCB-9	chr22	22888133	22888133	T	C	exonic	IGLL5	129	61	0.3211	Missense_Mutation	p.L27P	Targerted Panel Gene	GCB-DLBCL
GCB-9	chr6	37170360	37170360	G	A	exonic	PIM1	45	18	0.2857	Missense_Mutation	p.A20T	Targerted Panel Gene	GCB-DLBCL
GCB-9	chr6	37170484	37170484	G	A	exonic	PIM1	86	25	0.2252	Missense_Mutation	p.S61N	Targerted Panel Gene	GCB-DLBCL
GCB-9	chr6	37170993	37170993	C	T	exonic	PIM1	398	111	0.2181	Missense_Mutation	p.H68Y	Targerted Panel Gene	GCB-DLBCL
GCB-9	chr6	37170625	37170625	G	A	exonic	PIM1	134	37	0.2164	Missense_Mutation	p.C17Y	Targerted Panel Gene	GCB-DLBCL
GCB-9	chr6	37170486	37170486	C	T	exonic	PIM1	88	24	0.2143	Missense_Mutation	p.P62S	Targerted Panel Gene	GCB-DLBCL
GCB-9	chr6	37170485	37170485	C	G	exonic	PIM1	88	23	0.2054	Missense_Mutation	p.S61R	Targerted Panel Gene	GCB-DLBCL
GCB-9	chr8	109443714	109443714	G	C	exonic	PKHD1L1	673	170	0.2014	Missense_Mutation	p.G1535R	Targerted Panel Gene	GCB-DLBCL
GCB-9	chr10	87933126	87933126	C	T	exonic	PTEN	540	243	0.3103	Missense_Mutation	p.H296Y	Targerted Panel Gene	GCB-DLBCL
GCB-9	chr15	33724144	33724144	G	C	exonic	RYR3	302	82	0.2135	Missense_Mutation	p.D2294H	Targerted Panel Gene	GCB-DLBCL
GCB-9	chr16	11255366	11255366	C	T	exonic	SOCS1	77	49	0.3889	Missense_Mutation	p.R38H	Targerted Panel Gene	GCB-DLBCL
GCB-9	chr16	11255472	11255472	C	G	exonic	SOCS1	111	58	0.3412	Missense_Mutation	p.A3P	Targerted Panel Gene	GCB-DLBCL
GCB-9	chr16	11255453	11255453	G	C	exonic	SOCS1	115	57	0.3314	Missense_Mutation	p.A9G	Targerted Panel Gene	GCB-DLBCL
GCB-9	chr17	7674230	7674230	C	G	exonic	TP53	262	82	0.2384	Missense_Mutation	p.G206R	Targerted Panel Gene	GCB-DLBCL
GCB-9	chr13	44574241	44574241	G	T	exonic	TSC22D1	705	188	0.2105	Missense_Mutation	p.P612T	Targerted Panel Gene	GCB-DLBCL
GCB-9	chr8	102289485	102289485	G	C	exonic	UBR5	300	175	0.3676	Missense_Mutation	p.Q1561E	Targerted Panel Gene	GCB-DLBCL
GCB-9	chr2	61492337	61492337	C	T	exonic	XPO1	606	180	0.2287	Missense_Mutation	p.E571K	Targerted Panel Gene	GCB-DLBCL
GCB-9	chr15	44715613	44715613	C	A	exonic	B2M	477	207	0.3026	Nonsense_Mutation	p.Y86X	Targerted Panel Gene	GCB-DLBCL
GCB-9	chr17	65056395	65056395	G	A	exonic	GNA13	241	66	0.2143	Nonsense_Mutation	p.Q67X	Targerted Panel Gene	GCB-DLBCL
GCB-9	chr22	22888168	22888168	C	A	exonic	IGLL5	123	59	0.3242	Nonsense_Mutation	p.C3X	Targerted Panel Gene	GCB-DLBCL
GCB-9	chr12	124354927	124354927	G	A	exonic	NCOR2	153	41	0.2113	Nonsense_Mutation	p.Q1132X	Targerted Panel Gene	GCB-DLBCL

Table S5: List of Antibodies used for the Western Blot

Antibody	Catalog No.	Supplier
Actin	sc-47778	Santa Cruz
Btk	8547	Cell signaling Technology
Syk Antibody	2712	Cell signaling Technology
Phospho-Syk (Tyr525/526)	2751	Cell signaling Technology
Phospho-Btk (Tyr223)	5082	Cell signaling Technology
p65	sc-8008	Santa Cruz
Pim-1 (C93F2)	3247	Cell signaling Technology
Caspase-3 (D3R6Y)	14220	Cell signaling Technology
